# An open source tool for automatic spatiotemporal assessment of calcium transients and local ‘signal-close-to-noise’ activity in calcium imaging data

**DOI:** 10.1371/journal.pcbi.1006054

**Published:** 2018-03-30

**Authors:** Juan Prada, Manju Sasi, Corinna Martin, Sibylle Jablonka, Thomas Dandekar, Robert Blum

**Affiliations:** 1 Department of Bioinformatics, University of Würzburg, Würzburg, Germany; 2 Institute of Clinical Neurobiology, University Hospital Würzburg, Würzburg, Germany; Hebrew University of Jerusalem, ISRAEL

## Abstract

Local and spontaneous calcium signals play important roles in neurons and neuronal networks. Spontaneous or cell-autonomous calcium signals may be difficult to assess because they appear in an unpredictable spatiotemporal pattern and in very small neuronal loci of axons or dendrites. We developed an open source bioinformatics tool for an unbiased assessment of calcium signals in x,y-t imaging series. The tool bases its algorithm on a continuous wavelet transform-guided peak detection to identify calcium signal candidates. The highly sensitive calcium event definition is based on identification of peaks in 1D data through analysis of a 2D wavelet transform surface. For spatial analysis, the tool uses a grid to separate the x,y-image field in independently analyzed grid windows. A document containing a graphical summary of the data is automatically created and displays the loci of activity for a wide range of signal intensities. Furthermore, the number of activity events is summed up to create an estimated total activity value, which can be used to compare different experimental situations, such as calcium activity before or after an experimental treatment. All traces and data of active loci become documented. The tool can also compute the signal variance in a sliding window to visualize activity-dependent signal fluctuations. We applied the calcium signal detector to monitor activity states of cultured mouse neurons. Our data show that both the total activity value and the variance area created by a sliding window can distinguish experimental manipulations of neuronal activity states. Notably, the tool is powerful enough to compute local calcium events and ‘signal-close-to-noise’ activity in small loci of distal neurites of neurons, which remain during pharmacological blockade of neuronal activity with inhibitors such as tetrodotoxin, to block action potential firing, or inhibitors of ionotropic glutamate receptors. The tool can also offer information about local homeostatic calcium activity events in neurites.

## Author summary

Calcium imaging has become a standard tool to investigate local, spontaneous, or cell-autonomous calcium signals in neurons. Some of these calcium signals are fast and ‘small’, thus making it difficult to identify real signaling events due to an unavoidable signal noise. Therefore, it is difficult to assess the spatiotemporal activity footprint of individual neurons or a neuronal network. We developed this open source tool to automatically extract, count, and localize calcium signals from the whole x,y-t image series. As demonstrated here, the tool is useful for an unbiased comparison of activity states of neurons, helps to assess local calcium transients, and even visualizes local homeostatic calcium activity. The tool is powerful enough to visualize signal-close-to-noise calcium activity.

This is a *PLOS Computational Biology* Software paper.

## Introduction

Calcium ions mediate fast signaling to regulate neuronal development, synaptic transmission, and synaptic plasticity [1–5]. Calcium imaging has become a standard technique for mapping neuronal activity in neurons *in vitro* and *in vivo* [6,7]. Although calcium signaling of neurons is well investigated, calcium-dependent mechanisms underlying spontaneous or cell-autonomous excitability are not well understood [8–11]. Investigating the molecular mechanisms underlying spontaneous calcium influx revealed two principle mechanisms of how spontaneous excitation is initiated. Either spontaneous excitation is ligand-dependent and caused by the non-synaptic release of transmitters such as glutamate or GABA [12,13]. Alternatively, excitability is part of a developmental program and is triggered by the neuron itself, meaning by cell-autonomous excitation using subthreshold active ion channels, or is caused by self-enhancement of intrinsic excitability through autocrine signaling [10,14,15]. Furthermore, homeostatic calcium fluxes are cell-autonomously controlled and occur in cellular subdomains [16]. Three major explanations of why signaling of spontaneous or cell-autonomous excitability is not well investigated can be proposed: (1) Calcium transients can appear at very local places, and their spatiotemporal footprint is not predictable. (2) Proteins involved in neuronal excitation show a high functional diversity, depending on their locus of action. (3) Neuronal signals can be very fast and ‘small’, thus making it difficult to identify real signaling events due to the unavoidable measurement noise.

Techniques to extract calcium signals from imaging data are commonly region of interest (ROI) analyses. ROIs are typically selected manually or in a semi-automated manner [6,17,18]. This approach is time-consuming in assessing the spatiotemporal activity pattern of neurons. Moreover, the selection of ROIs and the criteria to define activity events is rather heuristic and largely user-dependent [17,18]. Signal identification, not only in calcium imaging data, but also in mass spectrometric data, is often performed using noise filtering and peak identification algorithms. However, this strategy creates a high false-positive rate, especially when low amplitude peaks are analyzed [19–21]. Calcium signal identification and signal source separation are two separate problems in calcium signal analysis. To support the analysis of imaging data many helpful automated or semi-automated computational tools have been developed [18,21–28]. Therefore, advanced computational tools are helpful to localize, extract and sort cellular signals to detect neuronal calcium activity [18] or to strengthen the detection of calcium activity by signal demixing and denoising [22,23,26]. Multiple arithmetical strategies to read out calcium signals from raw data exist, and each of these methods has its advantages [18,21,23,29]. However, strategies to identify local activity close to the noise level with high sensitivity are not well developed. There is a certain risk that activity events, which are not spike-like, or are very close to the measurement noise, may be ignored by computational tools using denoising, or feature-dependent methods for calcium time series analysis. This can be of advantage, as this strategy creates robust data to describe spike-like activity in neurons. However, those strategies might ignore activity below the spike threshold, or activity that is local and not synchronous with the firing pattern, or not triggered by voltage-dependent mechanisms. Another problem is that local calcium transients are commonly not shaped as calcium spikes (fast onset followed by a slower decay of the signal) and are filtered out by algorithms that expect a calcium spike or are filtered out because of computational strategies that define noise levels or noise signals [23,27].

Many biologically relevant calcium signals are very small and close to the measurement noise. Here, we tackle the problem of computing “signal-close-to-noise” activity, which is a calcium signal under pharmacological blockade of neuronal activity, in the absence of any exogenous stimulation or treatment. Here we define an activity event (signal) as a calcium event carrying information, while a computed noise event does not carry biological information. Therefore, we assume that homeostatic calcium fluxes of resting neurons [30] should create calcium events.

In certain research questions, the analysis of local calcium transients offers important functional information. Local calcium activity regulates functions such as neurotransmitter specification, neurite extension, growth cone dynamics, activity-dependent axon growth, network wiring, as well as synaptic scaling [10,12,31–36]. While we know much about functions of local calcium signaling, the molecules triggering local or spontaneous activity are not well understood.

Here, we introduce an intuitive computational approach to assess the activity state of neurons and to visualize signal-close-to-noise activity. The motivation was to create this tool for an unbiased computing of local activity in neurites, to monitor local excitability events, and to visualize homeostatic calcium activity. The bioinformatical tool is a refined Continuous Wavelet Transform (CWT)-based algorithm which identifies peak candidates in calcium activity signals occurring in the x,y-t imaging data set. As local calcium transients are hardly predictable or regularly shaped, we create a grid-based activity pattern over the x,y-field of the raw image data. CWT-guided peak detection was used to compute calcium signals, because we found that it targets critical problems in signal-close-to-noise computing: (1.) Baseline removal and signal smoothing is not needed and even small signals are accurately computed. (2.) The algorithm can be directly applied to raw data. (3.) Low amplitude peaks can still be real, and peak detection through local signal-to-noise ratio computation above a heuristic threshold may become an insufficient criterion to detect small signals, or to discriminate real signals from high amplitudes of noise signals. Therefore, instead of directly detecting peaks in the calcium signal, the algorithm identifies ridges in a CWT coefficient matrix and utilizes these coefficients to create an effective SNR for peak identification. These coefficients effectively guide the search for peaks that correspond to real calcium activity on the neurons. This improves the robustness of peak detection and keeps the false positive rate low [20].

Here we confirm that our strategy enables the identification of calcium activity events independent of segmentation parameters and shape parameters. The tool is easy to handle, powerful over the complete intensity value range, and ignores baseline shifts. More importantly, the computation identifies local activity events, even under pharmacological blockade of neuronal activity, and gives new information on calcium activity close to the signal noise. The tool is an open source tool.

## Design and implementation

Basic principles of the computational tool are explained in the methods section. We first explain the basic concept of our activity analysis approach and illustrate its performance based on calcium imaging data. Then we compare the tool’s features to alternative approaches. This is followed by detailed evaluation of the bioinformatics approach with calcium imaging data. Next, we map signal-close-to noise activity in neurites in the presence of inhibitors of neuronal activity in order to visualize and localize spontaneous and local neuronal activity events. Finally, we confirm signal-close-to-noise activity induced by homeostatic calcium fluxes.

**Fig 1:**
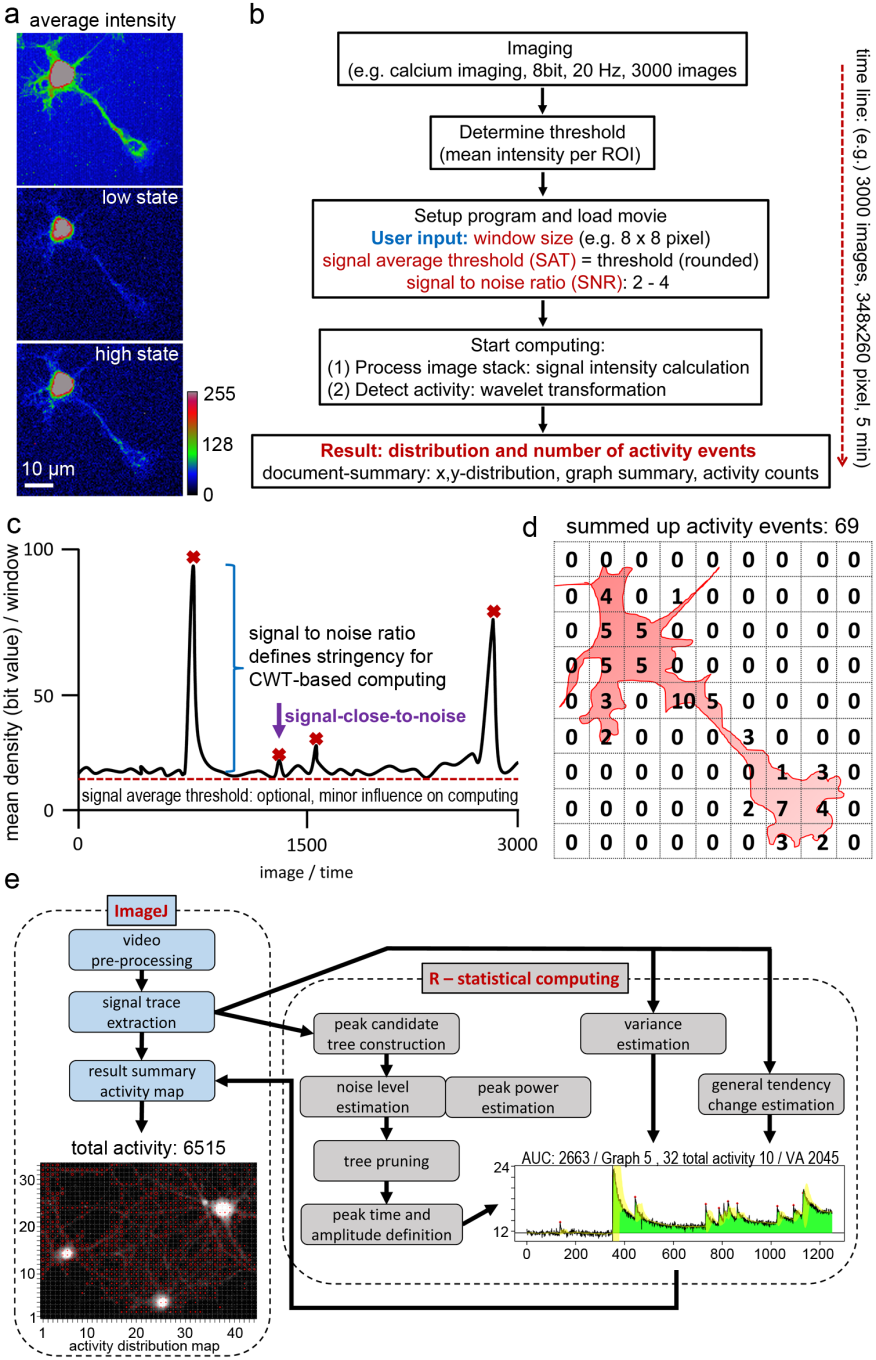
Overview of image analysis by NA^3^. **a**, Motoneuron loaded with a fluorescent calcium indicator. Average intensity projection of 1000 images to show the neuronal morphology. The neuron shifts spontaneously from a low activity state to a high activity state. Rainbow pseudocolor shows low intensity values in blue and high intensity values in red. **b**, Workflow for the analysis of calcium imaging raw data. After threshold determination according to a rule, the user defines the window size for x,y-grid, and chooses a signal-to-noise ratio value to tune the stringency of the tool. Two computations are started: (1) the signal intensity calculation, (2) the wavelet transform. A result pdf is automatically created. **c**, The signal-to-noise ratio (SNR) defines the stringency of the computation. The signal average threshold (SAT) can be used to set a signal threshold. The SAT can be close to the black level without having a strong impact on the computation result. **d**, The documentation pdf defines activity events, marks them, and counts them. All traces showing one or more activity events are given and used to count the total activity. An overview image is created that shows the grid over the first image of the movie, red circles to visualize the activity state of a grid window, and the total activity value, which is a computed value to describe the overall activity state of the neurons. The number of counted activity events per grid window events is shown in a text fil in the results folder. **e**, Overview of the NA^3^ workflow. The tool combines functions in ImageJ with “R”. Video processing and signal extraction occurs in ImageJ, before the signals are automatically transferred to “R”. In “R”, the event computing takes place. The result is created in “R” and exported as a pdf-file. The results are also transferred back to ImageJ to allow an interactive access to the data for image processing, ROI selection, or data evaluation.

To test our computational approach, we started with standard calcium imaging of cultured motoneurons. Motoneurons show spontaneous calcium transients commonly categorized as ‘global’ or ‘local’ activity [13]. In mouse motoneurons, this kind of activity has been shown to be cell-autonomous and to mediate activity-dependent axon growth [10,14,32]. We used the motoneuron system to see whether the computational tool is able to describe spontaneous shifts from a low activity state to a high activity state. In the low activity state, motoneurons show almost no spontaneous activity, before they change their activity state and show more than ten calcium transients per minute [10]. This experiment is used to explain the workflow of the calcium activity detector tool. We call the open source tool ‘*Neural Activity*^*3*^(*NA*^*3*^) (Fig 1).

### Basic concept and operation of the neuronal activity detection tool

Imaging can be performed with any imaging system that creates intensity values. Here, we used a CCD camera (8-bit) at 2.5 Hz (motoneurons), or 10–20 Hz (hippocampal neurons) (Fig 1a). Next, the image series is uploaded to the activity detector tool. After opening the image series, the program asks for three tuning parameters: the grid **window size (WS)**, the **signal-to-noise ratio (SNR)** and the **signal average threshold (SAT)** (Fig 1b and 1c; see S1 Manual). The window size (WS) will create a grid of ROIs with a defined size (e.g. 8x8 pixel, Fig 1d). The grid is used to localize activity events. The signal-to-noise ratio (SNR) is the most important value and defines the stringency of the calcium event detector. SNR values of 1.5 to 4 (or even 1.3) provide robust results on typical datasets acquired with the help of fluorescent calcium indicators. In our experience, computing of high signal-to-noise datasets, such as those obtained with modern genetically encoded calcium indicators [23,37], can be performed with much higher SNR values. The signal average threshold (SAT) can be used to threshold the signals or to remove background signals. The threshold is a mean fluorescence value in a selected ROI, which can be taken either from the cell-free background or from any low-activity area in the x,y-image series. The SAT can be determined by a simple rule; SAT is the rounded threshold value. Note that the SAT can be set close to the black level. The reason is that the tool works with a CWT-based detection algorithm [20] and therefore background removal with the help of a threshold value has only a minor influence on the final result. Calcium activity event identification is then performed by two operations: (1) ***Process image stack*** to read out the signal intensity values in each grid window; (2) ***Detect activity*** to perform a wavelet transformation and to detect activity peaks.

A pdf-document is automatically generated and put into the result folder of the activity tool (S1 Fig; computing WS8, SNR2, SAT7, MAC1). This document shows on page 1 an image of the time series, the grid, and loci of calcium activity indicated by red circles within the grid window. The diameter of the circles is bigger when the tool finds more activity events. All activity events are summed up to give a computed value, the ‘total activity’ value (Fig 1d). This value represents all calcium events in the whole x,y-t images series. The value represents the activity state under a specific experimental condition. Furthermore, the resulting pdf document shows all traces in which a calcium activity event was found, thus enabling fast access to the raw data and the interpretation of activity events by the tool. Activity events are marked and counted (Fig 1c and 1d). Finally, a txt-file is generated that shows the calculated numbers of activity per grid window in a x,y-table structure (S2 Fig). We also offer the possibility to increase the stringency of the tool. The user can select to count only those signal traces with more than one activity event (***Minimum activity counts*; MAC**). Furthermore, the tool is able to analyze signal fluctuations (***Include variance***). With this function, the tool offers a value called variance area. The idea is that signal fluctuations are bigger, when, for instance, homeostatic calcium fluxes are pronounced. This is very useful to experimentally test whether fast fluctuations in a calcium signal trace are activity-dependent or not. The tool also offers the possibility to estimate a point of change in the general behavior of the signal in order to compute states of long-lasting activity, for instance after stimulation of neurons with an agonist. This feature of the tool is based on a confidence test that recursively searches for the point in the signal where the biggest change in variance and average occurs. We also implemented a ROI tool, which allows the re-analysis of loci of interest. ROIs can be selected with the help of a ROI manager, an ImageJ function, and can be analyzed as described above. ROI information can also be imported to the ROI manager.

Calculation of 1300 images needed just about 60 seconds with the help of a standard desktop computer (Windows X64 Intel core i-5 machine with 4 Gigabyte of RAM memory). This short computing time allows fast testing of user-dependent tuning parameters, for instance to compute the same signals with different SNR values.

The tool processes the calcium imaging videos in ImageJ [38] for signal extraction. The data are automatically transferred to ‘R’ (https://www.r-project.org/) for the statistical computing (Fig 1e; details are explained in the methods section). For some applications, pre-processing of the videos might be necessary. For this purpose, it is of advantage that the presented tool is embedded in ImageJ, which gives the user access to a wide range of image processing tools and interactive access to the data.

In Table 1 the application features of our tool are compared to alternative approaches, delineating the areas where we find that our tool is used advantageously.

**Table 1:** Feature comparison.

Study	Features and advantages	Implementation
Romano et al., 2017	Toolbox including algorithms and interactive tools for image pre-processing and segmentation, estimation and labeling of significant single-neuron signals, mapping of neuronal responses, and detection of activity-correlated neuronal clusters. Automatic somatic ROI or hexagonal grid of ROIs for signal extraction and other ROI tools. Signal candidates are labeled in the trace.	Matlab
Pnevmatikakis et al., 2016	Cell detection or component segmentation with CNMF and signal extraction after spike detection with a spike model. Signal denoising and signal deconvolution implemented. Potent in demixing of overlapping signals, advanced framework for analysis of calcium activity of neurons. The tool is very powerful in extracting signals as a component.	Matlab Python
Patel et al., 2015	Analysis package (FluoroSNNAP) for semiautomatic cell detection with stCA, knowledge-based signal identification and extraction based on a user-defined calcium event waveform library. Spike and connectivity analysis are integrated. It uses signal templates to identify and label signal candidates at the onset point of the signal. The tool can also be used with a deconvolution strategy.	Matlab
Maruyama et al., 2014	Cell detection or component segmentation with plain NMF for demixing of slow and fast signal components in a x,y-field.	Matlab
Janicek et al., 2013	Spike-feature analysis based on a spike model.	Matlab
Mukamel et al., 2009	Cell detection or component segmentation with PCA/stICA; signal extraction, deconvolution, and spike detection, advanced for identification of calcium spikes and to extract individual signals.	Matlab
This study	Unbiased pixelwise x,y-grid, counts activity events, event identification by CWT-guided peak detection, uses a pruning strategy to value signal candidates, estimates signal variance to define activity-based signal fluctuations. Developed for local, spontaneous event detection in the whole x,y-field. Creates two natural numbers for activity state comparison: the total activity value and the variance area for signal fluctuation. Offers an activity image of the neurons and is useful to identify loci of activity in neurites or in the neuropil. The user can also use ROI information with the help of the ImageJ ROI manager.	ImageJ / R in BIO7 (open source,no software license required)

## Results

### Computation of high versus low activity in motoneurons

**Fig 2:**
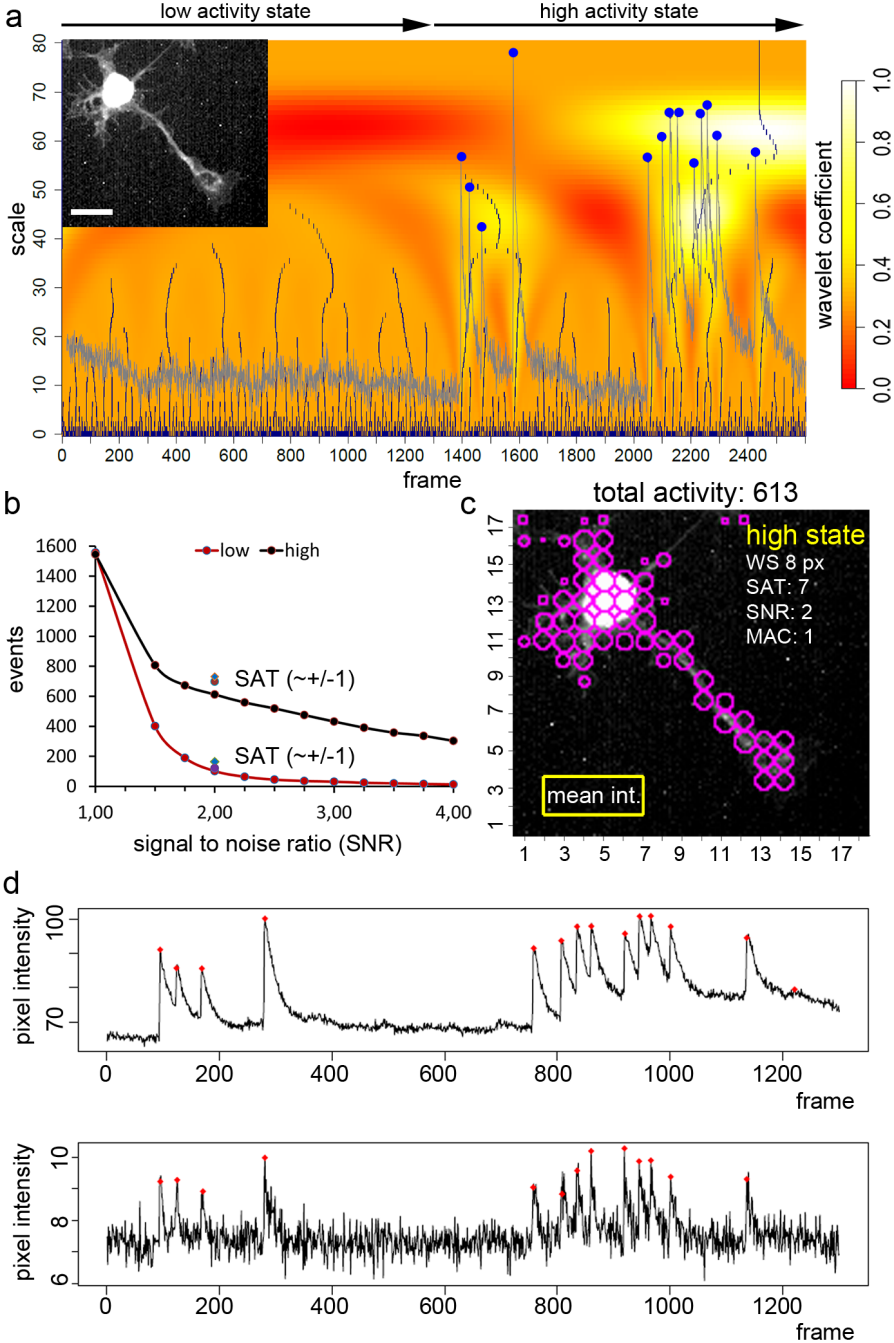
Calcium activity assessment with the activity detector tool. **a**, Principle of calcium activity event detection. Activity event identification is shown for a representative grid window (per se representing a ROI) on a single motoneuron. This motoneuron (shown in the inlet, and in Fig 1) shows global calcium transients. 2600 images (frames; x-axis) were analyzed. The grey trace shows the raw mean intensity values of a representative grid window. After extraction of the image signal in a grid window, all local maxima of the intensity signal are identified at several scales (y-axis) and signal candidates are selected and marked (blue dots). Details are explained in the methods: *Strategy for calcium event (peak) identification*. **b**, Effect of tuning parameters on calcium activity event detection. The total number of computed activity events (y-axis) in relation to changes in the user-dependent signal-to-noise ratio (SNR). Two activity stages of the motoneuron are compared. The low activity state (*in a*, frame 1–1300) and the high activity state (frame 1301–2600). Discrimination of the high activity state and the low activity state is very effective over a broad range of SNR values from 1.5 to 4. The signal average threshold was set to an intensity value of 6 (up-rounded mean intensity value seen in the background). A conservative SAT value was selected and modified at a SNR of 2 (blue square; SAT = 5 a.u.; purple circle; SAT = 6 a.u.). **c**, Data documentation 1: x,y-t summary. The image shows the distribution and number of calcium activity events raised by a spontaneously active motoneuron. Such an image is automatically generated by the program. The user-dependent tuning parameters for this analysis are given. The image field 142 x 130 pixel was automatically split in a grid of 8 x 8 pixel (WS 8 px). Magenta circles indicate areas with calcium events. The smaller the diameter, the less activity is found in the corresponding grid window. All detected calcium activity events are summed up to offer the value ‘total activity’. **d**, Data documentation 2: the individual traces represent changes in fluorescence in one grid window. The tool automatically generates traces (black line) representing a grid window and shows raw bit values (y-axis) over the frame number (x-axis). Calcium activity events detected by the tool are labeled with a little red square at the peak point. The upper panel describes the graph in grid 5/14 (x/y-axis) in the somatic region of the motoneuron. Here raw bit values ranged from about 65 to 110. In the lower panel a region in the growth cone of the motoneuron was analyzed (grid 13/4; x/y-axis). Here, raw mean bit values in the grid range from 6 to 10. Note the robust detection of global activity despite an almost 10-fold difference in the mean intensity values in the corresponding grid window.

Firstly, we asked whether the tuning parameters (SAT and SNR; Fig 1b) are robust enough to assess the activity state of a motoneuron. For convenience, this is again explained based on the single motoneuron (Fig 2). The movie was split into a low activity state (frame 1–1300) and a high activity state (frame 1301–2600). We compared the low activity state with the high activity state of this globally firing motoneuron over a wide range of SNR values between 1.5 and 4 (Fig 2b). Increasing the SNR increased the stringency of the tool, meaning that the tool preferentially identified stronger changes in fluorescence, or spike-like activity events (Fig 2b). The SAT can be determined according to a rule, but little changes in the SAT only have a minor influence on the total number of activity events (Fig 2b). As the calculation time is relatively short, the user can easily find a SNR, which fits best to the individual experimental condition or experimental setup. In this example, the high activity state of the motoneuron is defined by the total activity value of 613 (WS: 8; SNR: 2; SAT: 6; MAC: 1; Fig 2c). The low activity state is represented by a total activity value of 102 (S3 Fig). Although the underlying intensity signals within the grid windows are based on an almost 10-fold difference in the intensity values between the soma and the growth cone, the tool is able to compute relevant changes in signal intensity in the grid windows that superimpose the motoneuron (Fig 2d).

### Tool performance

**Fig 3:**
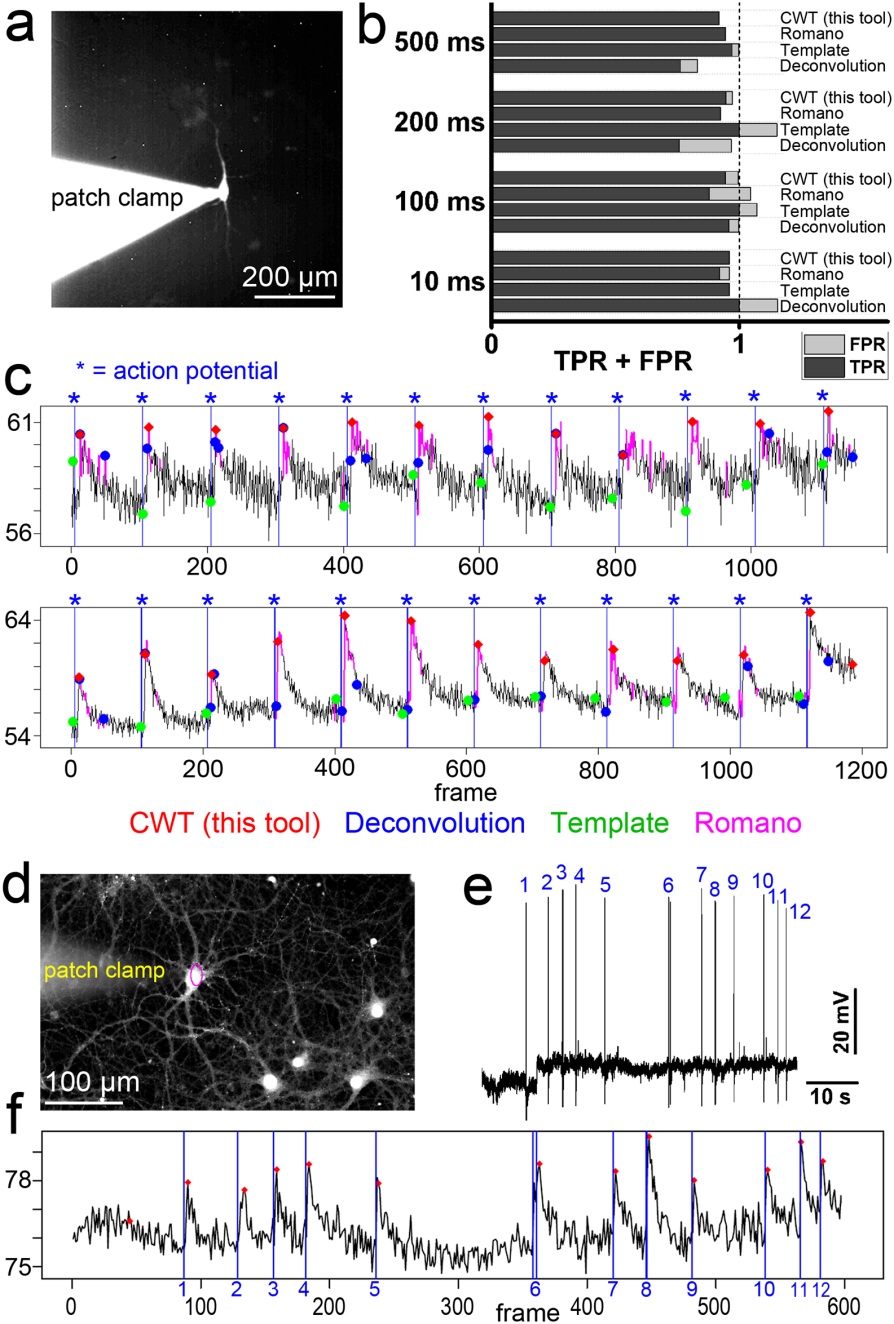
Computation of action potential-induced calcium events. a, Experimental approach. Whole-cell patch clamp recording and parallel calcium imaging of single cells. Here, the calcium indicator was applied with the help of the patch clamp pipette. **b,c**, Analysis of true-positive responses (TPR) and false-positive responses (FPR) based on parallel calcium imaging and patch clamp recording. Action potentials were induced by current injection (12 times, 200 pA, interstimulus interval: 5 s) for different times (10–500 ms). Current injection of 200 pA for 10 ms (upper panel in c) or 100 ms (lower panel in c) induced single action potentials (indicated by the blue label and vertical line. Calcium imaging was performed at 20 Hz. Calcium event labeling by four computational approaches. Four computational strategies for calcium event definition were applied; our CWT-approach, deconvolution, template-matching, and definition of significant signals above a computed baseline (‘Romano toolbox’). **d**, Experimental approach. Hippocampal neurons were cultured for 24 days in vitro. At this age, neurons develop glutamatergic synapses with mature hallmarks [39] and become spontaneously active. Cells were loaded with OGB1-AM and investigated with patch clamp recording and calcium imaging at 20 Hz. **e, f**, Electrophysiological recording of action potentials induced by spontaneous activity. In this trace, twelve action potentials are marked and correlate with AP-induced calcium spikes. All twelve calcium events (ROI, cell soma in a) were labeled by the CWT computation under these low SNR conditions.

To test the performance and reliability of our computation, we performed simultaneous whole-cell patch clamp recording and calcium imaging. First, we offered the calcium indicator dye with the intracellular patch clamp solution (Fig 3a) and stimulated the cells by twelve current injections for 10, 100, 200, or 500 ms to induce action potentials (AP) and AP-induced calcium transients. We extracted the calcium signals and determined whether the AP-induced calcium transients can be detected by our computation. Our computational strategy detected the AP-induced calcium event (true positive response, TPR) with high precision (Fig 3b and 3c). On average, at an imaging speed of one image per 50 ms, the time between the AP and the time point of computed AP-induced calcium peaks was 320 ms ± 65 ms (n = 192 events from 4 cells, mean value ± variance). False-positive events (FPR) were a minor problem under these circumstances. Other event detection methods also provided reliable results (Fig 3b). While a deconvolution method marked several false-positive events, template-matching strategy and a strategy to extract significant signals above a computed baseline [27] labelled the AP-induced calcium signal with high precision [21].

Next, we asked whether our computation could also detect spontaneous activity in long-term cultured hippocampal neurons (DIV 24) (Fig 3d–3f). APs generated by spontaneous activity were inducing typical calcium spikes, and these calcium events were detected by our computational approach (Fig 3f). S4 Fig shows how our tool and other computations identify or count activity events in a calibration dataset showing simultaneous imaging with loose-seal cell-attached recording in four GCaMP expressing neurons [37] (data taken from: https://crcns.org/data-sets/methods/cai-1/about-cai-1).

### Computing of calcium spikes (high SNR conditions)

**Fig 4:**
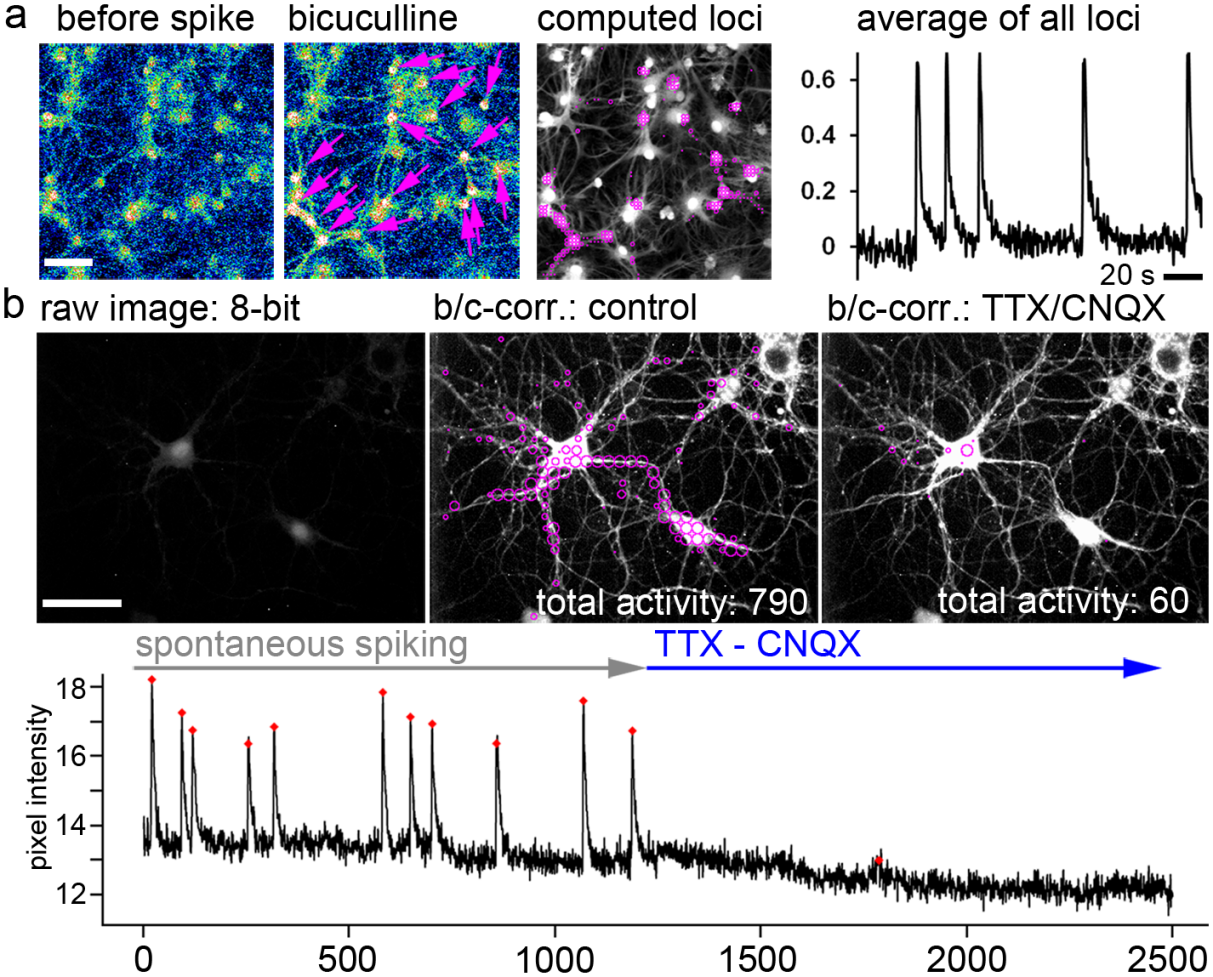
Calcium spike-detection under stringent computing conditions. **a**, Confocal imaging of synchronously spiking glia-derived neurons (re-computing of earlier data; [40]. Cells were loaded with calcium indicator OGB1 to label glial cells and neurons. Spontaneous activity was induced by inhibition of GABAergic signaling with bicuculline to induce the spiking behavior in the neural network. A magenta arrow labels all neurons. Computed loci are shown. The average signal trace representing all computed loci is shown. **b**, Imaging was performed under low-light conditions. The raw image represents this imaging situation. Loci of computed activity events are shown on a brightness-contrast corrected image of the neurons. Under control conditions, spontaneous spiking is observed on the somata, but also in the periphery of the neurons. Spike blockade (TTX, CNQX) correlates with a reduced number of computed activity events. Graph: Raw intensity values are plotted against the frame number. The calcium spikes are efficiently blocked by TTX and CNQX. Scale bar in a: 100μm; in b: 50 μm.

To see how the tool behaves under high signal-to-noise ratio conditions, we re-computed a recent dataset of typical calcium spikes generated by synchronously active neurons (Fig 4a) [40]. In this example, calcium signals of glia-derived neurons were investigated by confocal calcium imaging [40]. Under continuous perfusion, neurons were treated with 10 μM bicuculline to block gamma-aminobutyric acid (GABA)_A_ receptors and to induce calcium spikes (Fig 4a) [40,41]. By using high stringency settings, the tool automatically extracts the spike signal (Fig 4a, average of all computed loci) and marks the grid windows in which the neurons show spike behavior (Fig 4a, computed loci). Note that under these imaging conditions, calcium spikes are preferentially observed in the neuronal somata. Next, we computed spontaneously spiking primary hippocampal neurons (Fig 4b). Here, the bioinformatics tool is powerful enough to automatically mark typical calcium spikes and to count 790 activity events (Fig 4b, middle). Acute blockade of the calcium spikes with the inhibitors tetrodotoxin (TTX) to inhibit TTX-sensitive voltage-gated sodium channels, and CNQX (6-cyano-7-nitroquinoxaline-2,3-dione) to inhibit ionotropic glutamate receptors, blocked the generation of calcium spikes. A more than 10-fold lower total activity value and a much lower number of active loci illustrate the change in activity (Fig 4b, right). The decline in the cytosolic calcium signal after activity blockade may be due to the low activity of voltage-gated calcium channels and the inability of neurons to maintain high calcium in the ER and cytosol [4,30,42]. The experiment shows that the activity detector is powerful enough to identify global spike-activity over a broad range of intensity signals.

Next we asked how the tool behaves under more complex experimental conditions such as chemical LTP induction.

### Assessment of activity states in hippocampal neurons

**Fig 5:**
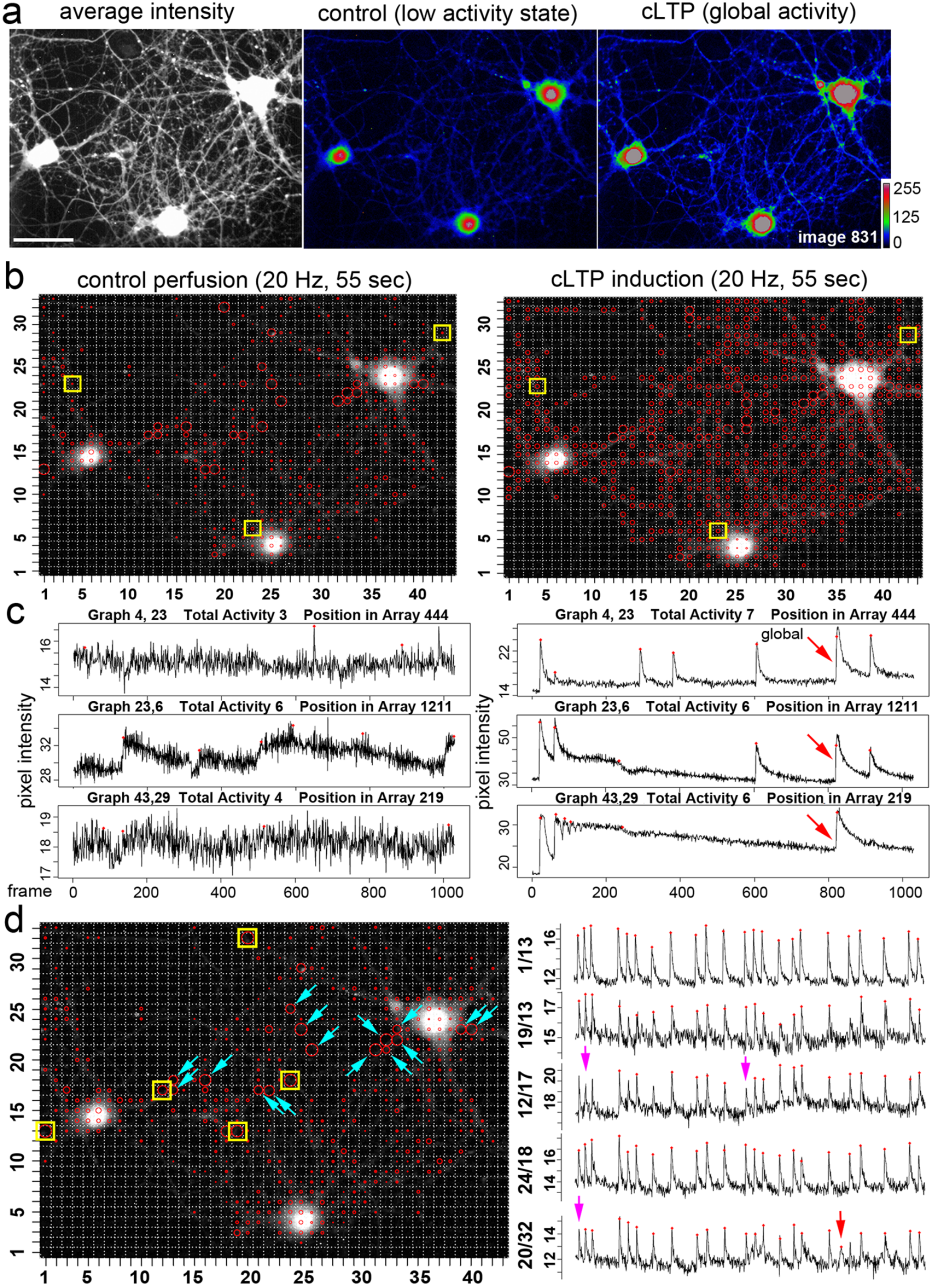
Activity profile of cultured hippocampal neurons before and after activity induction. **a**, Average intensity: Hippocampal neurons loaded with a fluorescent calcium indicator. Control; neurons at a low activity state. cLTP; neurons in a global activity state after treatment with a chemical LTP induction solution. **b**, Activity distribution under control conditions. Calcium imaging was performed for 55 seconds with a speed of 20 Hz. Virtual total activity number (WS8, SNR 2.5, SAT 11, MAC 2): 2135 activity events. Note that regions of high activity are indicated by circles with a larger diameter (Fig 5d). Under cLTP treatment, neurons increase their total activity number to 4785 events. Yellow marks point to grid windows shown in c. **c**, Under control conditions, activity events close to the baseline trace are found at some positions. After cLTP treatment local and global calcium signals (global: red arrow) are detected. Calcium spikes and local calcium transients are marked. **d**, Calcium spikes in loci of synchronous activity. (left) Spontaneous activity profile of hippocampal neurons under control conditions. Neuronal somata do not exhibit a spiking behavior. Calcium spikes are identified in the periphery in indicated grid windows. Yellow squares point to grid windows, which are shown on the right with the corresponding signal trace and the activity marks. Blue arrows point to other grid windows with this synchronous activity pattern. Out-of-synchronicity events are also detected (Red arrow on the right). Some obvious calcium spikes were overseen by the computation due to the stringency parameters used for this analysis (purple arrows). In Fig 6e we show that the spikes are detected when the same data are analyzed at SNR values of 2.0 and 1.5.

Next, we performed calcium imaging with hippocampal neurons at a speed of 20 Hz and acquired thousands of images (up to 10,000) per experiment under continuous perfusion. To induce increased activity, neurons were stimulated with an extracellular solution for chemical LTP induction (Fig 5). Two situations are compared; the control image sequence shows the time window before stimulation (Fig 5b and 5c, left panel), while the cLTP stimulation phase is an equally long image series to describe the acute stimulation (Fig 5b and 5c, right panel). Under control conditions, regions of high activity were preferentially identified in the neuronal periphery over neurites (Fig 5b). Many of the computed signals were very small and showed no typical shape of a classic calcium spike (Fig 5c; S5 Fig; WS8, SAT11, SNR 2.5; MAC2). Local activity with typical synchronous calcium spikes showed up at multiple positions of the x,y-field (Fig 5d, yellow and blue marks). A look at the underlying raw image data showed that this spiking activity is caused by multiple varicosities of an axon-like structure. During chemical LTP induction, neurons showed increased spike-like activity (S6 Fig; WS8, SAT11, SNR 2.5; MAC2), and even developed global synchronous activity over cell somata and neurites (Fig 5b). The increase in activity is described by the total activity values (2135 activity events versus 4785 activity events). The tool marked many activity events with a noise-like character (Fig 5c, example traces on the left side). During stimulation with the cLTP-inducing solution, the same neuronal structures increase their cytosolic calcium levels, some oscillated in their calcium signal, and also developed typical calcium spikes (Fig 5c, example traces on the right side), and the tool calculated an increased number of activity events.

### Camera noise and false negative event computation

**Fig 6:**
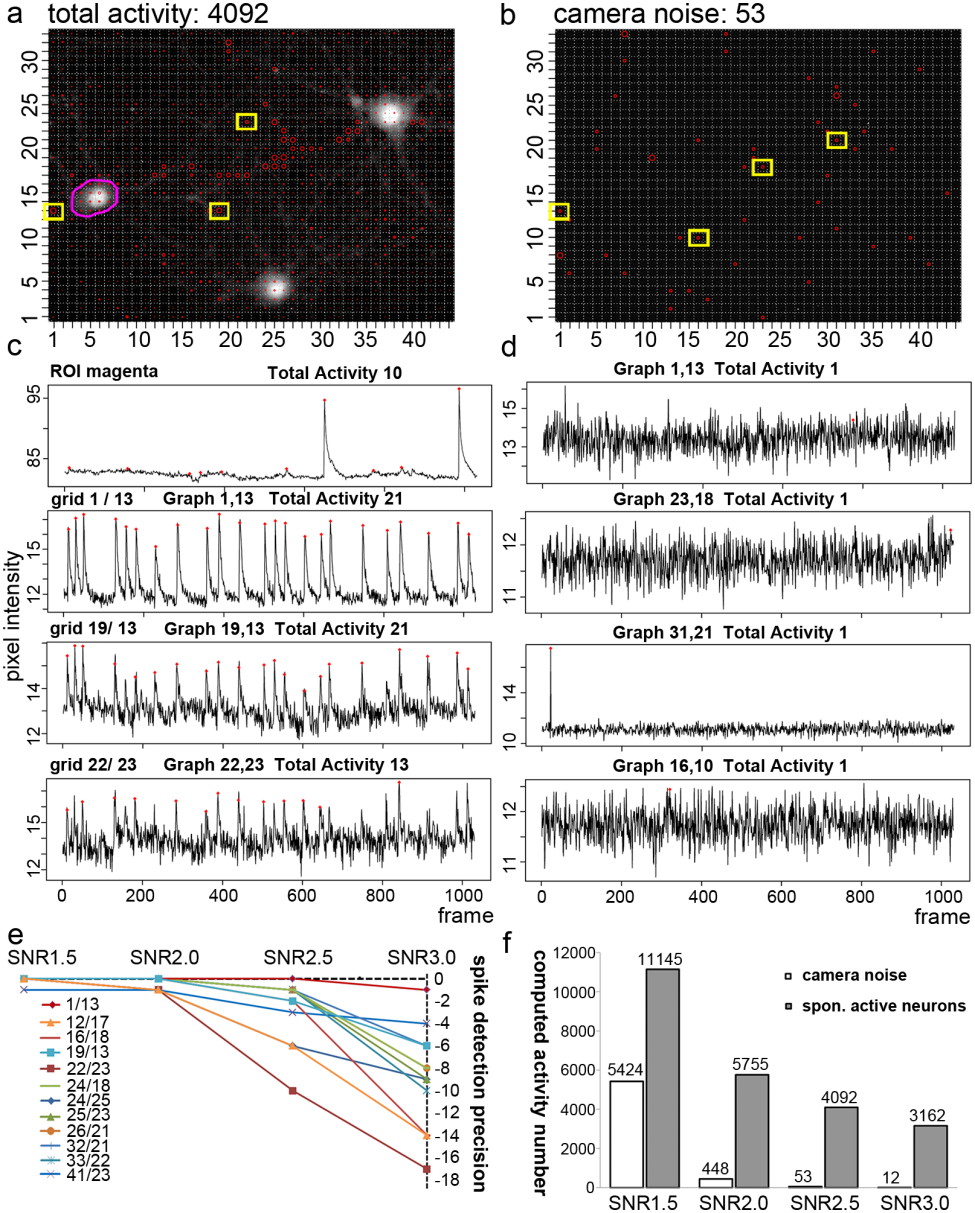
Wavelet-based activity detection in noise signals and spike-detection precision in a spontaneous active hippocampal neuron. **a** Activity distribution under control conditions (movie as in Fig 4b, left panel). Here, analysis was performed with the following parameters: WS8, SNR 2.5, SAT 2, MAC 1). 4092 activity events were computed. **b**, Noise video analysis. A homogenous fluorescence signal was imaged (identical camera settings). Analysis was performed with the following parameters: WS8, SNR 2.5, SAT 2, MAC 1). 53 noise events were computed. Some signals are camera-based (graph 31/21). **c**, Calcium spikes in loci of synchronous activity. Loci are marked in (a). All cell soma ROI (magenta) was computed with the ROI tool in NA^3^.**d**, Typical signal traces found in the noise video. Grid windows are indicated in (b). **e**, Spike-detection precision. Areas showing 23 synchronous spikes (1/13–41/23) are compared with a subthreshold SAT value, at different SNR values. The graph shows the underestimation in the number of spikes in the y-axis. **f**, Comparison of computed events in the noise video (in b) compared to spontaneous active neurons (in a). Settings were: WS8, SNR variable, SAT 2, MAC 1. All SNR values allow the discrimination between the noise state and the active state. The higher the SNR, the better is the stringency of the tool. Small activity events are underestimated under high SNR conditions.

The stringency of the tool depends on the wavelet algorithm and the chosen SNR. In Fig 5d, five signal traces are marked and show synchronous activity in the periphery. Purple arrows point to calcium spikes which were not counted by the activity detector, while the red arrow points to a local event that is only present in this trace (grid window 20/32). To show how the tool operates real noise in relation to noise-like activity, we compared the camera signal with real imaging data (Fig 6). For simplicity, this is explained based on the data in Fig 5. We selected 12 grids and the underlying signal traces showing synchronous activity (as in Fig 5d). In these traces, 23 calcium spikes can be identified in all of these grid windows. Grid windows of four representative loci are marked in Fig 6a, corresponding signal traces in Fig 6c. We put the SAT close to the black level to include all grid windows in the analysis. Next we determined the spike detection precision under different SNR values (SNR 1.5 –SNR 3.0) (Fig 6e). The tool computed all 23 events with a SNR of 1.5, but finds fewer events with an SNR of 3.0, which reflects the small change in signal intensity in these regions. For instance, in Fig 6c, grid window 22/23 shows a high sensitivity to high SNR values (‘worst case’). Here, the mean intensity signal lies between 12–14.5 arbitrary units (*de facto* reflecting the mean of the raw bit values), and therefore high SNR values ignore some calcium spikes. However, in the best case, in grid window 1/13, the spike detection precision is constant over a wide range of SNR values, because the signal is very clear. Here, the mean raw values of the signal shifts from about 10–16 arbitrary units during a calcium spike. An auto-correction removes border effects caused by the calculation itself. The missing calcium events in Fig 5d (purple arrows) were excluded because of a too stringent definition of the SNR. Furthermore, it shows that it can be useful to analyze signal traces of the same video under different SNR conditions in order to describe different experimental conditions, as, for instance, before or after activity blockade.

Next, we asked whether the tool computes too much noise. We imaged a homogenous light source, a fluorescent paper, with identical camera settings (Fig 6b) and computed the noise signal with different SNR values. The SAT was again set at 2, meaning close to the black level, while the mean intensity value in the image field was 13. All activity events were counted (MAC of 1). Representative signal traces are shown in Fig 6d. With a SNR of 1.5, 5524 events were computed in the noise video, while 11145 events were found in with neurons (Fig 6f). However, with an SNR of 2.5, the noise signals (53) were in a good balance compared to the signals found in neurons (4092). One has to consider that the program was computing 1452 grid windows of 1030 images. For this specific setting and imaging situation, this indicates that about 1.3% of the computed neuronal activity events reflected camera noise. Furthermore, this indicates that even with an SNR of 1.5, more than 5500 activity events in the whole x,y-t imaging raw data might be regarded as neural activity. The camera noise alone cannot be responsible for the high number of signal-close-to-noise activity events detected by the algorithm.

**Fig 7:**
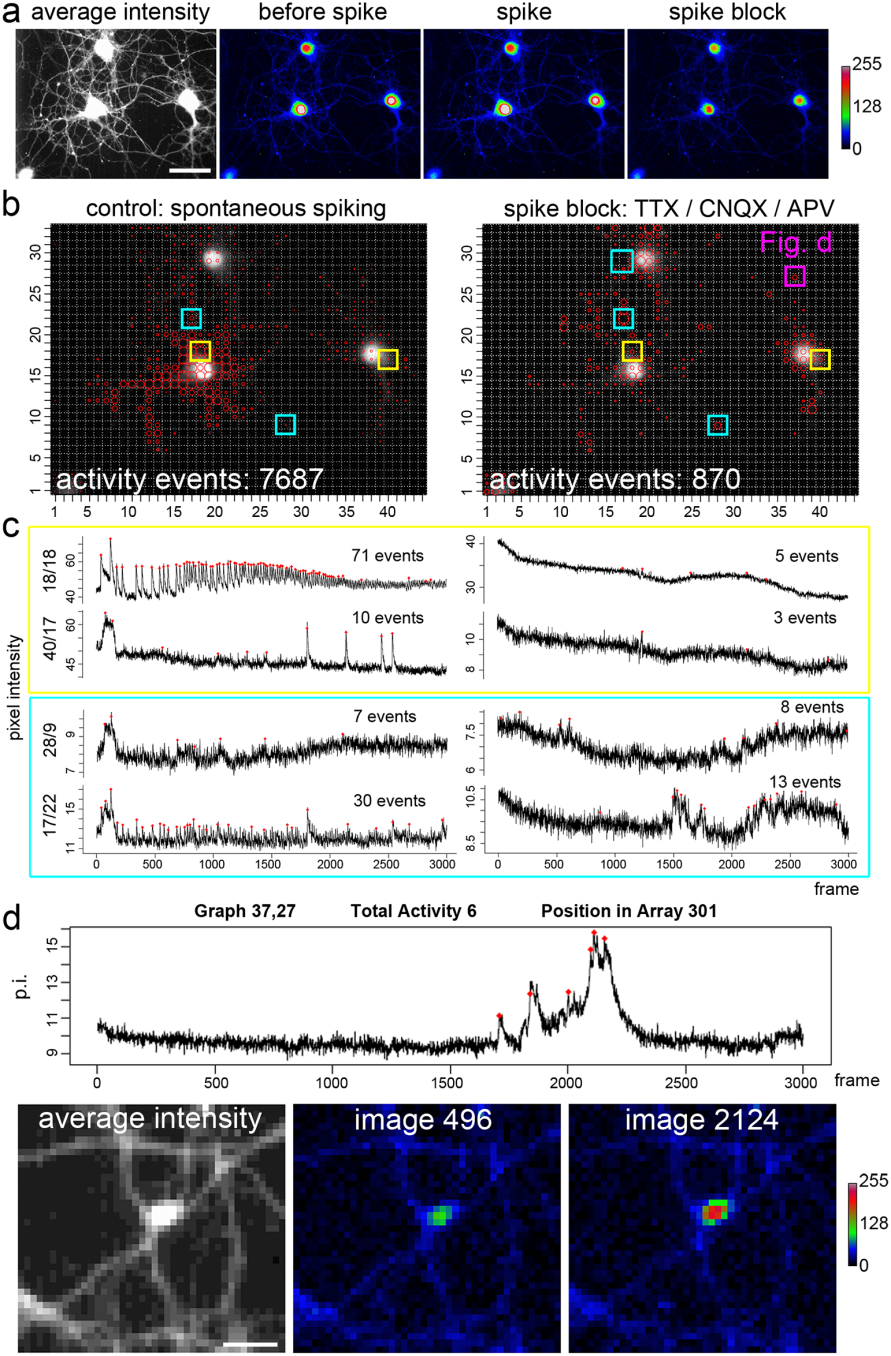
Activity profile of hippocampal neurons after calcium spike blockade. **a**, Average intensity: Hippocampal neurons loaded with a fluorescent calcium indicator. Pseudocolor images: neurons before a spike, during a calcium spike (seen in the lower left cell), and after spike blockade. 3000 images, acquired at 20Hz are analyzed (WS8, SAT7, SNR 3). **b**, Under control conditions, one neuron (the lower, left) shows a high spiking activity, as indicated by the activity pattern (red circles in the grid windows). After acute treatment with an inhibitor cocktail (TTX, CNQX, APV), spiking behavior is blocked (yellow squares; traces in c), non-spike-like activity events become visible on the soma and in the periphery. Shape and number of residual activity signals are quite diverse (bright blue squares; traces in c). **c**; Grid window-specific signal traces with the corresponding activity marks (red) as indicated in b. **d**, Activity hotspot in the periphery, in a varicosity-like structure. The activity hotspot is indicated by a contrast-enhanced average intensity projection. Image 496 shows the low activity state, while image 2124 indicates the high activity state. The corresponding grid window is indicated in (b).

However, this experiment does not prove whether very small signals identified by the algorithm reflect a biological phenomenon or not. To target this question, we performed the following experiments. First, we imaged hippocampal neurons in presence of specific inhibitors of neuronal activity (Fig 7). Furthermore, we investigated activity events and signal fluctuations of homeostatic calcium fluxes during acute withdrawal and re-addition of extracellular calcium under activity blockade [30].

### Computing of activity events under calcium spike-block conditions

To prove whether the activity detector marks too many false-positive events, we tested the tool on imaging data of spontaneously firing neurons, and blocked activity and excitatory neurotransmission with TTX, a powerful inhibitor of action potential firing, CNQX, and APV (D-2-amino-5-phosphonovalerate), to inhibit ionotropic glutamate receptors. Before activity blockade, the activity detection tool could well monitor the calcium-spike activity of a neuron (Fig 7), but also found many small and non-spike like activity events (Fig 7c, grid 28/9). Here, for simplicity, we use the term calcium spike to describe the typical waveform of AP-induced calcium spikes, with a fast onset and a slower decay of the signal. Acute perfusion with the activity inhibitor cocktail stopped the appearance of the classical calcium spike (Fig 7c), hence, less activity events were computed. However, many small activity signals were found in the neuronal periphery. This non-spiking activity was typically local, meaning not a global calcium signal, or signal component of the complete neuron. Many of these signals were not shaped like typical calcium spikes with a fast onset and a slow decay, and were not uniformly shaped (Fig 7d, purple rectangle to indicate a local activity hot spot).

### Detection of neuronal activity events close to the measurement noise

**Fig 8:**
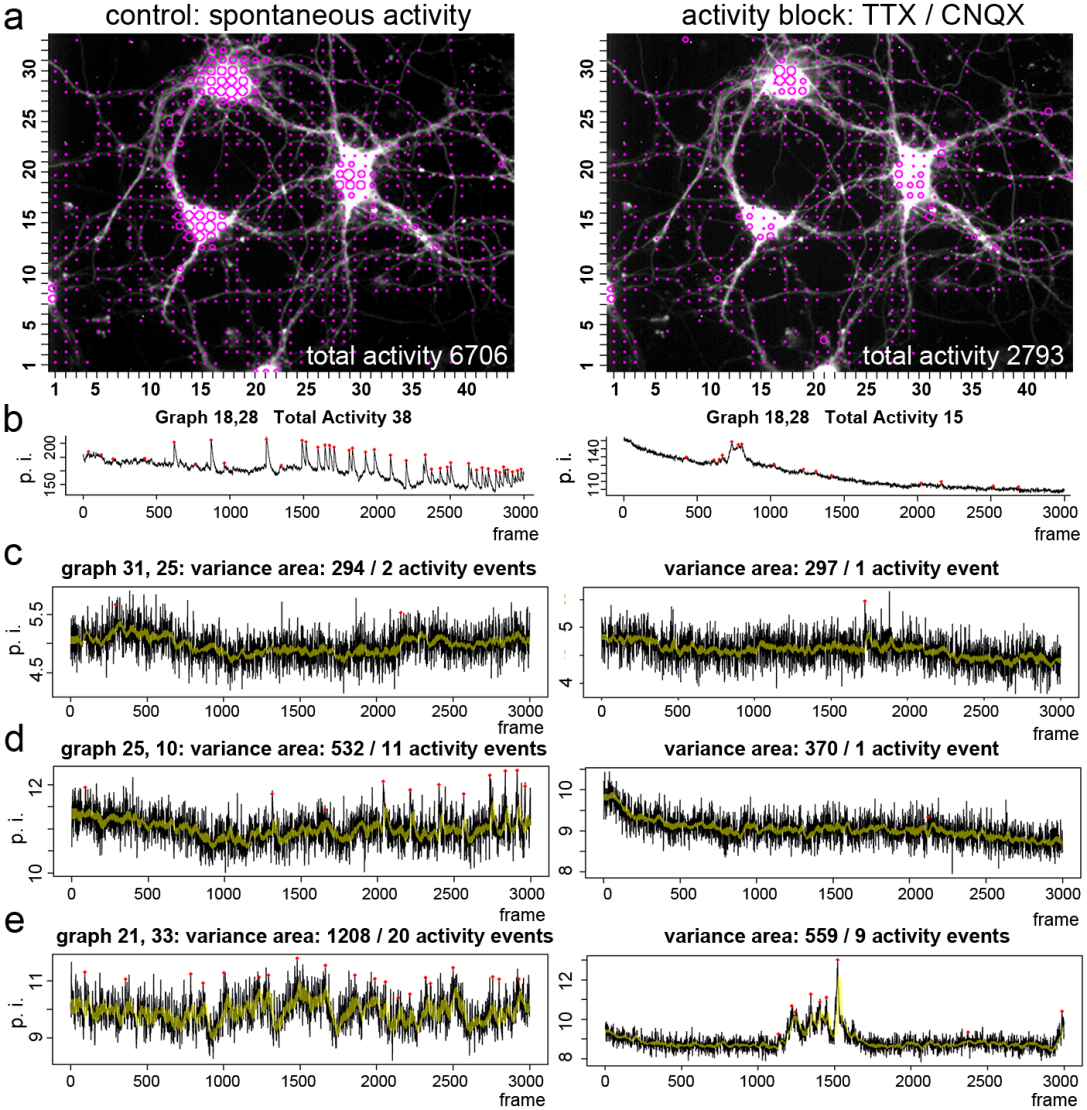
Parallel computing of activity events and variance area. **a**, Activity map of spontaneous activity of hippocampal neurons (DIV 10) before and after acute activity blockade. **b**, Calcium spike formation (left trace) is blocked by the inhibitor cocktail (right trace). **c—e**, Raw traces representing typical calcium signals in non-spiking areas (black trace). The yellow band indicates the variance area in a sliding window of 30 images. Activity marks are indicated as red dots. The activity state of the grid windows is described with two parameters, the variance area, and the number of activity events. **c**, Signal trace with one or two computed activity events. The variance area is almost identical before and after spike block. **d**, Signal fluctuations, represented by the variance area, become smaller under spike block conditions. Furthermore, the activity inhibitor cocktail reduces the number of activity events. **e**, This grid window over a neuritic element shows high signal fluctuation, which correlates with a higher number of activity events. Spike-blockade reduces the variance area and the number of activity events. However, a local activity event is not blocked by the inhibitor cocktail. Structural elements of local activity on the basis of this analysis are shown in Fig 9.

Next, we asked whether activity events close to the baseline are random fluctuations in the signal, or whether these small activity events reflect calcium fluxes or activity close to the baseline. Theoretically, when a neuron shows fluctuations in the calcium signal and many small calcium transients, but no typical calcium spikes, then the variance of the signal should be bigger than under conditions when activity is blocked. We again imaged hippocampal neurons (DIV10; 10.000 images, 20Hz), and compared neuronal activity under control conditions and after acute activity blockade with TTX and CNQX (Fig 8). Typical calcium spikes were detected (Fig 8a and 8b; left trace) and the pharmacological treatment was sufficient to block the typical calcium spikes with a fast onset followed by a decaying transient (Fig 8b, right trace). Then we calculated the variability of each signal using a sliding window of 30 frames and determined the variance and the mean of the signal (computed with the tool: WS8, SAT7, SNR 3; Include variance: 30). Finally, we determined the area of the variance around the mean in the signal trace (yellow band in Fig 8c–8e) and determined the number of activity events in the same traces with the activity detection tool. We call this value VA_30_, for variance area in a sliding window of 30 frames (S7 Fig, shortened example, 12 of 289 pages). In grid windows with a low number of activity events (none to two), the variance area value VA_30_ was almost identical between control conditions (Fig 8c, left trace, VA_30_ = 294, 2 events) and after TTX/CNQX block of neuronal activity (Fig 8c, right trace; VA_30_ = 297, 1 event). However, in another grid window (Fig 8d), the variance area shifted during activity blockade from VA_30_ = 532 to VA_30_ = 370, and activity events were reduced from 11 to 1, showing that many of the small activity events in the control condition (left trace) are sensitive against inhibitors of neural activity, and might be real calcium signal events. In Fig 8e, a high variance area value (VA_30_ = 1208) correlated with a high number of activity events (20 activity events). Activity blockade with TTX and CNQX reduced the variance area to VA_30_ = 559, indicating reduced calcium fluxes after activity blockade. However, despite the strong reduction in signal fluctuation, the activity detector identified a local (not global) TTX/CNQX-independent, long-lasting transient with 9 activity events (Fig 8e, lower trace, frames 1100–1700). This shows that the computational assay is able to describe small loci of activity with two different values, the activity event number and the variance area. We controlled that our protocols, meaning imaging over several minutes and blocking of typical calcium spike behavior is reversible and that calcium spike activity recovers upon wash out of the corresponding inhibitors (exemplarily shown in S8 Fig).

**Fig 9:**
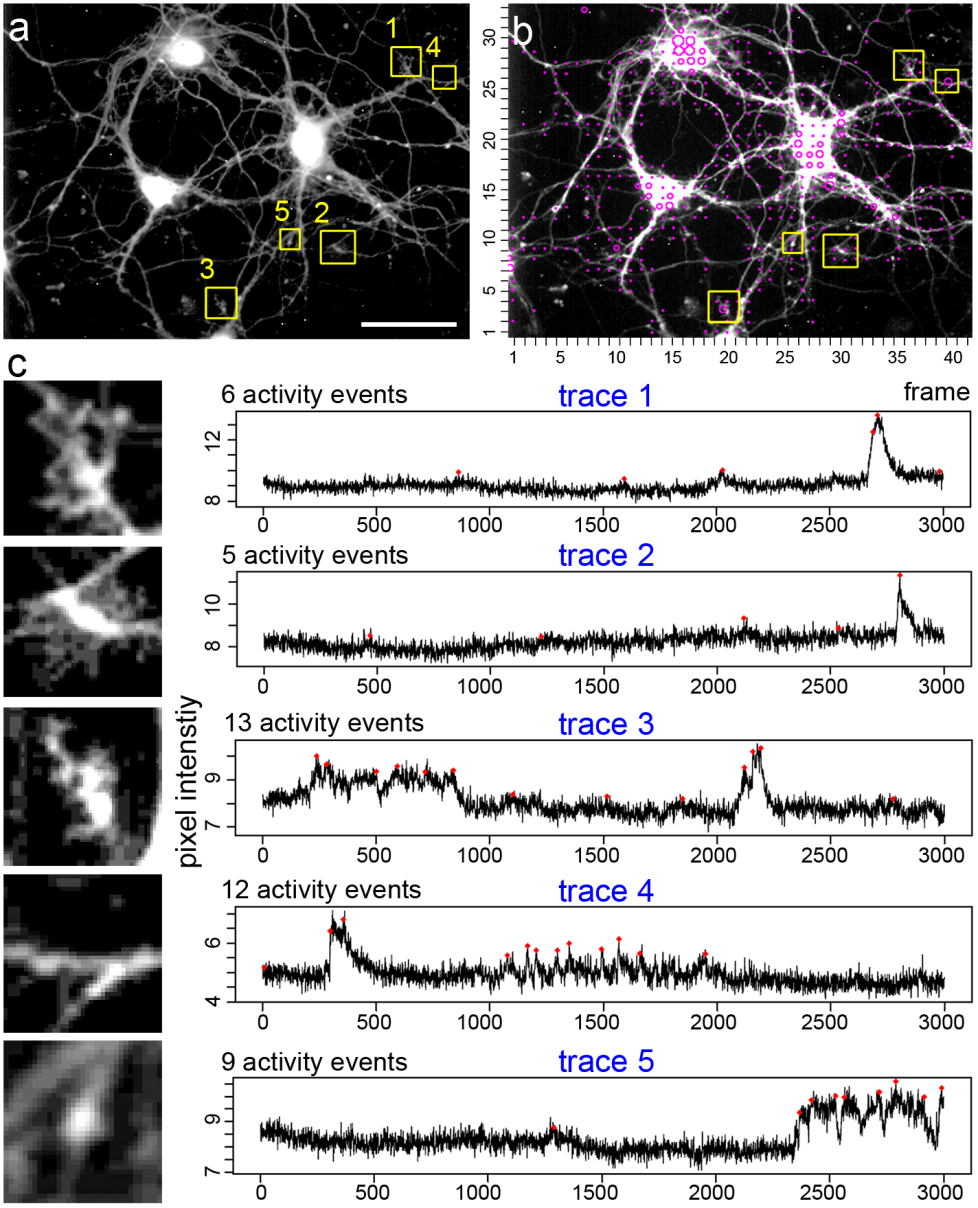
Structures with high rates of local activity after calcium spike blockade. **a**, Average intensity image of hippocampal neurons loaded with the calcium indicator. Grid windows with high rates of local activity are shown in yellow (see c). **b**, Activity map (see also Fig 8). **c**, Five regions of interest are indicated (trace 1 –trace 5). The upper three represent growth cone-like structures, the lower two traces represent hotspots on neurites. Note the variability and the diverse character of the calcium signal patterns detected by the computational approach.

Zooming in on corresponding loci (Fig 9) shows that growth cone-like structures (Fig 9c, trace 1–3), neuritic elements (Fig 9c, trace 4), or varicosity-like structures (Fig 9c, trace 5) form these local activity hotspots. Computed local activity events exhibit either a calcium spike-like shape (Fig 9c, trace 1, 2), or reflect a phase of increased fluctuations (e.g. Fig 9c, trace 4: images 1000–2000), or a sudden jump in the activity state (Fig 9c, trace 5: image 2300–3000). The local activity events with the shape of a ‘broad’ or ‘elongated’ calcium spike are reminiscent of typical cell-autonomous local calcium spikes in motoneurons, which are mediated by local activity of voltage-gated calcium channels. In motoneurons, these local calcium events are triggered by local activity of TTX-insensitive voltage-gated sodium channels and are mediated by the local activation of high-voltage activated calcium channels [10,14,32]. The data confirm that many ‘small’ activity events in an active locus of a neuron are not solely a misinterpretation of the measurement noise. We term these events in calcium imaging data as ‘signals-close-to-noise’ activity.

### ‘Signal-close-to-noise’ activity events are a biological phenomenon

**Fig 10:**
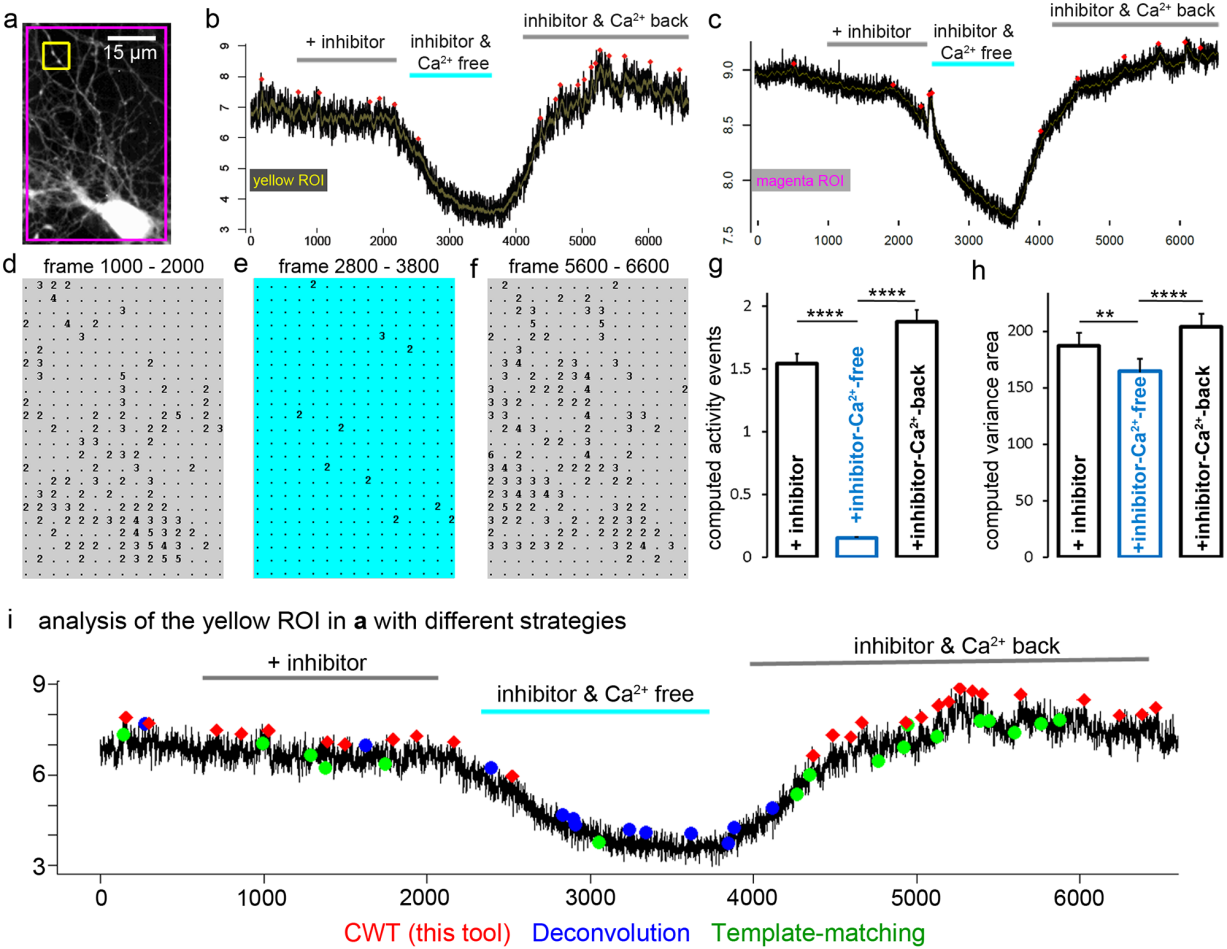
Computing of signals close to the noise level. **a**, Typical hippocampal neuron, loaded with calcium indicator. Two ROIs are indicated. **b,c**, Calcium traces representing the yellow and magenta ROI in a. Removal of extracellular calcium causes a decline in the calcium indicator signal. This correlates with a reduced number of computed activity events (red dots). **d-e**, Number of computed activity events are shown on the x,y-grid. Under calcium-free conditions (cyan), a low number of activity events was found in the signal trace. In the presence of extracellular calcium, more activity events are computed by the algorithm. Regions of activity are found in the soma, but also on distal neurites. **g,h**, Summary graphs for computed activity events and variance area. The virtual activity value has a strong discriminative power to describe the experimental situation (calcium-free versus calcium present). n = 164 ROIs, mean ± SEM; one way ANOVA (Kruskal-Wallis) followed by Dunn’s multiple comparison test; p-values: ** < 0.001; **** < 0.0001. **i**, Representative signal analysis (yellow ROI in a) with deconvolution (blue dots), template-matching (green dots), and our CWT-approach. Phases of homeostatic activity in presence of extracellular calcium are best described with help of template-matching and CWT-computation.

While some activity events under blockade of neuronal activity with TTX, CNQX, and APV can easily be regarded as real activity events (e.g. in Fig 8d or Fig 9e), many computed signals cannot easily been distinguished from noise. We asked whether statistical computing of activity events could be used to describe the biological phenomenon of homeostatic calcium fluxes in resting neurons [30]. Under conditions of activity blockade (TTX, CNQX, APV, 50 μM NiCl_2_), resting neurons show pronounced homeostatic calcium fluxes between the endoplasmic reticulum and the extracellular space, as recently shown by direct ER calcium imaging [30]. To compensate unavoidable loss of ER calcium over the plasma membrane, resting neurons balance ER calcium levels through a continuous calcium influx mechanism with properties of store-operated calcium entry (SOCE) [30,42–44]. To compute this calcium activity, we imaged calcium fluxes in resting hippocampal neurons and acquired 6600 images at a frequency of 10 Hz (Fig 10a). After one minute under steady-state conditions, neuronal activity was blocked with a high amount of TTX (500 nM), CNQX and APV (each 20 μM). Then extracellular calcium was withdrawn for more than three minutes, before extracellular calcium was re-added to stimulate neuronal SOCE. Computational analysis was then performed under medial stringency conditions (SAT 2, SNR 2.5, MAC2). As shown in the signal traces in Fig 10b and 10c, removal of extracellular calcium led to a brief decline in the cytosolic calcium signal (from bit value 7 to bit value 3), which was accompanied by a decline in computed activity events (Fig 10d and 10e). This was observed for individual grid windows (for instance Fig 10b, yellow ROI in a), or the whole image plane (Fig 10c, magenta ROI). Re-addition of extracellular calcium also restored the intensity of cytosolic calcium signals and more activity events were computed (Fig 10f).

We asked then whether the number of activity events or the change in the variance area is best suited to describe the changes in signal-close-to noise activity. We summed up the activity events (Fig 10g) and the computed VA_30_ area (Fig 10h) of all computed grid windows. The computed activity event value was well suited to distinguish signal-close-to-noise activity in the presence or absence of extracellular calcium. The tool identifies the calcium activity when the power of the signal is high enough in comparison to the intensity values in the neighborhood. Signal traces showing a decrease in cytosolic calcium concentration, as it happens to neurons under low calcium conditions (see Fig 10b, frames 2000–4000), will affect the CWT computation of the trace. Nevertheless, this has a minor influence in the activity event detection, because the algorithm constructs the ridges according to local maxima and estimates the power of an activity peak according to its close vicinity. The variance area value could also statistically distinguish between the two activity states, albeit with much less discriminatory power. The example shown here is representative of 15 imaging series performed with five independent neuron preparations. These results suggest that signal-close-to-noise activity can be regarded as a biological phenomenon, here triggered by homeostatic calcium fluxes [30], and can be visualized in an unbiased way with the bioinformatics tool described here and both computed values, the total activity value and the variance area.

Our tool identified a region of high activity in the neurites of the neuron. We used this ROI information (yellow ROI in Fig 10a) to show how other calcium event identification strategies analyze the signal (Fig 10i). A deconvolution strategy offered many events under calcium withdrawal conditions, suggesting that these signals may be false positive. Notably, template-matching [21] (green labels in Fig 10i) and our CWT/pruning strategy found signal events before calcium withdrawal and after re-addition of calcium, indicating that both computational approaches can be used to define phases of homeostatic calcium activity in neurites. Signal extraction with the help of the ‘Romano toolbox’ [27] failed to extract and define a significant signal above a computed background noise signal in phases of higher homeostatic activity in the presence of calcium and is therefore not marked in Fig 10i. Finally, we analyzed this calcium movie with a tool for component segmentation and signal extraction [23]. This tool extracted five signal components from the movie (S9 Fig). The typical signal pattern of calcium withdrawal and calcium re-addition was mirrored in four of five components. Notably, the computation indicates that the phase of calcium withdrawal is a phase of low activity (indicated by a straight line in the signal components 1, 3, 4; S9 Fig). This suggests that the computation underlying this signal demixing strategy is useful to extract and interpret homeostatic activity from neurites. However, tens of loci of local homeostatic calcium activity were overseen and not identified as a signal component (compare Fig 10d–10f with S9 Fig). Furthermore, the calcium withdrawal effect appears in the background signal and is erroneously considered to be a noise signal (S9 Fig). Here, we assume that an unbiased grid strategy might help to offer an initial segmentation of the image data, before signals are extracted and demixed from the corresponding structures in the grid.

In summary, CWT-based computation and signal pruning is suited to identify calcium activity components in loci of homeostatic calcium activity and the data can be used to count activity events for comparison of activity states. After minimal parameter tuning, two other strategies, template matching according to Patel et al. [21] and signal component analysis according to Pnevmatikakis et al. [23] would also be suited to compute signal-close-to-noise calcium signals. Here, some computational adaptations and tuning parameters might be computed and tested in the future. We found that counting of activity events in grid windows is useful to describe, in consideration of the pros and cons, local homeostatic calcium activity.

## Discussion

We developed and tested an open source bioinformatics tool to identify local calcium transients and ‘signal-close-to-noise’ activity from x,y-t imaging raw data. The tool is designed for an unbiased comparison of activity states of neurons to identify loci of activity in the whole x,y-t image material. Its good performance for this research question was meticulously tested (Figs 6–10), yielding unique features (Table 1) fruitful for high and low activity detection in motoneurons, activity state assessment in neurites of hippocampal neurons and monitoring local neuronal activity events close to the measurement noise.

After discussing performance and optimal parameter tuning, we explain choosing regions of interests and practical considerations. We believe that our tool is quite powerful and will even allow shifting the focus of neuronal activity detection to new biological questions, such as the contribution of subthreshold active ion channels in local neuronal excitation, or the contribution of local store-operated calcium homeostasis in neuronal excitability.

The tool can be used as a calcium spike detector, but is, in our opinion, more powerful in evaluating and computing calcium signaling events which are small, irregularly shaped, close to the measurement noise, and in the periphery of the neuron. Different strategies are already used to describe calcium activity automatically and each of the methods has specific advantages [18,21–23,25,45,46]. However, the individual essential requirements on an automated calcium signal detector are quite different, and therefore an all-purpose tool does not exist. We searched for a strategy powerful enough to create an overall census of calcium activity in a wider x,y-field. The tool described here looks highly sensitive for local, specific activity and rejects in each peak given the null hypothesis “all is just noise” after automatic testing. Our tool accepts that a signal trace is never smooth. Pre-processing of raw data is not needed and all information from a grid window is included. If there are ‘inactive’ pixels in a grid, then they do not significantly contribute to the signal. It is a strong feature of our strategy that it works close to the signal noise level and therefore sees signals even if there is an inactive pixel close to an active pixel. On the one hand our strategy sounds risky, as it is prone to overestimate activity events close to the measurement noise [18,23,25] under small SNR values. On the other hand, it does not ignore local activity close to the measurement noise. The tool is not based on simple peak candidate filtering above an arbitrary noise threshold. It is based on continuous wavelet transform-based guided peak detection with a second derivative Gaussian as mother wavelet. This mother wavelet is appropriate for peak detection, as it enhances the peak and diminishes the neighboring signal values. For this reason, our tool is able to identify signal-close-to-noise calcium activity.

### Tuning parameters to change the stringency of activity detection

Specific parameter settings were important to achieve optimal performance; examples are given in the results (Figs 1, 2 and 5). Here we discuss this more in general. To find a balance between two extremes, namely overestimation of activity through the computation of signals within noise, and disregarding of real activity close to the noise, we included simple tuning parameters. These tuning parameters, the signal-average threshold, the signal-to-noise ratio, the minimal activity count number, and the window size to define an x,y-grid of ROIs, enable robust, transparent, and quantitative results on individual, comparative data sets. The most important tuning parameter is the signal-to-noise-ratio (SNR). The SNR defines the stringency of the detection tool. Analysis of our imaging data on cultured neurons with synthetic fluorescent calcium indicators (OGB1) shows that an SNR of 1.5 to 2.5 is useful in finding activity events close to the noise level. SNR values of 3 to 4 focus on calcium transients with higher amplitude, and tend to discard ‘smaller’ transient-like signals. The quite short computing time in combination with the flexibility of the tool makes it easy to adapt the parameters for personal use.

### Signal fluctuations and activity detection

When we analyzed calcium imaging data from neurites of hippocampal neurons, it was obvious that some traces exhibited more and higher signal variations than other neuronal loci. For simplicity, we term this common phenomenon as signal fluctuation. To describe signal fluctuations, we calculated the variance in a sliding window of 30 frames, and found that fluctuations in the calcium signal became smaller when we blocked calcium spikes. The tool can be tuned to use longer or shorter sliding windows. In loci with intensive signal fluctuations, the wavelet analysis found a certain number of activity events. We therefore suggest using two signal trace features to compare neuronal activity mediated by signals close to the noise level: (1.) the number of total activity events in a grid window, and (2.) the variance area in a sliding window of a calcium trace. As shown in Fig 10, even activity caused by homeostatic calcium fluxes can be described by the computed activity number and variance area in a signal trace. Both values are ideally taken from a dataset before and after pharmacological treatment, or from a wildtype neuron in comparison to genetically modified neurons, or can be determined in comparison to a control region in the same image series.

### ROI definition versus a grid of ROIs

Powerful strategies to extract cellular calcium signals are based on multiplication of two matrices to encode the spatial and temporal signal information [18,23,47]. A critical problem with this is to identify the spatial footprint of activity loci, meaning how to select the region of interest (ROI) or image segment for the subsequent calcium signal analysis. Independent component analysis (ICA) for matrix factorization [18,21], non-negative matrix factorization (NMF) [47], and constrained non-negative matrix factorization (CNMF) are powerful methods for extracting cells’ location and automated signal extraction. To target the problem of overlapping loci of activity, CNMF was shown to be eminently powerful [23]. While these strategies are all excellent in defining somatic calcium events and global activity of neurons, the tools may disregard small and local calcium events, which happen outside of the computed image component or ROI or have different time signatures. Furthermore, to better define calcium spikes, the true calcium signal traces are commonly ‘smoothed’ and therefore activity events close to the measurement noise may be ignored, or defined as noise [18,21,23]. We found that CNMF can be powerful to extract homeostatic signals from neuronal segments (S9 Fig). However, new tuning criteria may be needed for improved separation of local activity from background signals. Maybe a grid strategy would be useful to support this analysis, thus simply separating a neuron in unbiased subcompartments. Furthermore, an event definition and a counter would be helpful to offer a natural number representing calcium activity.

In our approach here, we intended to go one step back and to pattern the x,y-field with a pixel-wise grid, to avoid trace smoothing, and to paint an activity pattern over the x,y-grid field, thus making the activity detection independent of a user- or computation-based ROI definition. The user gets an idea of how activity is distributed over the image field and gets new information about local calcium activity events. Theoretically, it is a disadvantage that the locus of analysis is not drawn by the underlying neuronal morphology and therefore only partially fits the underlying structure. It is also a fact that the SNR of the extracted signal in the grid is not ideal, since the temporal traces are extracted not only from pixels belonging to one compartment, but also from pixels from another structure in the same grid window. The grid idea also causes that the same activity event, e.g. a spike, is counted in many grid windows if the activity spreads over a region bigger than the grid size. However, for this tool and our research question it is a powerful and robust strategy to identify activity loci and to count a computed activity event. This is because spontaneous activity is astonishingly diverse, occurs at very small loci, and shows signals which do not appear in the close neighborhood. These events reflect microdomain activity. The grid concept is a simple way to compute total activity patterns of all loci. The output image creates an activity image, which rebuilds the gross structure of the underlying activity centers (e.g. Figs 3, 4 and 9). The implemented ROI tool and the window size parameters allow pixel-wise re-analysis of image segments. The simplistic concept makes the tool user-friendly and robust.

Calcium transient identification can be computed with different algorithms and wavelet-based detection methods are useful for peak detection, even though variants of the algorithm produce a high false positive rate and introduce small phase-shifts [21], while other algorithms are well suited to reduce the false-positive rate [20,48]. We show by analyzing real data that the computing is quite precise when the tool is well tuned. Knowledge-based activity event detection (template-matching) uses a database of calcium signal waveforms or synthetic datasets and proves whether there is a similarity between a waveform and the more noisy true calcium signal trace [21,25]. We avoided knowledge-based strategies on the basis of pre-defined calcium signal waveforms, as we cannot easily create knowledge-based masks for the non-spiking activity observed in calcium imaging data of spontaneously active neurons or in the homeostatic situation. At the same time our data suggest that template-matching [21] is suited to detect signal-close-to-noise activity by homeostatic calcium fluxes. Future tools might include both strategies and the CWT computing might support the creation of templates for template-matching.

One cannot easily decide whether computed calcium signals close to the noise are ‘real’ signals, or wrongly identified signals. Our tool accepts this problem and decides that it is better to label event candidates, than to ignore them. As already mentioned above, the tool tries to find calcium activity events. It is important to notice that the wavelet transform provides a set of point candidates that could be possibly labeled as activity peaks. However, this labeling depends on the pruning done to the tree formed with the set of candidates. This pruning process is done following the branches on the activity tree and such branches are formed according to the ridges of the wavelet transform. The pruning consists in discarding such points which are not present in more than one scale (meaning that they are just local maxima) and those points that do not have the minimally required SNR level. At the end of the pruning, all the remaining points are tagged as activity peaks.

For the CWT computation, we used a symmetric kernel. For this reason, the asymmetry of spike-induced calcium transients is a disadvantageous condition for our algorithm, not because of the wavelet transform algorithm itself, but because of the selection of the symmetric second derivative Gaussian as the mother wavelet. However, the second derivative Gaussian has the important property that it has an almost zero phase shift. This allows us to mark the point in time of the detected peak. Our results in Figs 3–5 confirm that the symmetric mother wavelet is suited to compute spike-induced calcium signals with high precision.

### Practical considerations

The tool is an open source tool, based on ImageJ and ‘R’, and does not need any commercial, license-protected computing environment. The tool is easily operated. A user manual is available (S1 Manual).

Based on our imaging data, we defined a minimum distance between peaks, which is a distance of five data points. When neurons show high activity rates, one can either increase the speed of image acquisition, or one can test beneficial properties of a specific calcium indicator to improve the temporal resolution or the signal-to-noise ratio [6,49]. In the manual we show how the tool can be tuned to better label higher event frequencies (S1 Manual, Chapter VIII). To compare different experimental conditions, we recommend starting with a bigger grid window, the SAT according to the provided thresholding rule, or close to the black level (see above) and an SNR of 2.5 as the default setting. The grid window and the SNR affect the computing time and for a first assessment of raw data these default settings worked very well in our hands with synthetic high affinity calcium indicators. For instance, using a standard desktop computer, 3,000 images (348 x 260 pixel) are computed for one minute to process the image stack, and for another three minutes to complete the wavelet transform and the generation of the documentation pdf. One thousand images are computed in less than two minutes.

Initially, we normally check the data with an SNR of 2, 2.5, and 3, and a grid window size that is small enough to cover the neurites. A second computation with another SNR value, e.g. to separate high SNR signals and low SNR signals, needs just the resetting of the computed values, the change in the SNR value and the restart of the “***Detect activity***” computing, to get the next signal documentation. This needs some seconds. After a first assessment, it is easy to tune the parameters for the final analysis or a specific image acquisition setup. Small grid windows will offer an intuitive image of the neuronal loci where activity events took place, but at the cost of a longer computing time.

### Typical biological questions for use of the activity detector NA^3^

This tool shifts the focus from spike analysis of cell bodies to local signal-close-to-noise analysis in any area of a neuron. Small and local calcium signals mediate important biological functions [9,12,32,50,51] and may be caused by a multiplicity of signaling mechanisms [1,2,4,52]. The molecules underlying local calcium signals are not well described, but are relevant target factors for potential protective and functionally restorative treatments in psychiatric and neurological disorders. One example is the identification of target factors to treat motoneuron diseases [53–55]. Motoneurons show cell-autonomous spontaneous calcium transients, which appear in an unpredictable spatiotemporal on-off, and high versus low frequency pattern [10,14]. By using calcium imaging and subsequent assessment of spontaneous calcium events, disease-relevant molecular factors have been identified. For instance, the local excitability pattern of motoneurons is triggered by a subthreshold active ion channel, the sodium channel Na_V_1.9 [32,56], and mediated by the N-type calcium channel Ca_V_2.2 [14]. This excitability signal cascade is disturbed in motoneurons from mouse models for spinal muscular atrophy [10,14,32,54], the most common genetic cause for infant mortality [57]. Screening-like approaches based on calcium imaging and automated excitability analysis with bioinformatics may offer new information on the role of these genetic factors in motoneurons and patient-derived induced neurons.

Furthermore, not much is known about the role of the excitability factors in neurons of the brain or within neural networks, which are triggering neural network oscillations [58,59]. Locally acting signaling factors such as neuropeptides, neurotrophins, or the contribution of subthreshold voltage changes on excitability are not well integrated in a functional concept of synaptic communication [60]. Furthermore, local tuning and scaling of excitability by calcium-dependent pathways plays an important role in synaptic development [9], and one has to consider that the neuronal excitability is also affected by homeostatic calcium fluxes at rest, which are maintained by a store-operated calcium entry mechanism [30,43,44].

### Conclusion

The software tool presented here operates on a wide range of neuronal activity detection tasks including very different signal intensities and assesses a broad visual field. It is able to offer an unbiased analysis of spontaneous and local neuronal activity. As it is not focused on calcium spikes or a computed ROI, it is able to offer initial insights into the total calcium activity pattern under different experimental conditions. The idea is to use an unbiased x,y-grid, to include all pixels in a imaging video and to offer an activity map. The tool avoids a preferential look at one activity mode of a neuron, e.g. the spiking behavior of neuronal somata, but instead tries to find any activity by detecting signals-close-to-noise.

## Materials and methods

### Primary hippocampal neurons

The animal welfare committee of the University of Würzburg, in accordance with European Union guidelines, approved all experimental procedures. Hippocampal neurons were prepared from CD1 mice of either sex, as described earlier [39]. Hippocampi of newborn mice were collected in Hank’s buffered saline solution (HBSS). Trypsin (Worthington) was added to a final concentration of 0.1% and the tissue incubated for 15 min at 37°C. The protease digestion was stopped with 0.1% Trypsin inhibitor (Sigma). After four steps of trituration in Neurobasal/B27 medium (Life Technologies), cells were plated on poly-L-lysine-coated glass coverslips in Neurobasal, 1× B27, 0.5% penicillin/streptomycin, 1% Glutamax, and 1× N2 supplement (all Life Technologies) and cultured at 37°C under an atmosphere of 5% CO_2_. Calcium imaging experiments were performed after indicated days in vitro (DIV).

### Primary motoneurons

Primary motoneurons were prepared from spinal cord [61]. The lumbar spinal cord of mouse embryos was dissected at embryonic day 13 or 14. Motoneurons were enriched by affinity-panning with antibodies against the p75^NTR^ receptor, and plated at a density of 1,000–2,000 cells on 10mm glass coverslips coated with polyornithine and laminin-1. Motoneurons were grown in Neurobasal/B27 medium (Life Technologies), 2% horse serum, 10 nM β-mercaptoethanol, and 1x GlutaMax. The neurotrophic factors BDNF and CNTF were added at a concentration of 5ng/ml. One day after motoneuron isolation, 40% of the medium was replaced. Calcium imaging was performed at DIV 3.

### Calcium imaging

Calcium indicator dye loading and Ca^2+^ imaging was performed in artificial cerebral spinal fluid (ACSF). For motoneuron imaging ACSF contained (in mM): 127 NaCl, 3 KCl, 2.5 NaH_2_PO_4_, 2 CaCl_2_, 1 MgCl_2_, 23 NaHCO_3_ and 25 D-glucose, bubbled with 95% O_2_/5% CO_2_. Hippocampal neurons were imaged under continuous perfusion with (in mM): 135 NaCl, 6 KCl, 1 MgCl_2_, 2 CaCl_2_, 5.5 D-glucose, 10 HEPES). For chemical LTP stimulation the following buffer composition was used: 128 NaCl, 13 KCl, 3 CaCl2, 5.5 D-glucose, 10 HEPES, 0.1 glycine (in mM). Calcium-free ACSF contained 0.1 mM EGTA. The calcium indicator Oregon Green 488 BAPTA-1, AM (OGB; Invitrogen) was prepared as 5mM stock solution in 20% Pluronic F-127 / DMSO (Invitrogen). For dye loading, 0.5μl of the OGB/Pluronic mixture was mixed in 500μl of ACSF and neurons were labeled for 15 minutes at 37°C and 5% CO_2_. Changes in OGB/calcium-fluorescence were monitored with the help of an upright microscope (BXWI, Olympus, objective: Olympus 40x LUMPlanFI/IR, 0.8 W), in a heated imaging chamber (Luigs & Neumann). Imaging was performed under continuous perfusion with ACSF. Images (8-bit) were captured at the indicated speed in a streaming approach, with a Rolera-XR camera (Qimaging) and StreamPix 4 software (Norpix) under continuous illumination with a 470nm LED light source (Visitron Systems). Fluorescence filters with the following parameters were used: excitation: 482 ± 35 nm; dichroic filter 506nm, emission filter 536 ± 40nm. The health status of the neurons is routinely tested by patch-clamp techniques, control stimuli, and washout controls.

### Simultaneous whole-cell patch clamp recordings and calcium imaging

Current clamp recordings were performed in *whole-cell* configuration. Pipettes with 2.5–5 MΩ resistances were pulled from borosilicate glass (GB 150-8P, Science Products) with a P-97 micropipette puller (Sutter Instruments). Data were acquired using a HEKA EPC-10 USB patch-clamp amplifier controlled by the PatchMaster software (HEKA Electronic) at 25°C. Raw data were continuously sampled at a frequency of 5 kHz and filtered at 2.9 kHz. Electrophysiological experiments with hippocampal neurons were performed with calcium imaging buffer as an external solution. For a steady flow of the external solution, a Minipuls 3 Peristaltic Pump (Gilson) was used. The internal solution contained 148 mM potassium gluconate, 10 mM HEPES, 10 mM NaCl, 0.5 mM MgCl_2_, 4 mM Mg-ATP, 0.4 mM Na_3_-GTP (pH 7.3 with KOH) and Oregon Green^™^ 488 BAPTA-1 hexa potassium salt (2 μM). In *whole-cell* configuration, 200 pA current injections were given for either 500 ms, 200 ms, 100 ms or 10 ms for 12 times at intervals of 10 s. Calcium imaging and patch clamp recording was synchronized via TTL (transistor-transistor-logic) signals and controlled with the help of a TTL-induced 10 ms light pulse (Thorlabs, T-cube LED driver LEDD1B). The light signal became visible in a single frame of the image series. Spontaneous activity was measured in the whole-cell current clamp configuration. Parallel calcium imaging was performed with a sample rate of 10 Hz. In some experiments, the calcium indicator loading was done with estered OGB, as described above.

### Neuronal activity detection tool

To compute calcium transients from raw image material the method was split into two stages. Signal extraction was computed on ImageJ [38] and activity events were calculated on ‘R’ (https://www.r-project.org). Both computations were embedded in the Bio7 environment, an open platform (http://bio7.org/). We call the presented application **NA**^**3**^ (spoken: NA cubic). It is a powerful software tool, combining different routines and languages for optimal neuronal activity detection. The open source tool consists of around 800 lines of code and the core functionality is about 140 lines long. The application was developed on a Windows X64 Intel core i-7 machine with 16 Gigabyte of RAM memory. For time series analysis, a Windows X64 Intel core i-5 machine with 4 Gigabyte of RAM memory was used. The tool is available on: https://github.com/jpits30/NeuronActivityTool. The tool containing the ROI feature is available at: https://github.com/jpits30/NeuronActivityTool_ROI.

### Wavelet transform algorithm

The intensity signals are analyzed in order to detect the peaks that correspond to neuronal activity. The event detection uses a modified wavelet transform algorithm for peak detection [20,48]. For a more detailed explanation refer to this reference [62]. One advantage of the wavelet transform over other spectral analysis techniques, like the Fourier analysis, is its multiscale feature. The mother wavelet is scaled. Therefore, it can fit peaks of different sizes. Our algorithm sums the information obtained from matching the calcium activity peak with several scaled versions of the mother wavelet and decides based on that whether that part of the trace contains a peak of activity or not.

The wavelet transform constructs a signal representation that changes with time and space. In order to construct such representation the signal is convolved with a pattern signal called mother wavelet. The mother wavelet is normalized such that ∥Ψ∥ = 1. Using the mother wavelet Ψ, the wavelet transformation of a one dimensional function *x*(*t*) is defined in the equation (Eq 1).

$$X\left(a,b\right)=\frac{1}{\sqrt{a}}\int_{-\infty }^{\infty }\Psi^{*}\left(\frac{t-b}{a}\right)x\left(t\right)dt$$ (1)

*a* denotes the scale parameter of the wavelet, *b* is the time shift parameter. The wavelet transformation can be understood as a matching procedure between the original signal and the mother wavelet, so with the objective of peak detection, the mother wavelet must be a signal with a clear peak and approximate symmetry. This computation uses a second derivative Gaussian as mother wavelet. This mother wavelet is appropriate for peak detection, as it enhances the peak and diminishes the neighboring signal values. The wavelet transform was chosen because the shifting and scaling of the basic function results in the time resolution property for peak detection within a noisy intensity signal, being able to recognize not only spiking neurons but also smaller peaks of calcium activity. The wavelet transformation maps the peak search into a search of ridges on the time-scale space, this space is smoother and easier to characterize. The wavelet transformation also enables that removal of the base line is not required because it is done intrinsically.

We suppose, with respect to earlier studies [20], that the calcium trace in the vicinity of a peak is composed of three components, the peak (p(t)), the base line (b(t)) and the noise (N) as shown in equation (Eq 2)

$$x_{cal}(t)=p(t)+b(t)+N$$ (2)

Then the wavelet transform of such signals would be (Eq 3)

$$Xcal\left(a,b\right)=\frac{1}{\sqrt{a}}\left(\int_{-\infty }^{\infty }\Psi^{*}\left(\frac{t-b}{a}\right)p\left(t\right)dt+\int_{-\infty }^{\infty }\Psi^{*}\left(\frac{t-b}{a}\right)b\left(t\right)dt+\int_{-\infty }^{\infty }\Psi^{*}\left(\frac{t-b}{a}\right)N\;dt\right)$$ (3)

The second and third integrals will be approximately zero because of the symmetry of the mother wavelet and because of the low level of matching between the base line and a mother wavelet such as the second derivative Gaussian. That means that the only term left is the convolution between the peak and the mother wavelet.

### Strategy for calcium event (peak) identification

As mentioned before, once the original calcium trace is transformed, the peak search problem turns into a problem of finding the ridges of the wavelet transformation and determining which of those ridges corresponds to peaks of interest for us. A ridge is conform with a subset of local maxima, which are nearby in time and belong to consecutive scales [63]. Fig 2a shows how the computation extracts a set of signal peaks to define calcium transient candidates. The wavelet transform has a set of ridges, which are used to construct a tree. Using that tree, the computation detects the peaks in the signal. In the coefficient space of a wavelet transform (Fig 2a), a group of local maxima can be identified. In Fig 2a, the ridges are the brighter yellow areas (almost white in some points) under the blue vertical lines (branches).

The branches of the tree are assembled as follows. Starting at the highest scale (top of Fig 2a), a local maximum within the coefficients is defined. This coefficient is set to be the starting point of a branch of a tree. This branch is elongated by adding it to the rest of the coefficients in the same ridge. This procedure is repeated, starting from the second largest scale and is then repeated until all the local maximum values in the wavelet transform were considered. Some scales need to be evaluated several times, because they contain several local maximum values. This procedure is done by using the *wavCWTTree* function from the *wmtsa* package in R [64]. At the end of this procedure, we have a set of branches, which are further pruned according to the length of the branch.

The pruning of the branches is efficient and is performed as follows. Each branch (the blue vertical lines) extends over some scale values and has a particular length. Some of the branches are discarded if they do not meet certain criteria for their length. If a branch extends over less than one octave of the scale range, then it is discarded. The rest of the branches (those long enough) form the tree that we use to search for the peaks. In our experiments, we used one octave as the threshold. In the user manual, Chapter VIII (S1 Manual), we show how this pruning can be modified by the user.

Next, the tool searches each branch of the tree to find out whether it contains a peak. This search occurs according to two conditions. The first is the signal-to-noise ratio of the peak candidate and the second is whether the peak candidate lies on a scale higher than a certain value. The first condition, the SNR, is the most important and it is used in our algorithm as a tuning parameter. The signal-to-noise ratio is estimated with the help of the coefficients of the wavelet transform. The wavelet coefficients of the lowest scale near the branch are used to estimate the noise power. Similarly, the highest coefficient in the branch represents the power of the peak. Hence, the signal-to-noise ratio of the peak is simply the ratio of the power of the signal to the power of the noise. This signal-to-noise ratio has to be higher than the user-defined SNR; otherwise, the peak is discarded. This can be observed in Fig 2a, where some of the branches end up with an activity event (peak), while other branches are not considered as an activity event. The tool has a border effect of three to five images, where it cannot find activity.

### Variance area calculation

To describe signal fluctuations in a signal trace, the mean of the signal with the corresponding variance is calculated in a sliding window. The number of window frames can be selected by the user. The variance window is visualized as a yellow band (Fig 7), whereas the yellow area is the variance area, describing the mean +/- of the variance, centered on the mean. This variance area is a virtual value to describe changes in signal fluctuations. The variance area value is used to compare two experimental conditions, for instance, the variance of the mean of a calcium imaging signal in a grid window before and after pharmacological treatments.

### General tendency change estimation

The tool also offers the possibility to estimate a change in the general tendency of the signal. Such estimation is done with the help of the *changepoint* package in R. The estimated point of change in the general tendency is the point that splits the signal in the two parts with the most significant statistical difference in average and variance. It means that the signal before the point and after the point are the two most different parts of the signal which can be found, no other cut on the signal would produce a more different pair of signals. This tendency change estimation proves useful, for example when the researcher wants to see the time of action that an inhibitor needs to affect the behavior of cultures neurons.

## Availability and future directions

The application **NA**^**3**^ (NA cubic) is an open source software tool and is available at: https://github.com/jpits30/NeuronActivityTool. The tool containing an additional ROI feature is available at: https://github.com/jpits30/NeuronActivityTool_ROI. Updates and further information will be released at GitHub. Additional information, test data and raw data can be found at: https://www.biozentrum.uni-wuerzburg.de/bioinfo/computing/neuralactivitycubic/. The primary data are useful as standardization data to compute global and local calcium activity, signal-close-to-noise activity, signal components, or can be used to create signal templates for local activity events.

### Outlook and future

We will test the option for automated constrained non-negative matrix factorization (CNMF) to see whether such algorithms will help to better define the signal source. Furthermore, we will test optional asymmetric kernels for CWT computation and, depending on the data, which combination of symmetric and asymmetric kernels is optimal. Numerous other options are available to further develop our tool and new applications will be investigated. Users and developers are invited to develop and test new options for NA^3^ and use our test data and code.

## Supporting information

S1 Fig Output dataset. Computation of a spontaneously active motoneuron; trace and activity counts.
(PDF)

S2 Fig Output dataset. Computation of a spontaneously active motoneuron; text-file with the grid-specific activity counts.
(TXT)

S3 Fig Output dataset. Computation of a motoneuron in the low activity phase.
(PDF)

S4 Fig Tool comparison. Comparison of CWT (our tool, red rhombus), deconvolution (blue dots), template-matching (green dots) and signal significance computation (“Romano toolbox”; magenta line) in a calibration dataset showing simultaneous imaging with loose-seal cell-attached recording in four GCaMP expressing neurons [37]. Black line: Calcium signal trace (raw data), blue vertical lines: spike information. The TIFF format image stacks were taken from: https://crcns.org/data-sets/methods/cai-1/about-cai-1 [37]. This dataset contains raw data information showing the signal behavior of GCamp5k-expressing neurons. The spike-data were extracted from http://spikefinder.codeneuro.org/, dataset 06 [28]. We analyzed four videos (called cells 0, 1, 5, and 8 in the spikefinder training data). The names of the underlying image stack raw data are: cell1005, cell2002, cell1002, and cell4003. Please note that the original dataset contains 24.000 images acquired in two color channels (green and red). The calcium signal is only present in the green channel. Therefore every second image was removed for the computation.
(PDF)

S5 Fig Output dataset. Computation of spontaneously active hippocampal neurons, traces and activity counts.
(PDF)

S6 Fig Output dataset. Computation of hippocampal neurons during stimulation with a solution to induce chemical LTP; traces and activity counts.
(PDF)

S7 Fig Output dataset. Computation of spontaneously active hippocampal neurons; traces, activity counts, variance area. Shown are 12 of 289 pages of data summary.
(PDF)

S8 Fig Washout control. **a**, Hippocampal neurons loaded with OGB1-AM, average intensity projection, brightness and contrast adjusted. **b-d**, Three spontaneous active neurons (ROI 1–3) show typical calcium spike-behavior (fast onset, slower decaying of the signal). The neuronal activity inhibitors for glutamatergic transmission (CNQX, APV) were acutely applied to abruptly inhibit the calcium spiking behavior (upper trace in b–d). Upon washout of the inhibitors, cells recover from activity blockade and show again typical calcium spikes (lower traces in b–d).
(PDF)

S9 Fig Analysis of homeostatic calcium fluxes. Here, we show the performance of a tool that decomposes the spatiotemporal activity of a neuron into a spatial component to show the local neural structure and temporal components that model the local calcium dynamics [23]. This tool identified ten signal components from the movie (Fig 9) and computed five of these components.
(PDF)

S1 Manual Installing instructions and user manual. (PDF)
