# Supplementary material for: An open source tool for automatic spatiotemporal assessment of calcium transients and local ‘signal-close-to-noise’ activity in calcium imaging data
Source: PLoS Comput Biol. 2018 Mar 30;14(3):e1006054. doi: 10.1371/journal.pcbi.1006054 (PMC5895056; doi:10.1371/journal.pcbi.1006054)
Supplement: S1 Fig — Computation of a spontaneously active motoneuron; trace and activity counts. (PDF) [file pcbi.1006054.s001.pdf]

Total activity 613

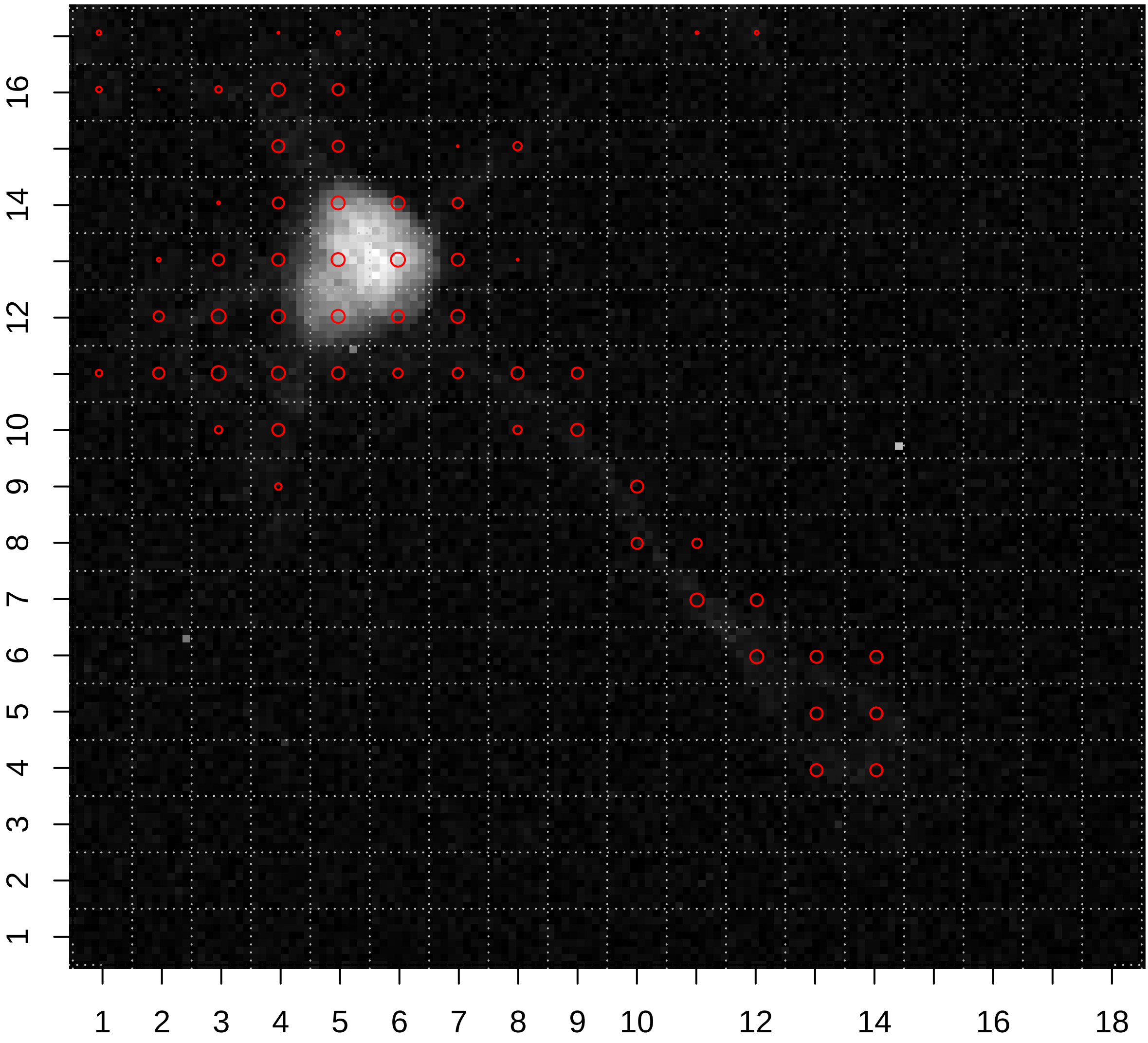

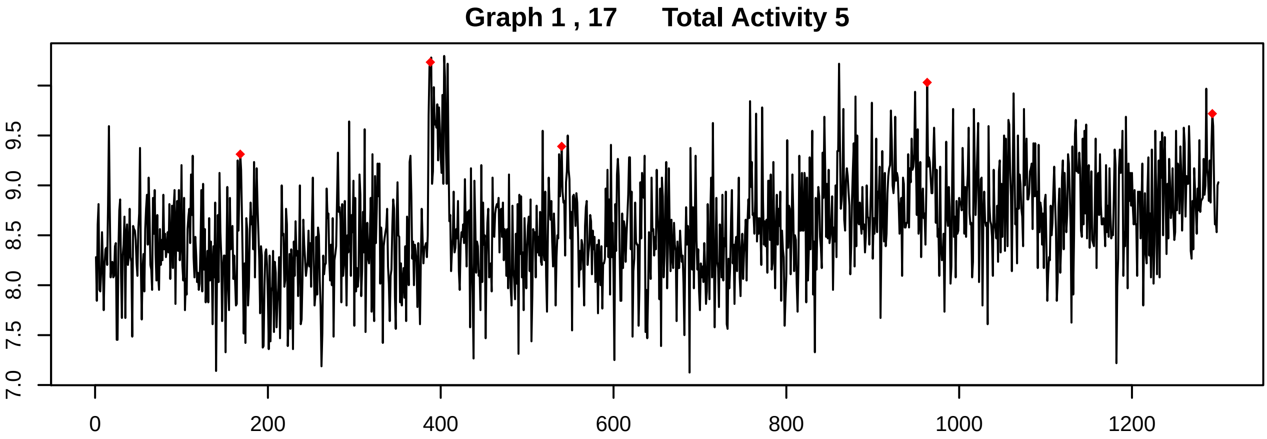
Graph 1 , 17 Total Activity 5
7.0
7.5
8.0
8.5
9.0
9.5
0
200
400
600
800
1000
1200

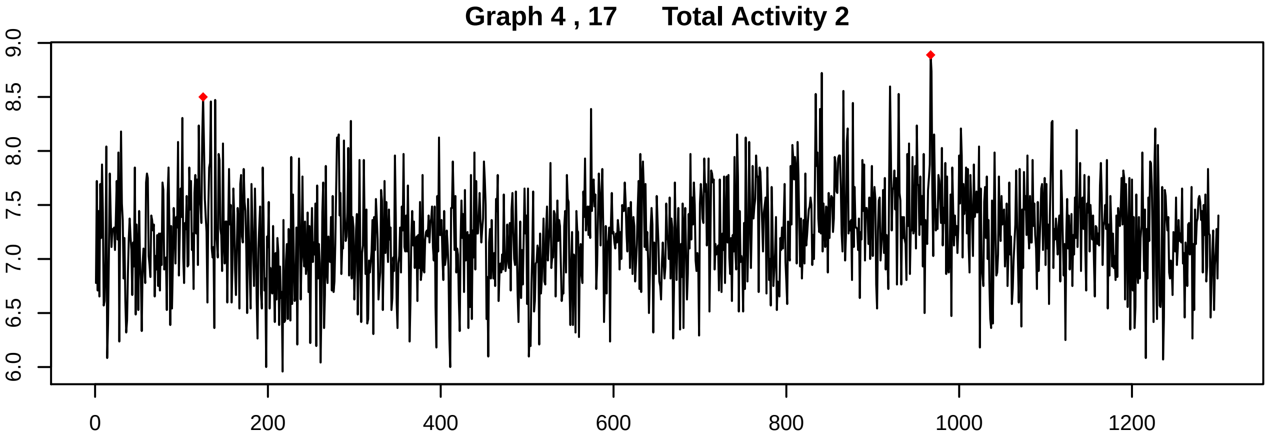
Graph 4 , 17 Total Activity 2
6.0
6.5
7.0
7.5
8.0
8.5
9.0
0
200
400
600
800
1000
1200

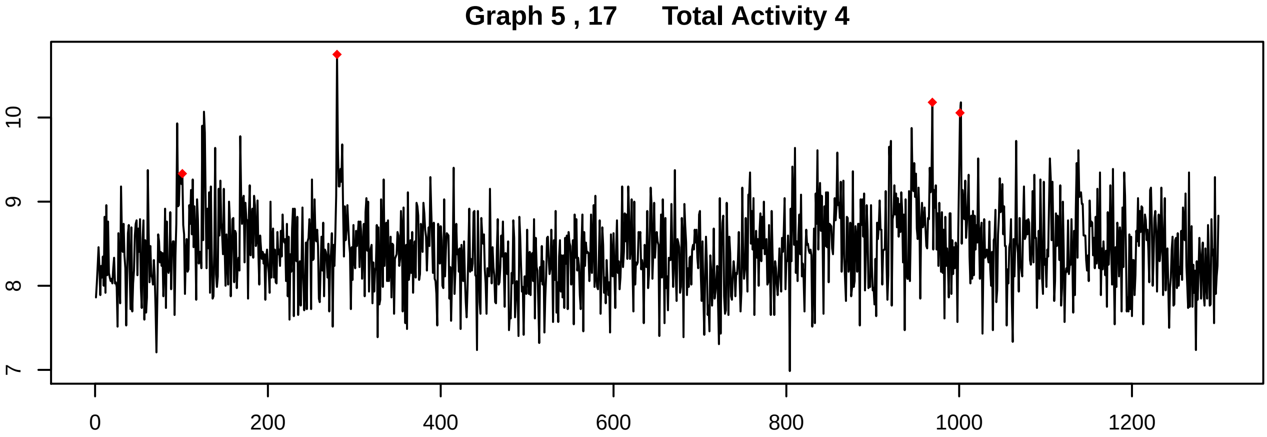
Graph 5 , 17 Total Activity 4
7
8
9
10
0
200
400
600
800
1000
1200

Graph 11 , 17   Total Activity 3

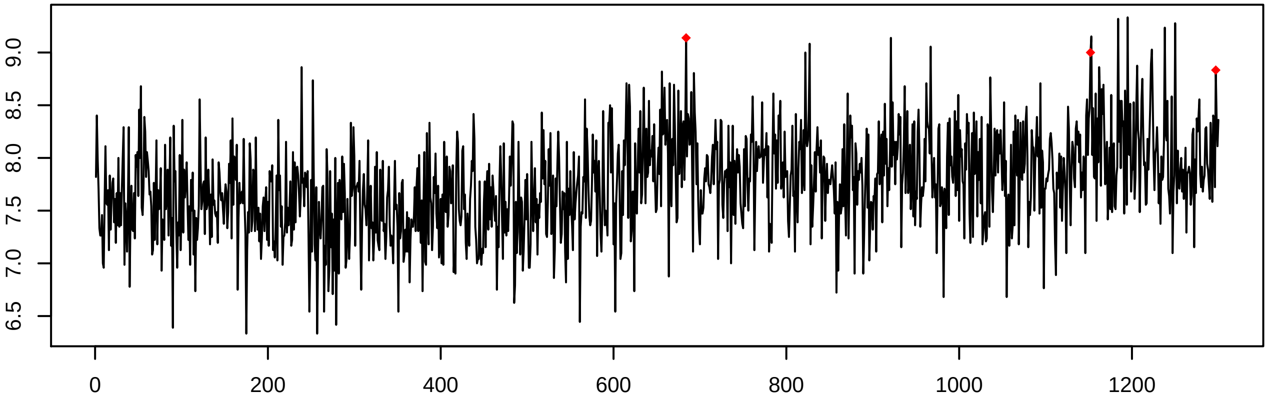

Graph 12 , 17   Total Activity 4

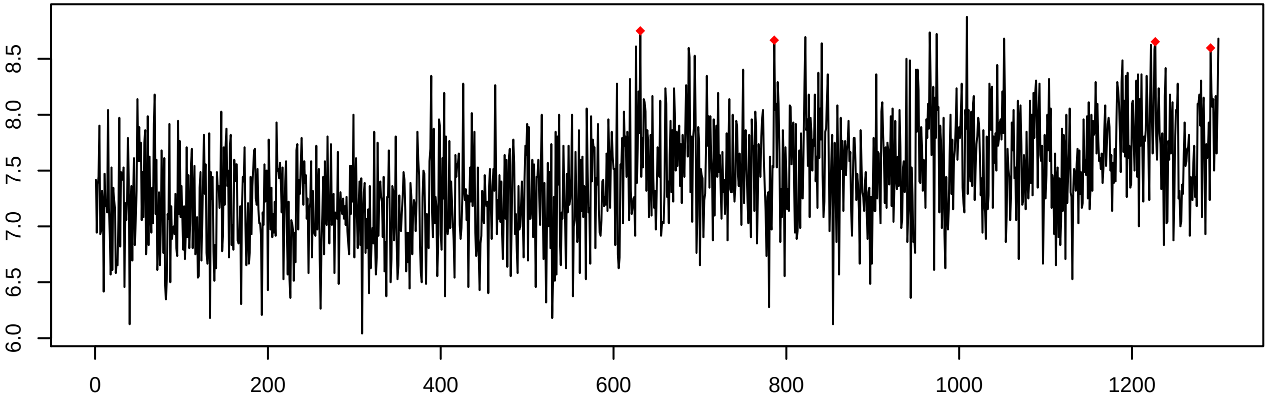

Graph 1 , 16   Total Activity 6

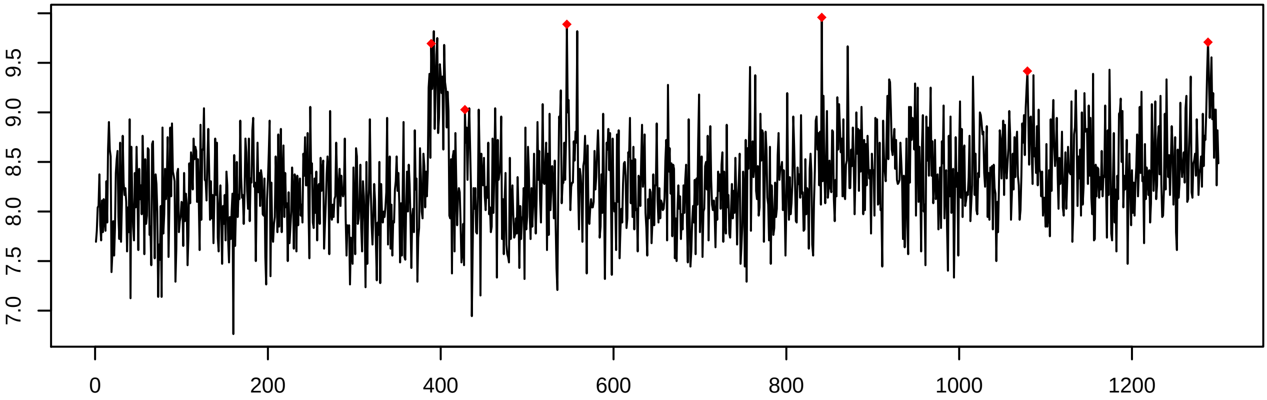

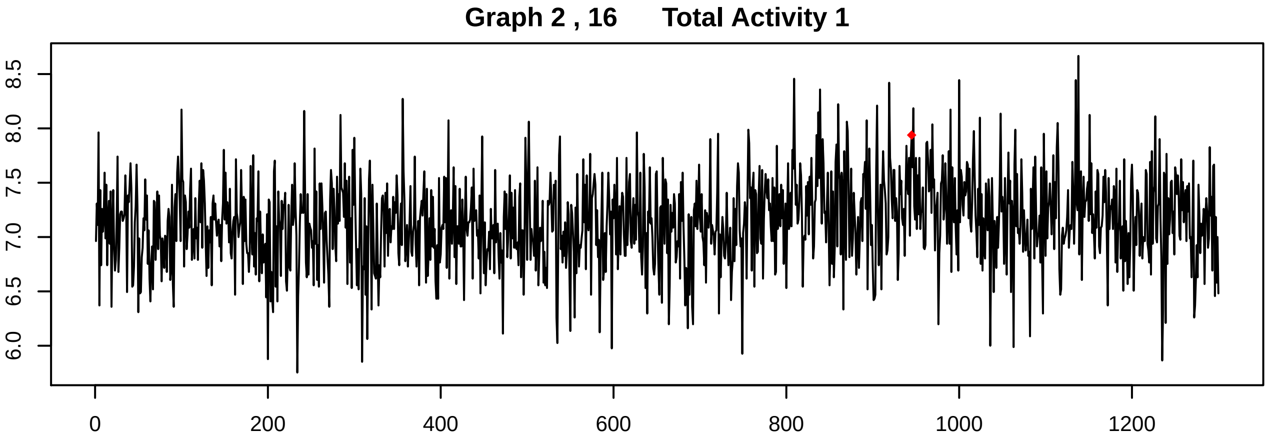
Graph 2 , 16 Total Activity 1
6.0
6.5
7.0
7.5
8.0
8.5
0
200
400
600
800
1000
1200

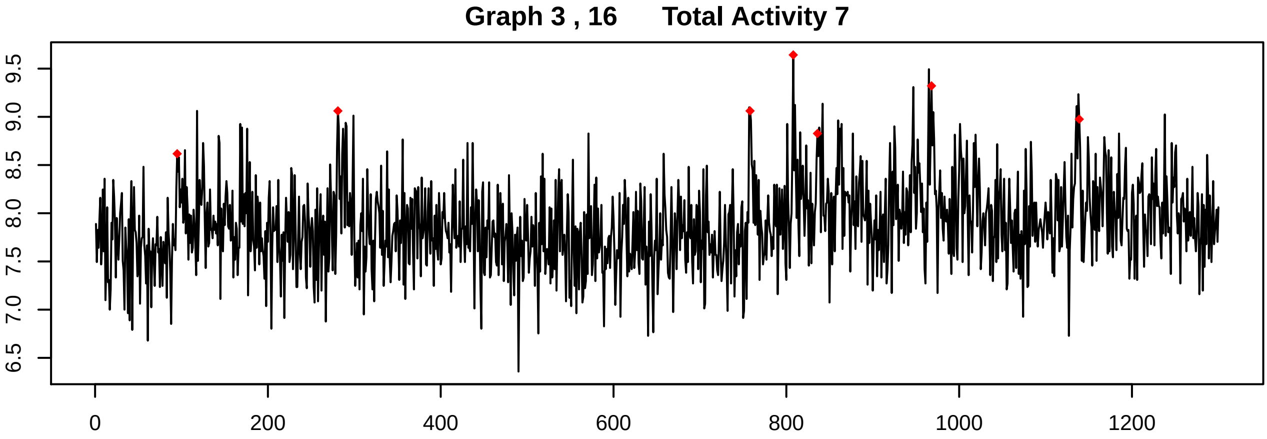
Graph 3 , 16 Total Activity 7
6.5
7.0
7.5
8.0
8.5
9.0
9.5
0
200
400
600
800
1000
1200

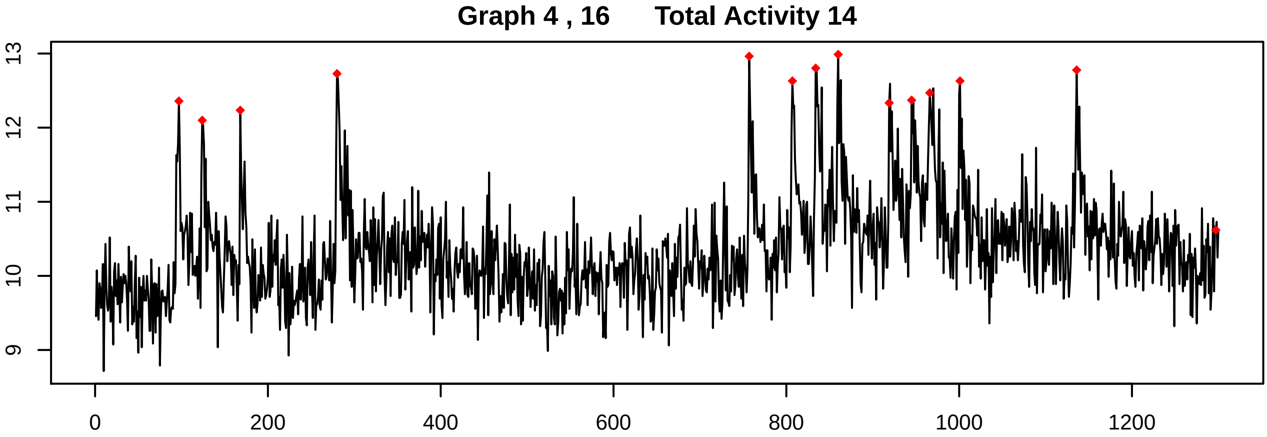
Graph 4 , 16 Total Activity 14
9
10
11
12
13
0
200
400
600
800
1000
1200

Graph 5 , 16   Total Activity 12

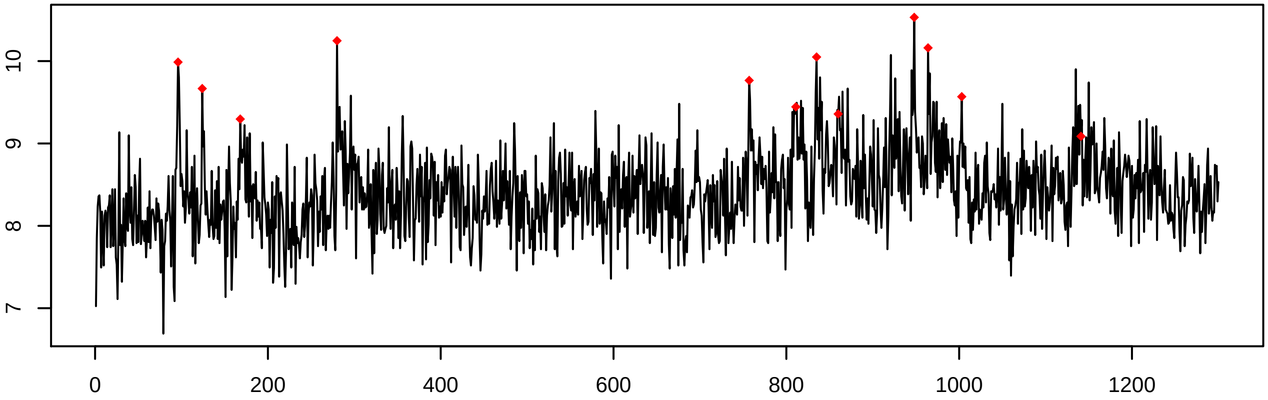

Graph 4 , 15   Total Activity 13

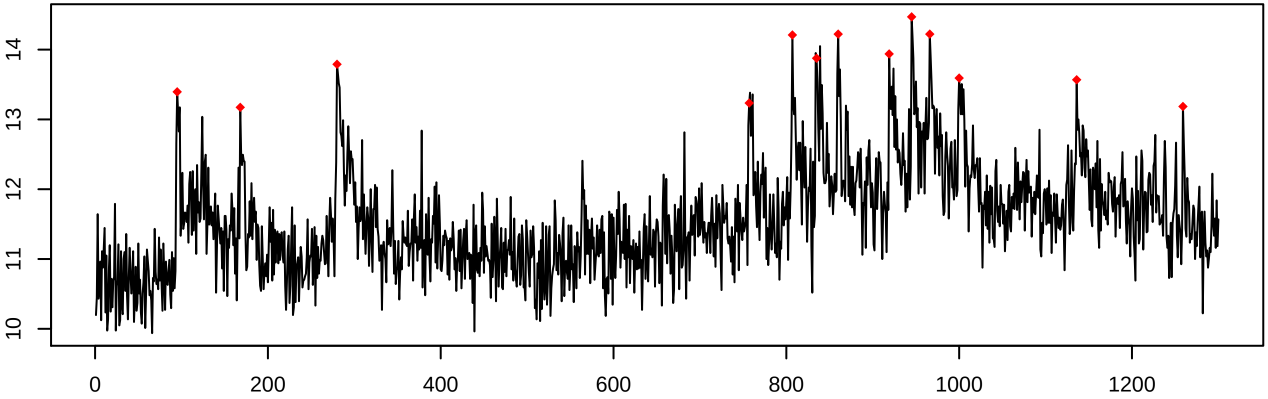

Graph 5 , 15   Total Activity 12

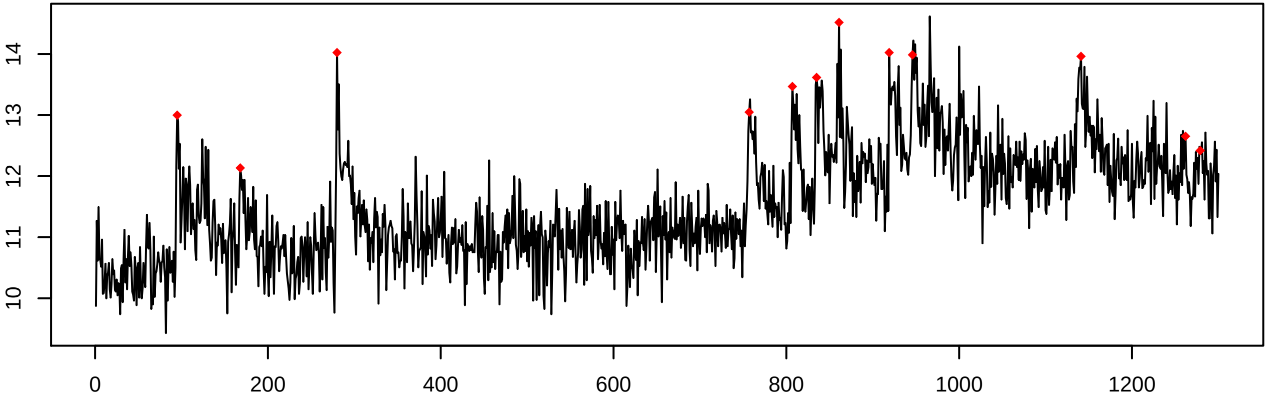

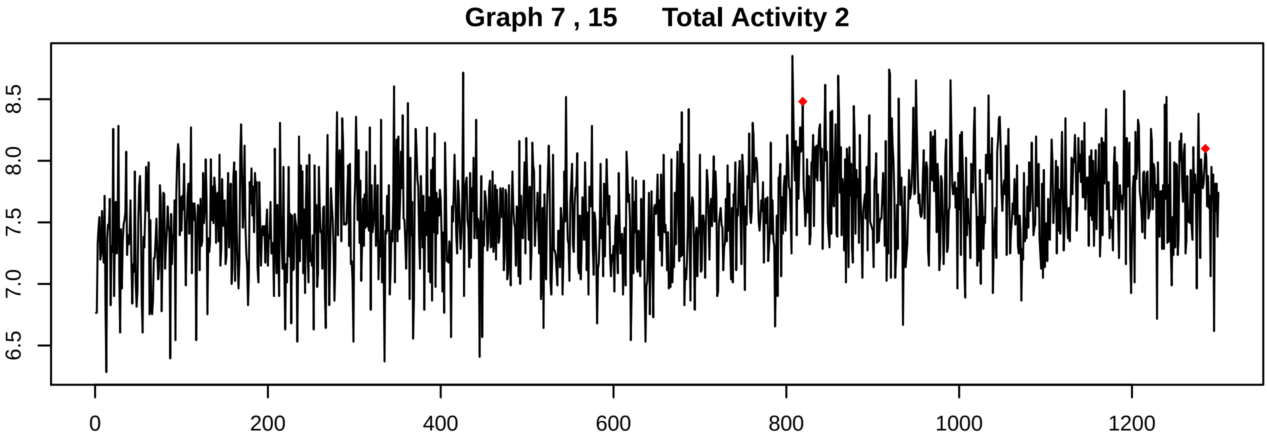
Graph 7 , 15 Total Activity 2
6.5
7.0
7.5
8.0
8.5
0
200
400
600
800
1000
1200

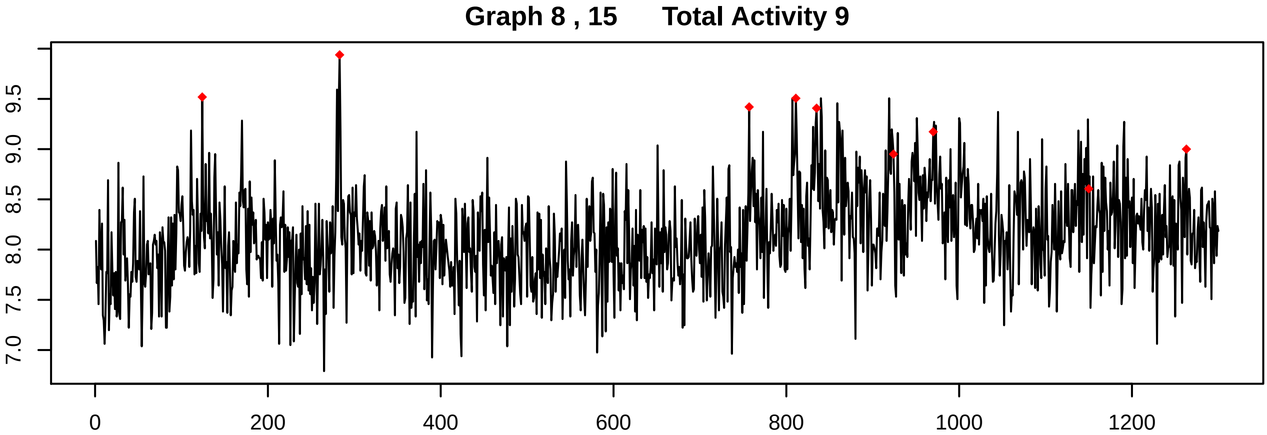
Graph 8 , 15 Total Activity 9
7.0
7.5
8.0
8.5
9.0
9.5
0
200
400
600
800
1000
1200

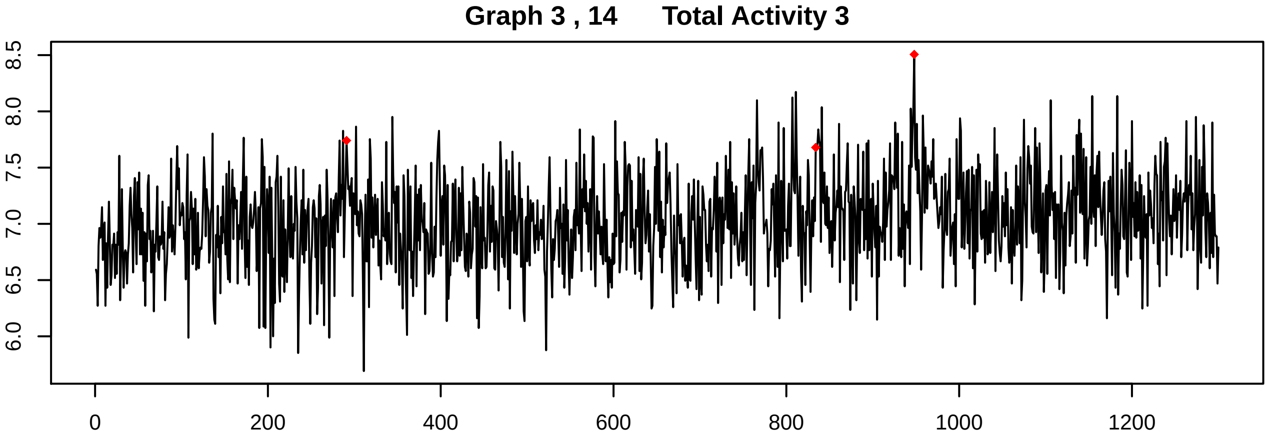
Graph 3 , 14 Total Activity 3
6.0
6.5
7.0
7.5
8.0
8.5
0
200
400
600
800
1000
1200

Graph 4 , 14   Total Activity 12

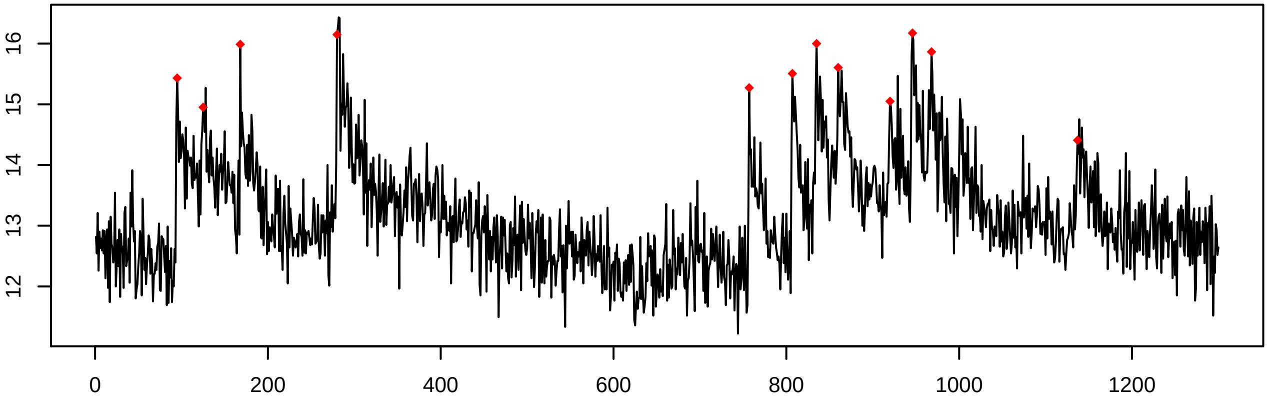

Graph 5 , 14   Total Activity 14

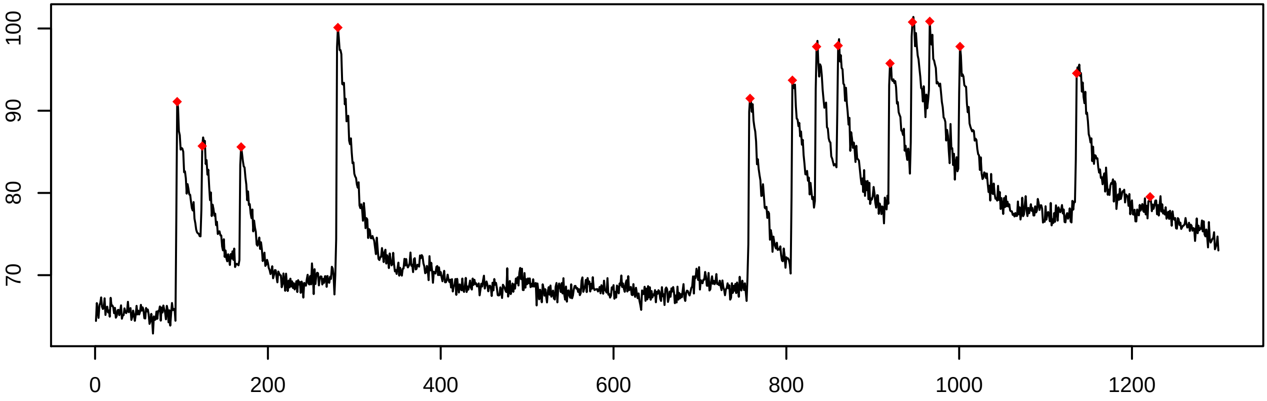

Graph 6 , 14   Total Activity 14

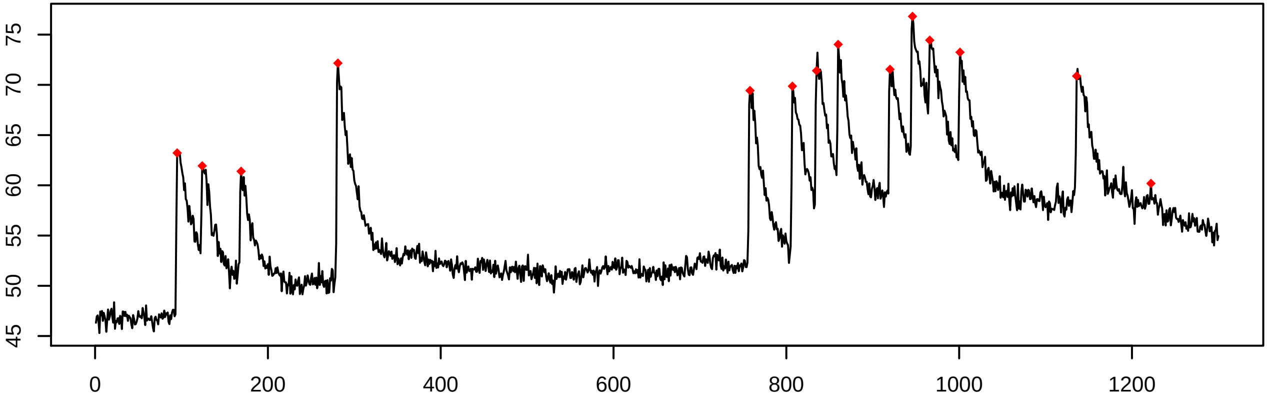

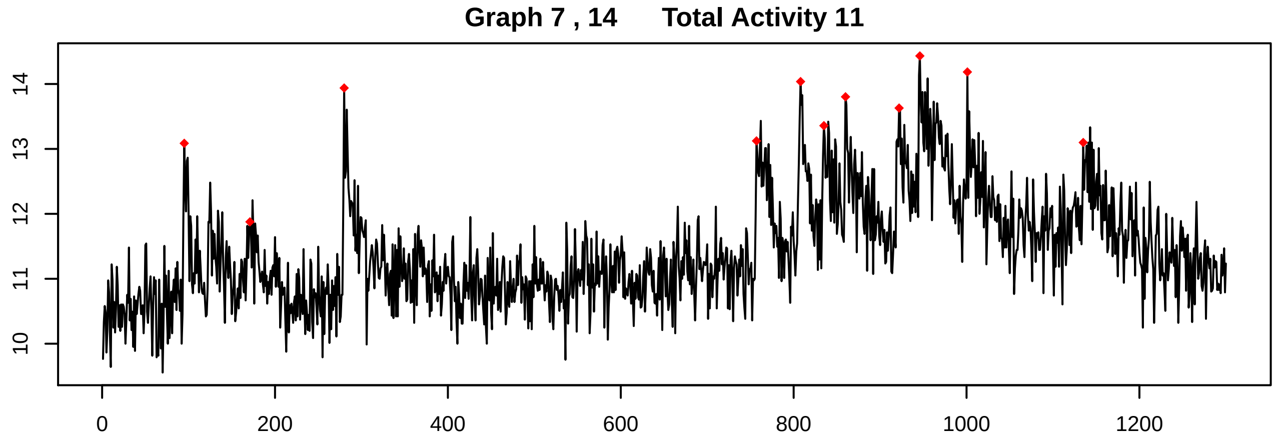
Graph 7 , 14 Total Activity 11
10
11
12
13
14
0
200
400
600
800
1000
1200

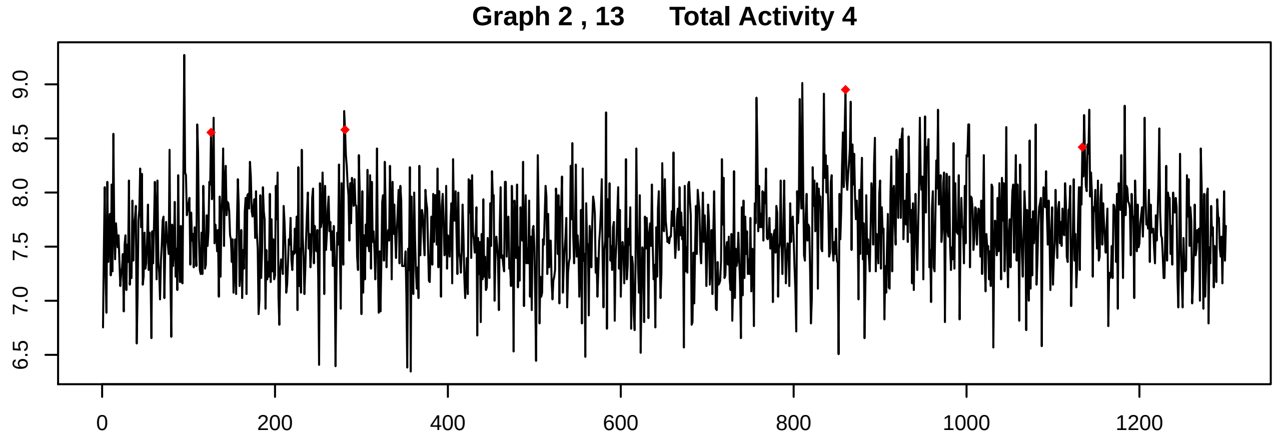
Graph 2 , 13 Total Activity 4
6.5
7.0
7.5
8.0
8.5
9.0
0
200
400
600
800
1000
1200

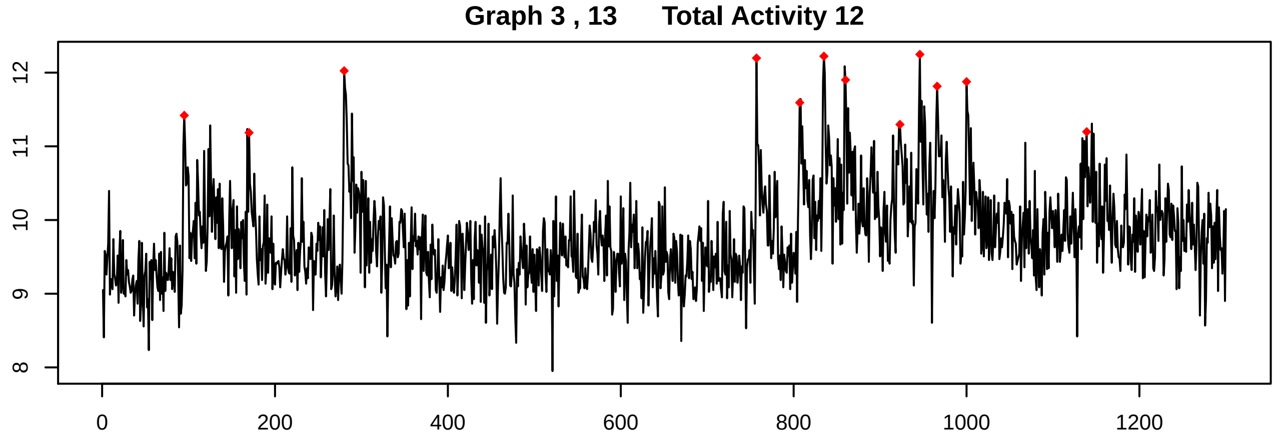
Graph 3 , 13 Total Activity 12
8
9
10
11
12
0
200
400
600
800
1000
1200

Graph 4 , 13   Total Activity 13

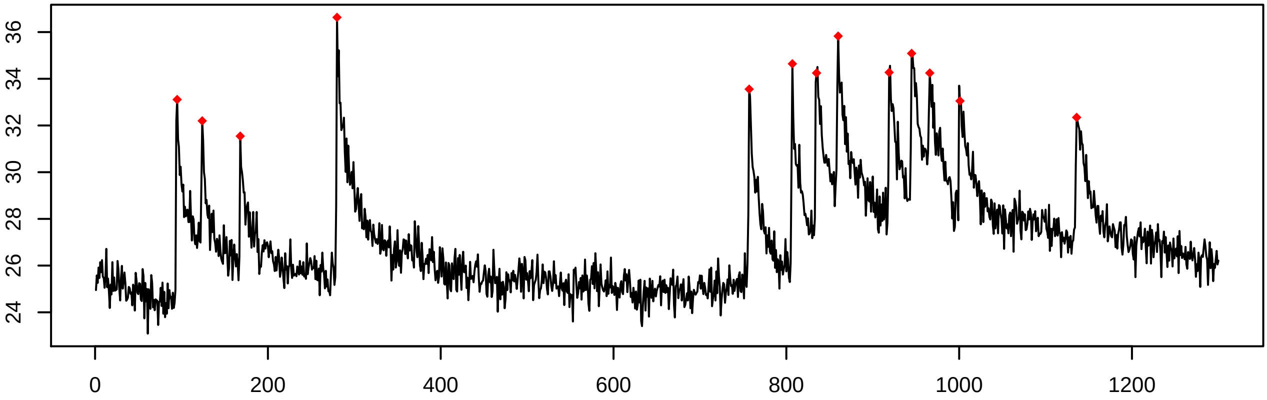

Graph 5 , 13   Total Activity 14

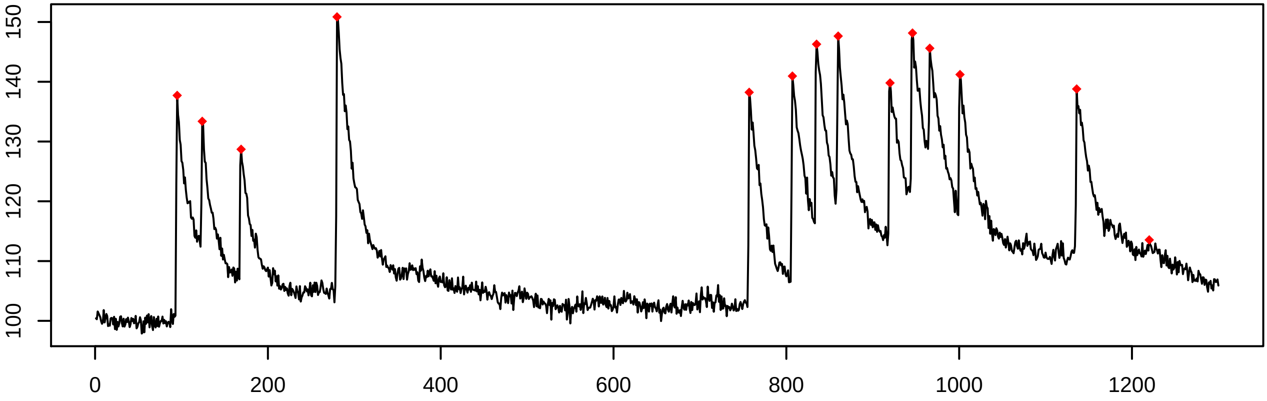

Graph 6 , 13   Total Activity 15

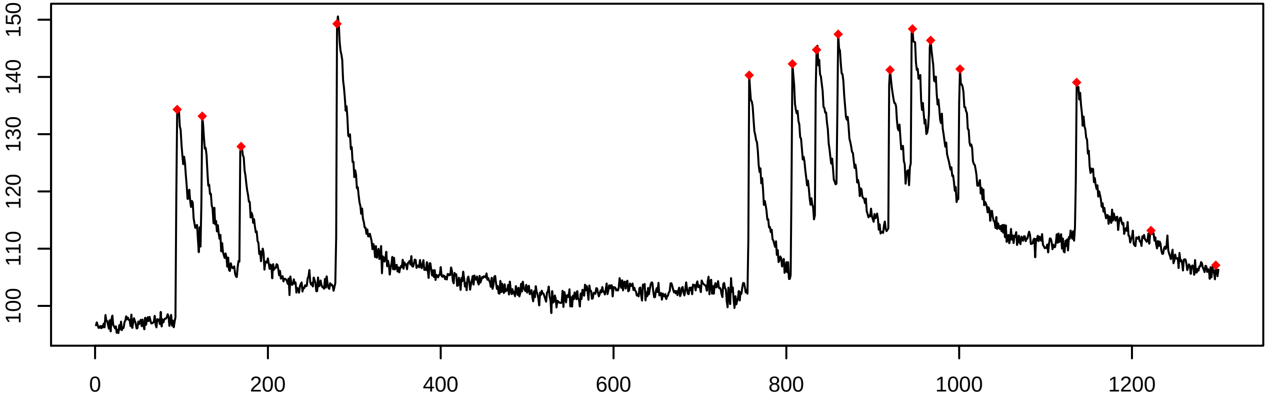

Graph 7 , 13   Total Activity 13

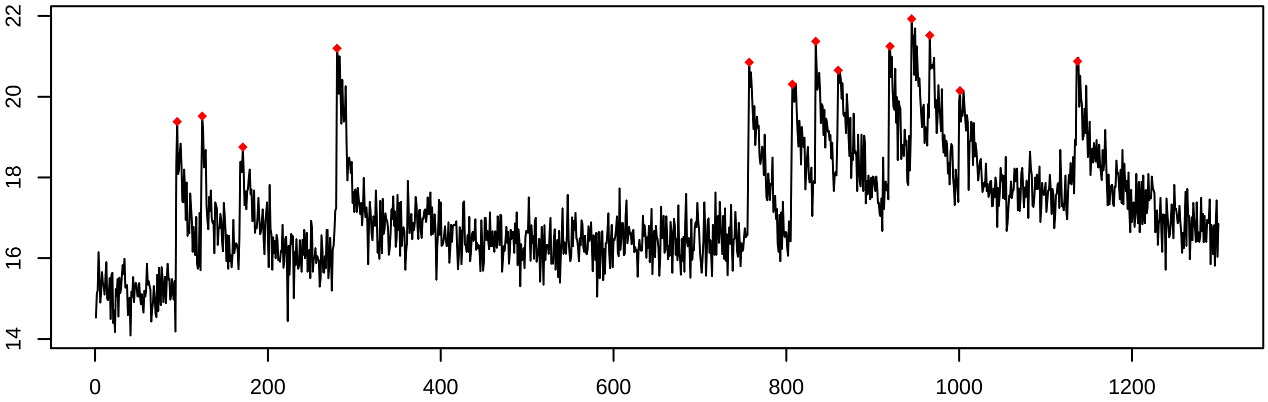

Graph 8 , 13   Total Activity 2

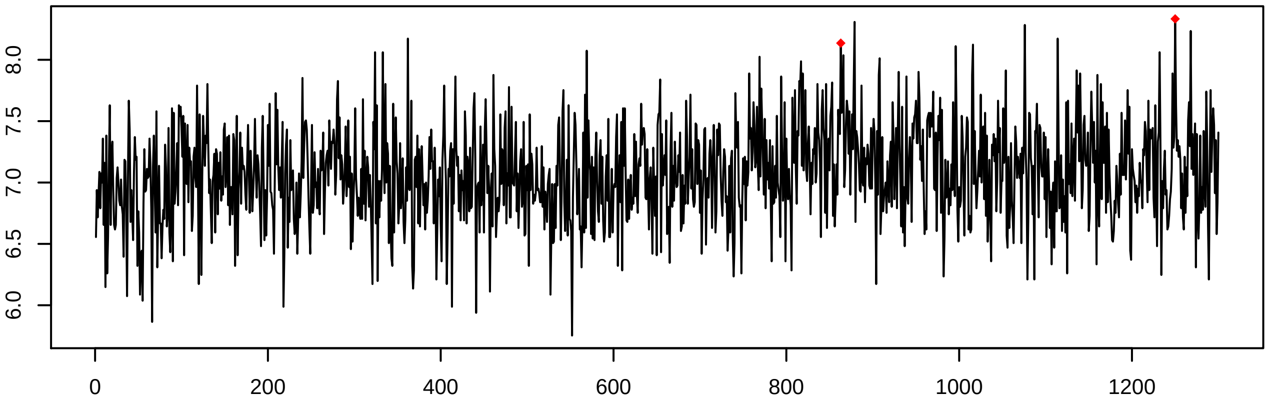

Graph 2 , 12   Total Activity 11

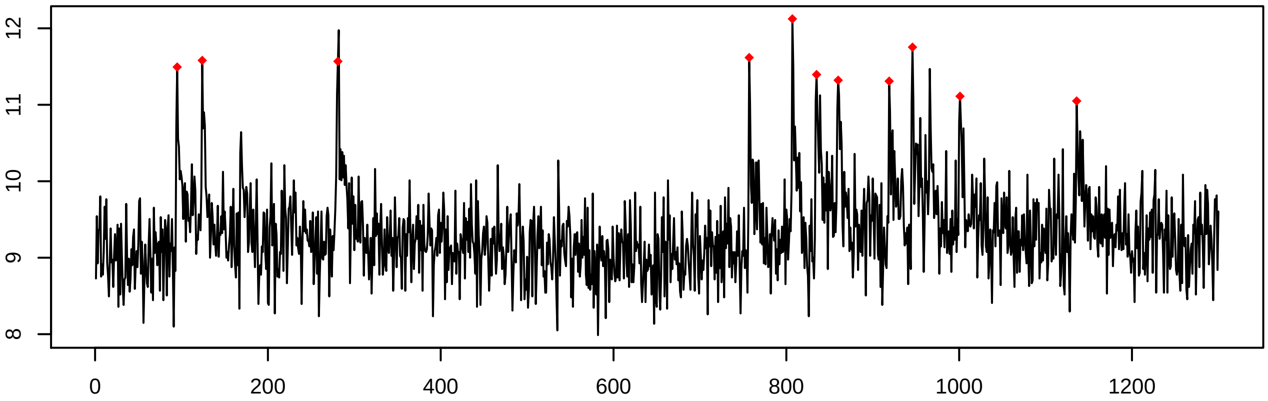

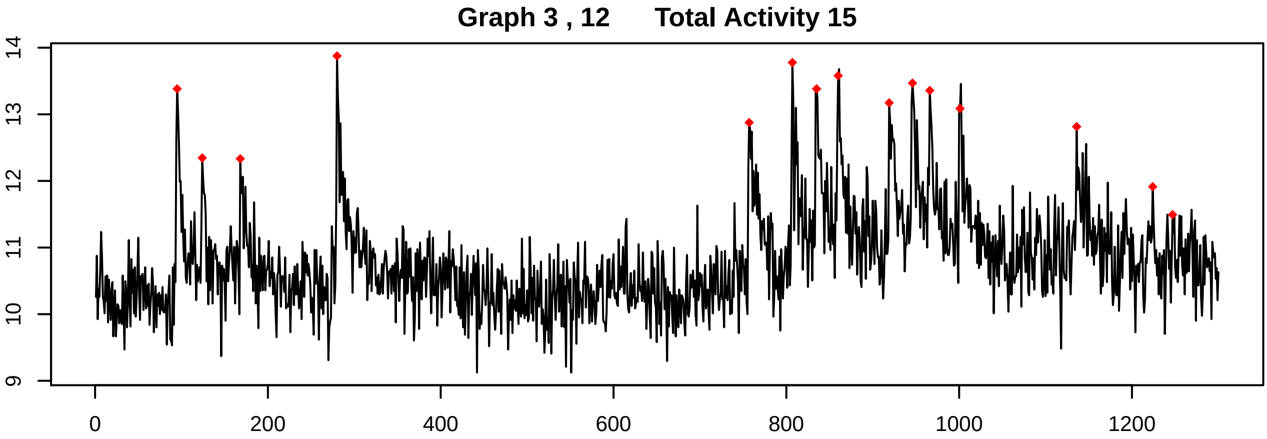
Graph 3 , 12   Total Activity 15
9
10
11
12
13
14
0
200
400
600
800
1000
1200

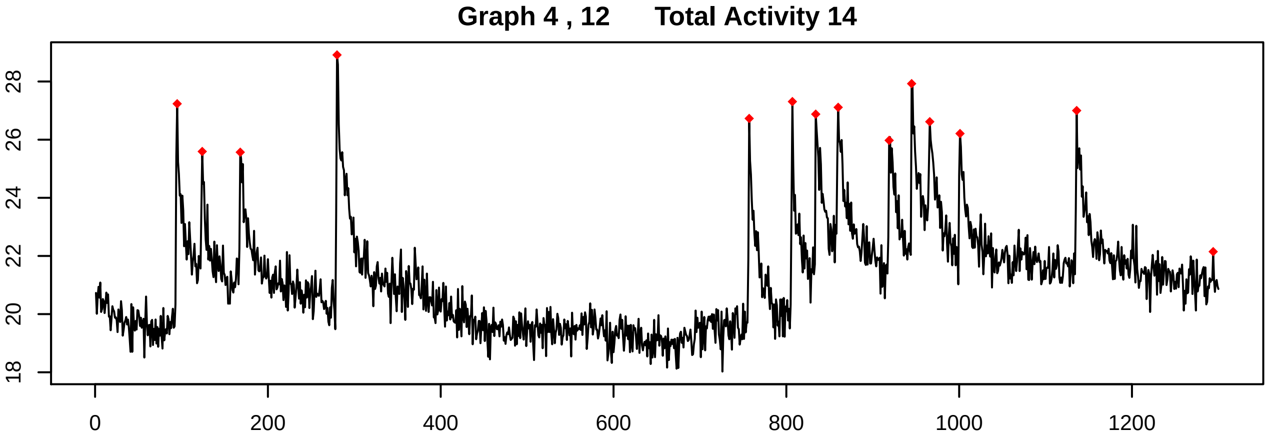
Graph 4 , 12   Total Activity 14
18
20
22
24
26
28
0
200
400
600
800
1000
1200

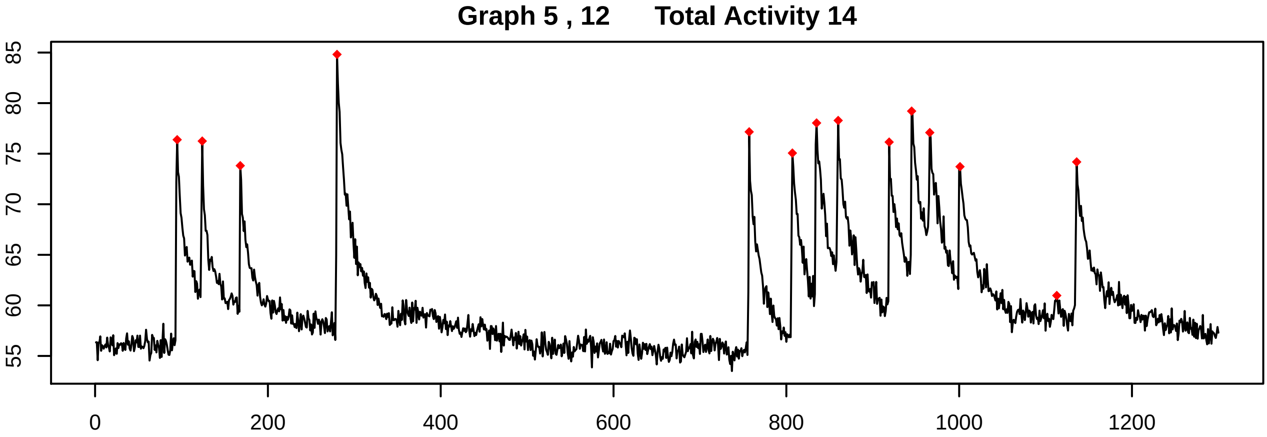
Graph 5 , 12   Total Activity 14
55
60
65
70
75
80
85
0
200
400
600
800
1000
1200

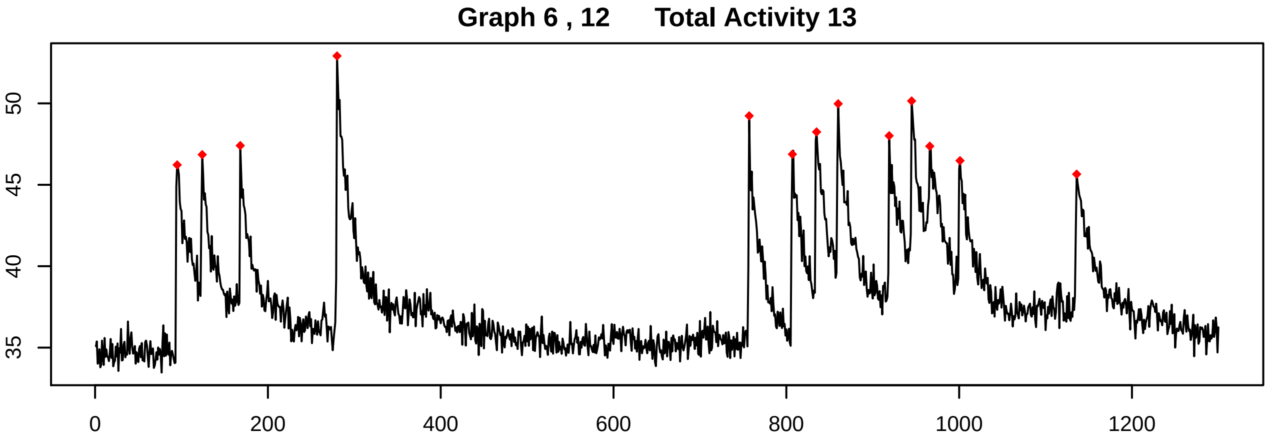
Graph 6 , 12 Total Activity 13
35
40
45
50
0
200
400
600
800
1000
1200

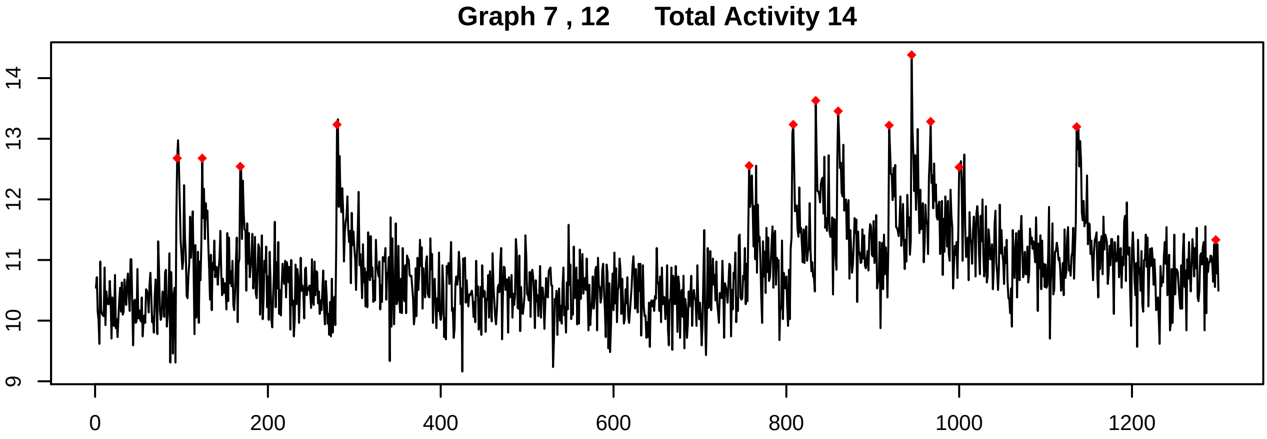
Graph 7 , 12 Total Activity 14
9
10
11
12
13
14
0
200
400
600
800
1000
1200

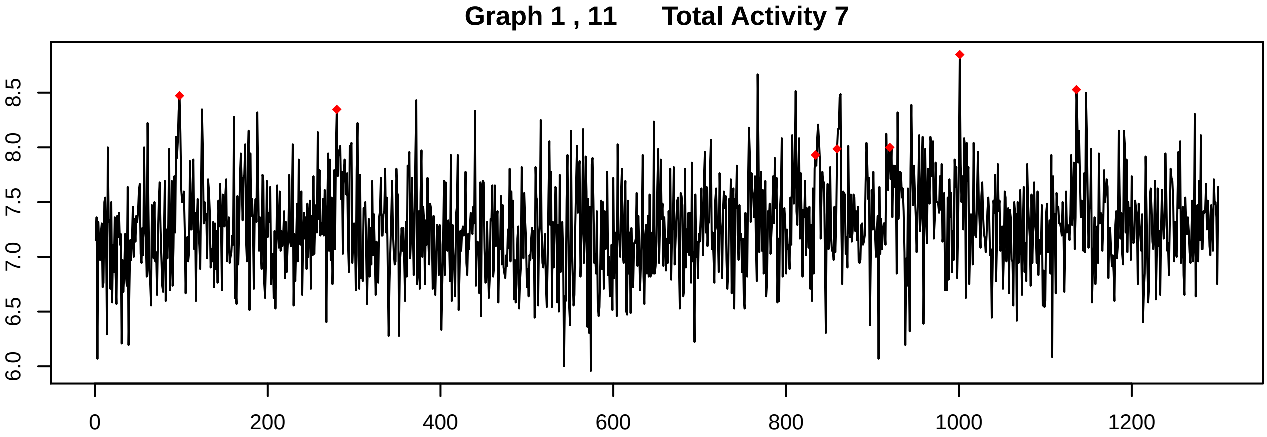
Graph 1 , 11 Total Activity 7
6.0
6.5
7.0
7.5
8.0
8.5
0
200
400
600
800
1000
1200

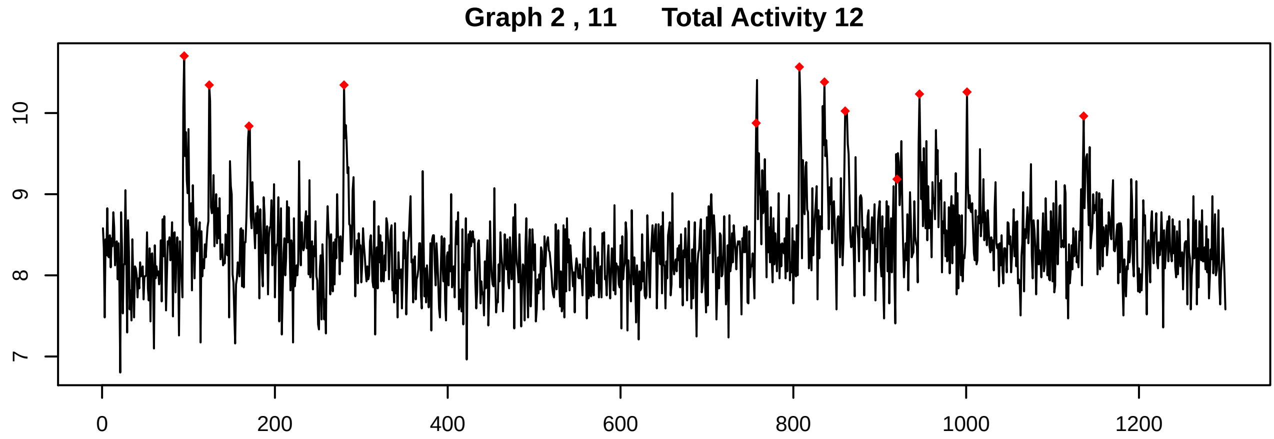
Graph 2 , 11 Total Activity 12
7
8
9
10
0
200
400
600
800
1000
1200

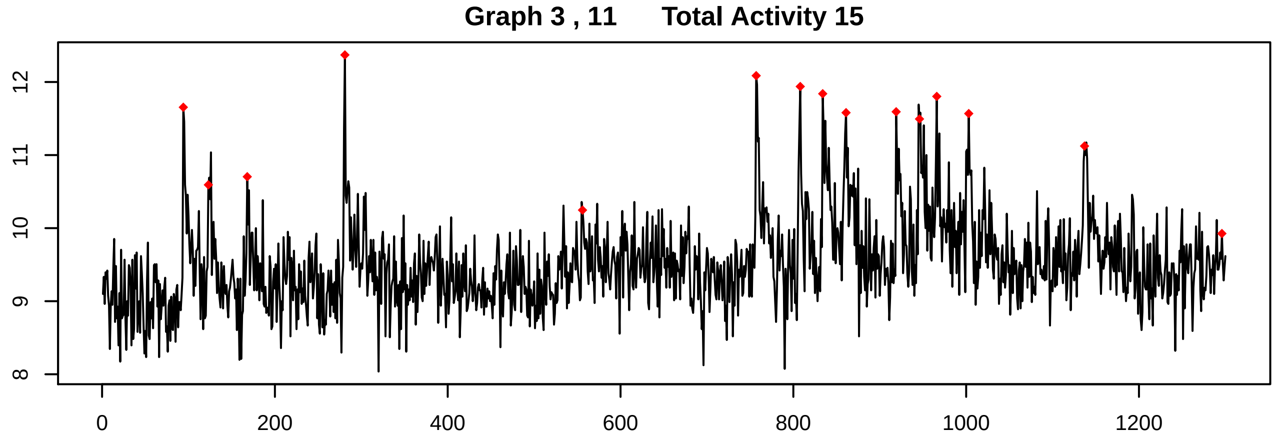
Graph 3 , 11 Total Activity 15
8
9
10
11
12
0
200
400
600
800
1000
1200

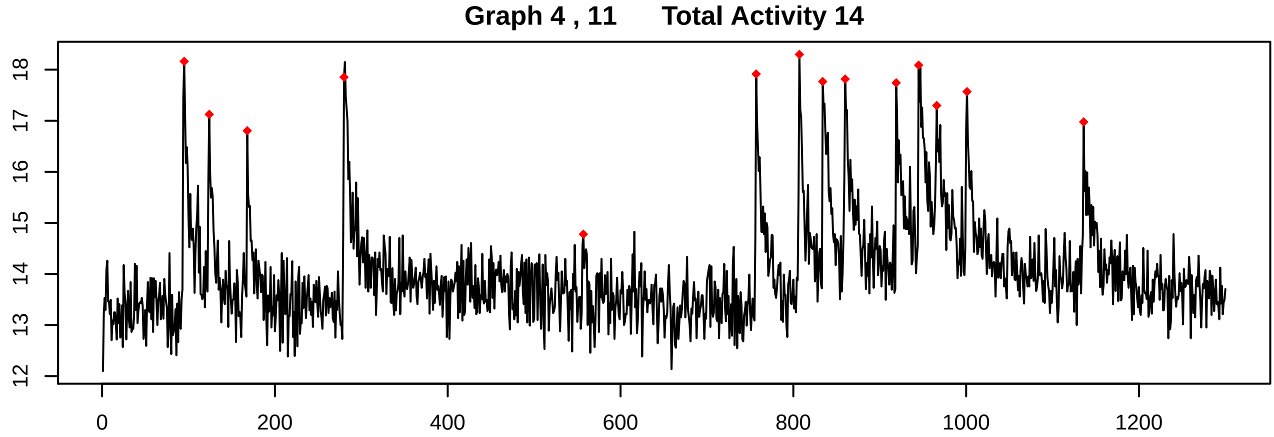
Graph 4 , 11 Total Activity 14
12
13
14
15
16
17
18
0
200
400
600
800
1000
1200

Graph 5 , 11   Total Activity 13

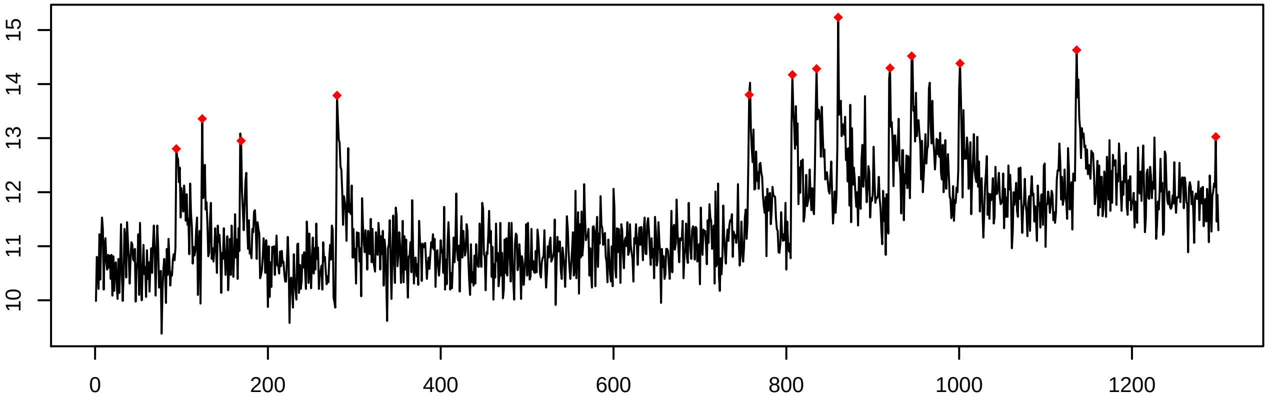

Graph 6 , 11   Total Activity 10

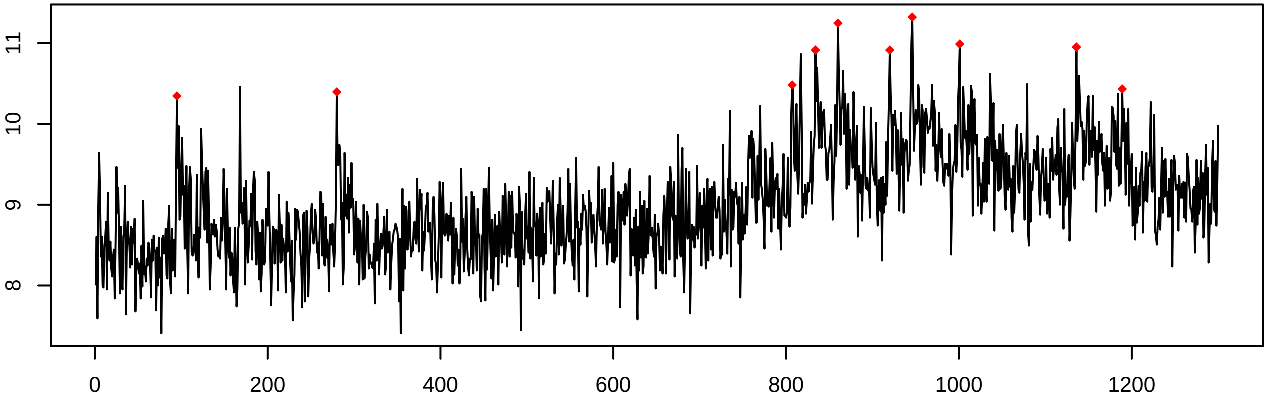

Graph 7 , 11   Total Activity 11

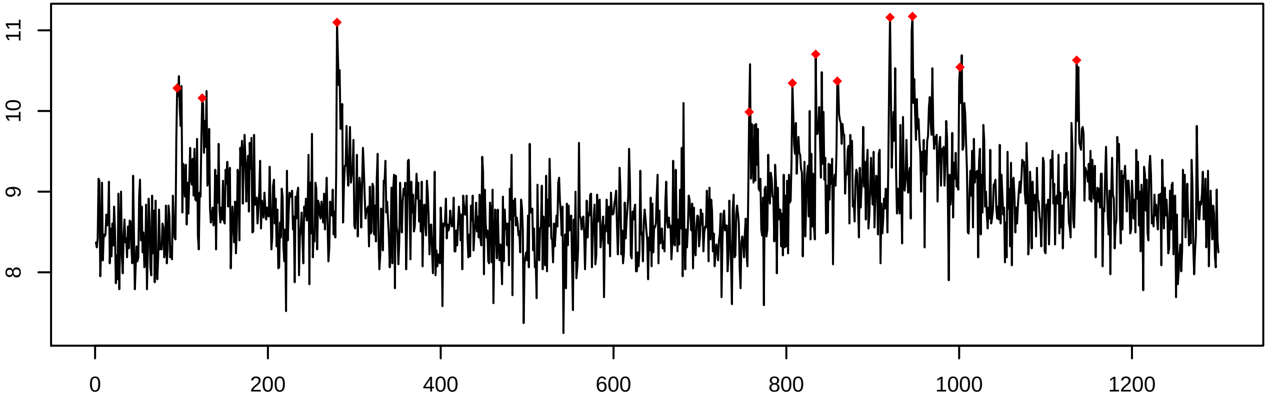

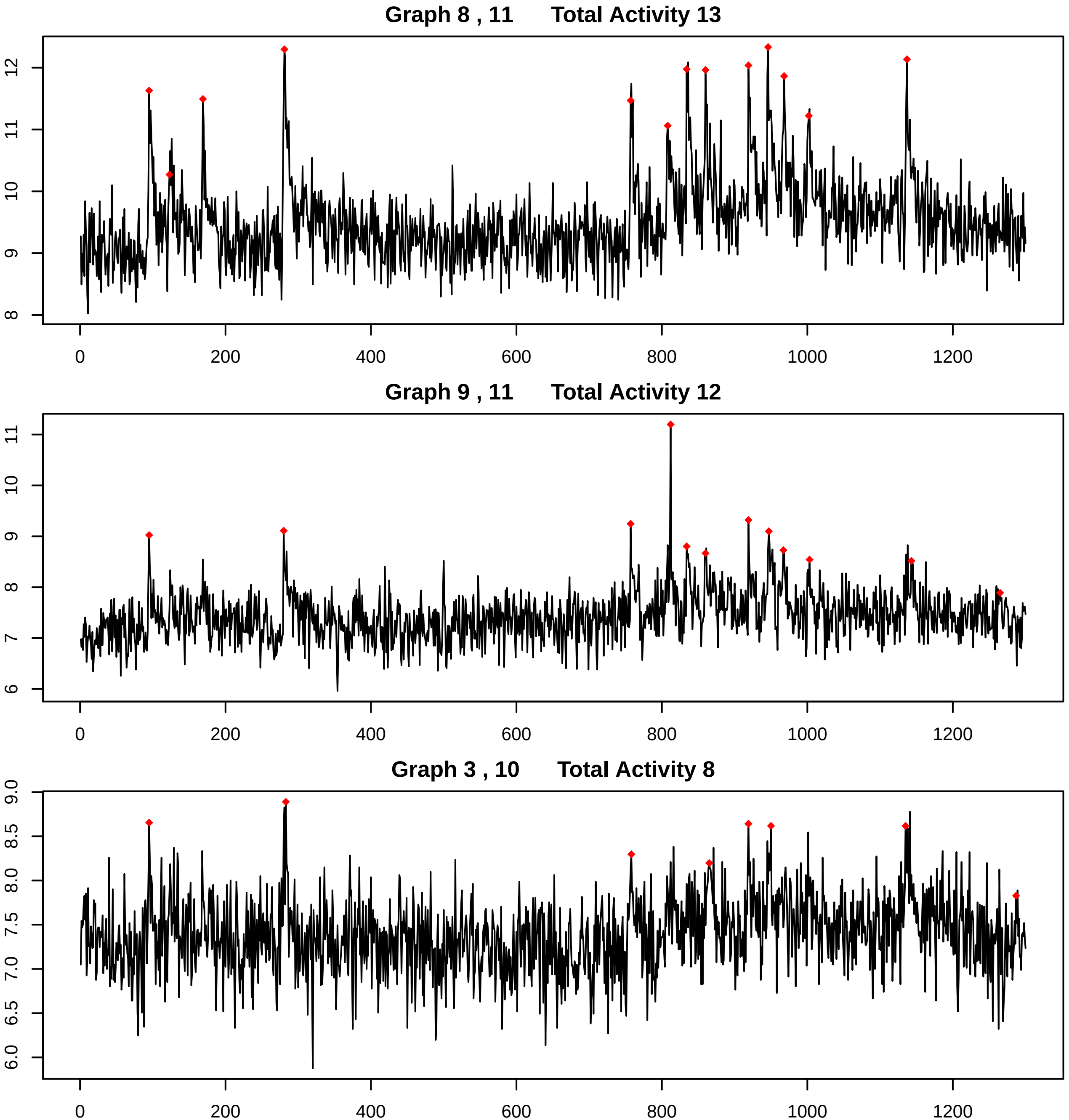
Graph 8 , 11 Total Activity 13
8 9 10 11 12
0 200 400 600 800 1000 1200
Graph 9 , 11 Total Activity 12
6 7 8 9 10 11
0 200 400 600 800 1000 1200
Graph 3 , 10 Total Activity 8
6.0 6.5 7.0 7.5 8.0 8.5 9.0
0 200 400 600 800 1000 1200

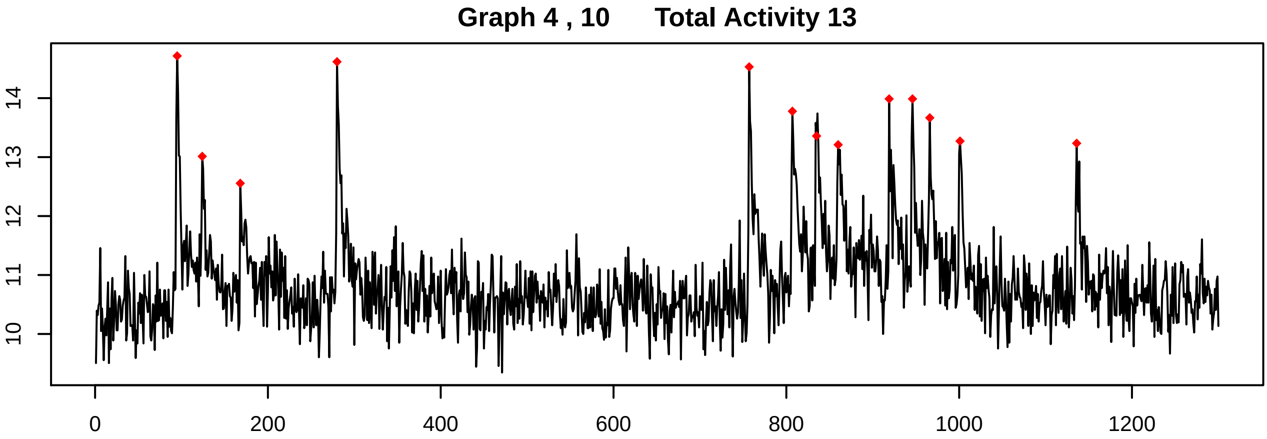
Graph 4 , 10 Total Activity 13
10
11
12
13
14
0
200
400
600
800
1000
1200

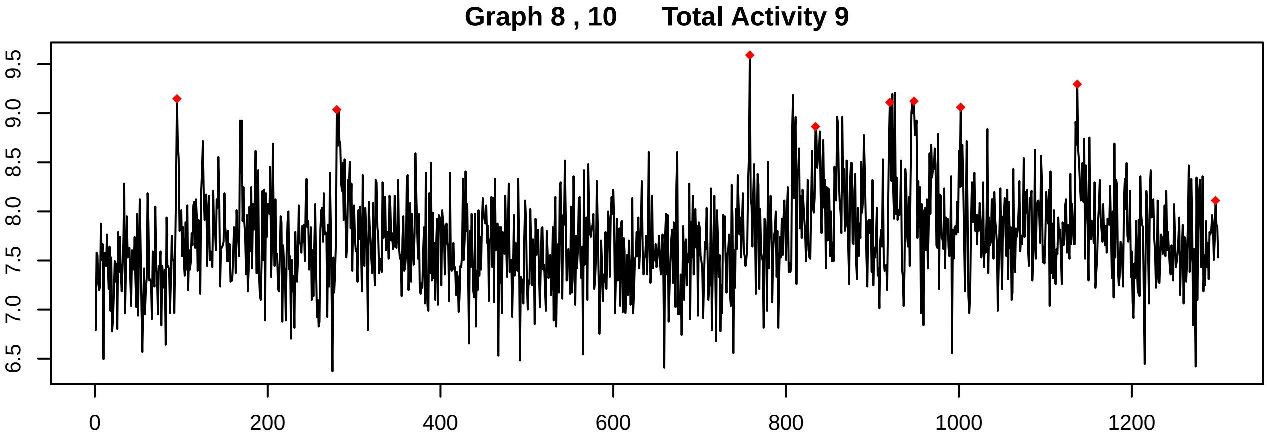
Graph 8 , 10 Total Activity 9
6.5
7.0
7.5
8.0
8.5
9.0
9.5
0
200
400
600
800
1000
1200

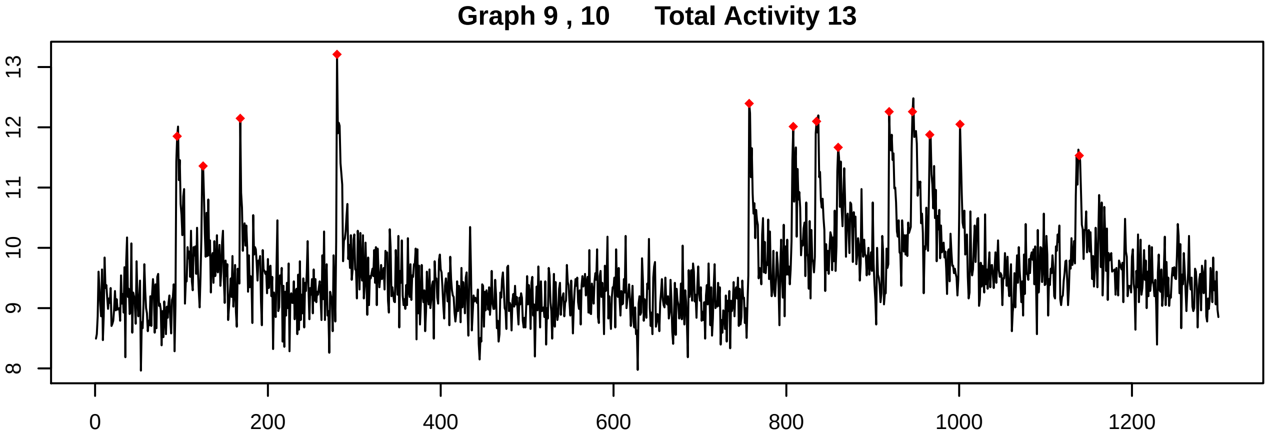
Graph 9 , 10 Total Activity 13
8
9
10
11
12
13
0
200
400
600
800
1000
1200

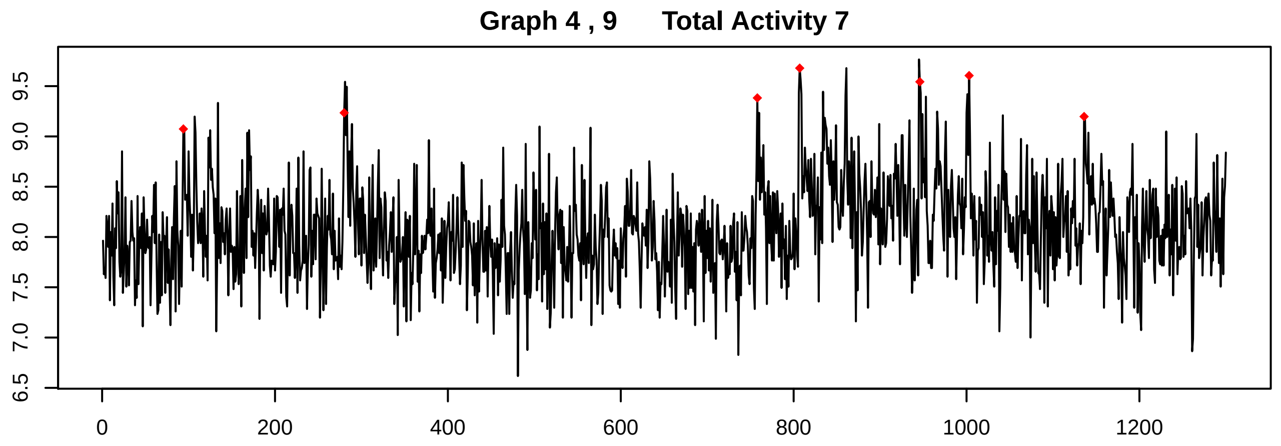
Graph 4 , 9   Total Activity 7
6.5
7.0
7.5
8.0
8.5
9.0
9.5
0
200
400
600
800
1000
1200

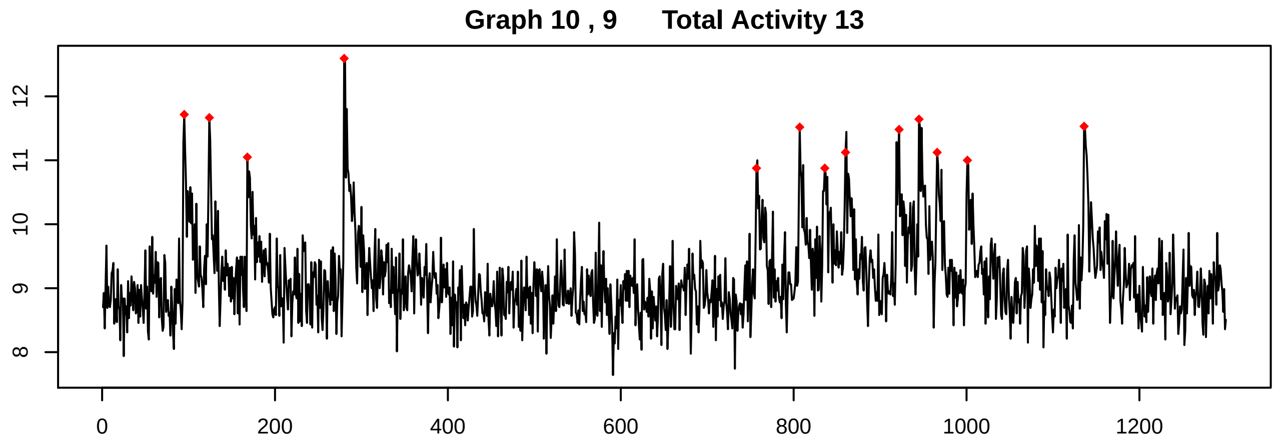
Graph 10 , 9   Total Activity 13
8
9
10
11
12
0
200
400
600
800
1000
1200

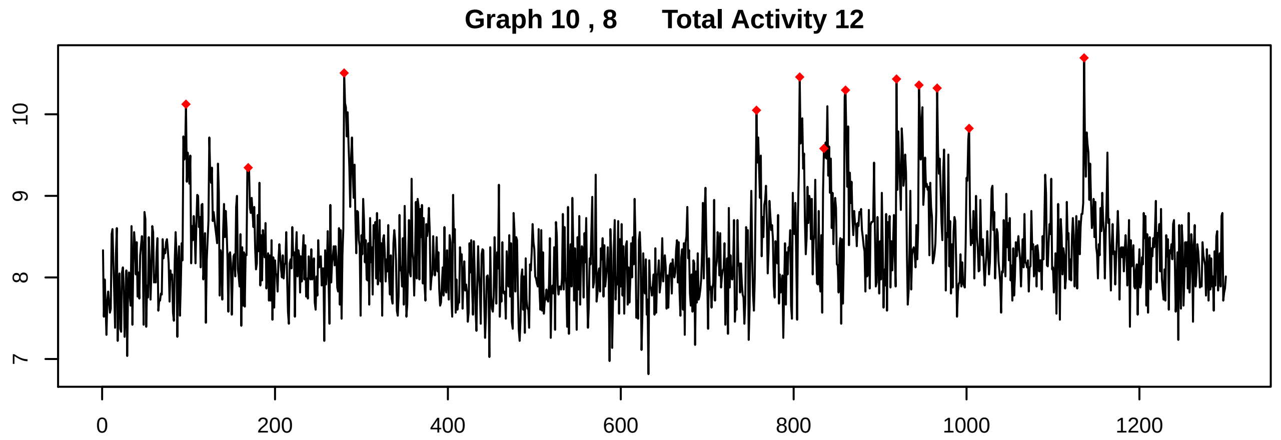
Graph 10 , 8   Total Activity 12
7
8
9
10
0
200
400
600
800
1000
1200

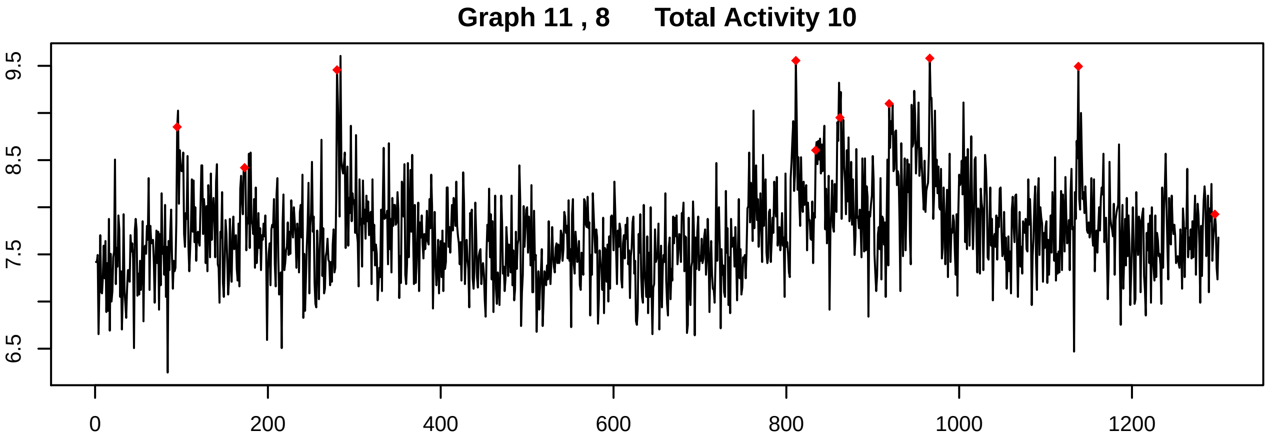
Graph 11 , 8   Total Activity 10
9.5
8.5
7.5
6.5
0
200
400
600
800
1000
1200

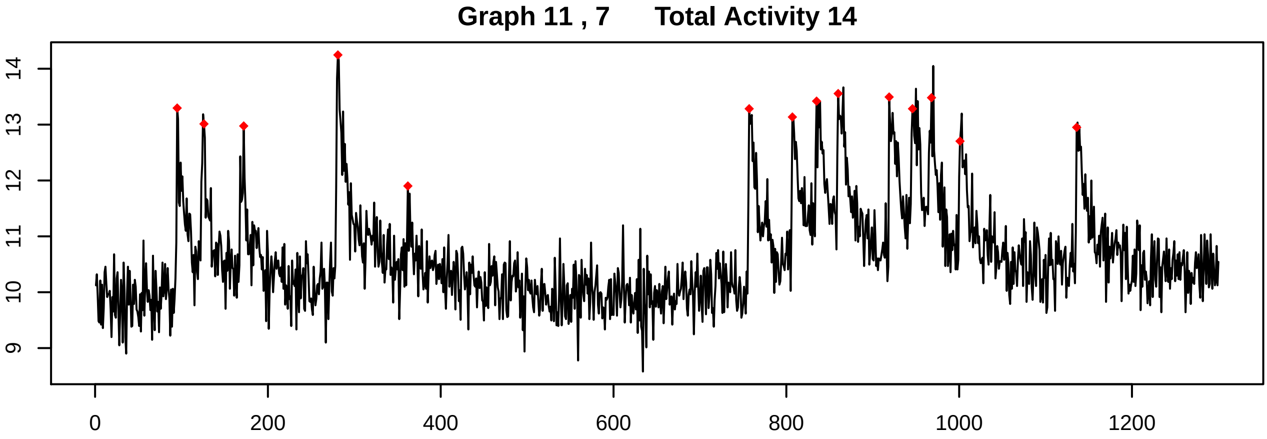
Graph 11 , 7   Total Activity 14
14
13
12
11
10
9
0
200
400
600
800
1000
1200

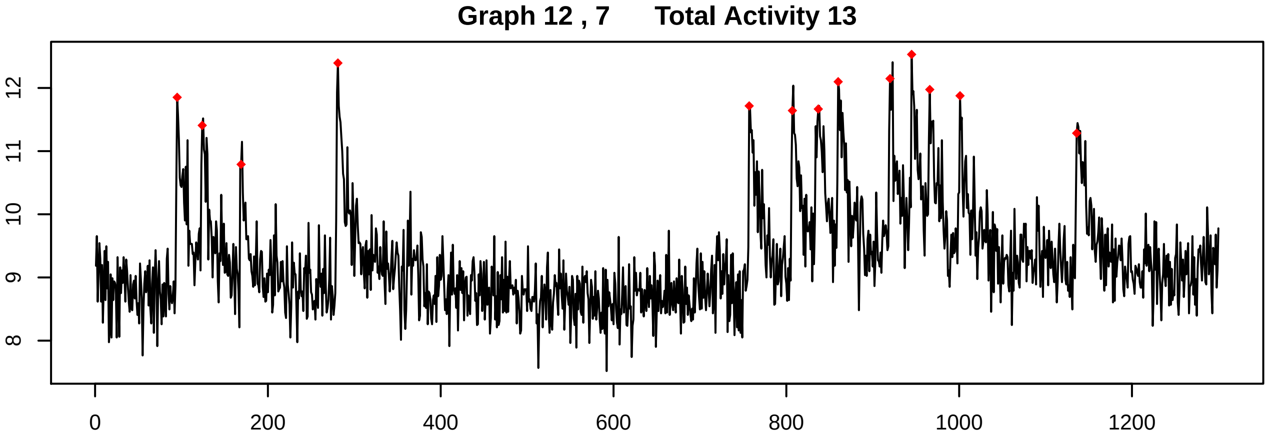
Graph 12 , 7   Total Activity 13
12
11
10
9
8
0
200
400
600
800
1000
1200

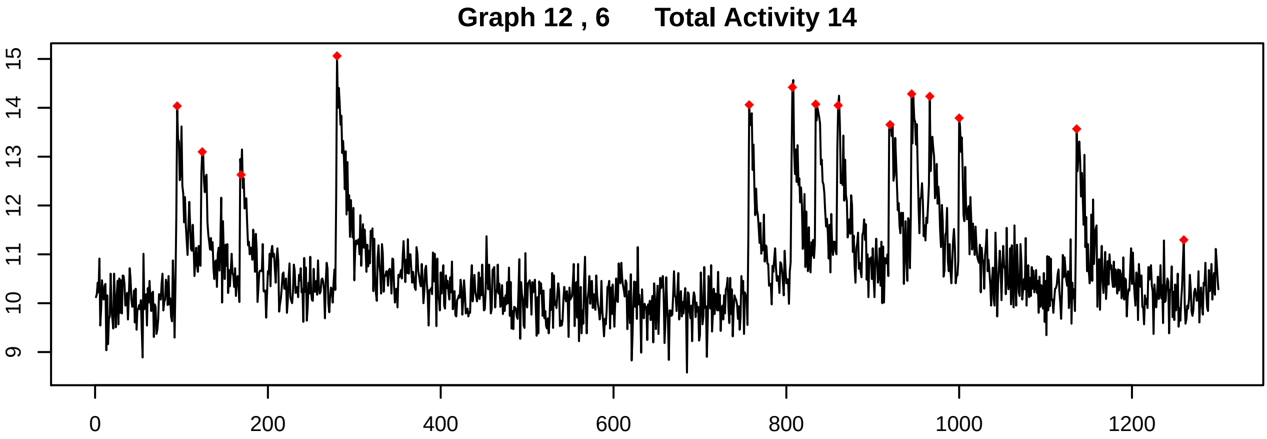
Graph 12 , 6 Total Activity 14
9
10
11
12
13
14
15
0
200
400
600
800
1000
1200

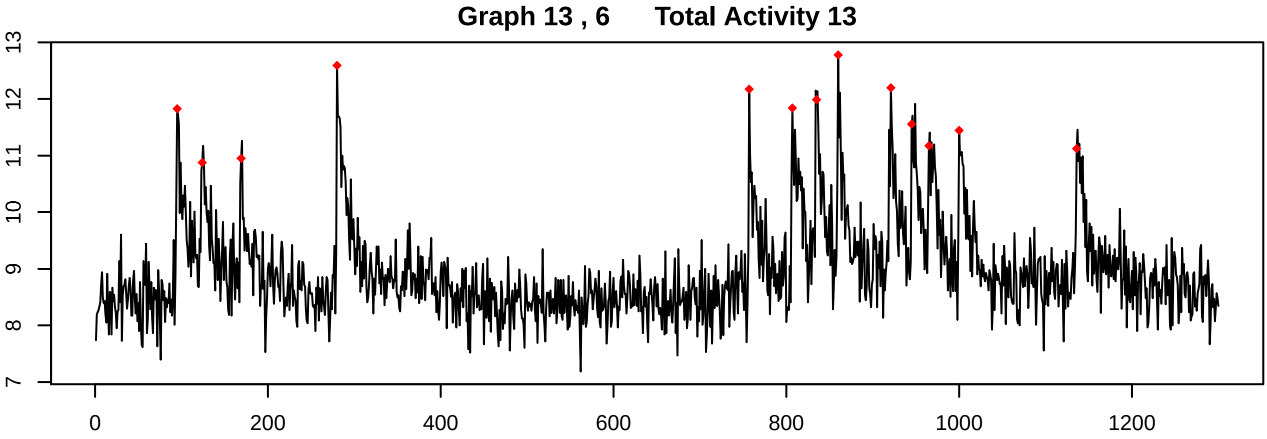
Graph 13 , 6 Total Activity 13
7
8
9
10
11
12
13
0
200
400
600
800
1000
1200

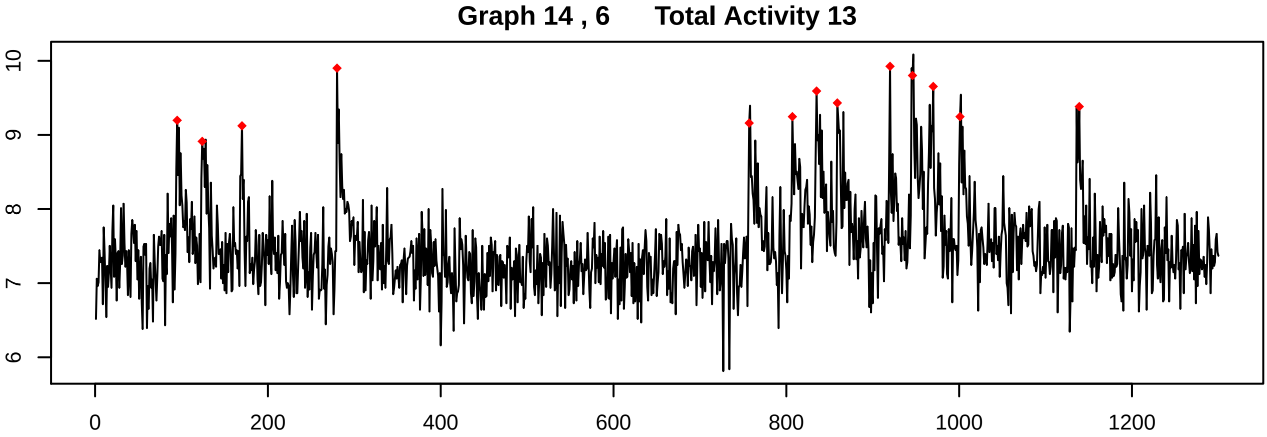
Graph 14 , 6 Total Activity 13
6
7
8
9
10
0
200
400
600
800
1000
1200

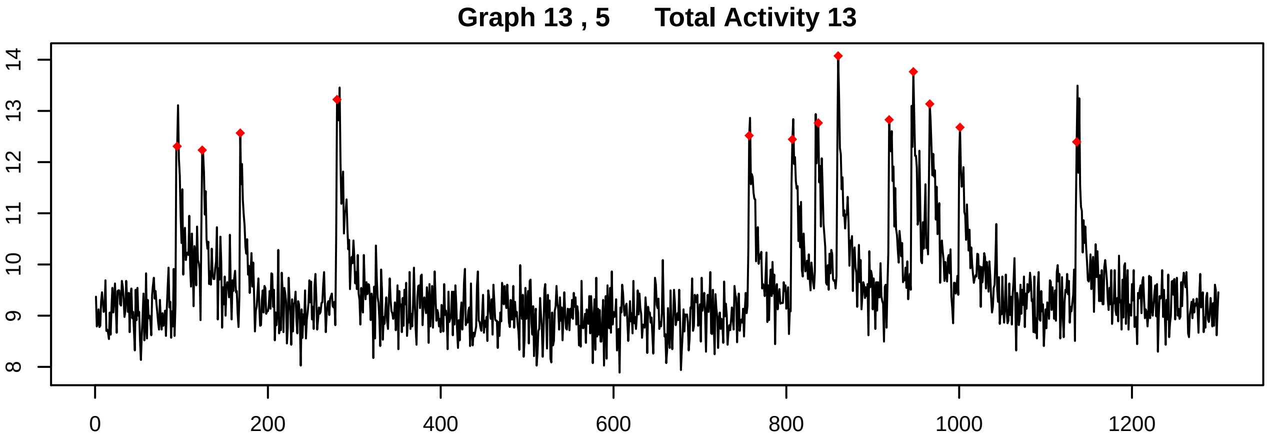
Graph 13 , 5 Total Activity 13
8
9
10
11
12
13
14
0
200
400
600
800
1000
1200

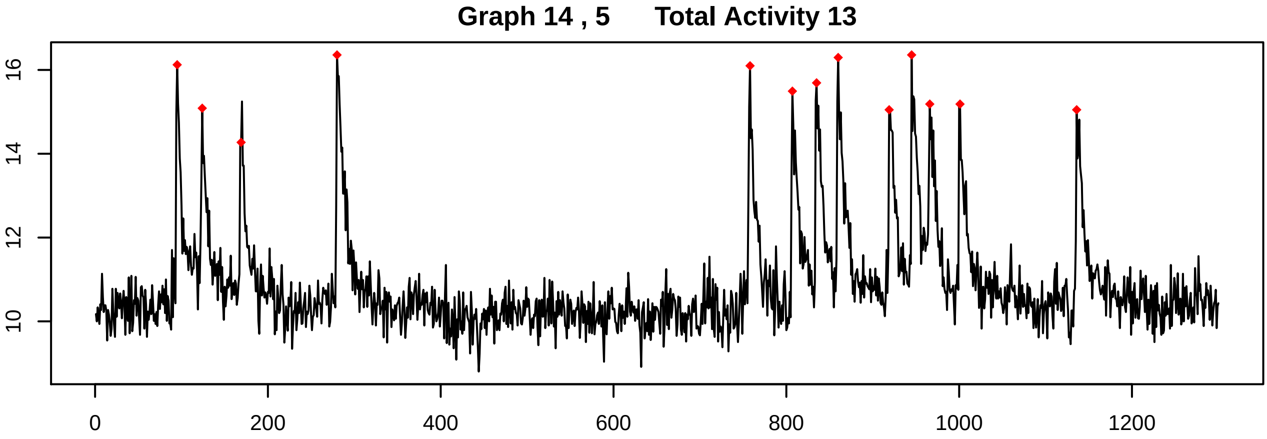
Graph 14 , 5 Total Activity 13
10
12
14
16
0
200
400
600
800
1000
1200

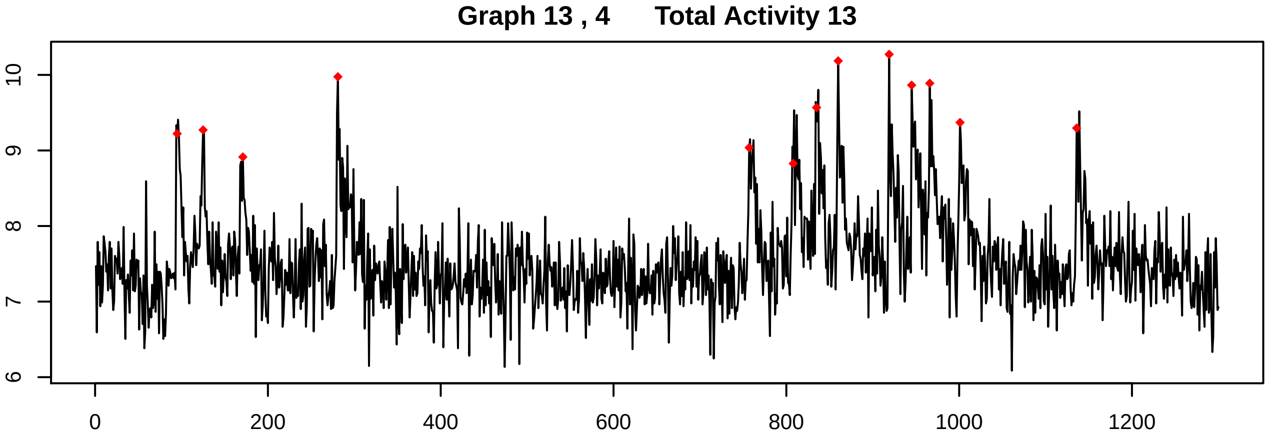
Graph 13 , 4 Total Activity 13
6
7
8
9
10
0
200
400
600
800
1000
1200

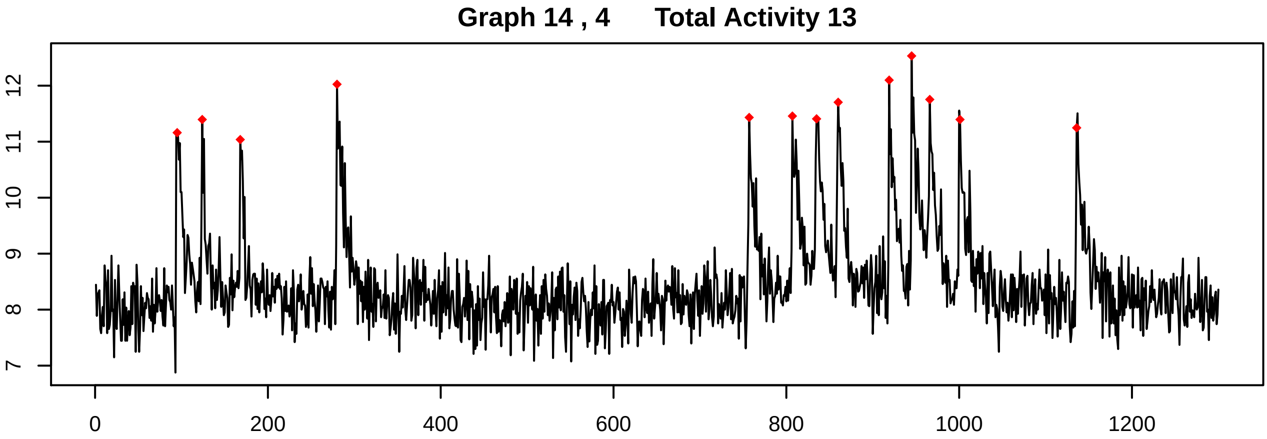

Graph 14 , 4 Total Activity 13
7
8
9
10
11
12
0
200
400
600
800
1000
1200
