# Supplementary material for: An open source tool for automatic spatiotemporal assessment of calcium transients and local ‘signal-close-to-noise’ activity in calcium imaging data
Source: PLoS Comput Biol. 2018 Mar 30;14(3):e1006054. doi: 10.1371/journal.pcbi.1006054 (PMC5895056; doi:10.1371/journal.pcbi.1006054)
Supplement: S3 Fig — Computation of a motoneuron in the low activity phase. (PDF) [file pcbi.1006054.s003.pdf]

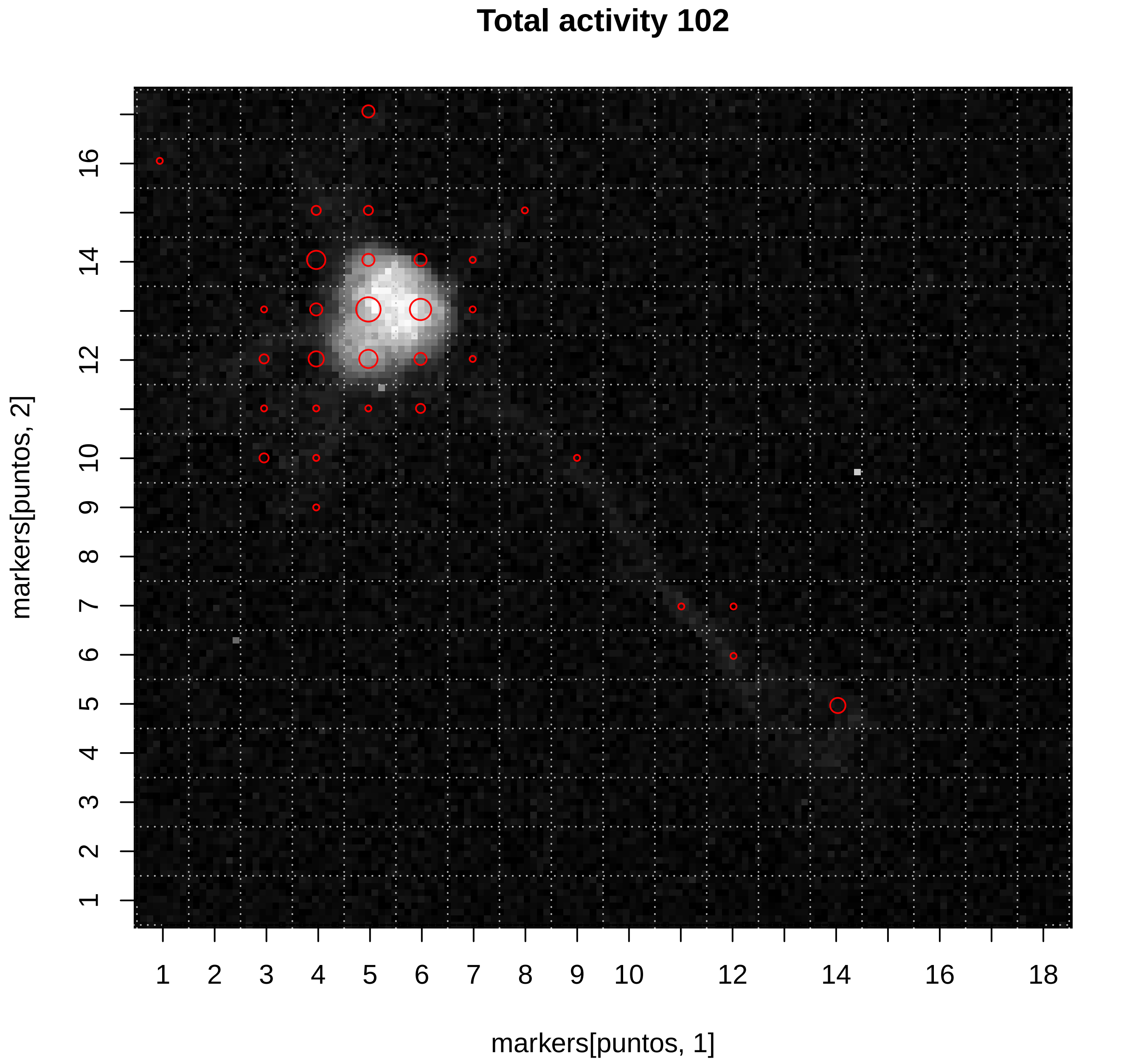

Total activity 102
markers[puntos, 2]
16
14
12
10
9
8
7
6
5
4
3
2
1
markers[puntos, 1]
1
2
3
4
5
6
7
8
9
10
12
14
16
18

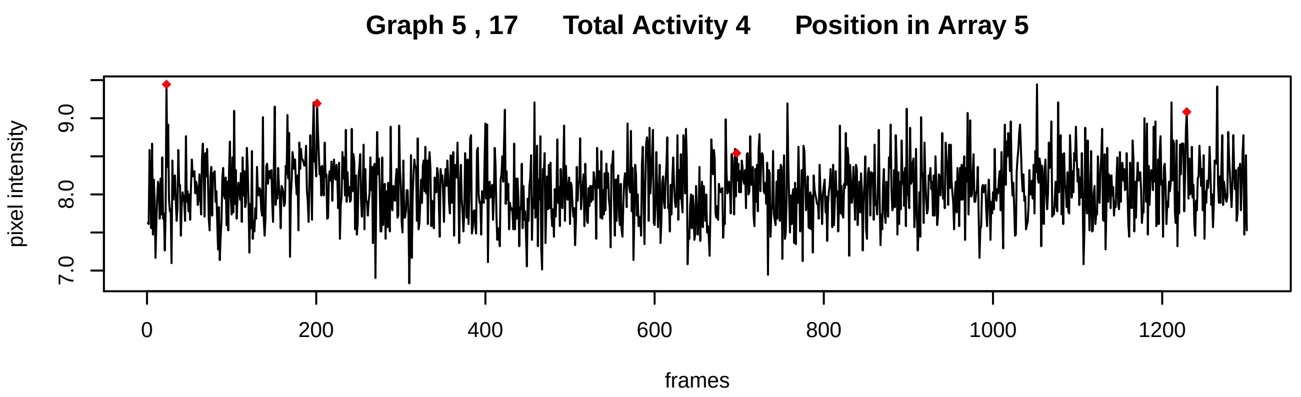
Graph 5 , 17 Total Activity 4 Position in Array 5
pixel intensity
7.0
8.0
9.0
0
200
400
600
800
1000
1200
frames

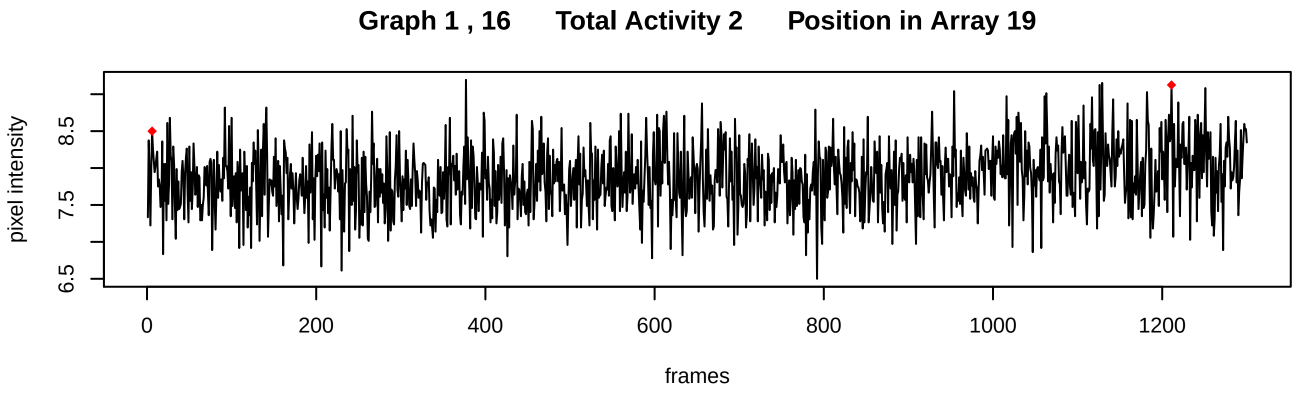
Graph 1 , 16 Total Activity 2 Position in Array 19
pixel intensity
6.5
7.5
8.5
0
200
400
600
800
1000
1200
frames

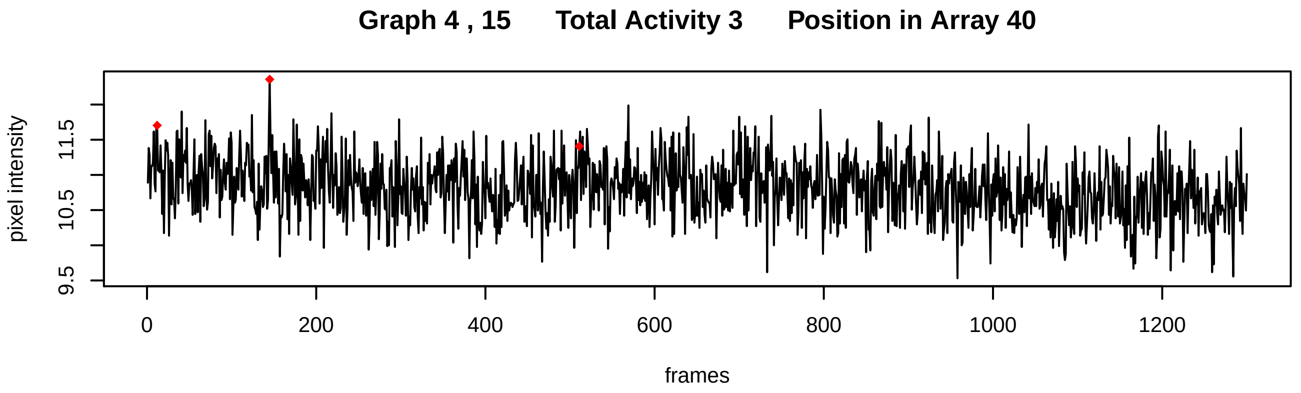
Graph 4 , 15 Total Activity 3 Position in Array 40
pixel intensity
9.5
10.5
11.5
0
200
400
600
800
1000
1200
frames

Graph 5 , 15 Total Activity 3 Position in Array 41

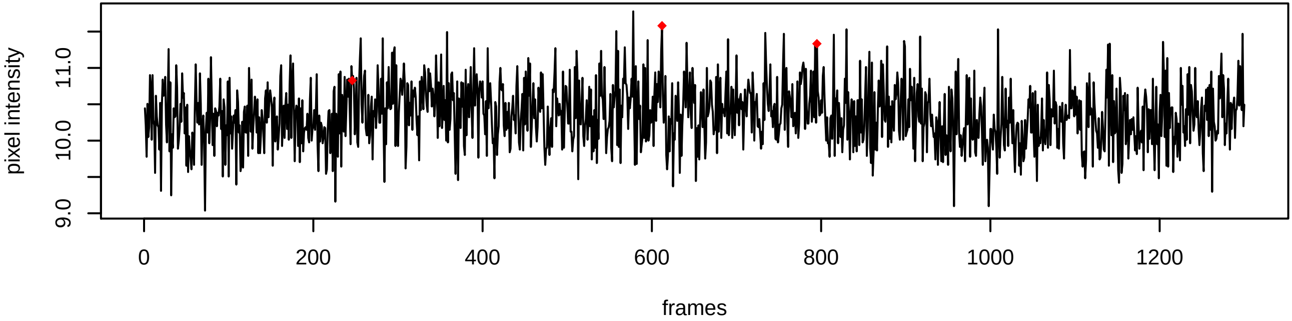

Graph 8 , 15 Total Activity 2 Position in Array 44

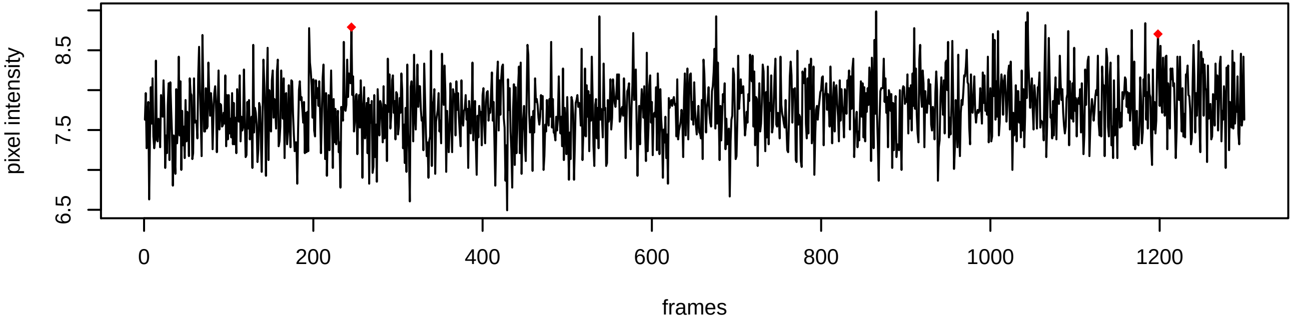

Graph 4 , 14 Total Activity 6 Position in Array 58

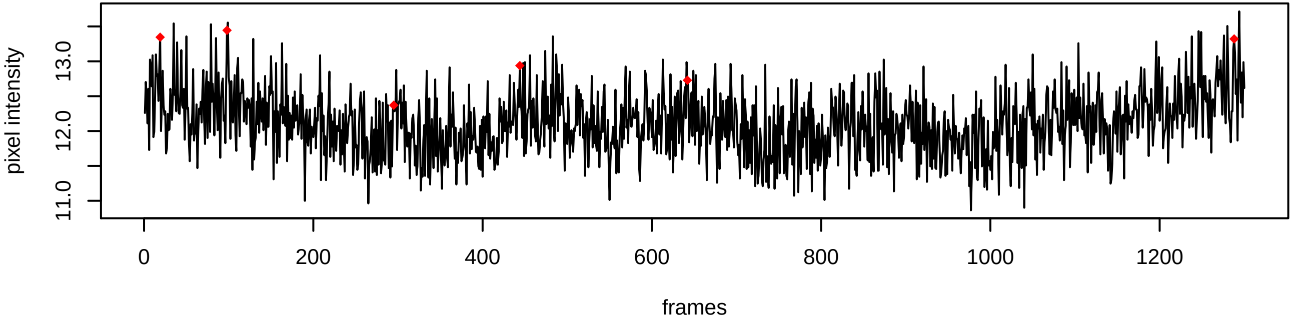

Graph 5 , 14 Total Activity 4 Position in Array 59

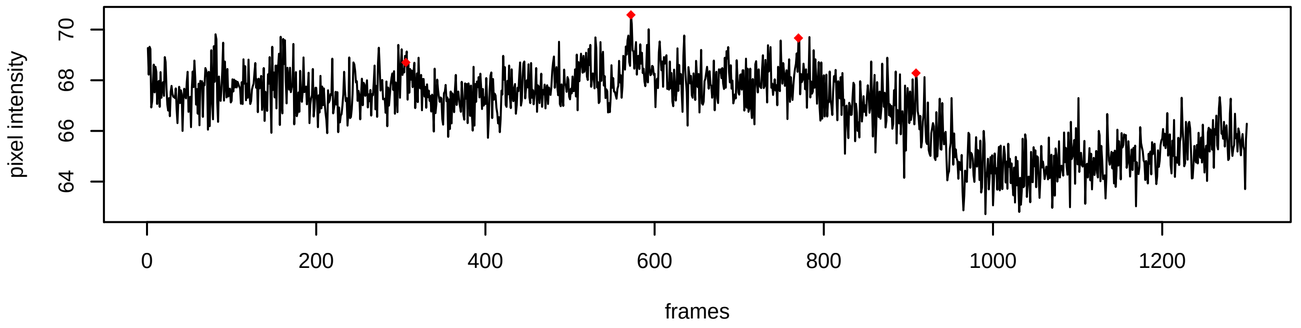

Graph 6 , 14 Total Activity 4 Position in Array 60

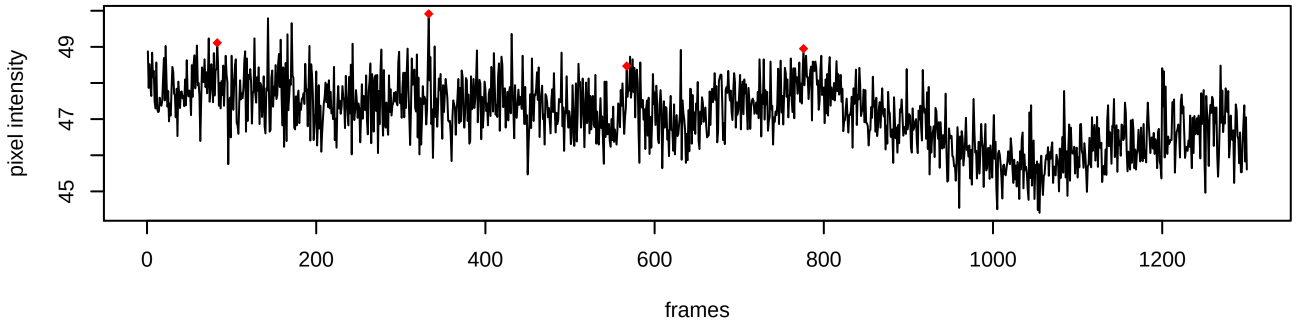

Graph 7 , 14 Total Activity 2 Position in Array 61

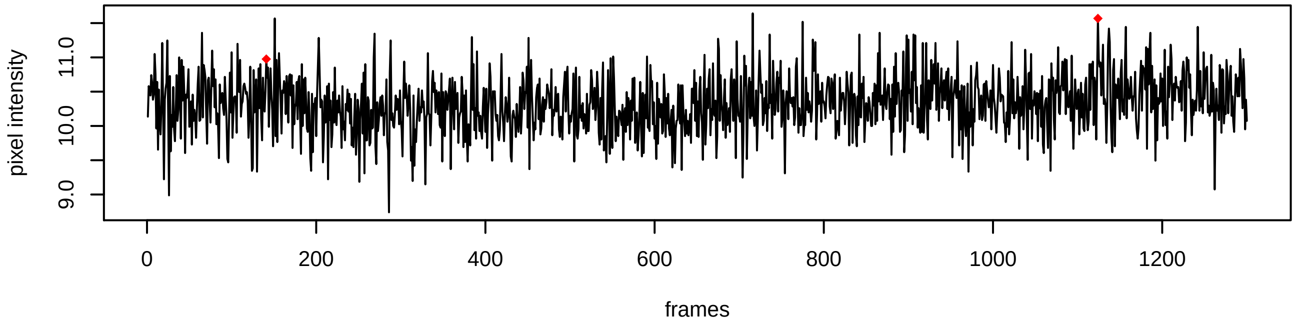

Graph 3 , 13   Total Activity 2   Position in Array 75

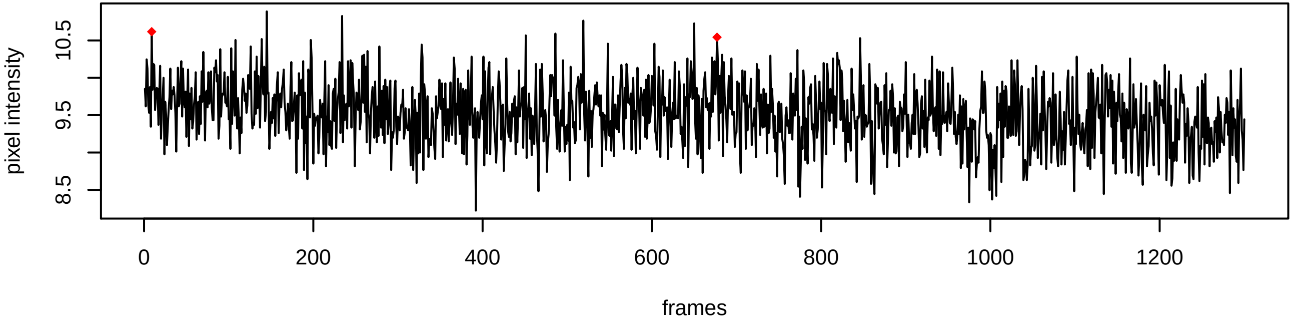

Graph 4 , 13   Total Activity 4   Position in Array 76

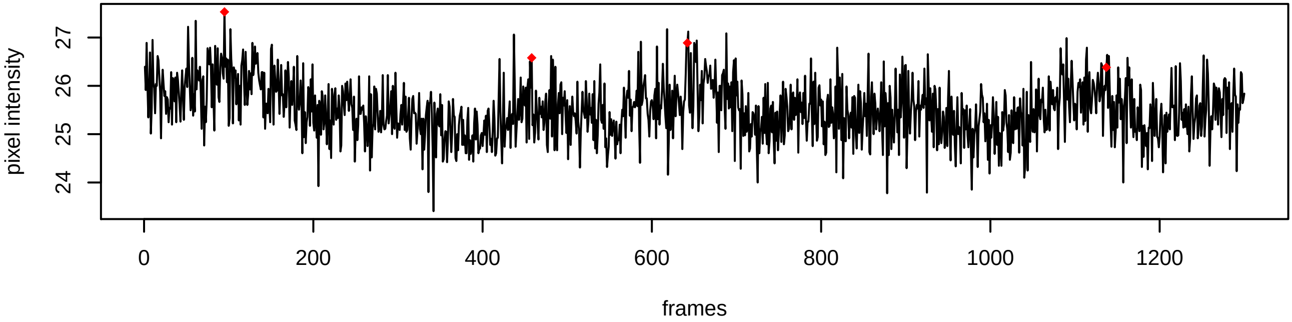

Graph 5 , 13   Total Activity 8   Position in Array 77

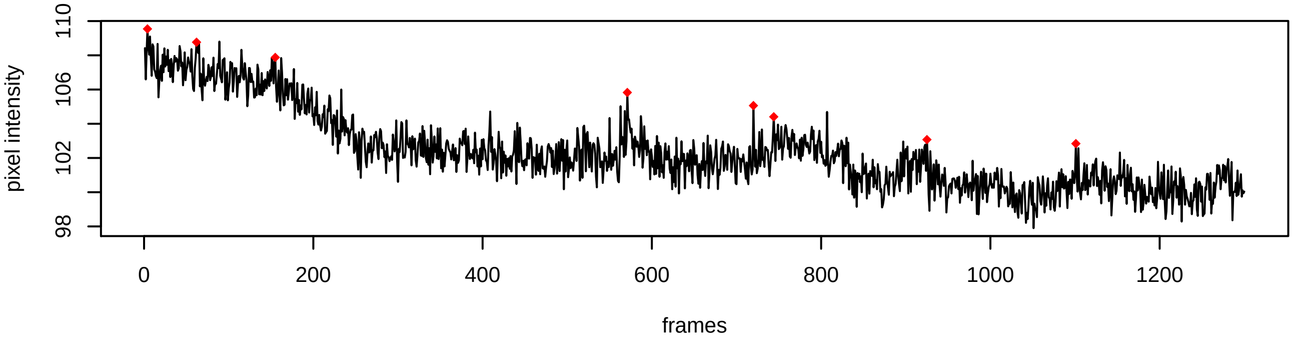

Graph 6 , 13   Total Activity 7   Position in Array 78

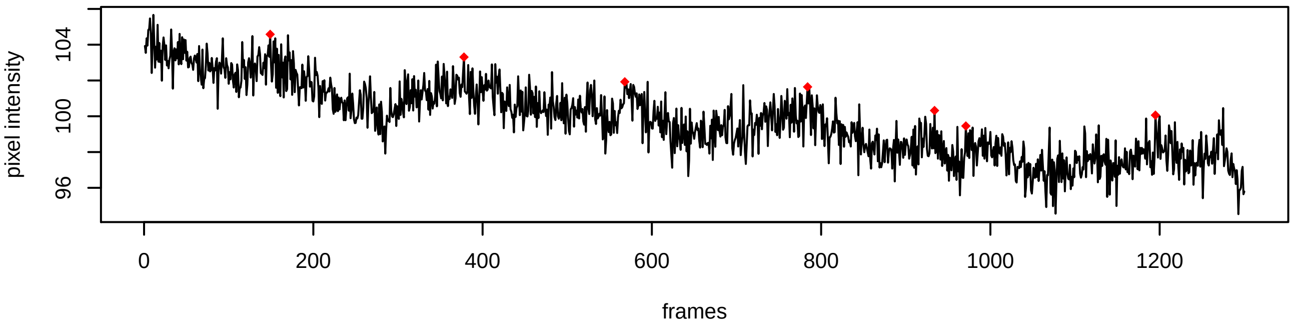

Graph 7 , 13   Total Activity 2   Position in Array 79

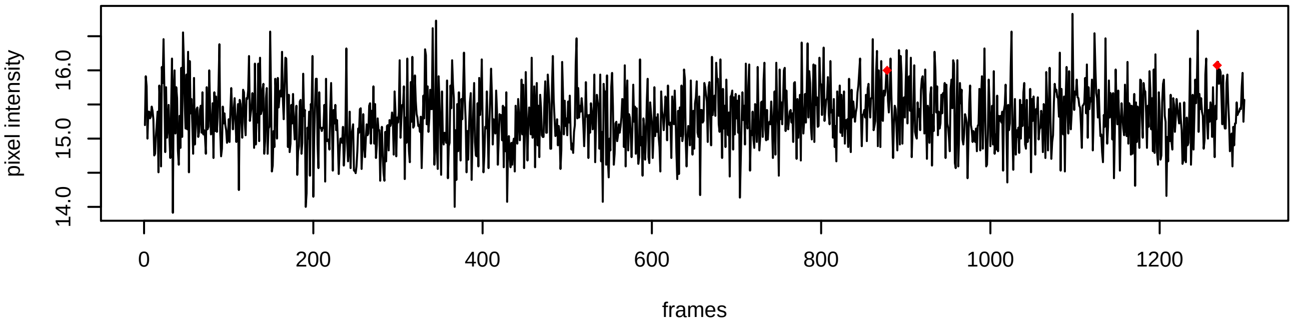

Graph 3 , 12   Total Activity 3   Position in Array 93

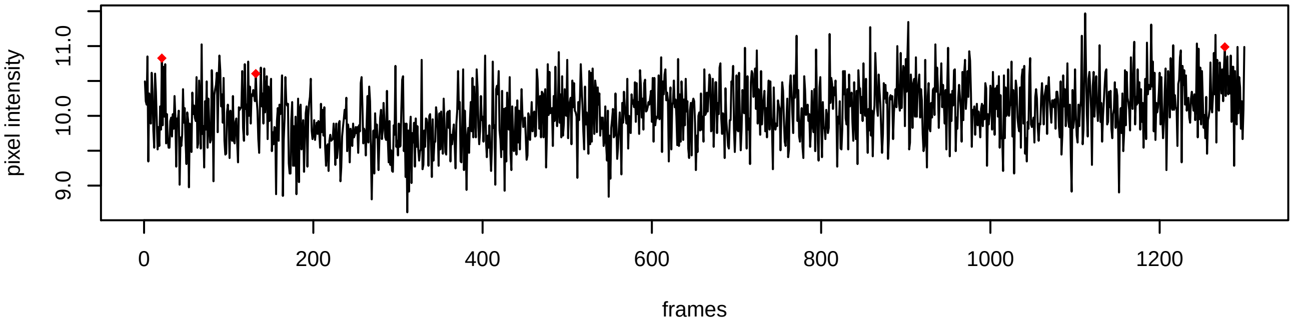

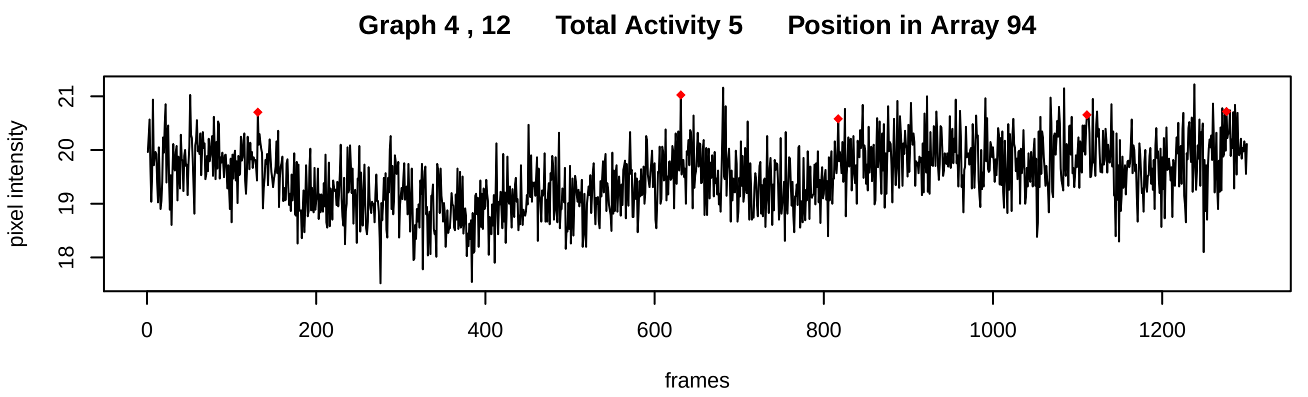
Graph 4 , 12 Total Activity 5 Position in Array 94
pixel intensity
18
19
20
21
0
200
400
600
800
1000
1200
frames

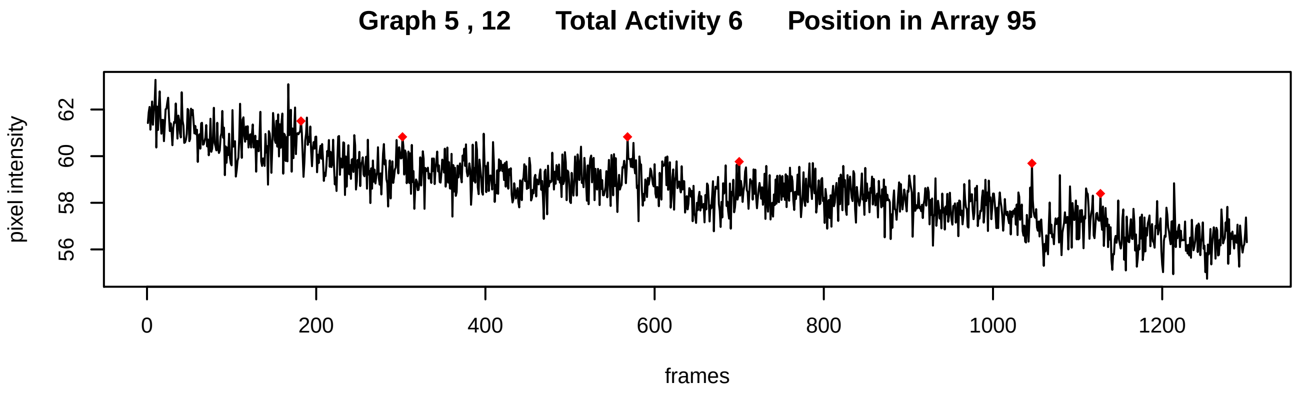
Graph 5 , 12 Total Activity 6 Position in Array 95
pixel intensity
56
58
60
62
0
200
400
600
800
1000
1200
frames

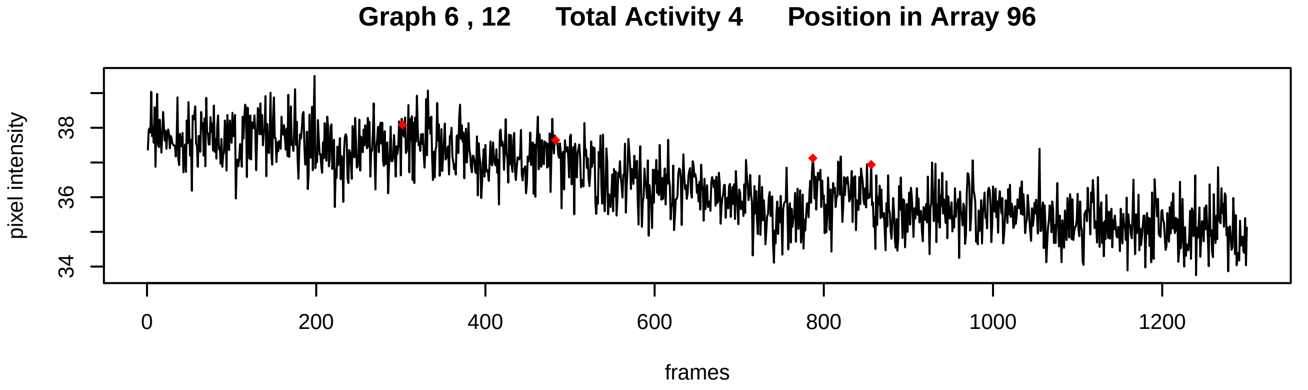
Graph 6 , 12 Total Activity 4 Position in Array 96
pixel intensity
34
36
38
0
200
400
600
800
1000
1200
frames

Graph 7 , 12   Total Activity 2   Position in Array 97

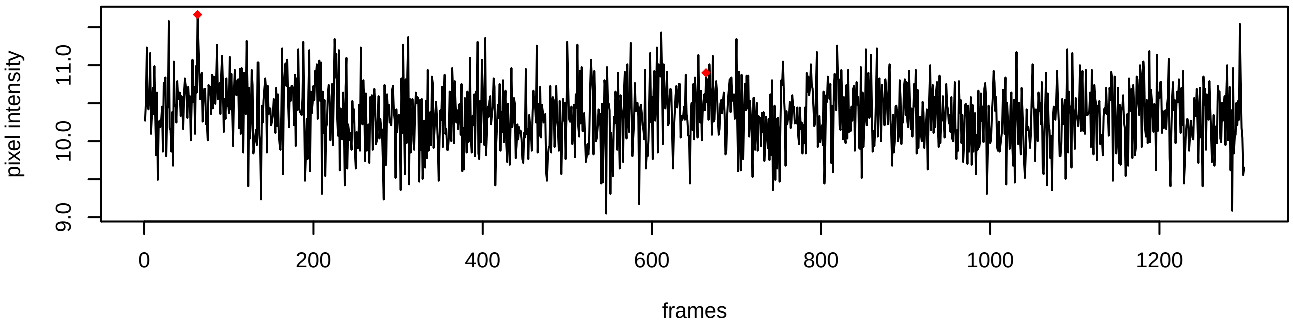

Graph 3 , 11   Total Activity 2   Position in Array 111

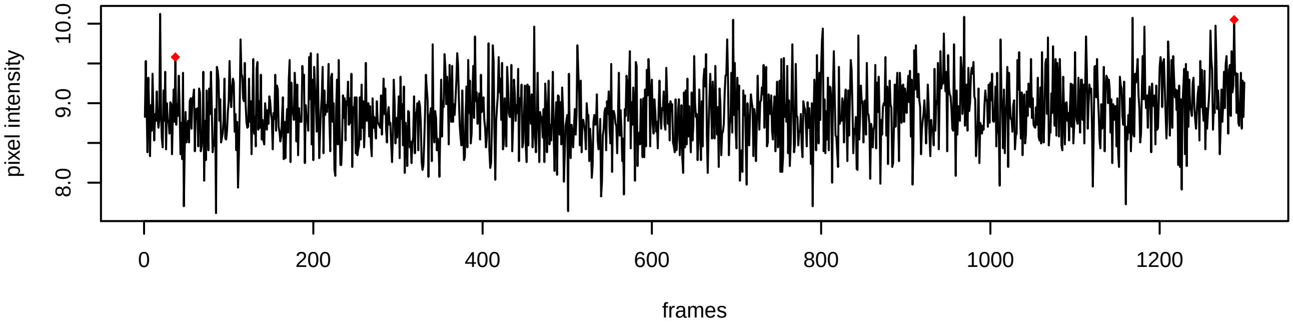

Graph 4 , 11   Total Activity 2   Position in Array 112

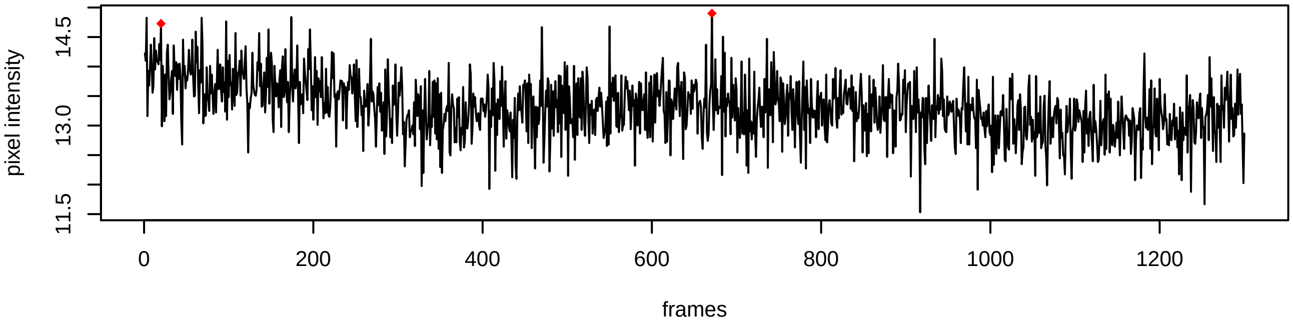

Graph 5 , 11 Total Activity 2 Position in Array 113

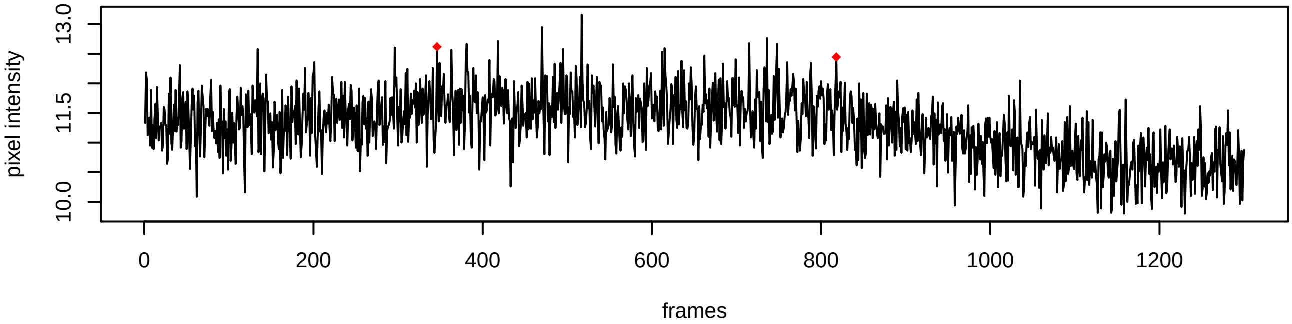

Graph 6 , 11 Total Activity 3 Position in Array 114

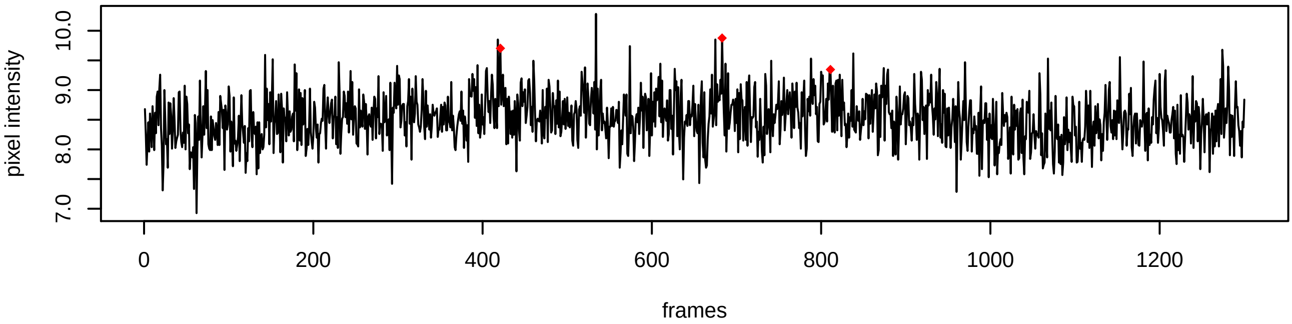

Graph 3 , 10 Total Activity 3 Position in Array 129

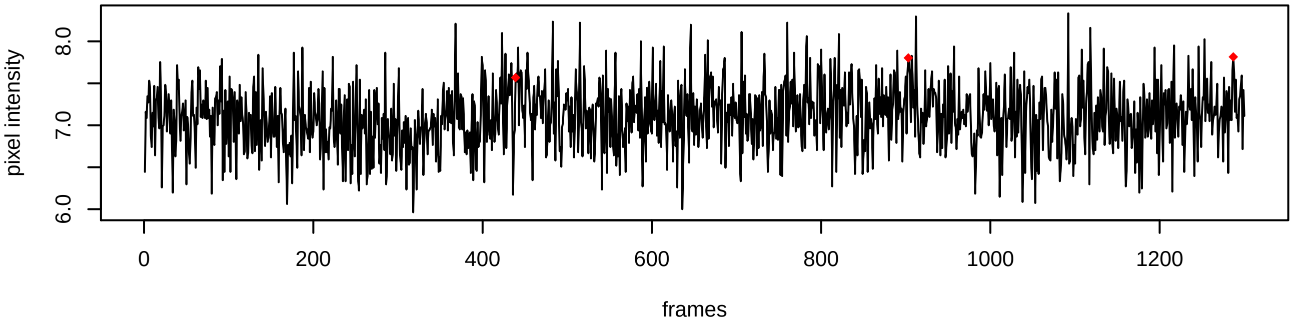

Graph 4 , 10   Total Activity 2   Position in Array 130

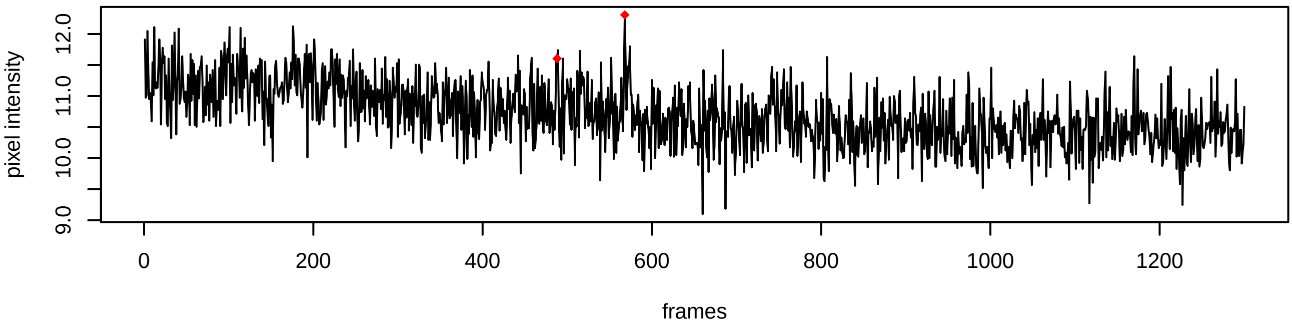

Graph 9 , 10   Total Activity 2   Position in Array 135

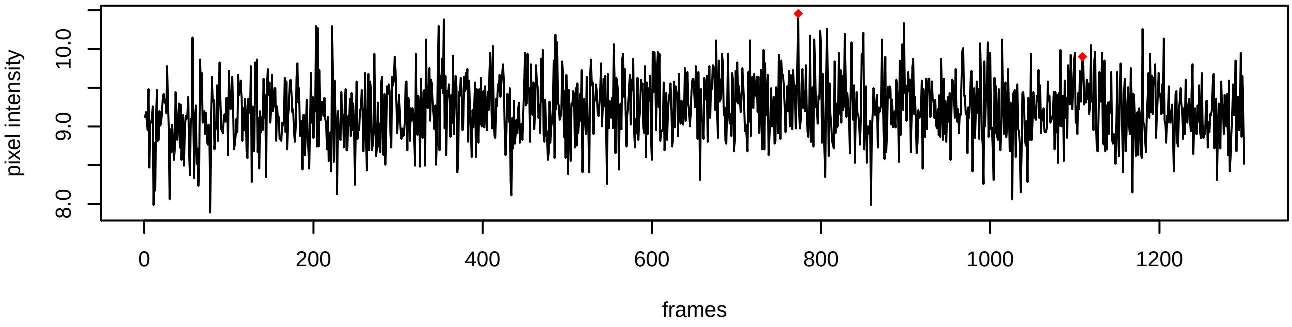

Graph 4 , 9   Total Activity 2   Position in Array 148

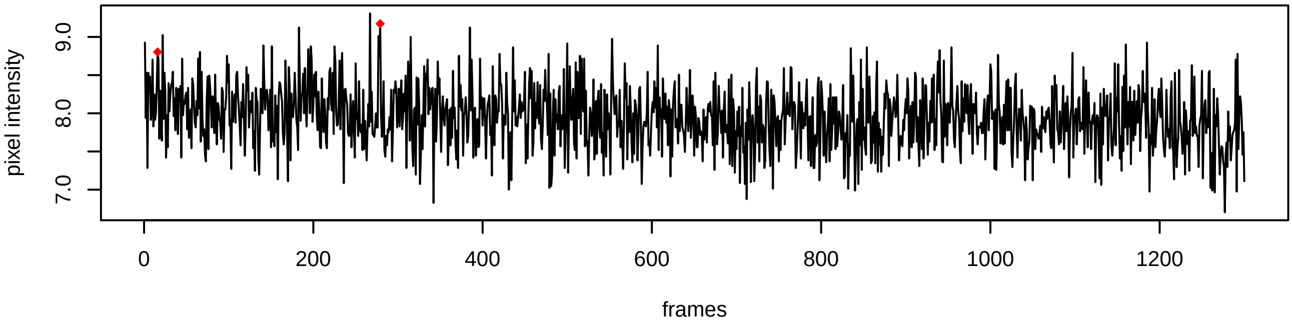

Graph 11 , 7 Total Activity 2 Position in Array 191

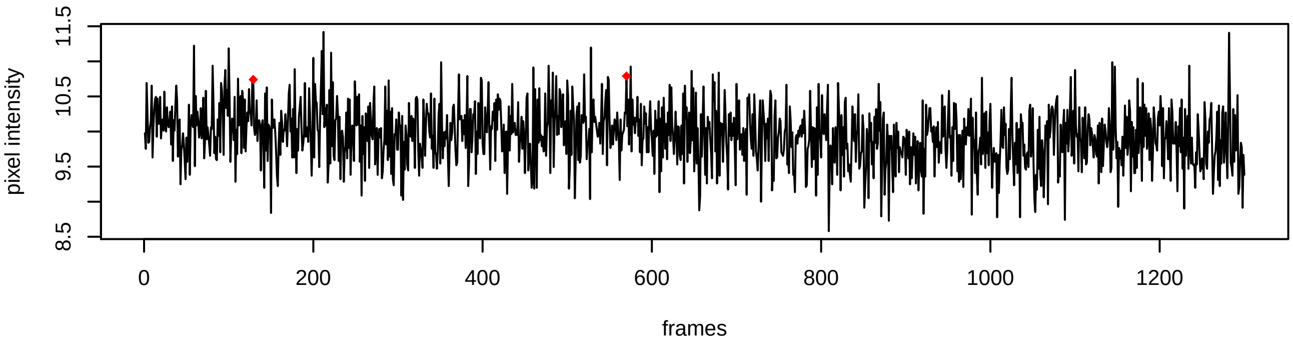

Graph 12 , 7 Total Activity 2 Position in Array 192

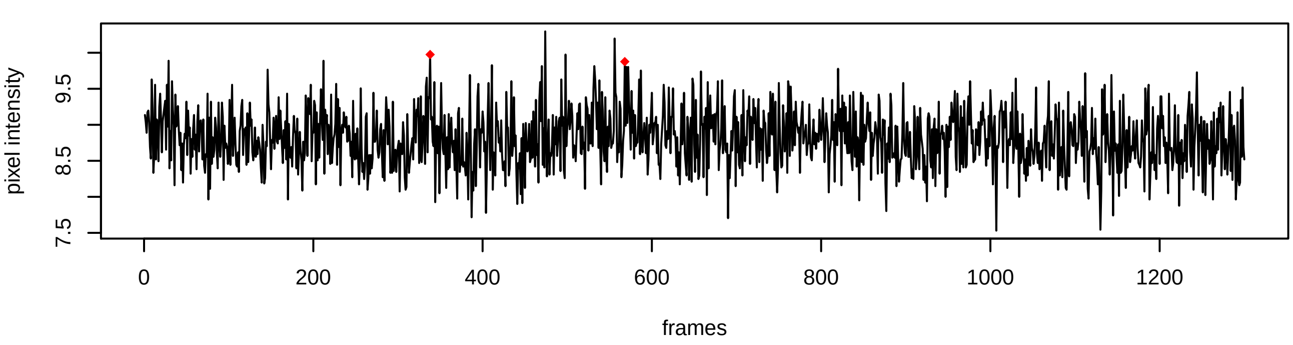

Graph 12 , 6 Total Activity 2 Position in Array 210

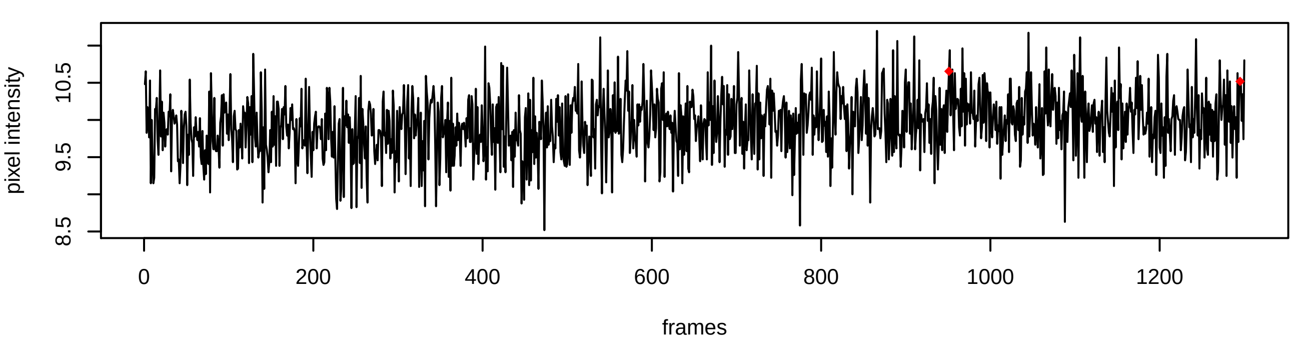

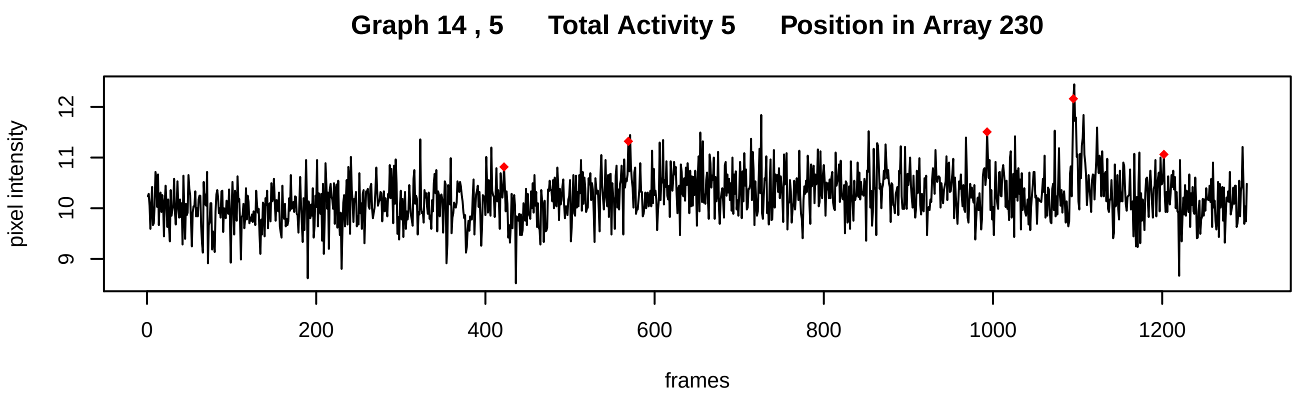
Graph 14 , 5 Total Activity 5 Position in Array 230
pixel intensity
9
10
11
12
0
200
400
600
800
1000
1200
frames
