# Supplementary figures and images for: An open source tool for automatic spatiotemporal assessment of calcium transients and local ‘signal-close-to-noise’ activity in calcium imaging data
Source: PLoS Comput Biol. 2018 Mar 30;14(3):e1006054. doi: 10.1371/journal.pcbi.1006054 (PMC5895056; doi:10.1371/journal.pcbi.1006054)

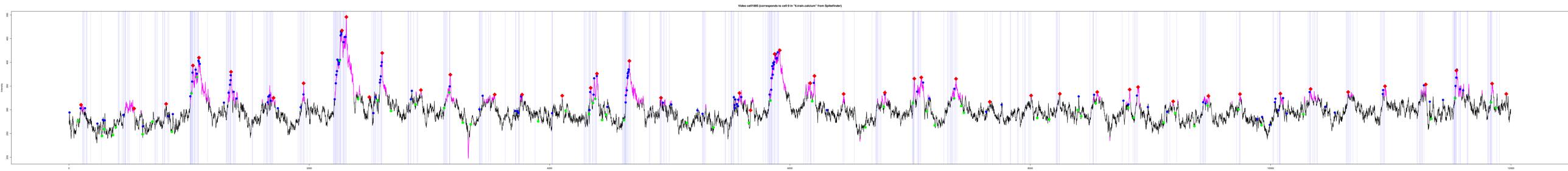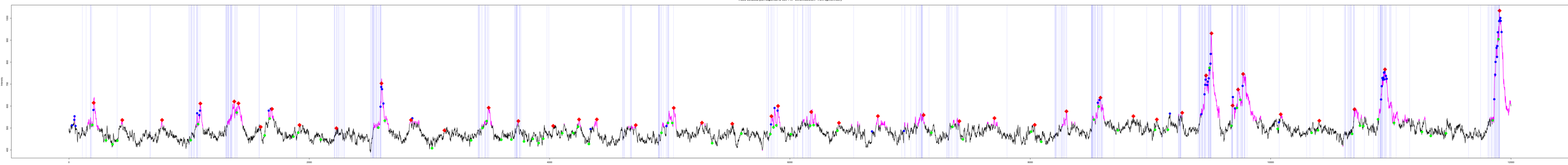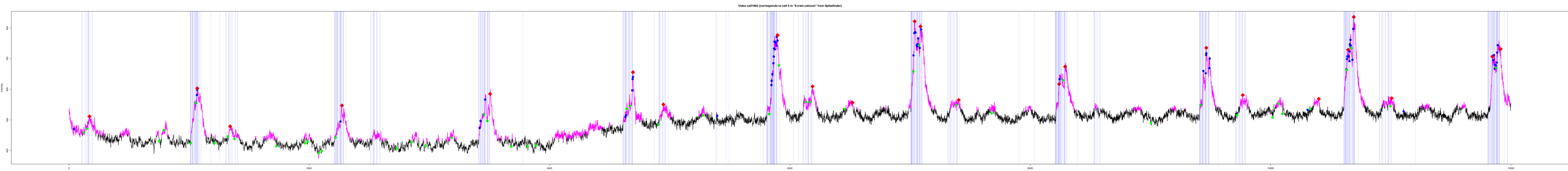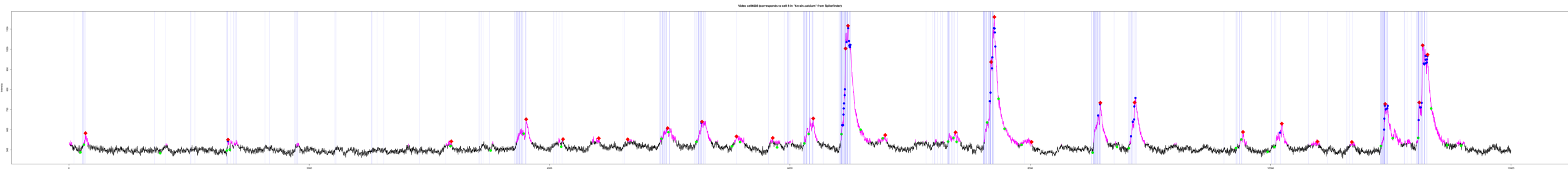

Supplement: S4 Fig — Comparison of CWT (our tool, red rhombus), deconvolution (blue dots), template-matching (green dots) and signal significance computation (“Romano toolbox”; magenta line) in a calibration dataset showing simultaneous imaging with loose-seal cell-attached recording in four GCaMP expressing neurons [37]. Black line: Calcium signal trace (raw data), blue vertical lines: spike information. The TIFF format image stacks were taken from: https://crcns.org/data-sets/methods/cai-1/about-cai-1 [37]. This dataset contains raw data information showing the signal behavior of GCamp5k-expressing neurons. The spike-data were extracted from http://spikefinder.codeneuro.org/, dataset 06 [28]. We analyzed four videos (called cells 0, 1, 5, and 8 in the spikefinder training data). The names of the underlying image stack raw data are: cell1005, cell2002, cell1002, and cell4003. Please note that the original dataset contains 24.000 images acquired in two color channels (green and red). The calcium signal is only present in the green channel. Therefore every second image was removed for the computation. (PDF) [file pcbi.1006054.s004.pdf]

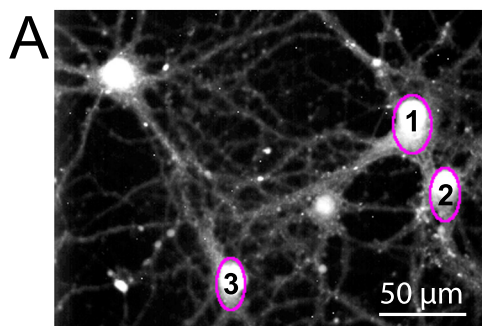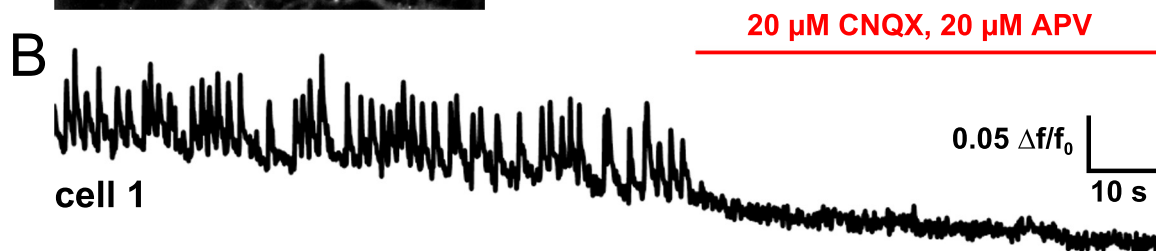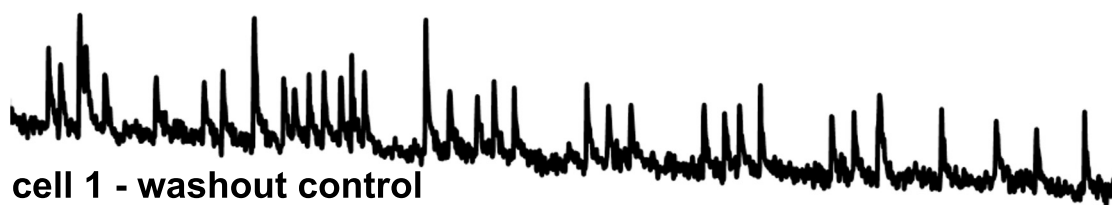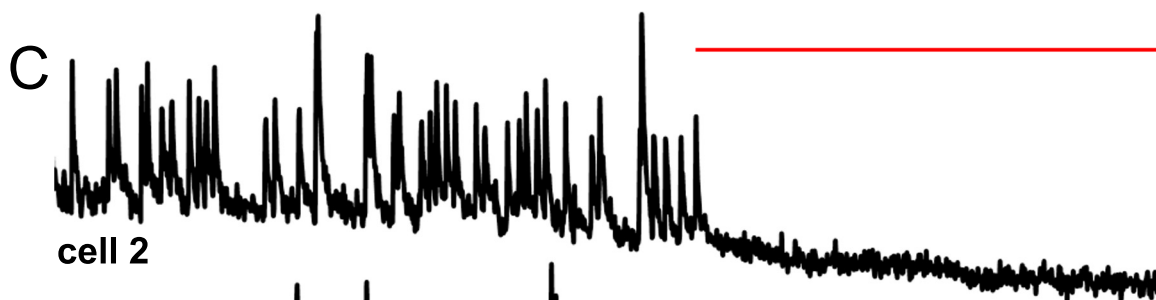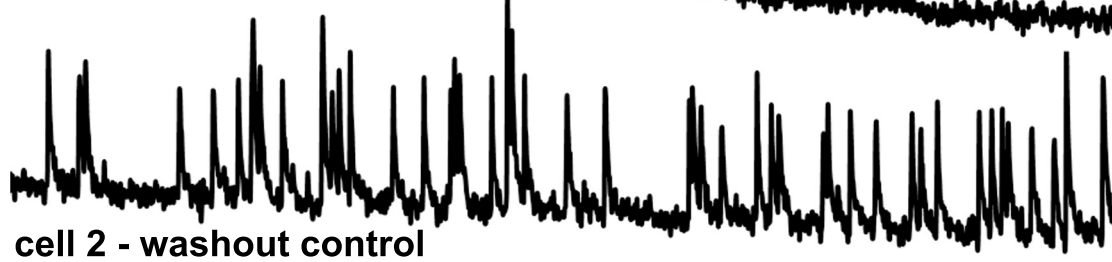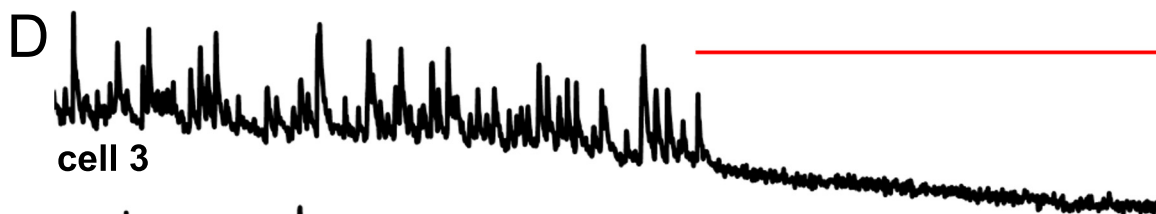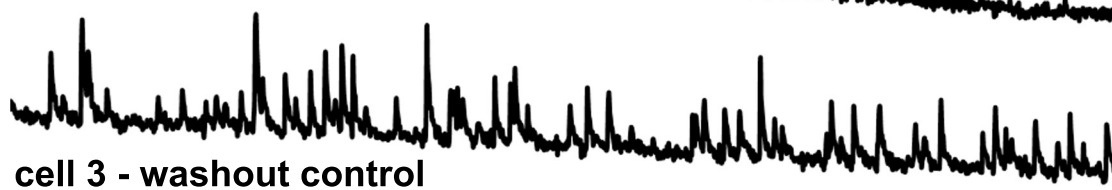

Supplement: S8 Fig — a, Hippocampal neurons loaded with OGB1-AM, average intensity projection, brightness and contrast adjusted. b-d, Three spontaneous active neurons (ROI 1–3) show typical calcium spike-behavior (fast onset, slower decaying of the signal). The neuronal activity inhibitors for glutamatergic transmission (CNQX, APV) were acutely applied to abruptly inhibit the calcium spiking behavior (upper trace in b–d). Upon washout of the inhibitors, cells recover from activity blockade and show again typical calcium spikes (lower traces in b–d). (PDF) [file pcbi.1006054.s008.pdf]
