# Supplementary material for: An open source tool for automatic spatiotemporal assessment of calcium transients and local ‘signal-close-to-noise’ activity in calcium imaging data
Source: PLoS Comput Biol. 2018 Mar 30;14(3):e1006054. doi: 10.1371/journal.pcbi.1006054 (PMC5895056; doi:10.1371/journal.pcbi.1006054)

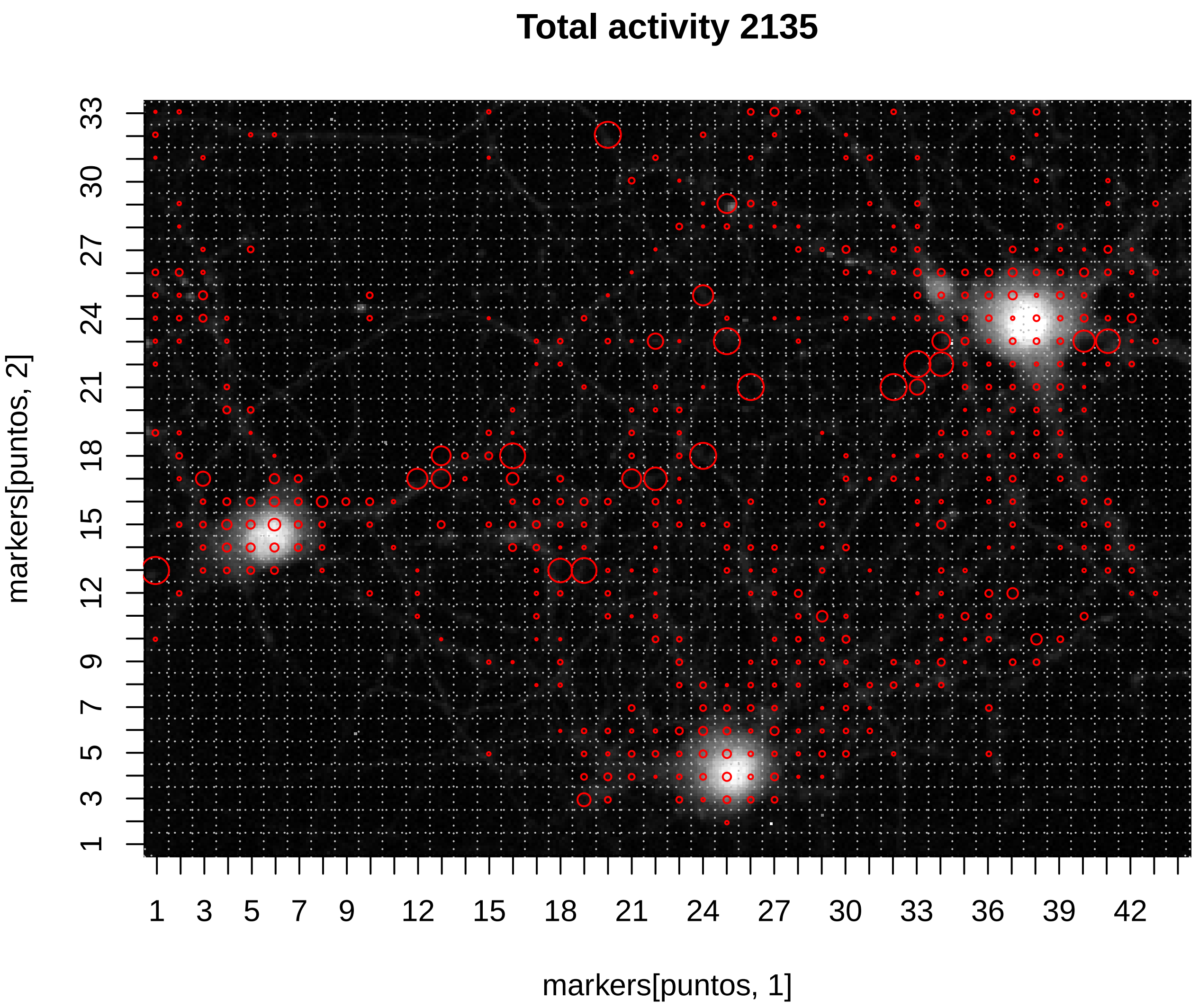
Total activity 2135
markers[puntos, 2]
1 3 5 7 9 12 15 18 21 24 27 30 33
markers[puntos, 1]
1 3 5 7 9 12 15 18 21 24 27 30 33 36 39 42

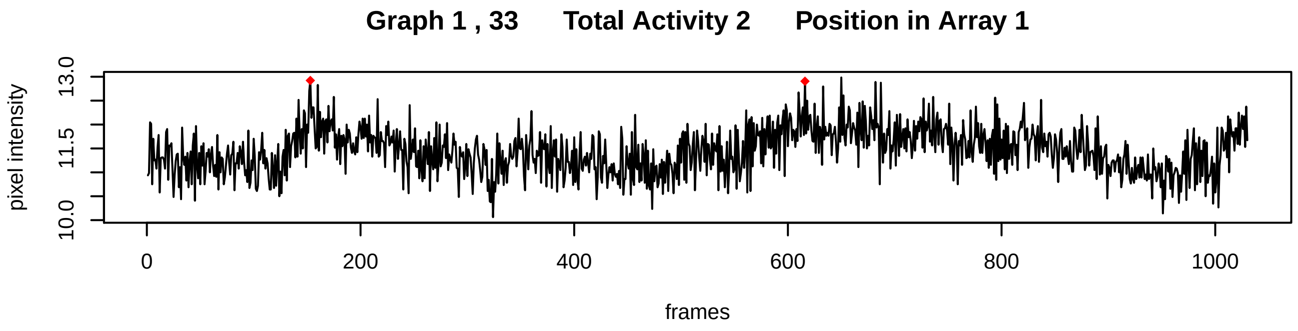
Graph 1 , 33 Total Activity 2 Position in Array 1
pixel intensity
10.0
11.5
13.0
0
200
400
600
800
1000
frames

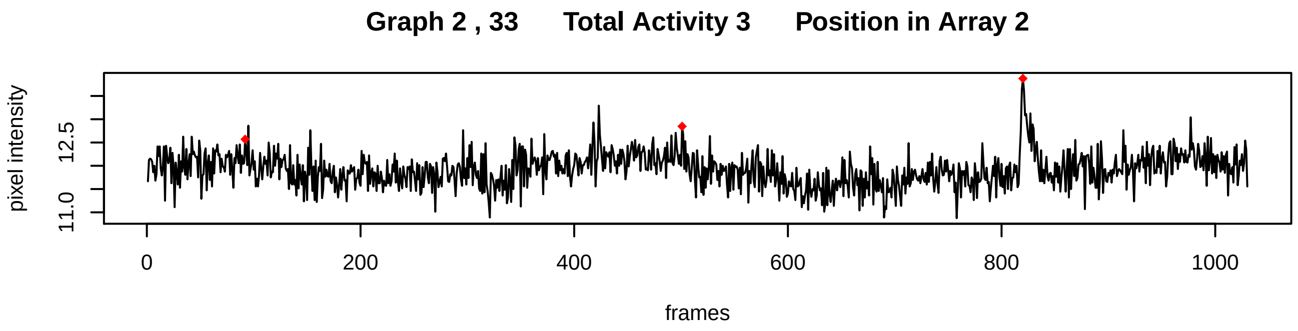
Graph 2 , 33 Total Activity 3 Position in Array 2
pixel intensity
11.0
12.5
0
200
400
600
800
1000
frames

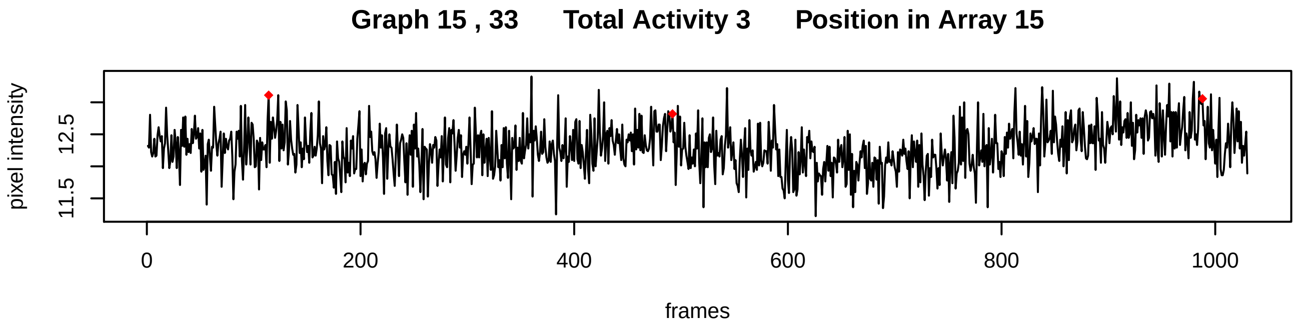
Graph 15 , 33 Total Activity 3 Position in Array 15
pixel intensity
11.5
12.5
0
200
400
600
800
1000
frames

Graph 26 , 33 Total Activity 5 Position in Array 26

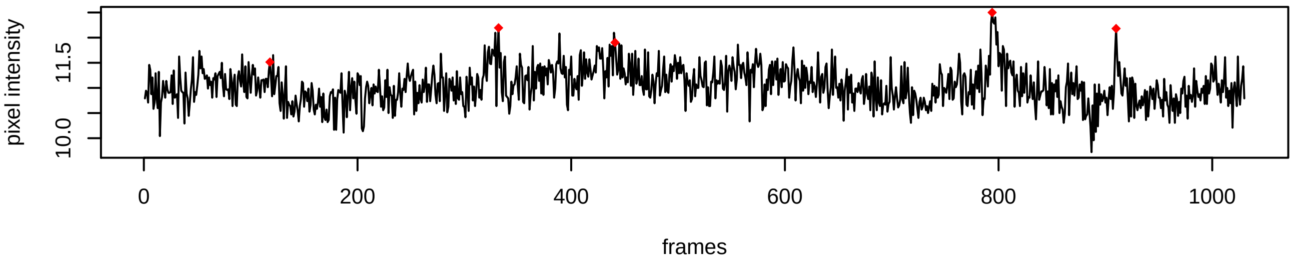

Graph 27 , 33 Total Activity 7 Position in Array 27

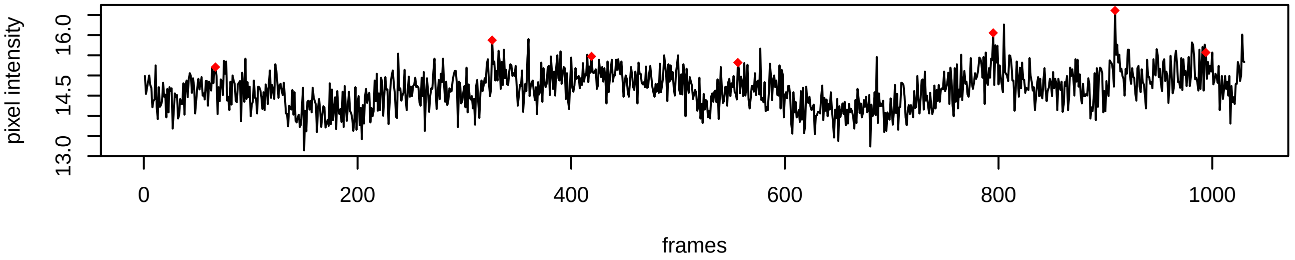

Graph 28 , 33 Total Activity 3 Position in Array 28

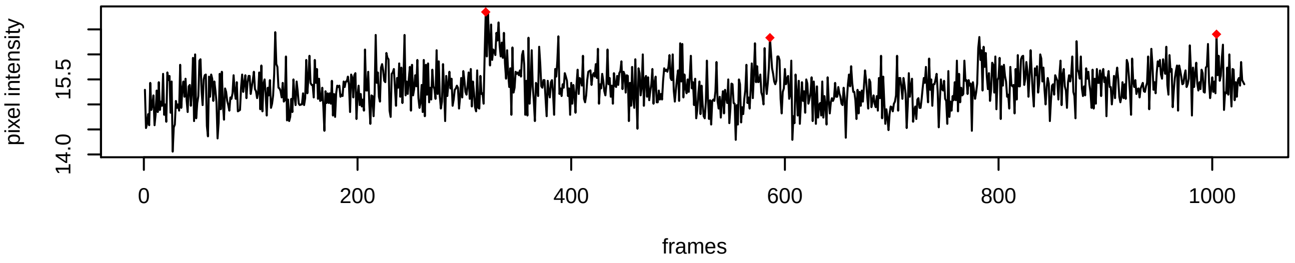

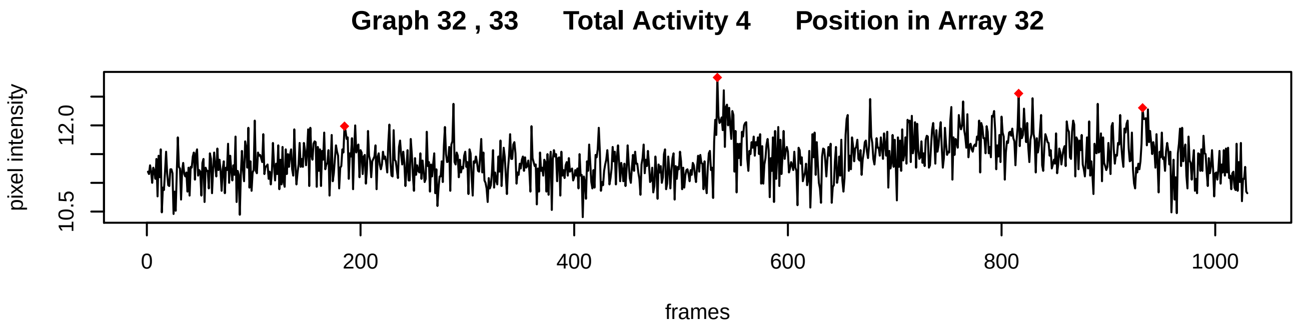
Graph 32 , 33 Total Activity 4 Position in Array 32
pixel intensity
10.5
12.0
0
200
400
600
800
1000
frames

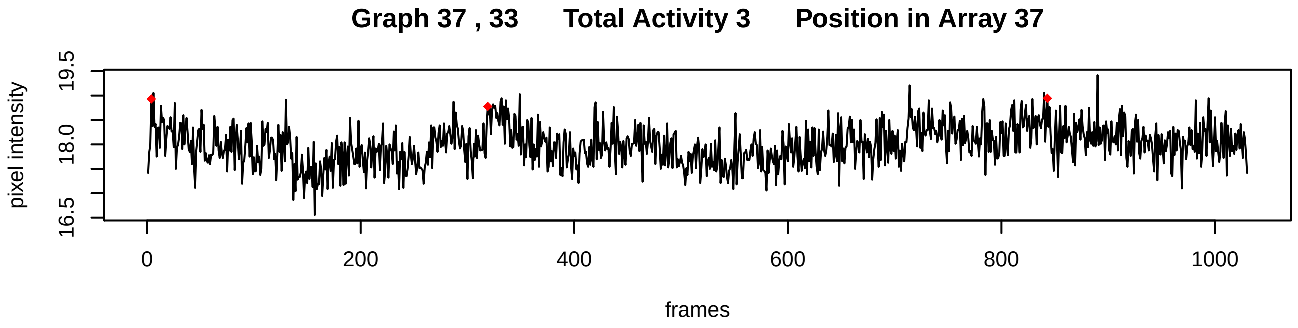
Graph 37 , 33 Total Activity 3 Position in Array 37
pixel intensity
16.5
18.0
19.5
0
200
400
600
800
1000
frames

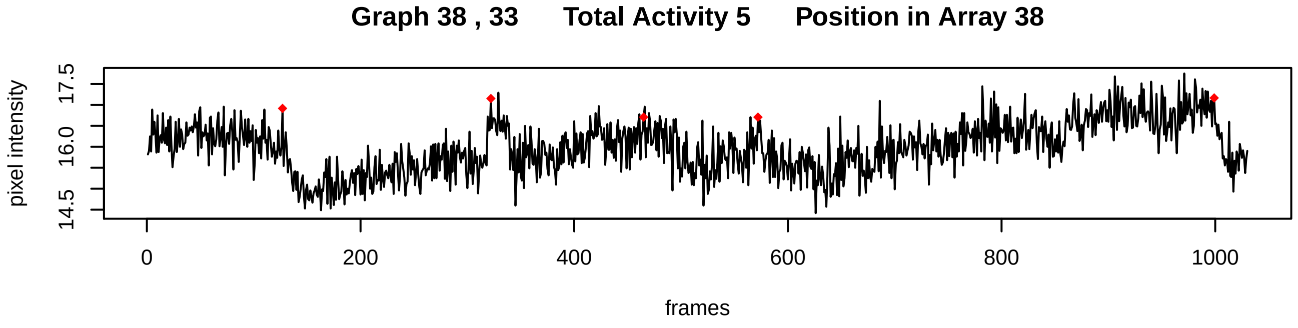
Graph 38 , 33 Total Activity 5 Position in Array 38
pixel intensity
14.5
16.0
17.5
0
200
400
600
800
1000
frames

Graph 1 , 32   Total Activity 4   Position in Array 45

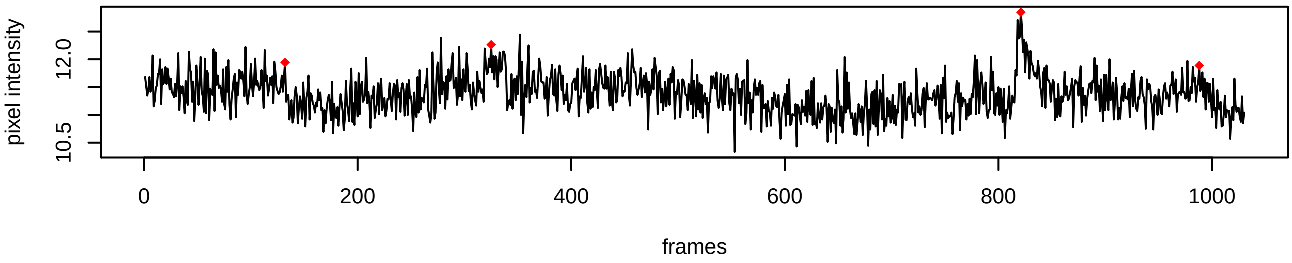

Graph 5 , 32   Total Activity 3   Position in Array 49

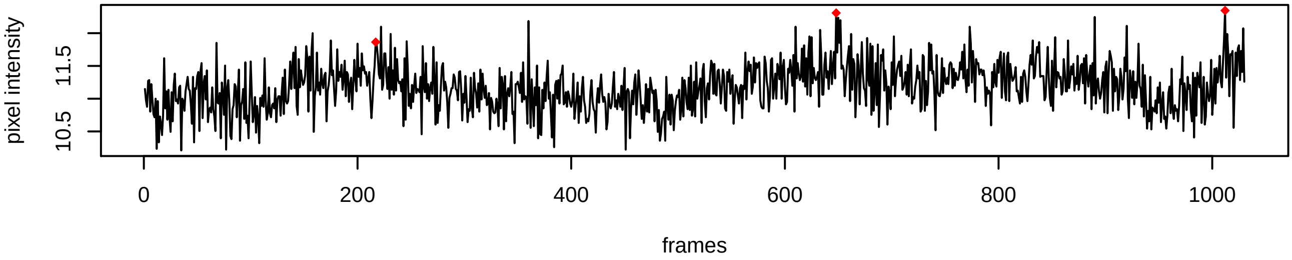

Graph 6 , 32   Total Activity 3   Position in Array 50

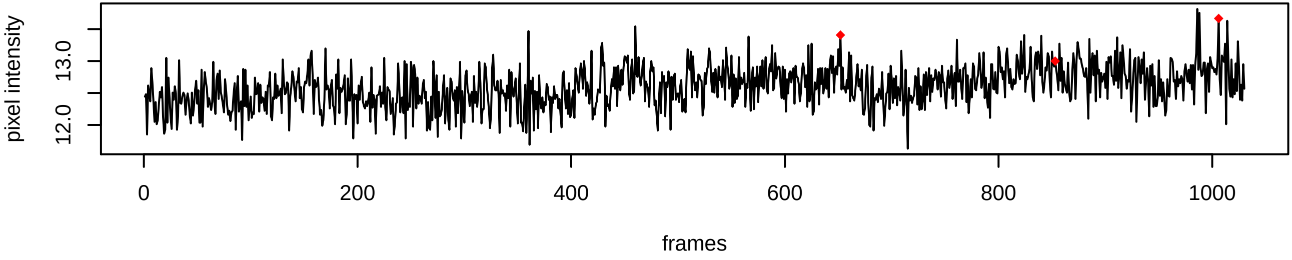

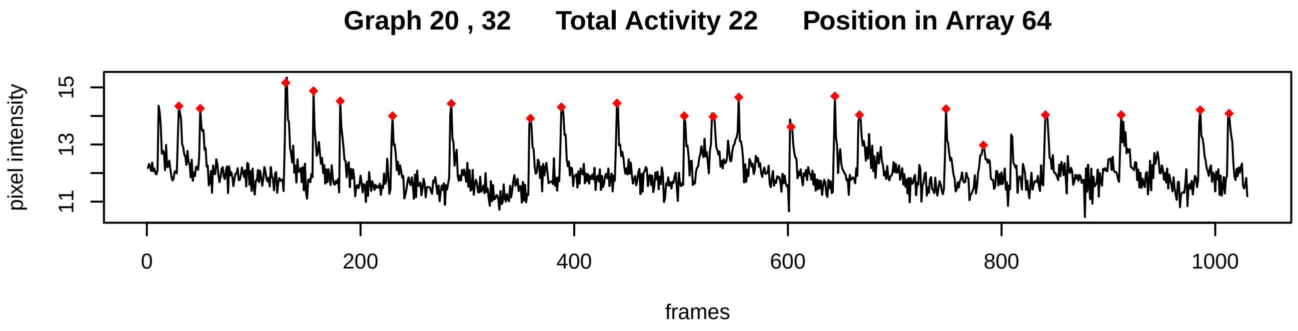
Graph 20 , 32 Total Activity 22 Position in Array 64
pixel intensity
11
13
15
0
200
400
600
800
1000
frames

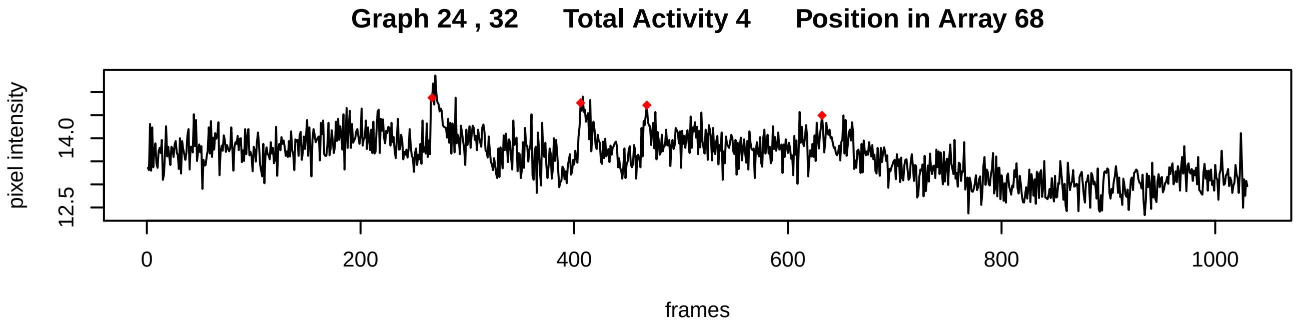
Graph 24 , 32 Total Activity 4 Position in Array 68
pixel intensity
12.5
14.0
0
200
400
600
800
1000
frames

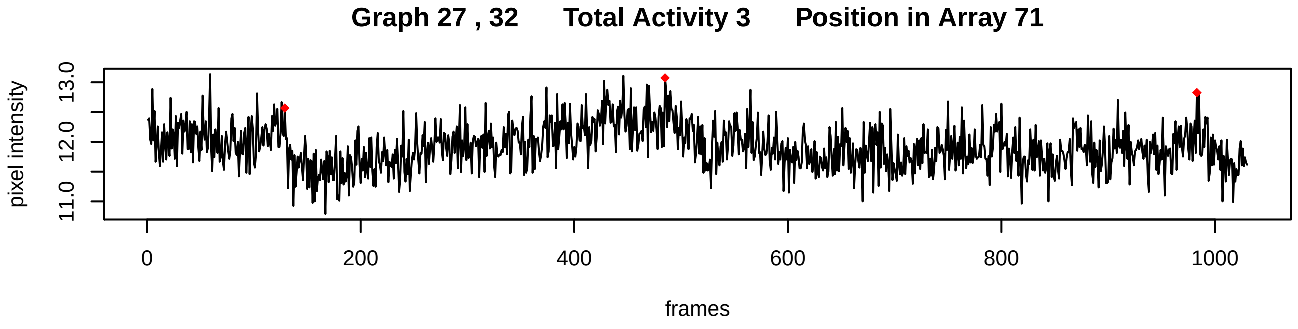
Graph 27 , 32 Total Activity 3 Position in Array 71
pixel intensity
11.0
12.0
13.0
0
200
400
600
800
1000
frames

Graph 30 , 32   Total Activity 2   Position in Array 74

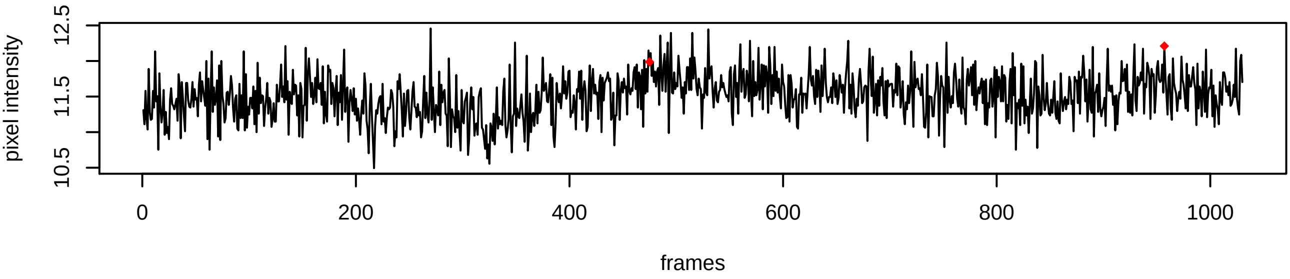

Graph 38 , 32   Total Activity 2   Position in Array 82

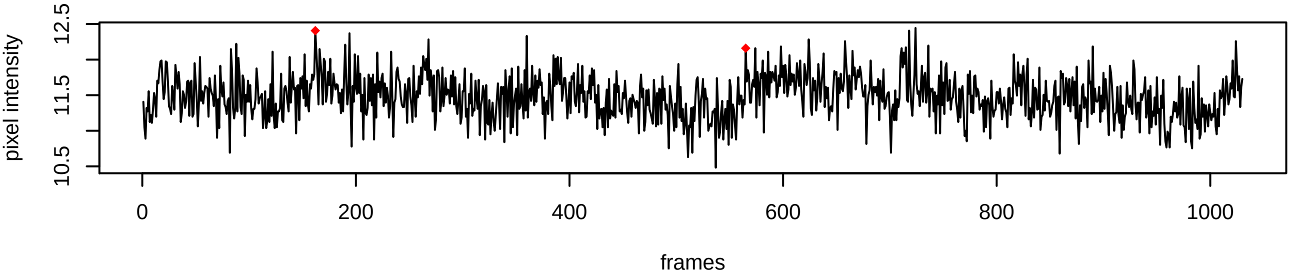

Graph 1 , 31   Total Activity 2   Position in Array 89

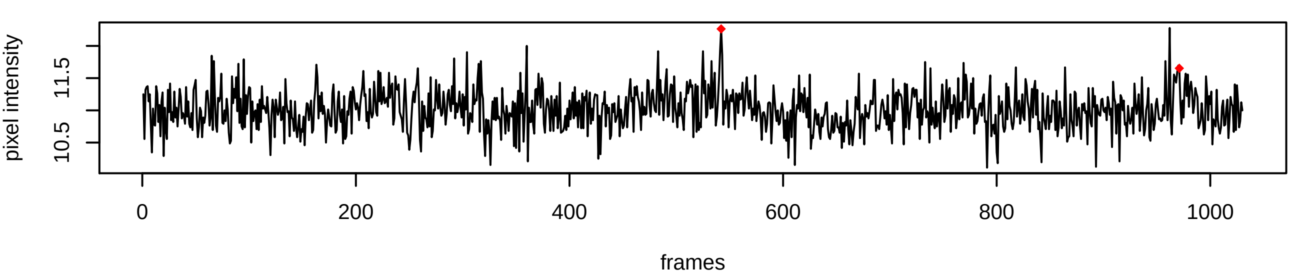

Graph 3 , 31   Total Activity 3   Position in Array 91

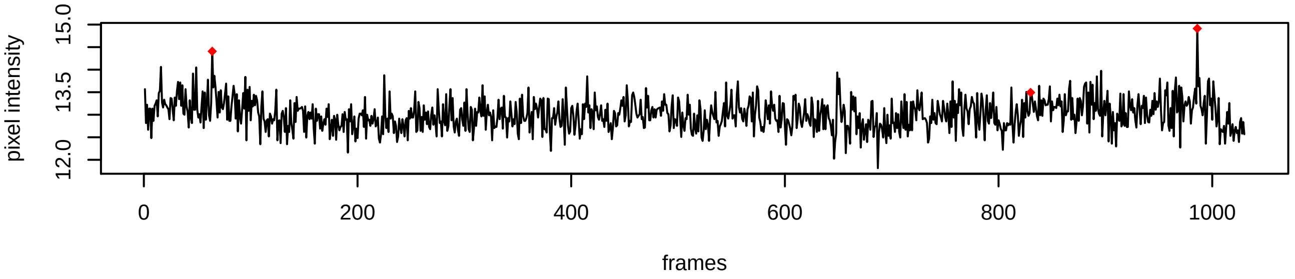

Graph 15 , 31   Total Activity 2   Position in Array 103

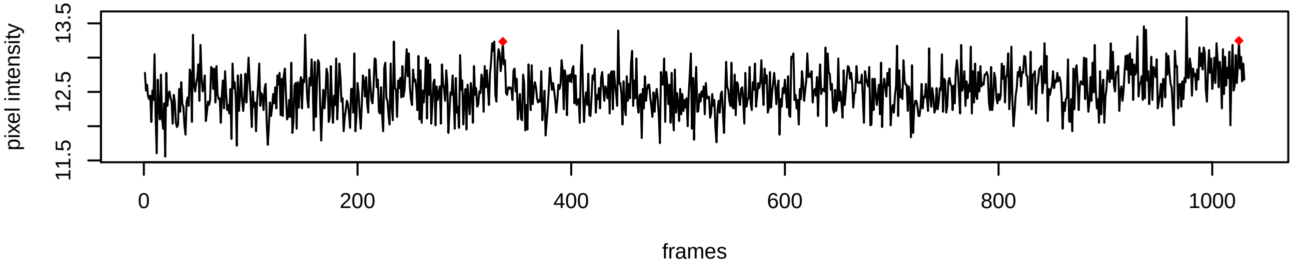

Graph 22 , 31   Total Activity 4   Position in Array 110

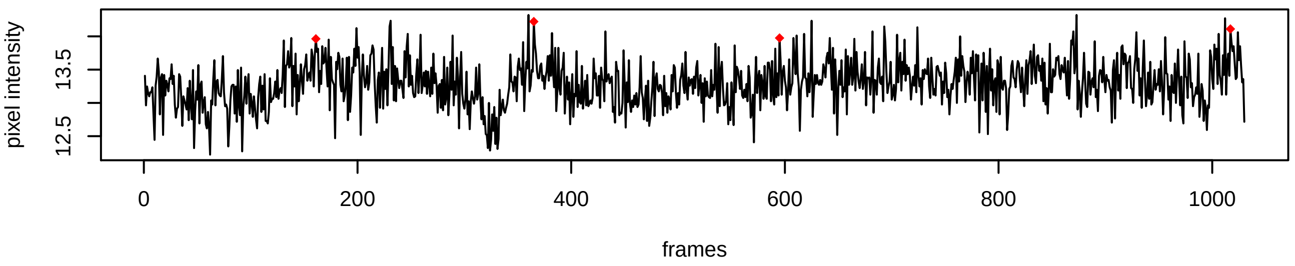

Graph 26 , 31 Total Activity 3 Position in Array 114

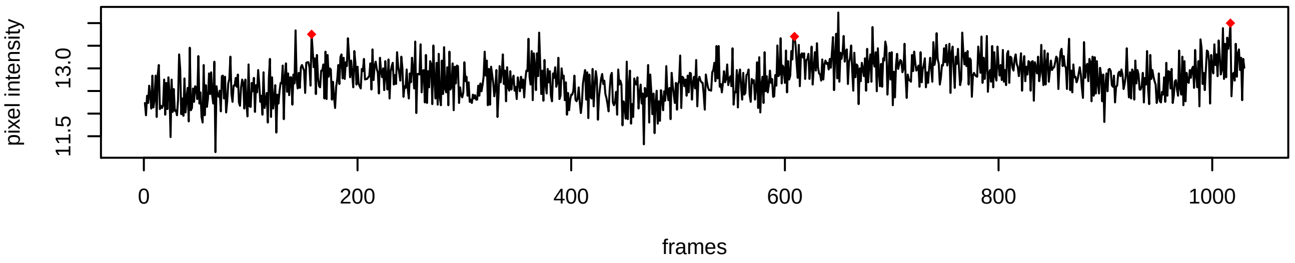

Graph 30 , 31 Total Activity 3 Position in Array 118

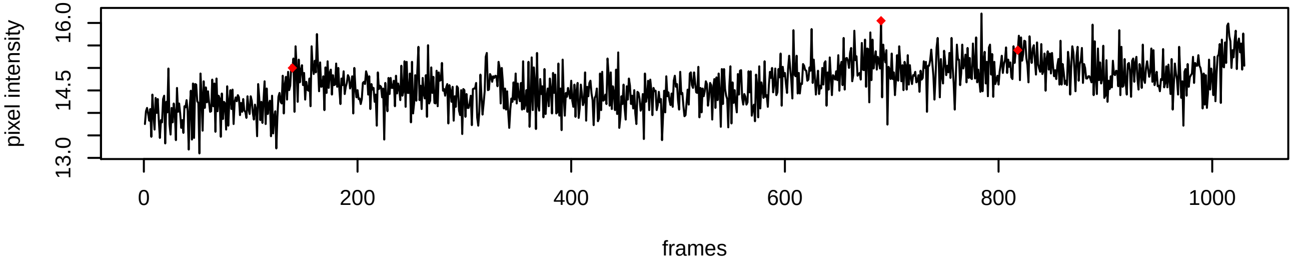

Graph 31 , 31 Total Activity 4 Position in Array 119

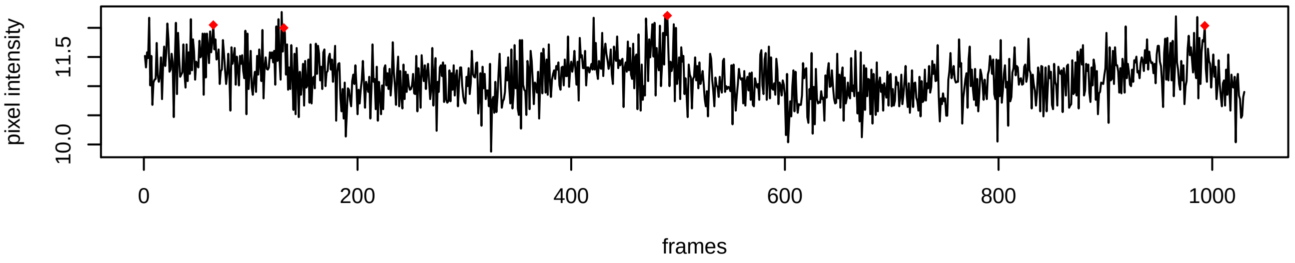

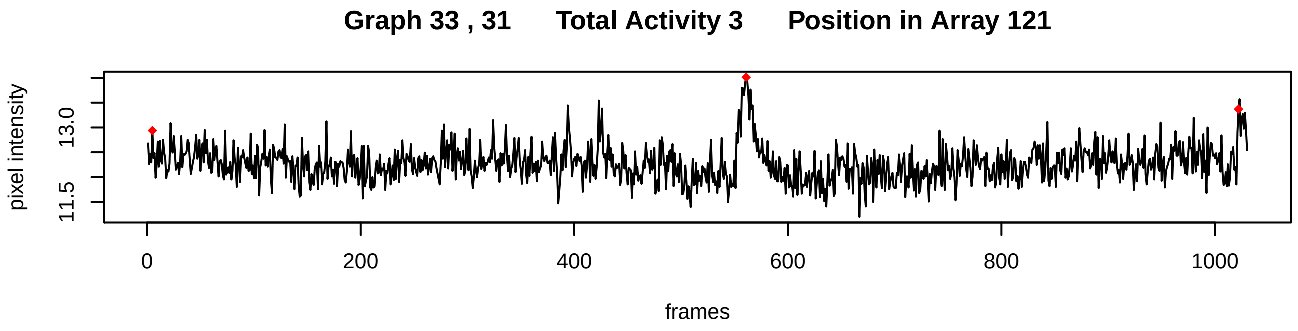
Graph 33 , 31 Total Activity 3 Position in Array 121
pixel intensity
11.5
13.0
0
200
400
600
800
1000
frames

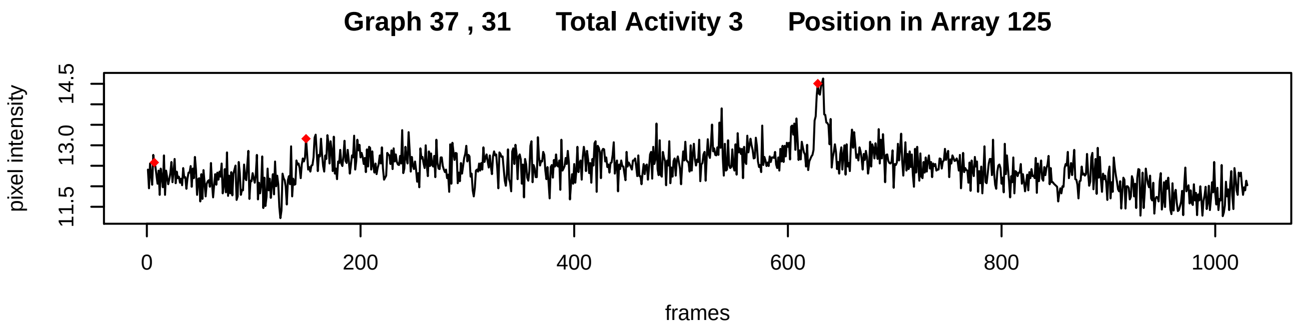
Graph 37 , 31 Total Activity 3 Position in Array 125
pixel intensity
11.5
13.0
14.5
0
200
400
600
800
1000
frames

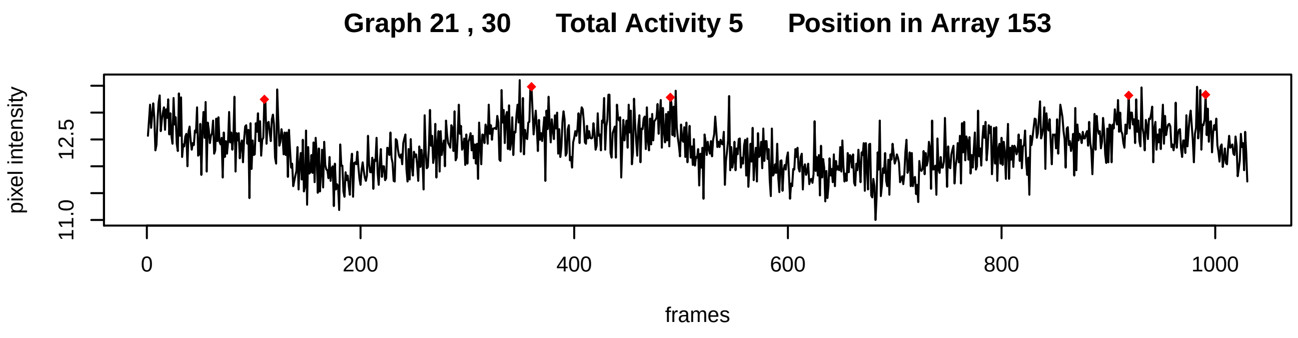
Graph 21 , 30 Total Activity 5 Position in Array 153
pixel intensity
11.0
12.5
0
200
400
600
800
1000
frames

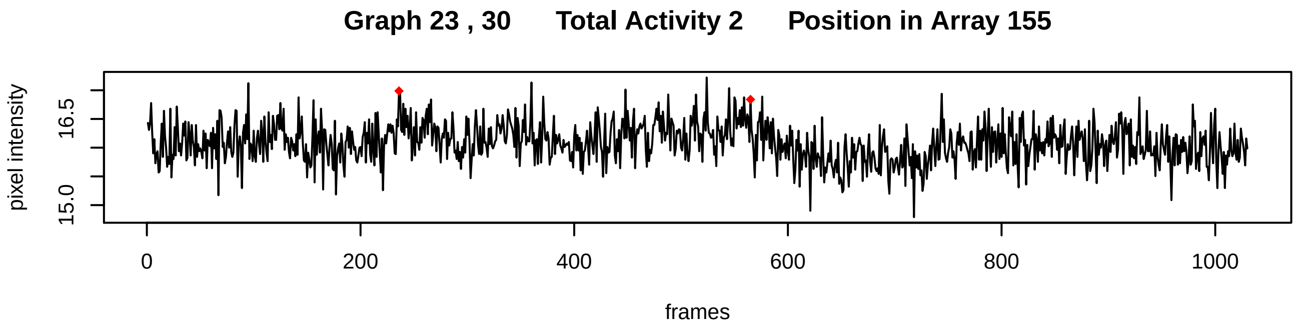
Graph 23 , 30 Total Activity 2 Position in Array 155
pixel intensity
15.0
16.5
0
200
400
600
800
1000
frames

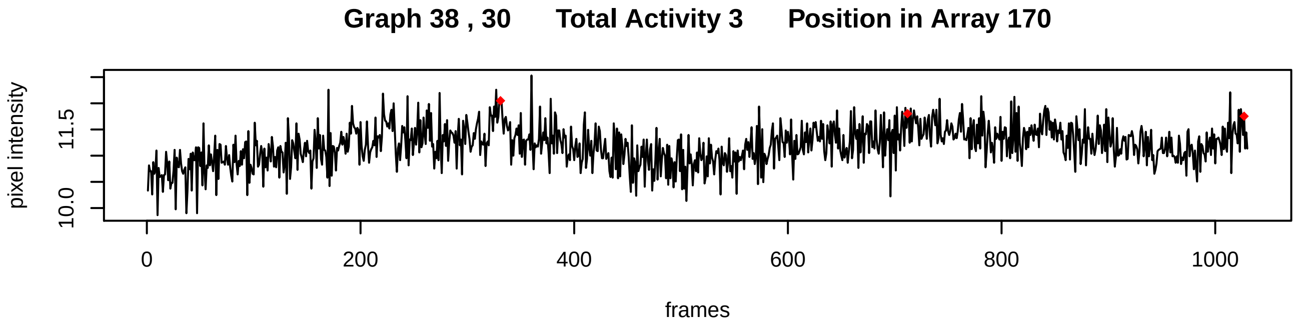
Graph 38 , 30 Total Activity 3 Position in Array 170
pixel intensity
10.0
11.5
0
200
400
600
800
1000
frames

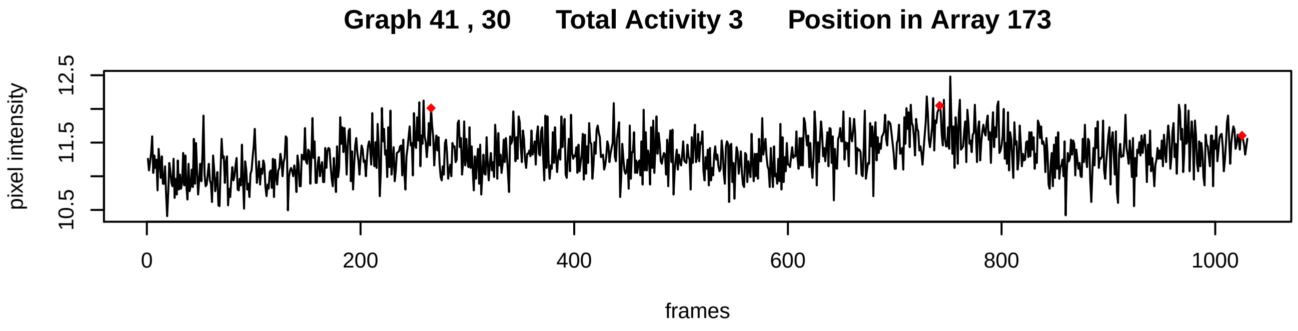
Graph 41 , 30 Total Activity 3 Position in Array 173
pixel intensity
10.5
11.5
12.5
0
200
400
600
800
1000
frames

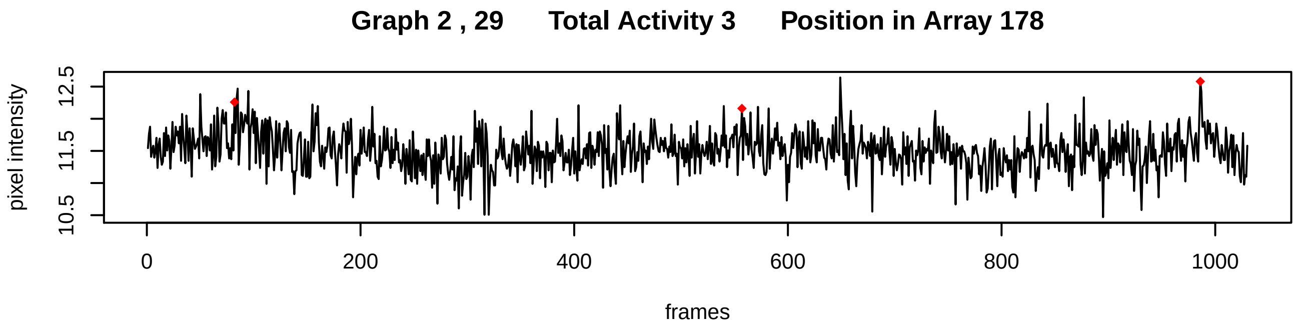
Graph 2 , 29 Total Activity 3 Position in Array 178
pixel intensity
10.5
11.5
12.5
0
200
400
600
800
1000
frames

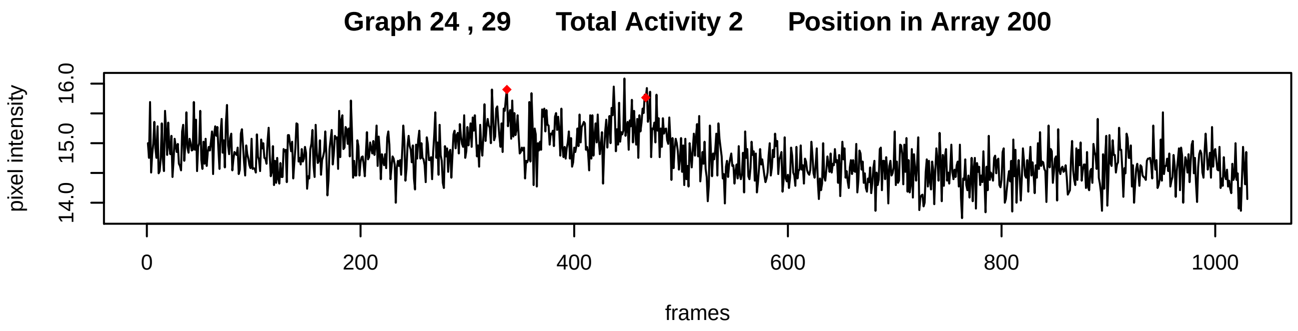
Graph 24 , 29 Total Activity 2 Position in Array 200
pixel intensity
14.0
15.0
16.0
0
200
400
600
800
1000
frames

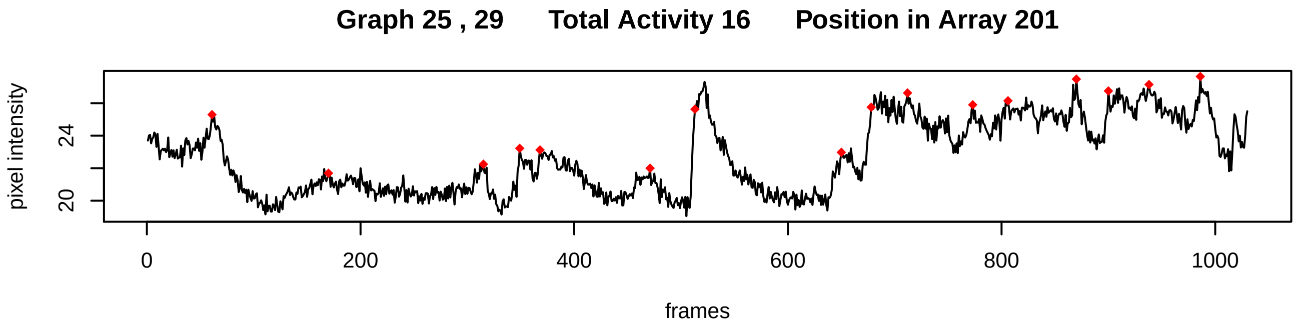
Graph 25 , 29 Total Activity 16 Position in Array 201
pixel intensity
20
24
0
200
400
600
800
1000
frames

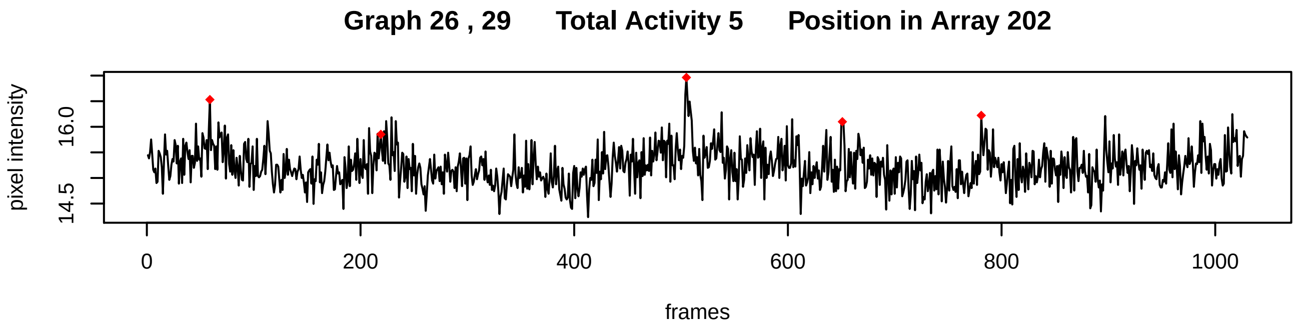
Graph 26 , 29 Total Activity 5 Position in Array 202
pixel intensity
14.5
16.0
0
200
400
600
800
1000
frames

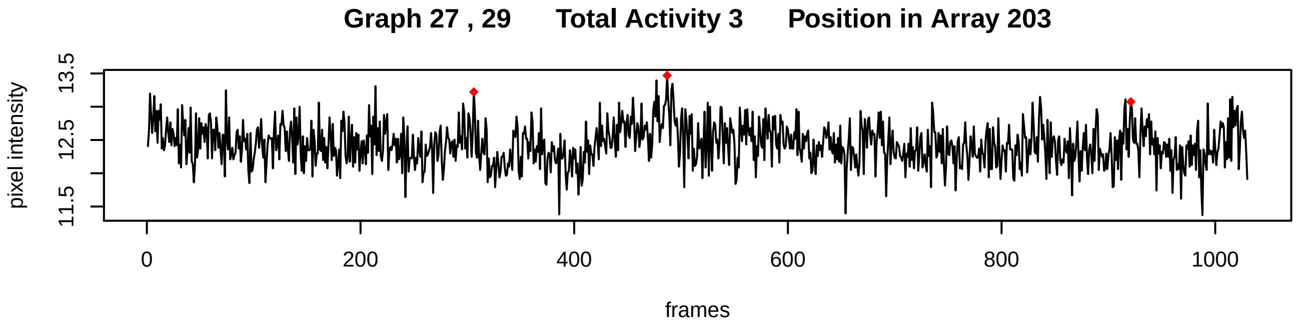
Graph 27 , 29 Total Activity 3 Position in Array 203
pixel intensity
11.5
12.5
13.5
0
200
400
600
800
1000
frames

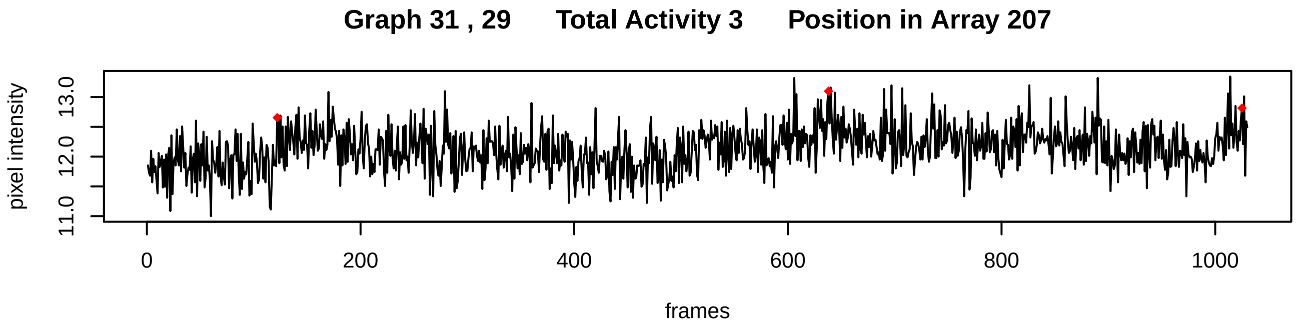
Graph 31 , 29 Total Activity 3 Position in Array 207
pixel intensity
11.0
12.0
13.0
0
200
400
600
800
1000
frames

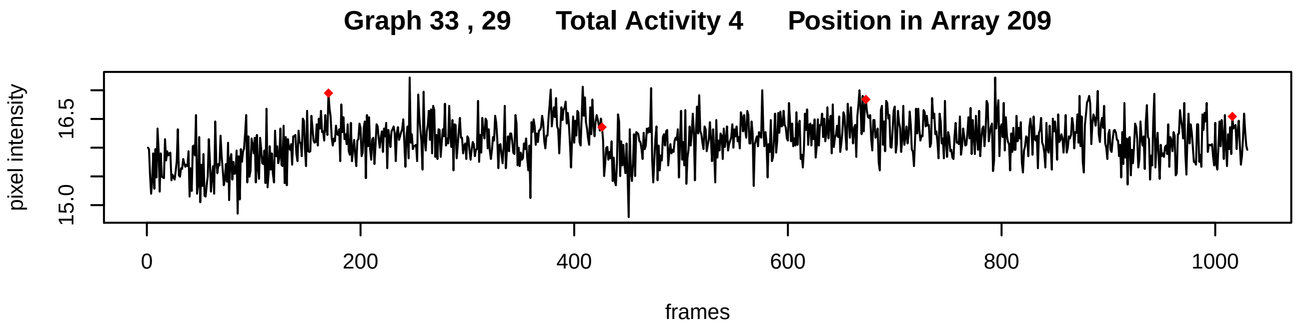
Graph 33 , 29 Total Activity 4 Position in Array 209
pixel intensity
15.0
16.5
0
200
400
600
800
1000
frames

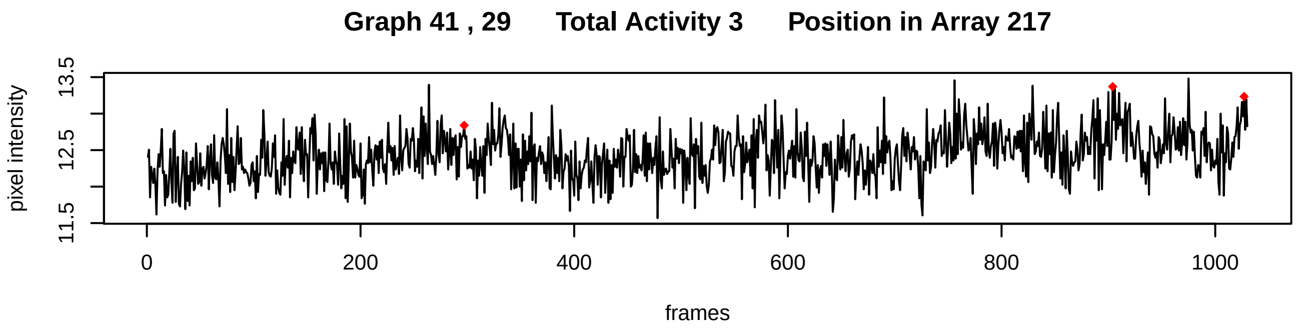
Graph 41 , 29 Total Activity 3 Position in Array 217
pixel intensity
11.5
12.5
13.5
0
200
400
600
800
1000
frames

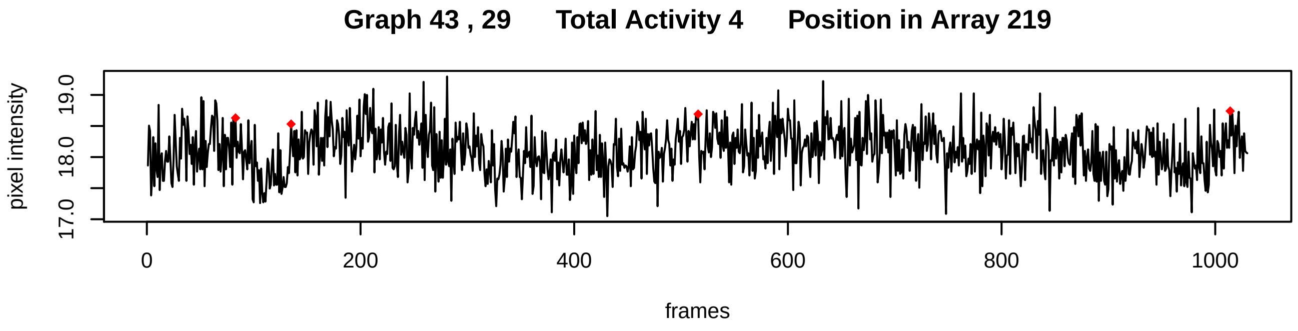
Graph 43 , 29 Total Activity 4 Position in Array 219
pixel intensity
17.0
18.0
19.0
0
200
400
600
800
1000
frames

Graph 2 , 28   Total Activity 2   Position in Array 222

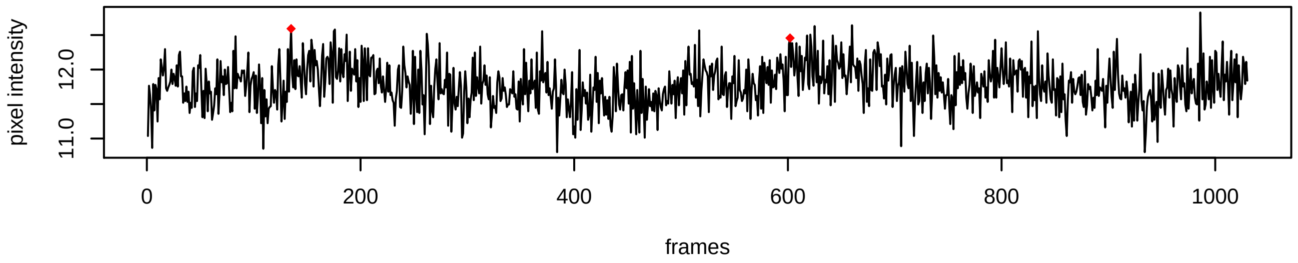

Graph 23 , 28   Total Activity 5   Position in Array 243

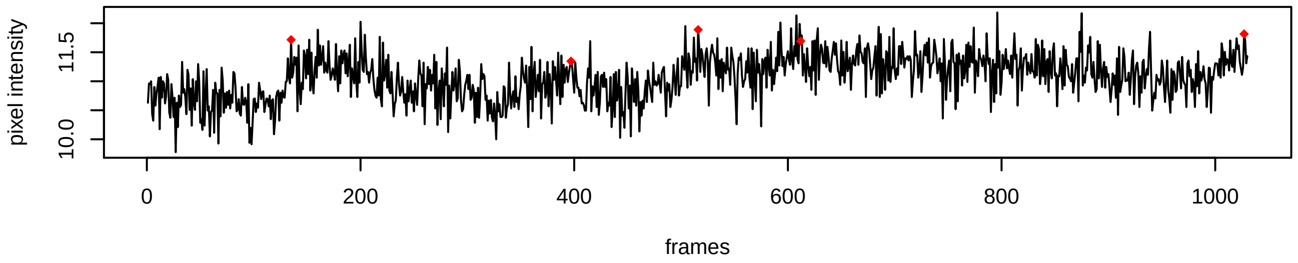

Graph 24 , 28   Total Activity 2   Position in Array 244

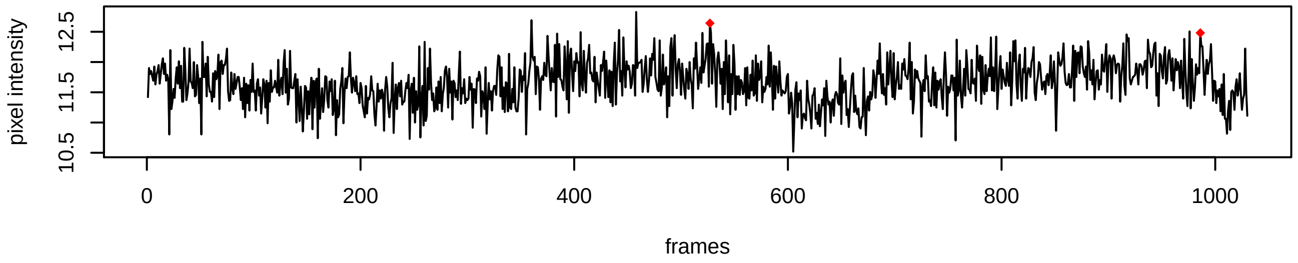

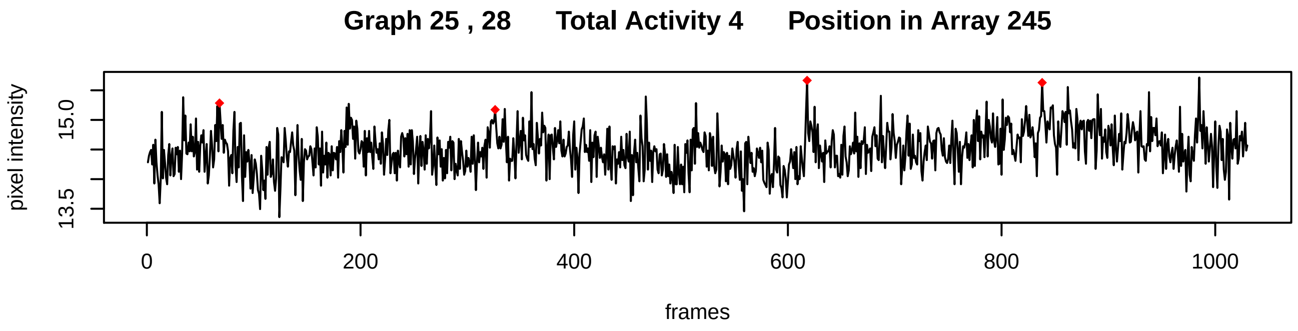
Graph 25 , 28 Total Activity 4 Position in Array 245
pixel intensity
13.5
15.0
0
200
400
600
800
1000
frames

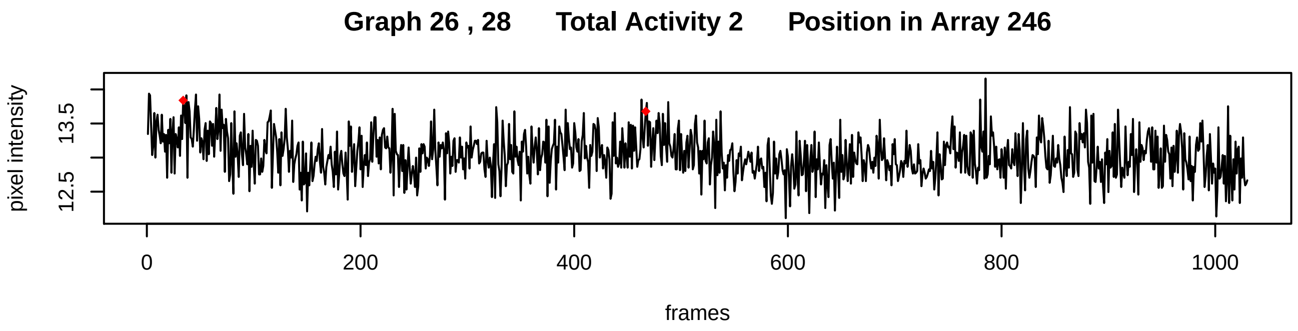
Graph 26 , 28 Total Activity 2 Position in Array 246
pixel intensity
12.5
13.5
0
200
400
600
800
1000
frames

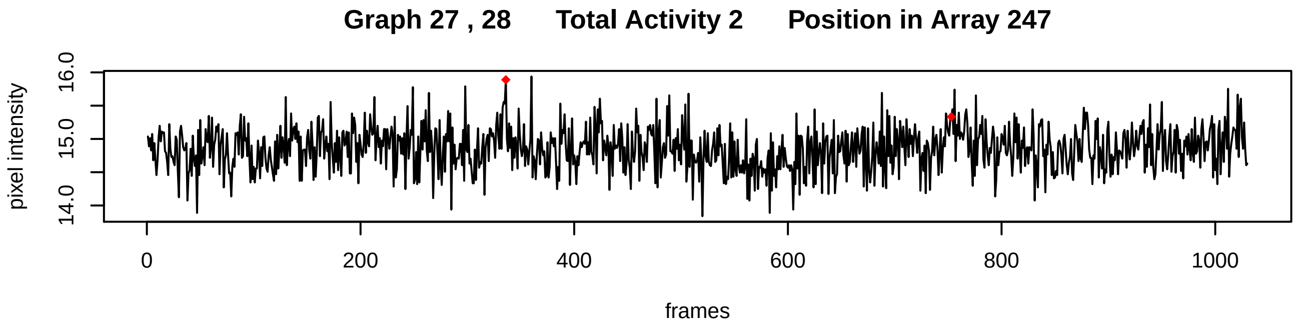
Graph 27 , 28 Total Activity 2 Position in Array 247
pixel intensity
14.0
15.0
16.0
0
200
400
600
800
1000
frames

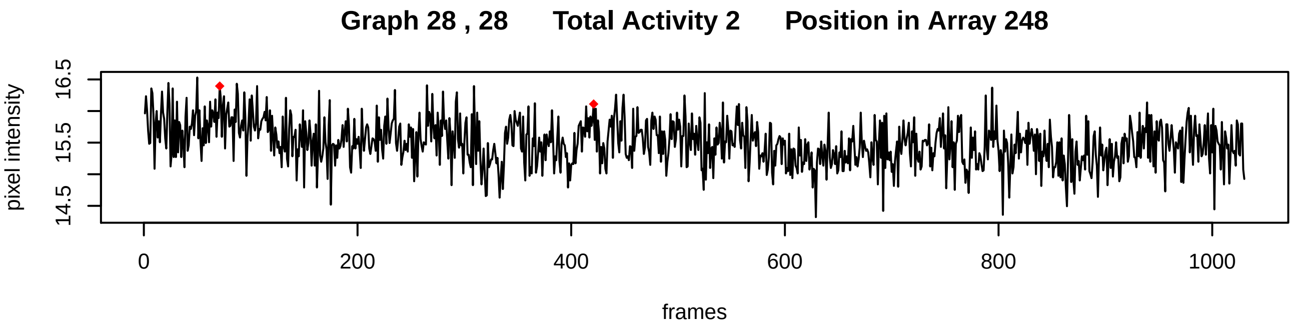
Graph 28 , 28 Total Activity 2 Position in Array 248
pixel intensity
14.5
15.5
16.5
0
200
400
600
800
1000
frames

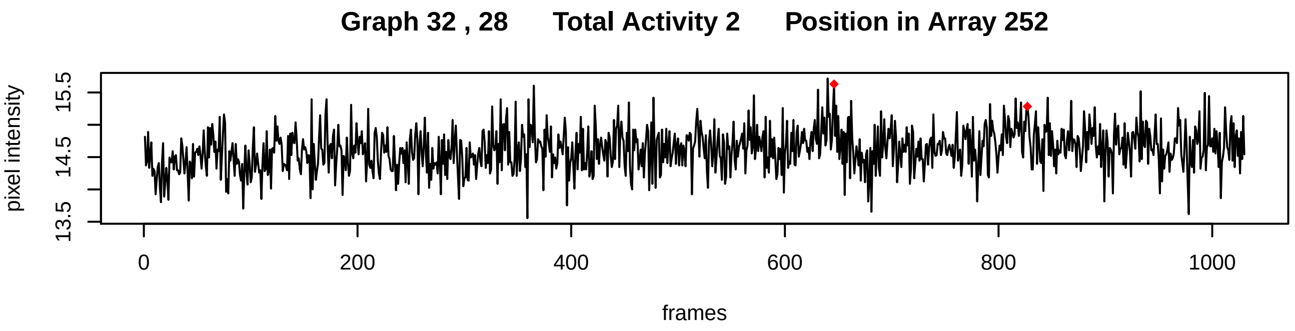
Graph 32 , 28 Total Activity 2 Position in Array 252
pixel intensity
13.5
14.5
15.5
0
200
400
600
800
1000
frames

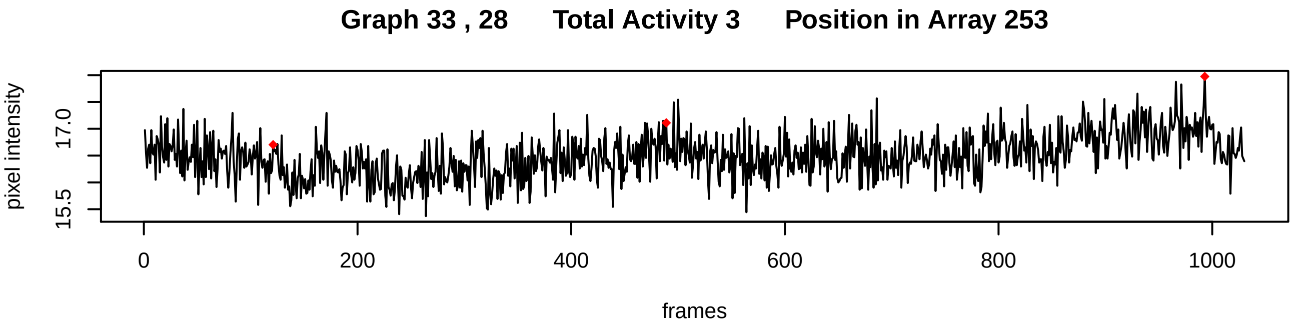
Graph 33 , 28 Total Activity 3 Position in Array 253
pixel intensity
15.5
17.0
0
200
400
600
800
1000
frames

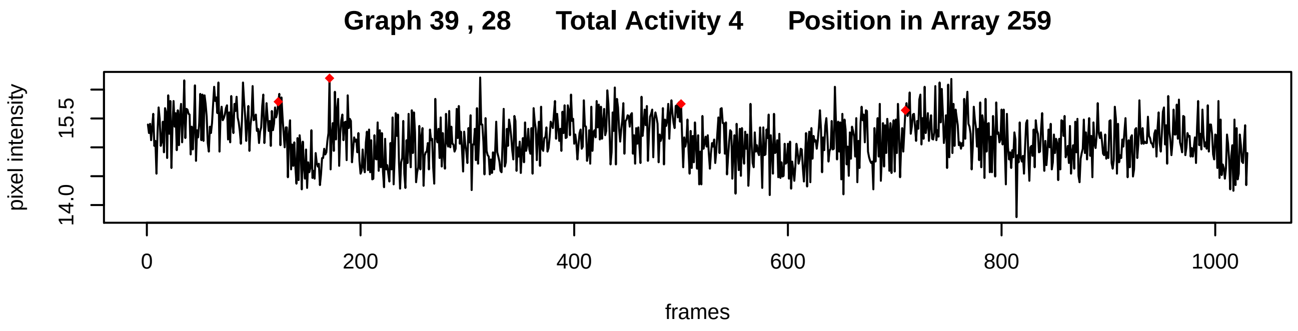
Graph 39 , 28 Total Activity 4 Position in Array 259
pixel intensity
14.0
15.5
0
200
400
600
800
1000
frames

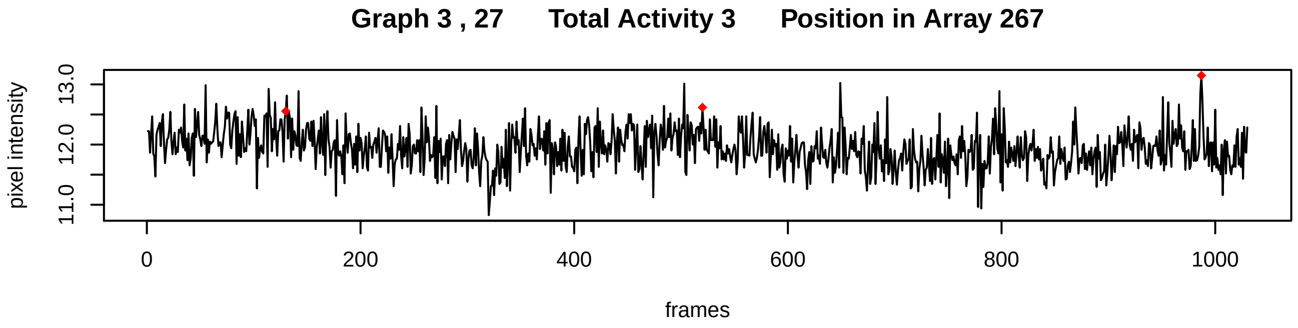
Graph 3 , 27 Total Activity 3 Position in Array 267
pixel intensity
11.0
12.0
13.0
0
200
400
600
800
1000
frames

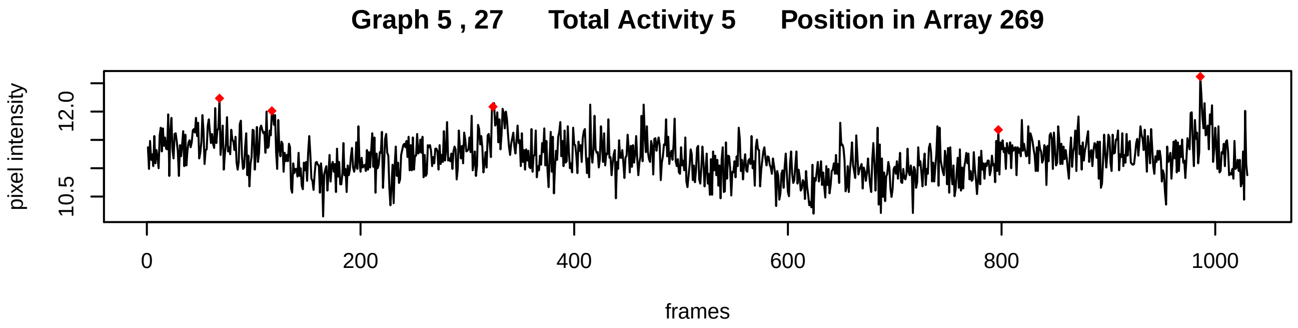
Graph 5 , 27 Total Activity 5 Position in Array 269
pixel intensity
10.5
12.0
0
200
400
600
800
1000
frames

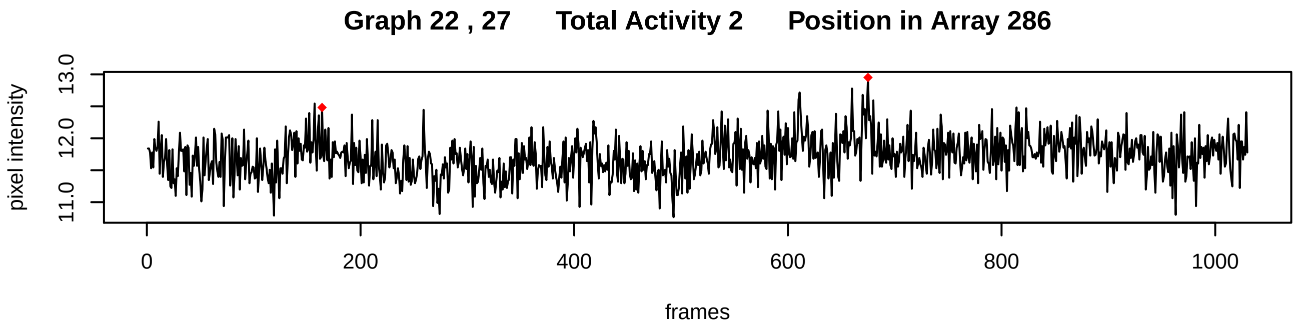
Graph 22 , 27 Total Activity 2 Position in Array 286
pixel intensity
11.0
12.0
13.0
0
200
400
600
800
1000
frames

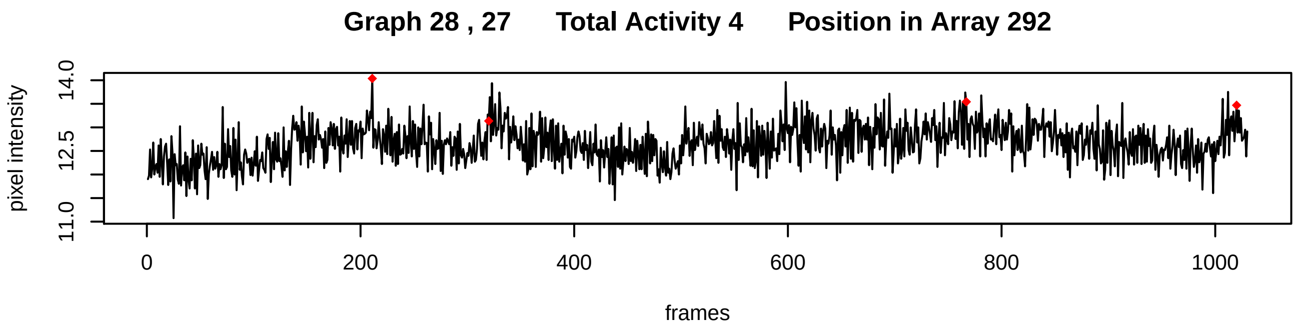
Graph 28 , 27 Total Activity 4 Position in Array 292
pixel intensity
11.0
12.5
14.0
0
200
400
600
800
1000
frames

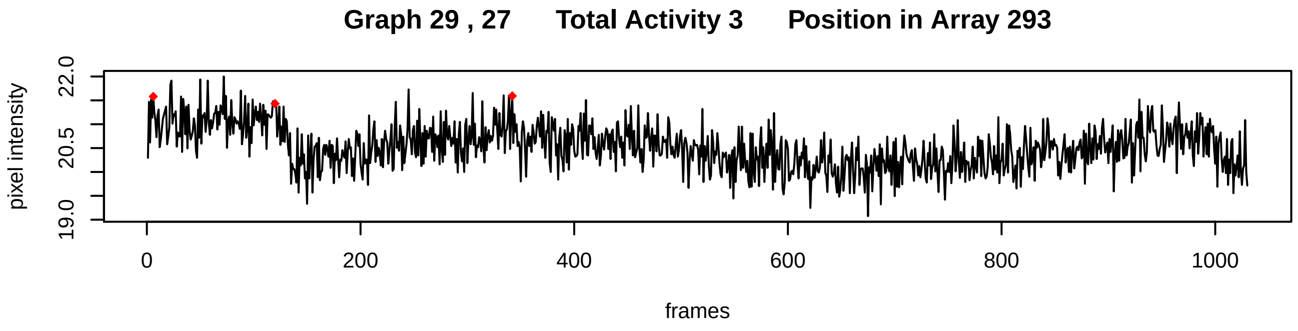
Graph 29 , 27 Total Activity 3 Position in Array 293
pixel intensity
19.0
20.5
22.0
0
200
400
600
800
1000
frames

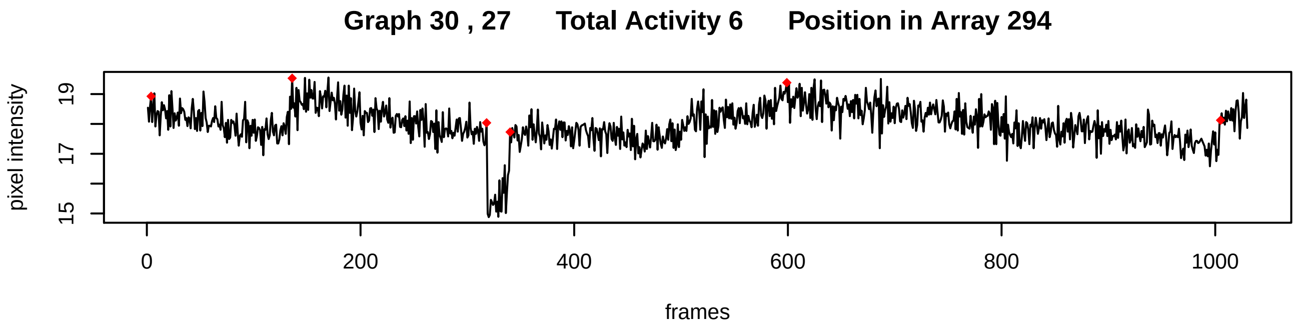
Graph 30 , 27 Total Activity 6 Position in Array 294
pixel intensity
15
17
19
0
200
400
600
800
1000
frames

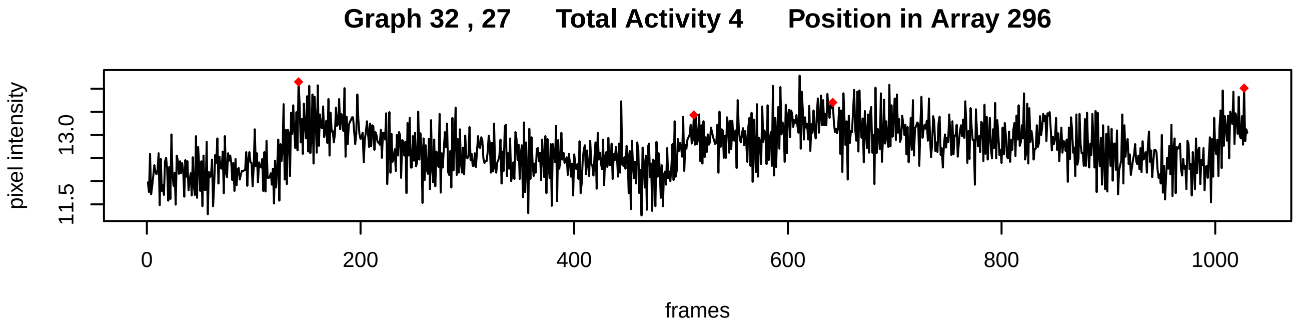
Graph 32 , 27 Total Activity 4 Position in Array 296
pixel intensity
11.5
13.0
0
200
400
600
800
1000
frames

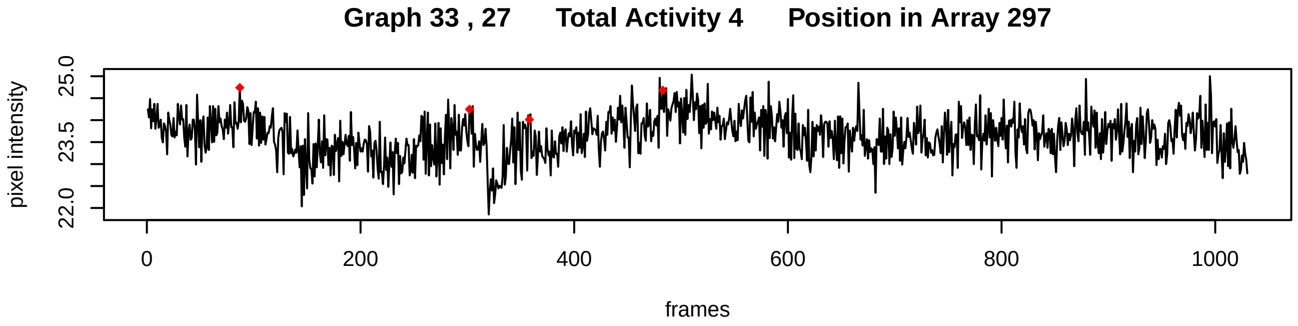
Graph 33 , 27 Total Activity 4 Position in Array 297
pixel intensity
22.0
23.5
25.0
0
200
400
600
800
1000
frames

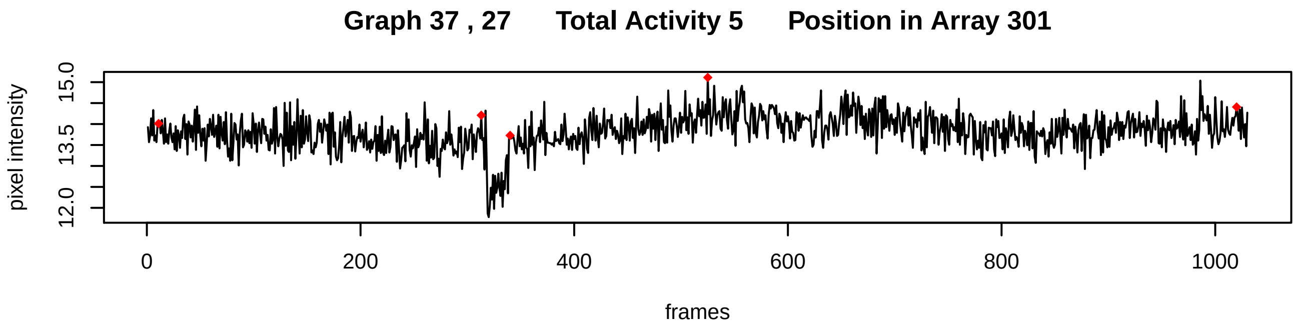
Graph 37 , 27 Total Activity 5 Position in Array 301
pixel intensity
12.0
13.5
15.0
0
200
400
600
800
1000
frames

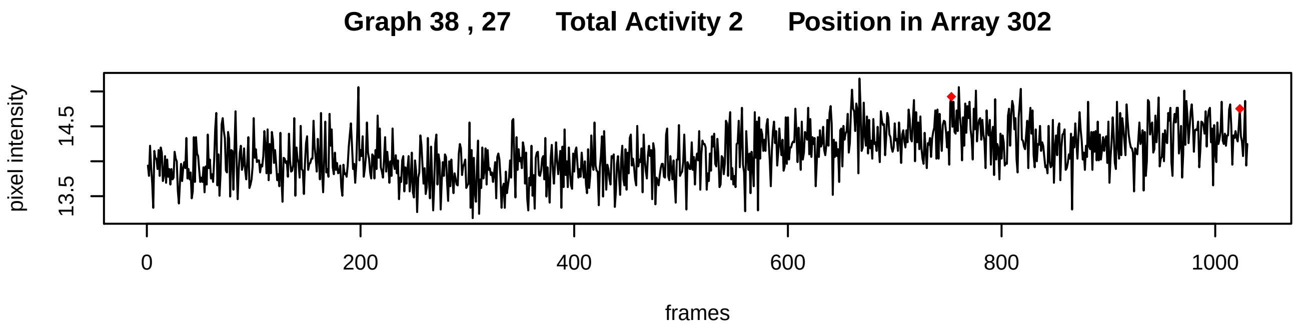
Graph 38 , 27 Total Activity 2 Position in Array 302
pixel intensity
13.5
14.5
0
200
400
600
800
1000
frames

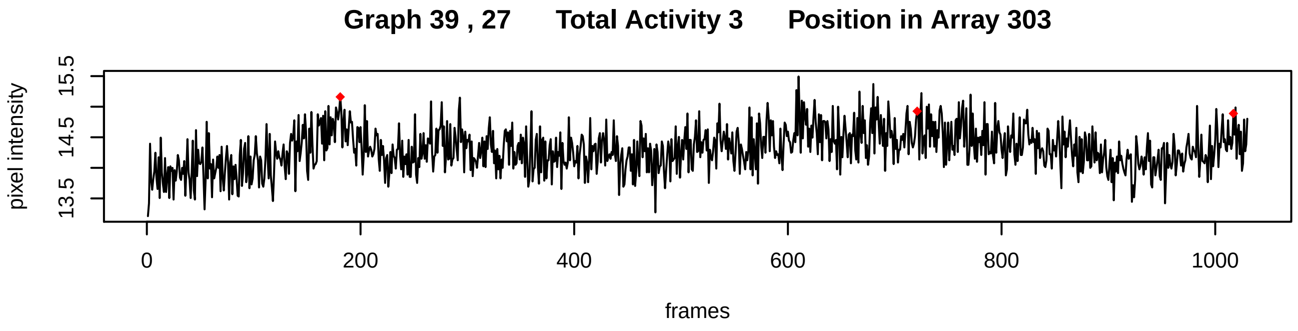
Graph 39 , 27 Total Activity 3 Position in Array 303
pixel intensity
13.5
14.5
15.5
0
200
400
600
800
1000
frames

Graph 40 , 27 Total Activity 2 Position in Array 304

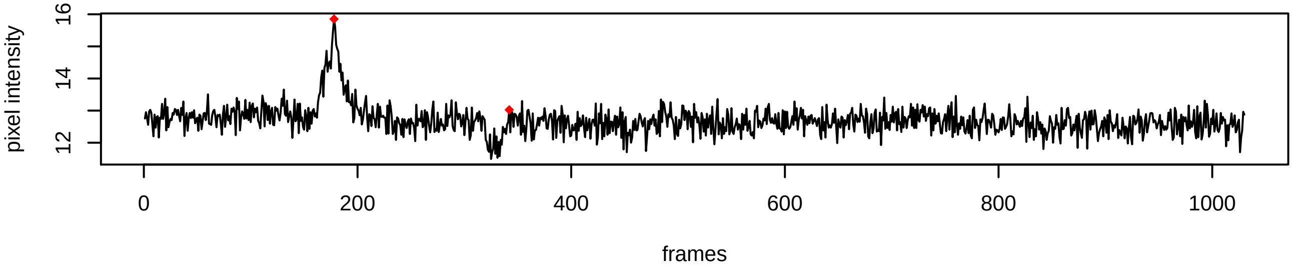

Graph 41 , 27 Total Activity 6 Position in Array 305

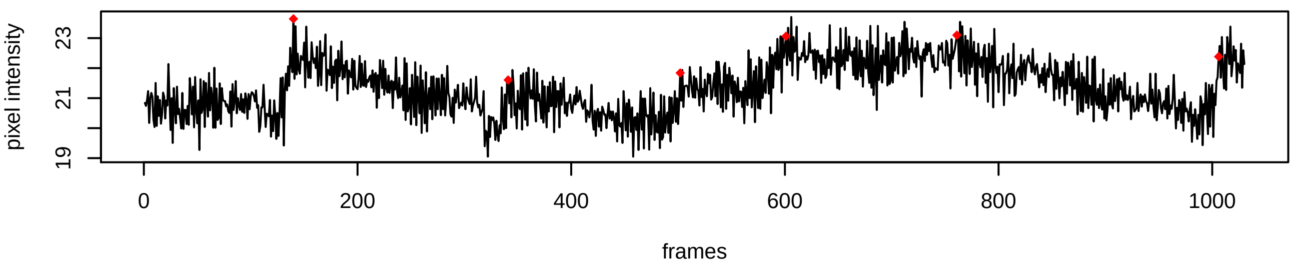

Graph 42 , 27 Total Activity 2 Position in Array 306

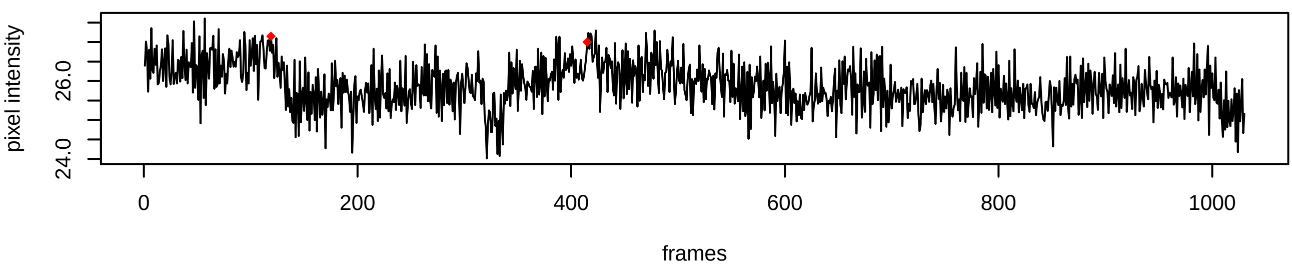

Graph 1 , 26   Total Activity 5   Position in Array 309

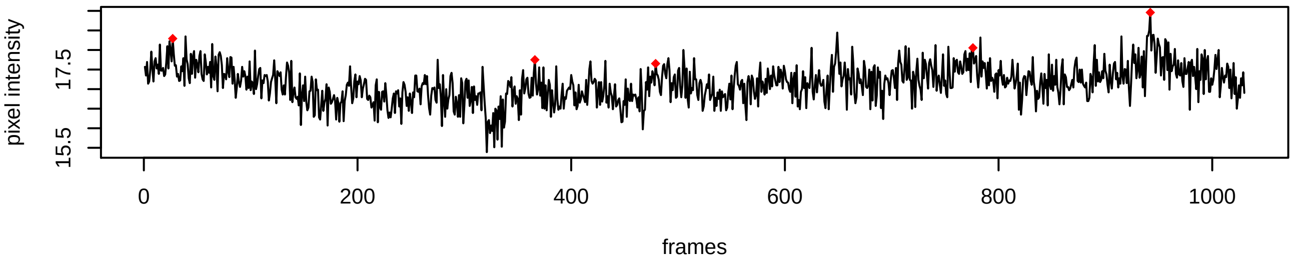

Graph 2 , 26   Total Activity 6   Position in Array 310

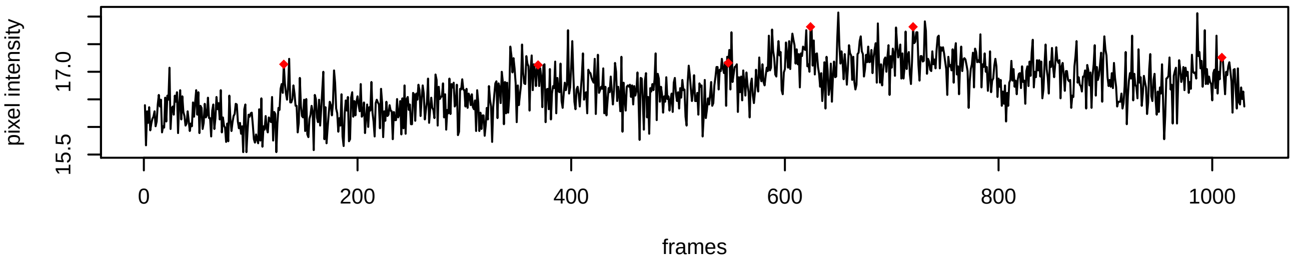

Graph 3 , 26   Total Activity 3   Position in Array 311

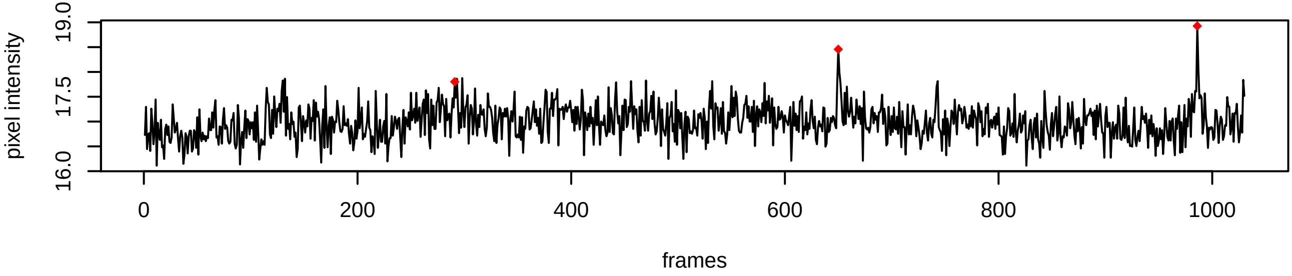

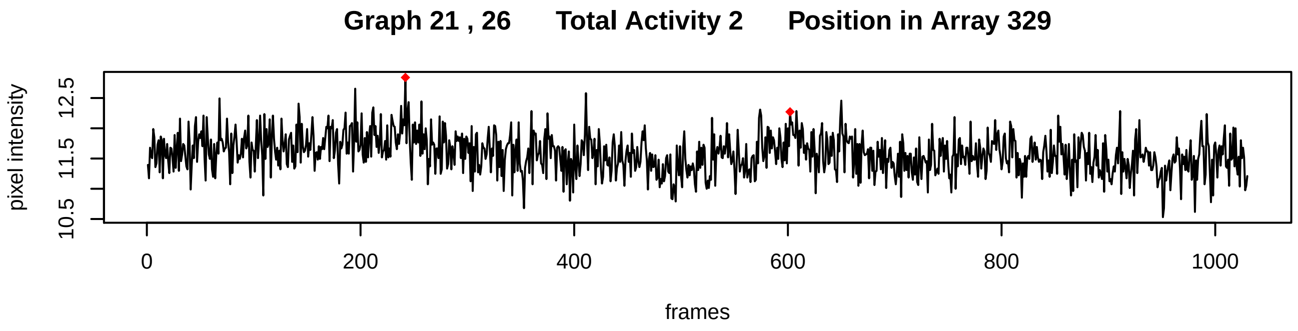
Graph 21 , 26 Total Activity 2 Position in Array 329
pixel intensity
10.5
11.5
12.5
0
200
400
600
800
1000
frames

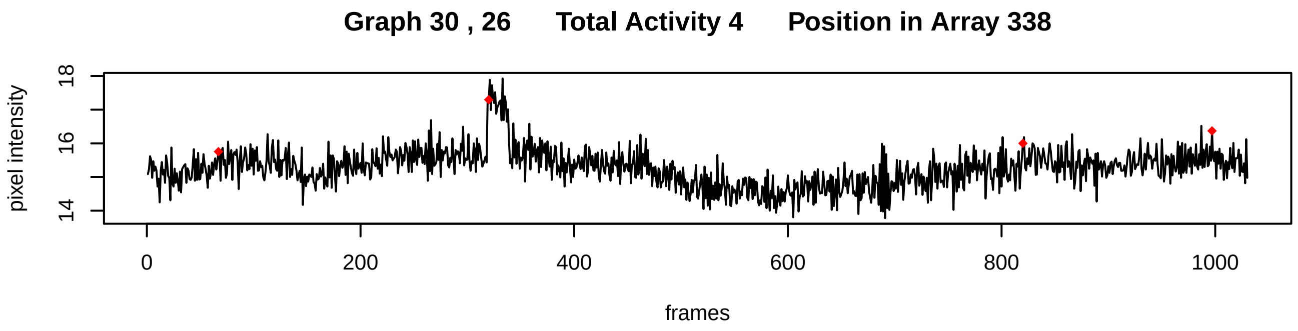
Graph 30 , 26 Total Activity 4 Position in Array 338
pixel intensity
14
16
18
0
200
400
600
800
1000
frames

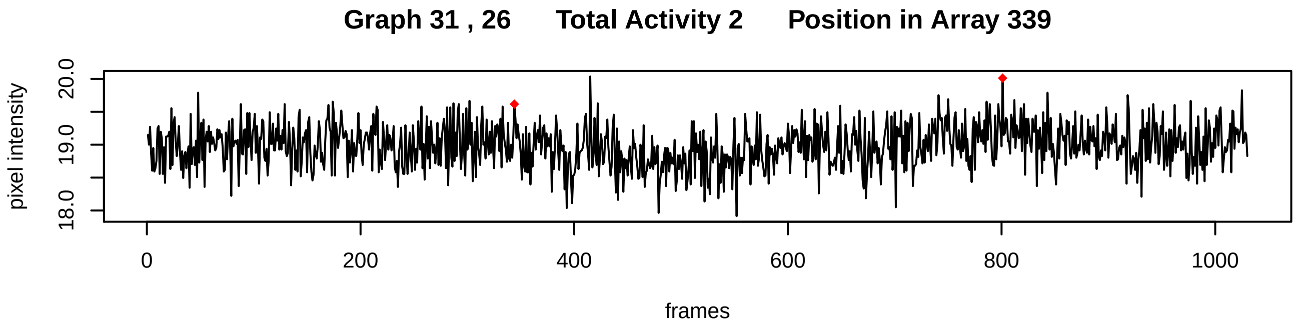
Graph 31 , 26 Total Activity 2 Position in Array 339
pixel intensity
18.0
19.0
20.0
0
200
400
600
800
1000
frames

Graph 32 , 26   Total Activity 3   Position in Array 340

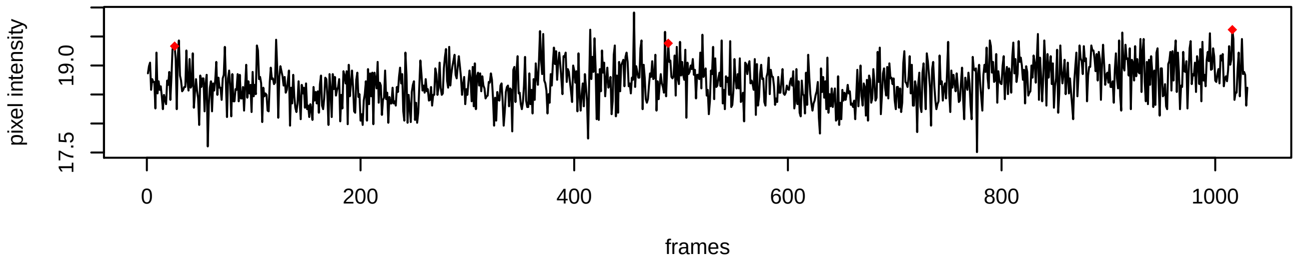

Graph 33 , 26   Total Activity 6   Position in Array 341

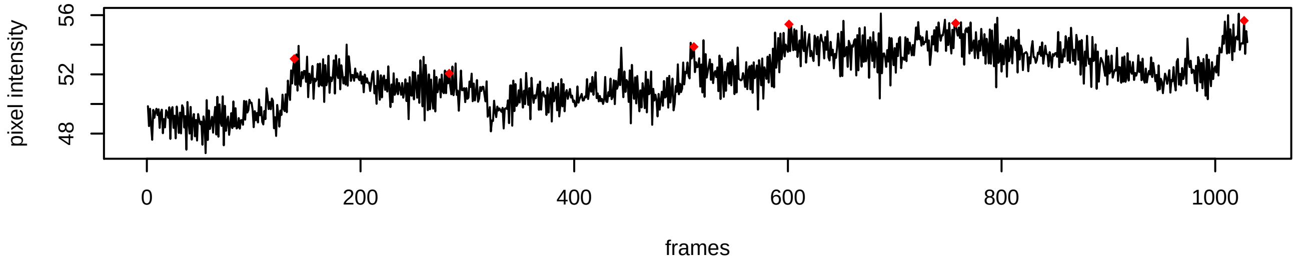

Graph 34 , 26   Total Activity 6   Position in Array 342

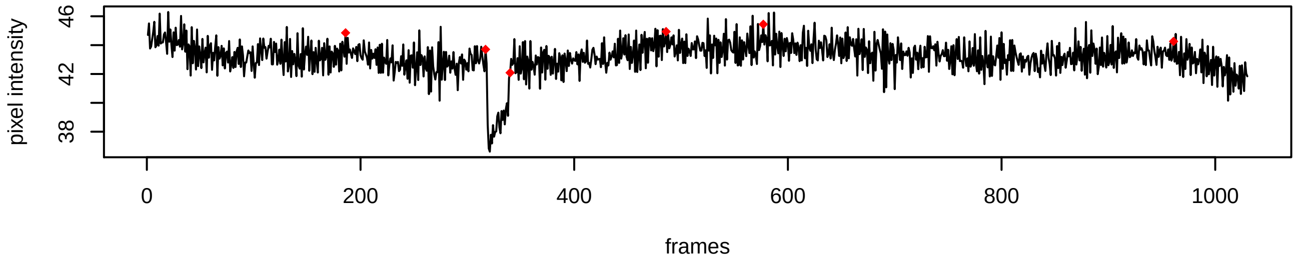

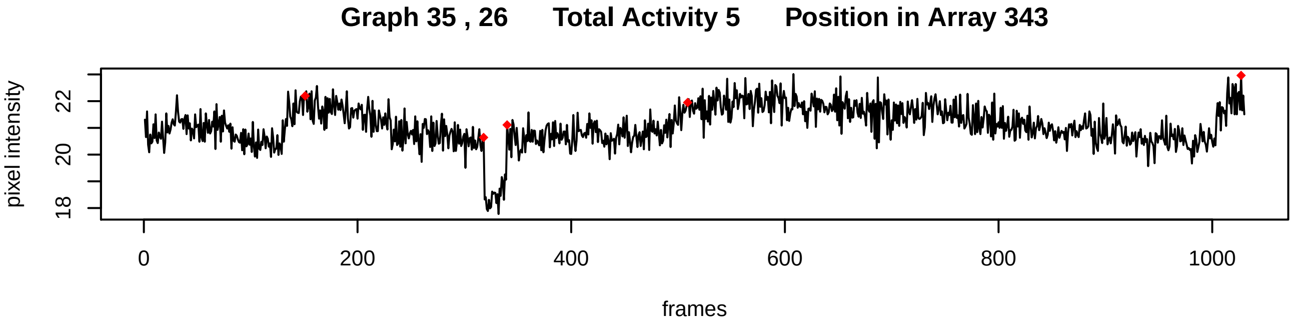
Graph 35 , 26 Total Activity 5 Position in Array 343
pixel intensity
18
20
22
0
200
400
600
800
1000
frames

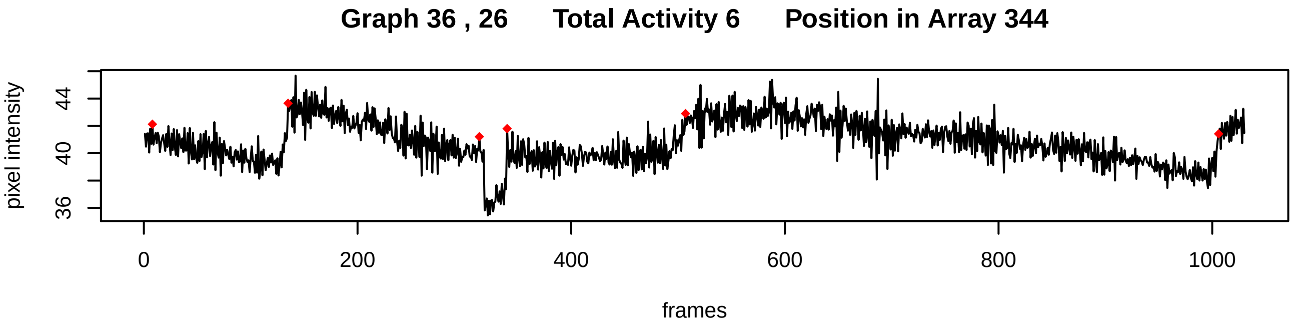
Graph 36 , 26 Total Activity 6 Position in Array 344
pixel intensity
36
40
44
0
200
400
600
800
1000
frames

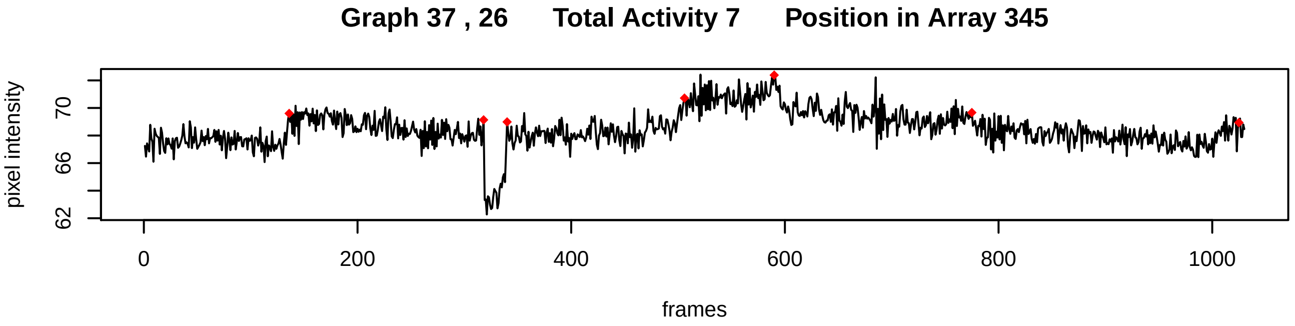
Graph 37 , 26 Total Activity 7 Position in Array 345
pixel intensity
62
66
70
0
200
400
600
800
1000
frames

Graph 38 , 26   Total Activity 5   Position in Array 346

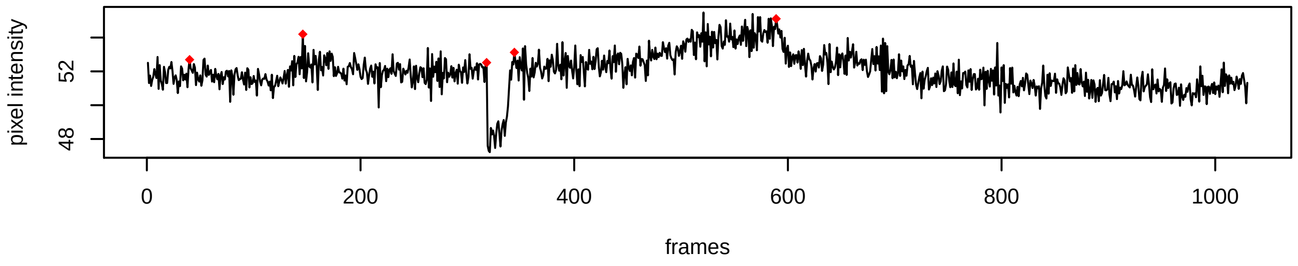

Graph 39 , 26   Total Activity 5   Position in Array 347

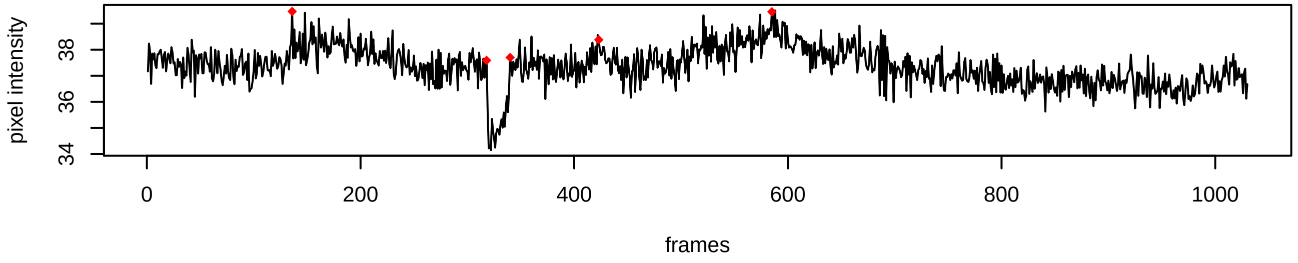

Graph 40 , 26   Total Activity 7   Position in Array 348

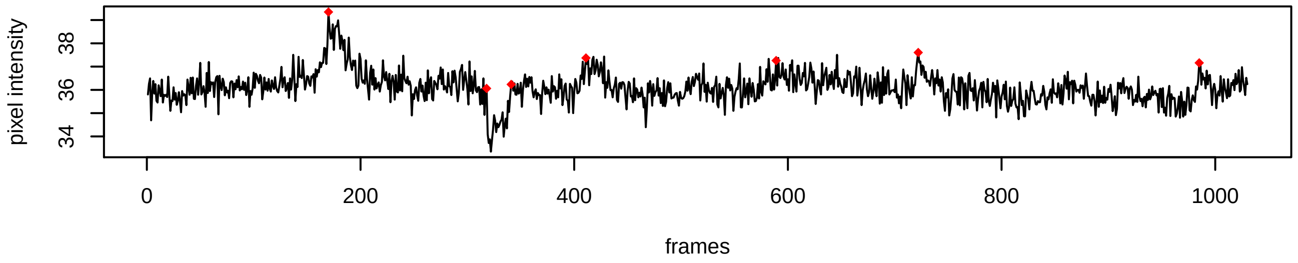

Graph 41 , 26   Total Activity 5   Position in Array 349

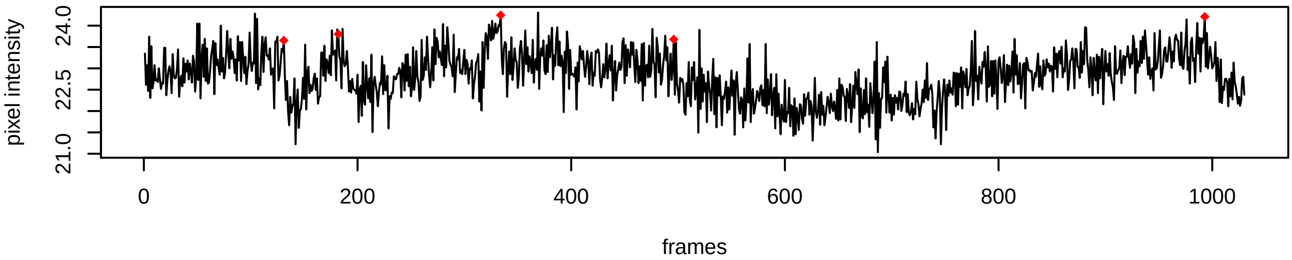

Graph 42 , 26   Total Activity 3   Position in Array 350

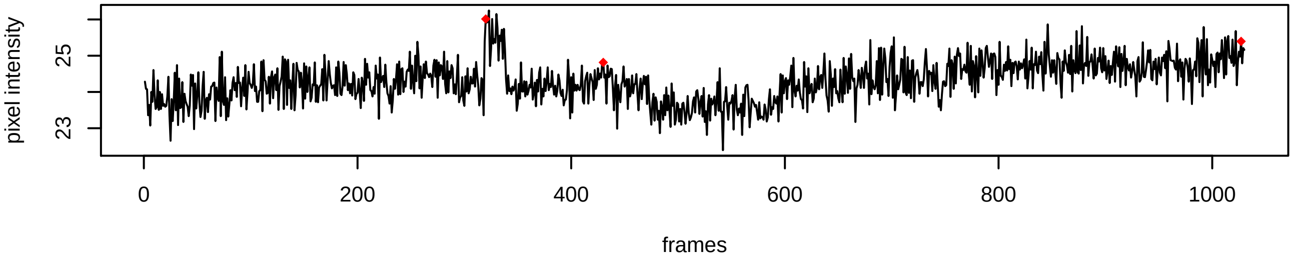

Graph 43 , 26   Total Activity 4   Position in Array 351

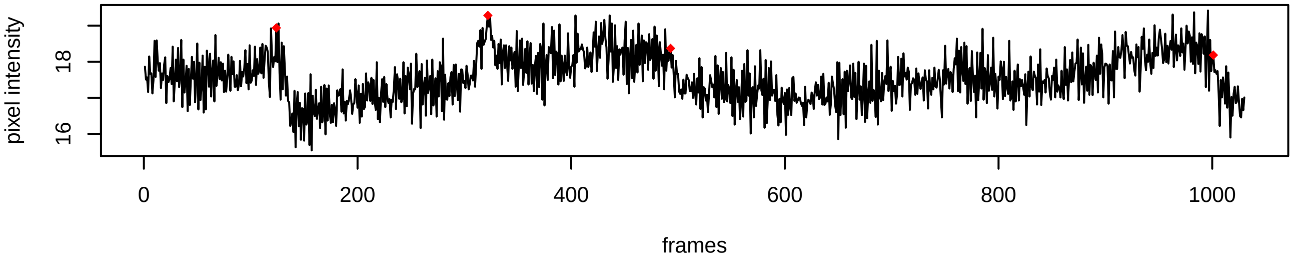

Graph 1 , 25 Total Activity 4 Position in Array 353

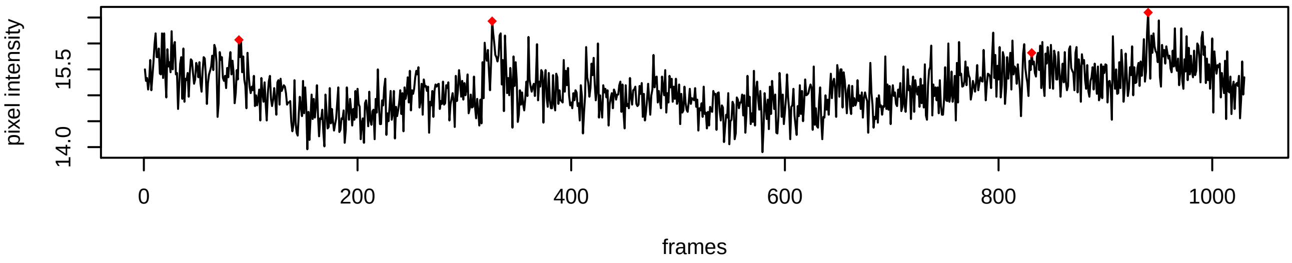

Graph 2 , 25 Total Activity 3 Position in Array 354

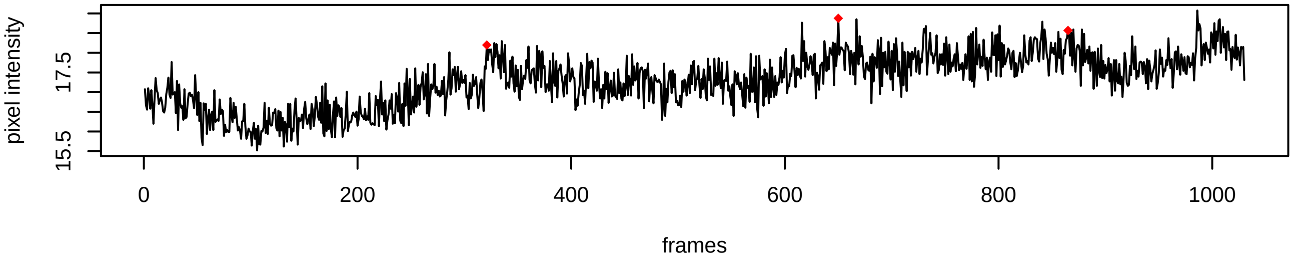

Graph 3 , 25 Total Activity 7 Position in Array 355

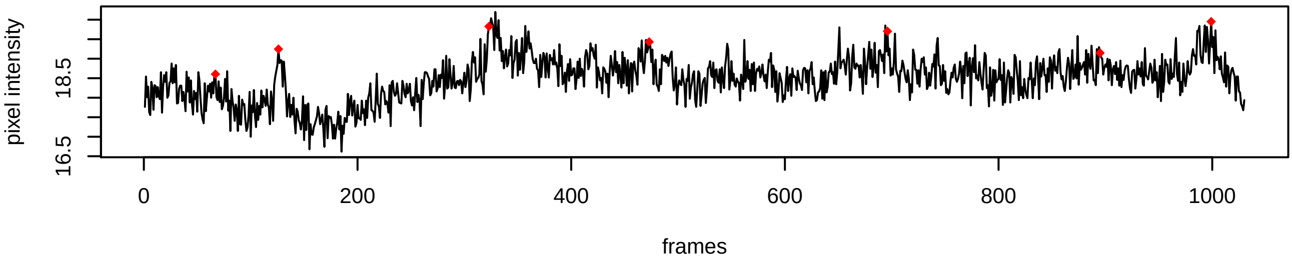

Graph 10 , 25   Total Activity 5   Position in Array 362

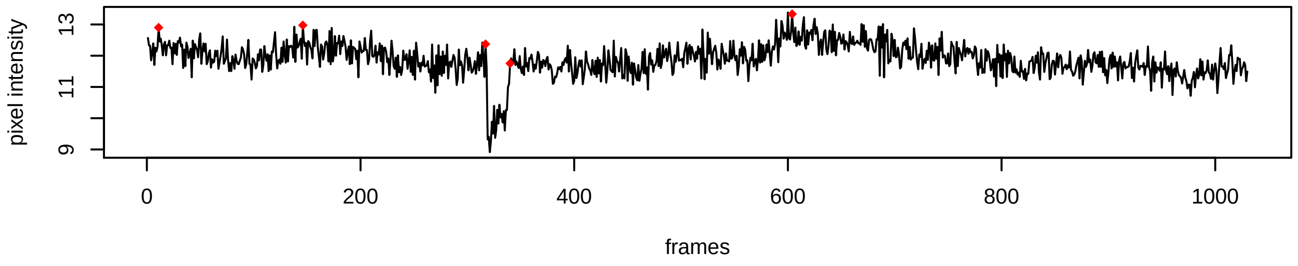

Graph 20 , 25   Total Activity 2   Position in Array 372

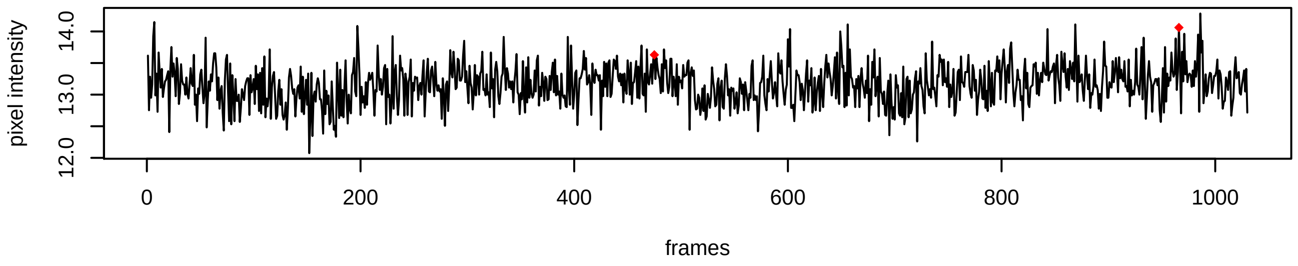

Graph 24 , 25   Total Activity 17   Position in Array 376

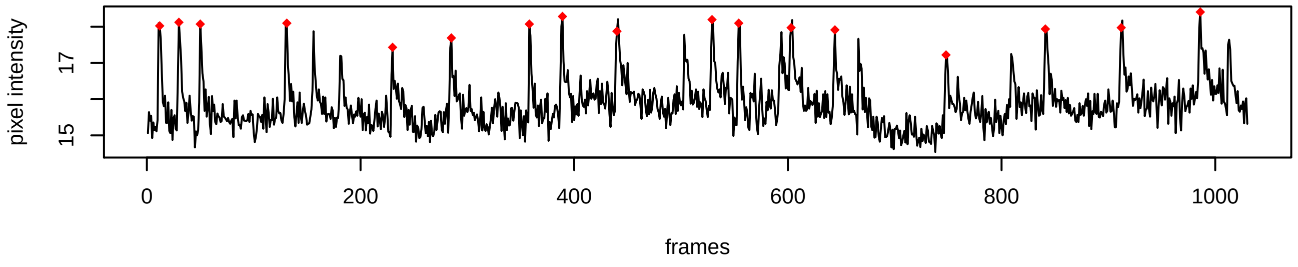

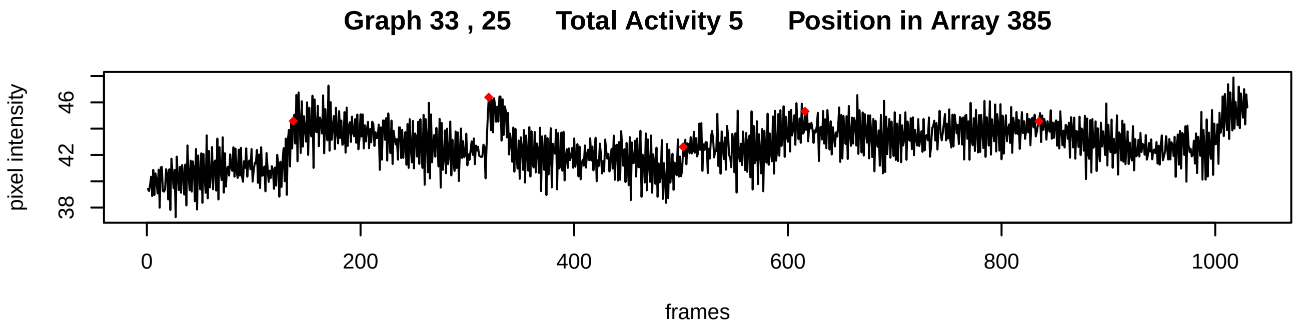
Graph 33 , 25 Total Activity 5 Position in Array 385
pixel intensity
38
42
46
0
200
400
600
800
1000
frames

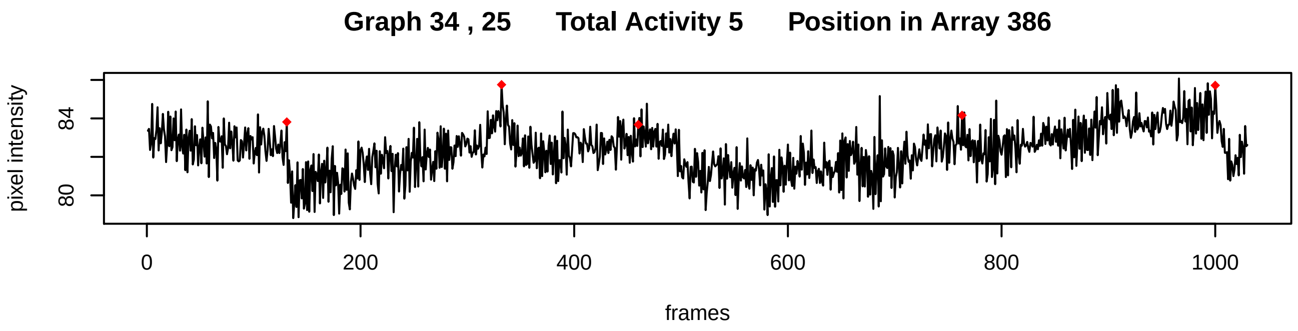
Graph 34 , 25 Total Activity 5 Position in Array 386
pixel intensity
80
84
0
200
400
600
800
1000
frames

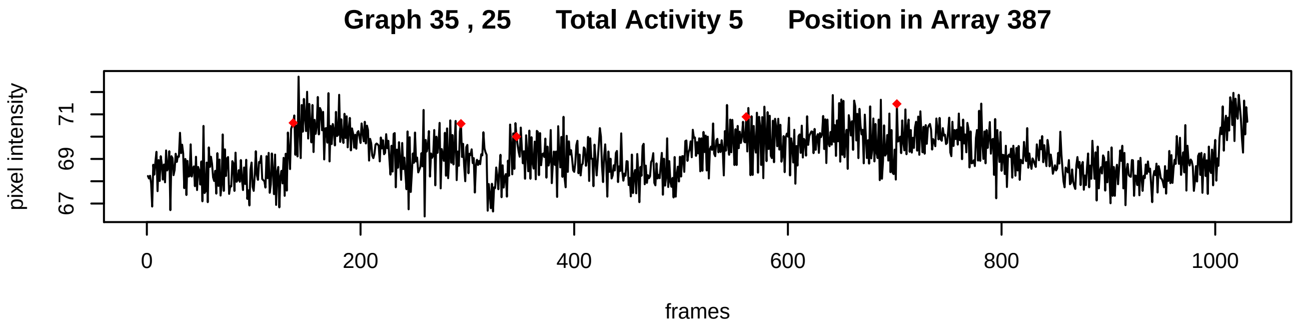
Graph 35 , 25 Total Activity 5 Position in Array 387
pixel intensity
67
69
71
0
200
400
600
800
1000
frames

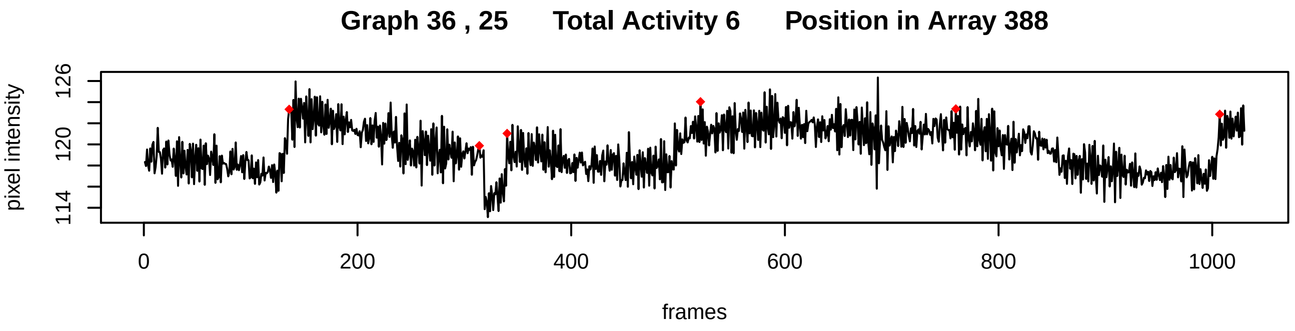
Graph 36 , 25 Total Activity 6 Position in Array 388
pixel intensity
126
120
114
0
200
400
600
800
1000
frames

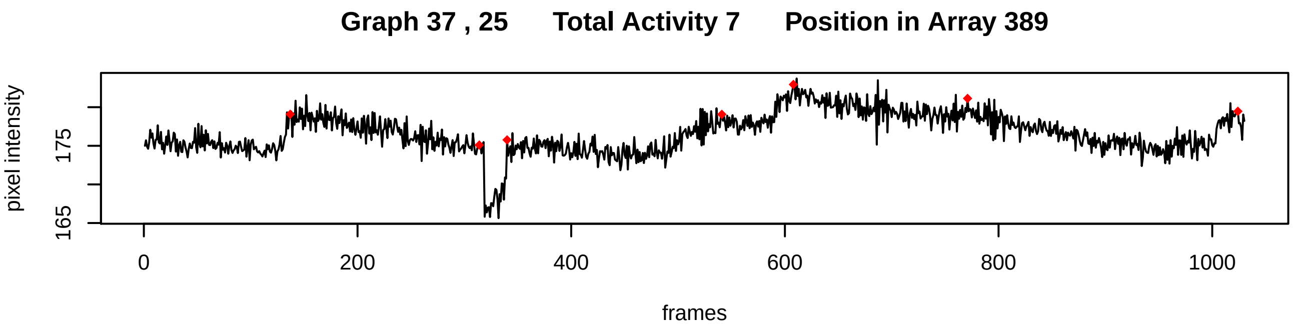
Graph 37 , 25 Total Activity 7 Position in Array 389
pixel intensity
175
165
0
200
400
600
800
1000
frames

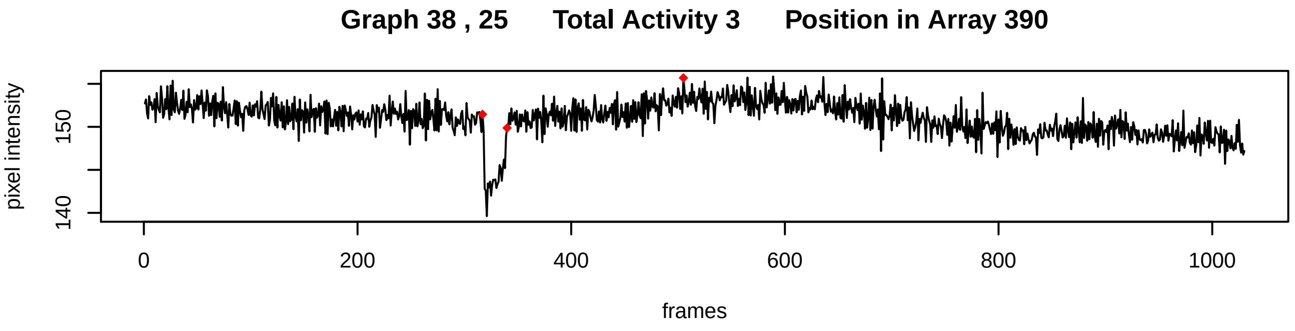
Graph 38 , 25 Total Activity 3 Position in Array 390
pixel intensity
150
140
0
200
400
600
800
1000
frames

Graph 39 , 25 Total Activity 6 Position in Array 391

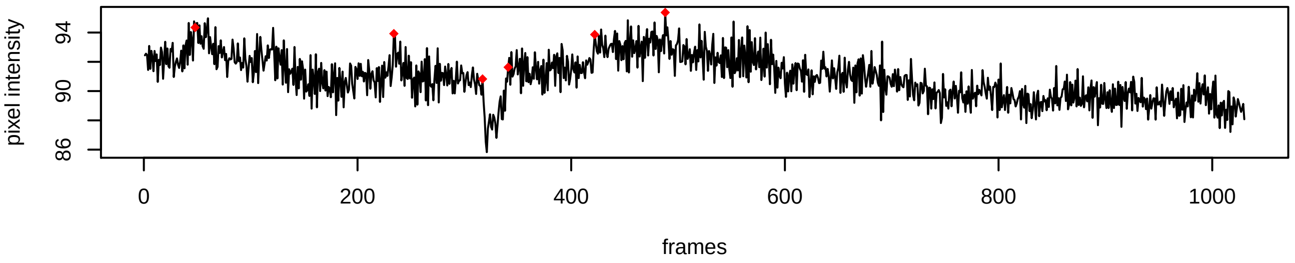

Graph 40 , 25 Total Activity 4 Position in Array 392

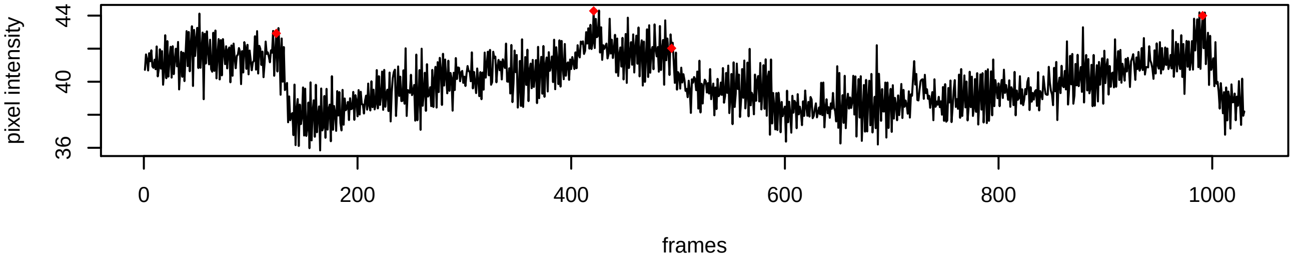

Graph 42 , 25 Total Activity 3 Position in Array 394

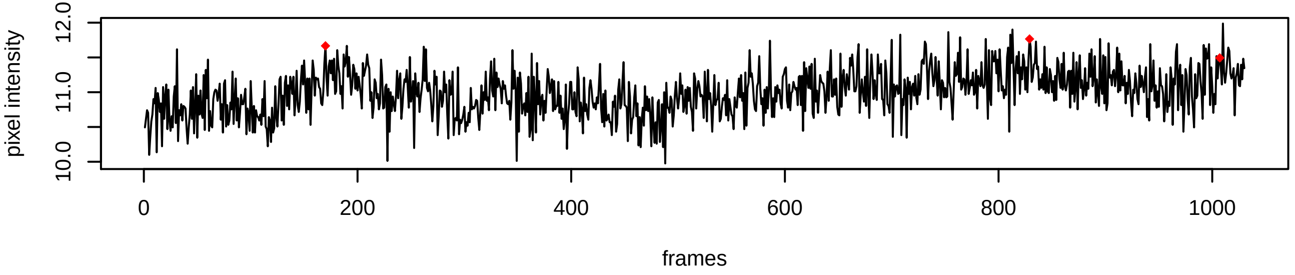

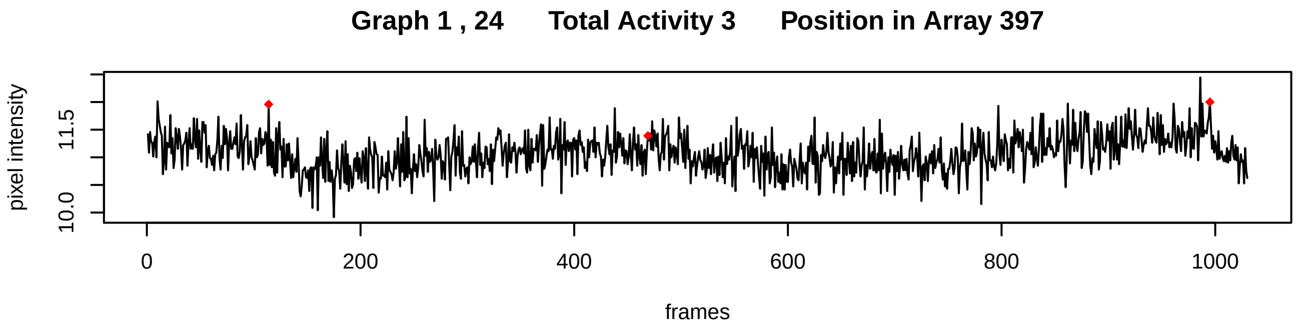
Graph 1 , 24 Total Activity 3 Position in Array 397
pixel intensity
10.0
11.5
0
200
400
600
800
1000
frames

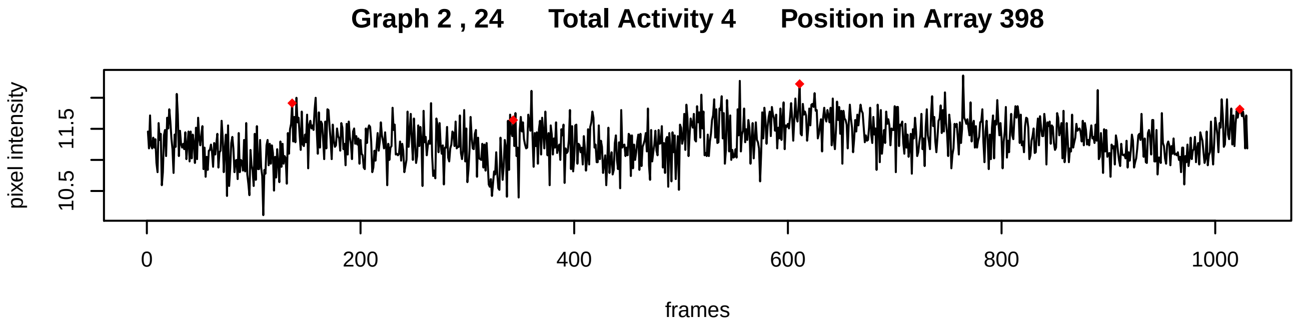
Graph 2 , 24 Total Activity 4 Position in Array 398
pixel intensity
10.5
11.5
0
200
400
600
800
1000
frames

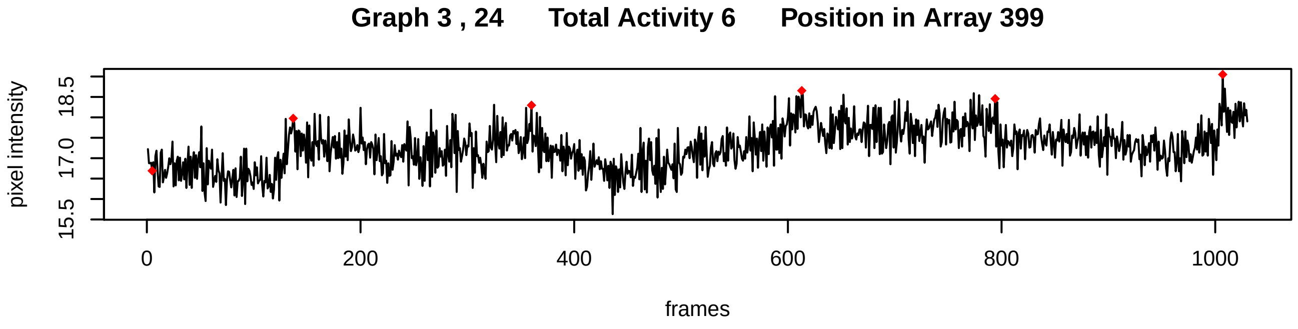
Graph 3 , 24 Total Activity 6 Position in Array 399
pixel intensity
15.5
17.0
18.5
0
200
400
600
800
1000
frames

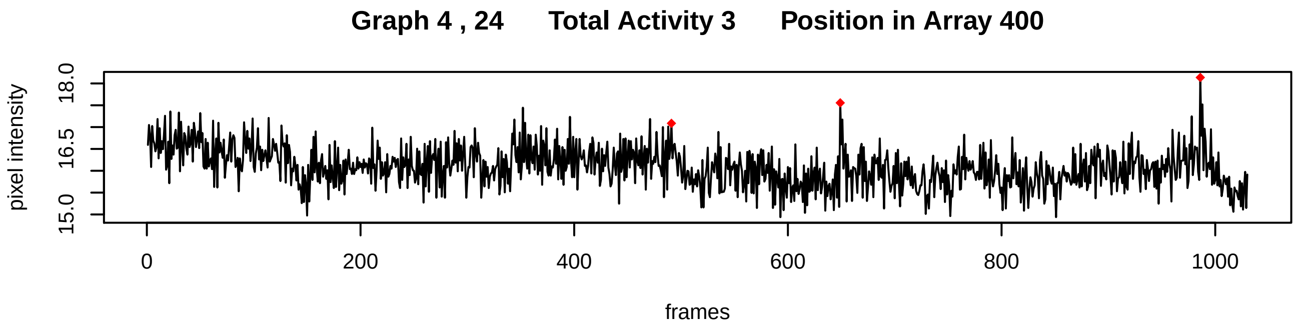
Graph 4 , 24 Total Activity 3 Position in Array 400
pixel intensity
15.0
16.5
18.0
0
200
400
600
800
1000
frames

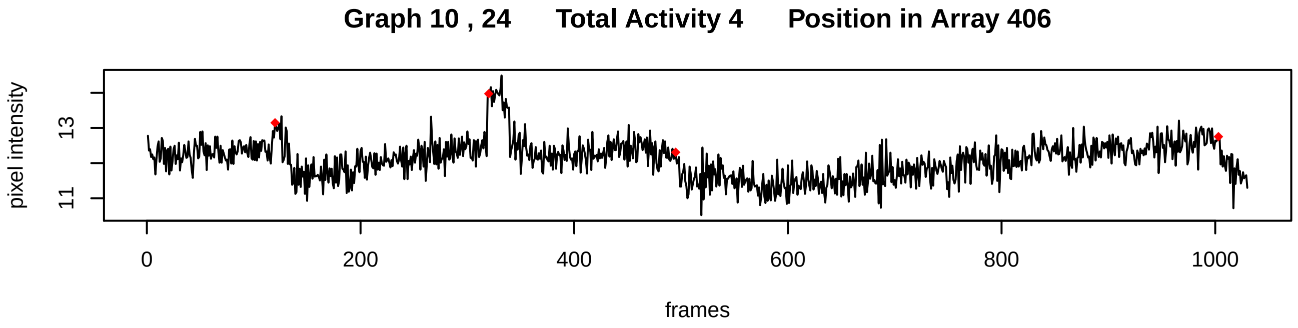
Graph 10 , 24 Total Activity 4 Position in Array 406
pixel intensity
11
13
0
200
400
600
800
1000
frames

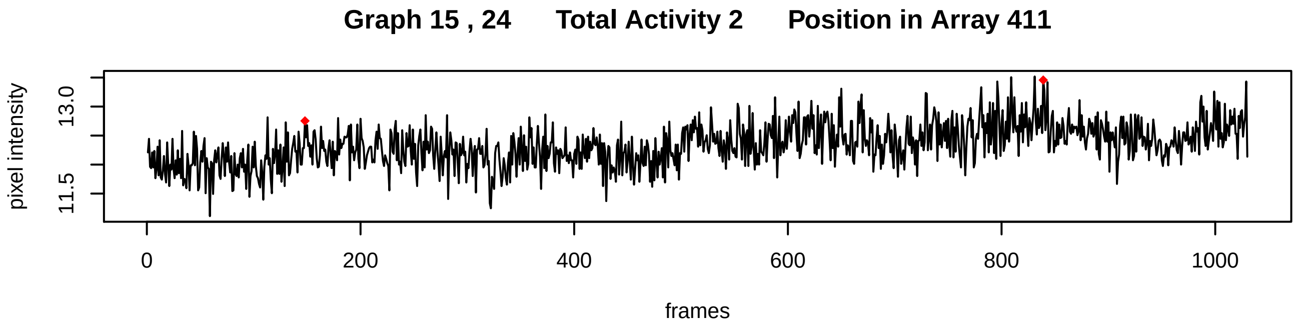
Graph 15 , 24 Total Activity 2 Position in Array 411
pixel intensity
11.5
13.0
0
200
400
600
800
1000
frames

Graph 19 , 24 Total Activity 4 Position in Array 415

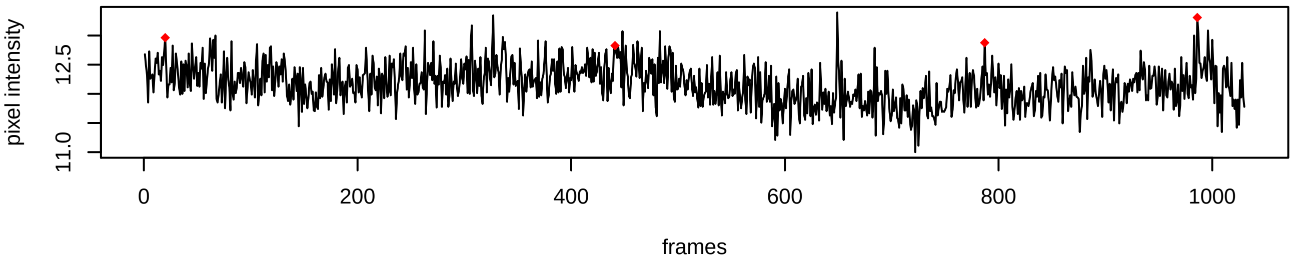

Graph 25 , 24 Total Activity 3 Position in Array 421

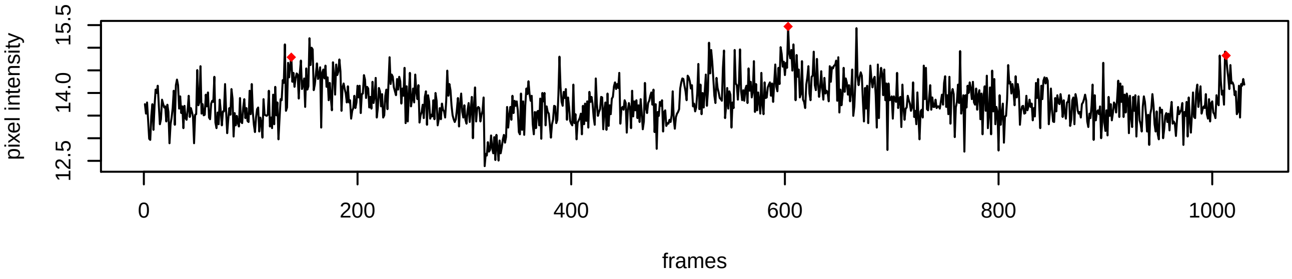

Graph 27 , 24 Total Activity 2 Position in Array 423

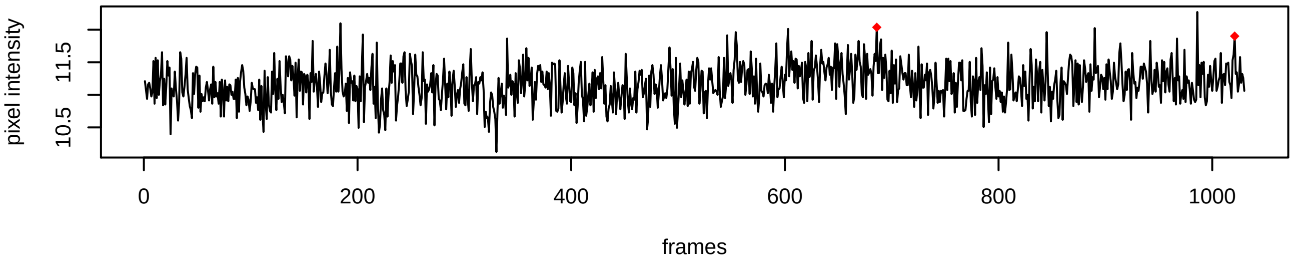

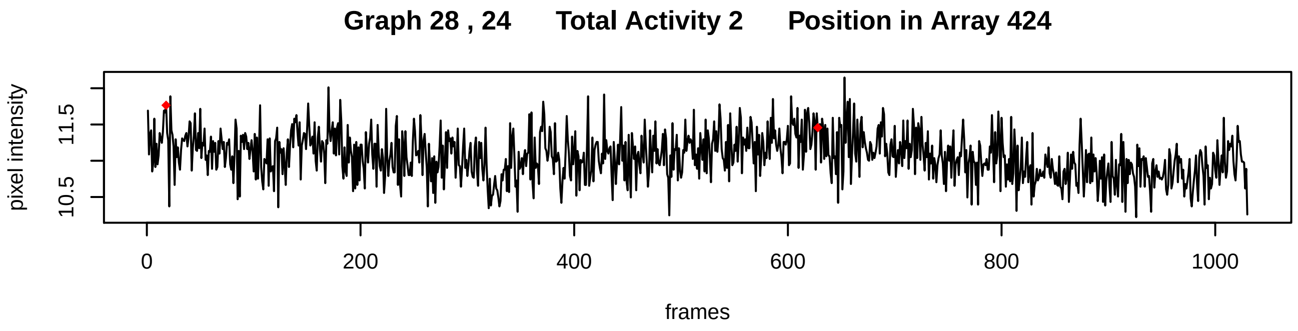
Graph 28 , 24 Total Activity 2 Position in Array 424
pixel intensity
10.5
11.5
0
200
400
600
800
1000
frames

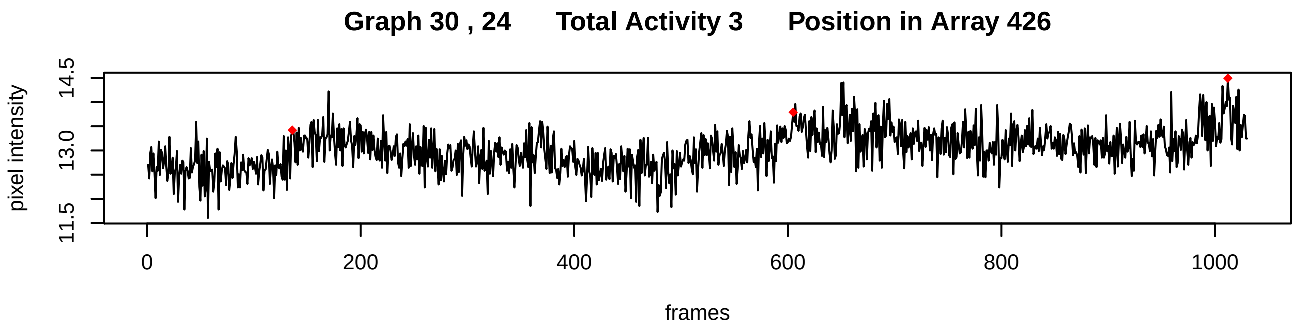
Graph 30 , 24 Total Activity 3 Position in Array 426
pixel intensity
11.5
13.0
14.5
0
200
400
600
800
1000
frames

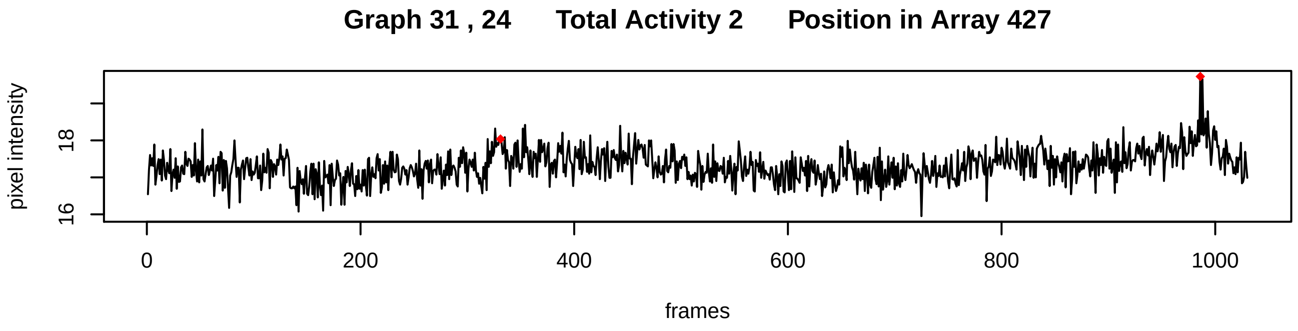
Graph 31 , 24 Total Activity 2 Position in Array 427
pixel intensity
16
18
0
200
400
600
800
1000
frames

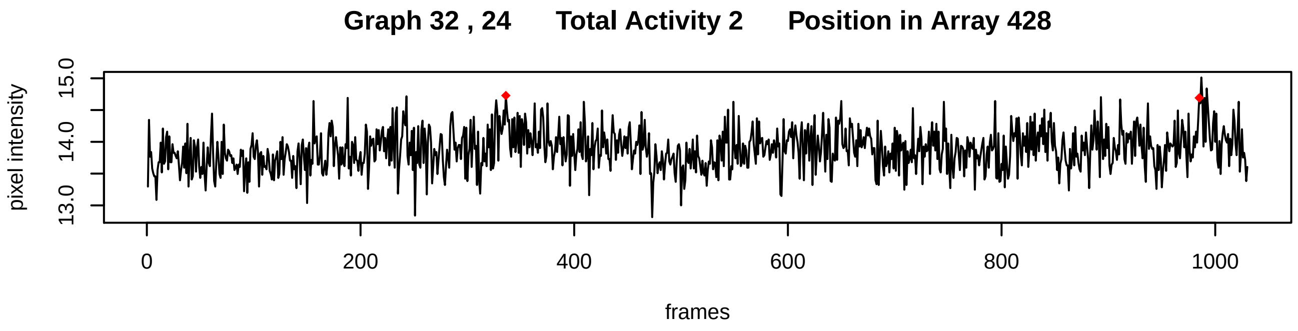
Graph 32 , 24 Total Activity 2 Position in Array 428
pixel intensity
13.0
14.0
15.0
0
200
400
600
800
1000
frames

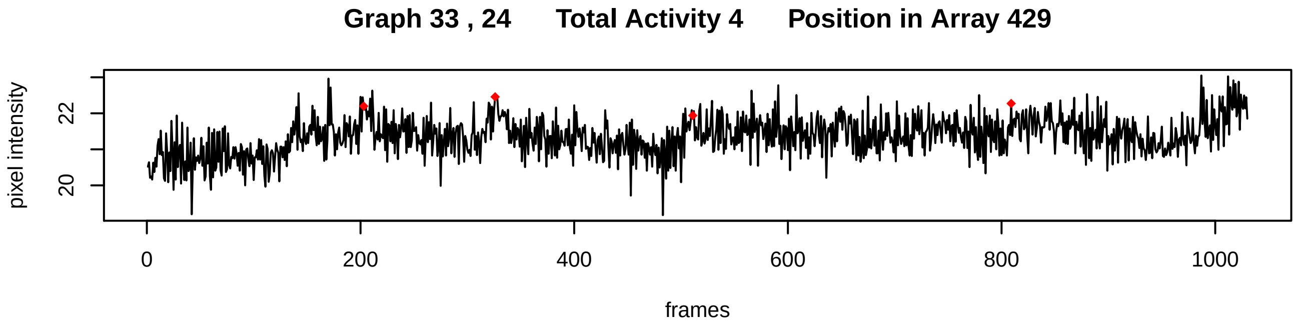
Graph 33 , 24 Total Activity 4 Position in Array 429
pixel intensity
20
22
0
200
400
600
800
1000
frames

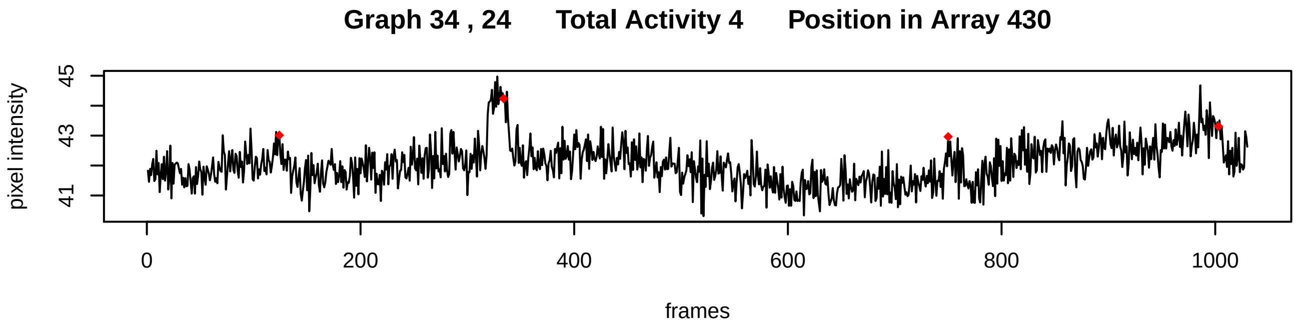
Graph 34 , 24 Total Activity 4 Position in Array 430
pixel intensity
41
43
45
0
200
400
600
800
1000
frames

Graph 35 , 24   Total Activity 4   Position in Array 431

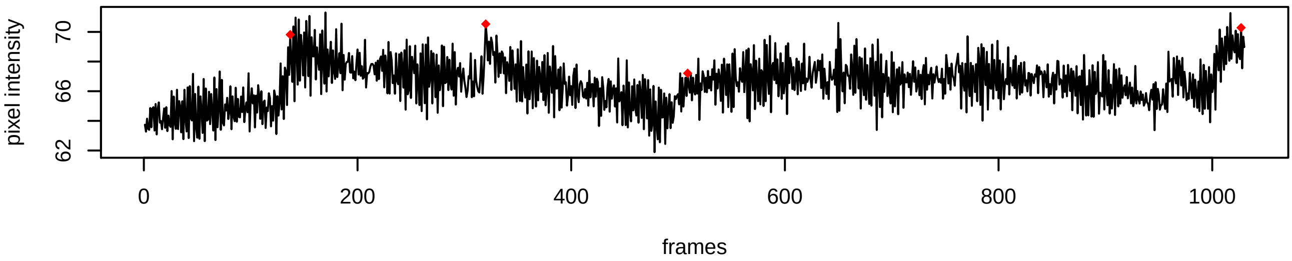

Graph 36 , 24   Total Activity 5   Position in Array 432

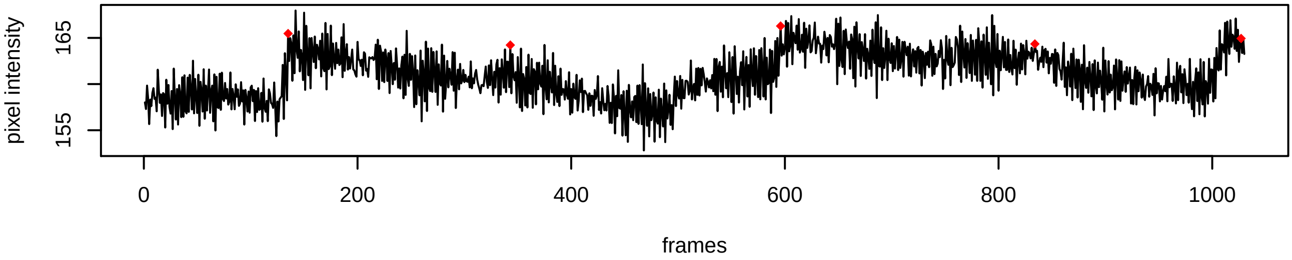

Graph 37 , 24   Total Activity 3   Position in Array 433

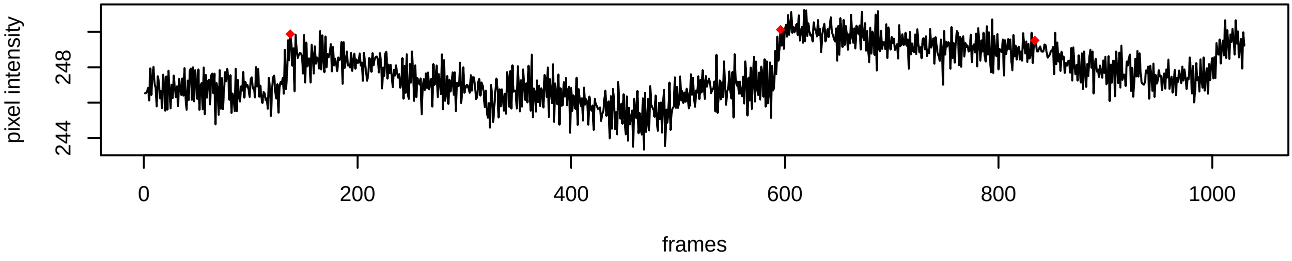

Graph 38 , 24 Total Activity 5 Position in Array 434

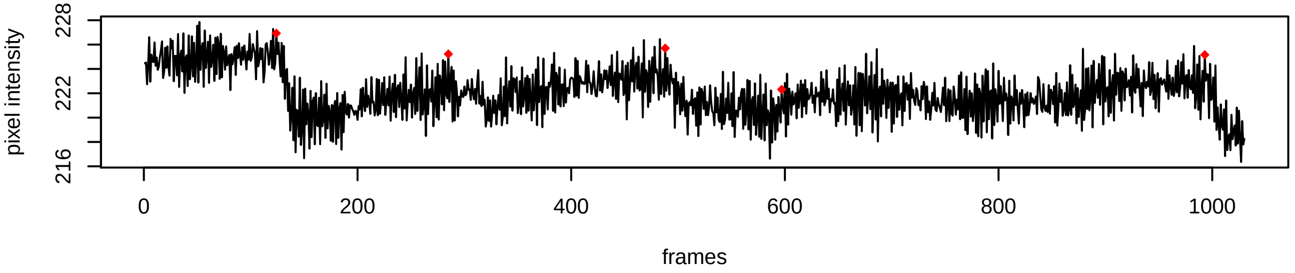

Graph 39 , 24 Total Activity 4 Position in Array 435

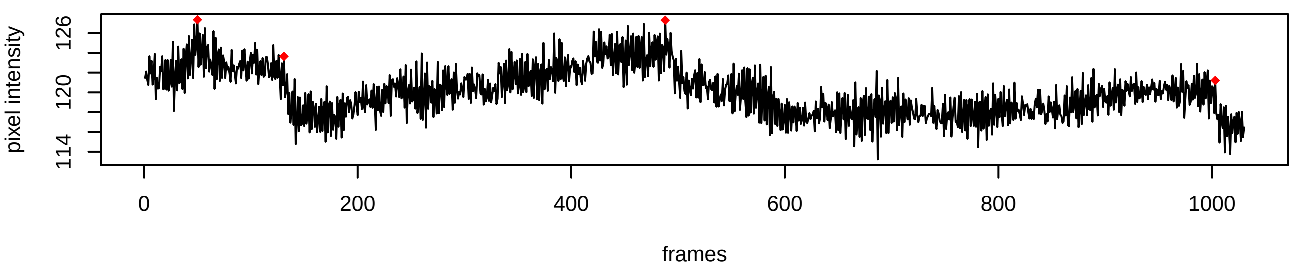

Graph 40 , 24 Total Activity 6 Position in Array 436

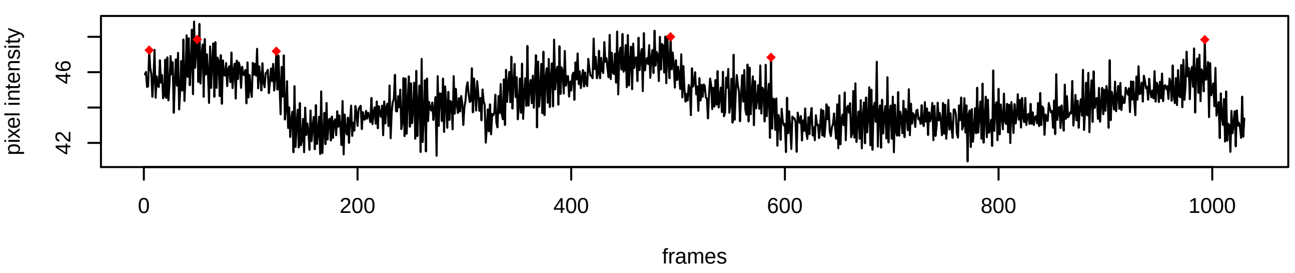

Graph 41 , 24 Total Activity 4 Position in Array 437

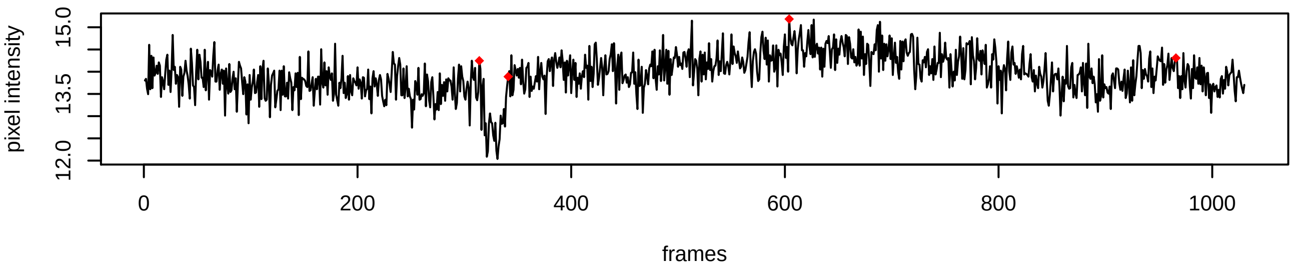

Graph 42 , 24 Total Activity 7 Position in Array 438

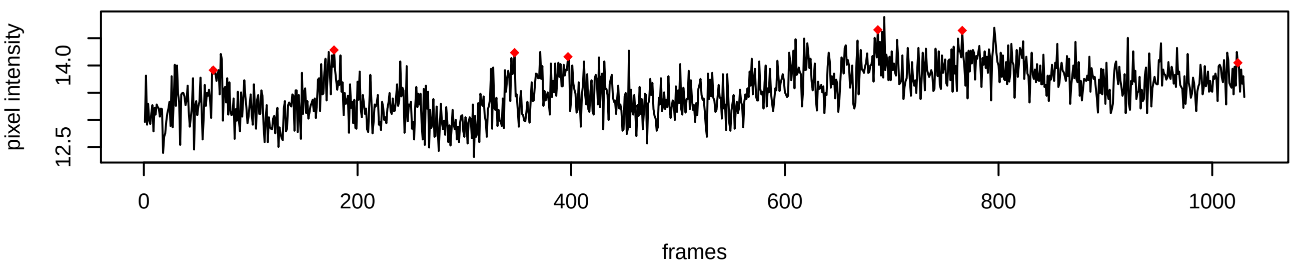

Graph 1 , 23 Total Activity 3 Position in Array 441

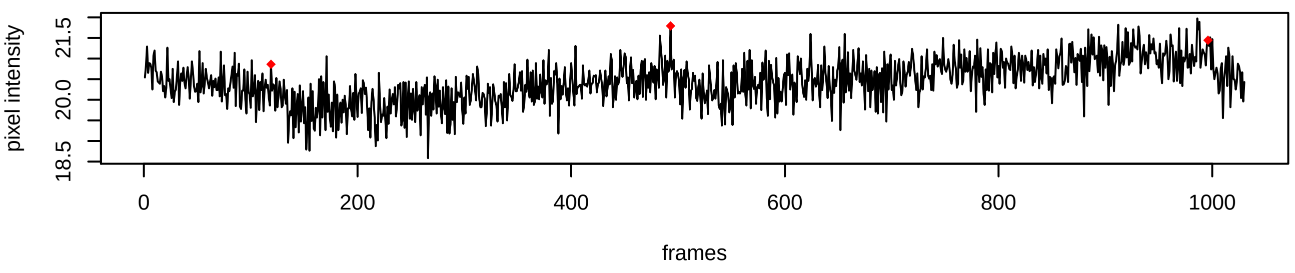

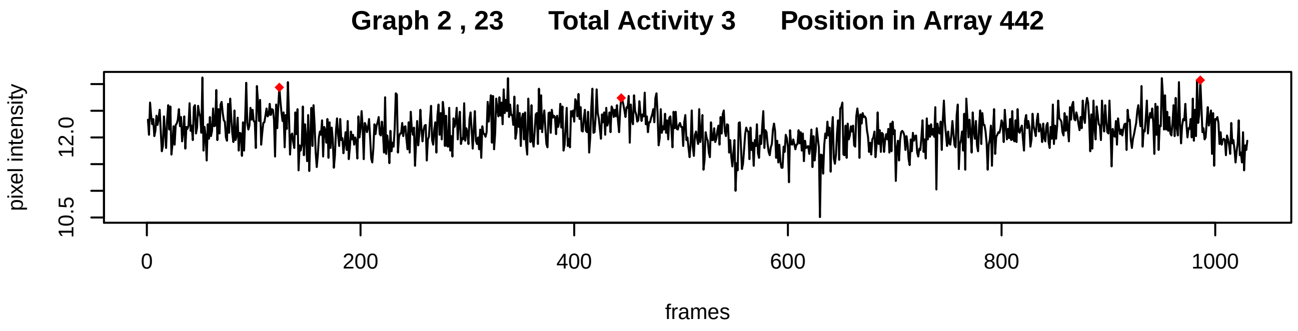
Graph 2 , 23 Total Activity 3 Position in Array 442
pixel intensity
12.0
10.5
0
200
400
600
800
1000
frames

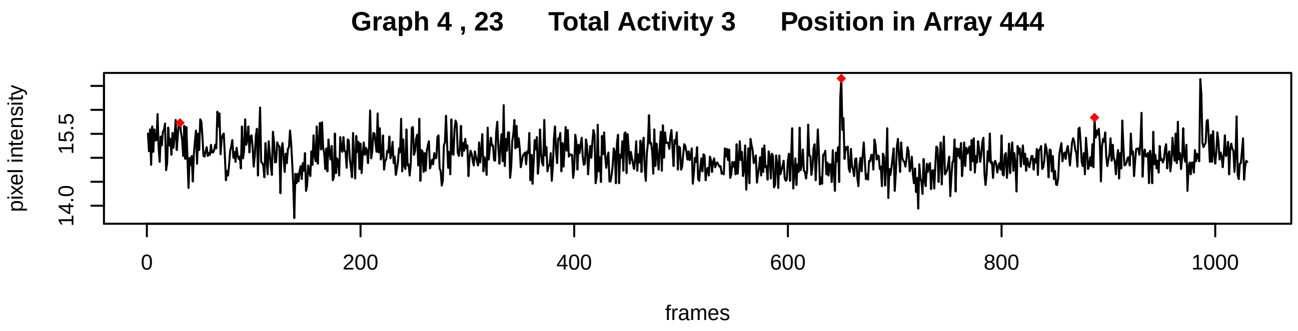
Graph 4 , 23 Total Activity 3 Position in Array 444
pixel intensity
15.5
14.0
0
200
400
600
800
1000
frames

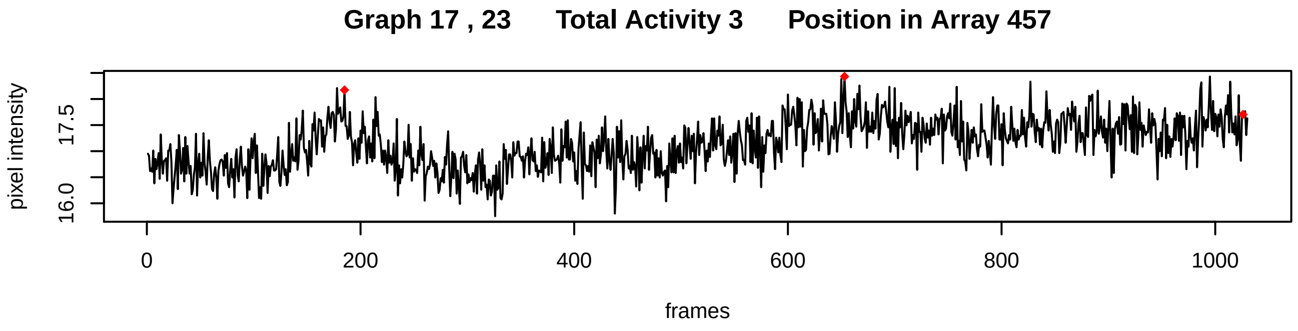
Graph 17 , 23 Total Activity 3 Position in Array 457
pixel intensity
17.5
16.0
0
200
400
600
800
1000
frames

Graph 18 , 23   Total Activity 4   Position in Array 458

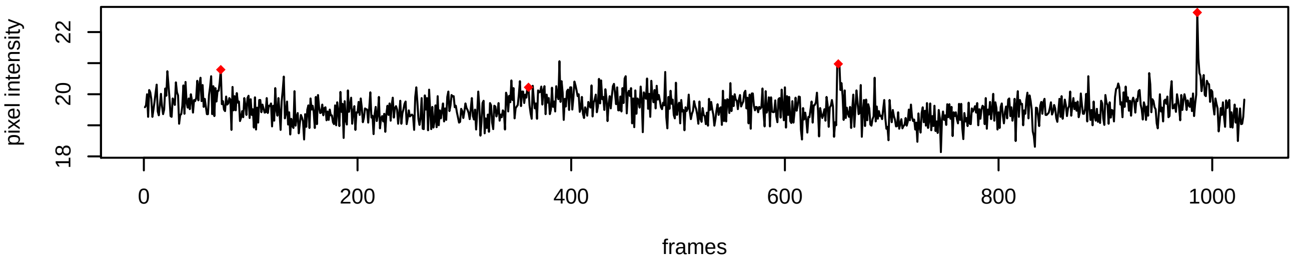

Graph 20 , 23   Total Activity 4   Position in Array 460

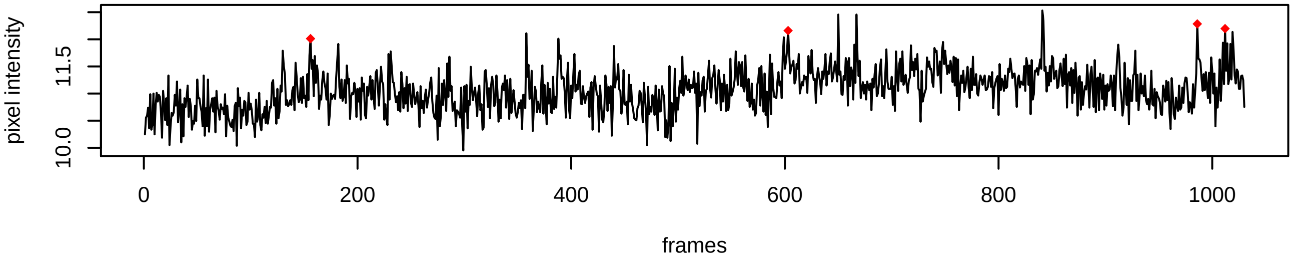

Graph 21 , 23   Total Activity 2   Position in Array 461

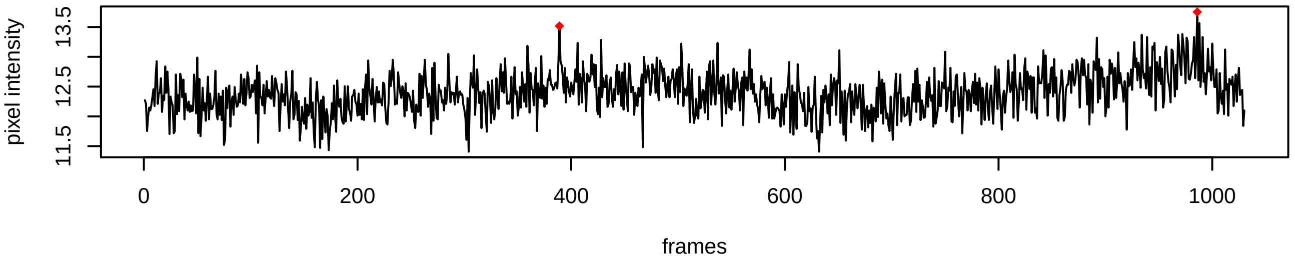

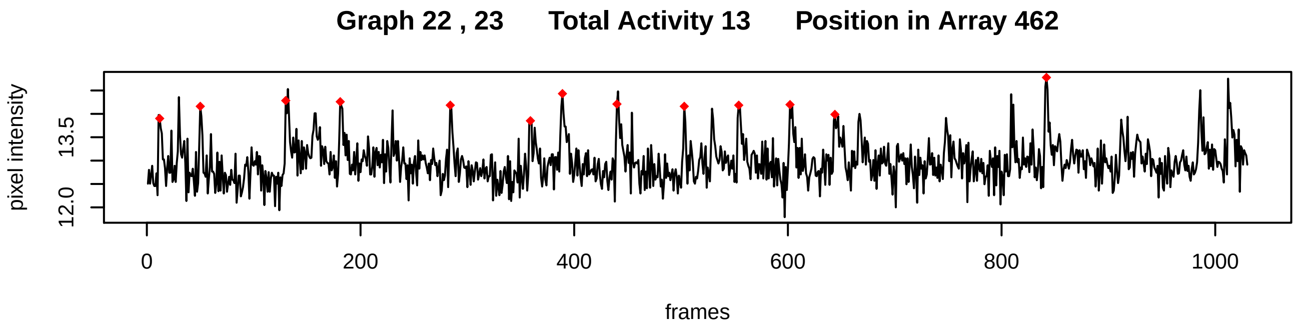
Graph 22 , 23 Total Activity 13 Position in Array 462
pixel intensity
12.0
13.5
0
200
400
600
800
1000
frames

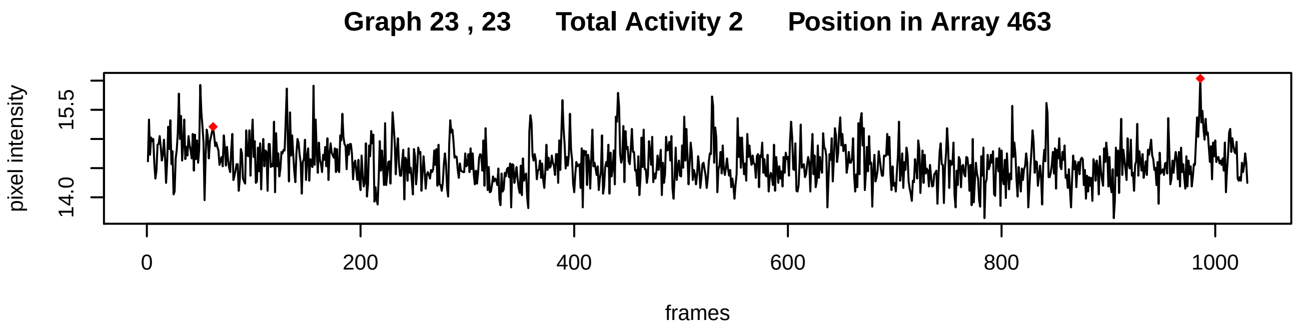
Graph 23 , 23 Total Activity 2 Position in Array 463
pixel intensity
14.0
15.5
0
200
400
600
800
1000
frames

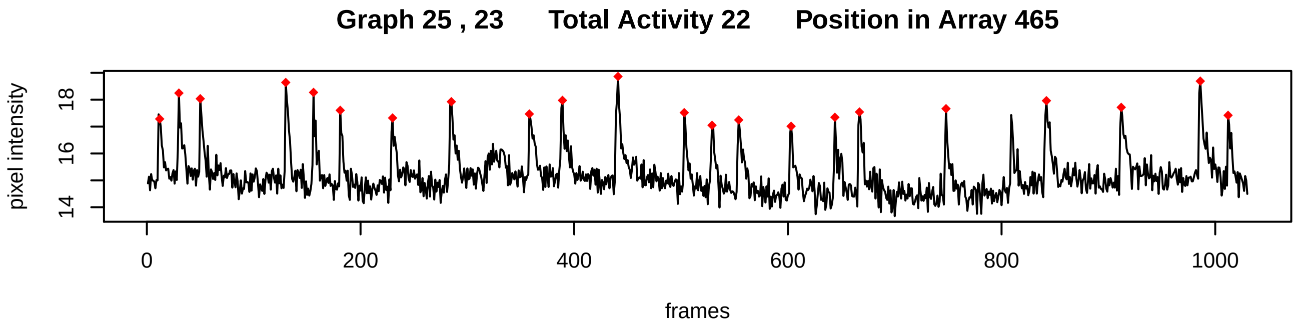
Graph 25 , 23 Total Activity 22 Position in Array 465
pixel intensity
14
16
18
0
200
400
600
800
1000
frames

Graph 28 , 23 Total Activity 3 Position in Array 468

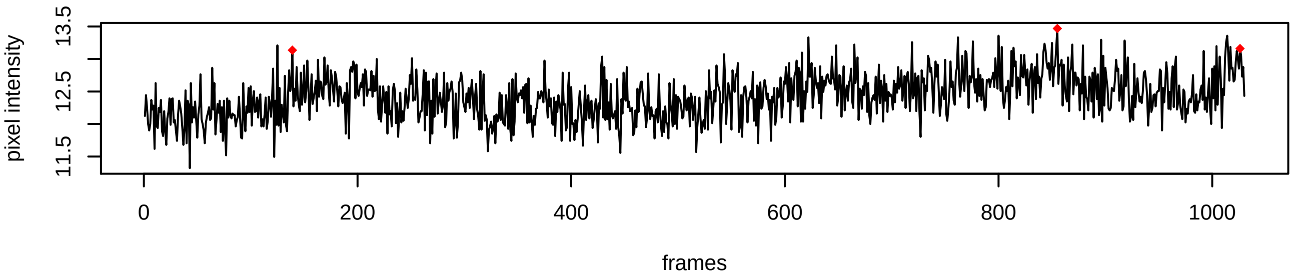

Graph 34 , 23 Total Activity 15 Position in Array 474

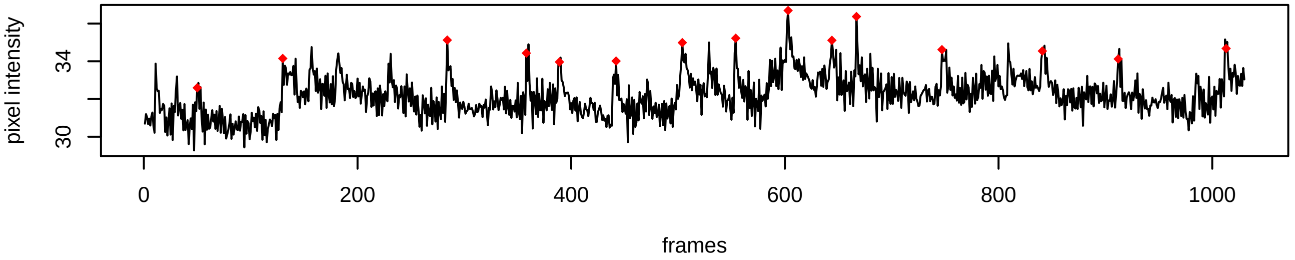

Graph 35 , 23 Total Activity 6 Position in Array 475

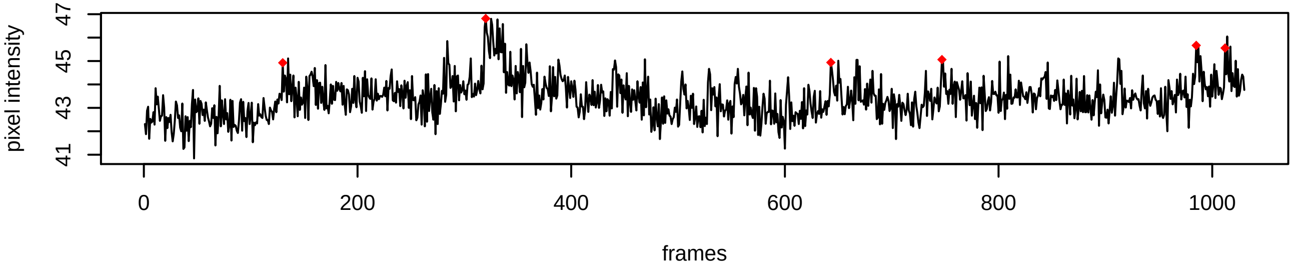

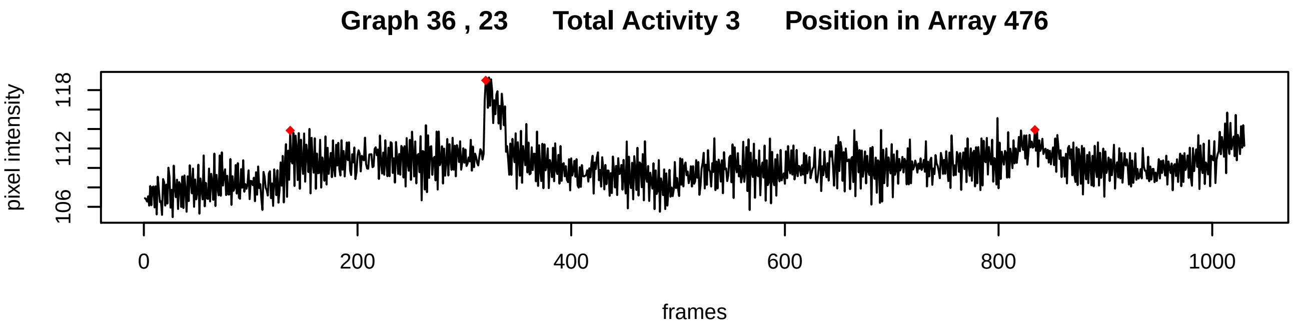
Graph 36 , 23 Total Activity 3 Position in Array 476
pixel intensity
106
112
118
0
200
400
600
800
1000
frames

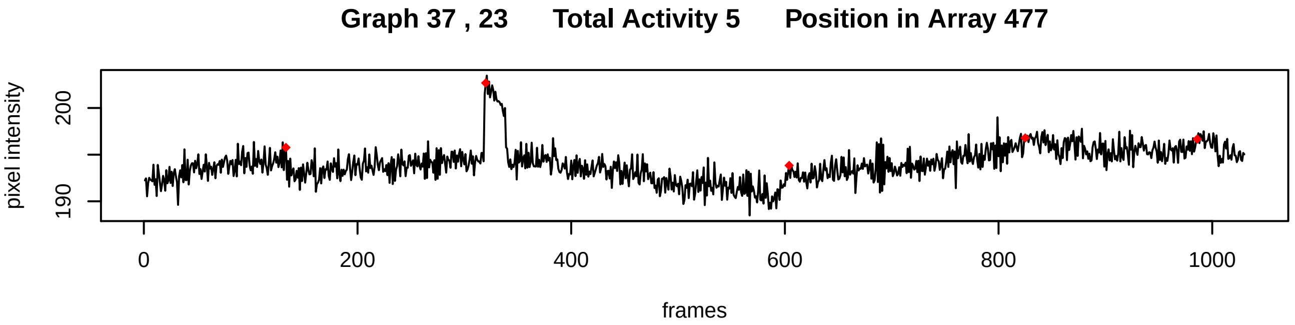
Graph 37 , 23 Total Activity 5 Position in Array 477
pixel intensity
190
200
0
200
400
600
800
1000
frames

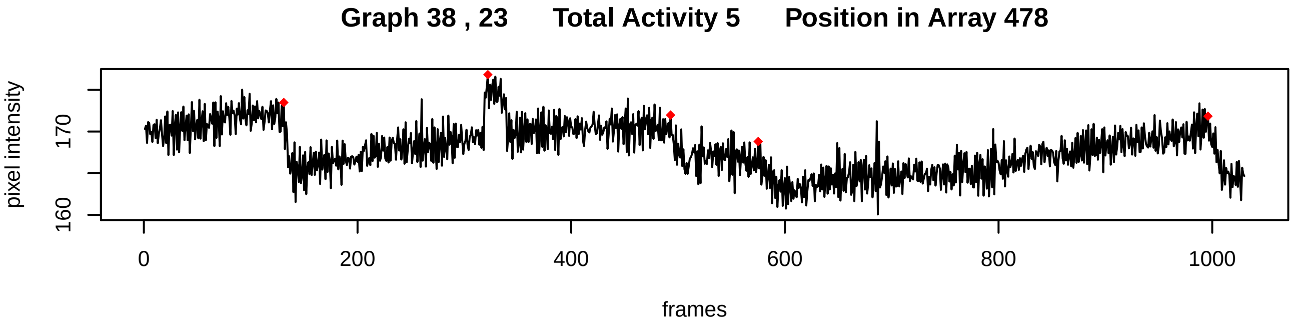
Graph 38 , 23 Total Activity 5 Position in Array 478
pixel intensity
160
170
0
200
400
600
800
1000
frames

Graph 39 , 23 Total Activity 5 Position in Array 479

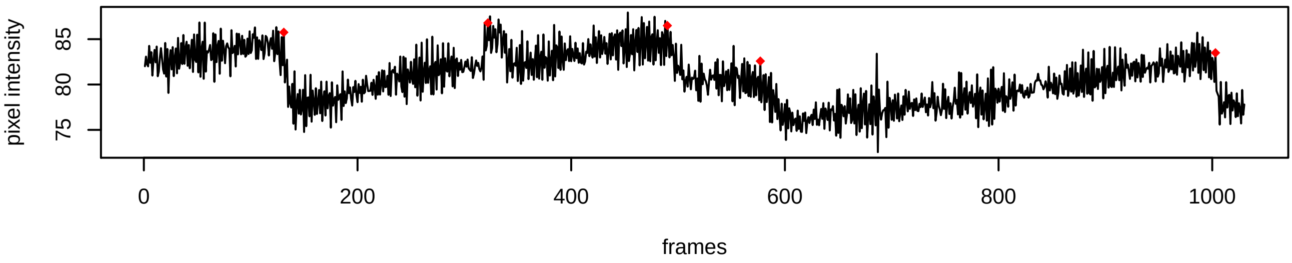

Graph 40 , 23 Total Activity 18 Position in Array 480

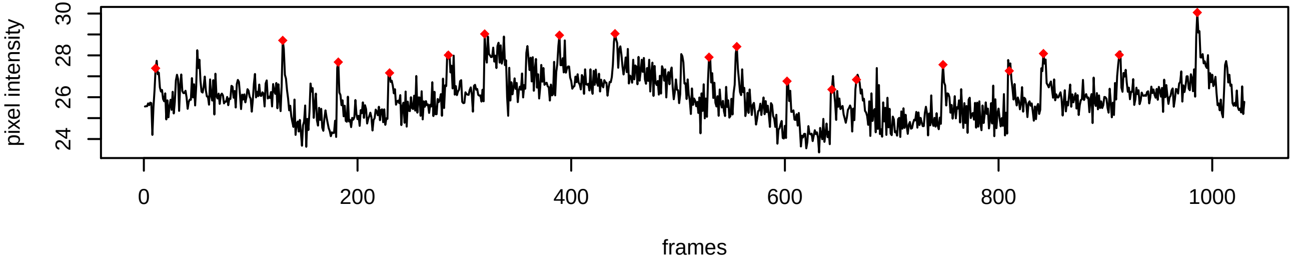

Graph 41 , 23 Total Activity 20 Position in Array 481

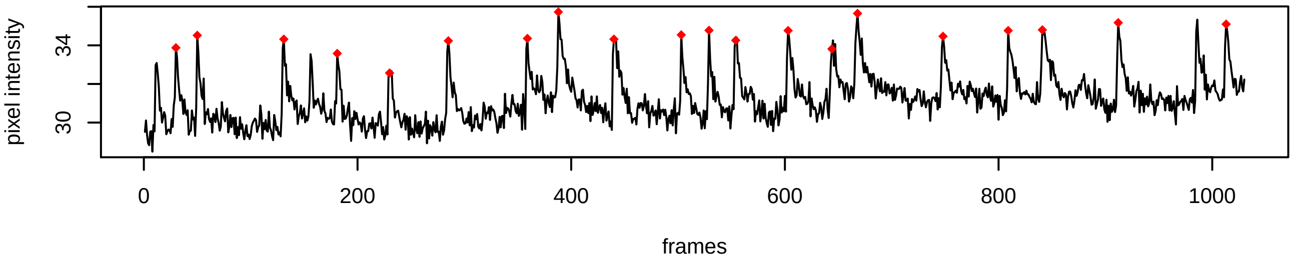

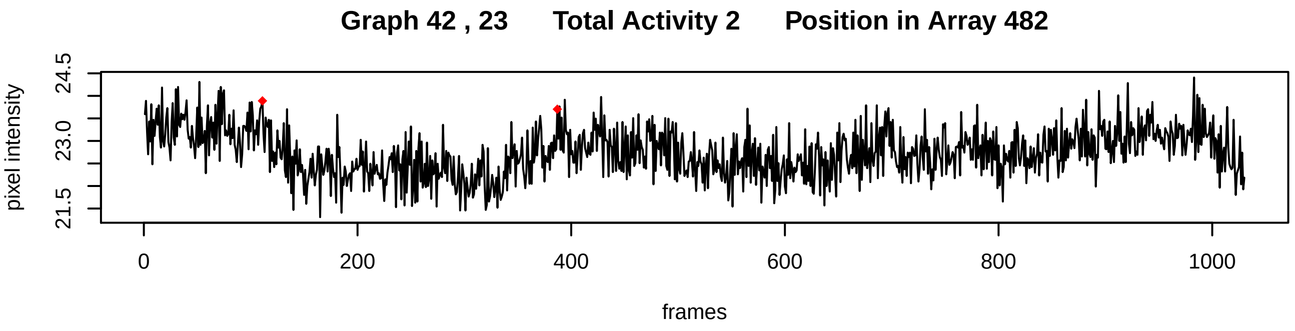
Graph 42 , 23 Total Activity 2 Position in Array 482
pixel intensity
21.5
23.0
24.5
0
200
400
600
800
1000
frames

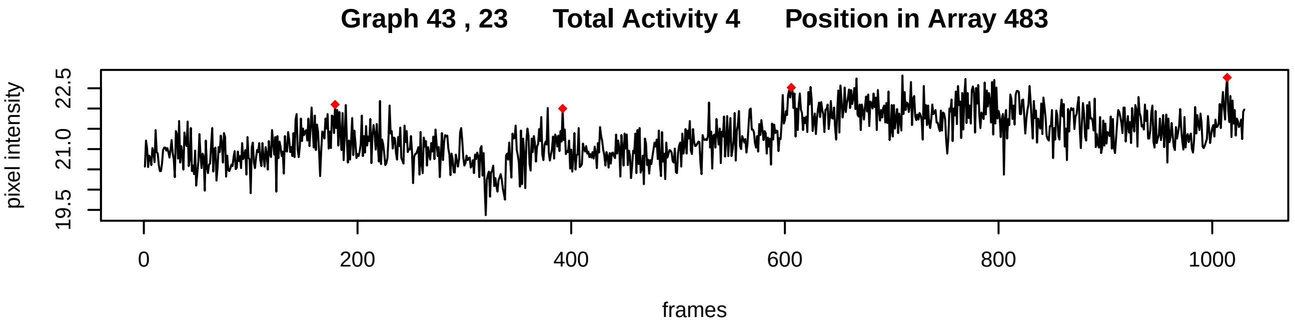
Graph 43 , 23 Total Activity 4 Position in Array 483
pixel intensity
19.5
21.0
22.5
0
200
400
600
800
1000
frames

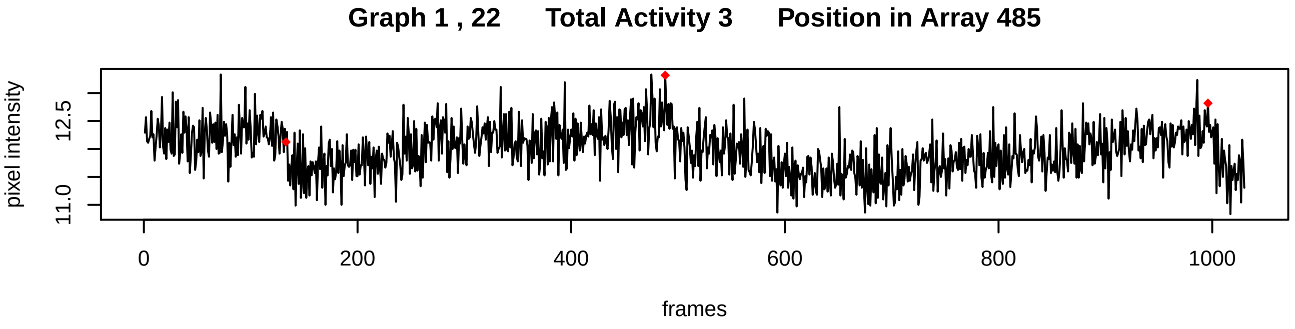
Graph 1 , 22 Total Activity 3 Position in Array 485
pixel intensity
11.0
12.5
0
200
400
600
800
1000
frames

Graph 17 , 22   Total Activity 2   Position in Array 501

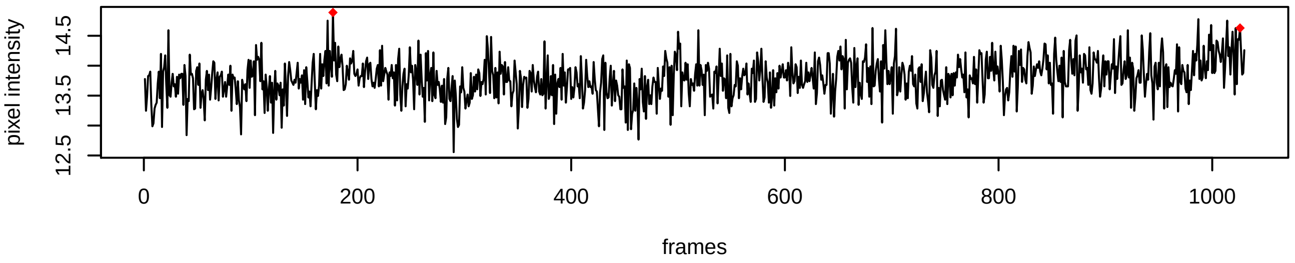

Graph 18 , 22   Total Activity 3   Position in Array 502

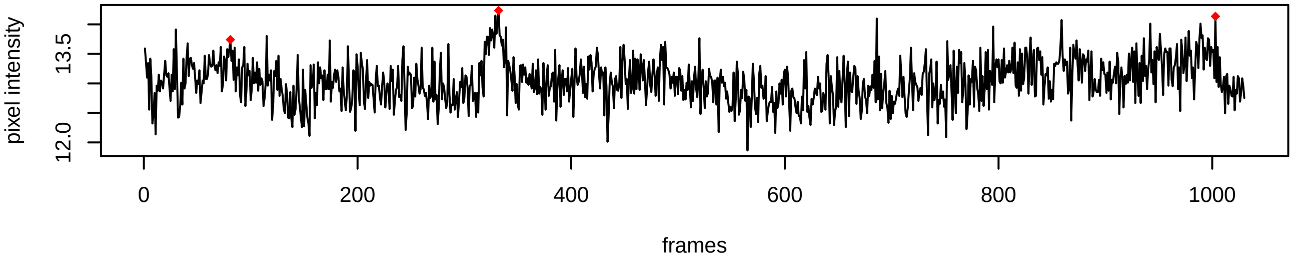

Graph 33 , 22   Total Activity 22   Position in Array 517

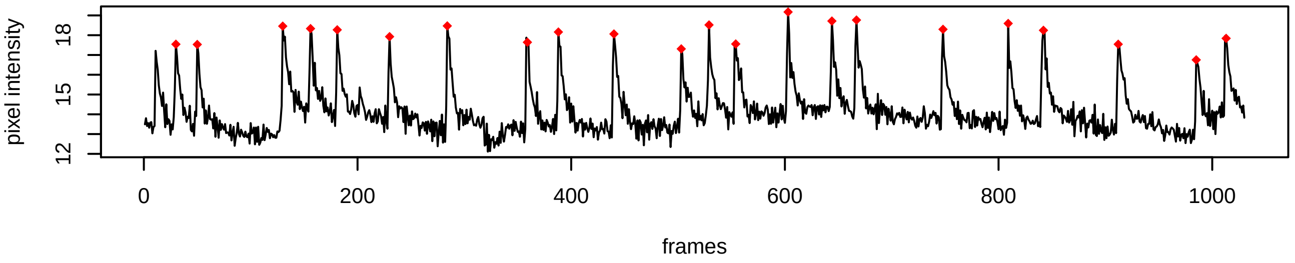

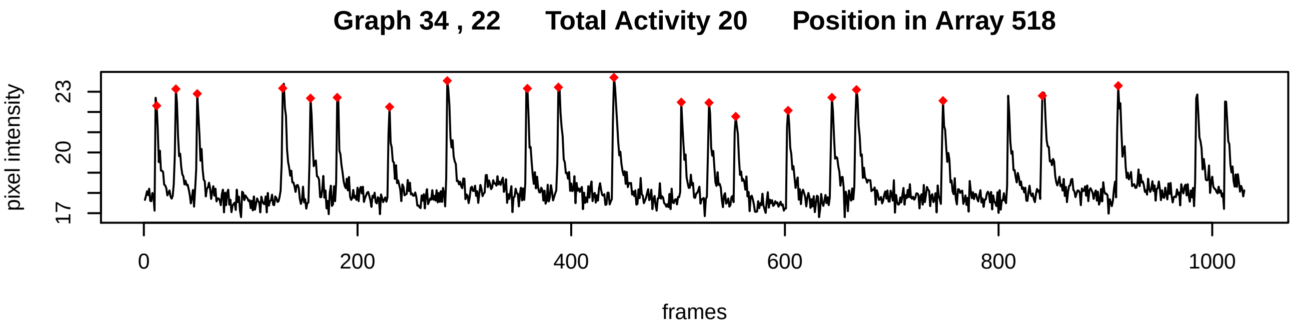
Graph 34 , 22 Total Activity 20 Position in Array 518
pixel intensity
17
20
23
0
200
400
600
800
1000
frames

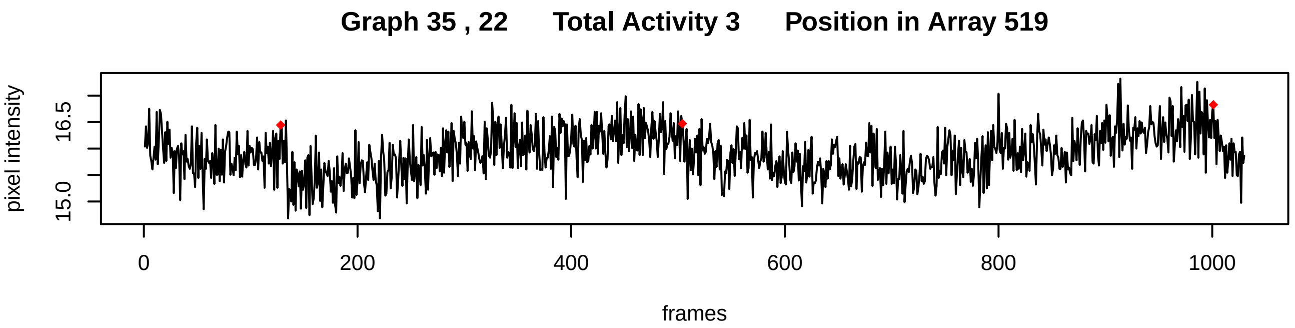
Graph 35 , 22 Total Activity 3 Position in Array 519
pixel intensity
15.0
16.5
0
200
400
600
800
1000
frames

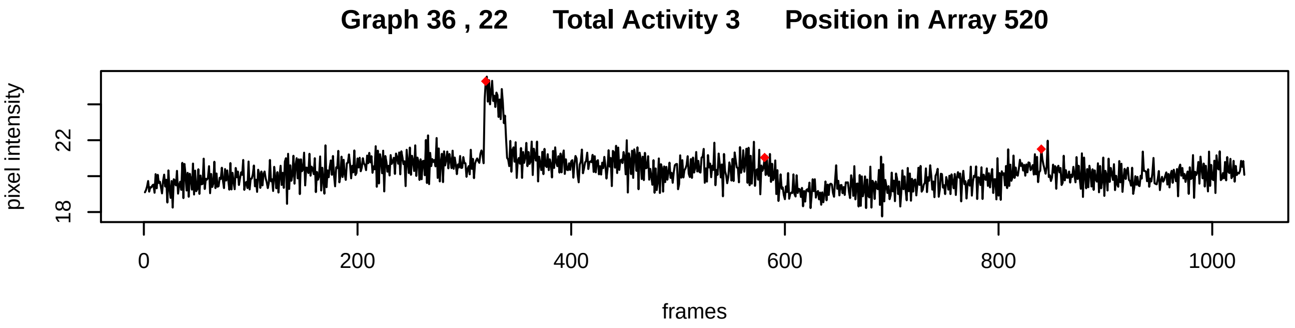
Graph 36 , 22 Total Activity 3 Position in Array 520
pixel intensity
18
22
0
200
400
600
800
1000
frames

Graph 37 , 22   Total Activity 4   Position in Array 521

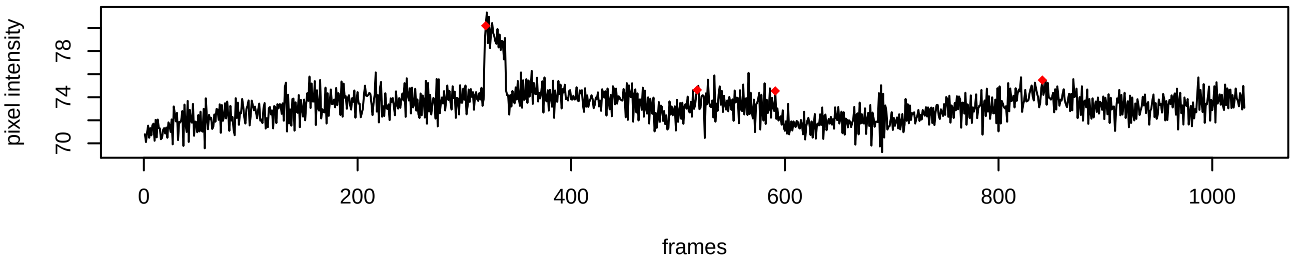

Graph 38 , 22   Total Activity 3   Position in Array 522

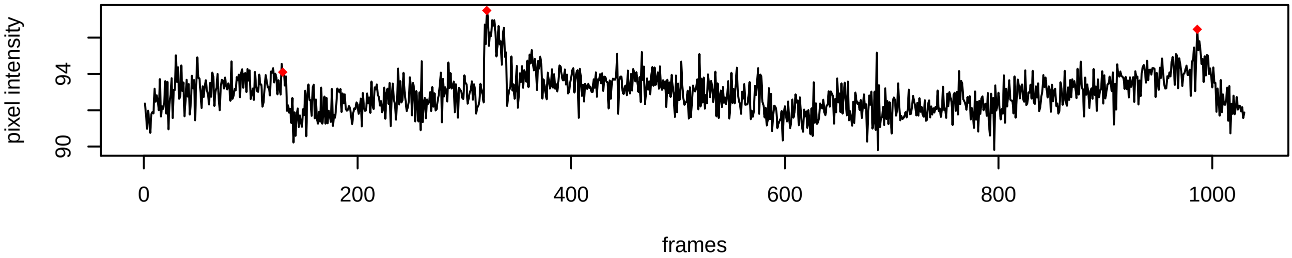

Graph 39 , 22   Total Activity 4   Position in Array 523

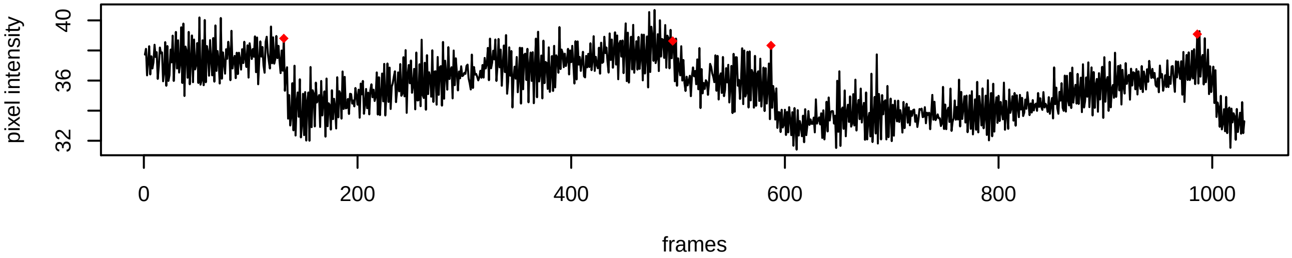

Graph 40 , 22 Total Activity 2 Position in Array 524

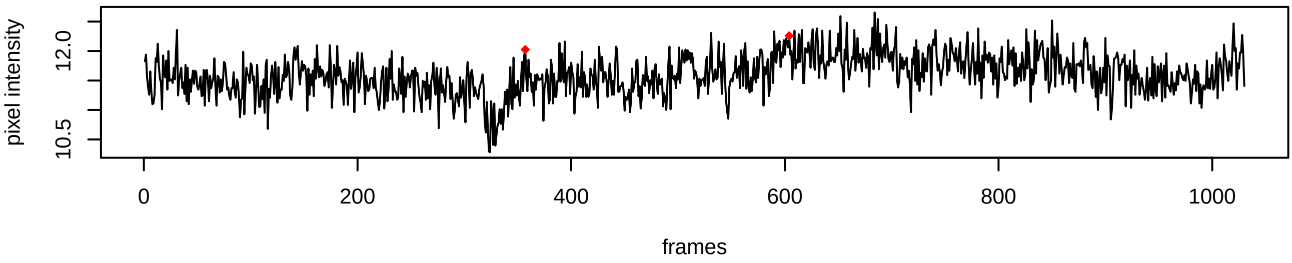

Graph 41 , 22 Total Activity 3 Position in Array 525

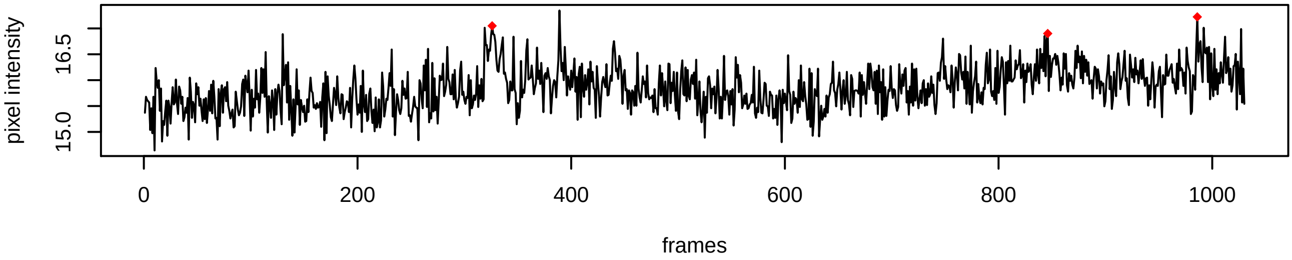

Graph 42 , 22 Total Activity 4 Position in Array 526

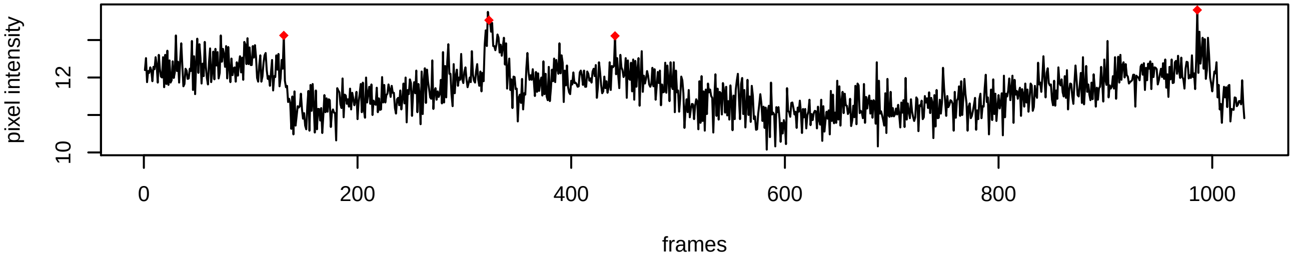

Graph 4 , 21 Total Activity 4 Position in Array 532

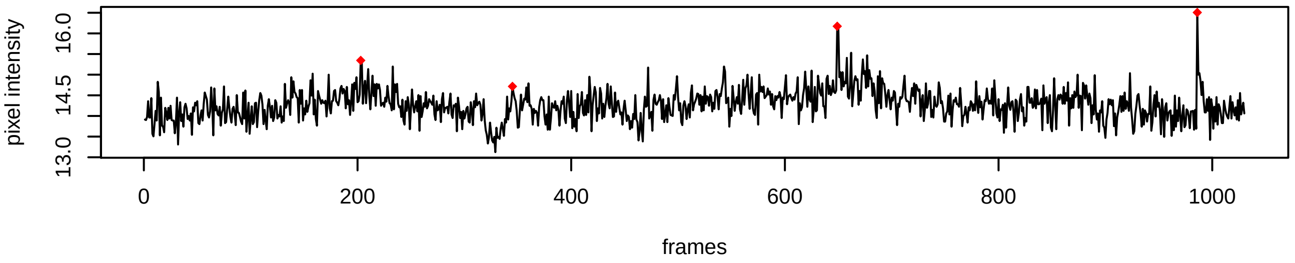

Graph 19 , 21 Total Activity 3 Position in Array 547

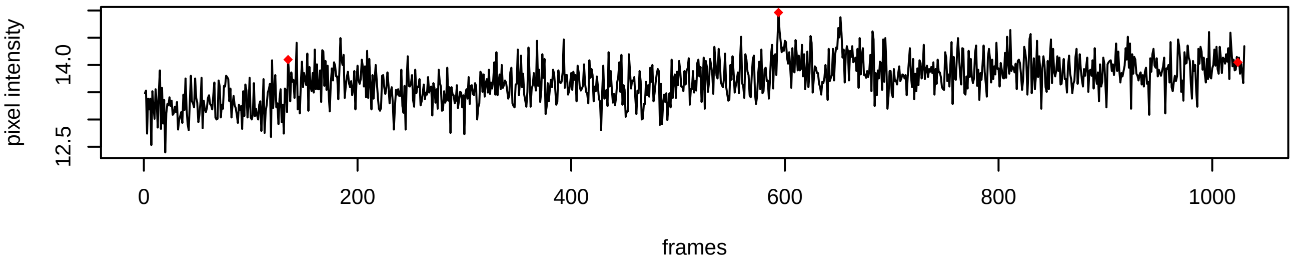

Graph 22 , 21 Total Activity 3 Position in Array 550

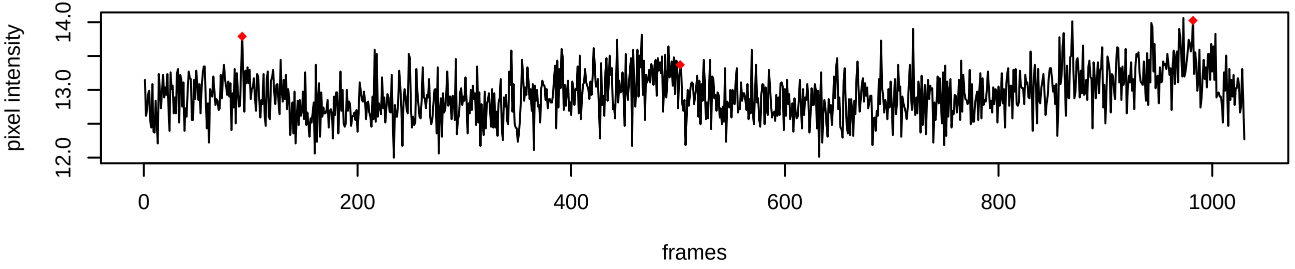

Graph 24 , 21   Total Activity 2   Position in Array 552

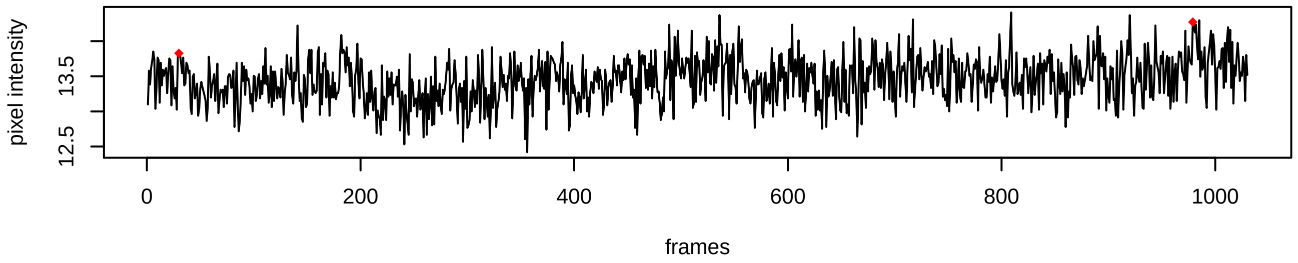

Graph 26 , 21   Total Activity 22   Position in Array 554

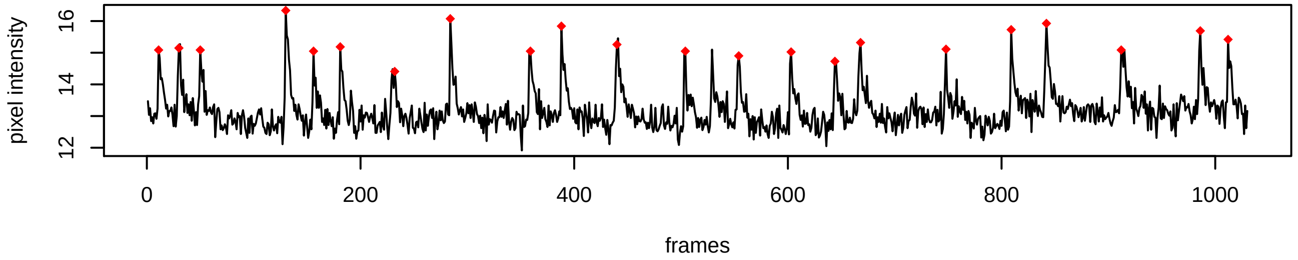

Graph 32 , 21   Total Activity 22   Position in Array 560

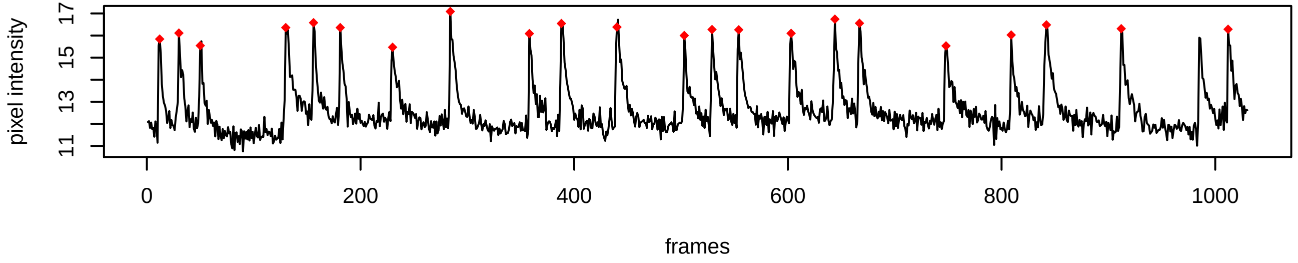

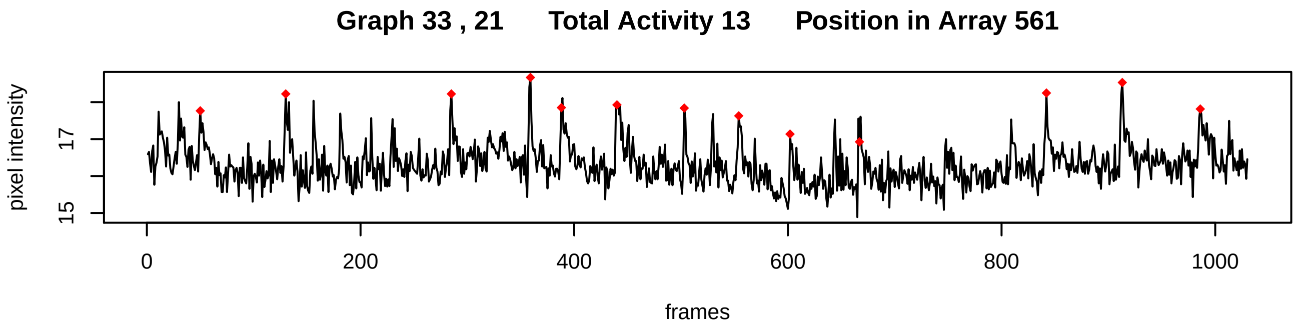
Graph 33 , 21 Total Activity 13 Position in Array 561
pixel intensity
15
17
0
200
400
600
800
1000
frames

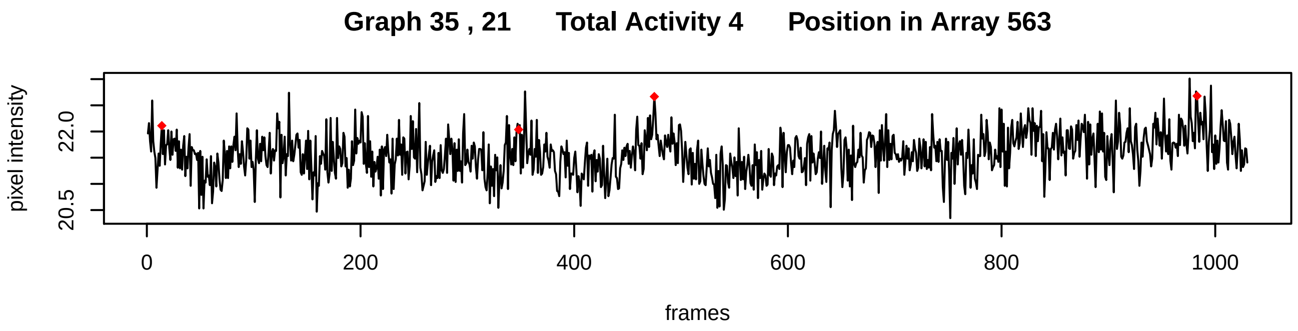
Graph 35 , 21 Total Activity 4 Position in Array 563
pixel intensity
20.5
22.0
0
200
400
600
800
1000
frames

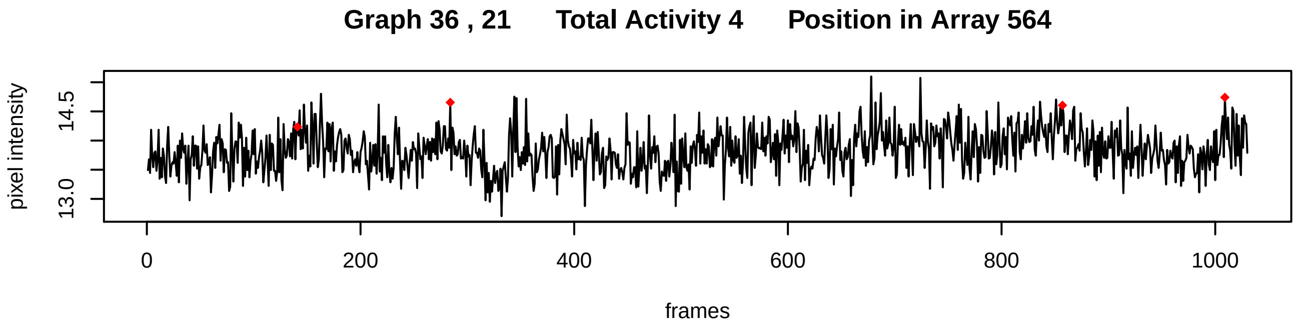
Graph 36 , 21 Total Activity 4 Position in Array 564
pixel intensity
13.0
14.5
0
200
400
600
800
1000
frames

Graph 37 , 21 Total Activity 4 Position in Array 565

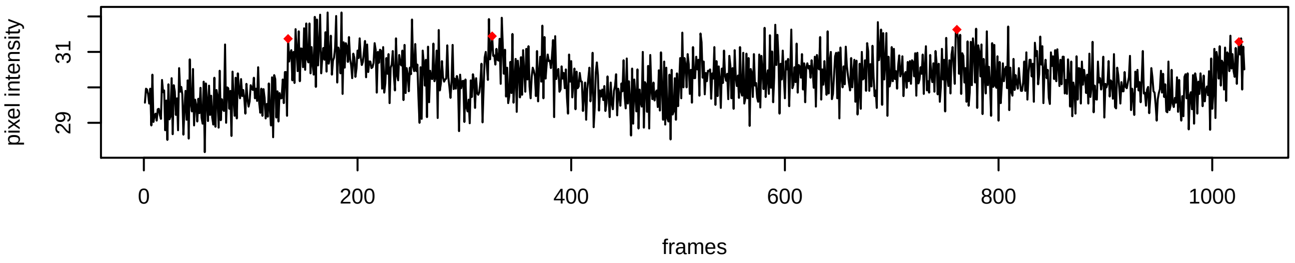

Graph 38 , 21 Total Activity 5 Position in Array 566

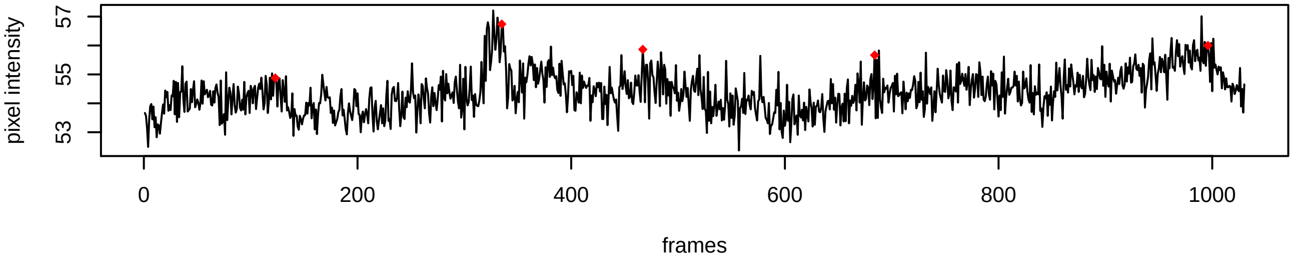

Graph 39 , 21 Total Activity 5 Position in Array 567

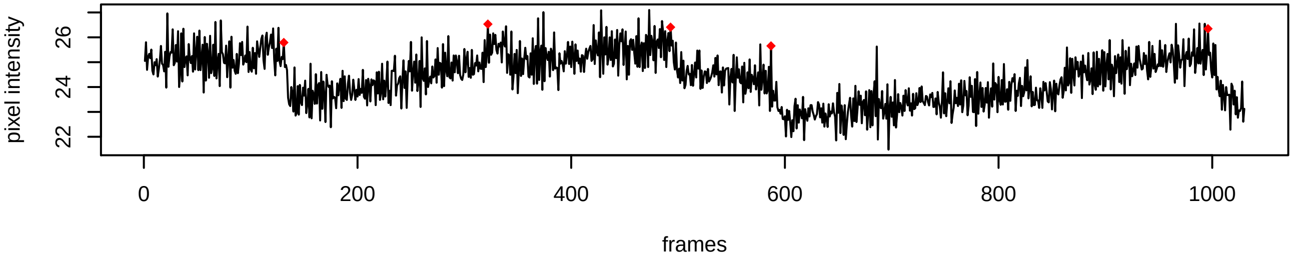

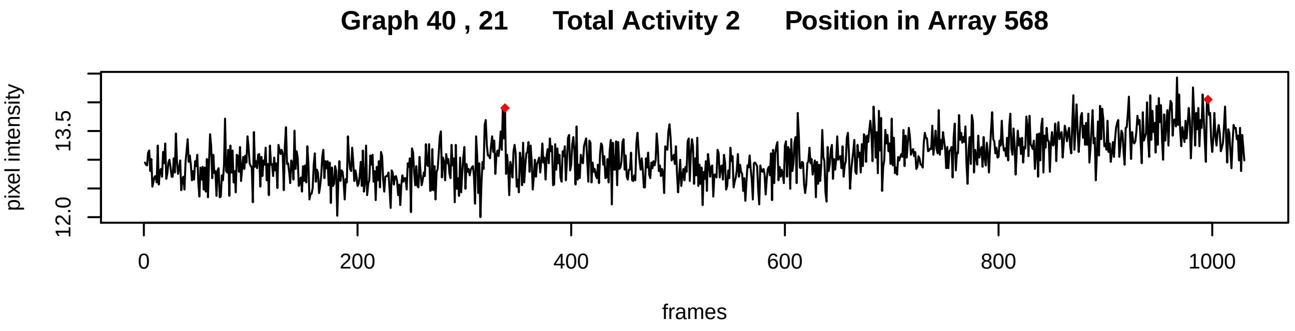
Graph 40 , 21 Total Activity 2 Position in Array 568
pixel intensity
12.0
13.5
frames
0
200
400
600
800
1000

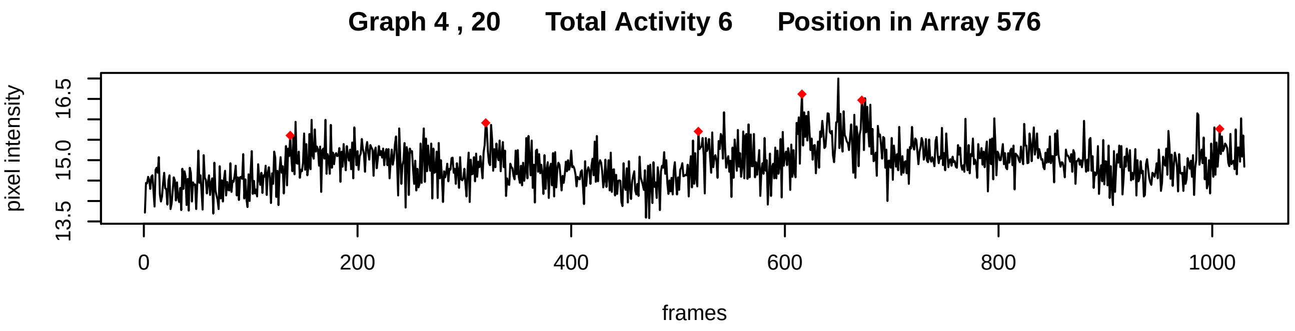
Graph 4 , 20 Total Activity 6 Position in Array 576
pixel intensity
13.5
15.0
16.5
frames
0
200
400
600
800
1000

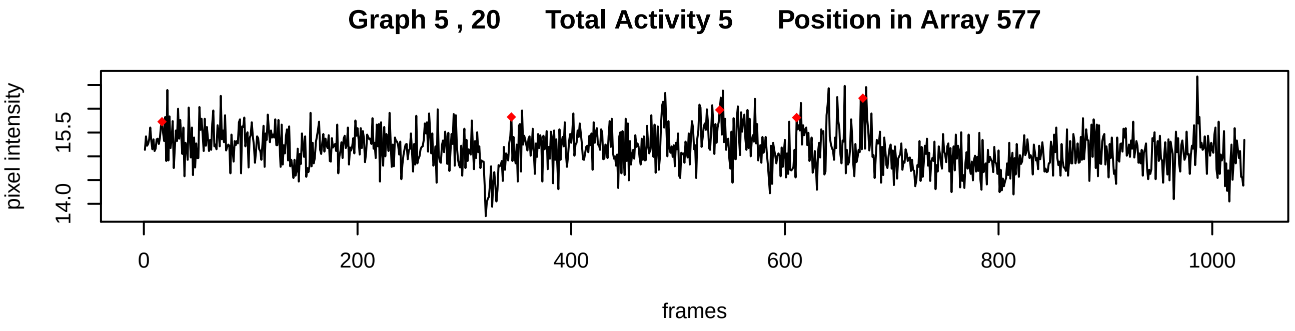
Graph 5 , 20 Total Activity 5 Position in Array 577
pixel intensity
14.0
15.5
frames
0
200
400
600
800
1000

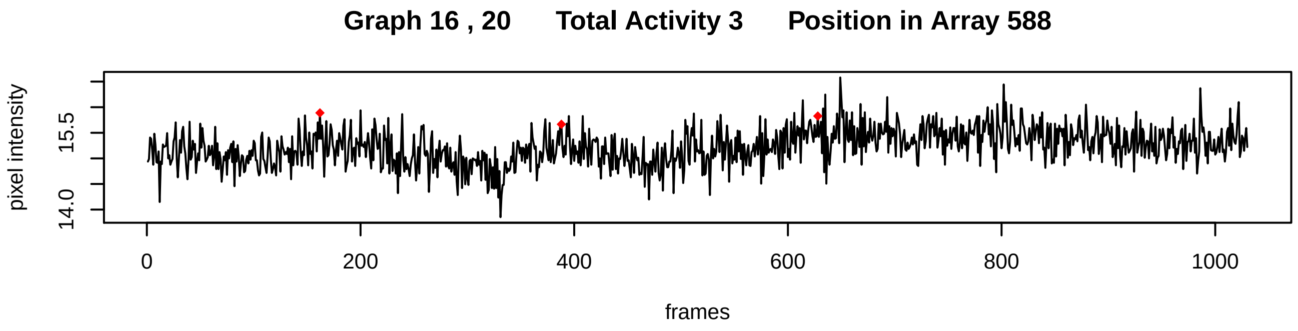
Graph 16 , 20 Total Activity 3 Position in Array 588
pixel intensity
14.0
15.5
0
200
400
600
800
1000
frames

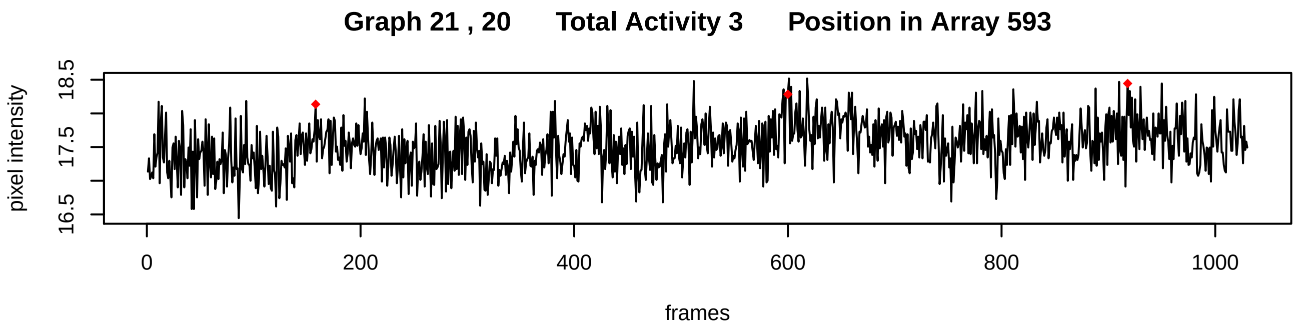
Graph 21 , 20 Total Activity 3 Position in Array 593
pixel intensity
16.5
17.5
18.5
0
200
400
600
800
1000
frames

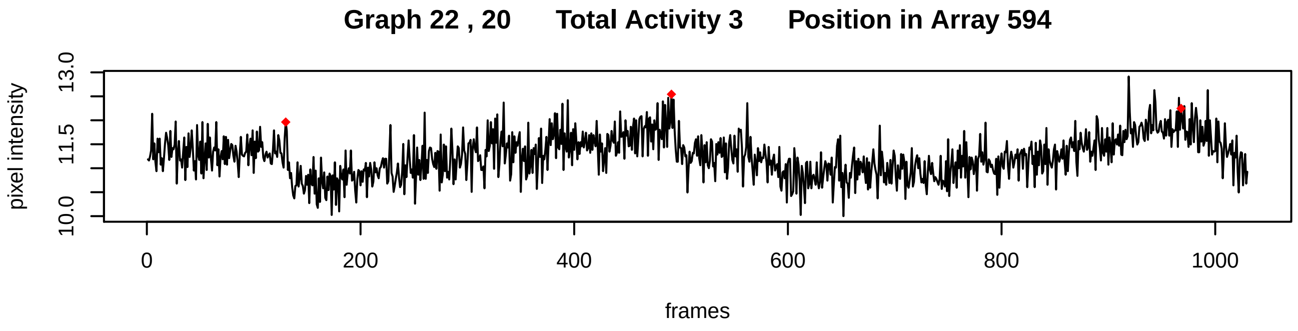
Graph 22 , 20 Total Activity 3 Position in Array 594
pixel intensity
10.0
11.5
13.0
0
200
400
600
800
1000
frames

Graph 23 , 20   Total Activity 4   Position in Array 595

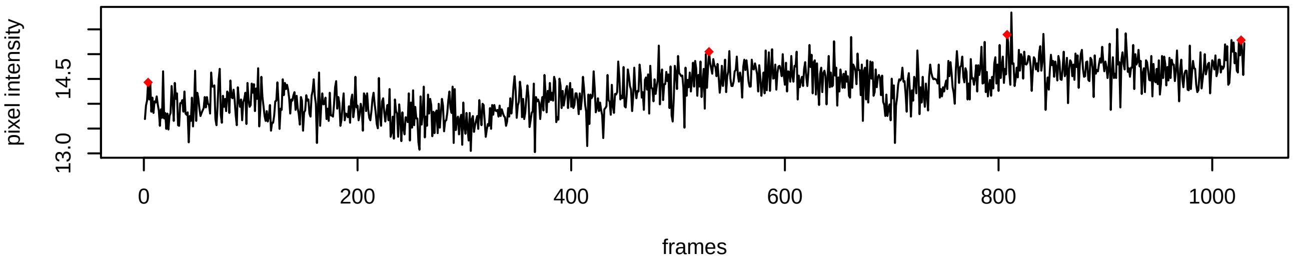

Graph 35 , 20   Total Activity 2   Position in Array 607

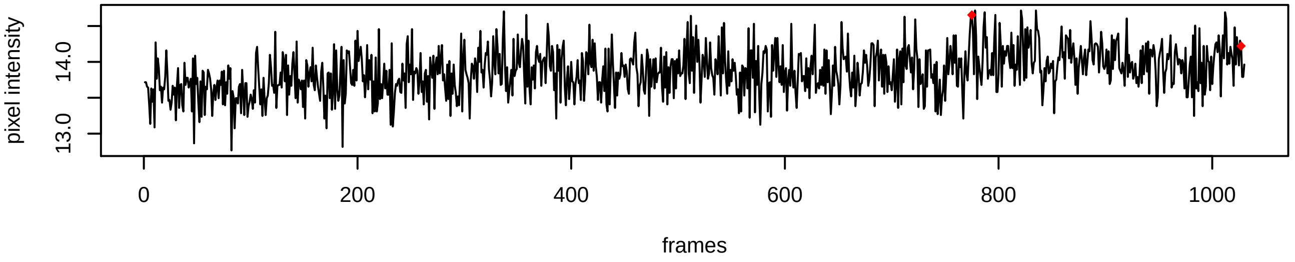

Graph 36 , 20   Total Activity 2   Position in Array 608

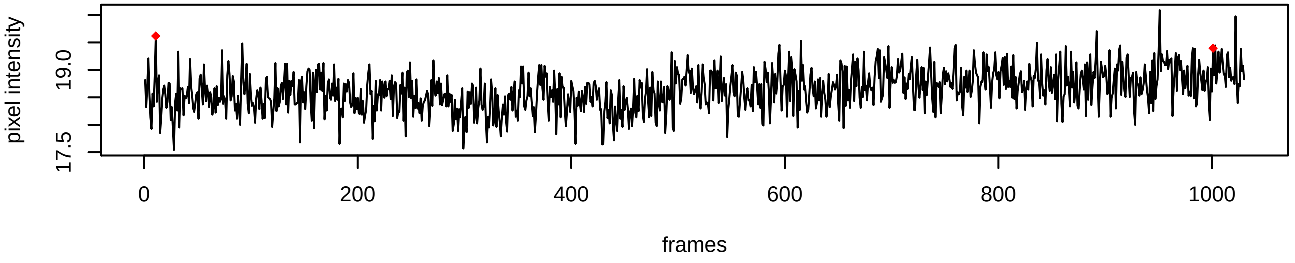

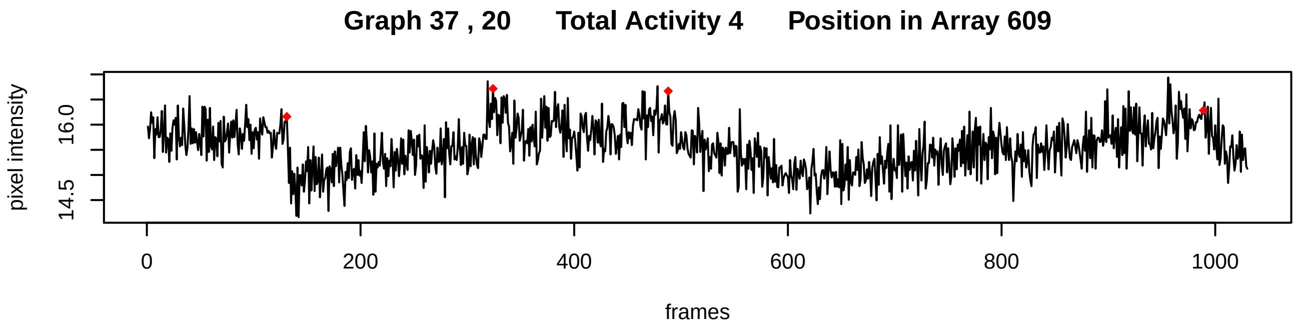
Graph 37 , 20 Total Activity 4 Position in Array 609
pixel intensity
16.0
14.5
0
200
400
600
800
1000
frames

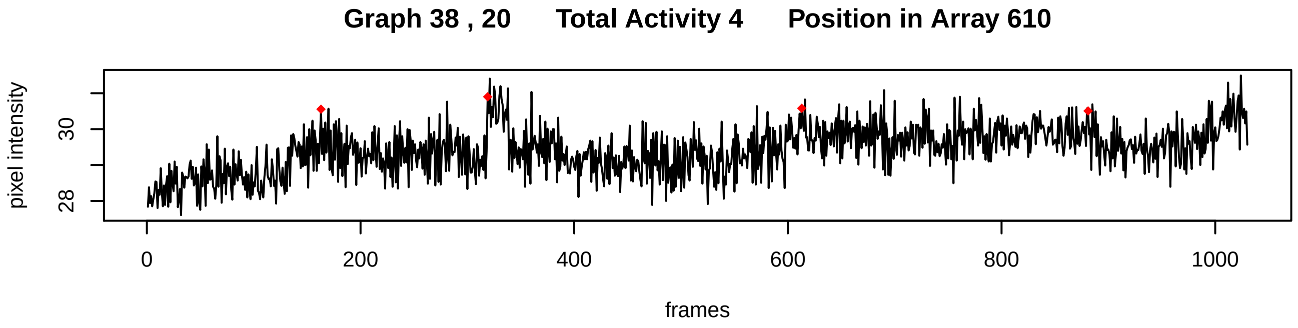
Graph 38 , 20 Total Activity 4 Position in Array 610
pixel intensity
30
28
0
200
400
600
800
1000
frames

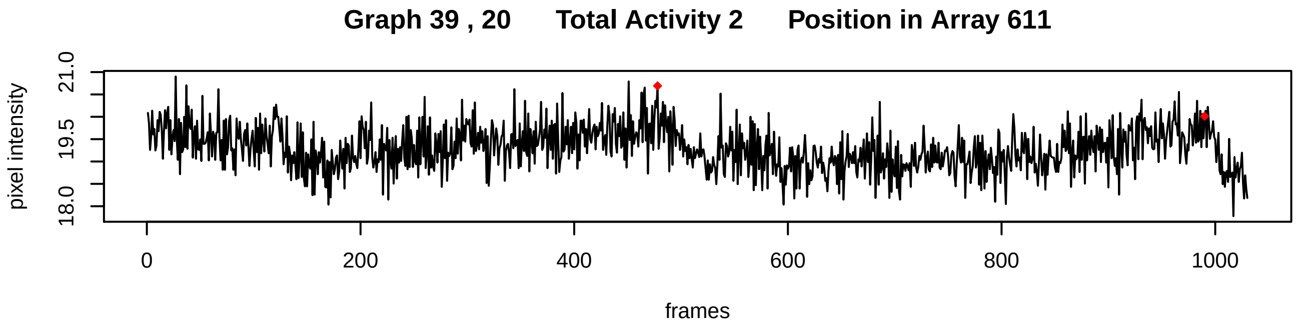
Graph 39 , 20 Total Activity 2 Position in Array 611
pixel intensity
21.0
19.5
18.0
0
200
400
600
800
1000
frames

Graph 40 , 20   Total Activity 3   Position in Array 612

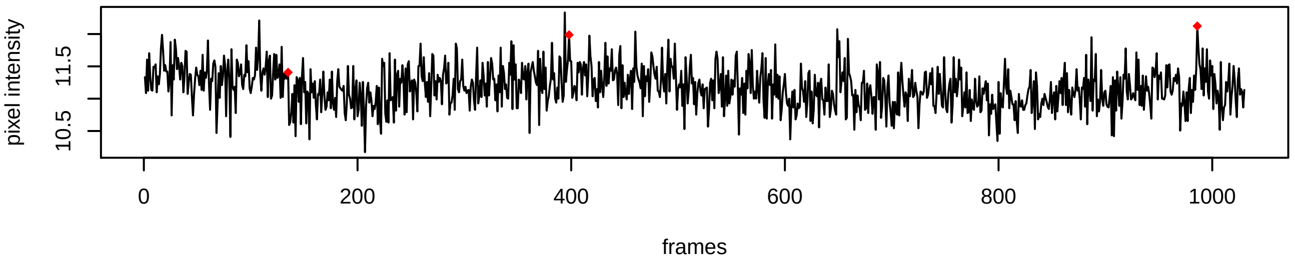

Graph 1 , 19   Total Activity 5   Position in Array 617

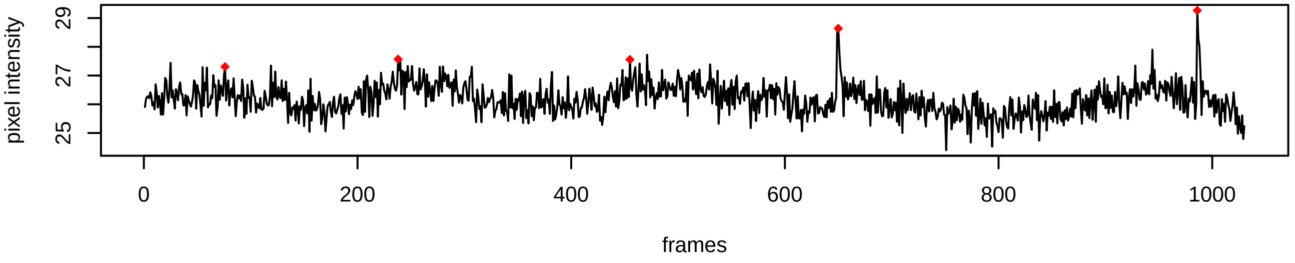

Graph 2 , 19   Total Activity 3   Position in Array 618

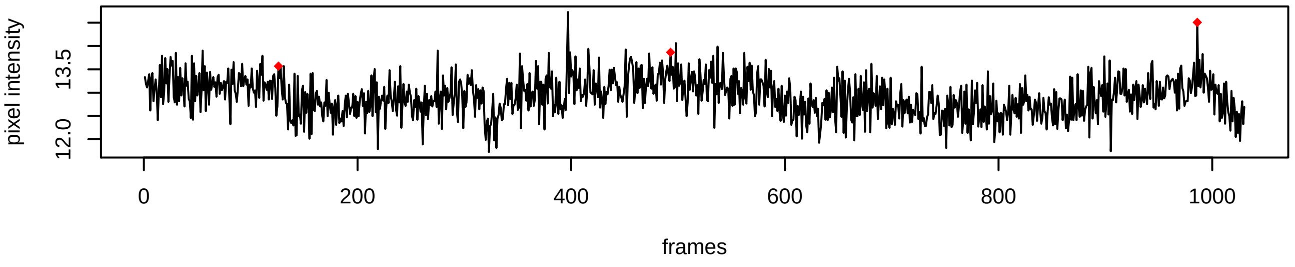

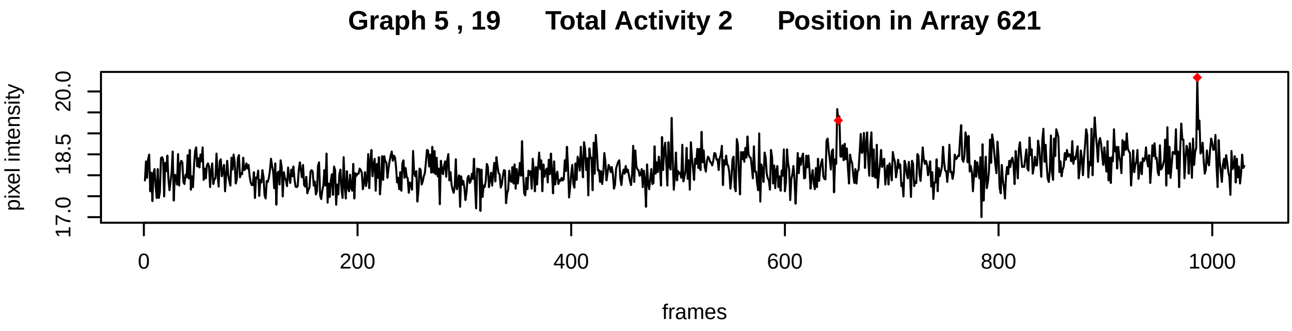
Graph 5 , 19 Total Activity 2 Position in Array 621
pixel intensity
17.0
18.5
20.0
0
200
400
600
800
1000
frames

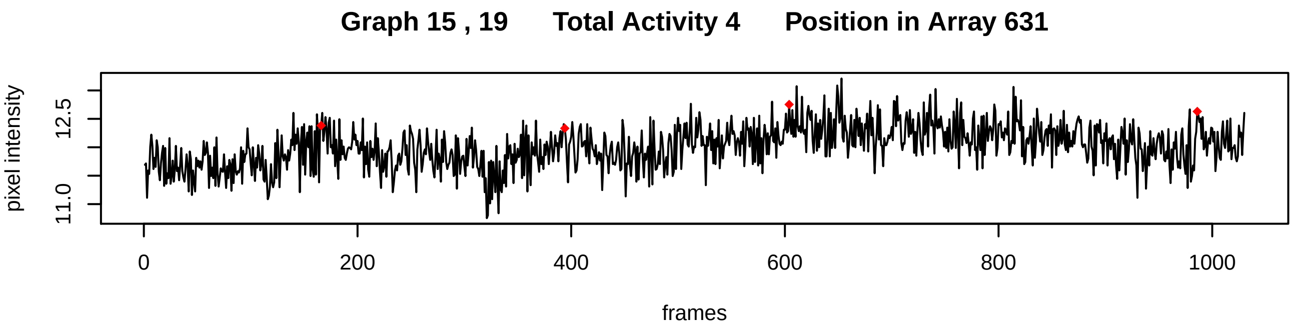
Graph 15 , 19 Total Activity 4 Position in Array 631
pixel intensity
11.0
12.5
0
200
400
600
800
1000
frames

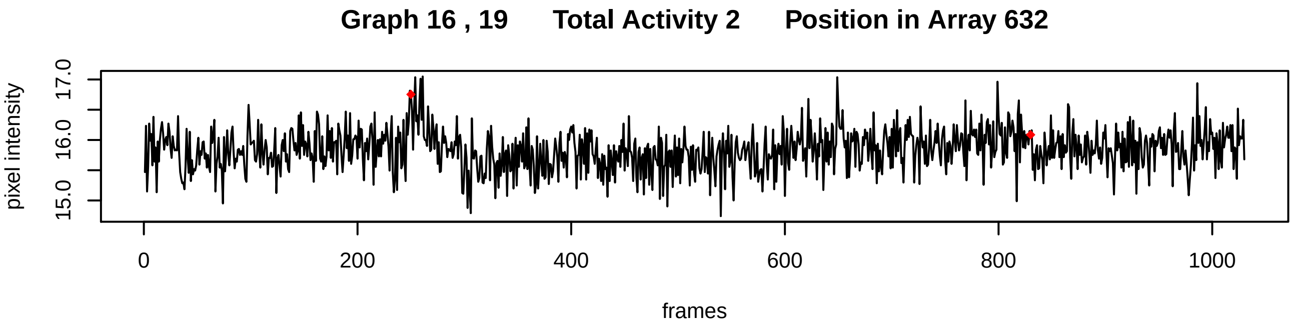
Graph 16 , 19 Total Activity 2 Position in Array 632
pixel intensity
15.0
16.0
17.0
0
200
400
600
800
1000
frames

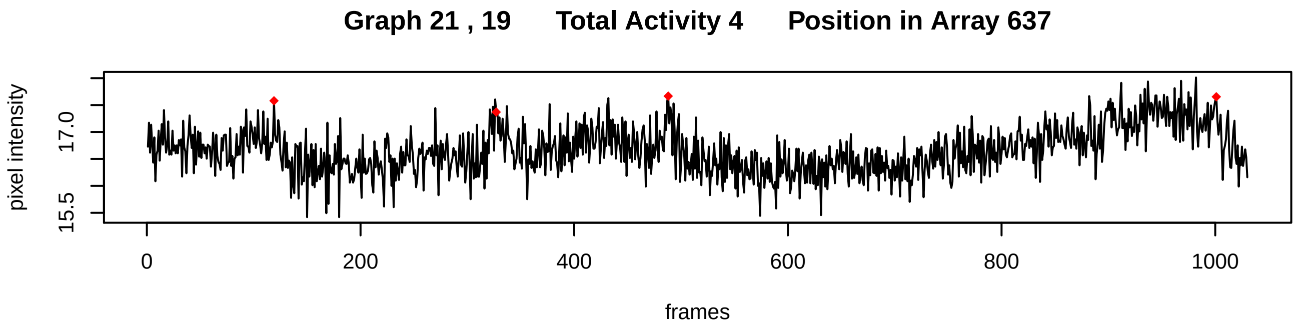
Graph 21 , 19 Total Activity 4 Position in Array 637
pixel intensity
15.5
17.0
frames
0
200
400
600
800
1000

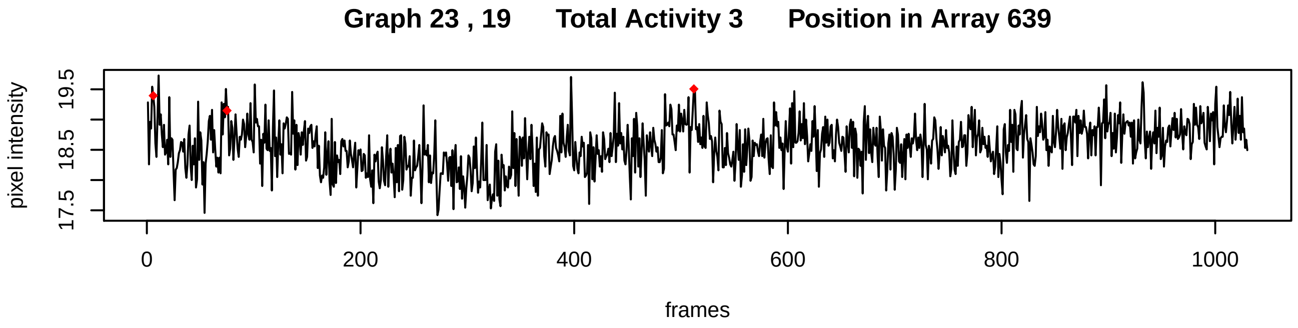
Graph 23 , 19 Total Activity 3 Position in Array 639
pixel intensity
17.5
18.5
19.5
frames
0
200
400
600
800
1000

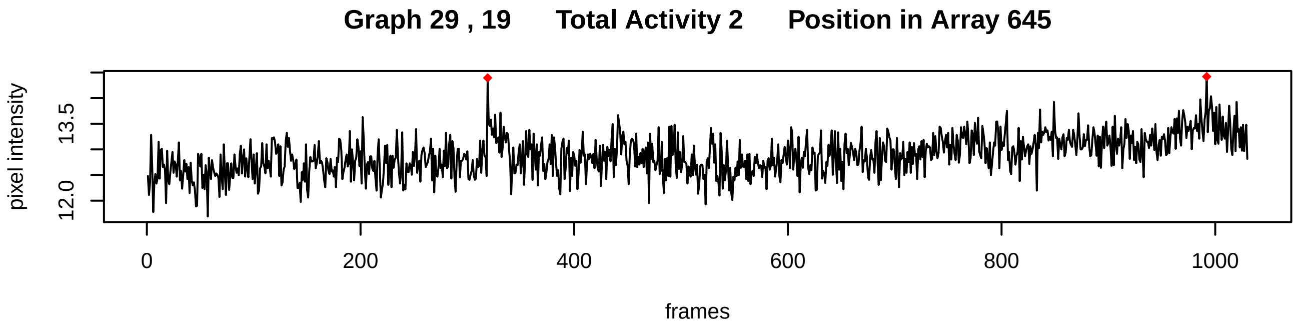
Graph 29 , 19 Total Activity 2 Position in Array 645
pixel intensity
12.0
13.5
frames
0
200
400
600
800
1000

Graph 34 , 19 Total Activity 4 Position in Array 650

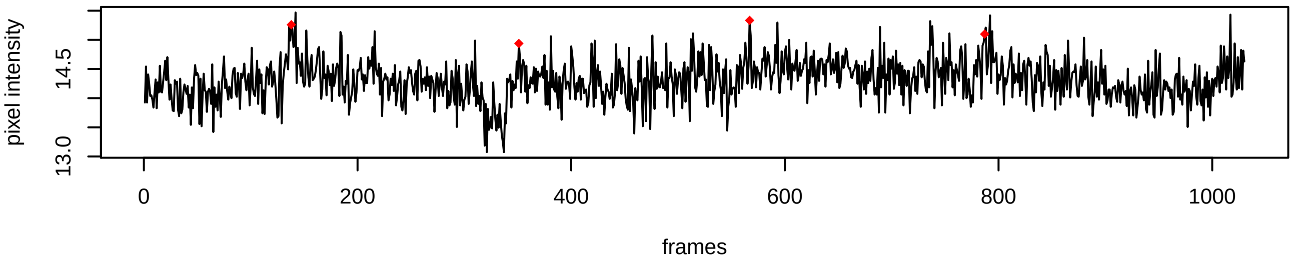

Graph 35 , 19 Total Activity 4 Position in Array 651

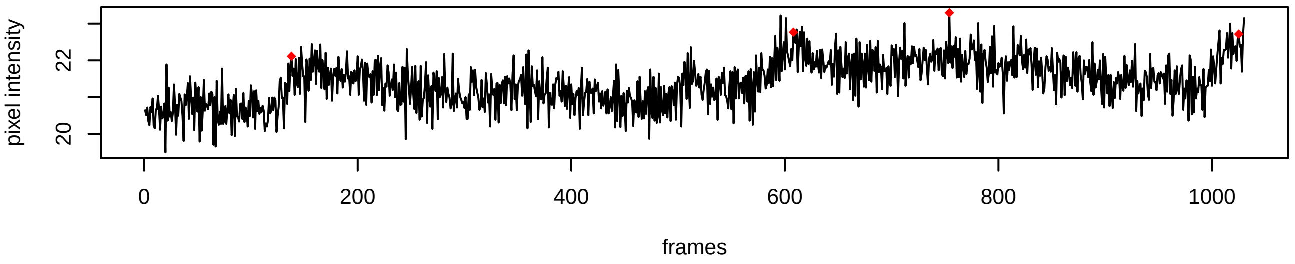

Graph 36 , 19 Total Activity 3 Position in Array 652

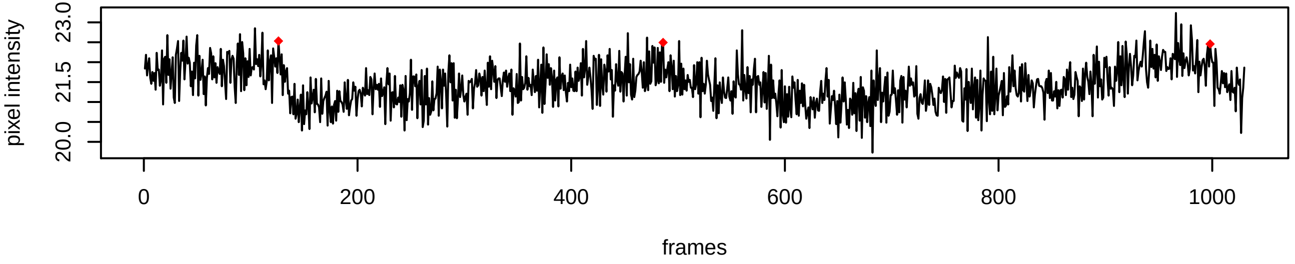

Graph 37 , 19 Total Activity 2 Position in Array 653

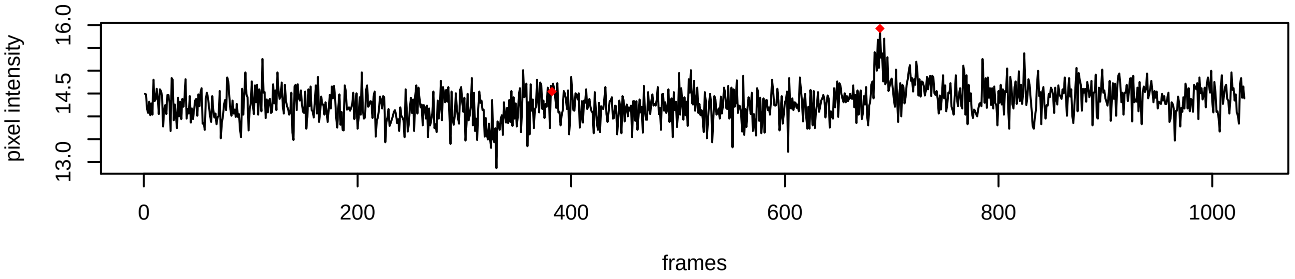

Graph 38 , 19 Total Activity 4 Position in Array 654

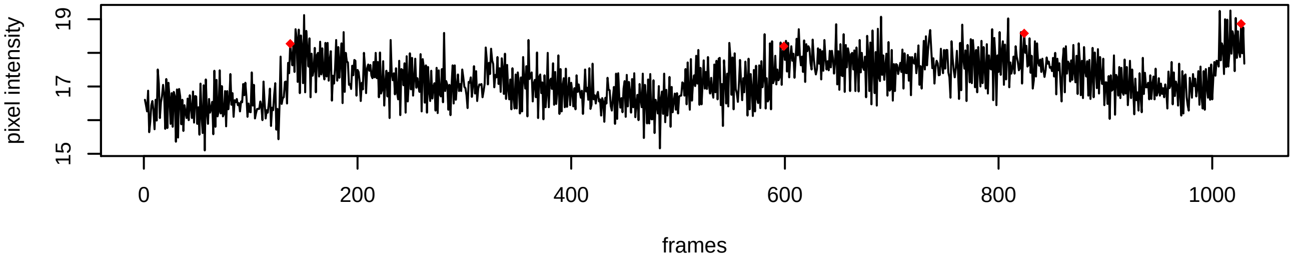

Graph 39 , 19 Total Activity 4 Position in Array 655

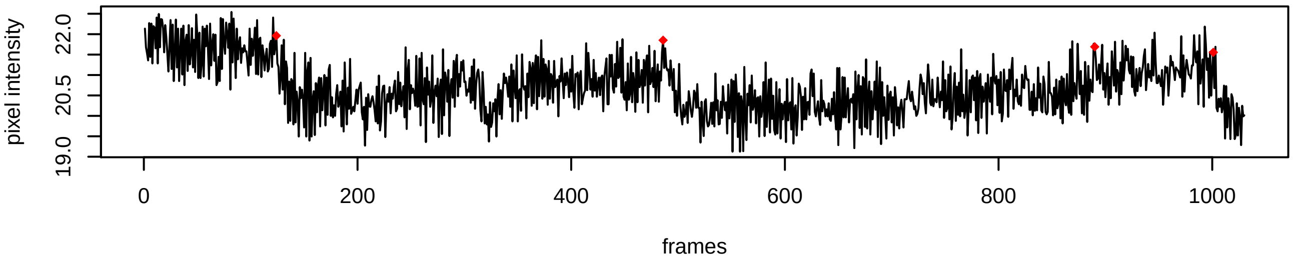

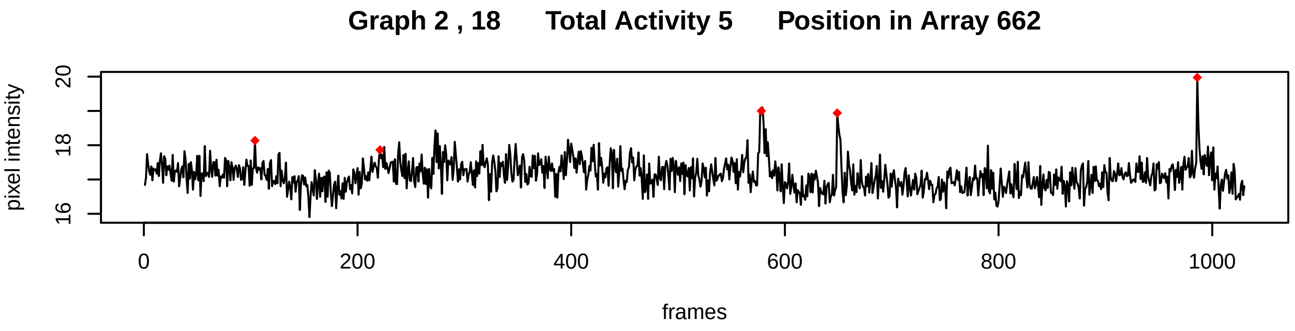
Graph 2 , 18 Total Activity 5 Position in Array 662
pixel intensity
16
18
20
0
200
400
600
800
1000
frames

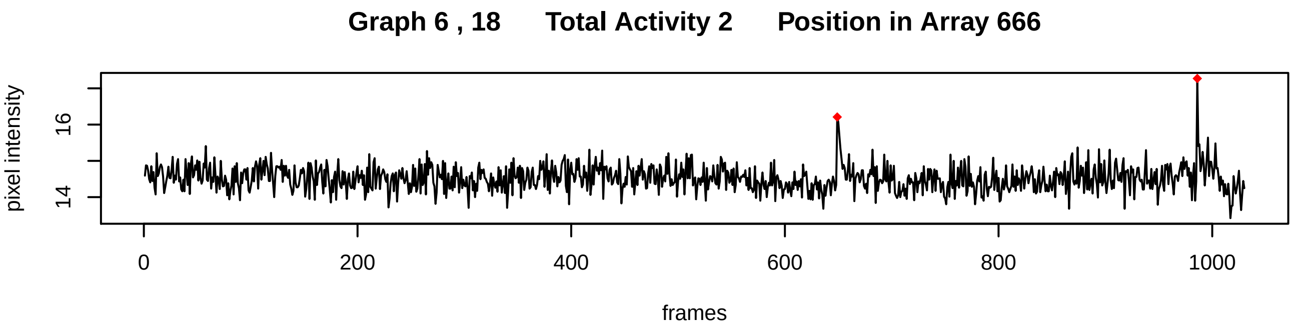
Graph 6 , 18 Total Activity 2 Position in Array 666
pixel intensity
14
16
0
200
400
600
800
1000
frames

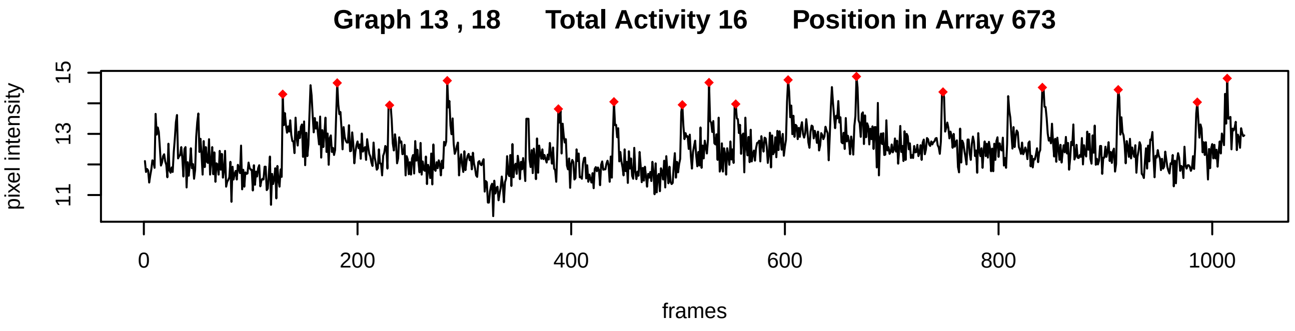
Graph 13 , 18 Total Activity 16 Position in Array 673
pixel intensity
11
13
15
0
200
400
600
800
1000
frames

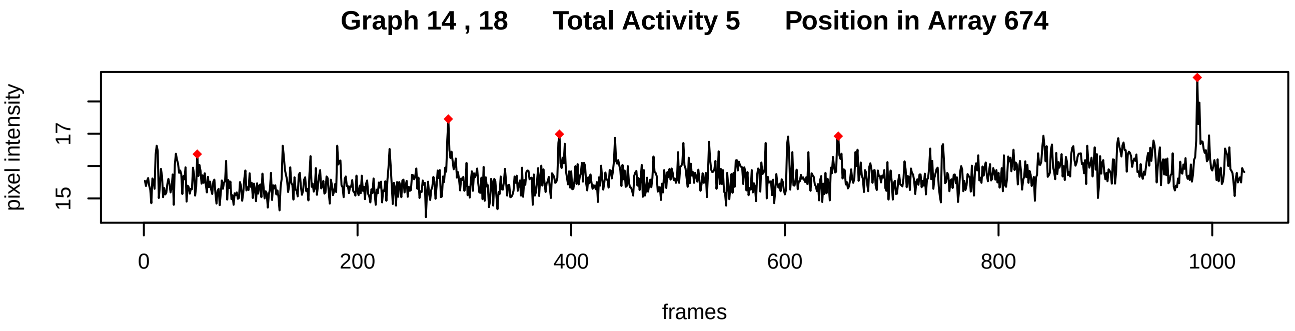
Graph 14 , 18 Total Activity 5 Position in Array 674
pixel intensity
15
17
0
200
400
600
800
1000
frames

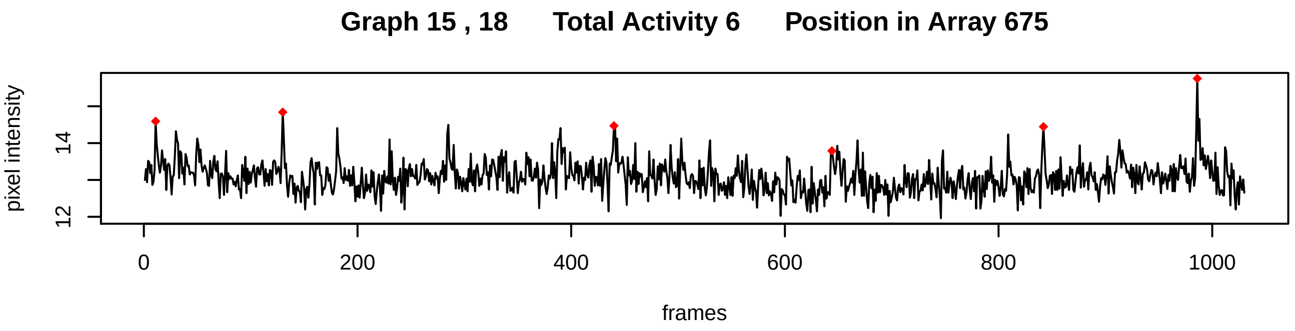
Graph 15 , 18 Total Activity 6 Position in Array 675
pixel intensity
12
14
0
200
400
600
800
1000
frames

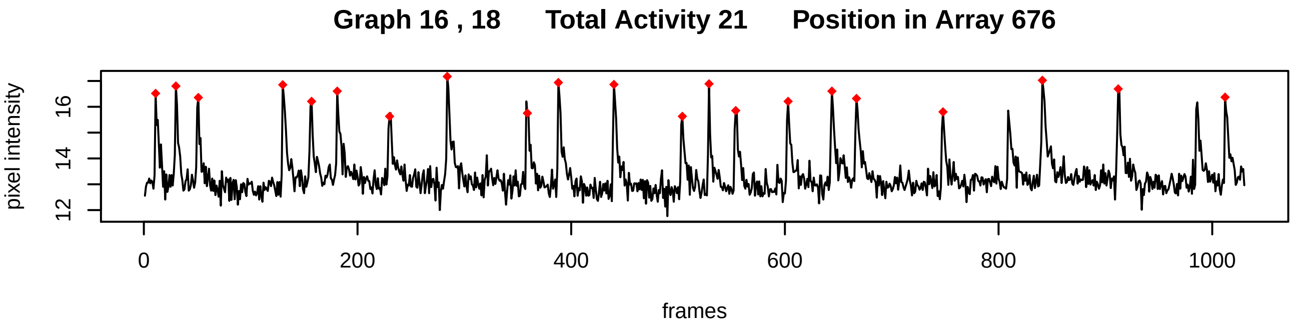
Graph 16 , 18 Total Activity 21 Position in Array 676
pixel intensity
12
14
16
0
200
400
600
800
1000
frames

Graph 21 , 18 Total Activity 4 Position in Array 681

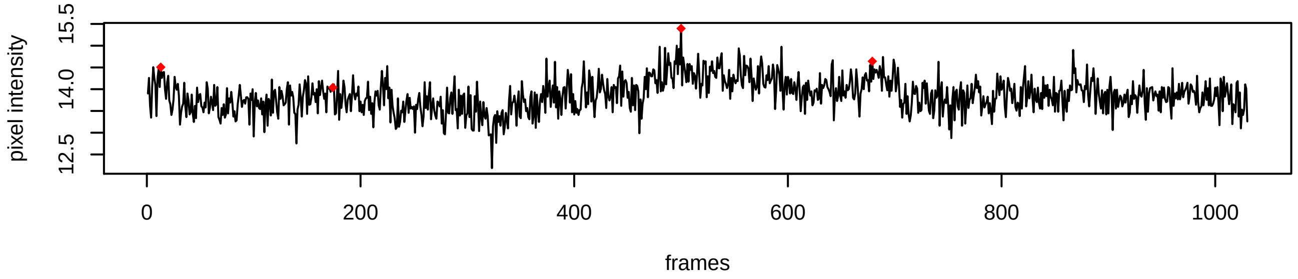

Graph 23 , 18 Total Activity 4 Position in Array 683

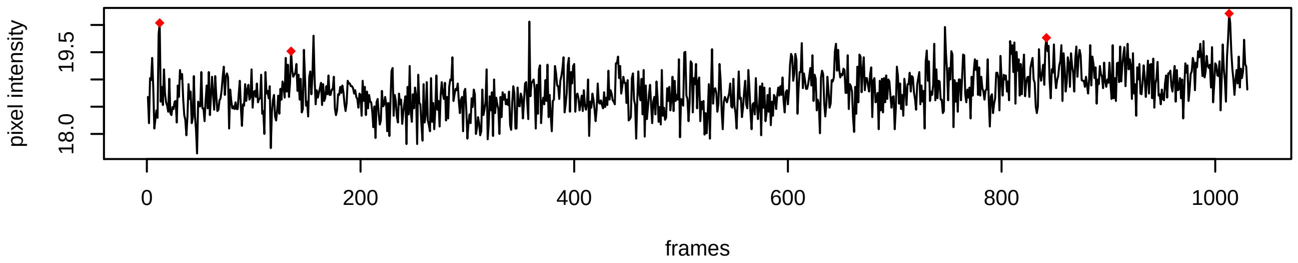

Graph 24 , 18 Total Activity 22 Position in Array 684

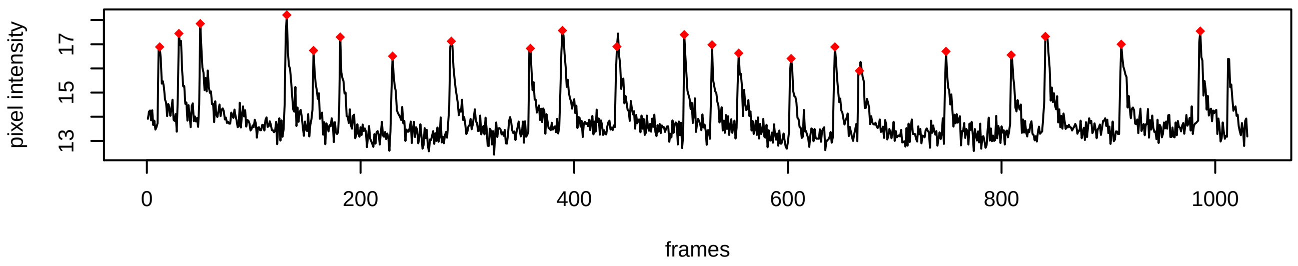

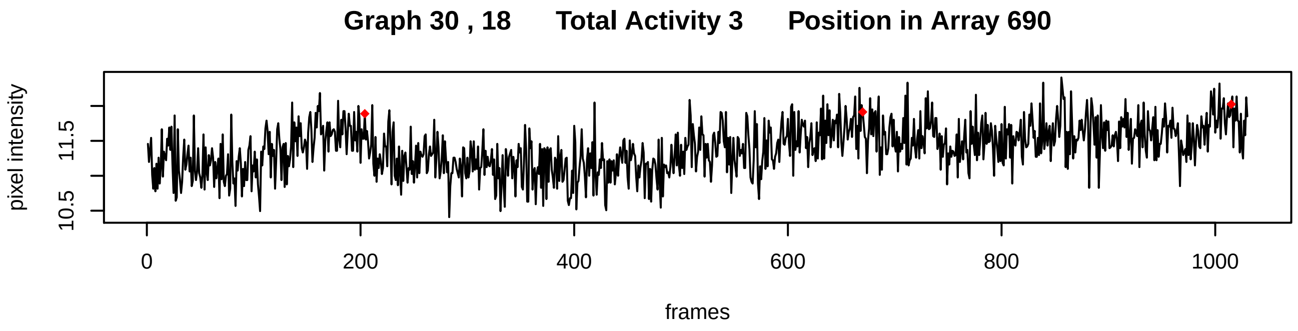
Graph 30 , 18 Total Activity 3 Position in Array 690
pixel intensity
10.5
11.5
0
200
400
600
800
1000
frames

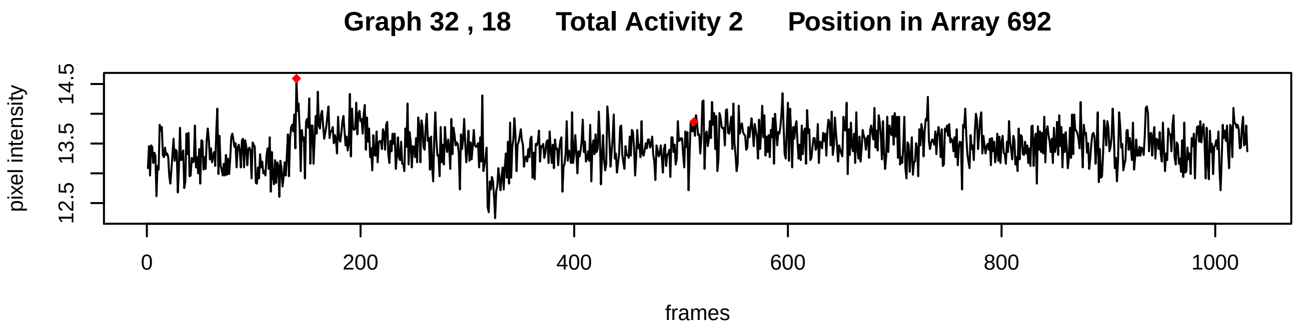
Graph 32 , 18 Total Activity 2 Position in Array 692
pixel intensity
12.5
13.5
14.5
0
200
400
600
800
1000
frames

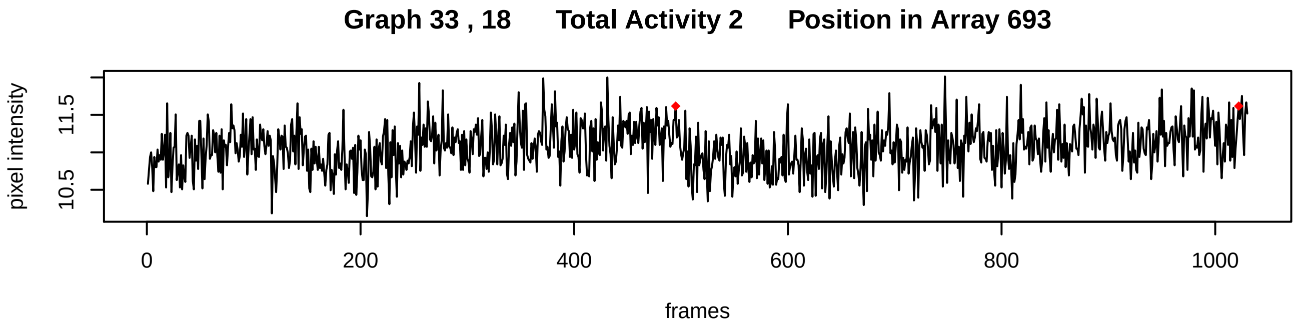
Graph 33 , 18 Total Activity 2 Position in Array 693
pixel intensity
10.5
11.5
0
200
400
600
800
1000
frames

Graph 34 , 18   Total Activity 3   Position in Array 694

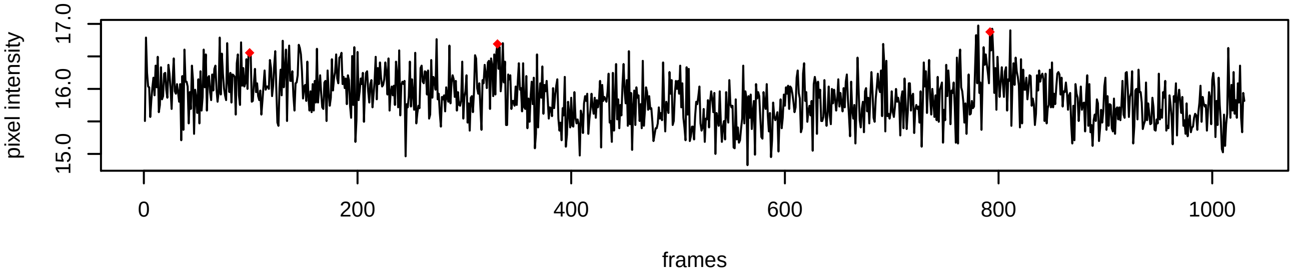

Graph 35 , 18   Total Activity 4   Position in Array 695

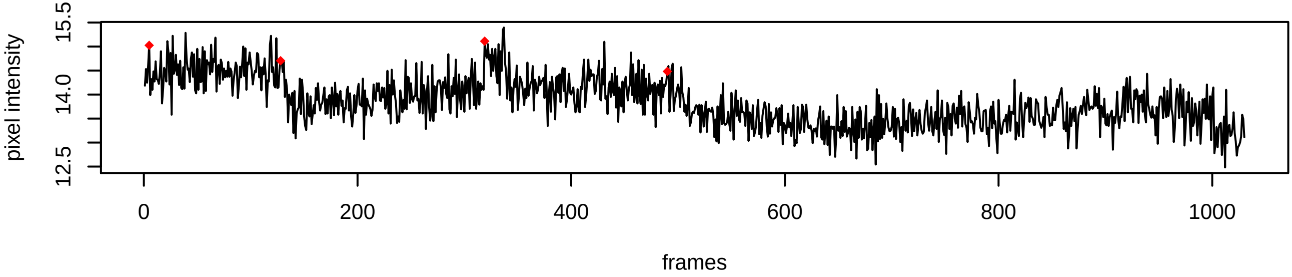

Graph 36 , 18   Total Activity 2   Position in Array 696

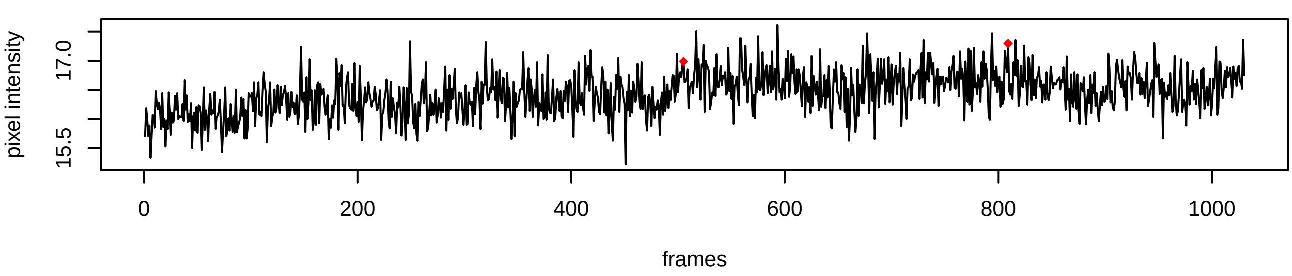

Graph 37 , 18   Total Activity 4   Position in Array 697

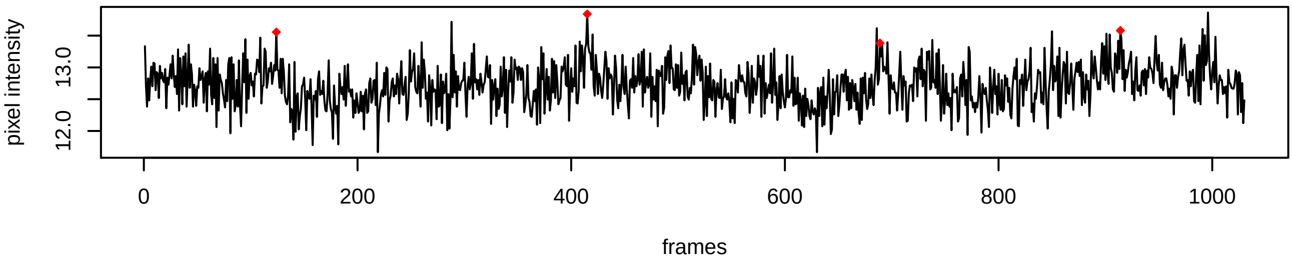

Graph 38 , 18   Total Activity 4   Position in Array 698

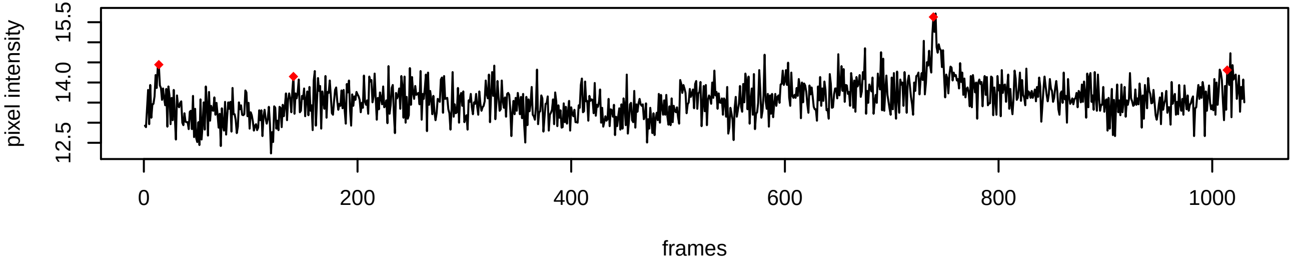

Graph 39 , 18   Total Activity 3   Position in Array 699

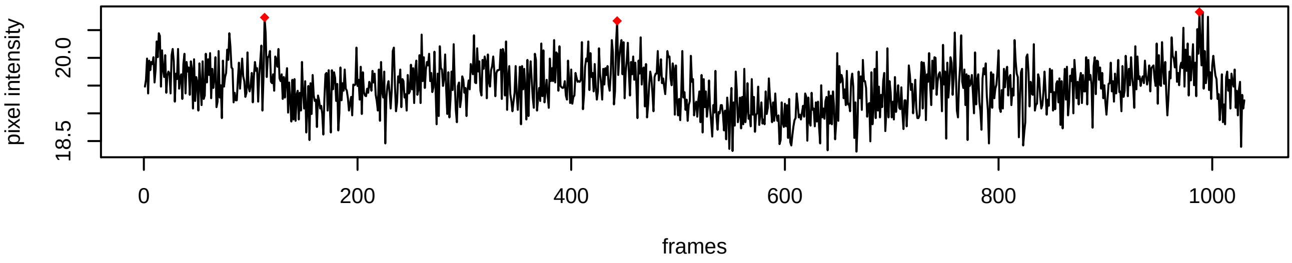

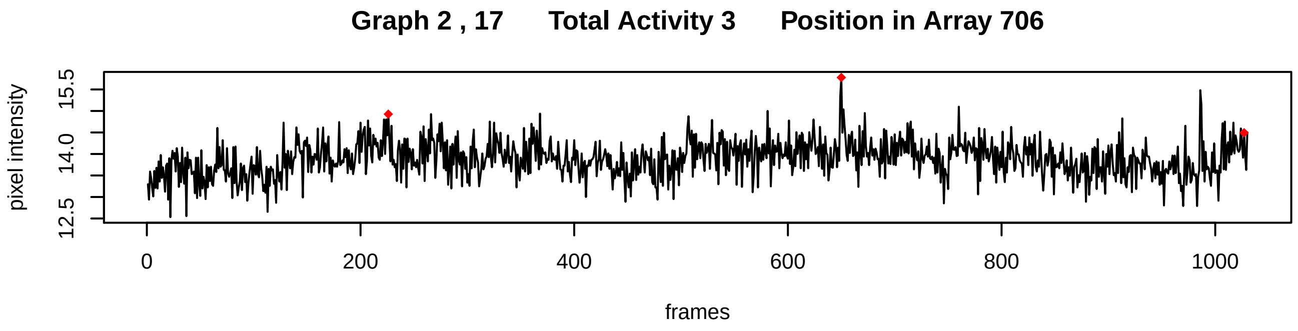
Graph 2 , 17 Total Activity 3 Position in Array 706
pixel intensity
12.5
14.0
15.5
0
200
400
600
800
1000
frames

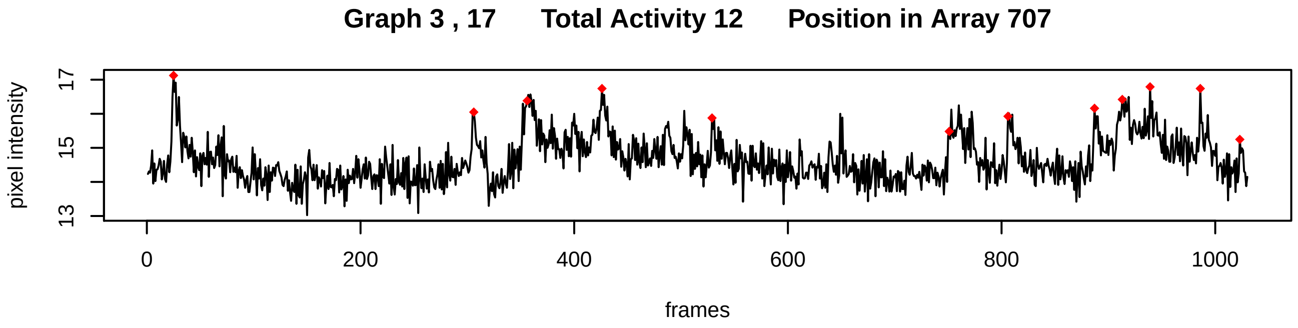
Graph 3 , 17 Total Activity 12 Position in Array 707
pixel intensity
13
15
17
0
200
400
600
800
1000
frames

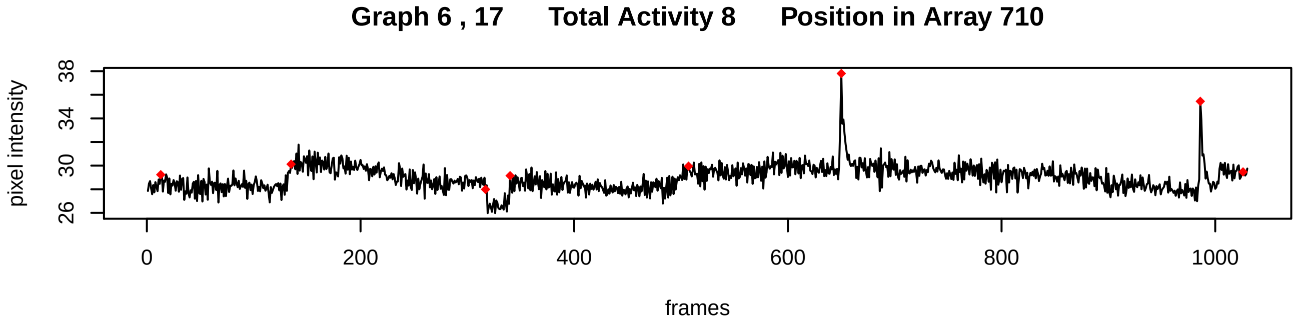
Graph 6 , 17 Total Activity 8 Position in Array 710
pixel intensity
26
30
34
38
0
200
400
600
800
1000
frames

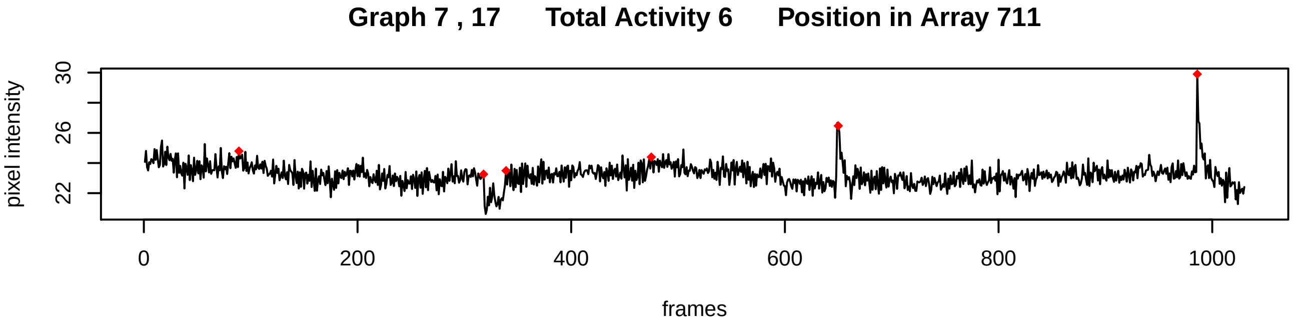
Graph 7 , 17 Total Activity 6 Position in Array 711
pixel intensity
22
26
30
0
200
400
600
800
1000
frames

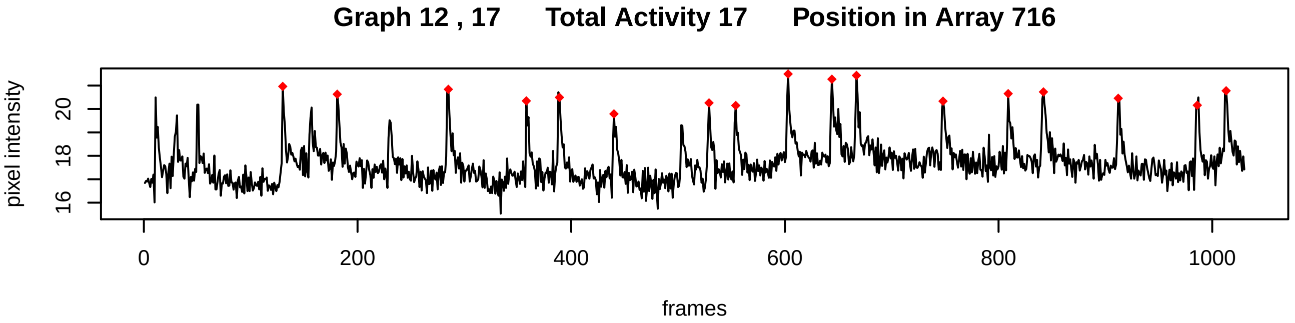
Graph 12 , 17 Total Activity 17 Position in Array 716
pixel intensity
16
18
20
0
200
400
600
800
1000
frames

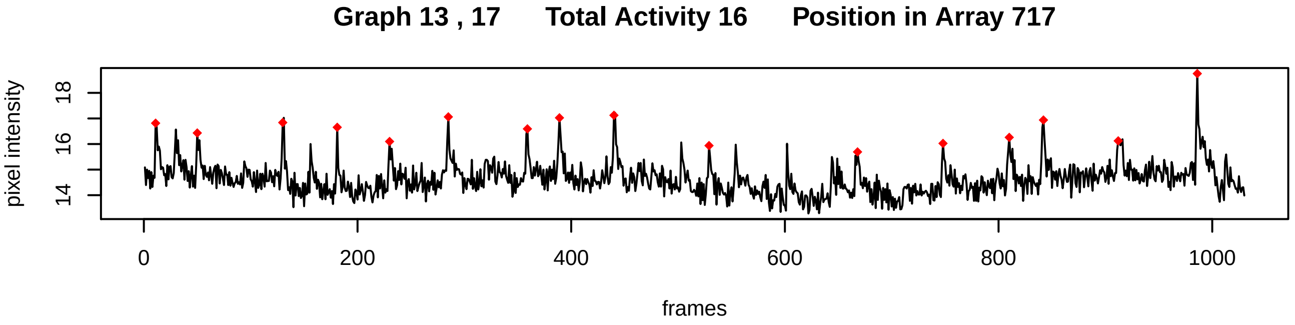
Graph 13 , 17 Total Activity 16 Position in Array 717
pixel intensity
14
16
18
0
200
400
600
800
1000
frames

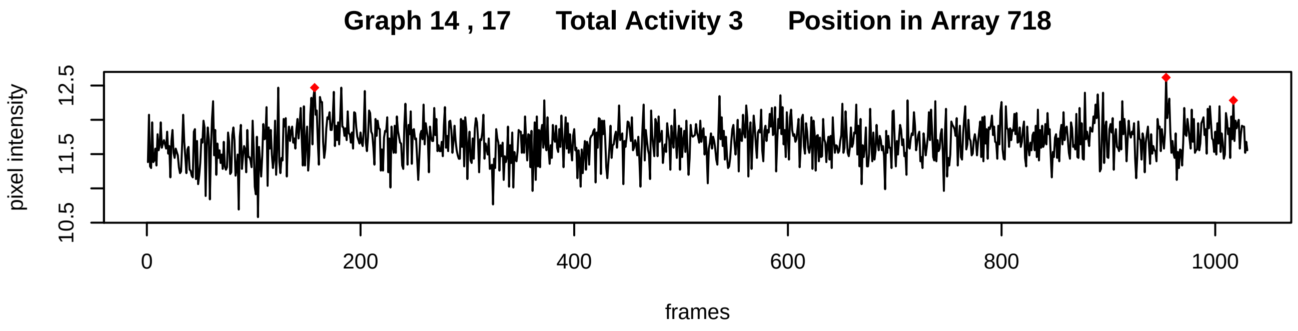
Graph 14 , 17 Total Activity 3 Position in Array 718
pixel intensity
10.5
11.5
12.5
frames
0
200
400
600
800
1000

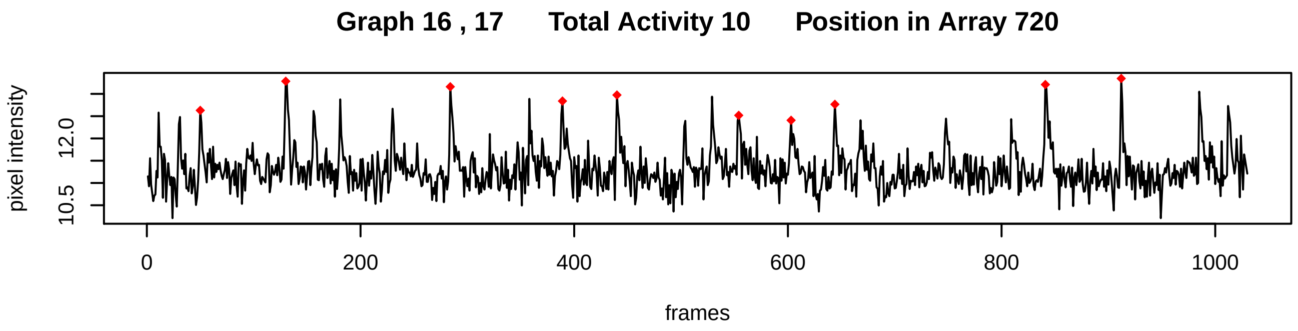
Graph 16 , 17 Total Activity 10 Position in Array 720
pixel intensity
10.5
12.0
frames
0
200
400
600
800
1000

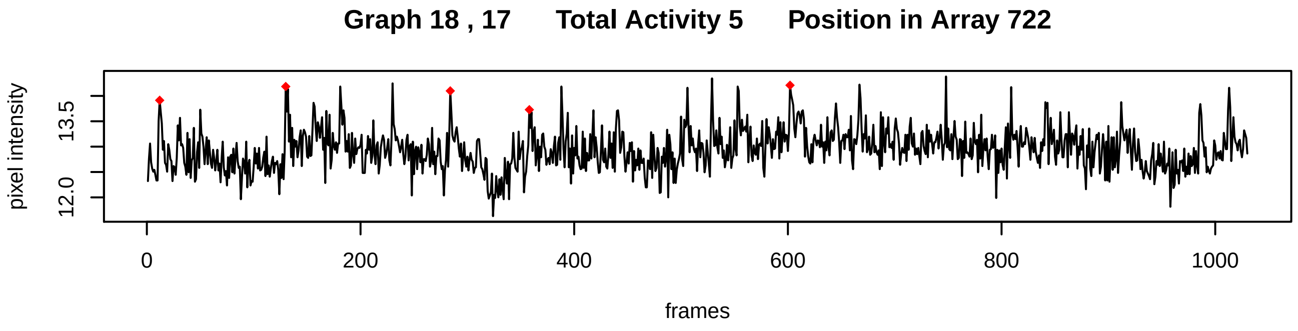
Graph 18 , 17 Total Activity 5 Position in Array 722
pixel intensity
12.0
13.5
frames
0
200
400
600
800
1000

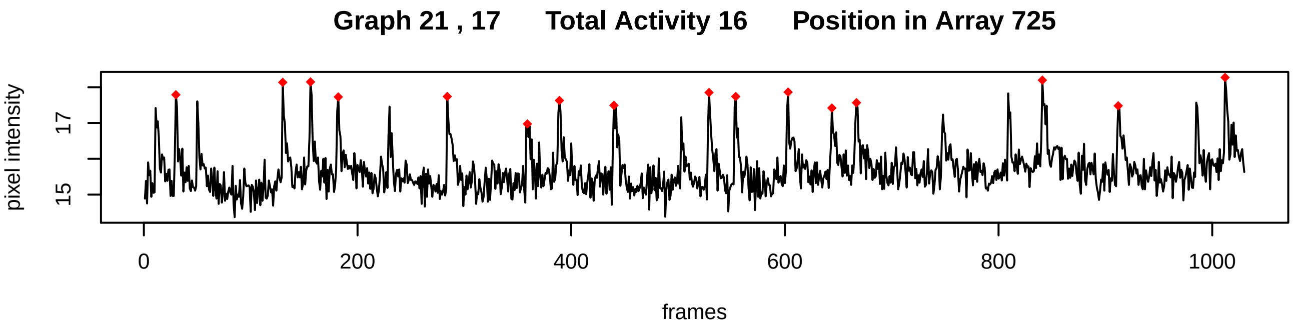
Graph 21 , 17 Total Activity 16 Position in Array 725
pixel intensity
15
17
0
200
400
600
800
1000
frames

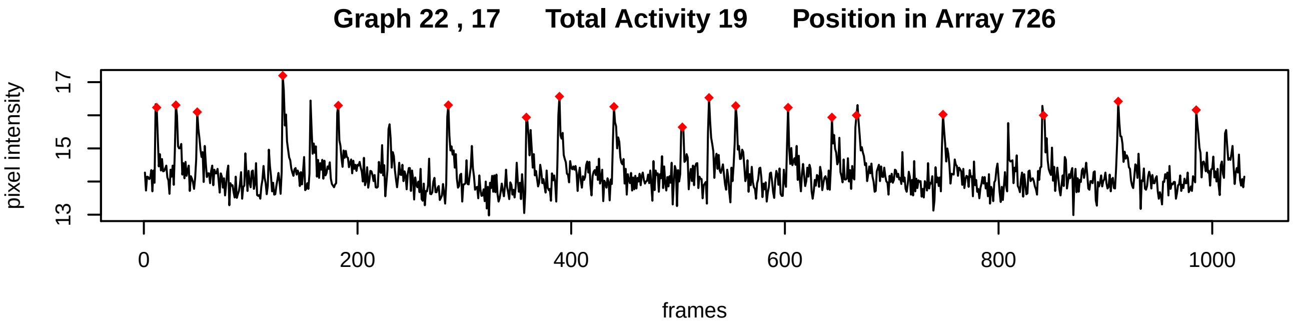
Graph 22 , 17 Total Activity 19 Position in Array 726
pixel intensity
13
15
17
0
200
400
600
800
1000
frames

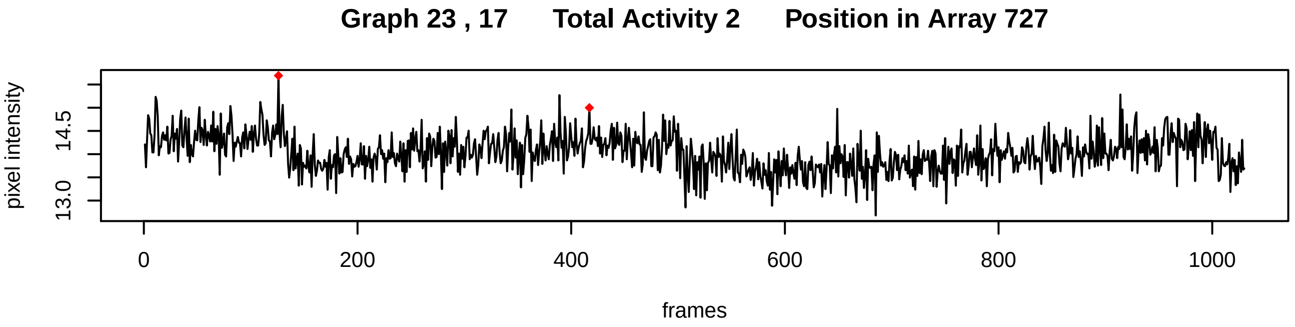
Graph 23 , 17 Total Activity 2 Position in Array 727
pixel intensity
13.0
14.5
0
200
400
600
800
1000
frames

Graph 30 , 17   Total Activity 4   Position in Array 734

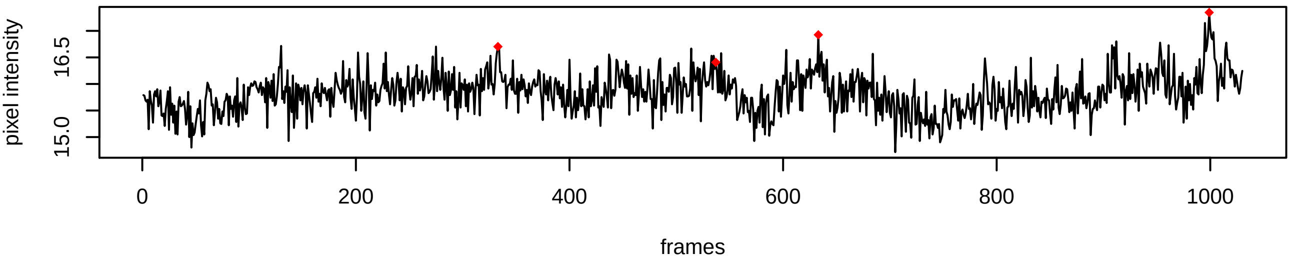

Graph 31 , 17   Total Activity 2   Position in Array 735

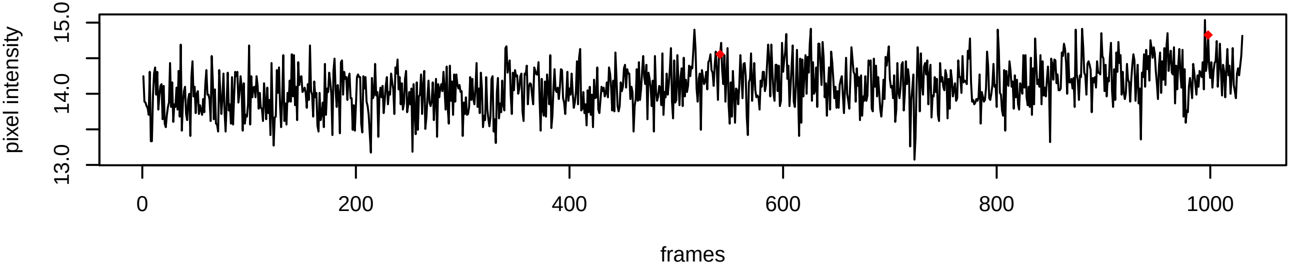

Graph 32 , 17   Total Activity 4   Position in Array 736

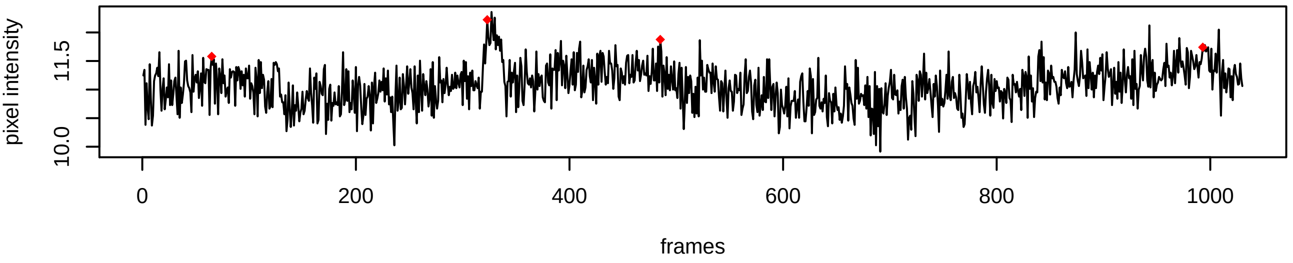

Graph 33 , 17 Total Activity 2 Position in Array 737

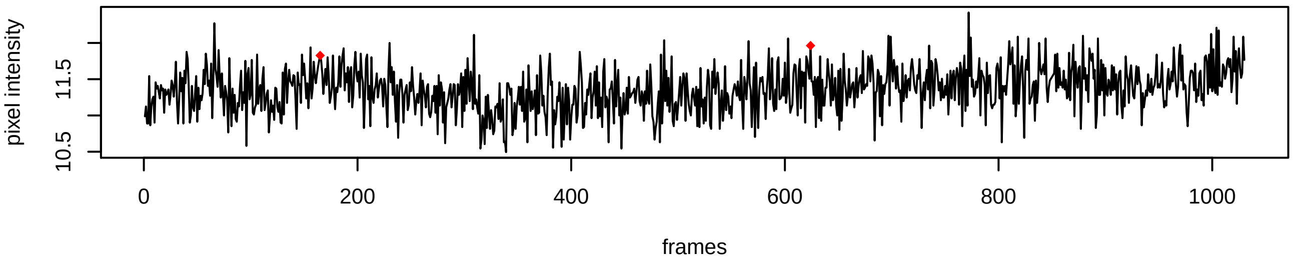

Graph 36 , 17 Total Activity 3 Position in Array 740

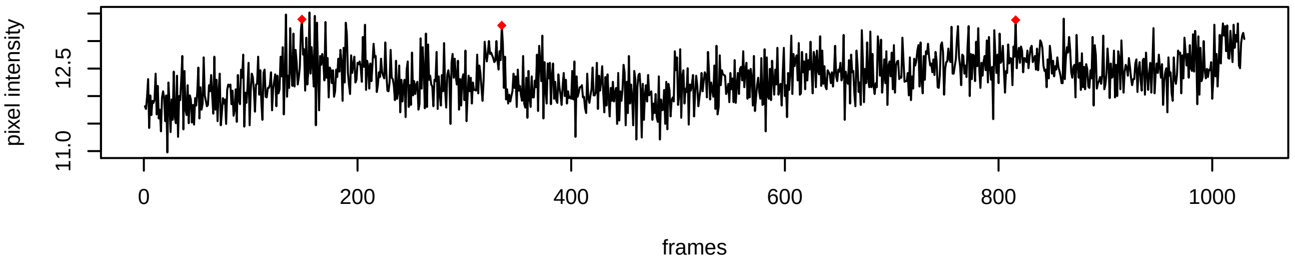

Graph 37 , 17 Total Activity 5 Position in Array 741

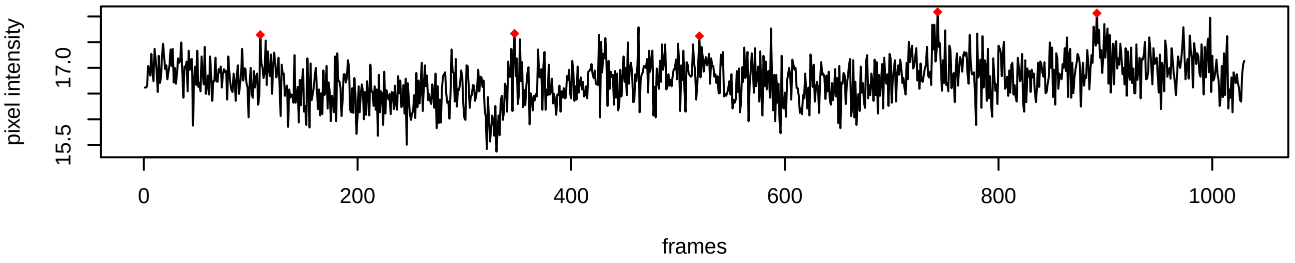

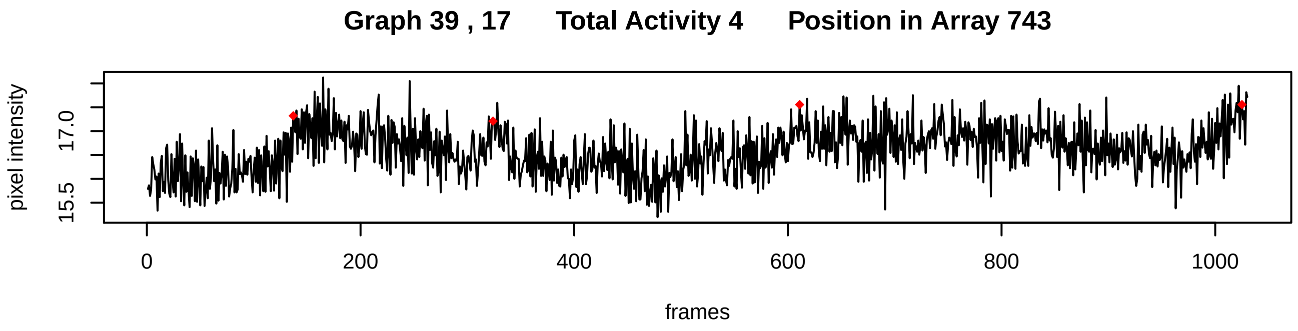
Graph 39 , 17 Total Activity 4 Position in Array 743
pixel intensity
15.5
17.0
0
200
400
600
800
1000
frames

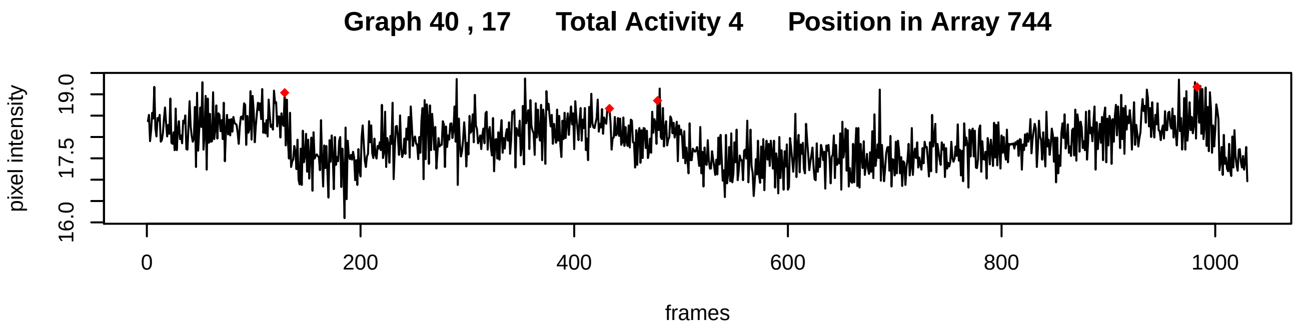
Graph 40 , 17 Total Activity 4 Position in Array 744
pixel intensity
16.0
17.5
19.0
0
200
400
600
800
1000
frames

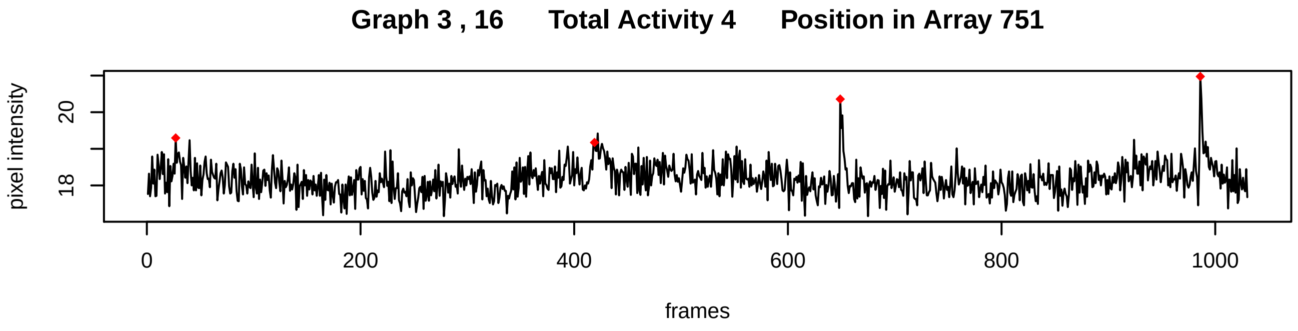
Graph 3 , 16 Total Activity 4 Position in Array 751
pixel intensity
18
20
0
200
400
600
800
1000
frames

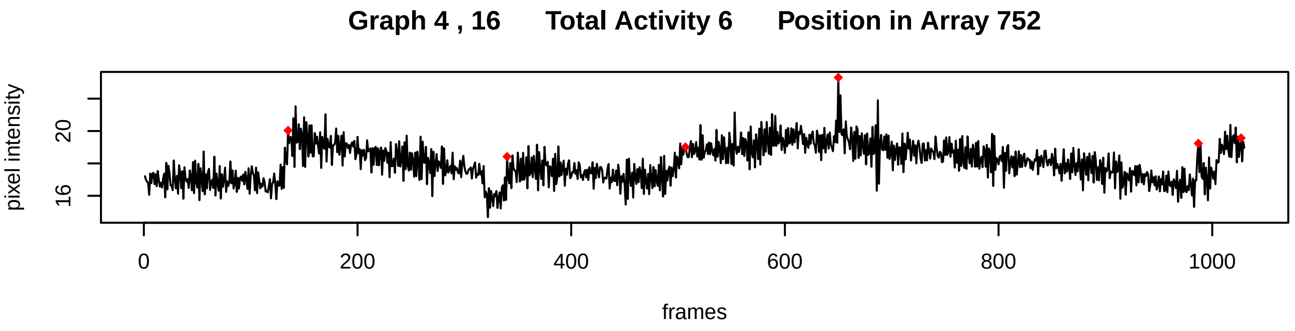
Graph 4 , 16 Total Activity 6 Position in Array 752
pixel intensity
16
20
0
200
400
600
800
1000
frames

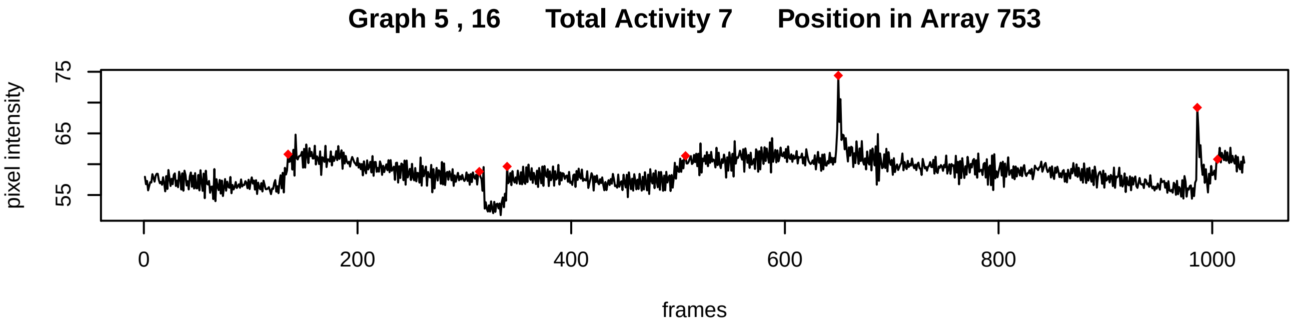
Graph 5 , 16 Total Activity 7 Position in Array 753
pixel intensity
55
65
75
0
200
400
600
800
1000
frames

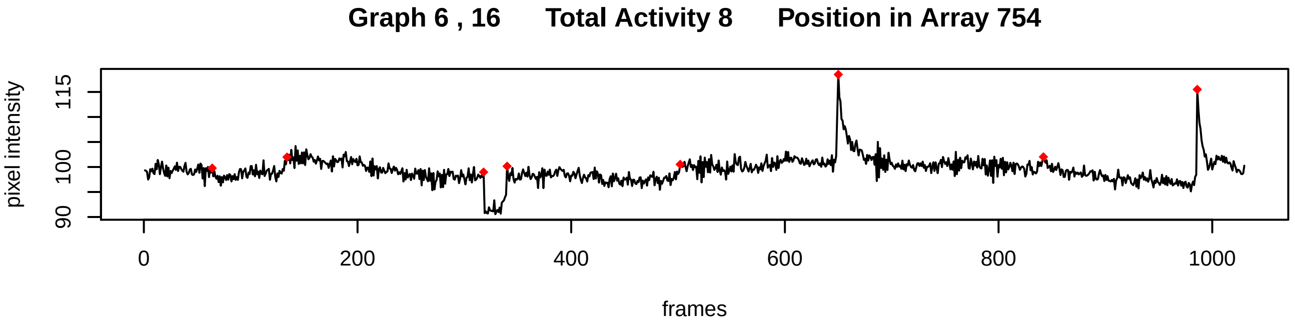
Graph 6 , 16 Total Activity 8 Position in Array 754
pixel intensity
90
100
115
0
200
400
600
800
1000
frames

Graph 7 , 16   Total Activity 6   Position in Array 755

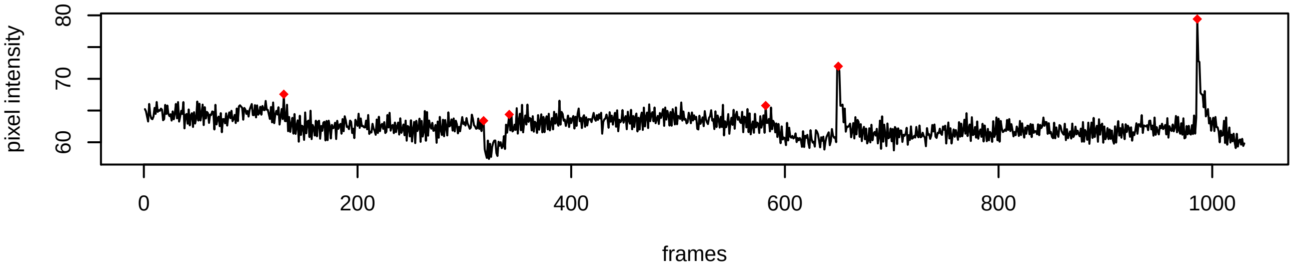

Graph 8 , 16   Total Activity 9   Position in Array 756

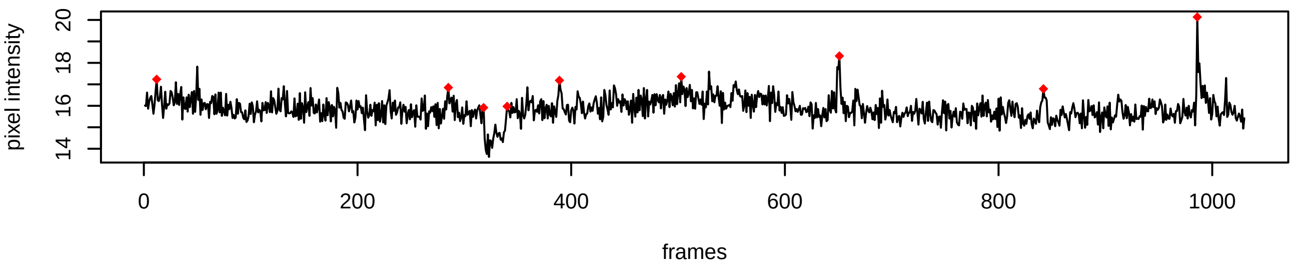

Graph 9 , 16   Total Activity 6   Position in Array 757

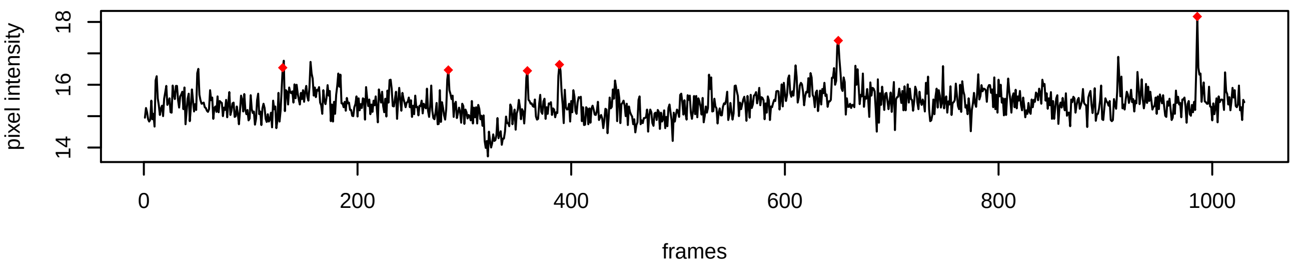

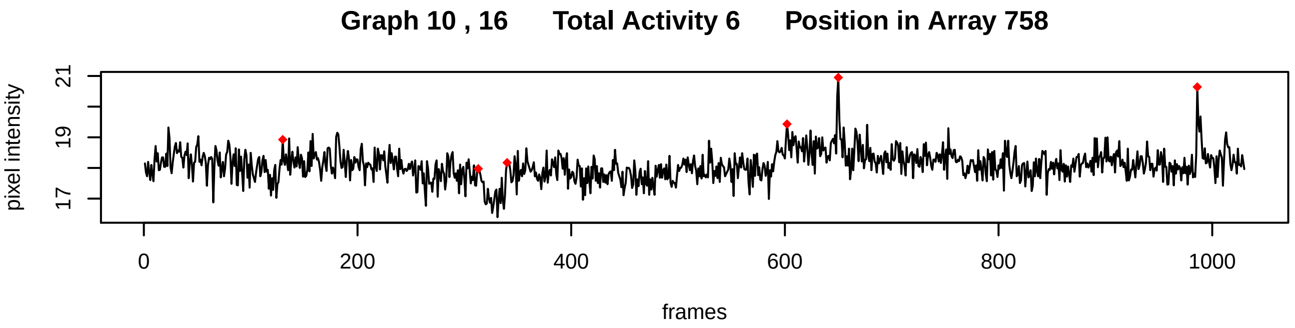
Graph 10 , 16 Total Activity 6 Position in Array 758
pixel intensity
17
19
21
0
200
400
600
800
1000
frames

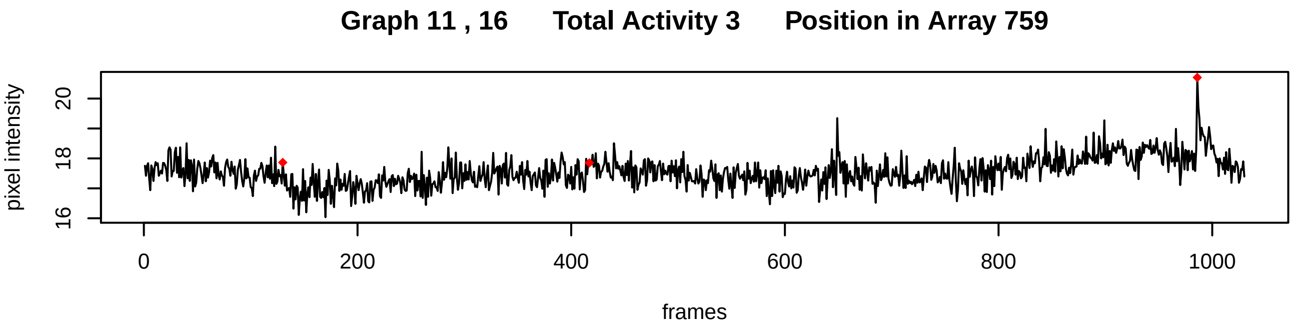
Graph 11 , 16 Total Activity 3 Position in Array 759
pixel intensity
16
18
20
0
200
400
600
800
1000
frames

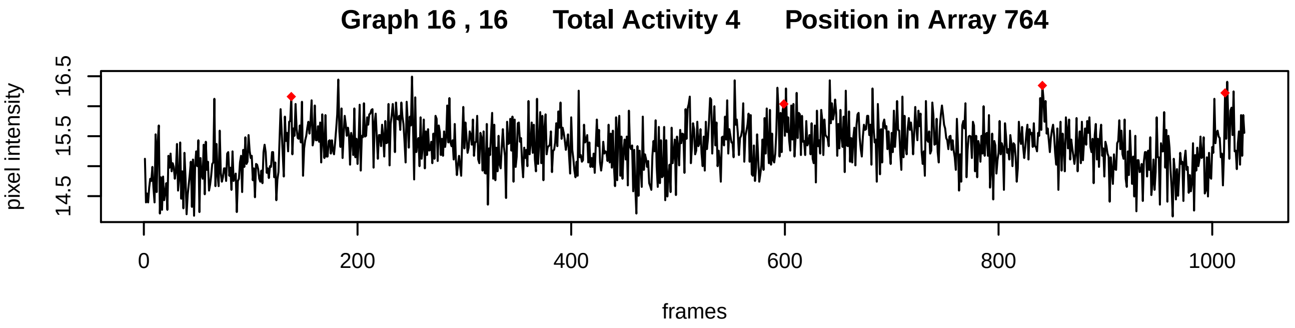
Graph 16 , 16 Total Activity 4 Position in Array 764
pixel intensity
14.5
15.5
16.5
0
200
400
600
800
1000
frames

Graph 17 , 16 Total Activity 5 Position in Array 765

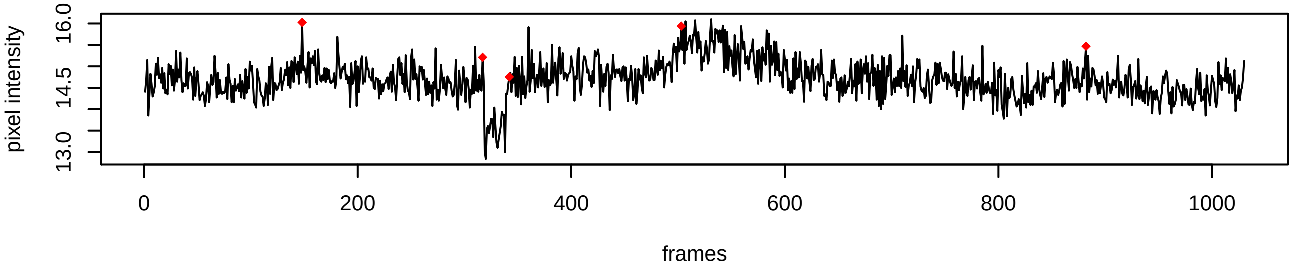

Graph 18 , 16 Total Activity 5 Position in Array 766

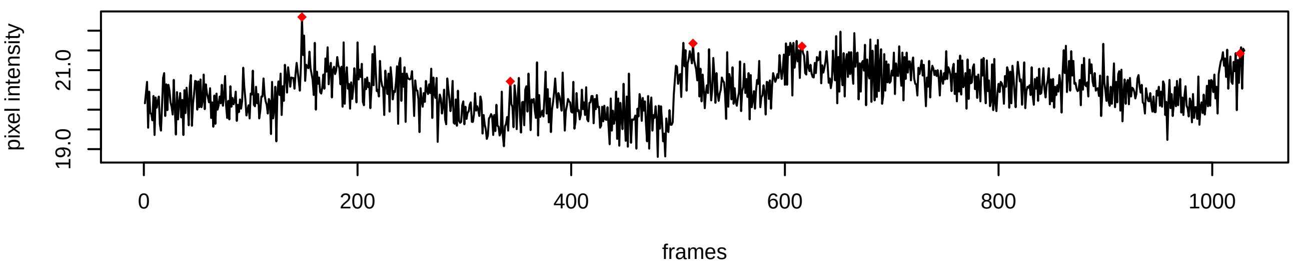

Graph 19 , 16 Total Activity 6 Position in Array 767

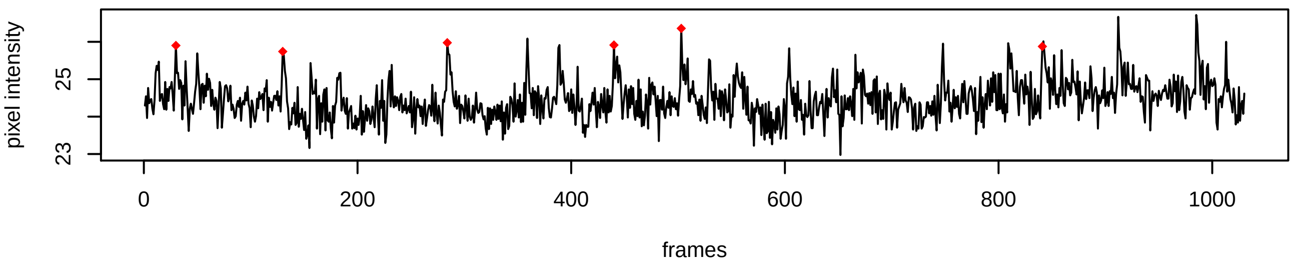

Graph 20 , 16   Total Activity 5   Position in Array 768

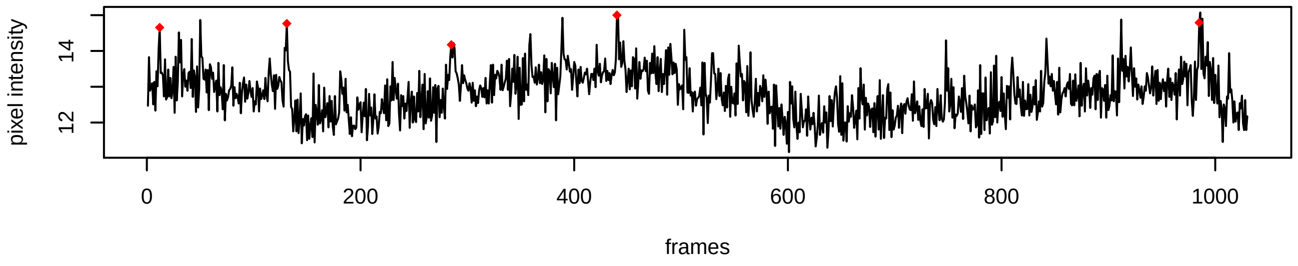

Graph 22 , 16   Total Activity 5   Position in Array 770

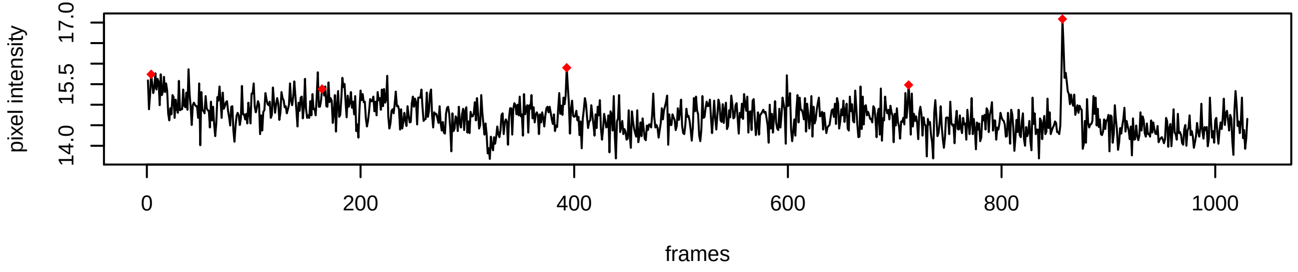

Graph 23 , 16   Total Activity 3   Position in Array 771

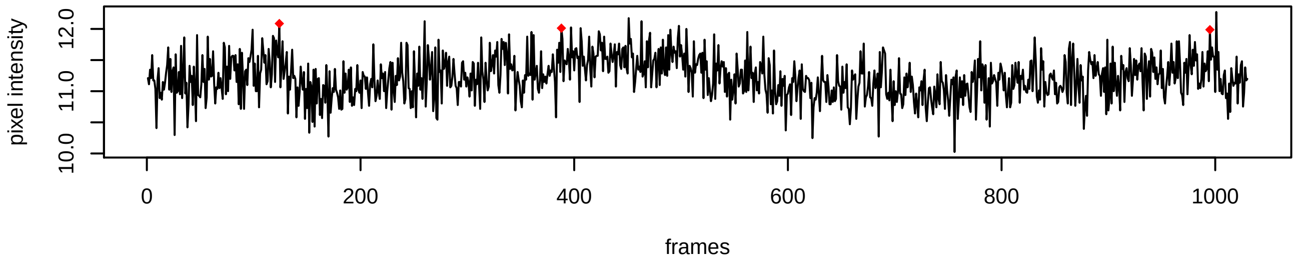

Graph 26 , 16 Total Activity 4 Position in Array 774

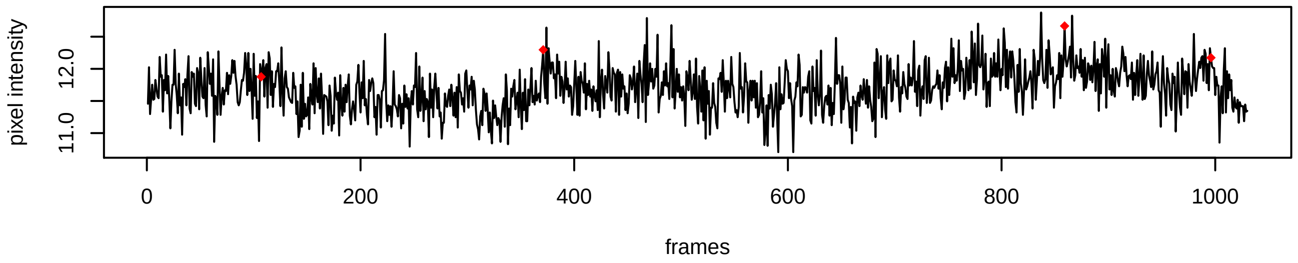

Graph 29 , 16 Total Activity 5 Position in Array 777

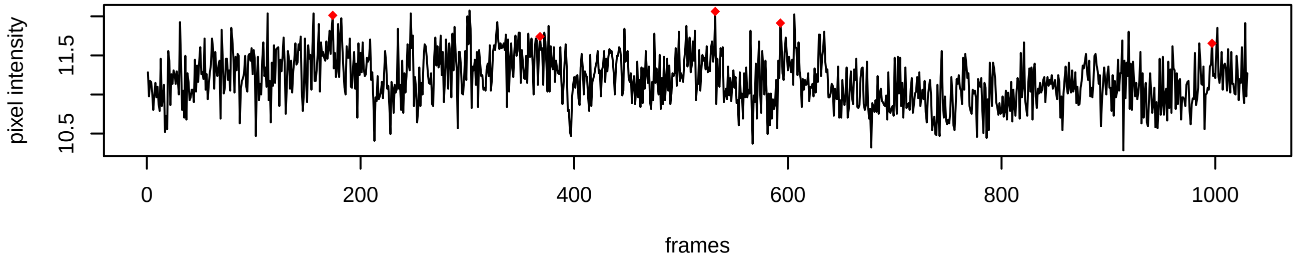

Graph 33 , 16 Total Activity 3 Position in Array 781

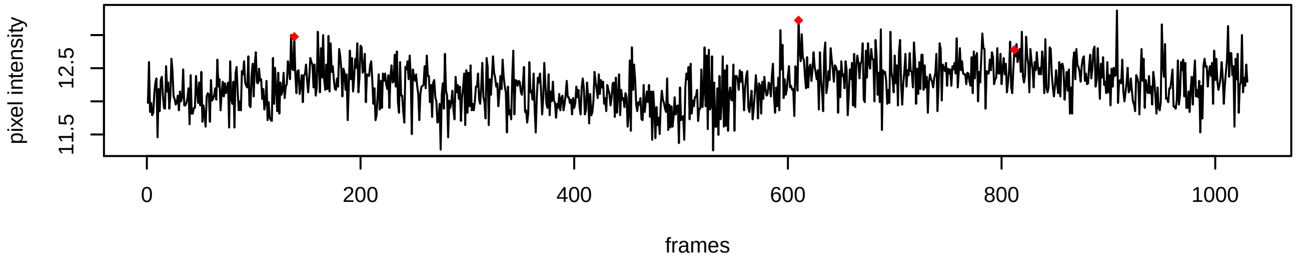

Graph 34 , 16 Total Activity 3 Position in Array 782

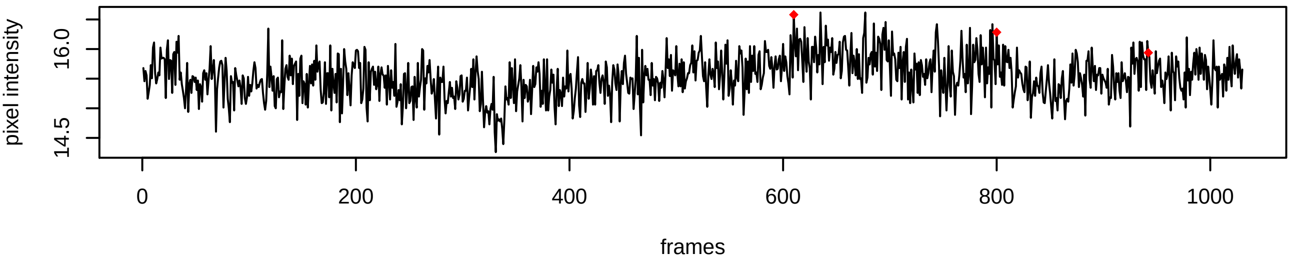

Graph 36 , 16 Total Activity 3 Position in Array 784

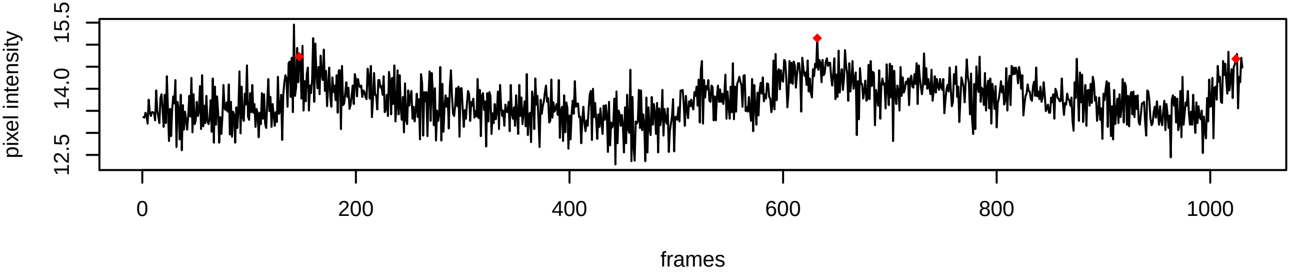

Graph 37 , 16 Total Activity 4 Position in Array 785

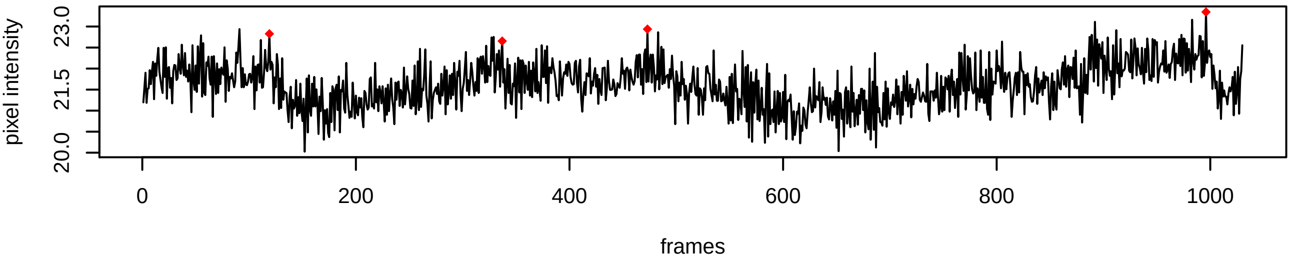

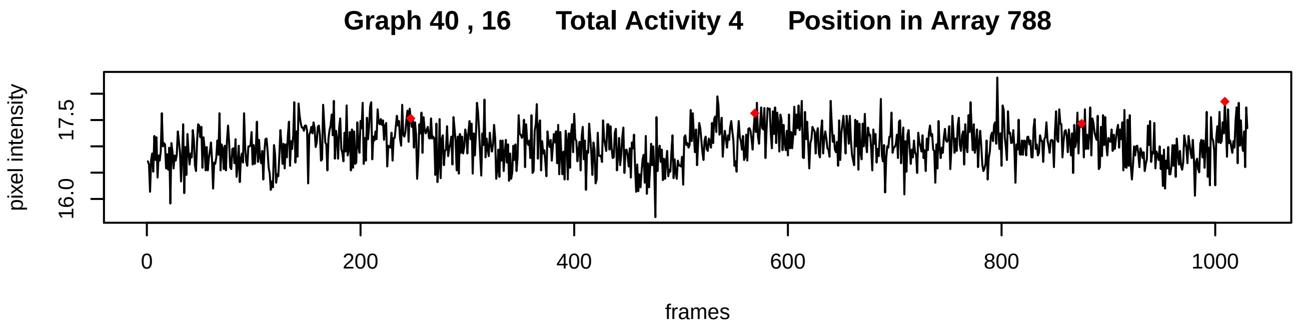
Graph 40 , 16 Total Activity 4 Position in Array 788
pixel intensity
16.0
17.5
0
200
400
600
800
1000
frames

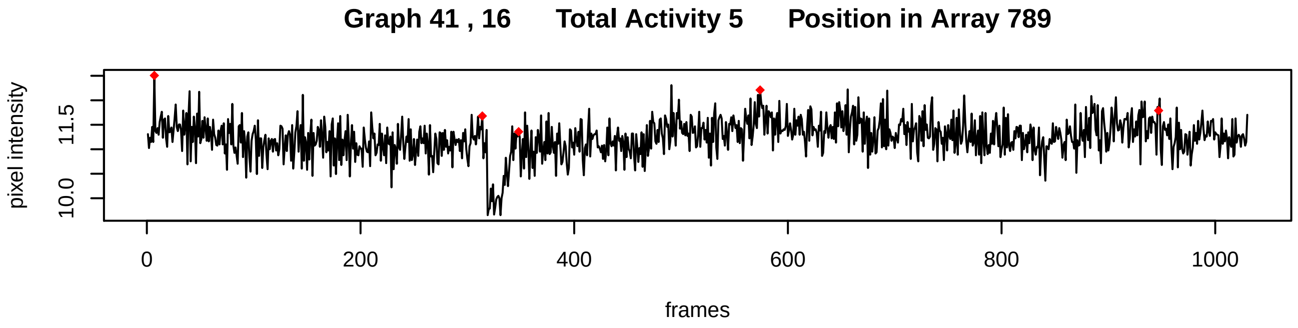
Graph 41 , 16 Total Activity 5 Position in Array 789
pixel intensity
10.0
11.5
0
200
400
600
800
1000
frames

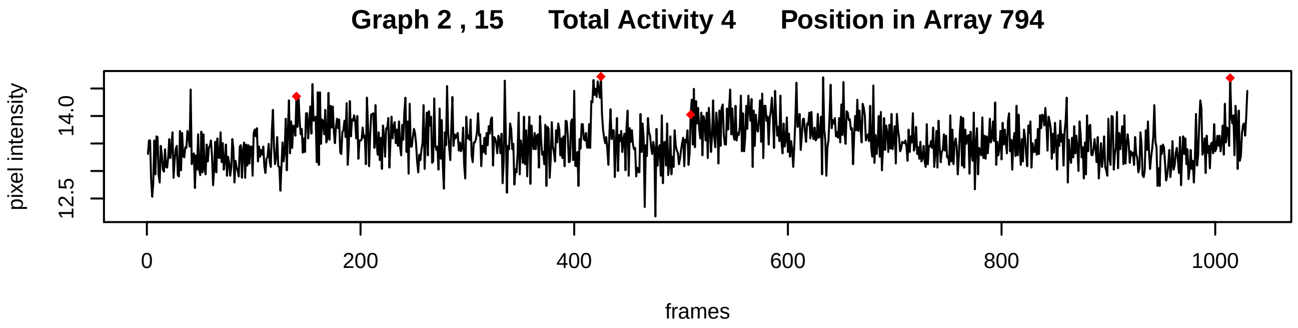
Graph 2 , 15 Total Activity 4 Position in Array 794
pixel intensity
12.5
14.0
0
200
400
600
800
1000
frames

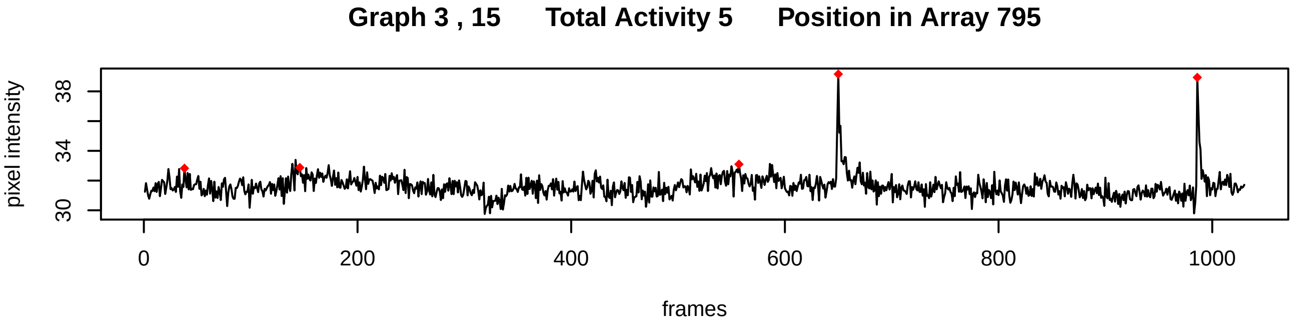
Graph 3 , 15 Total Activity 5 Position in Array 795
pixel intensity
30
34
38
0
200
400
600
800
1000
frames

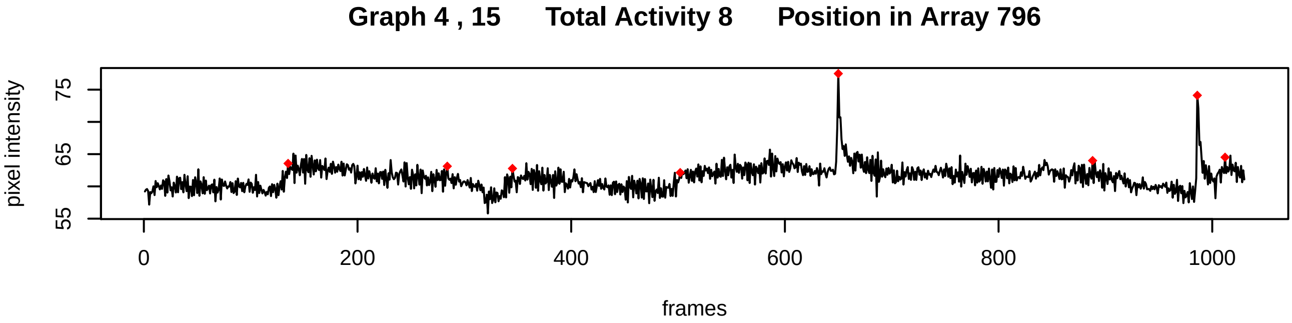
Graph 4 , 15 Total Activity 8 Position in Array 796
pixel intensity
55
65
75
0
200
400
600
800
1000
frames

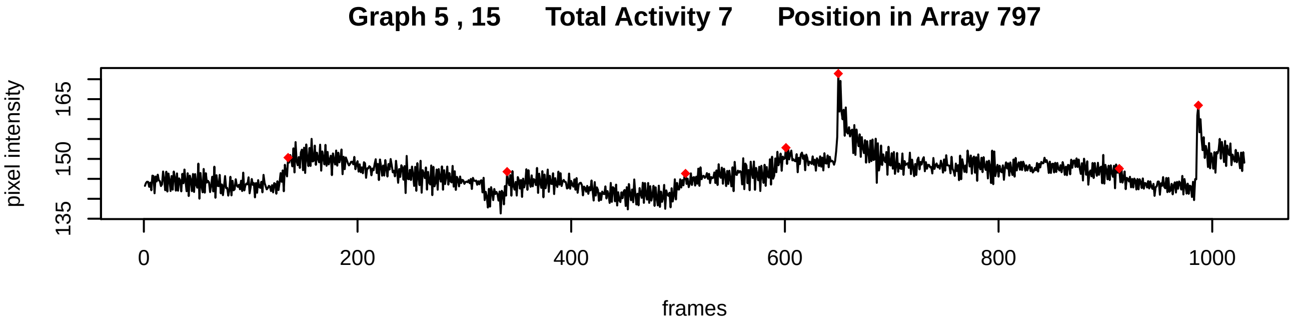
Graph 5 , 15 Total Activity 7 Position in Array 797
pixel intensity
135
150
165
0
200
400
600
800
1000
frames

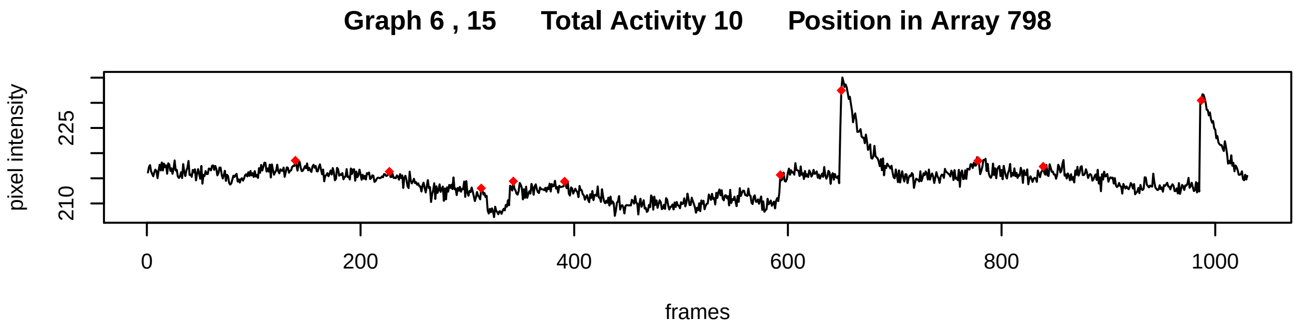
Graph 6 , 15 Total Activity 10 Position in Array 798
pixel intensity
210
225
0
200
400
600
800
1000
frames

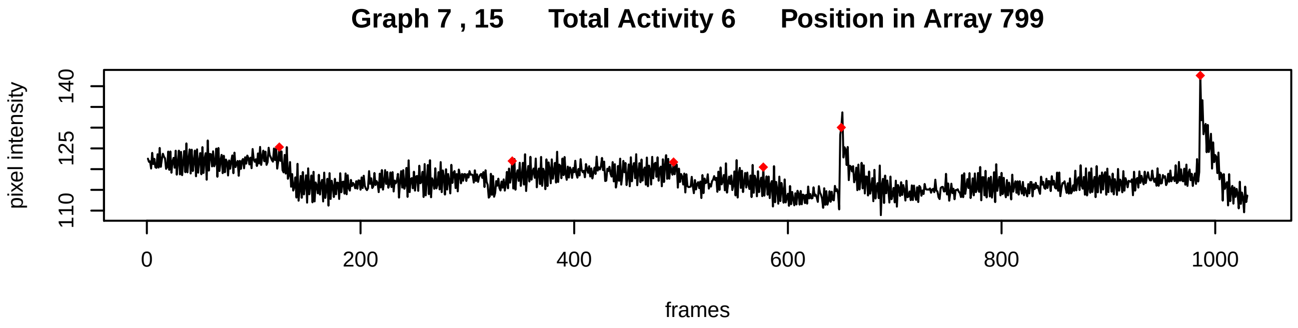
Graph 7 , 15 Total Activity 6 Position in Array 799
pixel intensity
110
125
140
0
200
400
600
800
1000
frames

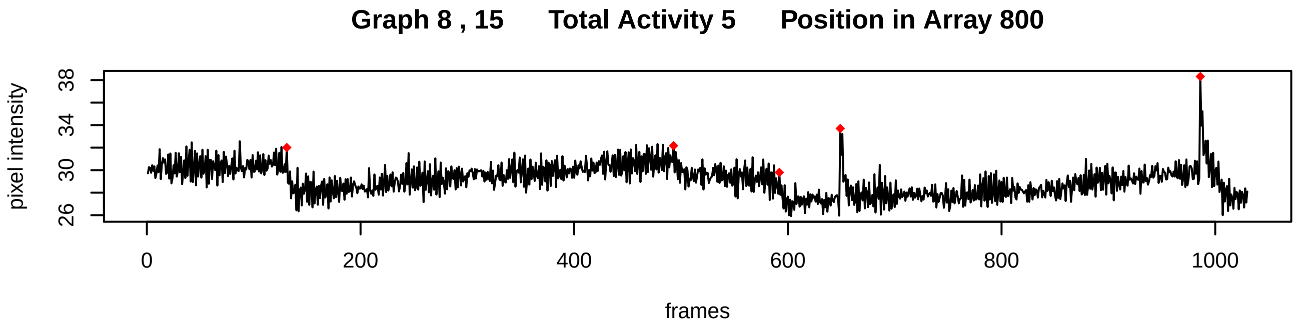
Graph 8 , 15 Total Activity 5 Position in Array 800
pixel intensity
26
30
34
38
0
200
400
600
800
1000
frames

Graph 10 , 15   Total Activity 4   Position in Array 802

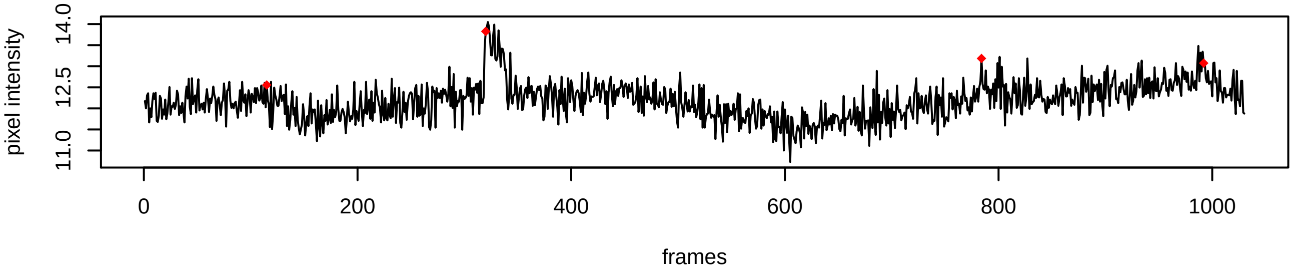

Graph 13 , 15   Total Activity 6   Position in Array 805

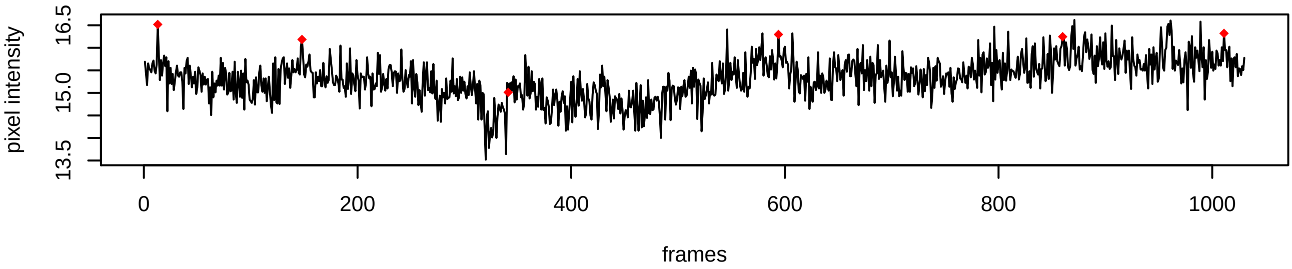

Graph 15 , 15   Total Activity 4   Position in Array 807

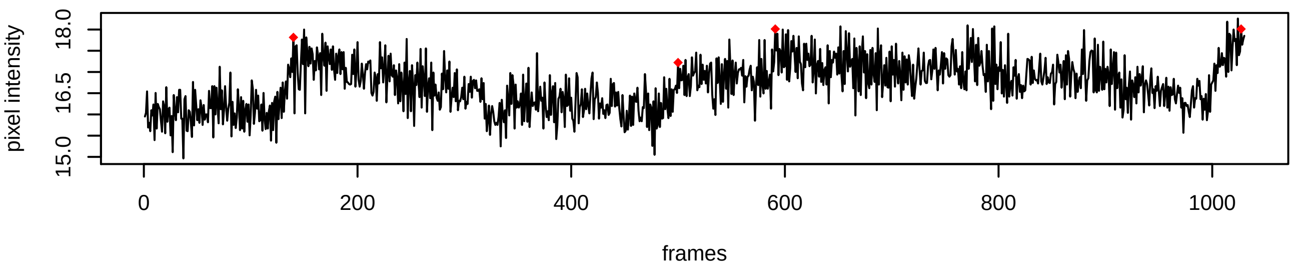

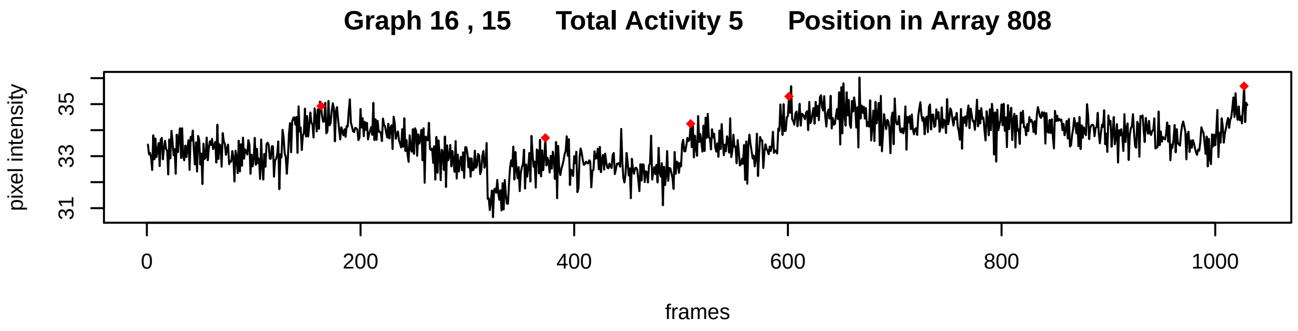
Graph 16 , 15 Total Activity 5 Position in Array 808
pixel intensity
31
33
35
0
200
400
600
800
1000
frames

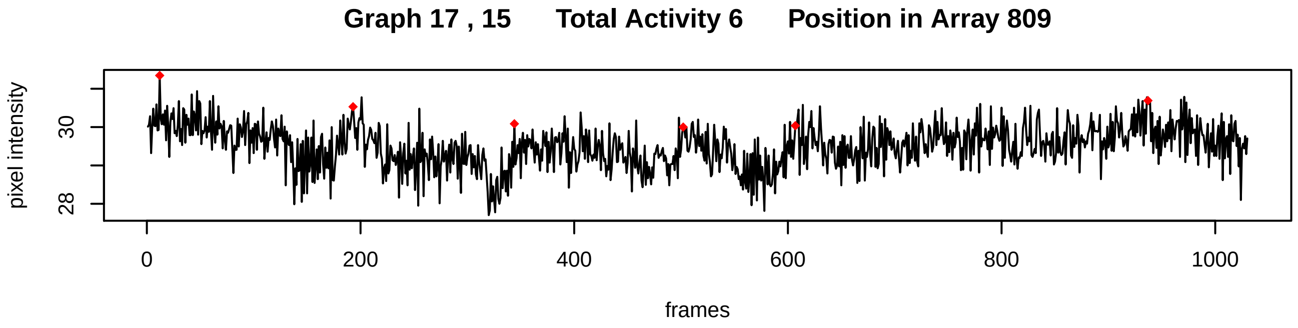
Graph 17 , 15 Total Activity 6 Position in Array 809
pixel intensity
28
30
0
200
400
600
800
1000
frames

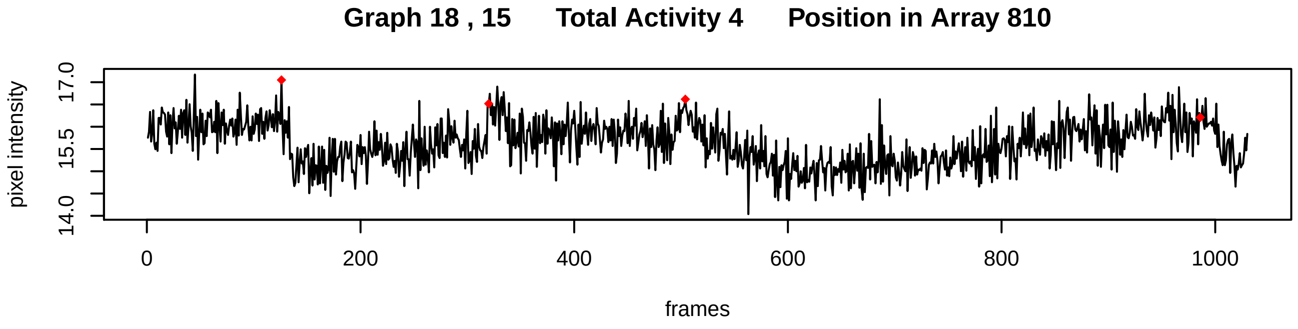
Graph 18 , 15 Total Activity 4 Position in Array 810
pixel intensity
14.0
15.5
17.0
0
200
400
600
800
1000
frames

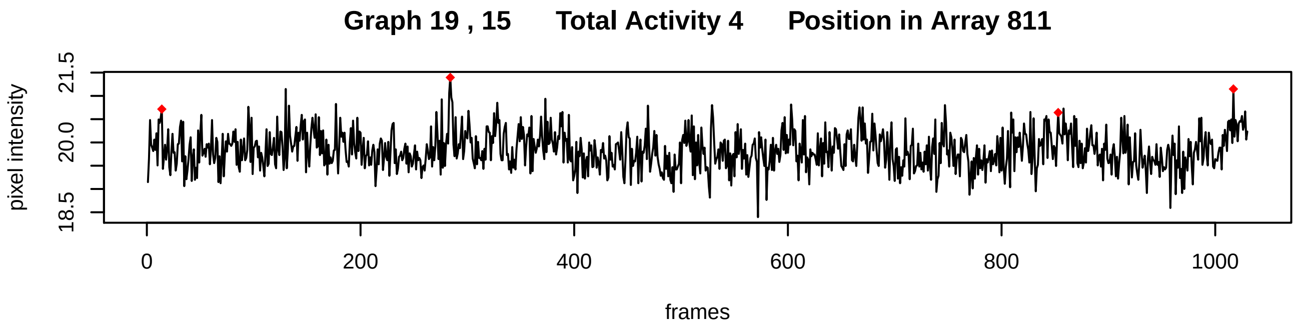
Graph 19 , 15 Total Activity 4 Position in Array 811
pixel intensity
18.5
20.0
21.5
0
200
400
600
800
1000
frames

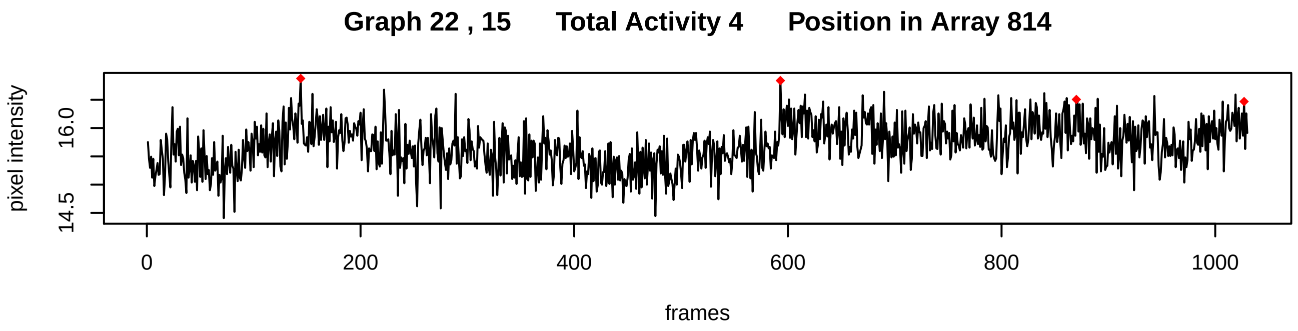
Graph 22 , 15 Total Activity 4 Position in Array 814
pixel intensity
14.5
16.0
0
200
400
600
800
1000
frames

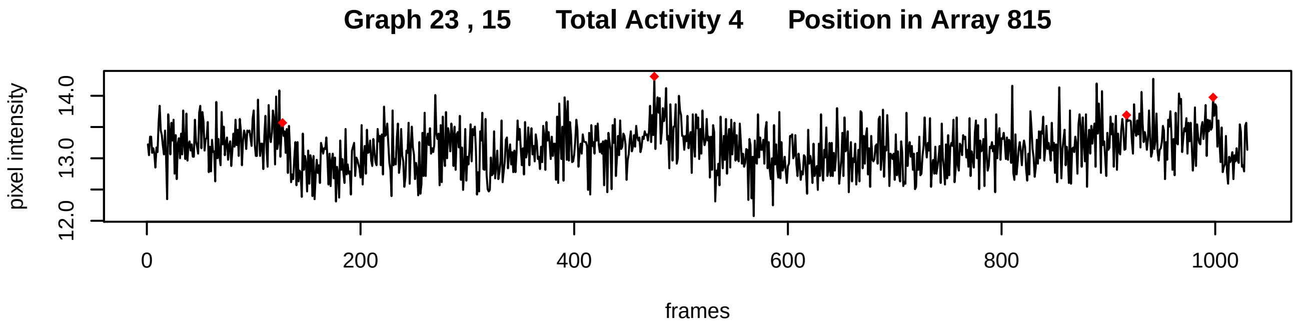
Graph 23 , 15 Total Activity 4 Position in Array 815
pixel intensity
12.0
13.0
14.0
0
200
400
600
800
1000
frames

Graph 24 , 15   Total Activity 3   Position in Array 816

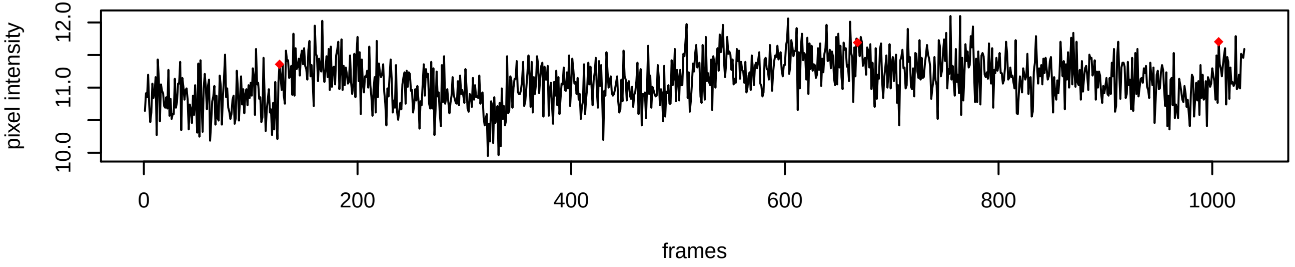

Graph 25 , 15   Total Activity 4   Position in Array 817

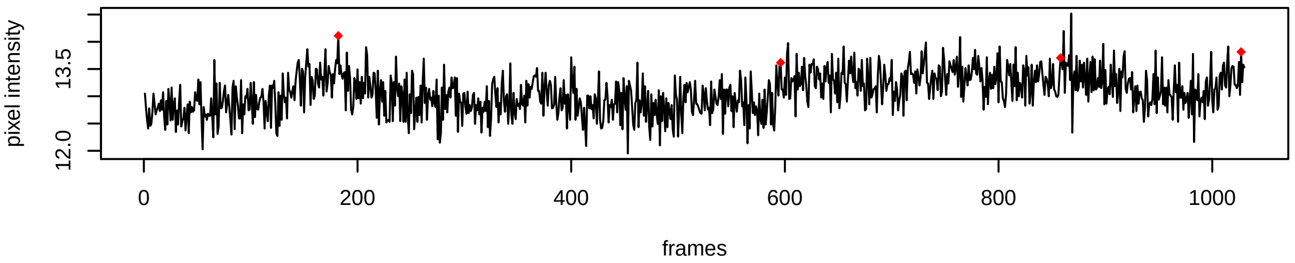

Graph 29 , 15   Total Activity 4   Position in Array 821

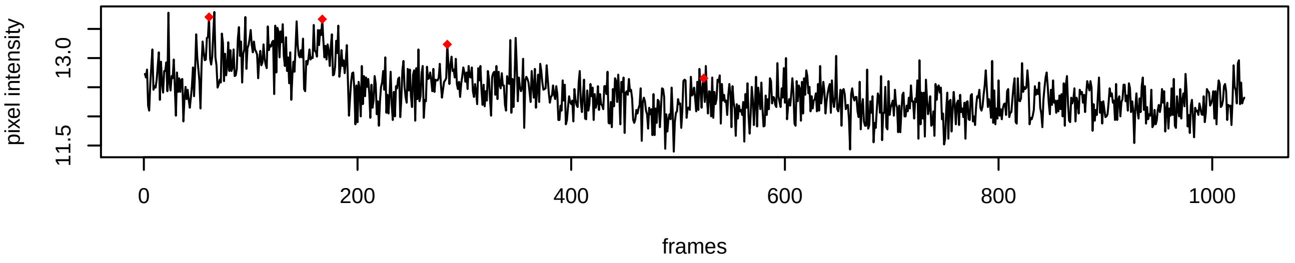

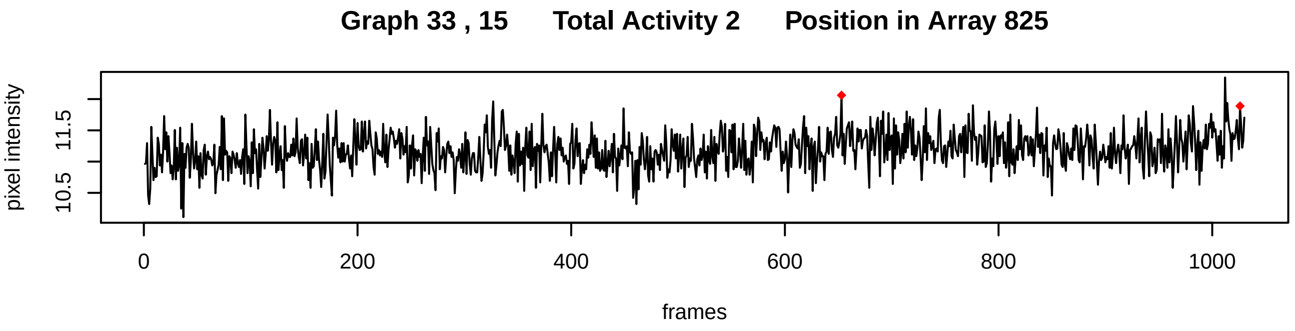
Graph 33 , 15 Total Activity 2 Position in Array 825
pixel intensity
10.5
11.5
0
200
400
600
800
1000
frames

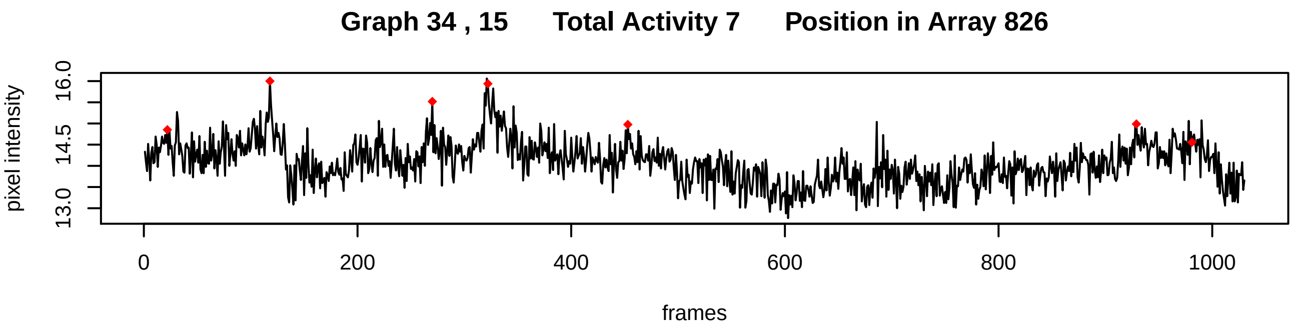
Graph 34 , 15 Total Activity 7 Position in Array 826
pixel intensity
13.0
14.5
16.0
0
200
400
600
800
1000
frames

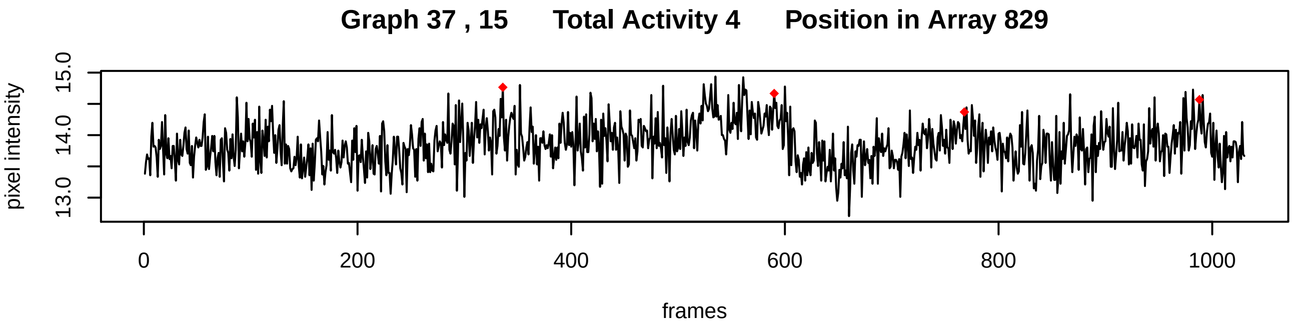
Graph 37 , 15 Total Activity 4 Position in Array 829
pixel intensity
13.0
14.0
15.0
0
200
400
600
800
1000
frames

Graph 40 , 15 Total Activity 4 Position in Array 832

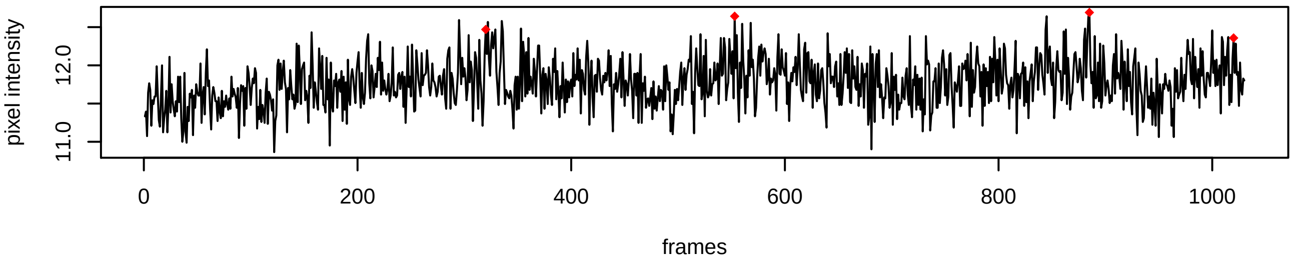

Graph 41 , 15 Total Activity 4 Position in Array 833

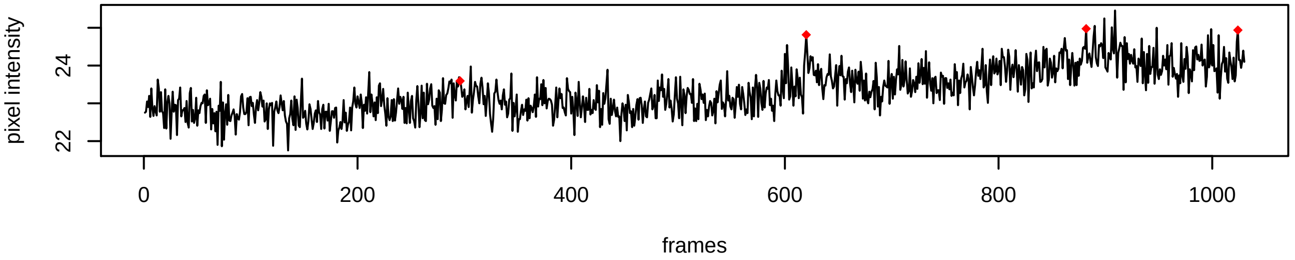

Graph 3 , 14 Total Activity 4 Position in Array 839

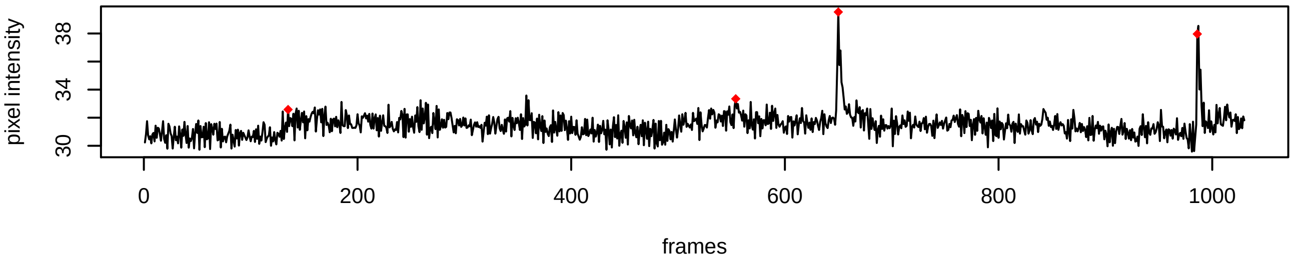

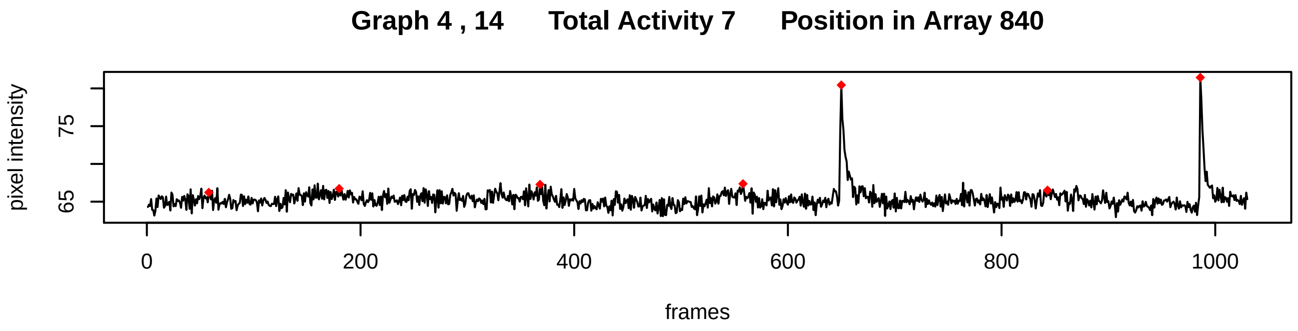
Graph 4 , 14 Total Activity 7 Position in Array 840
pixel intensity
65
75
0
200
400
600
800
1000
frames

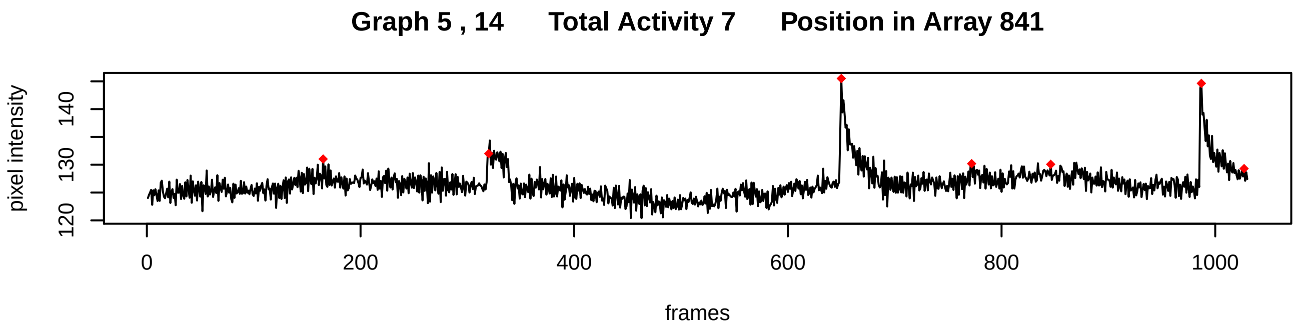
Graph 5 , 14 Total Activity 7 Position in Array 841
pixel intensity
120
130
140
0
200
400
600
800
1000
frames

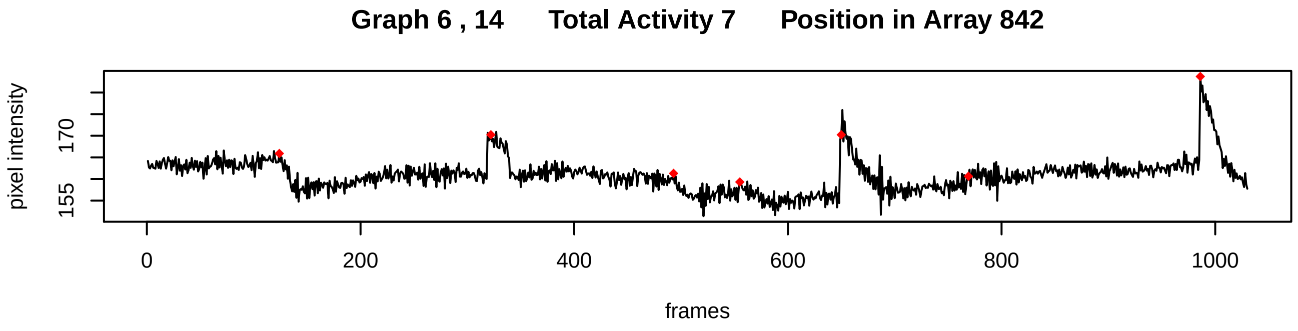
Graph 6 , 14 Total Activity 7 Position in Array 842
pixel intensity
155
170
0
200
400
600
800
1000
frames

Graph 7 , 14 Total Activity 6 Position in Array 843

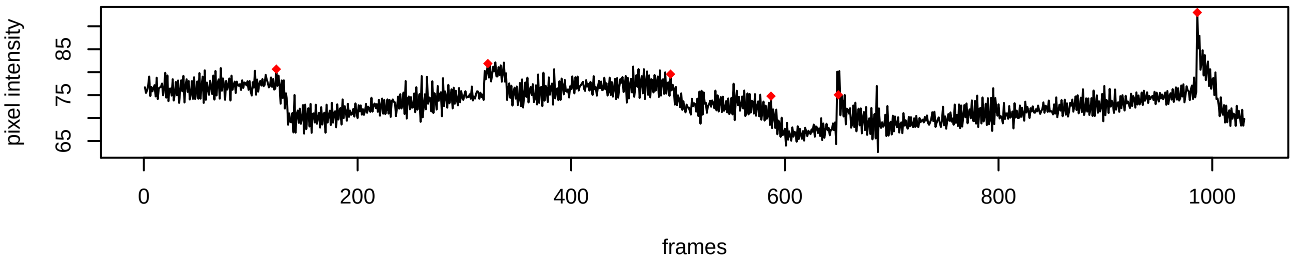

Graph 8 , 14 Total Activity 4 Position in Array 844

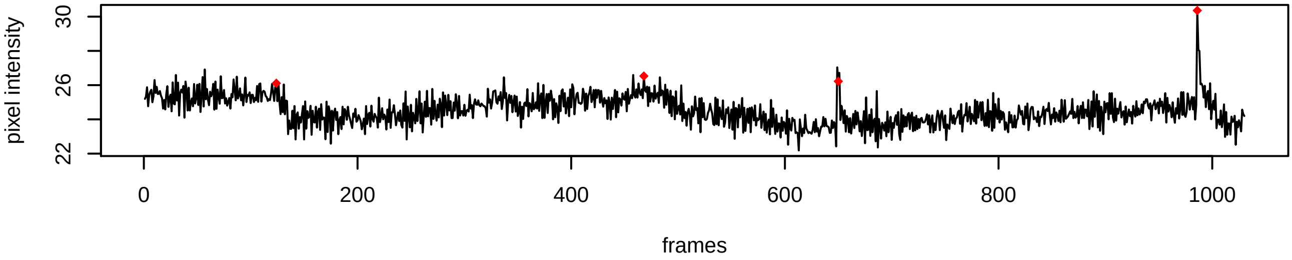

Graph 11 , 14 Total Activity 3 Position in Array 847

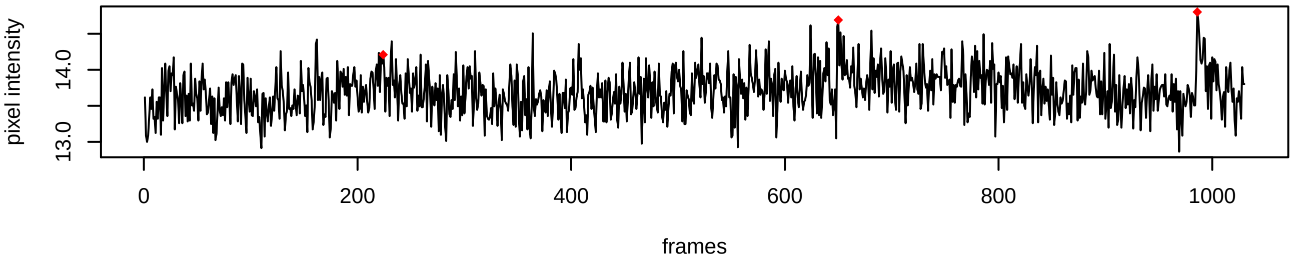

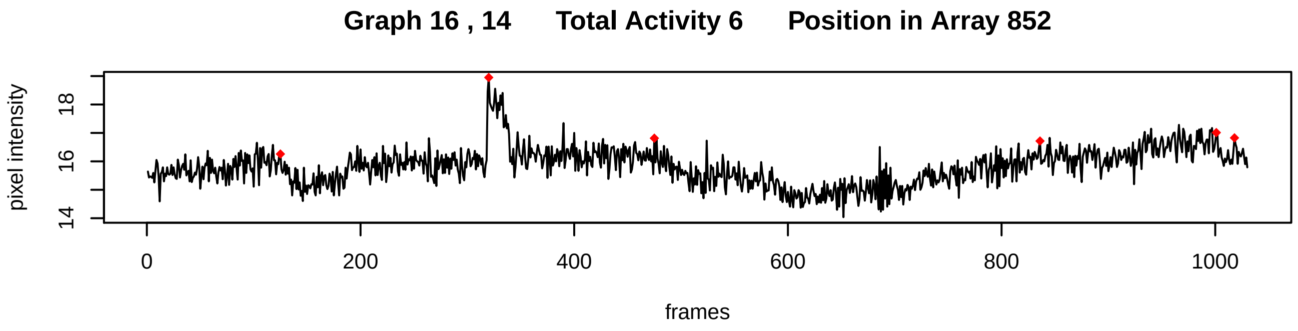
Graph 16 , 14 Total Activity 6 Position in Array 852
pixel intensity
14
16
18
0
200
400
600
800
1000
frames

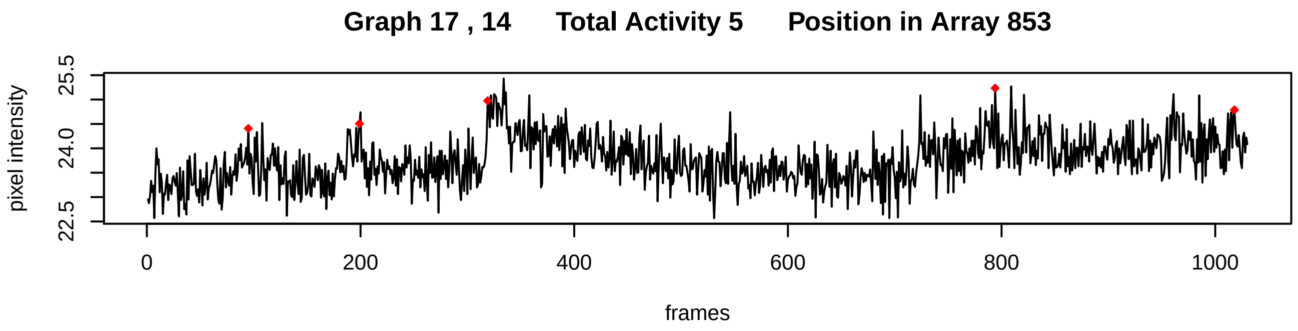
Graph 17 , 14 Total Activity 5 Position in Array 853
pixel intensity
22.5
24.0
25.5
0
200
400
600
800
1000
frames

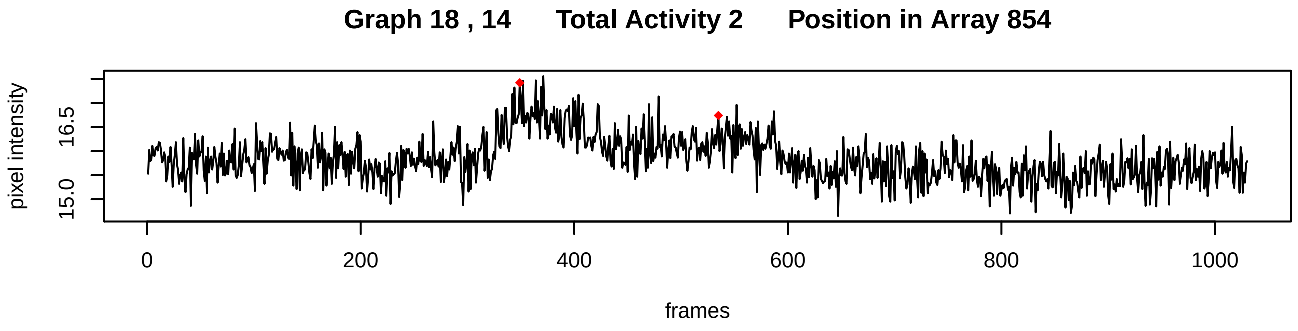
Graph 18 , 14 Total Activity 2 Position in Array 854
pixel intensity
15.0
16.5
0
200
400
600
800
1000
frames

Graph 19 , 14 Total Activity 3 Position in Array 855

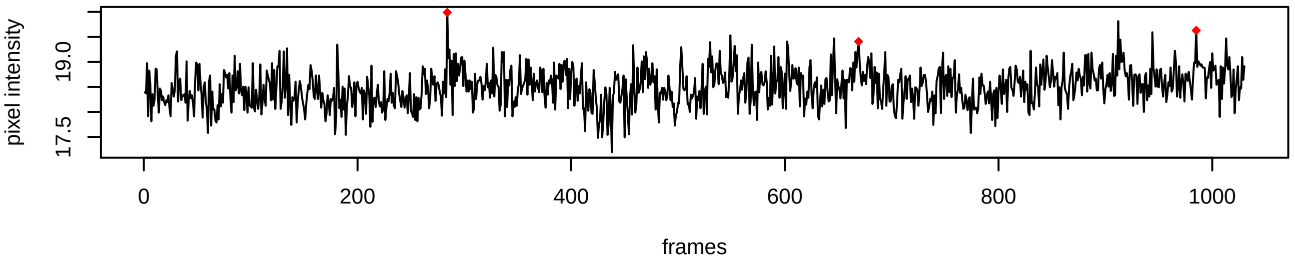

Graph 22 , 14 Total Activity 2 Position in Array 858

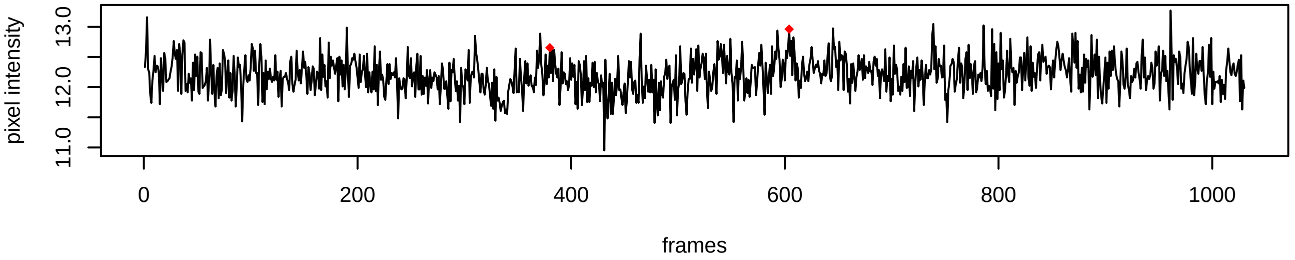

Graph 25 , 14 Total Activity 4 Position in Array 861

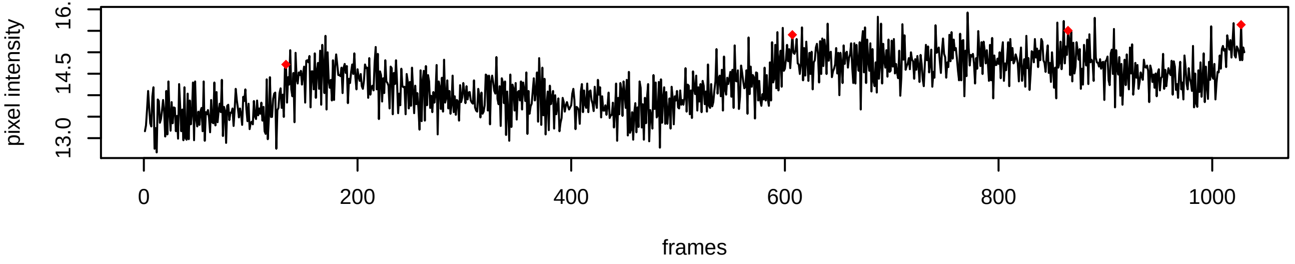

Graph 26 , 14   Total Activity 4   Position in Array 862

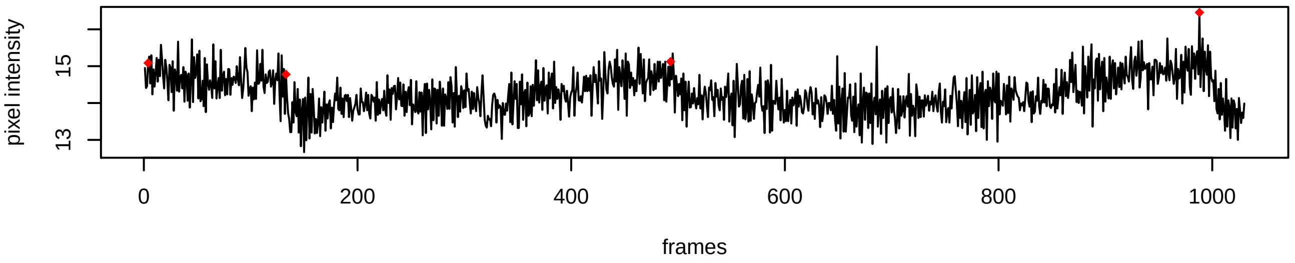

Graph 27 , 14   Total Activity 4   Position in Array 863

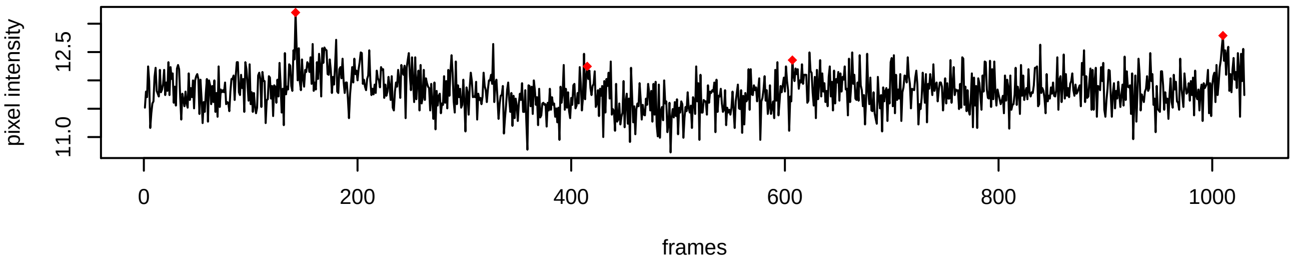

Graph 29 , 14   Total Activity 2   Position in Array 865

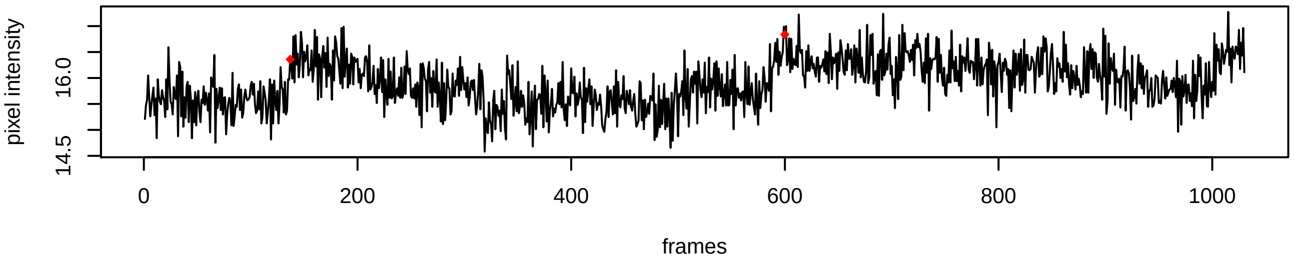

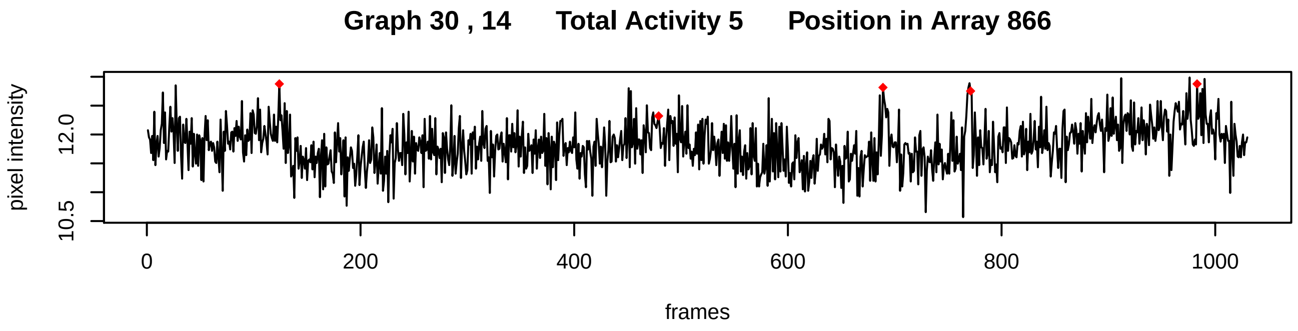
Graph 30 , 14 Total Activity 5 Position in Array 866
pixel intensity
10.5
12.0
0
200
400
600
800
1000
frames

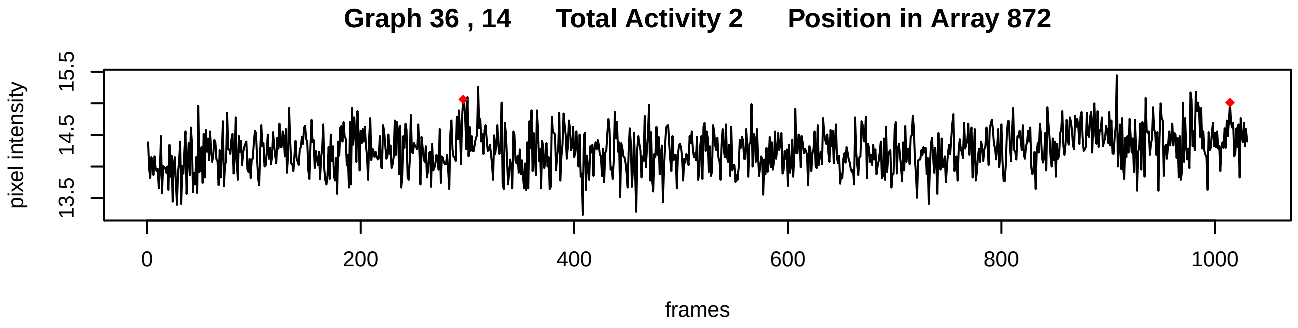
Graph 36 , 14 Total Activity 2 Position in Array 872
pixel intensity
13.5
14.5
15.5
0
200
400
600
800
1000
frames

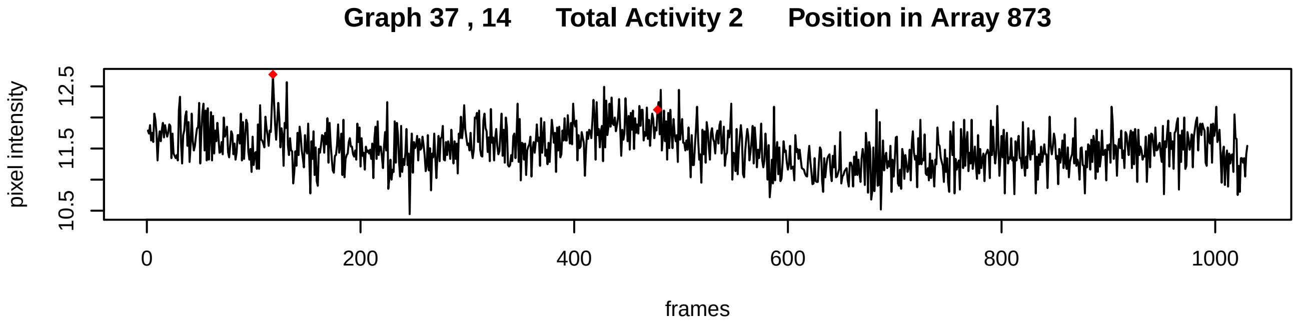
Graph 37 , 14 Total Activity 2 Position in Array 873
pixel intensity
10.5
11.5
12.5
0
200
400
600
800
1000
frames

Graph 39 , 14   Total Activity 3   Position in Array 875

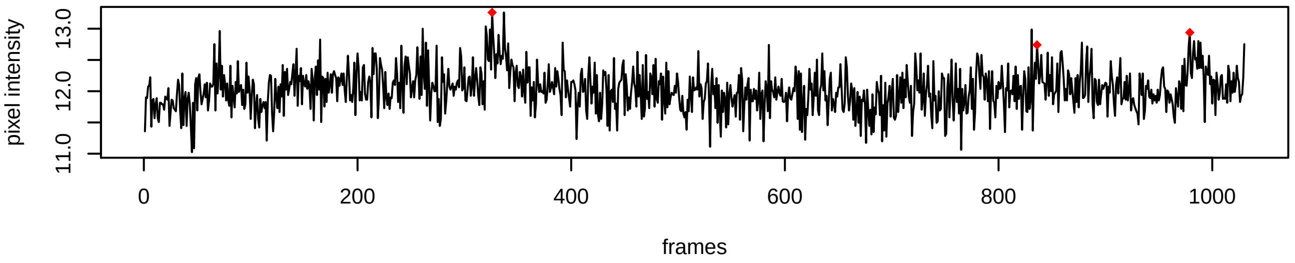

Graph 40 , 14   Total Activity 3   Position in Array 876

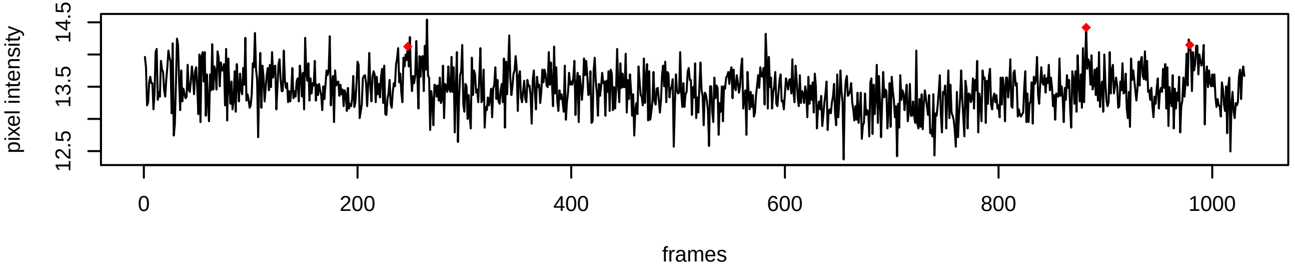

Graph 41 , 14   Total Activity 4   Position in Array 877

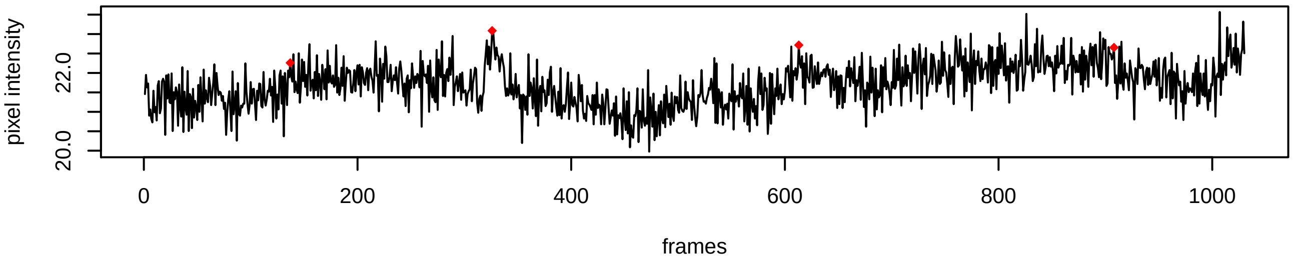

Graph 42 , 14 Total Activity 4 Position in Array 878

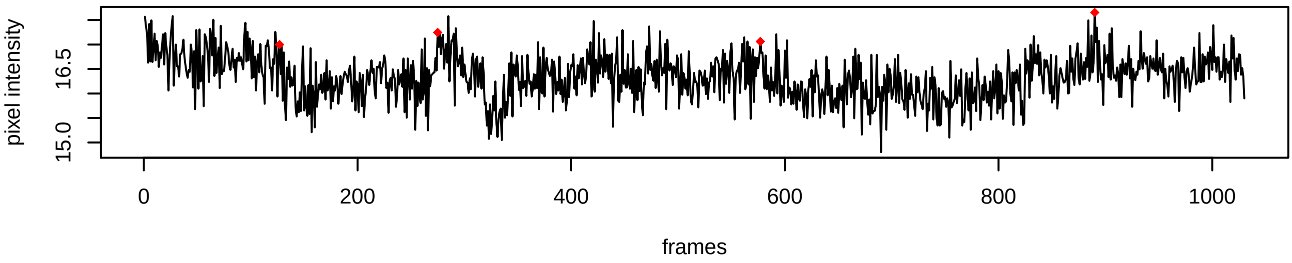

Graph 1 , 13 Total Activity 23 Position in Array 881

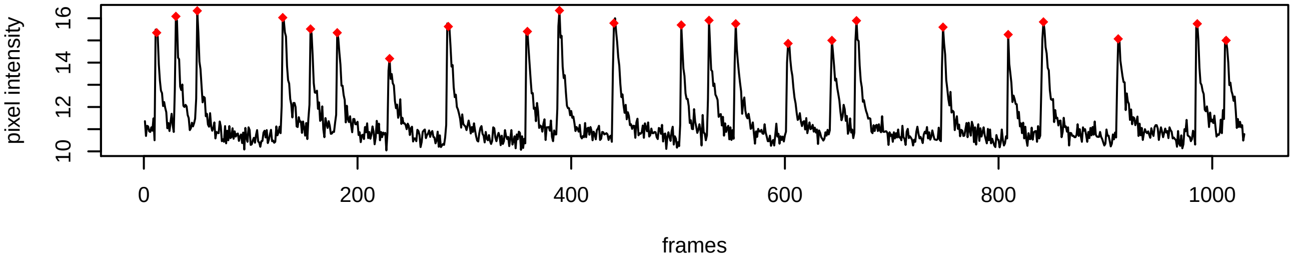

Graph 3 , 13 Total Activity 4 Position in Array 883

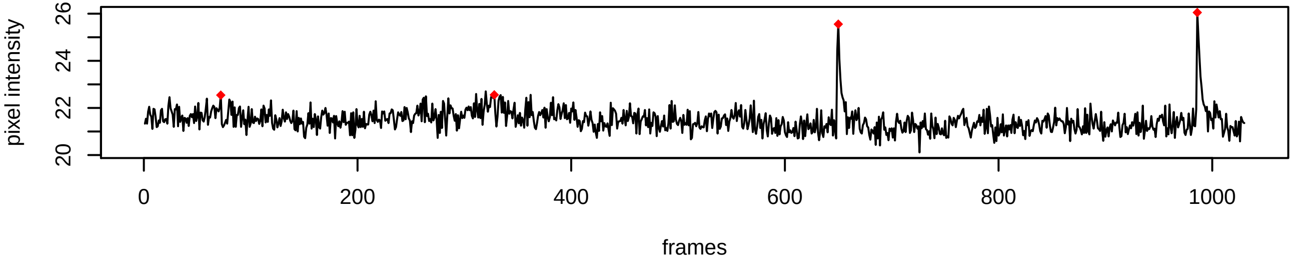

Graph 4 , 13   Total Activity 5   Position in Array 884

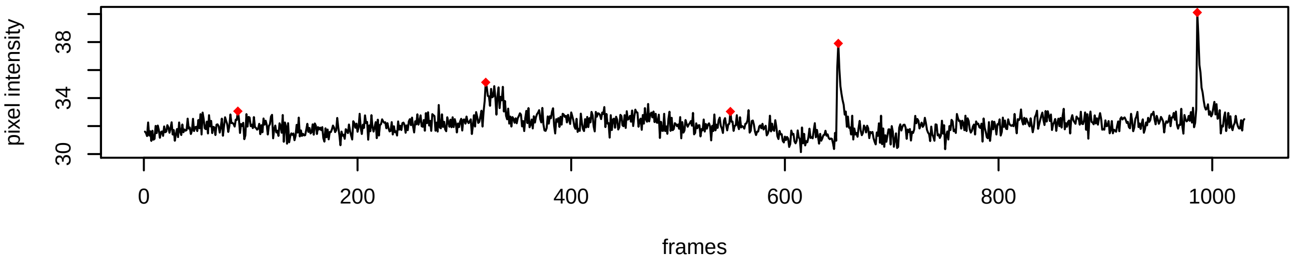

Graph 5 , 13   Total Activity 6   Position in Array 885

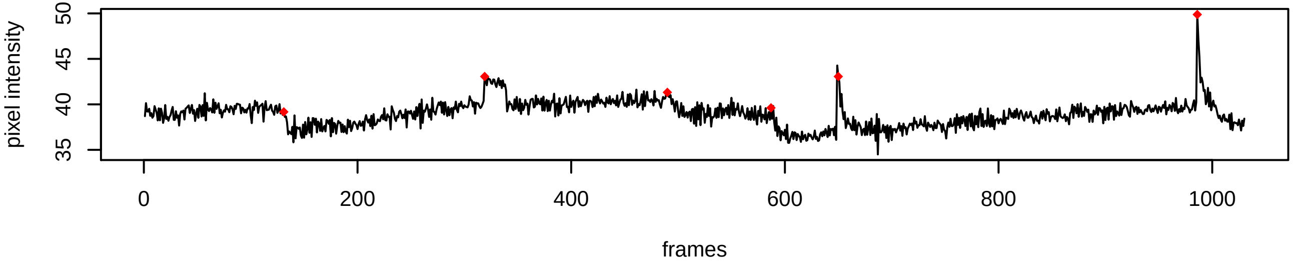

Graph 6 , 13   Total Activity 6   Position in Array 886

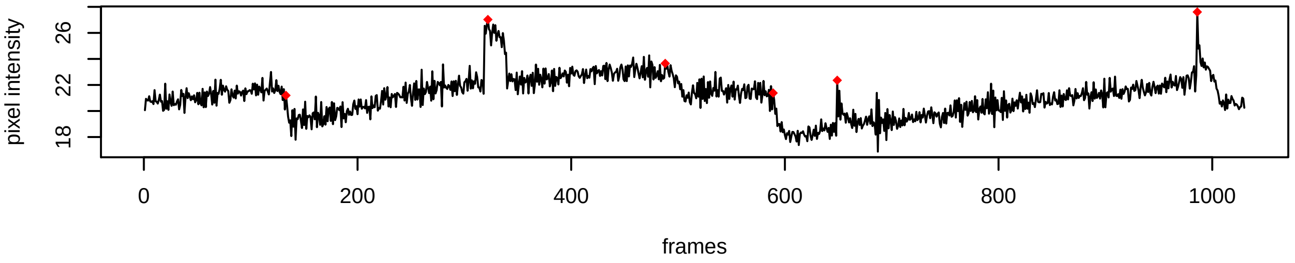

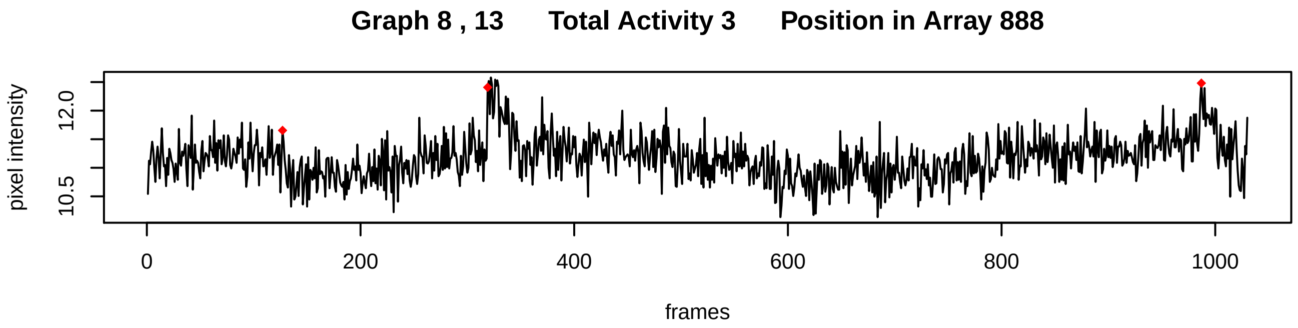
Graph 8 , 13 Total Activity 3 Position in Array 888
pixel intensity
10.5
12.0
0
200
400
600
800
1000
frames

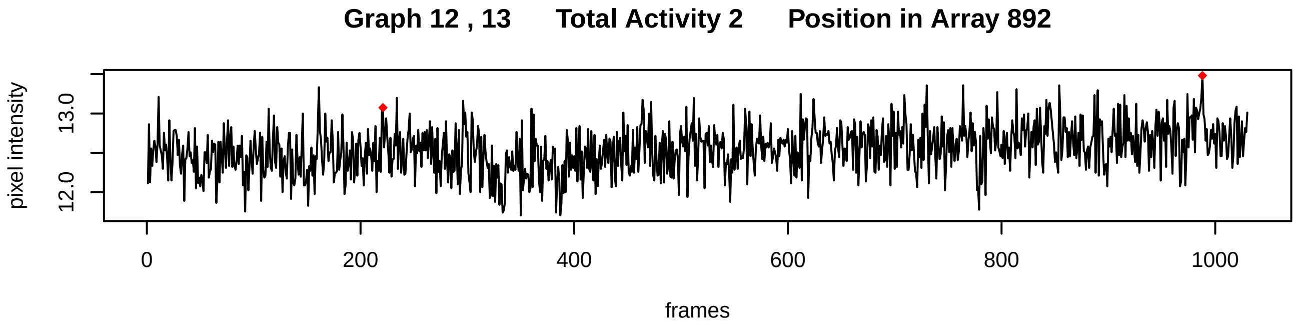
Graph 12 , 13 Total Activity 2 Position in Array 892
pixel intensity
12.0
13.0
0
200
400
600
800
1000
frames

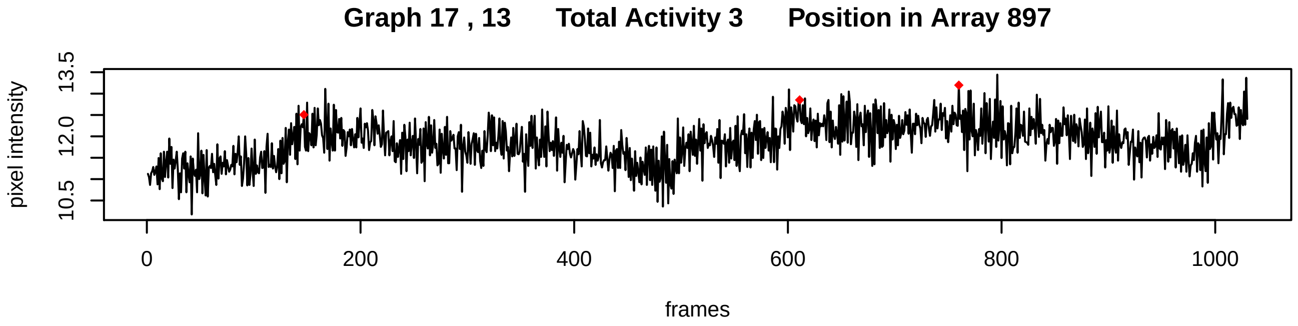
Graph 17 , 13 Total Activity 3 Position in Array 897
pixel intensity
10.5
12.0
13.5
0
200
400
600
800
1000
frames

Graph 18 , 13   Total Activity 20   Position in Array 898

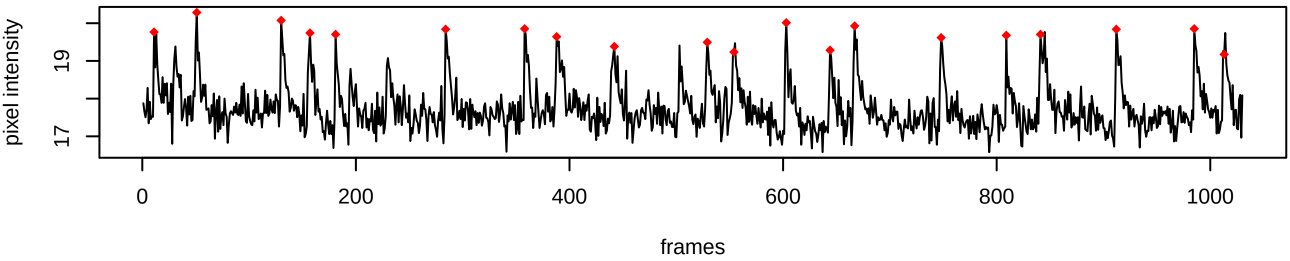

Graph 19 , 13   Total Activity 21   Position in Array 899

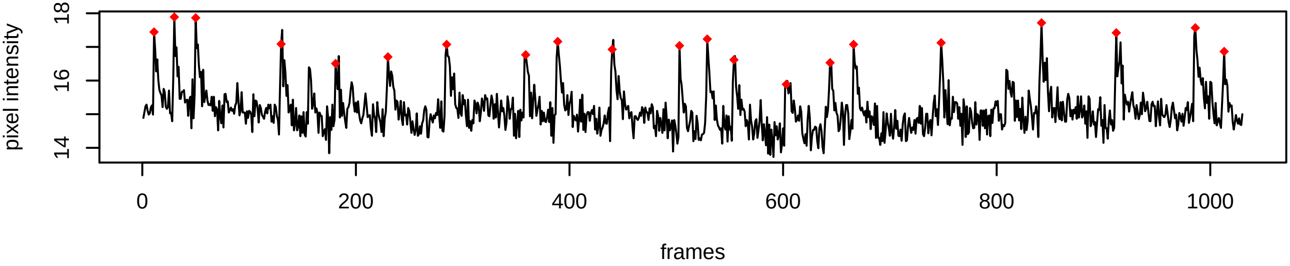

Graph 20 , 13   Total Activity 3   Position in Array 900

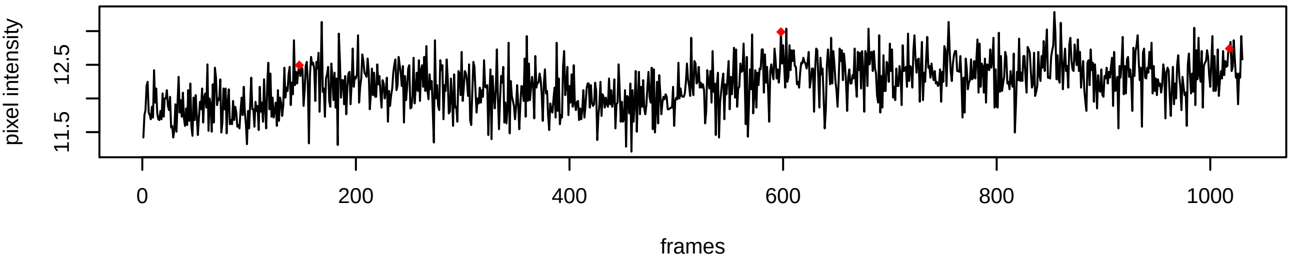

Graph 21 , 13   Total Activity 2   Position in Array 901

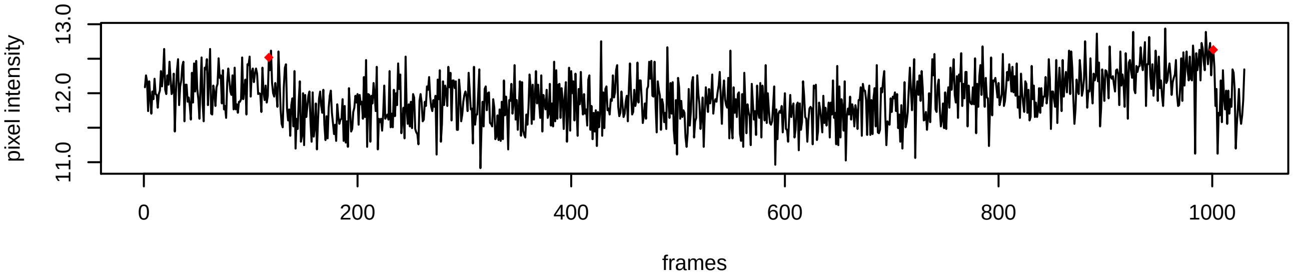

Graph 22 , 13   Total Activity 3   Position in Array 902

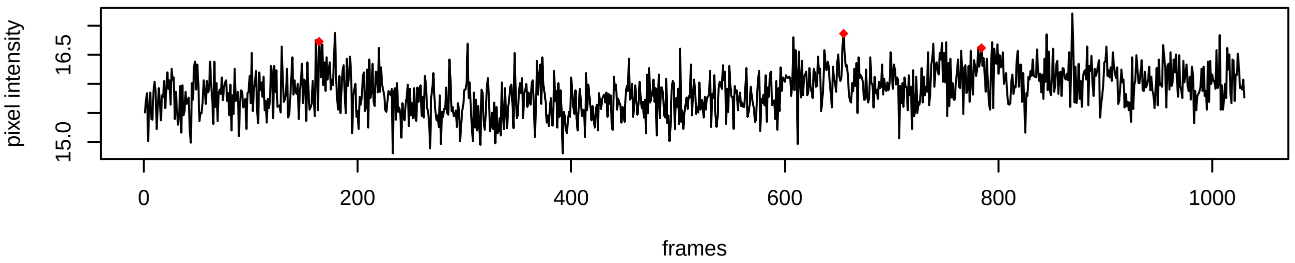

Graph 25 , 13   Total Activity 4   Position in Array 905

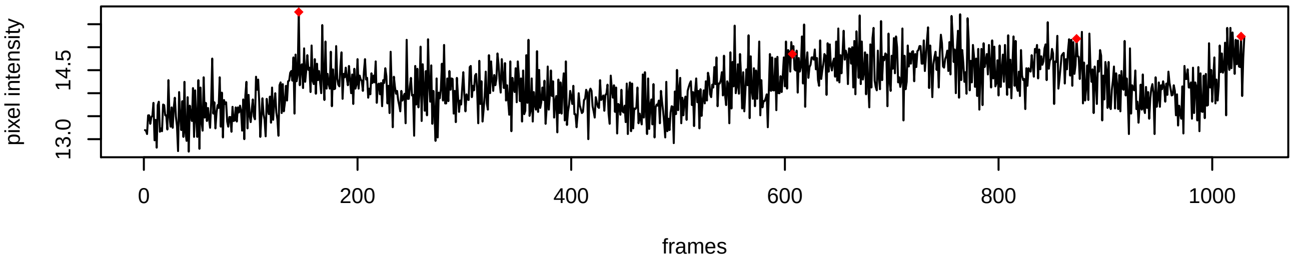

Graph 26 , 13   Total Activity 2   Position in Array 906

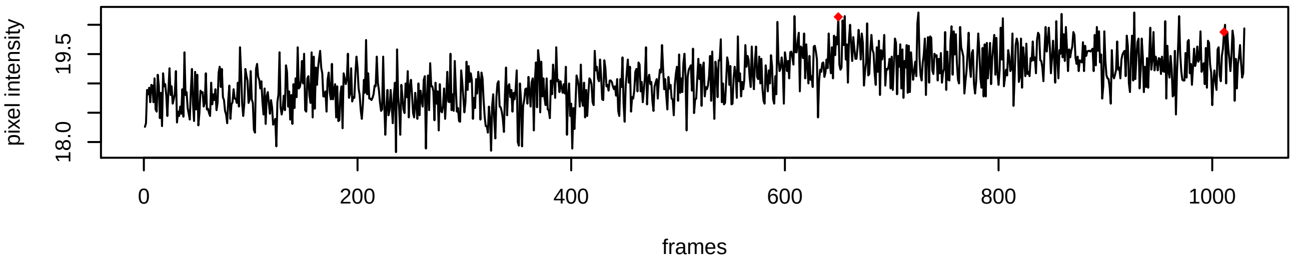

Graph 27 , 13   Total Activity 3   Position in Array 907

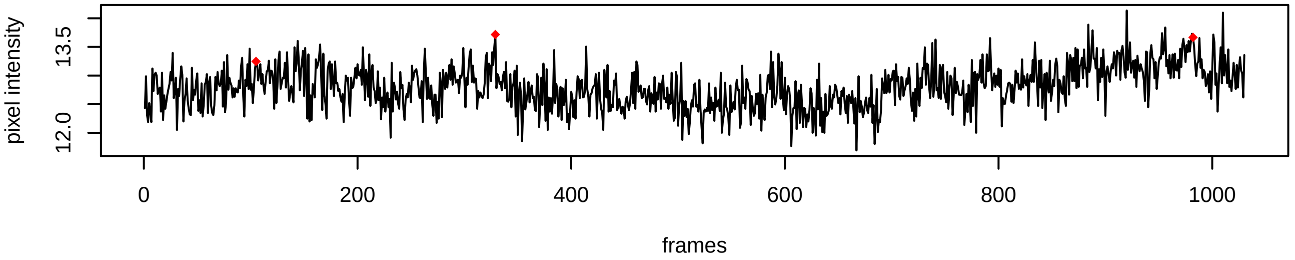

Graph 29 , 13   Total Activity 4   Position in Array 909

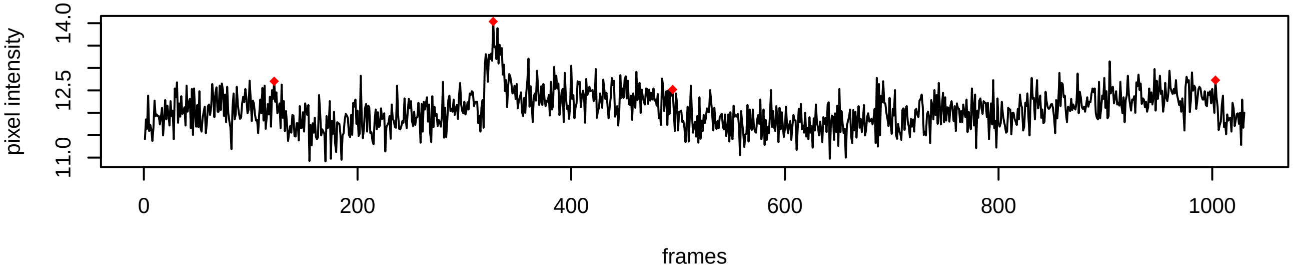

Graph 31 , 13 Total Activity 2 Position in Array 911

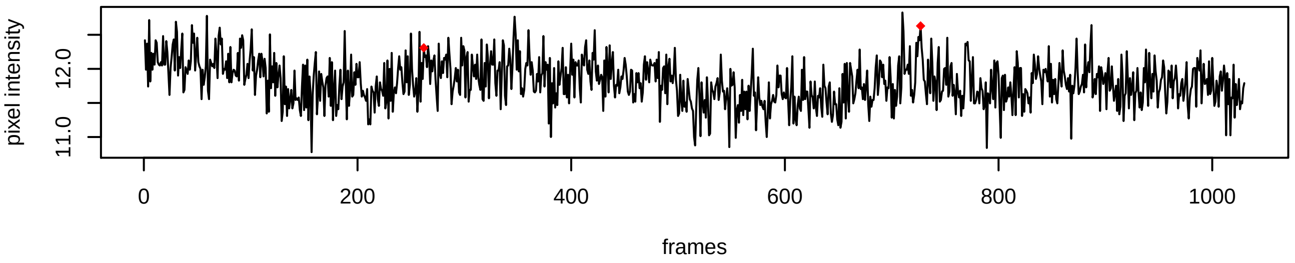

Graph 34 , 13 Total Activity 4 Position in Array 914

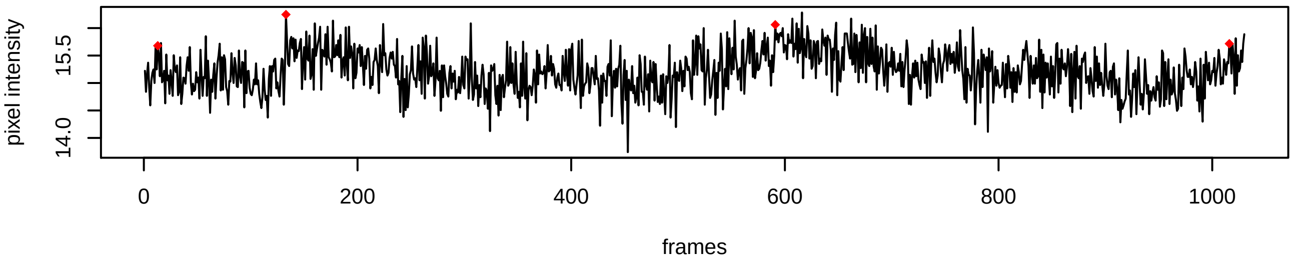

Graph 35 , 13 Total Activity 3 Position in Array 915

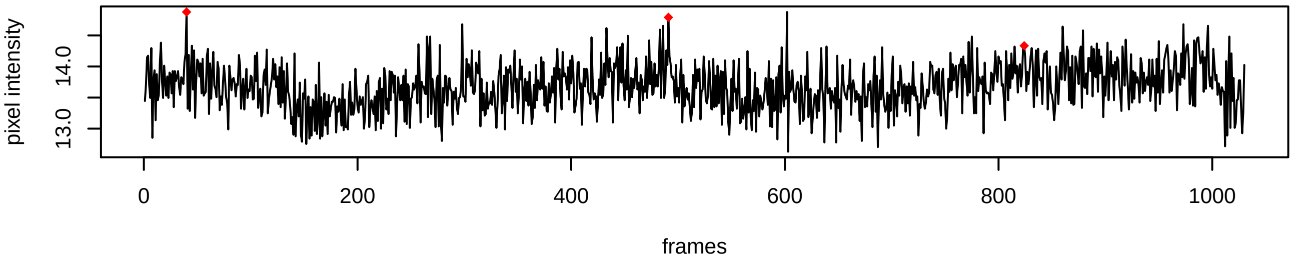

Graph 40 , 13 Total Activity 3 Position in Array 920

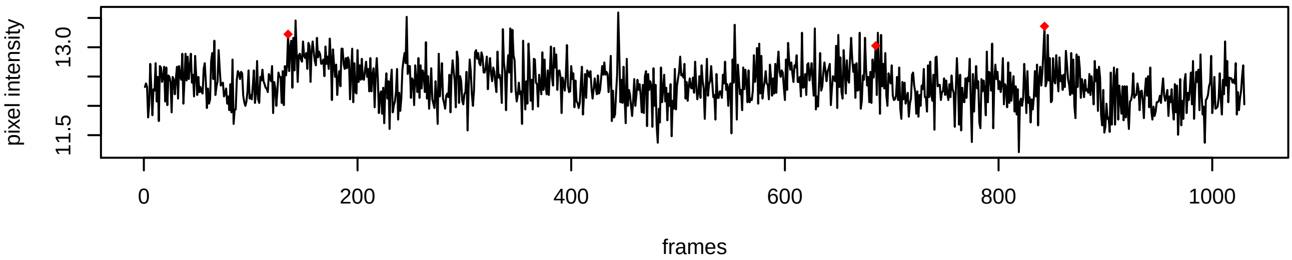

Graph 41 , 13 Total Activity 4 Position in Array 921

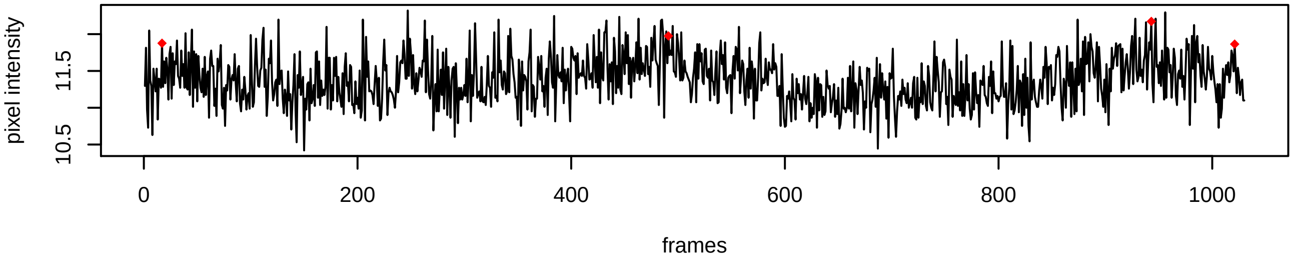

Graph 42 , 13 Total Activity 4 Position in Array 922

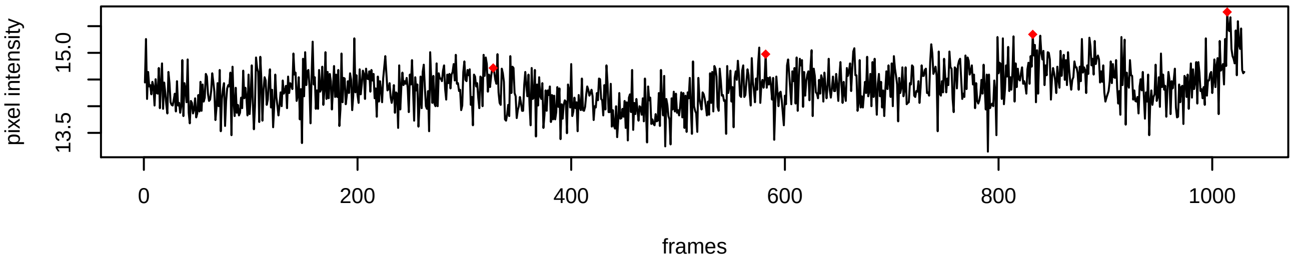

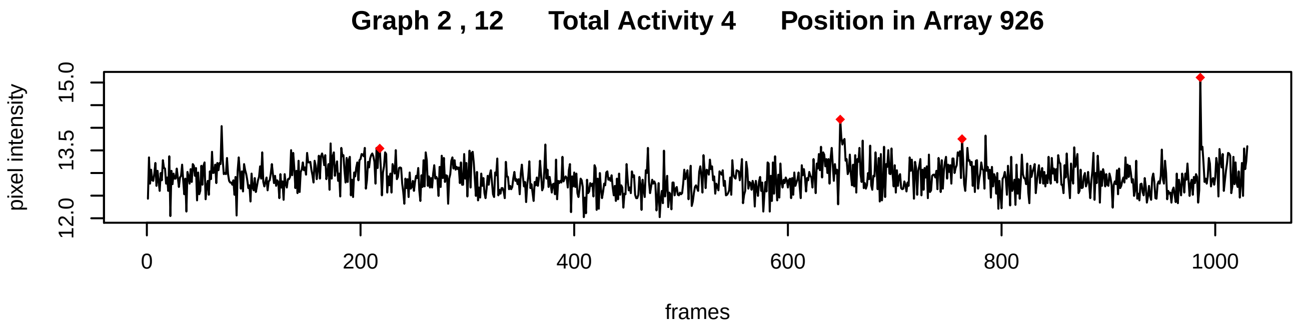
Graph 2 , 12 Total Activity 4 Position in Array 926
pixel intensity
12.0
13.5
15.0
0
200
400
600
800
1000
frames

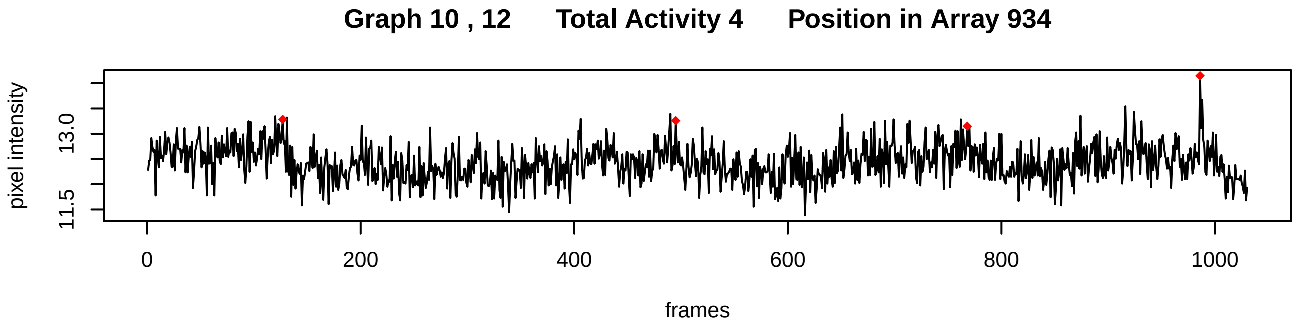
Graph 10 , 12 Total Activity 4 Position in Array 934
pixel intensity
11.5
13.0
0
200
400
600
800
1000
frames

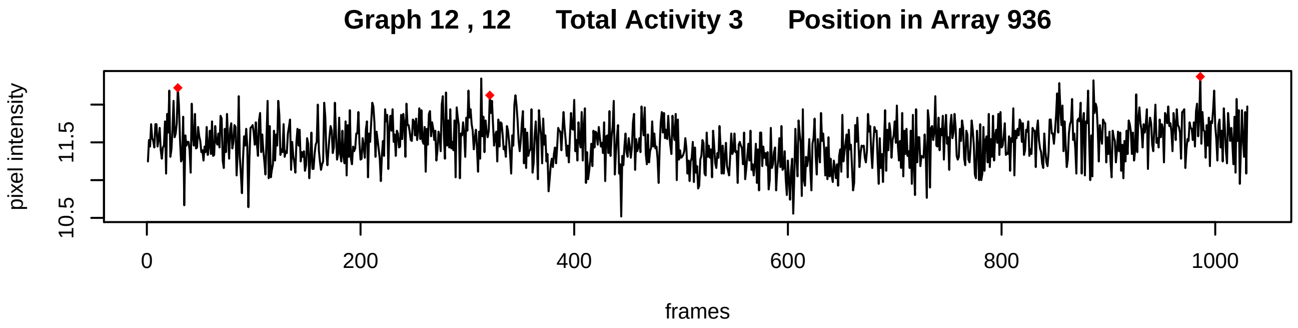
Graph 12 , 12 Total Activity 3 Position in Array 936
pixel intensity
10.5
11.5
0
200
400
600
800
1000
frames

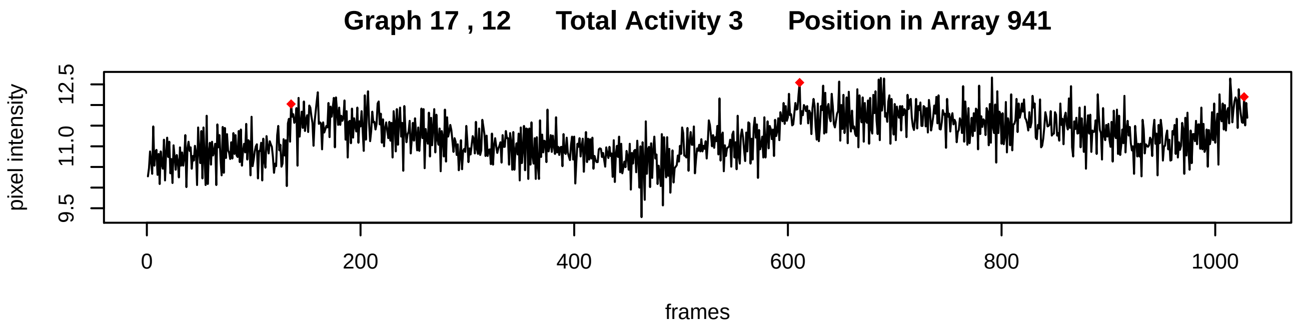
Graph 17 , 12 Total Activity 3 Position in Array 941
pixel intensity
9.5
11.0
12.5
0
200
400
600
800
1000
frames

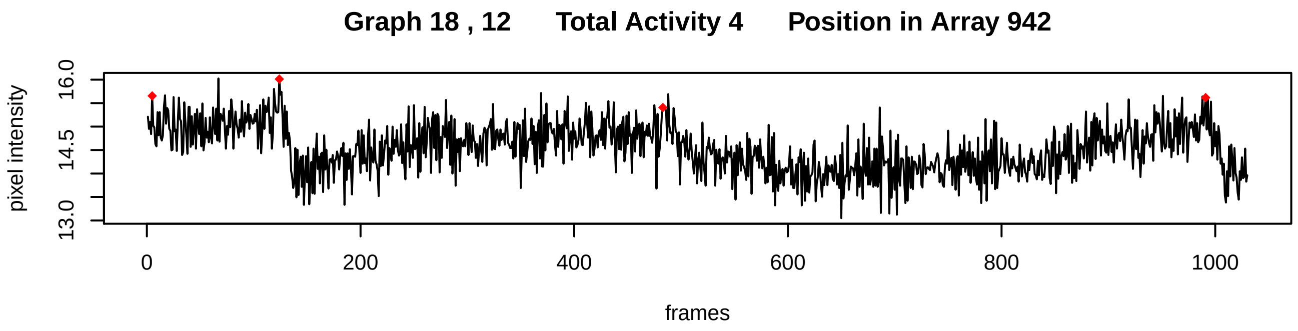
Graph 18 , 12 Total Activity 4 Position in Array 942
pixel intensity
13.0
14.5
16.0
0
200
400
600
800
1000
frames

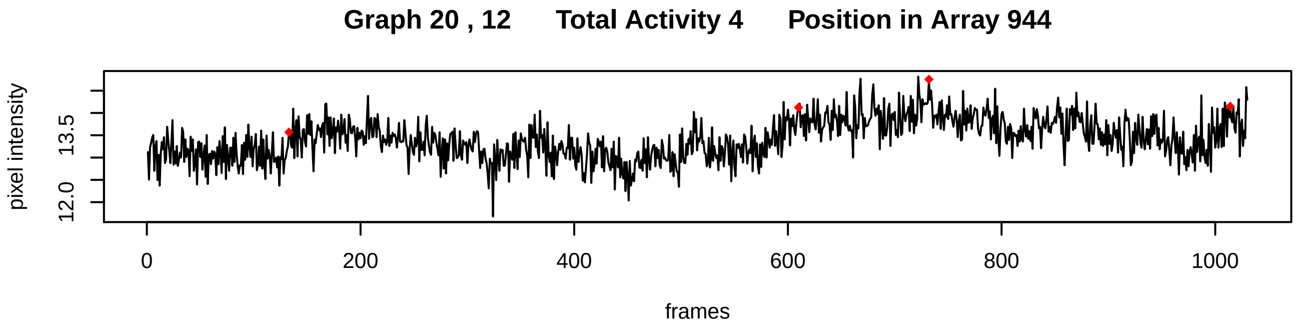
Graph 20 , 12 Total Activity 4 Position in Array 944
pixel intensity
12.0
13.5
0
200
400
600
800
1000
frames

Graph 22 , 12 Total Activity 2 Position in Array 946

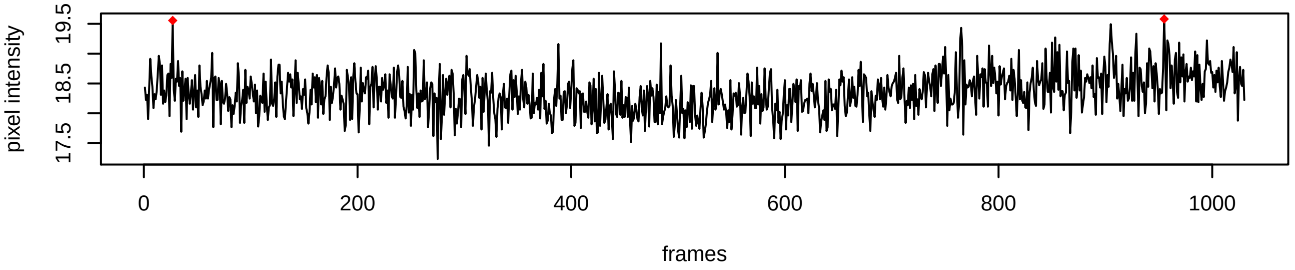

Graph 26 , 12 Total Activity 3 Position in Array 950

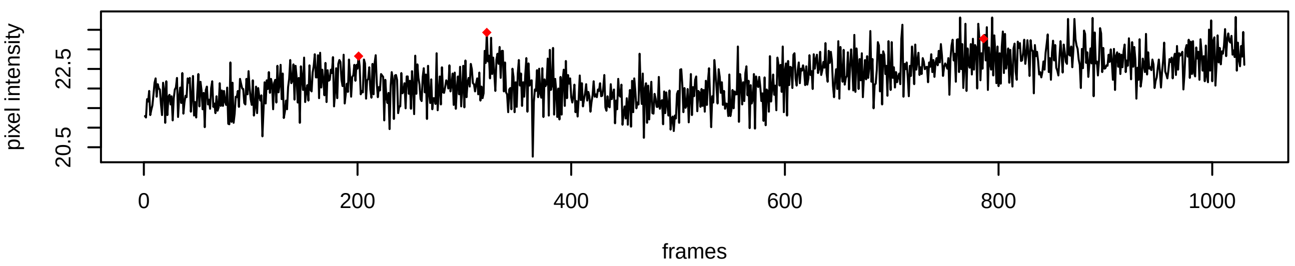

Graph 27 , 12 Total Activity 3 Position in Array 951

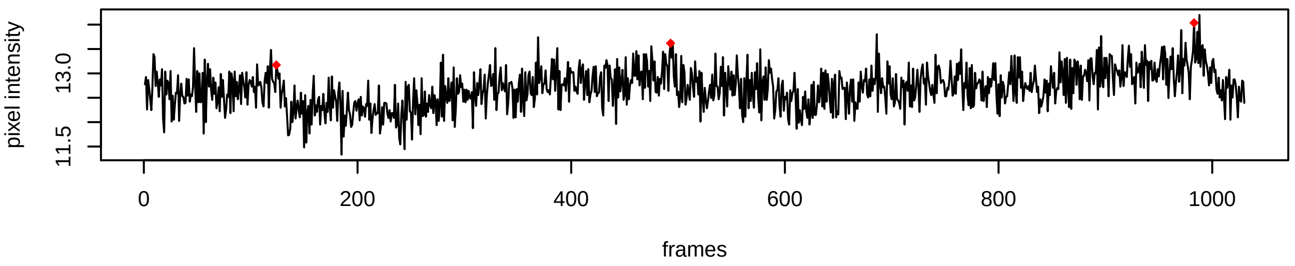

Graph 28 , 12 Total Activity 6 Position in Array 952

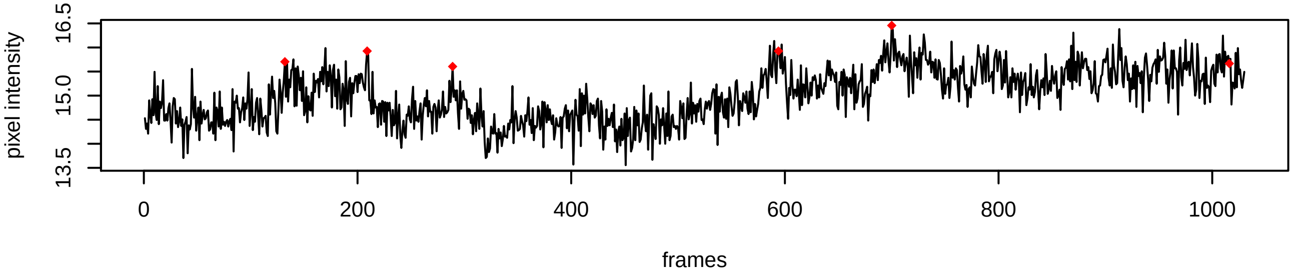

Graph 33 , 12 Total Activity 2 Position in Array 957

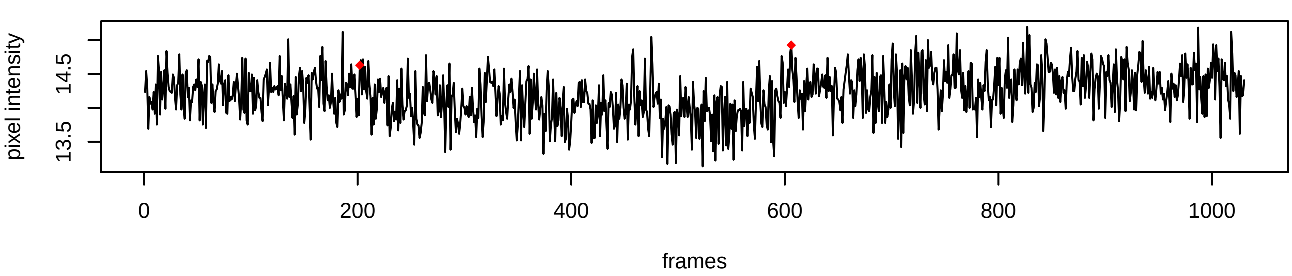

Graph 34 , 12 Total Activity 3 Position in Array 958

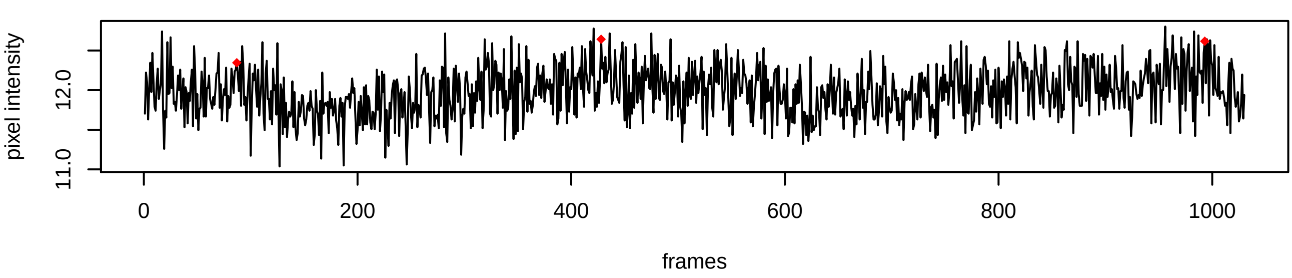

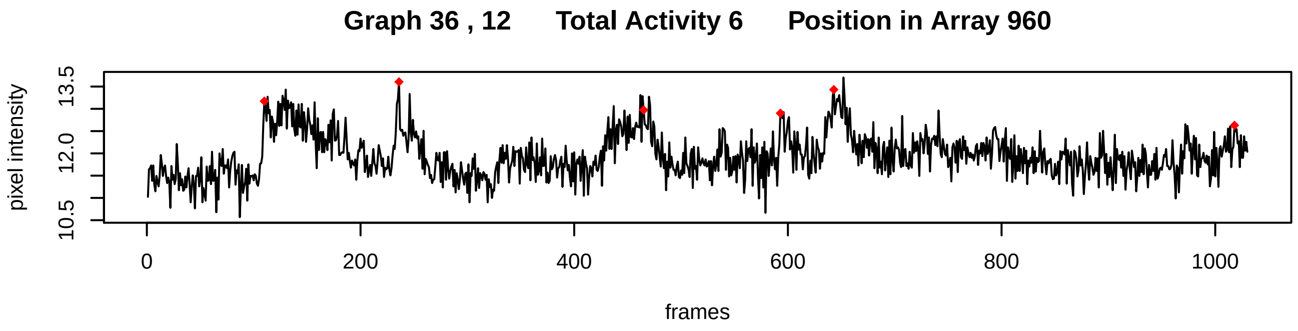
Graph 36 , 12 Total Activity 6 Position in Array 960
pixel intensity
10.5
12.0
13.5
0
200
400
600
800
1000
frames

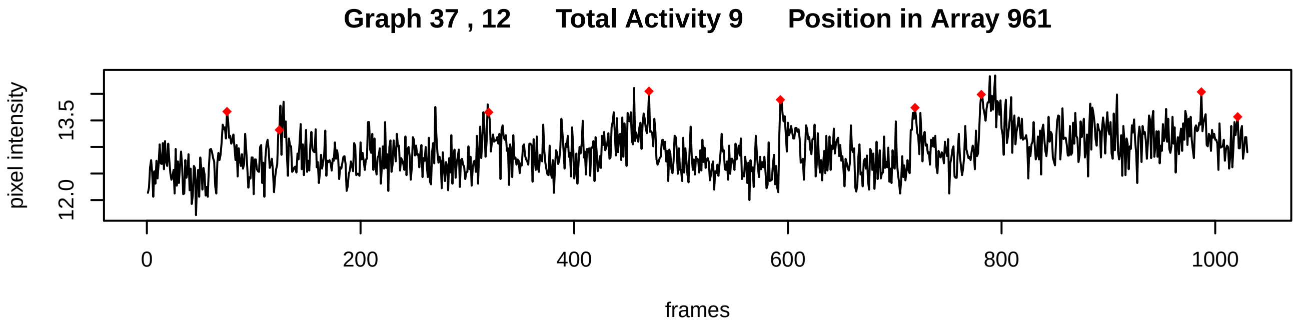
Graph 37 , 12 Total Activity 9 Position in Array 961
pixel intensity
12.0
13.5
0
200
400
600
800
1000
frames

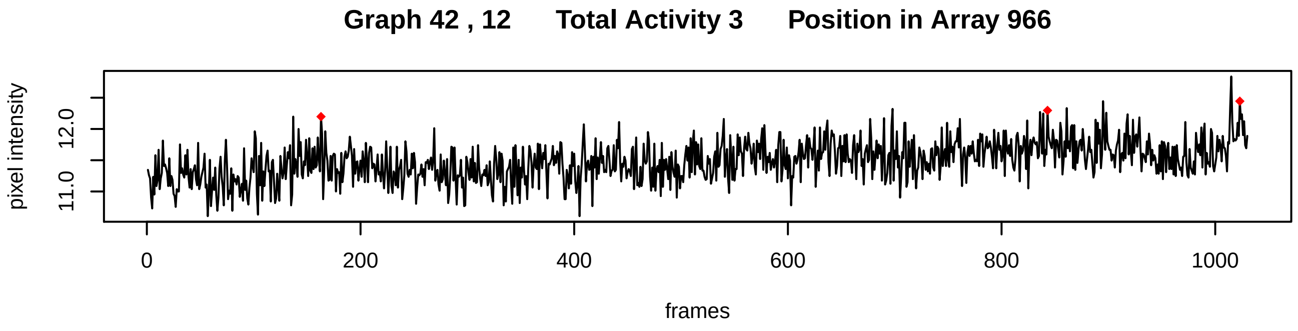
Graph 42 , 12 Total Activity 3 Position in Array 966
pixel intensity
11.0
12.0
0
200
400
600
800
1000
frames

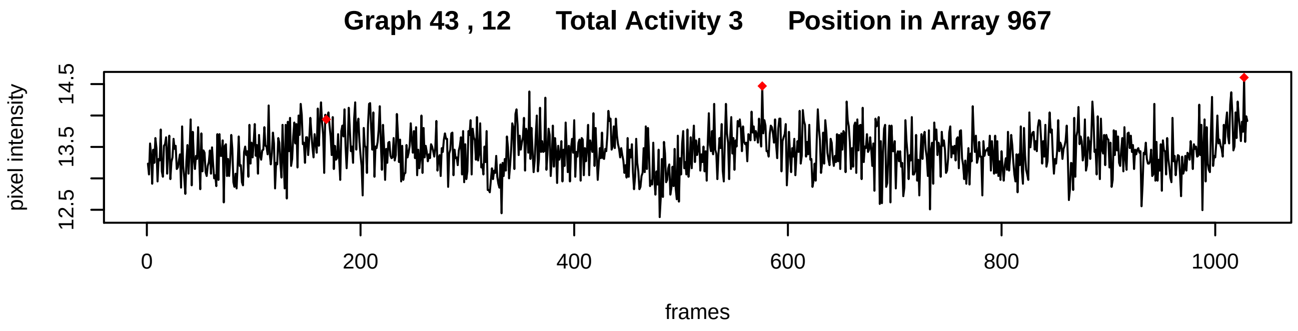
Graph 43 , 12 Total Activity 3 Position in Array 967
pixel intensity
12.5
13.5
14.5
0
200
400
600
800
1000
frames

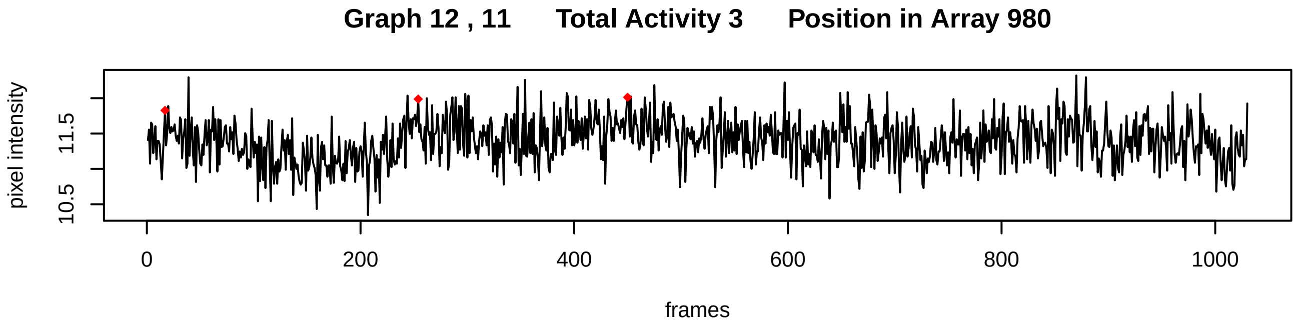
Graph 12 , 11 Total Activity 3 Position in Array 980
pixel intensity
10.5
11.5
0
200
400
600
800
1000
frames

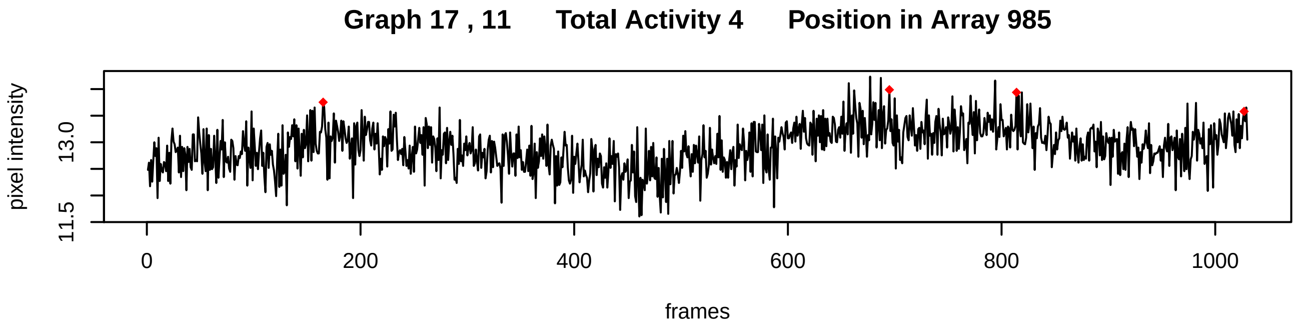
Graph 17 , 11 Total Activity 4 Position in Array 985
pixel intensity
11.5
13.0
0
200
400
600
800
1000
frames

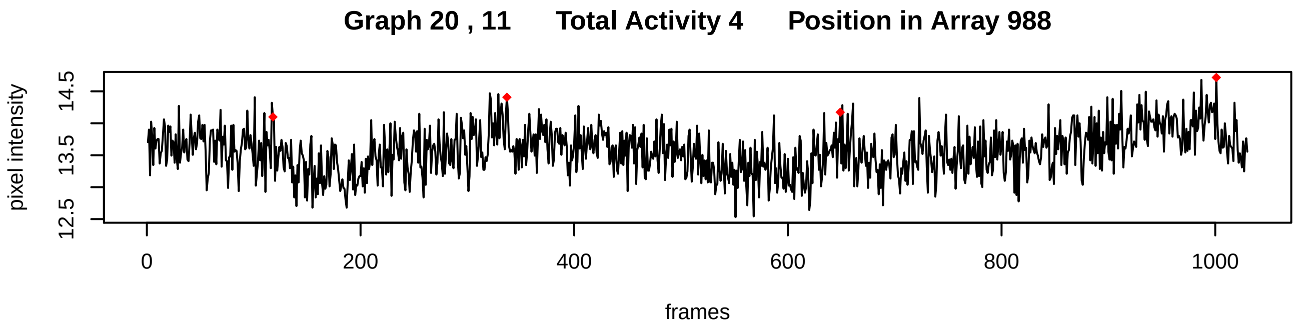
Graph 20 , 11 Total Activity 4 Position in Array 988
pixel intensity
12.5
13.5
14.5
0
200
400
600
800
1000
frames

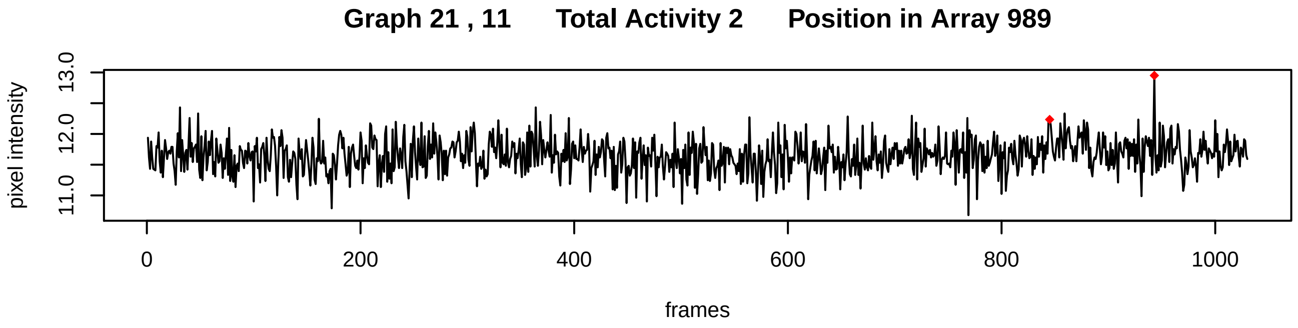
Graph 21 , 11 Total Activity 2 Position in Array 989
pixel intensity
11.0
12.0
13.0
0
200
400
600
800
1000
frames

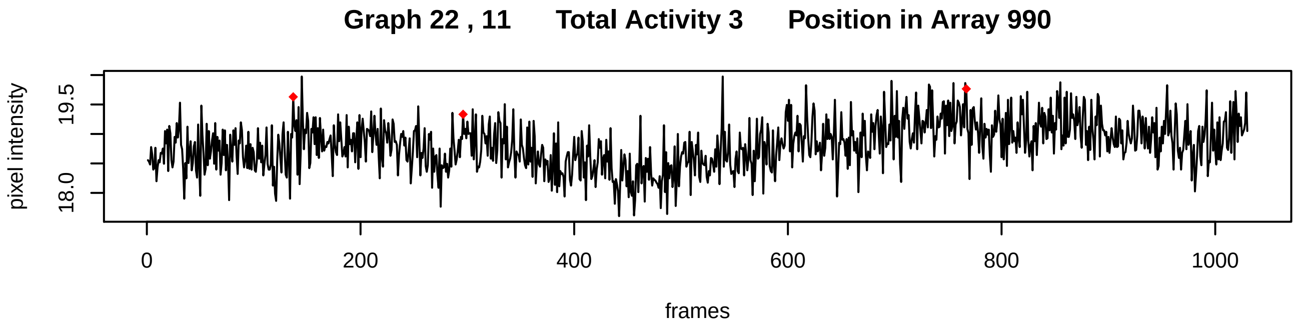
Graph 22 , 11 Total Activity 3 Position in Array 990
pixel intensity
18.0
19.5
0
200
400
600
800
1000
frames

Graph 28 , 11   Total Activity 4   Position in Array 996

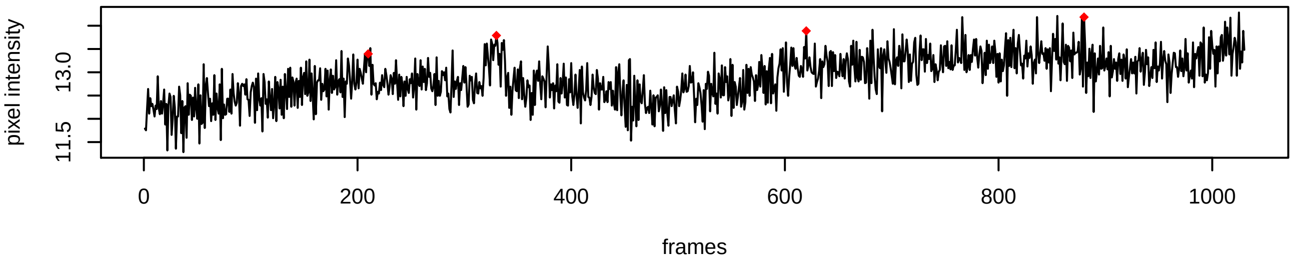

Graph 29 , 11   Total Activity 9   Position in Array 997

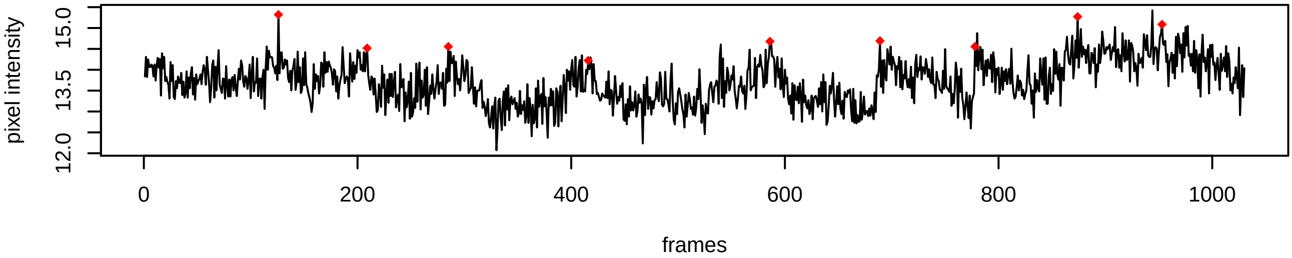

Graph 30 , 11   Total Activity 3   Position in Array 998

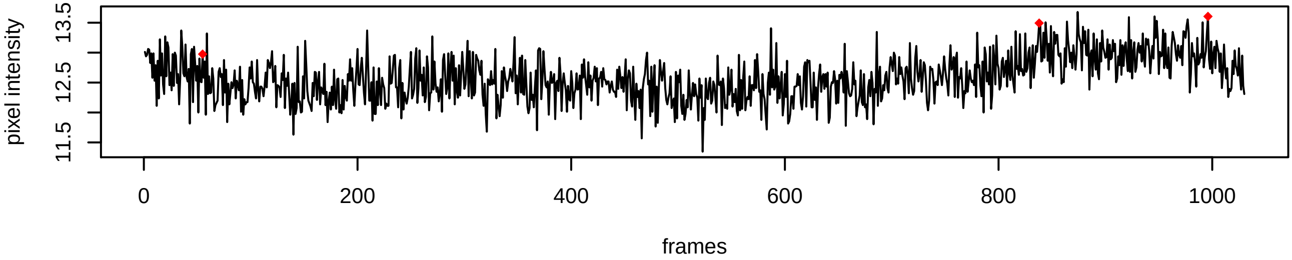

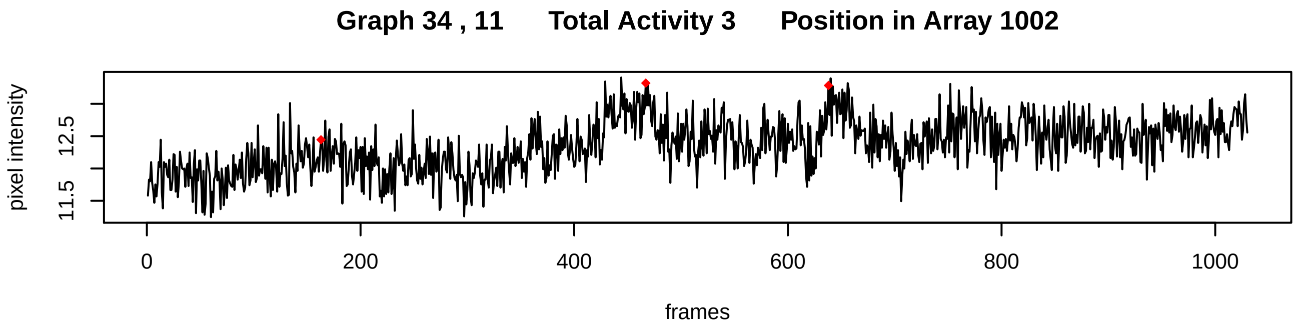
Graph 34 , 11 Total Activity 3 Position in Array 1002
pixel intensity
11.5
12.5
0
200
400
600
800
1000
frames

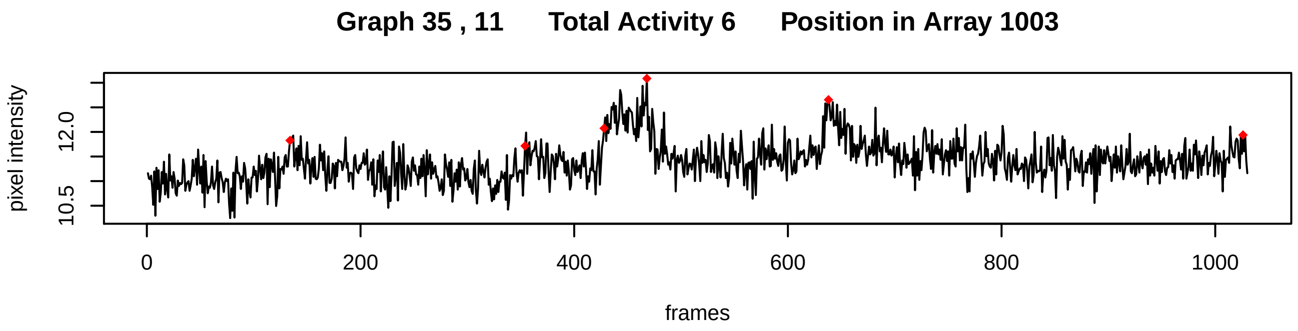
Graph 35 , 11 Total Activity 6 Position in Array 1003
pixel intensity
10.5
12.0
0
200
400
600
800
1000
frames

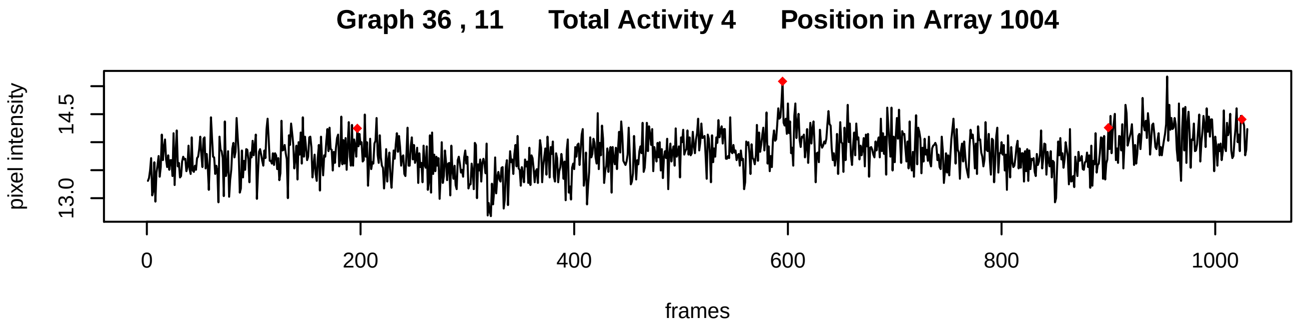
Graph 36 , 11 Total Activity 4 Position in Array 1004
pixel intensity
13.0
14.5
0
200
400
600
800
1000
frames

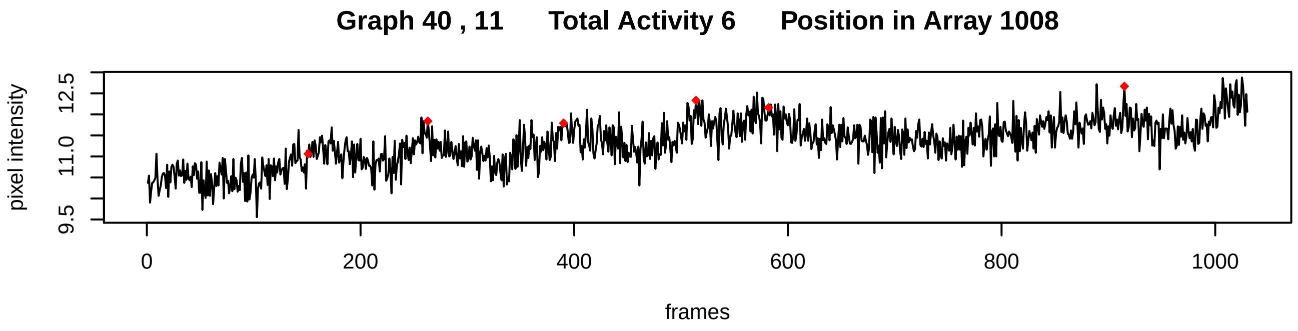
Graph 40 , 11 Total Activity 6 Position in Array 1008
pixel intensity
9.5
11.0
12.5
0
200
400
600
800
1000
frames

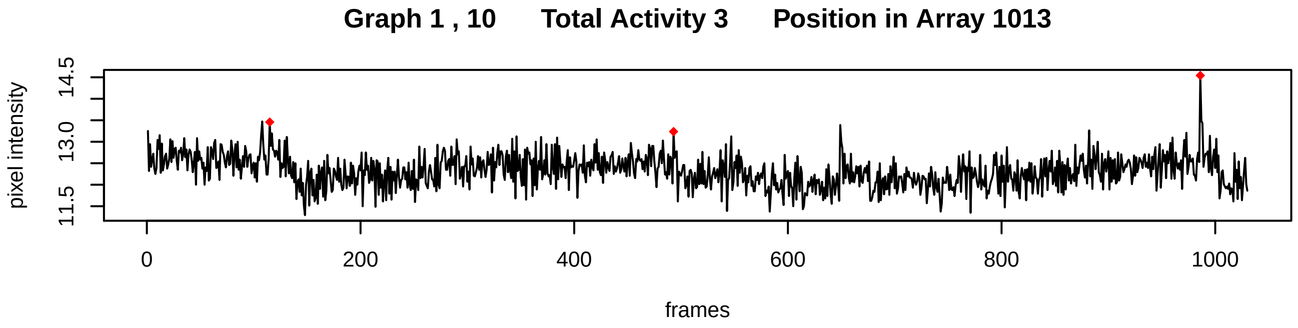
Graph 1 , 10 Total Activity 3 Position in Array 1013
pixel intensity
11.5
13.0
14.5
0
200
400
600
800
1000
frames

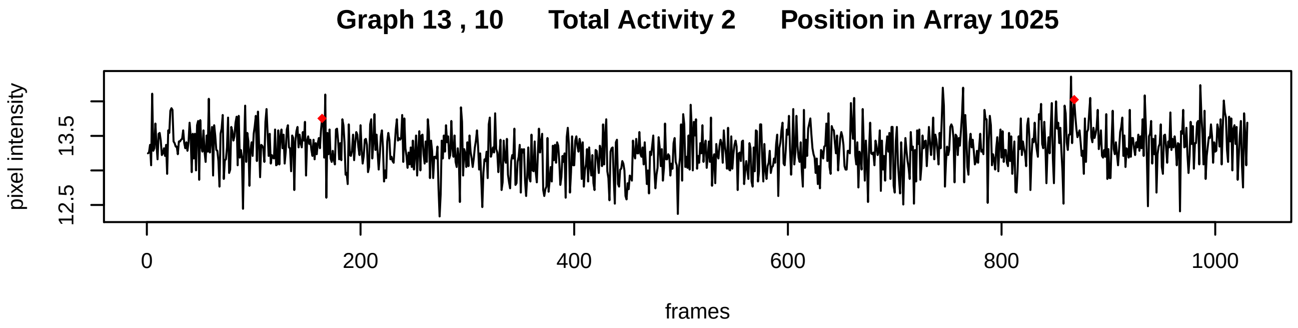
Graph 13 , 10 Total Activity 2 Position in Array 1025
pixel intensity
12.5
13.5
0
200
400
600
800
1000
frames

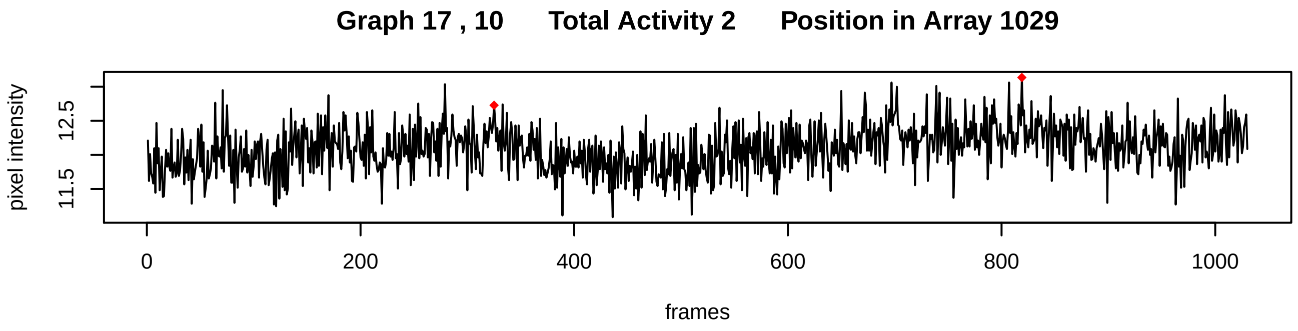
Graph 17 , 10 Total Activity 2 Position in Array 1029
pixel intensity
11.5
12.5
0
200
400
600
800
1000
frames

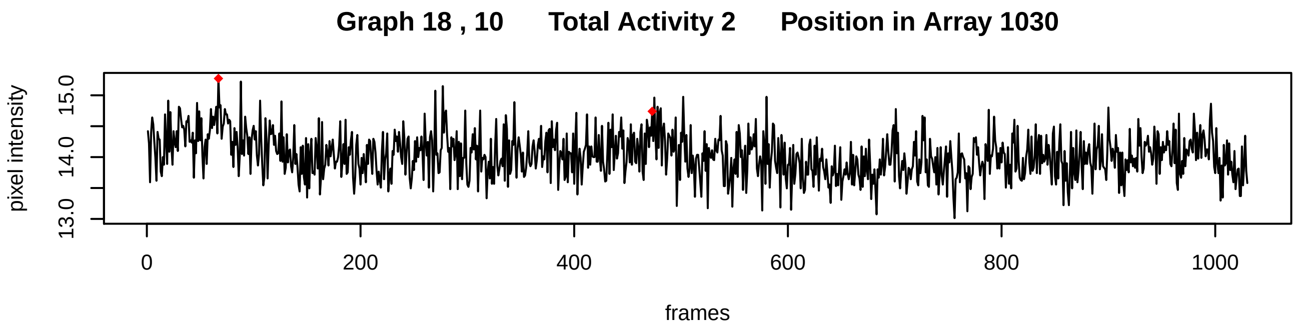
Graph 18 , 10 Total Activity 2 Position in Array 1030
pixel intensity
13.0
14.0
15.0
0
200
400
600
800
1000
frames

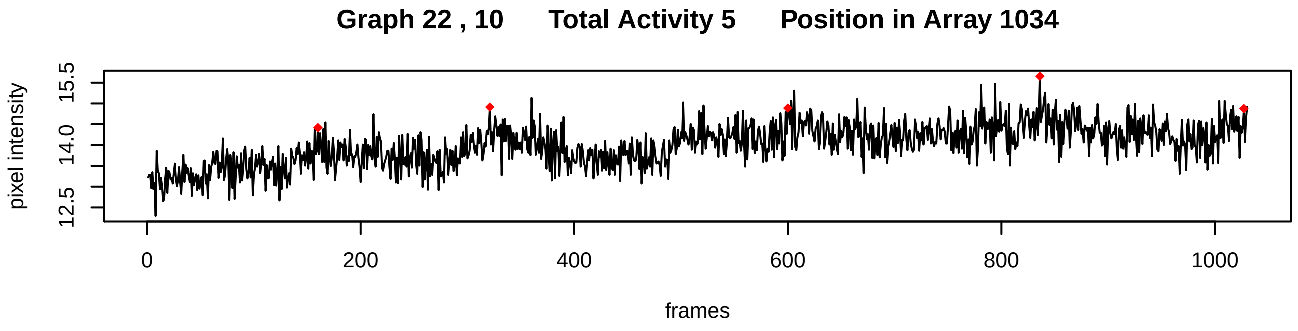
Graph 22 , 10 Total Activity 5 Position in Array 1034
pixel intensity
12.5
14.0
15.5
0
200
400
600
800
1000
frames

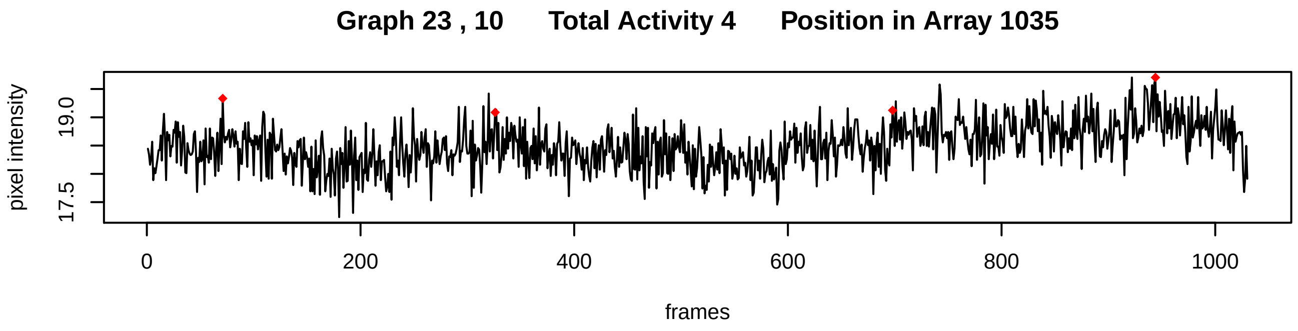
Graph 23 , 10 Total Activity 4 Position in Array 1035
pixel intensity
17.5
19.0
0
200
400
600
800
1000
frames

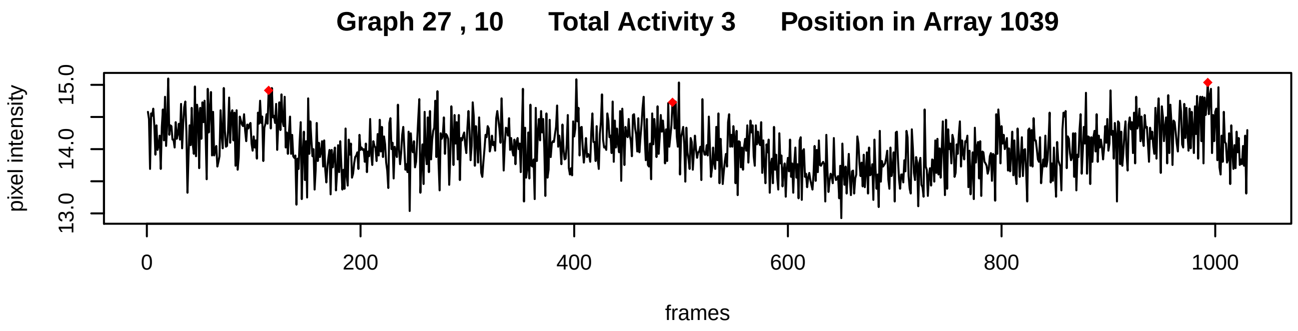
Graph 27 , 10 Total Activity 3 Position in Array 1039
pixel intensity
13.0
14.0
15.0
0
200
400
600
800
1000
frames

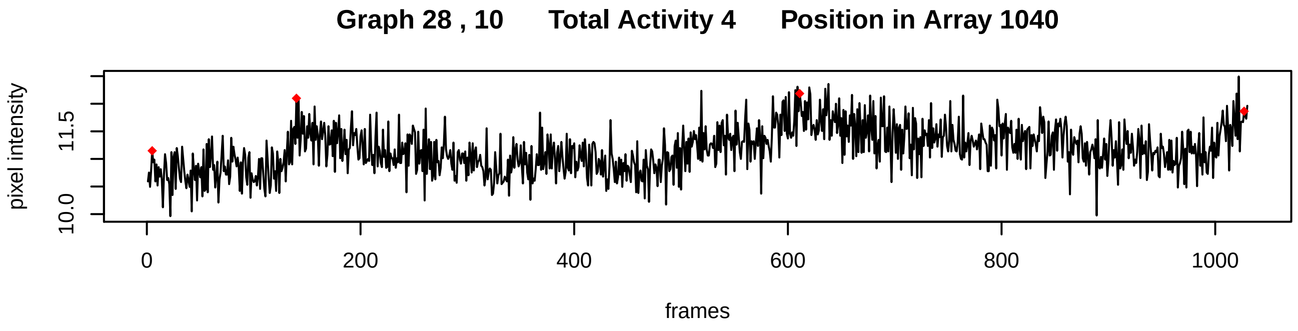
Graph 28 , 10 Total Activity 4 Position in Array 1040
pixel intensity
10.0
11.5
0
200
400
600
800
1000
frames

Graph 29 , 10 Total Activity 3 Position in Array 1041

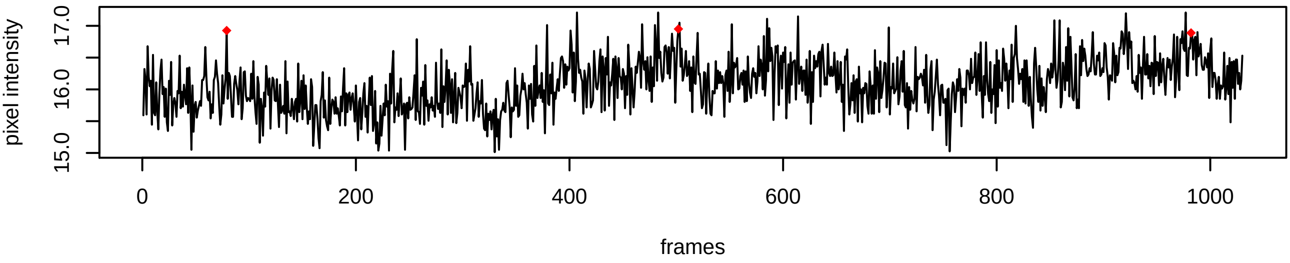

Graph 30 , 10 Total Activity 6 Position in Array 1042

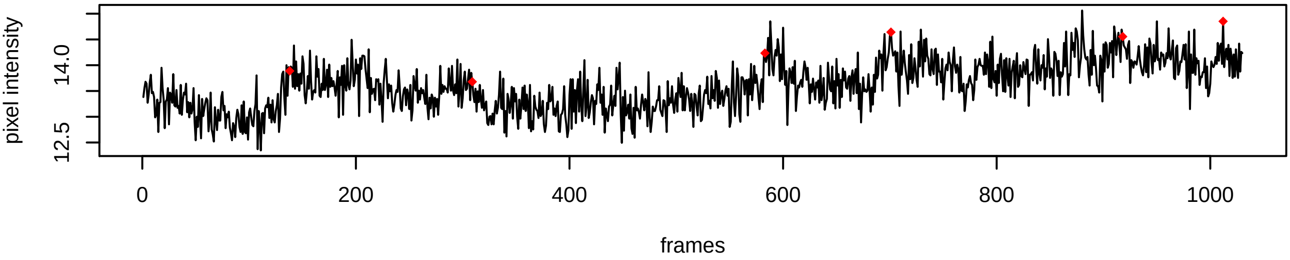

Graph 34 , 10 Total Activity 2 Position in Array 1046

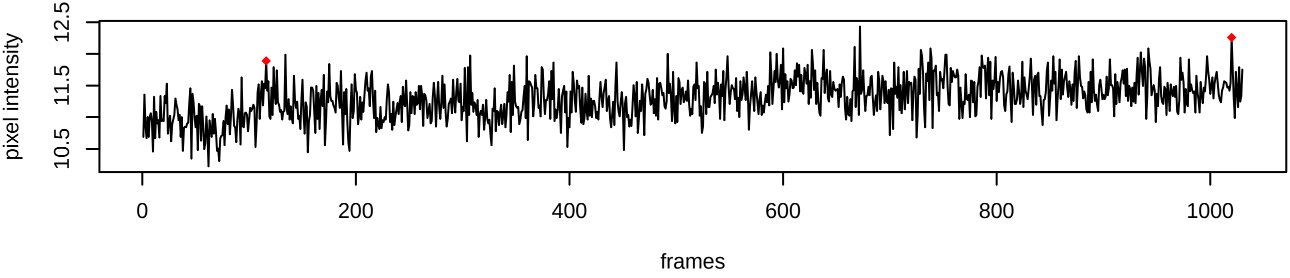

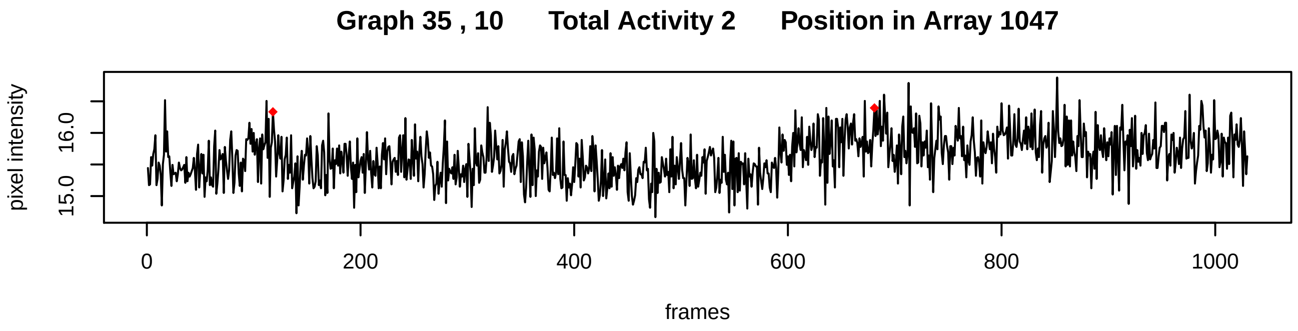
Graph 35 , 10 Total Activity 2 Position in Array 1047
pixel intensity
15.0
16.0
0
200
400
600
800
1000
frames

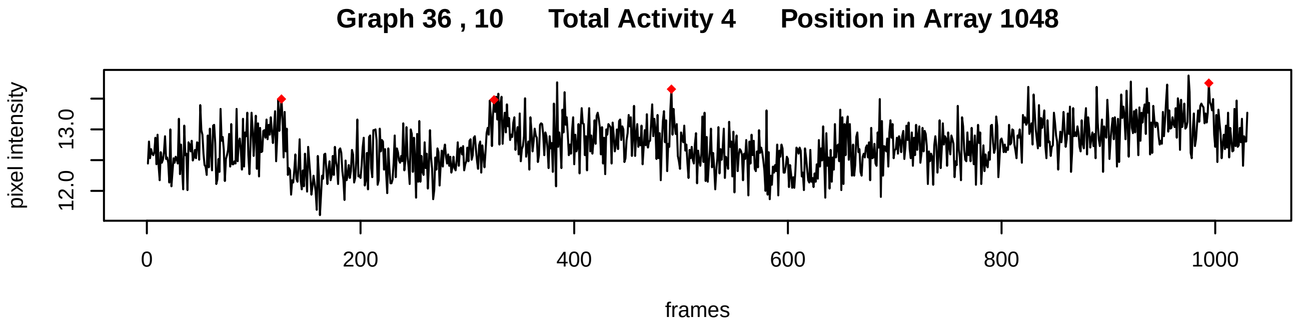
Graph 36 , 10 Total Activity 4 Position in Array 1048
pixel intensity
12.0
13.0
0
200
400
600
800
1000
frames

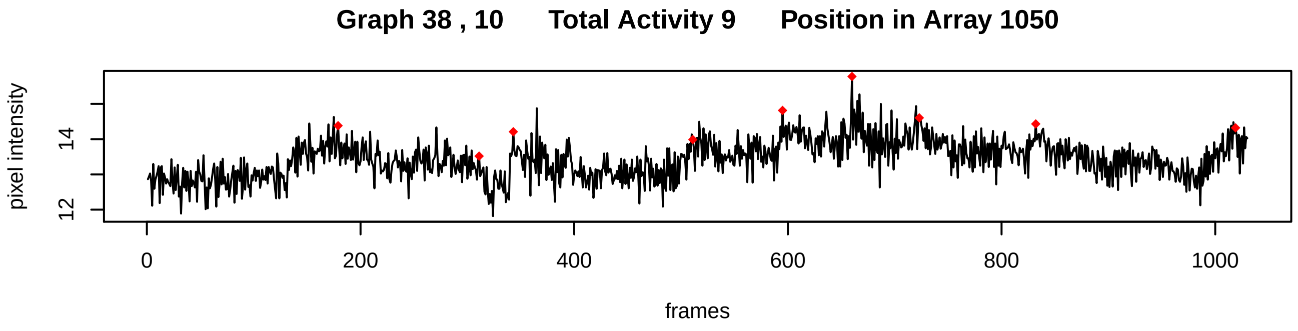
Graph 38 , 10 Total Activity 9 Position in Array 1050
pixel intensity
12
14
0
200
400
600
800
1000
frames

Graph 39 , 10   Total Activity 5   Position in Array 1051

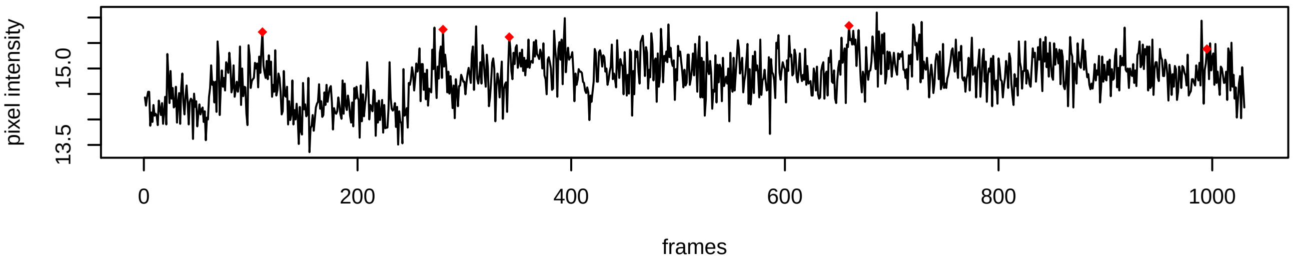

Graph 15 , 9   Total Activity 3   Position in Array 1071

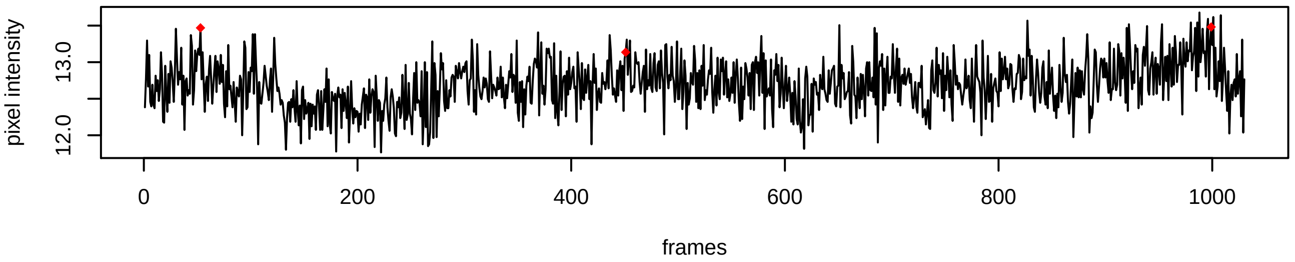

Graph 16 , 9   Total Activity 2   Position in Array 1072

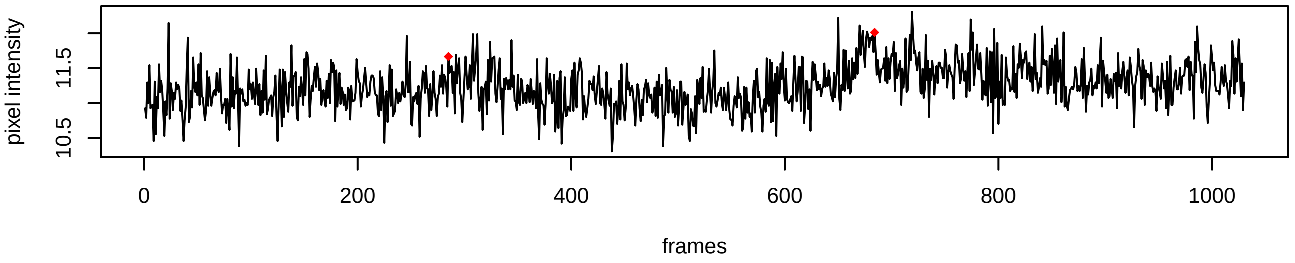

Graph 18 , 9 Total Activity 4 Position in Array 1074

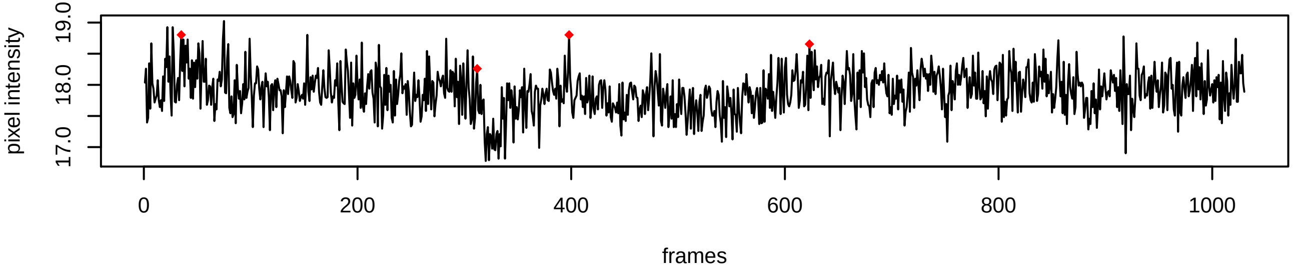

Graph 23 , 9 Total Activity 5 Position in Array 1079

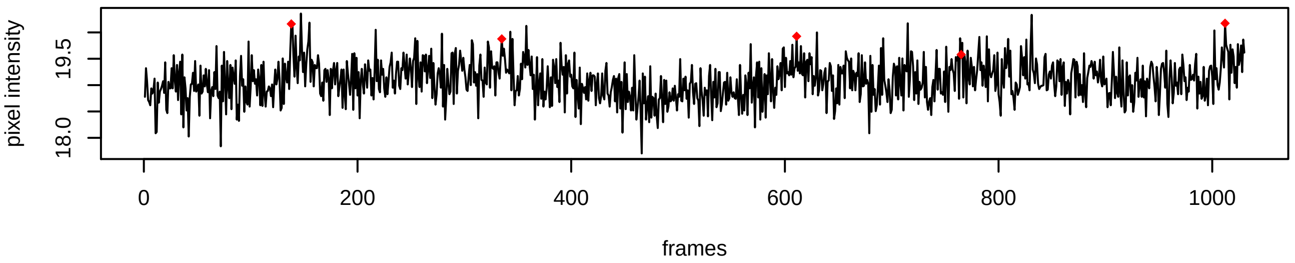

Graph 26 , 9 Total Activity 3 Position in Array 1082

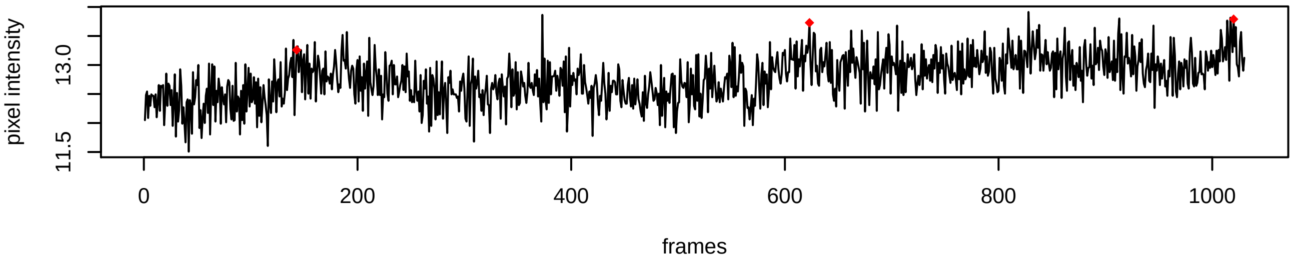

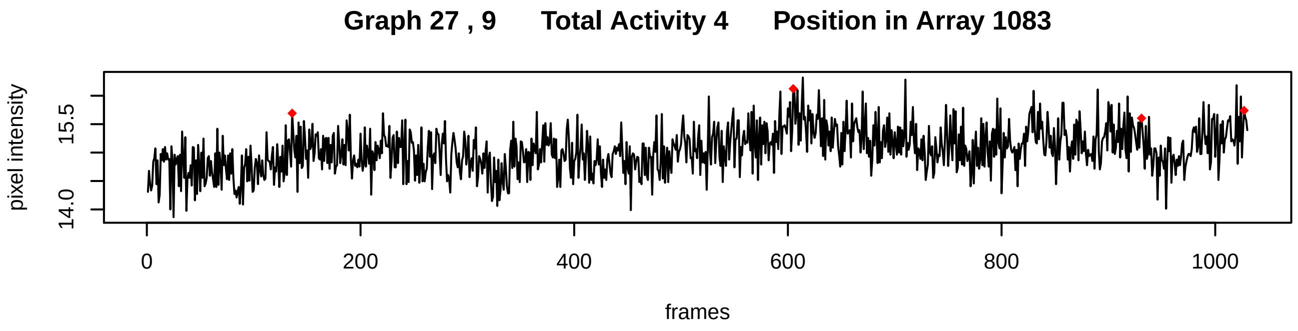
Graph 27 , 9 Total Activity 4 Position in Array 1083
pixel intensity
14.0
15.5
0
200
400
600
800
1000
frames

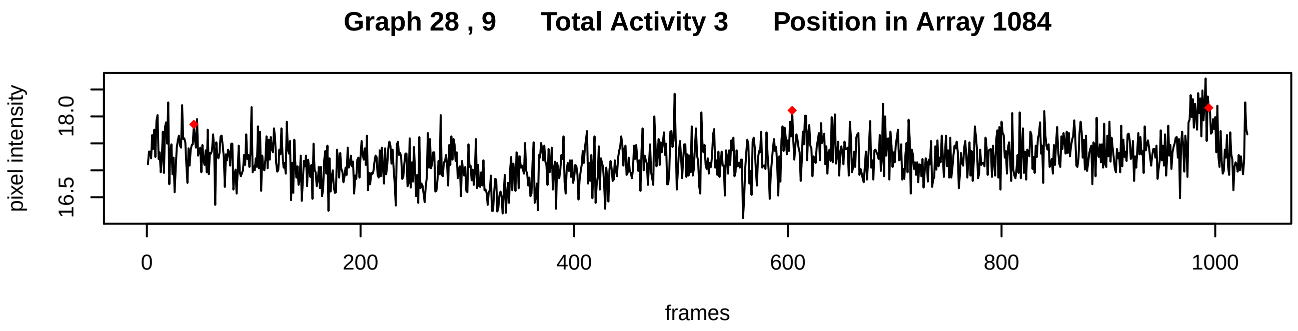
Graph 28 , 9 Total Activity 3 Position in Array 1084
pixel intensity
16.5
18.0
0
200
400
600
800
1000
frames

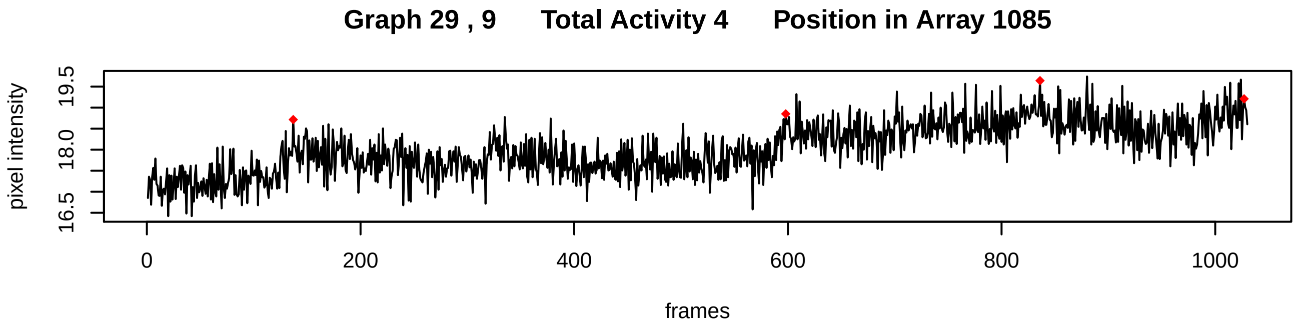
Graph 29 , 9 Total Activity 4 Position in Array 1085
pixel intensity
16.5
18.0
18.0
19.5
0
200
400
600
800
1000
frames

Graph 30 , 9   Total Activity 3   Position in Array 1086

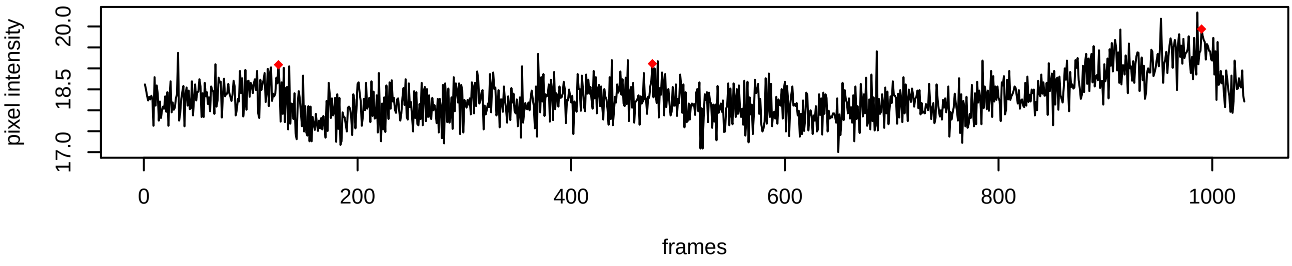

Graph 32 , 9   Total Activity 4   Position in Array 1088

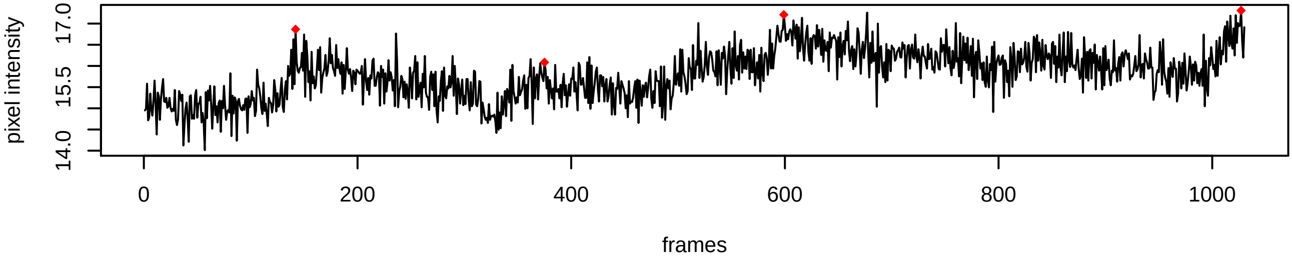

Graph 33 , 9   Total Activity 3   Position in Array 1089

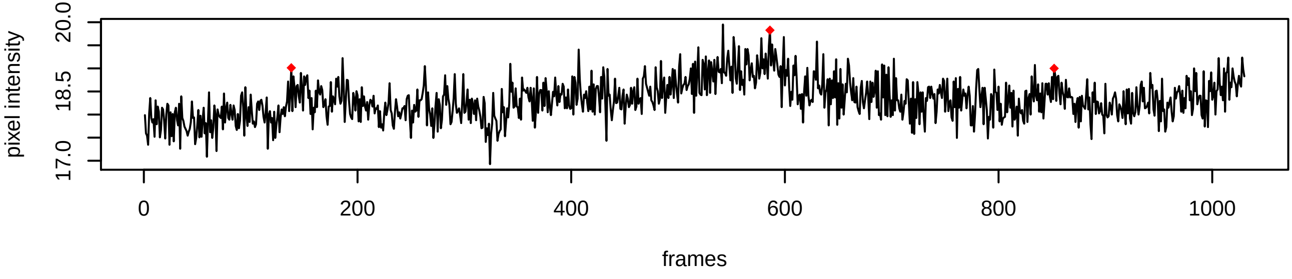

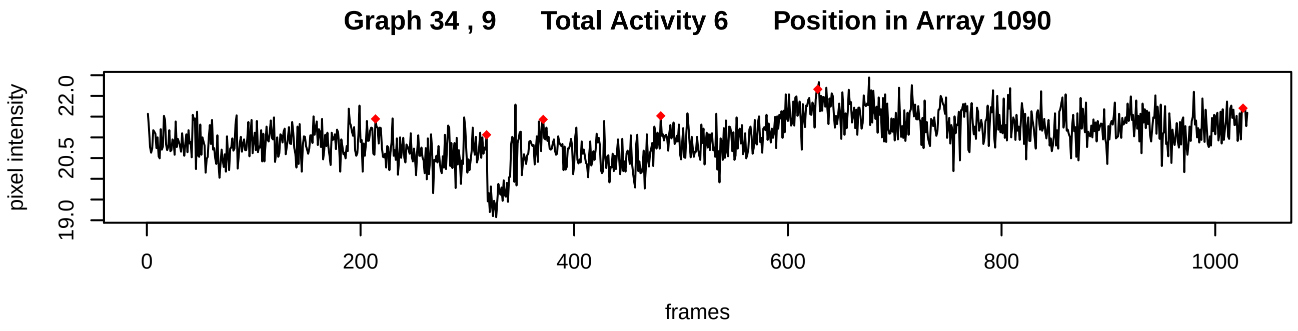
Graph 34 , 9 Total Activity 6 Position in Array 1090
pixel intensity
19.0
20.5
22.0
0
200
400
600
800
1000
frames

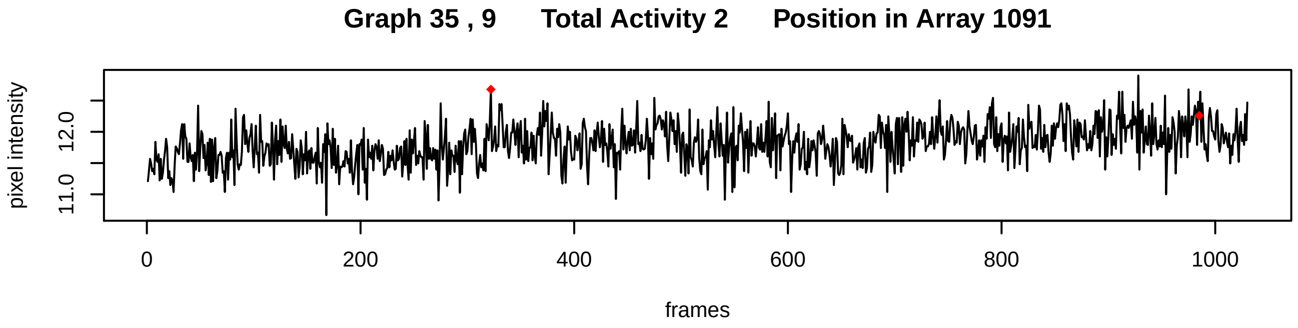
Graph 35 , 9 Total Activity 2 Position in Array 1091
pixel intensity
11.0
12.0
0
200
400
600
800
1000
frames

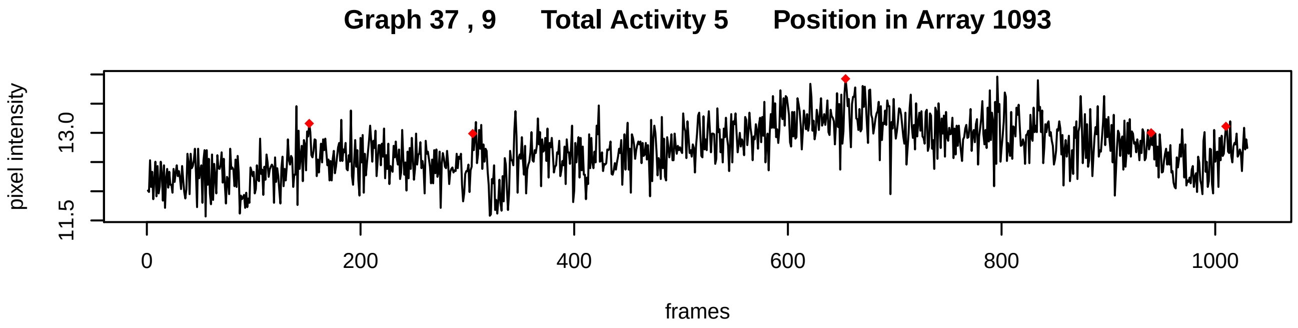
Graph 37 , 9 Total Activity 5 Position in Array 1093
pixel intensity
11.5
13.0
0
200
400
600
800
1000
frames

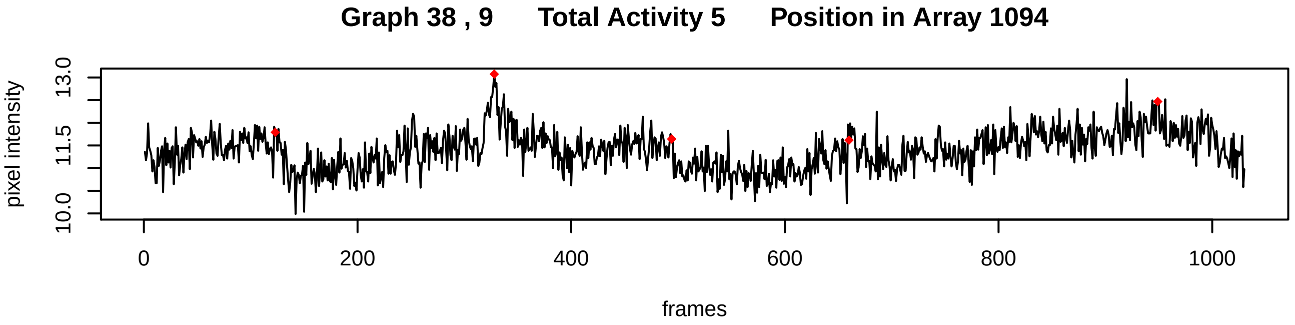
Graph 38 , 9 Total Activity 5 Position in Array 1094
pixel intensity
10.0
11.5
13.0
0
200
400
600
800
1000
frames

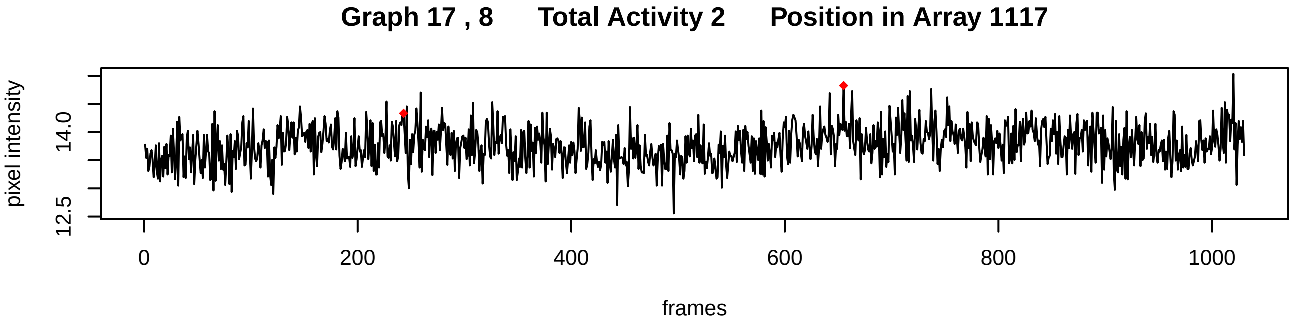
Graph 17 , 8 Total Activity 2 Position in Array 1117
pixel intensity
12.5
14.0
0
200
400
600
800
1000
frames

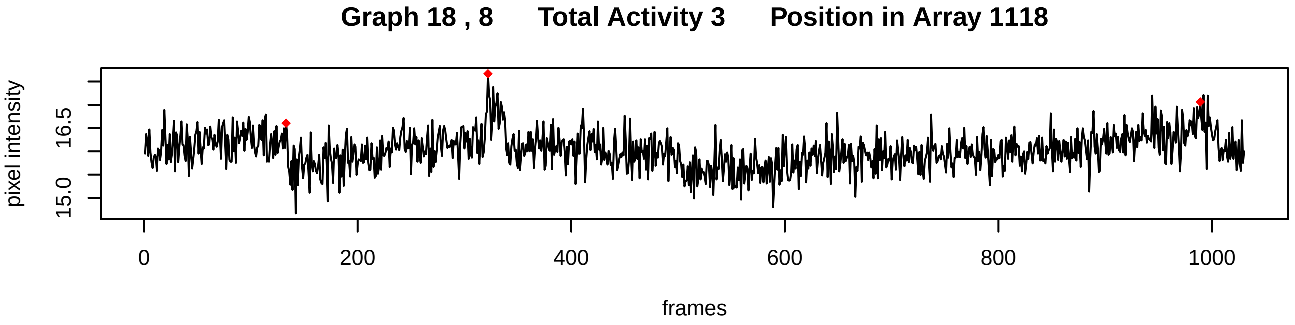
Graph 18 , 8 Total Activity 3 Position in Array 1118
pixel intensity
15.0
16.5
0
200
400
600
800
1000
frames

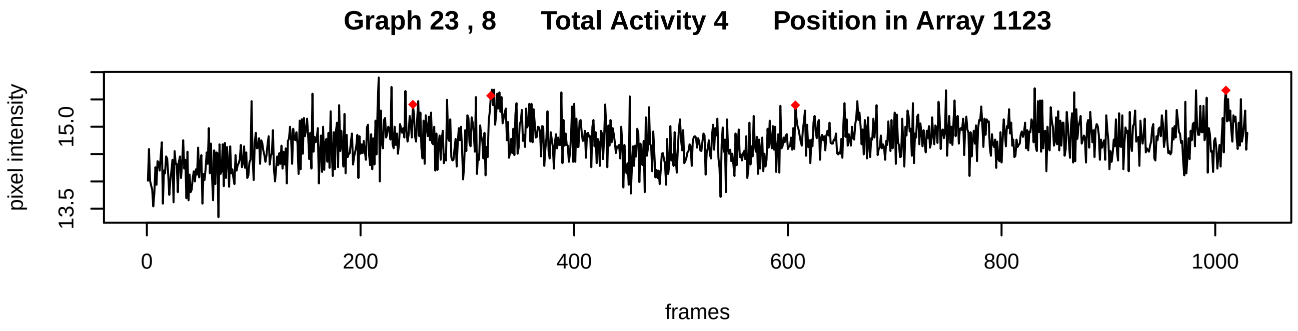
Graph 23 , 8 Total Activity 4 Position in Array 1123
pixel intensity
13.5
15.0
0
200
400
600
800
1000
frames

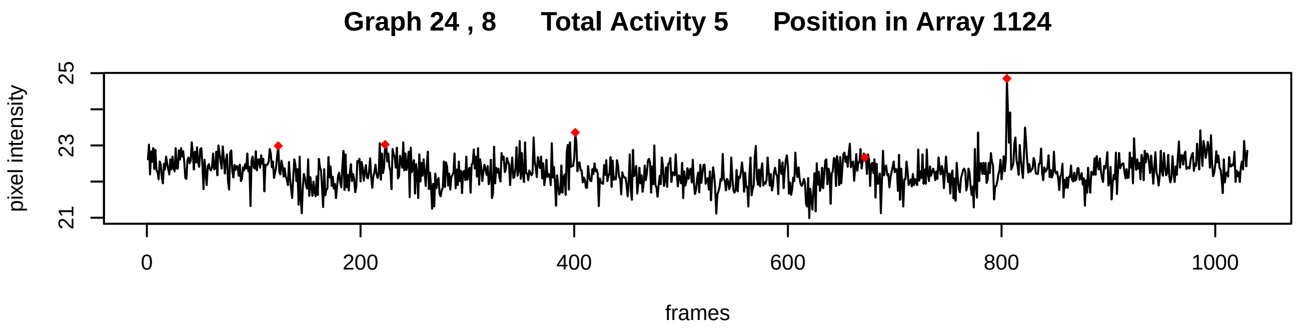
Graph 24 , 8 Total Activity 5 Position in Array 1124
pixel intensity
21
23
25
0
200
400
600
800
1000
frames

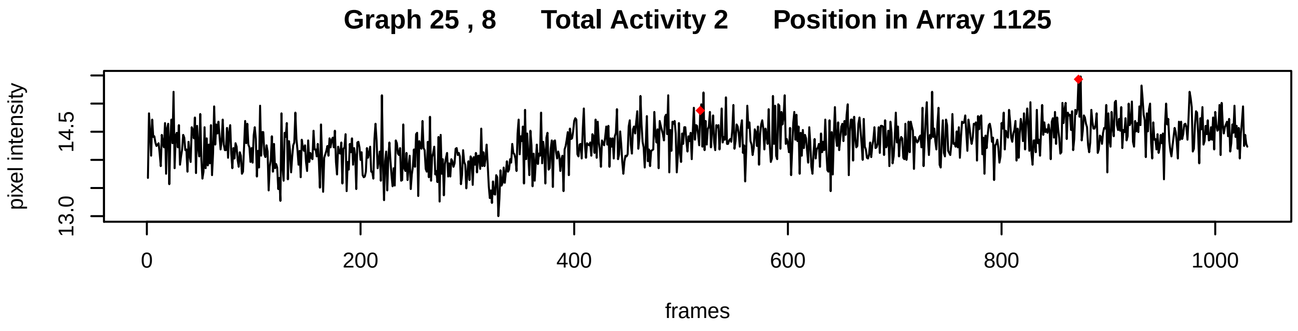
Graph 25 , 8 Total Activity 2 Position in Array 1125
pixel intensity
13.0
14.5
0
200
400
600
800
1000
frames

Graph 26 , 8   Total Activity 4   Position in Array 1126

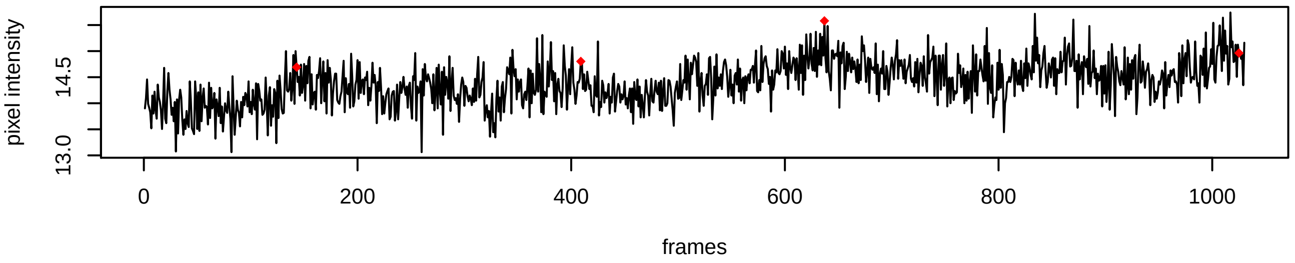

Graph 27 , 8   Total Activity 3   Position in Array 1127

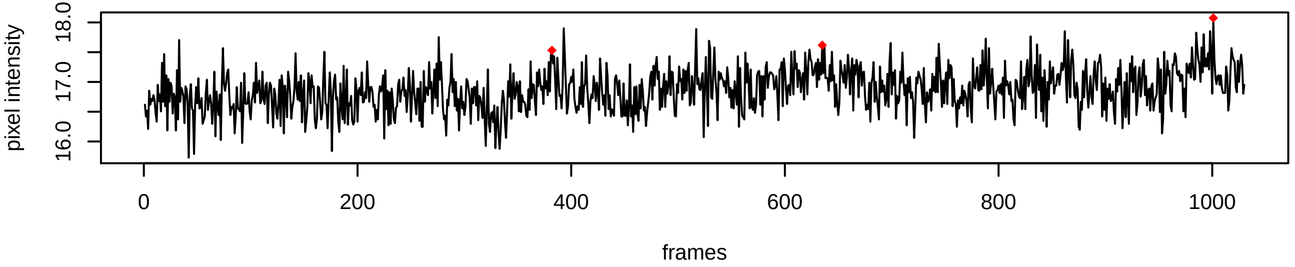

Graph 28 , 8   Total Activity 3   Position in Array 1128

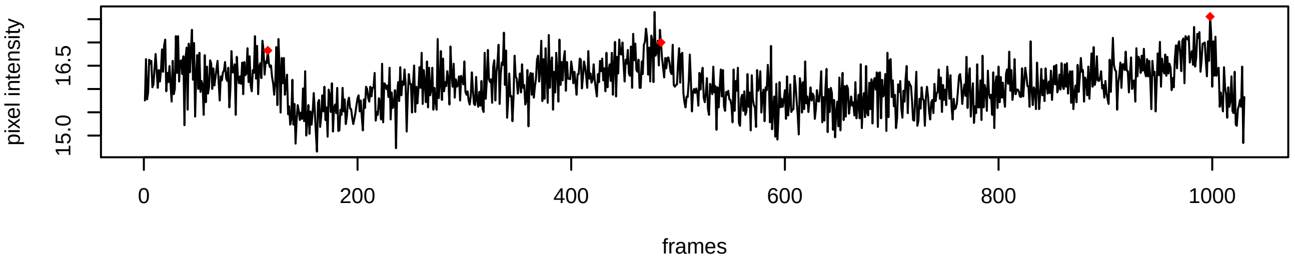

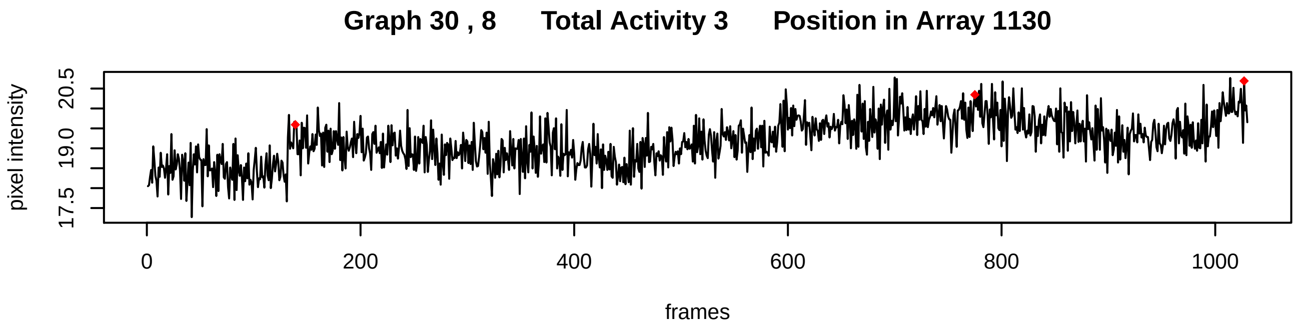
Graph 30 , 8   Total Activity 3   Position in Array 1130
pixel intensity
17.5
19.0
20.5
0
200
400
600
800
1000
frames

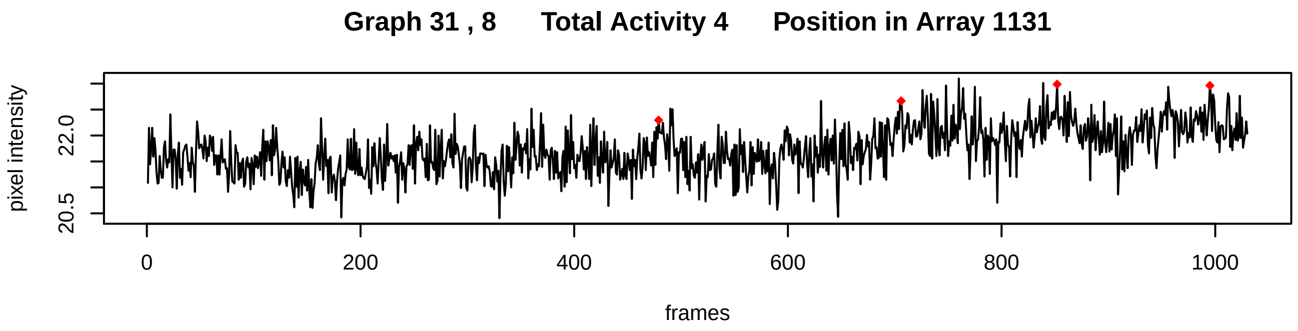
Graph 31 , 8   Total Activity 4   Position in Array 1131
pixel intensity
20.5
22.0
0
200
400
600
800
1000
frames

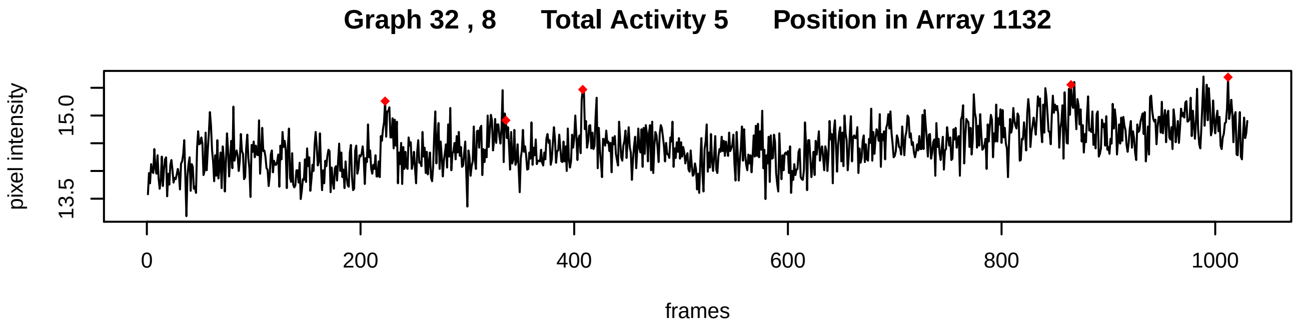
Graph 32 , 8   Total Activity 5   Position in Array 1132
pixel intensity
13.5
15.0
0
200
400
600
800
1000
frames

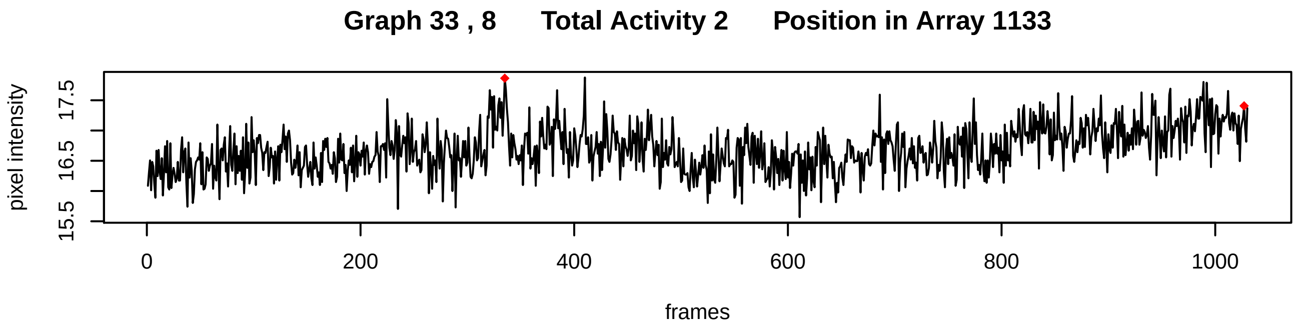
Graph 33 , 8 Total Activity 2 Position in Array 1133
pixel intensity
15.5
16.5
17.5
0
200
400
600
800
1000
frames

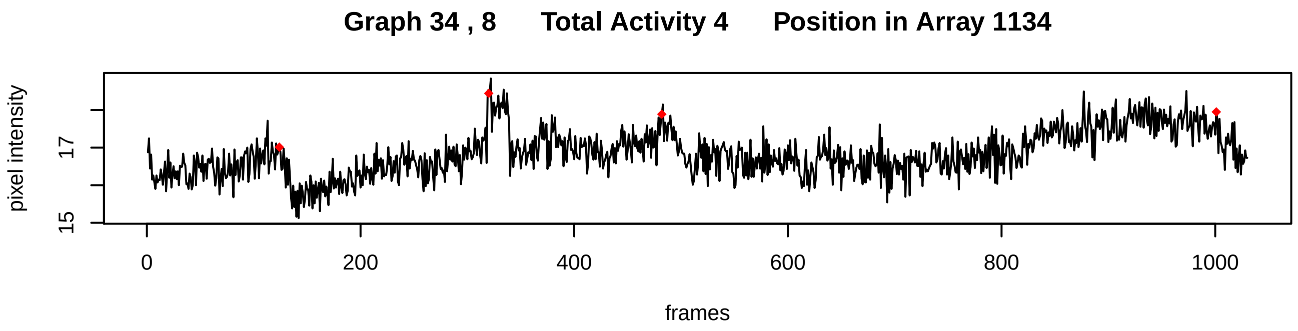
Graph 34 , 8 Total Activity 4 Position in Array 1134
pixel intensity
15
17
0
200
400
600
800
1000
frames

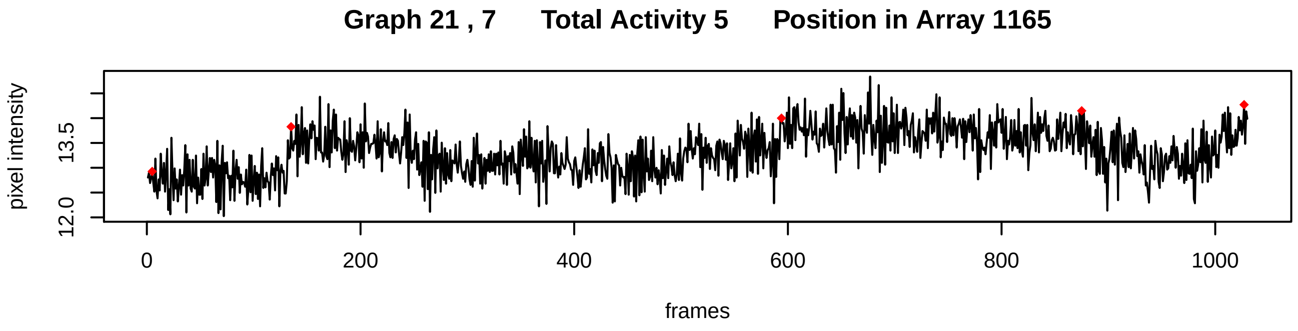
Graph 21 , 7 Total Activity 5 Position in Array 1165
pixel intensity
12.0
13.5
0
200
400
600
800
1000
frames

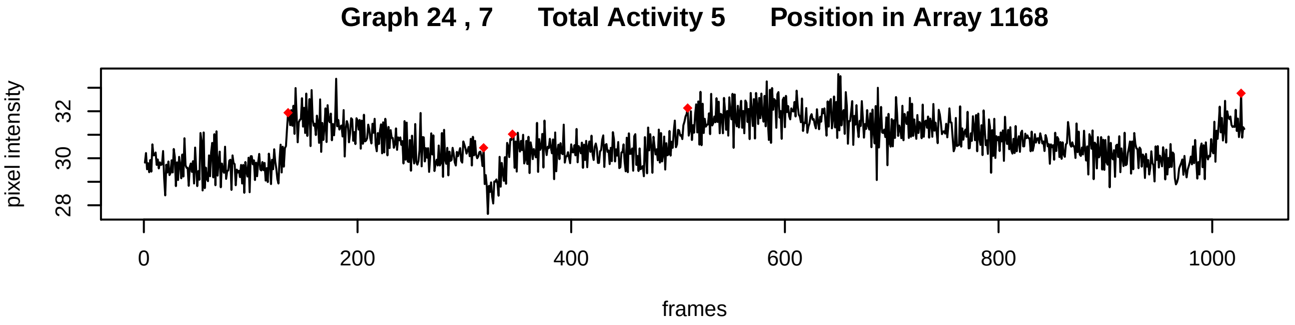
Graph 24 , 7 Total Activity 5 Position in Array 1168
pixel intensity
28
30
32
0
200
400
600
800
1000
frames

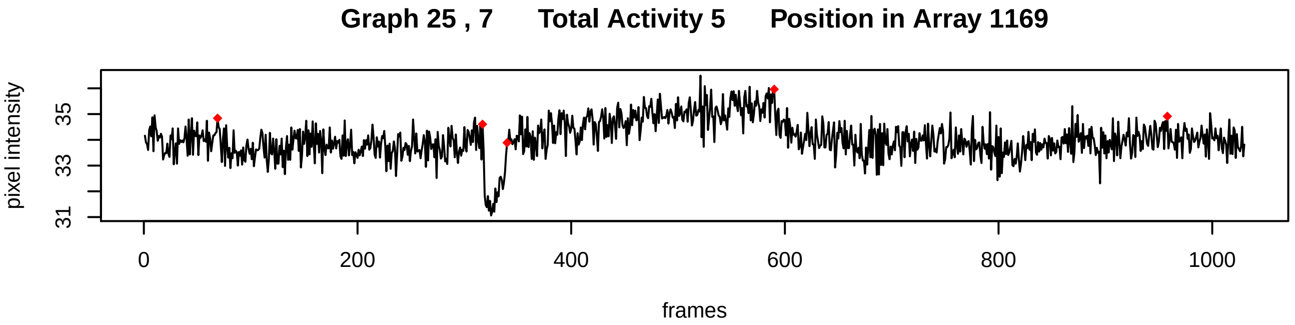
Graph 25 , 7 Total Activity 5 Position in Array 1169
pixel intensity
31
33
35
0
200
400
600
800
1000
frames

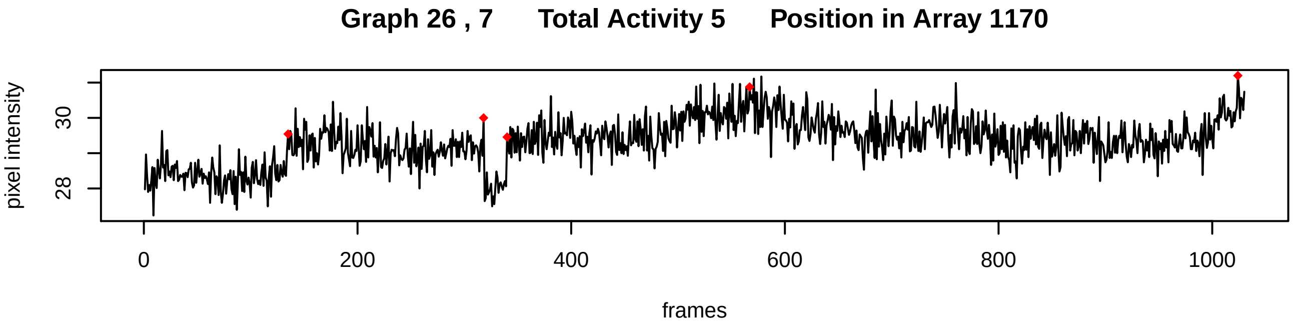
Graph 26 , 7 Total Activity 5 Position in Array 1170
pixel intensity
28
30
0
200
400
600
800
1000
frames

Graph 27 , 7 Total Activity 4 Position in Array 1171

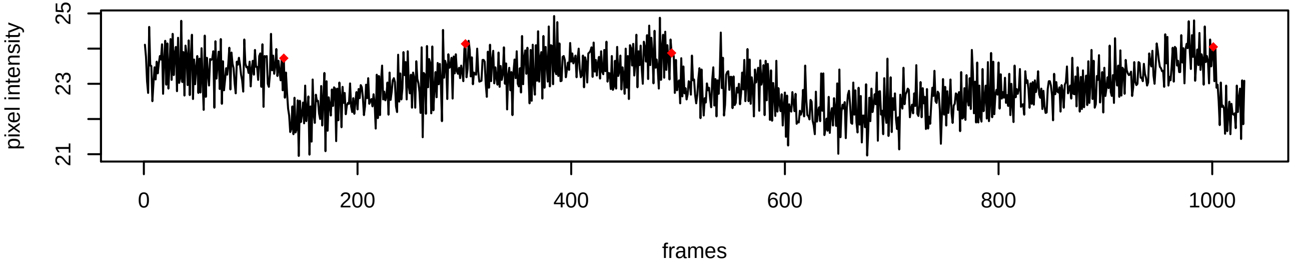

Graph 29 , 7 Total Activity 2 Position in Array 1173

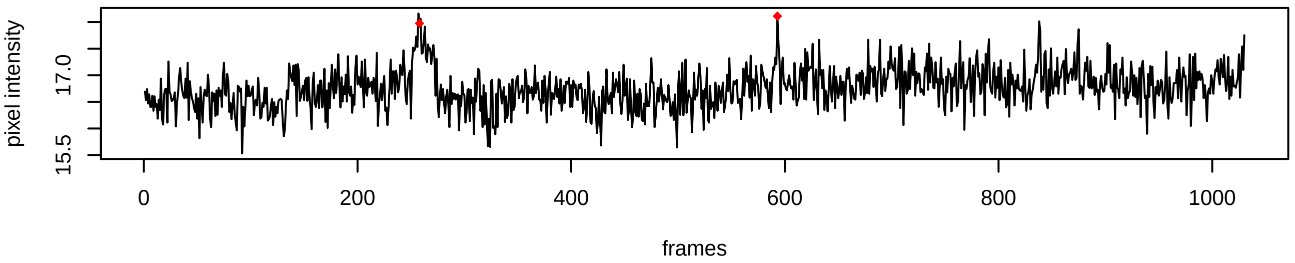

Graph 30 , 7 Total Activity 4 Position in Array 1174

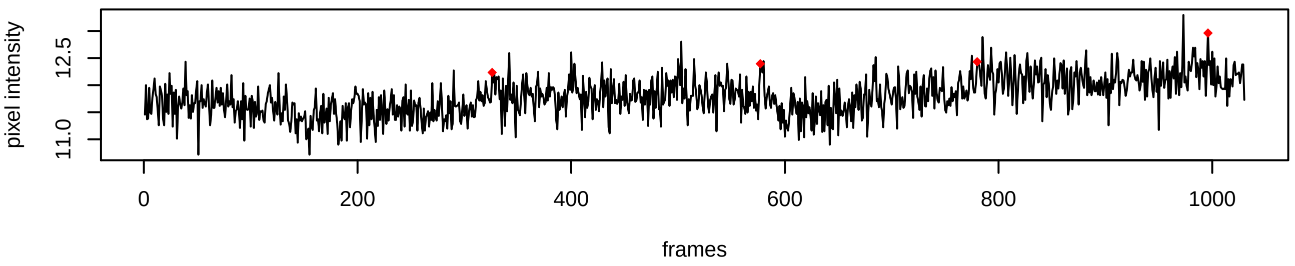

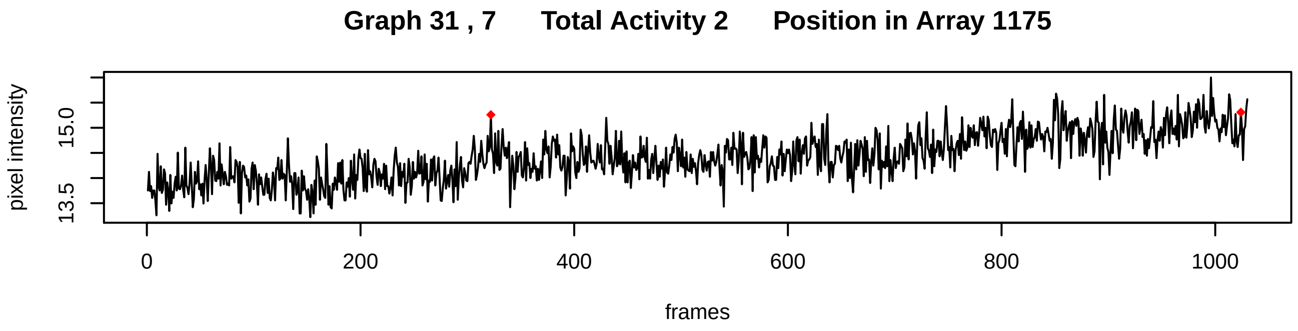
Graph 31 , 7 Total Activity 2 Position in Array 1175
pixel intensity
13.5
15.0
0
200
400
600
800
1000
frames

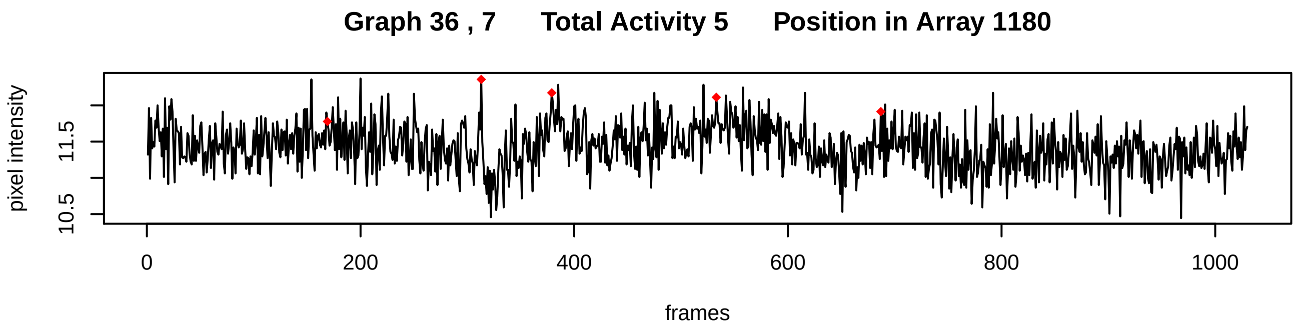
Graph 36 , 7 Total Activity 5 Position in Array 1180
pixel intensity
10.5
11.5
0
200
400
600
800
1000
frames

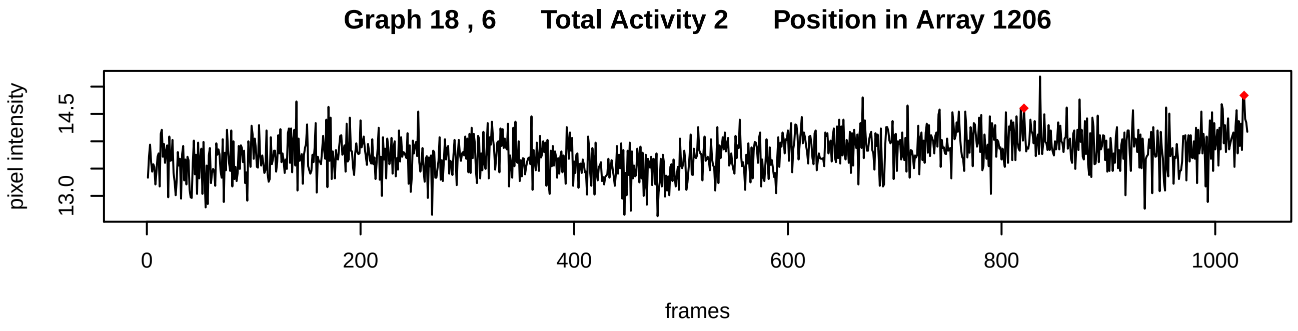
Graph 18 , 6 Total Activity 2 Position in Array 1206
pixel intensity
13.0
14.5
0
200
400
600
800
1000
frames

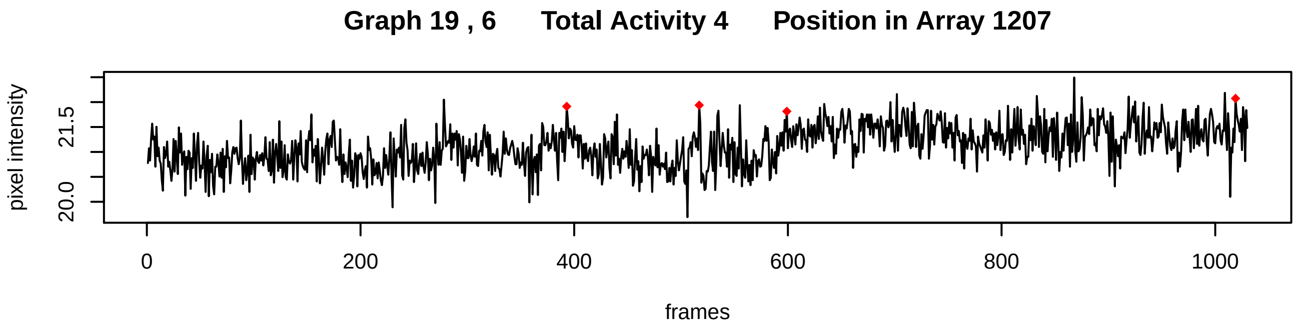
Graph 19 , 6 Total Activity 4 Position in Array 1207
pixel intensity
20.0
21.5
0
200
400
600
800
1000
frames

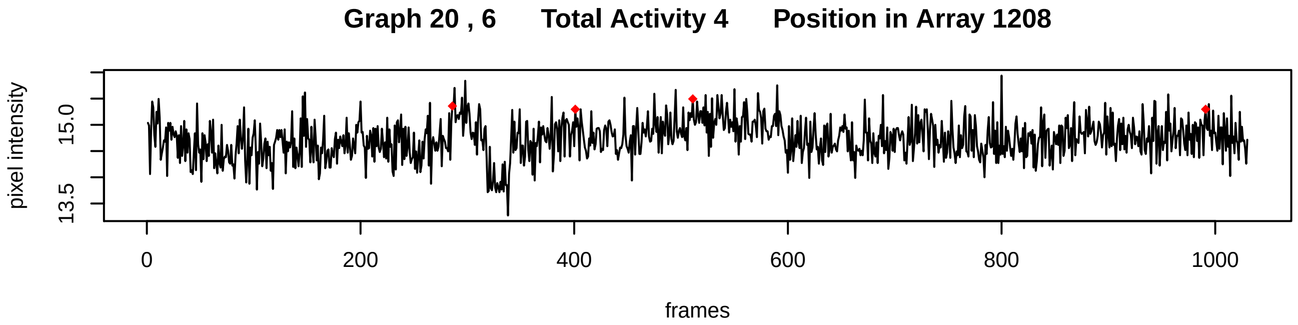
Graph 20 , 6 Total Activity 4 Position in Array 1208
pixel intensity
13.5
15.0
0
200
400
600
800
1000
frames

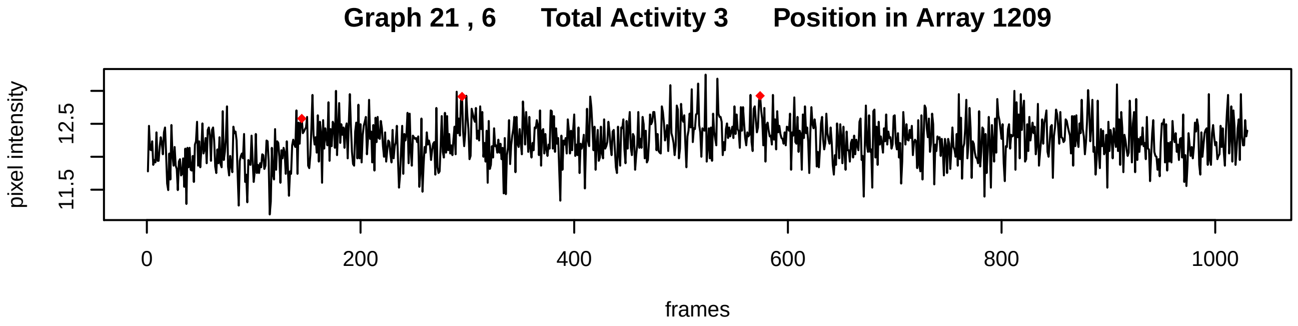
Graph 21 , 6 Total Activity 3 Position in Array 1209
pixel intensity
11.5
12.5
0
200
400
600
800
1000
frames

Graph 22 , 6   Total Activity 3   Position in Array 1210

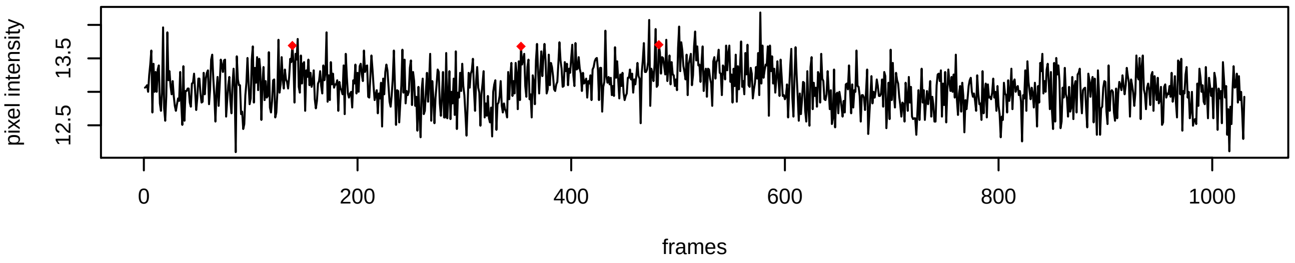

Graph 23 , 6   Total Activity 6   Position in Array 1211

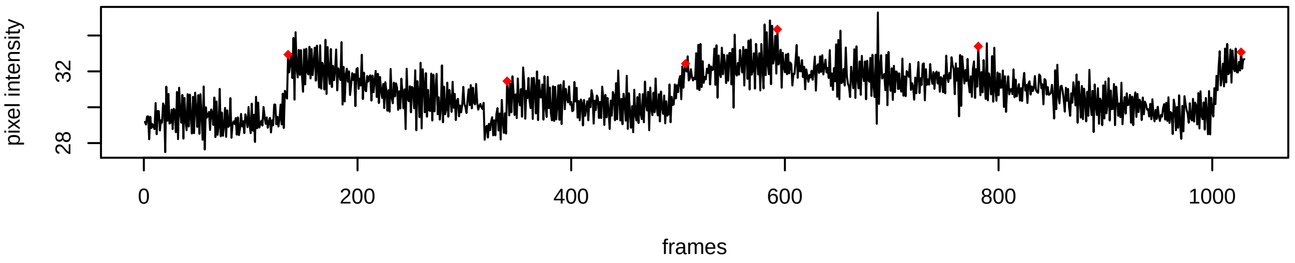

Graph 24 , 6   Total Activity 7   Position in Array 1212

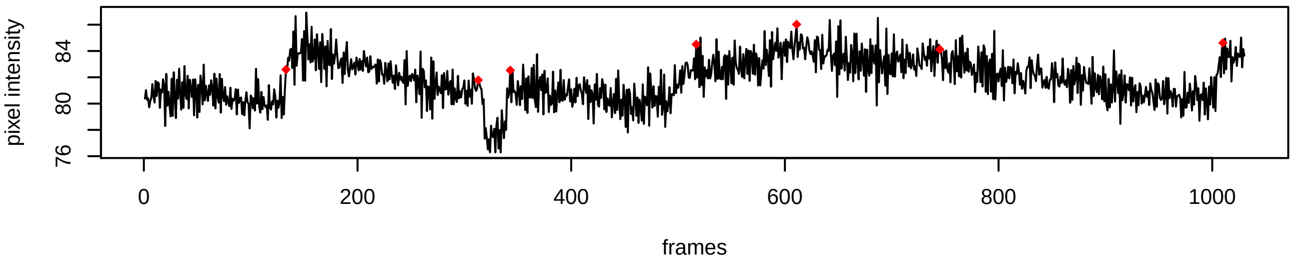

Graph 25 , 6   Total Activity 6   Position in Array 1213

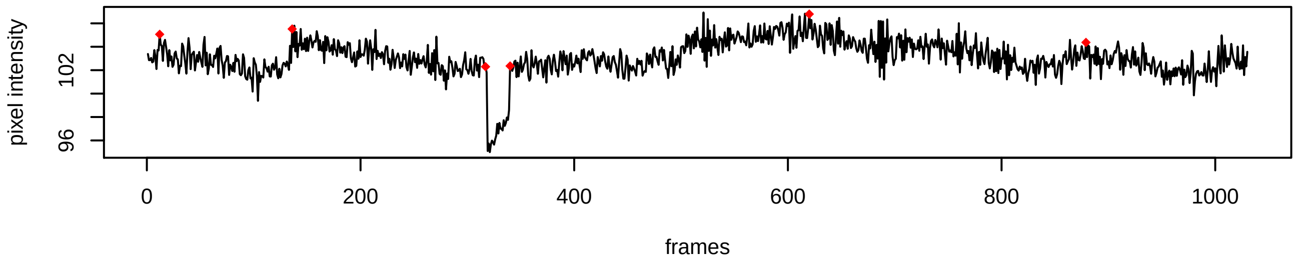

Graph 26 , 6   Total Activity 3   Position in Array 1214

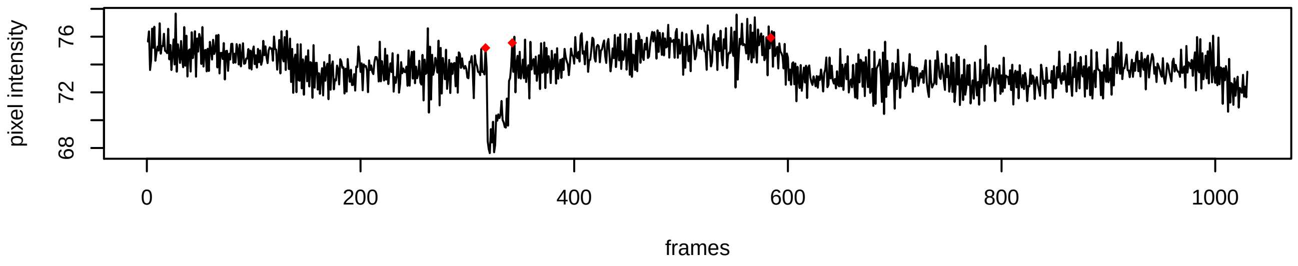

Graph 27 , 6   Total Activity 7   Position in Array 1215

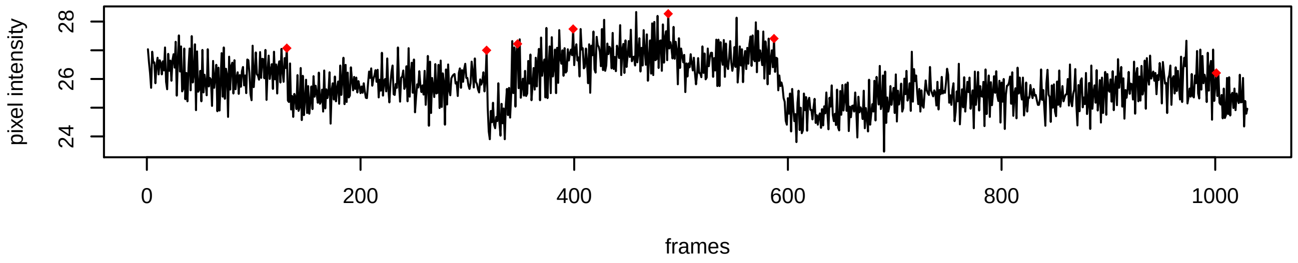

Graph 28 , 6 Total Activity 3 Position in Array 1216

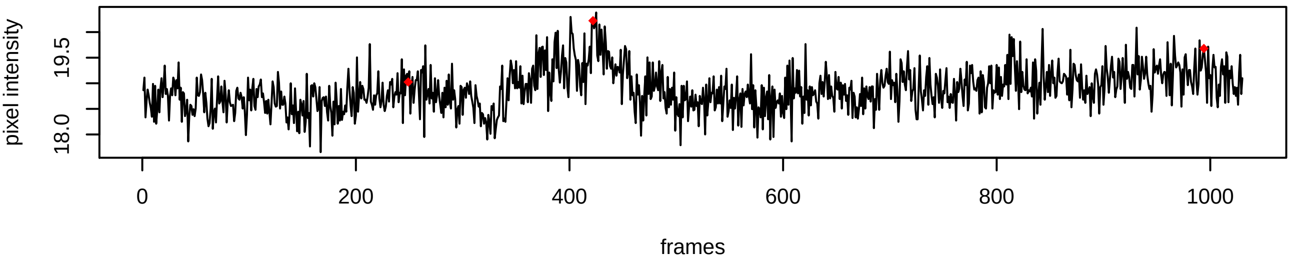

Graph 29 , 6 Total Activity 3 Position in Array 1217

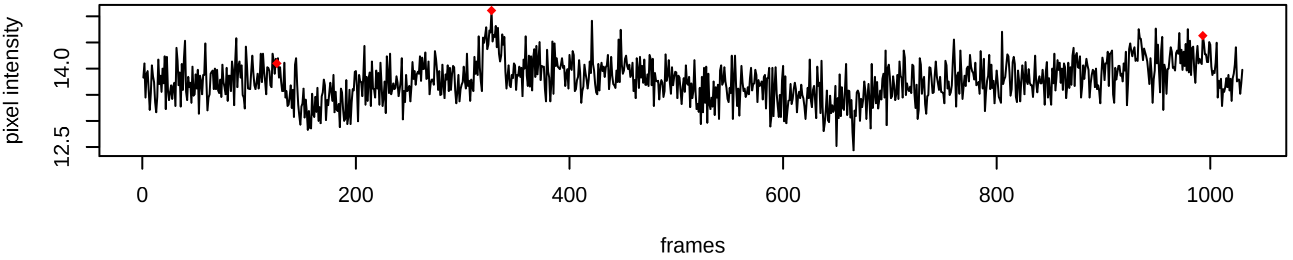

Graph 30 , 6 Total Activity 4 Position in Array 1218

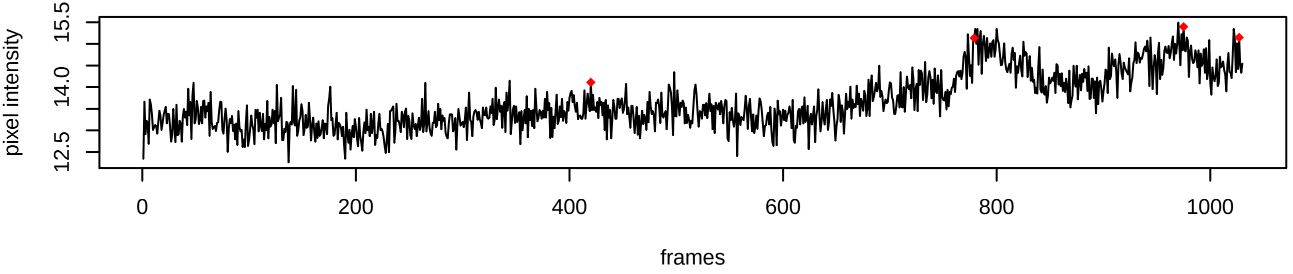

Graph 31 , 6 Total Activity 4 Position in Array 1219

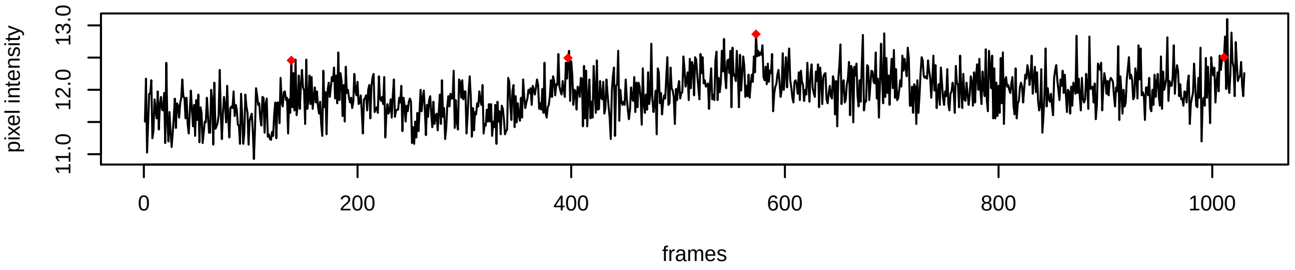

Graph 15 , 5 Total Activity 3 Position in Array 1247

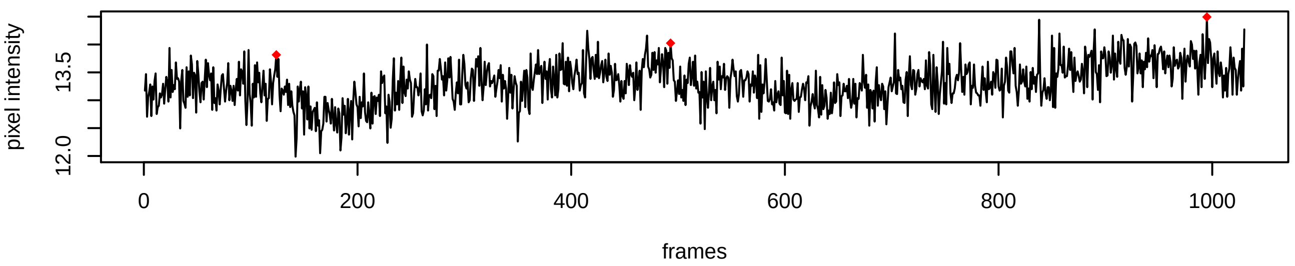

Graph 19 , 5 Total Activity 4 Position in Array 1251

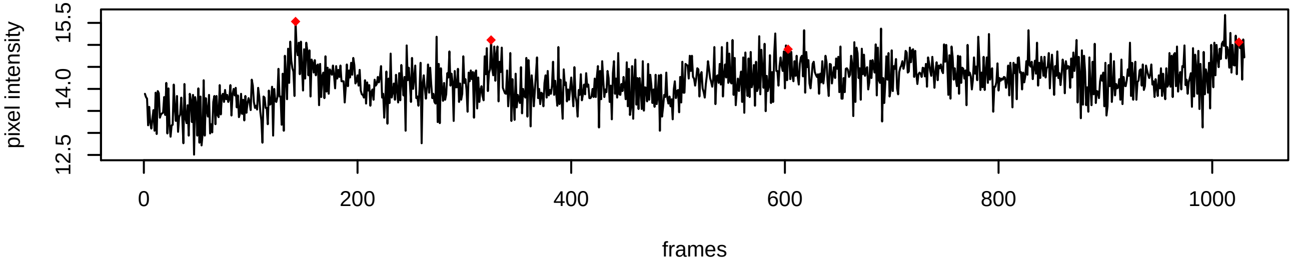

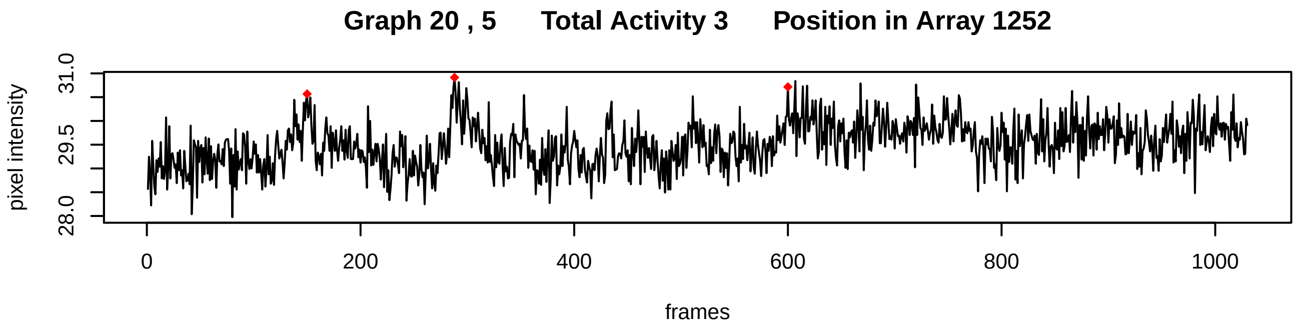
Graph 20 , 5 Total Activity 3 Position in Array 1252
pixel intensity
28.0
29.5
31.0
0
200
400
600
800
1000
frames

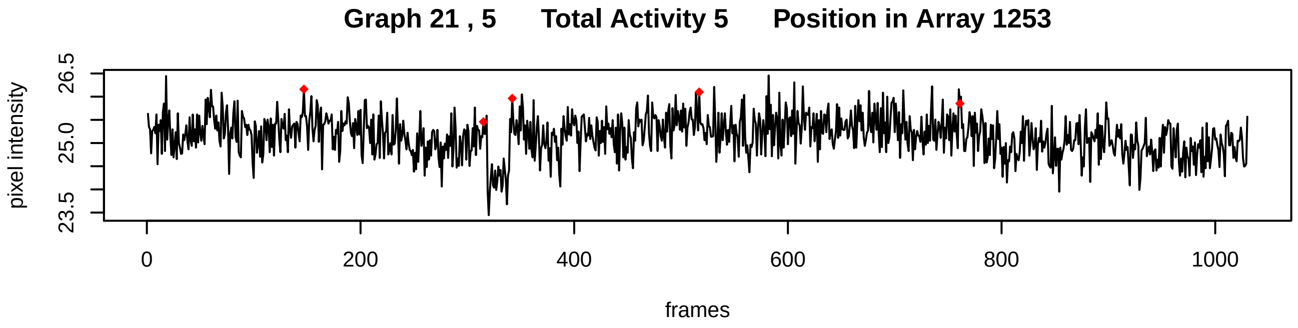
Graph 21 , 5 Total Activity 5 Position in Array 1253
pixel intensity
23.5
25.0
26.5
0
200
400
600
800
1000
frames

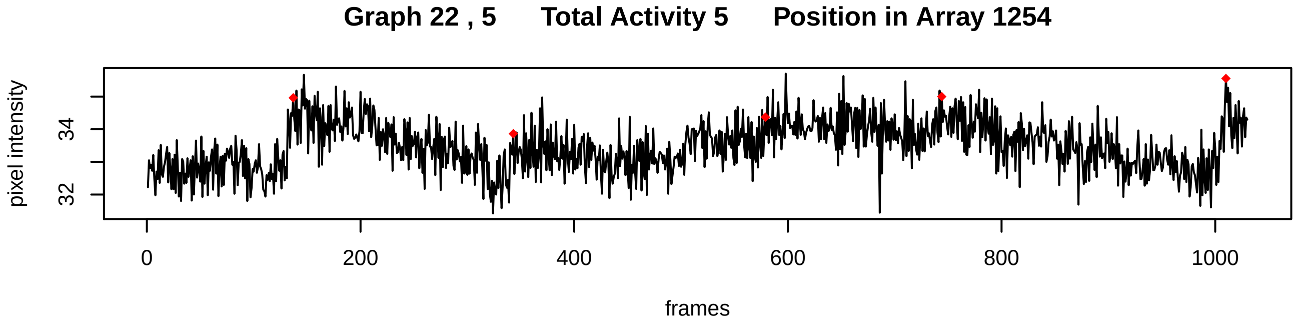
Graph 22 , 5 Total Activity 5 Position in Array 1254
pixel intensity
32
34
0
200
400
600
800
1000
frames

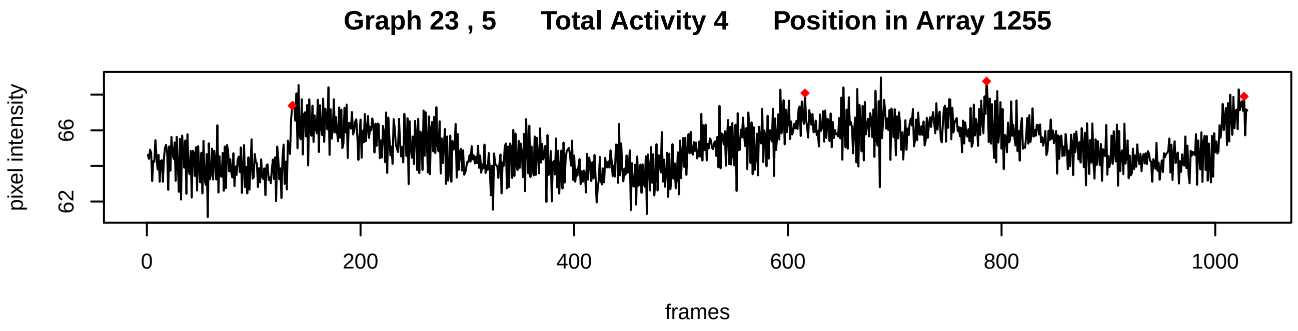
Graph 23 , 5 Total Activity 4 Position in Array 1255
pixel intensity
62
66
0
200
400
600
800
1000
frames

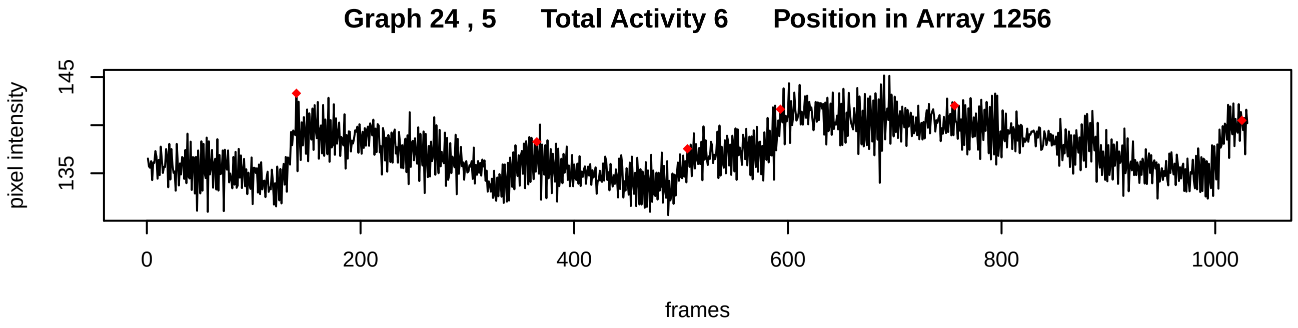
Graph 24 , 5 Total Activity 6 Position in Array 1256
pixel intensity
135
145
0
200
400
600
800
1000
frames

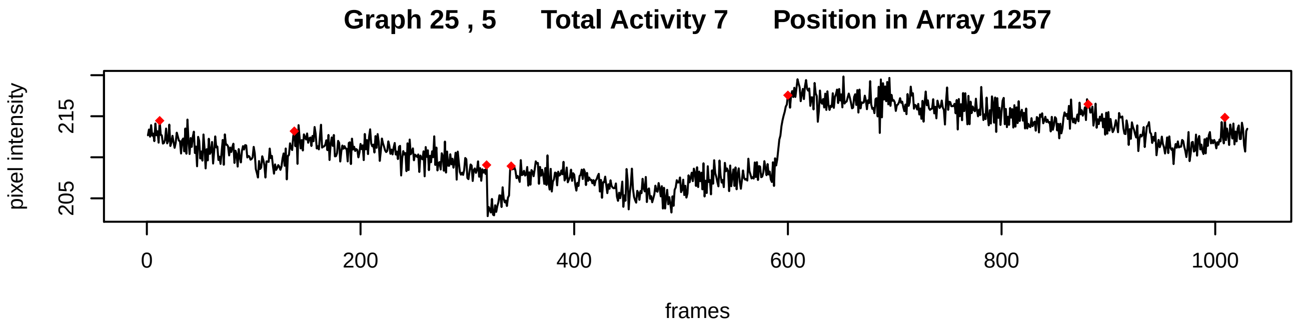
Graph 25 , 5 Total Activity 7 Position in Array 1257
pixel intensity
205
215
0
200
400
600
800
1000
frames

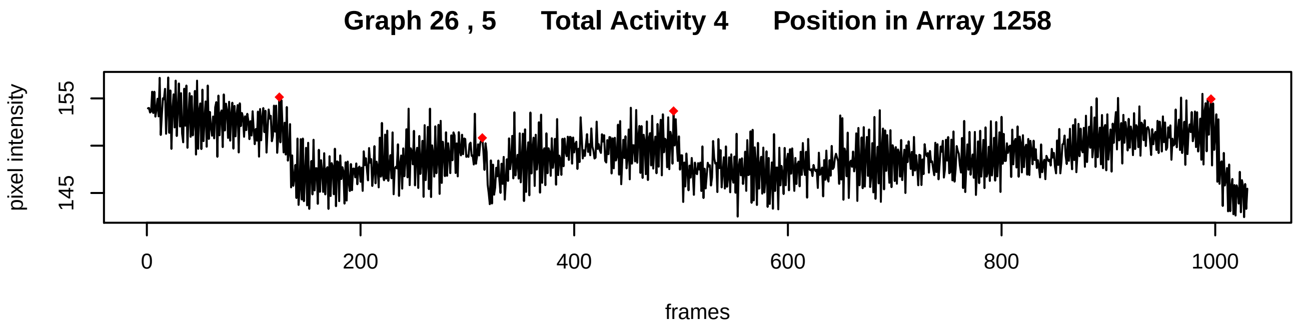
Graph 26 , 5 Total Activity 4 Position in Array 1258
pixel intensity
145
155
0
200
400
600
800
1000
frames

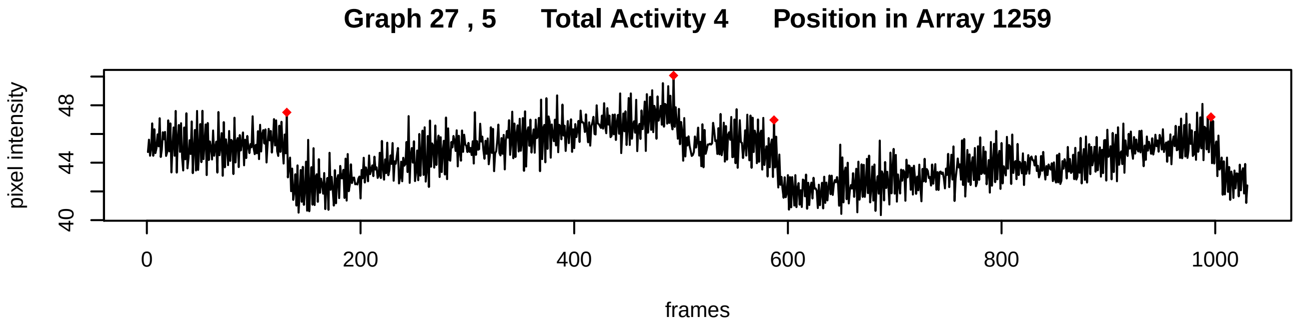
Graph 27 , 5 Total Activity 4 Position in Array 1259
pixel intensity
40
44
48
0
200
400
600
800
1000
frames

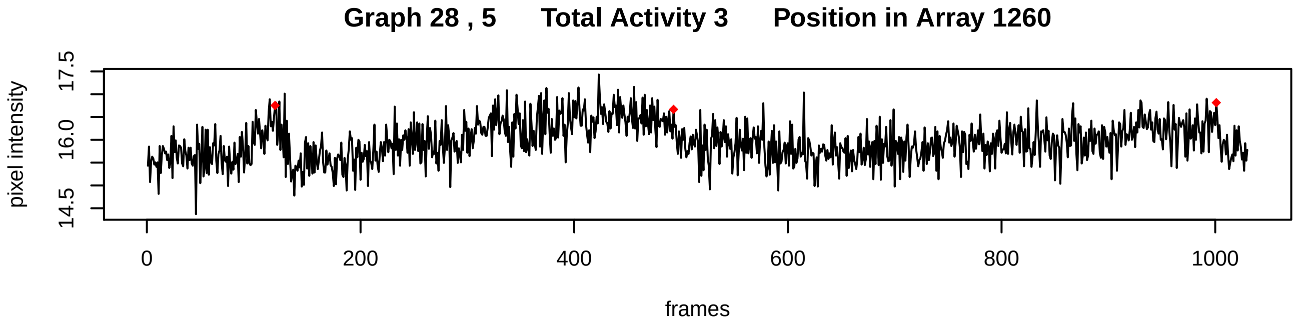
Graph 28 , 5 Total Activity 3 Position in Array 1260
pixel intensity
14.5
16.0
17.5
0
200
400
600
800
1000
frames

Graph 29 , 5 Total Activity 5 Position in Array 1261

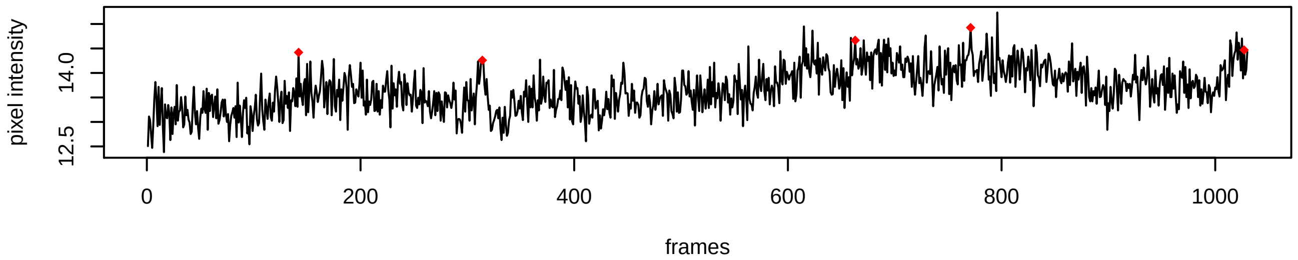

Graph 30 , 5 Total Activity 5 Position in Array 1262

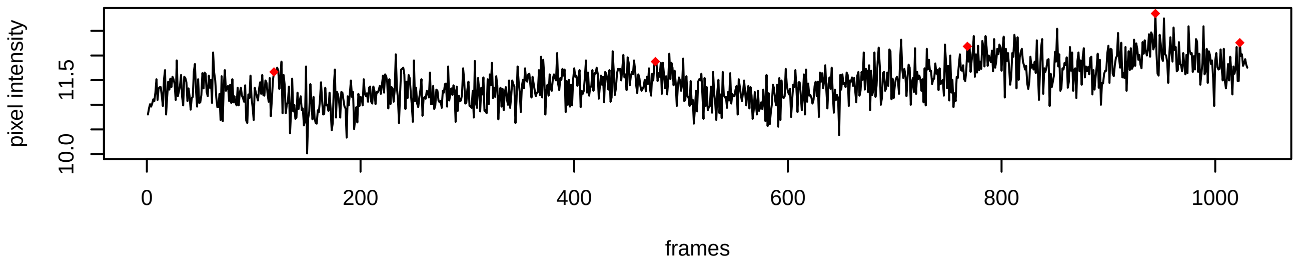

Graph 32 , 5 Total Activity 3 Position in Array 1264

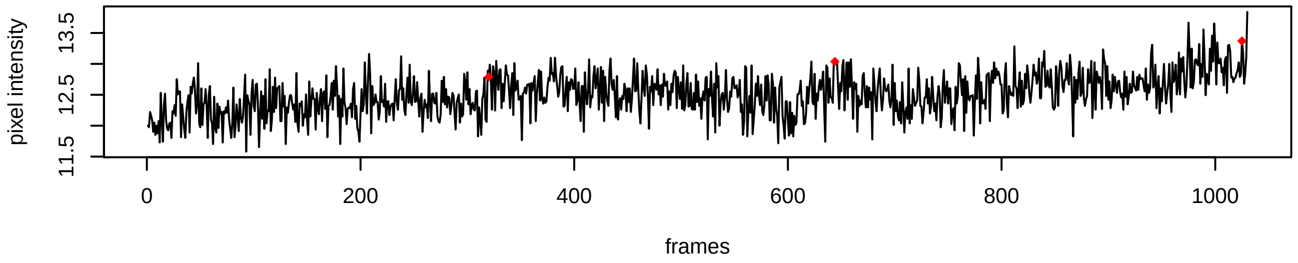

Graph 36 , 5   Total Activity 4   Position in Array 1268

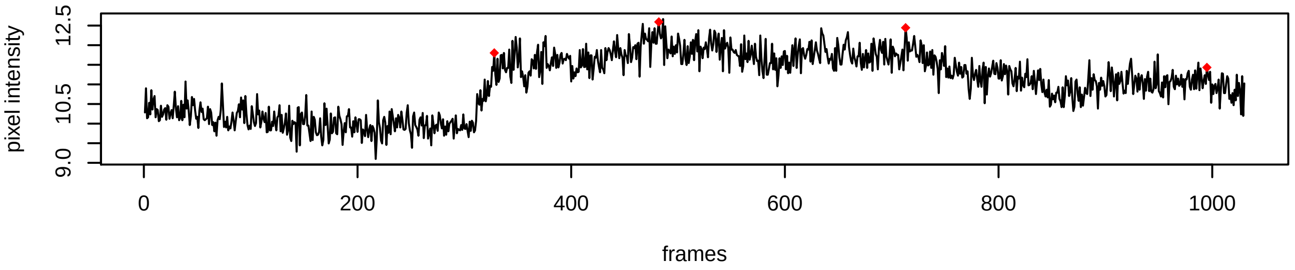

Graph 19 , 4   Total Activity 5   Position in Array 1295

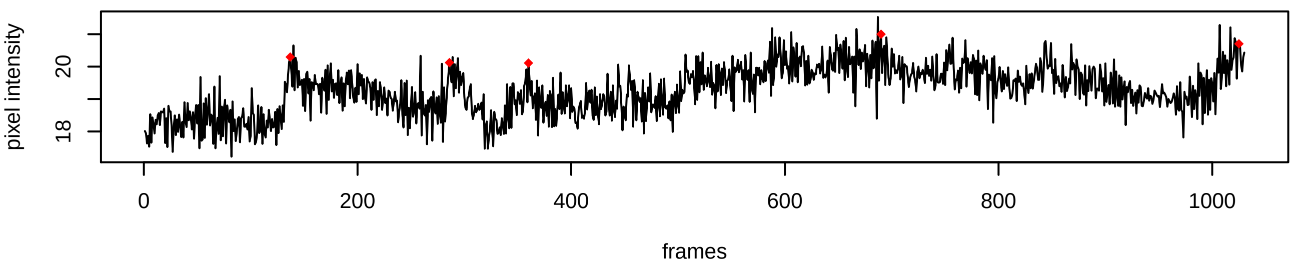

Graph 20 , 4   Total Activity 6   Position in Array 1296

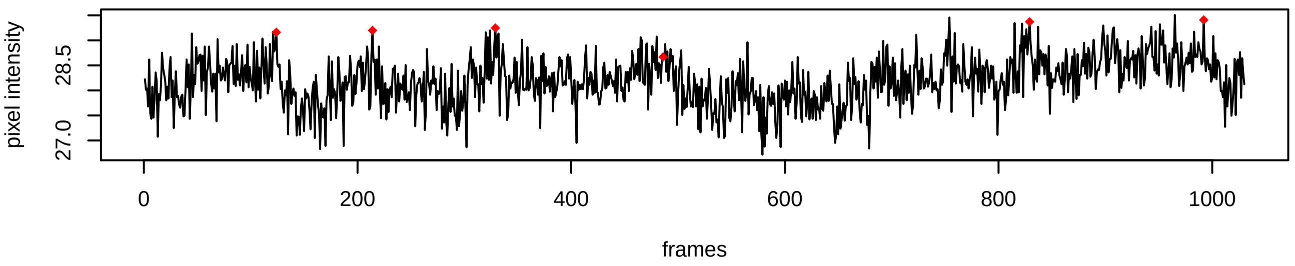

Graph 21 , 4 Total Activity 5 Position in Array 1297

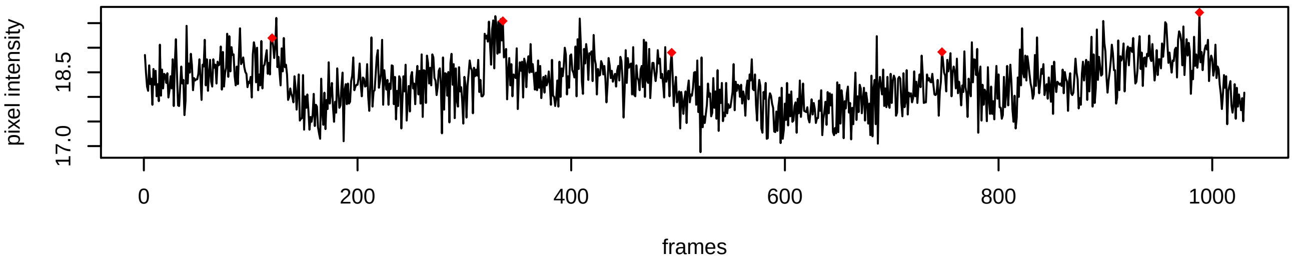

Graph 22 , 4 Total Activity 2 Position in Array 1298

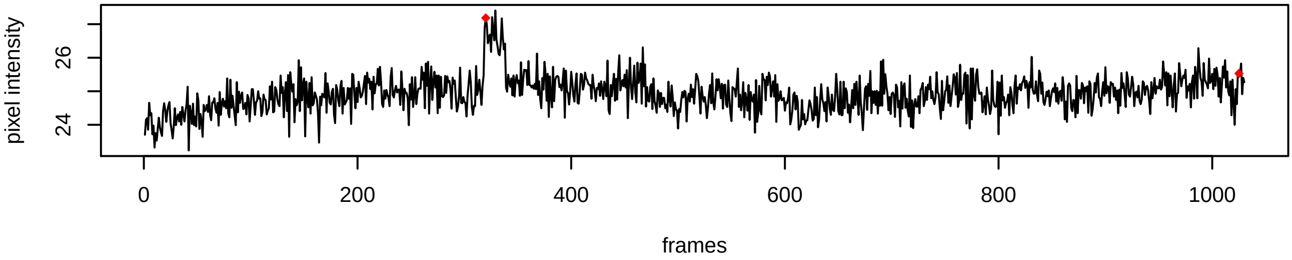

Graph 23 , 4 Total Activity 4 Position in Array 1299

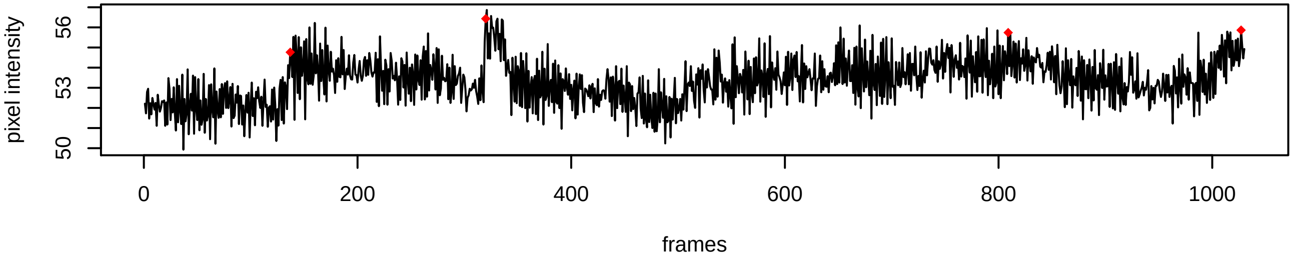

Graph 24 , 4   Total Activity 5   Position in Array 1300

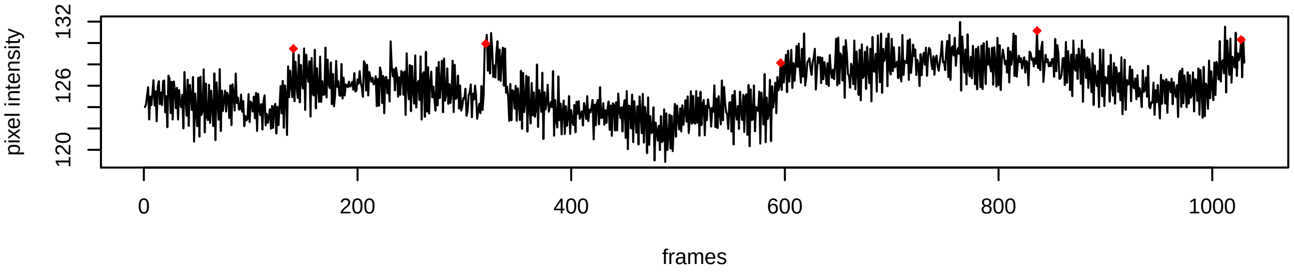

Graph 25 , 4   Total Activity 7   Position in Array 1301

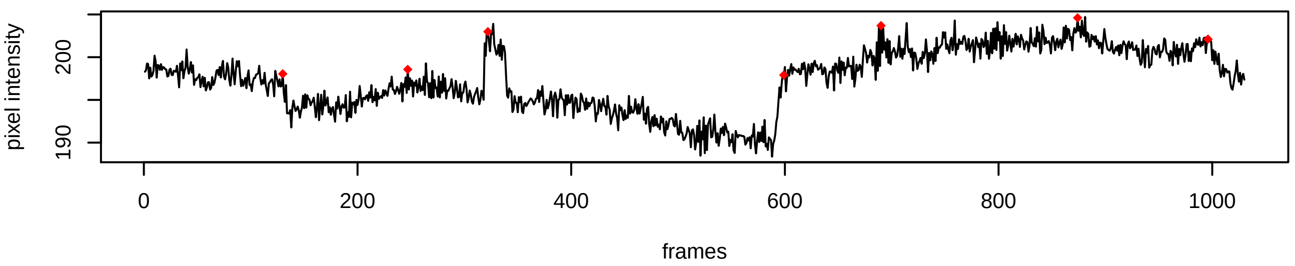

Graph 26 , 4   Total Activity 4   Position in Array 1302

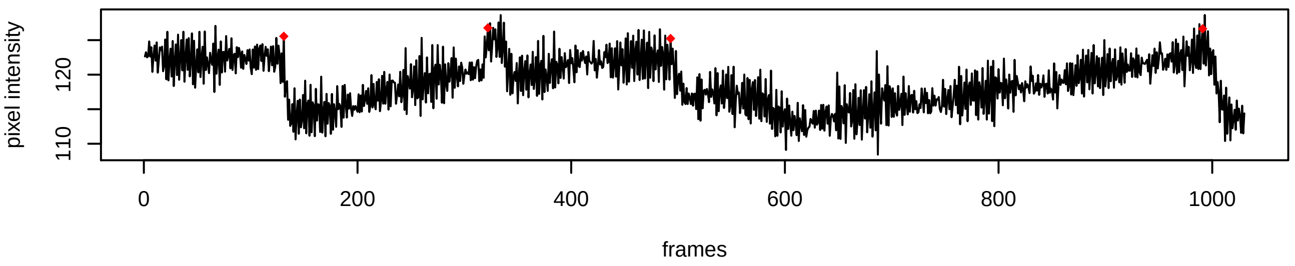

Graph 27 , 4   Total Activity 6   Position in Array 1303

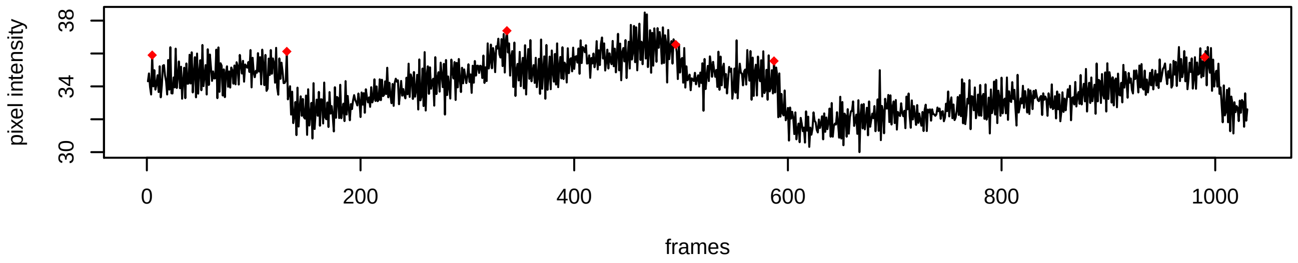

Graph 28 , 4   Total Activity 2   Position in Array 1304

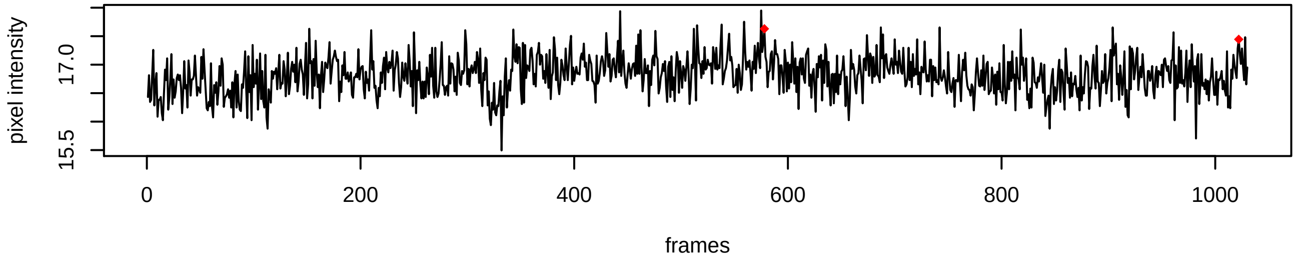

Graph 29 , 4   Total Activity 2   Position in Array 1305

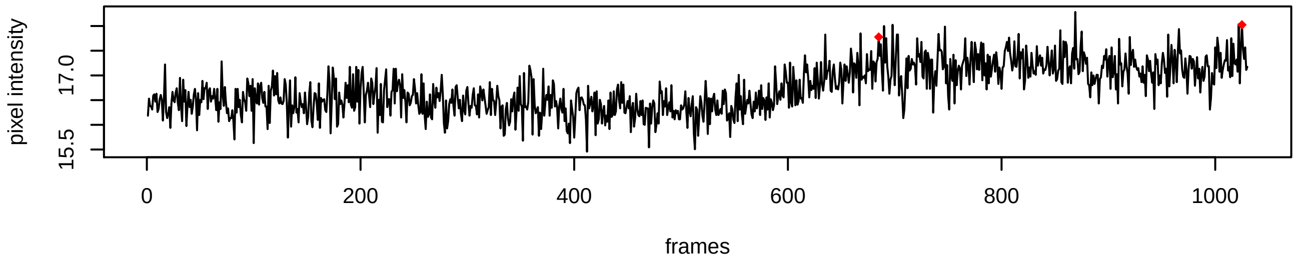

Graph 19 , 3 Total Activity 11 Position in Array 1339

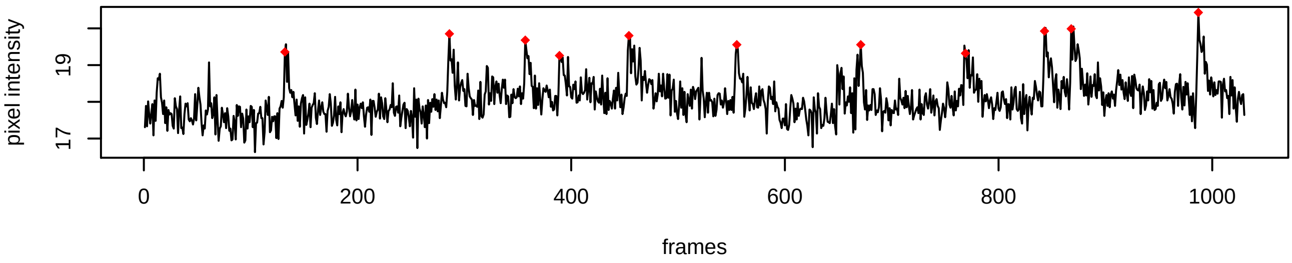

Graph 20 , 3 Total Activity 5 Position in Array 1340

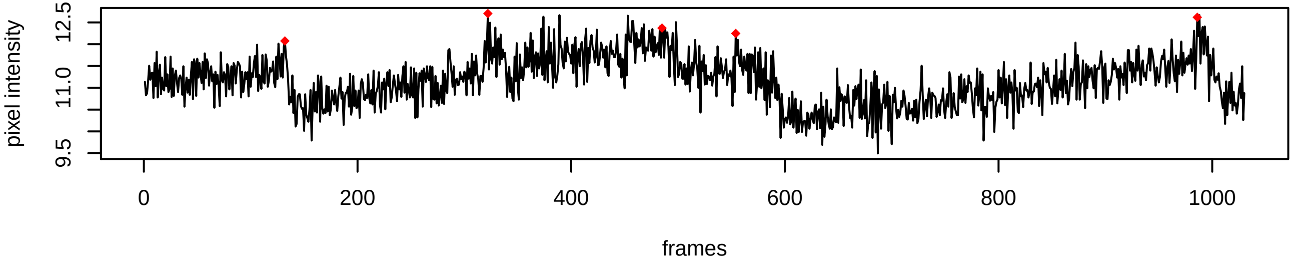

Graph 23 , 3 Total Activity 5 Position in Array 1343

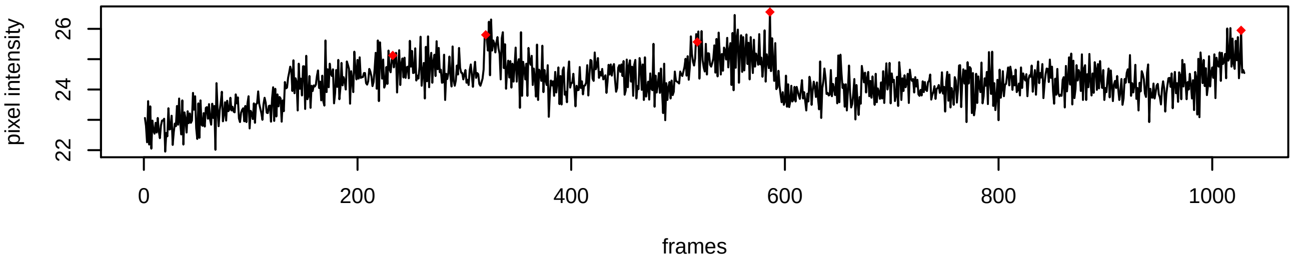

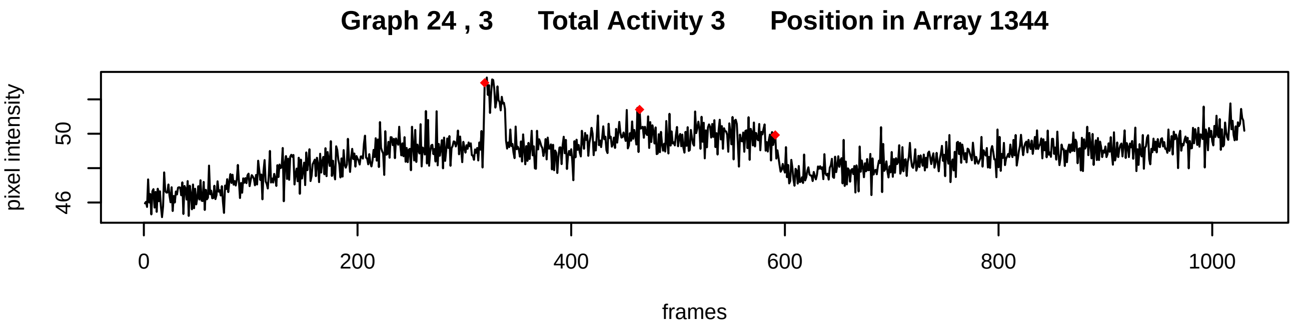
Graph 24 , 3 Total Activity 3 Position in Array 1344
pixel intensity
46
50
0
200
400
600
800
1000
frames

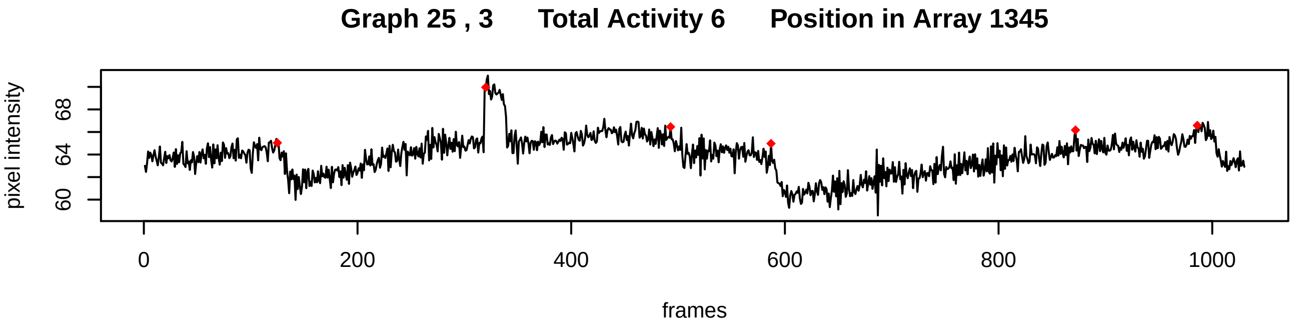
Graph 25 , 3 Total Activity 6 Position in Array 1345
pixel intensity
60
64
68
0
200
400
600
800
1000
frames

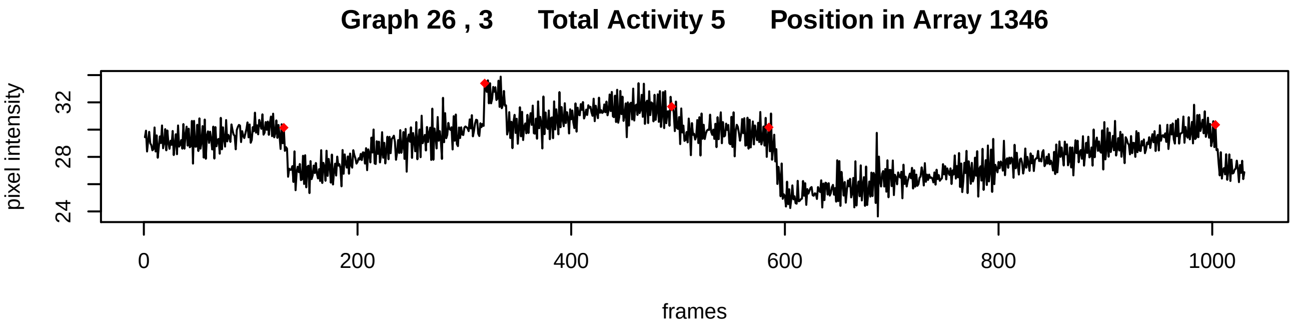
Graph 26 , 3 Total Activity 5 Position in Array 1346
pixel intensity
24
28
32
0
200
400
600
800
1000
frames

Graph 27 , 3   Total Activity 5   Position in Array 1347

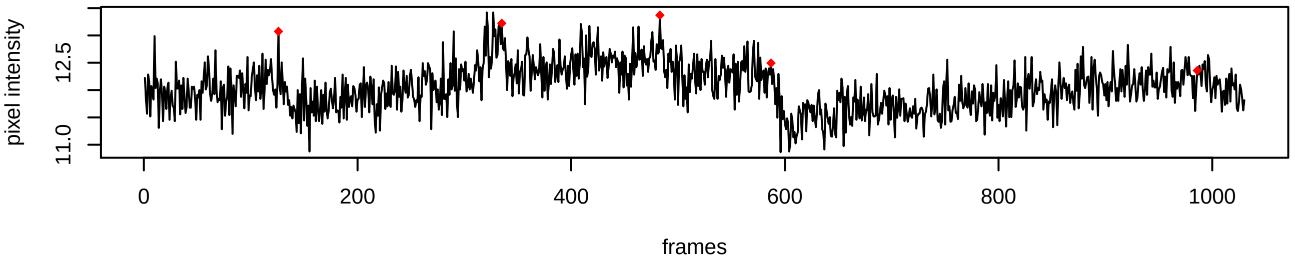

Graph 25 , 2   Total Activity 3   Position in Array 1389

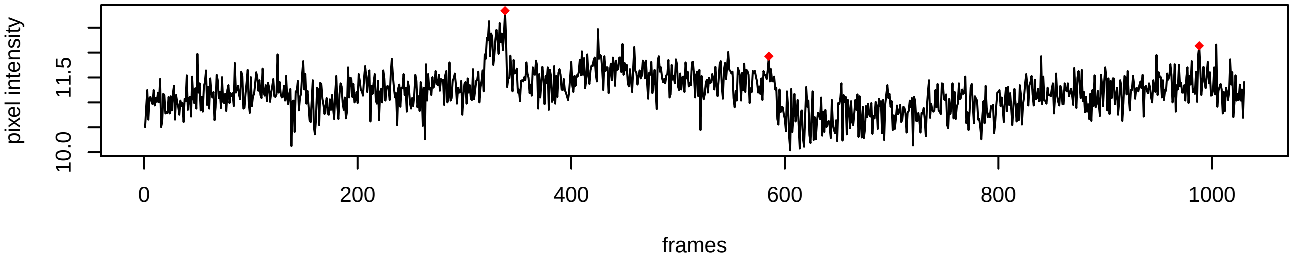

Supplement: S5 Fig — Computation of spontaneously active hippocampal neurons, traces and activity counts. (PDF) [file pcbi.1006054.s005.pdf]
