# Supplementary material for: An open source tool for automatic spatiotemporal assessment of calcium transients and local ‘signal-close-to-noise’ activity in calcium imaging data
Source: PLoS Comput Biol. 2018 Mar 30;14(3):e1006054. doi: 10.1371/journal.pcbi.1006054 (PMC5895056; doi:10.1371/journal.pcbi.1006054)

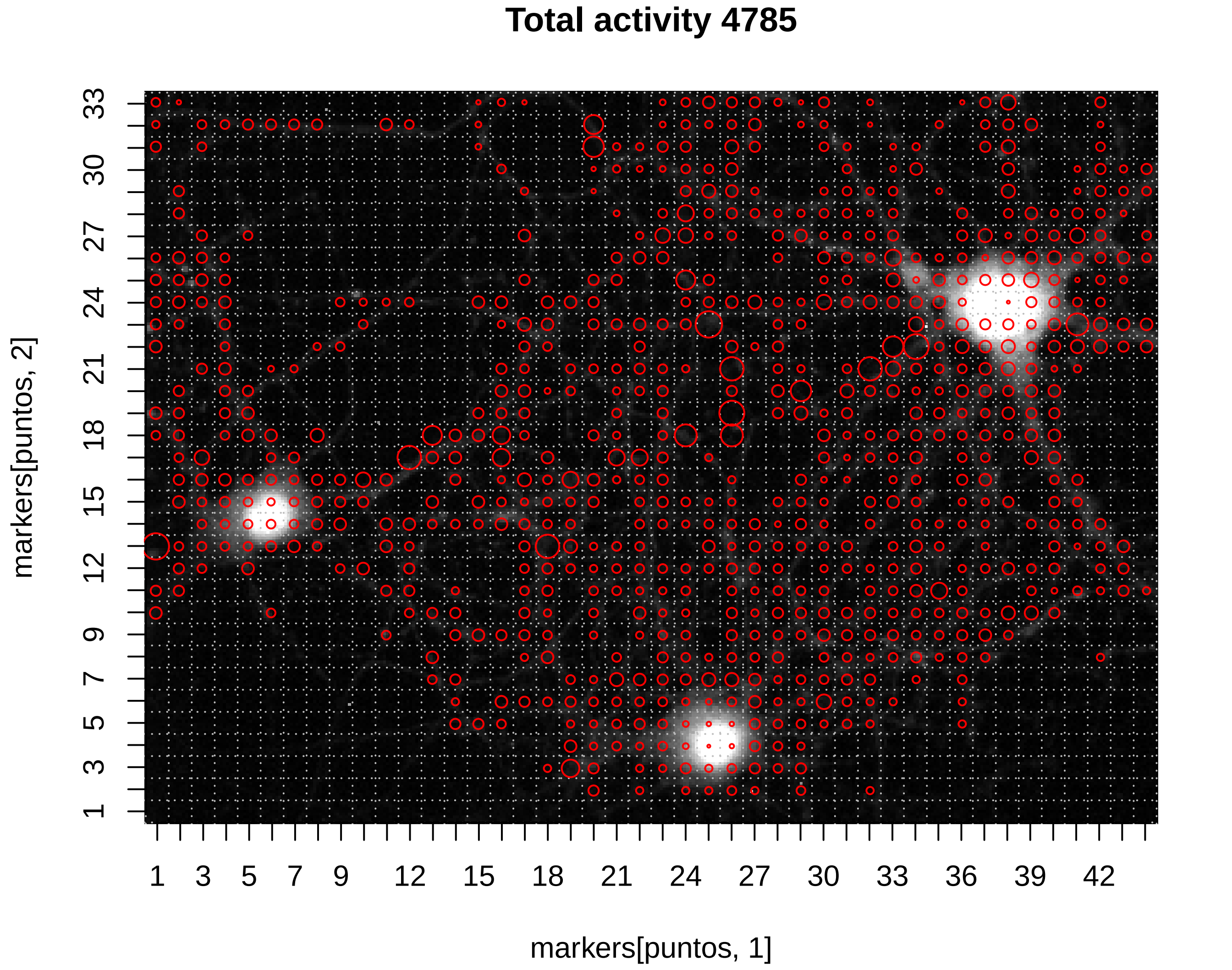
Total activity 4785
markers[puntos, 2]
markers[puntos, 1]
1
3
5
7
9
12
15
18
21
24
27
30
33
36
39
42

Graph 1 , 33 Total Activity 6 Position in Array 1

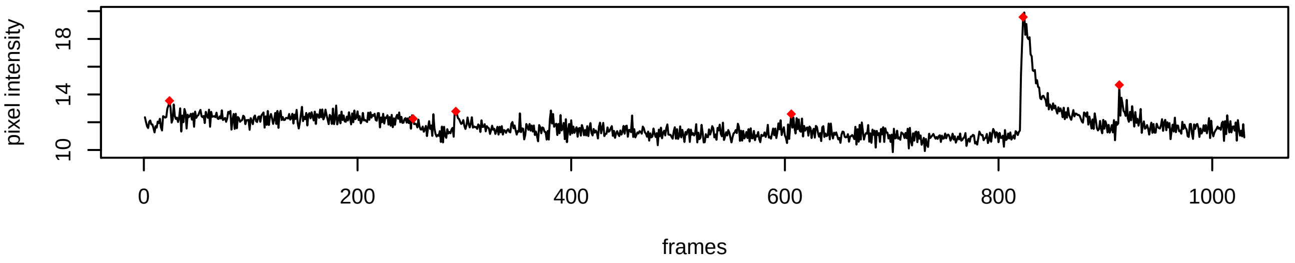

Graph 2 , 33 Total Activity 3 Position in Array 2

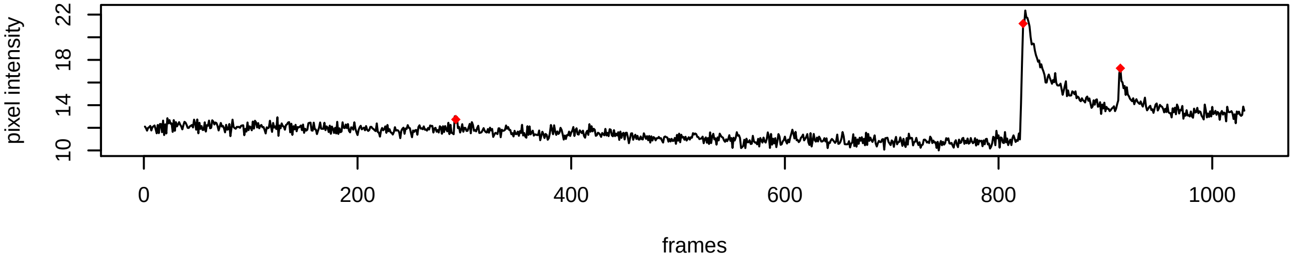

Graph 15 , 33 Total Activity 3 Position in Array 15

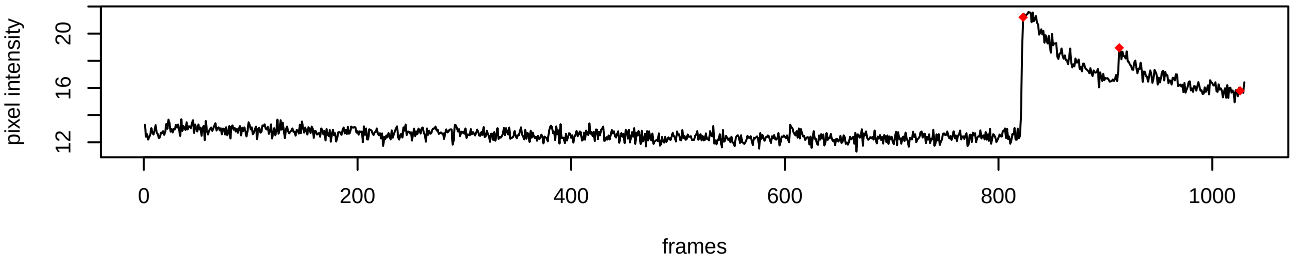

Graph 16 , 33 Total Activity 5 Position in Array 16

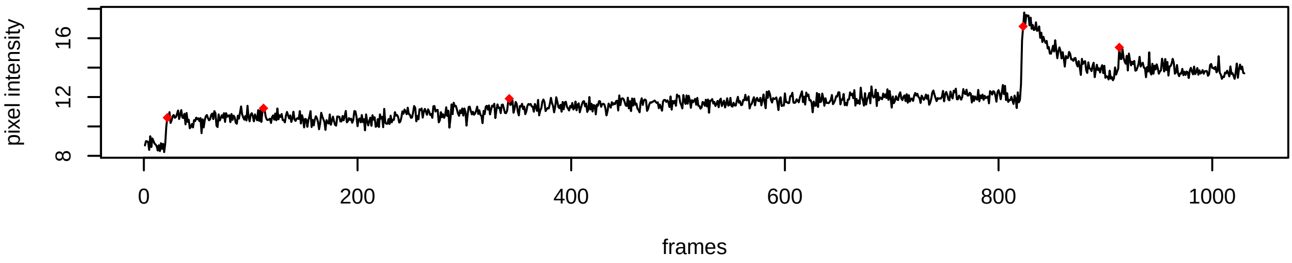

Graph 17 , 33 Total Activity 3 Position in Array 17

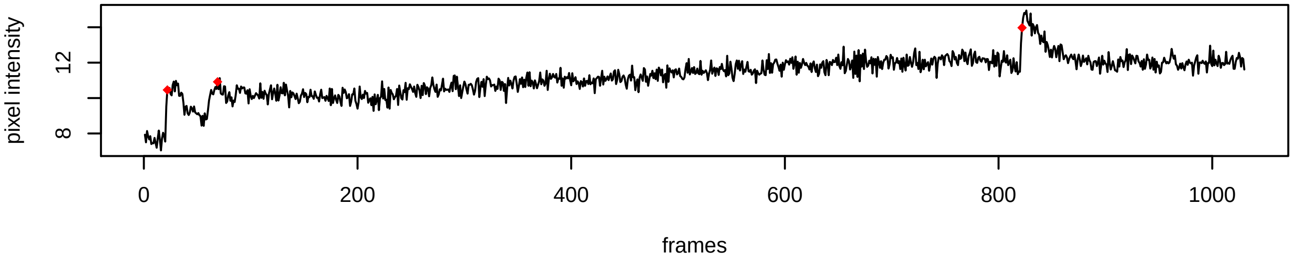

Graph 23 , 33 Total Activity 4 Position in Array 23

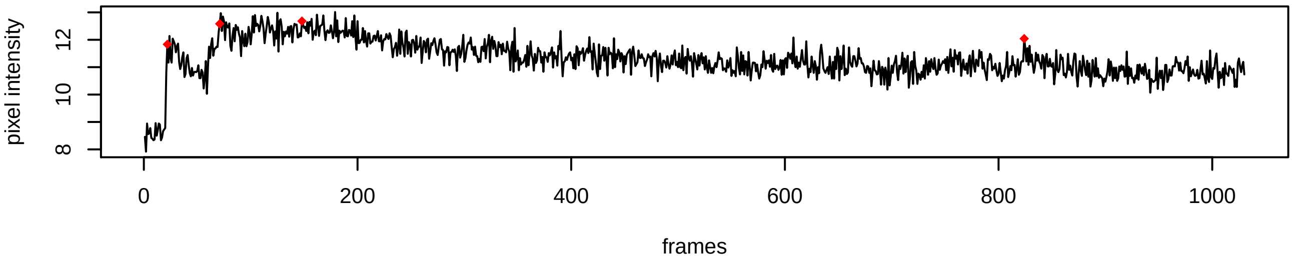

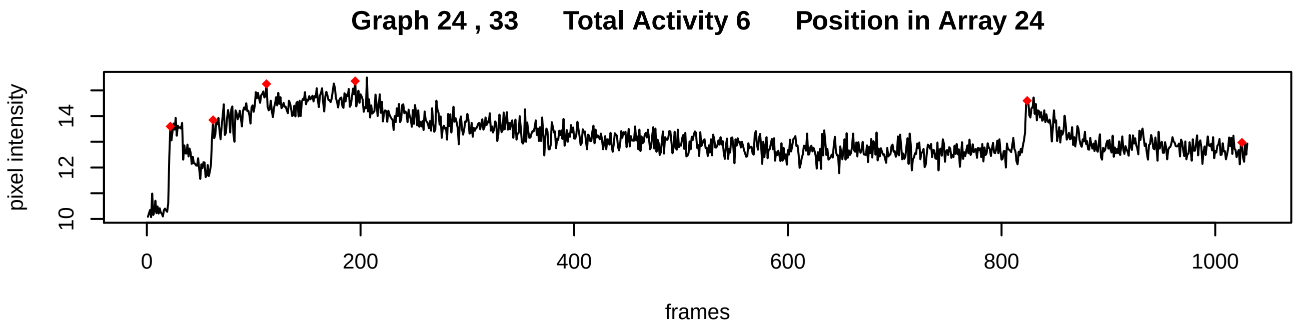
Graph 24 , 33 Total Activity 6 Position in Array 24
pixel intensity
10
12
14
0
200
400
600
800
1000
frames

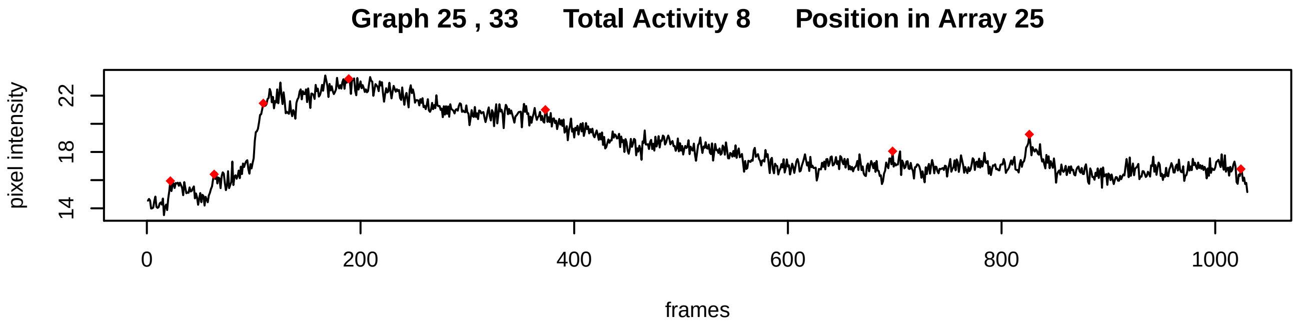
Graph 25 , 33 Total Activity 8 Position in Array 25
pixel intensity
14
18
22
0
200
400
600
800
1000
frames

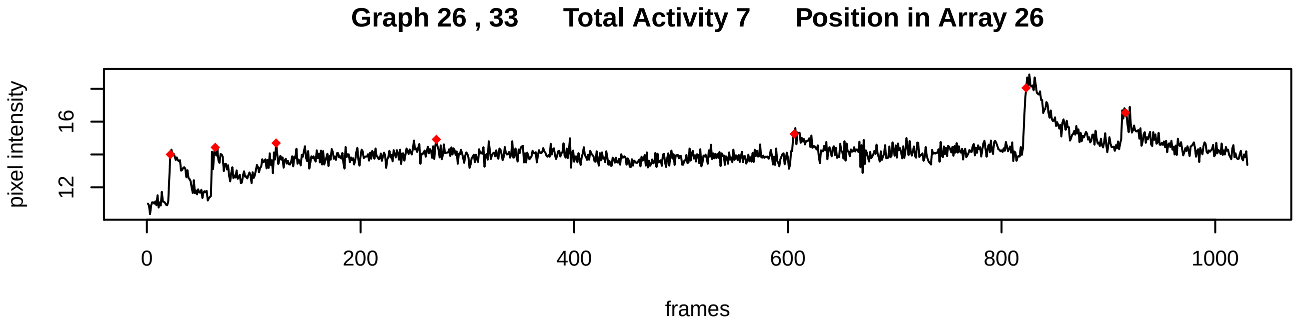
Graph 26 , 33 Total Activity 7 Position in Array 26
pixel intensity
12
16
0
200
400
600
800
1000
frames

Graph 27 , 33 Total Activity 7 Position in Array 27

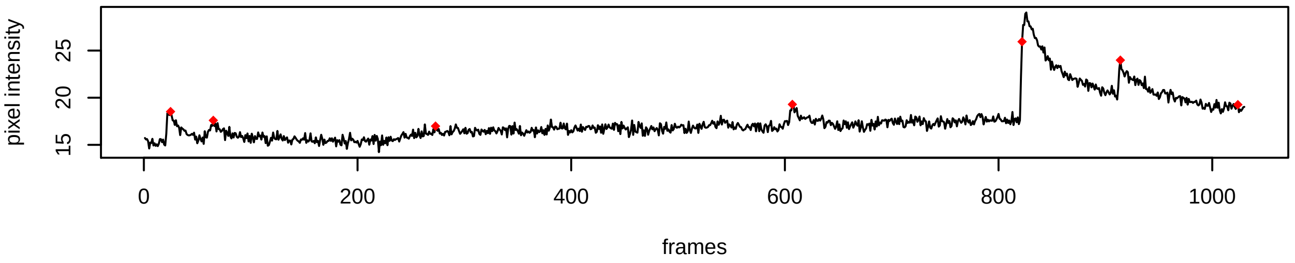

Graph 28 , 33 Total Activity 5 Position in Array 28

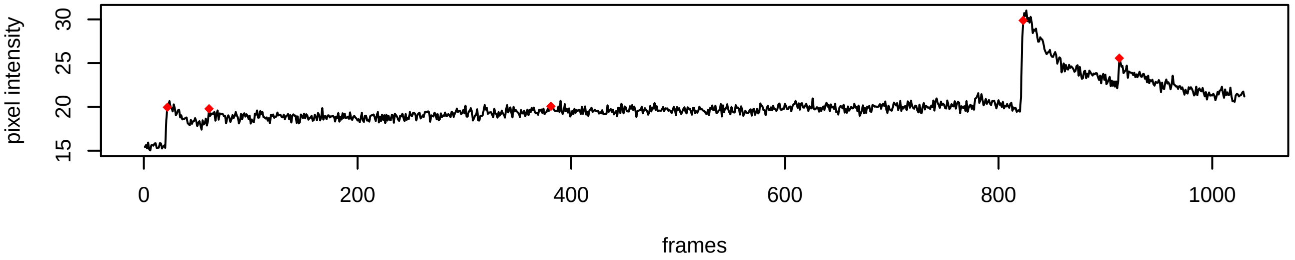

Graph 29 , 33 Total Activity 3 Position in Array 29

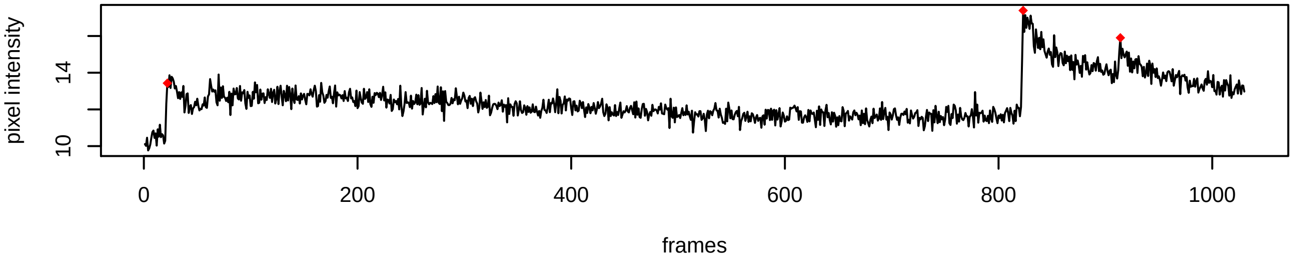

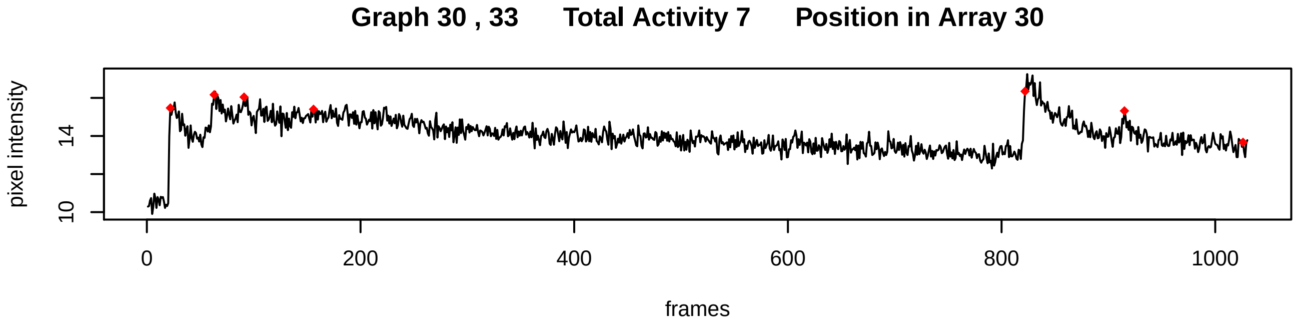

Graph 30 , 33 Total Activity 7 Position in Array 30
pixel intensity
10
14
0
200
400
600
800
1000
frames

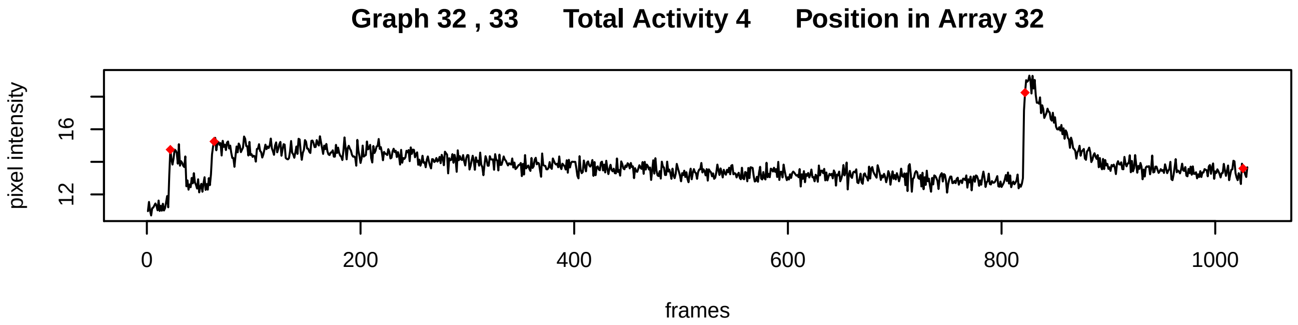

Graph 32 , 33 Total Activity 4 Position in Array 32
pixel intensity
12
16
0
200
400
600
800
1000
frames

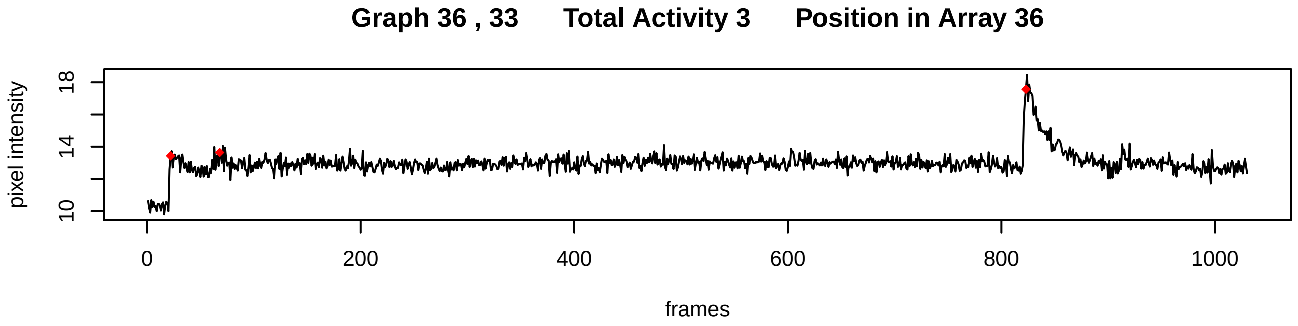

Graph 36 , 33 Total Activity 3 Position in Array 36
pixel intensity
10
14
18
0
200
400
600
800
1000
frames

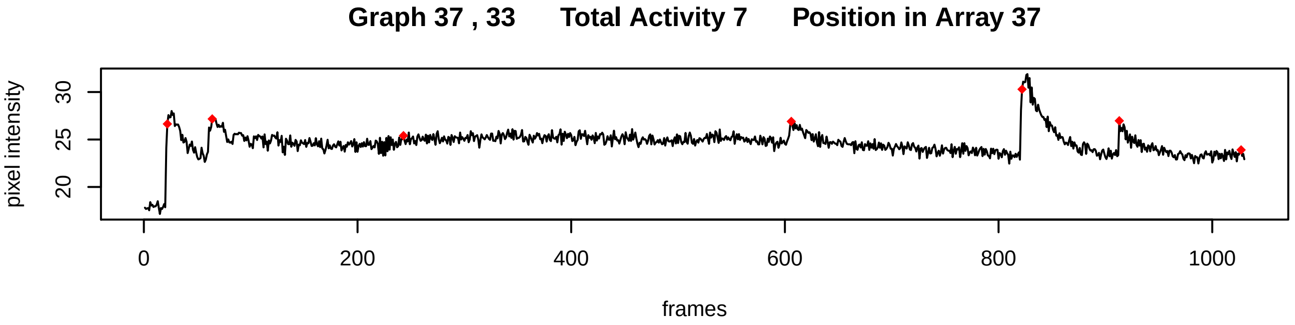
Graph 37 , 33 Total Activity 7 Position in Array 37
pixel intensity
20
25
30
0
200
400
600
800
1000
frames

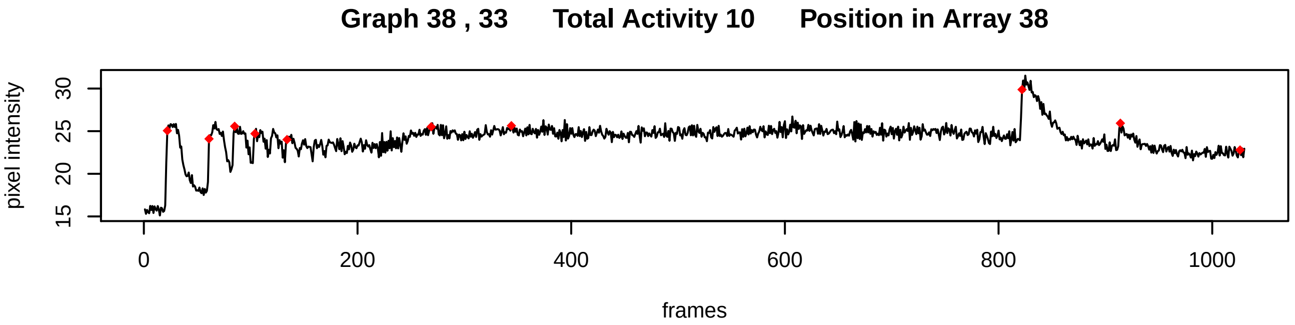
Graph 38 , 33 Total Activity 10 Position in Array 38
pixel intensity
15
20
25
30
0
200
400
600
800
1000
frames

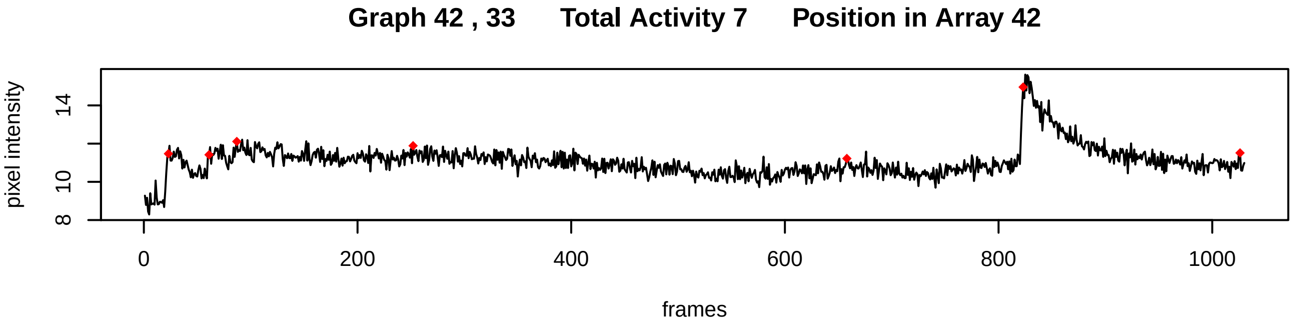
Graph 42 , 33 Total Activity 7 Position in Array 42
pixel intensity
8
10
14
0
200
400
600
800
1000
frames

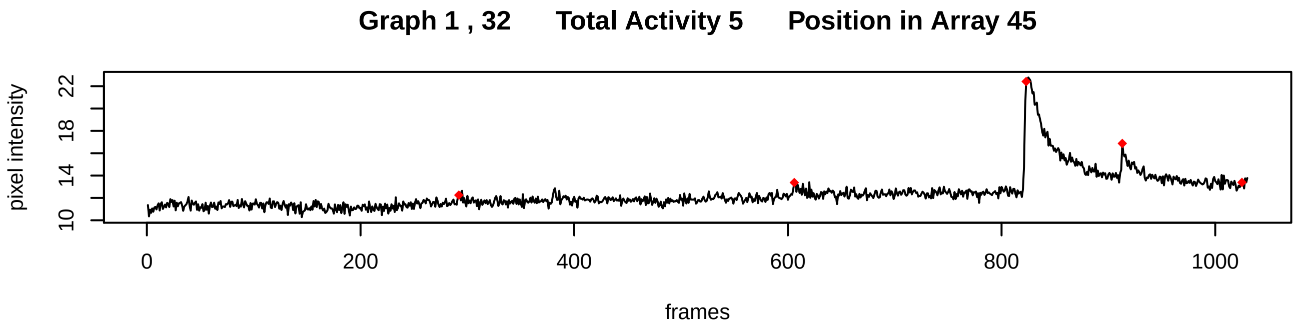
Graph 1 , 32 Total Activity 5 Position in Array 45
pixel intensity
10
14
18
22
0
200
400
600
800
1000
frames

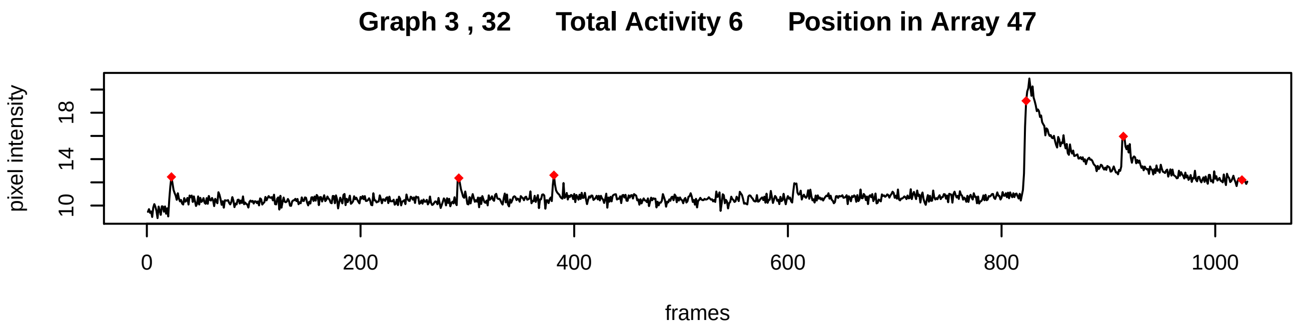
Graph 3 , 32 Total Activity 6 Position in Array 47
pixel intensity
10
14
18
0
200
400
600
800
1000
frames

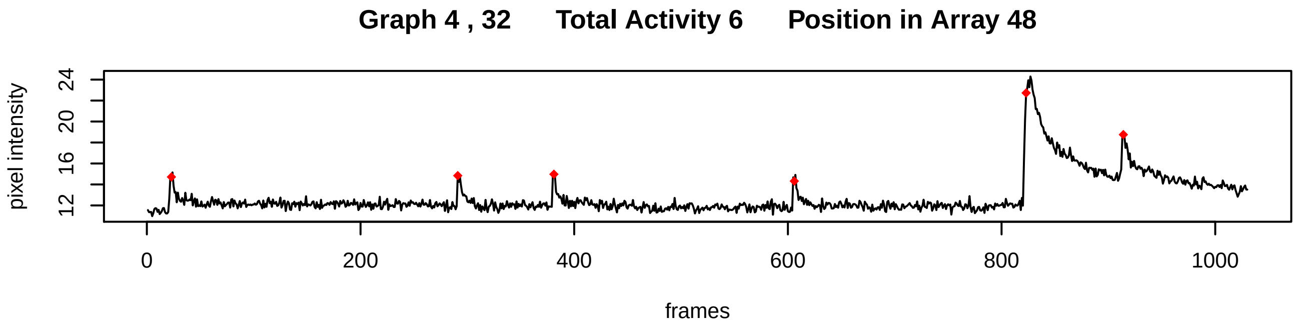
Graph 4 , 32 Total Activity 6 Position in Array 48
pixel intensity
12
16
20
24
0
200
400
600
800
1000
frames

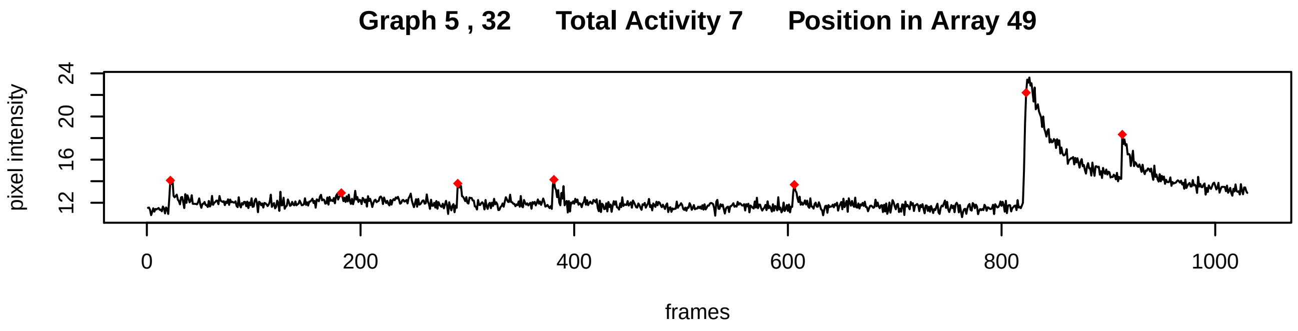
Graph 5 , 32 Total Activity 7 Position in Array 49
pixel intensity
12
16
20
24
0
200
400
600
800
1000
frames

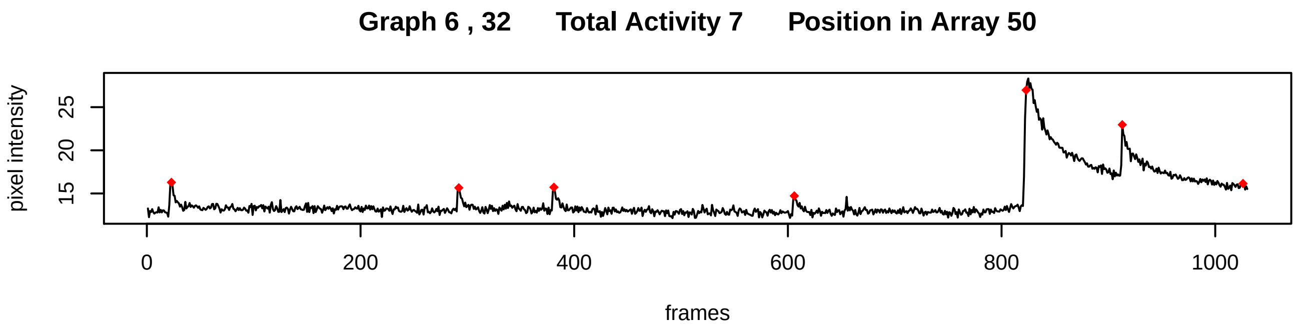
Graph 6 , 32 Total Activity 7 Position in Array 50
pixel intensity
15
20
25
0
200
400
600
800
1000
frames

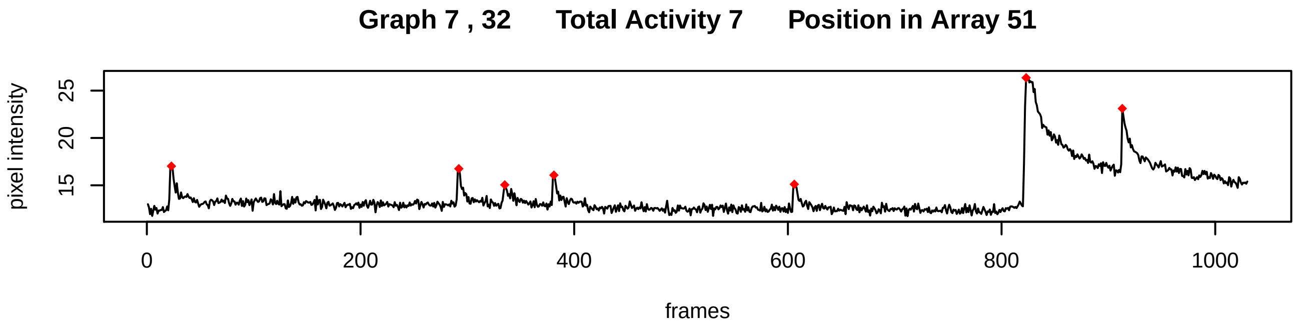
Graph 7 , 32 Total Activity 7 Position in Array 51
pixel intensity
15
20
25
0
200
400
600
800
1000
frames

Graph 8 , 32   Total Activity 7   Position in Array 52

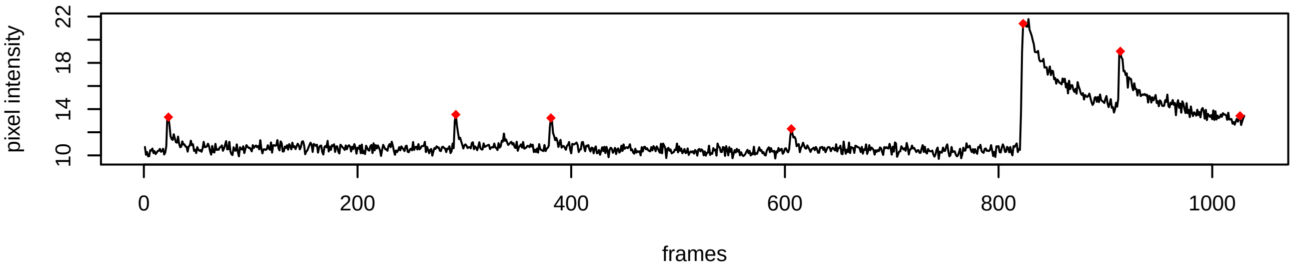

Graph 11 , 32   Total Activity 8   Position in Array 55

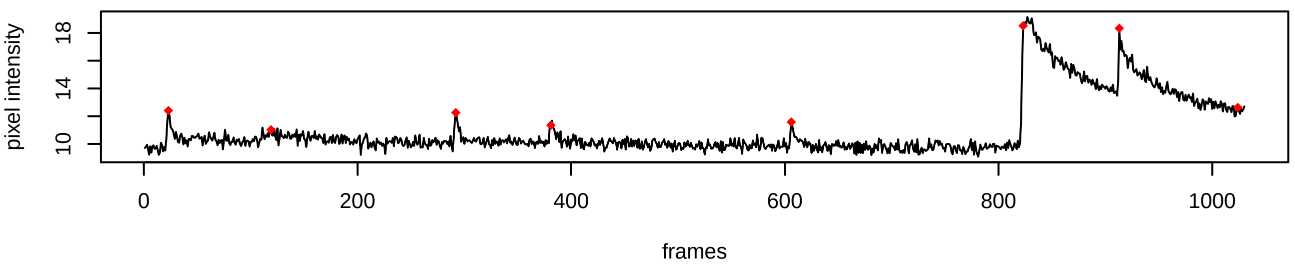

Graph 12 , 32   Total Activity 6   Position in Array 56

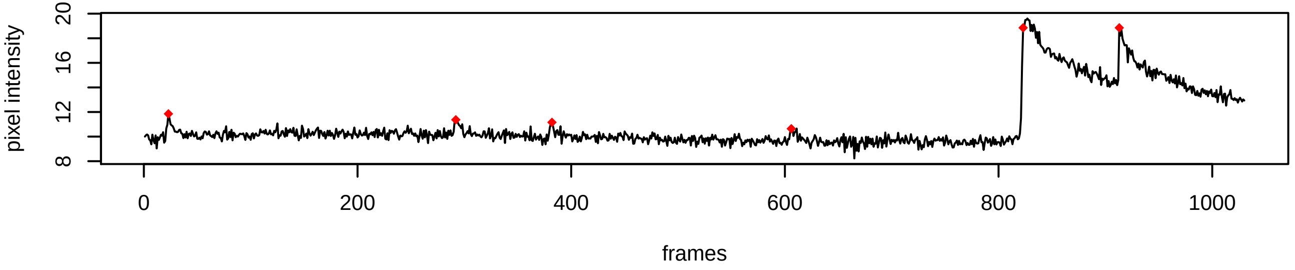

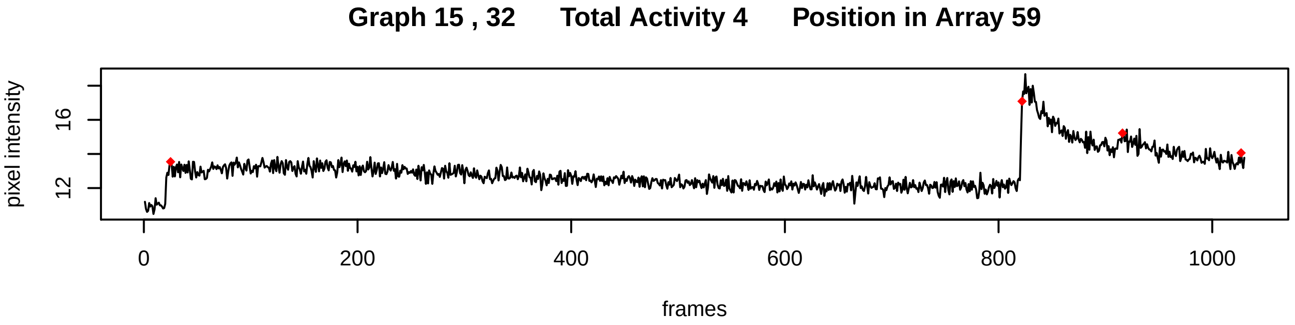
Graph 15 , 32 Total Activity 4 Position in Array 59
pixel intensity
12
16
0
200
400
600
800
1000
frames

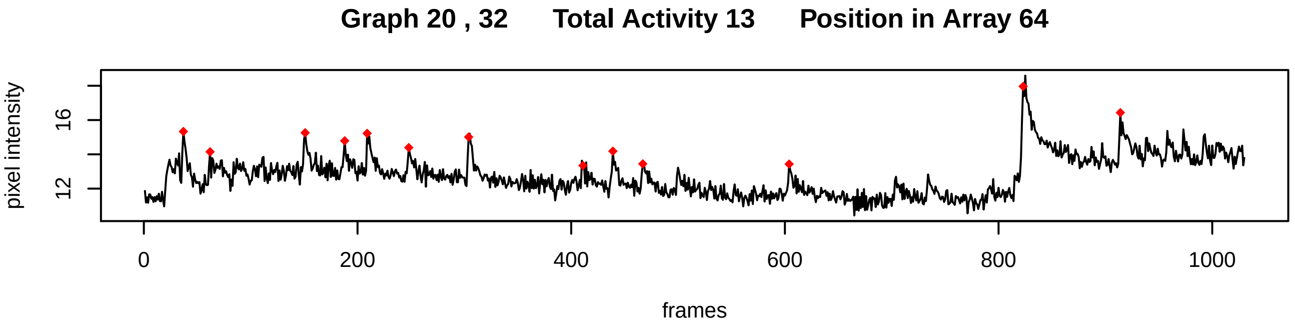
Graph 20 , 32 Total Activity 13 Position in Array 64
pixel intensity
12
16
0
200
400
600
800
1000
frames

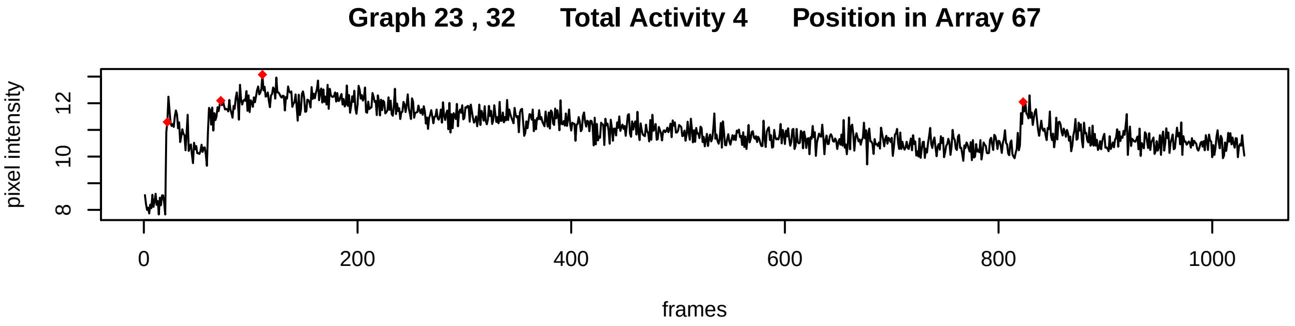
Graph 23 , 32 Total Activity 4 Position in Array 67
pixel intensity
8
10
12
0
200
400
600
800
1000
frames

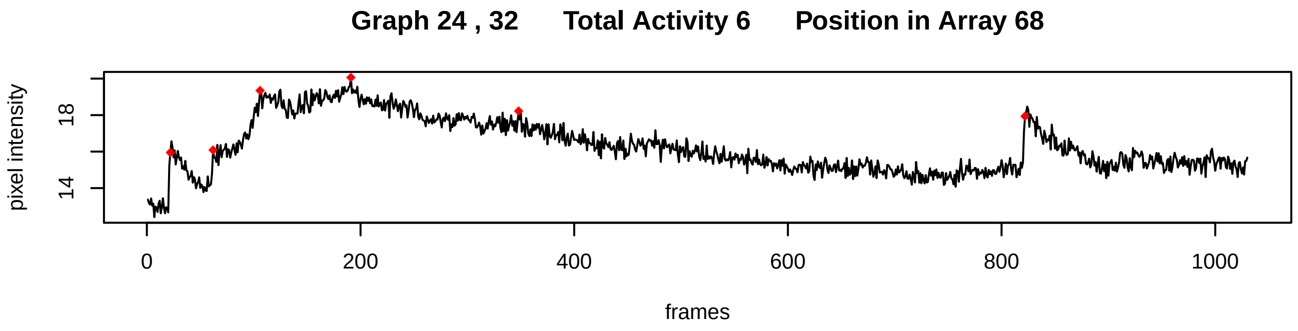
Graph 24 , 32 Total Activity 6 Position in Array 68
pixel intensity
14
16
18
0
200
400
600
800
1000
frames

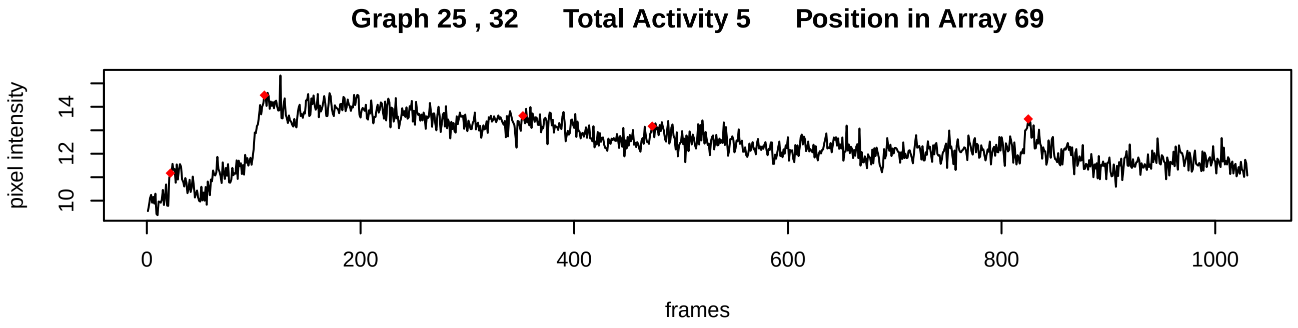
Graph 25 , 32 Total Activity 5 Position in Array 69
pixel intensity
10
12
14
0
200
400
600
800
1000
frames

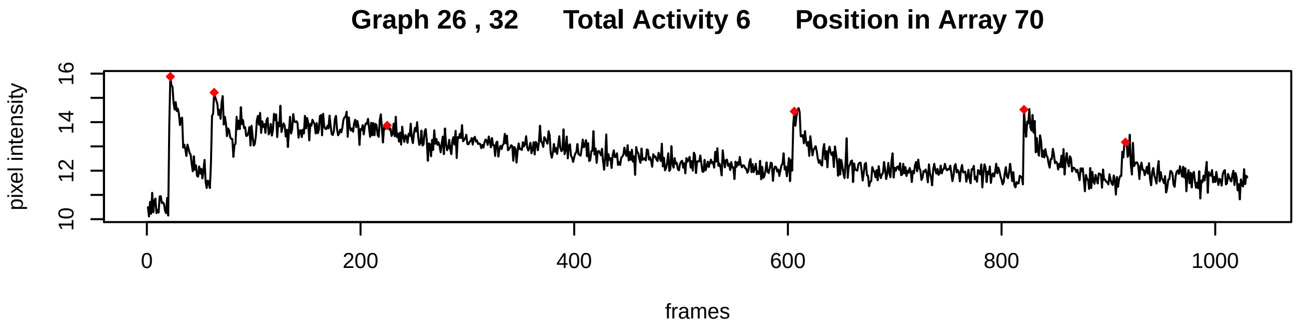
Graph 26 , 32 Total Activity 6 Position in Array 70
pixel intensity
10
12
14
16
0
200
400
600
800
1000
frames

Graph 27 , 32   Total Activity 8   Position in Array 71

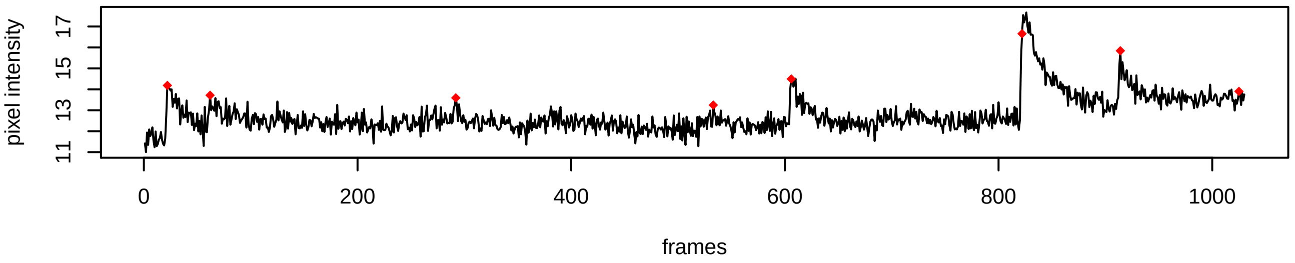

Graph 29 , 32   Total Activity 4   Position in Array 73

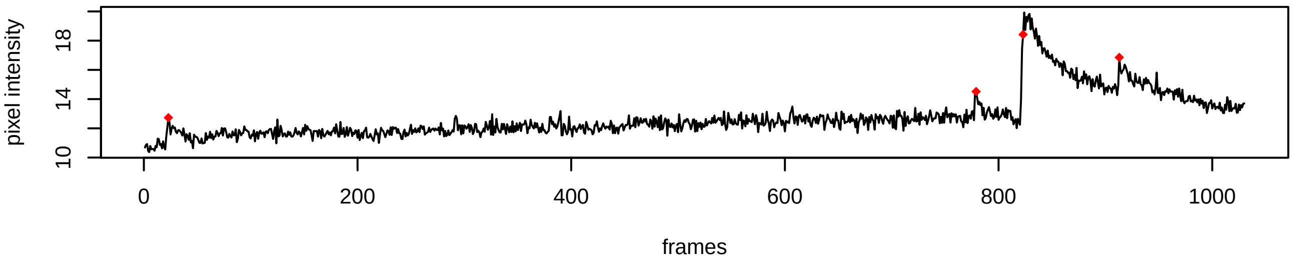

Graph 30 , 32   Total Activity 5   Position in Array 74

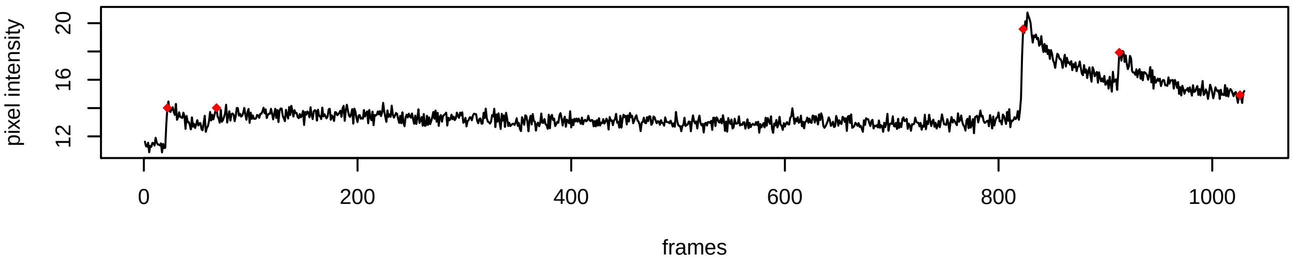

Graph 32 , 32   Total Activity 3   Position in Array 76

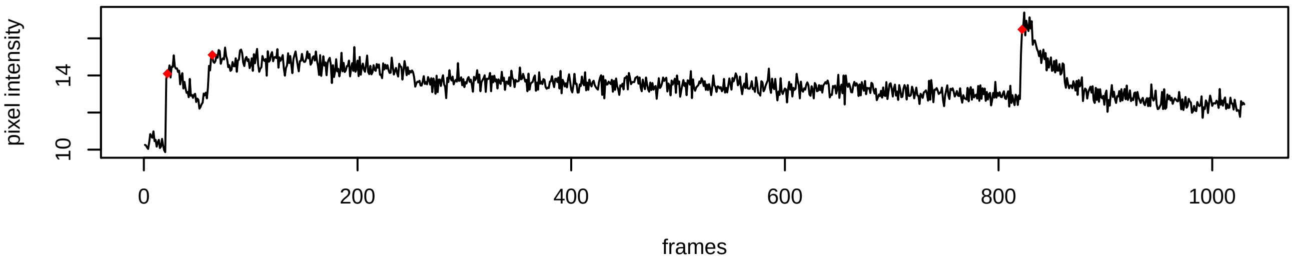

Graph 35 , 32   Total Activity 5   Position in Array 79

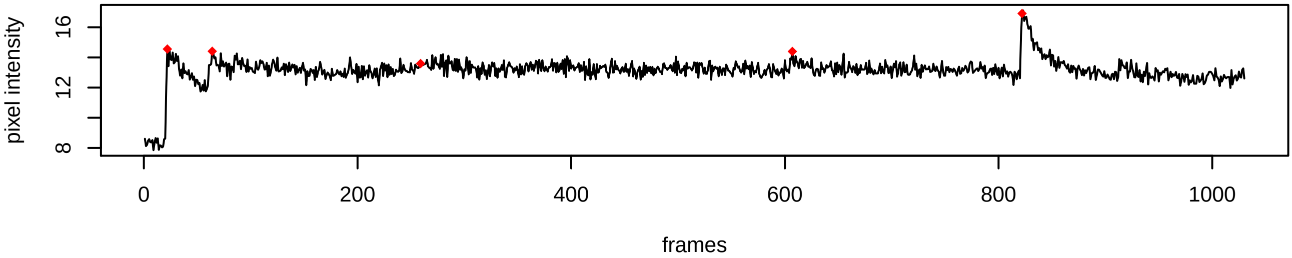

Graph 37 , 32   Total Activity 6   Position in Array 81

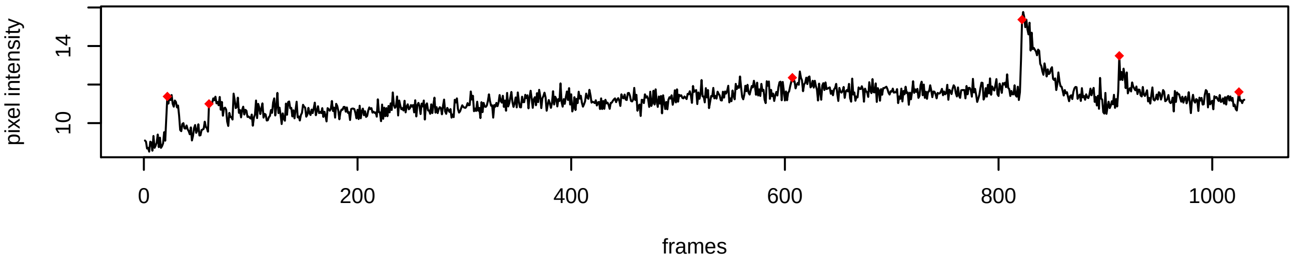

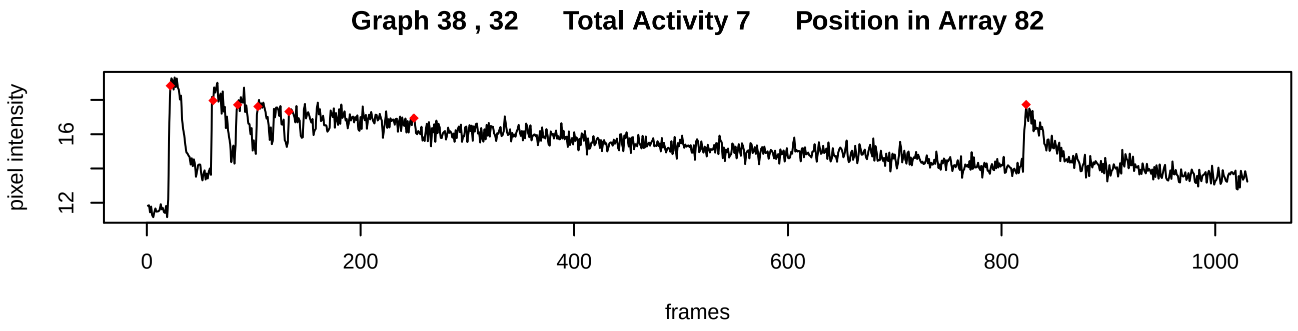
Graph 38 , 32 Total Activity 7 Position in Array 82
pixel intensity
12
16
0
200
400
600
800
1000
frames

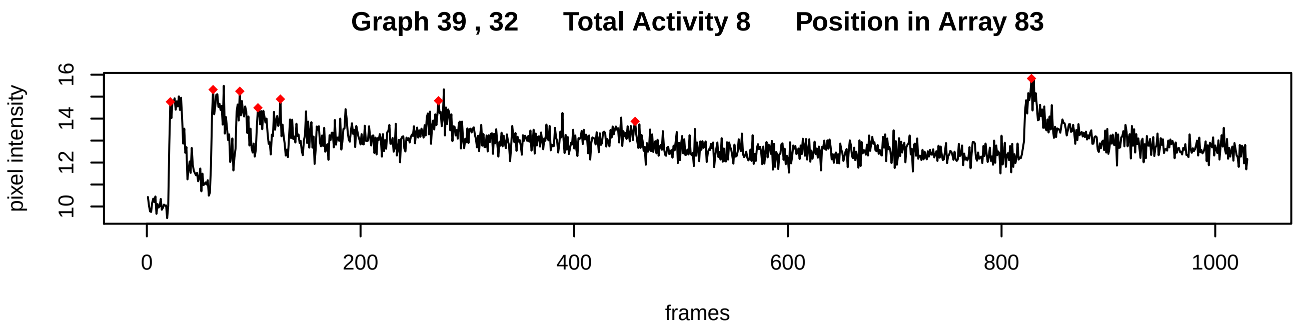
Graph 39 , 32 Total Activity 8 Position in Array 83
pixel intensity
10
12
14
16
0
200
400
600
800
1000
frames

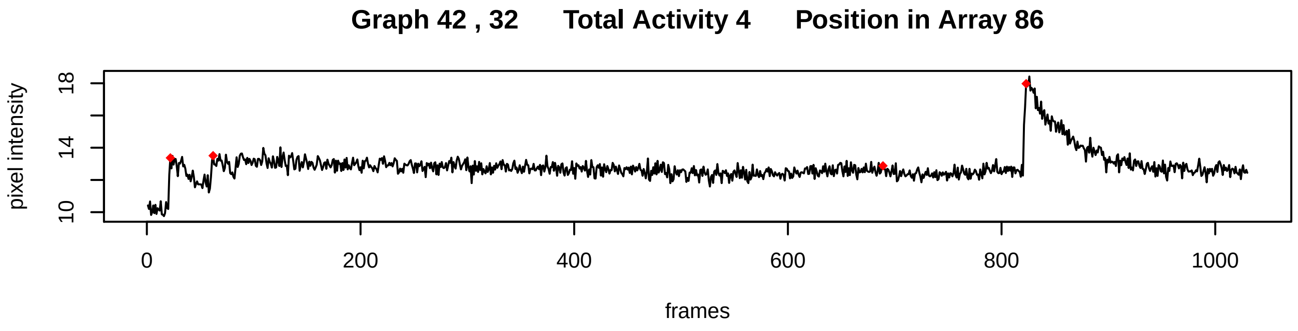
Graph 42 , 32 Total Activity 4 Position in Array 86
pixel intensity
10
14
18
0
200
400
600
800
1000
frames

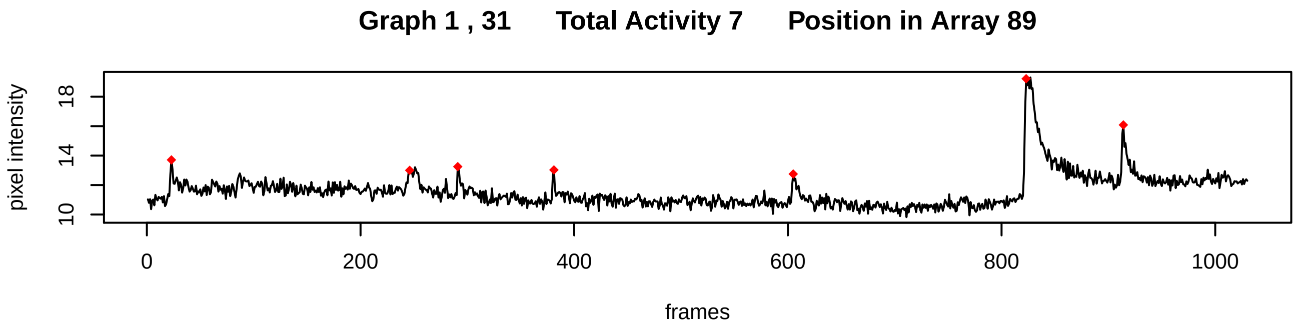
Graph 1 , 31 Total Activity 7 Position in Array 89
pixel intensity
10
14
18
0
200
400
600
800
1000
frames

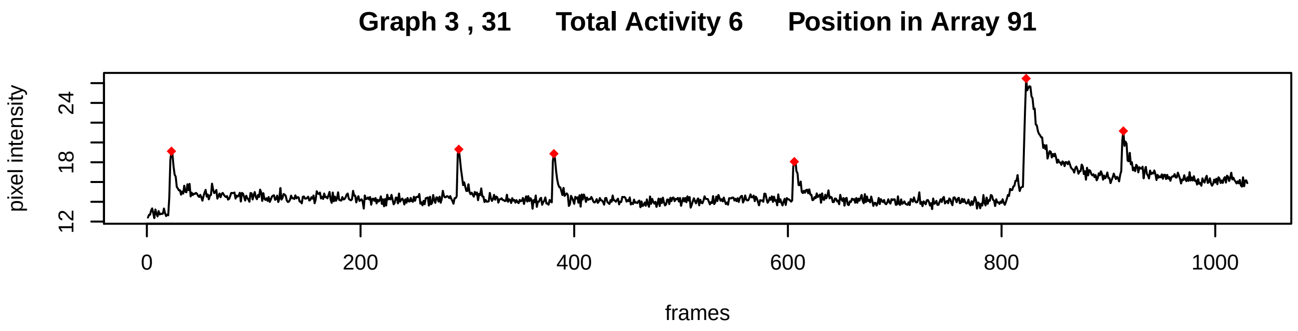
Graph 3 , 31 Total Activity 6 Position in Array 91
pixel intensity
12
18
24
0
200
400
600
800
1000
frames

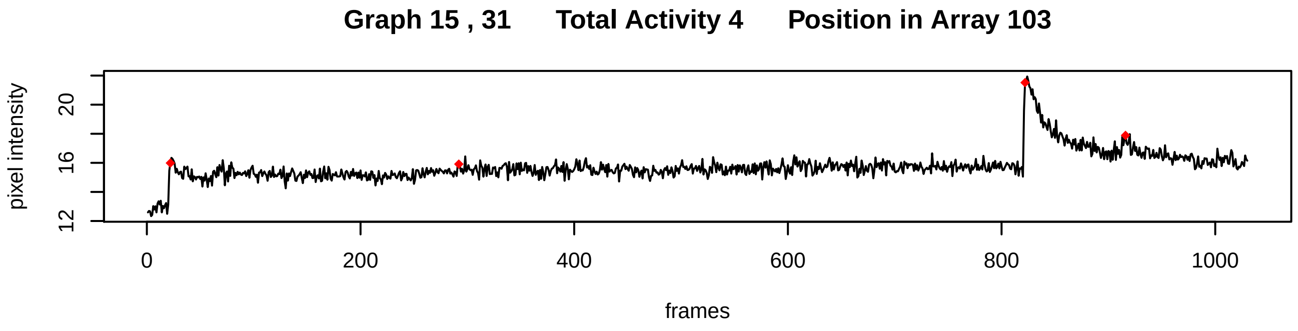
Graph 15 , 31 Total Activity 4 Position in Array 103
pixel intensity
12
16
20
0
200
400
600
800
1000
frames

Graph 20 , 31 Total Activity 14 Position in Array 108

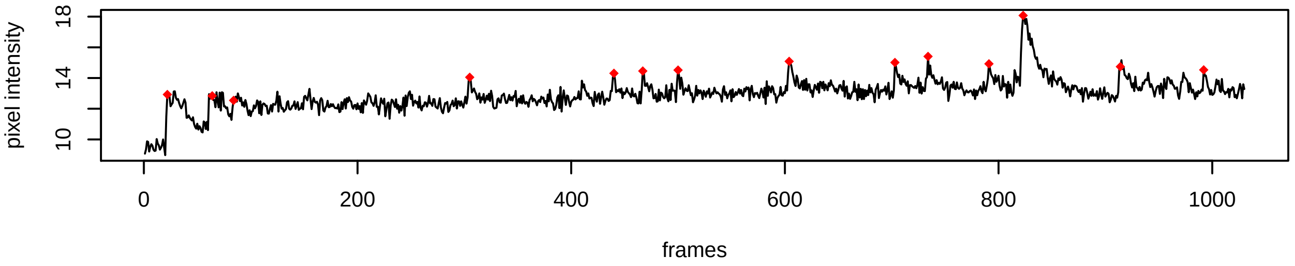

Graph 21 , 31 Total Activity 5 Position in Array 109

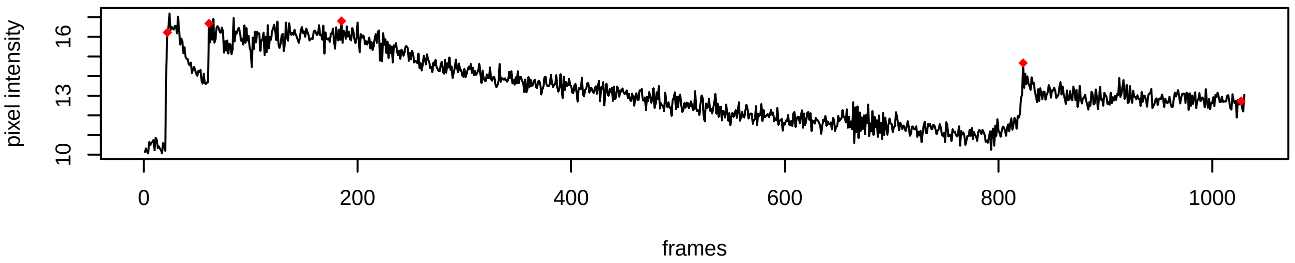

Graph 22 , 31 Total Activity 5 Position in Array 110

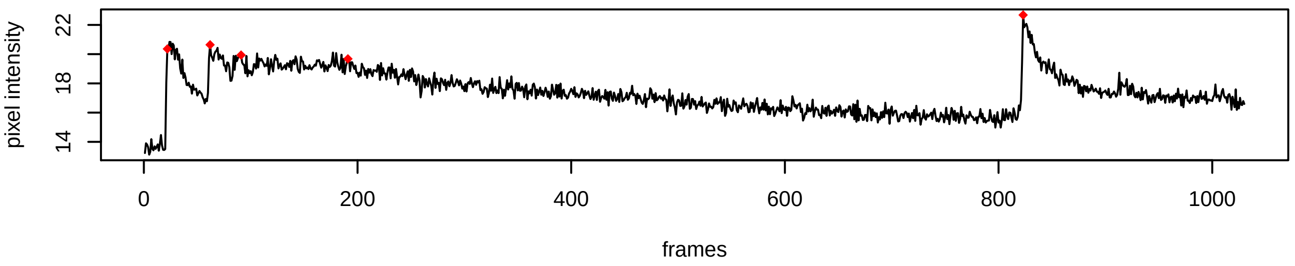

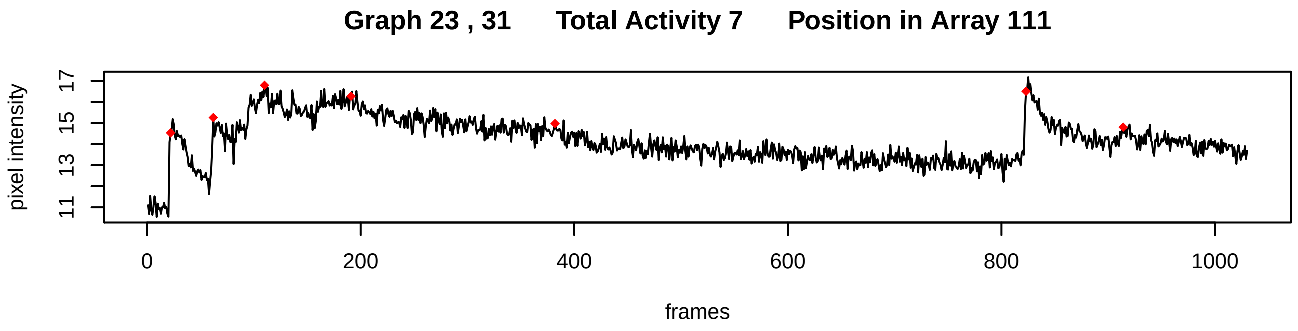
Graph 23 , 31 Total Activity 7 Position in Array 111
pixel intensity
11
13
15
17
0
200
400
600
800
1000
frames

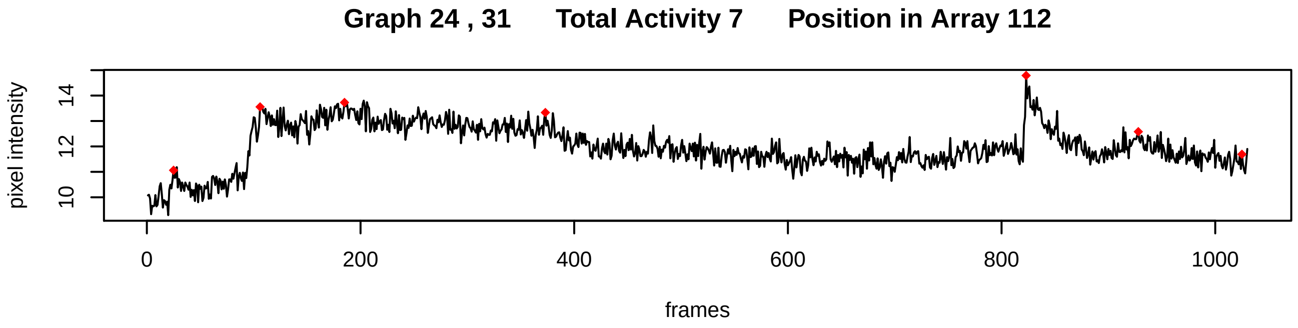
Graph 24 , 31 Total Activity 7 Position in Array 112
pixel intensity
10
12
14
0
200
400
600
800
1000
frames

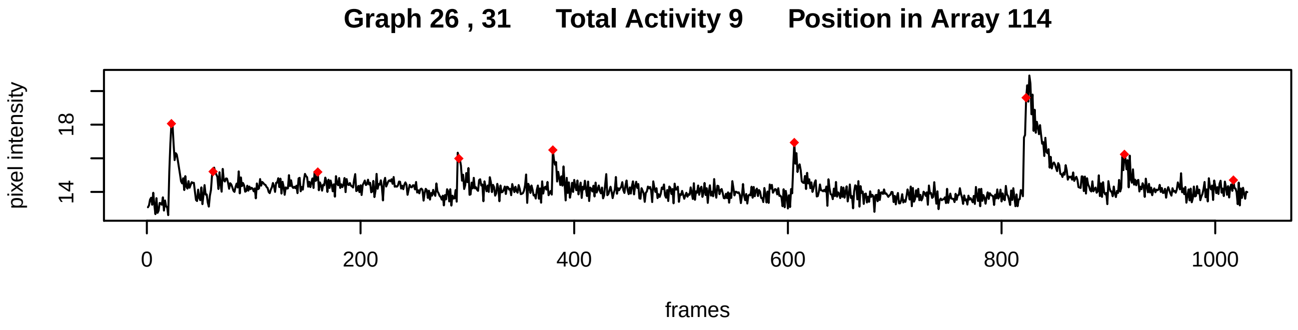
Graph 26 , 31 Total Activity 9 Position in Array 114
pixel intensity
14
18
0
200
400
600
800
1000
frames

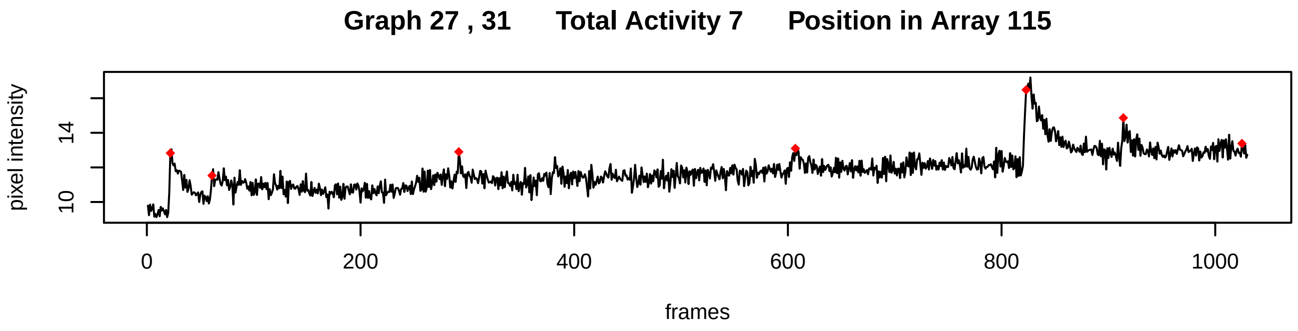
Graph 27 , 31 Total Activity 7 Position in Array 115
pixel intensity
10
14
0
200
400
600
800
1000
frames

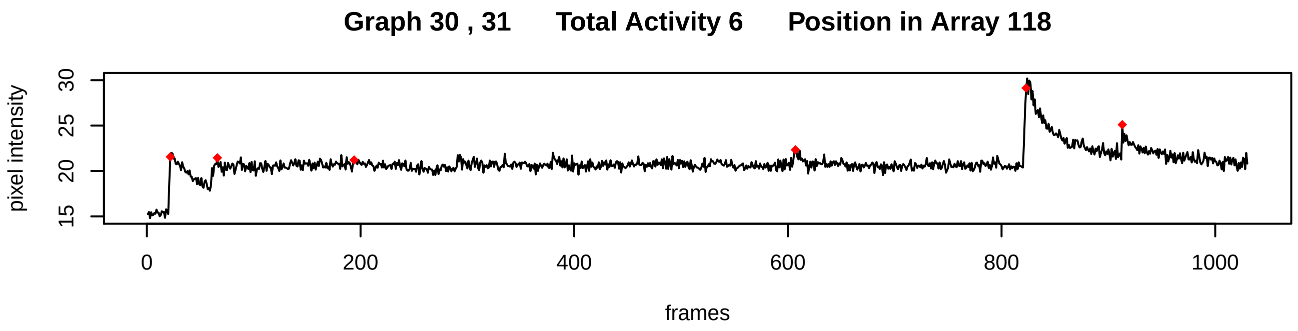
Graph 30 , 31 Total Activity 6 Position in Array 118
pixel intensity
15
20
25
30
0
200
400
600
800
1000
frames

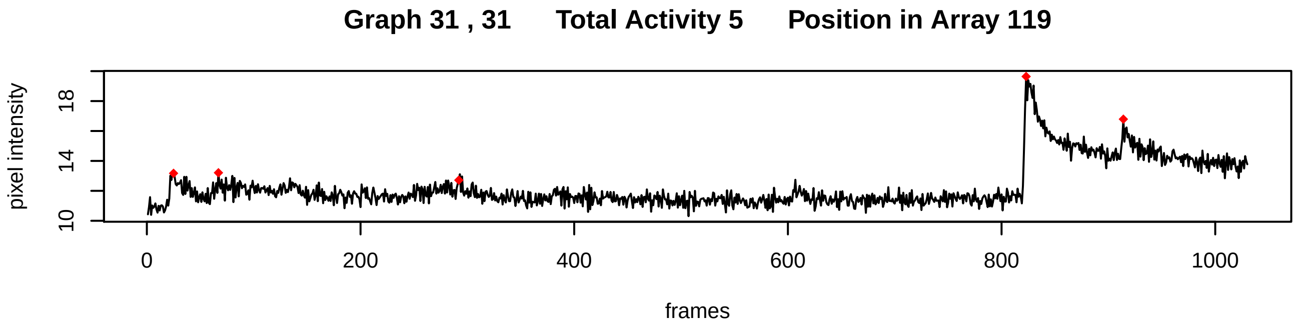
Graph 31 , 31 Total Activity 5 Position in Array 119
pixel intensity
10
14
18
0
200
400
600
800
1000
frames

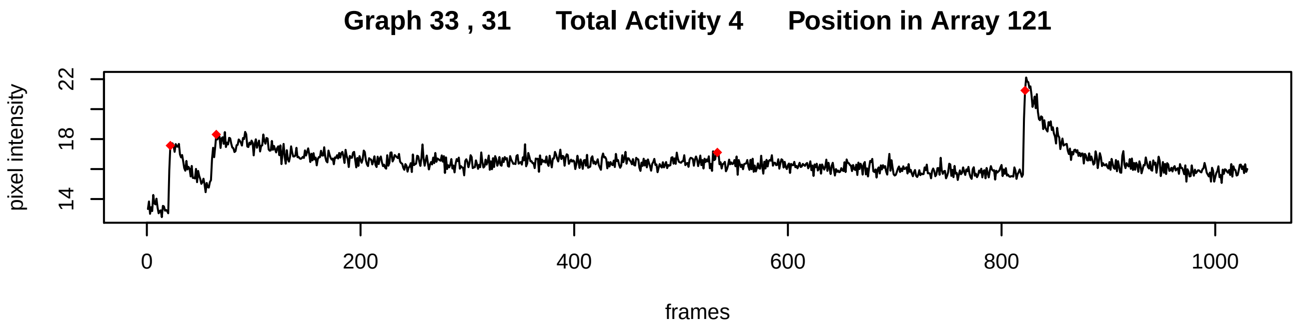

Graph 33 , 31 Total Activity 4 Position in Array 121
pixel intensity
14
18
22
0
200
400
600
800
1000
frames

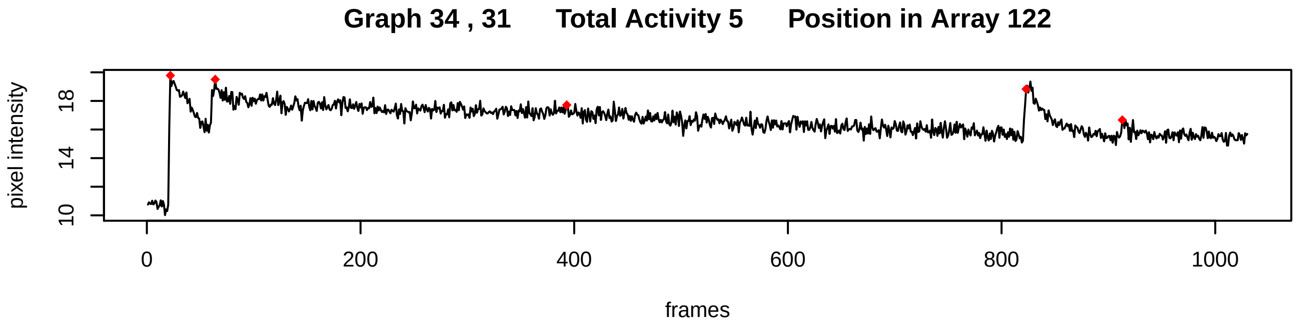

Graph 34 , 31 Total Activity 5 Position in Array 122
pixel intensity
10
14
18
0
200
400
600
800
1000
frames

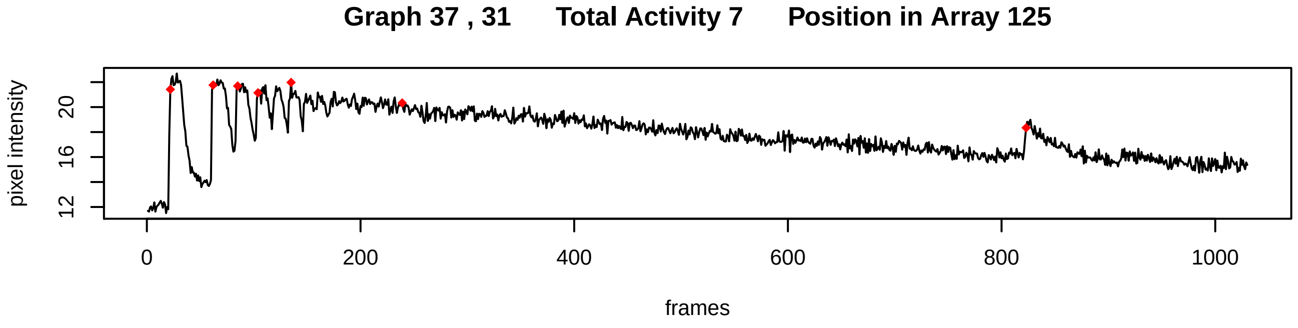

Graph 37 , 31 Total Activity 7 Position in Array 125
pixel intensity
12
16
20
0
200
400
600
800
1000
frames

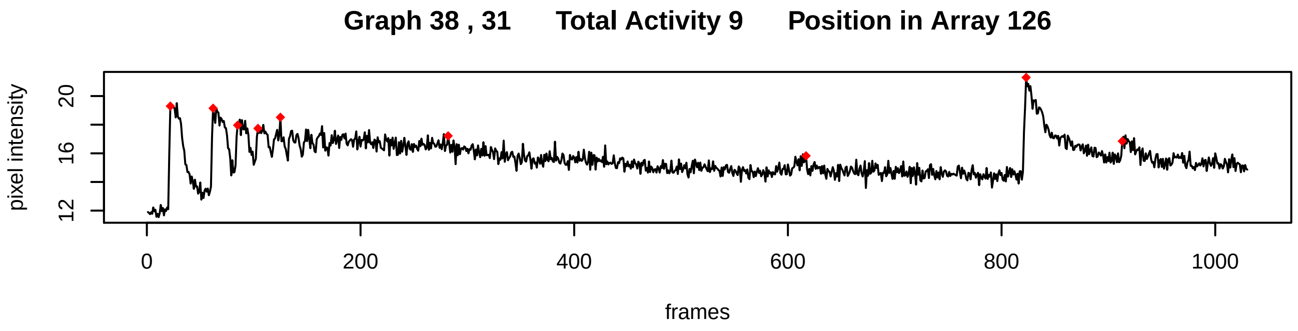
Graph 38 , 31 Total Activity 9 Position in Array 126
pixel intensity
12
16
20
0
200
400
600
800
1000
frames

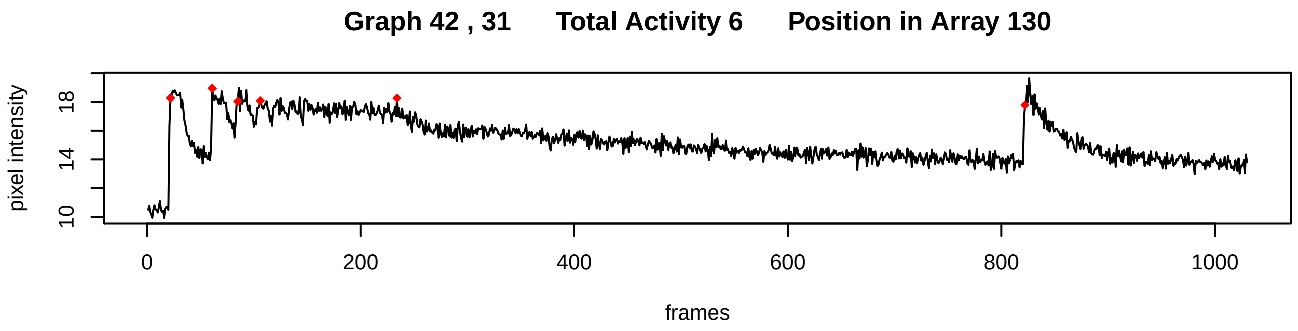
Graph 42 , 31 Total Activity 6 Position in Array 130
pixel intensity
10
14
18
0
200
400
600
800
1000
frames

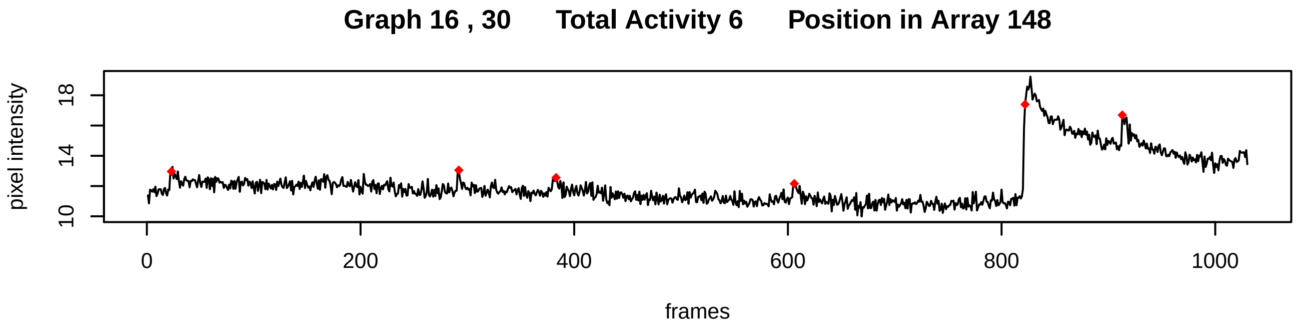
Graph 16 , 30 Total Activity 6 Position in Array 148
pixel intensity
10
14
18
0
200
400
600
800
1000
frames

Graph 20 , 30 Total Activity 3 Position in Array 152

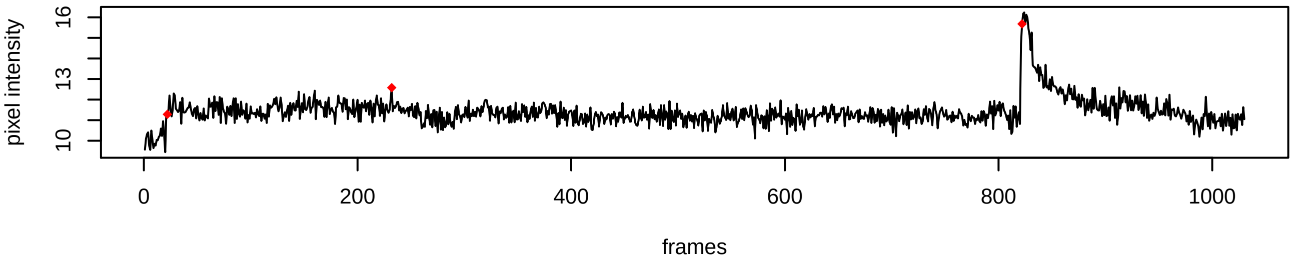

Graph 21 , 30 Total Activity 5 Position in Array 153

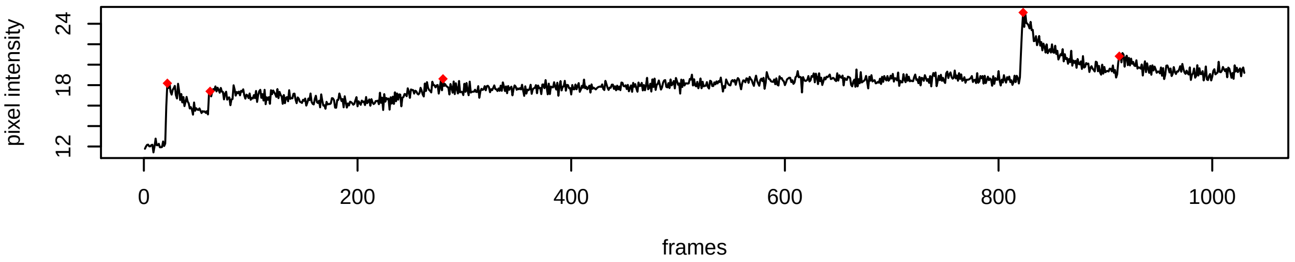

Graph 22 , 30 Total Activity 4 Position in Array 154

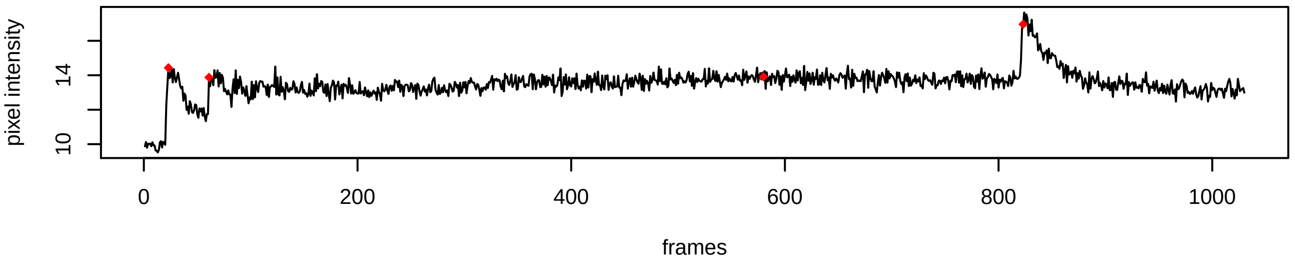

Graph 23 , 30 Total Activity 4 Position in Array 155

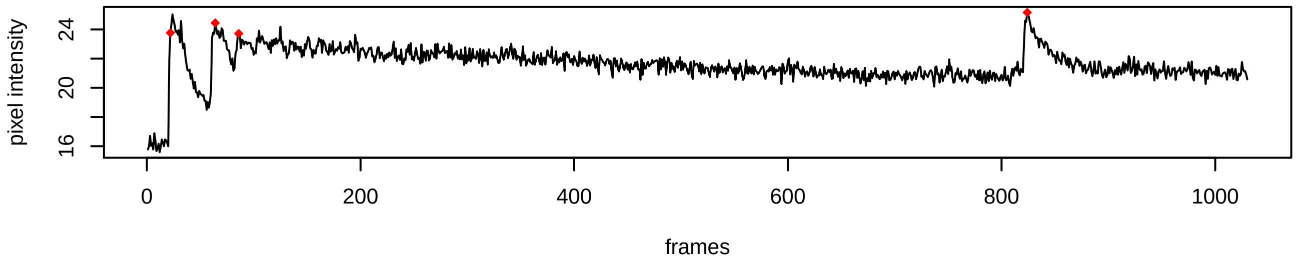

Graph 24 , 30 Total Activity 6 Position in Array 156

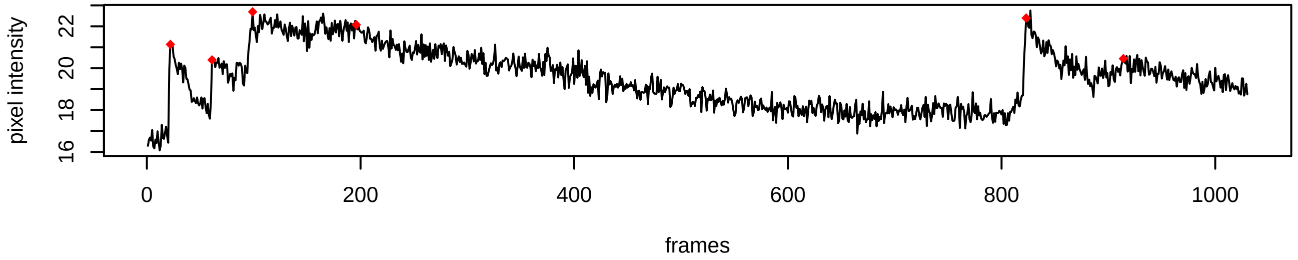

Graph 25 , 30 Total Activity 6 Position in Array 157

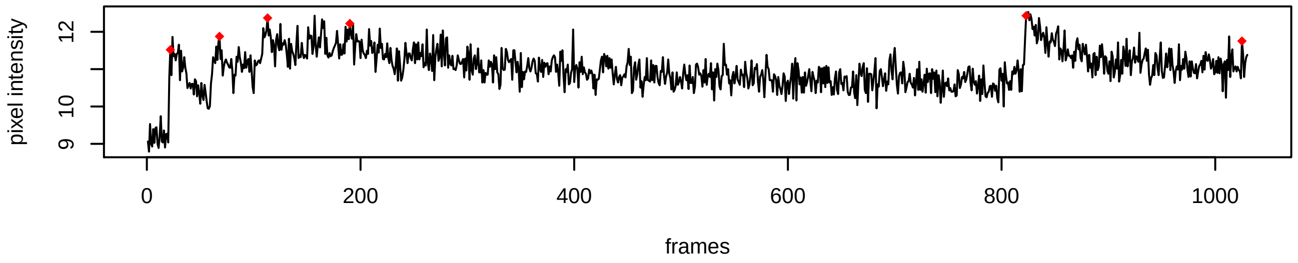

Graph 26 , 30 Total Activity 8 Position in Array 158

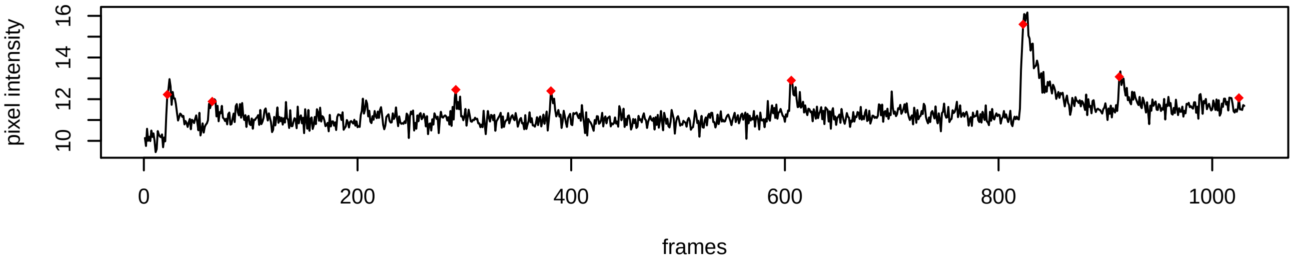

Graph 31 , 30 Total Activity 6 Position in Array 163

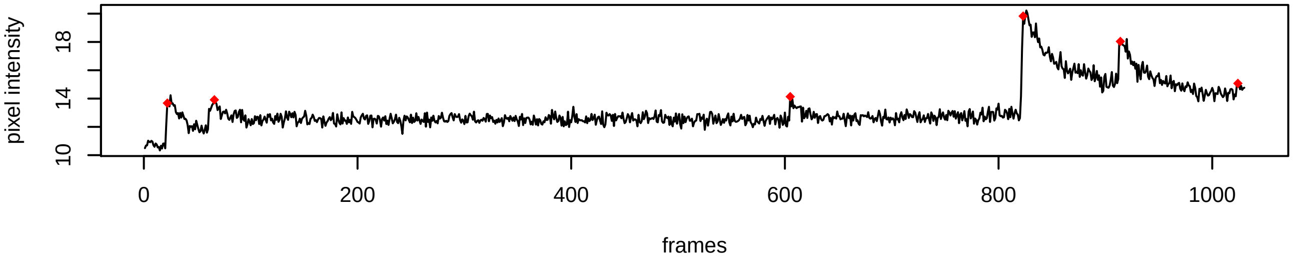

Graph 33 , 30 Total Activity 4 Position in Array 165

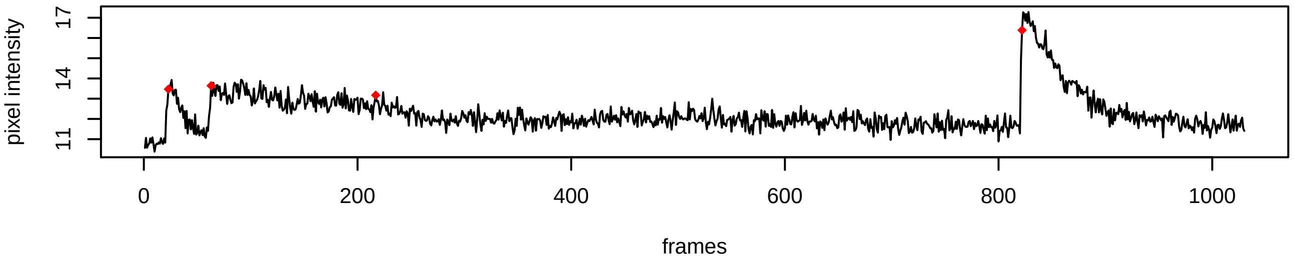

Graph 34 , 30   Total Activity 8   Position in Array 166

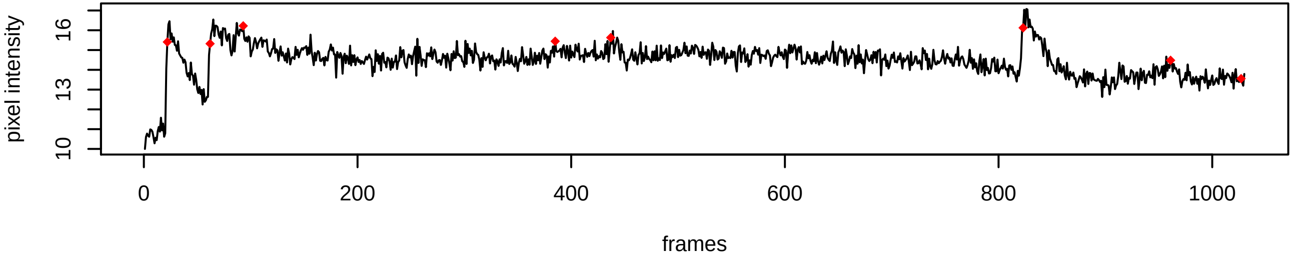

Graph 38 , 30   Total Activity 8   Position in Array 170

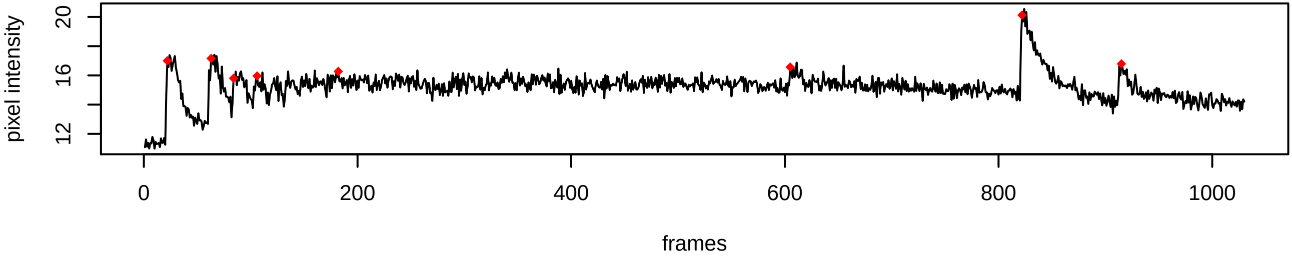

Graph 41 , 30   Total Activity 4   Position in Array 173

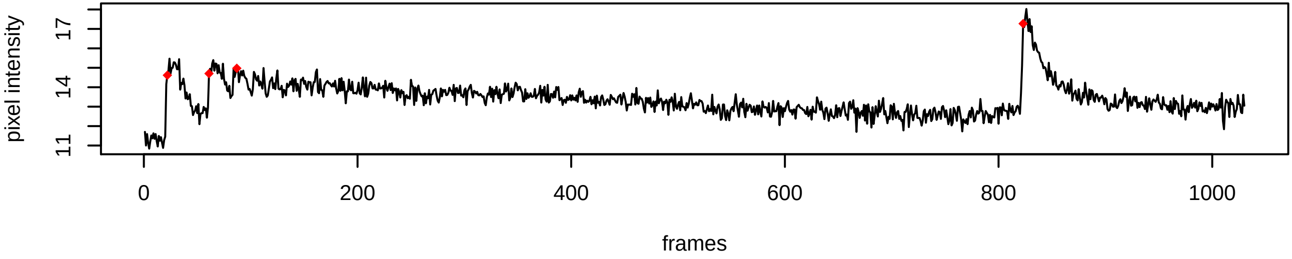

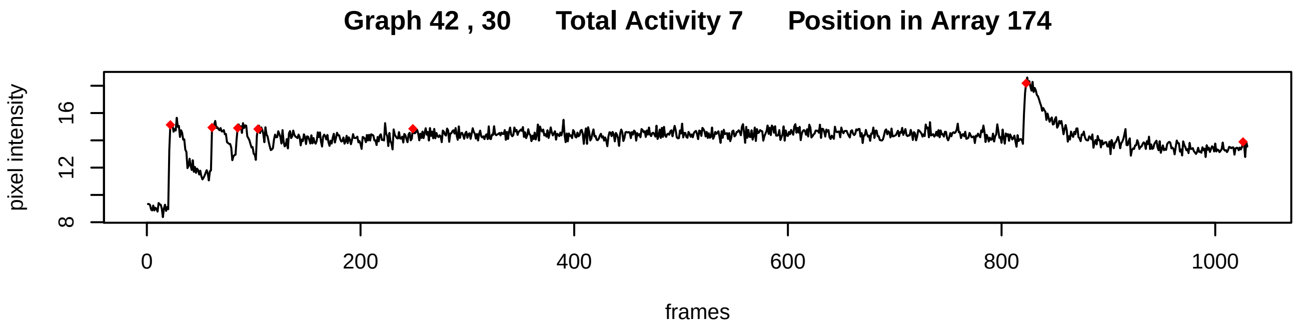
Graph 42 , 30 Total Activity 7 Position in Array 174
pixel intensity
8
12
16
0
200
400
600
800
1000
frames

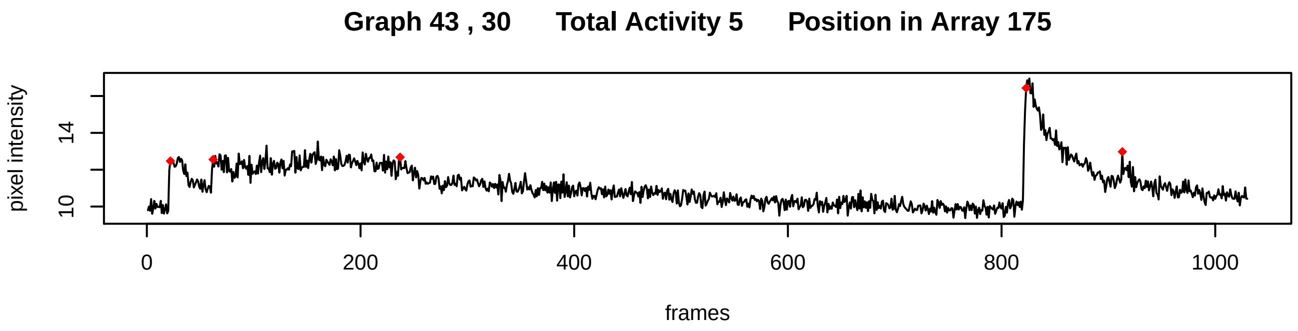
Graph 43 , 30 Total Activity 5 Position in Array 175
pixel intensity
10
14
0
200
400
600
800
1000
frames

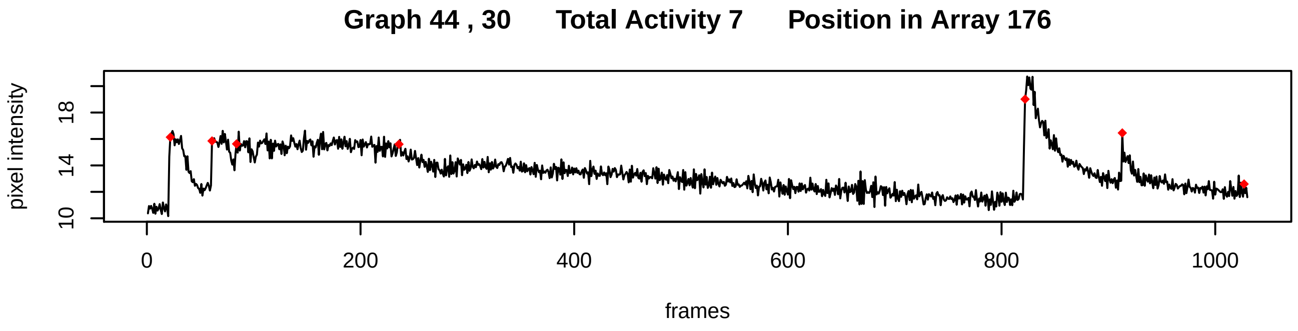
Graph 44 , 30 Total Activity 7 Position in Array 176
pixel intensity
10
14
18
0
200
400
600
800
1000
frames

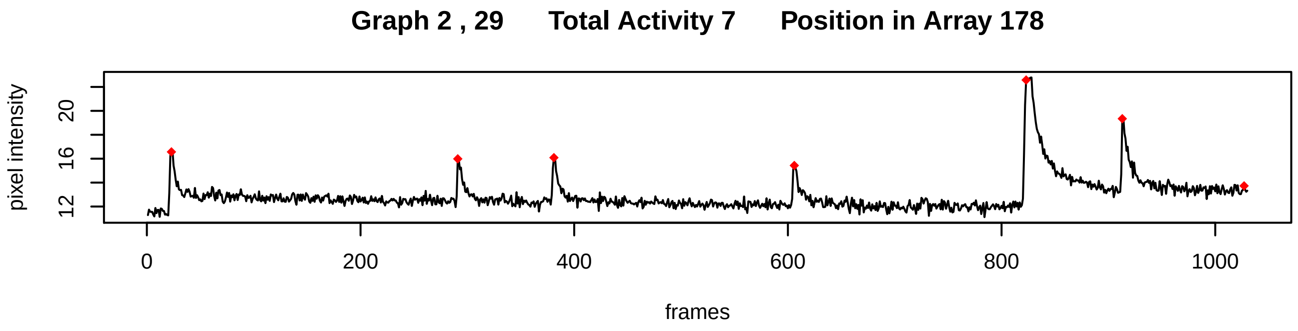
Graph 2 , 29 Total Activity 7 Position in Array 178
pixel intensity
12
16
20
0
200
400
600
800
1000
frames

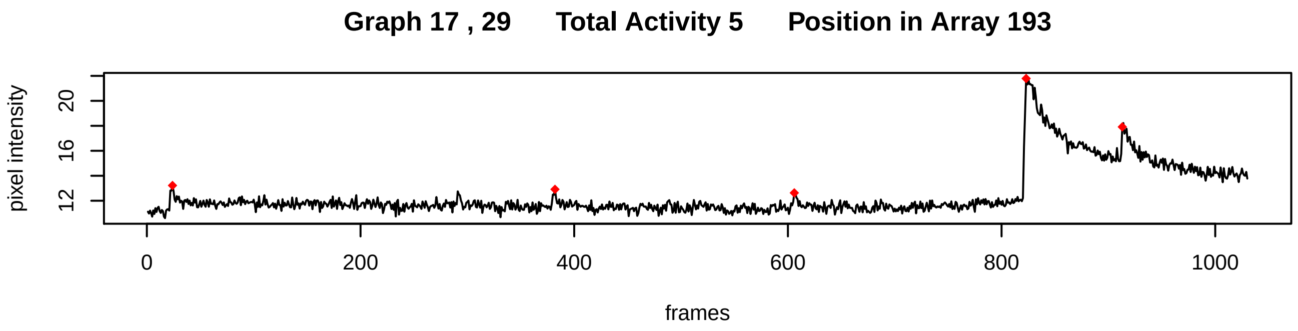
Graph 17 , 29 Total Activity 5 Position in Array 193
pixel intensity
12
16
20
0
200
400
600
800
1000
frames

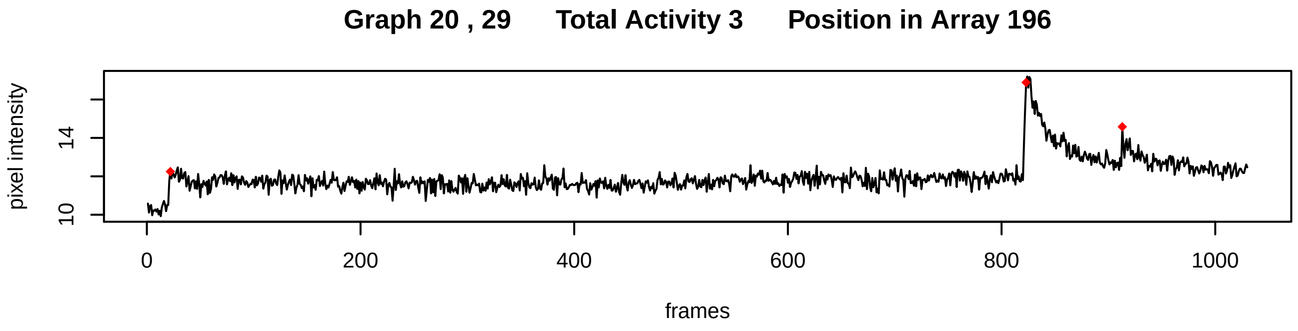
Graph 20 , 29 Total Activity 3 Position in Array 196
pixel intensity
10
14
0
200
400
600
800
1000
frames

Graph 24 , 29   Total Activity 7   Position in Array 200

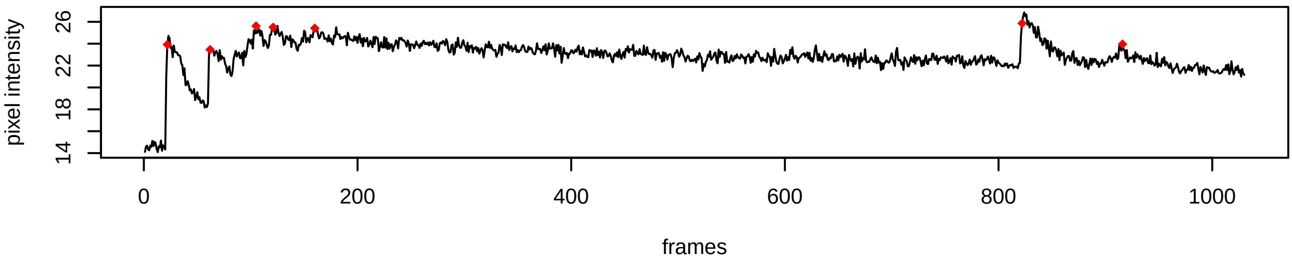

Graph 25 , 29   Total Activity 9   Position in Array 201

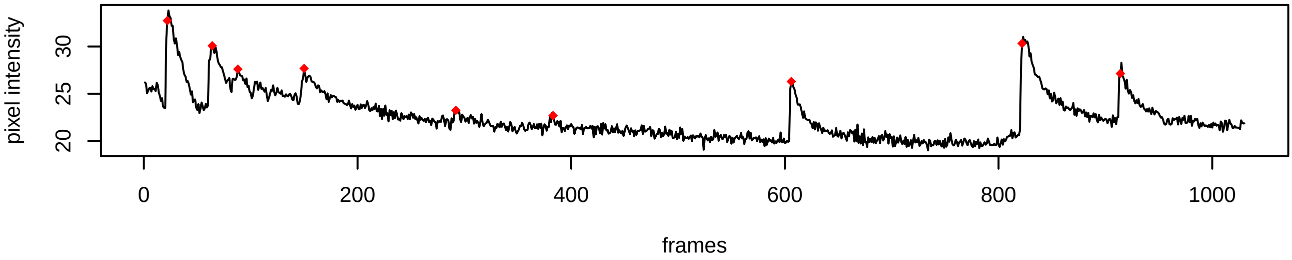

Graph 26 , 29   Total Activity 8   Position in Array 202

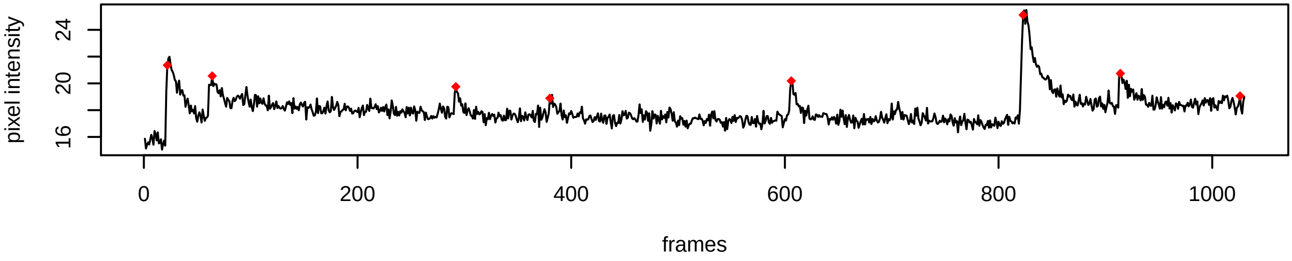

Graph 27 , 29   Total Activity 5   Position in Array 203

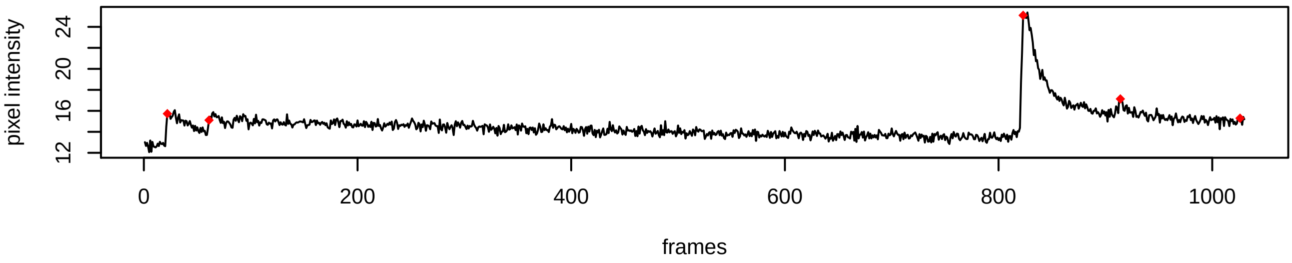

Graph 30 , 29   Total Activity 5   Position in Array 206

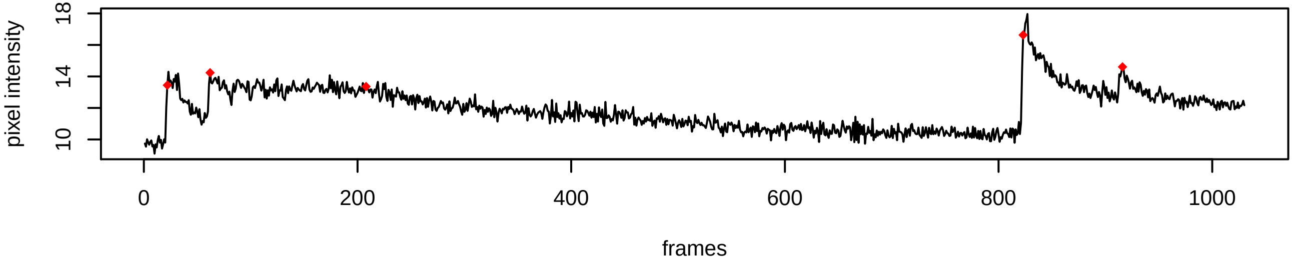

Graph 31 , 29   Total Activity 6   Position in Array 207

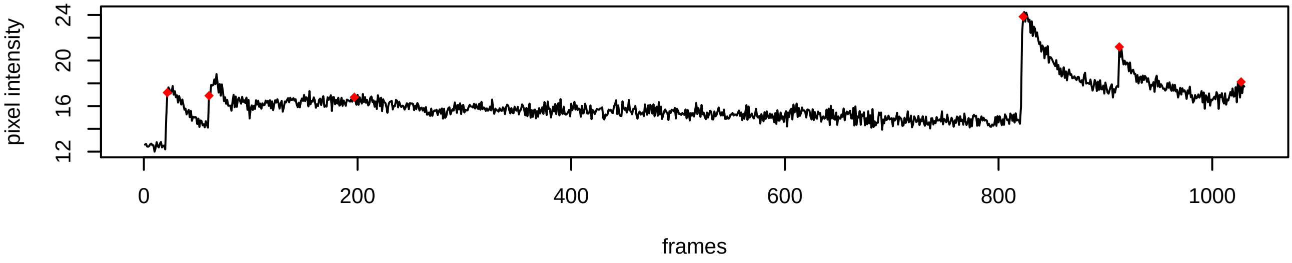

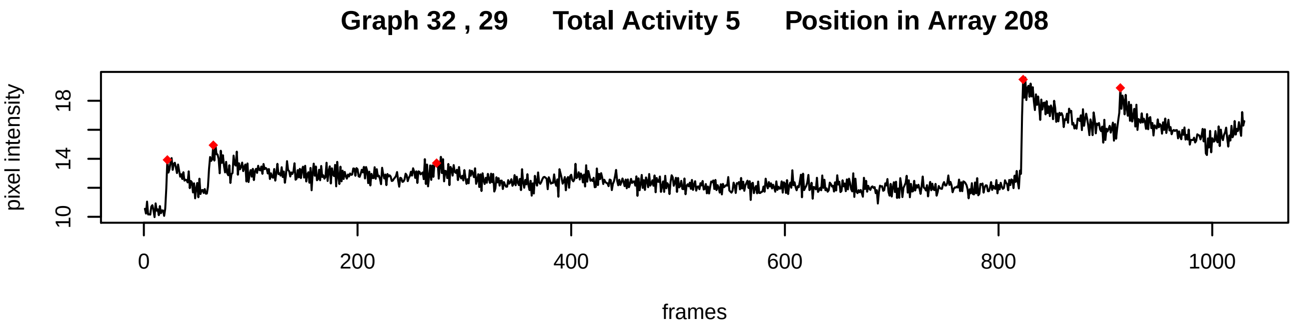
Graph 32 , 29 Total Activity 5 Position in Array 208
pixel intensity
10
14
18
0
200
400
600
800
1000
frames

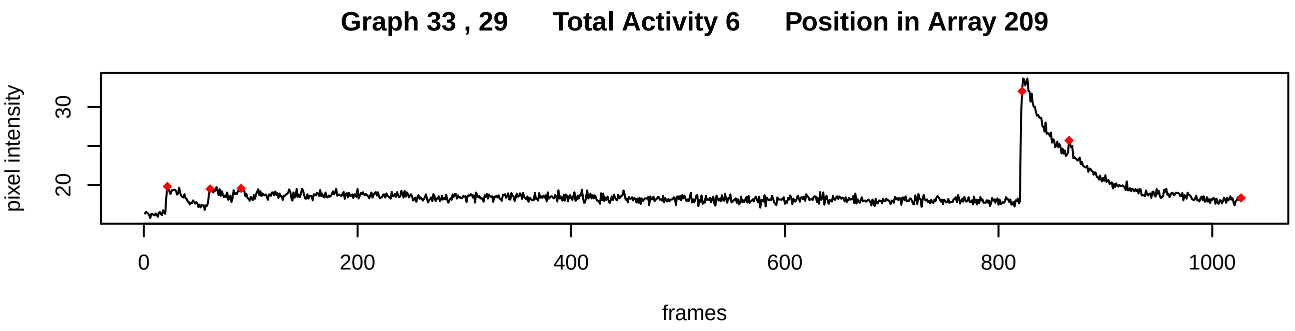
Graph 33 , 29 Total Activity 6 Position in Array 209
pixel intensity
20
30
0
200
400
600
800
1000
frames

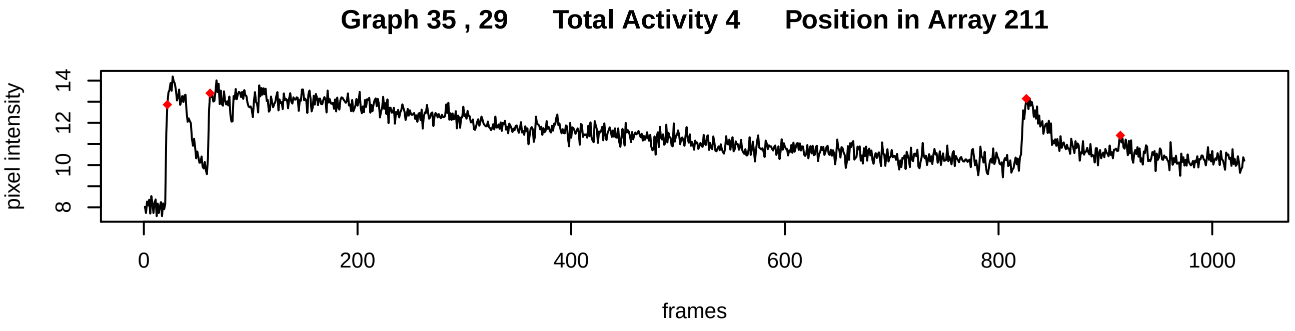
Graph 35 , 29 Total Activity 4 Position in Array 211
pixel intensity
8
10
12
14
0
200
400
600
800
1000
frames

Graph 38 , 29   Total Activity 9   Position in Array 214

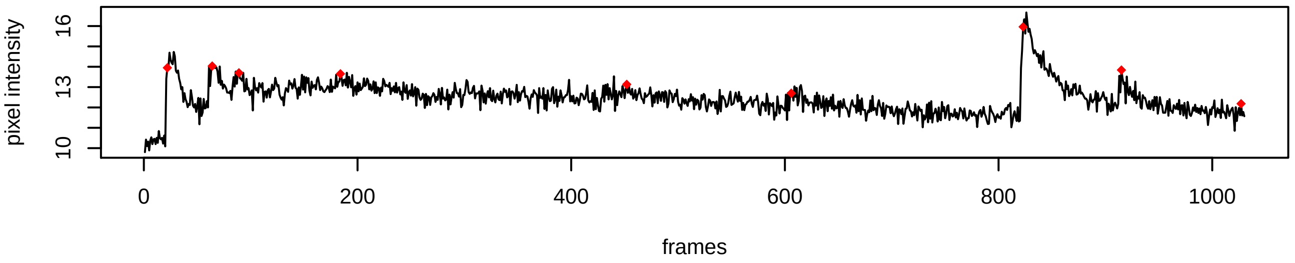

Graph 41 , 29   Total Activity 4   Position in Array 217

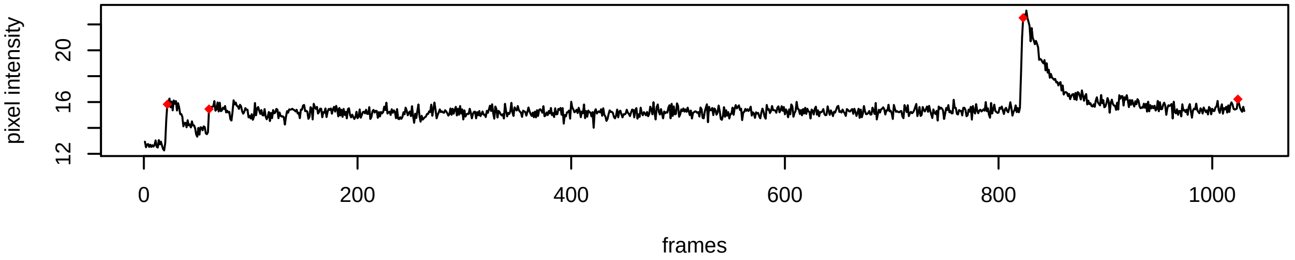

Graph 42 , 29   Total Activity 7   Position in Array 218

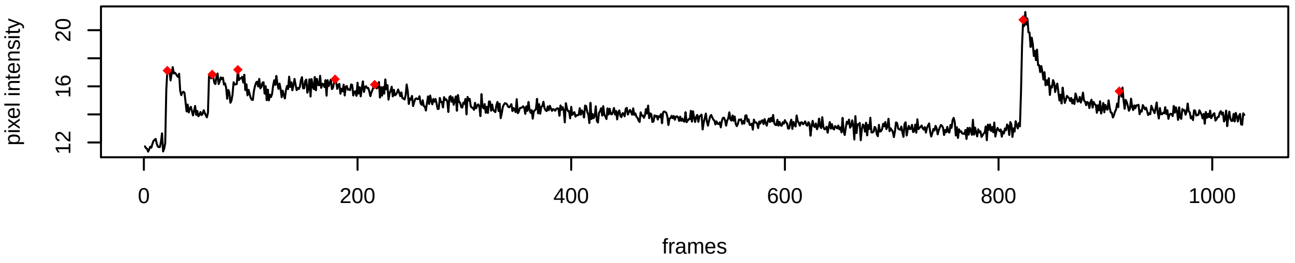

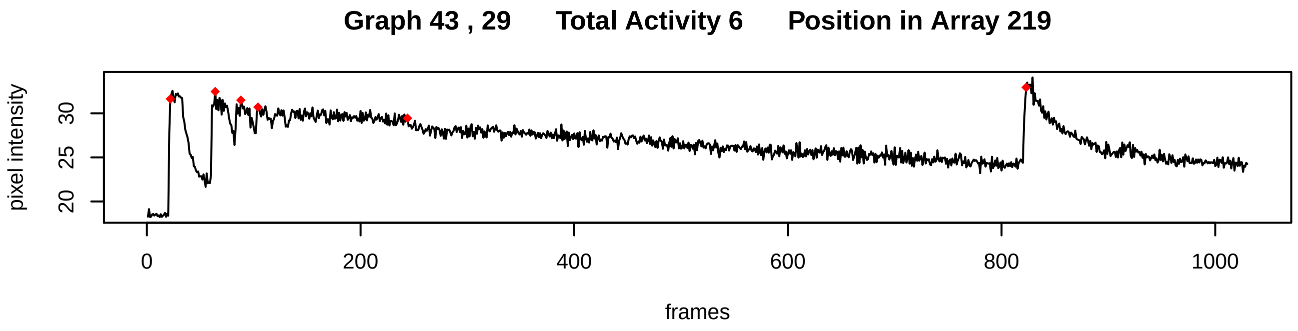
Graph 43 , 29 Total Activity 6 Position in Array 219
pixel intensity
20
25
30
0
200
400
600
800
1000
frames

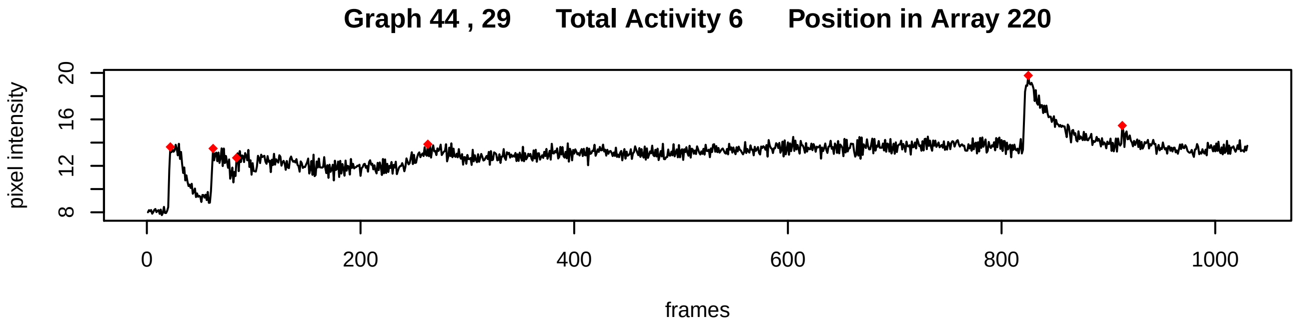
Graph 44 , 29 Total Activity 6 Position in Array 220
pixel intensity
8
12
16
20
0
200
400
600
800
1000
frames

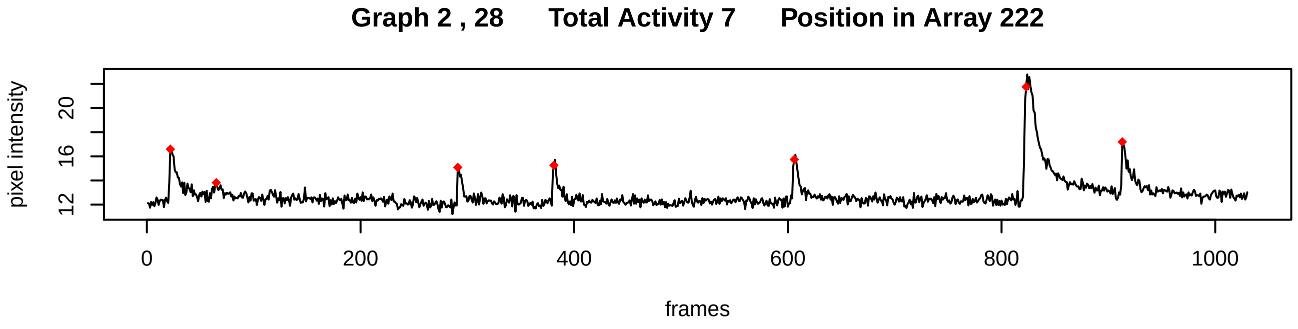
Graph 2 , 28 Total Activity 7 Position in Array 222
pixel intensity
12
16
20
0
200
400
600
800
1000
frames

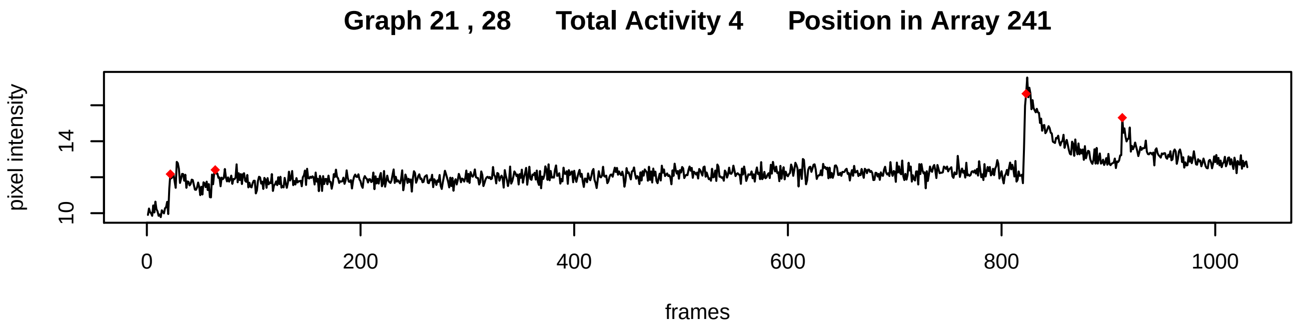
Graph 21 , 28 Total Activity 4 Position in Array 241
pixel intensity
10
14
0
200
400
600
800
1000
frames

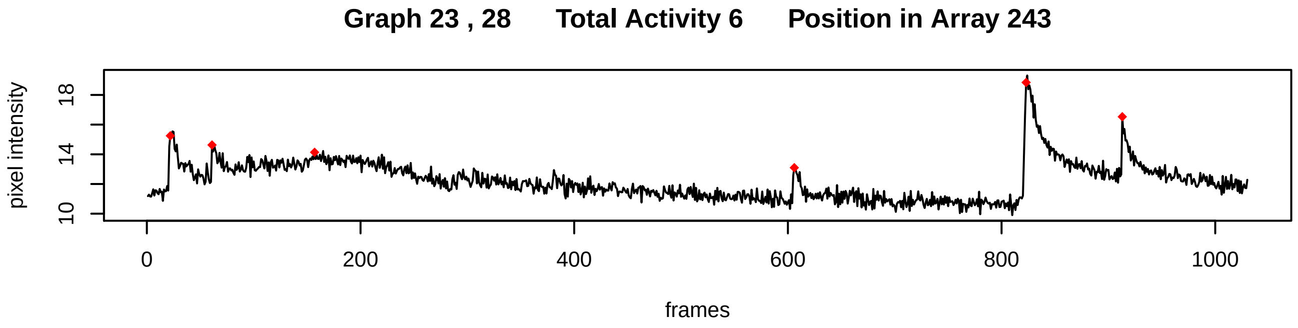
Graph 23 , 28 Total Activity 6 Position in Array 243
pixel intensity
10
14
18
0
200
400
600
800
1000
frames

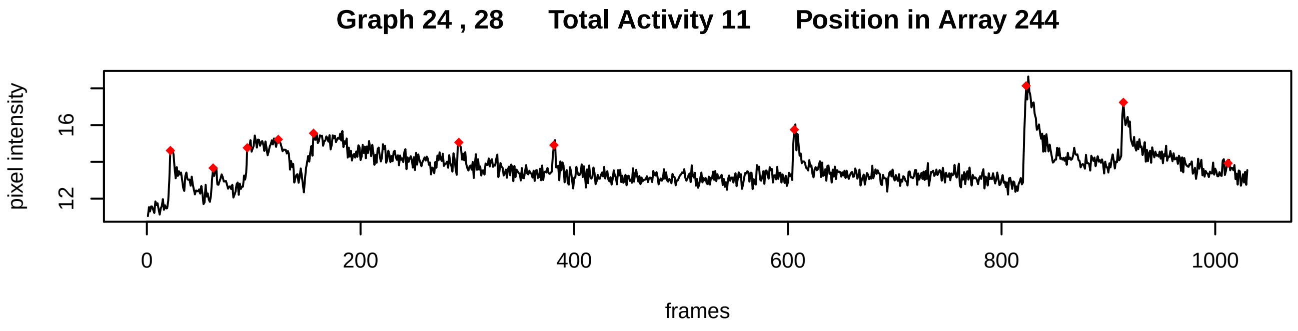
Graph 24 , 28 Total Activity 11 Position in Array 244
pixel intensity
12
16
0
200
400
600
800
1000
frames

Graph 25 , 28 Total Activity 6 Position in Array 245

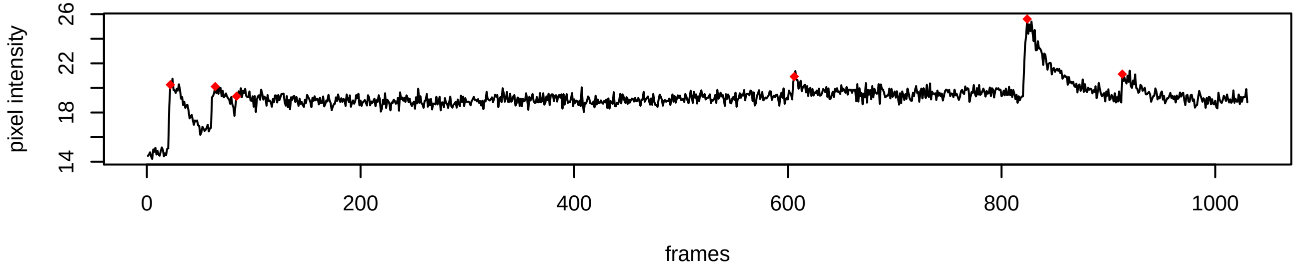

Graph 26 , 28 Total Activity 7 Position in Array 246

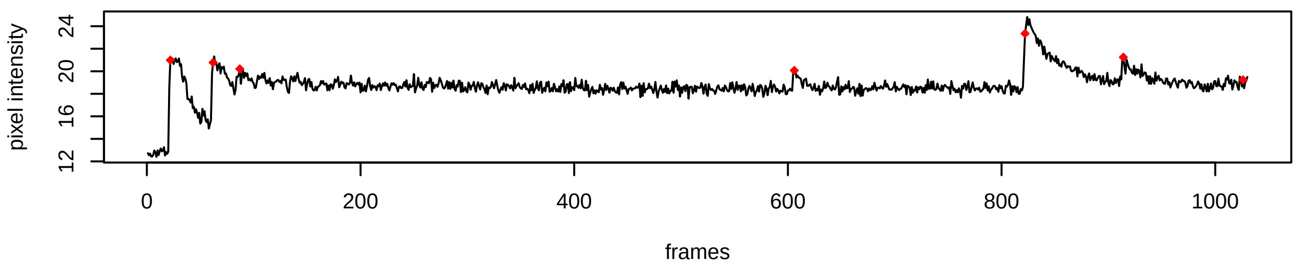

Graph 27 , 28 Total Activity 6 Position in Array 247

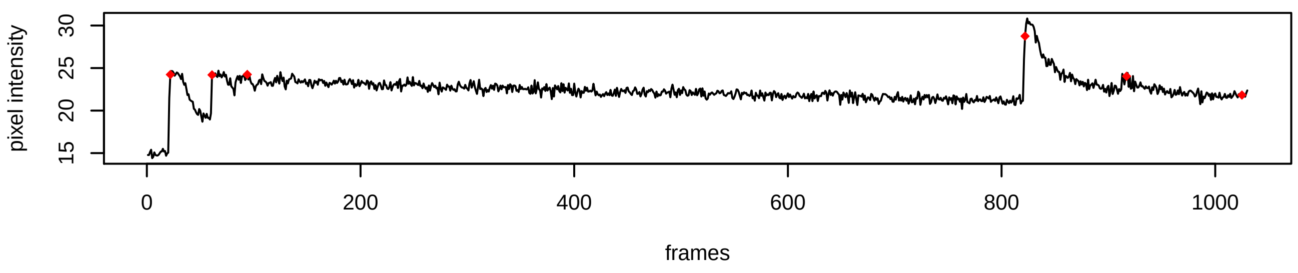

Graph 28 , 28 Total Activity 5 Position in Array 248

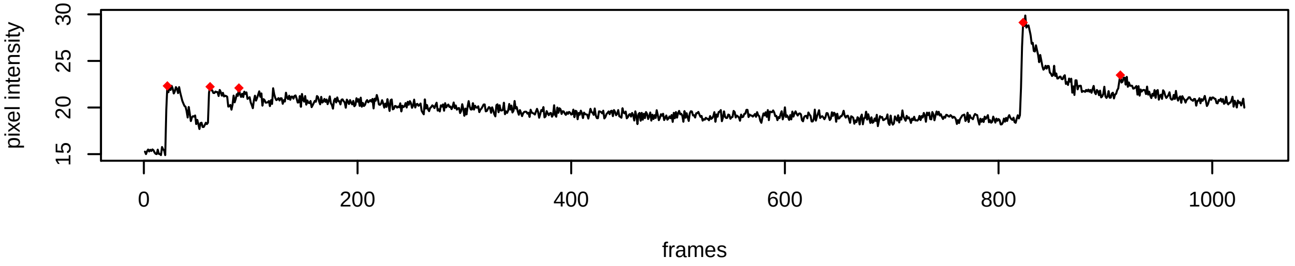

Graph 29 , 28 Total Activity 5 Position in Array 249

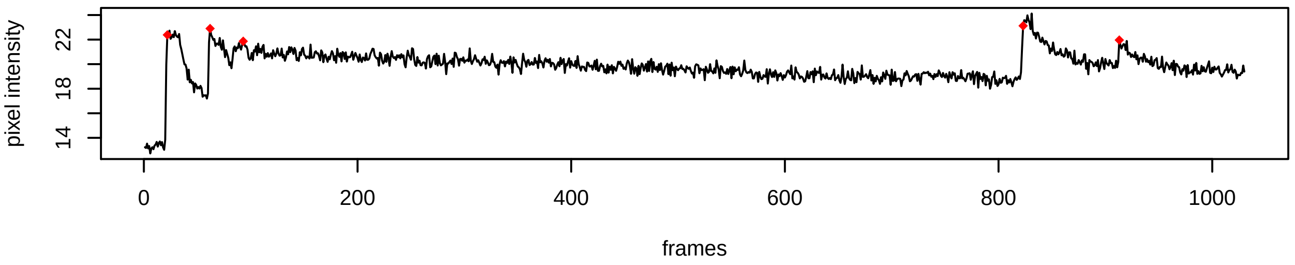

Graph 30 , 28 Total Activity 6 Position in Array 250

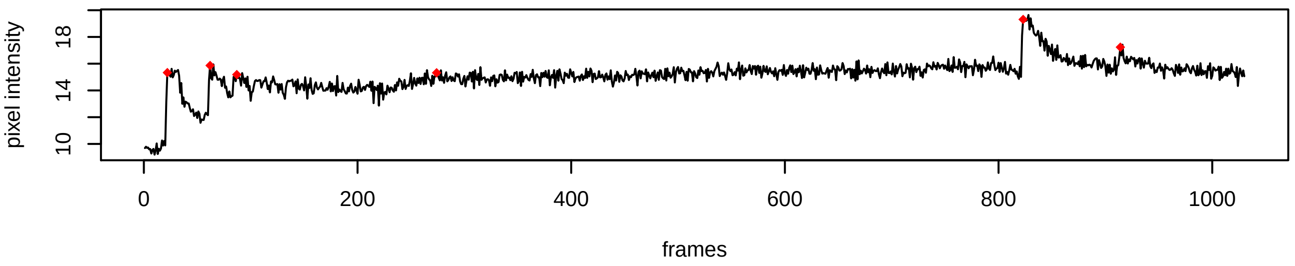

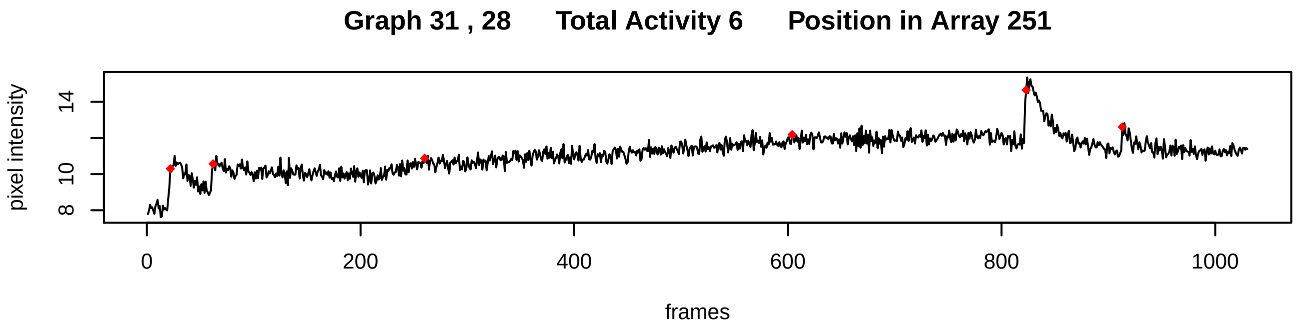
Graph 31 , 28 Total Activity 6 Position in Array 251
pixel intensity
8
10
14
0
200
400
600
800
1000
frames

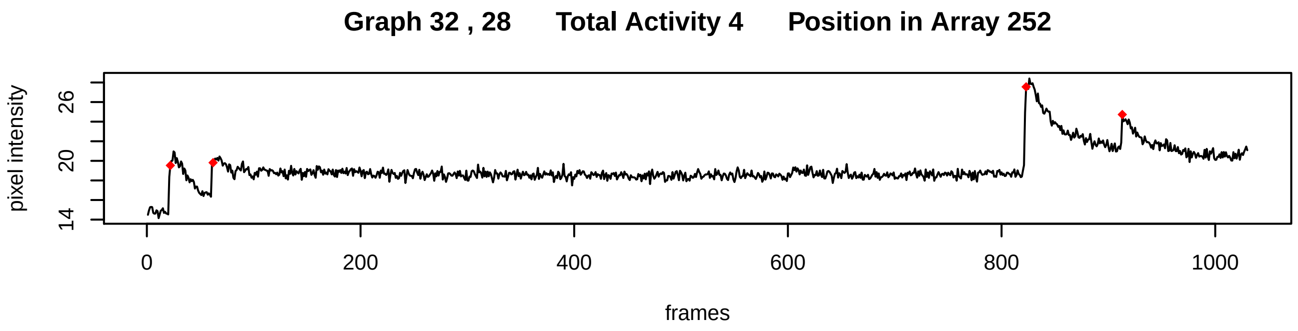
Graph 32 , 28 Total Activity 4 Position in Array 252
pixel intensity
14
20
26
0
200
400
600
800
1000
frames

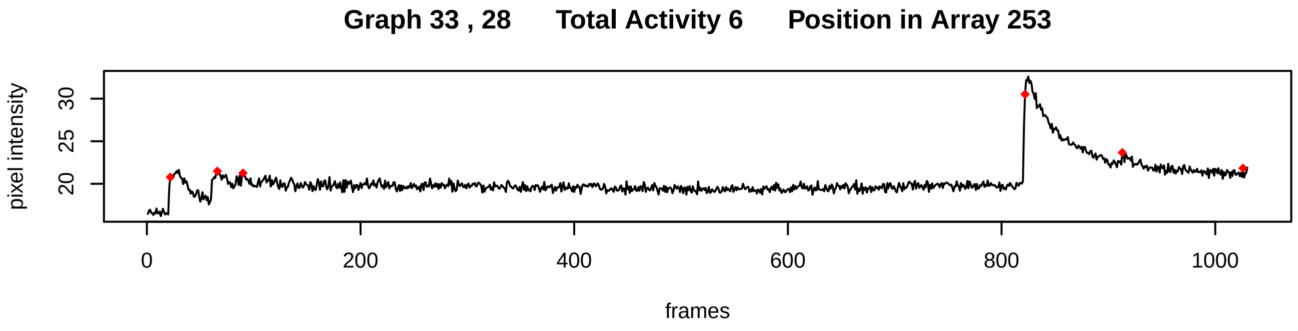
Graph 33 , 28 Total Activity 6 Position in Array 253
pixel intensity
20
25
30
0
200
400
600
800
1000
frames

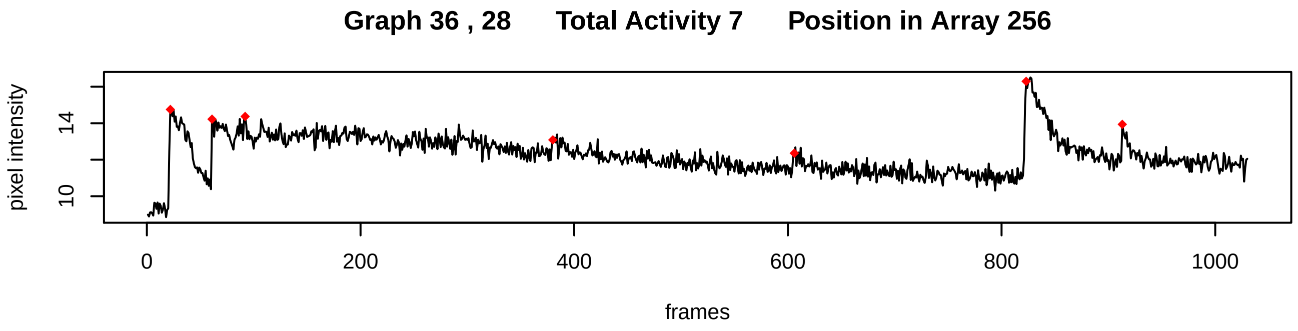
Graph 36 , 28 Total Activity 7 Position in Array 256
pixel intensity
10
14
0
200
400
600
800
1000
frames

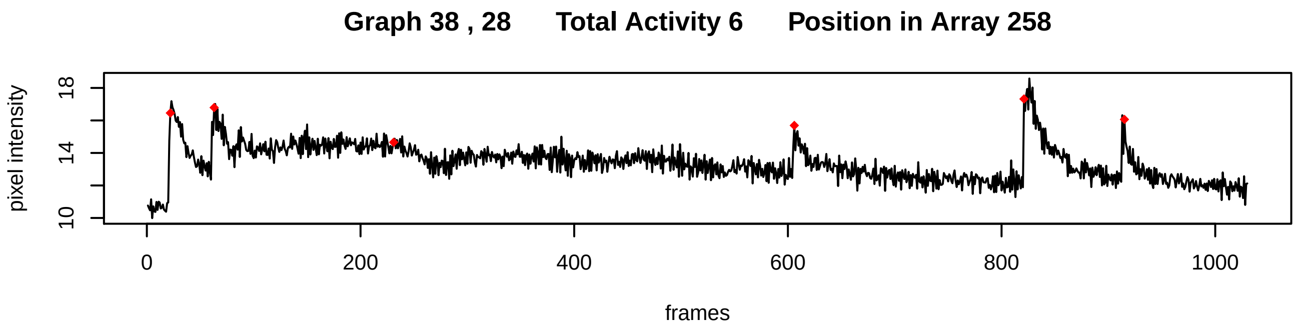
Graph 38 , 28 Total Activity 6 Position in Array 258
pixel intensity
10
14
18
0
200
400
600
800
1000
frames

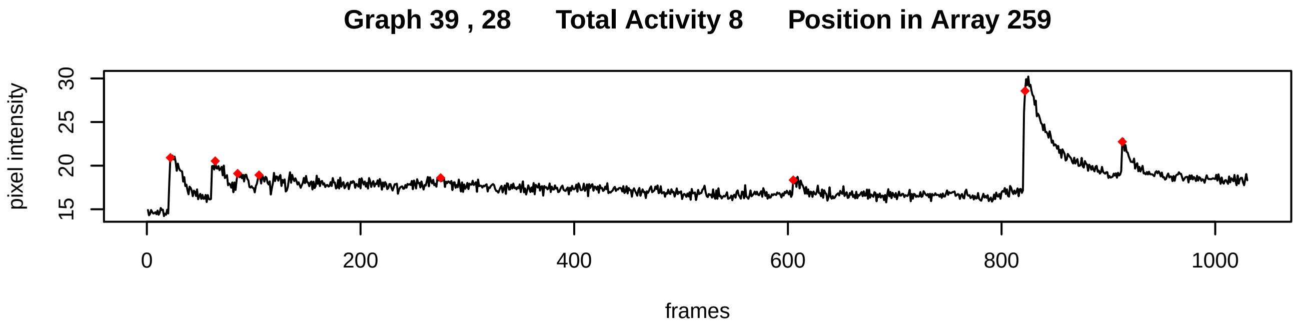
Graph 39 , 28 Total Activity 8 Position in Array 259
pixel intensity
15
20
25
30
0
200
400
600
800
1000
frames

Graph 40 , 28 Total Activity 5 Position in Array 260

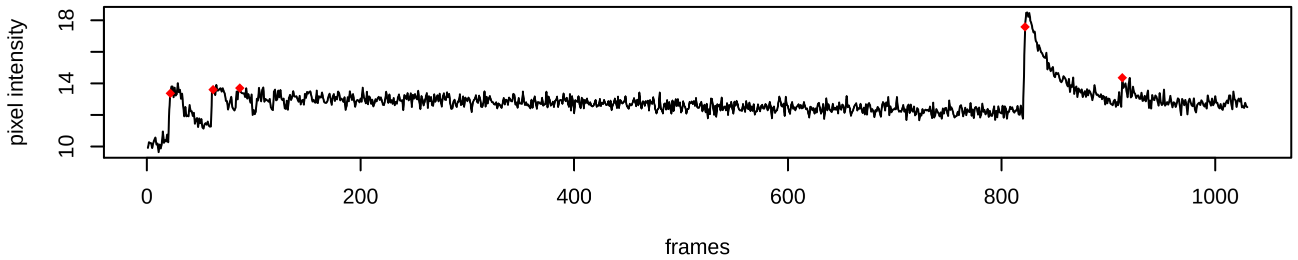

Graph 41 , 28 Total Activity 7 Position in Array 261

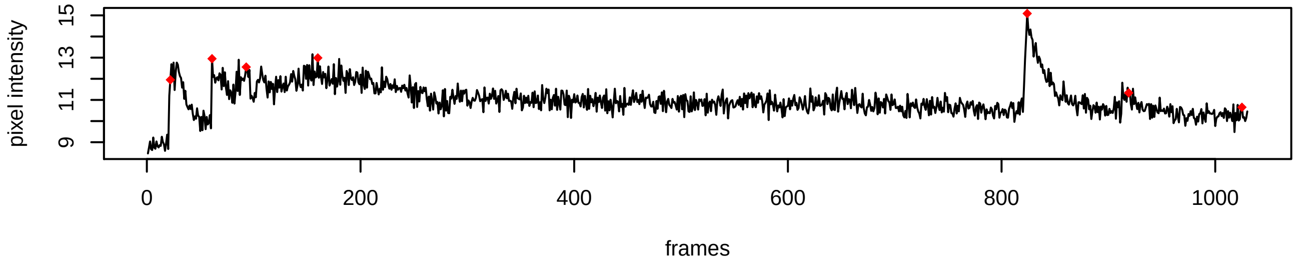

Graph 42 , 28 Total Activity 6 Position in Array 262

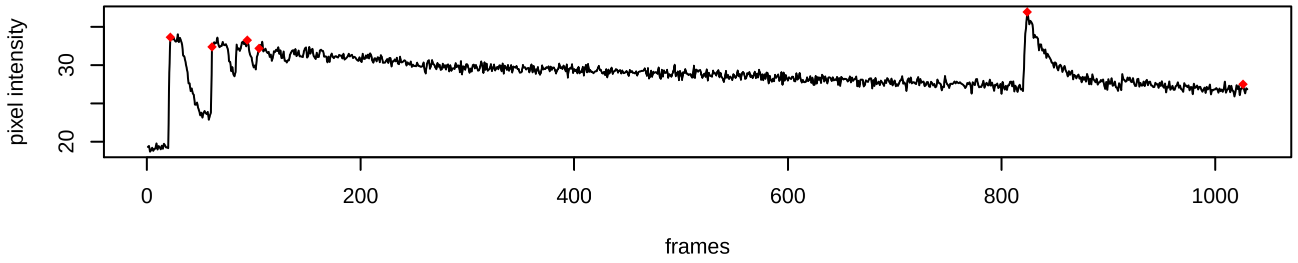

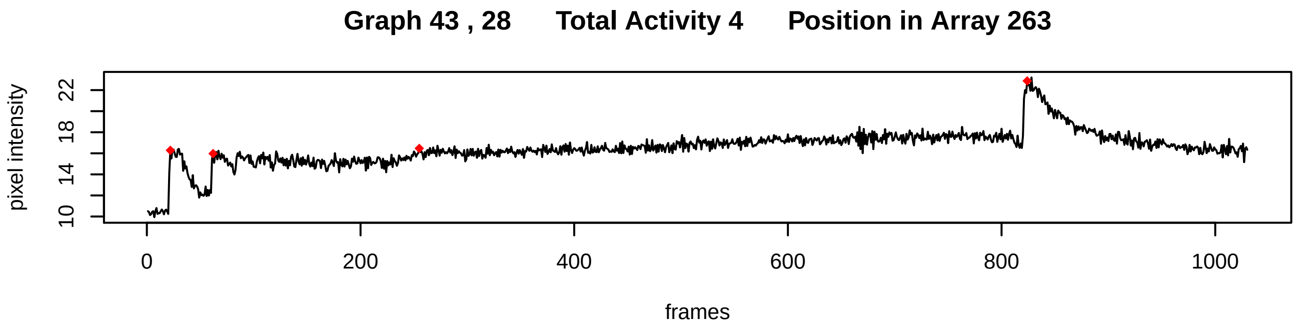
Graph 43 , 28 Total Activity 4 Position in Array 263
pixel intensity
10
14
18
22
0
200
400
600
800
1000
frames

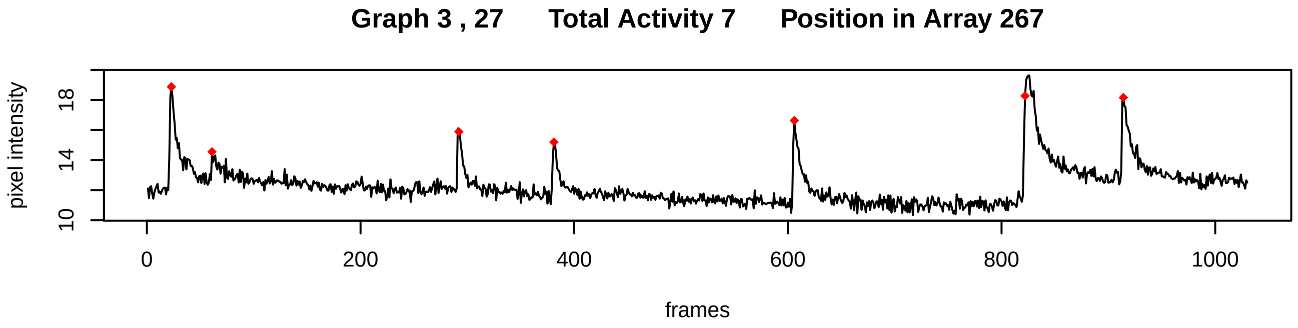
Graph 3 , 27 Total Activity 7 Position in Array 267
pixel intensity
10
14
18
0
200
400
600
800
1000
frames

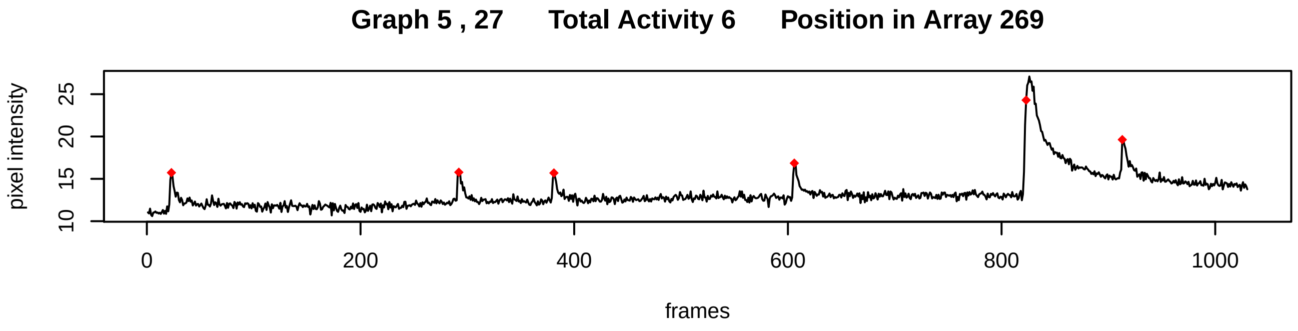
Graph 5 , 27 Total Activity 6 Position in Array 269
pixel intensity
10
15
20
25
0
200
400
600
800
1000
frames

Graph 17 , 27 Total Activity 8 Position in Array 281

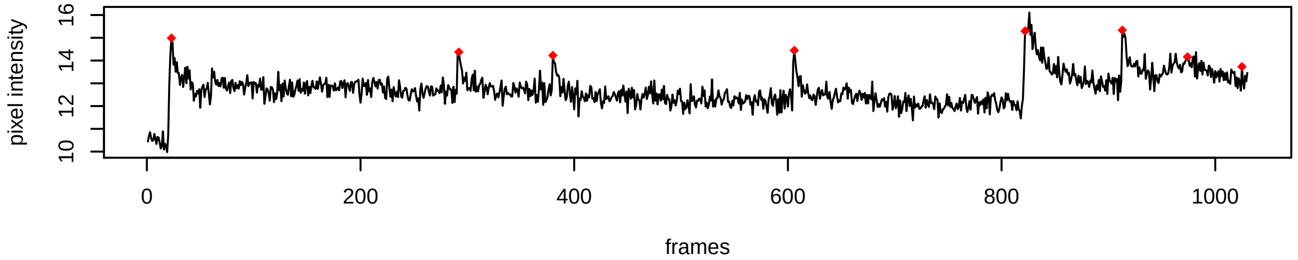

Graph 22 , 27 Total Activity 5 Position in Array 286

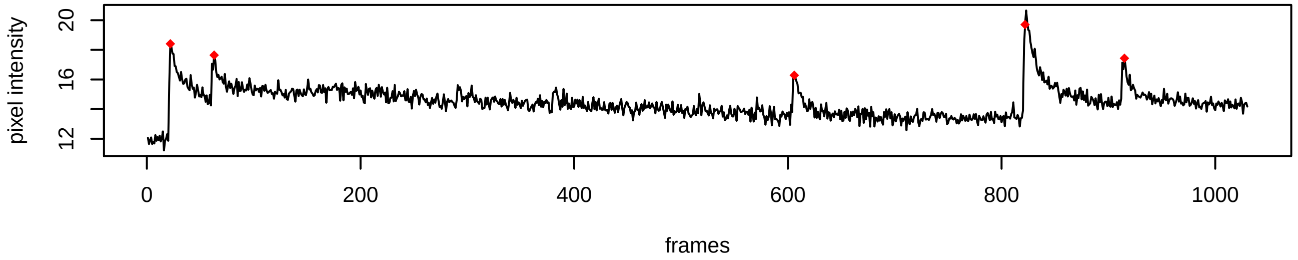

Graph 23 , 27 Total Activity 10 Position in Array 287

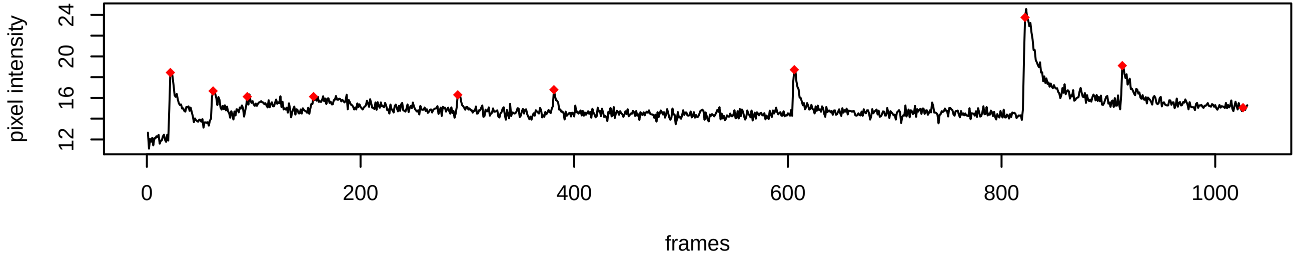

Graph 24 , 27 Total Activity 10 Position in Array 288

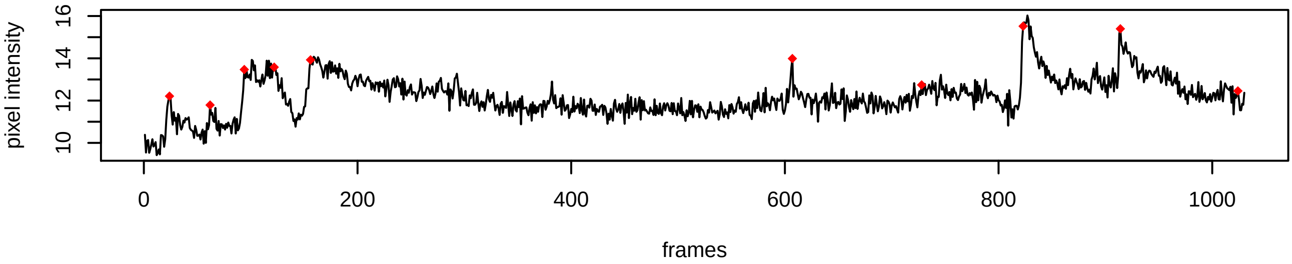

Graph 25 , 27 Total Activity 5 Position in Array 289

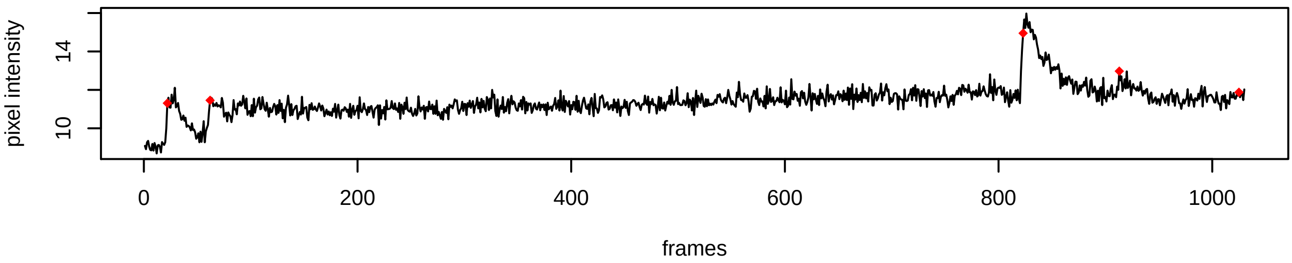

Graph 26 , 27 Total Activity 6 Position in Array 290

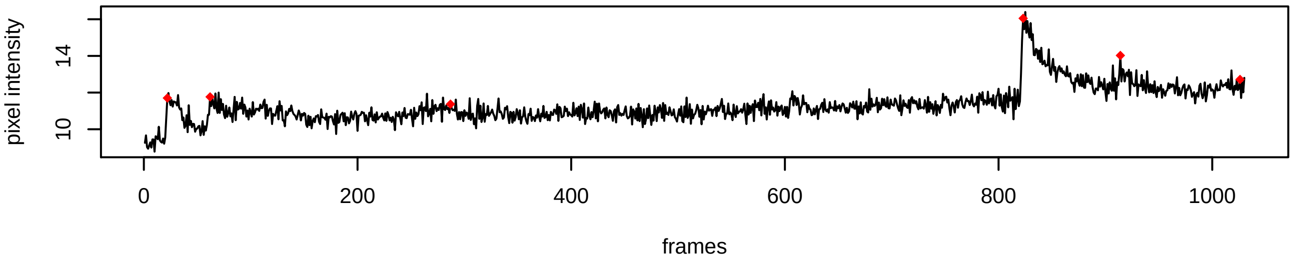

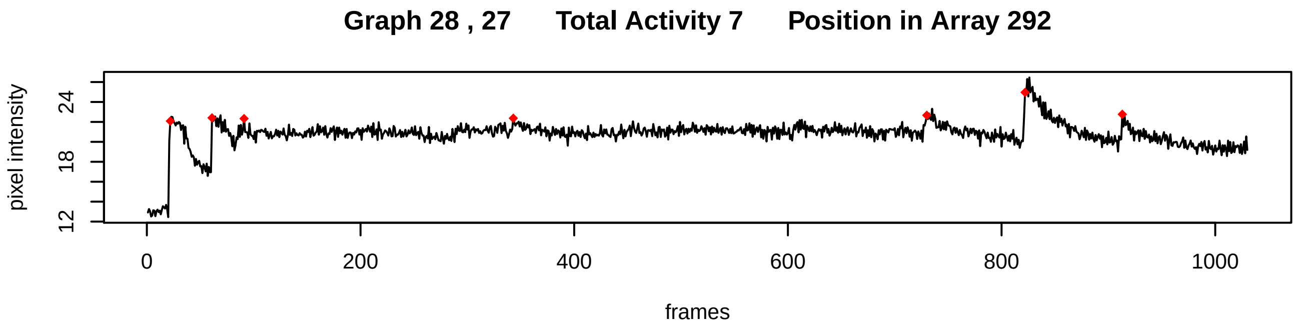
Graph 28 , 27 Total Activity 7 Position in Array 292
pixel intensity
12
18
24
0
200
400
600
800
1000
frames

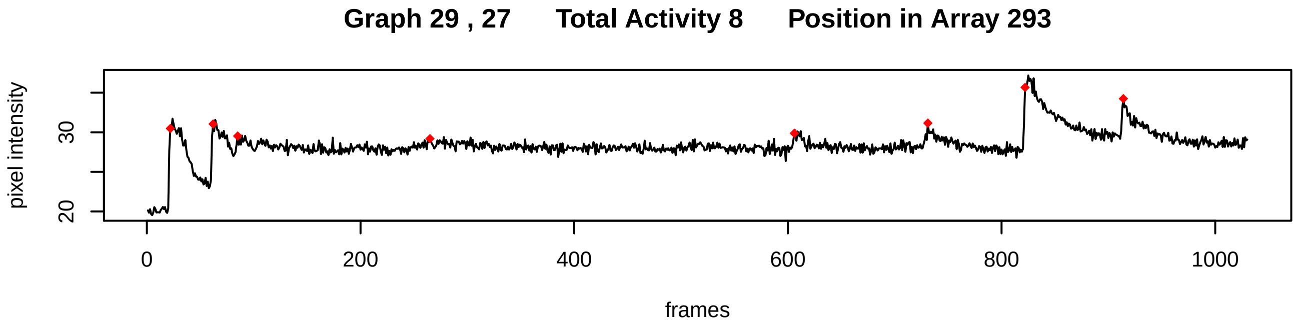
Graph 29 , 27 Total Activity 8 Position in Array 293
pixel intensity
20
30
0
200
400
600
800
1000
frames

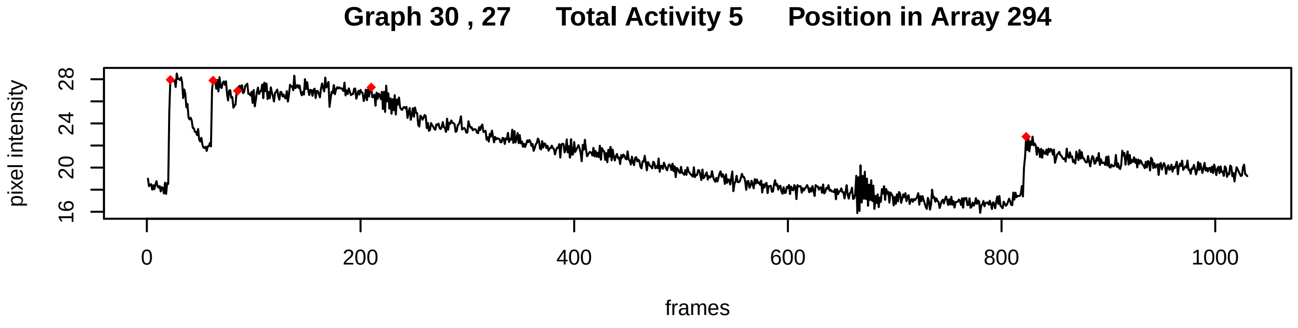
Graph 30 , 27 Total Activity 5 Position in Array 294
pixel intensity
16
20
24
28
0
200
400
600
800
1000
frames

Graph 31 , 27   Total Activity 5   Position in Array 295

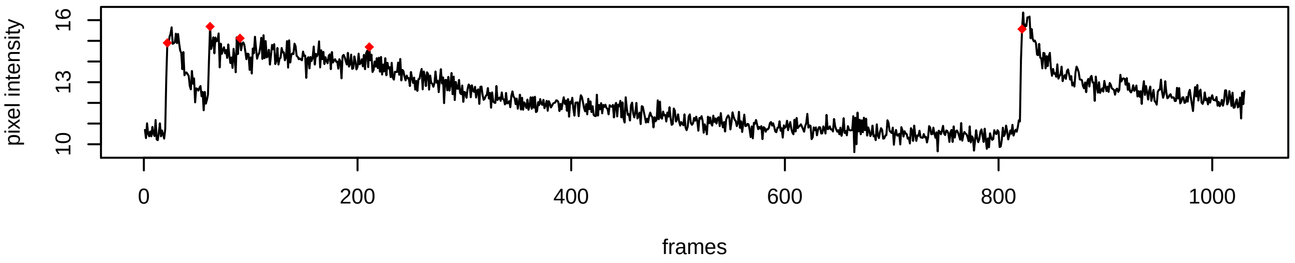

Graph 32 , 27   Total Activity 6   Position in Array 296

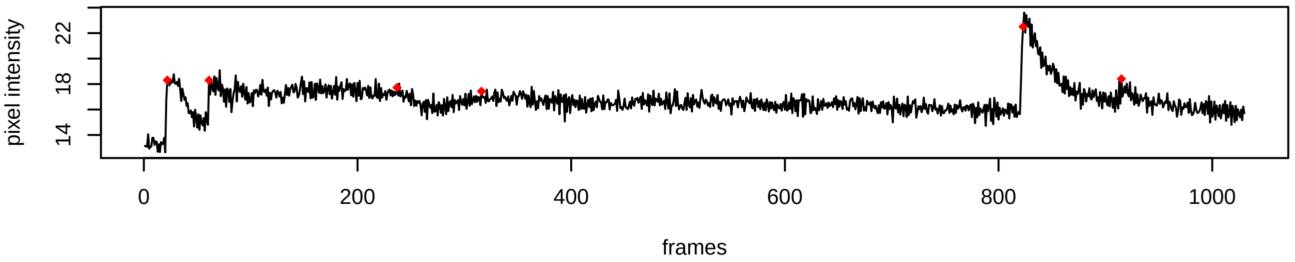

Graph 33 , 27   Total Activity 7   Position in Array 297

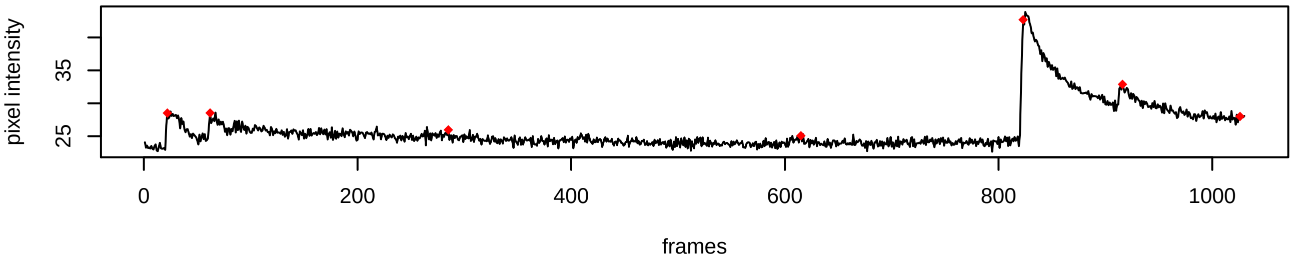

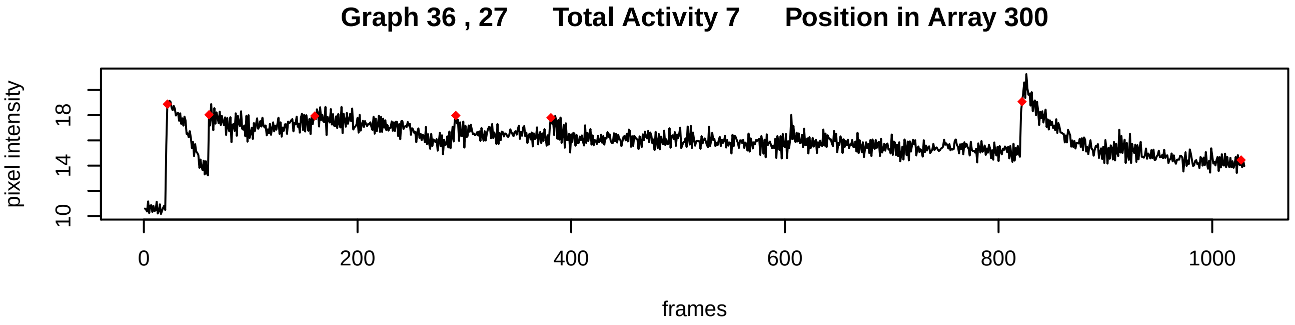
Graph 36 , 27 Total Activity 7 Position in Array 300
pixel intensity
10
14
18
0
200
400
600
800
1000
frames

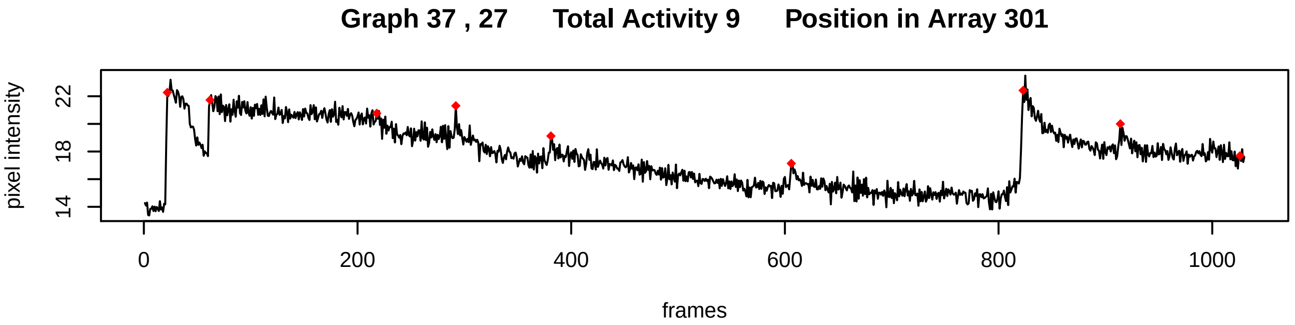
Graph 37 , 27 Total Activity 9 Position in Array 301
pixel intensity
14
18
22
0
200
400
600
800
1000
frames

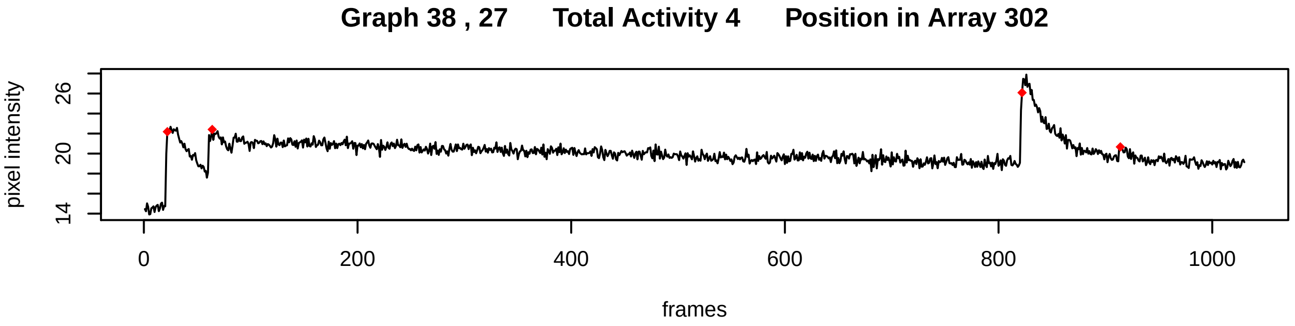
Graph 38 , 27 Total Activity 4 Position in Array 302
pixel intensity
14
20
26
0
200
400
600
800
1000
frames

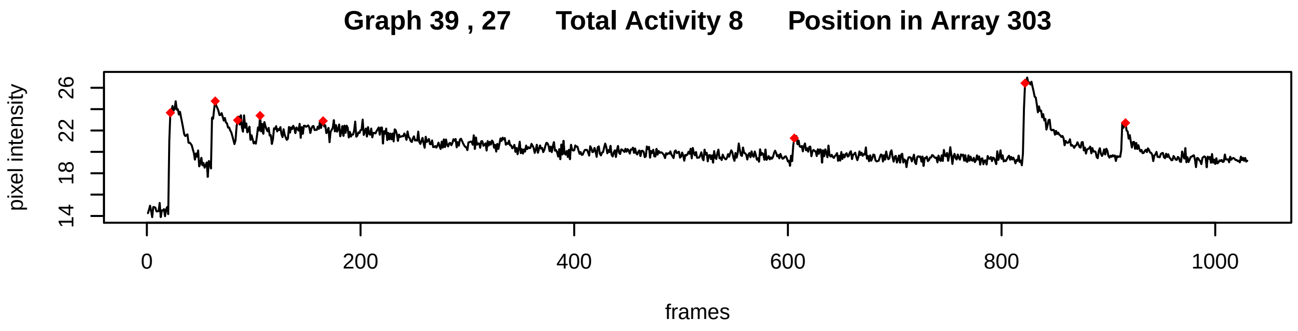
Graph 39 , 27 Total Activity 8 Position in Array 303
pixel intensity
14
18
22
26
0
200
400
600
800
1000
frames

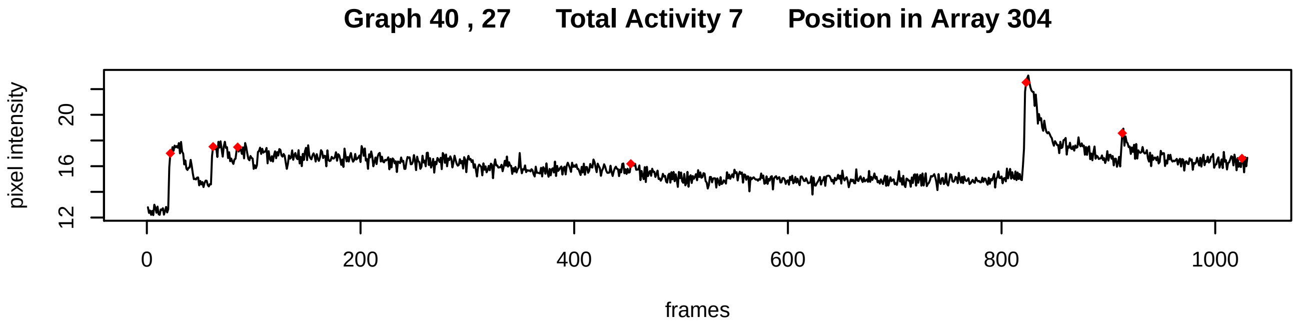
Graph 40 , 27 Total Activity 7 Position in Array 304
pixel intensity
12
16
20
0
200
400
600
800
1000
frames

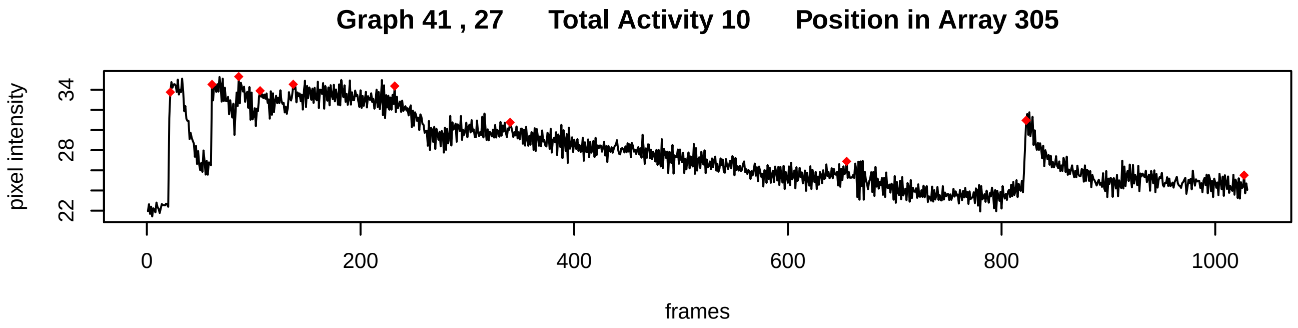
Graph 41 , 27 Total Activity 10 Position in Array 305
pixel intensity
22
28
34
0
200
400
600
800
1000
frames

Graph 42 , 27   Total Activity 7   Position in Array 306

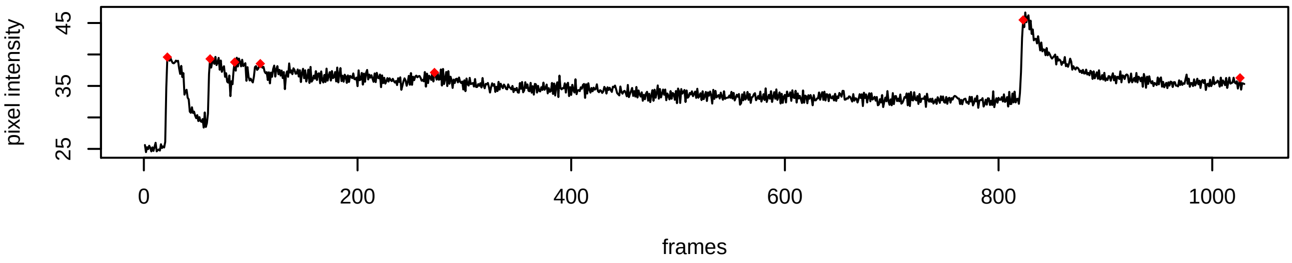

Graph 44 , 27   Total Activity 6   Position in Array 308

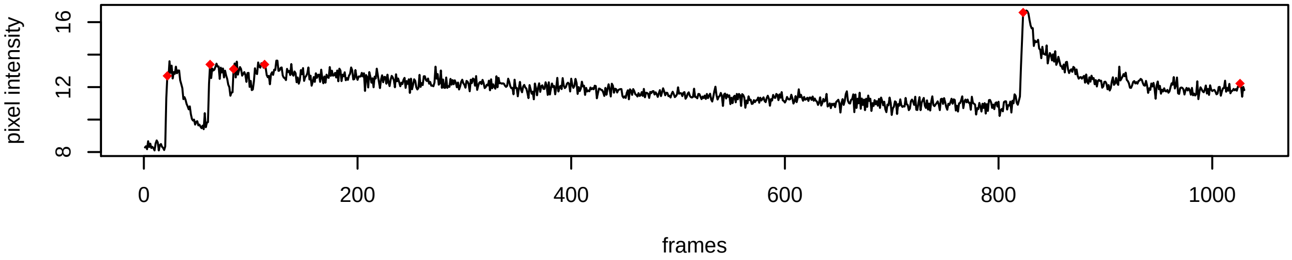

Graph 1 , 26   Total Activity 6   Position in Array 309

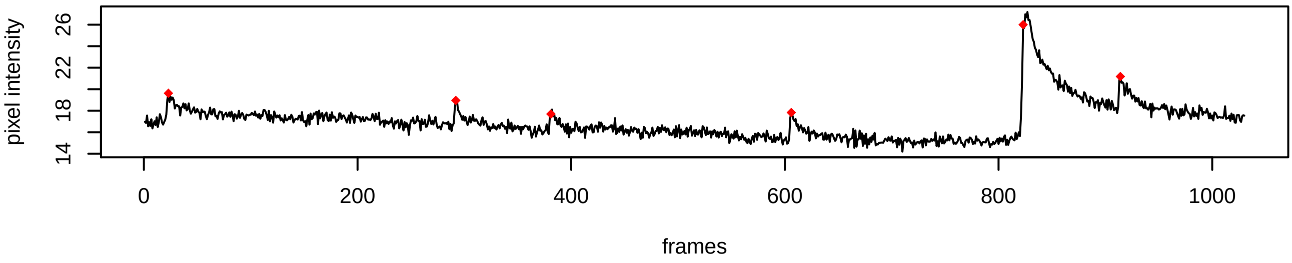

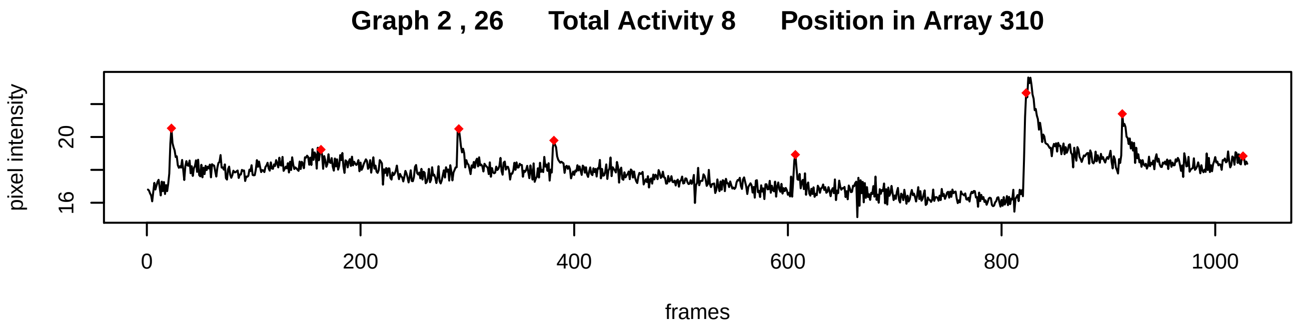
Graph 2 , 26 Total Activity 8 Position in Array 310
pixel intensity
16
20
0
200
400
600
800
1000
frames

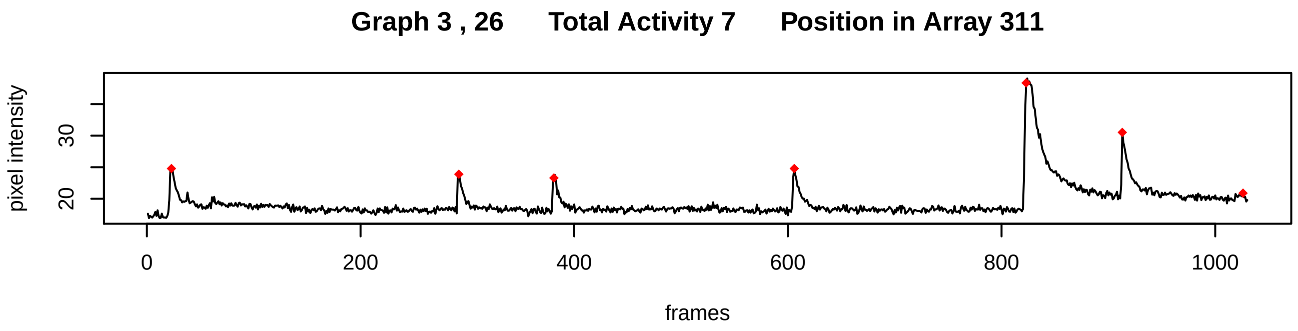
Graph 3 , 26 Total Activity 7 Position in Array 311
pixel intensity
20
30
0
200
400
600
800
1000
frames

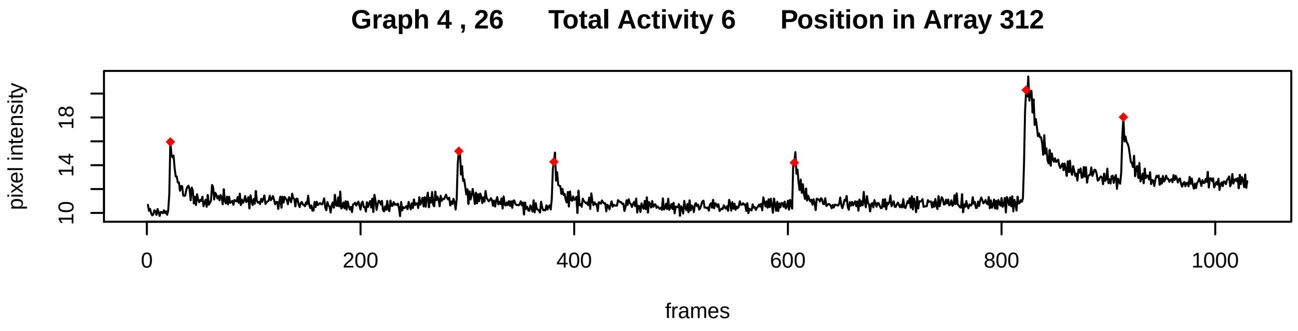
Graph 4 , 26 Total Activity 6 Position in Array 312
pixel intensity
10
14
18
0
200
400
600
800
1000
frames

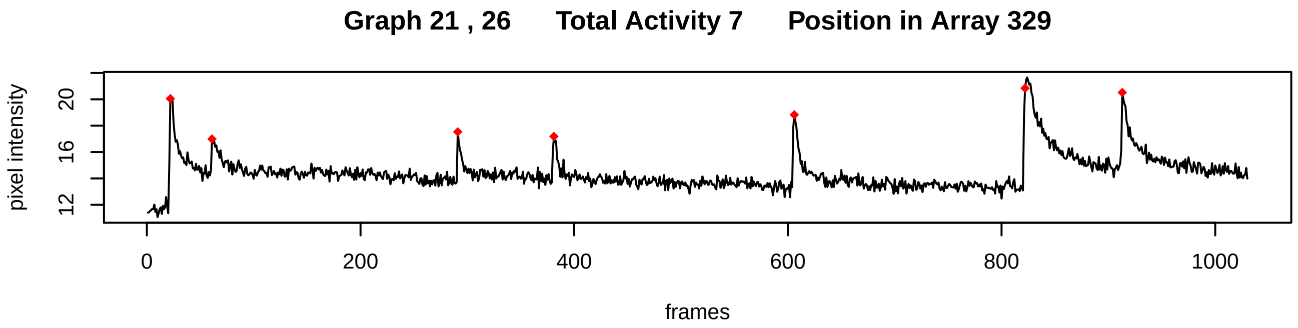
Graph 21 , 26 Total Activity 7 Position in Array 329
pixel intensity
12
16
20
0
200
400
600
800
1000
frames

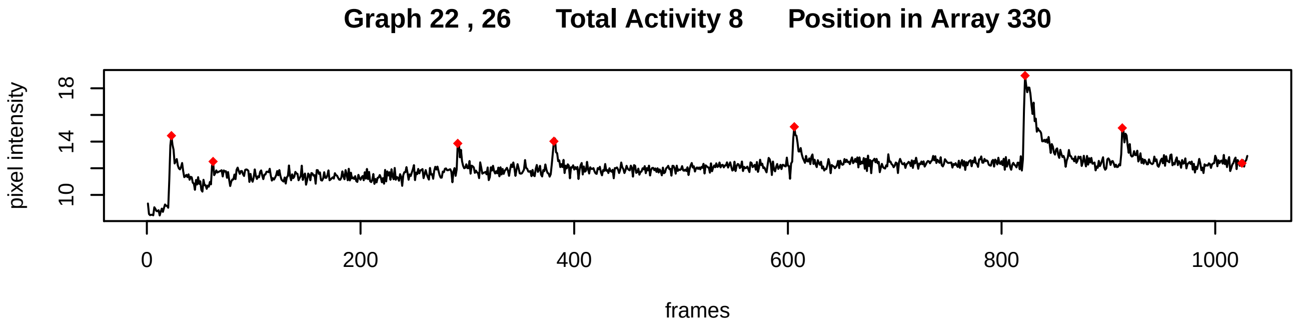
Graph 22 , 26 Total Activity 8 Position in Array 330
pixel intensity
10
14
18
0
200
400
600
800
1000
frames

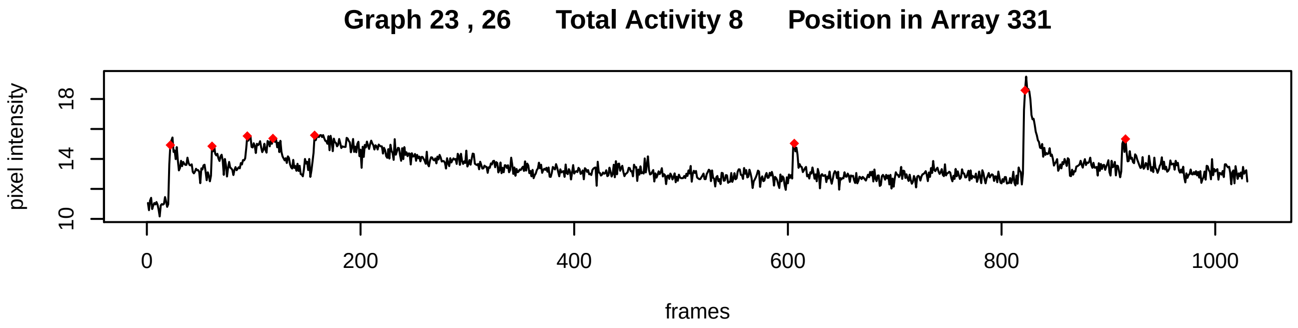
Graph 23 , 26 Total Activity 8 Position in Array 331
pixel intensity
10
14
18
0
200
400
600
800
1000
frames

Graph 28 , 26 Total Activity 6 Position in Array 336

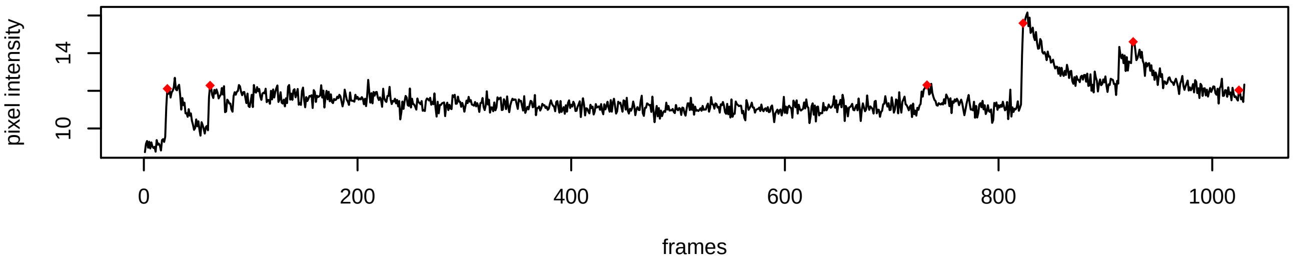

Graph 30 , 26 Total Activity 8 Position in Array 338

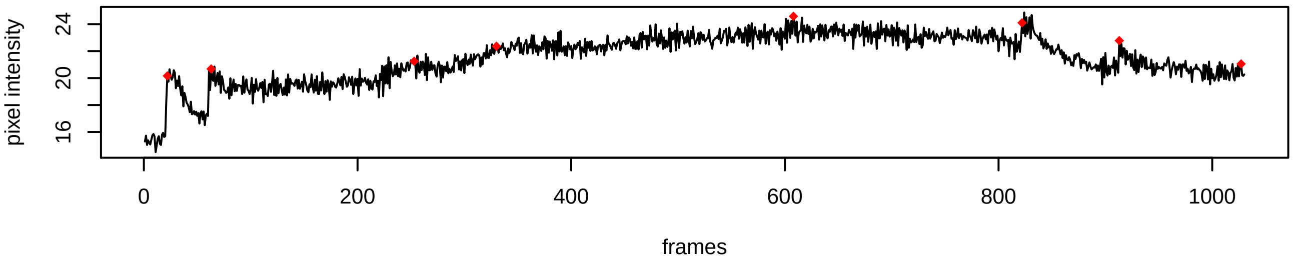

Graph 31 , 26 Total Activity 7 Position in Array 339

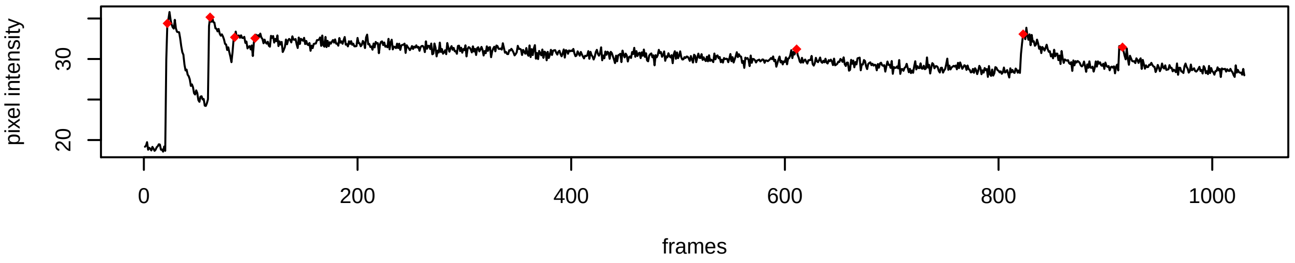

Graph 32 , 26   Total Activity 6   Position in Array 340

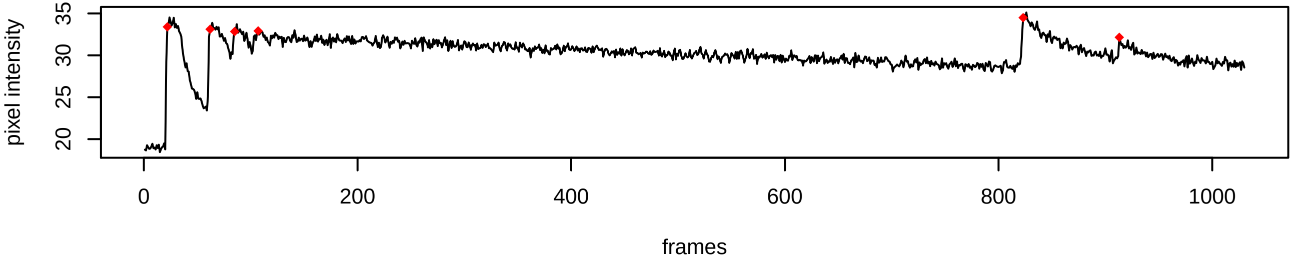

Graph 33 , 26   Total Activity 11   Position in Array 341

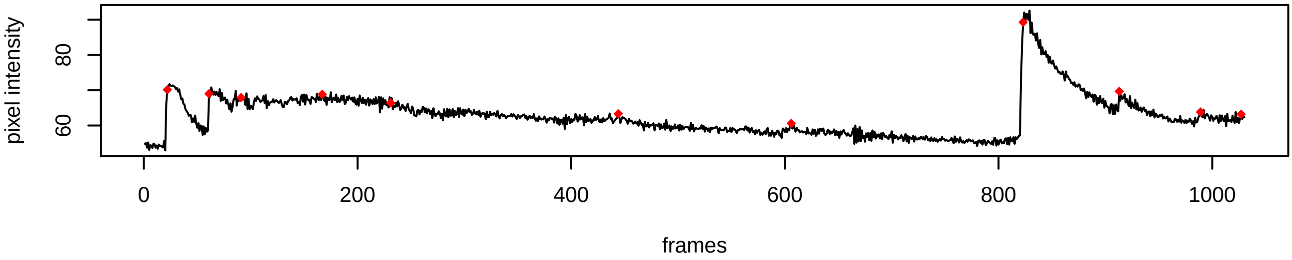

Graph 34 , 26   Total Activity 6   Position in Array 342

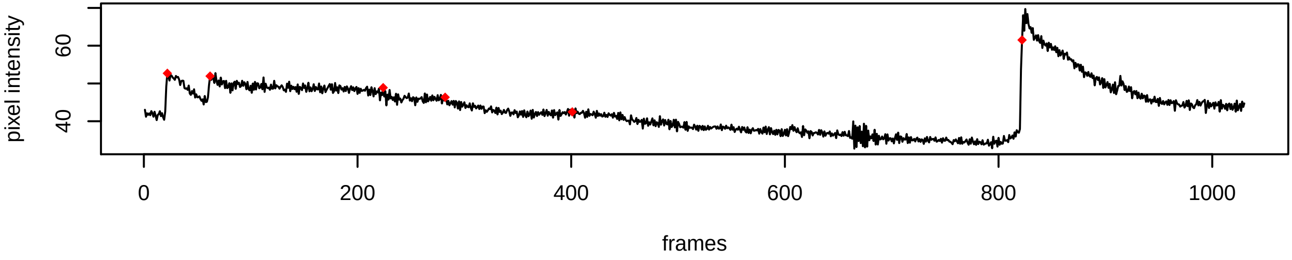

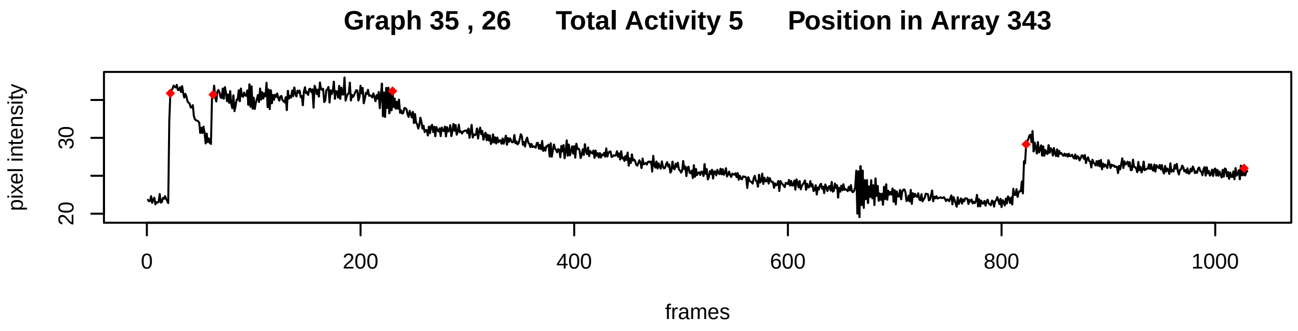
Graph 35 , 26 Total Activity 5 Position in Array 343
pixel intensity
20
30
0
200
400
600
800
1000
frames

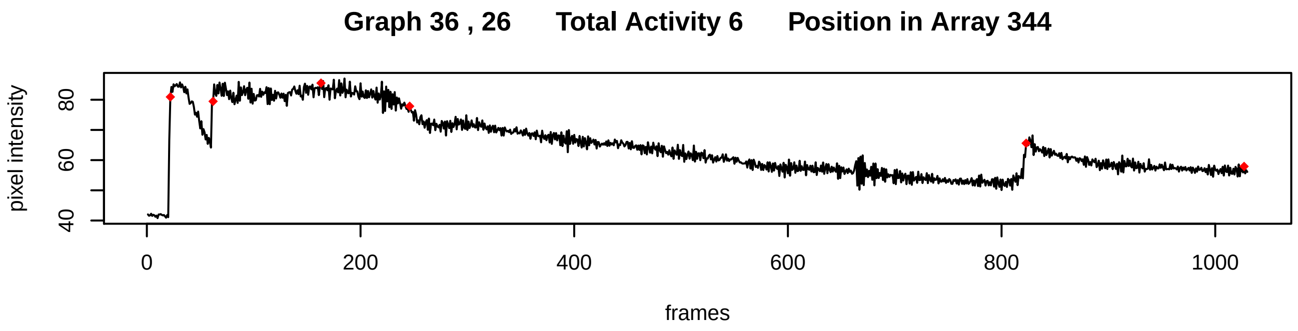
Graph 36 , 26 Total Activity 6 Position in Array 344
pixel intensity
40
60
80
0
200
400
600
800
1000
frames

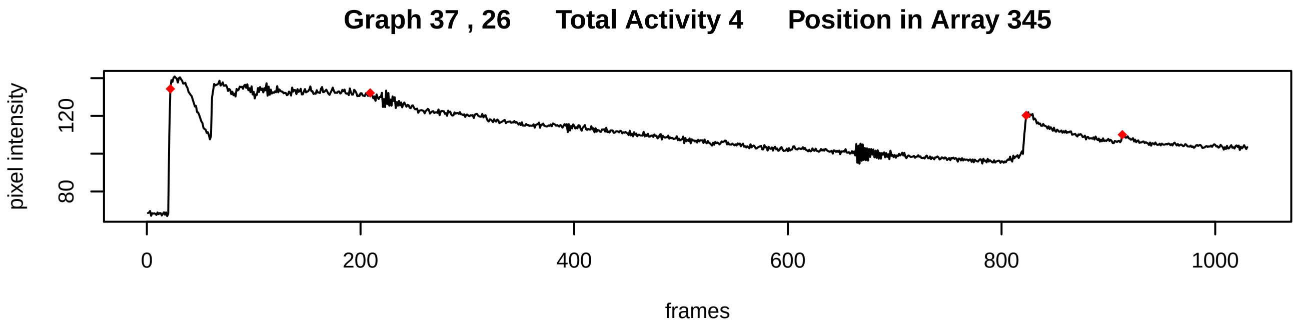
Graph 37 , 26 Total Activity 4 Position in Array 345
pixel intensity
80
120
0
200
400
600
800
1000
frames

Graph 38 , 26   Total Activity 8   Position in Array 346

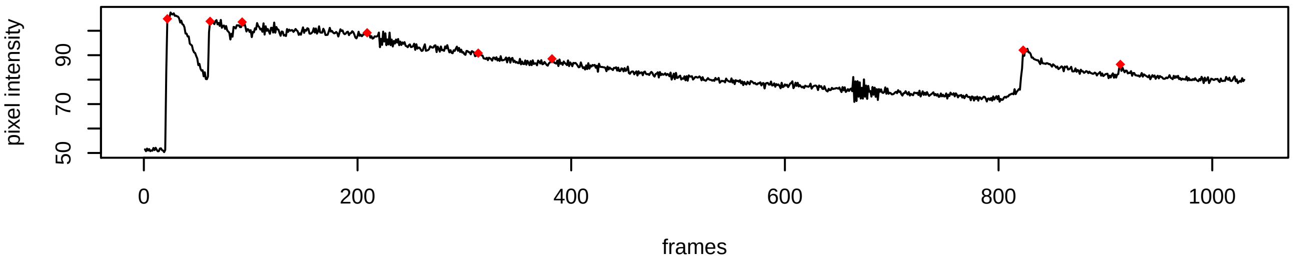

Graph 39 , 26   Total Activity 8   Position in Array 347

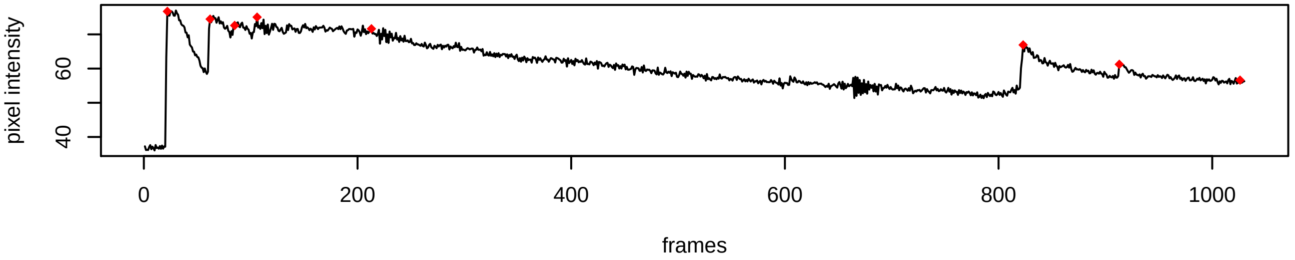

Graph 40 , 26   Total Activity 9   Position in Array 348

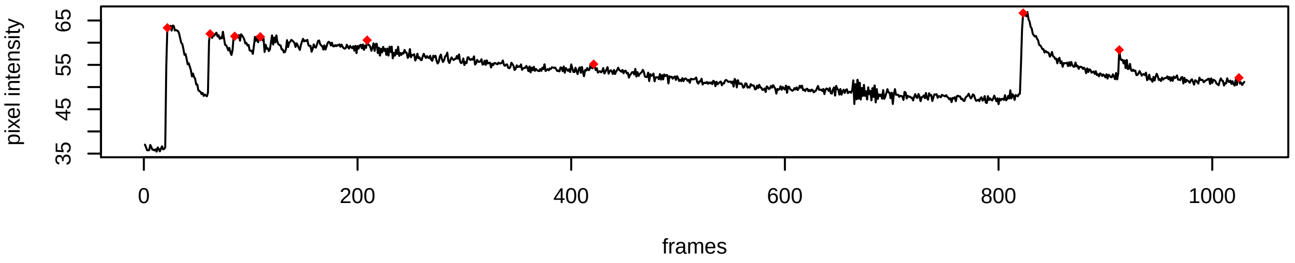

Graph 41 , 26   Total Activity 7   Position in Array 349

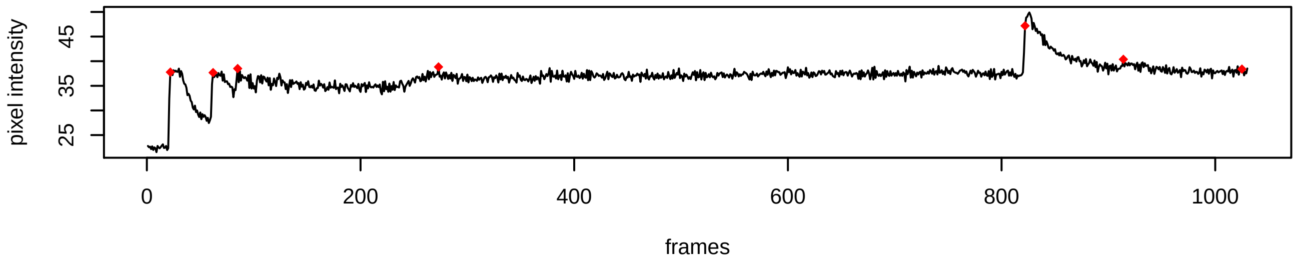

Graph 42 , 26   Total Activity 7   Position in Array 350

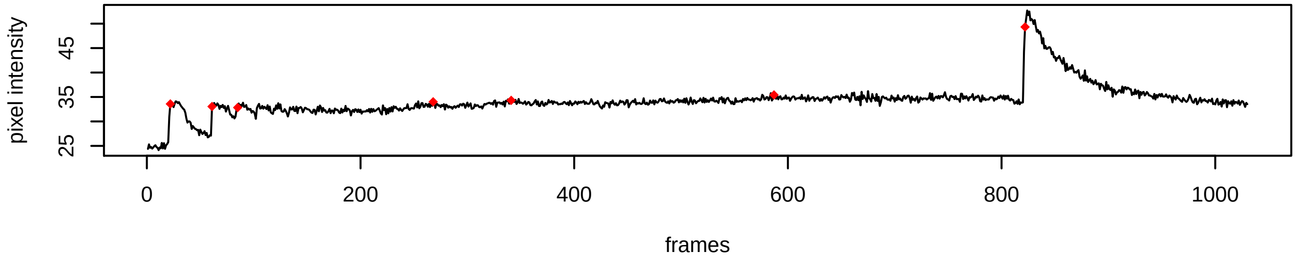

Graph 43 , 26   Total Activity 8   Position in Array 351

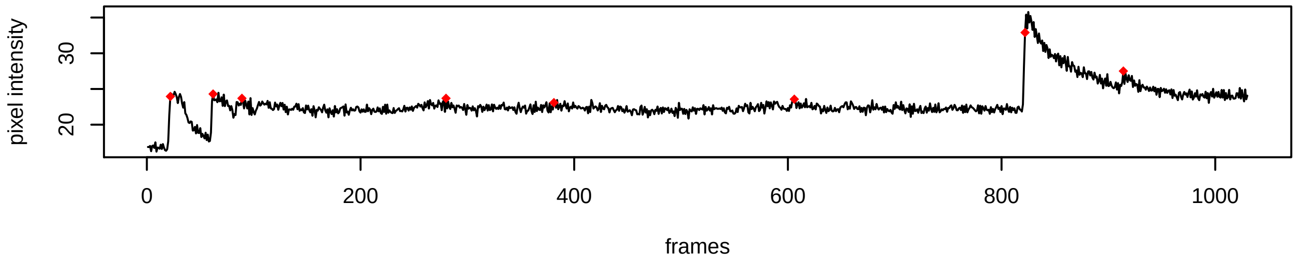

Graph 44 , 26   Total Activity 6   Position in Array 352

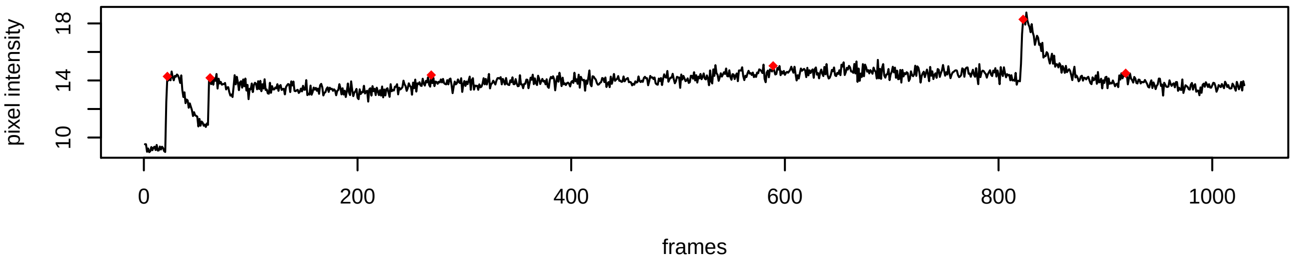

Graph 1 , 25   Total Activity 7   Position in Array 353

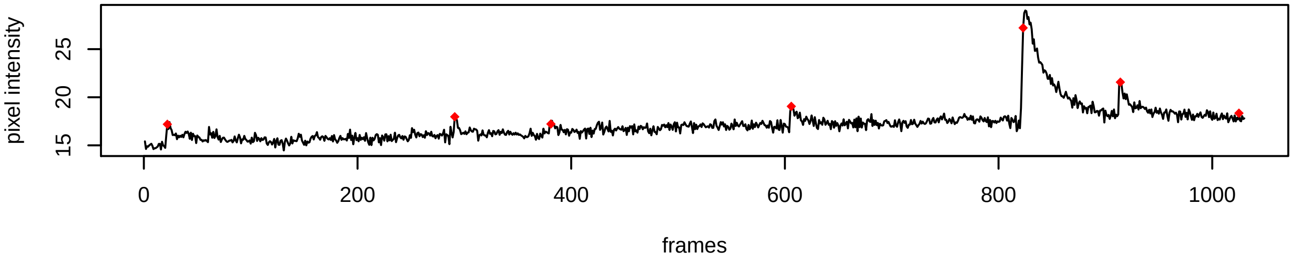

Graph 2 , 25   Total Activity 7   Position in Array 354

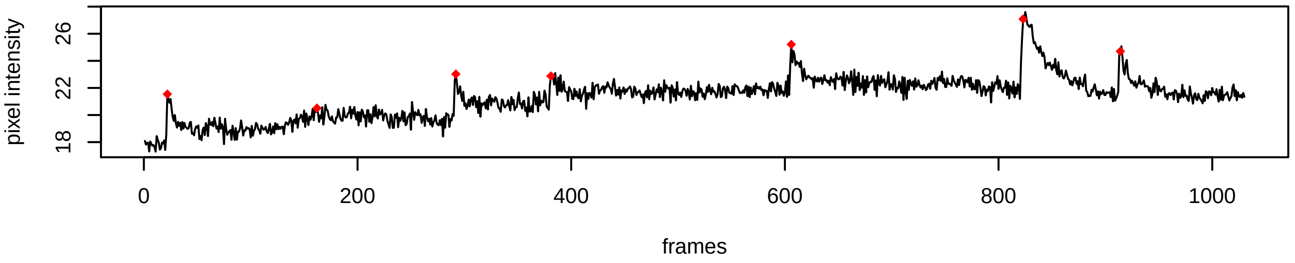

Graph 3 , 25 Total Activity 8 Position in Array 355

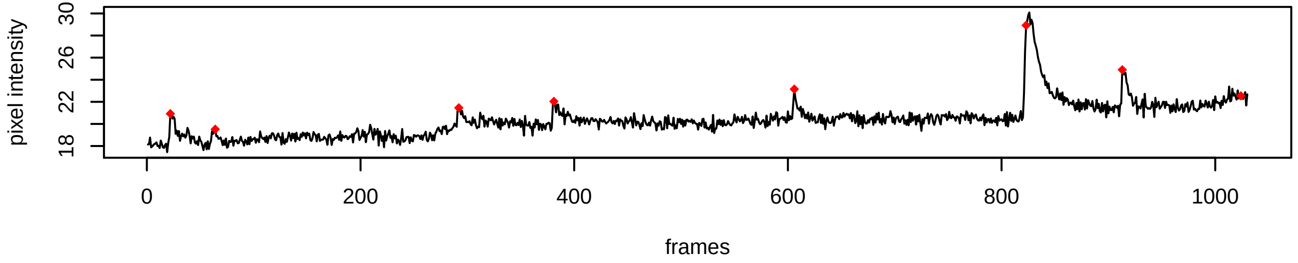

Graph 4 , 25 Total Activity 7 Position in Array 356

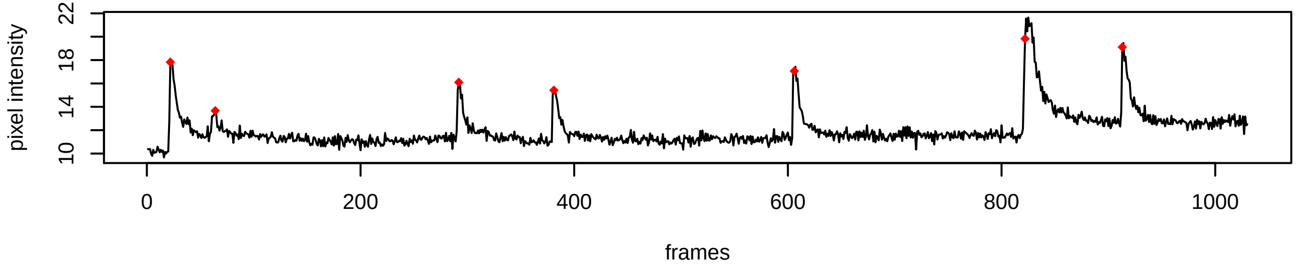

Graph 17 , 25 Total Activity 7 Position in Array 369

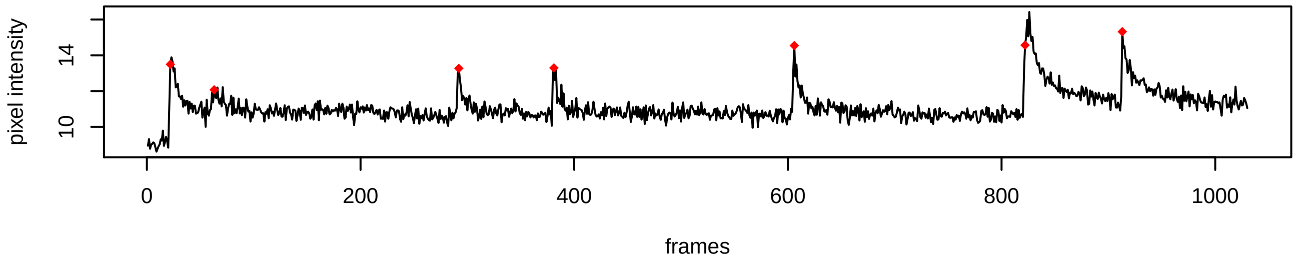

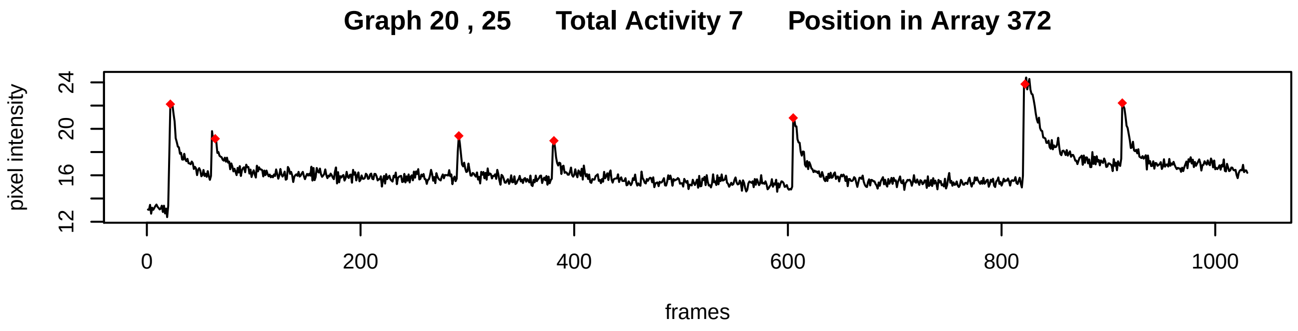
Graph 20 , 25   Total Activity 7   Position in Array 372
pixel intensity
12
16
20
24
0
200
400
600
800
1000
frames

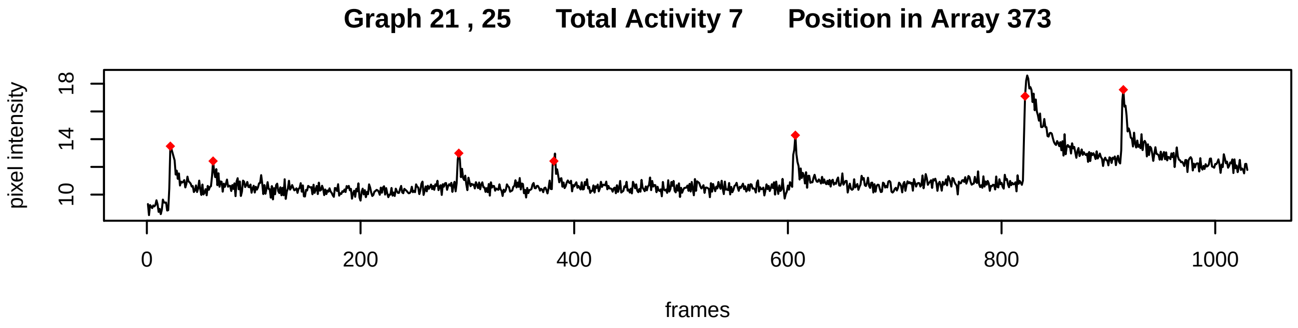
Graph 21 , 25   Total Activity 7   Position in Array 373
pixel intensity
10
14
18
0
200
400
600
800
1000
frames

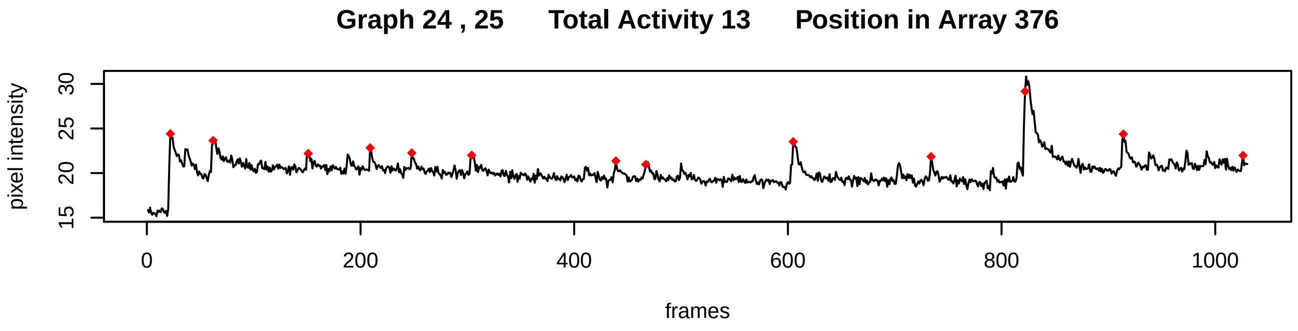
Graph 24 , 25   Total Activity 13   Position in Array 376
pixel intensity
15
20
25
30
0
200
400
600
800
1000
frames

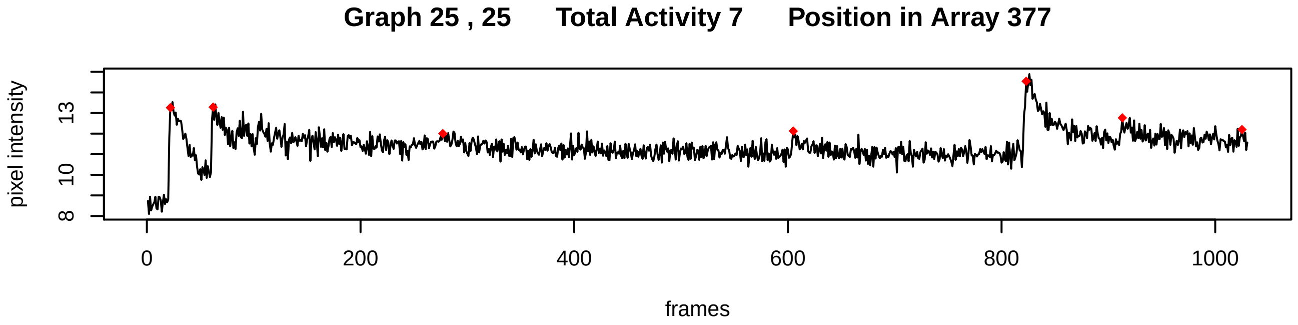
Graph 25 , 25 Total Activity 7 Position in Array 377
pixel intensity
8
10
13
0
200
400
600
800
1000
frames

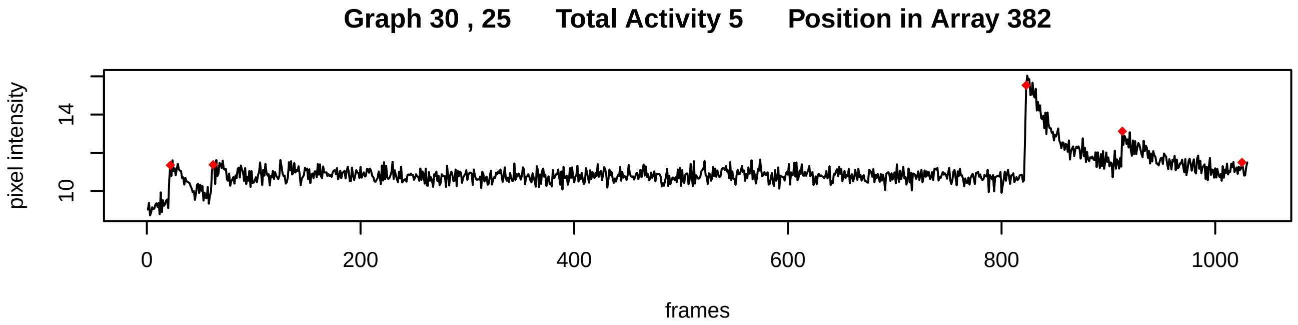
Graph 30 , 25 Total Activity 5 Position in Array 382
pixel intensity
10
14
0
200
400
600
800
1000
frames

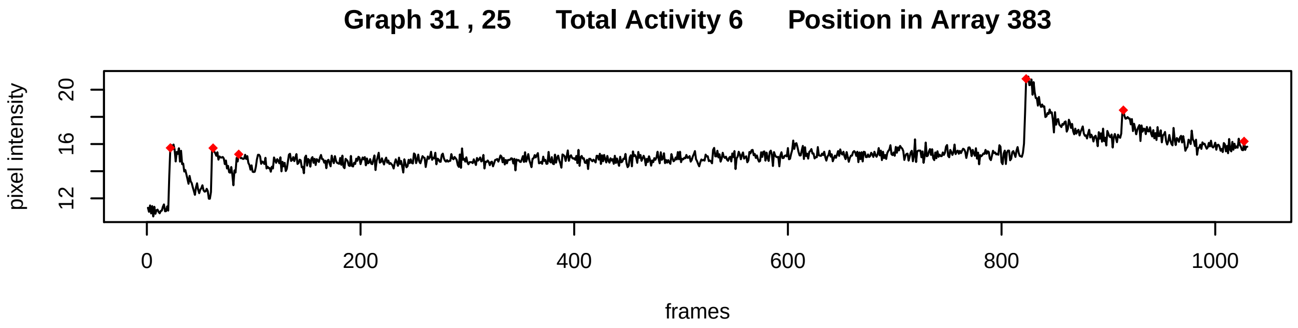
Graph 31 , 25 Total Activity 6 Position in Array 383
pixel intensity
12
16
20
0
200
400
600
800
1000
frames

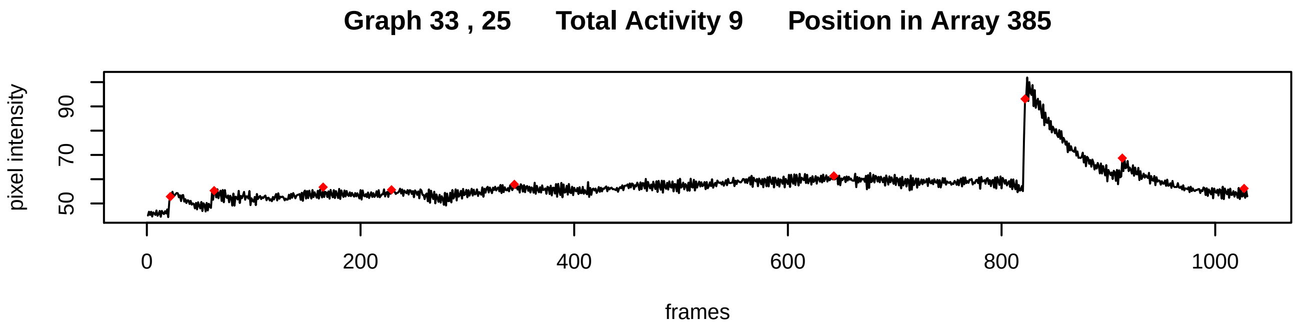
Graph 33 , 25 Total Activity 9 Position in Array 385
pixel intensity
50
70
90
0
200
400
600
800
1000
frames

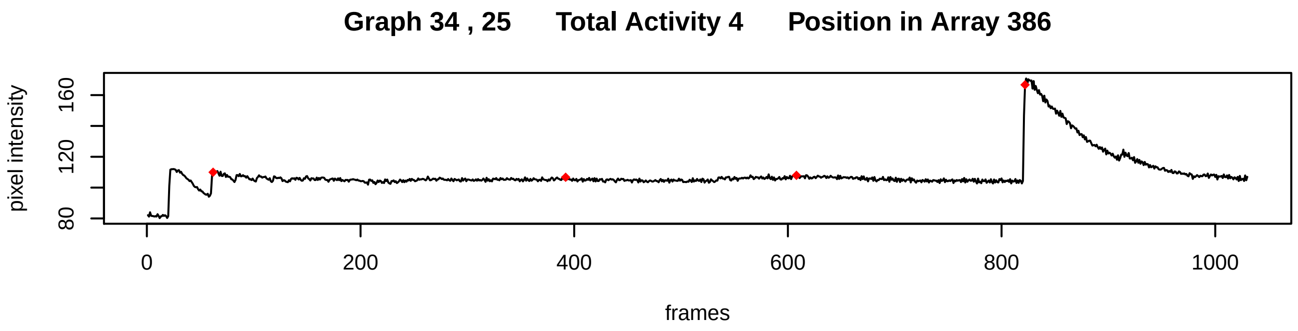
Graph 34 , 25 Total Activity 4 Position in Array 386
pixel intensity
80
120
160
0
200
400
600
800
1000
frames

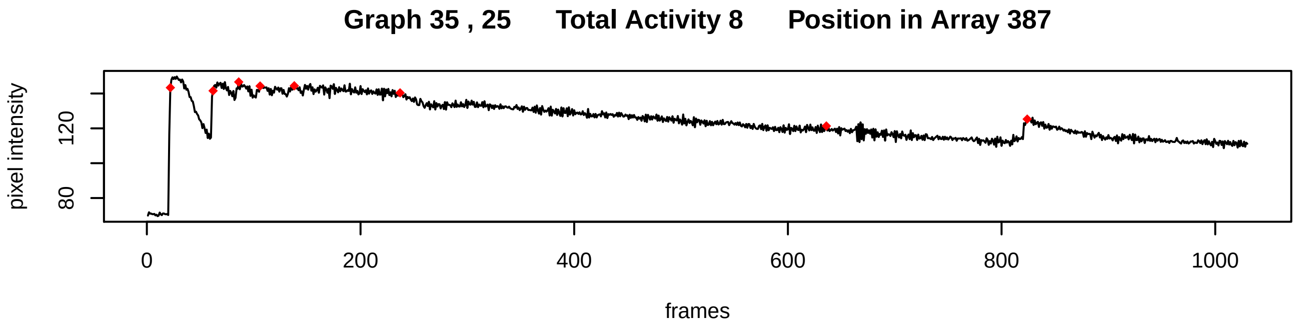
Graph 35 , 25 Total Activity 8 Position in Array 387
pixel intensity
80
120
0
200
400
600
800
1000
frames

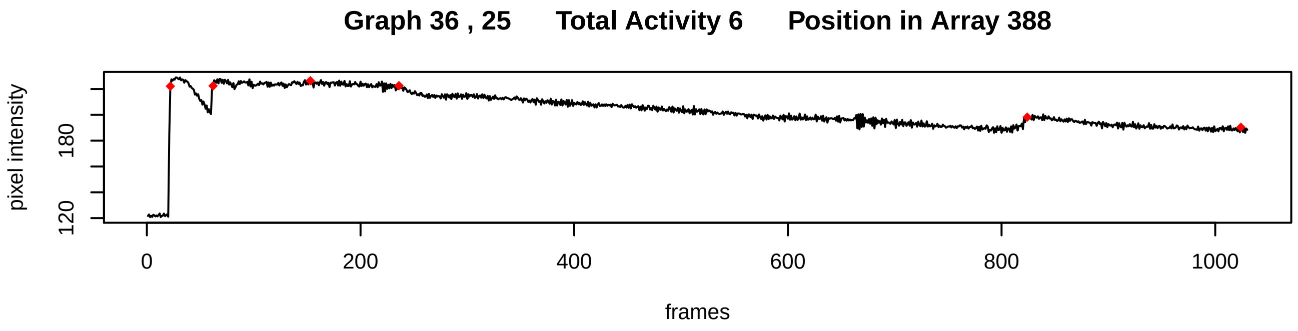
Graph 36 , 25 Total Activity 6 Position in Array 388
pixel intensity
120
180
0
200
400
600
800
1000
frames

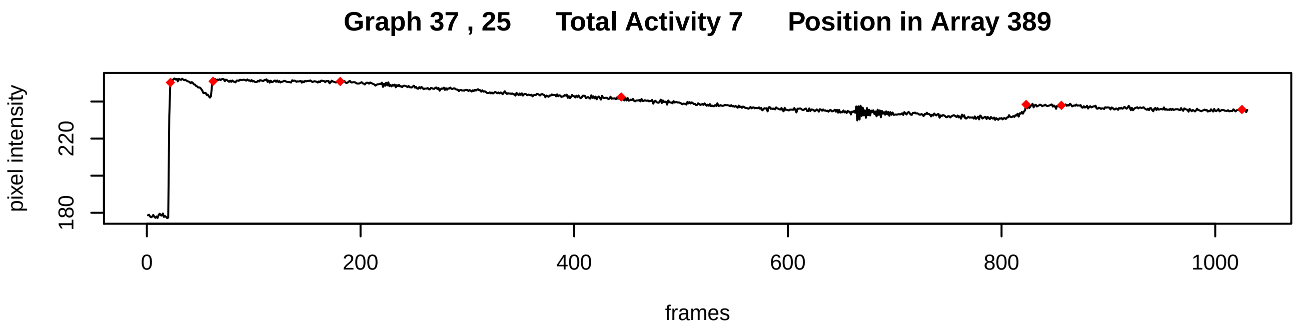
Graph 37 , 25 Total Activity 7 Position in Array 389
pixel intensity
180
220
0
200
400
600
800
1000
frames

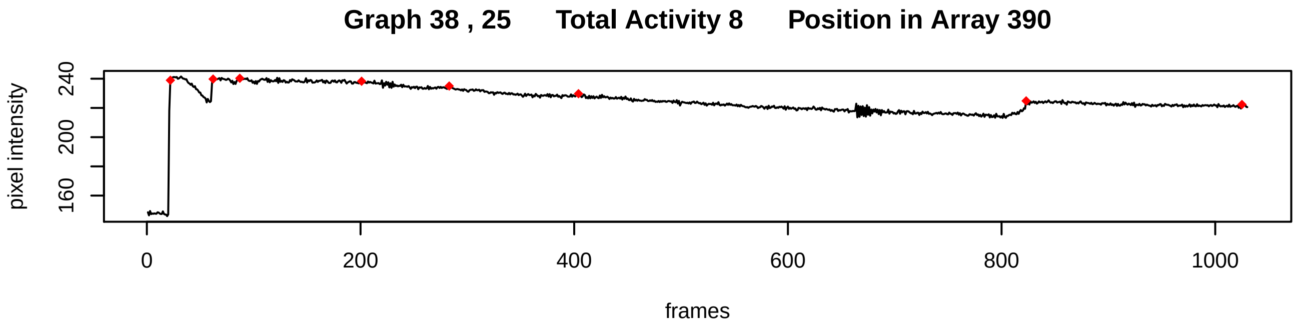
Graph 38 , 25 Total Activity 8 Position in Array 390
pixel intensity
160
200
240
0
200
400
600
800
1000
frames

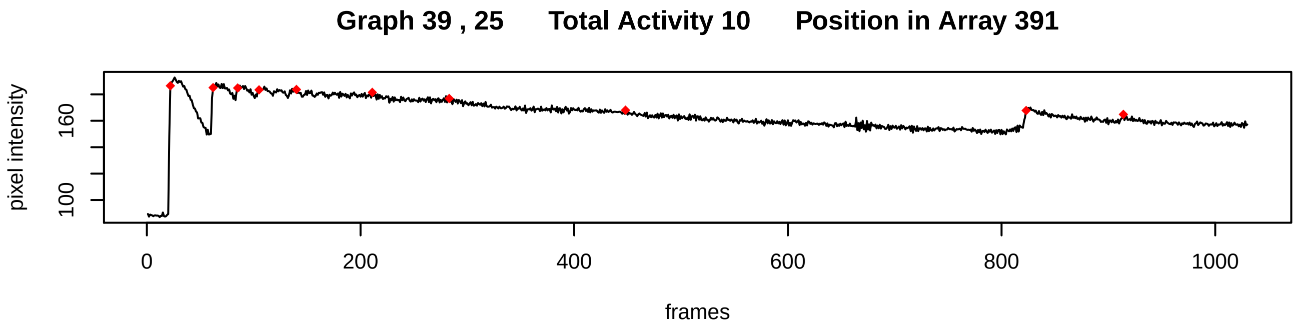
Graph 39 , 25 Total Activity 10 Position in Array 391
pixel intensity
100
160
0
200
400
600
800
1000
frames

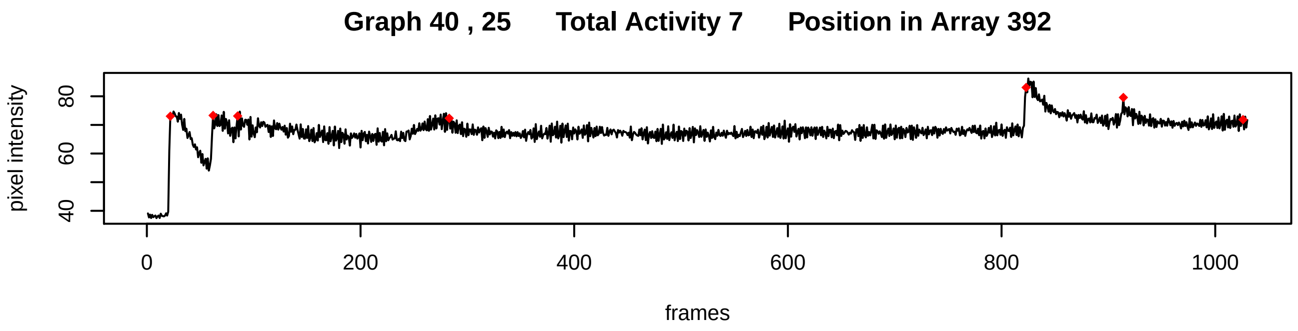
Graph 40 , 25 Total Activity 7 Position in Array 392
pixel intensity
40
60
80
0
200
400
600
800
1000
frames

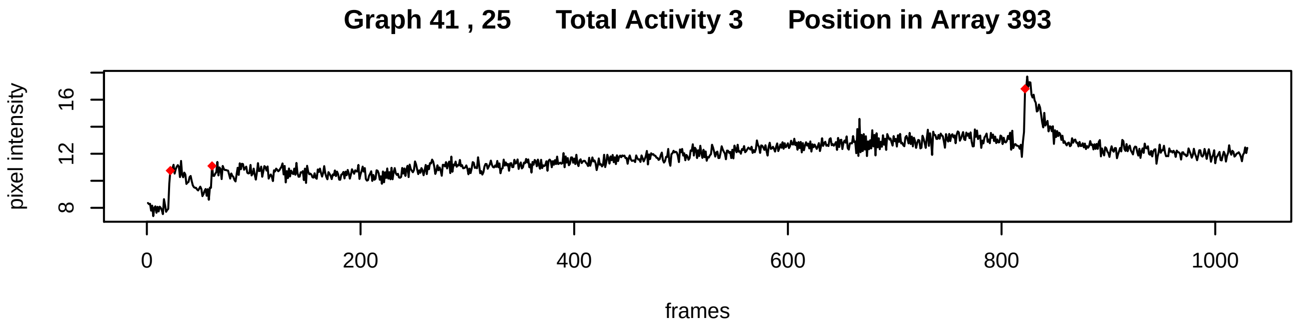
Graph 41 , 25 Total Activity 3 Position in Array 393
pixel intensity
8
12
16
0
200
400
600
800
1000
frames

Graph 42 , 25 Total Activity 6 Position in Array 394

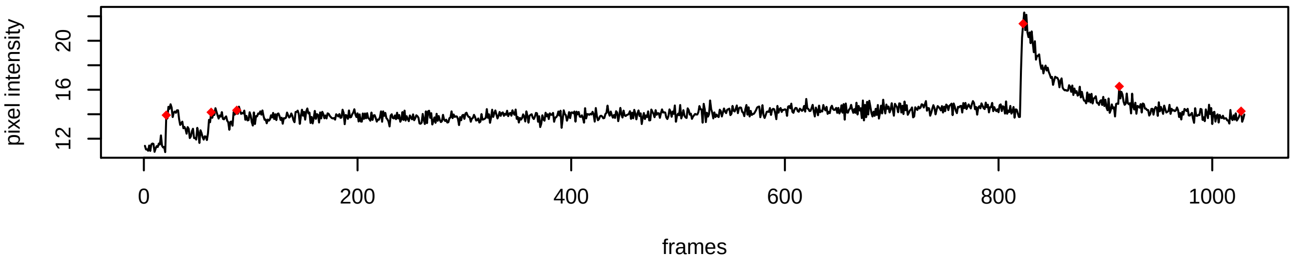

Graph 43 , 25 Total Activity 5 Position in Array 395

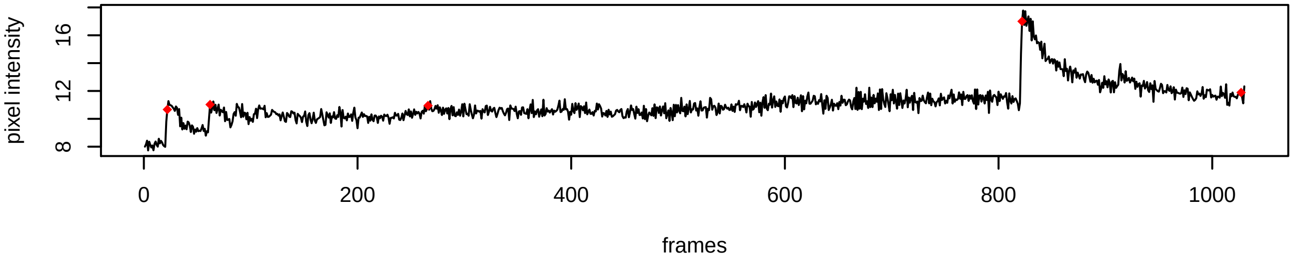

Graph 1 , 24 Total Activity 7 Position in Array 397

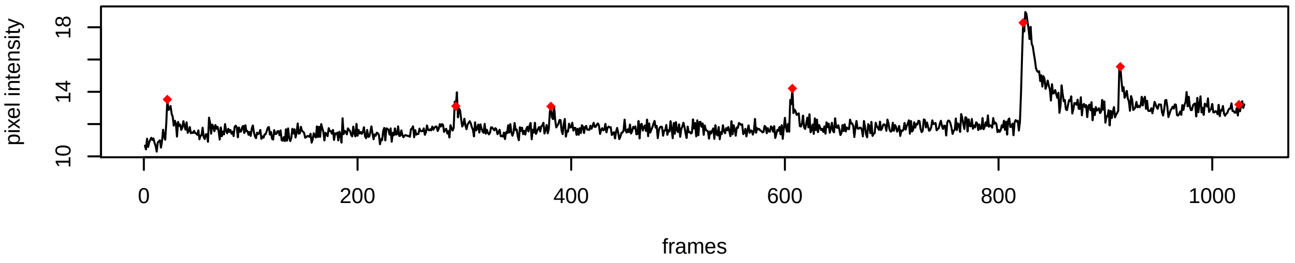

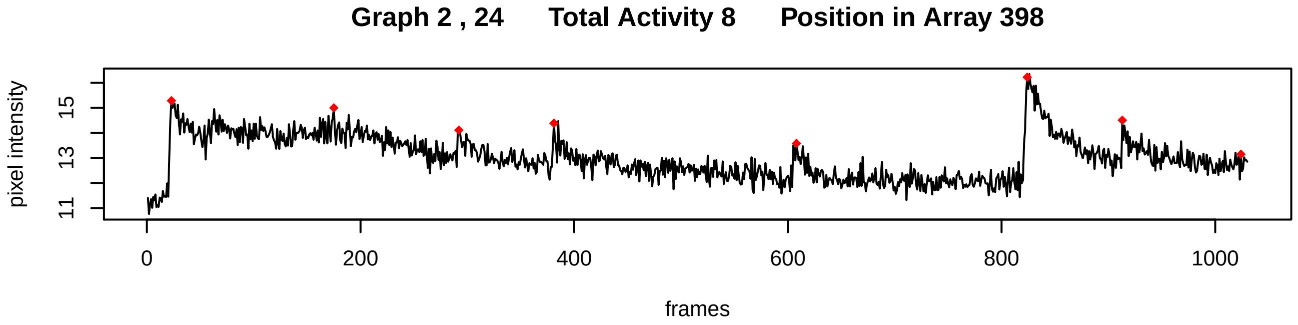
Graph 2 , 24 Total Activity 8 Position in Array 398
pixel intensity
11
13
15
0
200
400
600
800
1000
frames

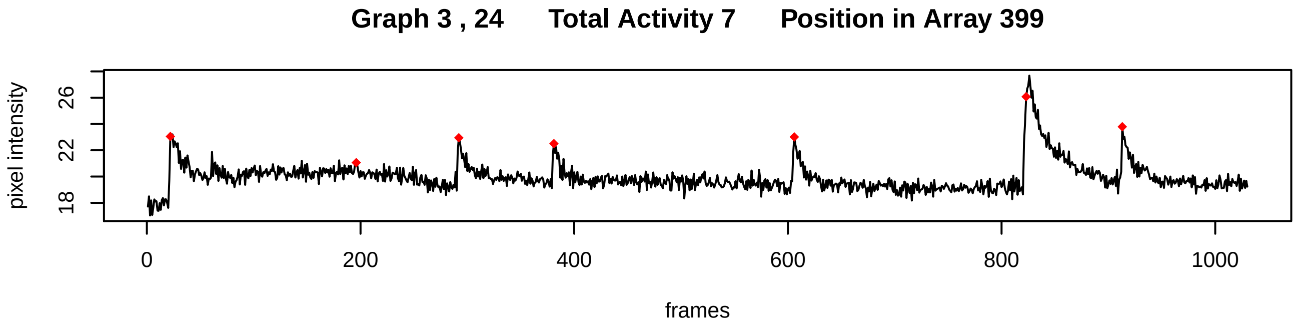
Graph 3 , 24 Total Activity 7 Position in Array 399
pixel intensity
18
22
26
0
200
400
600
800
1000
frames

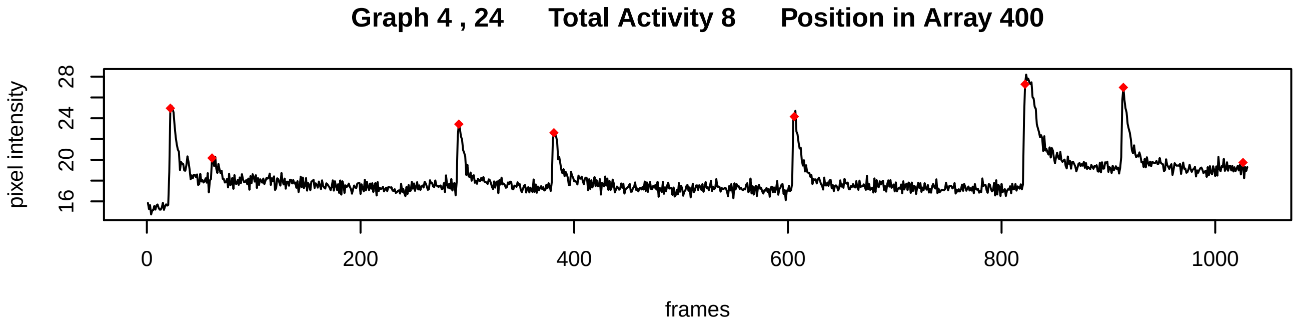
Graph 4 , 24 Total Activity 8 Position in Array 400
pixel intensity
16
20
24
28
0
200
400
600
800
1000
frames

Graph 9 , 24 Total Activity 6 Position in Array 405

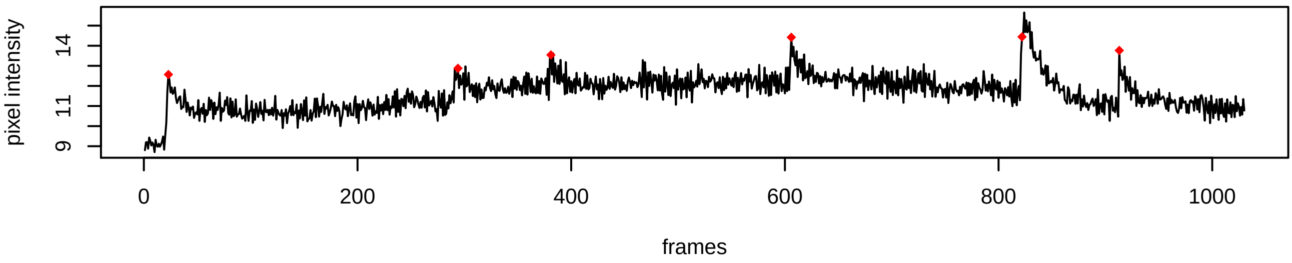

Graph 10 , 24 Total Activity 5 Position in Array 406

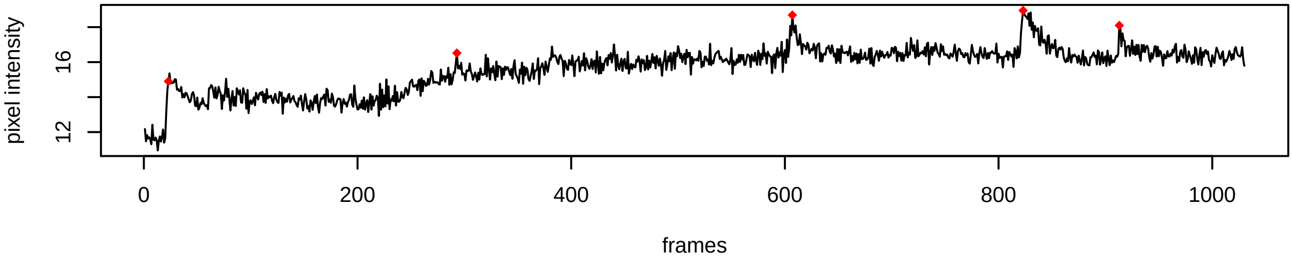

Graph 11 , 24 Total Activity 5 Position in Array 407

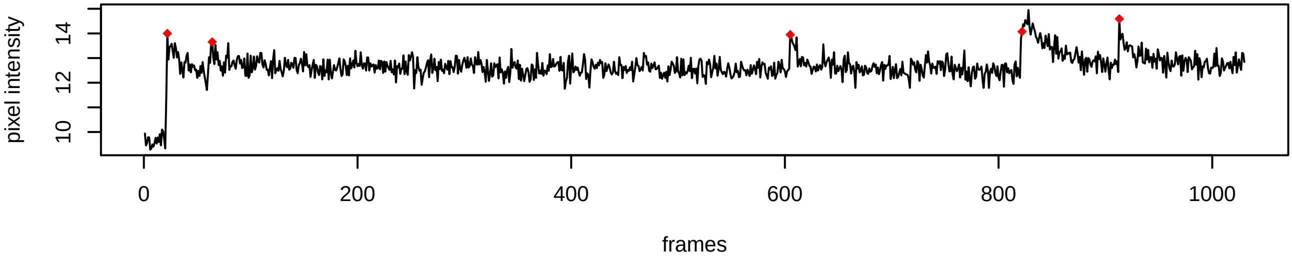

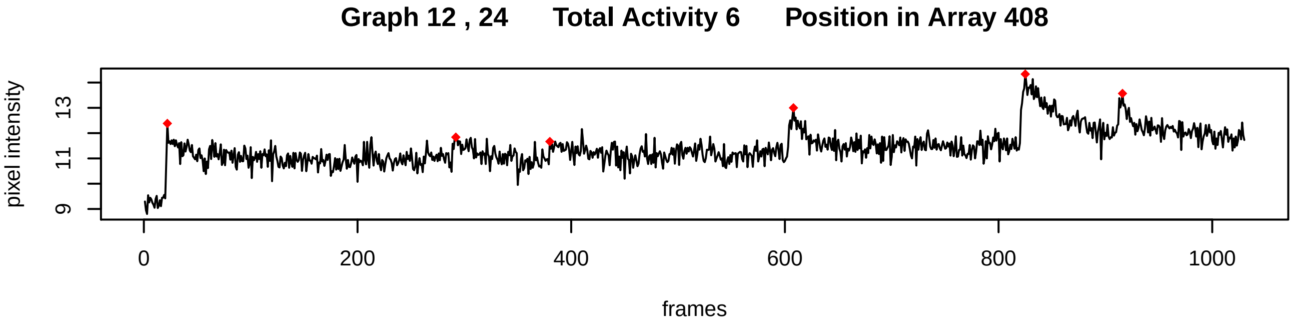

Graph 12 , 24 Total Activity 6 Position in Array 408
pixel intensity
9
11
13
0
200
400
600
800
1000
frames

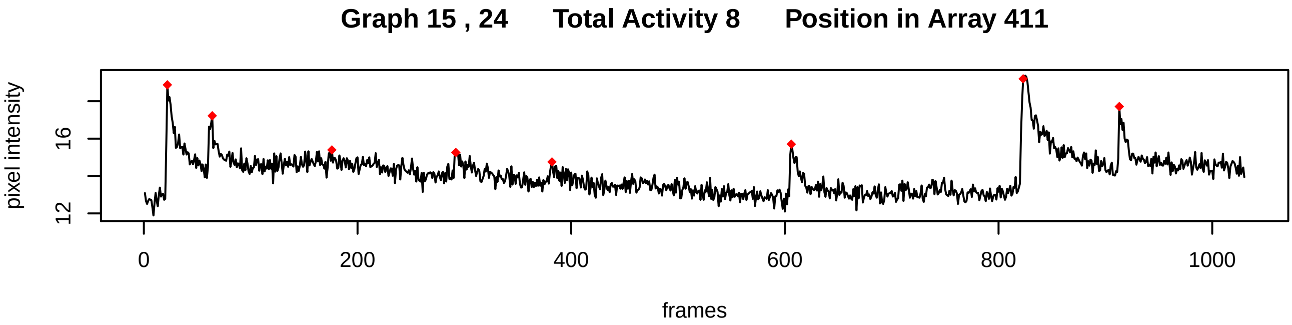

Graph 15 , 24 Total Activity 8 Position in Array 411
pixel intensity
12
16
0
200
400
600
800
1000
frames

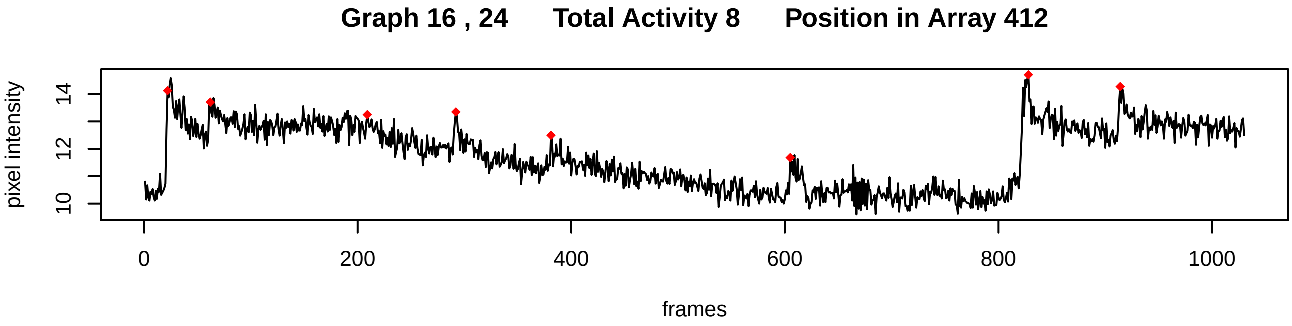

Graph 16 , 24 Total Activity 8 Position in Array 412
pixel intensity
10
12
14
0
200
400
600
800
1000
frames

Graph 18 , 24 Total Activity 8 Position in Array 414

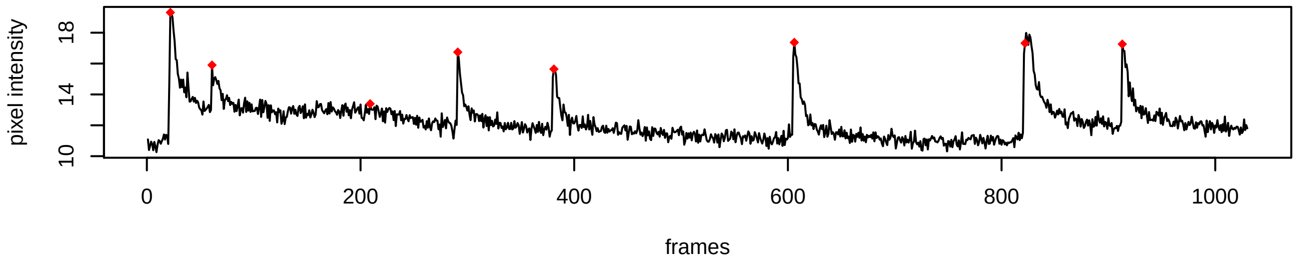

Graph 19 , 24 Total Activity 8 Position in Array 415

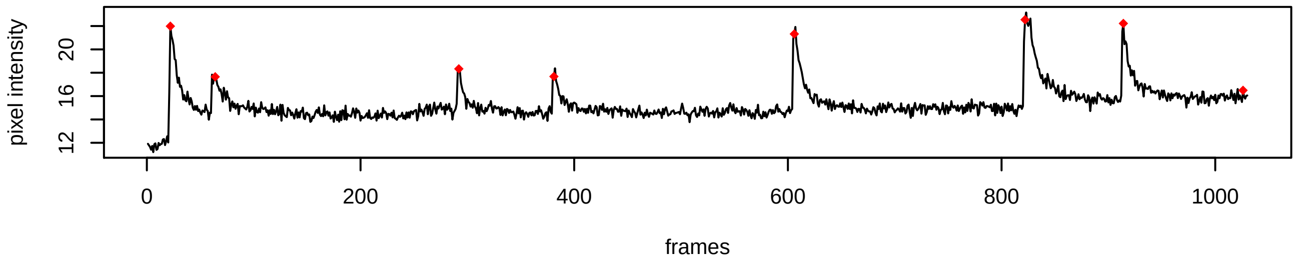

Graph 20 , 24 Total Activity 7 Position in Array 416

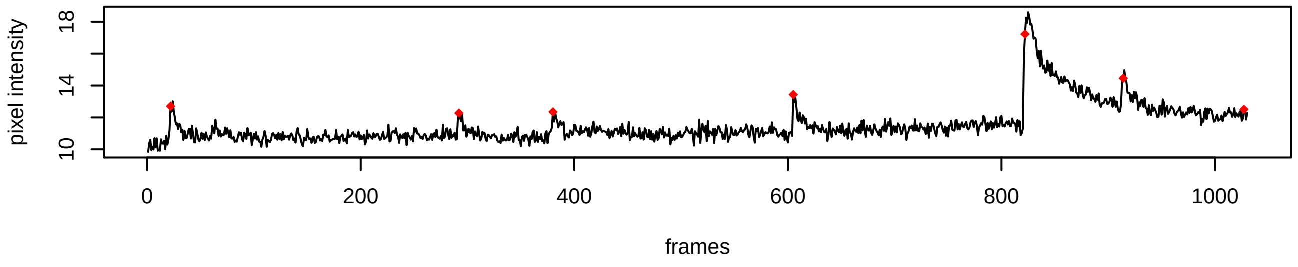

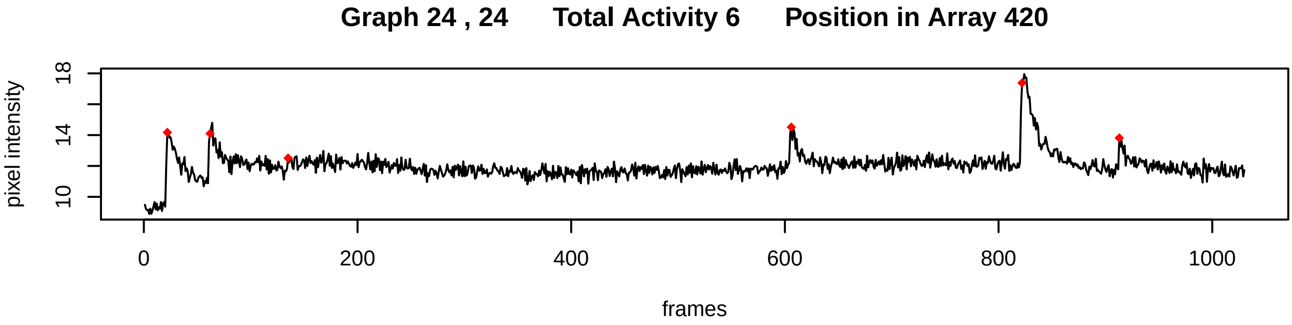
Graph 24 , 24 Total Activity 6 Position in Array 420
pixel intensity
10
14
18
0
200
400
600
800
1000
frames

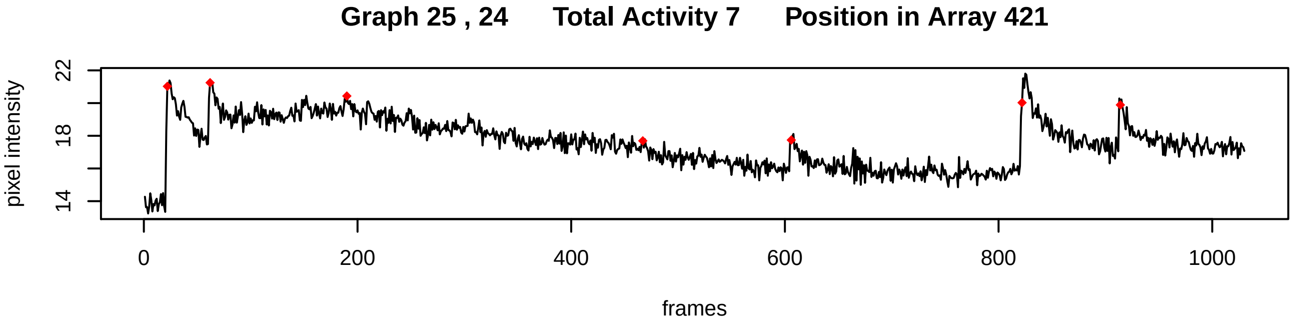
Graph 25 , 24 Total Activity 7 Position in Array 421
pixel intensity
14
18
22
0
200
400
600
800
1000
frames

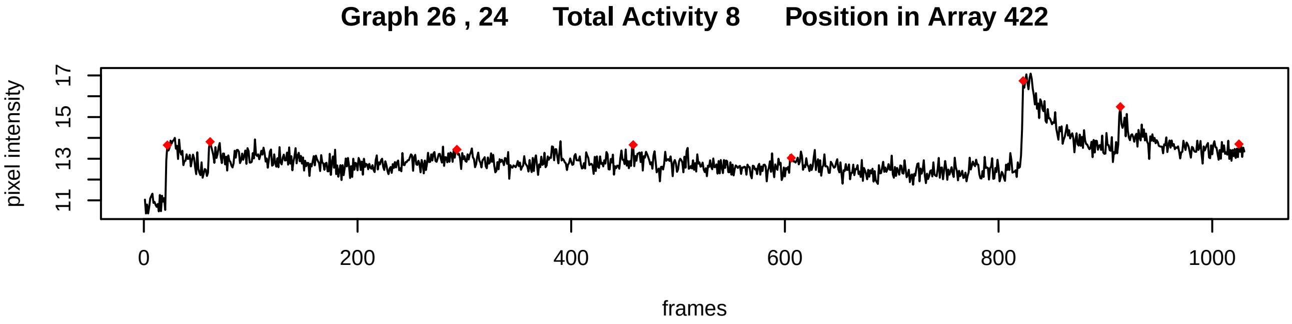
Graph 26 , 24 Total Activity 8 Position in Array 422
pixel intensity
11
13
15
17
0
200
400
600
800
1000
frames

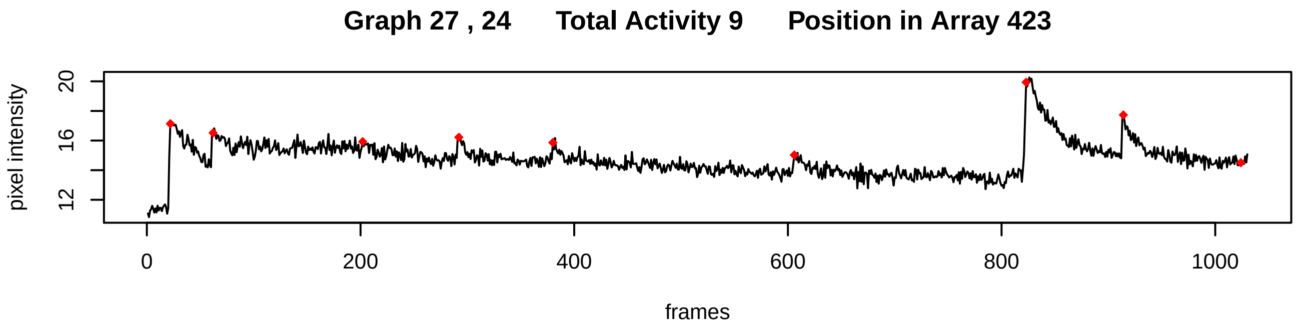
Graph 27 , 24 Total Activity 9 Position in Array 423
pixel intensity
12
16
20
0
200
400
600
800
1000
frames

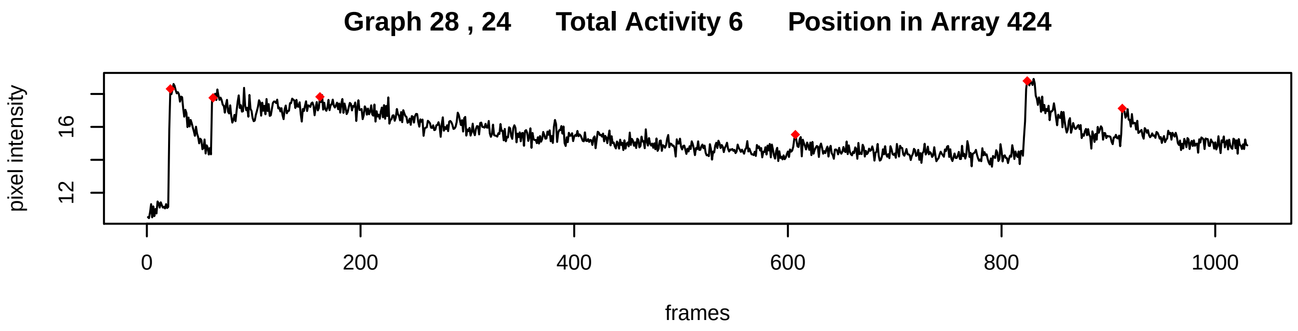
Graph 28 , 24 Total Activity 6 Position in Array 424
pixel intensity
12
16
0
200
400
600
800
1000
frames

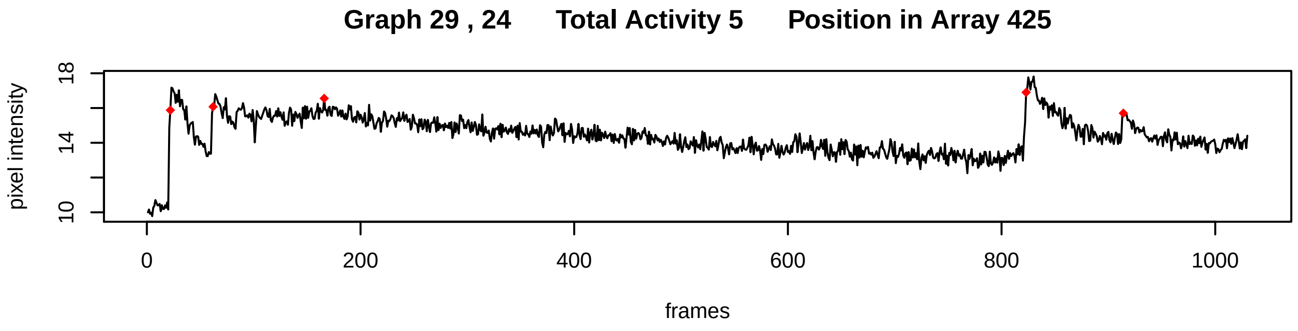
Graph 29 , 24 Total Activity 5 Position in Array 425
pixel intensity
10
14
18
0
200
400
600
800
1000
frames

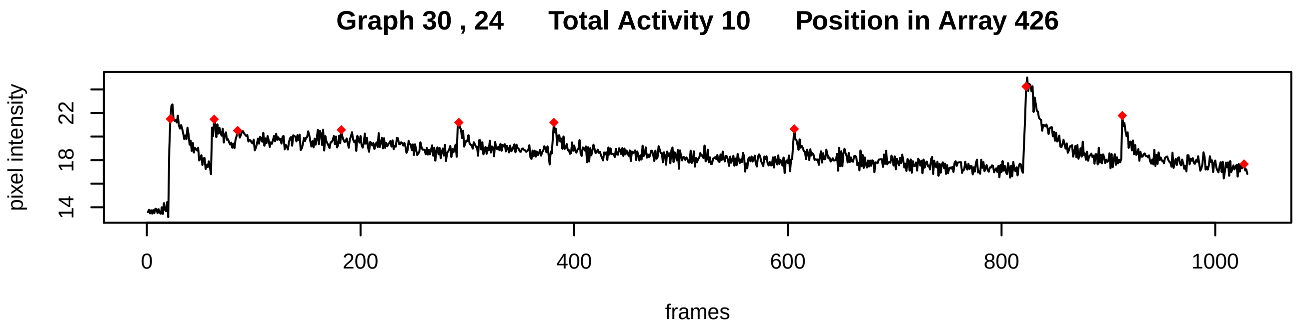
Graph 30 , 24 Total Activity 10 Position in Array 426
pixel intensity
14
18
22
0
200
400
600
800
1000
frames

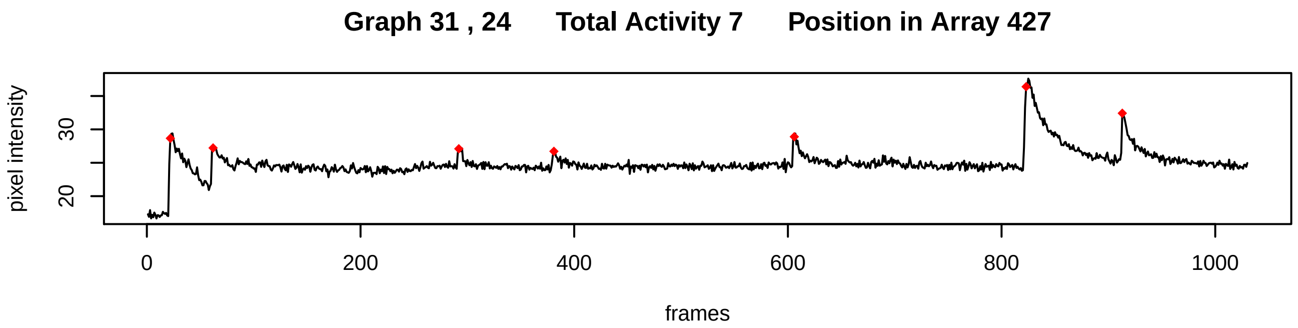
Graph 31 , 24 Total Activity 7 Position in Array 427
pixel intensity
20
30
0
200
400
600
800
1000
frames

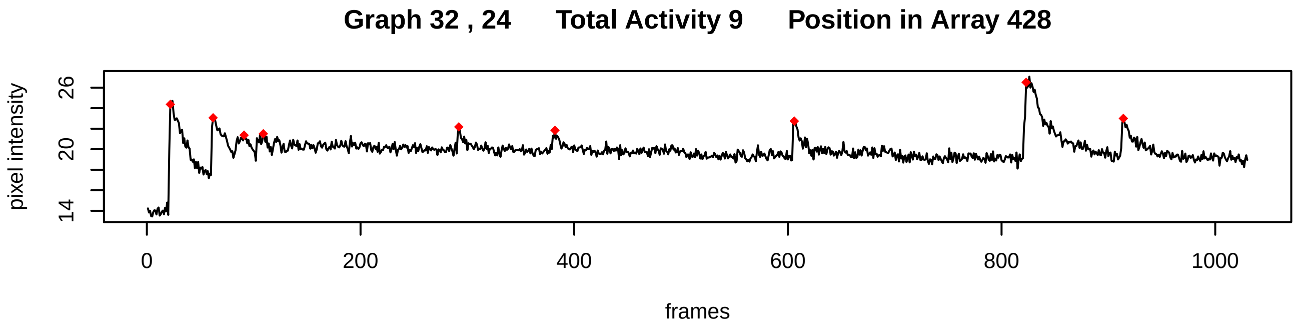
Graph 32 , 24 Total Activity 9 Position in Array 428
pixel intensity
14
20
26
0
200
400
600
800
1000
frames

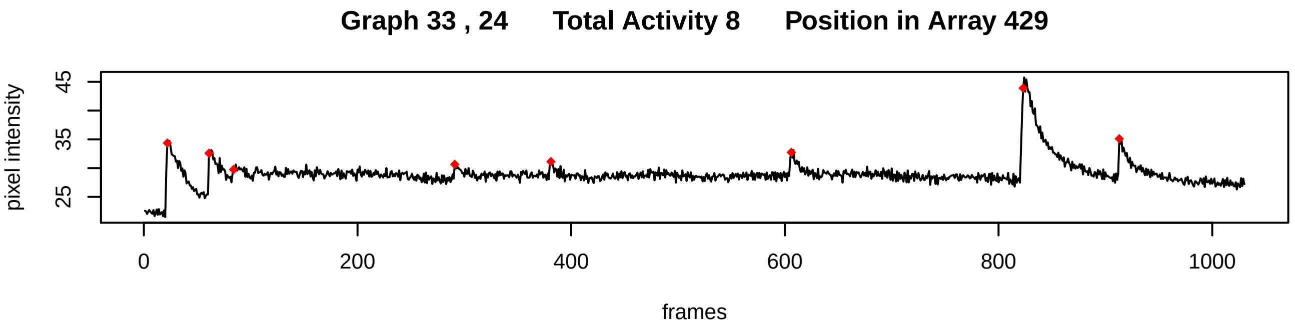
Graph 33 , 24 Total Activity 8 Position in Array 429
pixel intensity
25
35
45
0
200
400
600
800
1000
frames

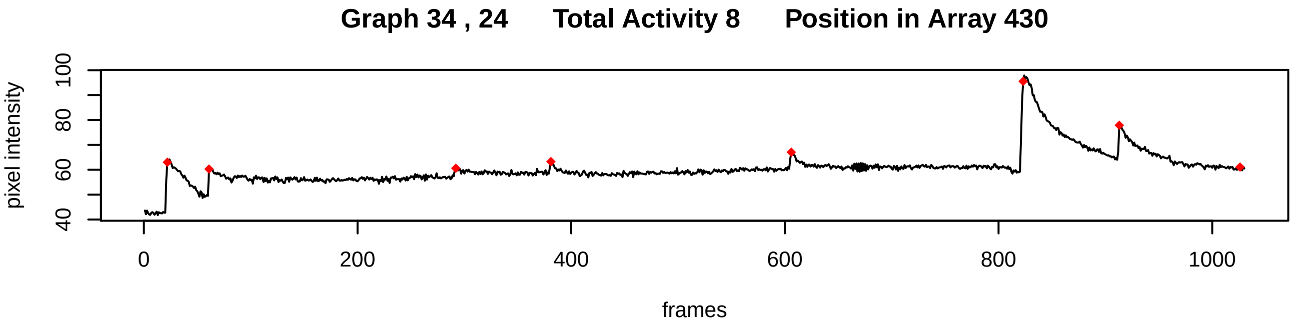
Graph 34 , 24 Total Activity 8 Position in Array 430
pixel intensity
40
60
80
100
0
200
400
600
800
1000
frames

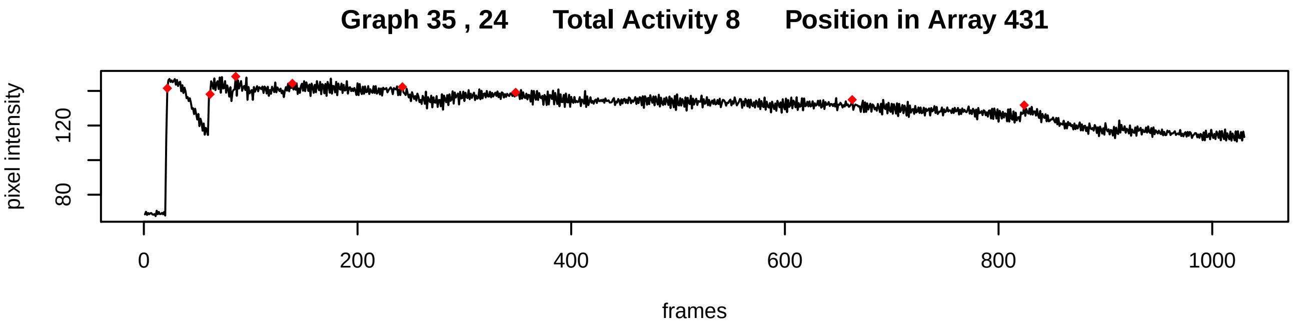
Graph 35 , 24 Total Activity 8 Position in Array 431
pixel intensity
80
120
0
200
400
600
800
1000
frames

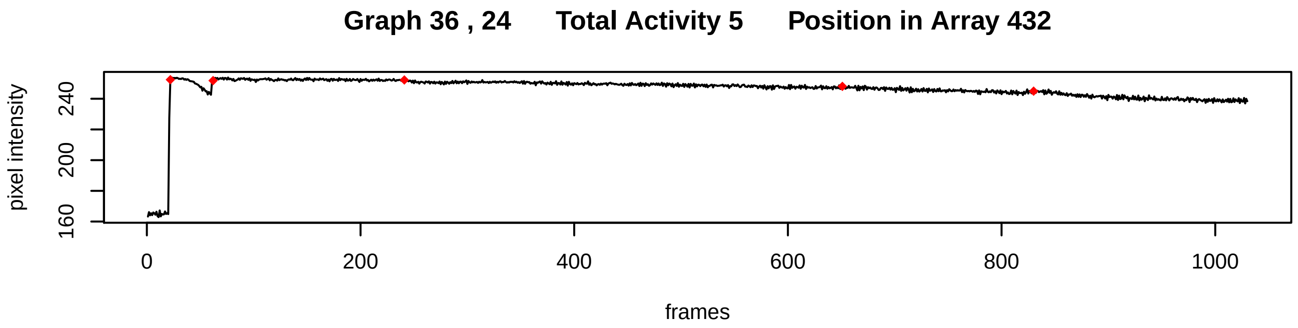
Graph 36 , 24 Total Activity 5 Position in Array 432
pixel intensity
160
200
240
0
200
400
600
800
1000
frames

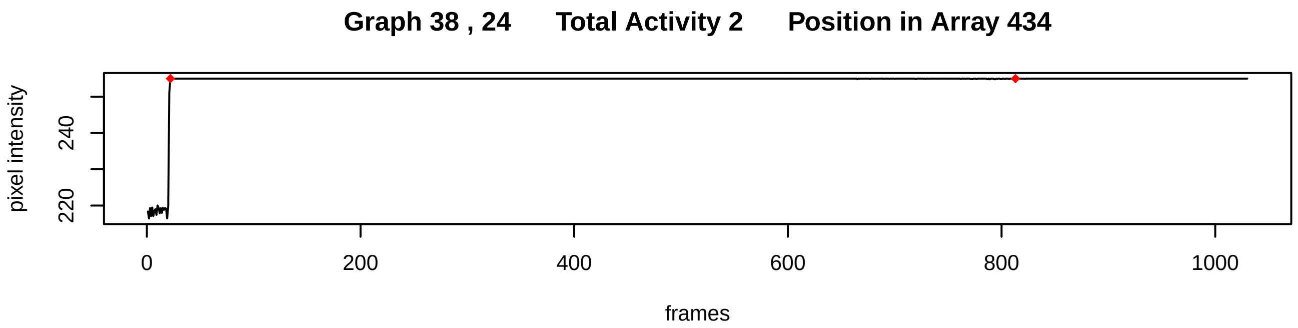
Graph 38 , 24 Total Activity 2 Position in Array 434
pixel intensity
220
240
0
200
400
600
800
1000
frames

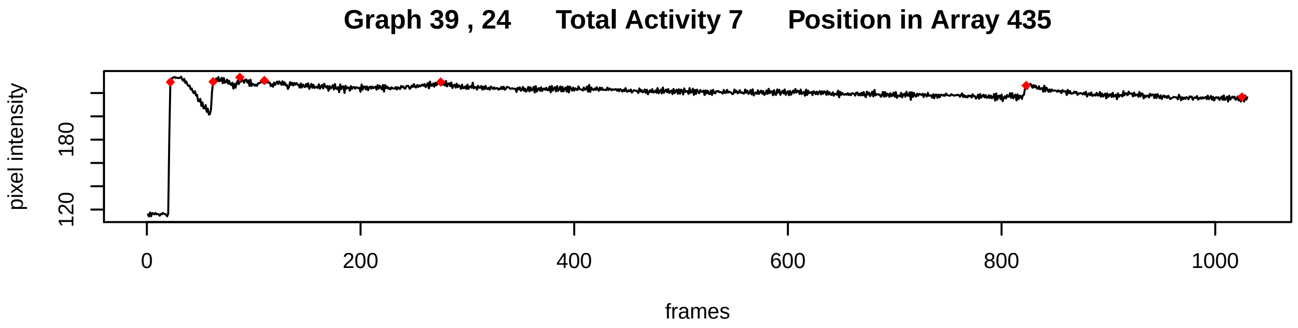
Graph 39 , 24 Total Activity 7 Position in Array 435
pixel intensity
120
180
0
200
400
600
800
1000
frames

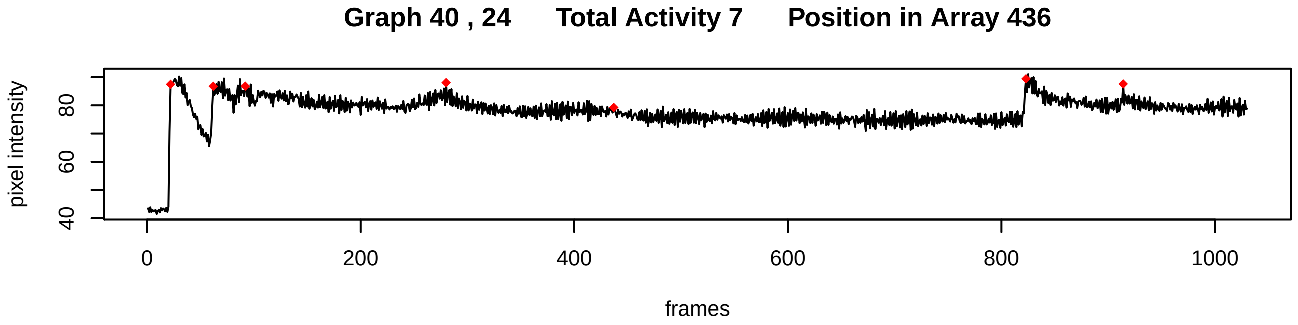
Graph 40 , 24 Total Activity 7 Position in Array 436
pixel intensity
40
60
80
0
200
400
600
800
1000
frames

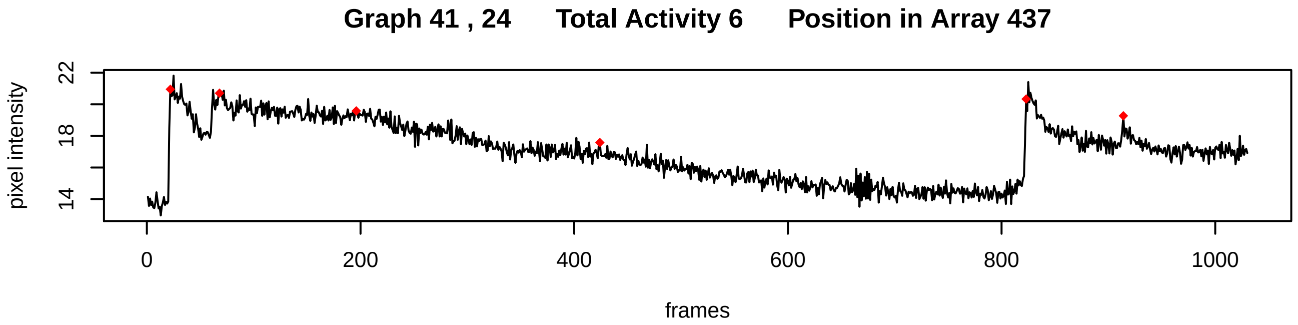
Graph 41 , 24 Total Activity 6 Position in Array 437
pixel intensity
14
18
22
0
200
400
600
800
1000
frames

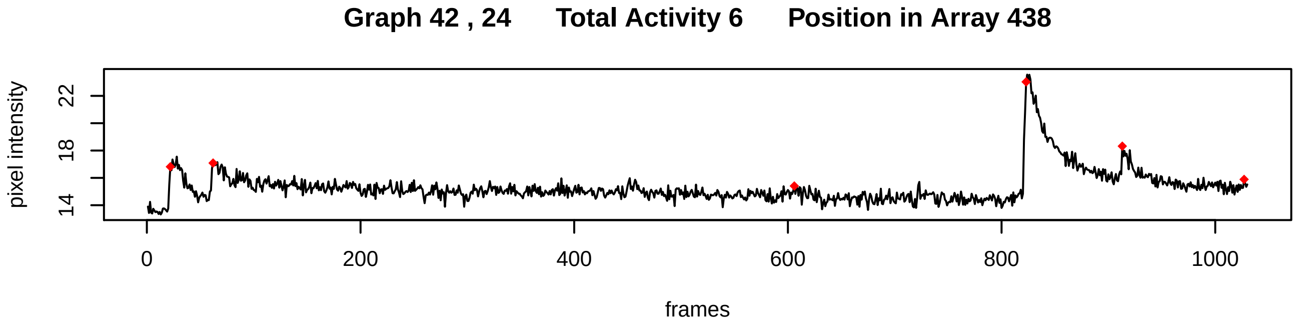
Graph 42 , 24 Total Activity 6 Position in Array 438
pixel intensity
14
18
22
0
200
400
600
800
1000
frames

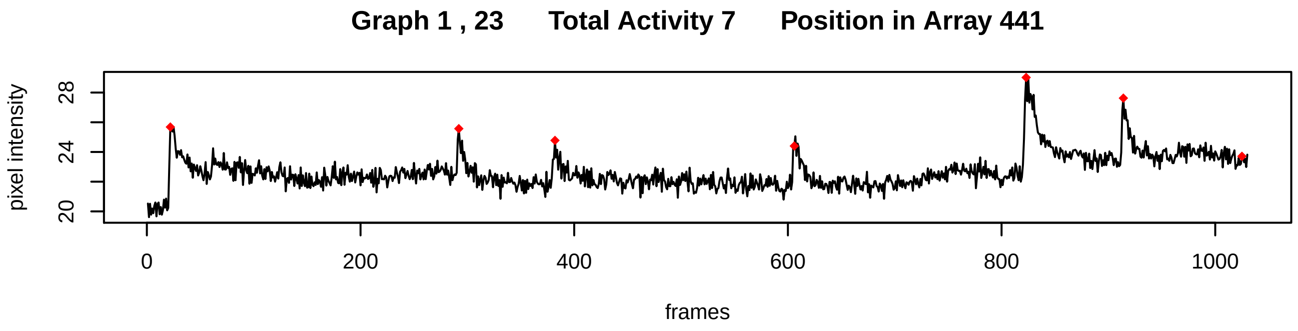
Graph 1 , 23 Total Activity 7 Position in Array 441
pixel intensity
frames
20
24
28
0
200
400
600
800
1000

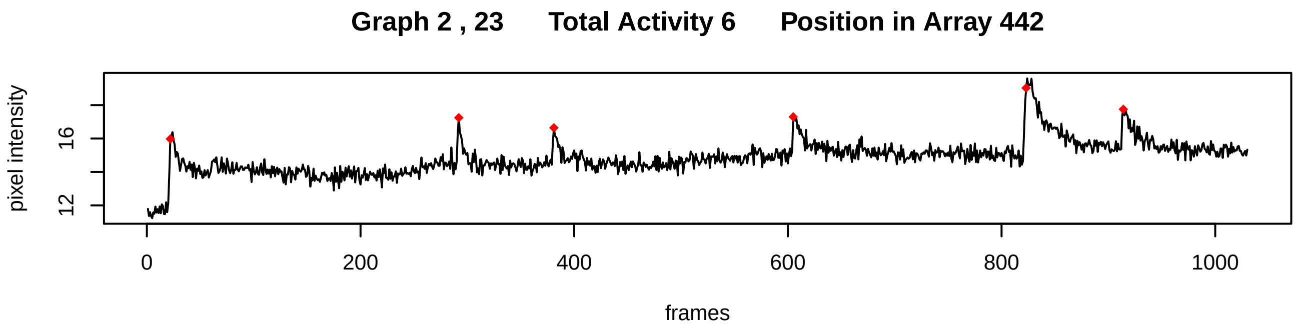
Graph 2 , 23 Total Activity 6 Position in Array 442
pixel intensity
frames
12
16
0
200
400
600
800
1000

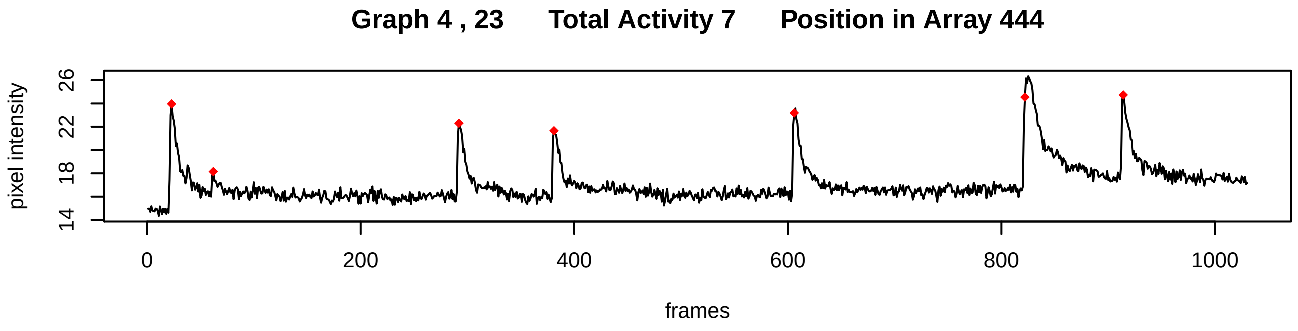
Graph 4 , 23 Total Activity 7 Position in Array 444
pixel intensity
frames
14
18
22
26
0
200
400
600
800
1000

Graph 10 , 23 Total Activity 6 Position in Array 450

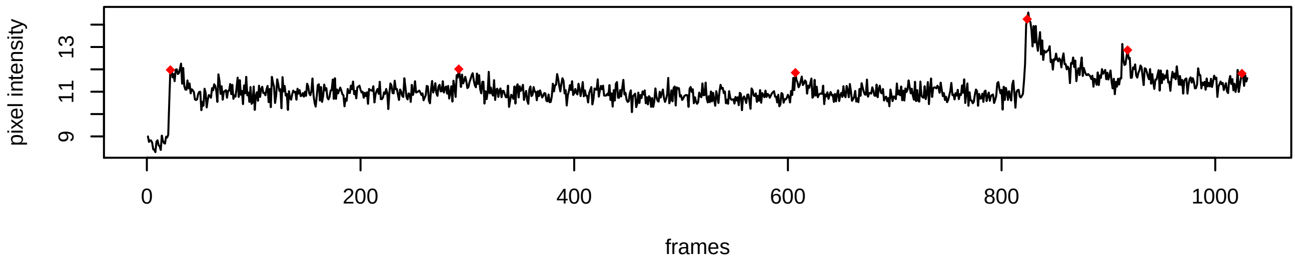

Graph 16 , 23 Total Activity 5 Position in Array 456

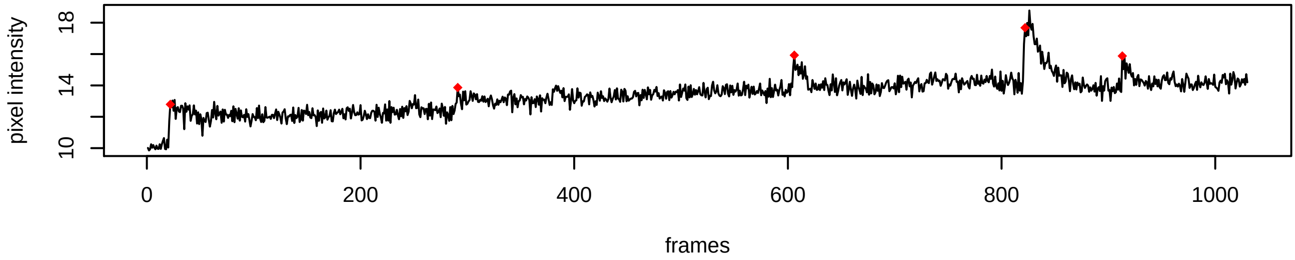

Graph 17 , 23 Total Activity 9 Position in Array 457

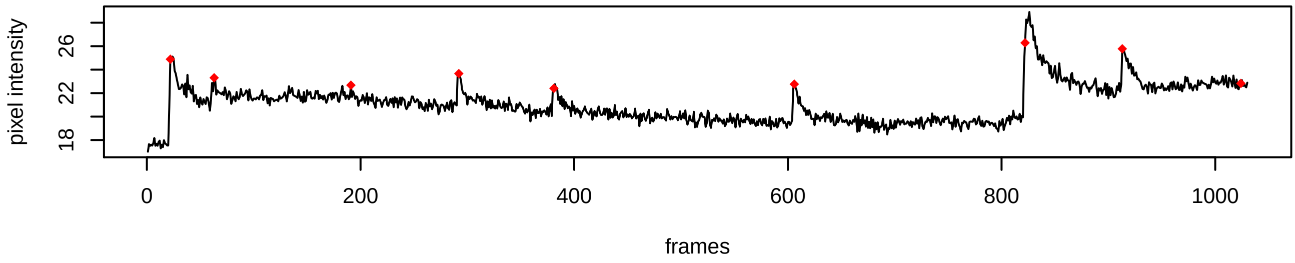

Graph 18 , 23   Total Activity 8   Position in Array 458

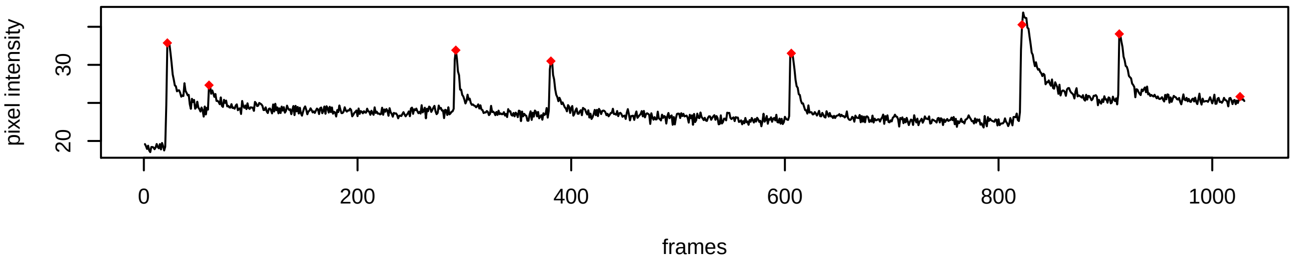

Graph 20 , 23   Total Activity 7   Position in Array 460

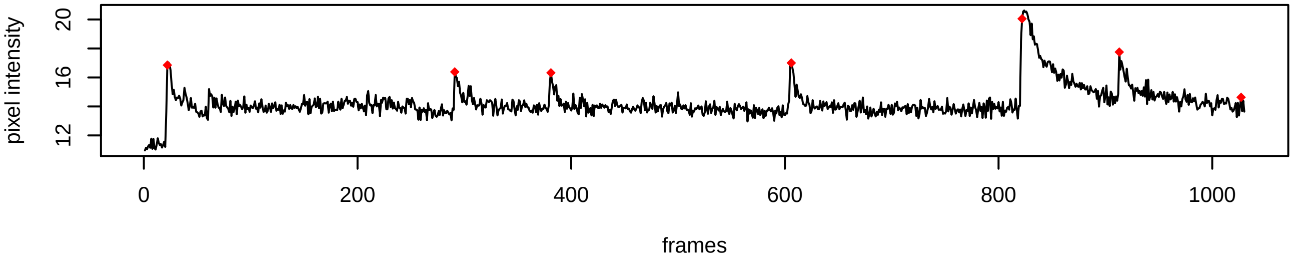

Graph 21 , 23   Total Activity 7   Position in Array 461

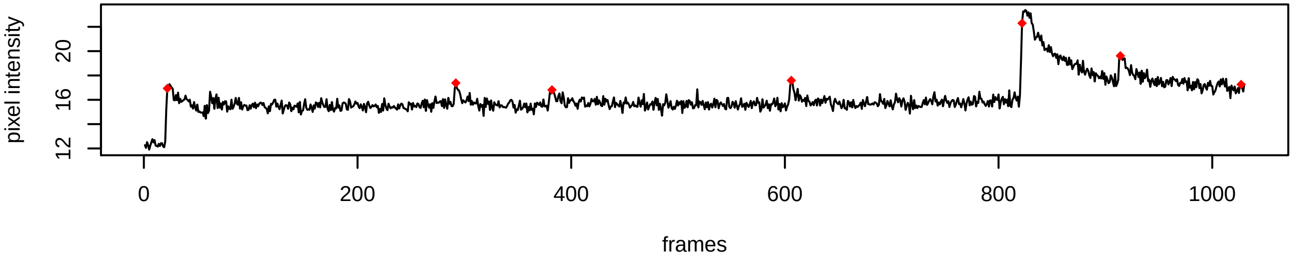

Graph 22 , 23 Total Activity 8 Position in Array 462

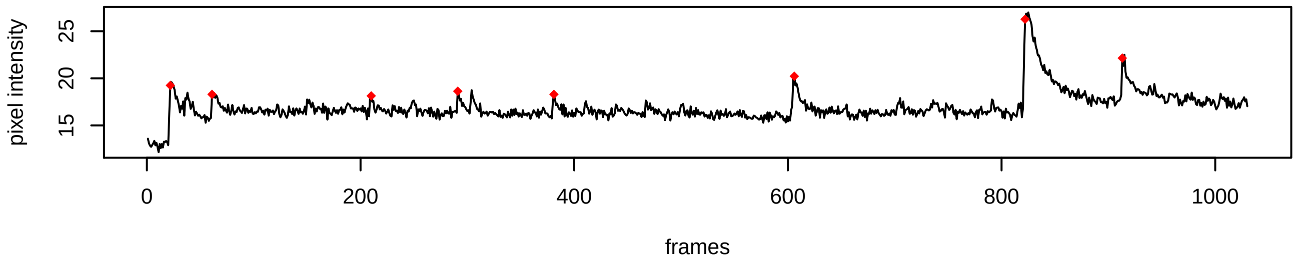

Graph 23 , 23 Total Activity 7 Position in Array 463

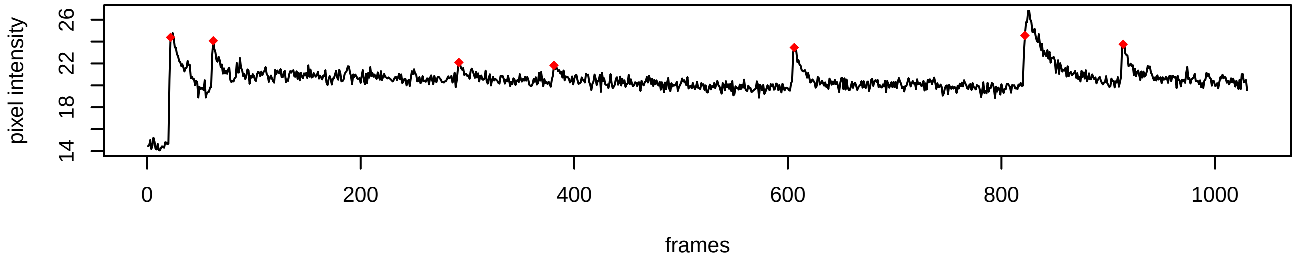

Graph 24 , 23 Total Activity 7 Position in Array 464

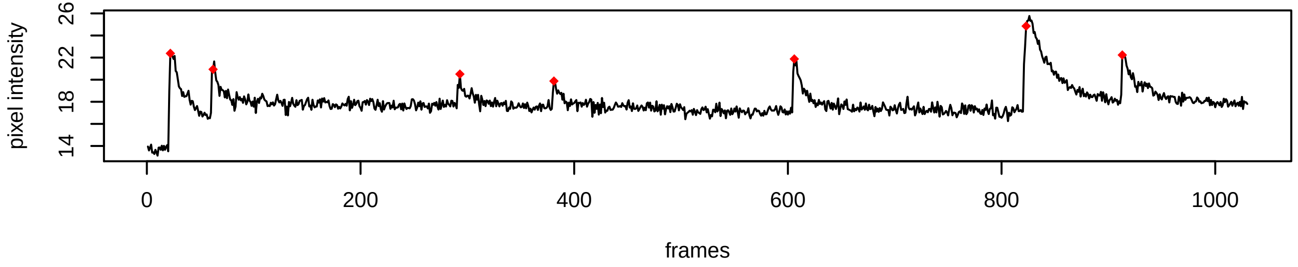

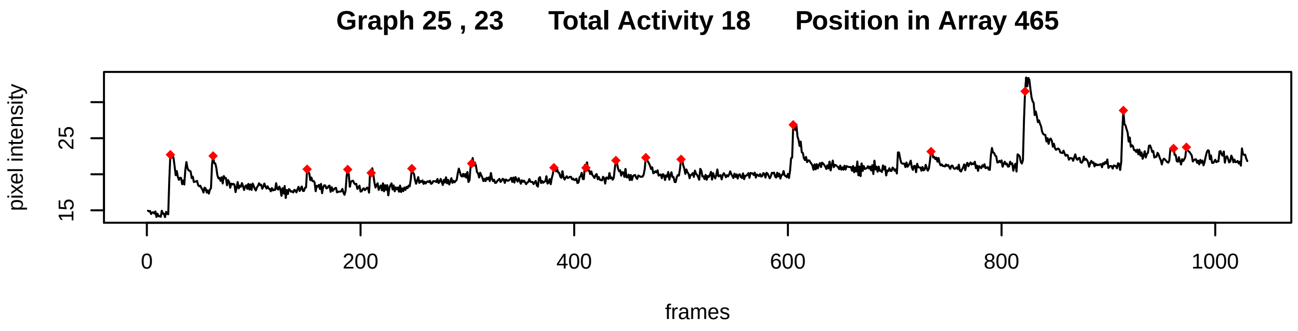
Graph 25 , 23 Total Activity 18 Position in Array 465
pixel intensity
15
25
0
200
400
600
800
1000
frames

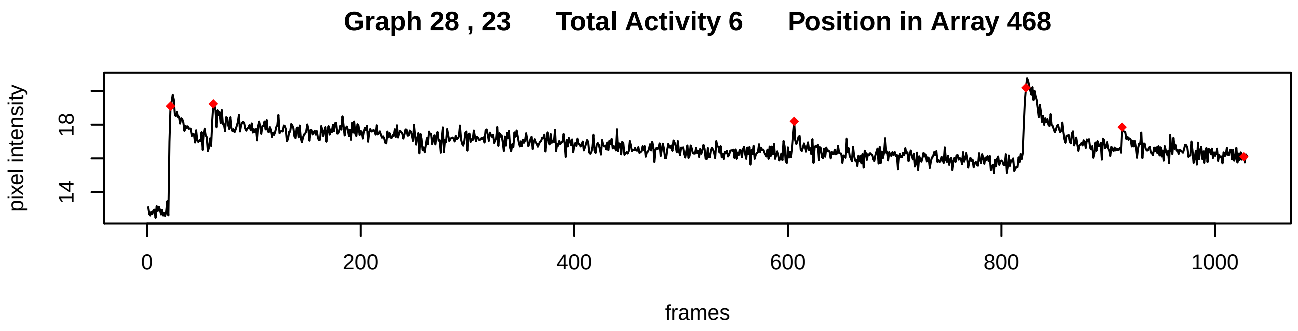
Graph 28 , 23 Total Activity 6 Position in Array 468
pixel intensity
14
18
0
200
400
600
800
1000
frames

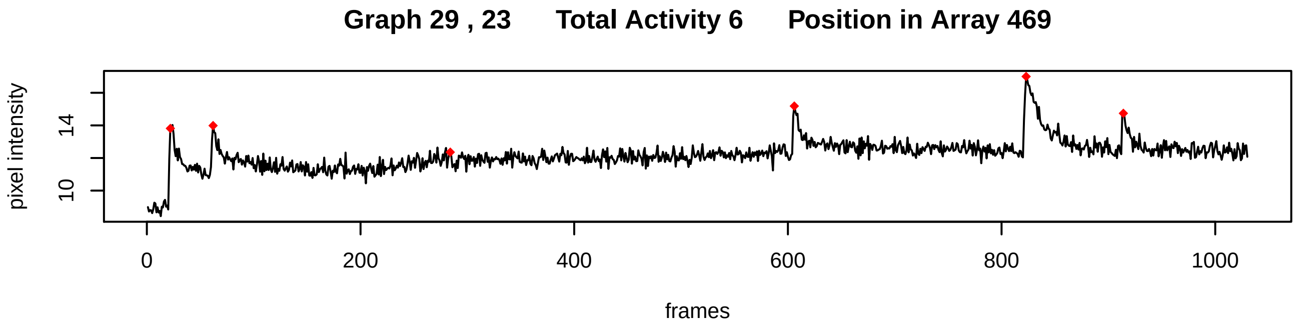
Graph 29 , 23 Total Activity 6 Position in Array 469
pixel intensity
10
14
0
200
400
600
800
1000
frames

Graph 34 , 23   Total Activity 10   Position in Array 474

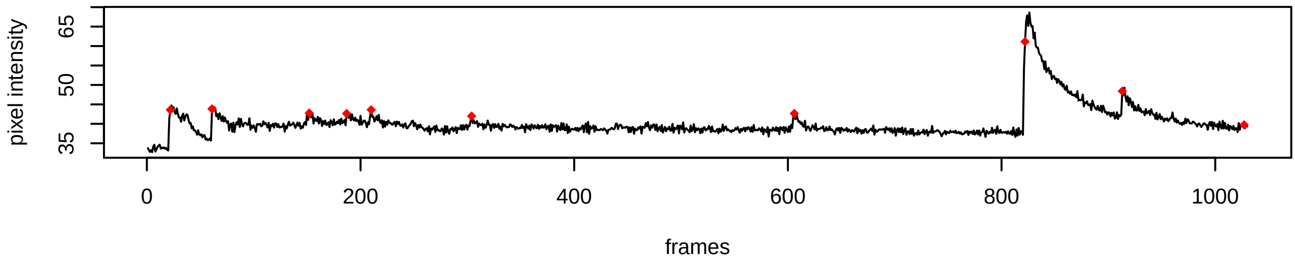

Graph 35 , 23   Total Activity 6   Position in Array 475

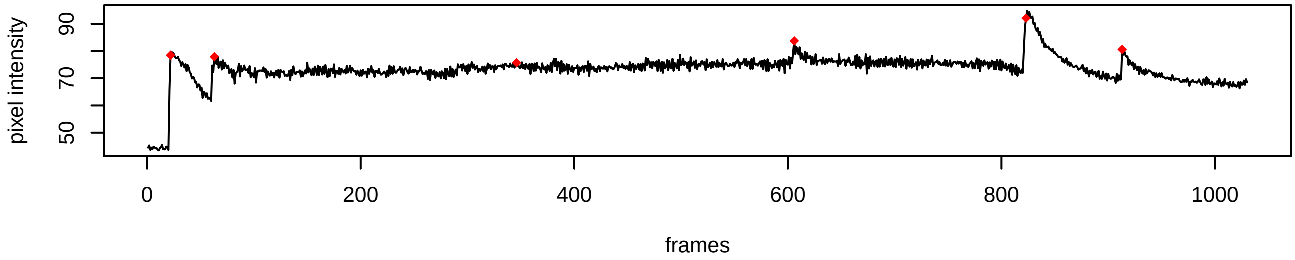

Graph 36 , 23   Total Activity 8   Position in Array 476

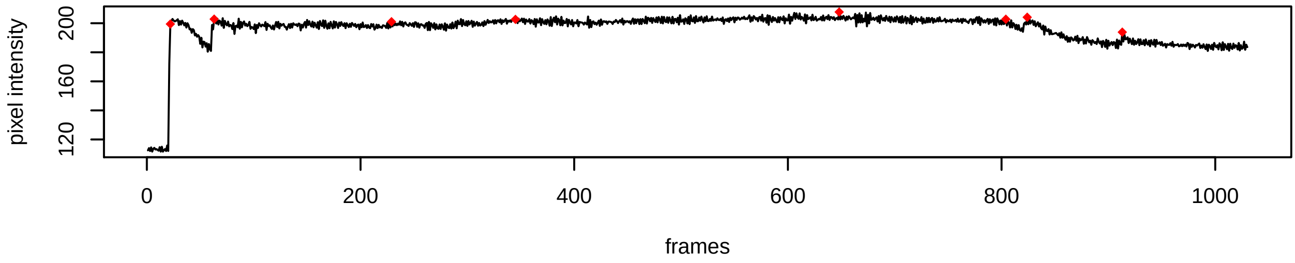

Graph 37 , 23 Total Activity 7 Position in Array 477

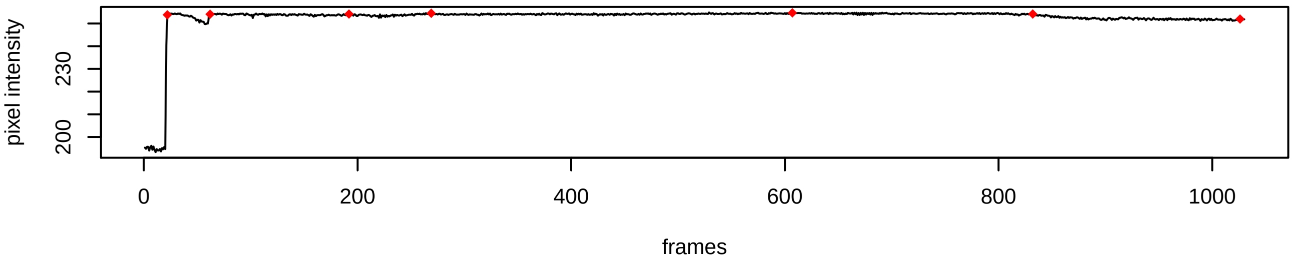

Graph 38 , 23 Total Activity 7 Position in Array 478

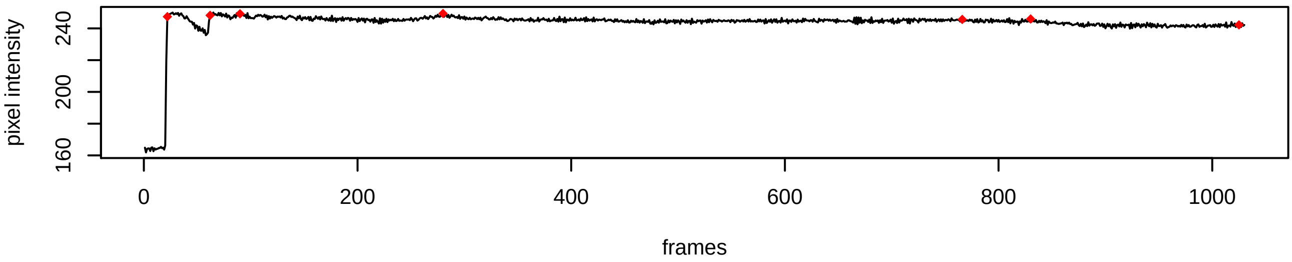

Graph 39 , 23 Total Activity 6 Position in Array 479

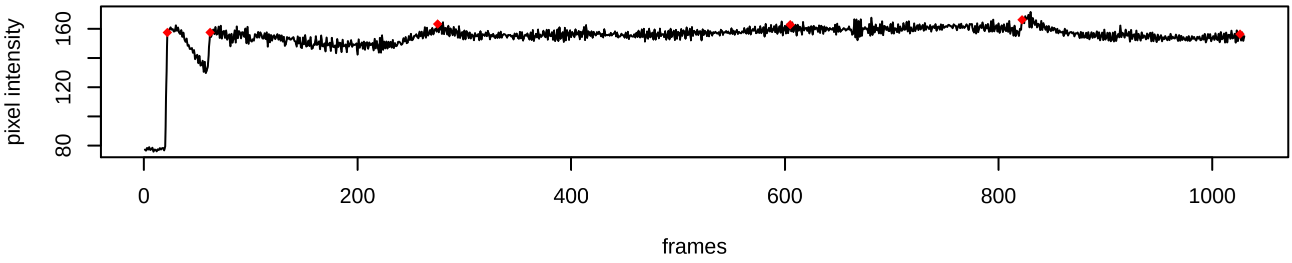

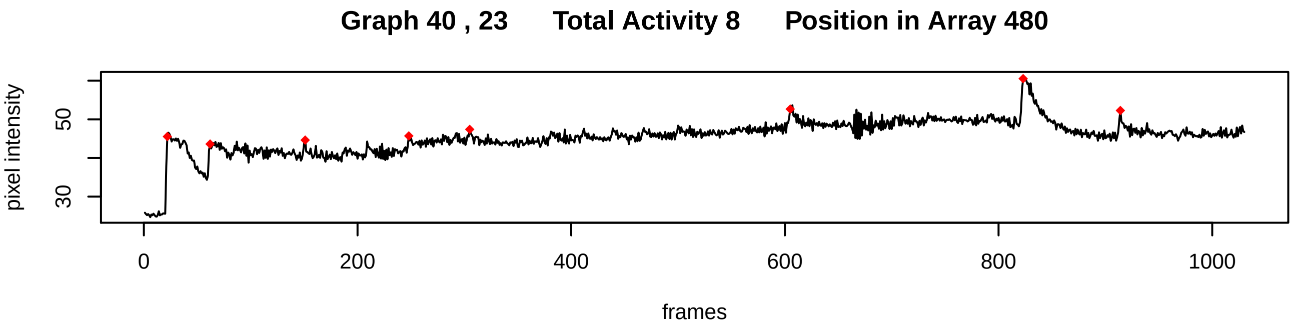
Graph 40 , 23 Total Activity 8 Position in Array 480
pixel intensity
30
50
0
200
400
600
800
1000
frames

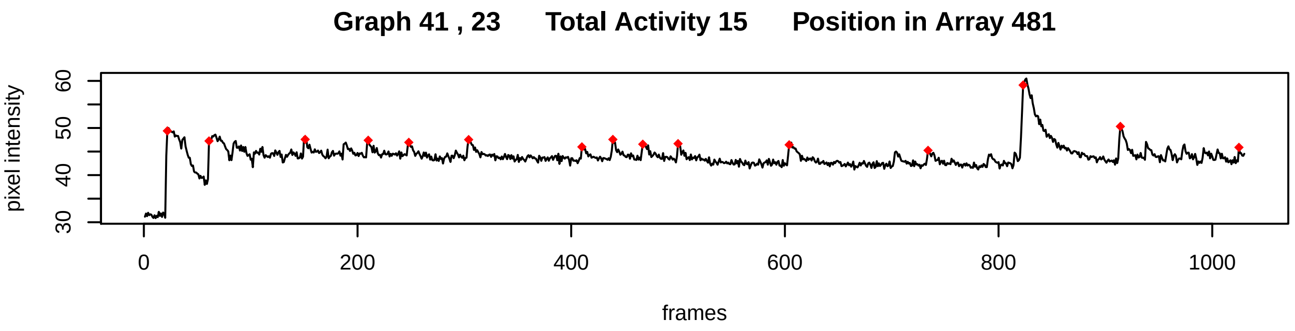
Graph 41 , 23 Total Activity 15 Position in Array 481
pixel intensity
30
40
50
60
0
200
400
600
800
1000
frames

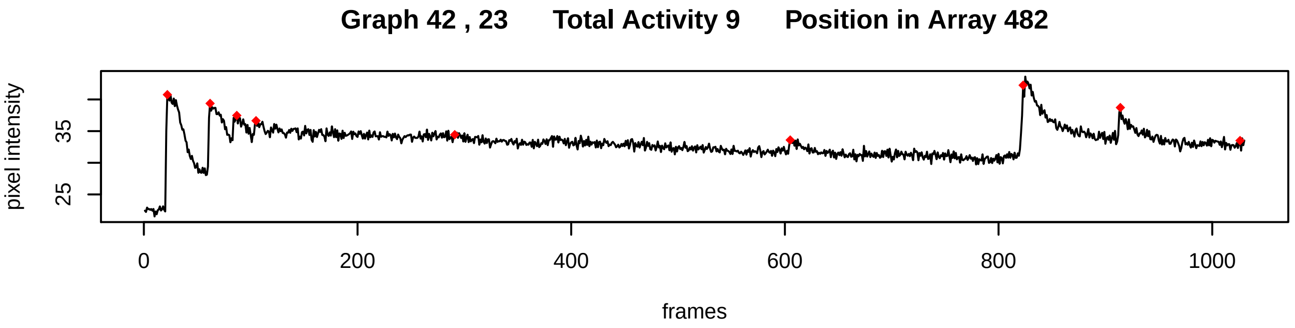
Graph 42 , 23 Total Activity 9 Position in Array 482
pixel intensity
25
35
0
200
400
600
800
1000
frames

Graph 43 , 23   Total Activity 8   Position in Array 483

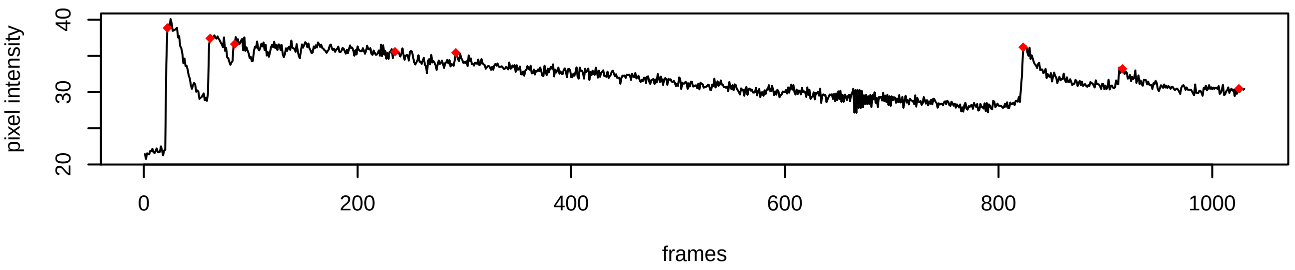

Graph 44 , 23   Total Activity 8   Position in Array 484

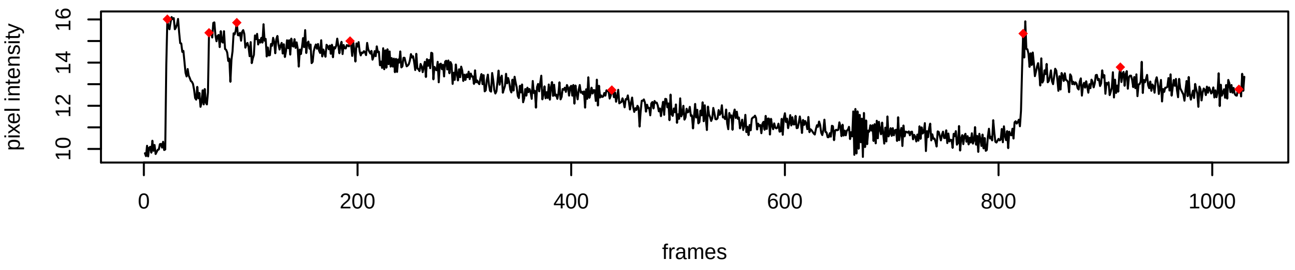

Graph 1 , 22   Total Activity 8   Position in Array 485

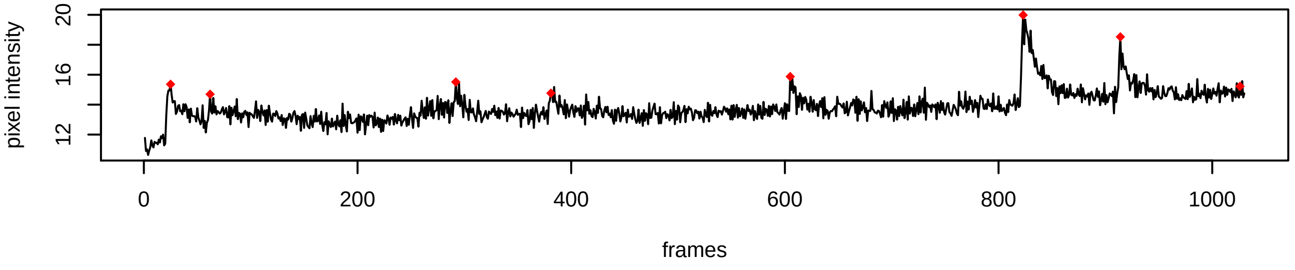

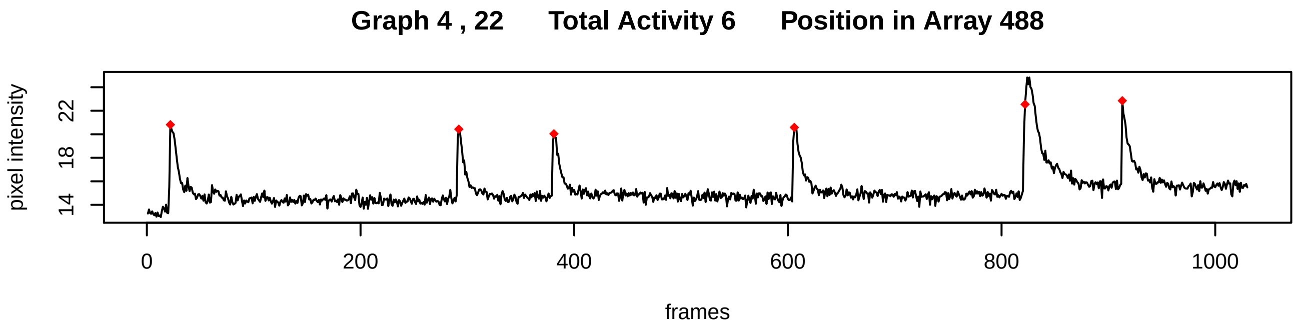
Graph 4 , 22 Total Activity 6 Position in Array 488
pixel intensity
14
18
22
0
200
400
600
800
1000
frames

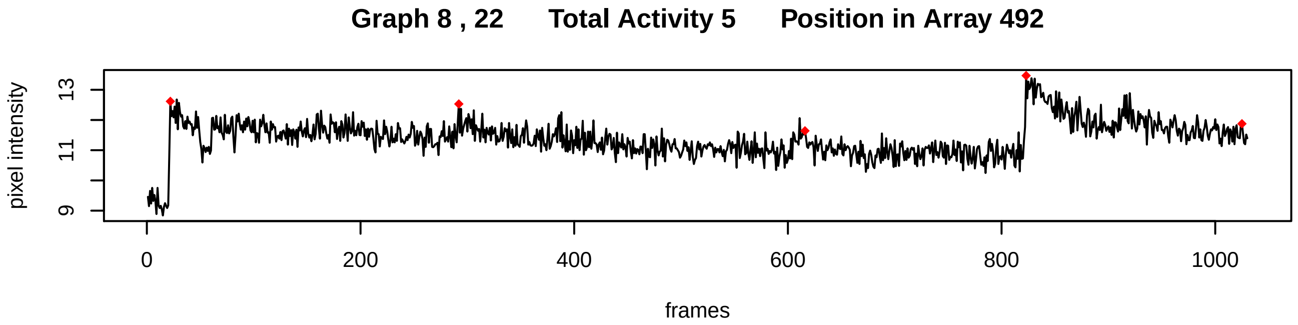
Graph 8 , 22 Total Activity 5 Position in Array 492
pixel intensity
9
11
13
0
200
400
600
800
1000
frames

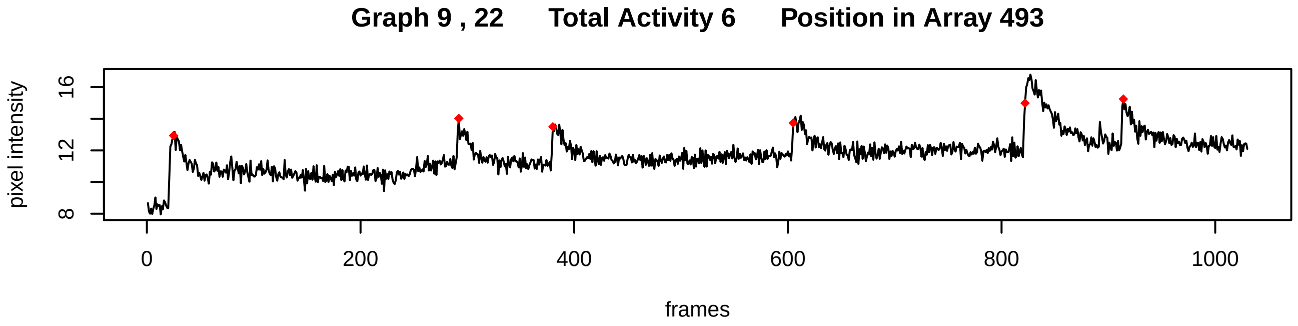
Graph 9 , 22 Total Activity 6 Position in Array 493
pixel intensity
8
12
16
0
200
400
600
800
1000
frames

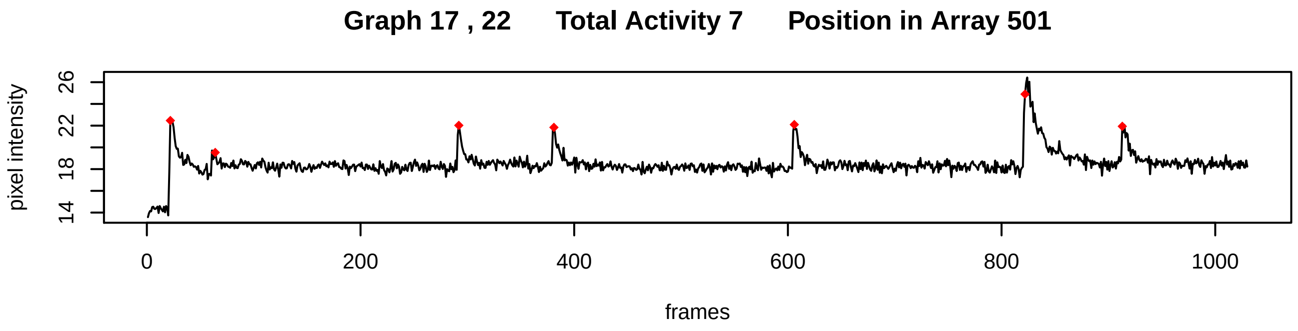
Graph 17 , 22 Total Activity 7 Position in Array 501
pixel intensity
14
18
22
26
0
200
400
600
800
1000
frames

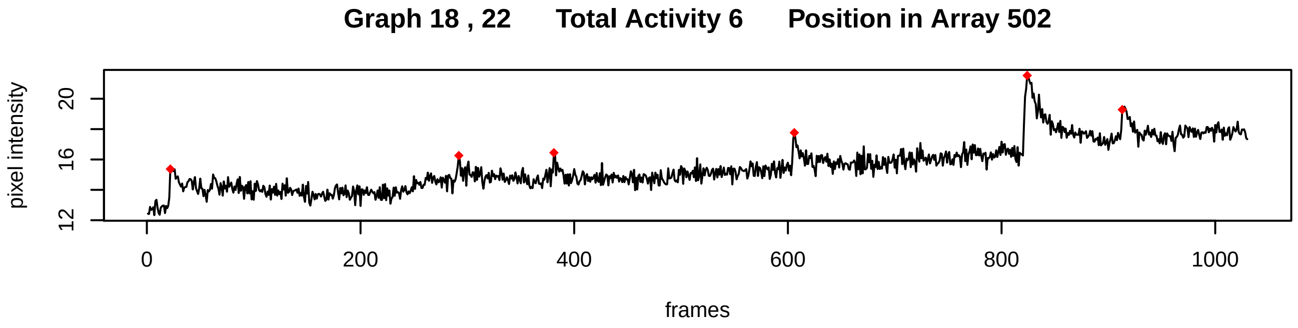
Graph 18 , 22 Total Activity 6 Position in Array 502
pixel intensity
12
16
20
0
200
400
600
800
1000
frames

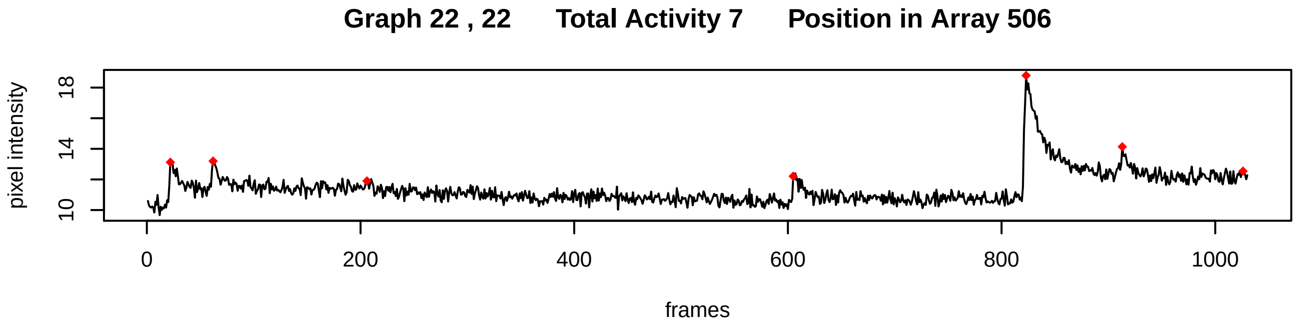
Graph 22 , 22 Total Activity 7 Position in Array 506
pixel intensity
10
14
18
0
200
400
600
800
1000
frames

Graph 26 , 22   Total Activity 8   Position in Array 510

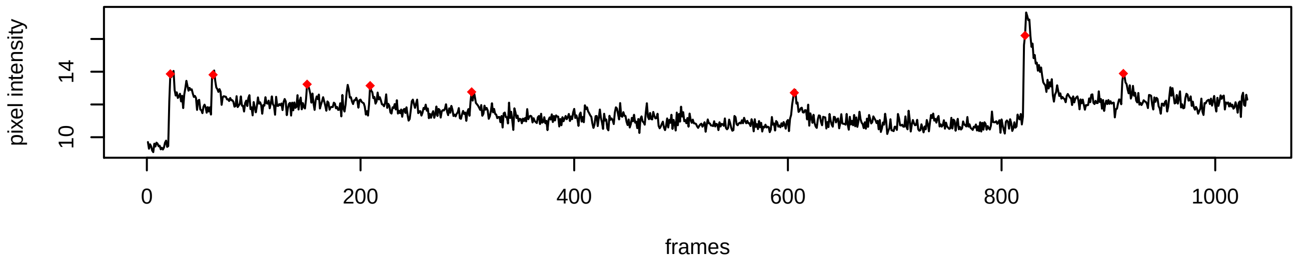

Graph 27 , 22   Total Activity 5   Position in Array 511

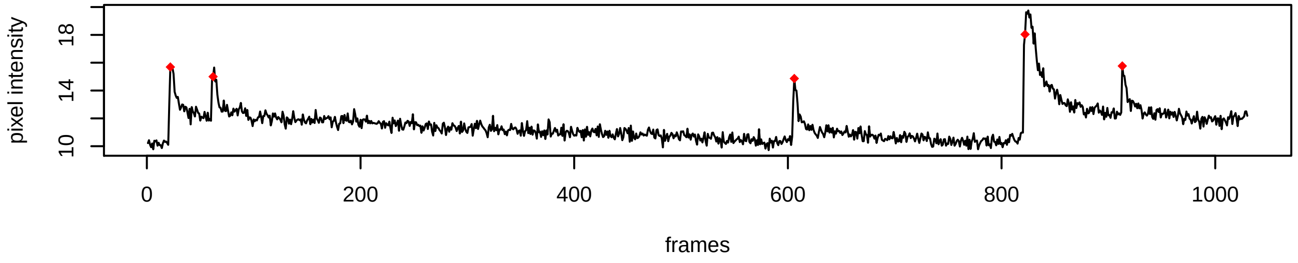

Graph 28 , 22   Total Activity 7   Position in Array 512

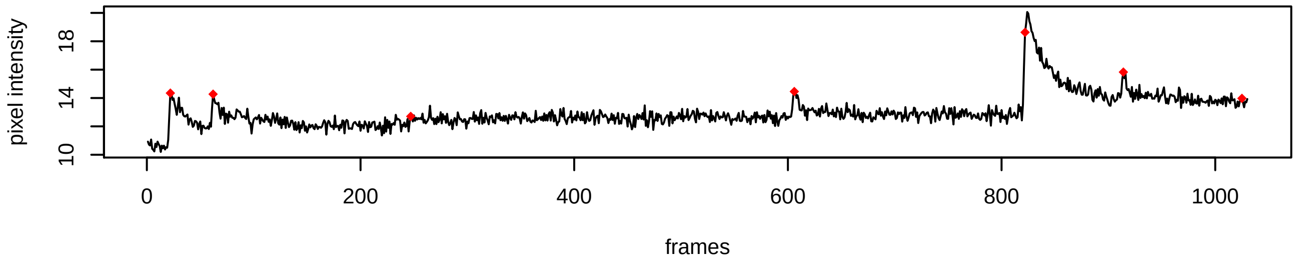

Graph 33 , 22 Total Activity 14 Position in Array 517

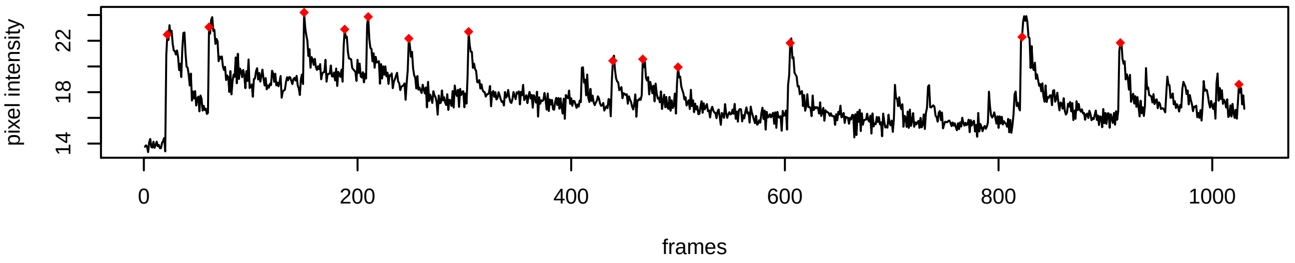

Graph 34 , 22 Total Activity 17 Position in Array 518

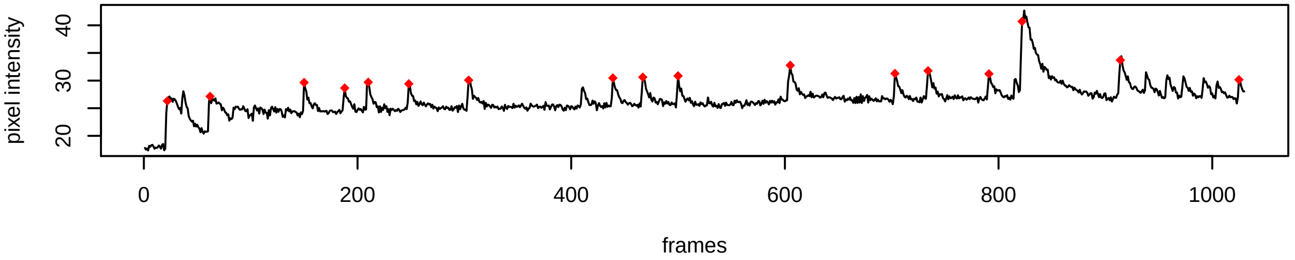

Graph 35 , 22 Total Activity 6 Position in Array 519

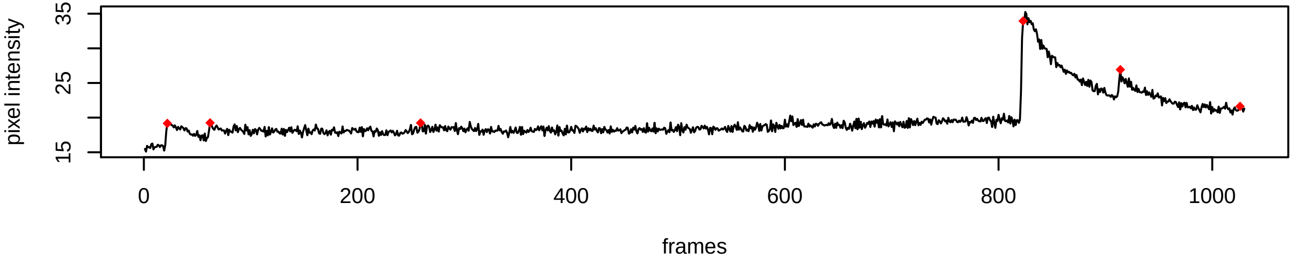

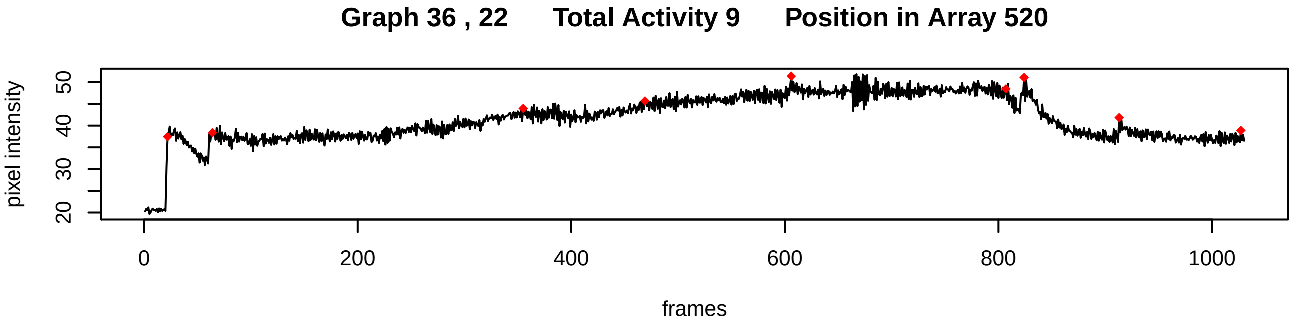

Graph 36 , 22 Total Activity 9 Position in Array 520
pixel intensity
frames

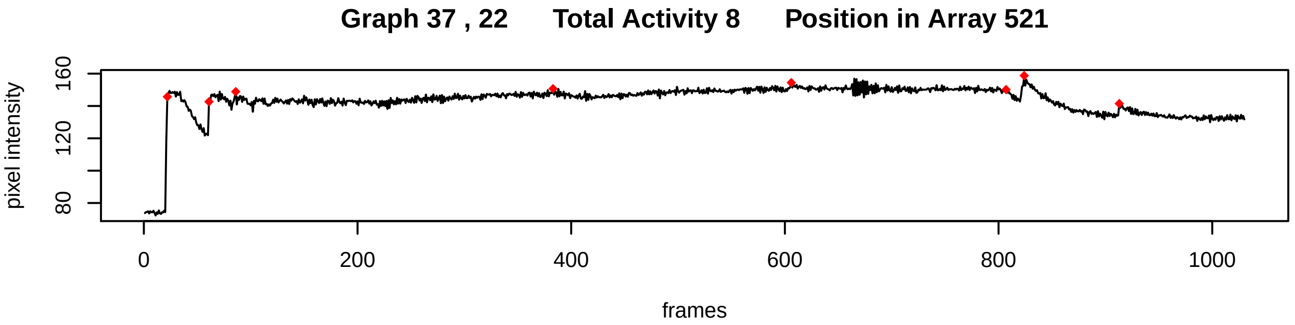

Graph 37 , 22 Total Activity 8 Position in Array 521
pixel intensity
frames

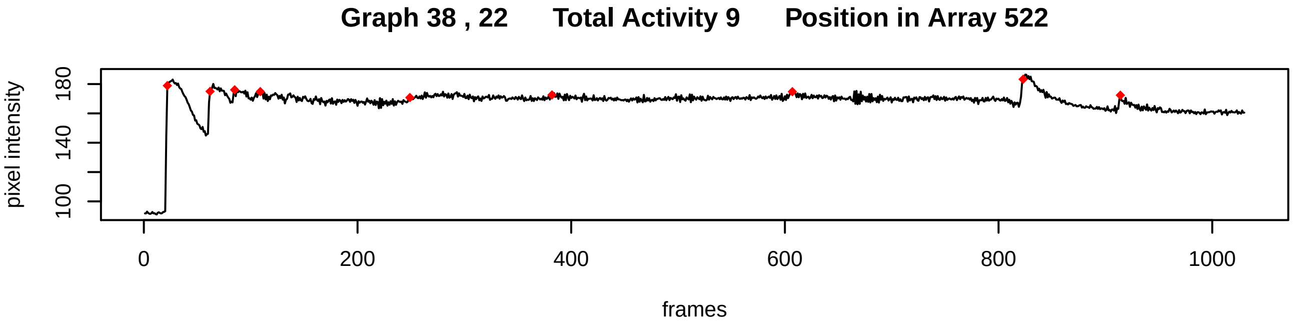

Graph 38 , 22 Total Activity 9 Position in Array 522
pixel intensity
frames

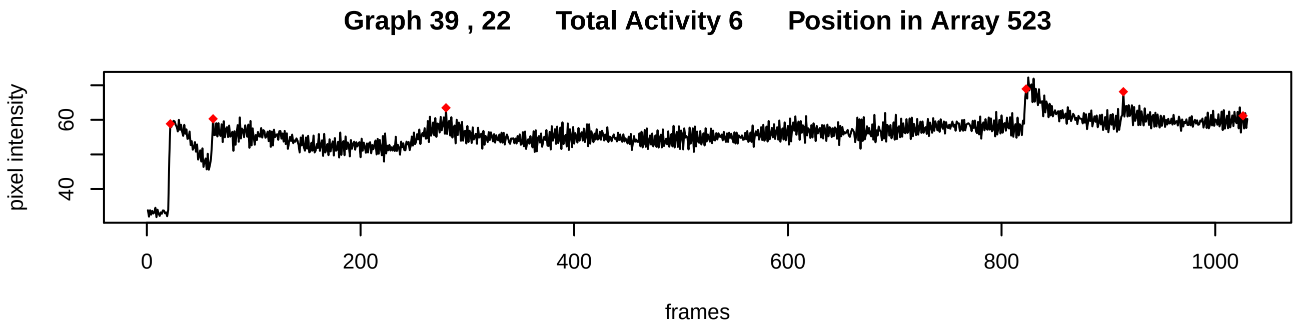

Graph 39 , 22 Total Activity 6 Position in Array 523
pixel intensity
40
60
0
200
400
600
800
1000
frames

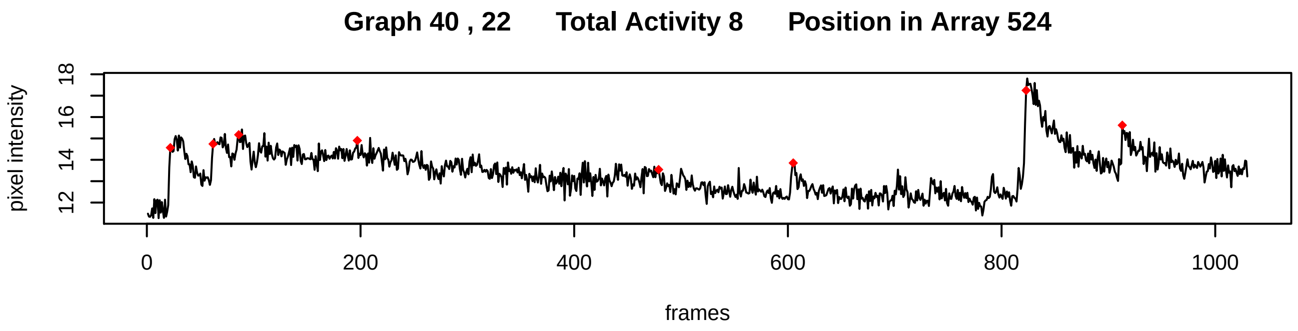

Graph 40 , 22 Total Activity 8 Position in Array 524
pixel intensity
12
14
16
18
0
200
400
600
800
1000
frames

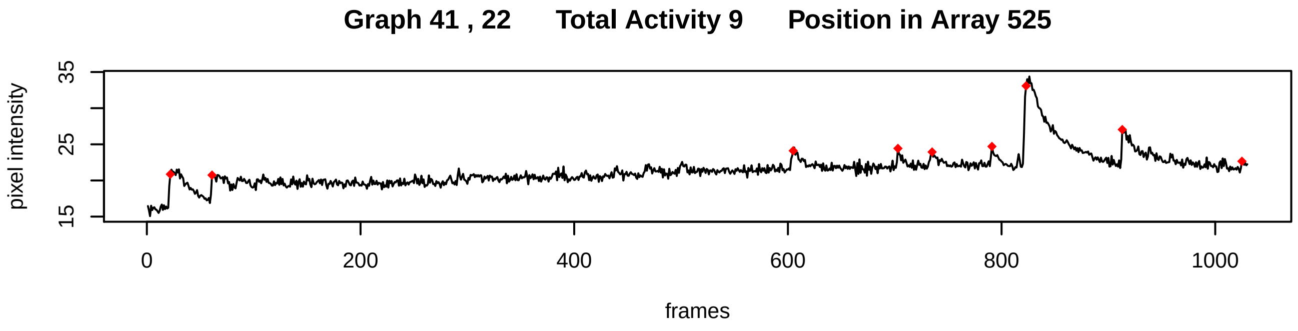

Graph 41 , 22 Total Activity 9 Position in Array 525
pixel intensity
15
25
35
0
200
400
600
800
1000
frames

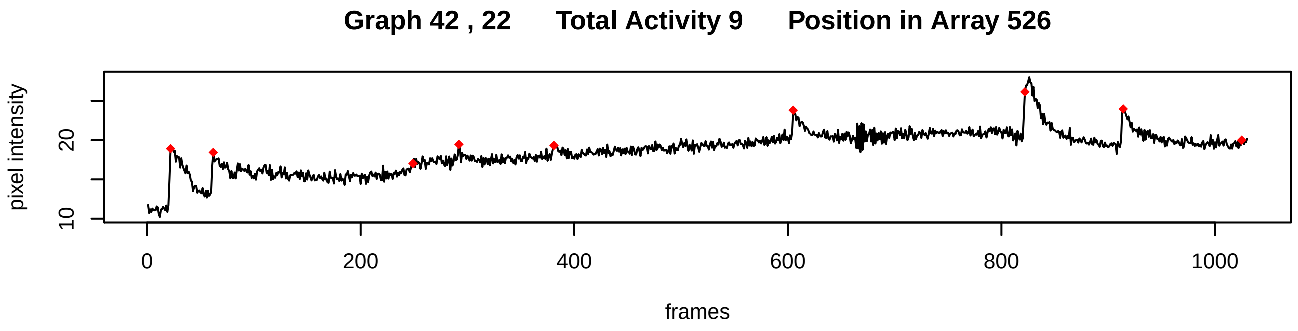
Graph 42 , 22 Total Activity 9 Position in Array 526
pixel intensity
10
20
0
200
400
600
800
1000
frames

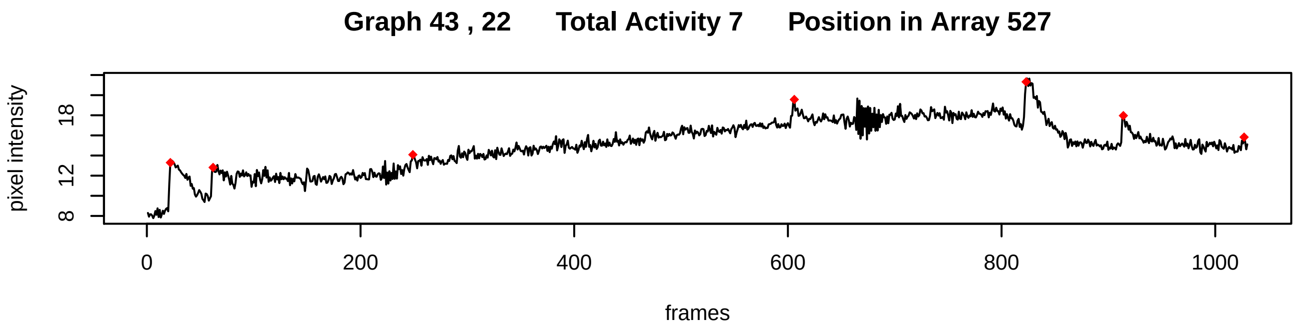
Graph 43 , 22 Total Activity 7 Position in Array 527
pixel intensity
8
12
18
0
200
400
600
800
1000
frames

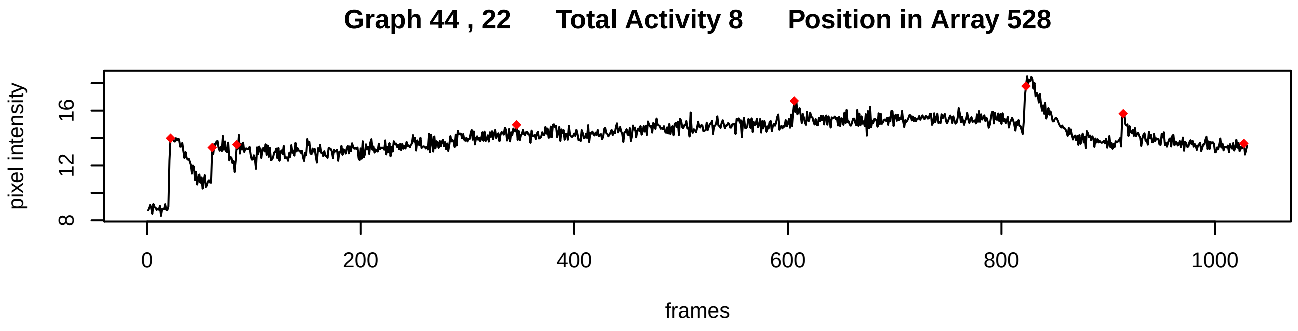
Graph 44 , 22 Total Activity 8 Position in Array 528
pixel intensity
8
12
16
0
200
400
600
800
1000
frames

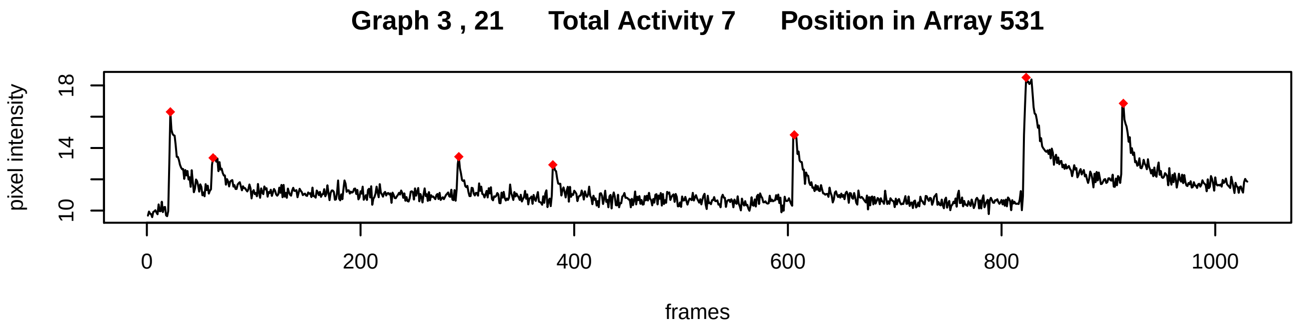
Graph 3 , 21   Total Activity 7   Position in Array 531
pixel intensity
10
14
18
0
200
400
600
800
1000
frames

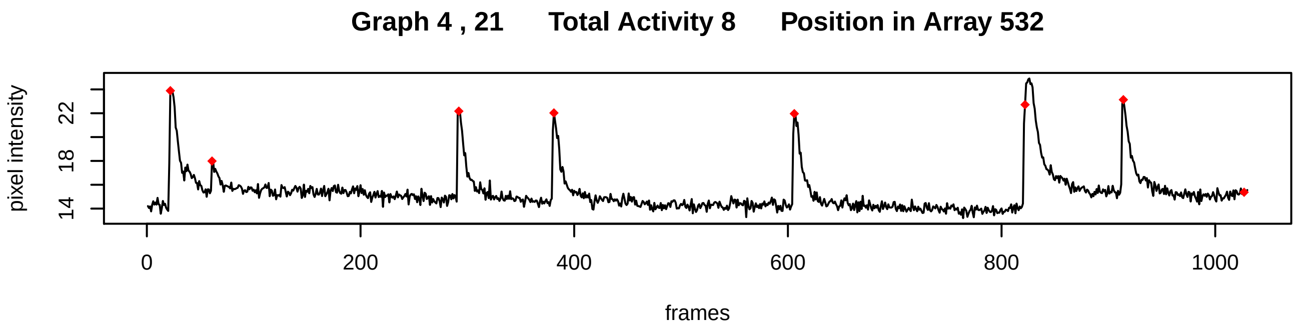
Graph 4 , 21   Total Activity 8   Position in Array 532
pixel intensity
14
18
22
0
200
400
600
800
1000
frames

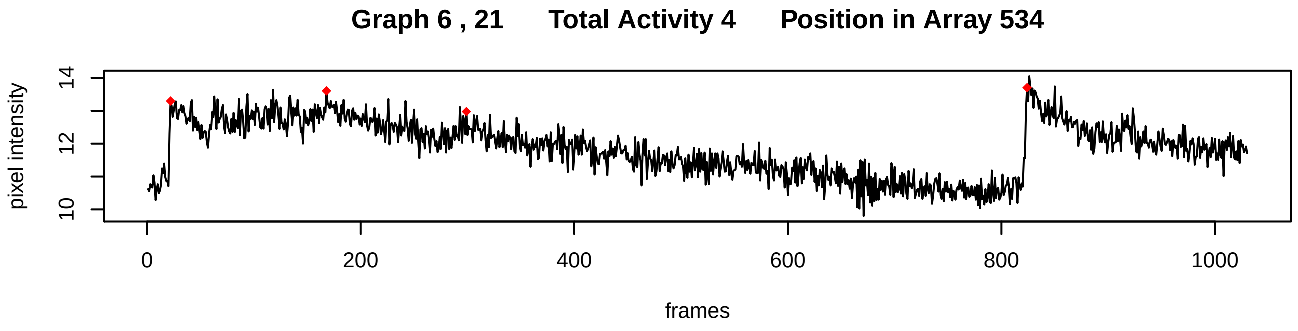
Graph 6 , 21   Total Activity 4   Position in Array 534
pixel intensity
10
12
14
0
200
400
600
800
1000
frames

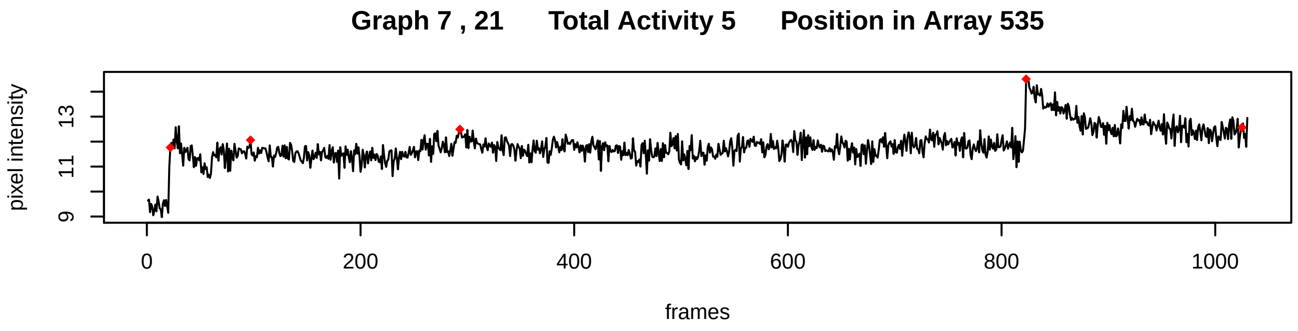
Graph 7 , 21 Total Activity 5 Position in Array 535
pixel intensity
9
11
13
0
200
400
600
800
1000
frames

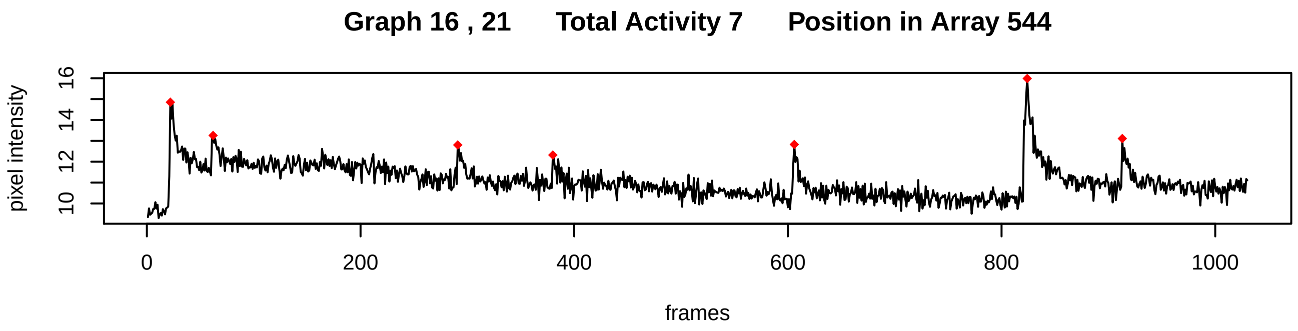
Graph 16 , 21 Total Activity 7 Position in Array 544
pixel intensity
10
12
14
16
0
200
400
600
800
1000
frames

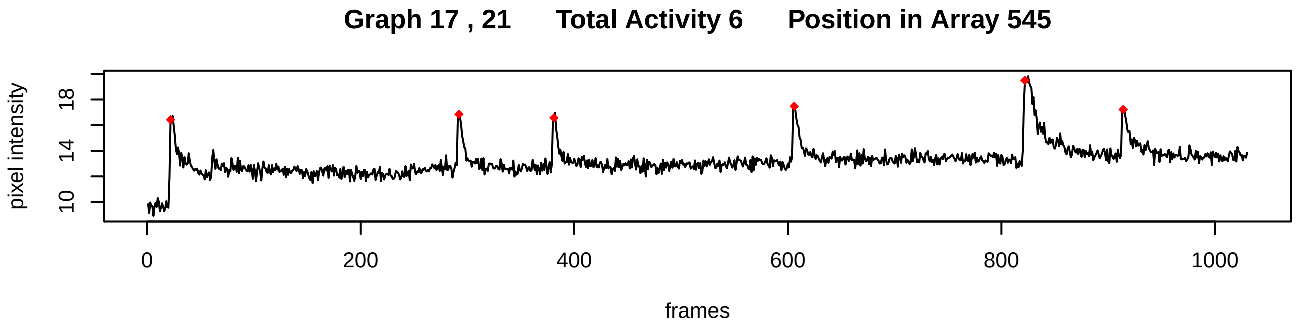
Graph 17 , 21 Total Activity 6 Position in Array 545
pixel intensity
10
14
18
0
200
400
600
800
1000
frames

Graph 19 , 21 Total Activity 6 Position in Array 547

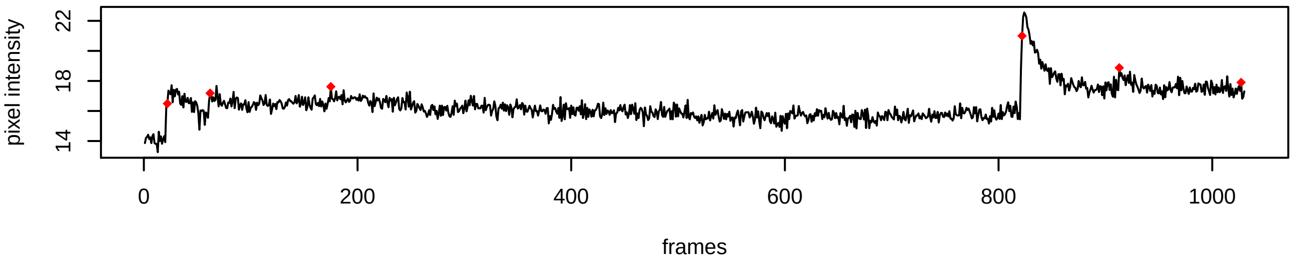

Graph 20 , 21 Total Activity 6 Position in Array 548

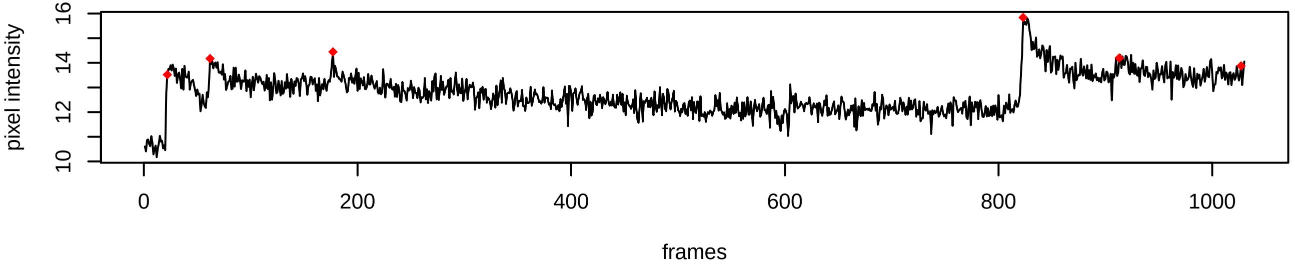

Graph 21 , 21 Total Activity 6 Position in Array 549

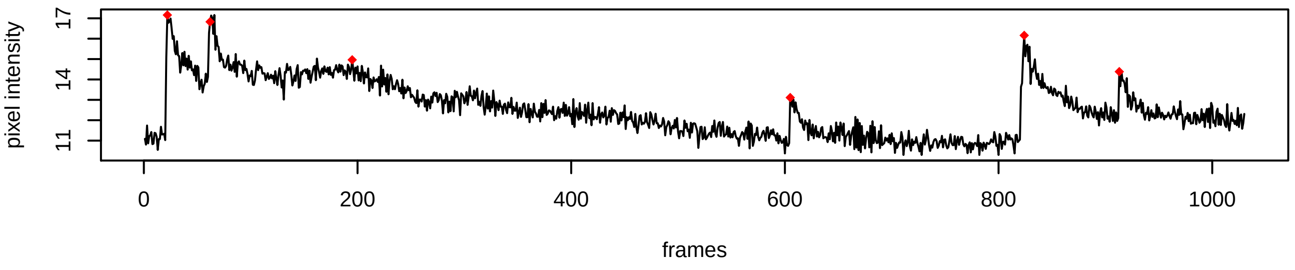

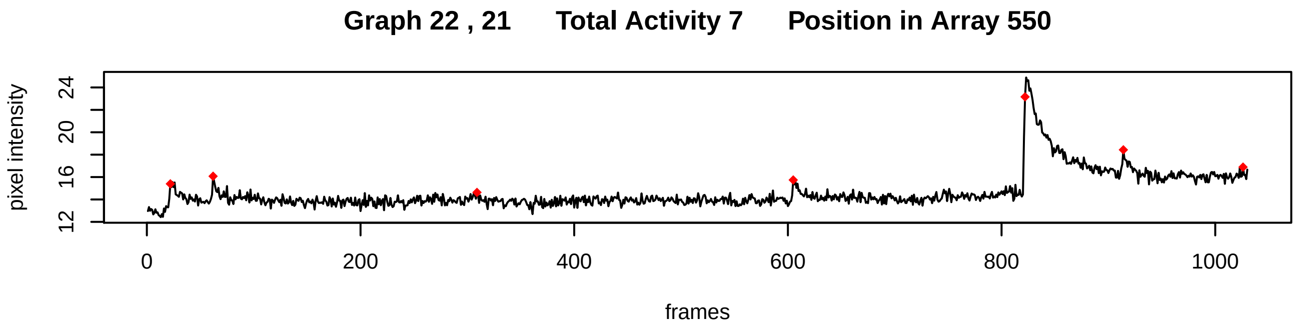
Graph 22 , 21 Total Activity 7 Position in Array 550
pixel intensity
12
16
20
24
0
200
400
600
800
1000
frames

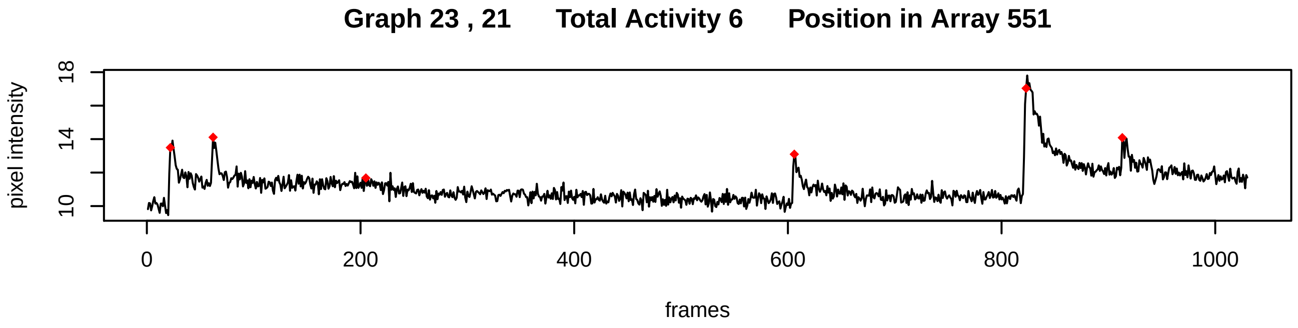
Graph 23 , 21 Total Activity 6 Position in Array 551
pixel intensity
10
14
18
0
200
400
600
800
1000
frames

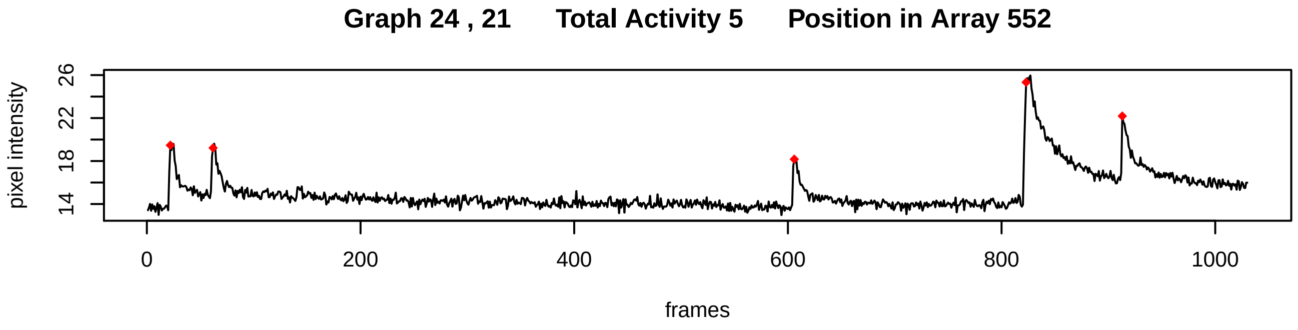
Graph 24 , 21 Total Activity 5 Position in Array 552
pixel intensity
14
18
22
26
0
200
400
600
800
1000
frames

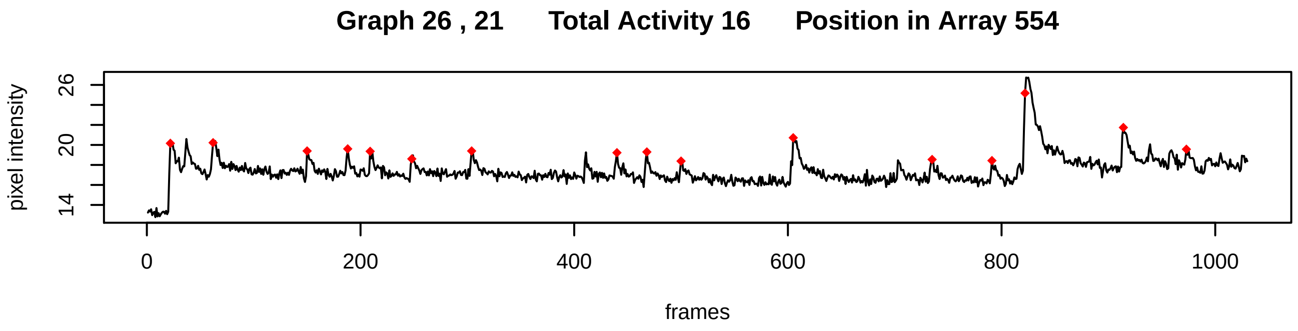
Graph 26 , 21 Total Activity 16 Position in Array 554
pixel intensity
14
20
26
0
200
400
600
800
1000
frames

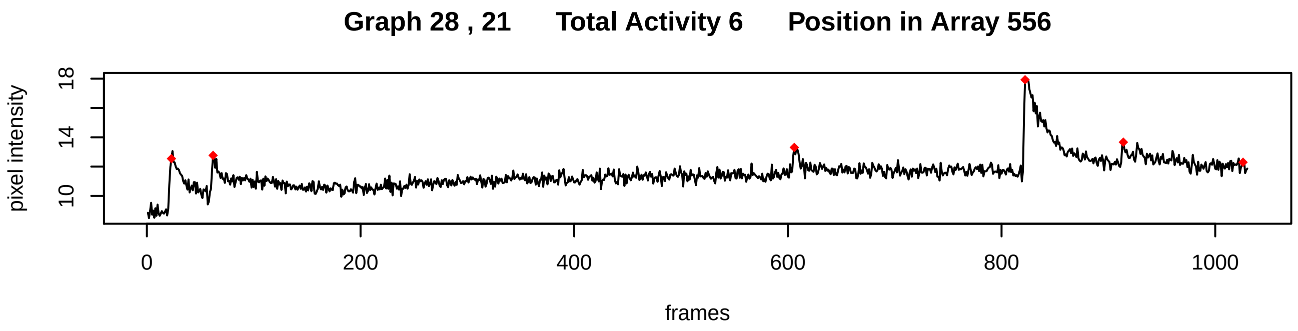
Graph 28 , 21 Total Activity 6 Position in Array 556
pixel intensity
10
14
18
0
200
400
600
800
1000
frames

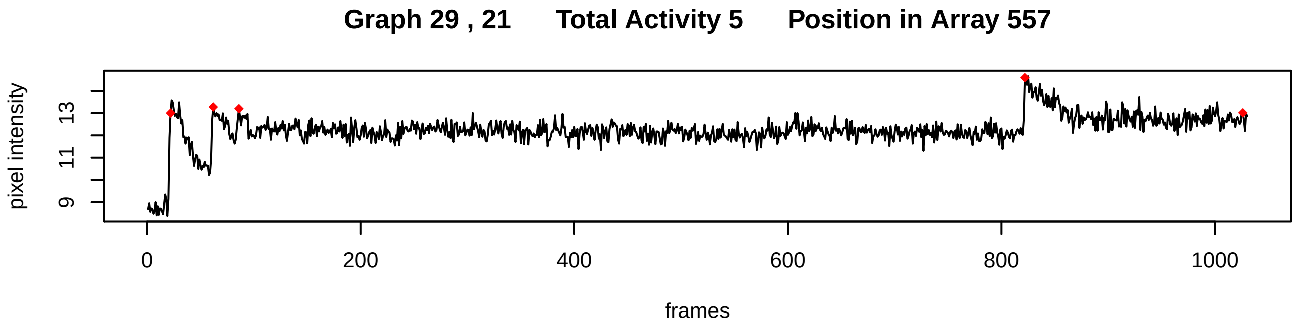
Graph 29 , 21 Total Activity 5 Position in Array 557
pixel intensity
9
11
13
0
200
400
600
800
1000
frames

Graph 31 , 21   Total Activity 6   Position in Array 559

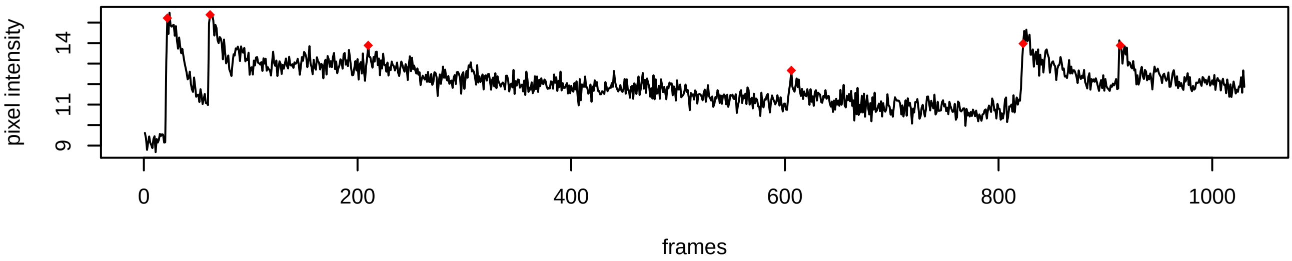

Graph 32 , 21   Total Activity 16   Position in Array 560

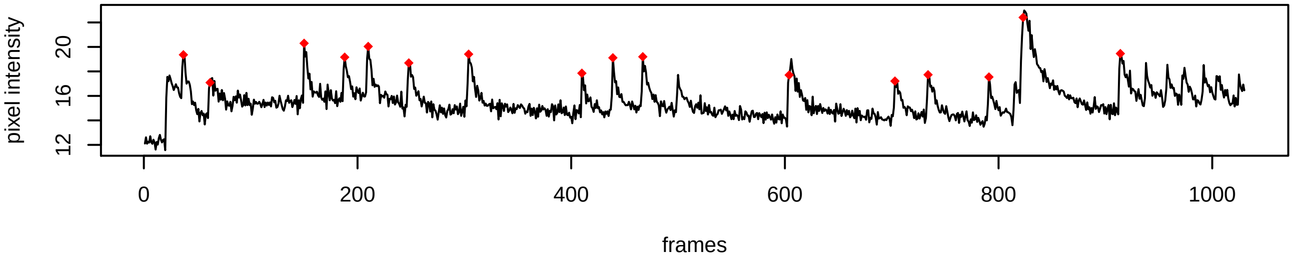

Graph 33 , 21   Total Activity 10   Position in Array 561

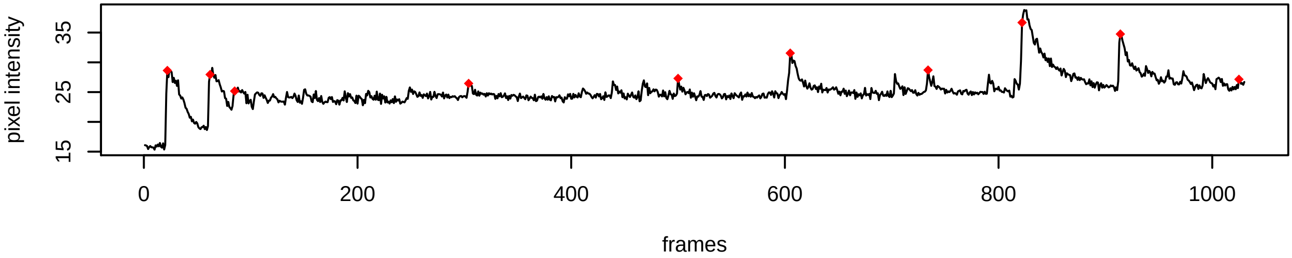

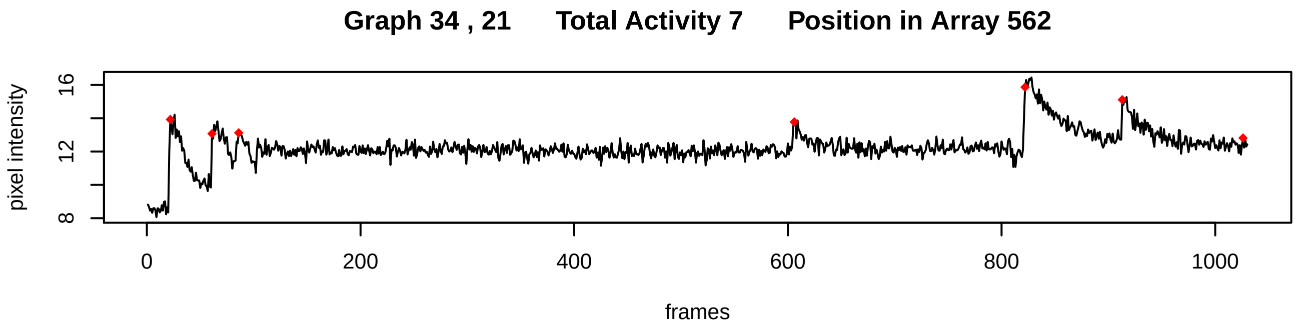
Graph 34 , 21 Total Activity 7 Position in Array 562
pixel intensity
8
12
16
0
200
400
600
800
1000
frames

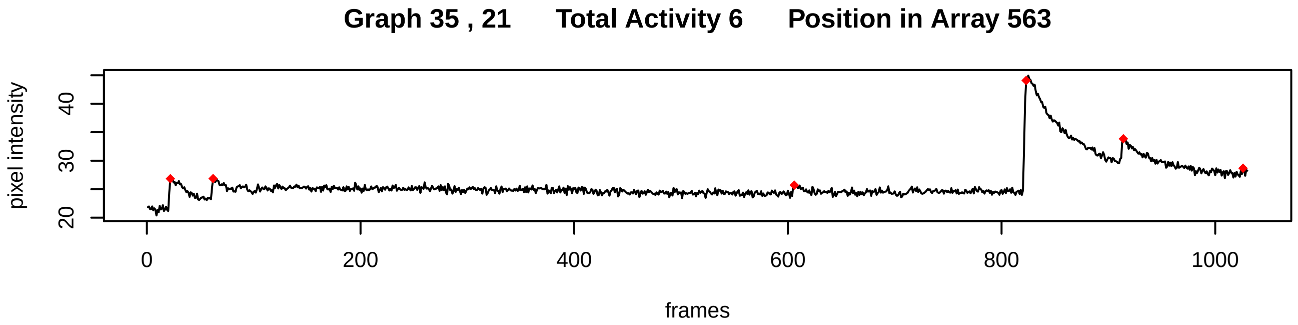
Graph 35 , 21 Total Activity 6 Position in Array 563
pixel intensity
20
30
40
0
200
400
600
800
1000
frames

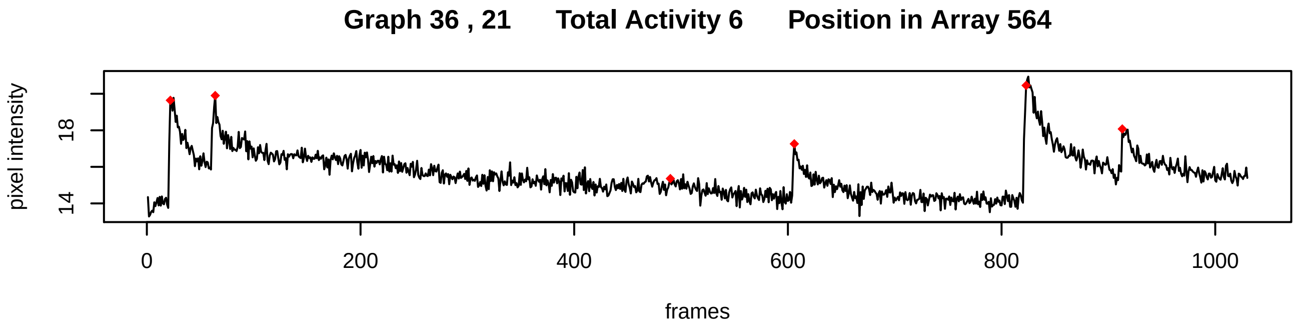
Graph 36 , 21 Total Activity 6 Position in Array 564
pixel intensity
14
18
0
200
400
600
800
1000
frames

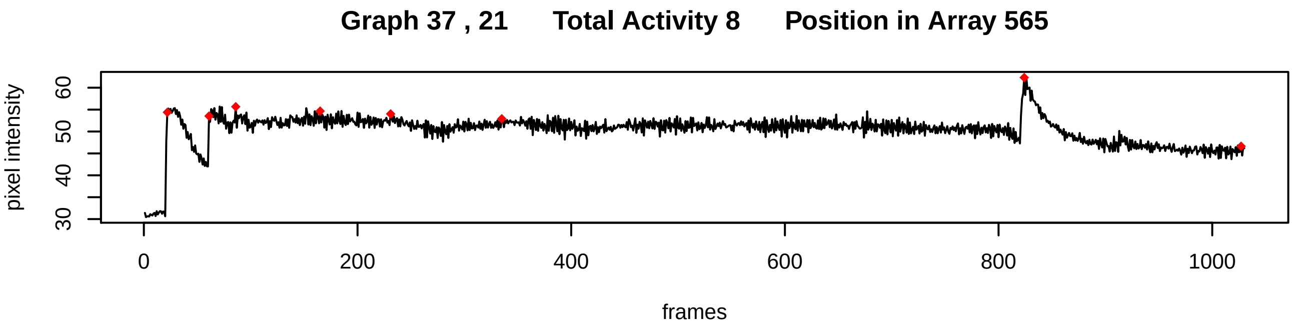
Graph 37 , 21 Total Activity 8 Position in Array 565
pixel intensity
30
40
50
60
0
200
400
600
800
1000
frames

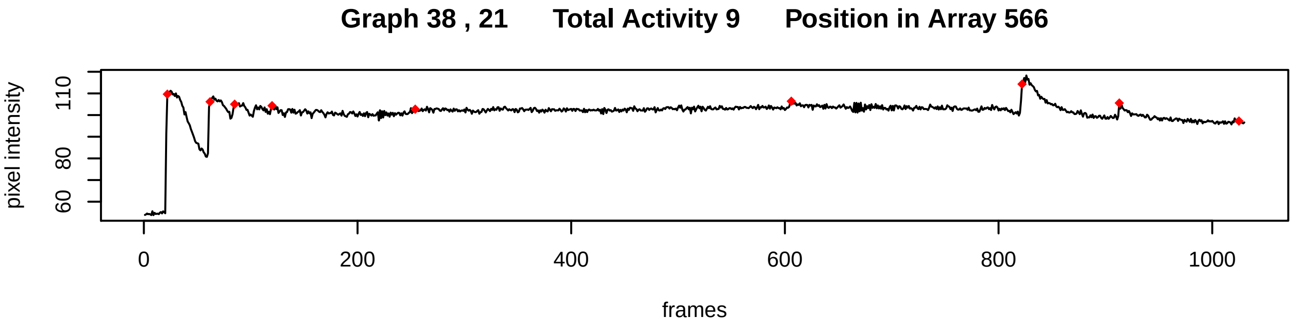
Graph 38 , 21 Total Activity 9 Position in Array 566
pixel intensity
60
80
110
0
200
400
600
800
1000
frames

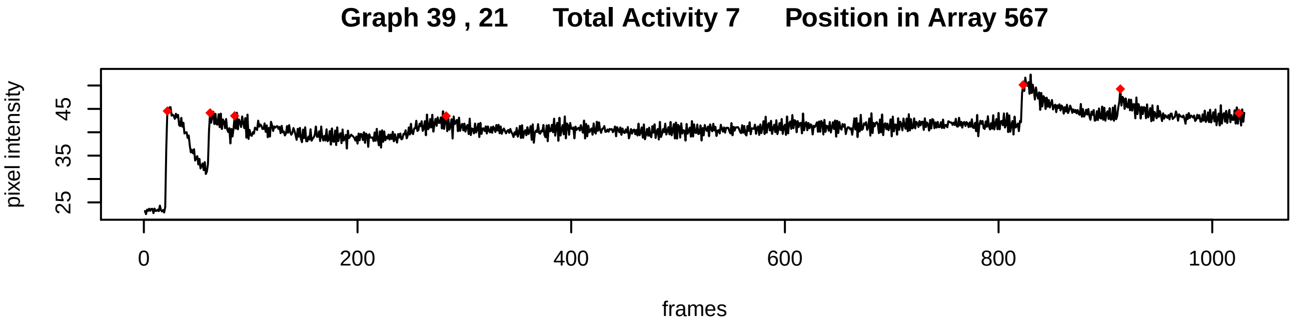
Graph 39 , 21 Total Activity 7 Position in Array 567
pixel intensity
25
35
45
0
200
400
600
800
1000
frames

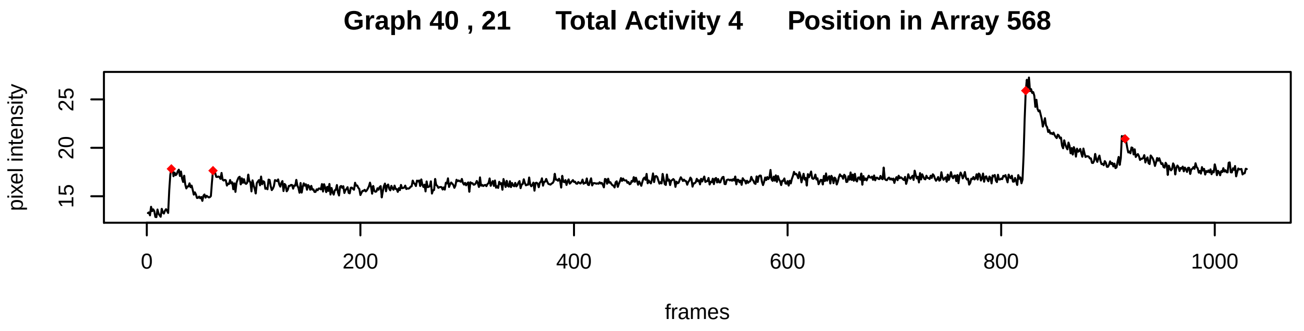
Graph 40 , 21 Total Activity 4 Position in Array 568
pixel intensity
15
20
25
0
200
400
600
800
1000
frames

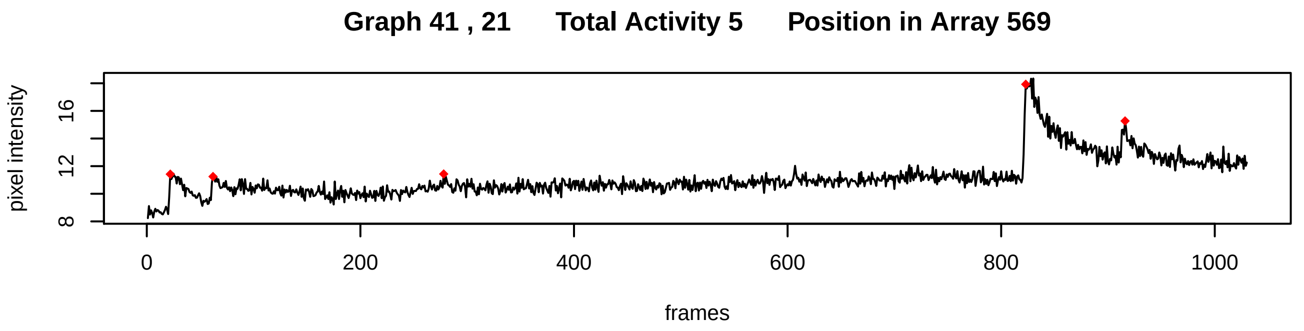
Graph 41 , 21 Total Activity 5 Position in Array 569
pixel intensity
8
12
16
0
200
400
600
800
1000
frames

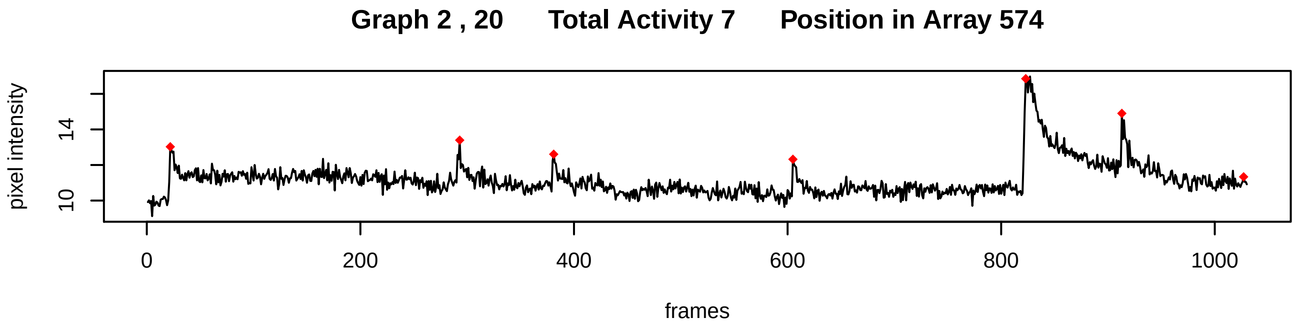
Graph 2 , 20 Total Activity 7 Position in Array 574
pixel intensity
10
14
0
200
400
600
800
1000
frames

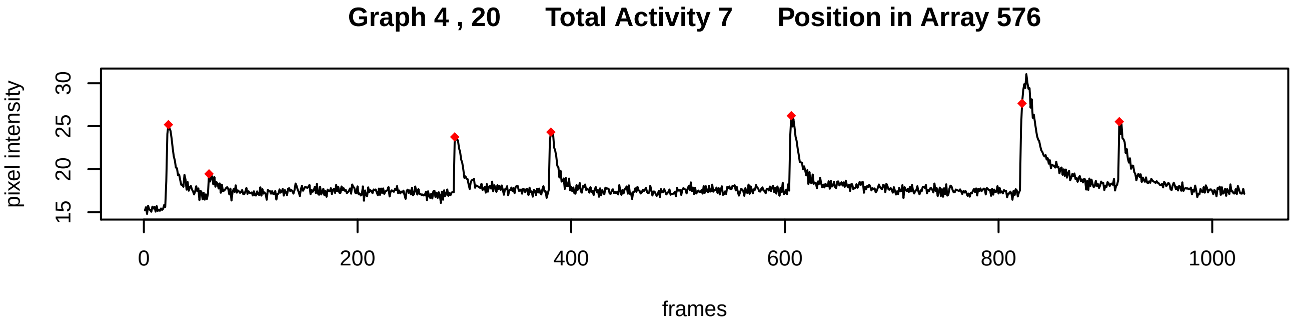
Graph 4 , 20 Total Activity 7 Position in Array 576
pixel intensity
15
20
25
30
0
200
400
600
800
1000
frames

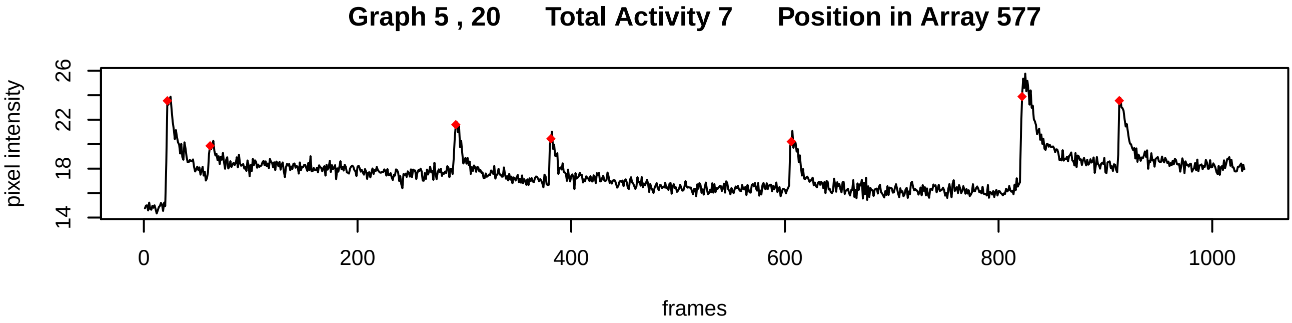
Graph 5 , 20 Total Activity 7 Position in Array 577
pixel intensity
14
18
22
26
0
200
400
600
800
1000
frames

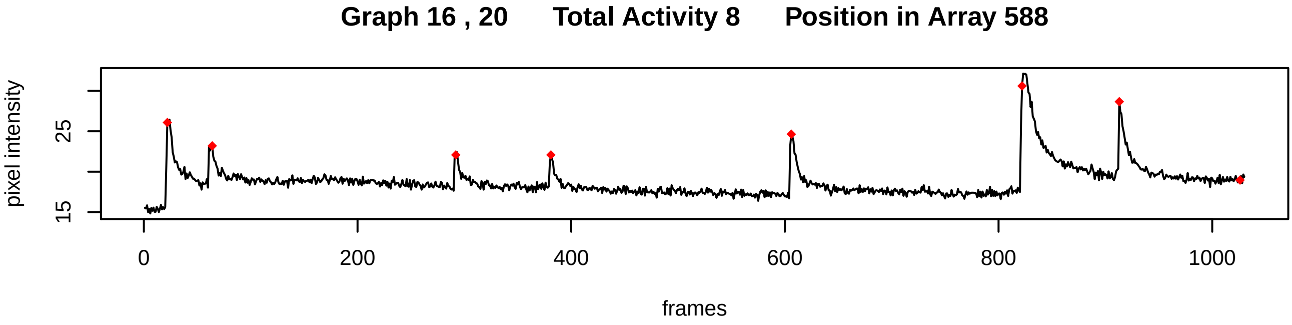
Graph 16 , 20 Total Activity 8 Position in Array 588
pixel intensity
15
25
0
200
400
600
800
1000
frames

Graph 17 , 20 Total Activity 8 Position in Array 589

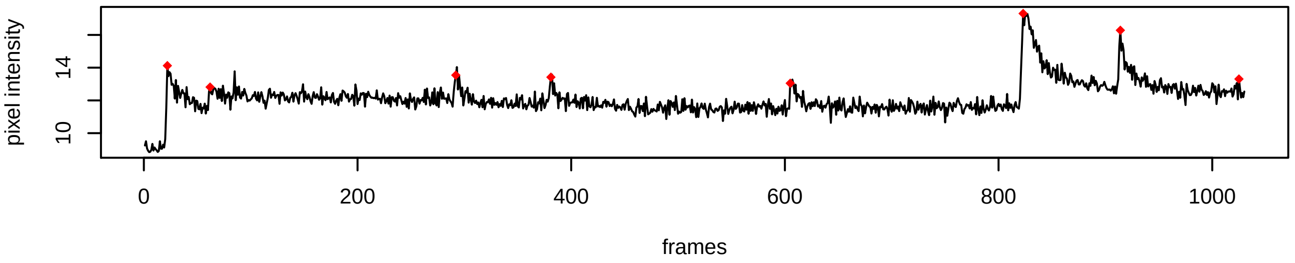

Graph 18 , 20 Total Activity 4 Position in Array 590

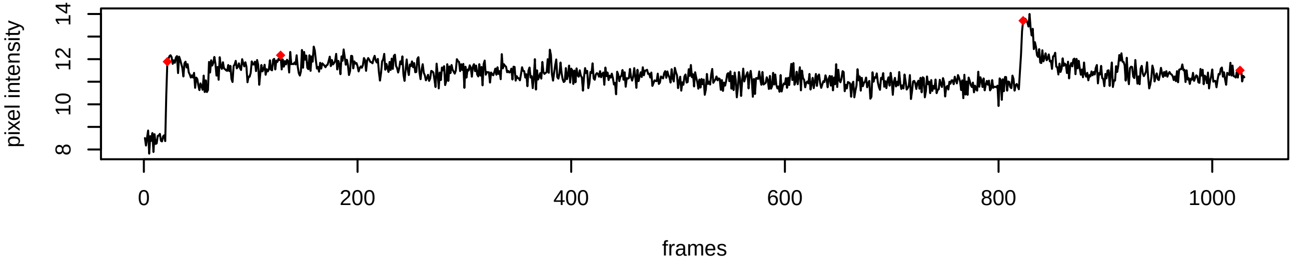

Graph 19 , 20 Total Activity 6 Position in Array 591

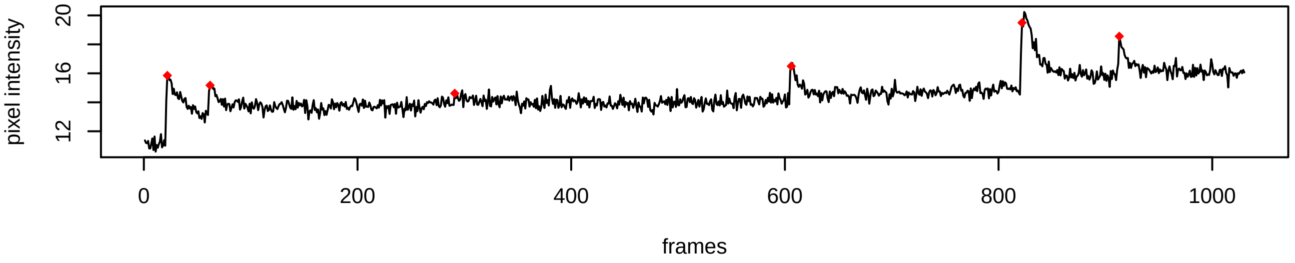

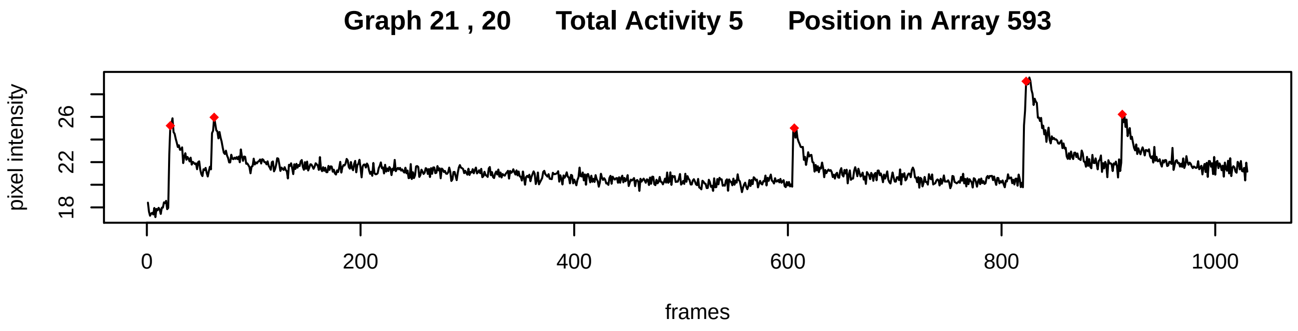
Graph 21 , 20 Total Activity 5 Position in Array 593
pixel intensity
18
22
26
0
200
400
600
800
1000
frames

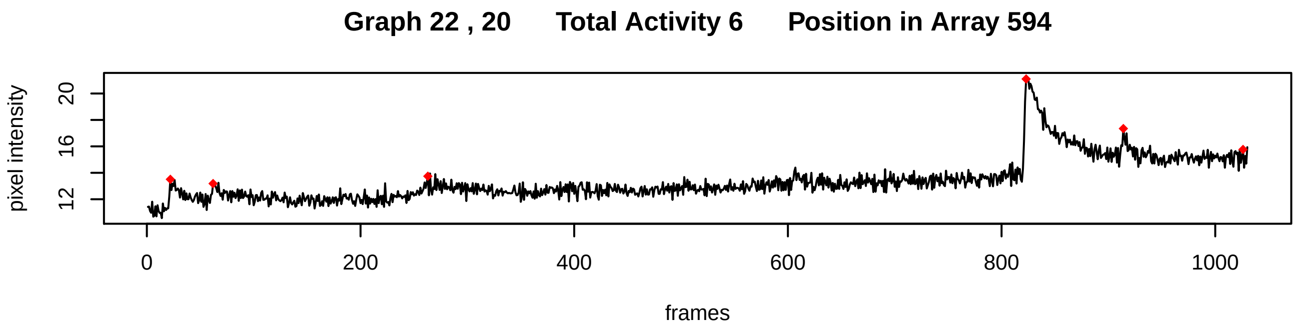
Graph 22 , 20 Total Activity 6 Position in Array 594
pixel intensity
12
16
20
0
200
400
600
800
1000
frames

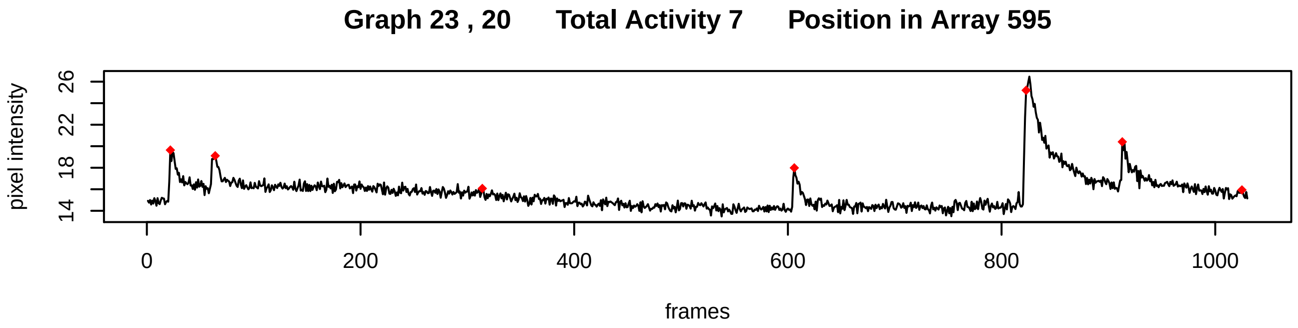
Graph 23 , 20 Total Activity 7 Position in Array 595
pixel intensity
14
18
22
26
0
200
400
600
800
1000
frames

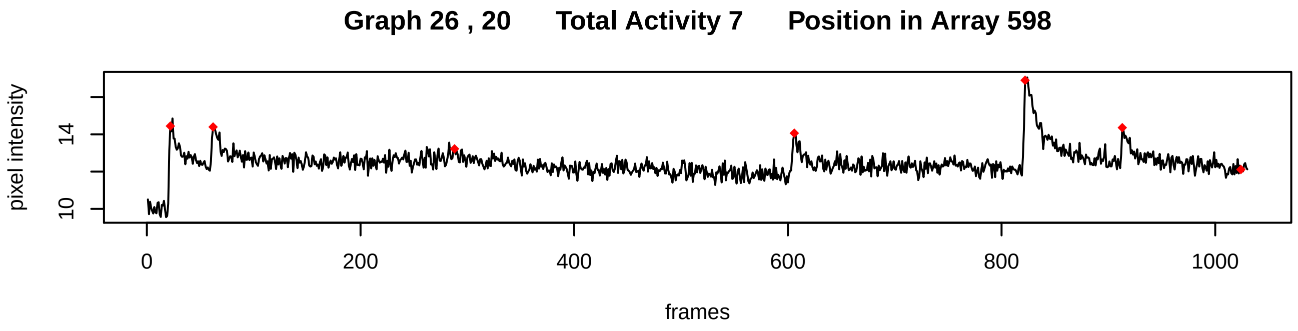
Graph 26 , 20 Total Activity 7 Position in Array 598
pixel intensity
10
14
0
200
400
600
800
1000
frames

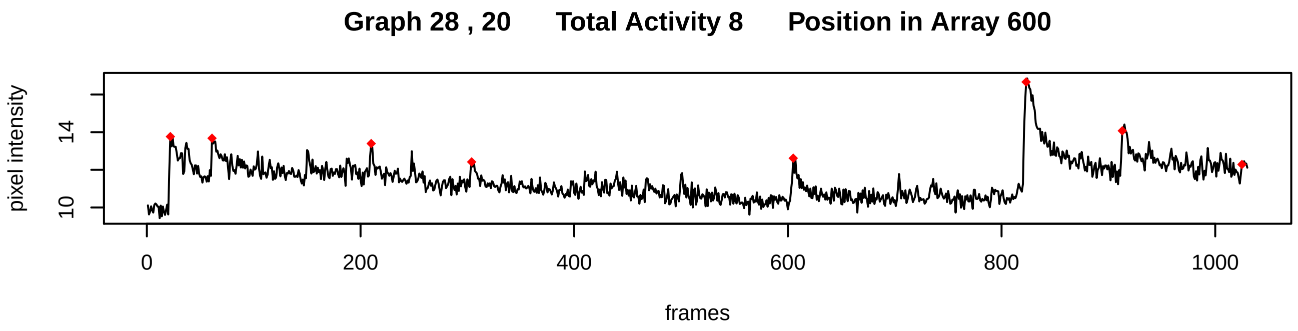
Graph 28 , 20 Total Activity 8 Position in Array 600
pixel intensity
10
14
0
200
400
600
800
1000
frames

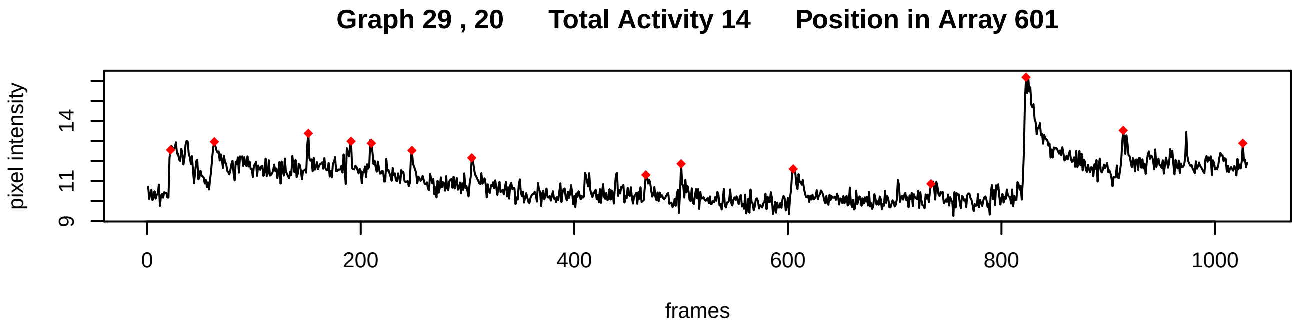
Graph 29 , 20 Total Activity 14 Position in Array 601
pixel intensity
9
11
14
0
200
400
600
800
1000
frames

Graph 31 , 20 Total Activity 9 Position in Array 603

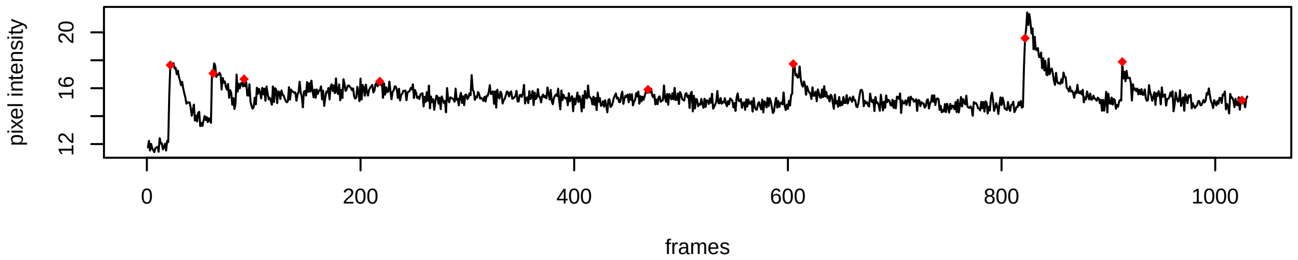

Graph 32 , 20 Total Activity 7 Position in Array 604

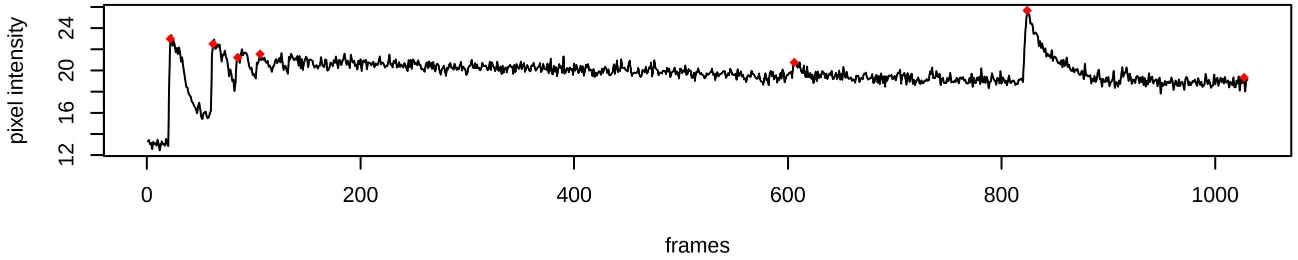

Graph 33 , 20 Total Activity 8 Position in Array 605

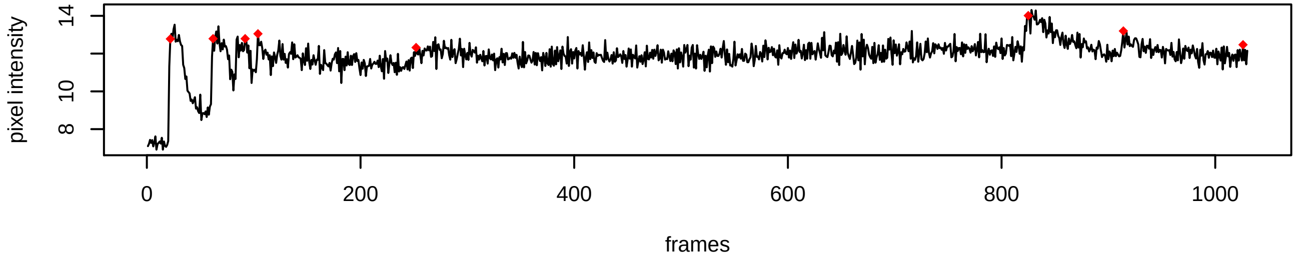

Graph 34 , 20   Total Activity 5   Position in Array 606

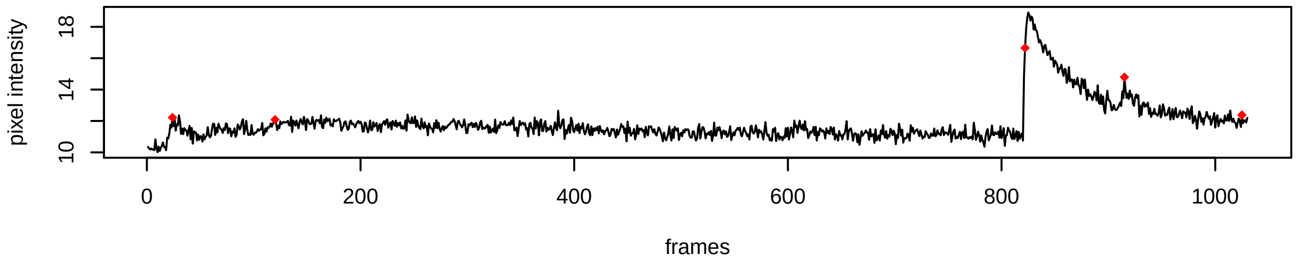

Graph 35 , 20   Total Activity 5   Position in Array 607

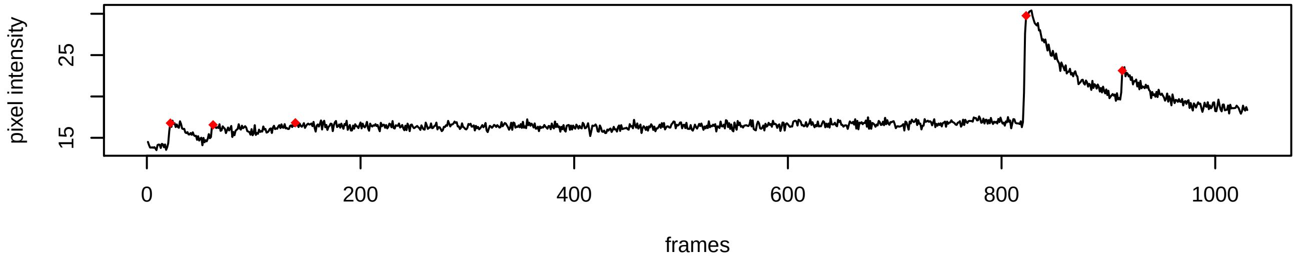

Graph 36 , 20   Total Activity 8   Position in Array 608

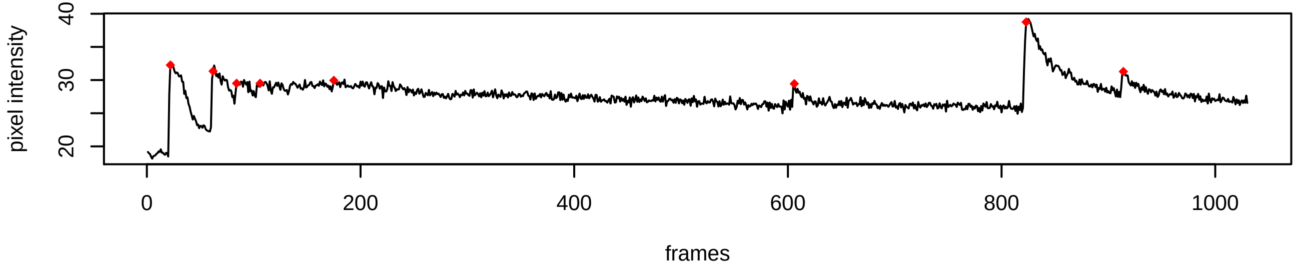

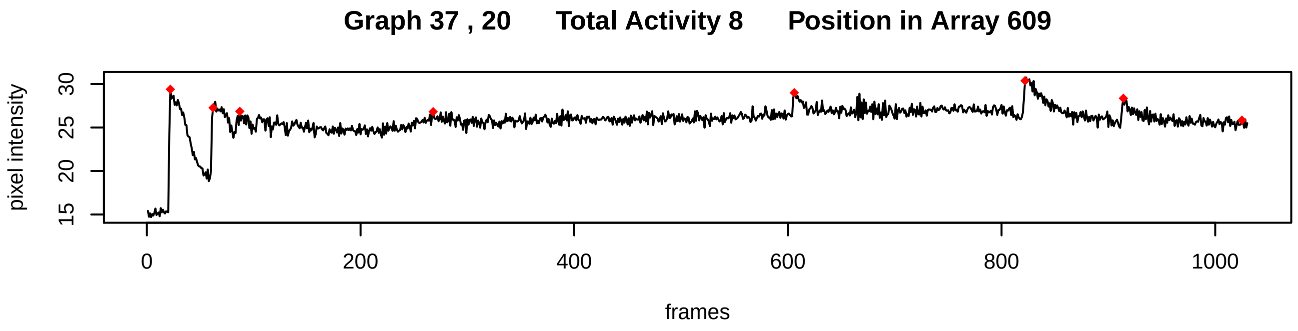
Graph 37 , 20 Total Activity 8 Position in Array 609
pixel intensity
frames
15
20
25
30
0
200
400
600
800
1000

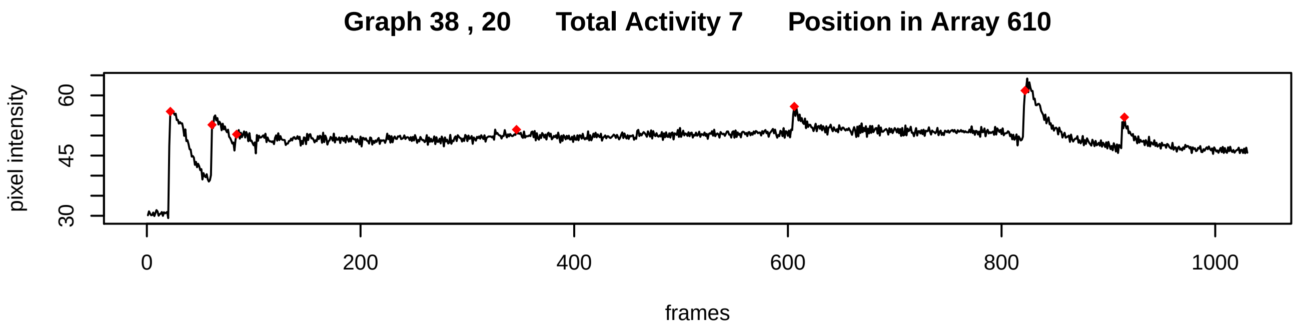
Graph 38 , 20 Total Activity 7 Position in Array 610
pixel intensity
frames
30
45
60
0
200
400
600
800
1000

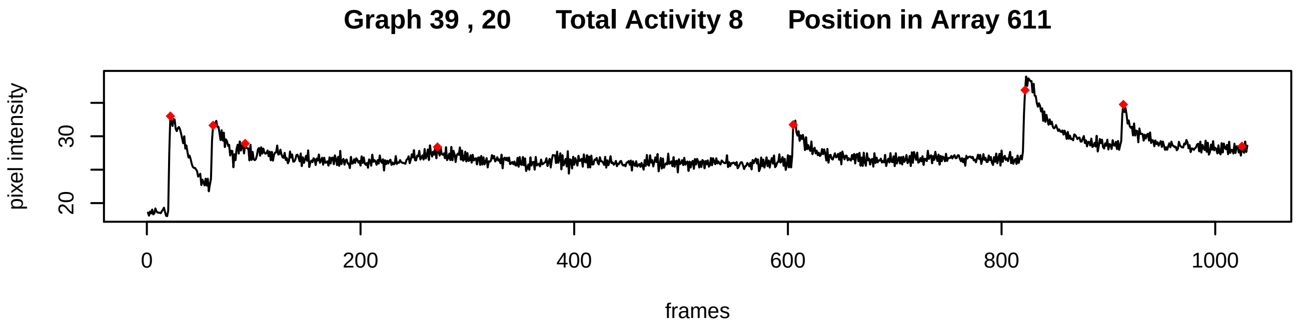
Graph 39 , 20 Total Activity 8 Position in Array 611
pixel intensity
frames
20
30
0
200
400
600
800
1000

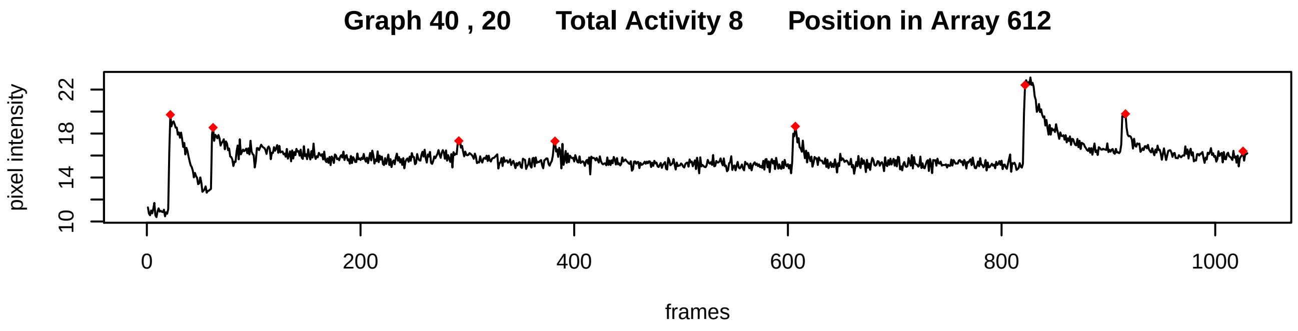
Graph 40 , 20 Total Activity 8 Position in Array 612
pixel intensity
10
14
18
22
0
200
400
600
800
1000
frames

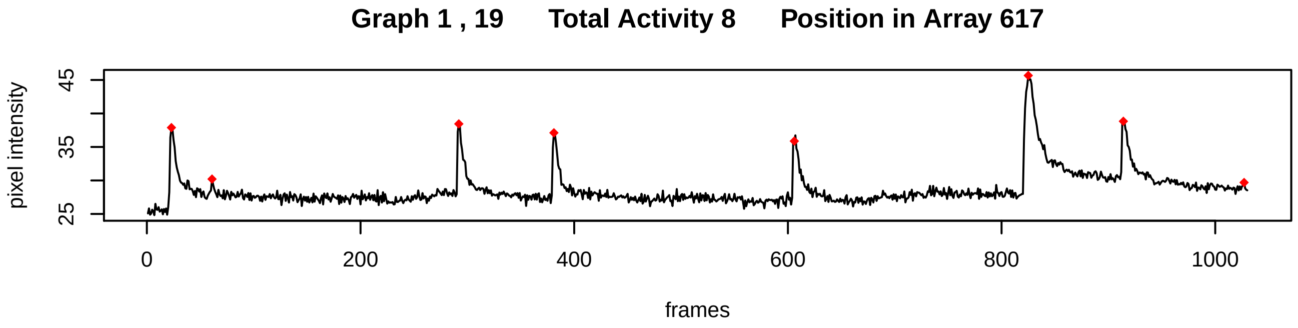
Graph 1 , 19 Total Activity 8 Position in Array 617
pixel intensity
25
35
45
0
200
400
600
800
1000
frames

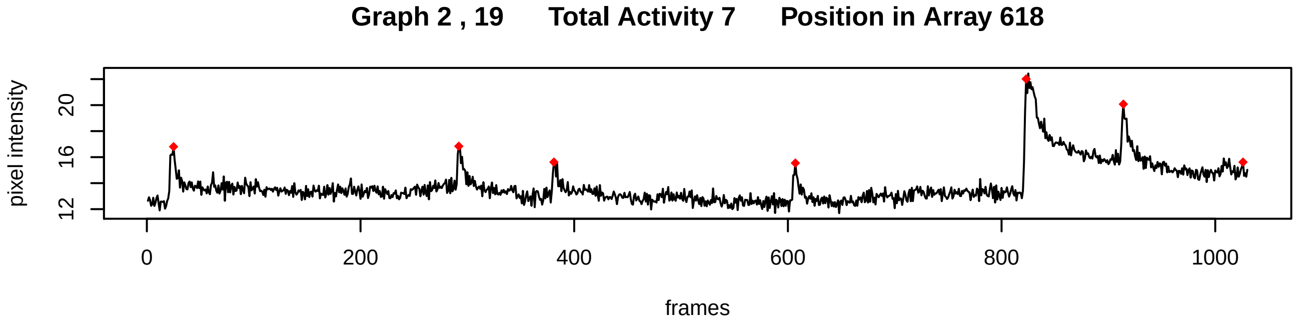
Graph 2 , 19 Total Activity 7 Position in Array 618
pixel intensity
12
16
20
0
200
400
600
800
1000
frames

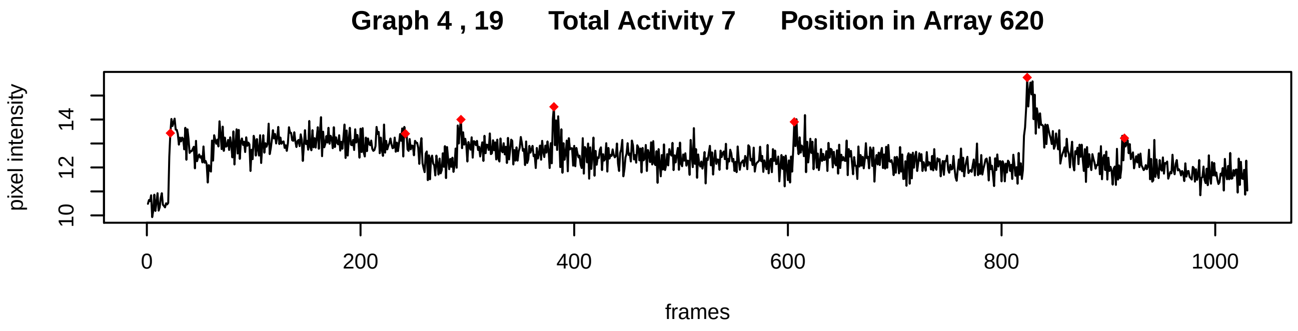
Graph 4 , 19 Total Activity 7 Position in Array 620
pixel intensity
10
12
14
0
200
400
600
800
1000
frames

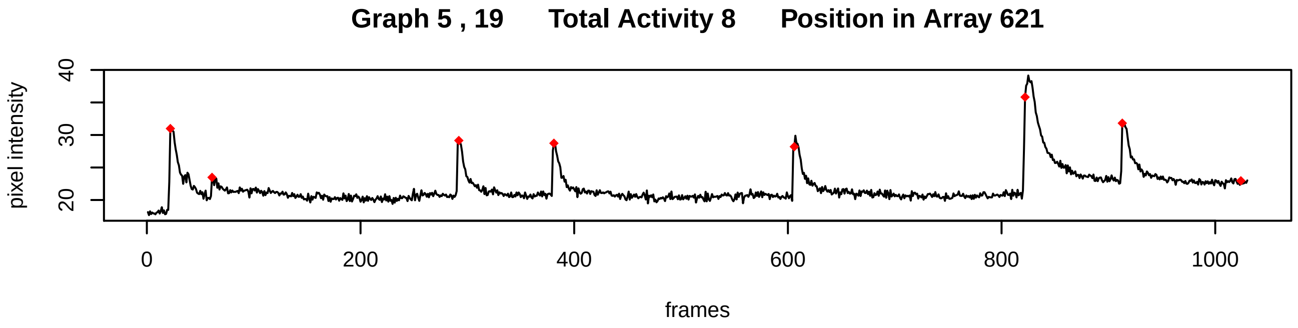
Graph 5 , 19 Total Activity 8 Position in Array 621
pixel intensity
20
30
40
0
200
400
600
800
1000
frames

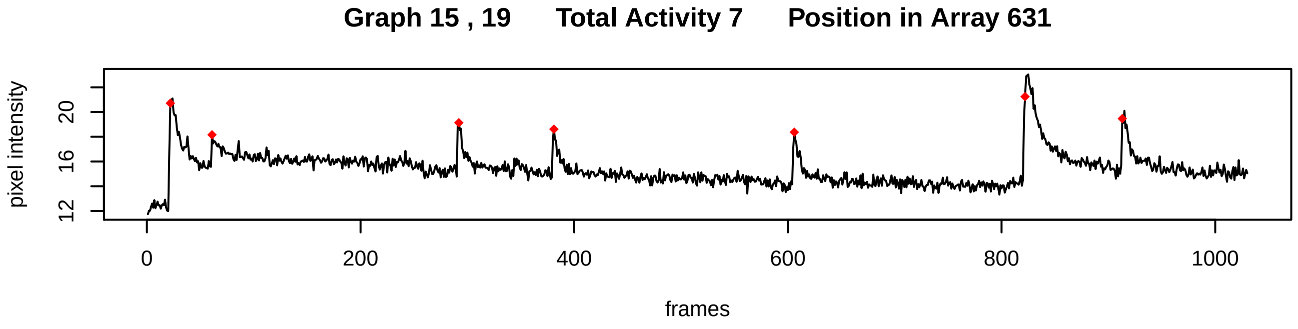
Graph 15 , 19 Total Activity 7 Position in Array 631
pixel intensity
12
16
20
0
200
400
600
800
1000
frames

Graph 16 , 19   Total Activity 7   Position in Array 632

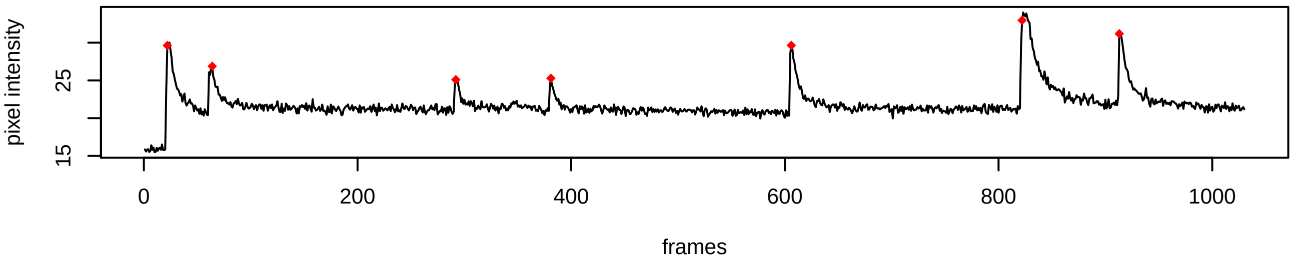

Graph 17 , 19   Total Activity 7   Position in Array 633

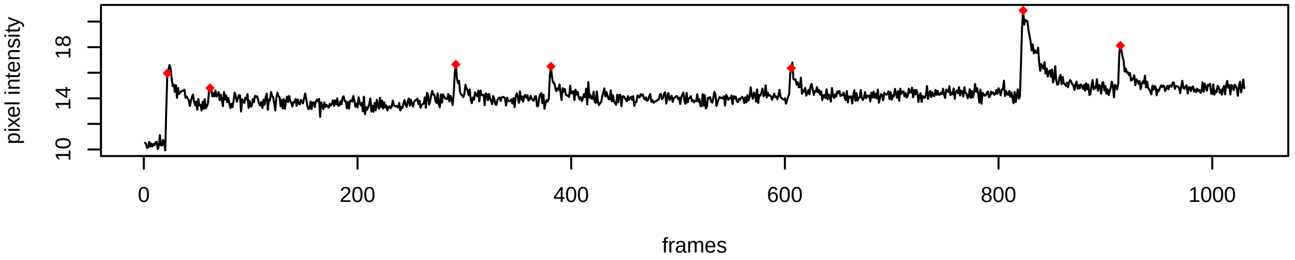

Graph 21 , 19   Total Activity 6   Position in Array 637

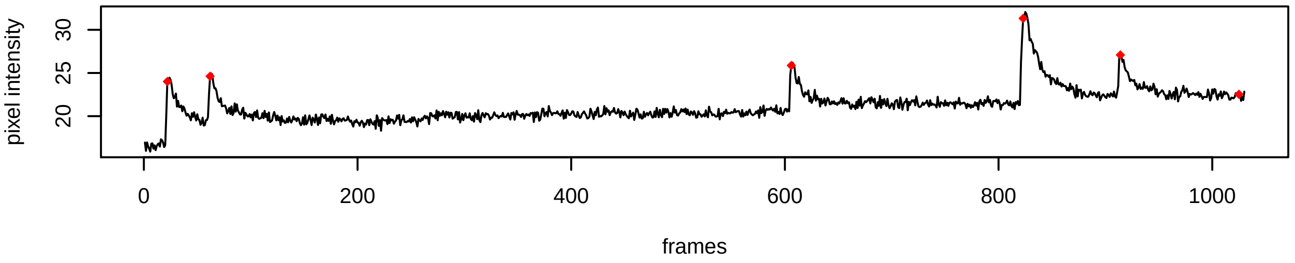

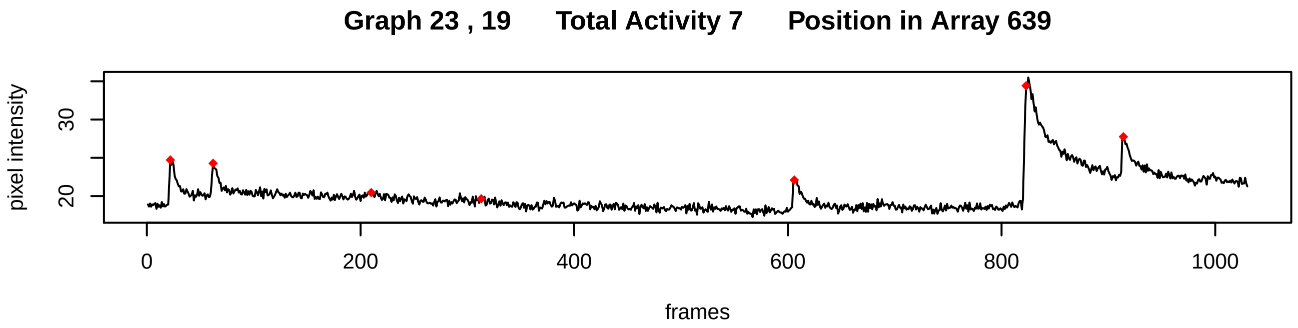
Graph 23 , 19 Total Activity 7 Position in Array 639
pixel intensity
20
30
0
200
400
600
800
1000
frames

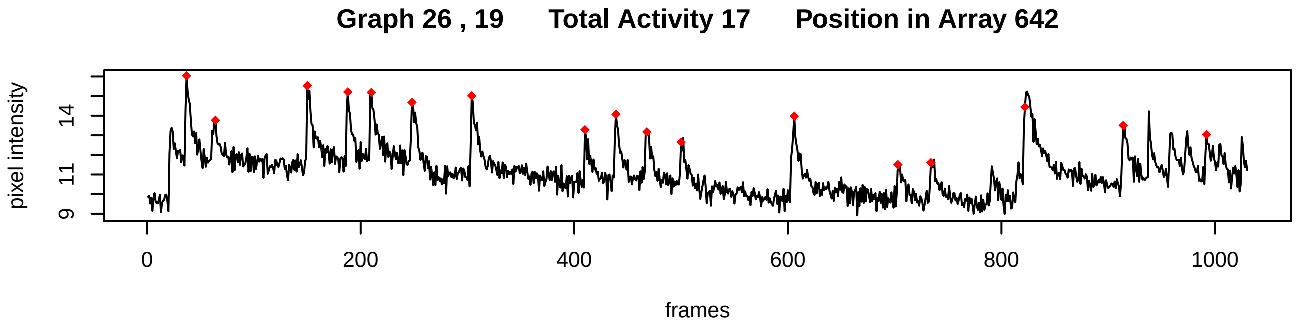
Graph 26 , 19 Total Activity 17 Position in Array 642
pixel intensity
9
11
14
0
200
400
600
800
1000
frames

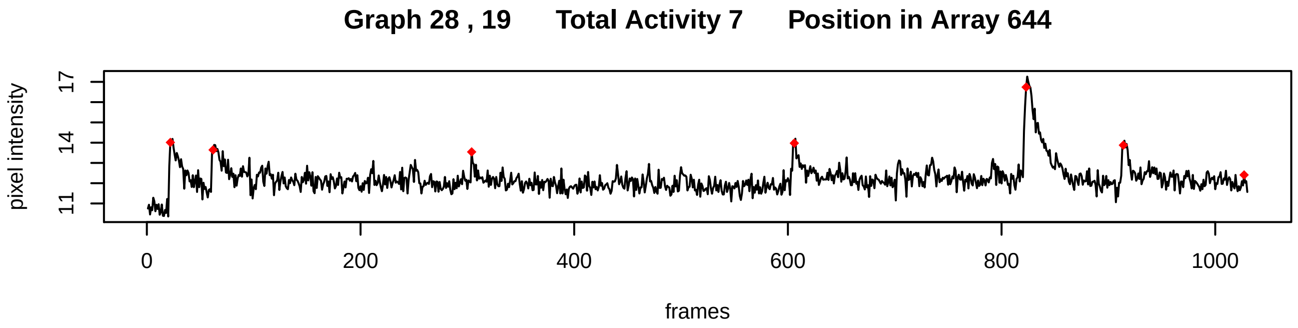
Graph 28 , 19 Total Activity 7 Position in Array 644
pixel intensity
11
14
17
0
200
400
600
800
1000
frames

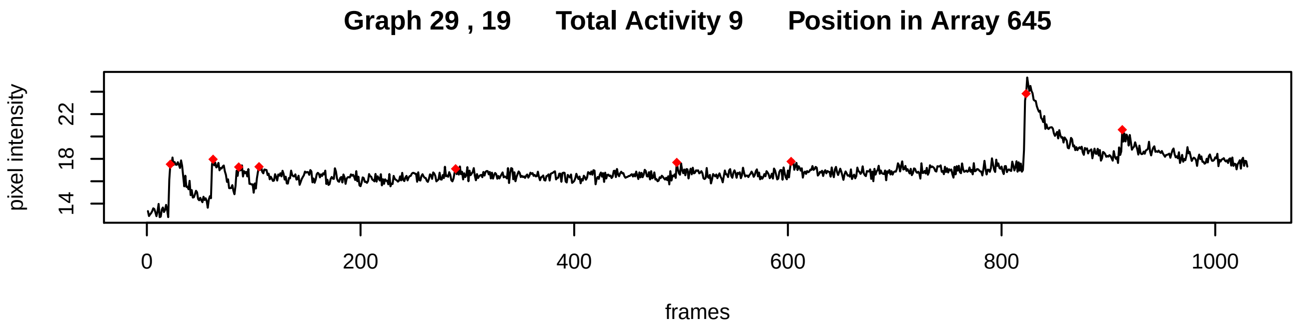
Graph 29 , 19 Total Activity 9 Position in Array 645
pixel intensity
14
18
22
0
200
400
600
800
1000
frames

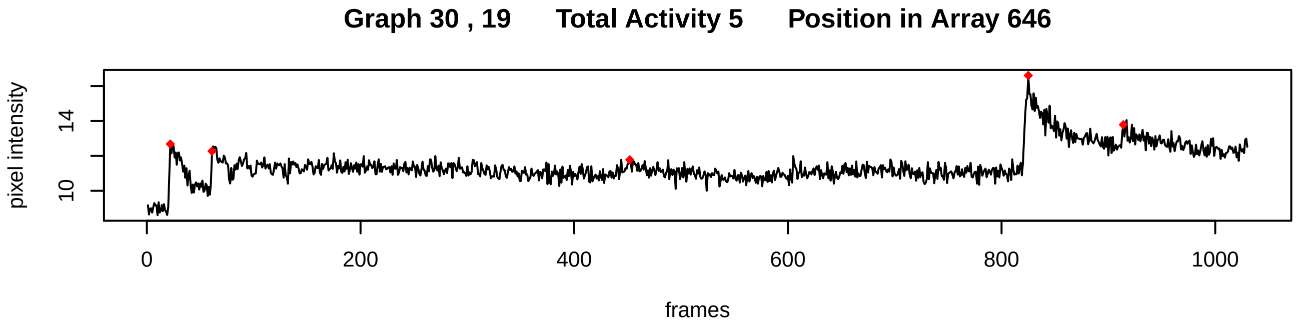
Graph 30 , 19 Total Activity 5 Position in Array 646
pixel intensity
10
14
0
200
400
600
800
1000
frames

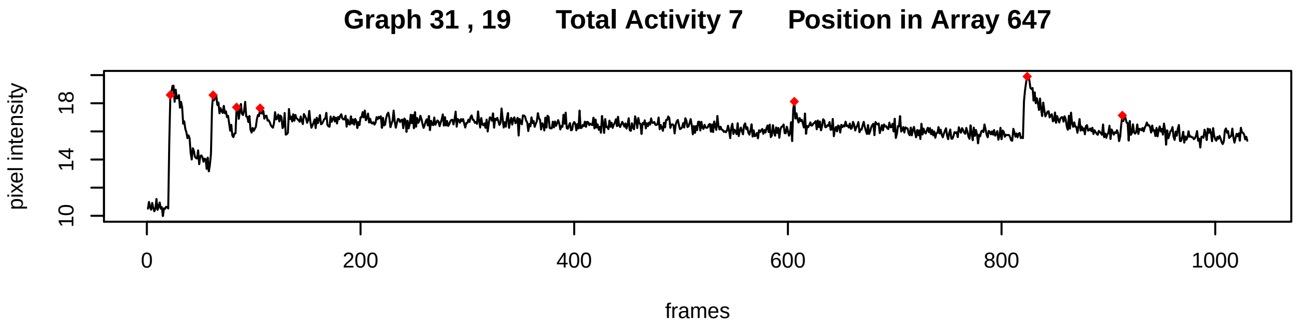
Graph 31 , 19 Total Activity 7 Position in Array 647
pixel intensity
10
14
18
0
200
400
600
800
1000
frames

Graph 34 , 19 Total Activity 8 Position in Array 650

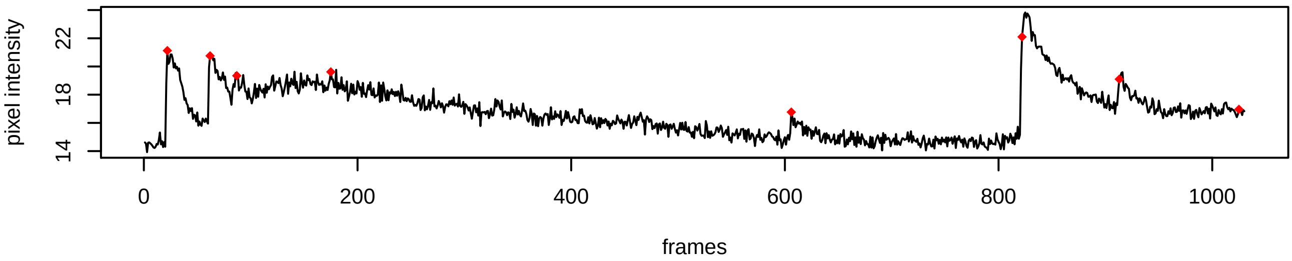

Graph 35 , 19 Total Activity 7 Position in Array 651

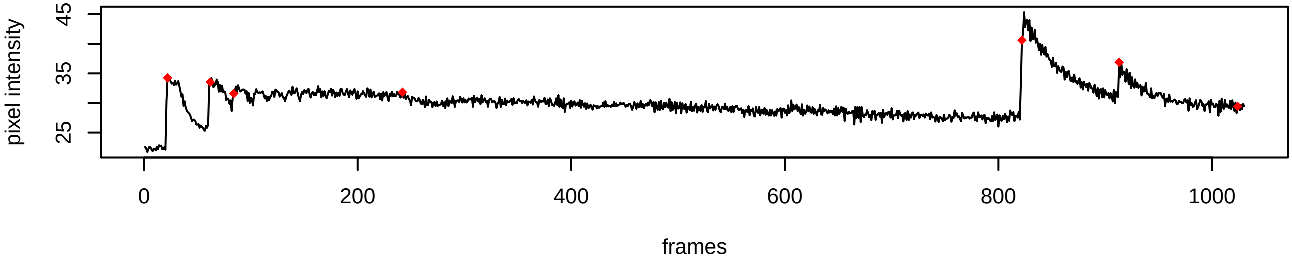

Graph 36 , 19 Total Activity 6 Position in Array 652

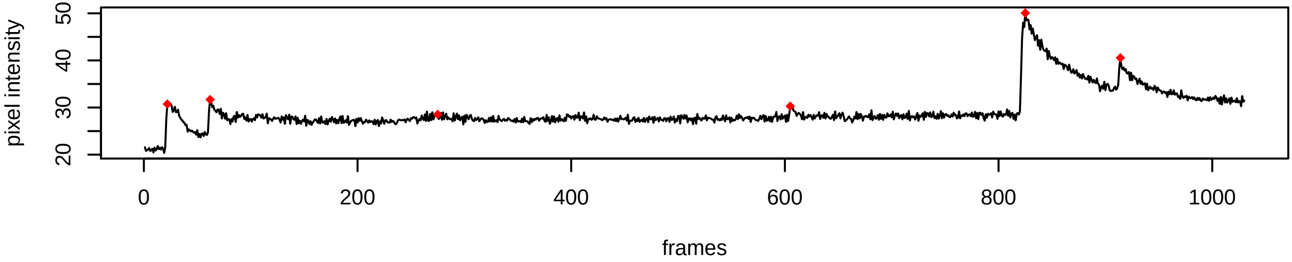

Graph 37 , 19   Total Activity 6   Position in Array 653

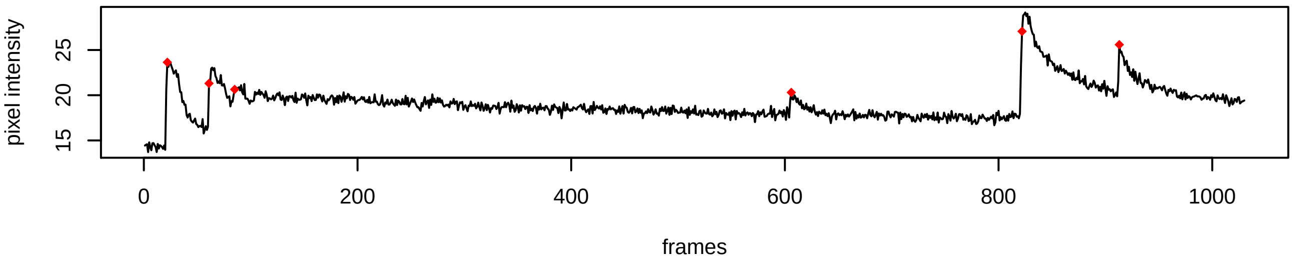

Graph 38 , 19   Total Activity 8   Position in Array 654

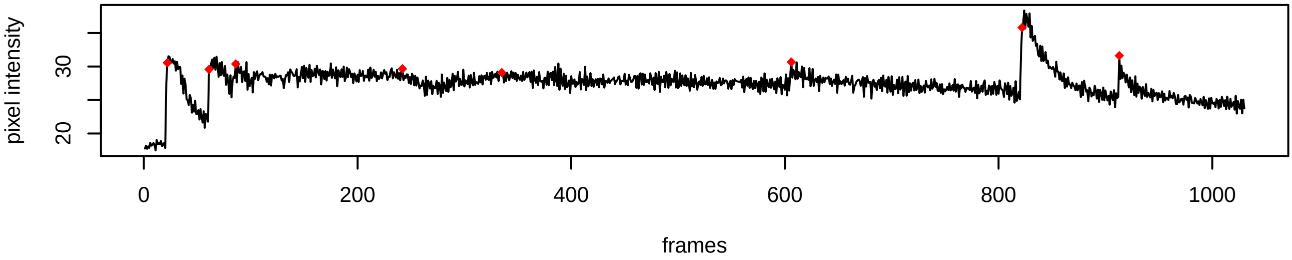

Graph 39 , 19   Total Activity 7   Position in Array 655

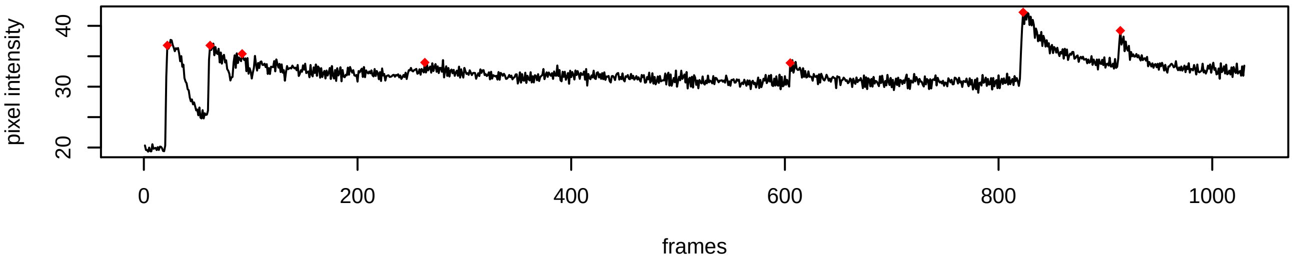

Graph 40 , 19 Total Activity 7 Position in Array 656

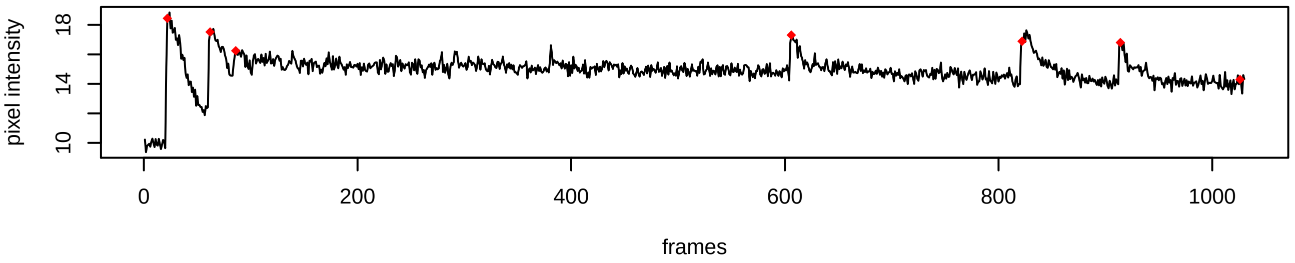

Graph 1 , 18 Total Activity 6 Position in Array 661

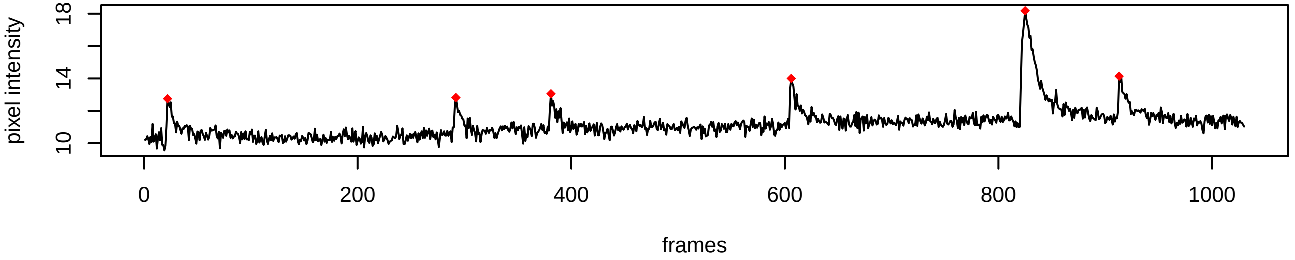

Graph 2 , 18 Total Activity 7 Position in Array 662

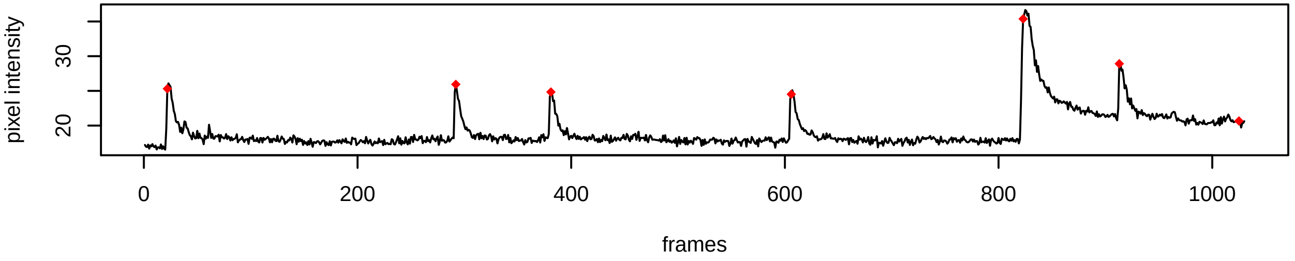

Graph 4 , 18 Total Activity 6 Position in Array 664

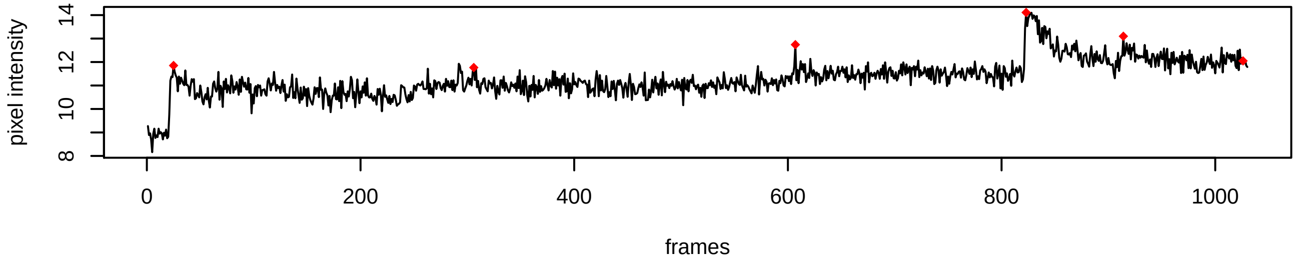

Graph 5 , 18 Total Activity 8 Position in Array 665

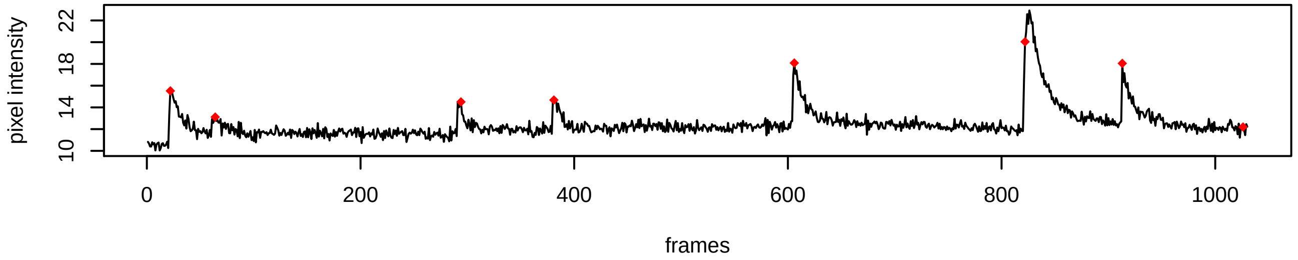

Graph 6 , 18 Total Activity 8 Position in Array 666

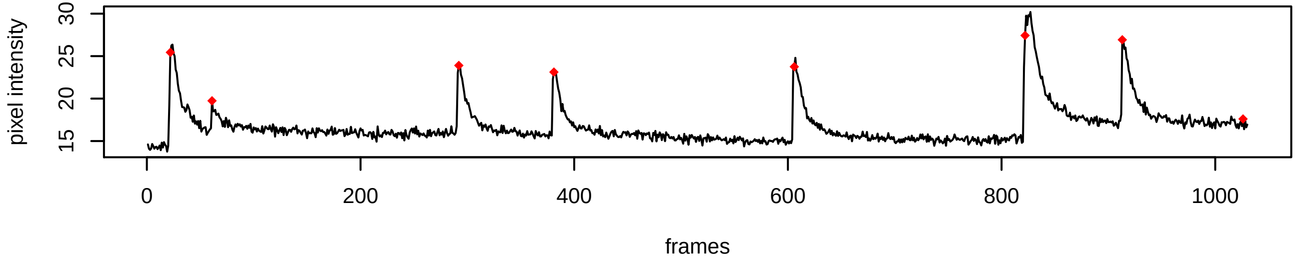

Graph 8 , 18   Total Activity 9   Position in Array 668

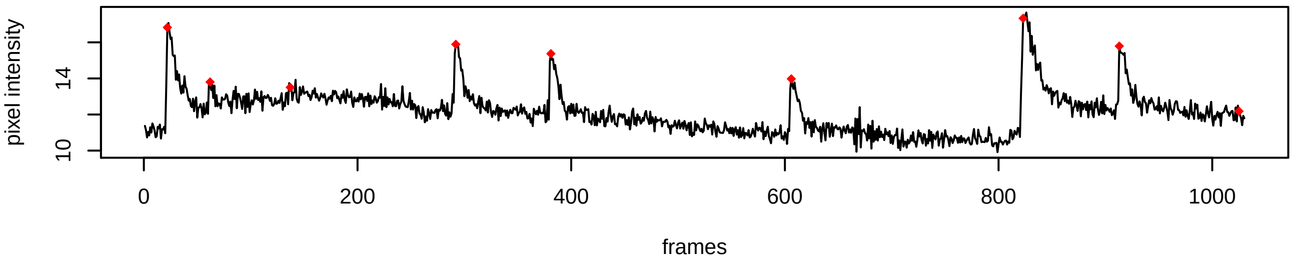

Graph 13 , 18   Total Activity 13   Position in Array 673

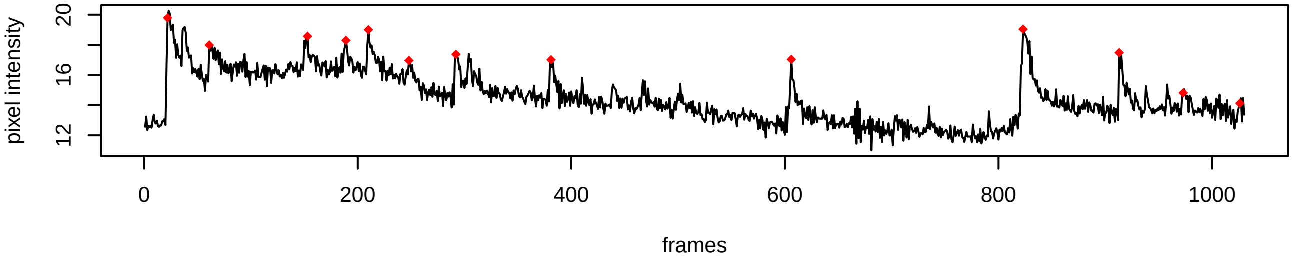

Graph 14 , 18   Total Activity 8   Position in Array 674

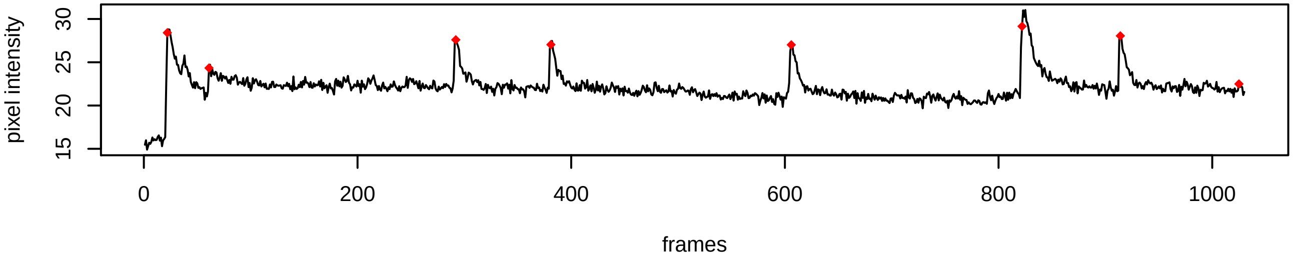

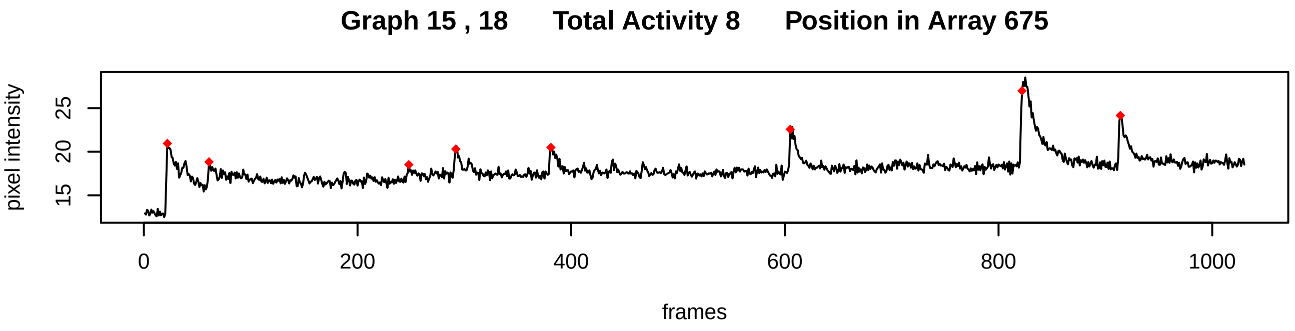
Graph 15 , 18 Total Activity 8 Position in Array 675
pixel intensity
15
20
25
0
200
400
600
800
1000
frames

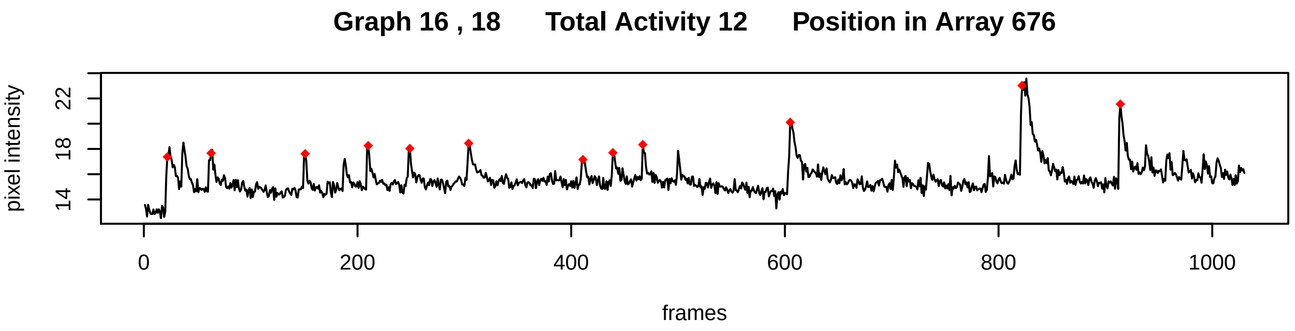
Graph 16 , 18 Total Activity 12 Position in Array 676
pixel intensity
14
18
22
0
200
400
600
800
1000
frames

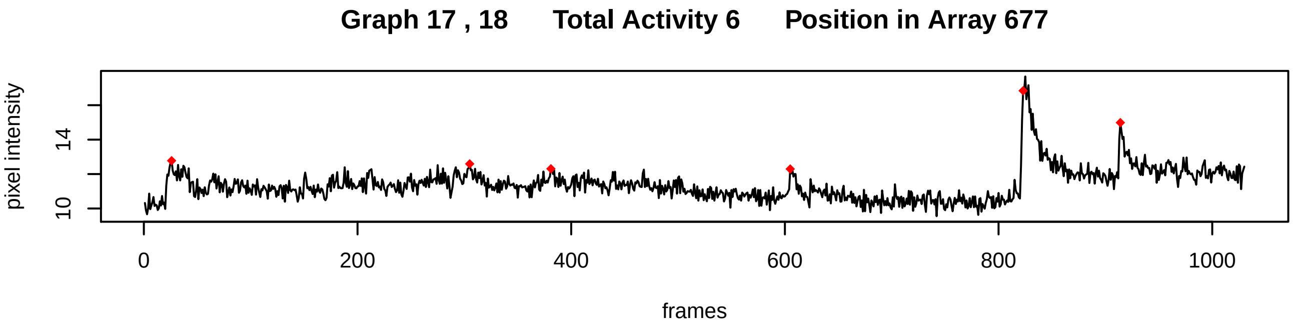
Graph 17 , 18 Total Activity 6 Position in Array 677
pixel intensity
10
14
0
200
400
600
800
1000
frames

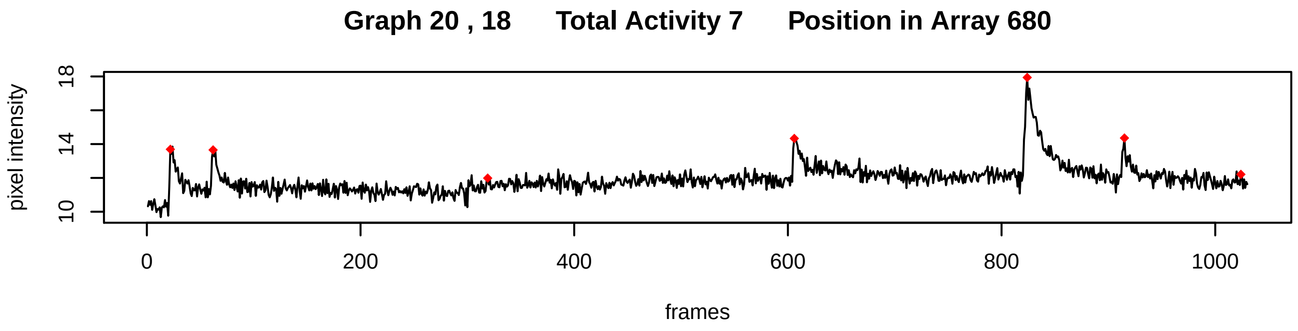
Graph 20 , 18 Total Activity 7 Position in Array 680
pixel intensity
10
14
18
0
200
400
600
800
1000
frames

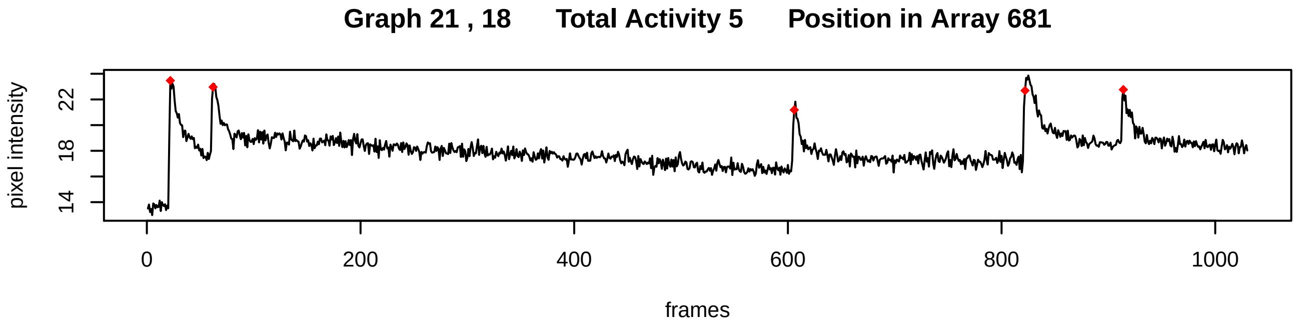
Graph 21 , 18 Total Activity 5 Position in Array 681
pixel intensity
14
18
22
0
200
400
600
800
1000
frames

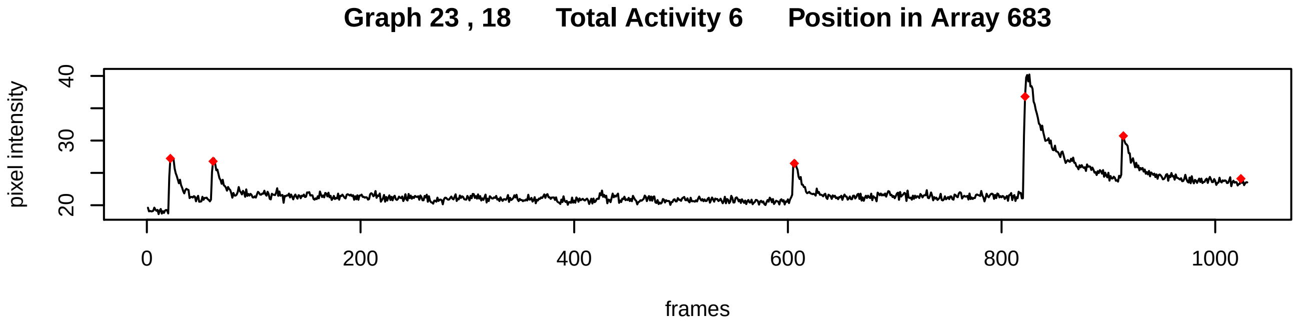
Graph 23 , 18 Total Activity 6 Position in Array 683
pixel intensity
20
30
40
0
200
400
600
800
1000
frames

Graph 24 , 18   Total Activity 15   Position in Array 684

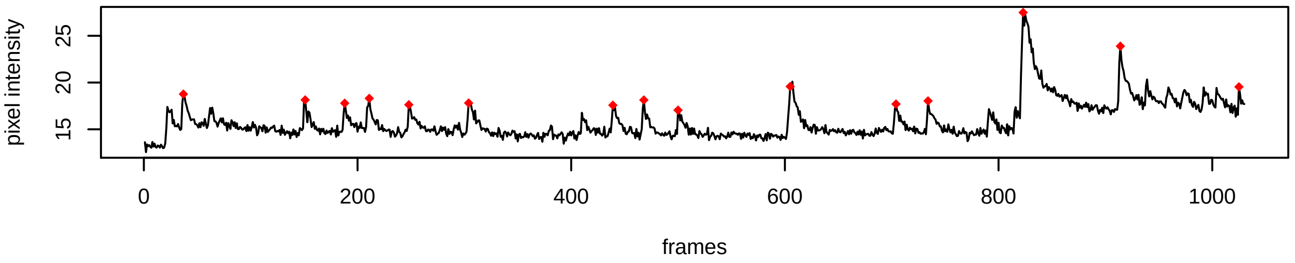

Graph 26 , 18   Total Activity 15   Position in Array 686

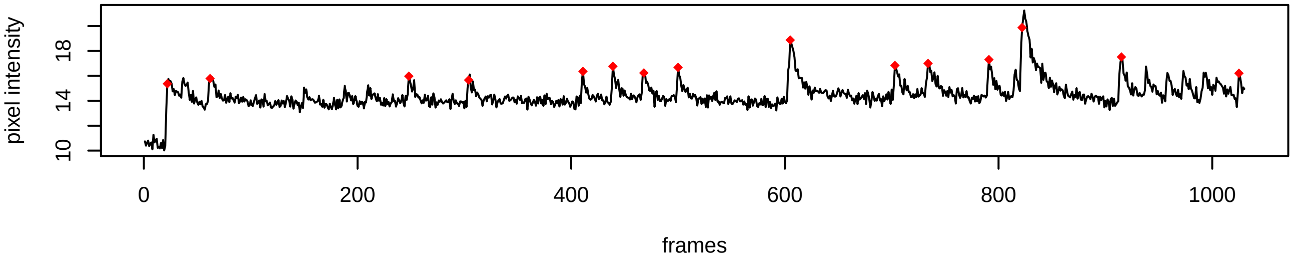

Graph 30 , 18   Total Activity 8   Position in Array 690

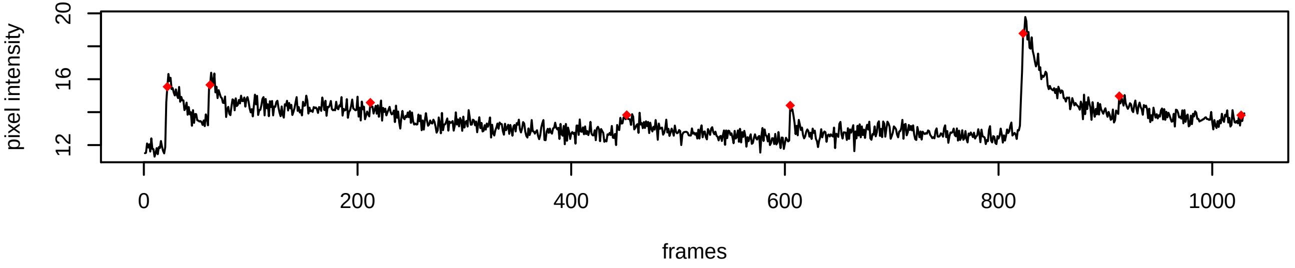

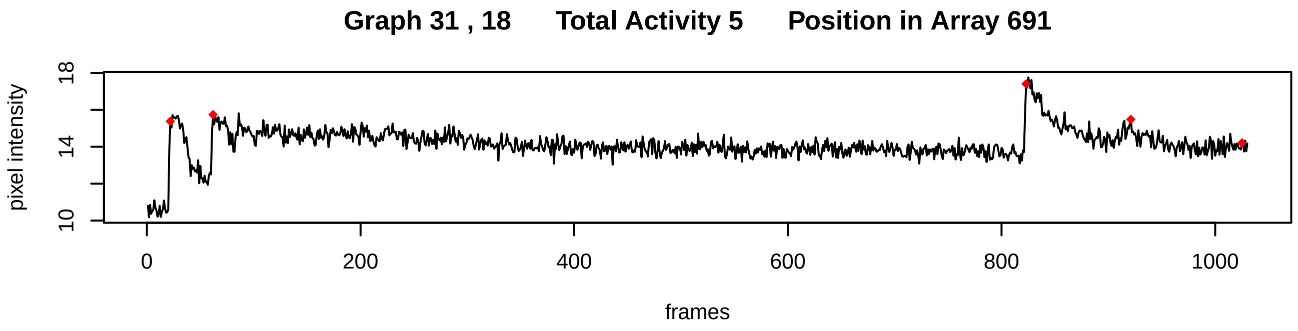
Graph 31 , 18 Total Activity 5 Position in Array 691
pixel intensity
10
14
18
0
200
400
600
800
1000
frames

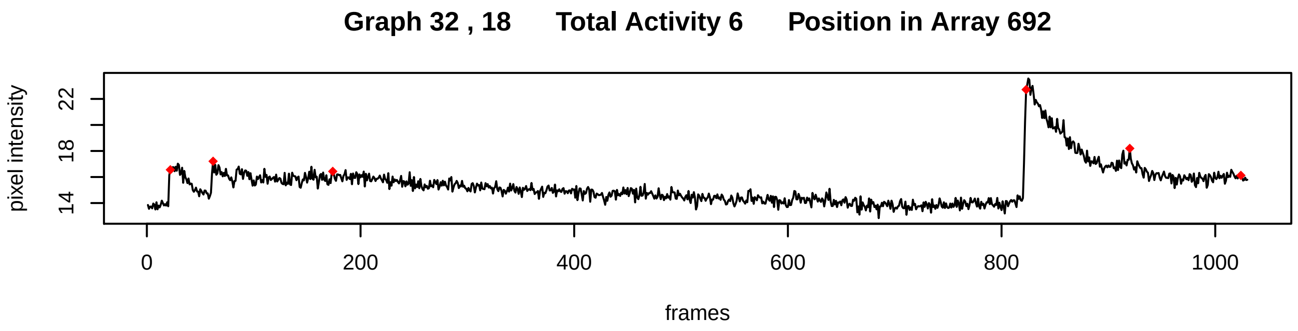
Graph 32 , 18 Total Activity 6 Position in Array 692
pixel intensity
14
18
22
0
200
400
600
800
1000
frames

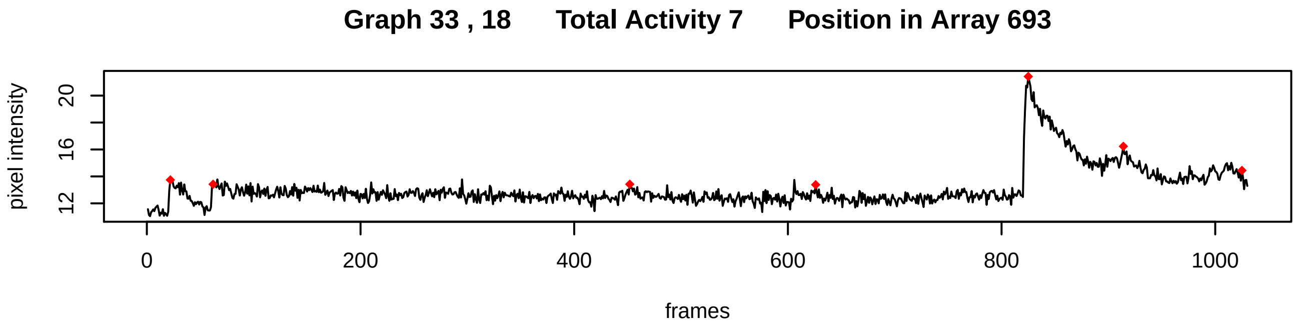
Graph 33 , 18 Total Activity 7 Position in Array 693
pixel intensity
12
16
20
0
200
400
600
800
1000
frames

Graph 34 , 18 Total Activity 7 Position in Array 694

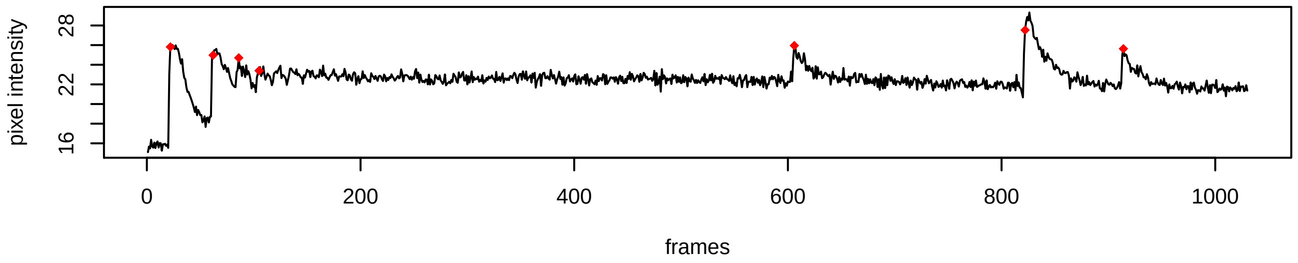

Graph 35 , 18 Total Activity 7 Position in Array 695

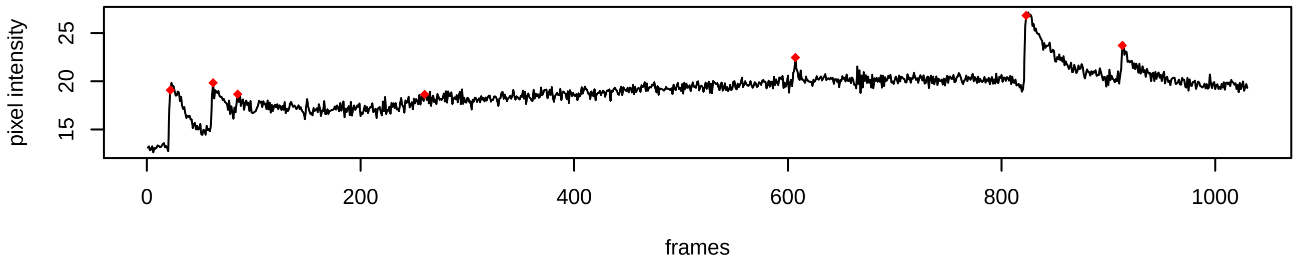

Graph 36 , 18 Total Activity 6 Position in Array 696

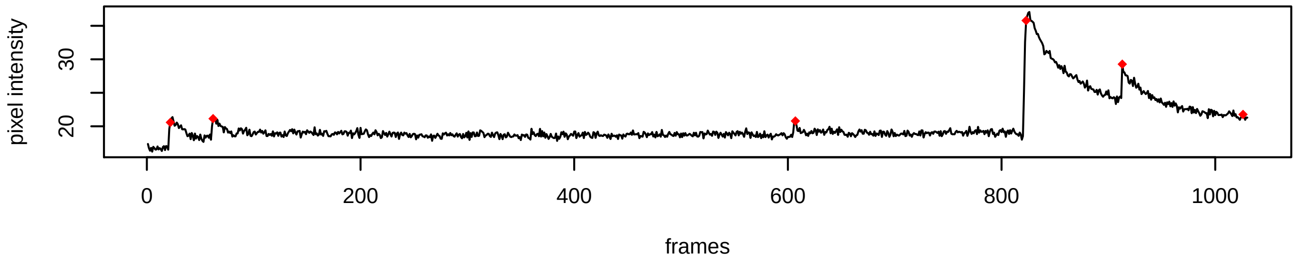

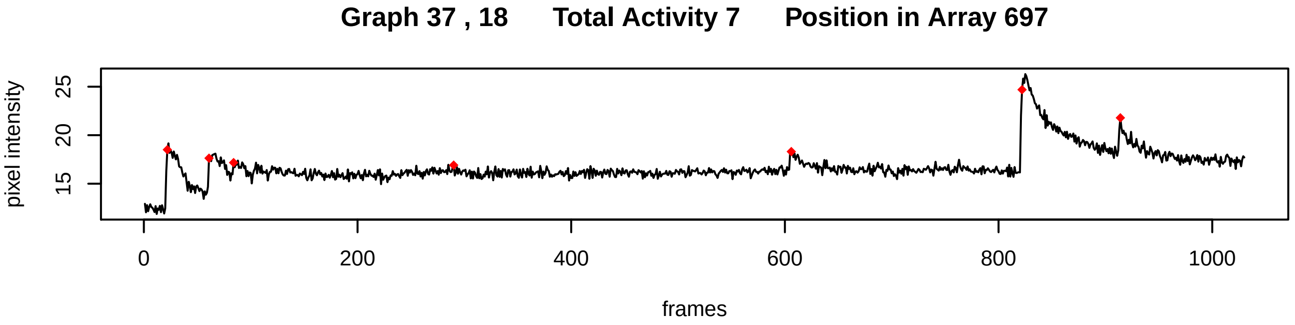

Graph 37 , 18 Total Activity 7 Position in Array 697
pixel intensity
15
20
25
0
200
400
600
800
1000
frames

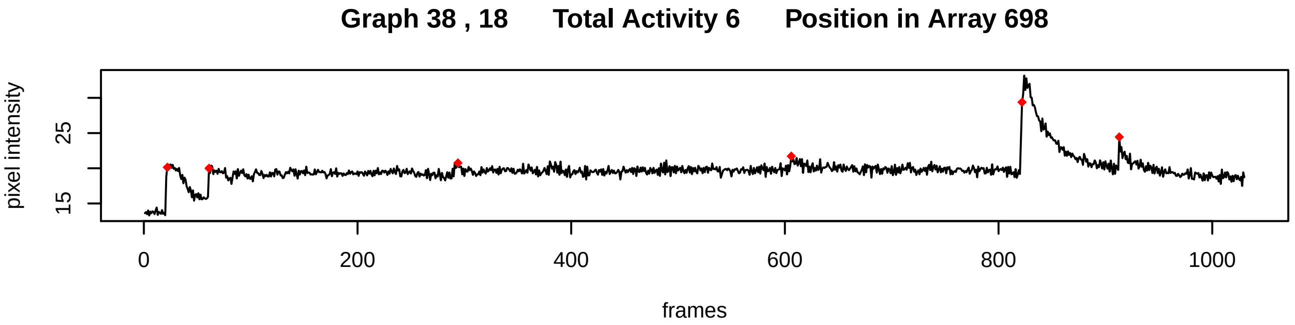

Graph 38 , 18 Total Activity 6 Position in Array 698
pixel intensity
15
25
0
200
400
600
800
1000
frames

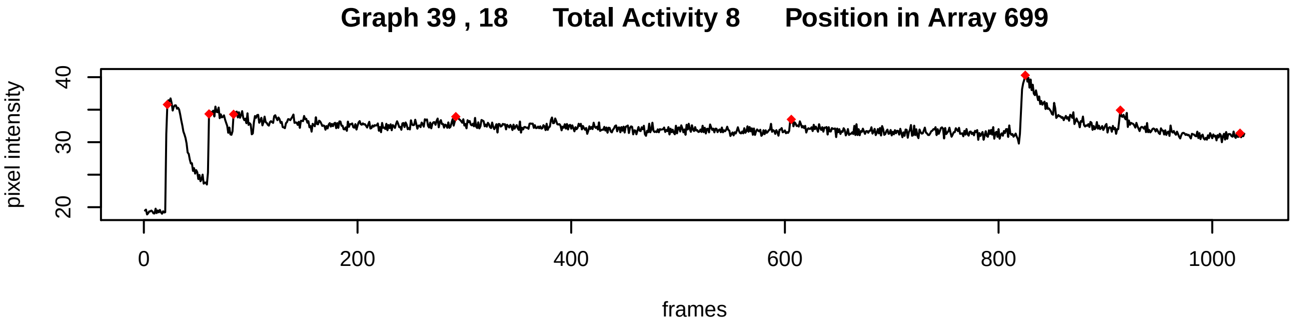

Graph 39 , 18 Total Activity 8 Position in Array 699
pixel intensity
20
30
40
0
200
400
600
800
1000
frames

Graph 40 , 18   Total Activity 8   Position in Array 700

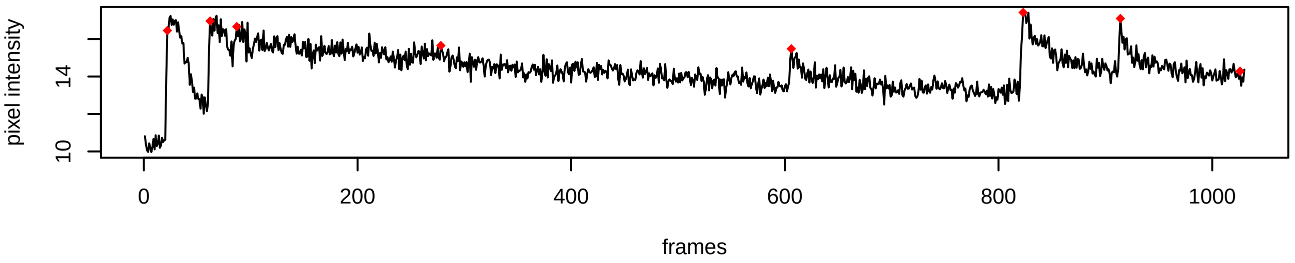

Graph 2 , 17   Total Activity 6   Position in Array 706

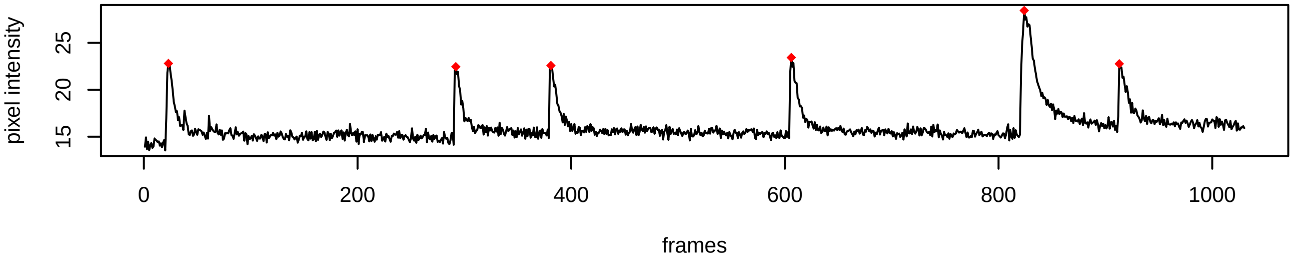

Graph 3 , 17   Total Activity 10   Position in Array 707

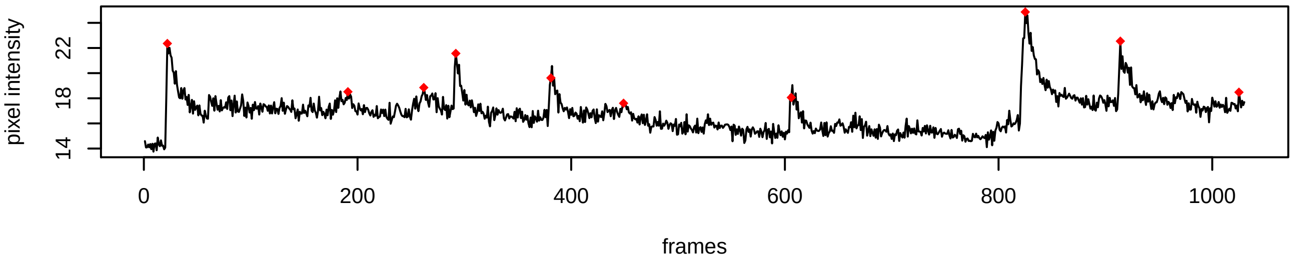

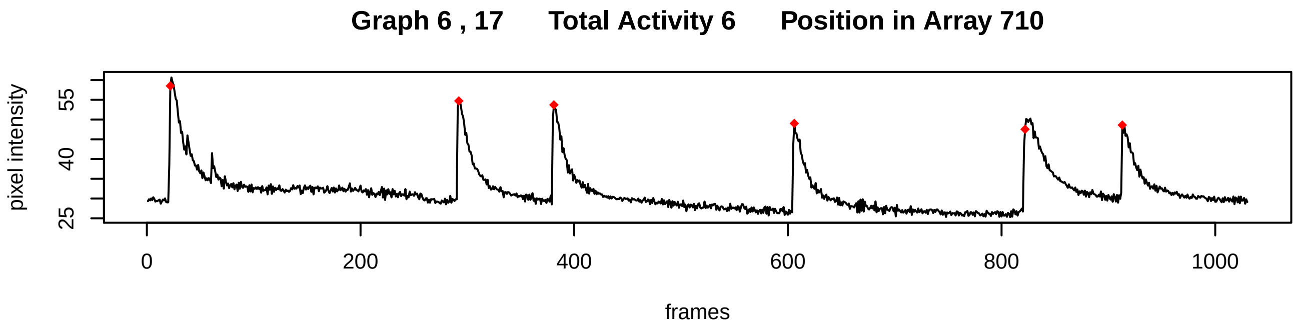
Graph 6 , 17 Total Activity 6 Position in Array 710
pixel intensity
25
40
55
0
200
400
600
800
1000
frames

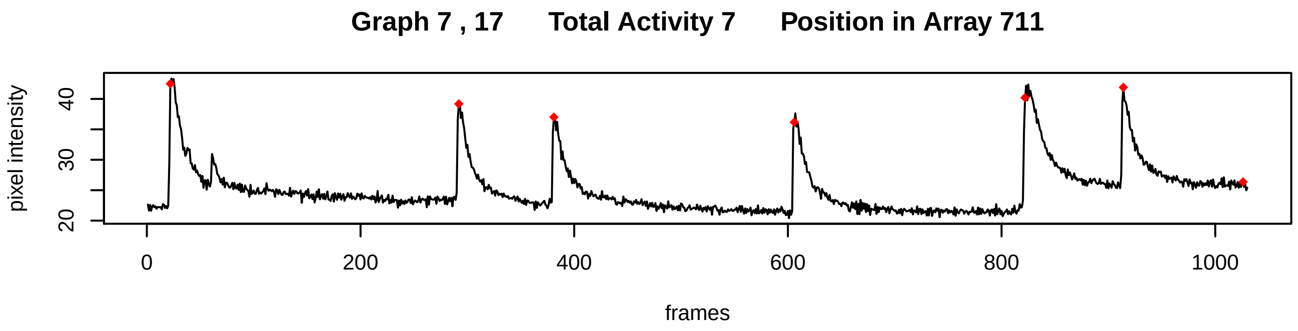
Graph 7 , 17 Total Activity 7 Position in Array 711
pixel intensity
20
30
40
0
200
400
600
800
1000
frames

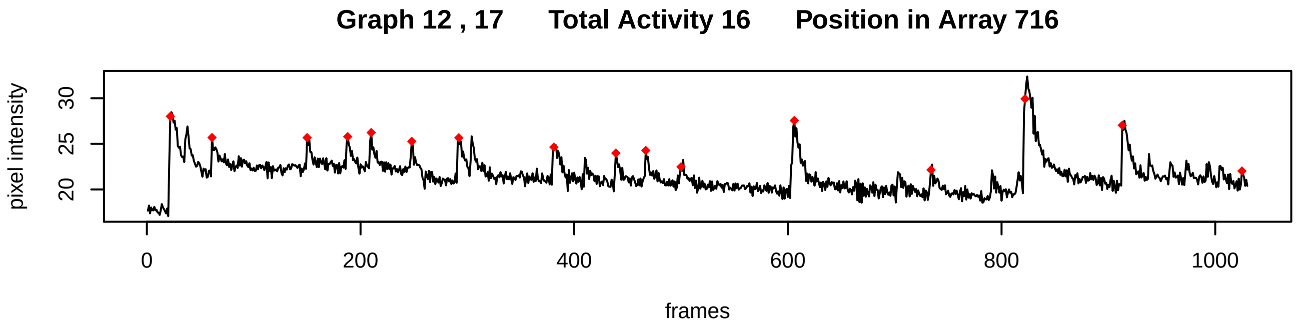
Graph 12 , 17 Total Activity 16 Position in Array 716
pixel intensity
20
25
30
0
200
400
600
800
1000
frames

Graph 13 , 17   Total Activity 8   Position in Array 717

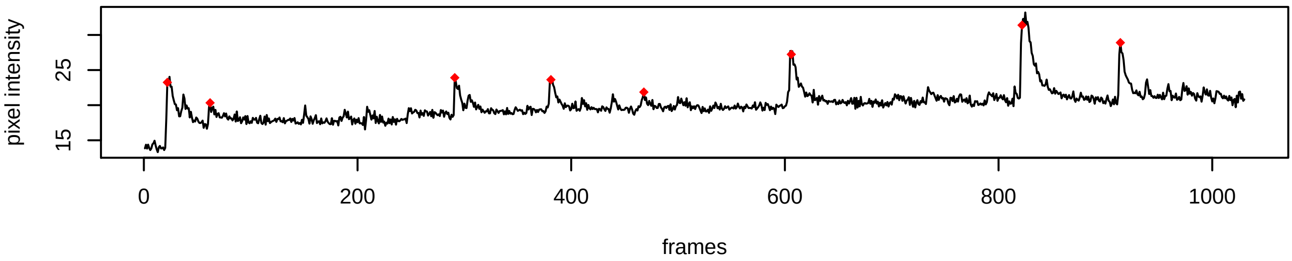

Graph 14 , 17   Total Activity 8   Position in Array 718

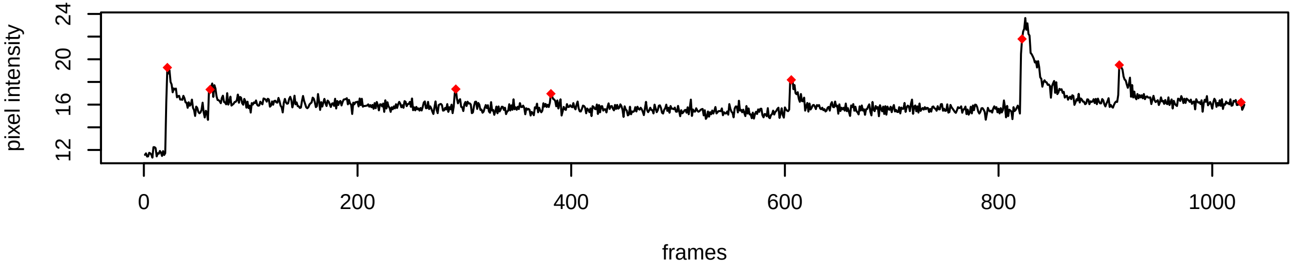

Graph 16 , 17   Total Activity 12   Position in Array 720

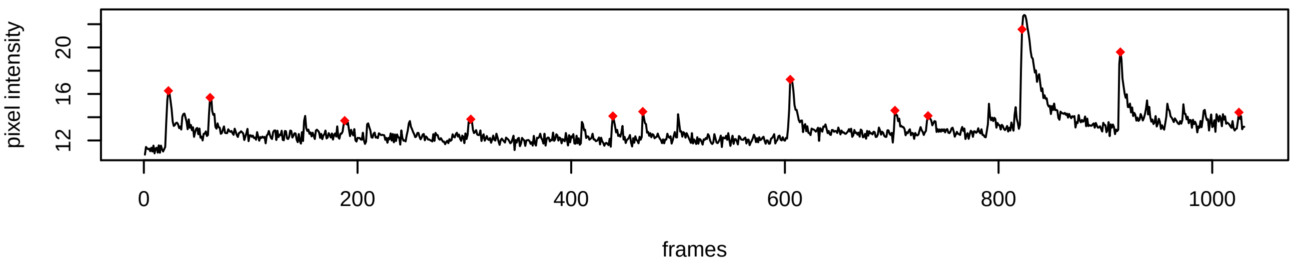

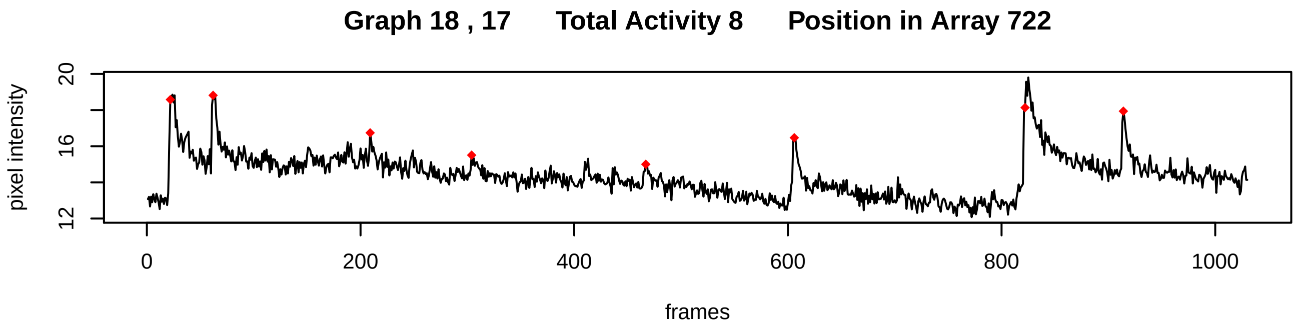
Graph 18 , 17 Total Activity 8 Position in Array 722
pixel intensity
12
16
20
0
200
400
600
800
1000
frames

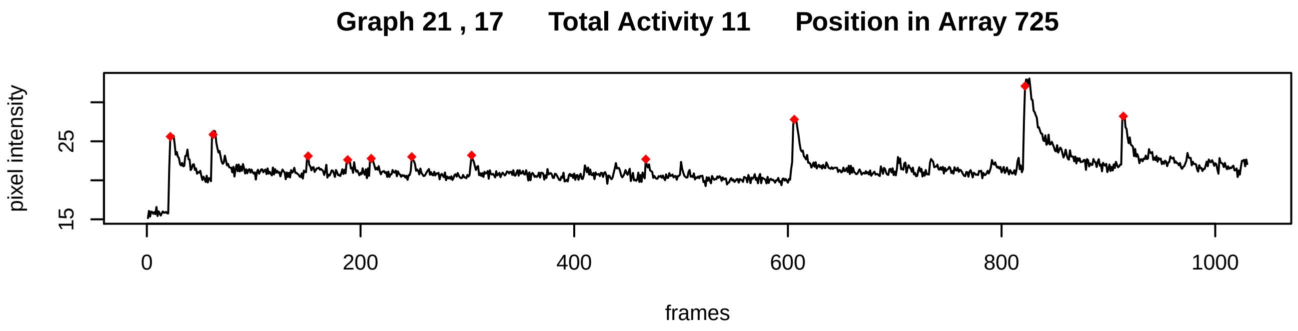
Graph 21 , 17 Total Activity 11 Position in Array 725
pixel intensity
15
25
0
200
400
600
800
1000
frames

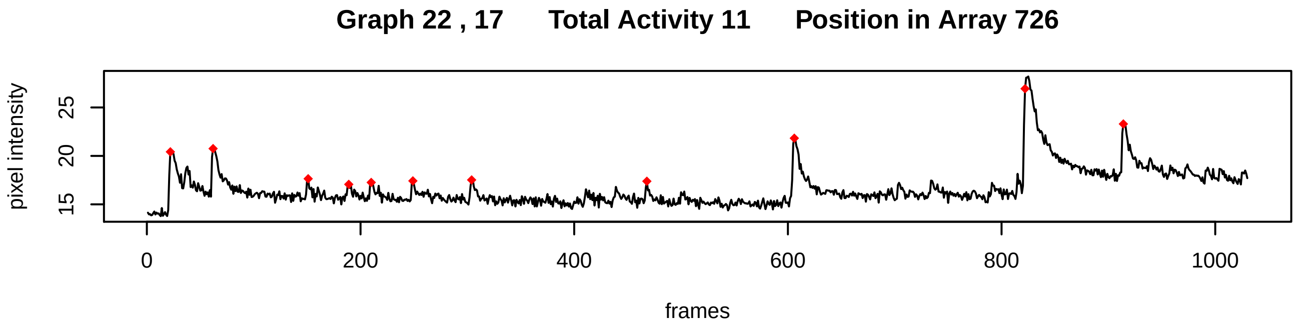
Graph 22 , 17 Total Activity 11 Position in Array 726
pixel intensity
15
20
25
0
200
400
600
800
1000
frames

Graph 23 , 17 Total Activity 7 Position in Array 727

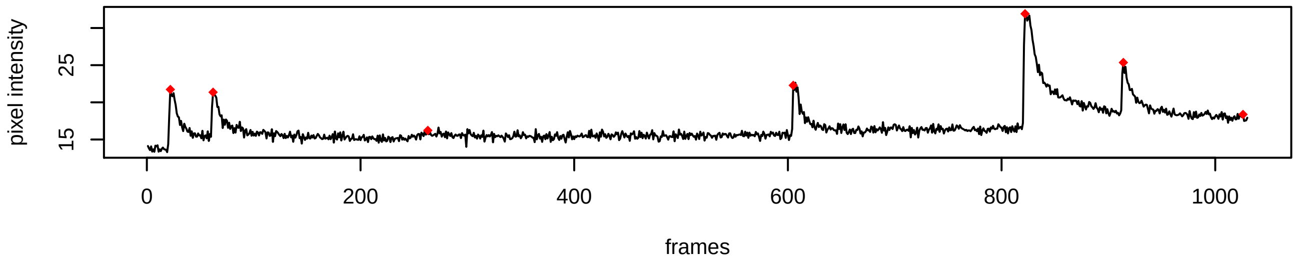

Graph 25 , 17 Total Activity 5 Position in Array 729

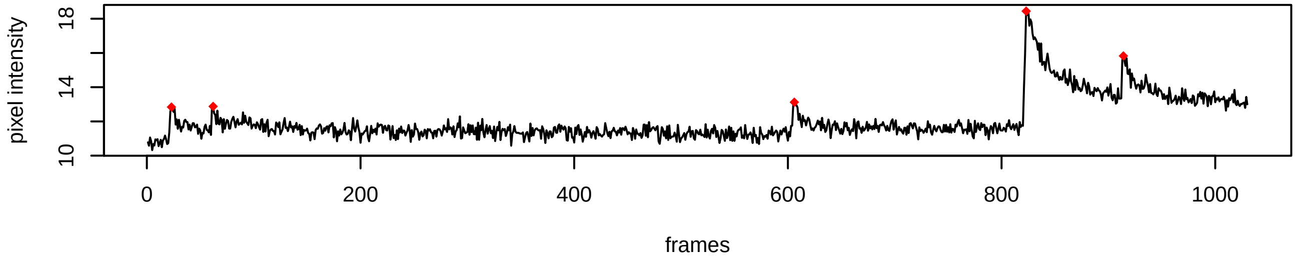

Graph 30 , 17 Total Activity 7 Position in Array 734

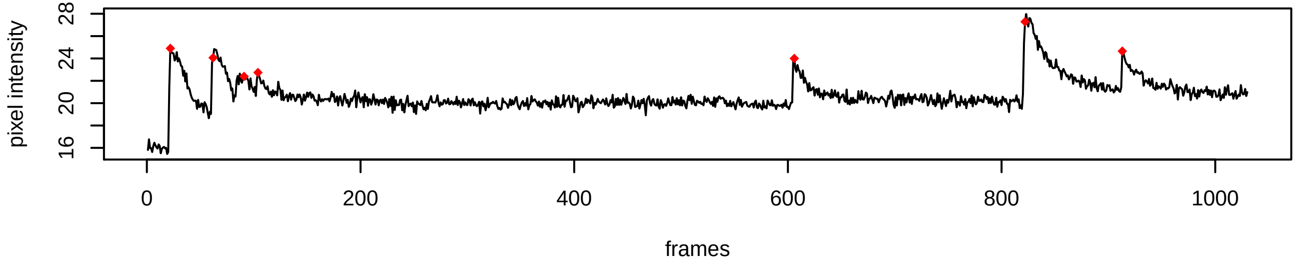

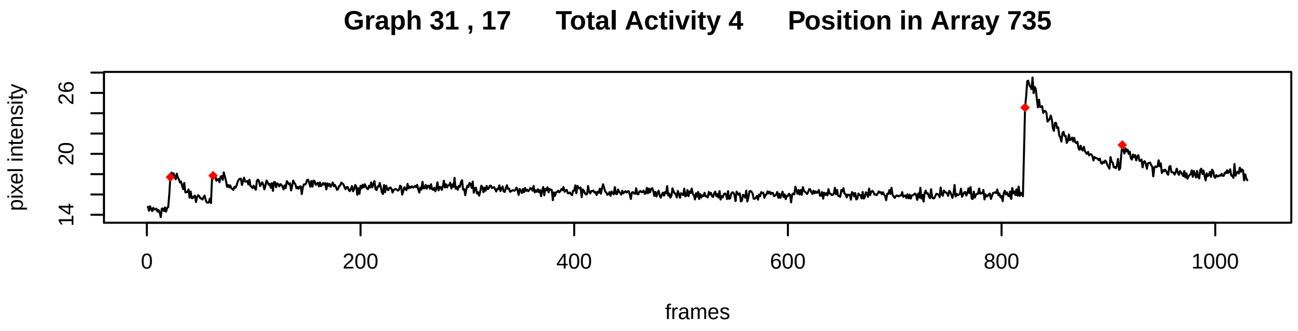
Graph 31 , 17   Total Activity 4   Position in Array 735
pixel intensity
14
20
26
0
200
400
600
800
1000
frames

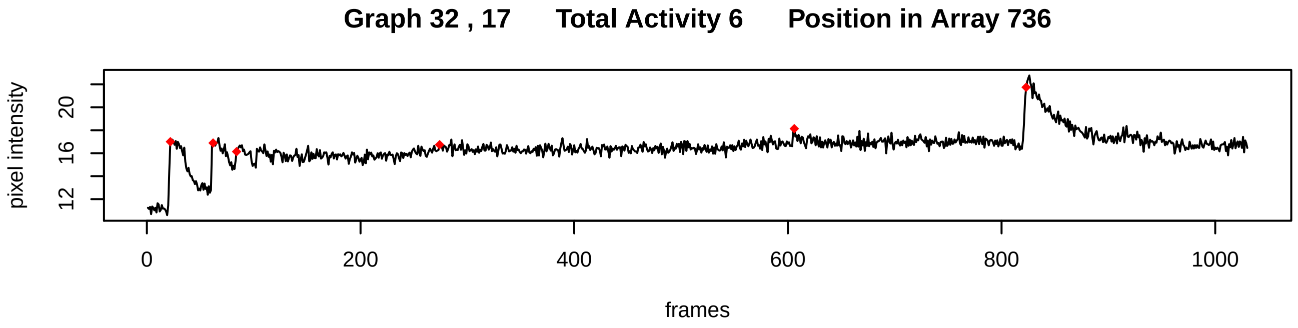
Graph 32 , 17   Total Activity 6   Position in Array 736
pixel intensity
12
16
20
0
200
400
600
800
1000
frames

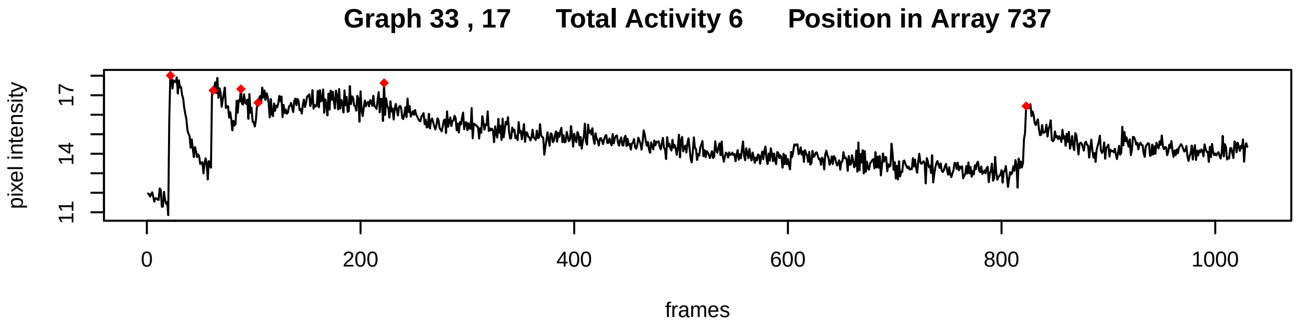
Graph 33 , 17   Total Activity 6   Position in Array 737
pixel intensity
11
14
17
0
200
400
600
800
1000
frames

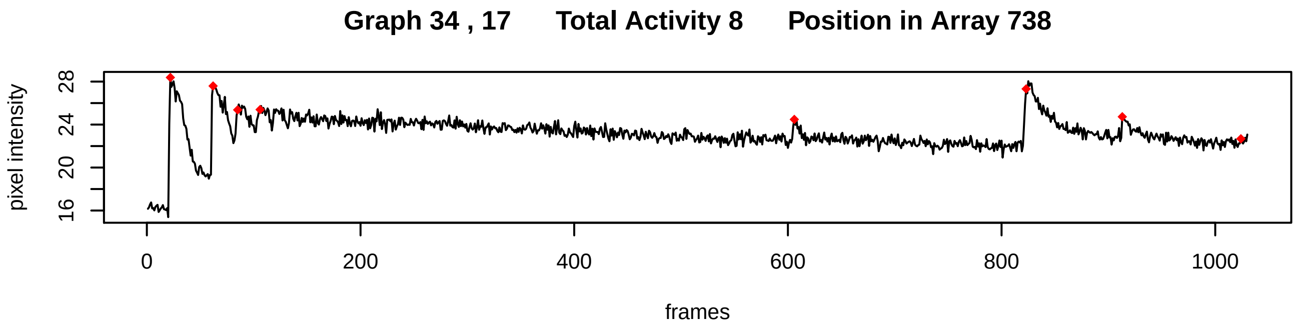
Graph 34 , 17 Total Activity 8 Position in Array 738
pixel intensity
16
20
24
28
0
200
400
600
800
1000
frames

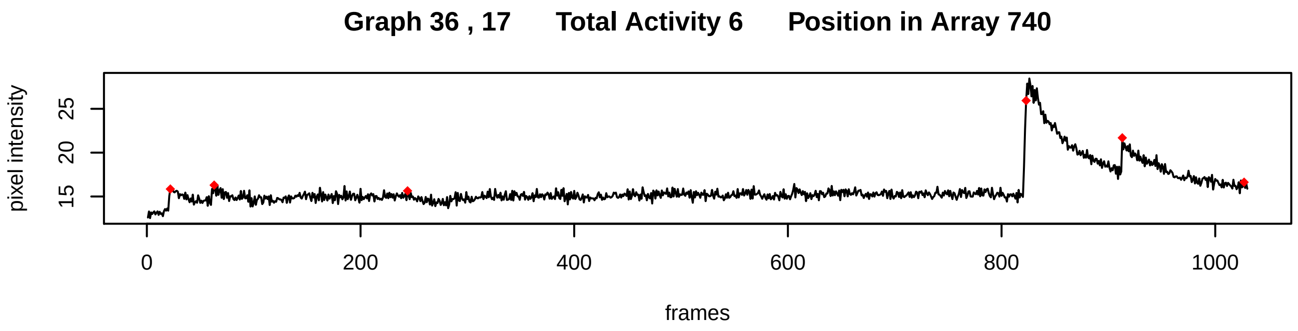
Graph 36 , 17 Total Activity 6 Position in Array 740
pixel intensity
15
20
25
0
200
400
600
800
1000
frames

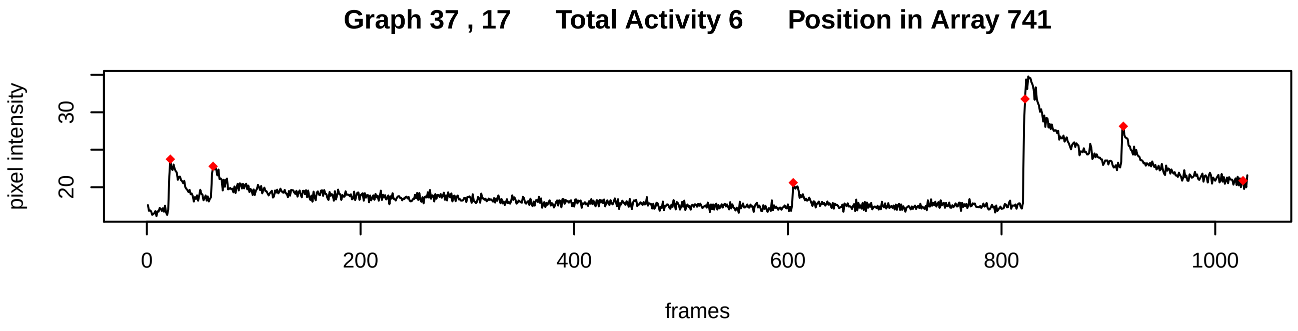
Graph 37 , 17 Total Activity 6 Position in Array 741
pixel intensity
20
30
0
200
400
600
800
1000
frames

Graph 39 , 17 Total Activity 9 Position in Array 743

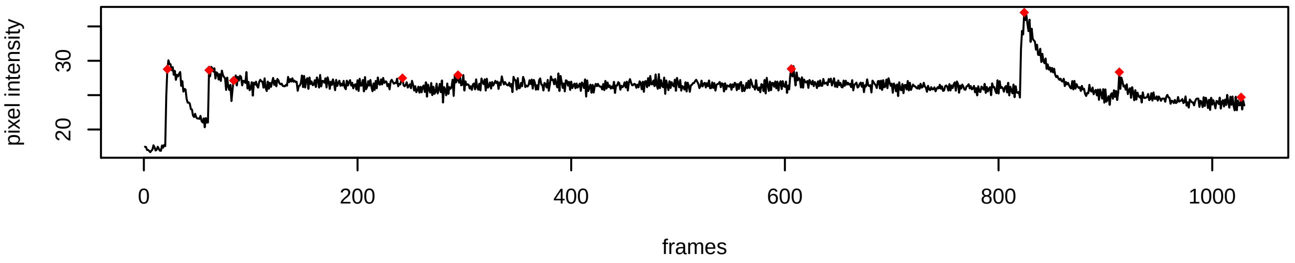

Graph 40 , 17 Total Activity 8 Position in Array 744

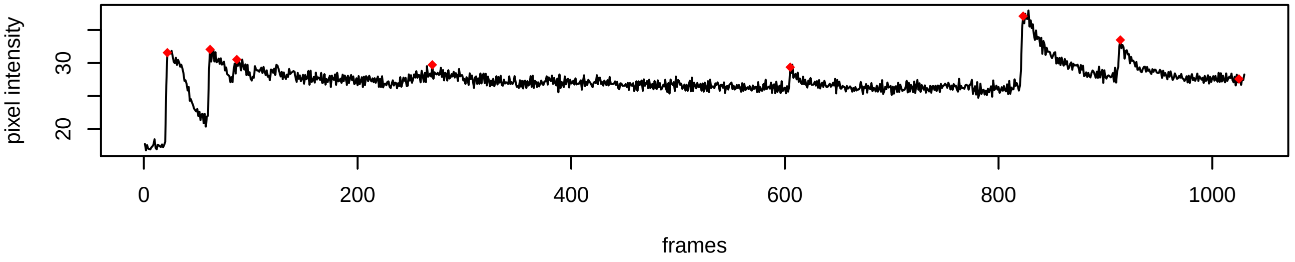

Graph 2 , 16 Total Activity 7 Position in Array 750

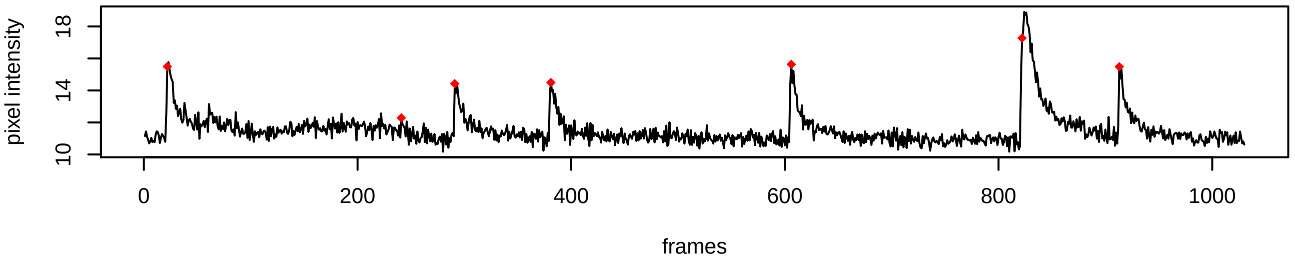

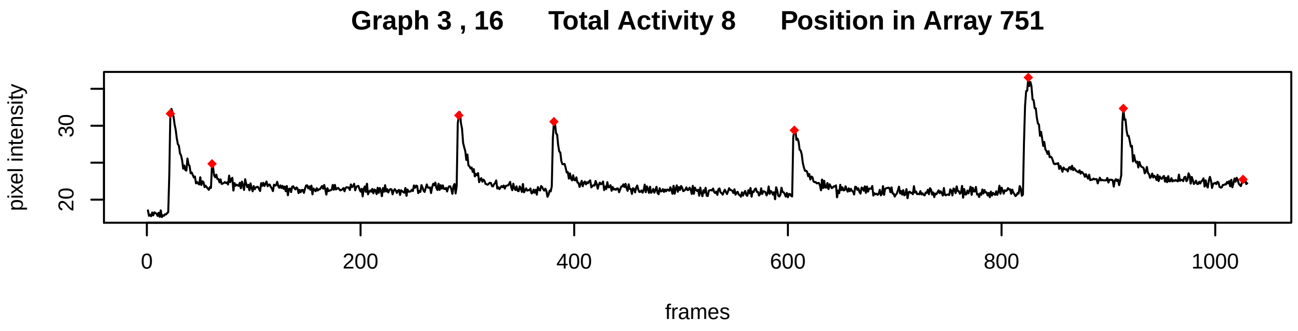
Graph 3 , 16 Total Activity 8 Position in Array 751
pixel intensity
20
30
0
200
400
600
800
1000
frames

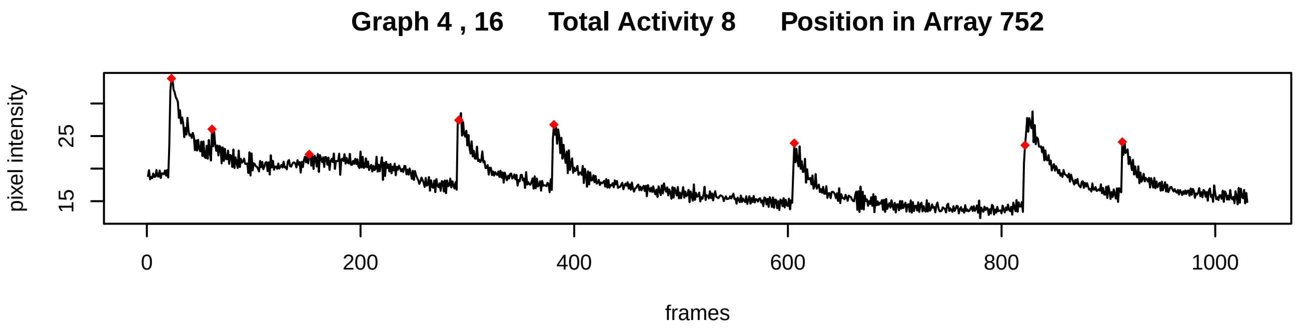
Graph 4 , 16 Total Activity 8 Position in Array 752
pixel intensity
15
25
0
200
400
600
800
1000
frames

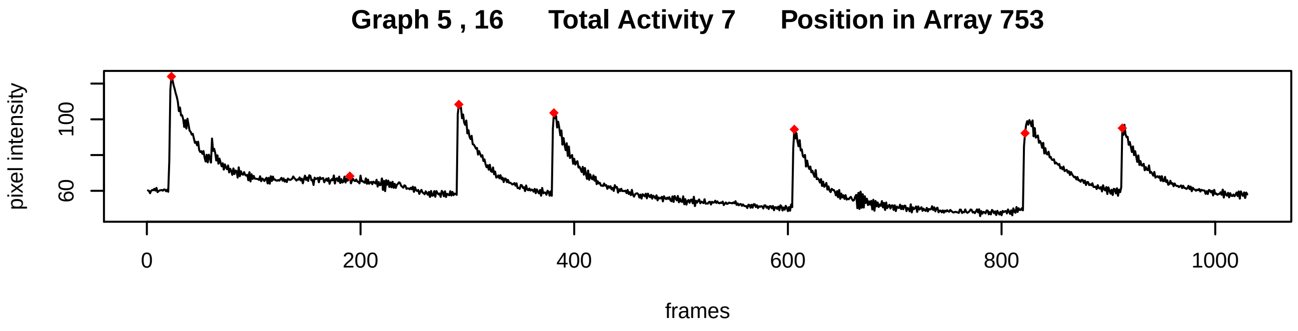
Graph 5 , 16 Total Activity 7 Position in Array 753
pixel intensity
60
100
0
200
400
600
800
1000
frames

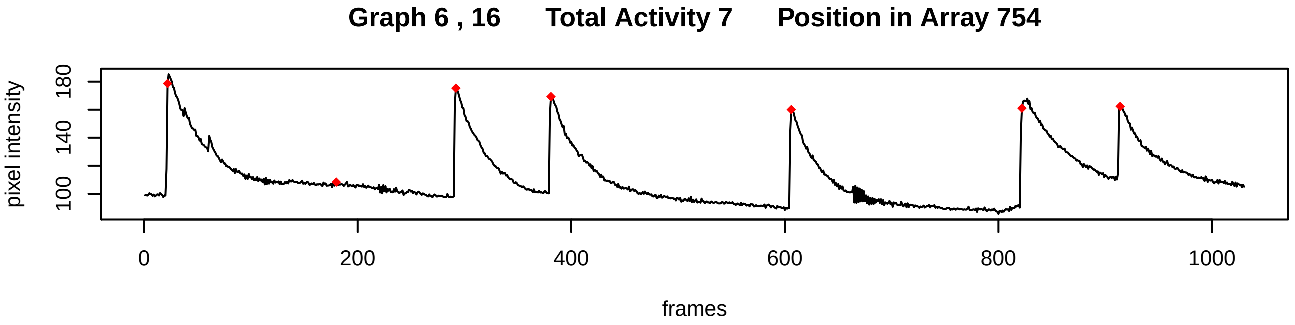
Graph 6 , 16 Total Activity 7 Position in Array 754
pixel intensity
100
140
180
0
200
400
600
800
1000
frames

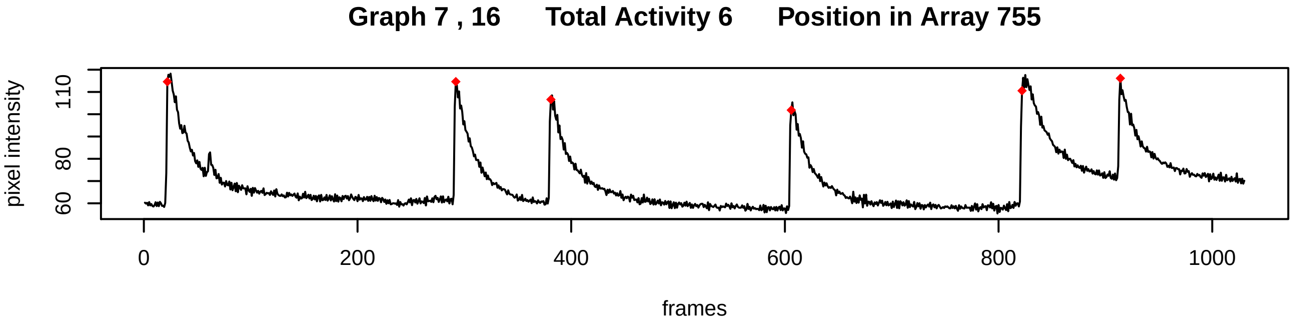
Graph 7 , 16 Total Activity 6 Position in Array 755
pixel intensity
60
80
110
0
200
400
600
800
1000
frames

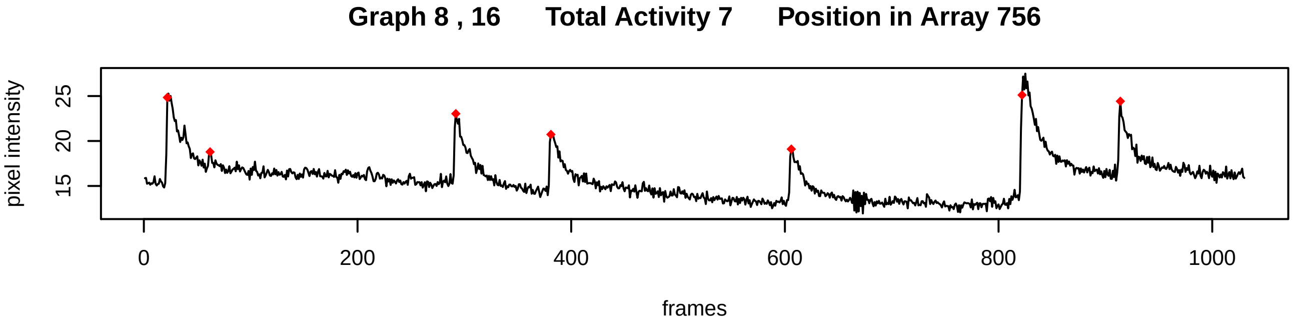
Graph 8 , 16 Total Activity 7 Position in Array 756
pixel intensity
15
20
25
0
200
400
600
800
1000
frames

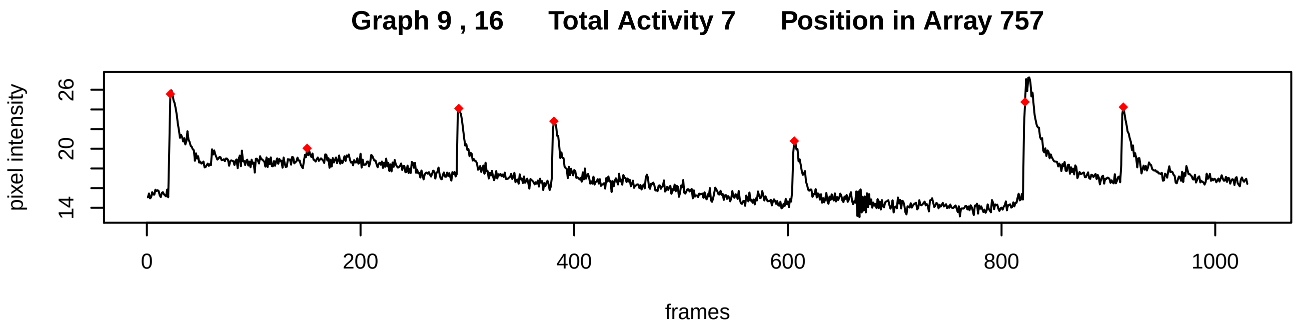

Graph 9 , 16 Total Activity 7 Position in Array 757
pixel intensity
14
20
26
0
200
400
600
800
1000
frames

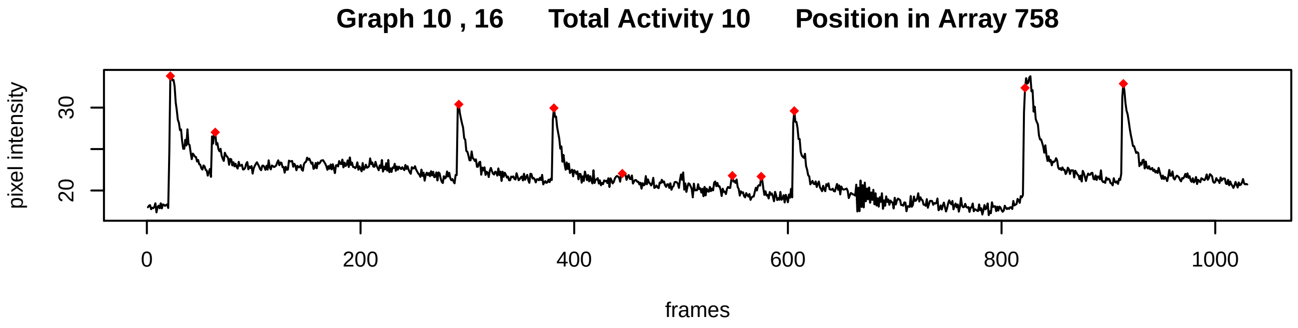

Graph 10 , 16 Total Activity 10 Position in Array 758
pixel intensity
20
30
0
200
400
600
800
1000
frames

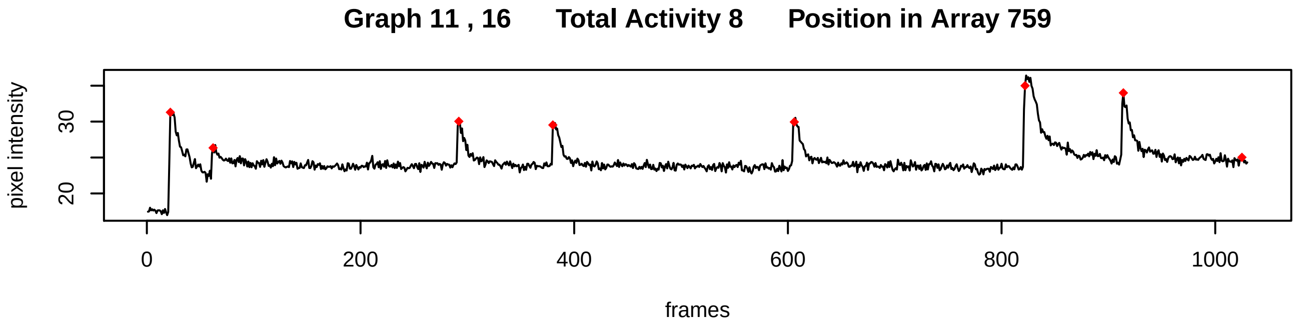

Graph 11 , 16 Total Activity 8 Position in Array 759
pixel intensity
20
30
0
200
400
600
800
1000
frames

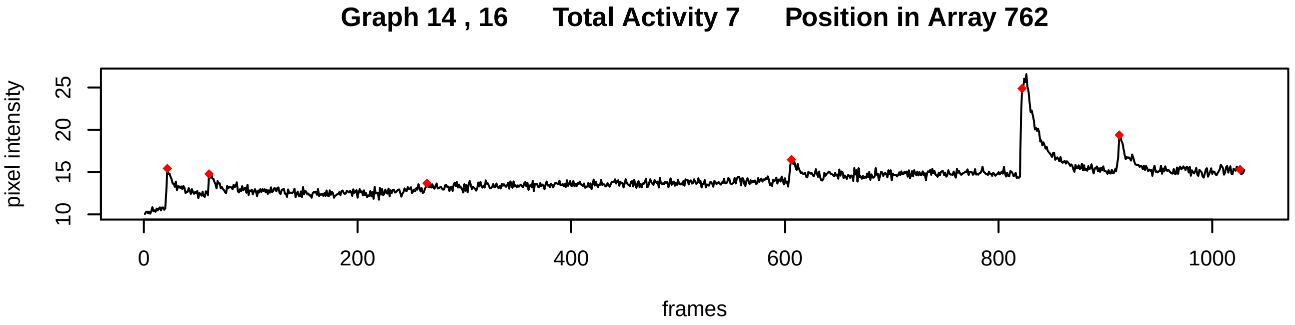
Graph 14 , 16 Total Activity 7 Position in Array 762
pixel intensity
10
15
20
25
0
200
400
600
800
1000
frames

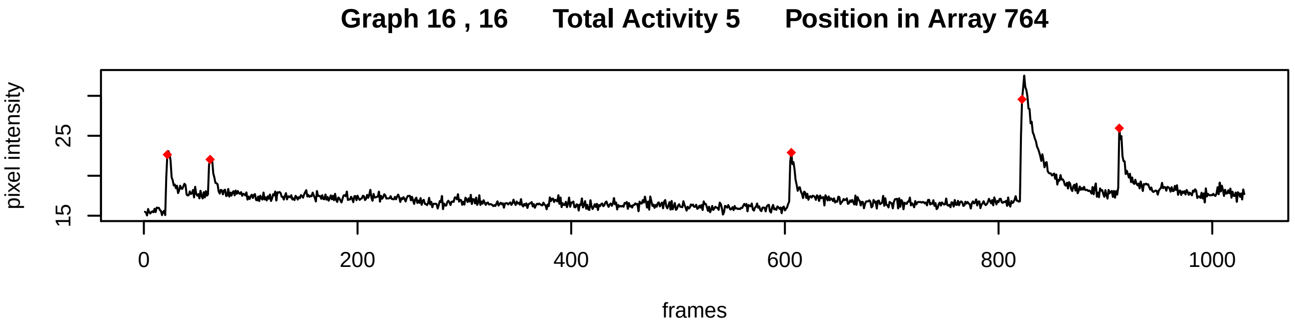
Graph 16 , 16 Total Activity 5 Position in Array 764
pixel intensity
15
25
0
200
400
600
800
1000
frames

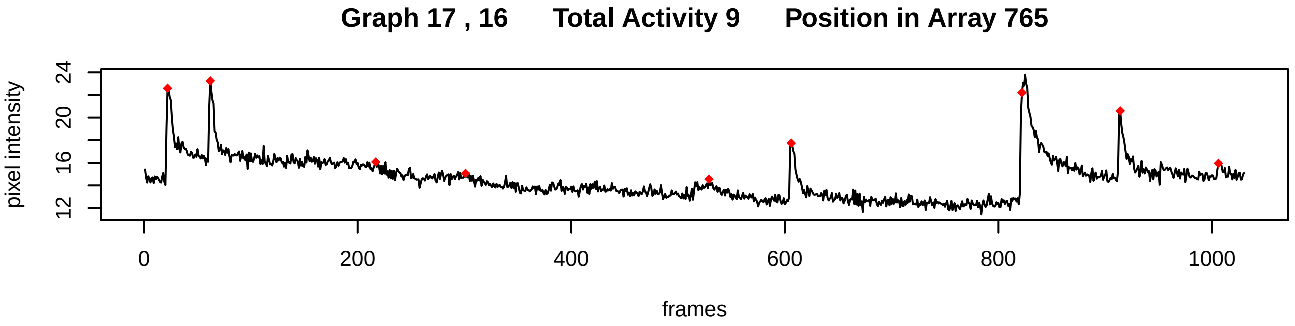
Graph 17 , 16 Total Activity 9 Position in Array 765
pixel intensity
12
16
20
24
0
200
400
600
800
1000
frames

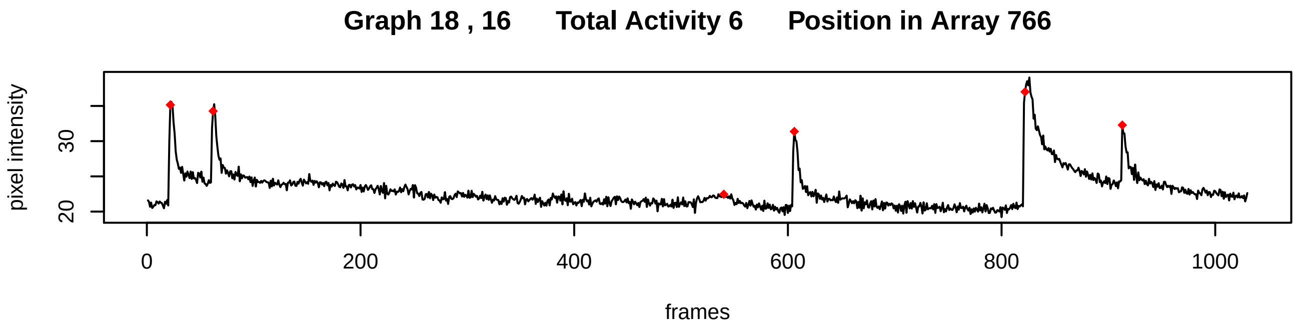
Graph 18 , 16 Total Activity 6 Position in Array 766
pixel intensity
20
30
0
200
400
600
800
1000
frames

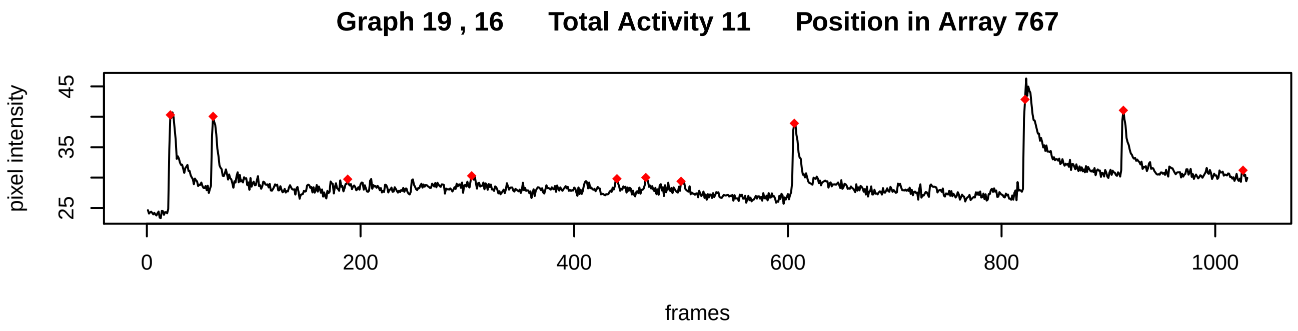
Graph 19 , 16 Total Activity 11 Position in Array 767
pixel intensity
25
35
45
0
200
400
600
800
1000
frames

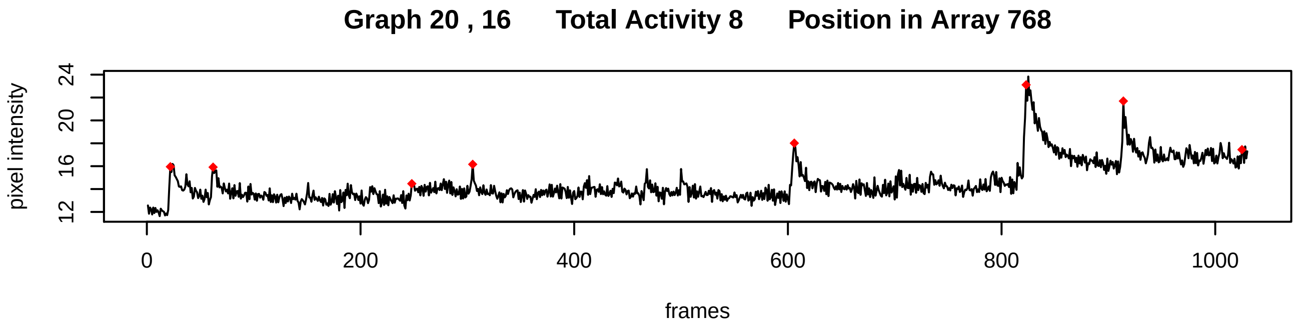
Graph 20 , 16 Total Activity 8 Position in Array 768
pixel intensity
12
16
20
24
0
200
400
600
800
1000
frames

Graph 21 , 16 Total Activity 5 Position in Array 769

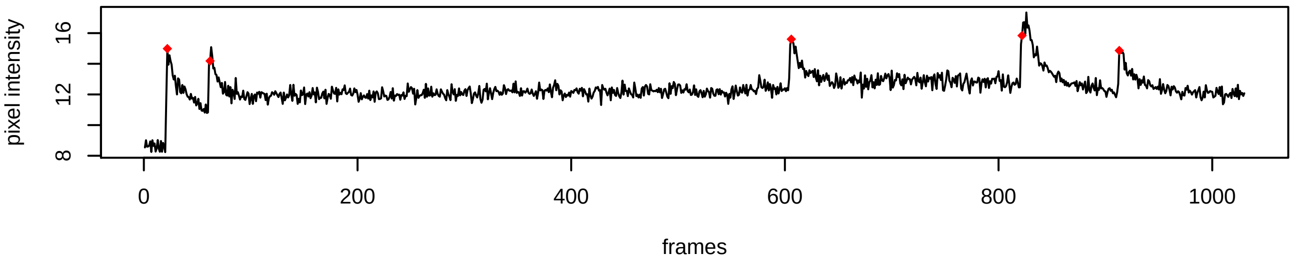

Graph 22 , 16 Total Activity 6 Position in Array 770

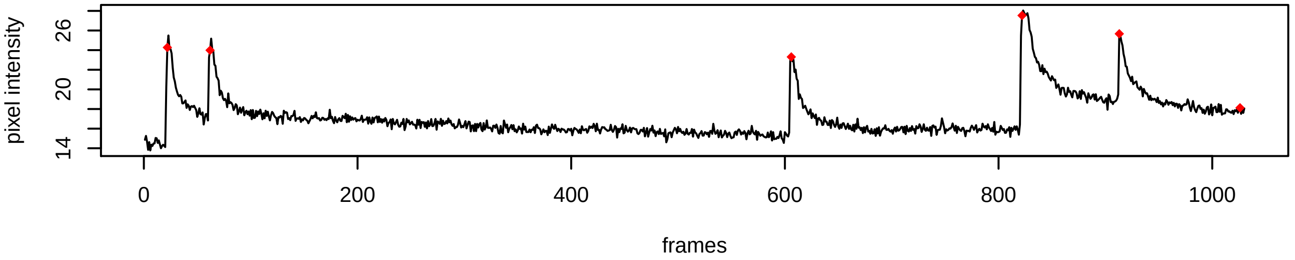

Graph 23 , 16 Total Activity 7 Position in Array 771

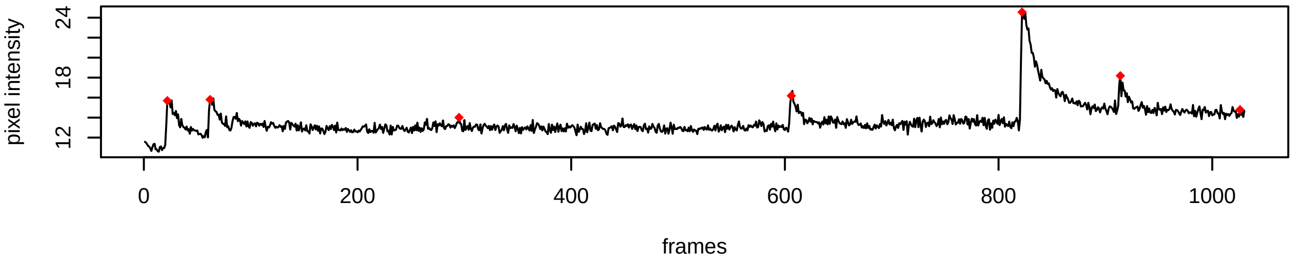

Graph 26 , 16 Total Activity 5 Position in Array 774

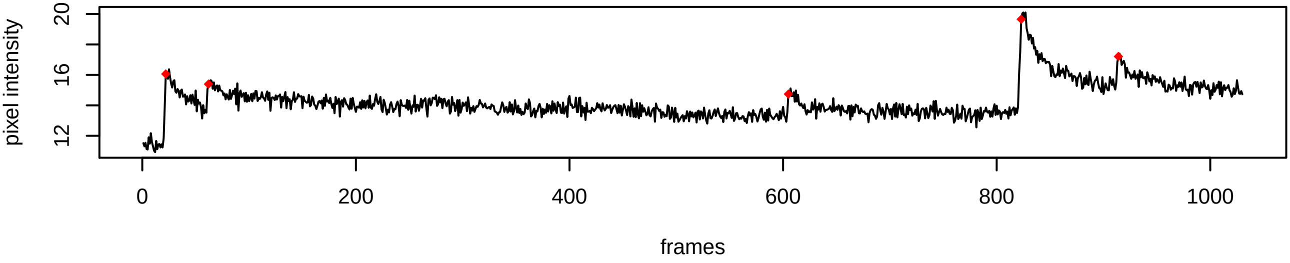

Graph 29 , 16 Total Activity 7 Position in Array 777

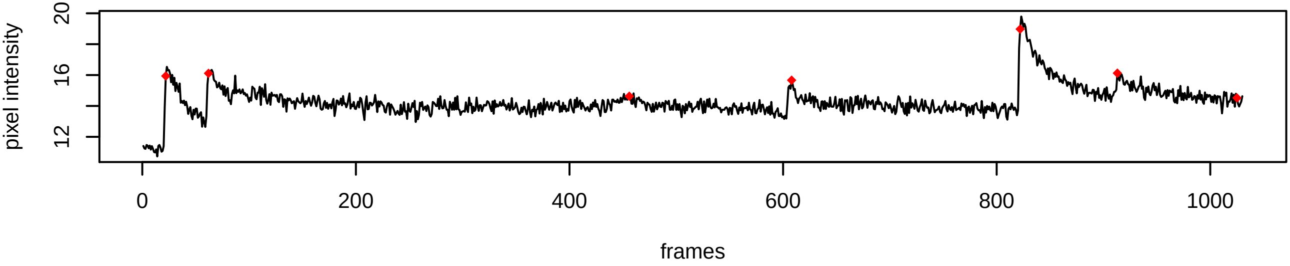

Graph 30 , 16 Total Activity 4 Position in Array 778

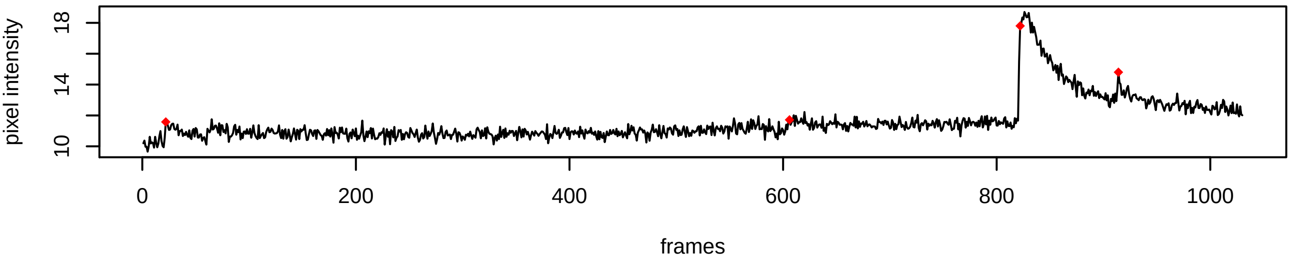

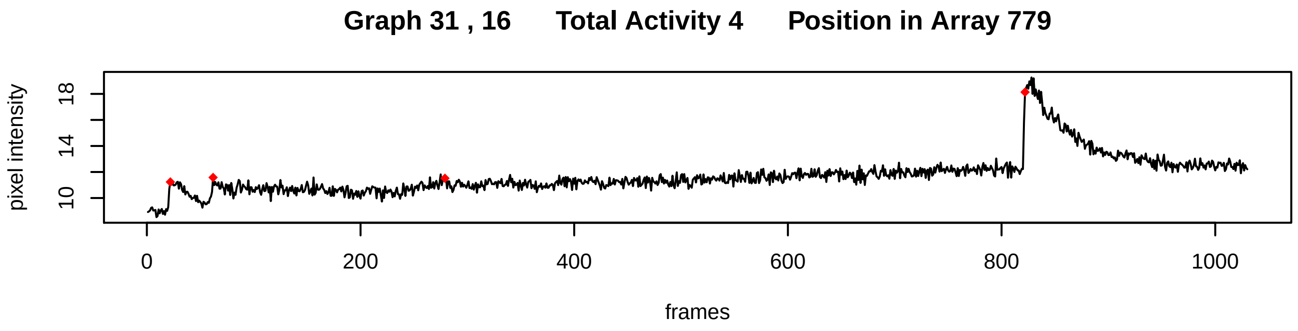
Graph 31 , 16 Total Activity 4 Position in Array 779
pixel intensity
10
14
18
0
200
400
600
800
1000
frames

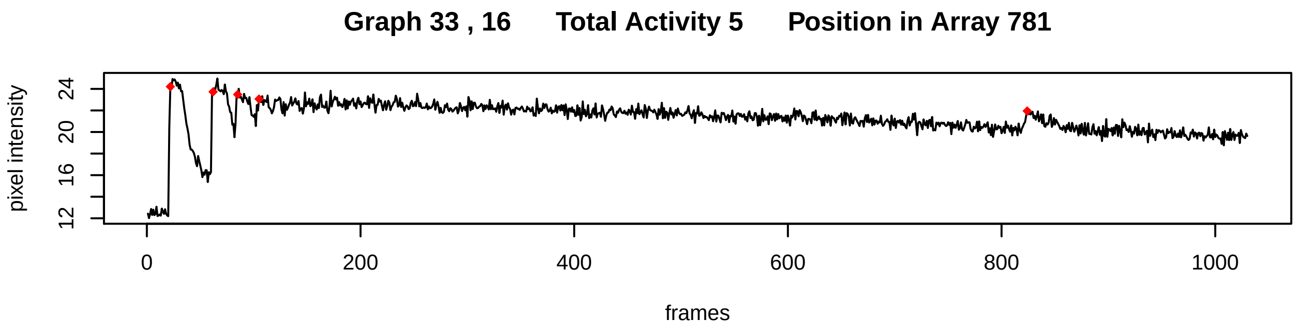
Graph 33 , 16 Total Activity 5 Position in Array 781
pixel intensity
12
16
20
24
0
200
400
600
800
1000
frames

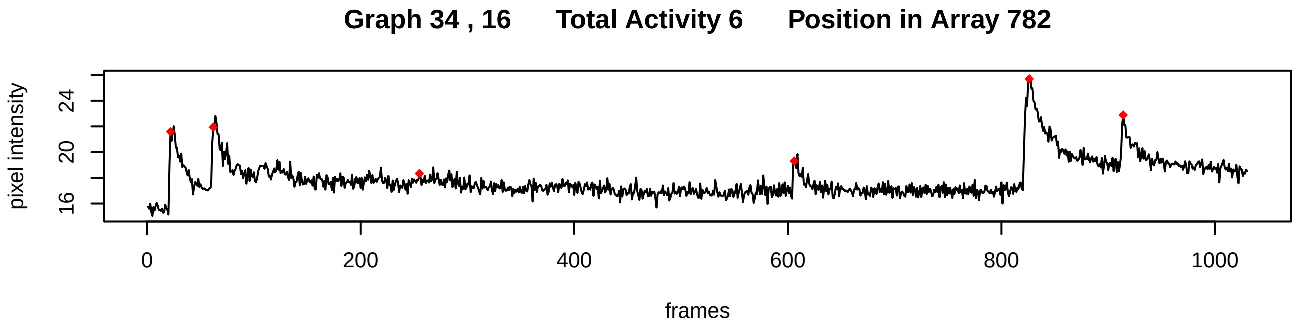
Graph 34 , 16 Total Activity 6 Position in Array 782
pixel intensity
16
20
24
0
200
400
600
800
1000
frames

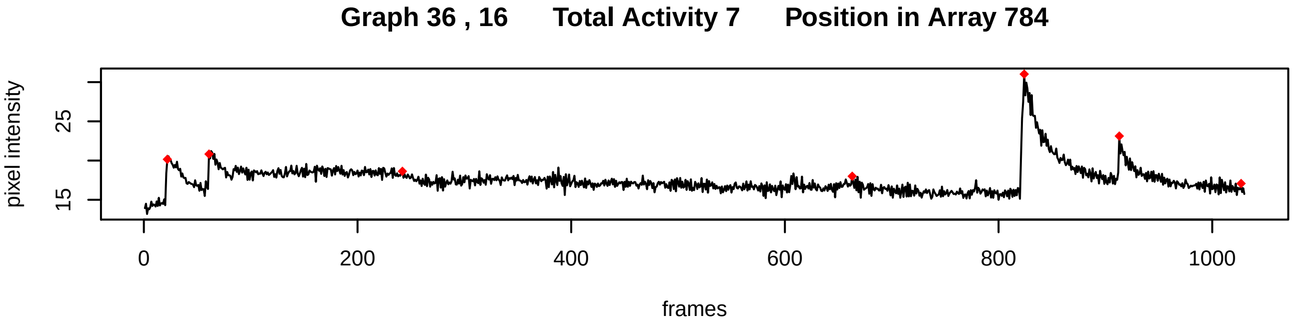
Graph 36 , 16 Total Activity 7 Position in Array 784
pixel intensity
15
25
0
200
400
600
800
1000
frames

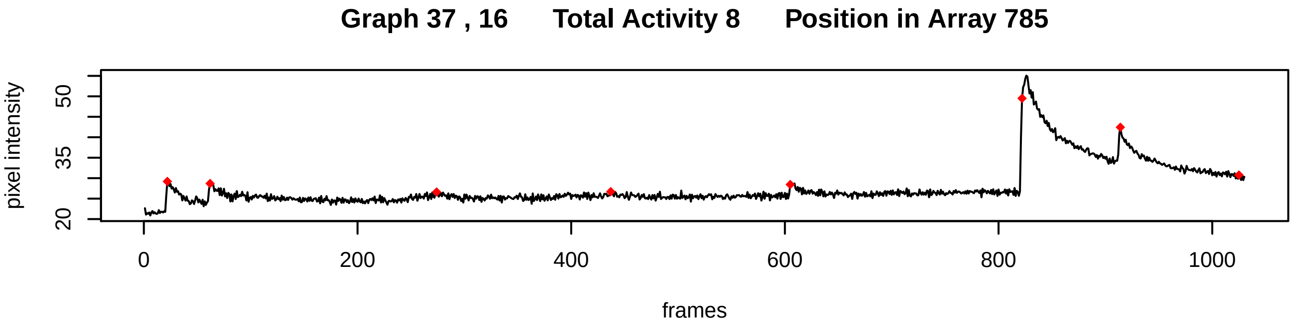
Graph 37 , 16 Total Activity 8 Position in Array 785
pixel intensity
20
35
50
0
200
400
600
800
1000
frames

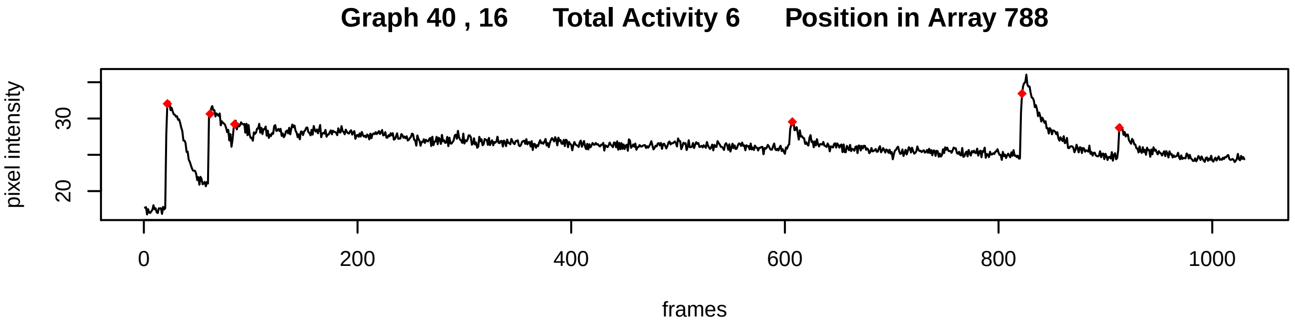
Graph 40 , 16 Total Activity 6 Position in Array 788
pixel intensity
20
30
0
200
400
600
800
1000
frames

Graph 41 , 16   Total Activity 7   Position in Array 789

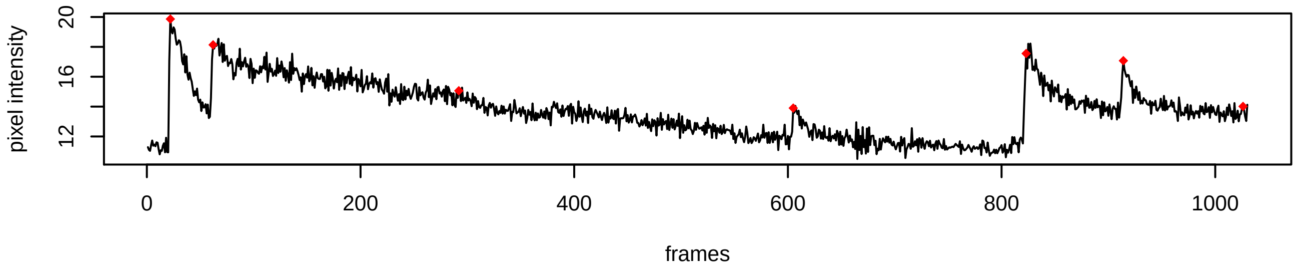

Graph 2 , 15   Total Activity 8   Position in Array 794

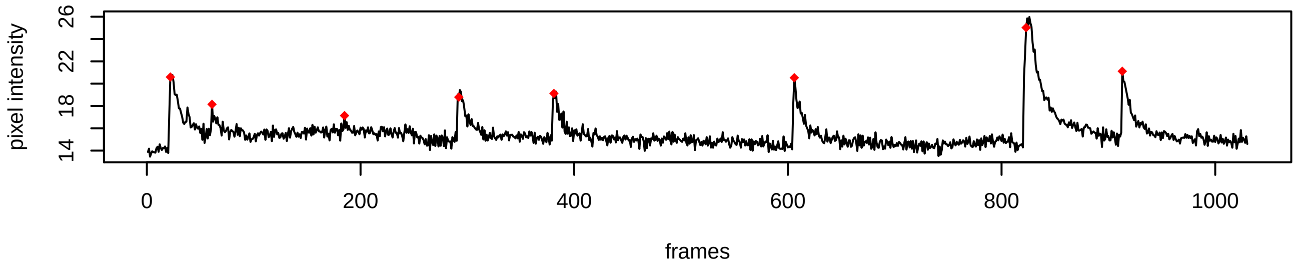

Graph 3 , 15   Total Activity 6   Position in Array 795

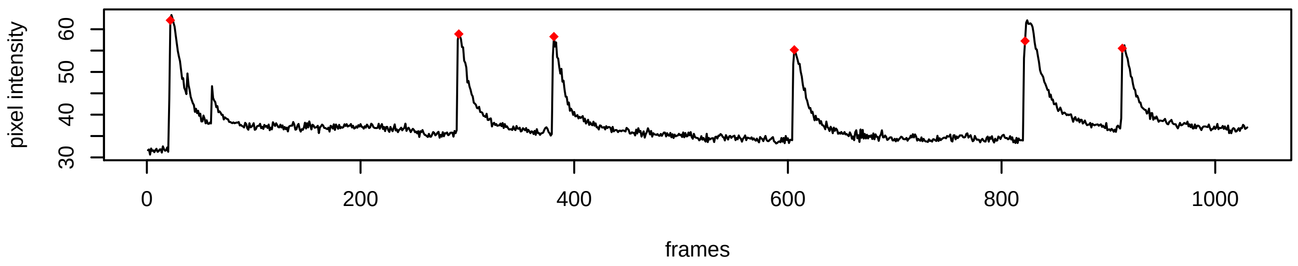

Graph 4 , 15 Total Activity 7 Position in Array 796

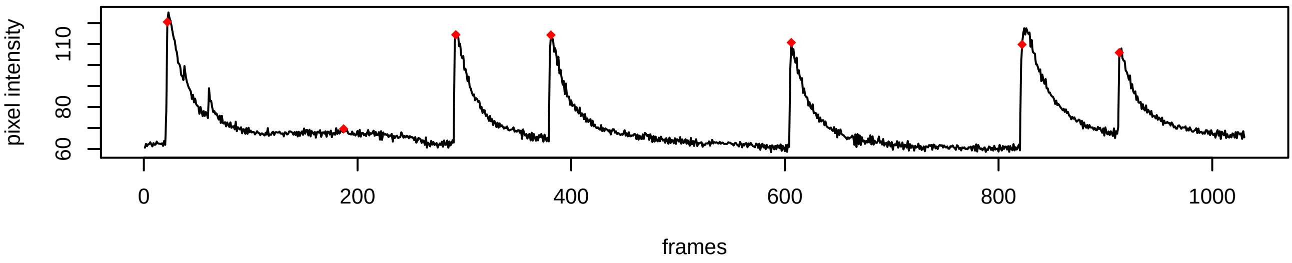

Graph 5 , 15 Total Activity 6 Position in Array 797

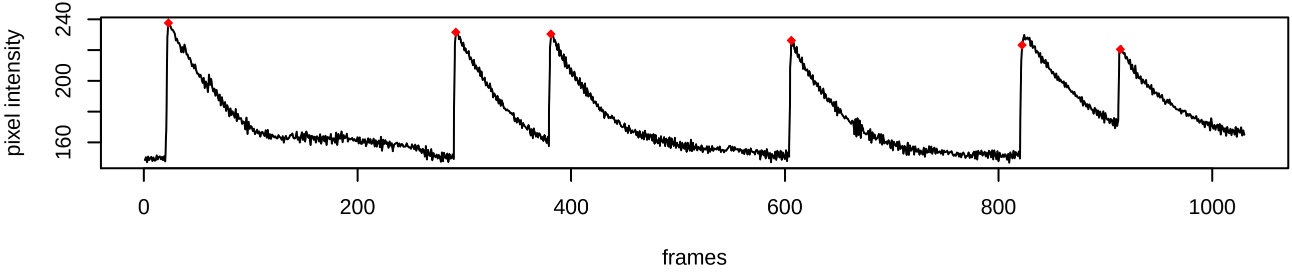

Graph 6 , 15 Total Activity 5 Position in Array 798

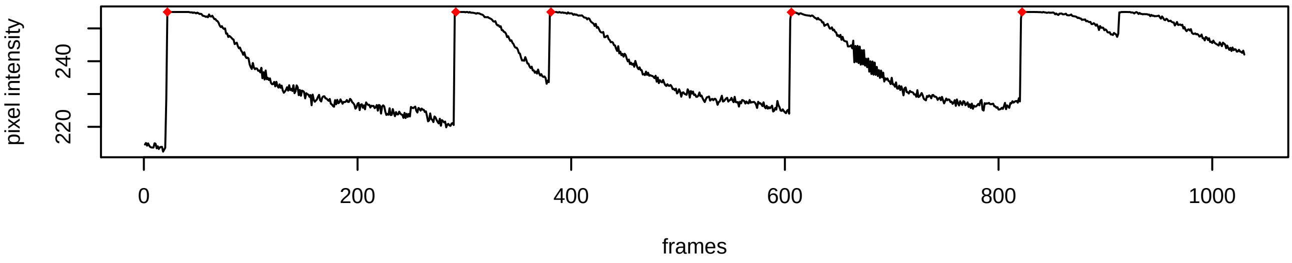

Graph 7 , 15   Total Activity 6   Position in Array 799

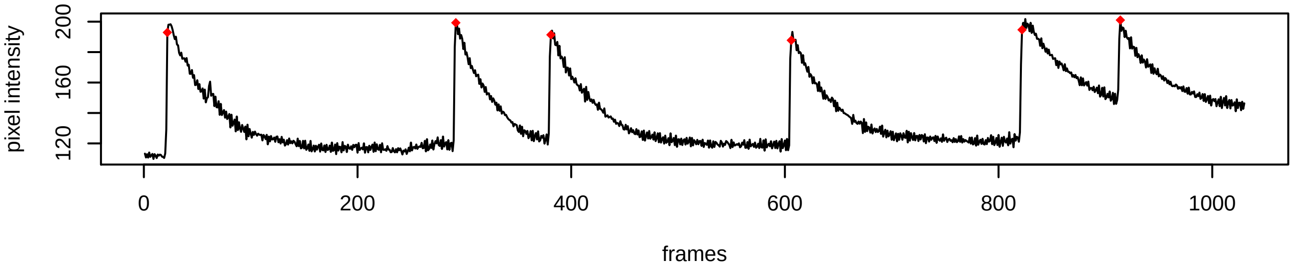

Graph 8 , 15   Total Activity 7   Position in Array 800

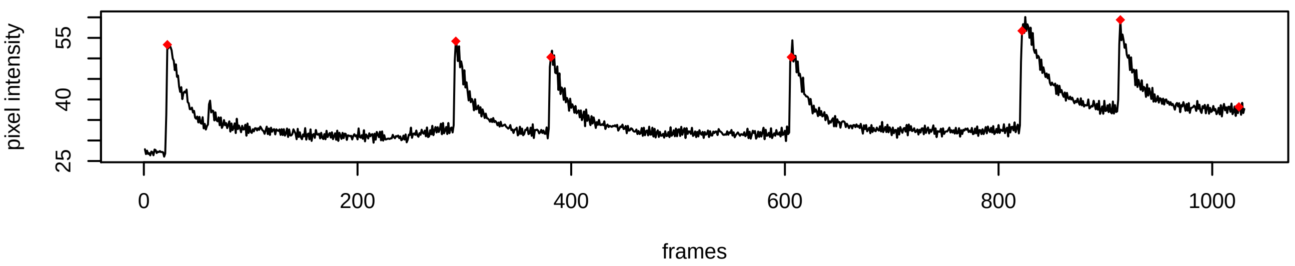

Graph 9 , 15   Total Activity 7   Position in Array 801

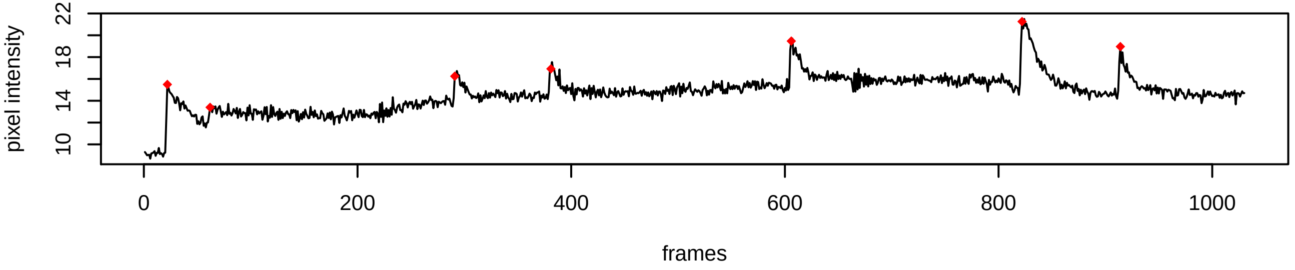

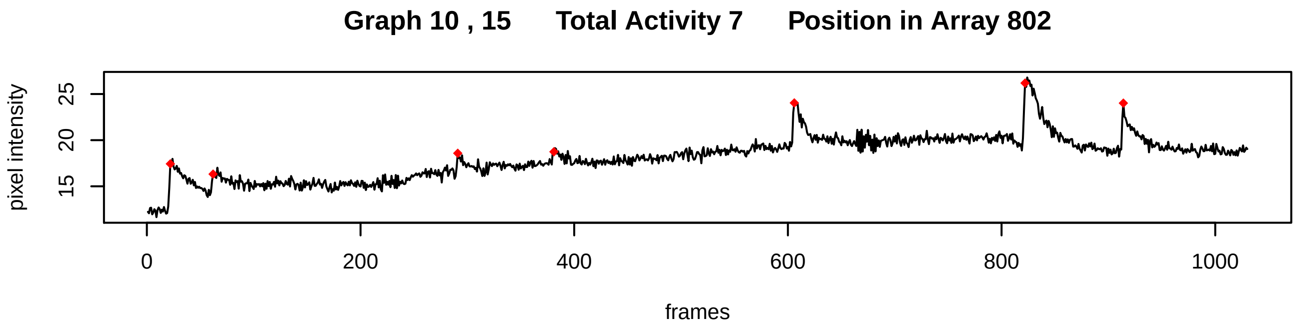
Graph 10 , 15 Total Activity 7 Position in Array 802
pixel intensity
15
20
25
0
200
400
600
800
1000
frames

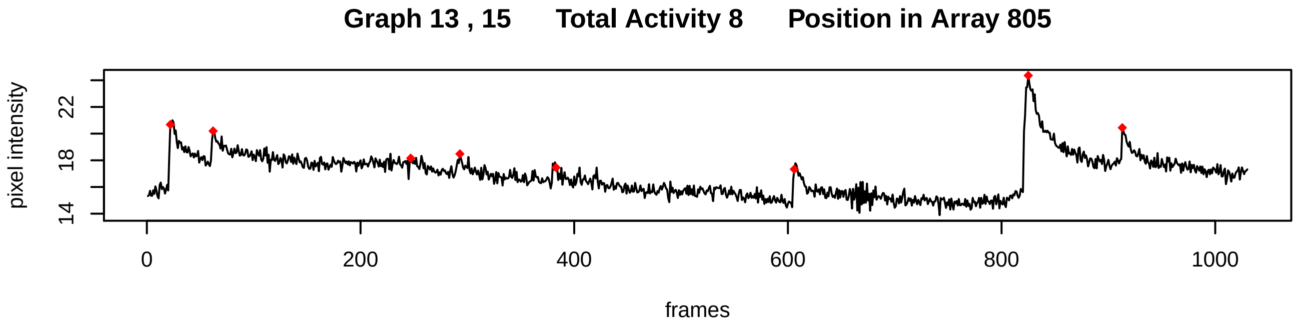
Graph 13 , 15 Total Activity 8 Position in Array 805
pixel intensity
14
18
22
0
200
400
600
800
1000
frames

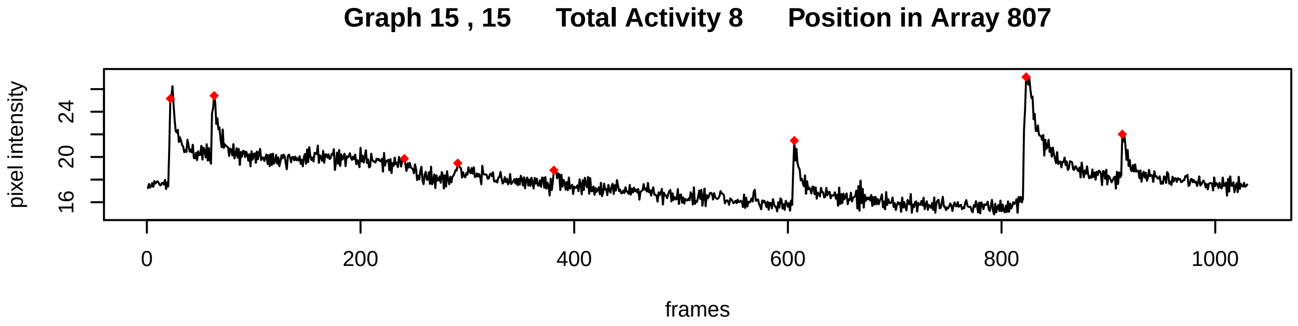
Graph 15 , 15 Total Activity 8 Position in Array 807
pixel intensity
16
20
24
0
200
400
600
800
1000
frames

Graph 16 , 15   Total Activity 6   Position in Array 808

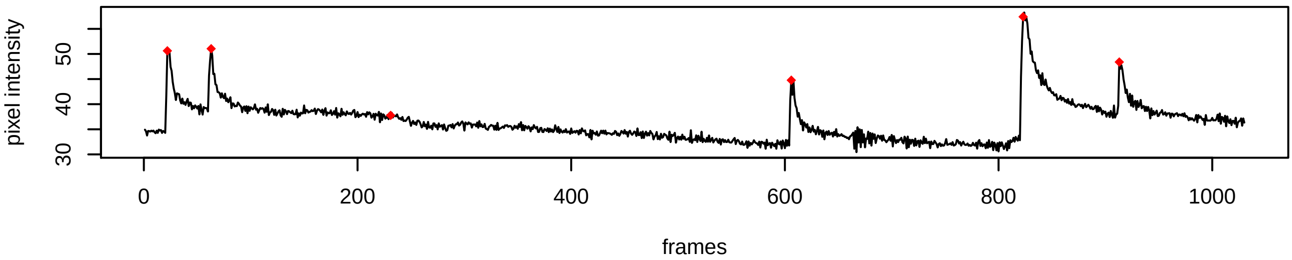

Graph 17 , 15   Total Activity 5   Position in Array 809

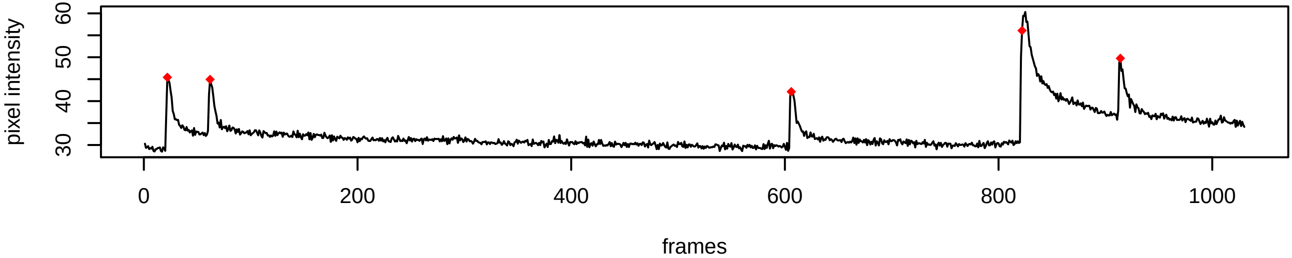

Graph 18 , 15   Total Activity 6   Position in Array 810

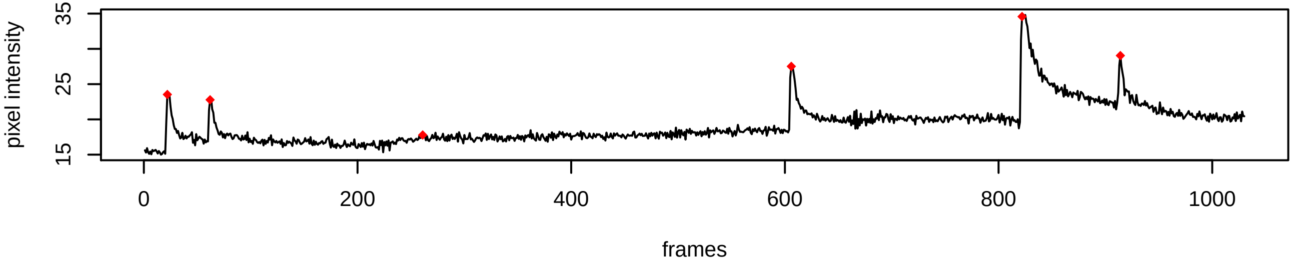

Graph 19 , 15 Total Activity 6 Position in Array 811

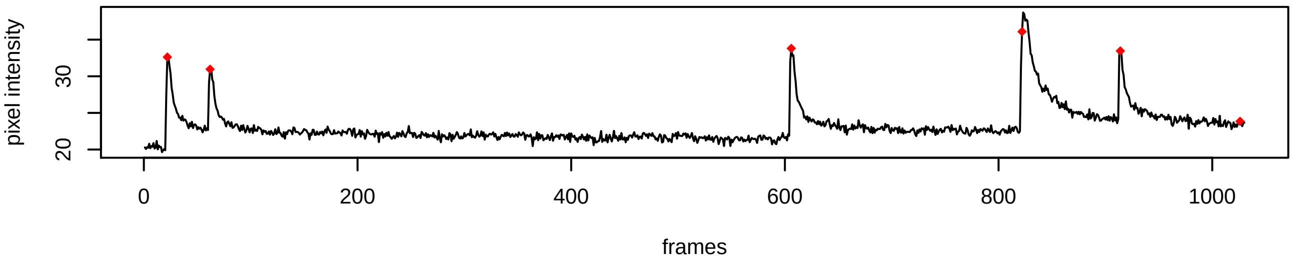

Graph 20 , 15 Total Activity 7 Position in Array 812

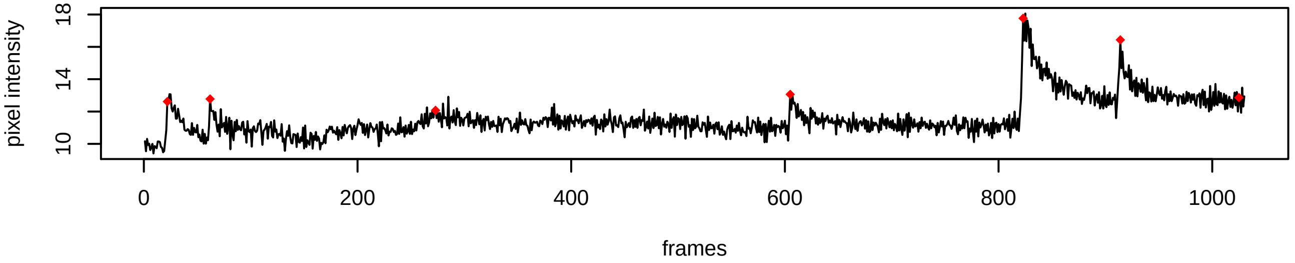

Graph 22 , 15 Total Activity 6 Position in Array 814

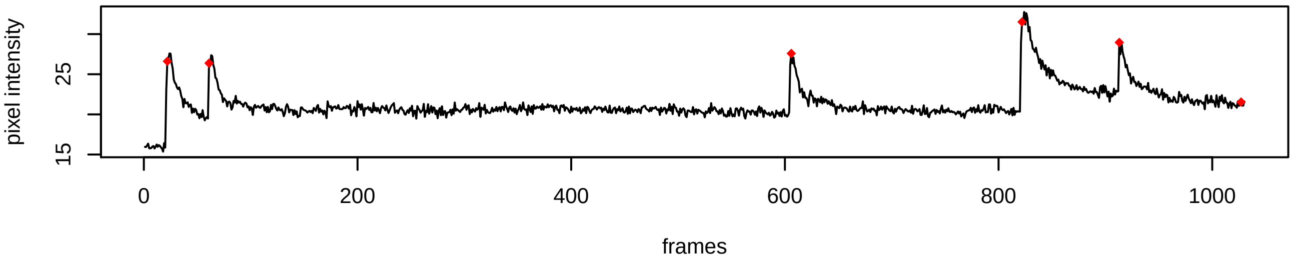

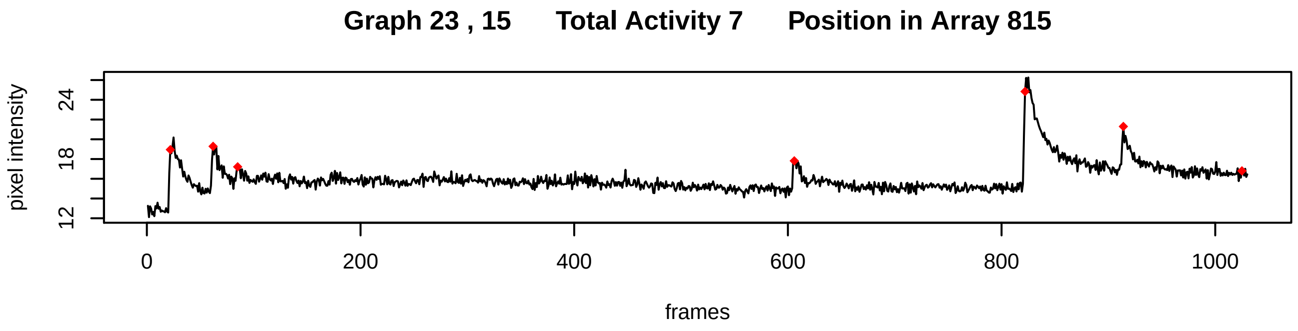
Graph 23 , 15 Total Activity 7 Position in Array 815
pixel intensity
12
18
24
0
200
400
600
800
1000
frames

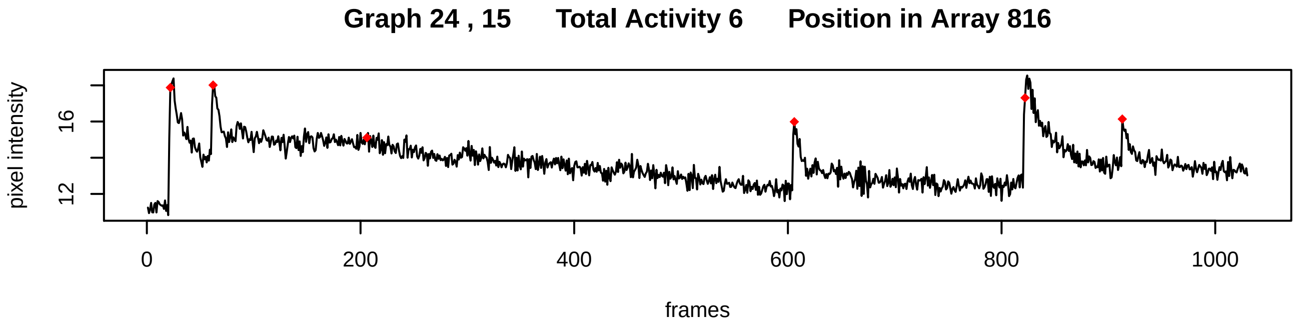
Graph 24 , 15 Total Activity 6 Position in Array 816
pixel intensity
12
16
0
200
400
600
800
1000
frames

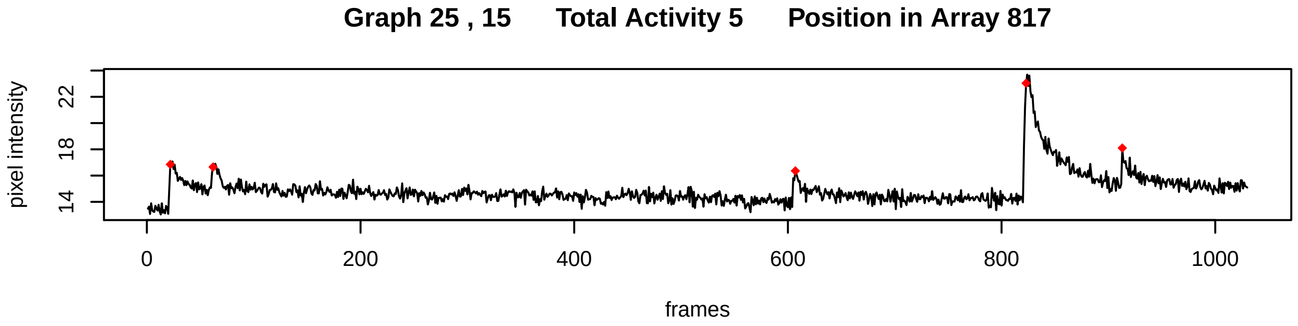
Graph 25 , 15 Total Activity 5 Position in Array 817
pixel intensity
14
18
22
0
200
400
600
800
1000
frames

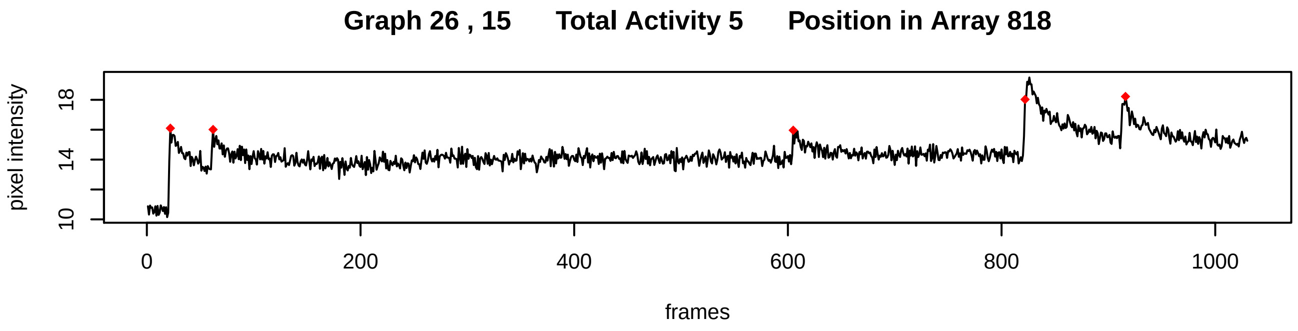
Graph 26 , 15 Total Activity 5 Position in Array 818
pixel intensity
10
14
18
0
200
400
600
800
1000
frames

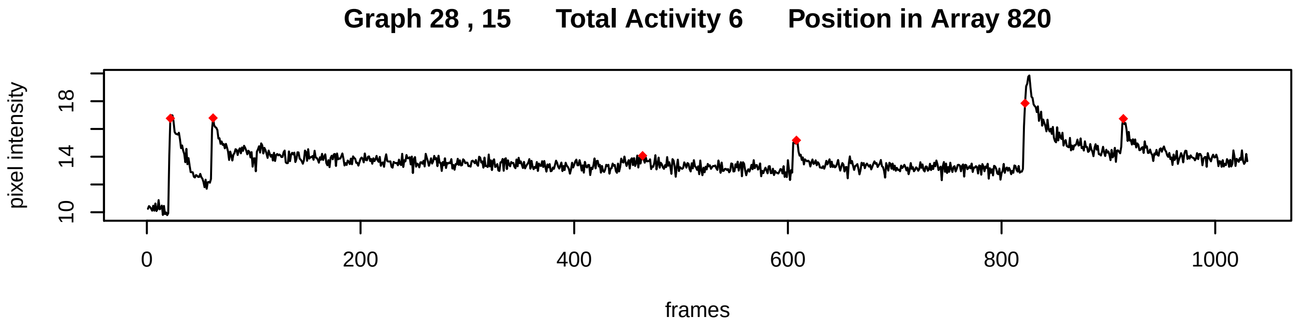
Graph 28 , 15 Total Activity 6 Position in Array 820
pixel intensity
10
14
18
0
200
400
600
800
1000
frames

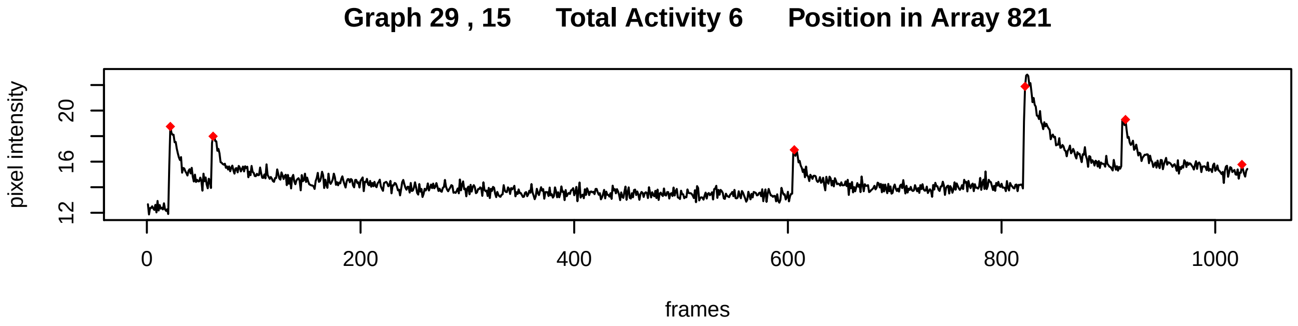
Graph 29 , 15 Total Activity 6 Position in Array 821
pixel intensity
12
16
20
0
200
400
600
800
1000
frames

Graph 30 , 15 Total Activity 5 Position in Array 822

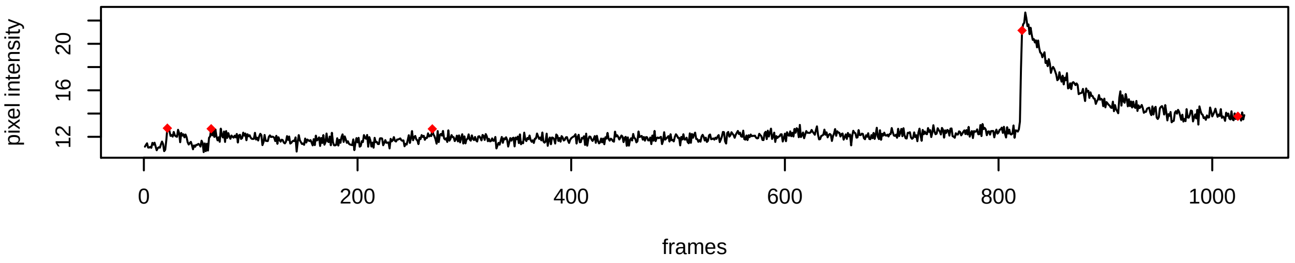

Graph 32 , 15 Total Activity 7 Position in Array 824

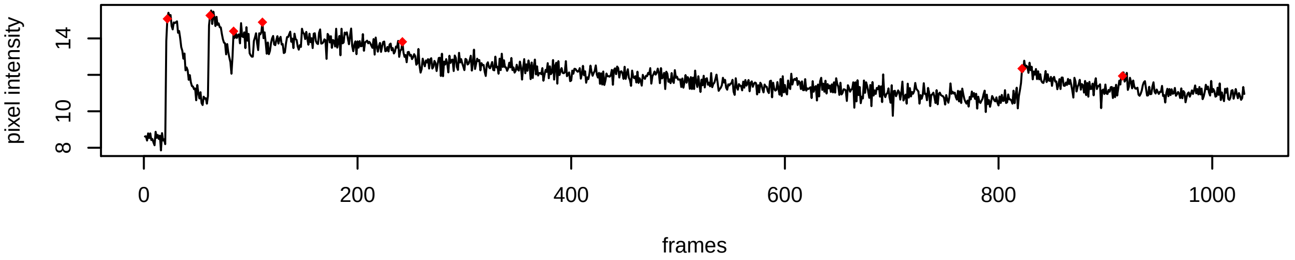

Graph 33 , 15 Total Activity 8 Position in Array 825

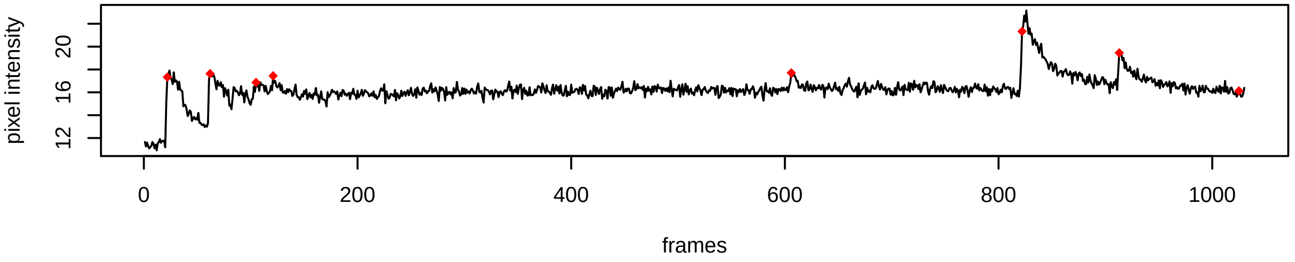

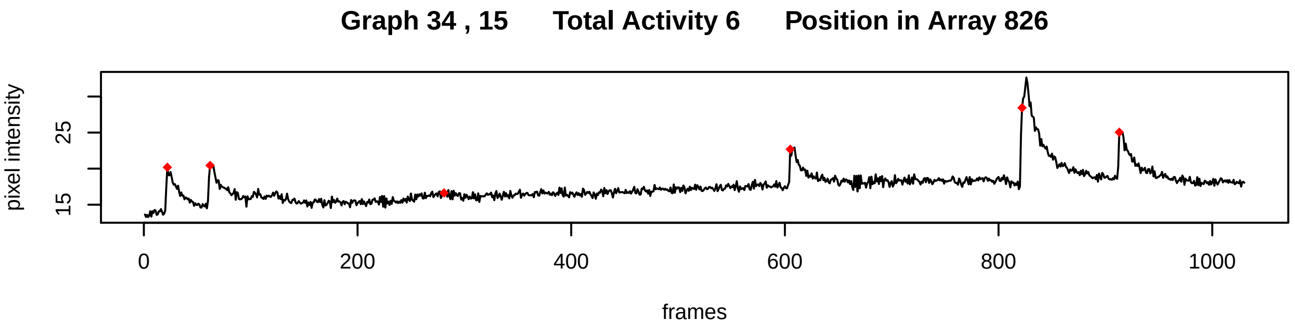
Graph 34 , 15 Total Activity 6 Position in Array 826
pixel intensity
15
25
0
200
400
600
800
1000
frames

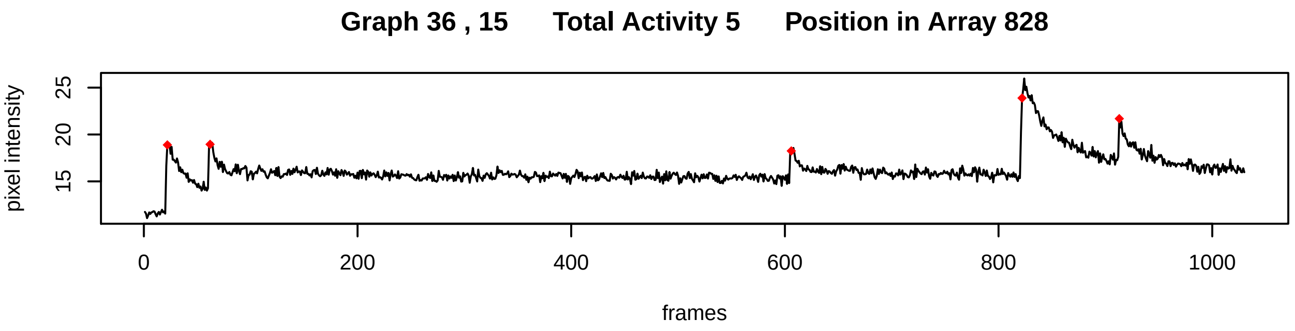
Graph 36 , 15 Total Activity 5 Position in Array 828
pixel intensity
15
20
25
0
200
400
600
800
1000
frames

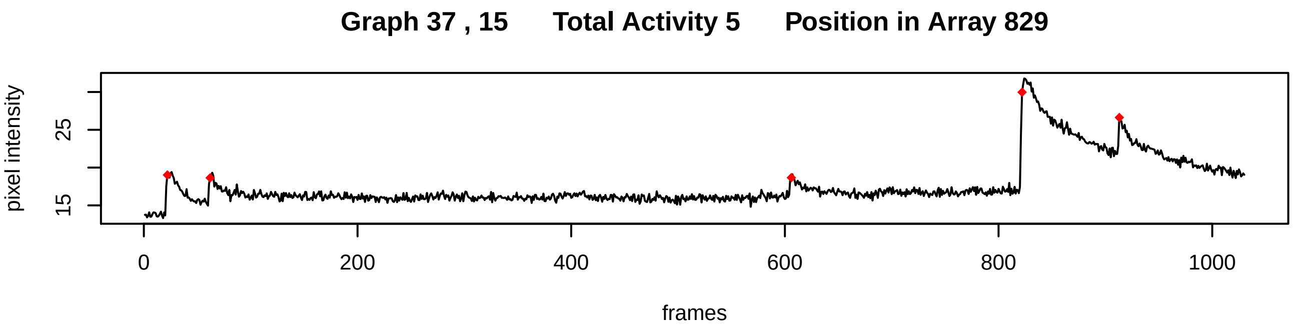
Graph 37 , 15 Total Activity 5 Position in Array 829
pixel intensity
15
25
0
200
400
600
800
1000
frames

Graph 38 , 15   Total Activity 7   Position in Array 830

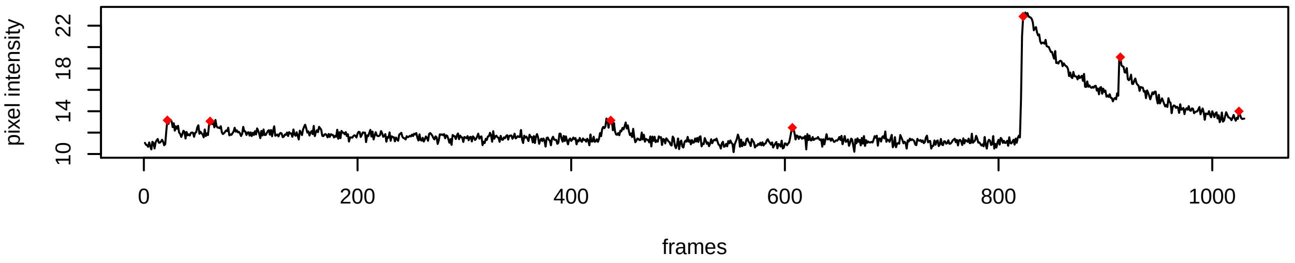

Graph 40 , 15   Total Activity 7   Position in Array 832

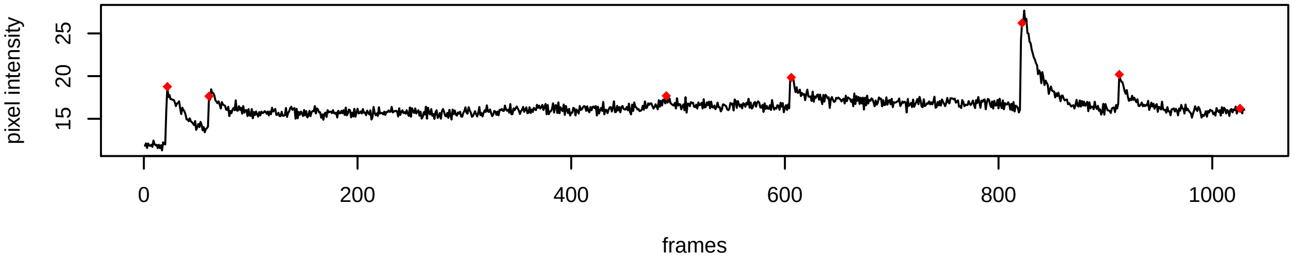

Graph 41 , 15   Total Activity 6   Position in Array 833

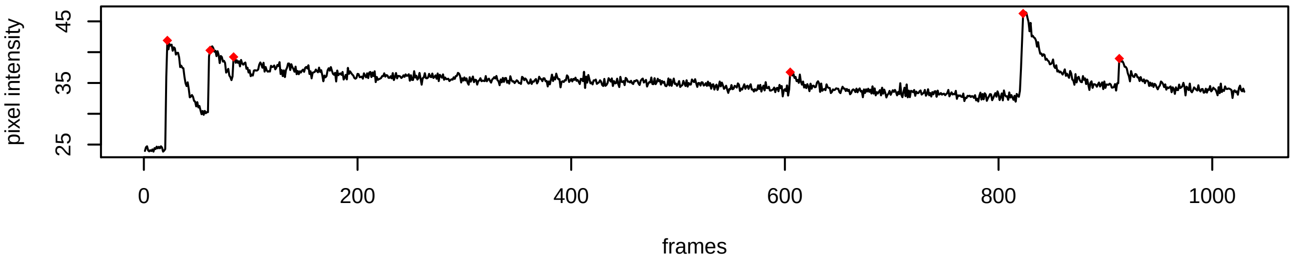

Graph 3 , 14 Total Activity 6 Position in Array 839

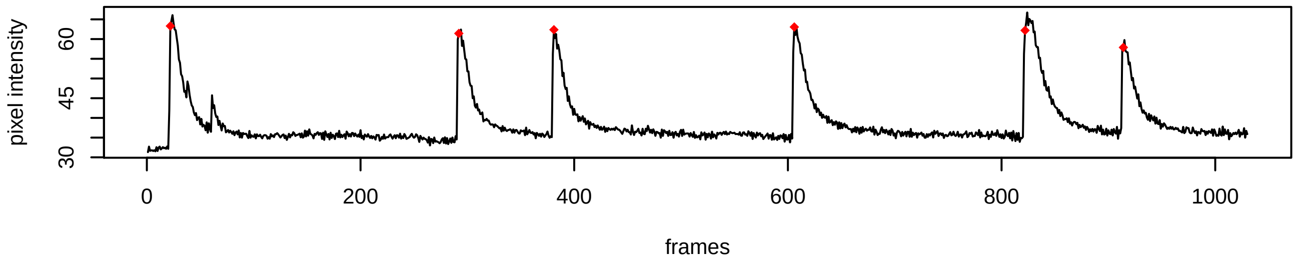

Graph 4 , 14 Total Activity 6 Position in Array 840

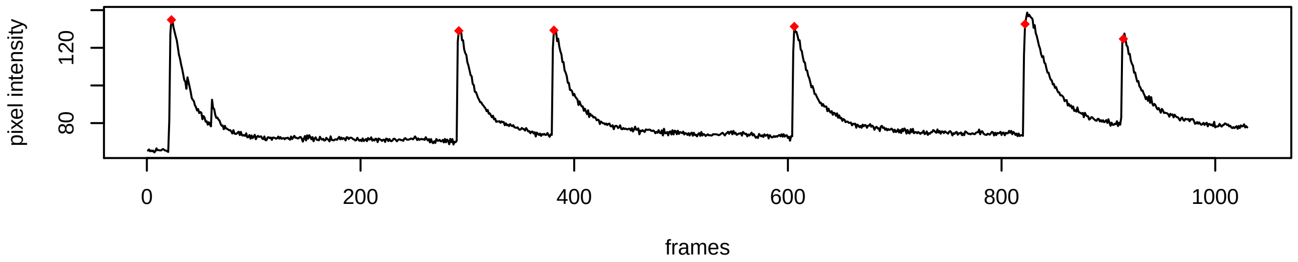

Graph 5 , 14 Total Activity 6 Position in Array 841

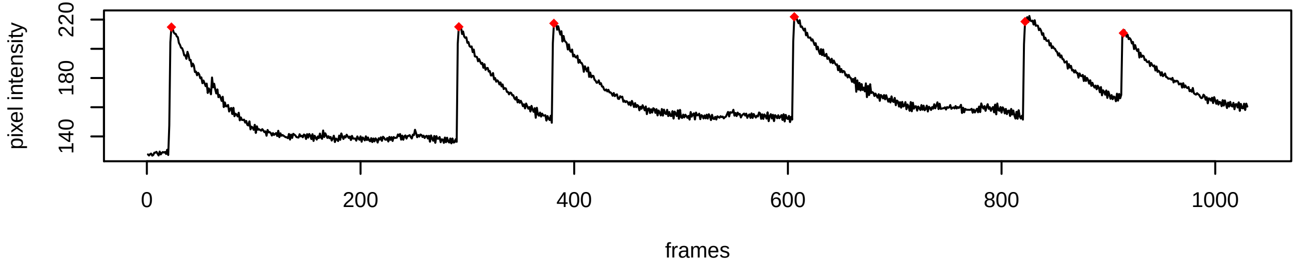

Graph 6 , 14   Total Activity 6   Position in Array 842

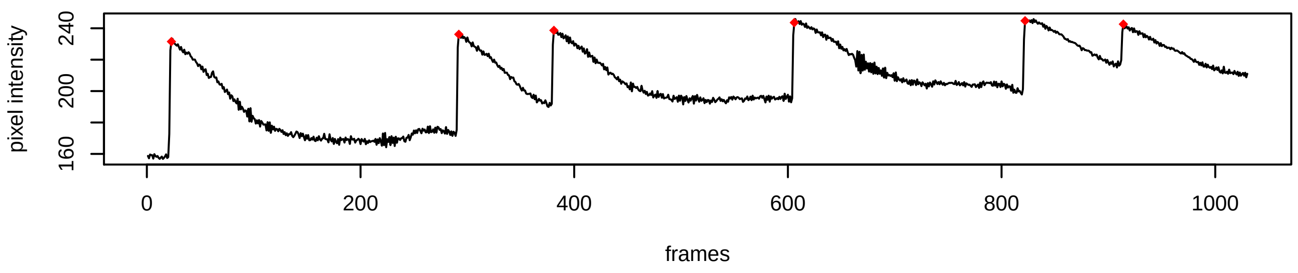

Graph 7 , 14   Total Activity 6   Position in Array 843

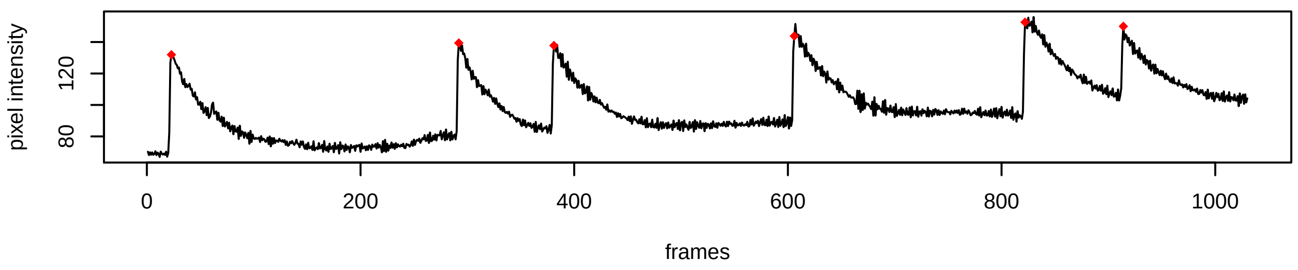

Graph 8 , 14   Total Activity 7   Position in Array 844

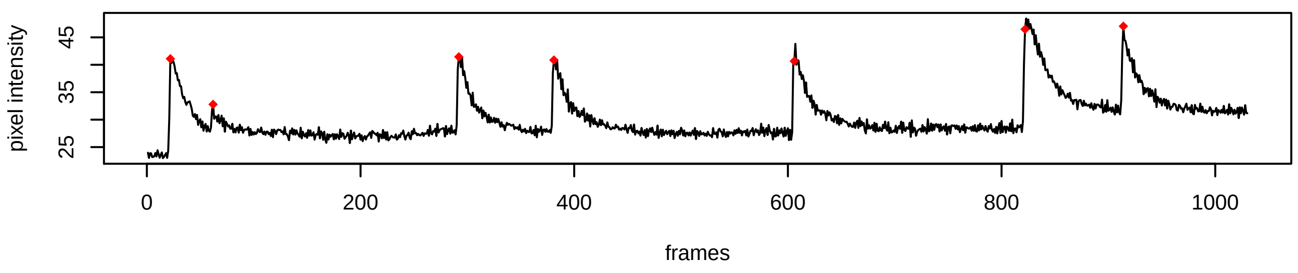

Graph 9 , 14 Total Activity 8 Position in Array 845

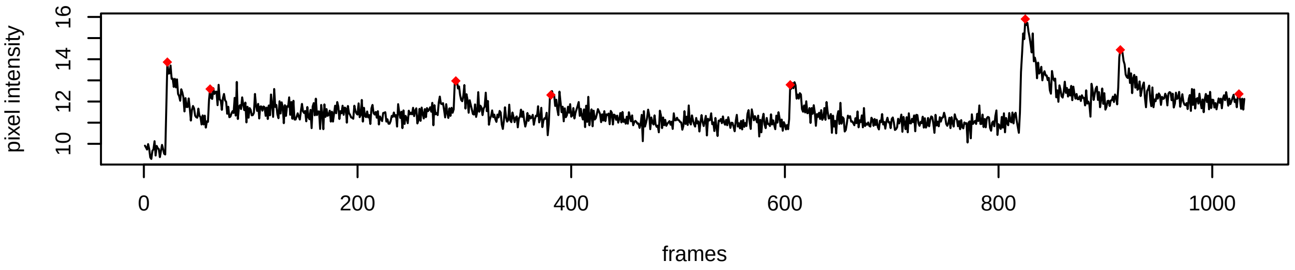

Graph 11 , 14 Total Activity 8 Position in Array 847

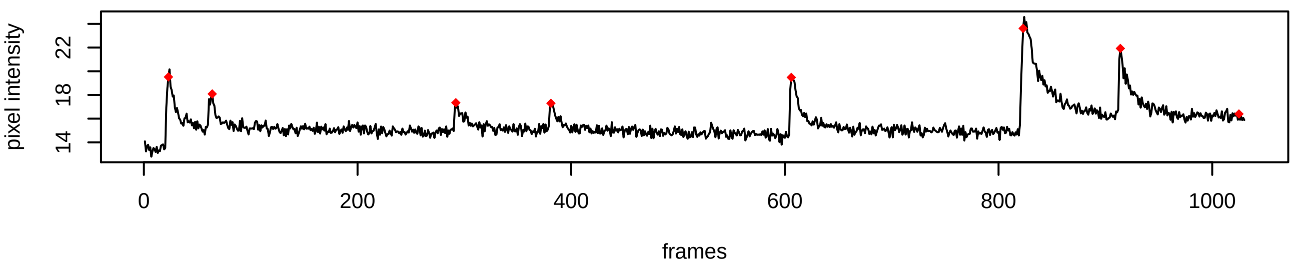

Graph 12 , 14 Total Activity 8 Position in Array 848

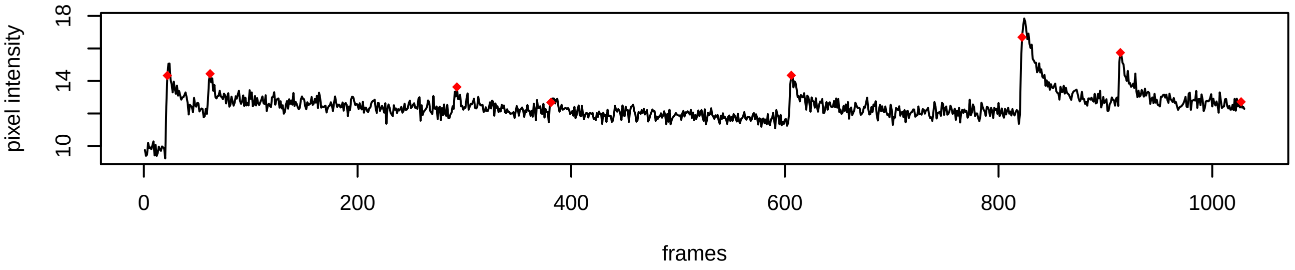

Graph 13 , 14 Total Activity 6 Position in Array 849

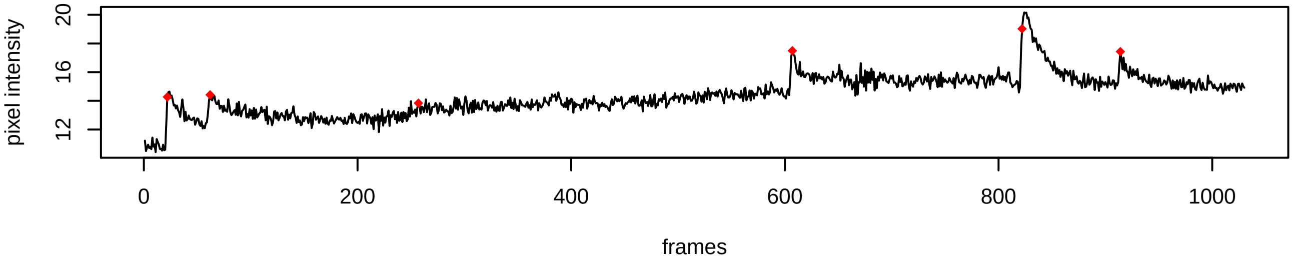

Graph 14 , 14 Total Activity 6 Position in Array 850

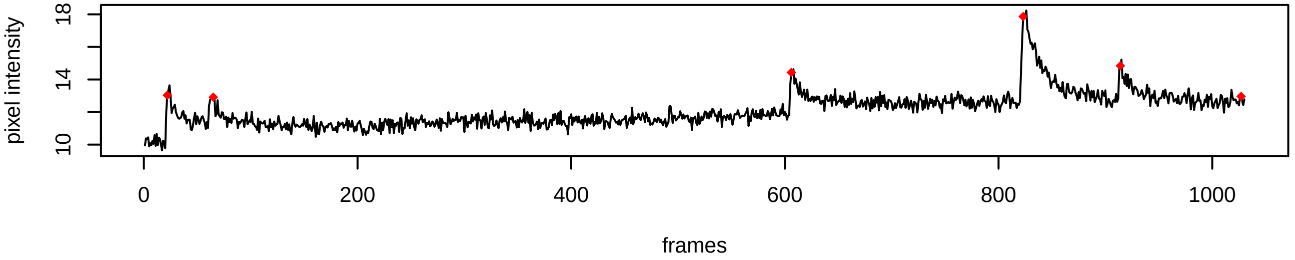

Graph 15 , 14 Total Activity 6 Position in Array 851

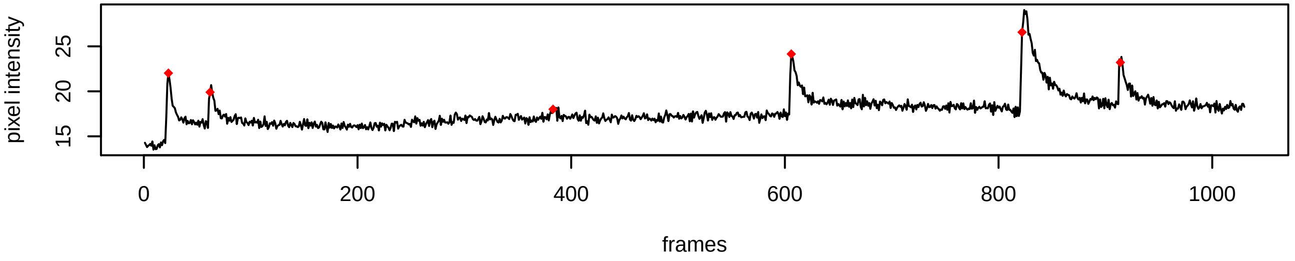

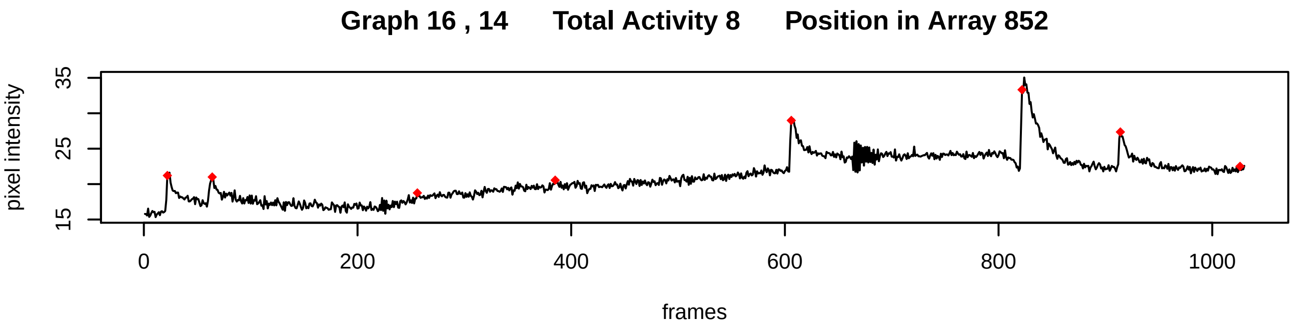
Graph 16 , 14 Total Activity 8 Position in Array 852
pixel intensity
15
25
35
0
200
400
600
800
1000
frames

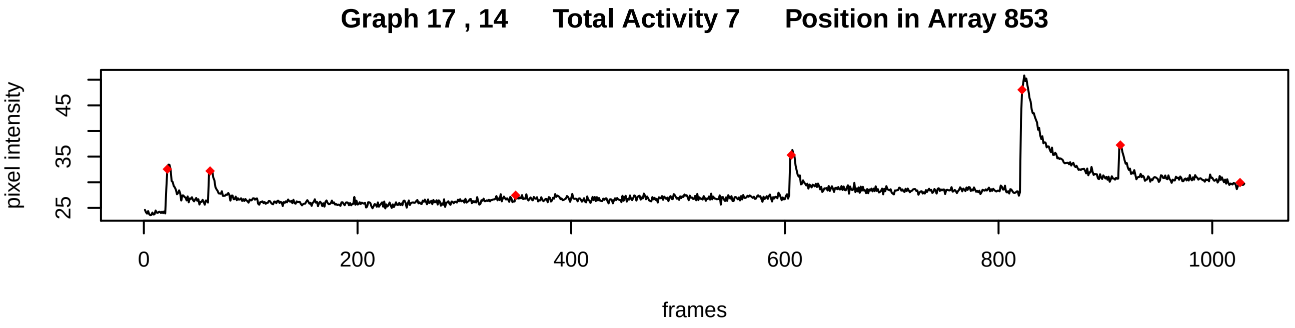
Graph 17 , 14 Total Activity 7 Position in Array 853
pixel intensity
25
35
45
0
200
400
600
800
1000
frames

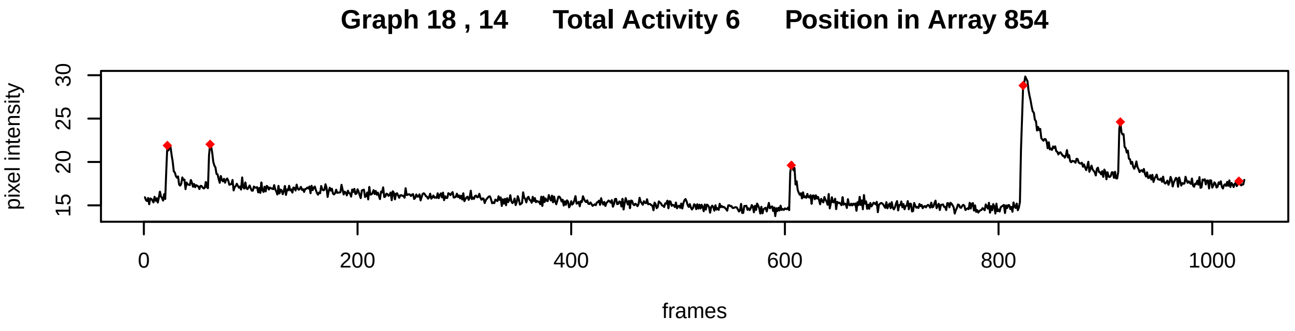
Graph 18 , 14 Total Activity 6 Position in Array 854
pixel intensity
15
20
25
30
0
200
400
600
800
1000
frames

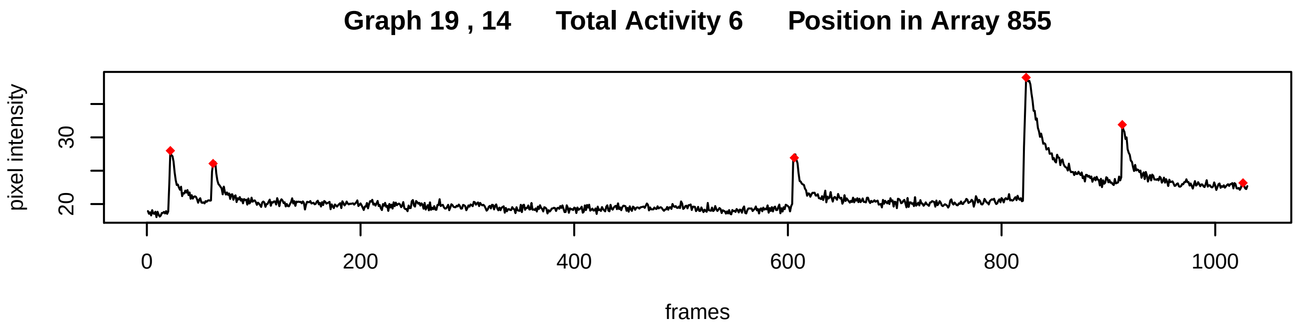
Graph 19 , 14 Total Activity 6 Position in Array 855
pixel intensity
20
30
0
200
400
600
800
1000
frames

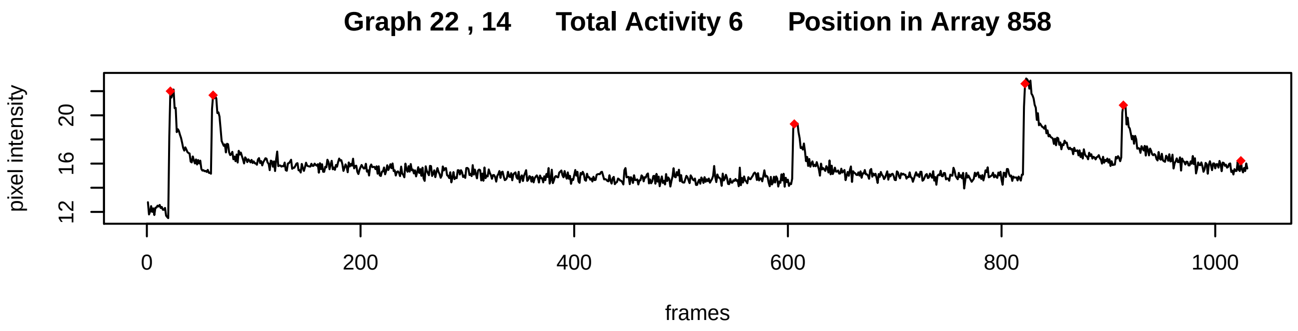
Graph 22 , 14 Total Activity 6 Position in Array 858
pixel intensity
12
16
20
0
200
400
600
800
1000
frames

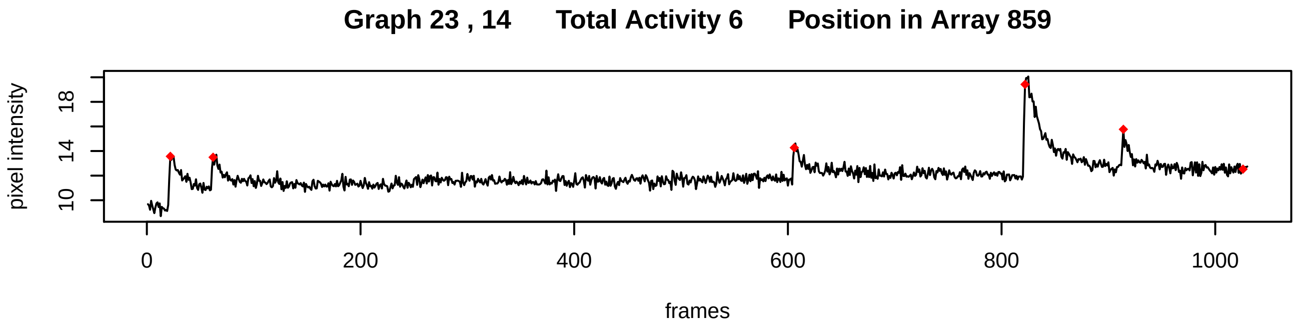
Graph 23 , 14 Total Activity 6 Position in Array 859
pixel intensity
10
14
18
0
200
400
600
800
1000
frames

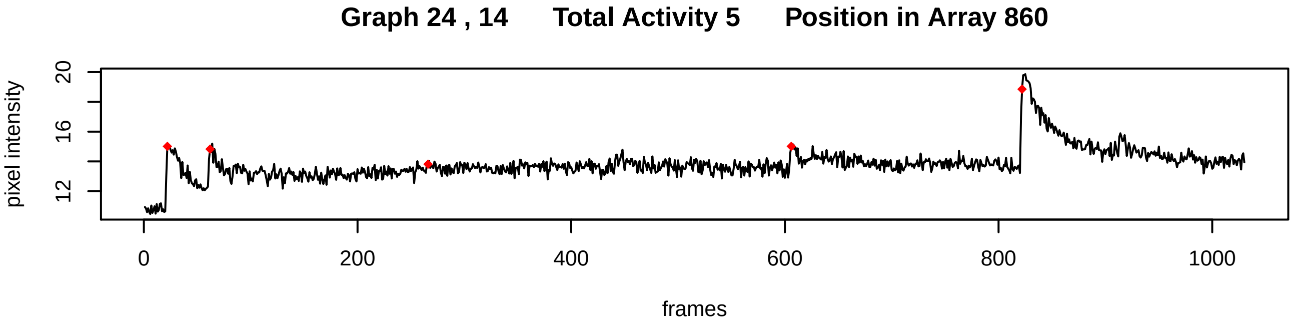
Graph 24 , 14 Total Activity 5 Position in Array 860
pixel intensity
12
16
20
0
200
400
600
800
1000
frames

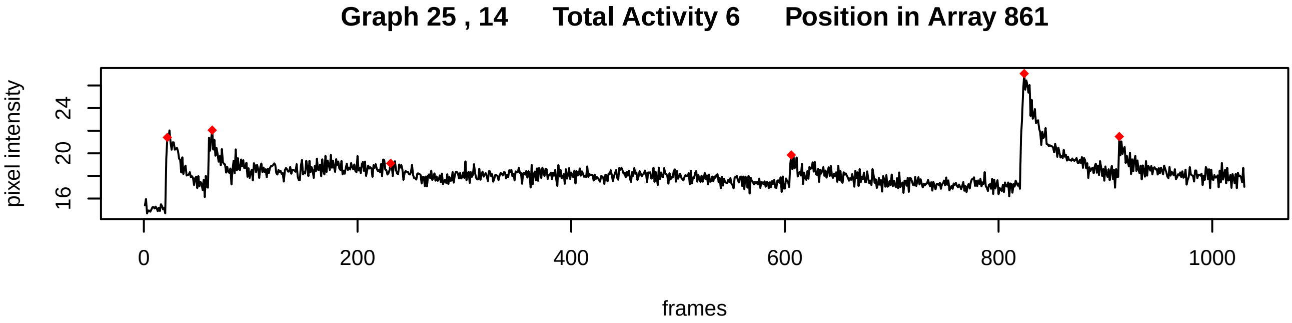
Graph 25 , 14 Total Activity 6 Position in Array 861
pixel intensity
16
20
24
0
200
400
600
800
1000
frames

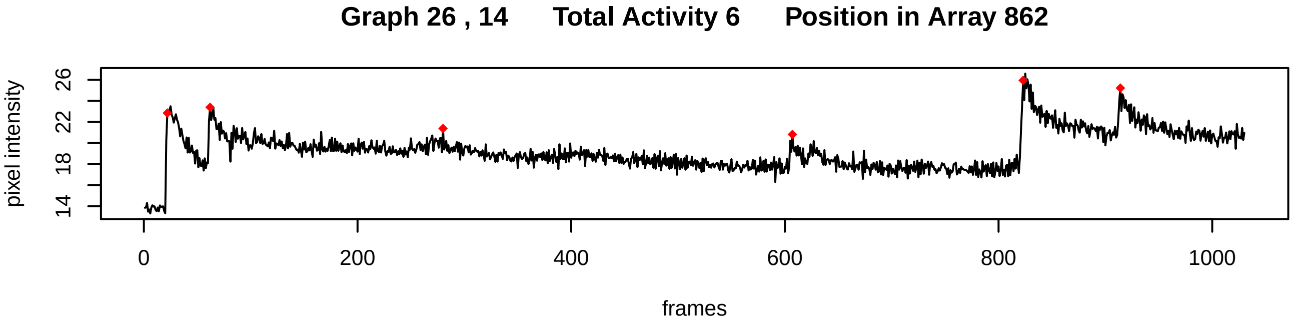
Graph 26 , 14 Total Activity 6 Position in Array 862
pixel intensity
14
18
22
26
0
200
400
600
800
1000
frames

Graph 27 , 14 Total Activity 7 Position in Array 863

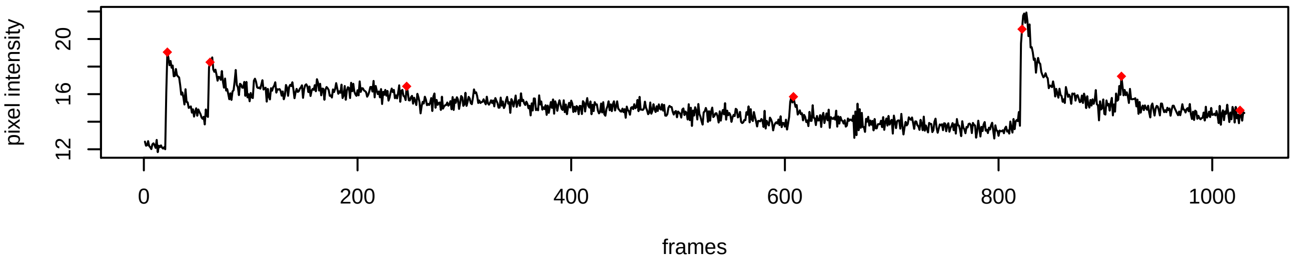

Graph 28 , 14 Total Activity 4 Position in Array 864

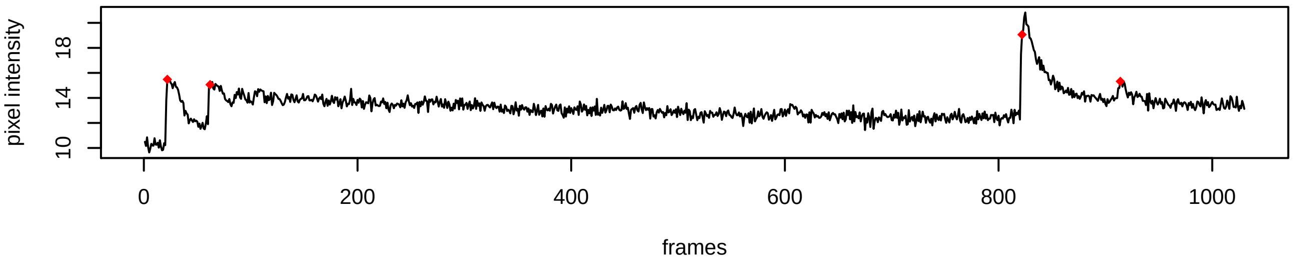

Graph 29 , 14 Total Activity 7 Position in Array 865

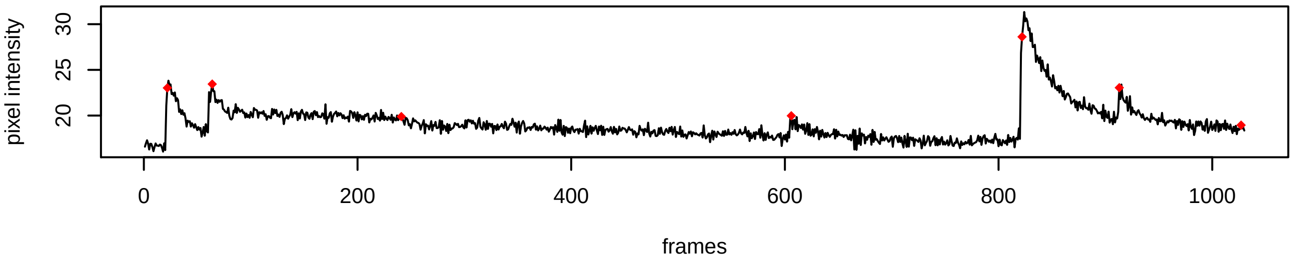

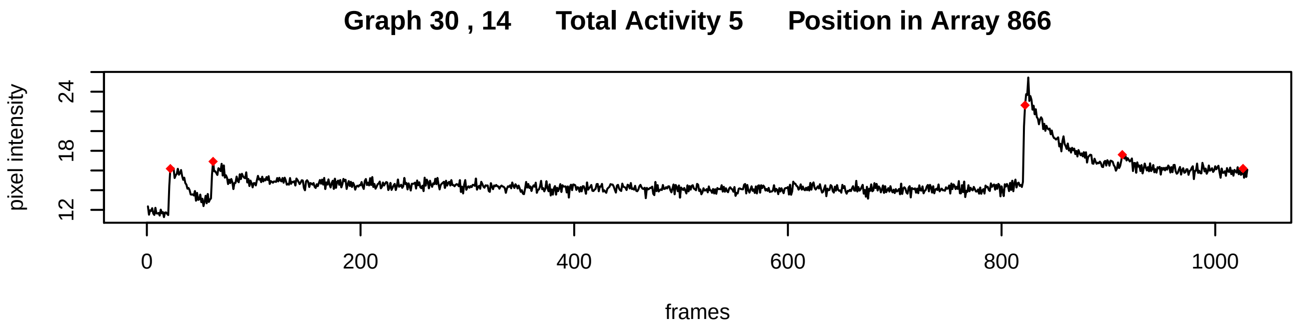
Graph 30 , 14 Total Activity 5 Position in Array 866
pixel intensity
12
18
24
0
200
400
600
800
1000
frames

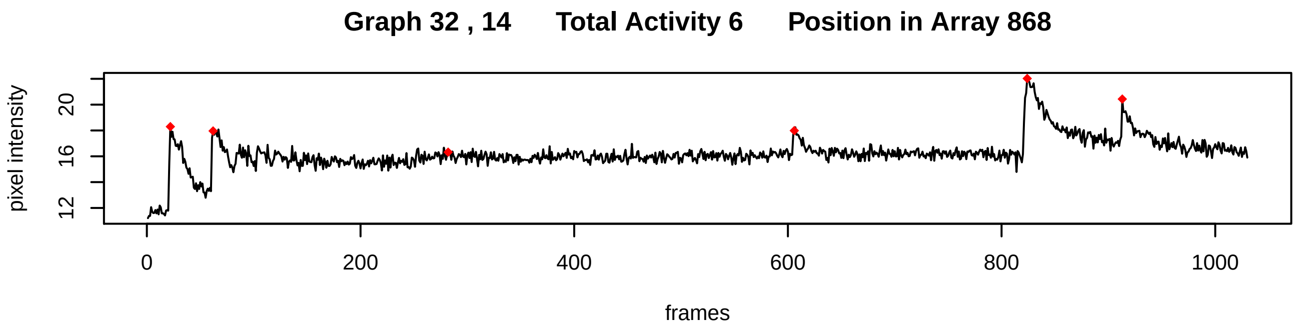
Graph 32 , 14 Total Activity 6 Position in Array 868
pixel intensity
12
16
20
0
200
400
600
800
1000
frames

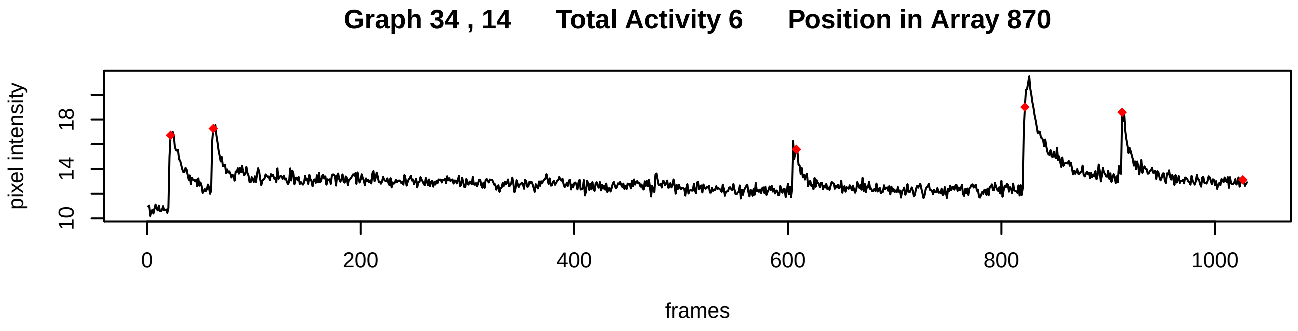
Graph 34 , 14 Total Activity 6 Position in Array 870
pixel intensity
10
14
18
0
200
400
600
800
1000
frames

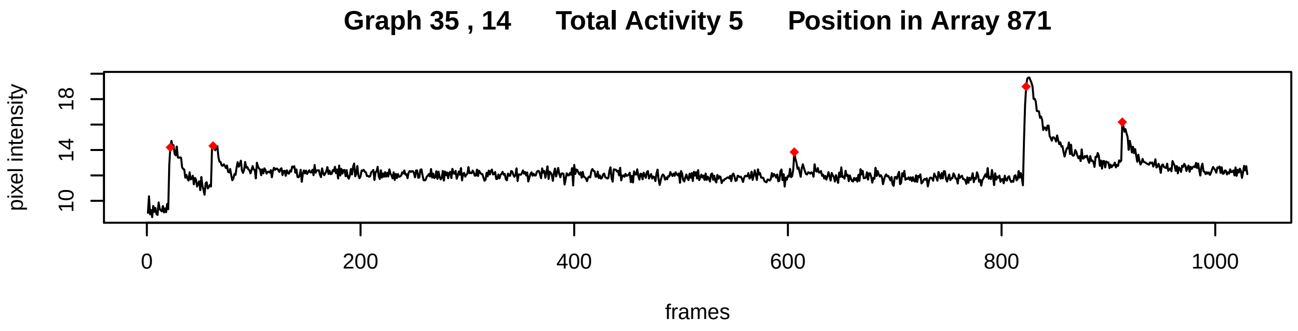
Graph 35 , 14 Total Activity 5 Position in Array 871
pixel intensity
frames
10
14
18
0
200
400
600
800
1000

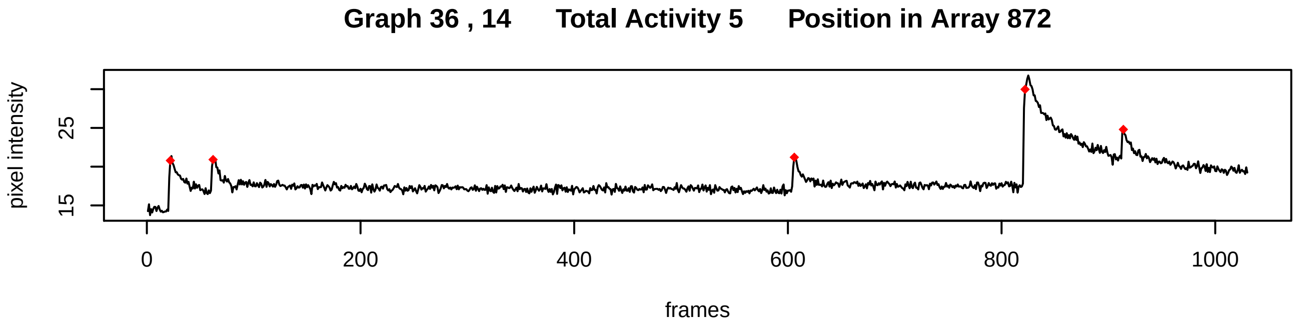
Graph 36 , 14 Total Activity 5 Position in Array 872
pixel intensity
frames
15
25
0
200
400
600
800
1000

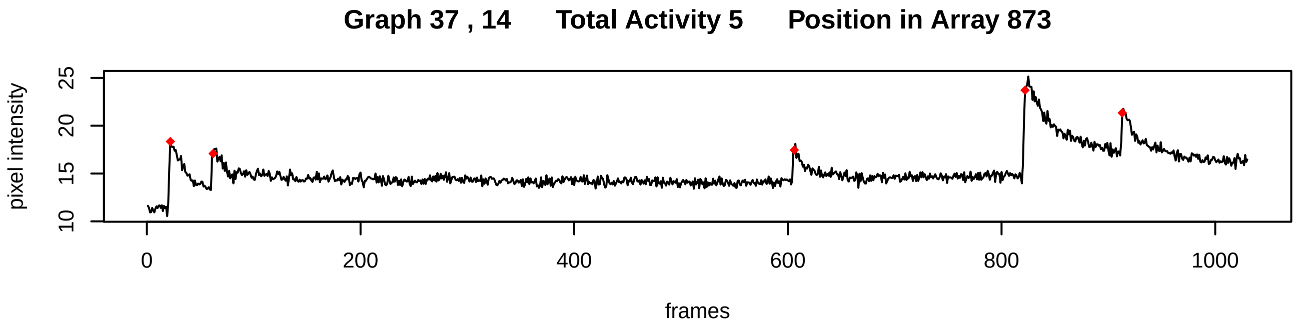
Graph 37 , 14 Total Activity 5 Position in Array 873
pixel intensity
frames
10
15
20
25
0
200
400
600
800
1000

Graph 39 , 14   Total Activity 6   Position in Array 875

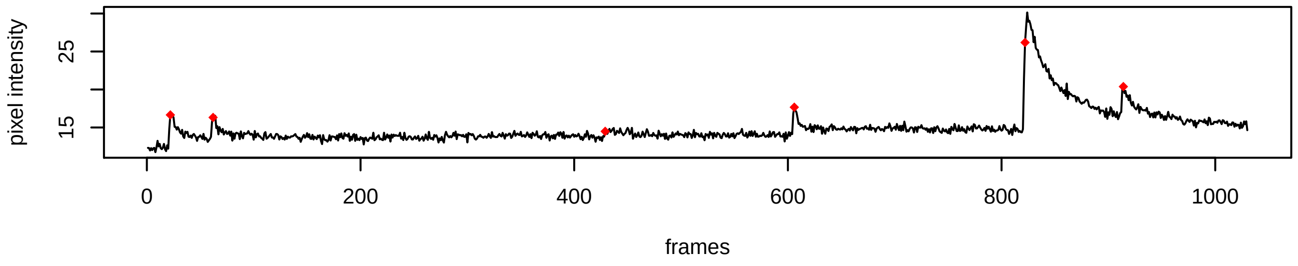

Graph 40 , 14   Total Activity 6   Position in Array 876

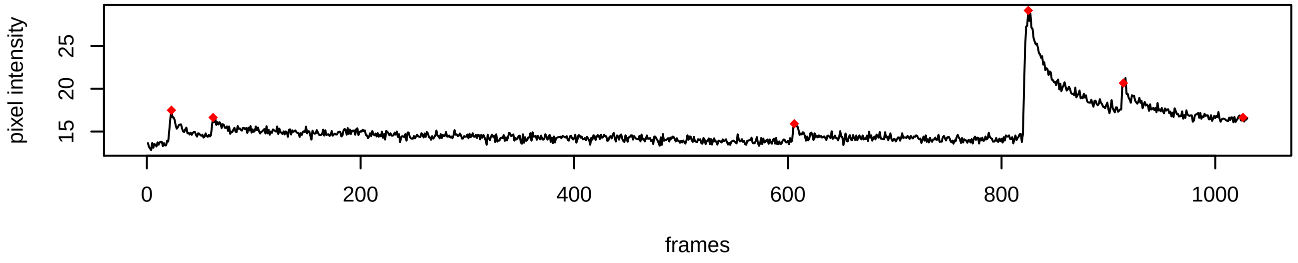

Graph 41 , 14   Total Activity 6   Position in Array 877

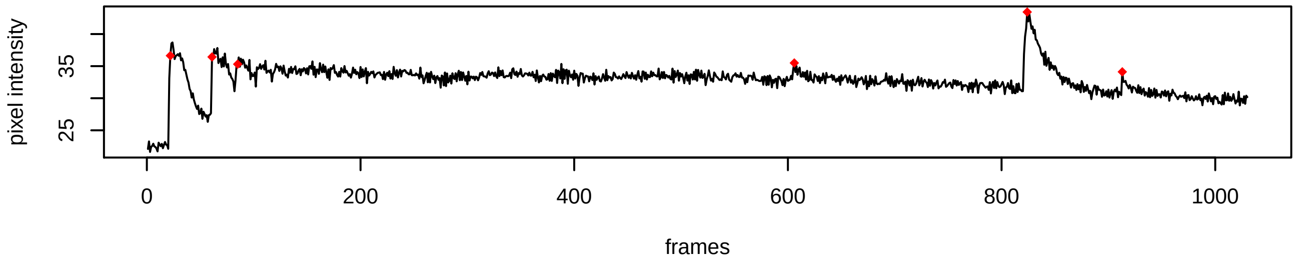

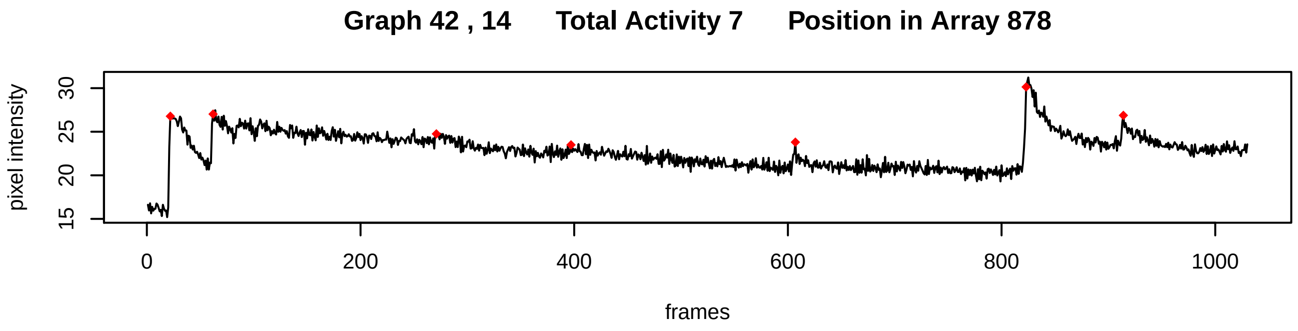

Graph 42 , 14 Total Activity 7 Position in Array 878
pixel intensity
15
20
25
30
0
200
400
600
800
1000
frames

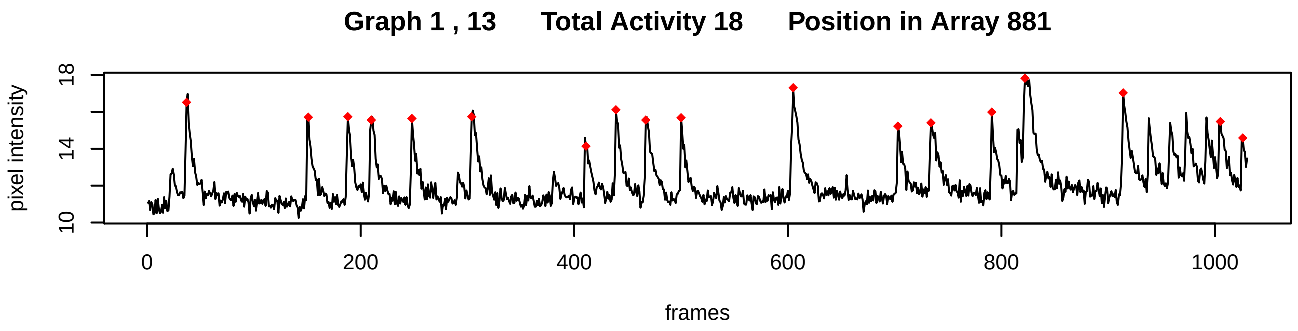

Graph 1 , 13 Total Activity 18 Position in Array 881
pixel intensity
10
14
18
0
200
400
600
800
1000
frames

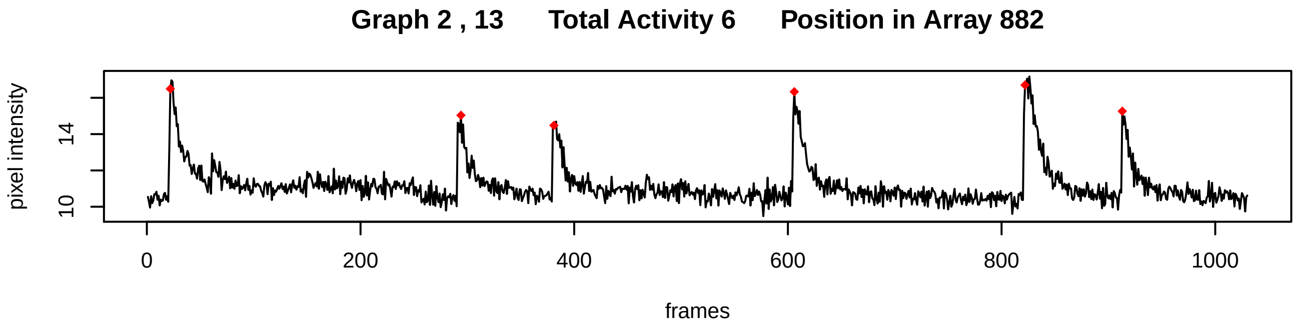

Graph 2 , 13 Total Activity 6 Position in Array 882
pixel intensity
10
14
0
200
400
600
800
1000
frames

Graph 3 , 13 Total Activity 6 Position in Array 883

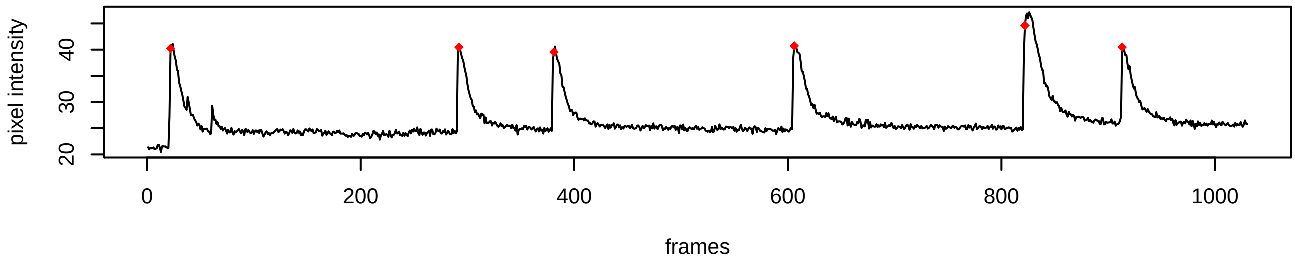

Graph 4 , 13 Total Activity 6 Position in Array 884

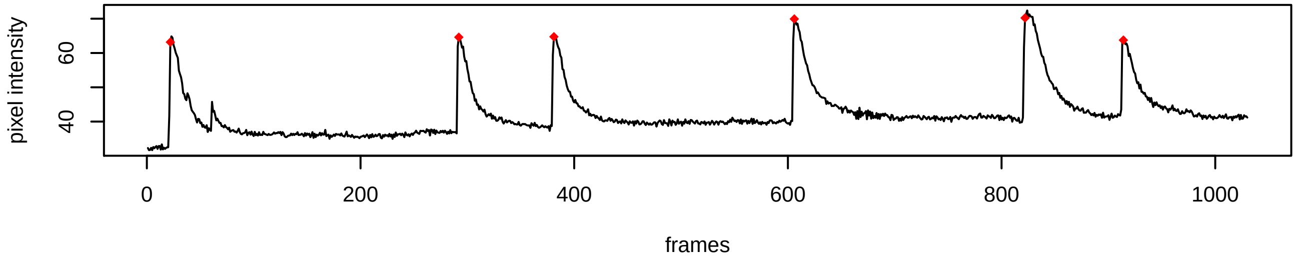

Graph 5 , 13 Total Activity 6 Position in Array 885

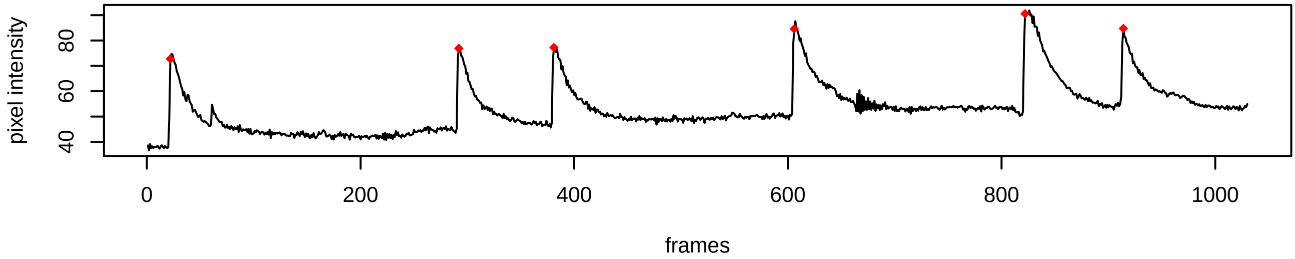

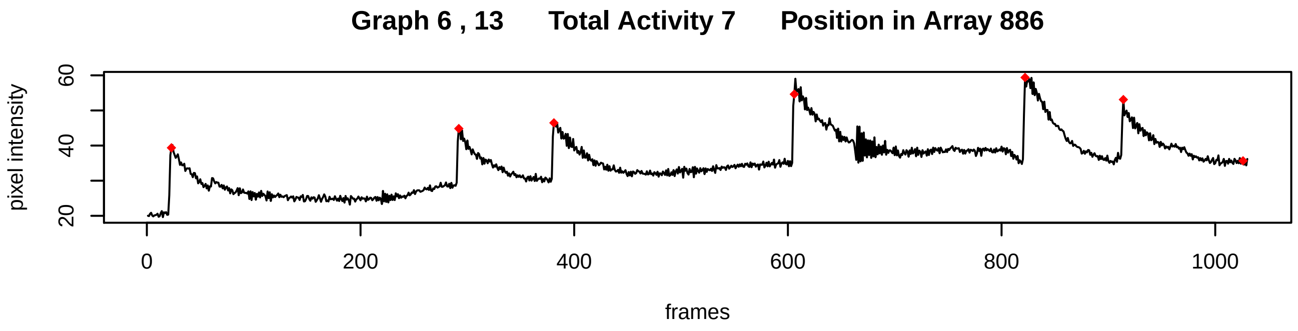
Graph 6 , 13 Total Activity 7 Position in Array 886
pixel intensity
20
40
60
0
200
400
600
800
1000
frames

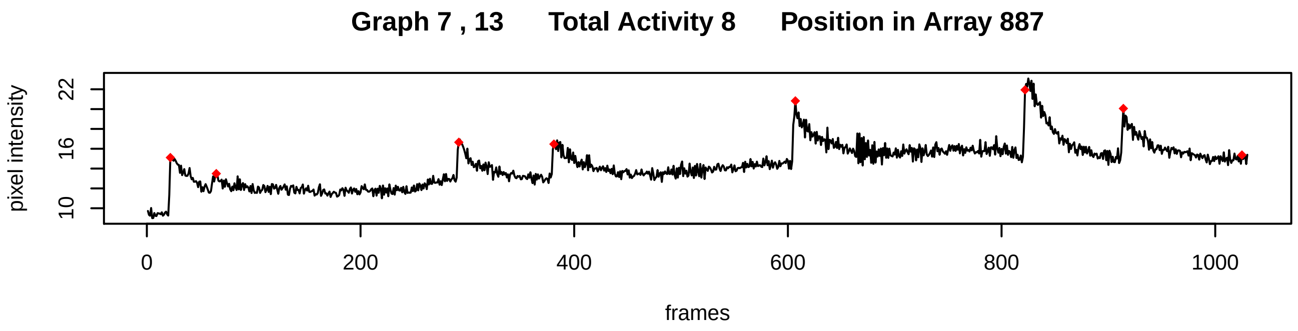
Graph 7 , 13 Total Activity 8 Position in Array 887
pixel intensity
10
16
22
0
200
400
600
800
1000
frames

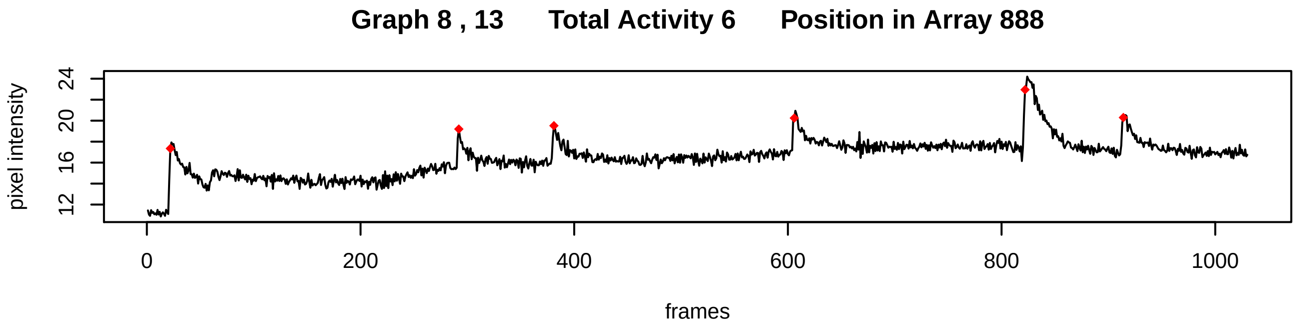
Graph 8 , 13 Total Activity 6 Position in Array 888
pixel intensity
12
16
20
24
0
200
400
600
800
1000
frames

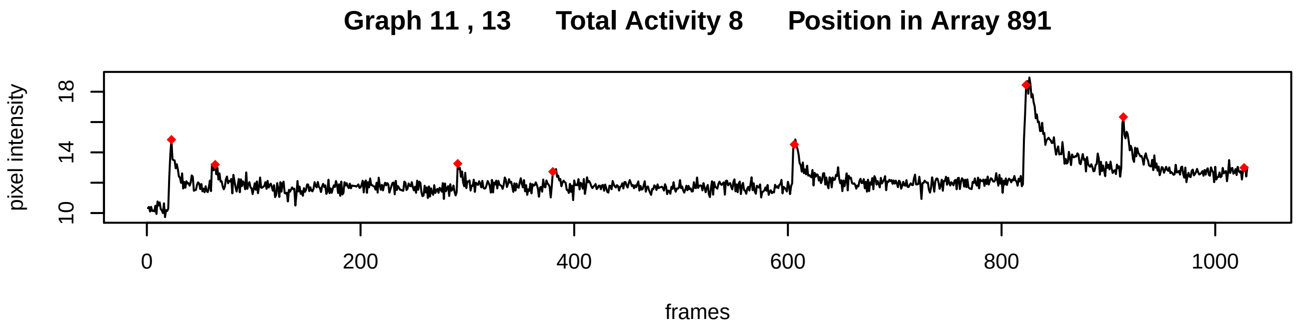
Graph 11 , 13 Total Activity 8 Position in Array 891
pixel intensity
10
14
18
0
200
400
600
800
1000
frames

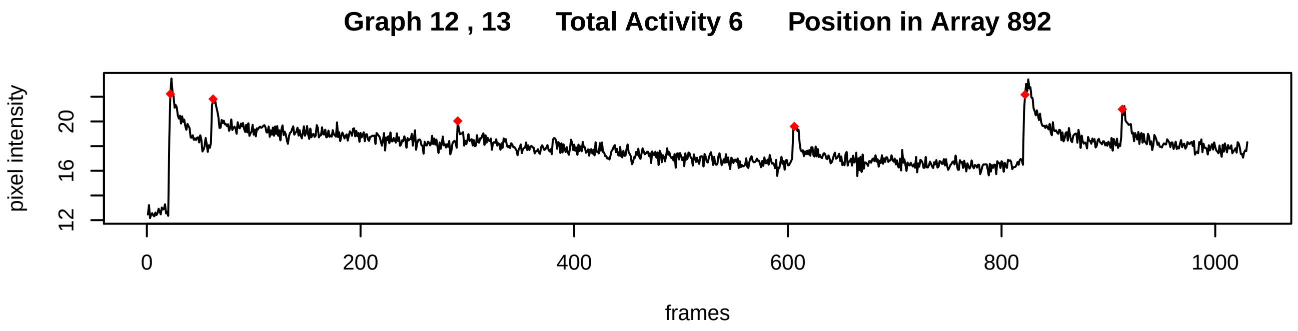
Graph 12 , 13 Total Activity 6 Position in Array 892
pixel intensity
12
16
20
0
200
400
600
800
1000
frames

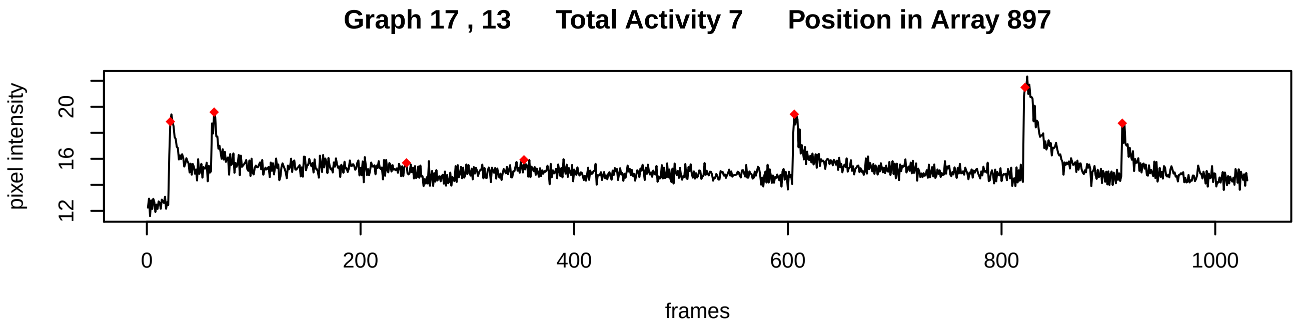
Graph 17 , 13 Total Activity 7 Position in Array 897
pixel intensity
12
16
20
0
200
400
600
800
1000
frames

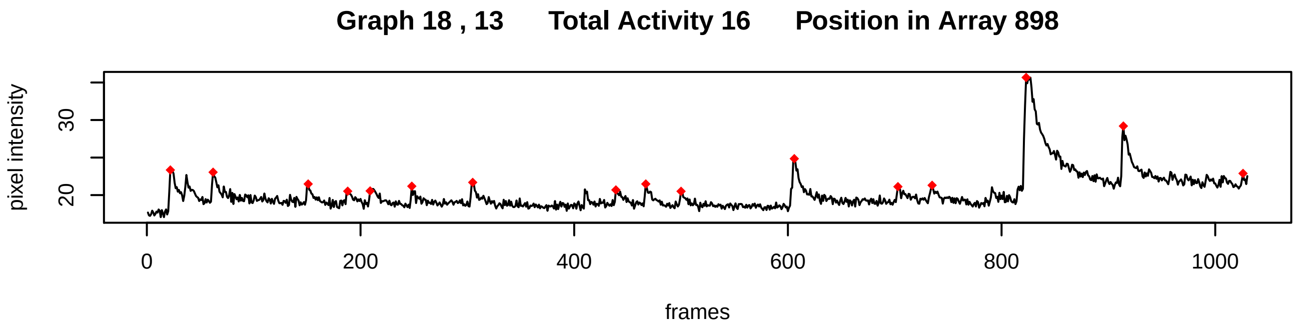
Graph 18 , 13 Total Activity 16 Position in Array 898
pixel intensity
20
30
0
200
400
600
800
1000
frames

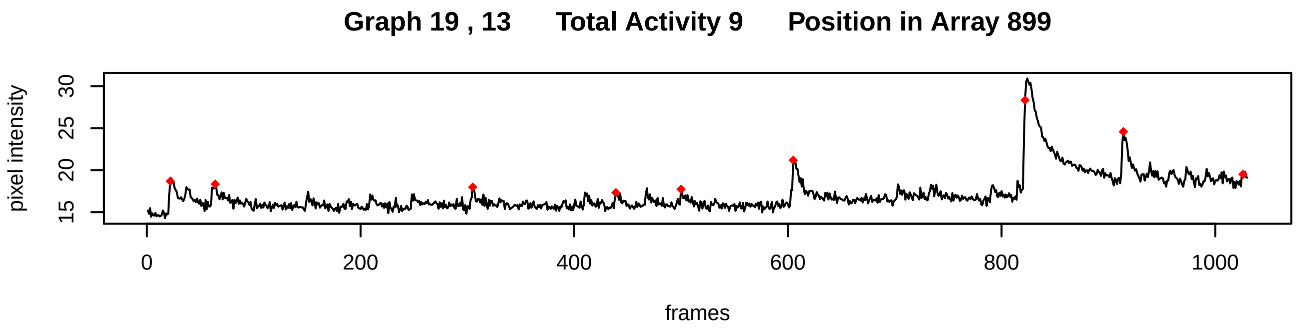
Graph 19 , 13 Total Activity 9 Position in Array 899
pixel intensity
15
20
25
30
0
200
400
600
800
1000
frames

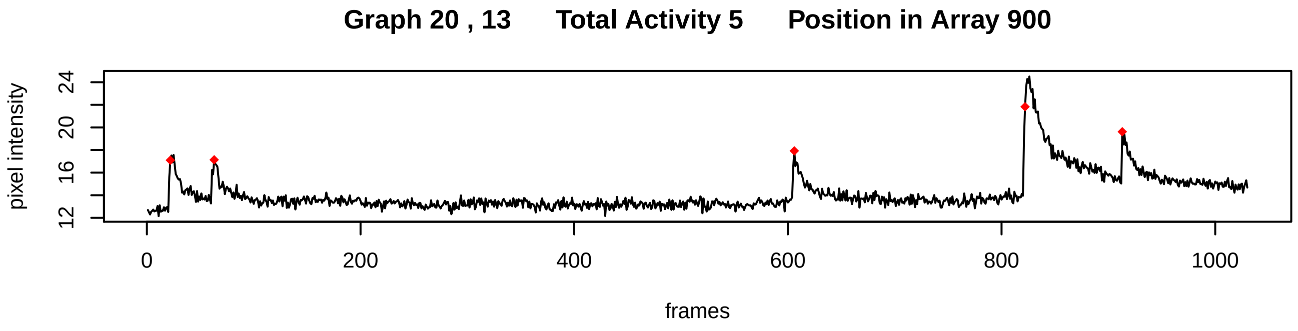
Graph 20 , 13 Total Activity 5 Position in Array 900
pixel intensity
12
16
20
24
0
200
400
600
800
1000
frames

Graph 21 , 13   Total Activity 6   Position in Array 901

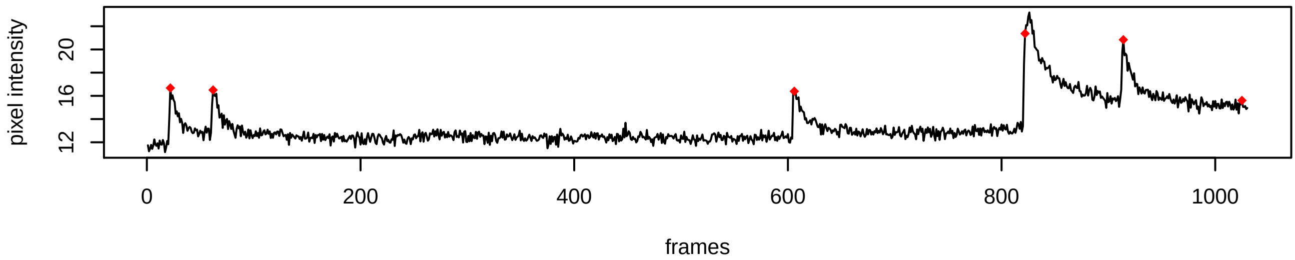

Graph 22 , 13   Total Activity 6   Position in Array 902

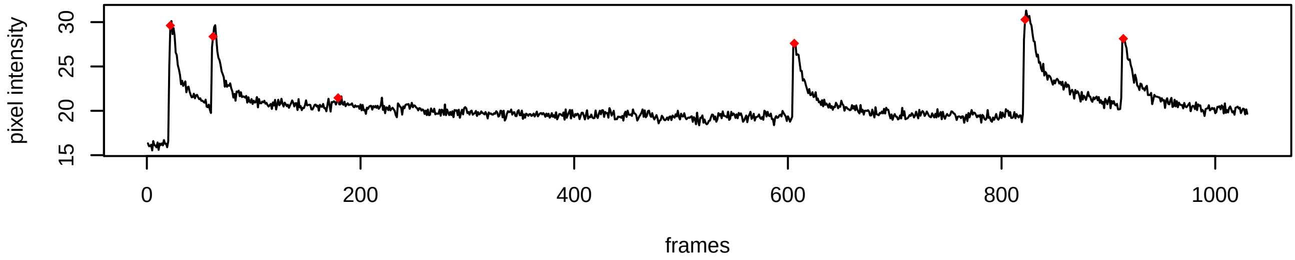

Graph 25 , 13   Total Activity 8   Position in Array 905

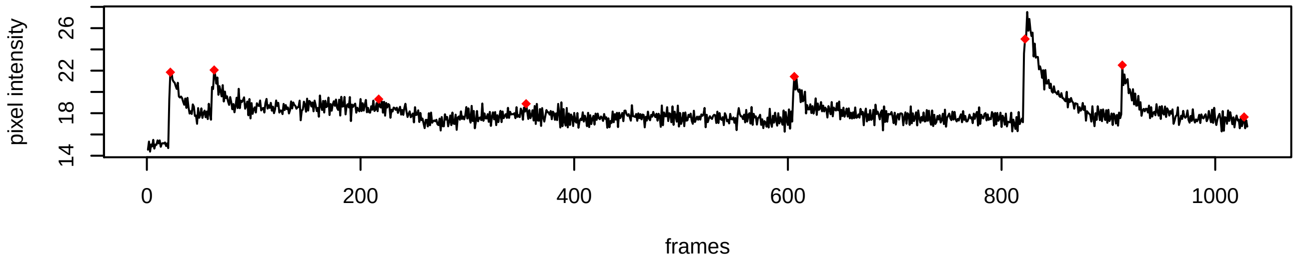

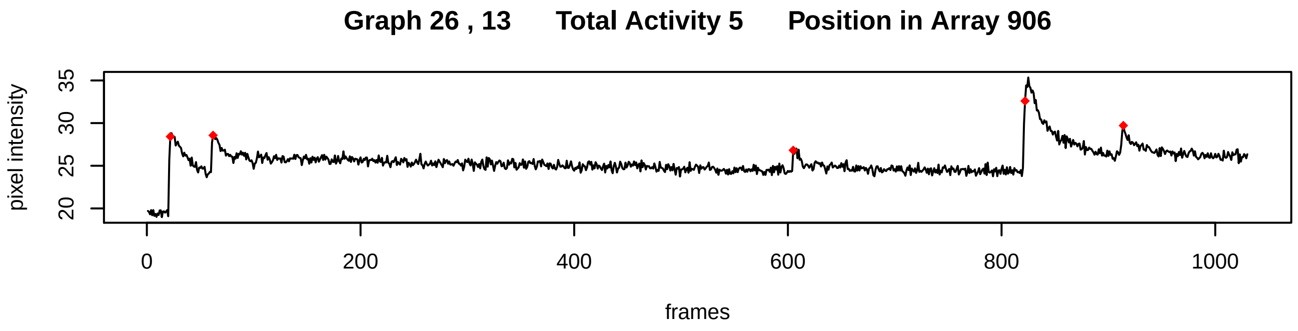
Graph 26 , 13 Total Activity 5 Position in Array 906
pixel intensity
20
25
30
35
0
200
400
600
800
1000
frames

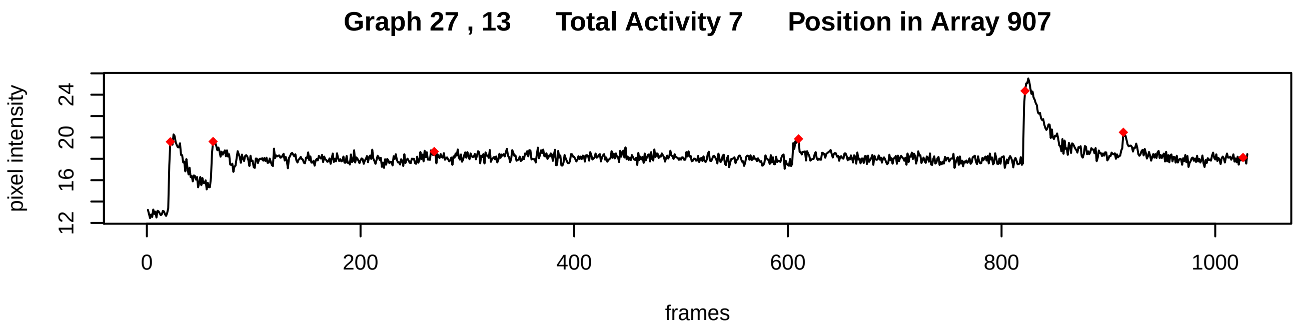
Graph 27 , 13 Total Activity 7 Position in Array 907
pixel intensity
12
16
20
24
0
200
400
600
800
1000
frames

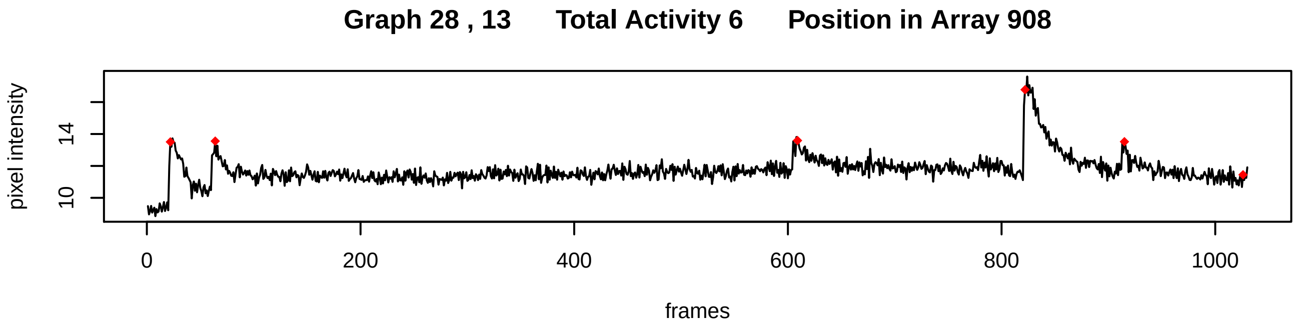
Graph 28 , 13 Total Activity 6 Position in Array 908
pixel intensity
10
14
0
200
400
600
800
1000
frames

Graph 29 , 13   Total Activity 6   Position in Array 909

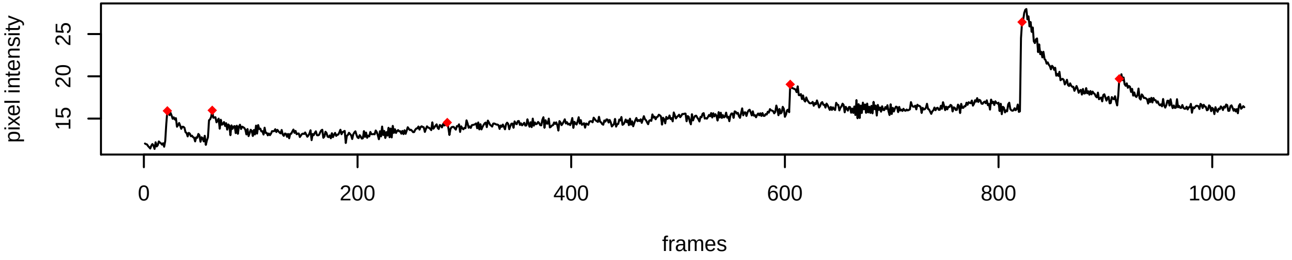

Graph 30 , 13   Total Activity 6   Position in Array 910

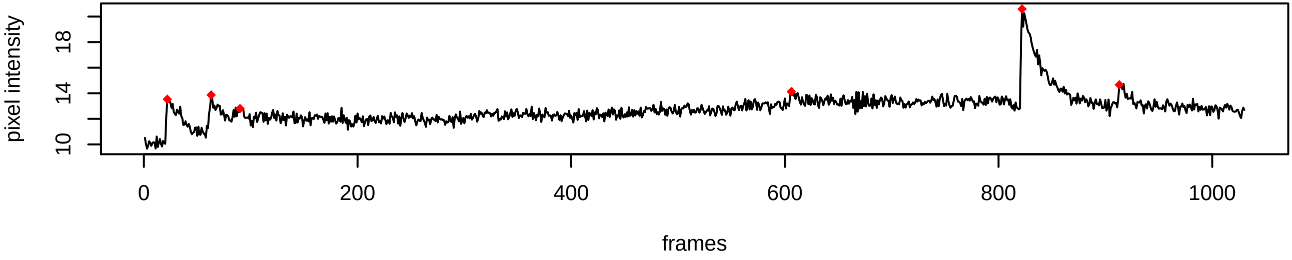

Graph 31 , 13   Total Activity 7   Position in Array 911

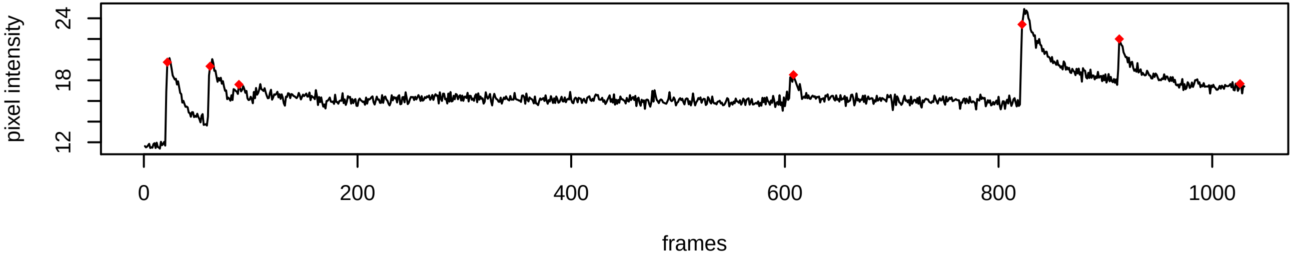

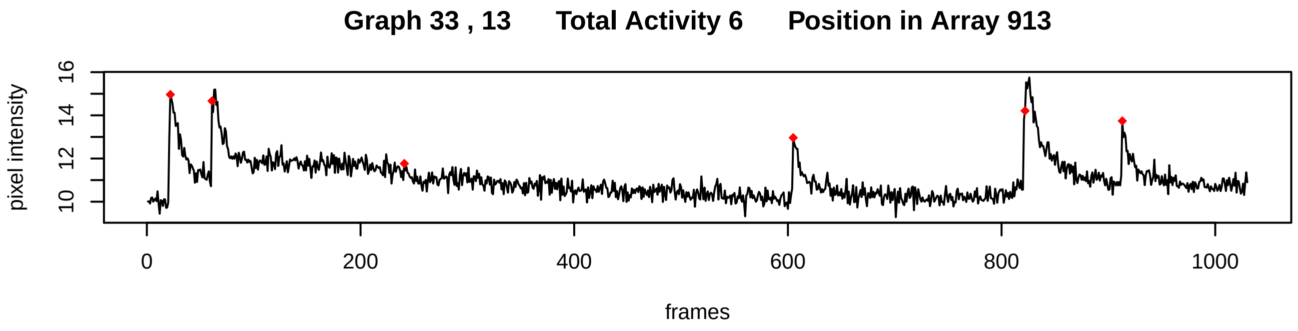
Graph 33 , 13 Total Activity 6 Position in Array 913
pixel intensity
10
12
14
16
0
200
400
600
800
1000
frames

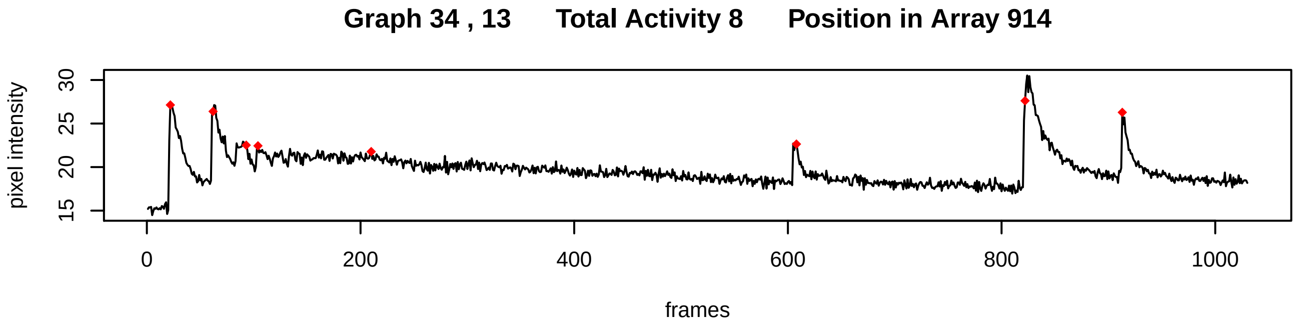
Graph 34 , 13 Total Activity 8 Position in Array 914
pixel intensity
15
20
25
30
0
200
400
600
800
1000
frames

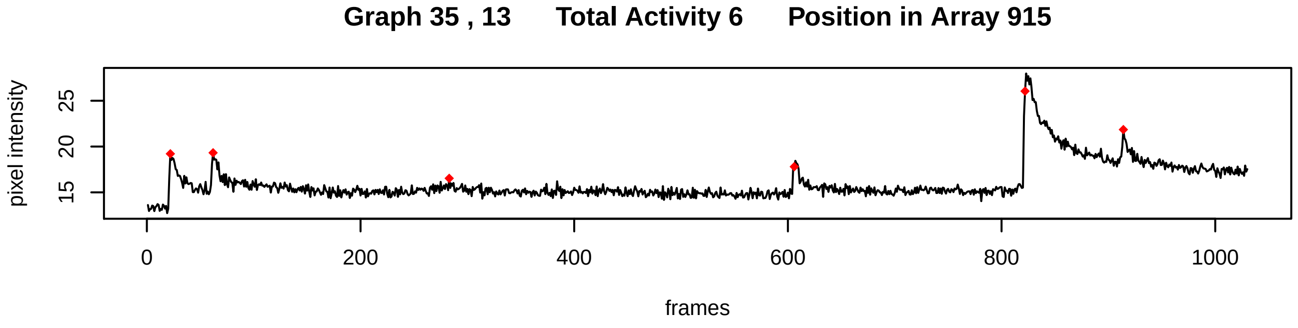
Graph 35 , 13 Total Activity 6 Position in Array 915
pixel intensity
15
20
25
0
200
400
600
800
1000
frames

Graph 37 , 13 Total Activity 5 Position in Array 917

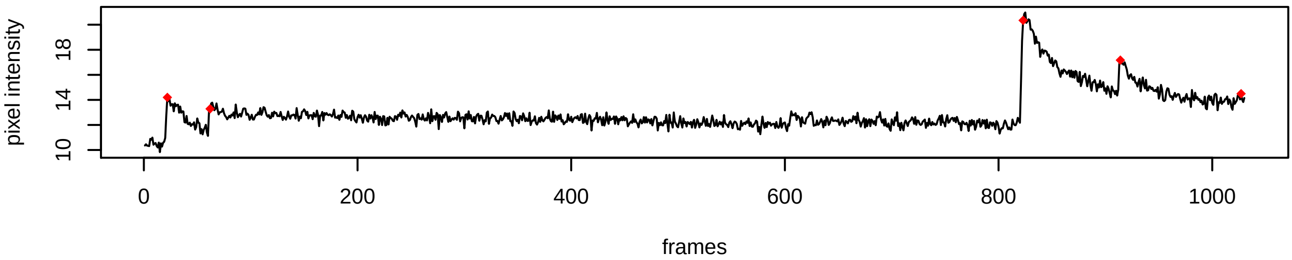

Graph 40 , 13 Total Activity 7 Position in Array 920

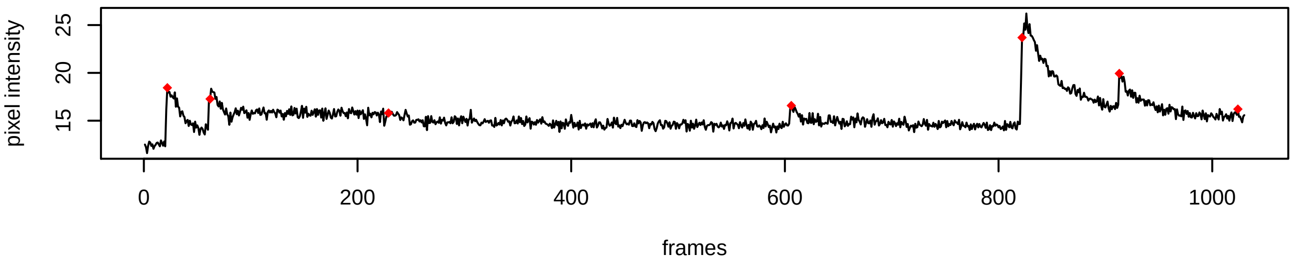

Graph 41 , 13 Total Activity 4 Position in Array 921

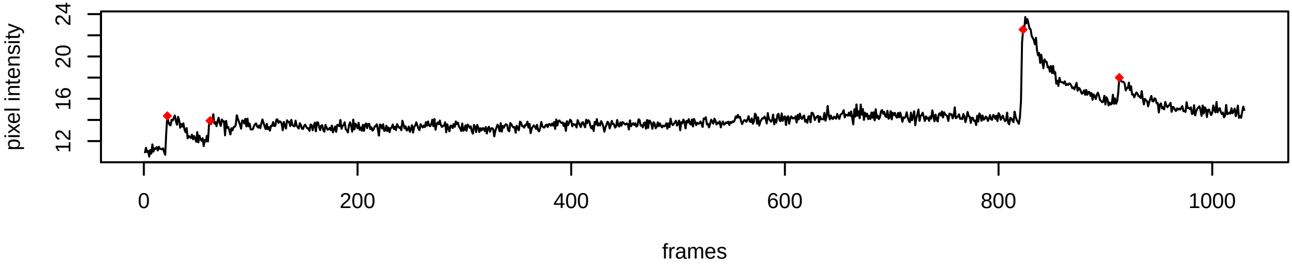

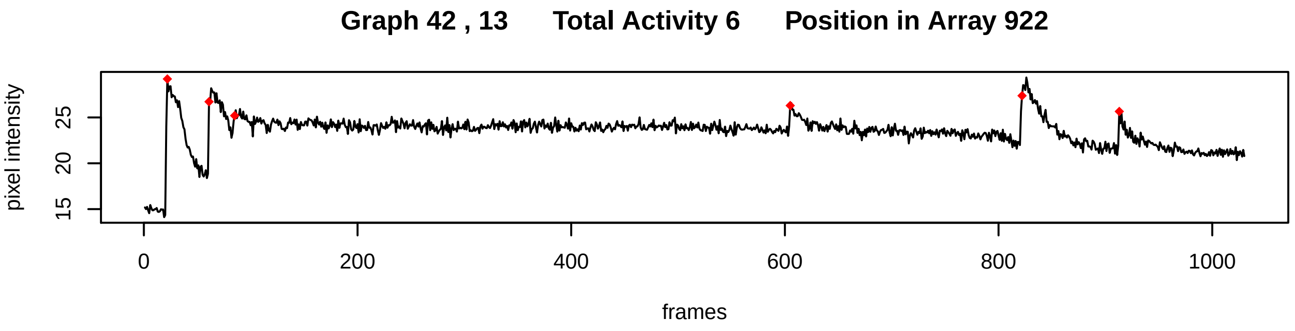
Graph 42 , 13 Total Activity 6 Position in Array 922
pixel intensity
15
20
25
0
200
400
600
800
1000
frames

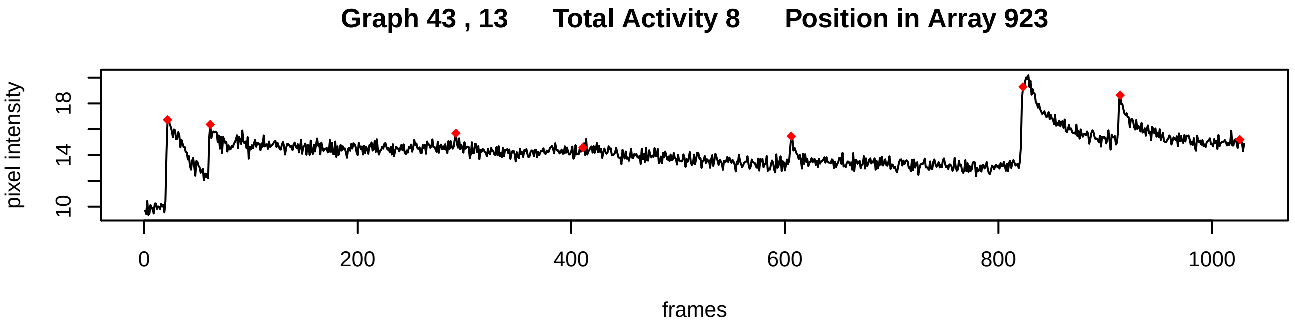
Graph 43 , 13 Total Activity 8 Position in Array 923
pixel intensity
10
14
18
0
200
400
600
800
1000
frames

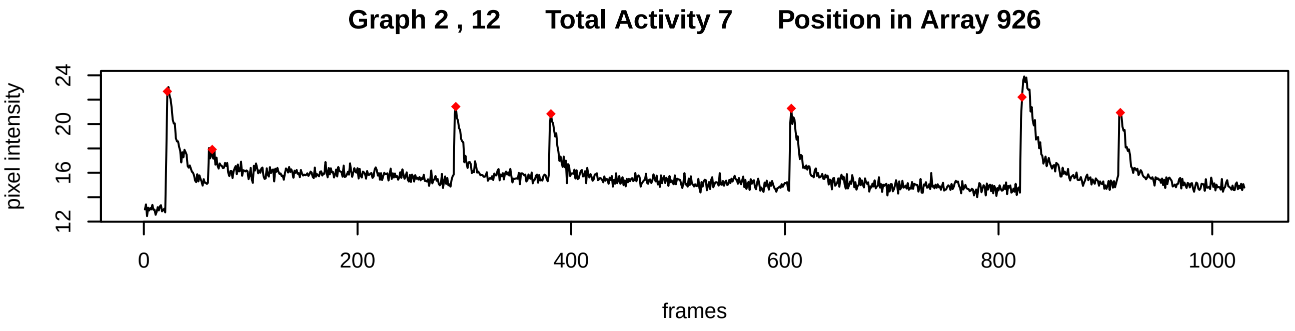
Graph 2 , 12 Total Activity 7 Position in Array 926
pixel intensity
12
16
20
24
0
200
400
600
800
1000
frames

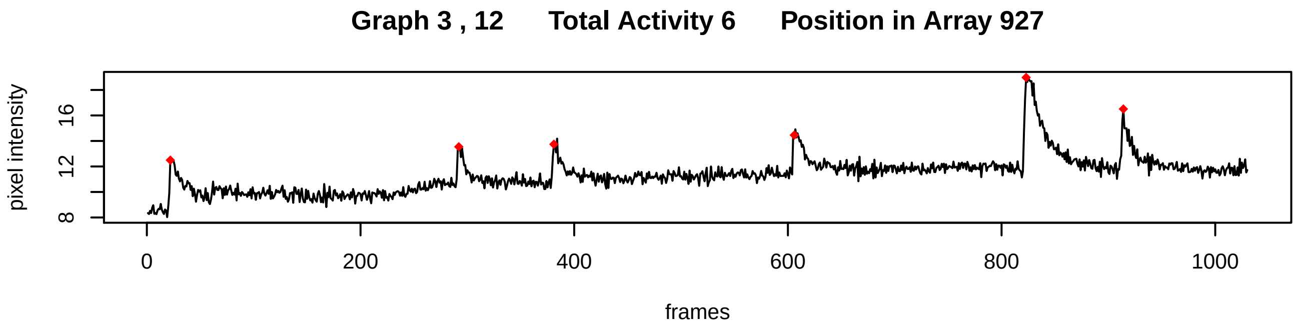
Graph 3 , 12 Total Activity 6 Position in Array 927
pixel intensity
8
12
16
0
200
400
600
800
1000
frames

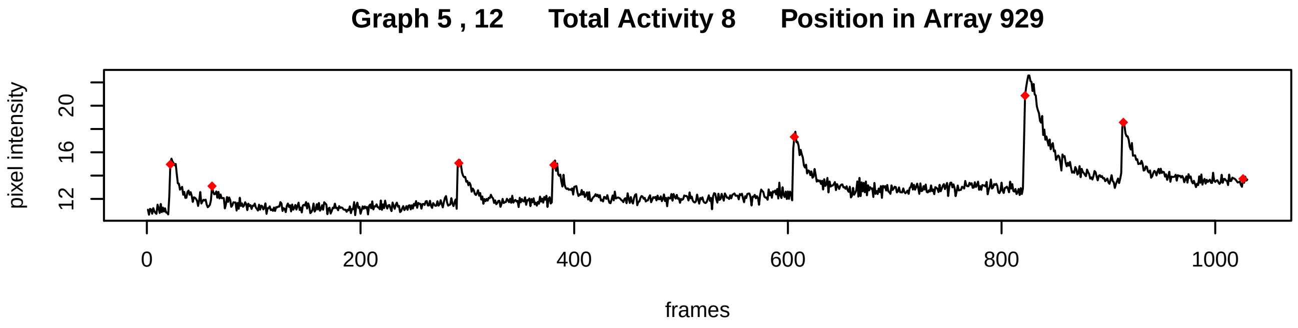
Graph 5 , 12 Total Activity 8 Position in Array 929
pixel intensity
12
16
20
0
200
400
600
800
1000
frames

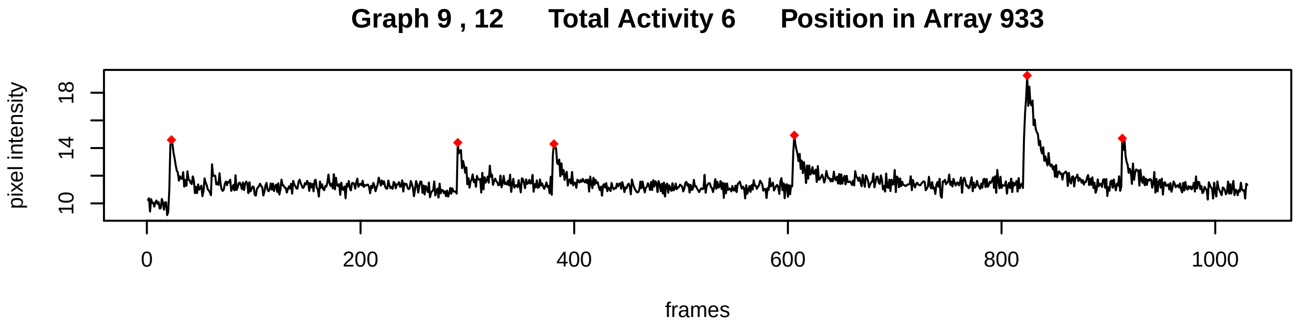
Graph 9 , 12 Total Activity 6 Position in Array 933
pixel intensity
10
14
18
0
200
400
600
800
1000
frames

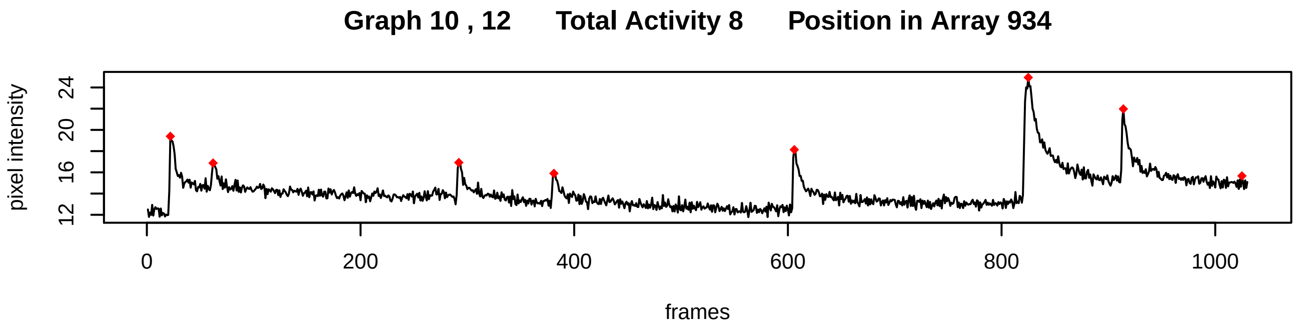
Graph 10 , 12 Total Activity 8 Position in Array 934
pixel intensity
12
16
20
24
0
200
400
600
800
1000
frames

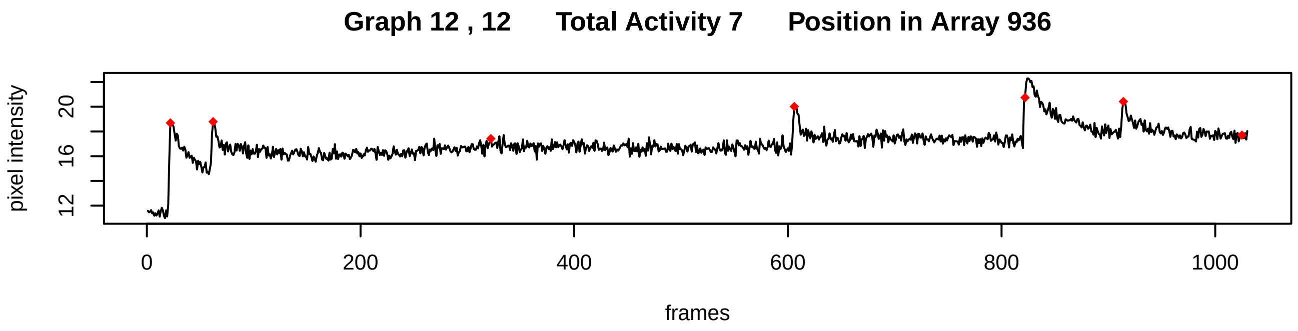
Graph 12 , 12 Total Activity 7 Position in Array 936
pixel intensity
12
16
20
0
200
400
600
800
1000
frames

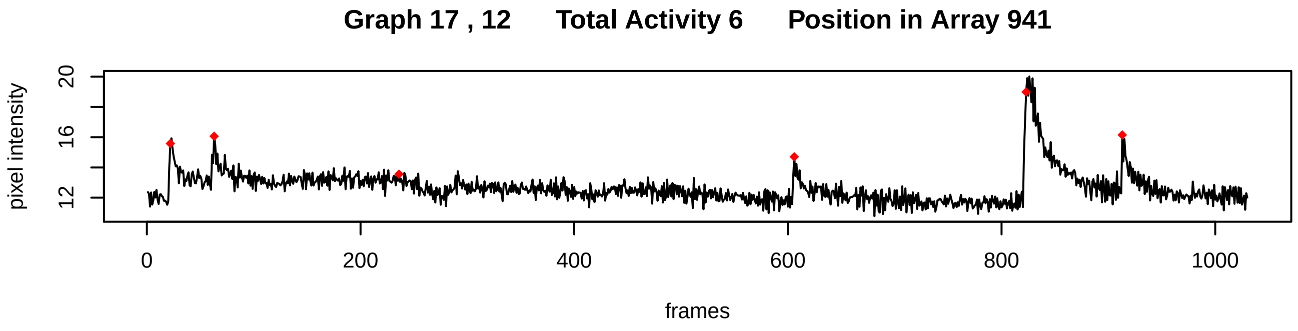
Graph 17 , 12 Total Activity 6 Position in Array 941
pixel intensity
12
16
20
0
200
400
600
800
1000
frames

Graph 18 , 12 Total Activity 7 Position in Array 942

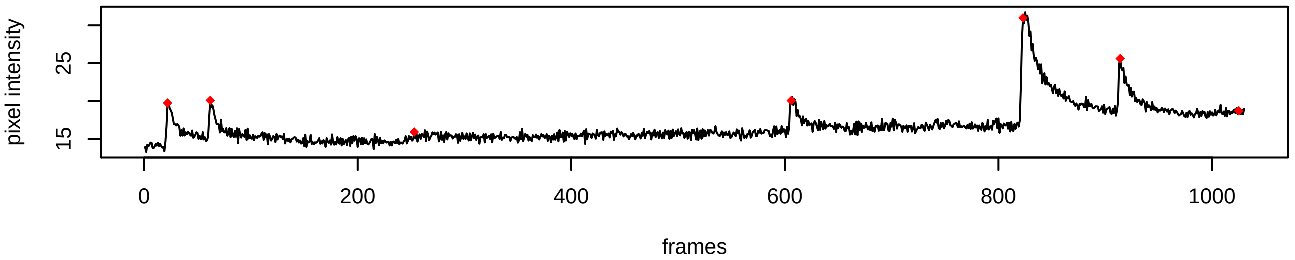

Graph 19 , 12 Total Activity 6 Position in Array 943

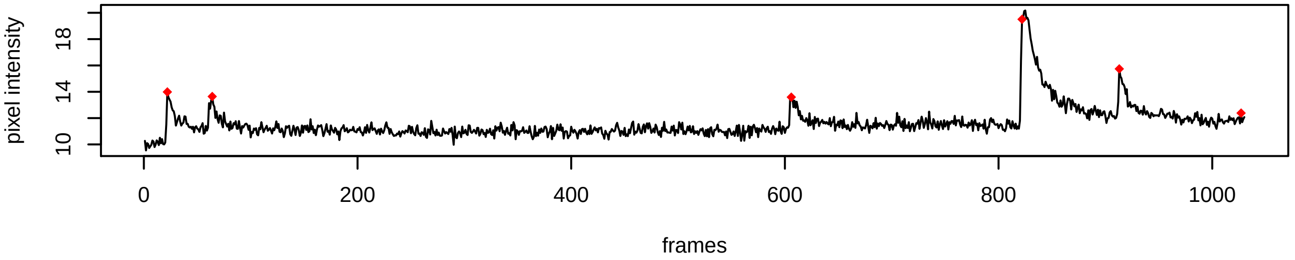

Graph 20 , 12 Total Activity 5 Position in Array 944

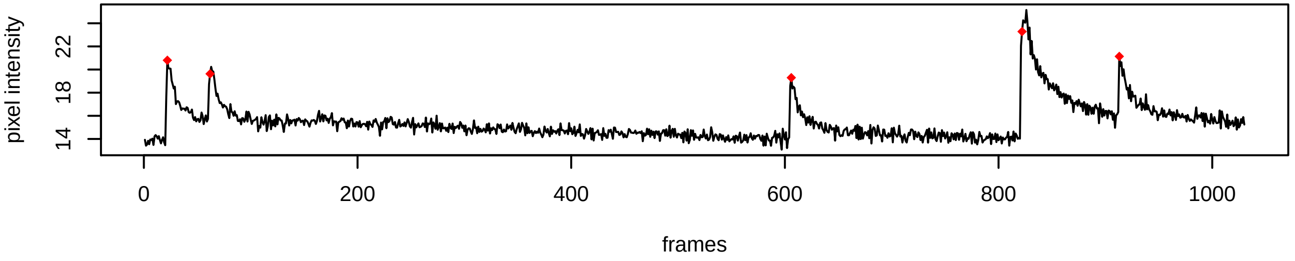

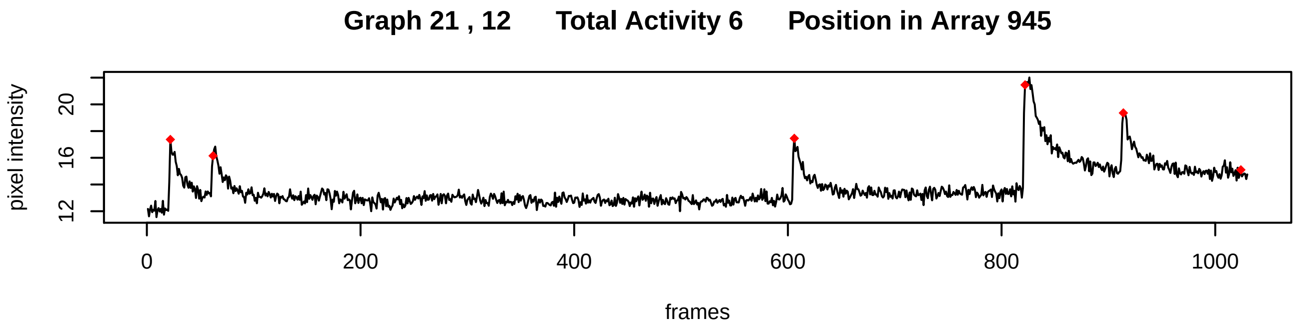
Graph 21 , 12 Total Activity 6 Position in Array 945
pixel intensity
12
16
20
0
200
400
600
800
1000
frames

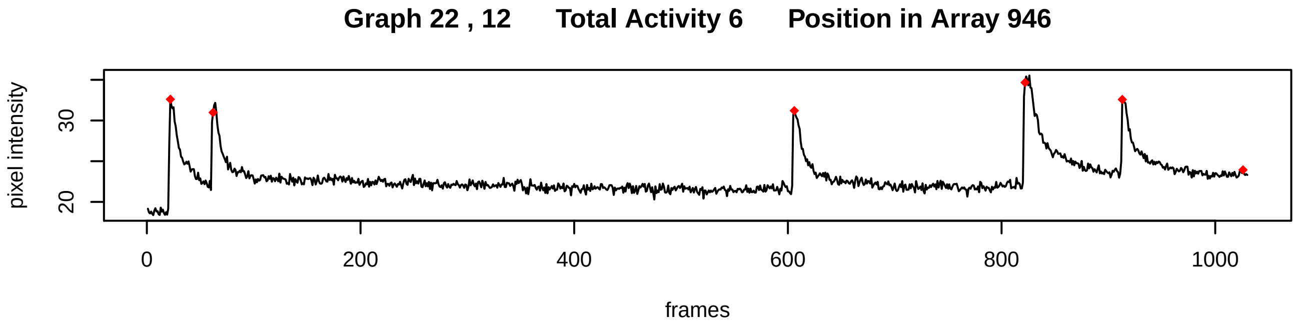
Graph 22 , 12 Total Activity 6 Position in Array 946
pixel intensity
20
30
0
200
400
600
800
1000
frames

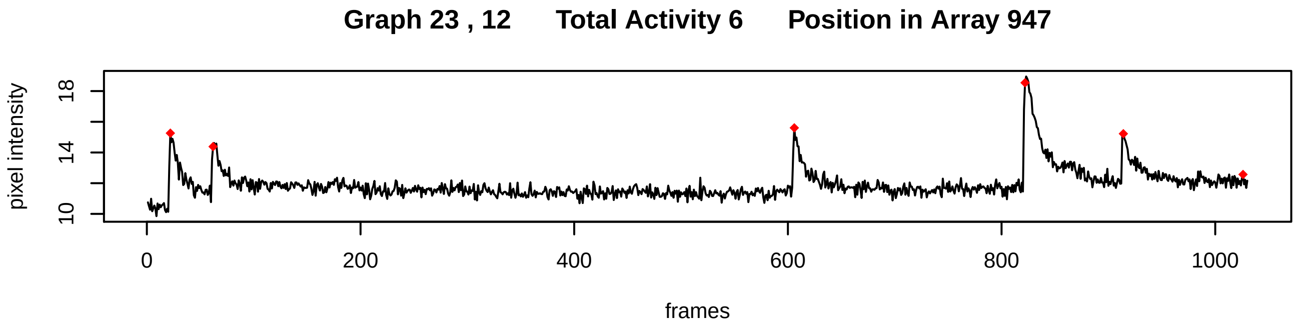
Graph 23 , 12 Total Activity 6 Position in Array 947
pixel intensity
10
14
18
0
200
400
600
800
1000
frames

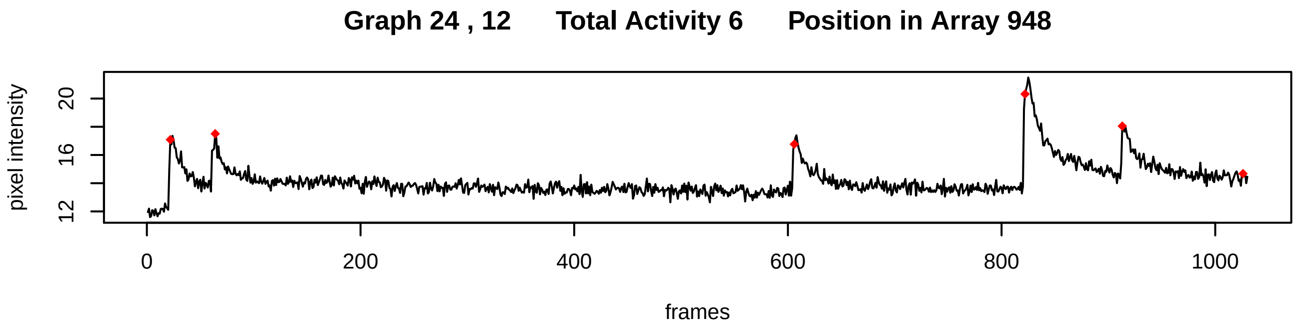
Graph 24 , 12   Total Activity 6   Position in Array 948
pixel intensity
12
16
20
0
200
400
600
800
1000
frames

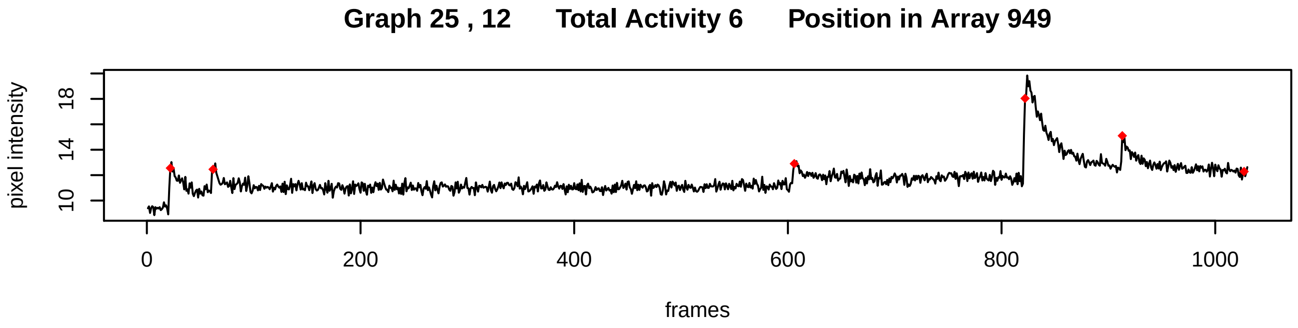
Graph 25 , 12   Total Activity 6   Position in Array 949
pixel intensity
10
14
18
0
200
400
600
800
1000
frames

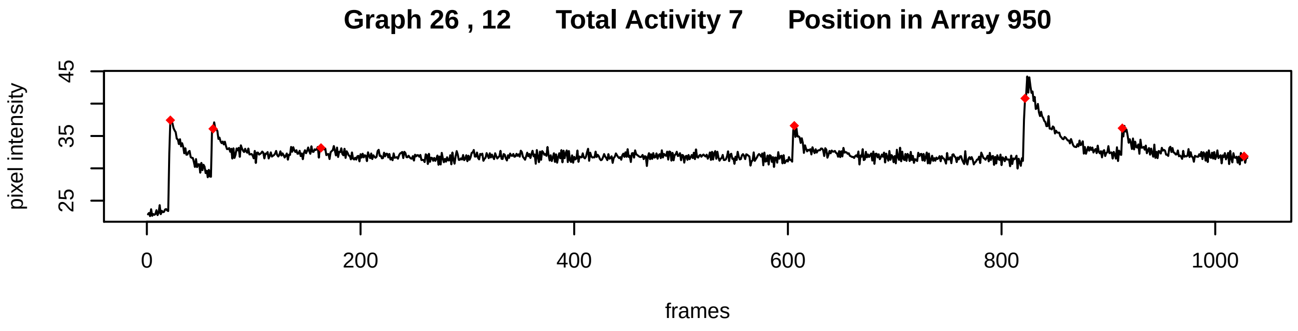
Graph 26 , 12   Total Activity 7   Position in Array 950
pixel intensity
25
35
45
0
200
400
600
800
1000
frames

Graph 27 , 12   Total Activity 7   Position in Array 951

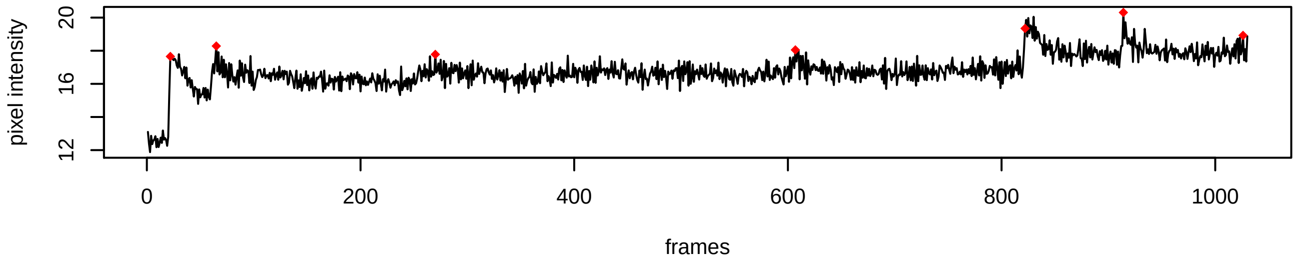

Graph 28 , 12   Total Activity 6   Position in Array 952

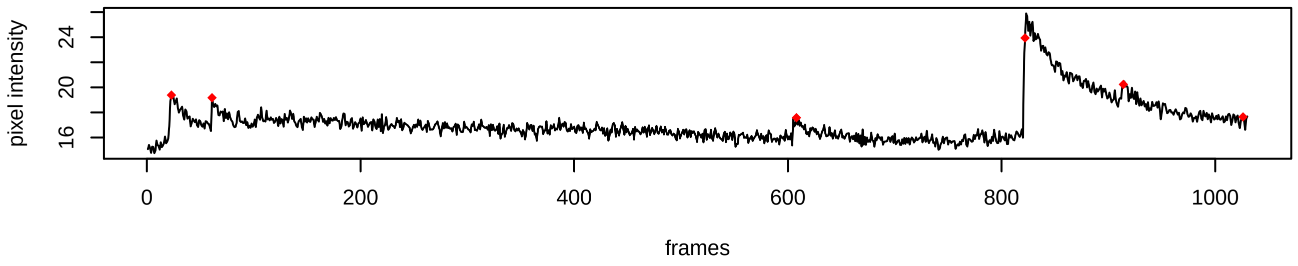

Graph 30 , 12   Total Activity 5   Position in Array 954

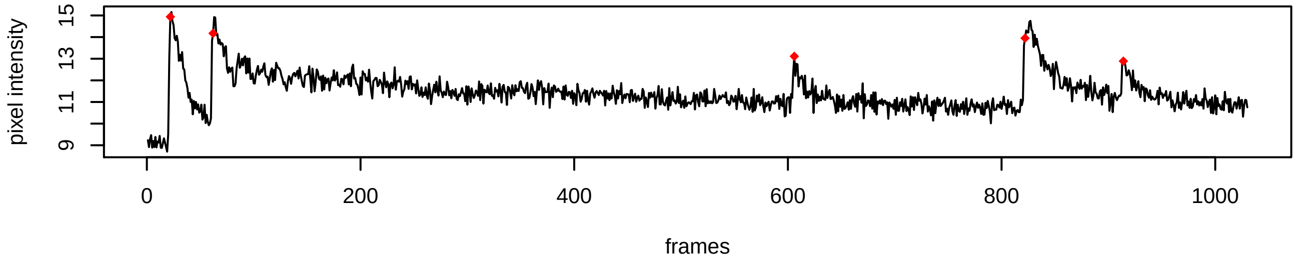

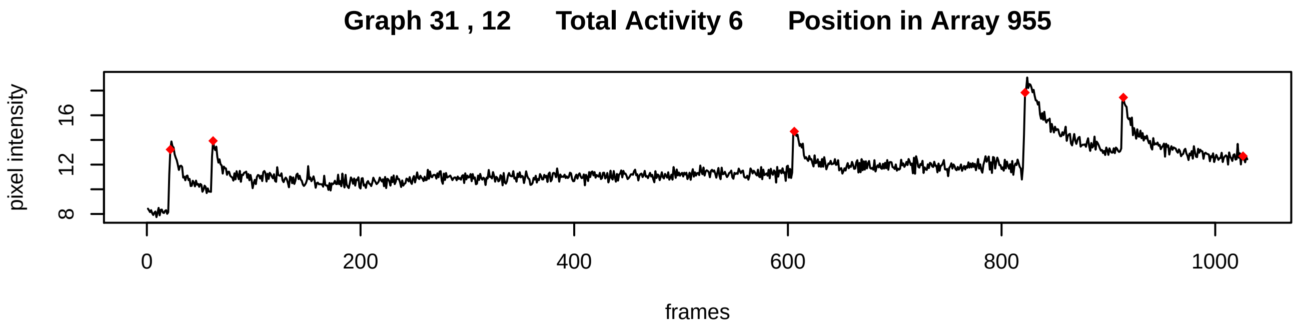
Graph 31 , 12 Total Activity 6 Position in Array 955
pixel intensity
8
12
16
0
200
400
600
800
1000
frames

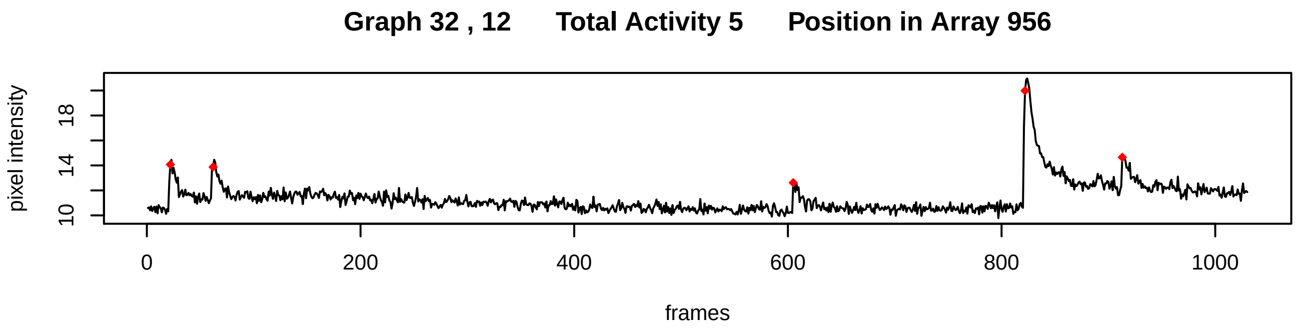
Graph 32 , 12 Total Activity 5 Position in Array 956
pixel intensity
10
14
18
0
200
400
600
800
1000
frames

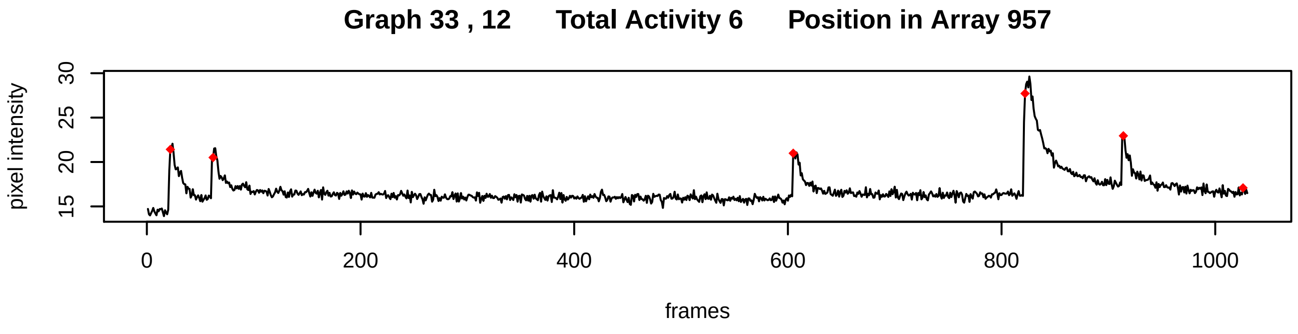
Graph 33 , 12 Total Activity 6 Position in Array 957
pixel intensity
15
20
25
30
0
200
400
600
800
1000
frames

Graph 34 , 12   Total Activity 7   Position in Array 958

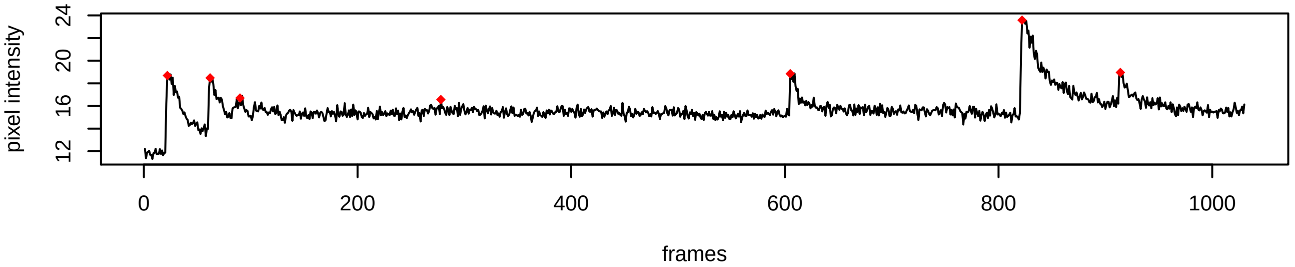

Graph 36 , 12   Total Activity 5   Position in Array 960

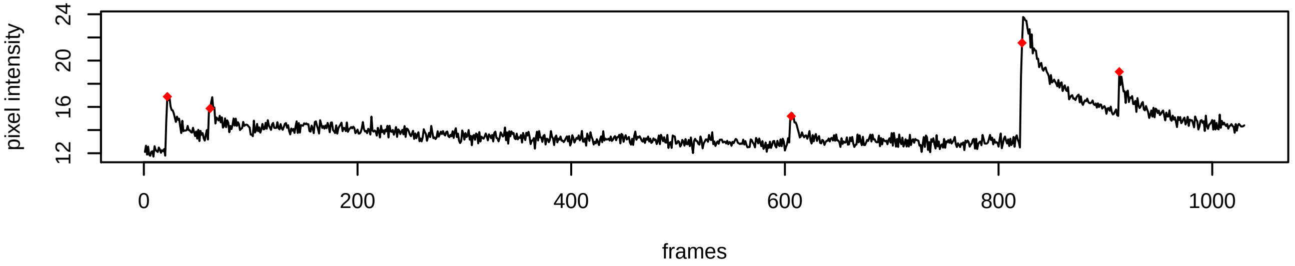

Graph 37 , 12   Total Activity 6   Position in Array 961

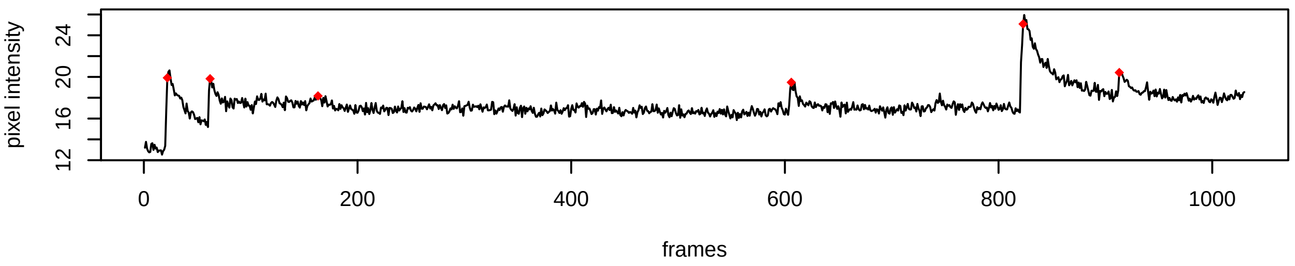

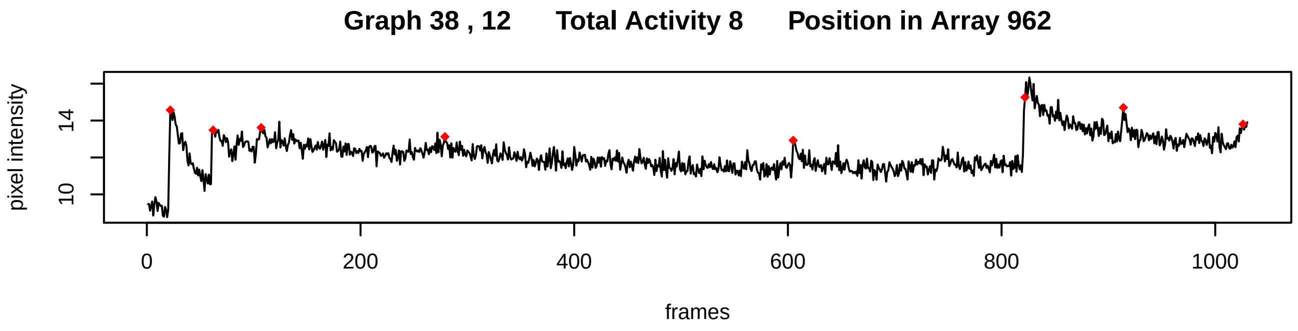
Graph 38 , 12 Total Activity 8 Position in Array 962
pixel intensity
10
14
0
200
400
600
800
1000
frames

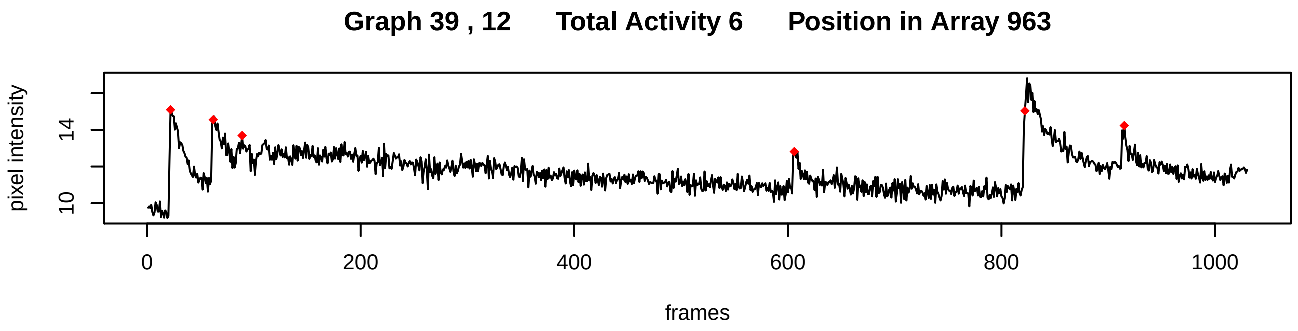
Graph 39 , 12 Total Activity 6 Position in Array 963
pixel intensity
10
14
0
200
400
600
800
1000
frames

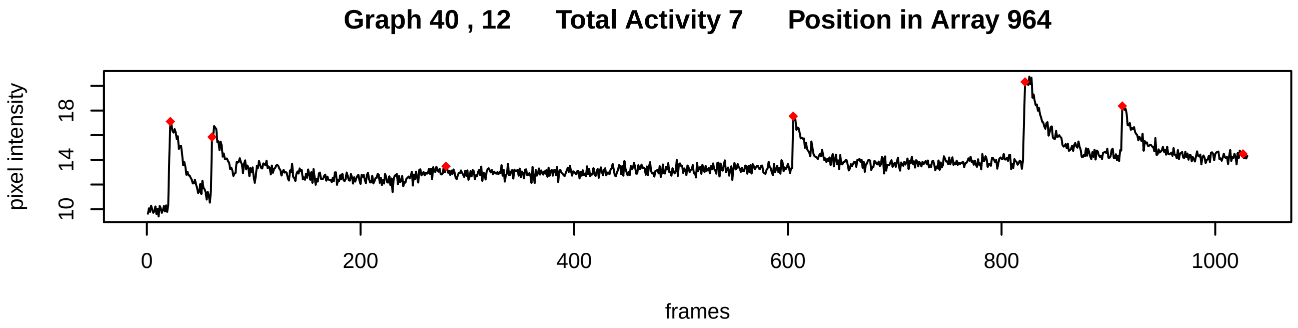
Graph 40 , 12 Total Activity 7 Position in Array 964
pixel intensity
10
14
18
0
200
400
600
800
1000
frames

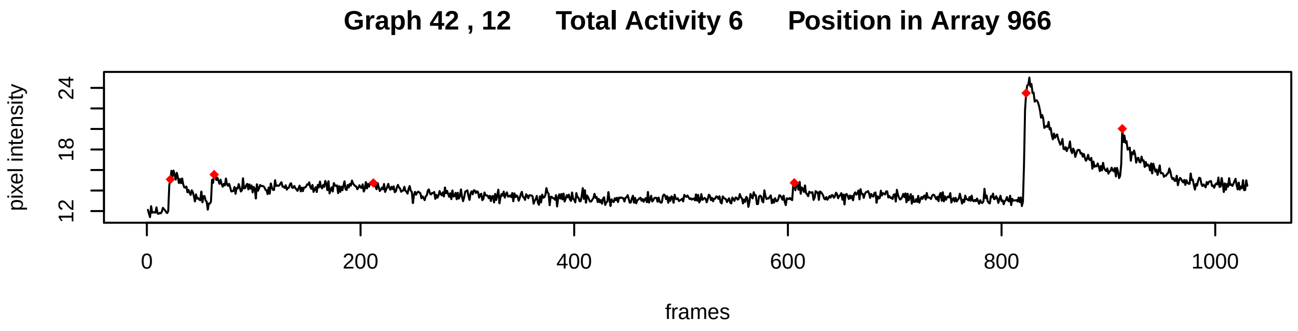
Graph 42 , 12 Total Activity 6 Position in Array 966
pixel intensity
12
18
24
0
200
400
600
800
1000
frames

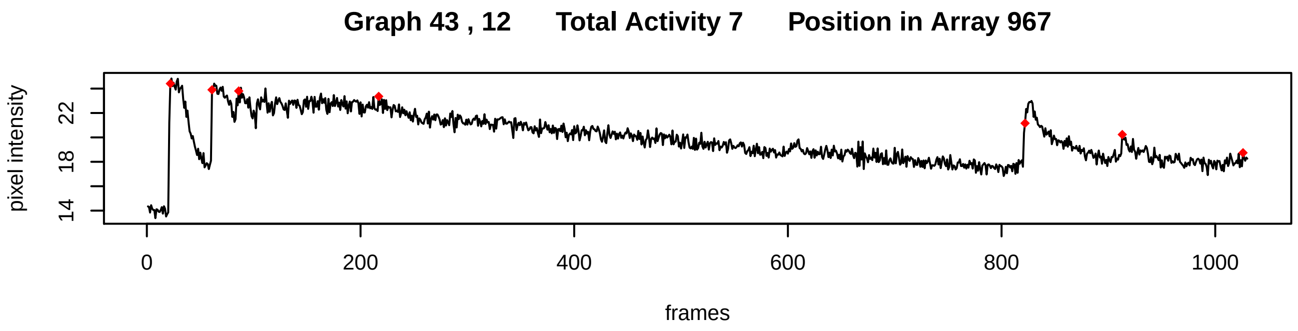
Graph 43 , 12 Total Activity 7 Position in Array 967
pixel intensity
14
18
22
0
200
400
600
800
1000
frames

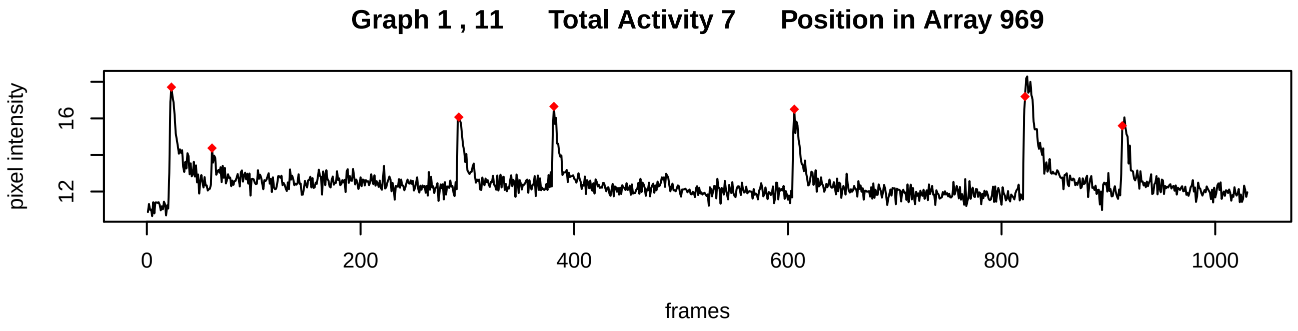
Graph 1 , 11 Total Activity 7 Position in Array 969
pixel intensity
12
16
0
200
400
600
800
1000
frames

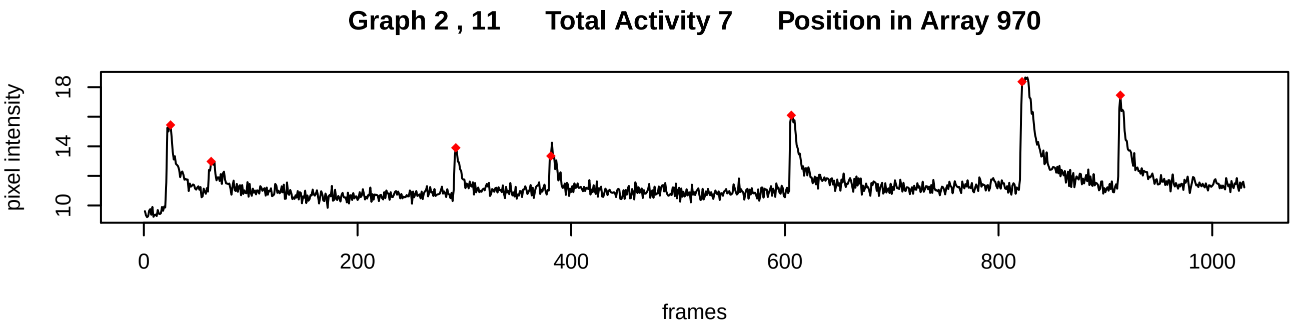
Graph 2 , 11 Total Activity 7 Position in Array 970
pixel intensity
10
14
18
0
200
400
600
800
1000
frames

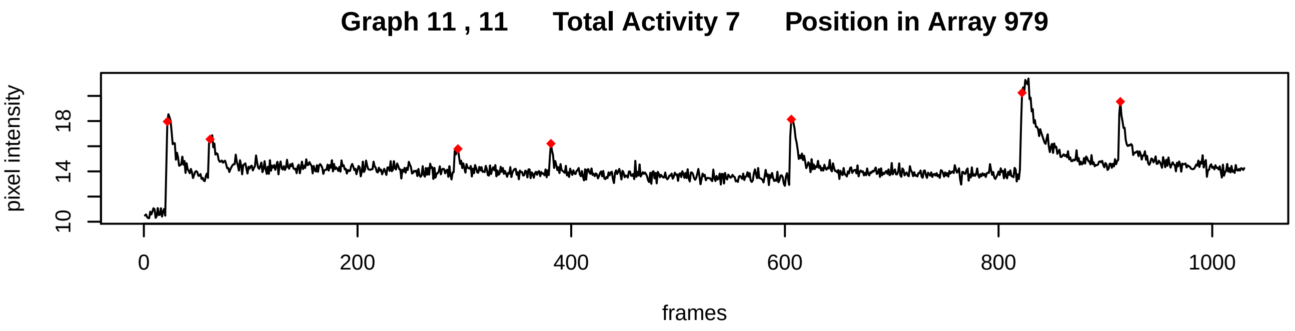
Graph 11 , 11 Total Activity 7 Position in Array 979
pixel intensity
10
14
18
0
200
400
600
800
1000
frames

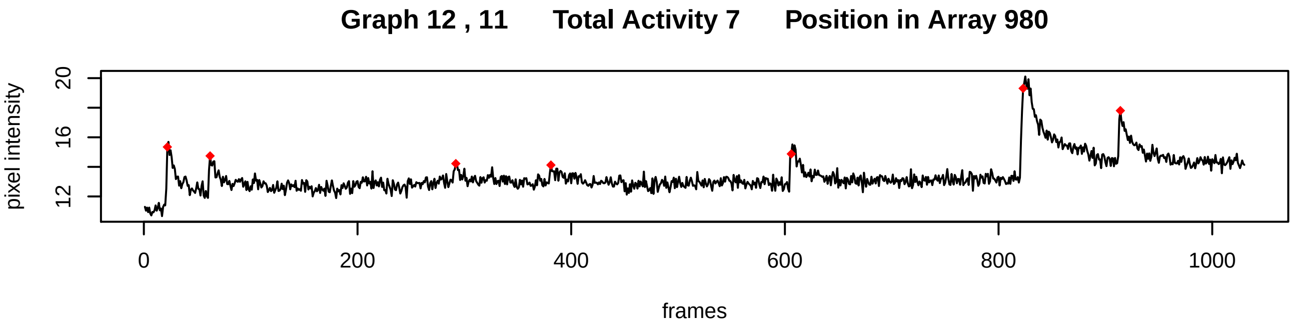
Graph 12 , 11 Total Activity 7 Position in Array 980
pixel intensity
12
16
20
0
200
400
600
800
1000
frames

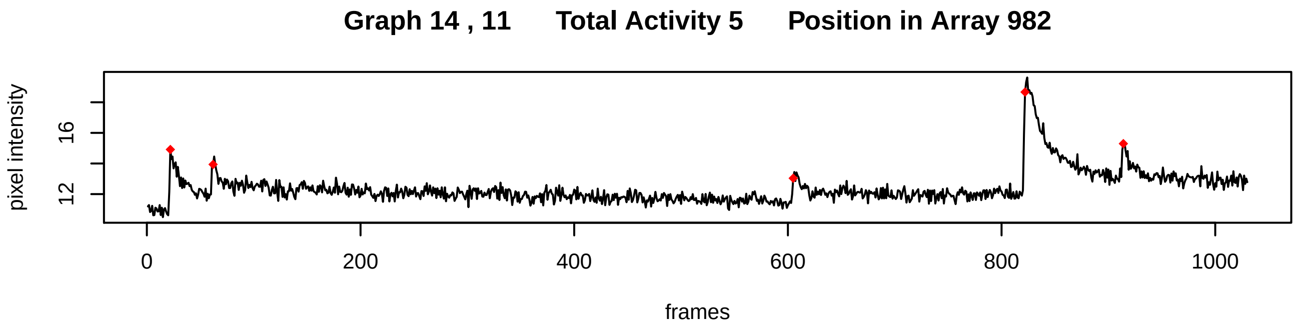
Graph 14 , 11 Total Activity 5 Position in Array 982
pixel intensity
12
16
0
200
400
600
800
1000
frames

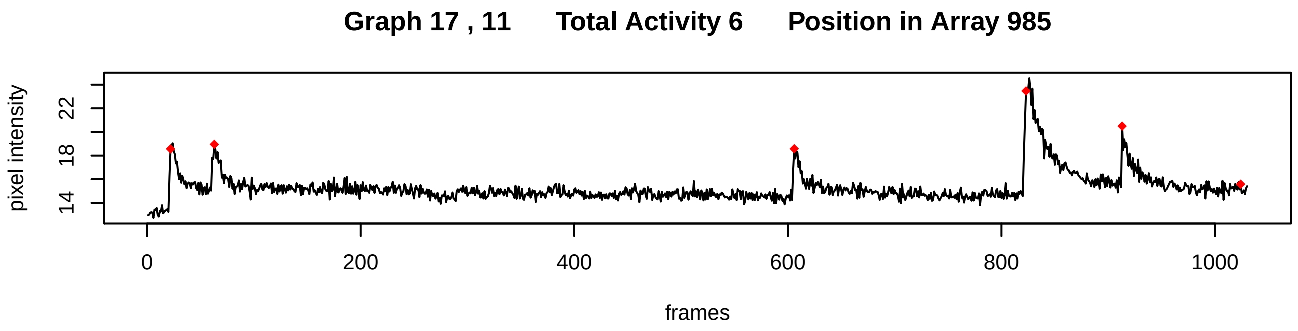
Graph 17 , 11 Total Activity 6 Position in Array 985
pixel intensity
14
18
22
0
200
400
600
800
1000
frames

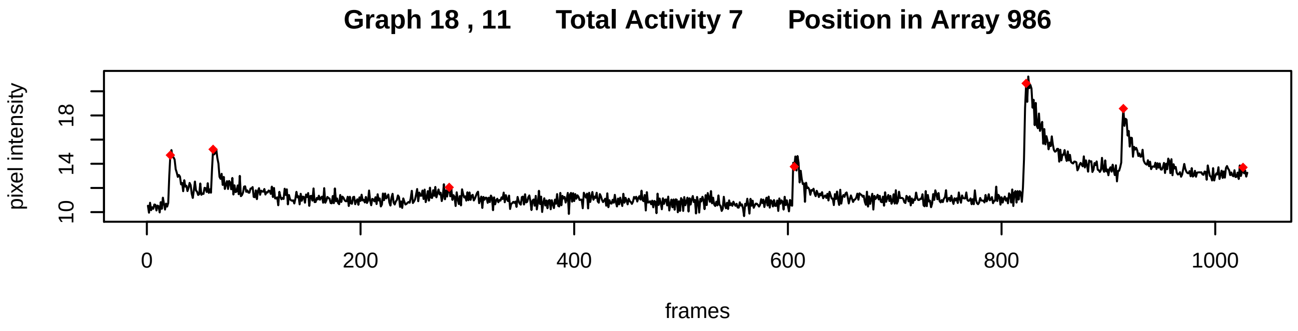
Graph 18 , 11 Total Activity 7 Position in Array 986
pixel intensity
10
14
18
0
200
400
600
800
1000
frames

Graph 20 , 11 Total Activity 6 Position in Array 988

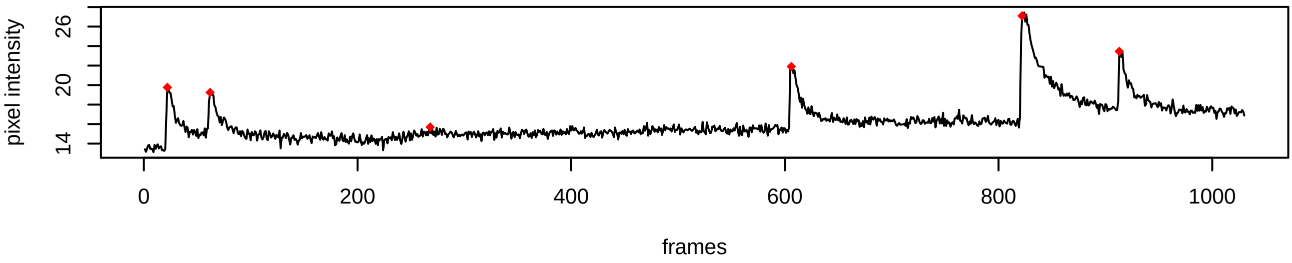

Graph 21 , 11 Total Activity 6 Position in Array 989

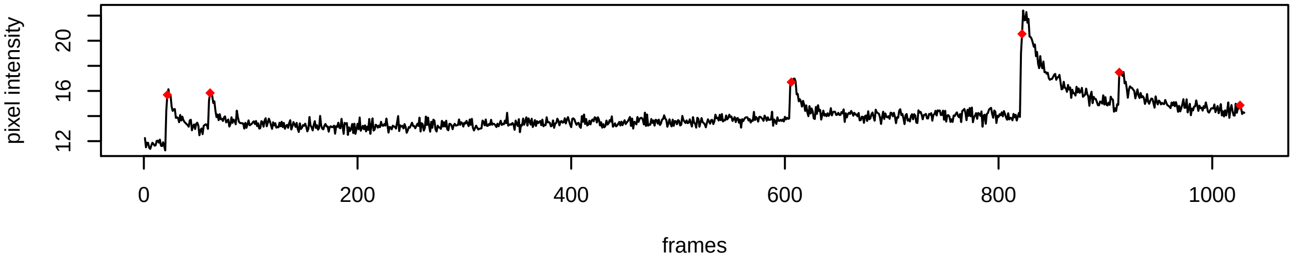

Graph 22 , 11 Total Activity 5 Position in Array 990

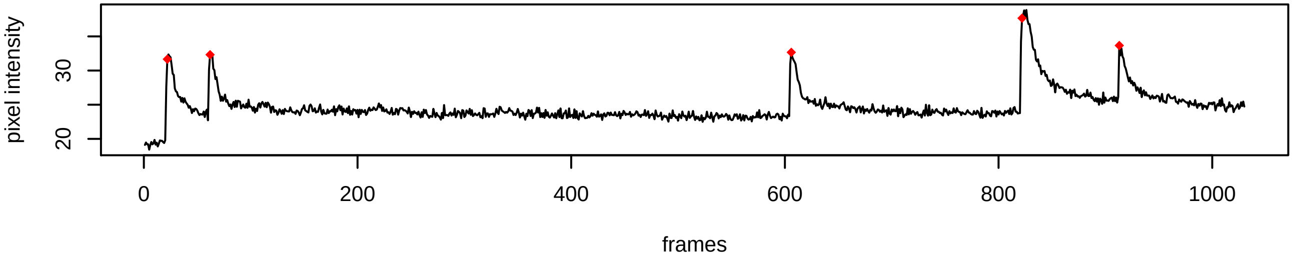

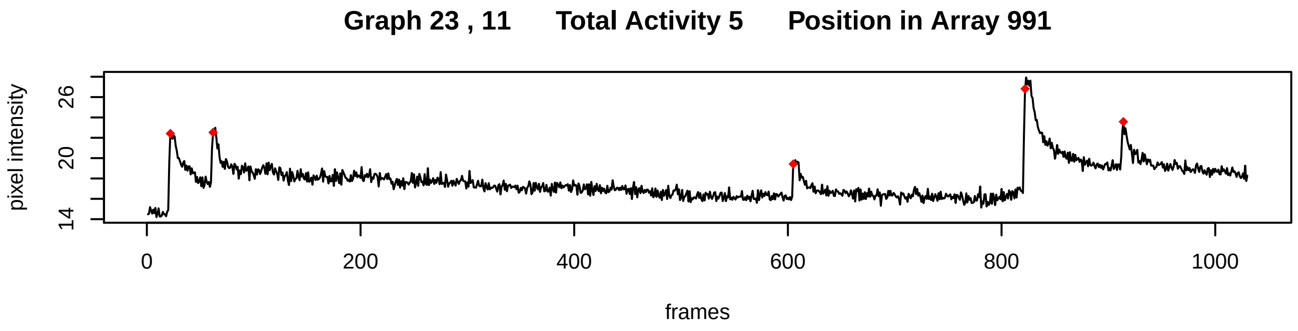
Graph 23 , 11 Total Activity 5 Position in Array 991
pixel intensity
14
20
26
0
200
400
600
800
1000
frames

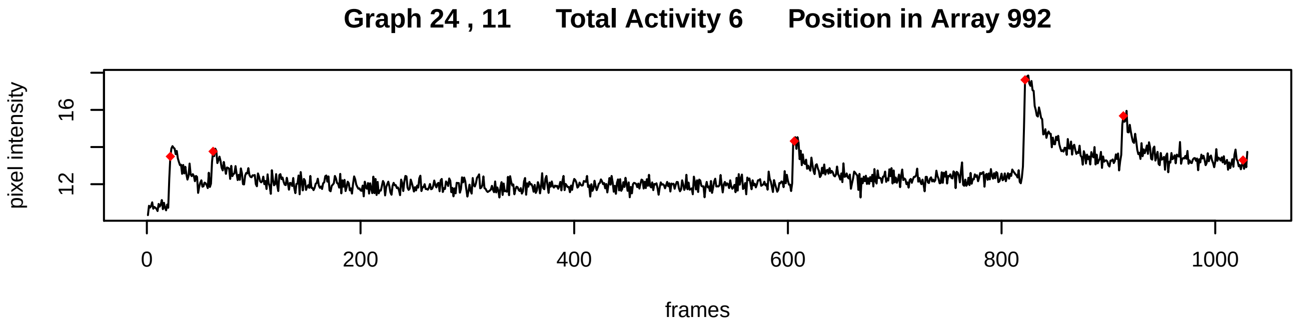
Graph 24 , 11 Total Activity 6 Position in Array 992
pixel intensity
12
16
0
200
400
600
800
1000
frames

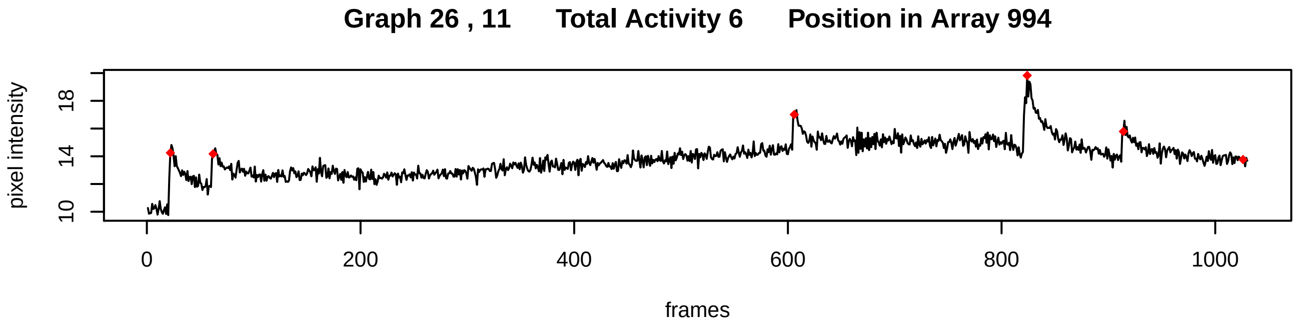
Graph 26 , 11 Total Activity 6 Position in Array 994
pixel intensity
10
14
18
0
200
400
600
800
1000
frames

Graph 27 , 11 Total Activity 5 Position in Array 995

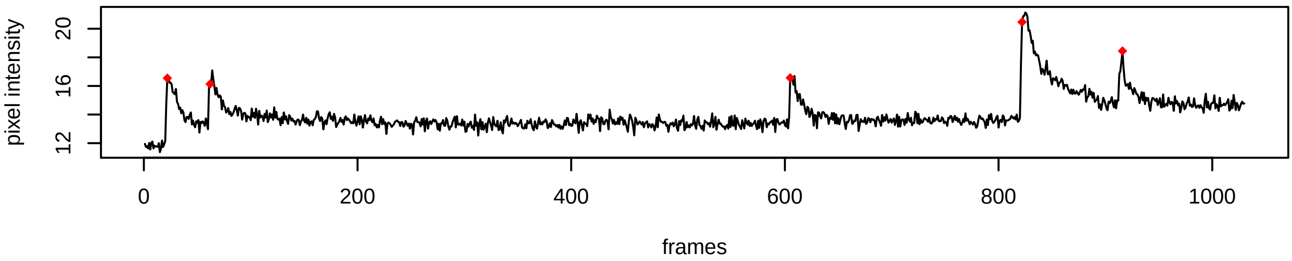

Graph 28 , 11 Total Activity 6 Position in Array 996

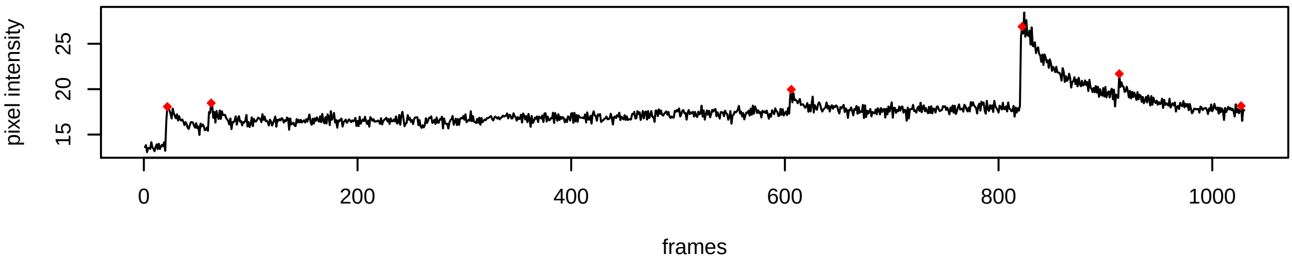

Graph 29 , 11 Total Activity 6 Position in Array 997

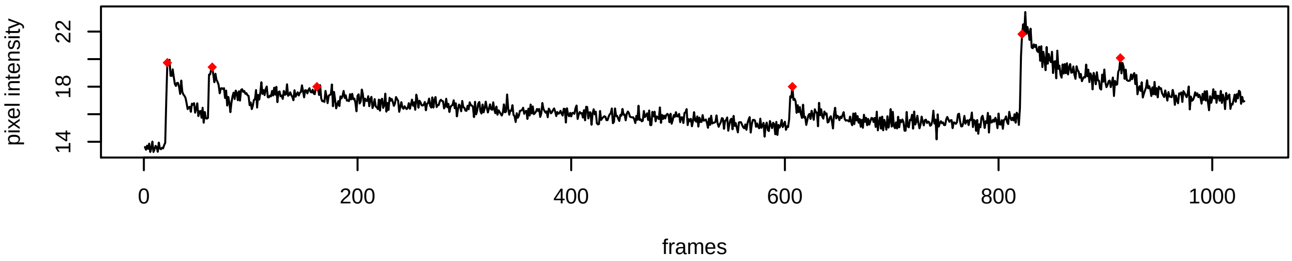

Graph 30 , 11 Total Activity 6 Position in Array 998

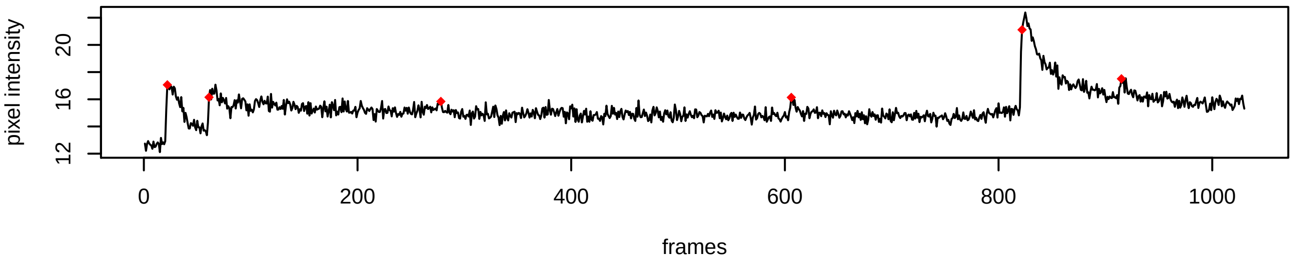

Graph 32 , 11 Total Activity 6 Position in Array 1000

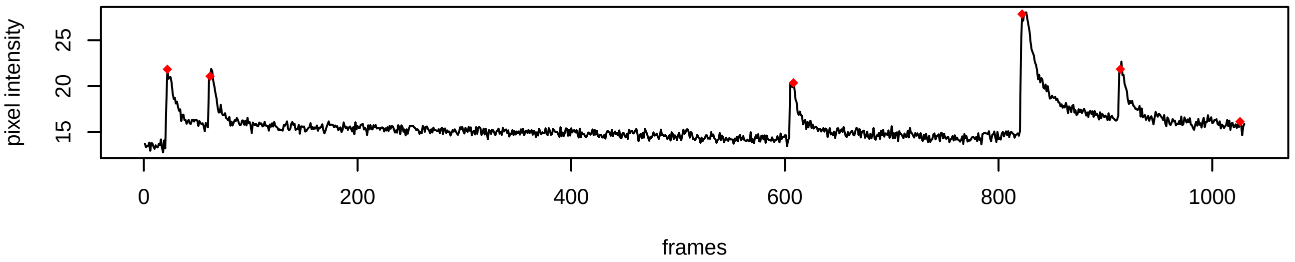

Graph 33 , 11 Total Activity 6 Position in Array 1001

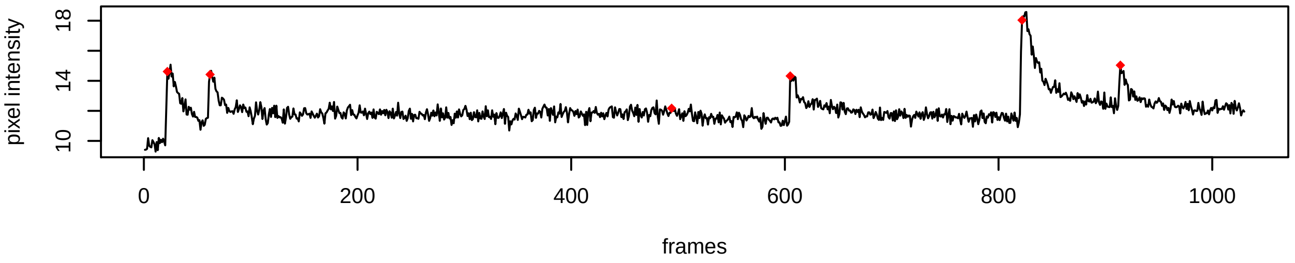

Graph 34 , 11   Total Activity 8   Position in Array 1002

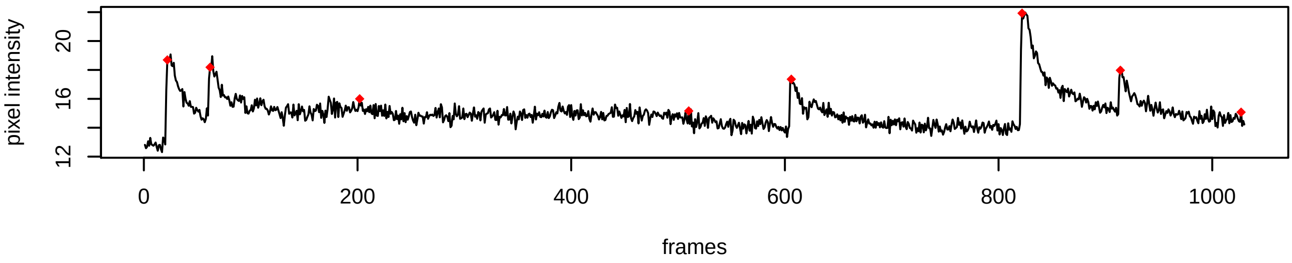

Graph 35 , 11   Total Activity 11   Position in Array 1003

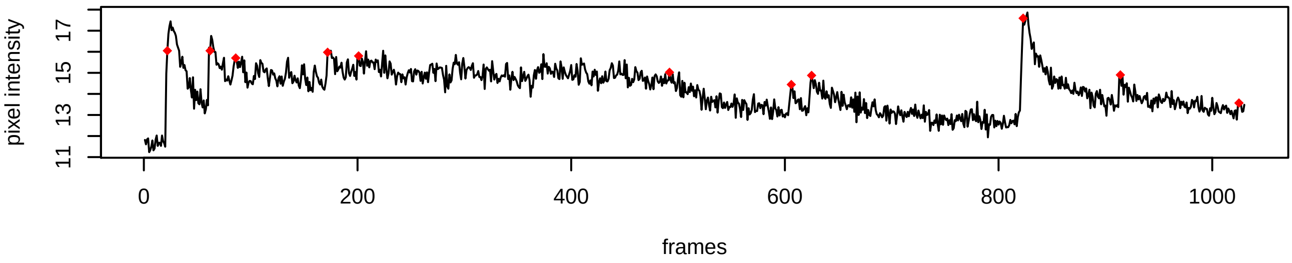

Graph 36 , 11   Total Activity 6   Position in Array 1004

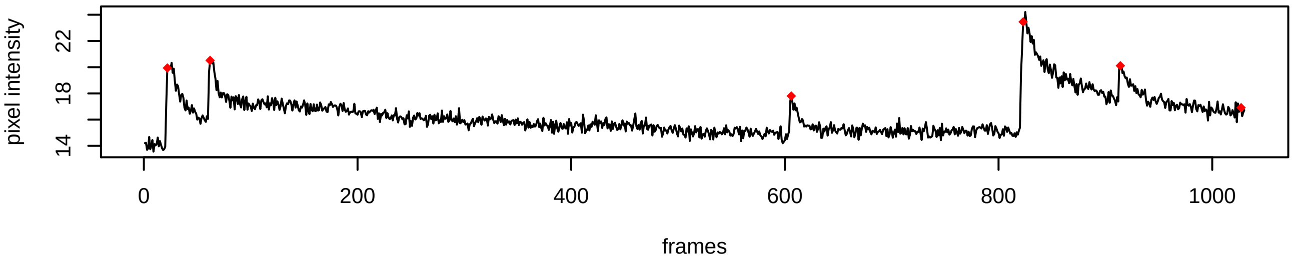

Graph 39 , 11 Total Activity 6 Position in Array 1007

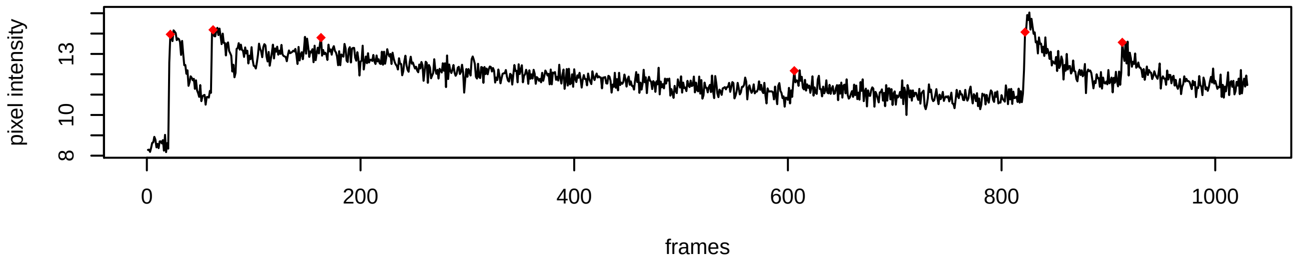

Graph 40 , 11 Total Activity 4 Position in Array 1008

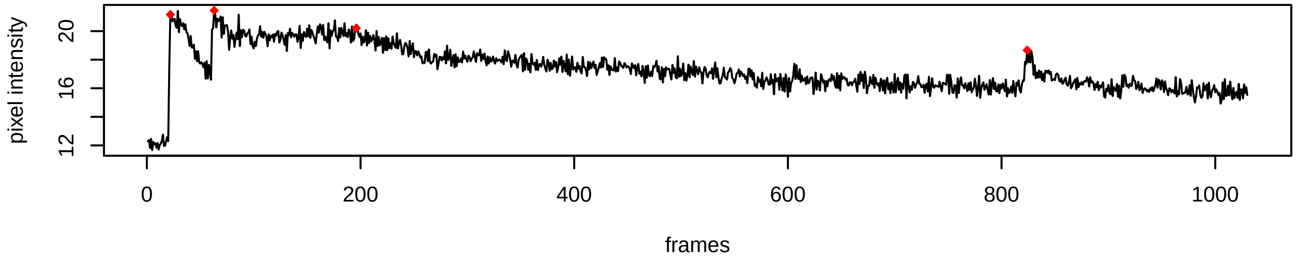

Graph 41 , 11 Total Activity 6 Position in Array 1009

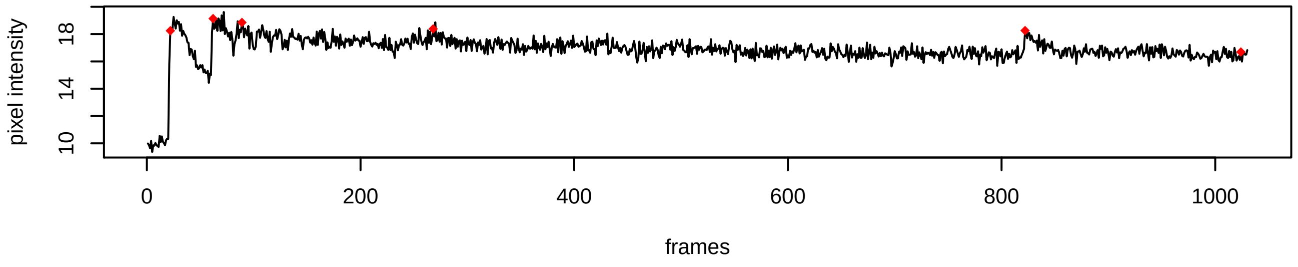

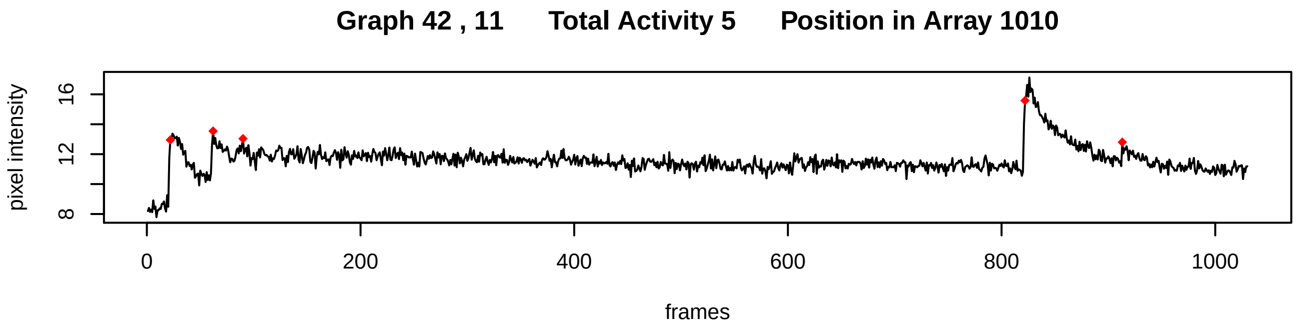
Graph 42 , 11 Total Activity 5 Position in Array 1010
pixel intensity
8
12
16
0
200
400
600
800
1000
frames

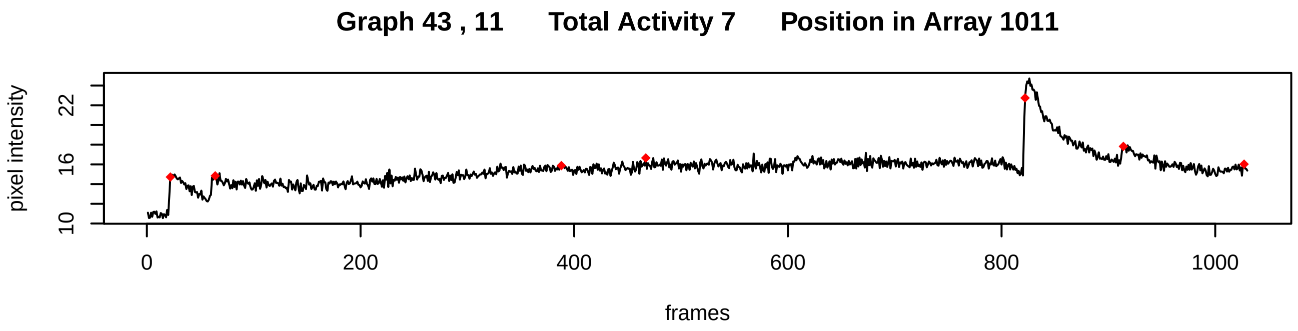
Graph 43 , 11 Total Activity 7 Position in Array 1011
pixel intensity
10
16
22
0
200
400
600
800
1000
frames

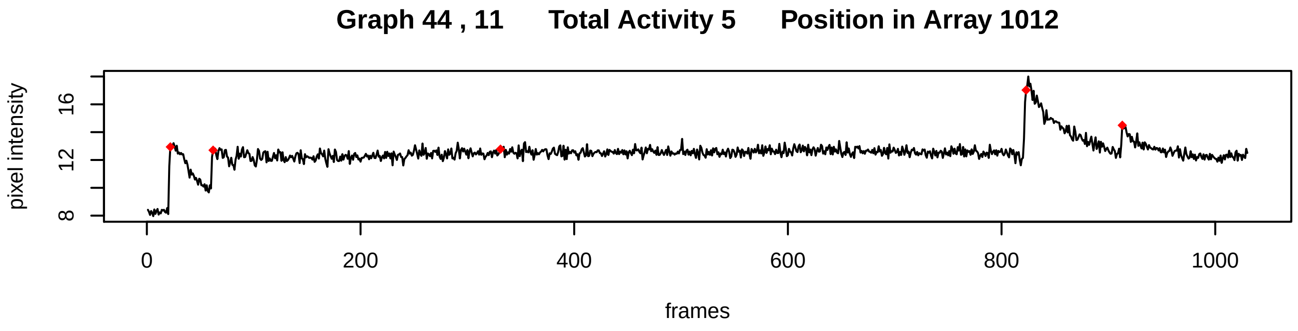
Graph 44 , 11 Total Activity 5 Position in Array 1012
pixel intensity
8
12
16
0
200
400
600
800
1000
frames

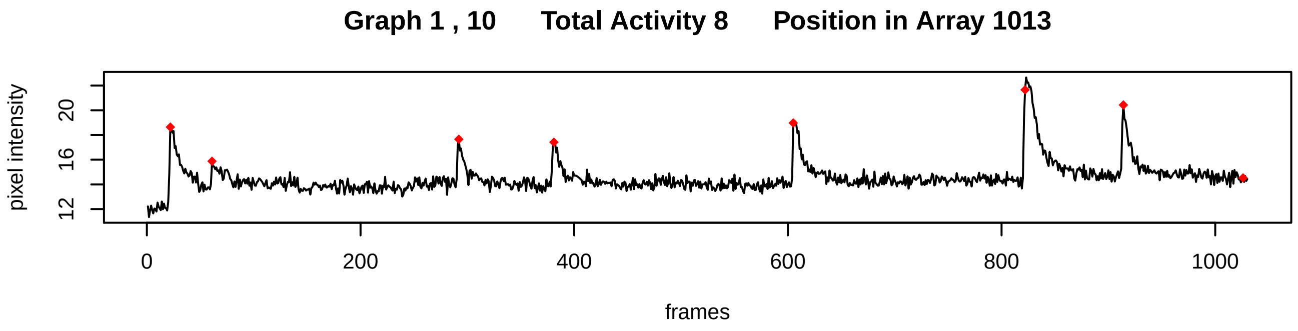
Graph 1 , 10 Total Activity 8 Position in Array 1013
pixel intensity
12
16
20
0
200
400
600
800
1000
frames

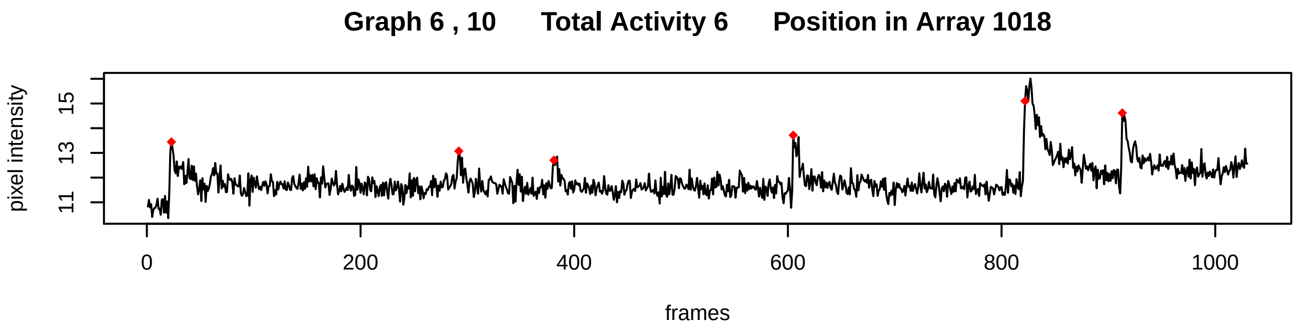
Graph 6 , 10 Total Activity 6 Position in Array 1018
pixel intensity
11
13
15
0
200
400
600
800
1000
frames

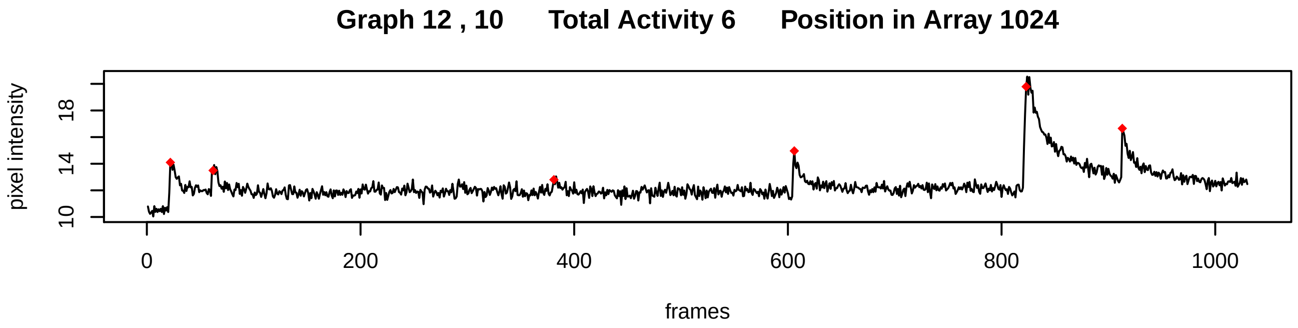
Graph 12 , 10 Total Activity 6 Position in Array 1024
pixel intensity
10
14
18
0
200
400
600
800
1000
frames

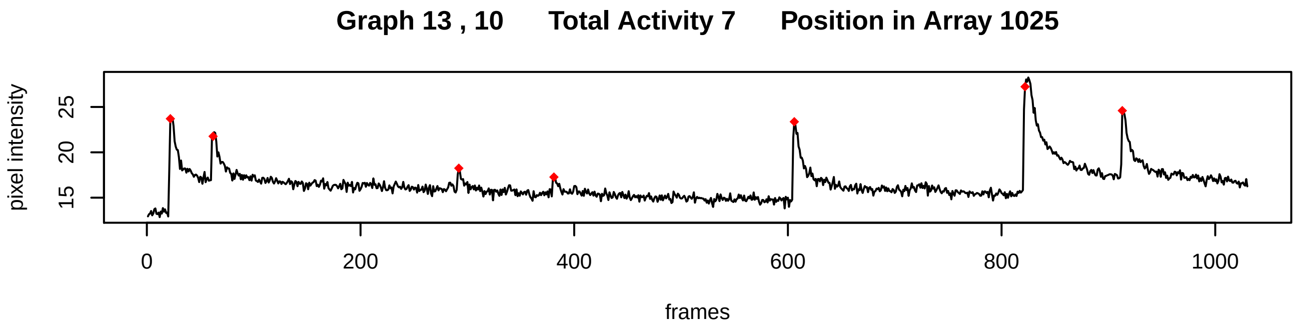
Graph 13 , 10 Total Activity 7 Position in Array 1025
pixel intensity
15
20
25
0
200
400
600
800
1000
frames

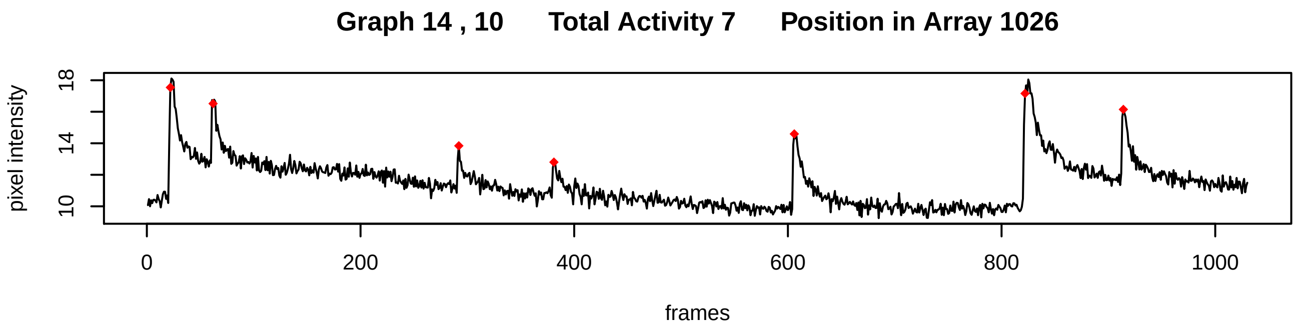
Graph 14 , 10 Total Activity 7 Position in Array 1026
pixel intensity
10
14
18
0
200
400
600
800
1000
frames

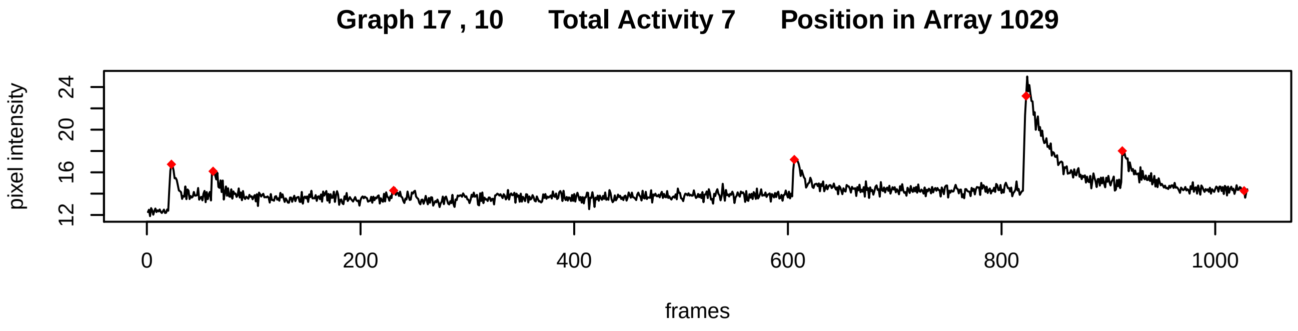
Graph 17 , 10 Total Activity 7 Position in Array 1029
pixel intensity
12
16
20
24
0
200
400
600
800
1000
frames

Graph 18 , 10 Total Activity 5 Position in Array 1030

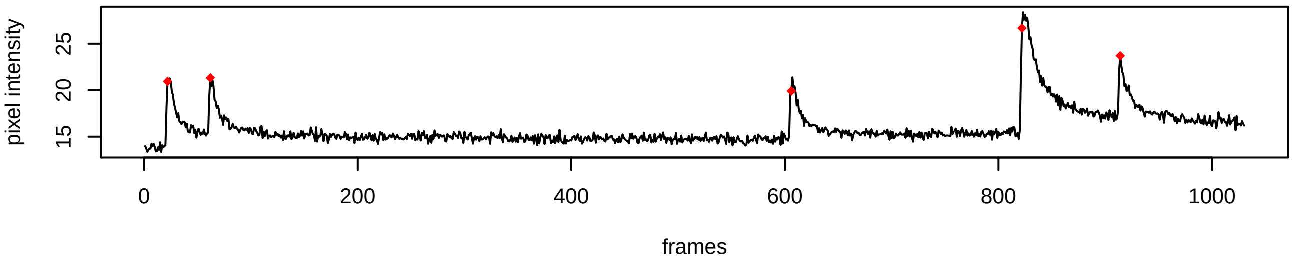

Graph 20 , 10 Total Activity 6 Position in Array 1032

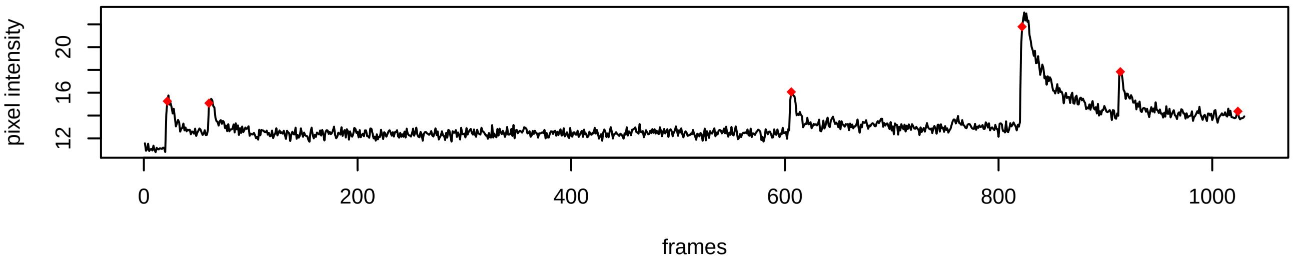

Graph 22 , 10 Total Activity 8 Position in Array 1034

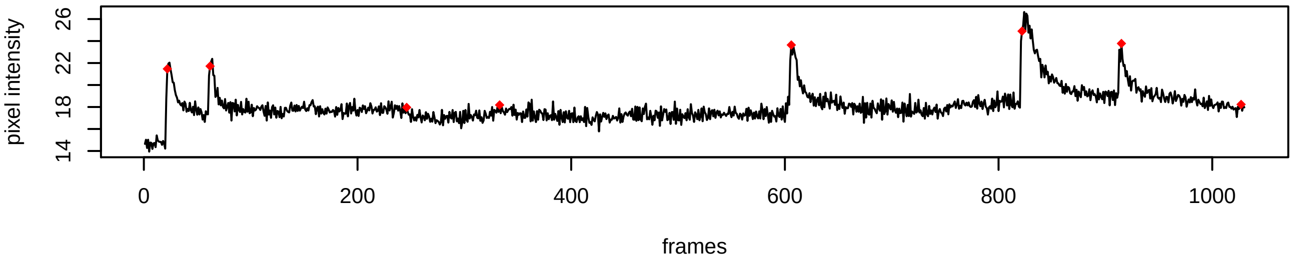

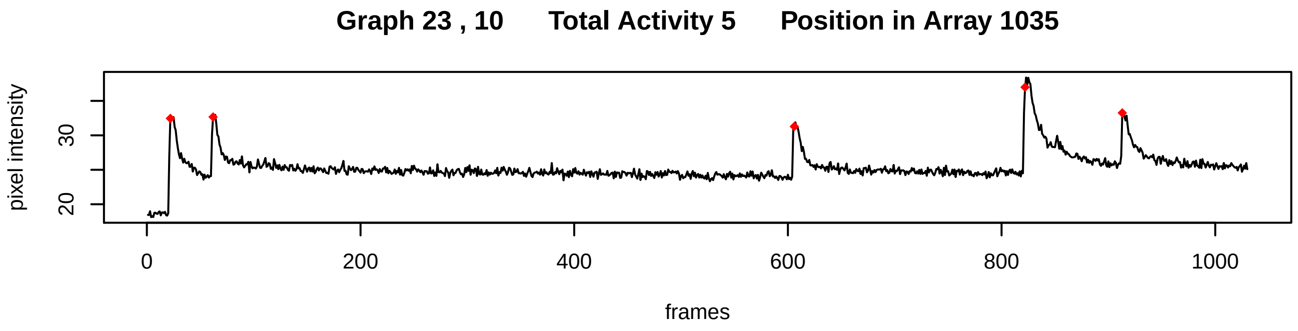
Graph 23 , 10 Total Activity 5 Position in Array 1035
pixel intensity
20
30
0
200
400
600
800
1000
frames

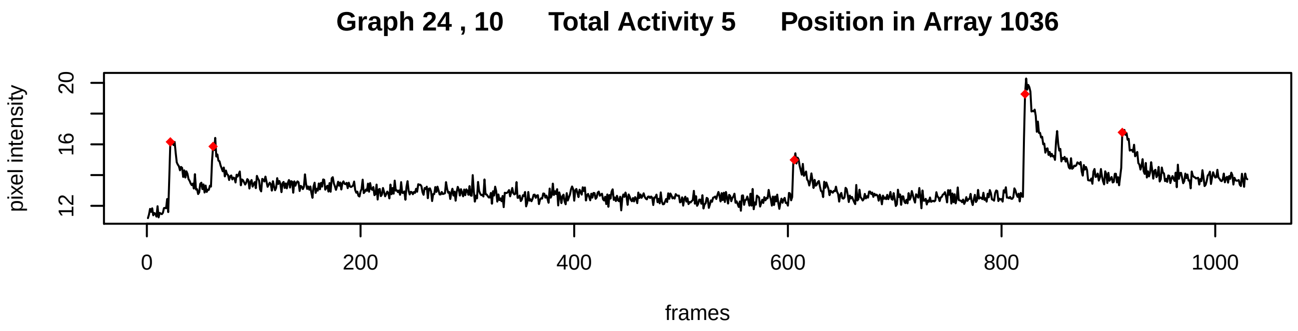
Graph 24 , 10 Total Activity 5 Position in Array 1036
pixel intensity
12
16
20
0
200
400
600
800
1000
frames

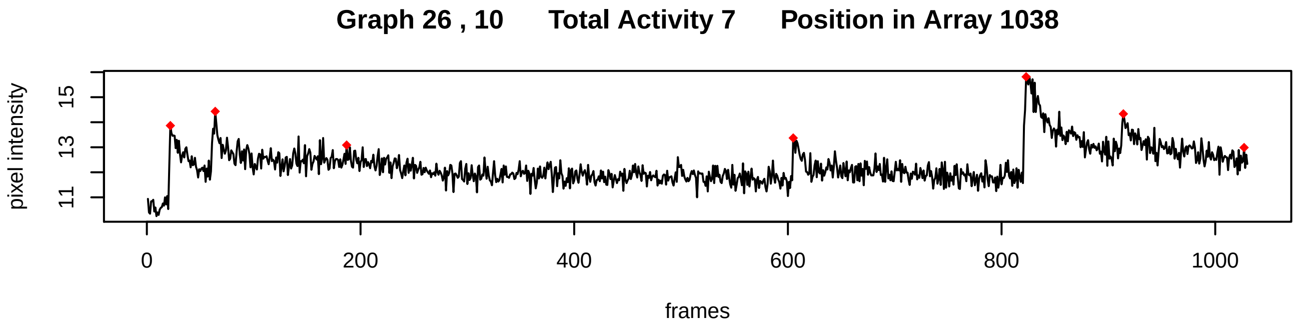
Graph 26 , 10 Total Activity 7 Position in Array 1038
pixel intensity
11
13
15
0
200
400
600
800
1000
frames

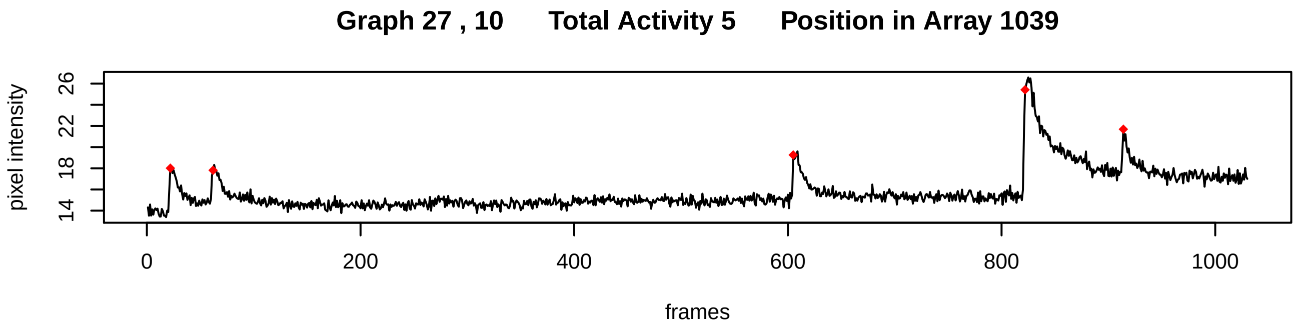
Graph 27 , 10 Total Activity 5 Position in Array 1039
pixel intensity
14
18
22
26
0
200
400
600
800
1000
frames

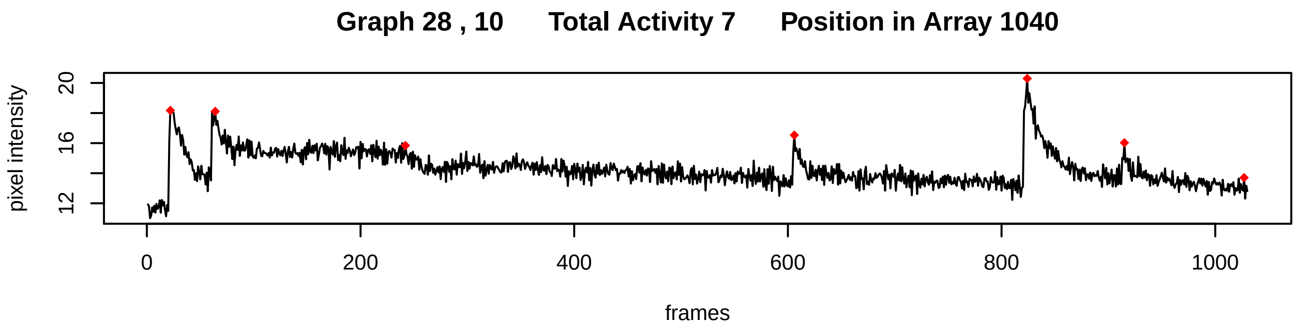
Graph 28 , 10 Total Activity 7 Position in Array 1040
pixel intensity
12
16
20
0
200
400
600
800
1000
frames

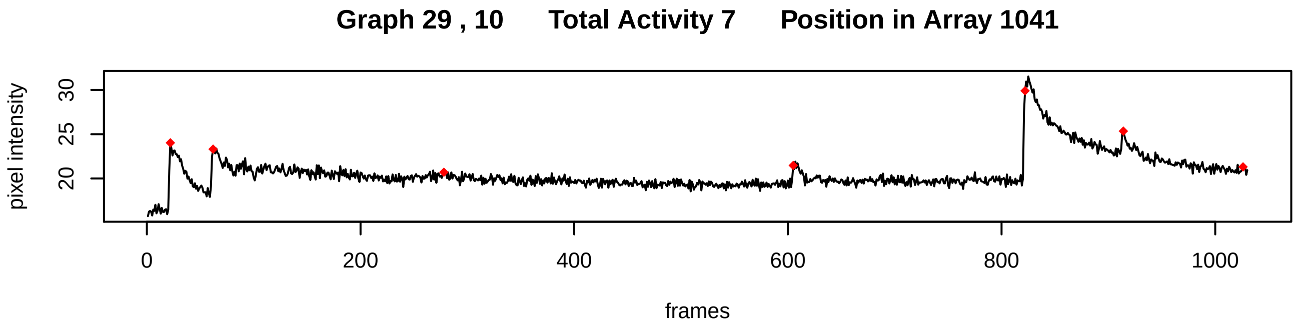
Graph 29 , 10 Total Activity 7 Position in Array 1041
pixel intensity
20
25
30
0
200
400
600
800
1000
frames

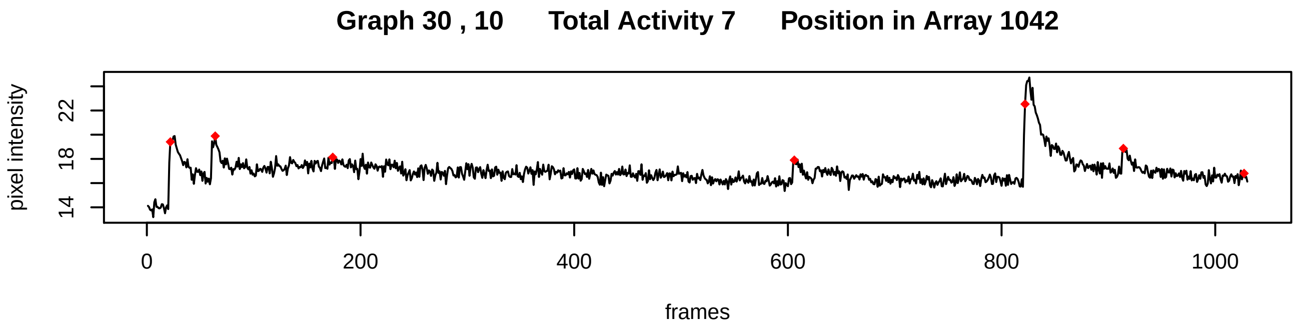
Graph 30 , 10 Total Activity 7 Position in Array 1042
pixel intensity
14
18
22
0
200
400
600
800
1000
frames

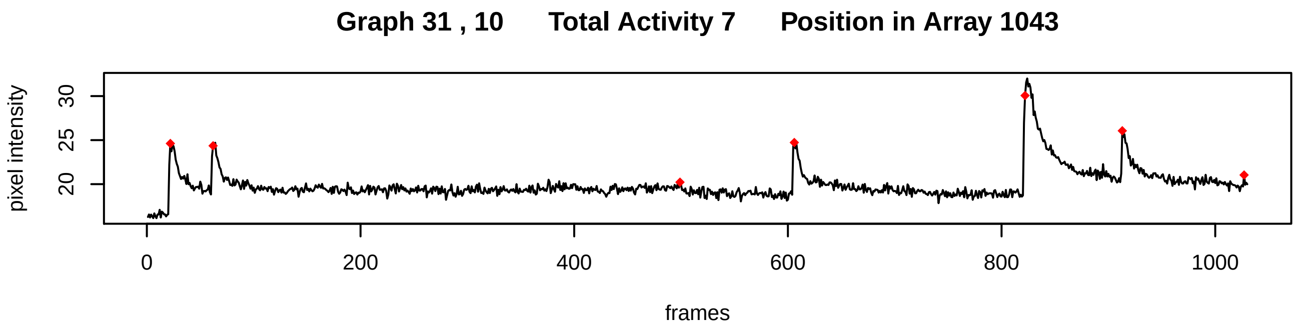
Graph 31 , 10 Total Activity 7 Position in Array 1043
pixel intensity
20
25
30
0
200
400
600
800
1000
frames

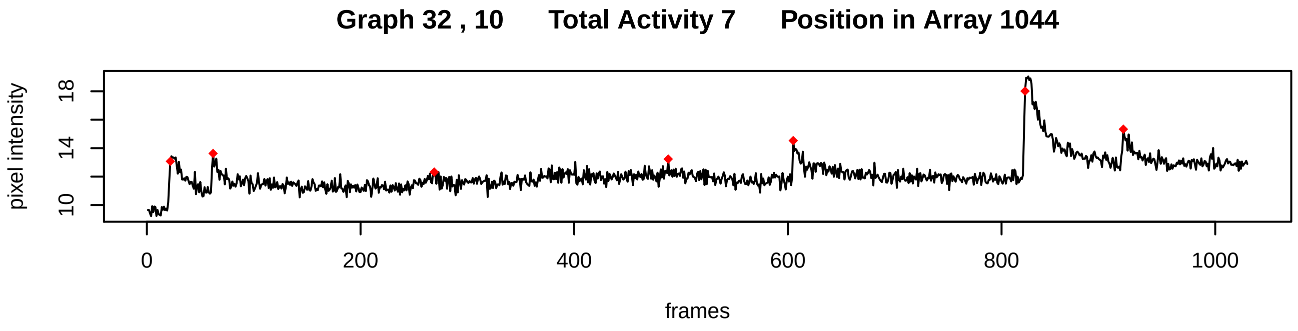
Graph 32 , 10 Total Activity 7 Position in Array 1044
pixel intensity
10
14
18
0
200
400
600
800
1000
frames

Graph 33 , 10 Total Activity 6 Position in Array 1045

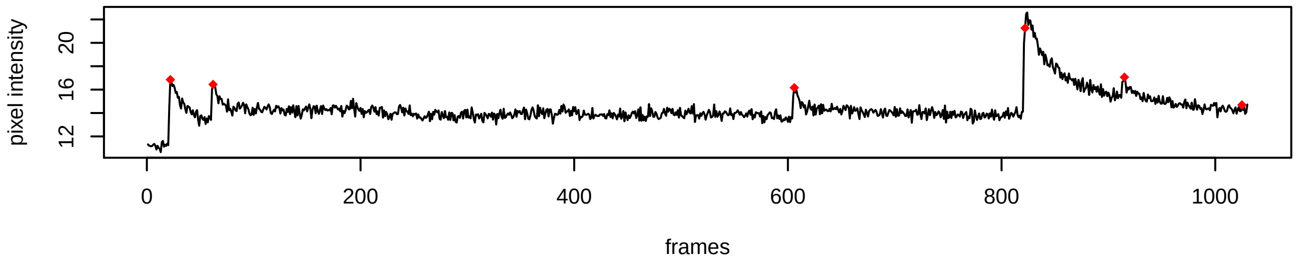

Graph 34 , 10 Total Activity 6 Position in Array 1046

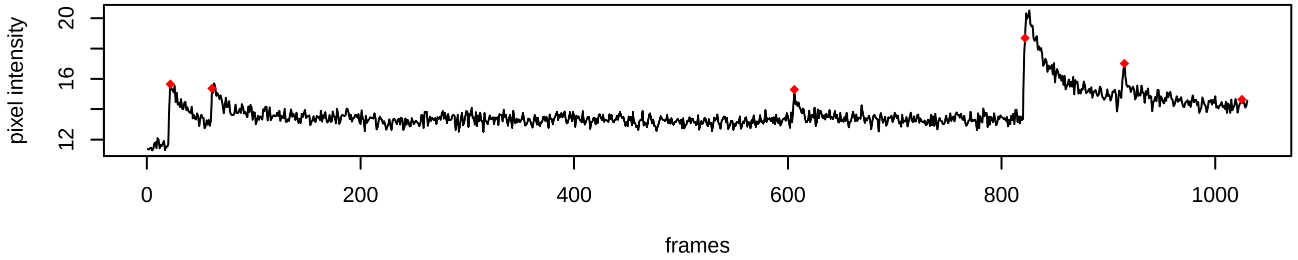

Graph 35 , 10 Total Activity 6 Position in Array 1047

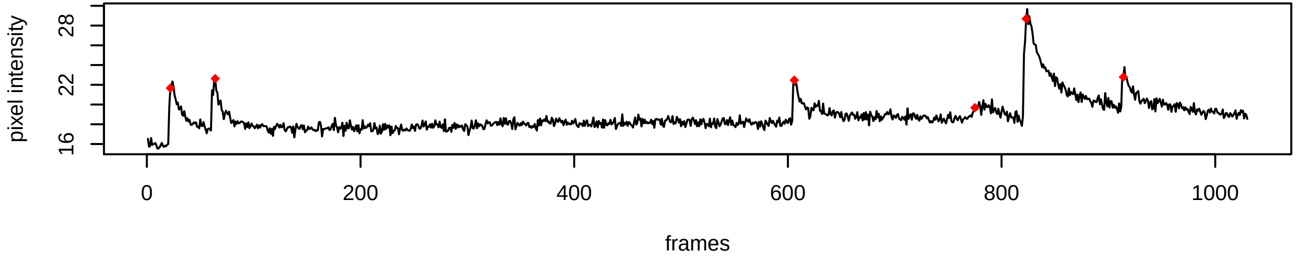

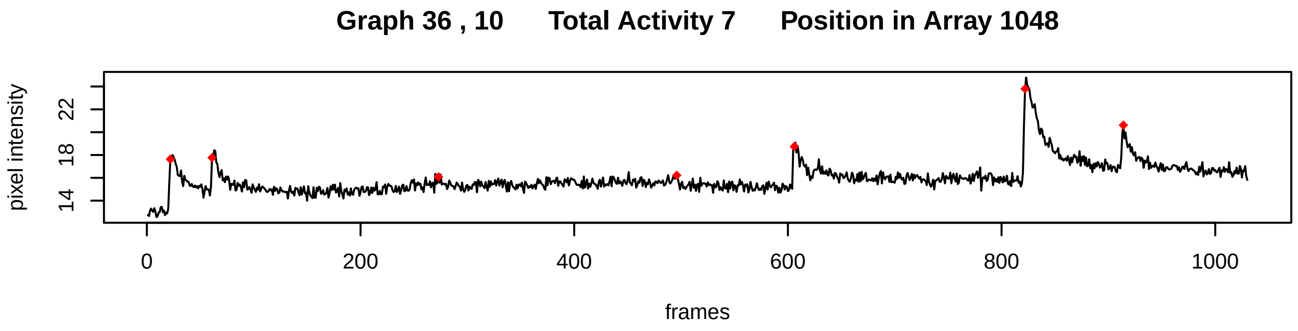
Graph 36 , 10 Total Activity 7 Position in Array 1048
pixel intensity
14
18
22
0
200
400
600
800
1000
frames

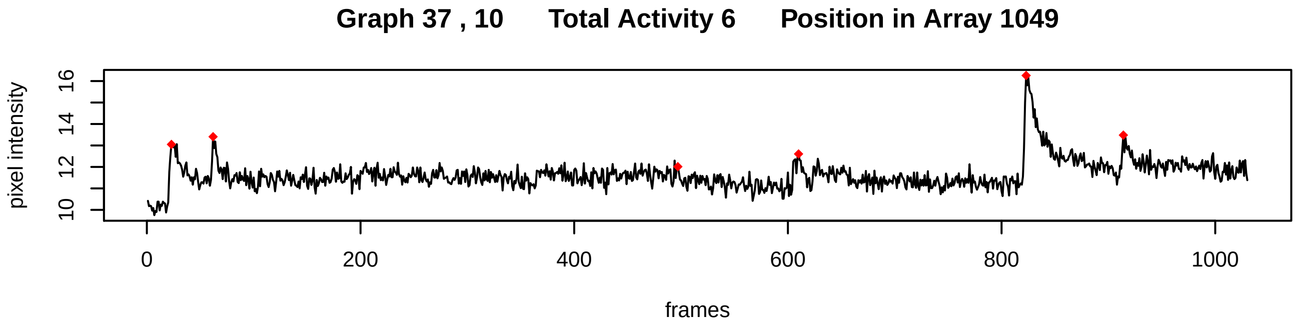
Graph 37 , 10 Total Activity 6 Position in Array 1049
pixel intensity
10
12
14
16
0
200
400
600
800
1000
frames

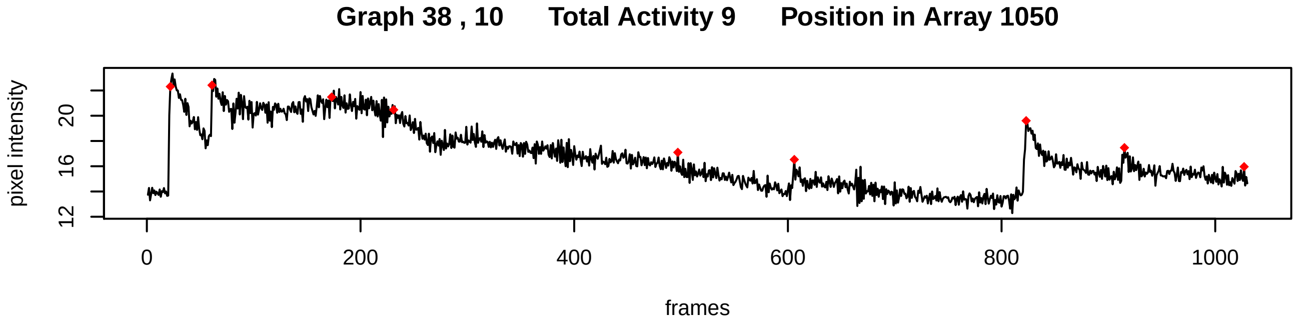
Graph 38 , 10 Total Activity 9 Position in Array 1050
pixel intensity
12
16
20
0
200
400
600
800
1000
frames

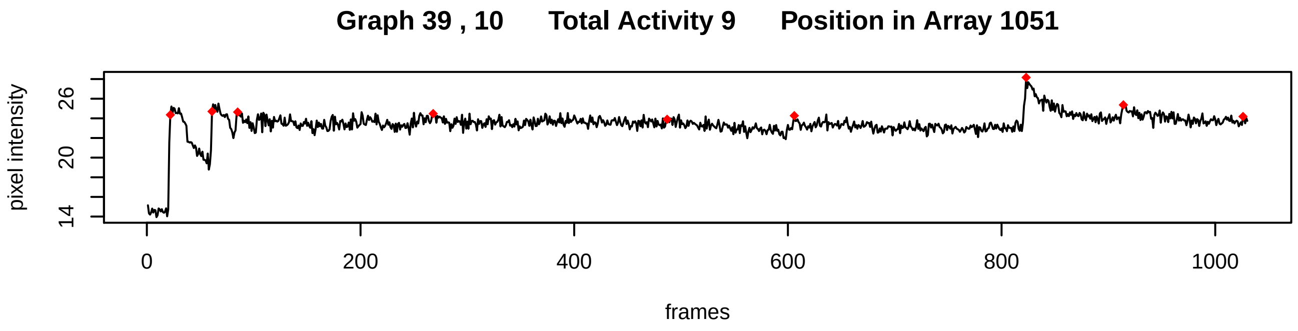
Graph 39 , 10 Total Activity 9 Position in Array 1051
pixel intensity
14
20
26
0
200
400
600
800
1000
frames

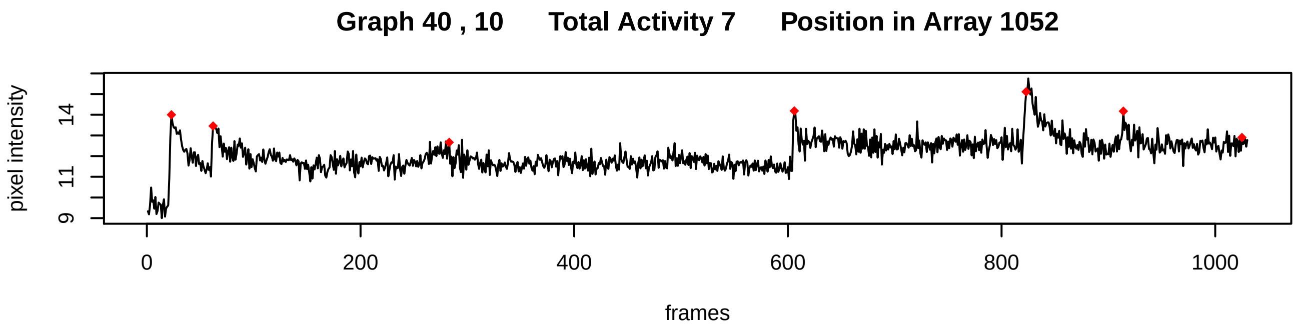
Graph 40 , 10 Total Activity 7 Position in Array 1052
pixel intensity
9
11
14
0
200
400
600
800
1000
frames

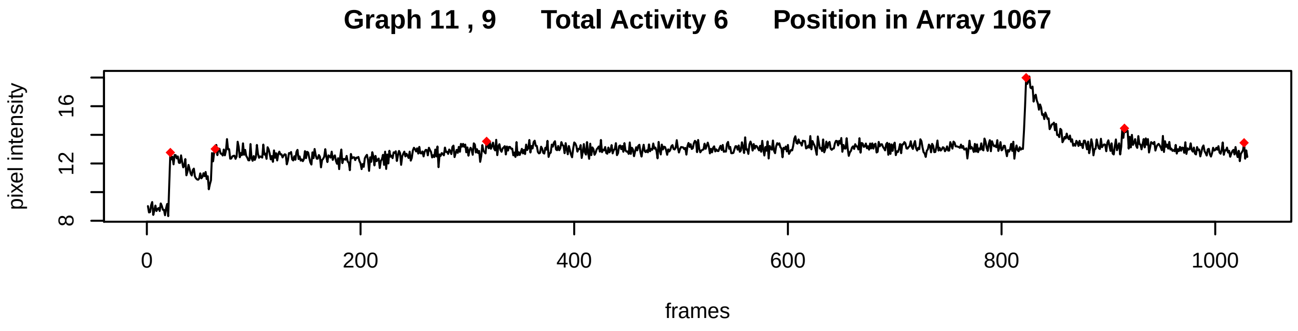
Graph 11 , 9 Total Activity 6 Position in Array 1067
pixel intensity
8
12
16
0
200
400
600
800
1000
frames

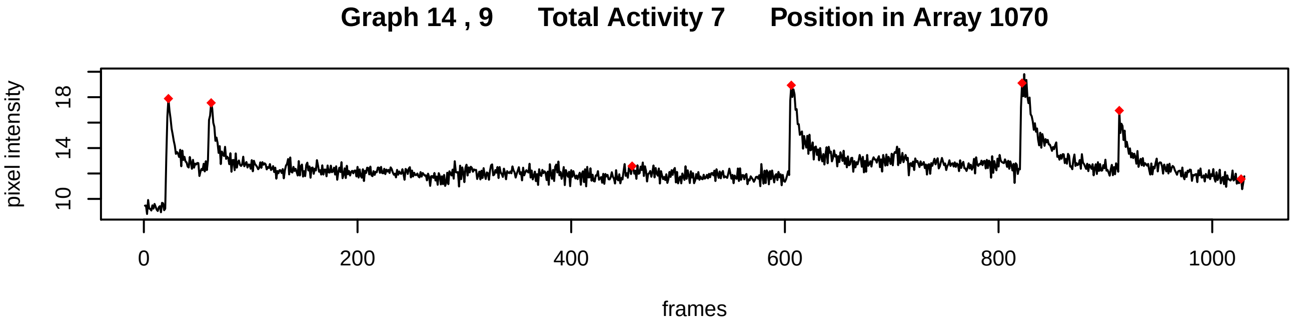
Graph 14 , 9 Total Activity 7 Position in Array 1070
pixel intensity
10
14
18
0
200
400
600
800
1000
frames

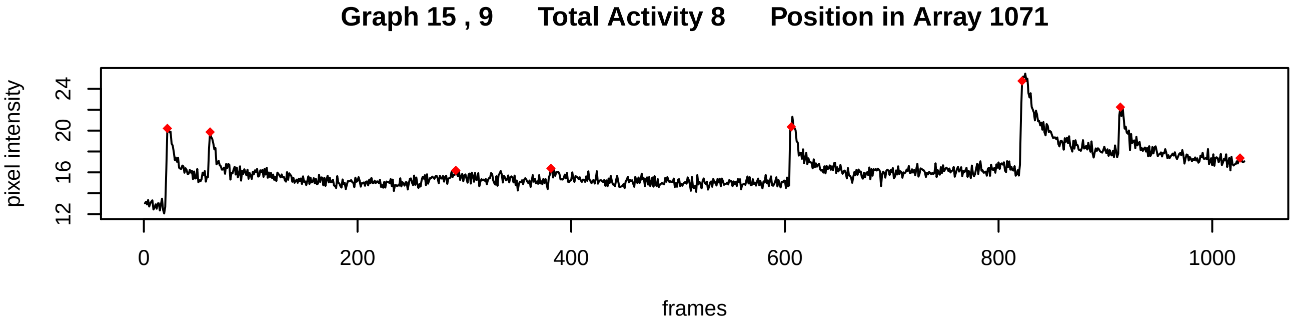
Graph 15 , 9 Total Activity 8 Position in Array 1071
pixel intensity
12
16
20
24
0
200
400
600
800
1000
frames

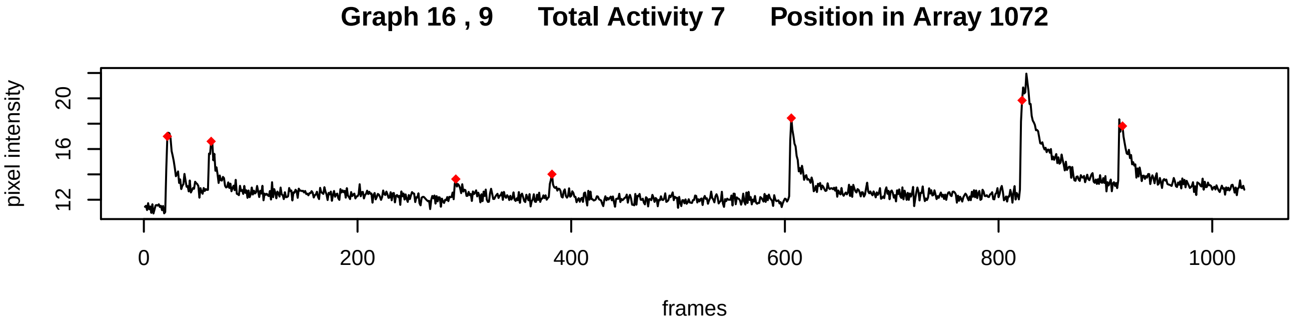
Graph 16 , 9 Total Activity 7 Position in Array 1072
pixel intensity
12
16
20
0
200
400
600
800
1000
frames

Graph 17 , 9 Total Activity 7 Position in Array 1073

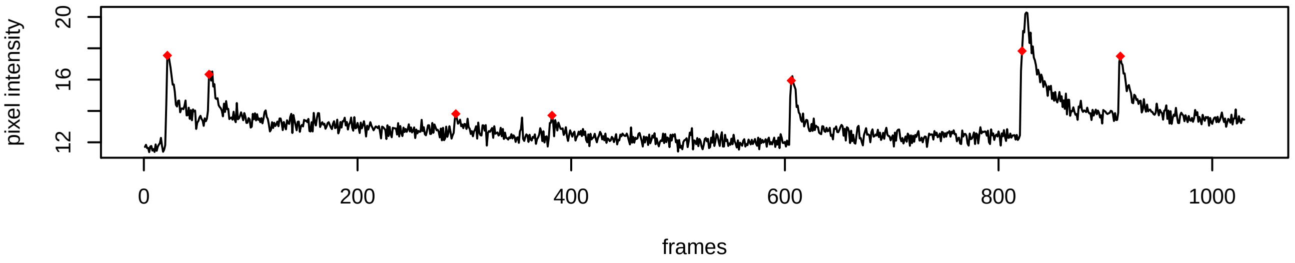

Graph 18 , 9 Total Activity 6 Position in Array 1074

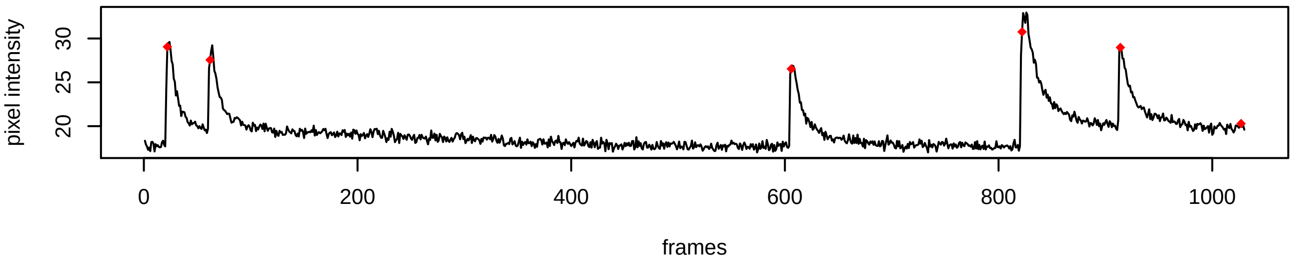

Graph 20 , 9 Total Activity 5 Position in Array 1076

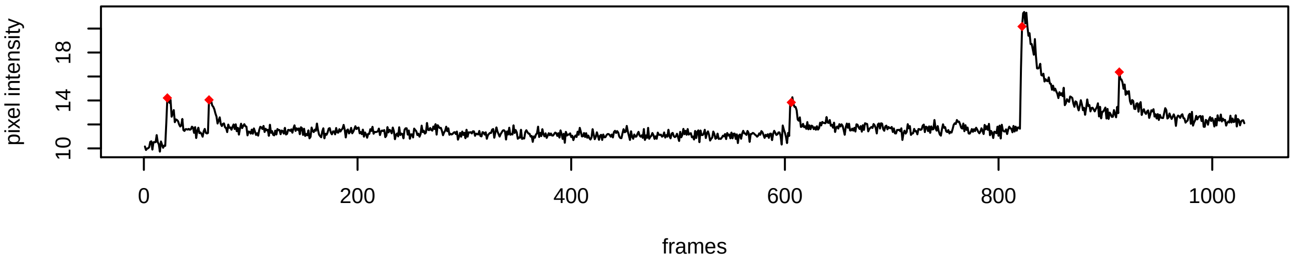

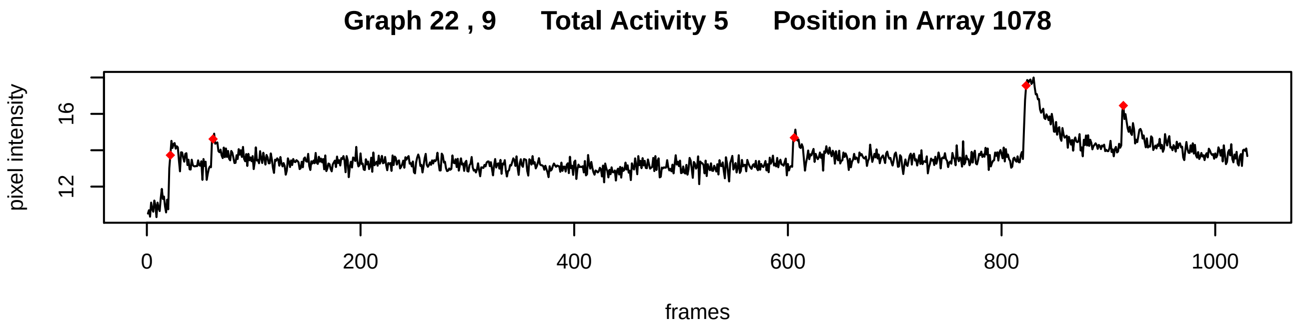
Graph 22 , 9 Total Activity 5 Position in Array 1078
pixel intensity
12
16
0
200
400
600
800
1000
frames

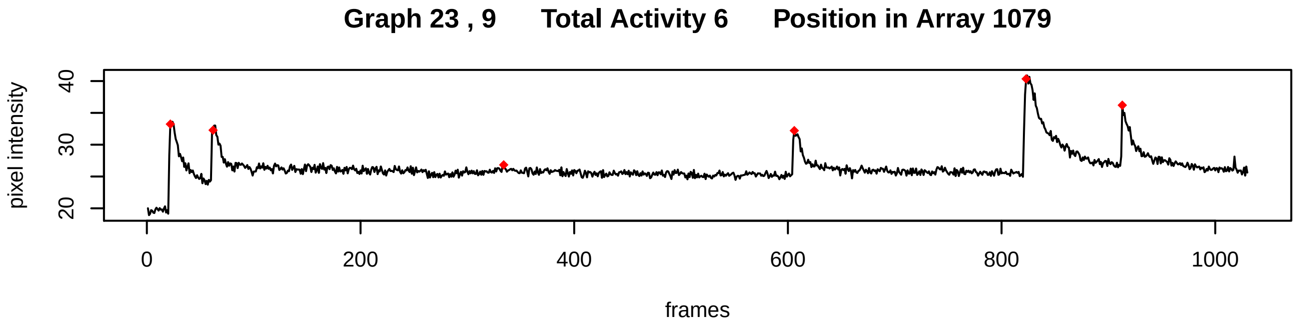
Graph 23 , 9 Total Activity 6 Position in Array 1079
pixel intensity
20
30
40
0
200
400
600
800
1000
frames

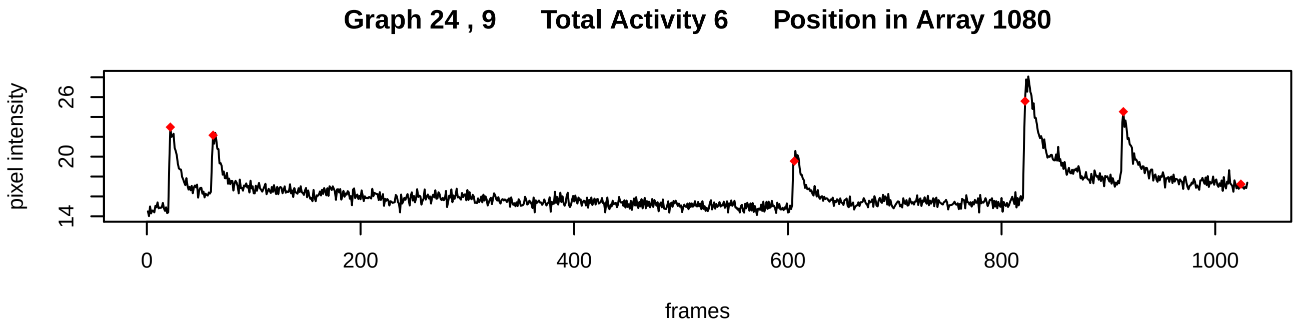
Graph 24 , 9 Total Activity 6 Position in Array 1080
pixel intensity
14
20
26
0
200
400
600
800
1000
frames

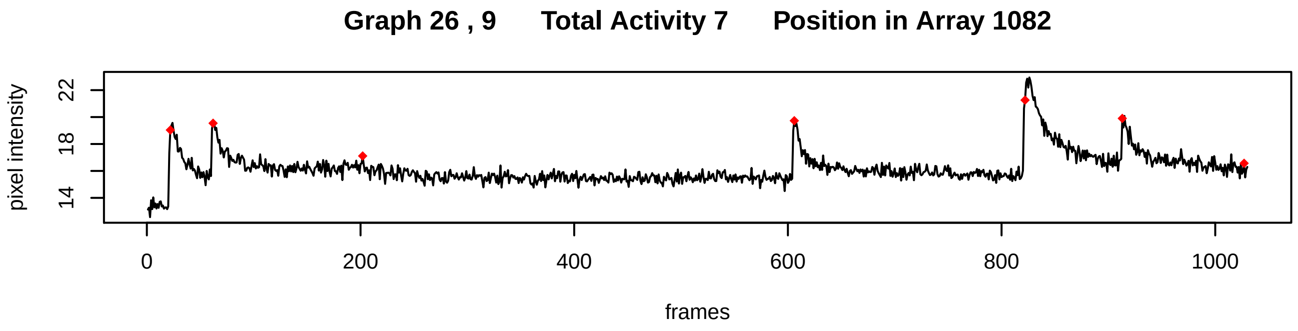

Graph 26 , 9 Total Activity 7 Position in Array 1082
pixel intensity
14
18
22
0
200
400
600
800
1000
frames

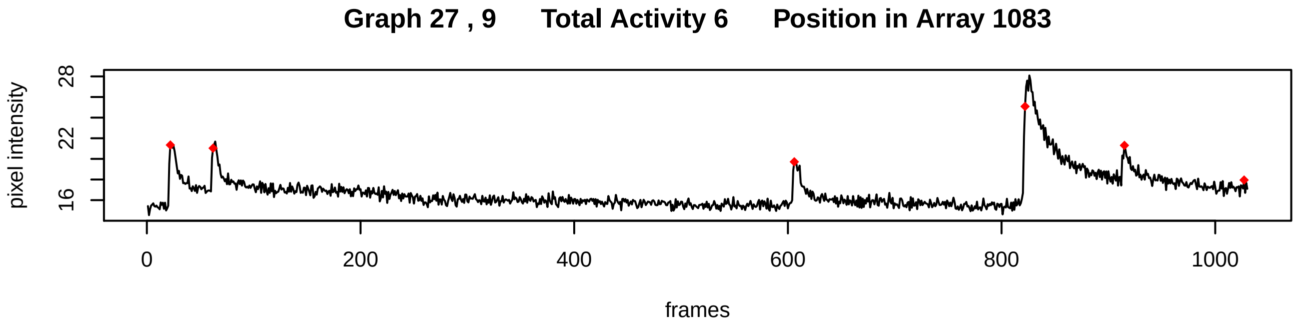

Graph 27 , 9 Total Activity 6 Position in Array 1083
pixel intensity
16
22
28
0
200
400
600
800
1000
frames

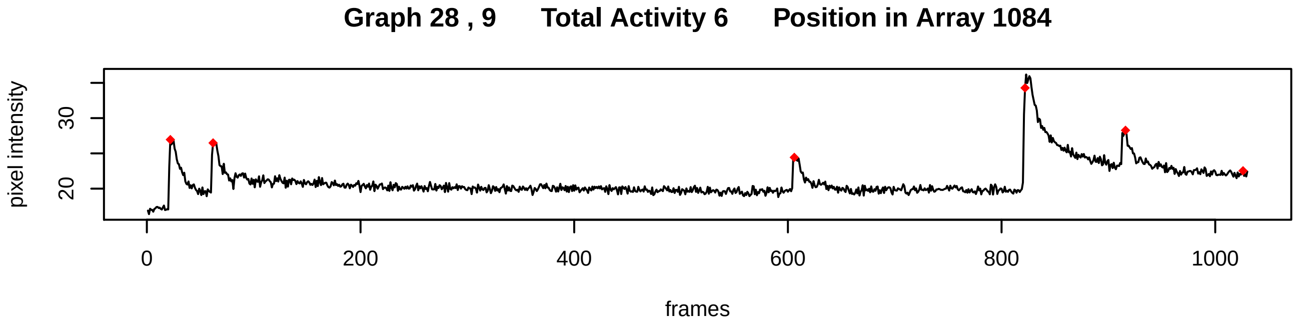

Graph 28 , 9 Total Activity 6 Position in Array 1084
pixel intensity
20
30
0
200
400
600
800
1000
frames

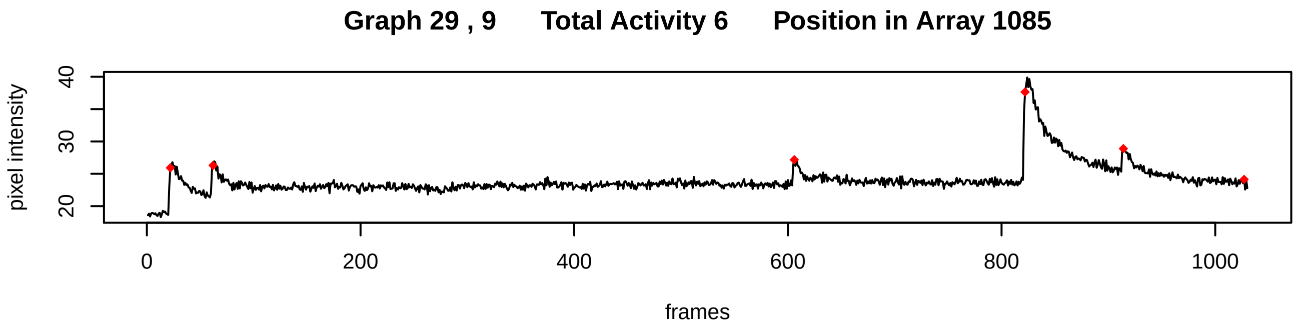
Graph 29 , 9 Total Activity 6 Position in Array 1085
pixel intensity
20
30
40
0
200
400
600
800
1000
frames

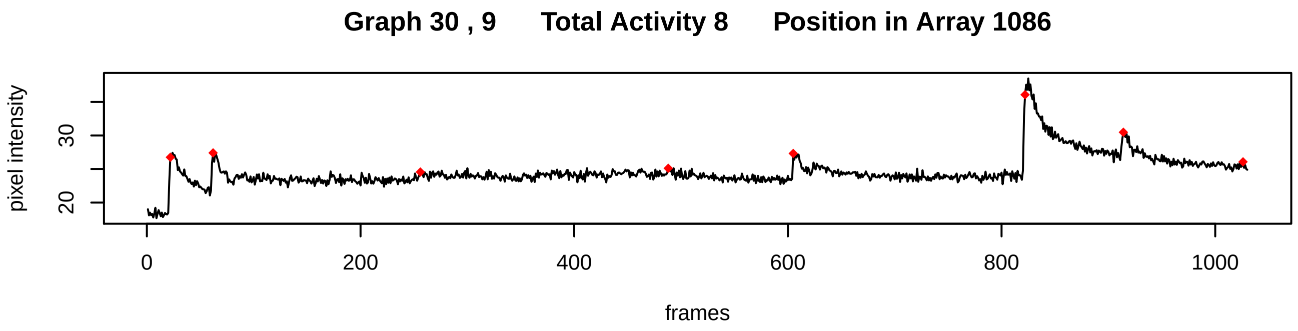
Graph 30 , 9 Total Activity 8 Position in Array 1086
pixel intensity
20
30
0
200
400
600
800
1000
frames

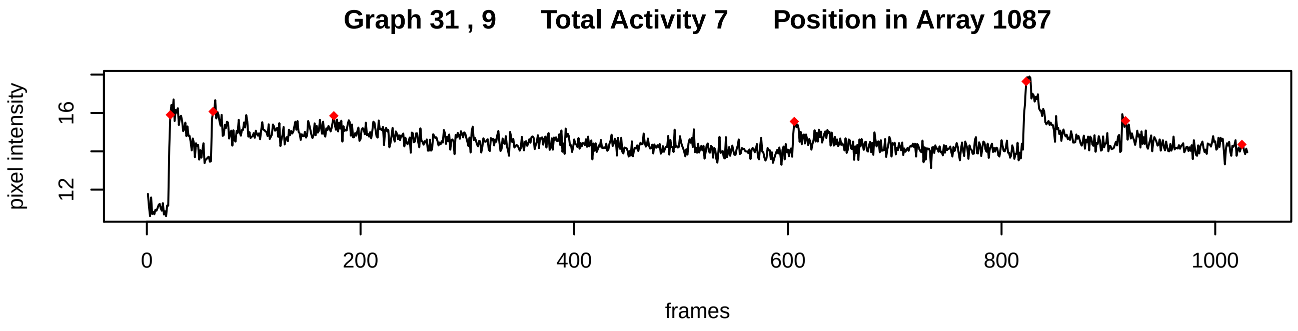
Graph 31 , 9 Total Activity 7 Position in Array 1087
pixel intensity
12
16
0
200
400
600
800
1000
frames

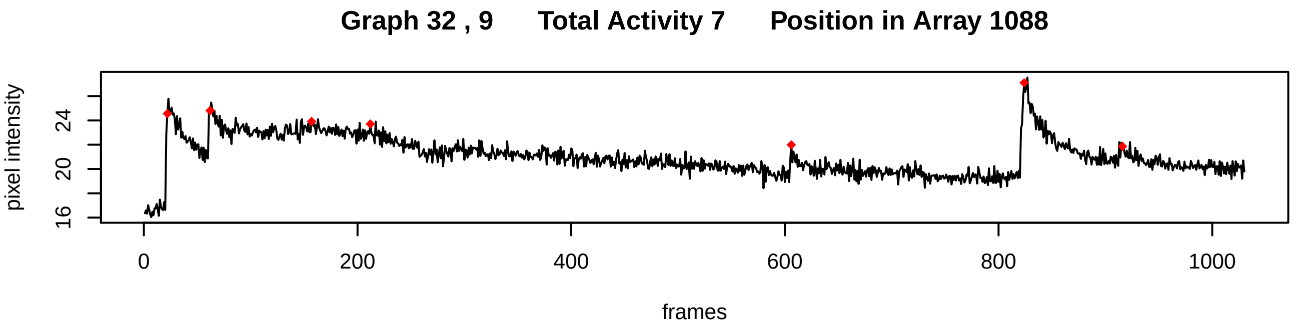
Graph 32 , 9 Total Activity 7 Position in Array 1088
pixel intensity
16
20
24
0
200
400
600
800
1000
frames

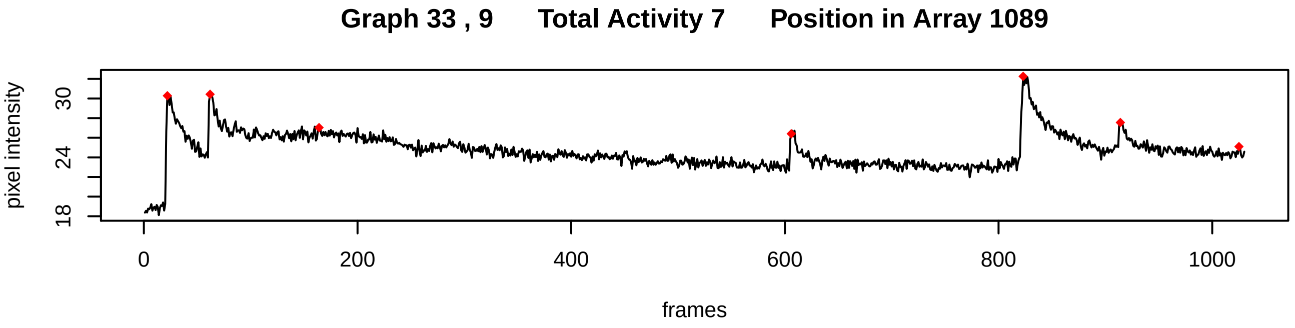
Graph 33 , 9 Total Activity 7 Position in Array 1089
pixel intensity
18
24
30
0
200
400
600
800
1000
frames

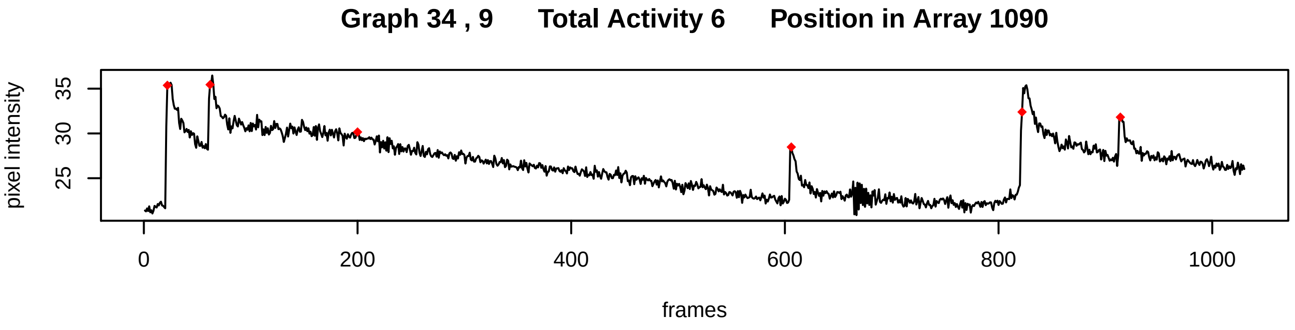
Graph 34 , 9 Total Activity 6 Position in Array 1090
pixel intensity
25
30
35
0
200
400
600
800
1000
frames

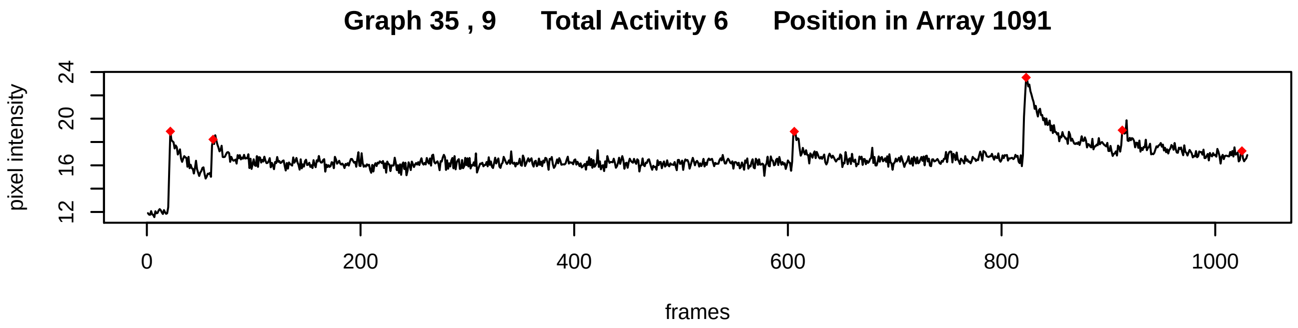
Graph 35 , 9 Total Activity 6 Position in Array 1091
pixel intensity
12
16
20
24
0
200
400
600
800
1000
frames

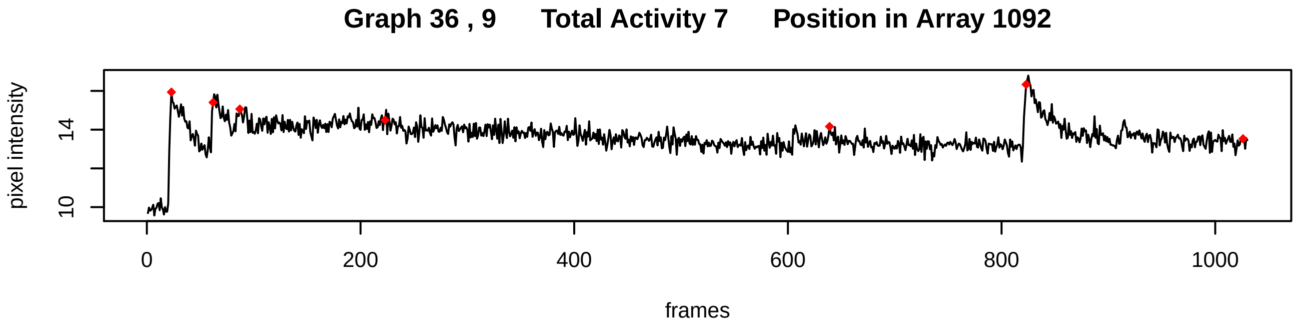
Graph 36 , 9 Total Activity 7 Position in Array 1092
pixel intensity
10
14
0
200
400
600
800
1000
frames

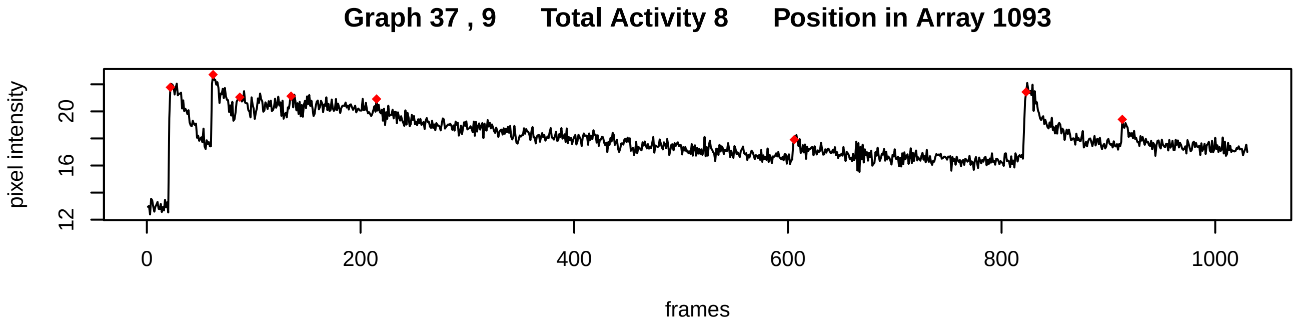
Graph 37 , 9 Total Activity 8 Position in Array 1093
pixel intensity
12
16
20
0
200
400
600
800
1000
frames

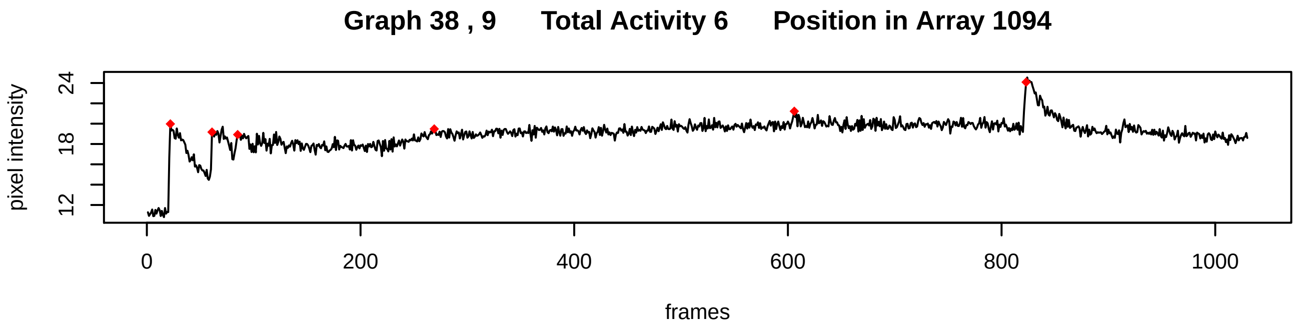
Graph 38 , 9 Total Activity 6 Position in Array 1094
pixel intensity
12
18
24
0
200
400
600
800
1000
frames

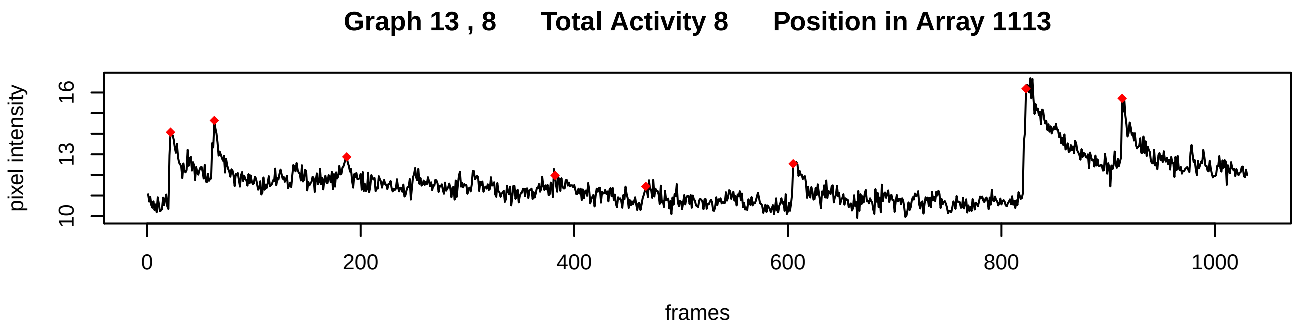
Graph 13 , 8 Total Activity 8 Position in Array 1113
pixel intensity
10
13
16
0
200
400
600
800
1000
frames

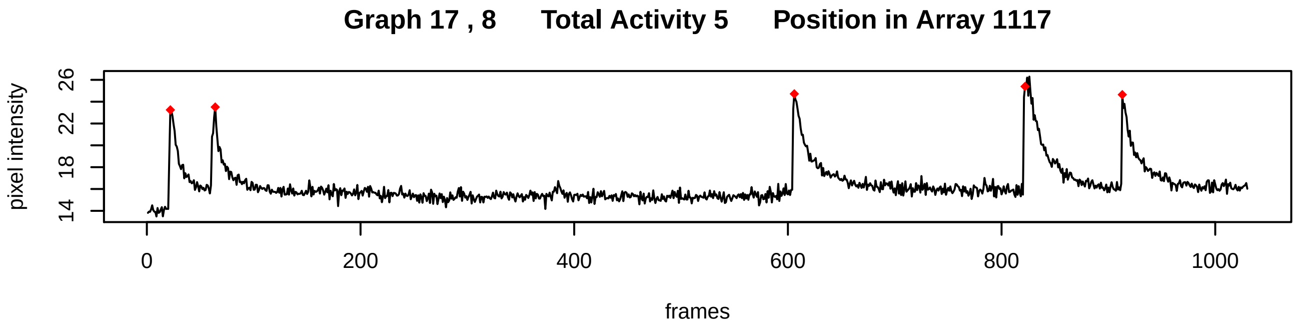
Graph 17 , 8 Total Activity 5 Position in Array 1117
pixel intensity
14
18
22
26
0
200
400
600
800
1000
frames

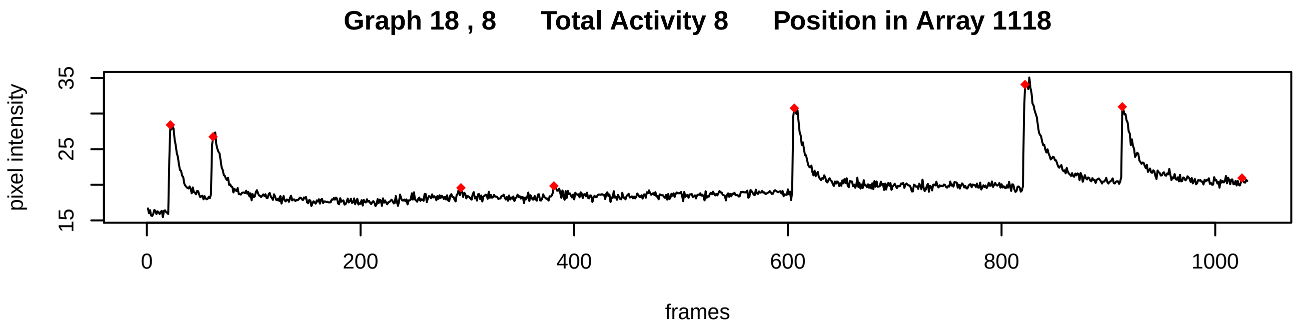
Graph 18 , 8 Total Activity 8 Position in Array 1118
pixel intensity
15
25
35
0
200
400
600
800
1000
frames

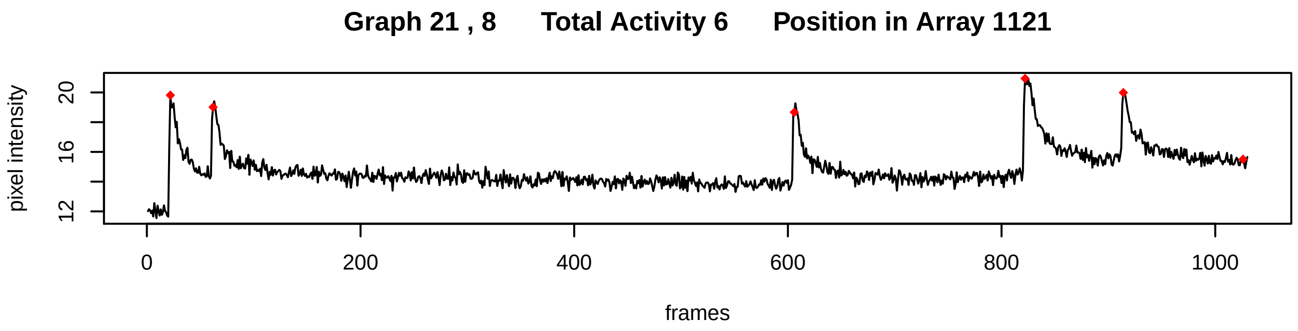
Graph 21 , 8 Total Activity 6 Position in Array 1121
pixel intensity
12
16
20
0
200
400
600
800
1000
frames

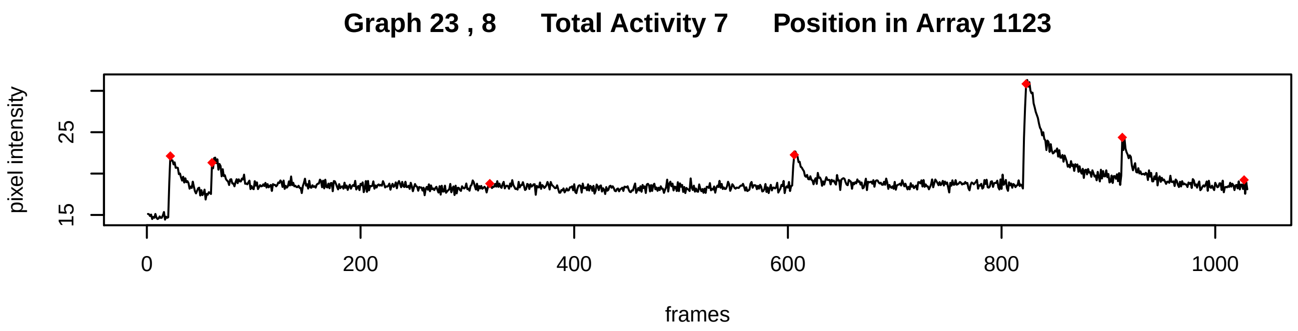
Graph 23 , 8 Total Activity 7 Position in Array 1123
pixel intensity
15
25
0
200
400
600
800
1000
frames

Graph 24 , 8   Total Activity 6   Position in Array 1124

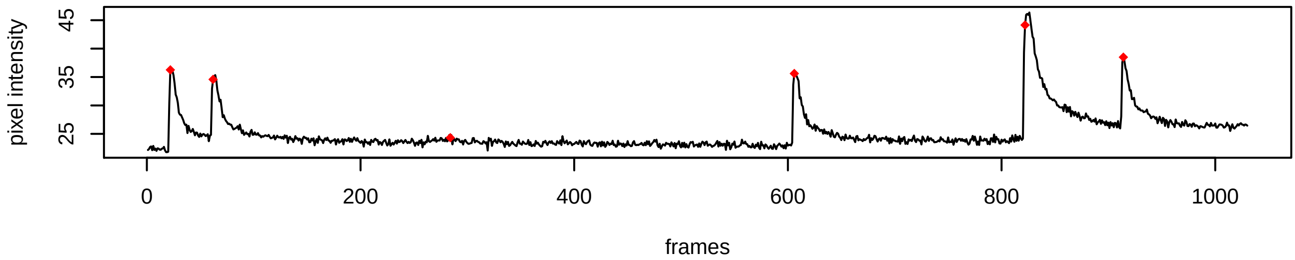

Graph 25 , 8   Total Activity 5   Position in Array 1125

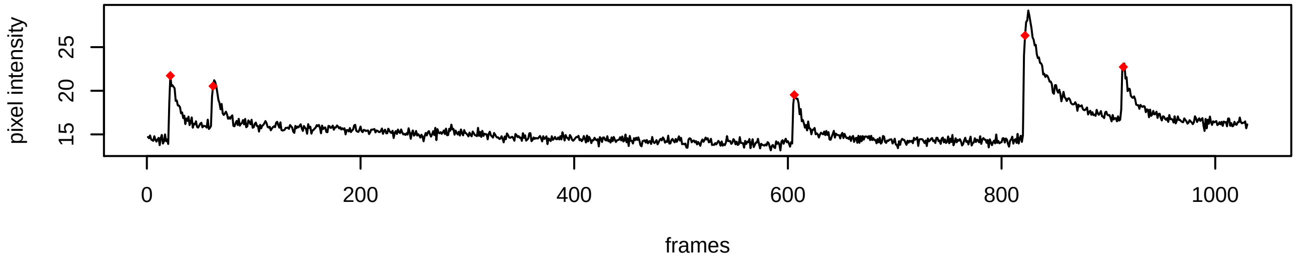

Graph 26 , 8   Total Activity 6   Position in Array 1126

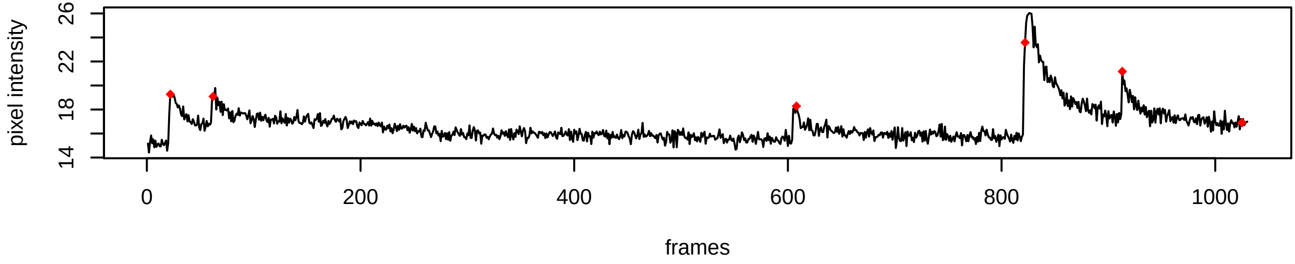

Graph 27 , 8   Total Activity 6   Position in Array 1127

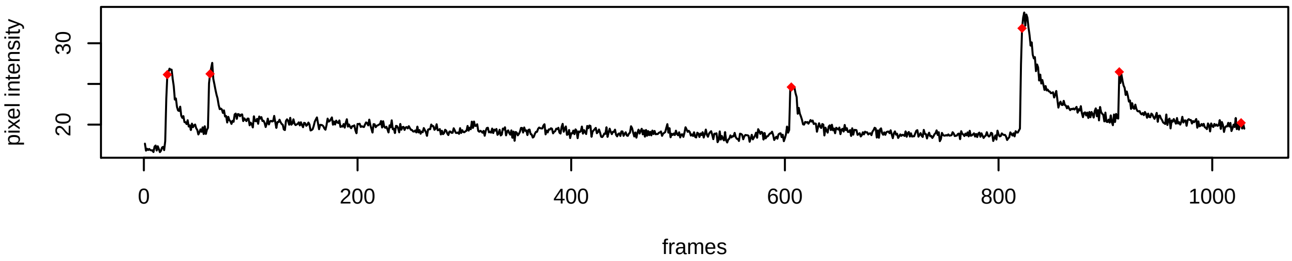

Graph 28 , 8   Total Activity 7   Position in Array 1128

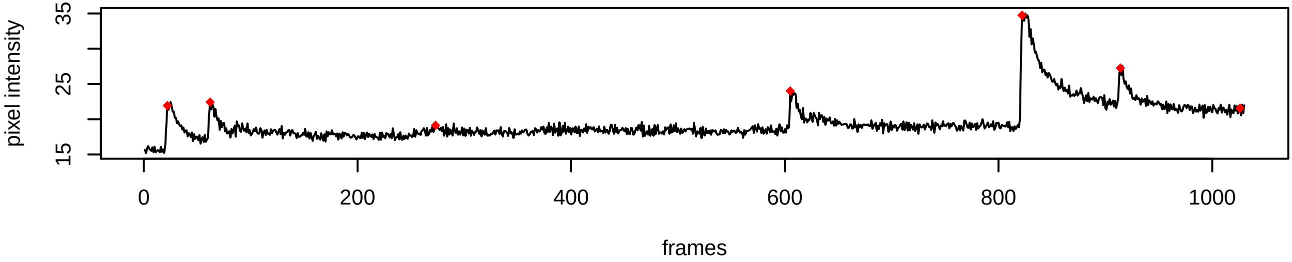

Graph 30 , 8   Total Activity 6   Position in Array 1130

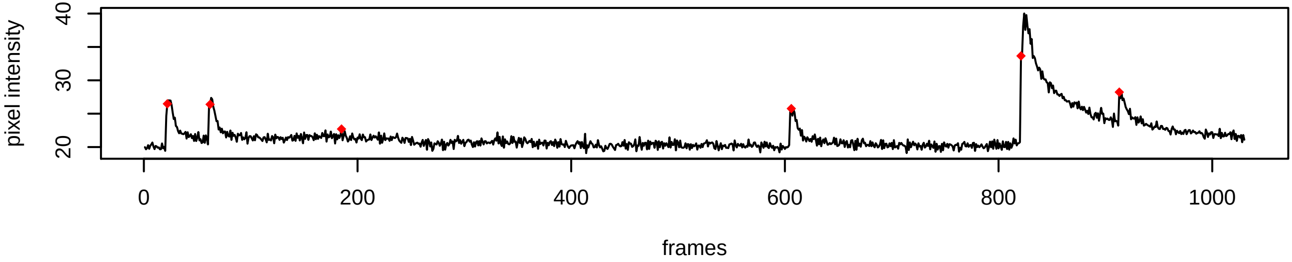

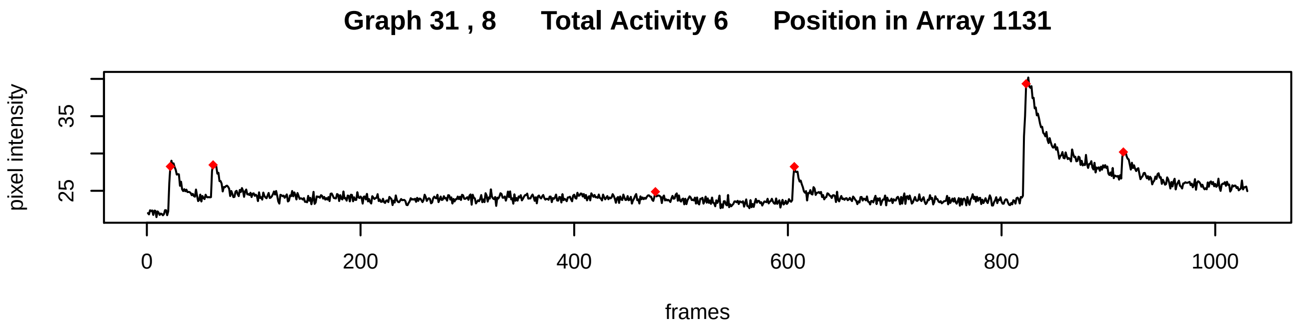
Graph 31 , 8 Total Activity 6 Position in Array 1131
pixel intensity
25
35
0
200
400
600
800
1000
frames

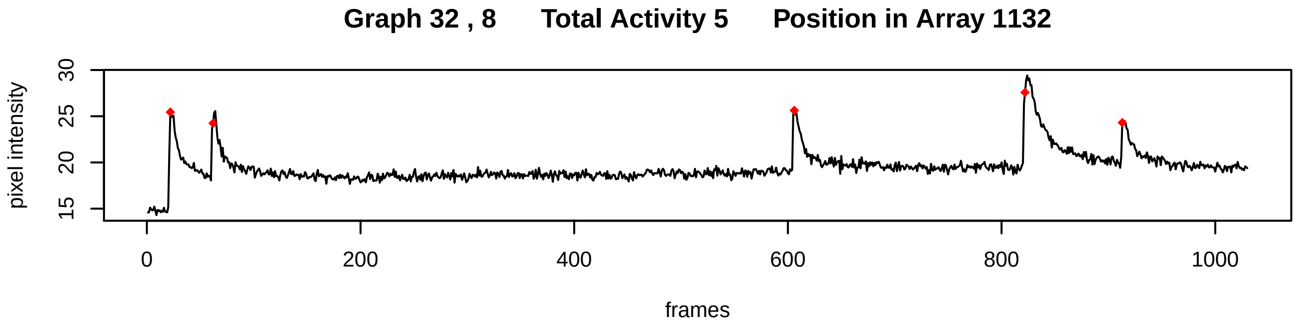
Graph 32 , 8 Total Activity 5 Position in Array 1132
pixel intensity
15
20
25
30
0
200
400
600
800
1000
frames

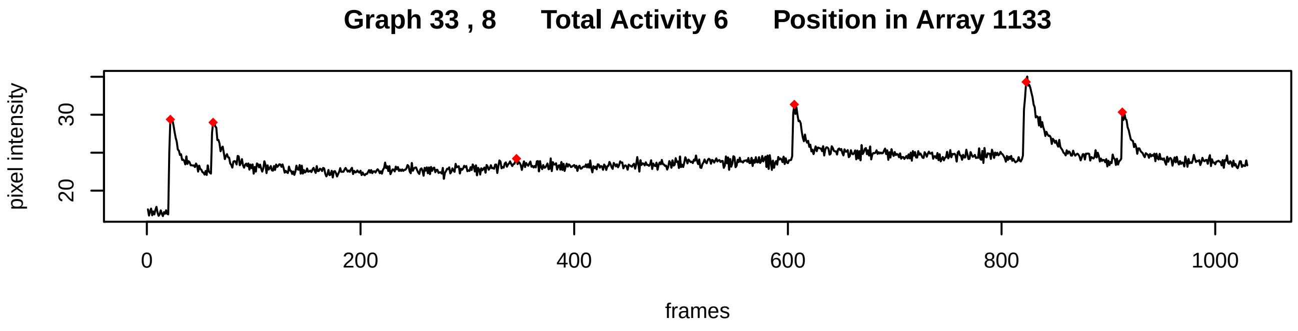
Graph 33 , 8 Total Activity 6 Position in Array 1133
pixel intensity
20
30
0
200
400
600
800
1000
frames

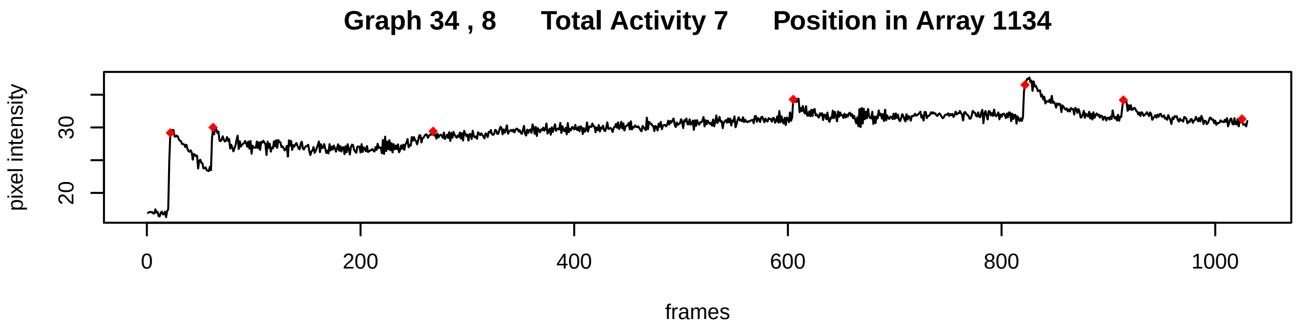
Graph 34 , 8 Total Activity 7 Position in Array 1134
pixel intensity
20
30
0
200
400
600
800
1000
frames

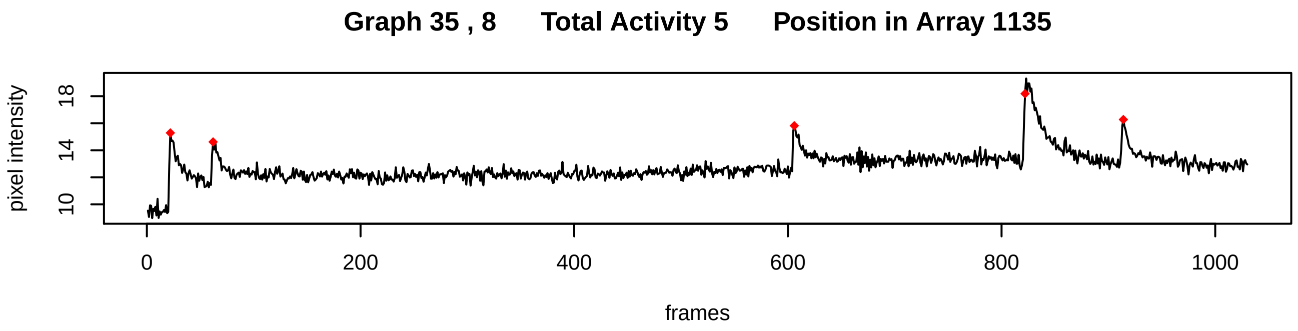
Graph 35 , 8 Total Activity 5 Position in Array 1135
pixel intensity
10
14
18
0
200
400
600
800
1000
frames

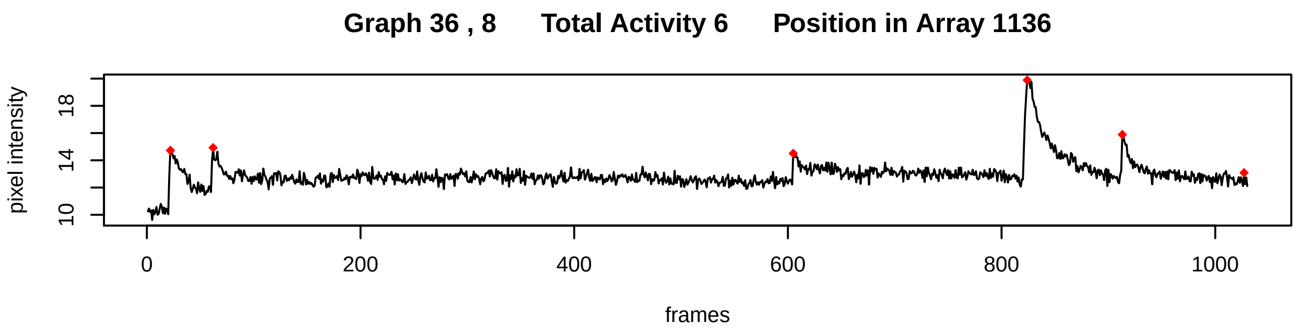
Graph 36 , 8 Total Activity 6 Position in Array 1136
pixel intensity
10
14
18
0
200
400
600
800
1000
frames

Graph 37 , 8   Total Activity 6   Position in Array 1137

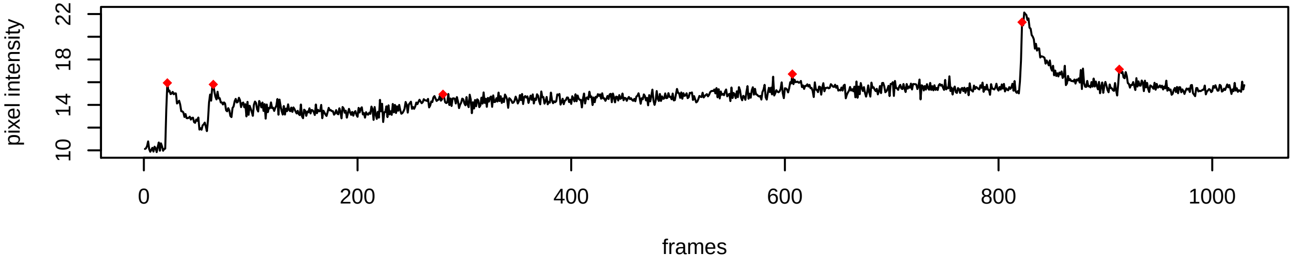

Graph 42 , 8   Total Activity 5   Position in Array 1142

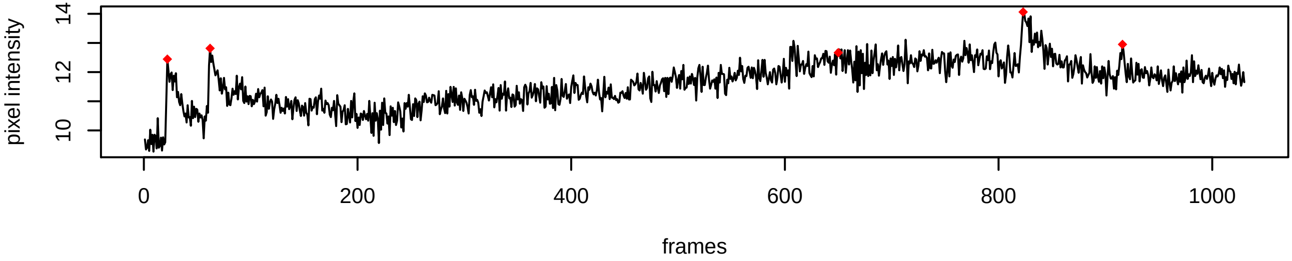

Graph 13 , 7   Total Activity 6   Position in Array 1157

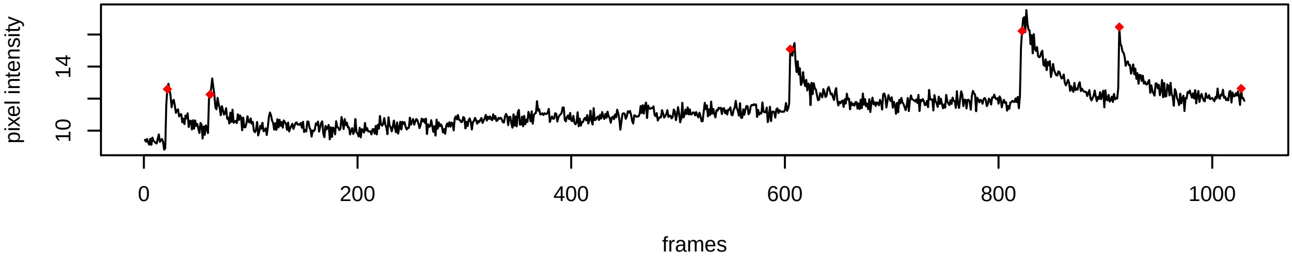

Graph 14 , 7   Total Activity 7   Position in Array 1158

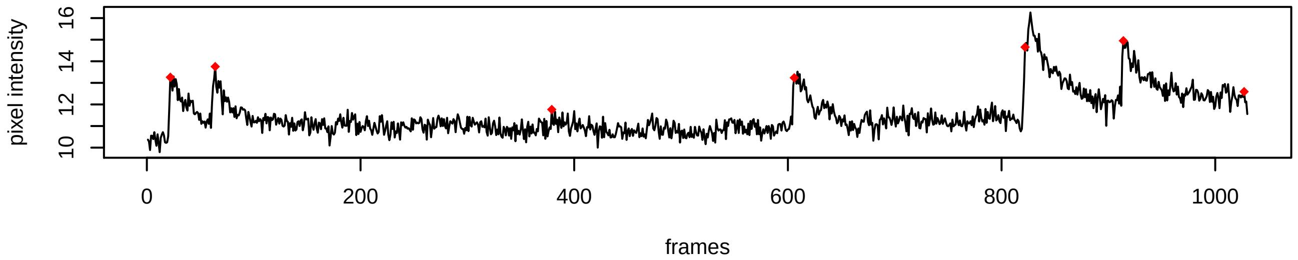

Graph 19 , 7   Total Activity 6   Position in Array 1163

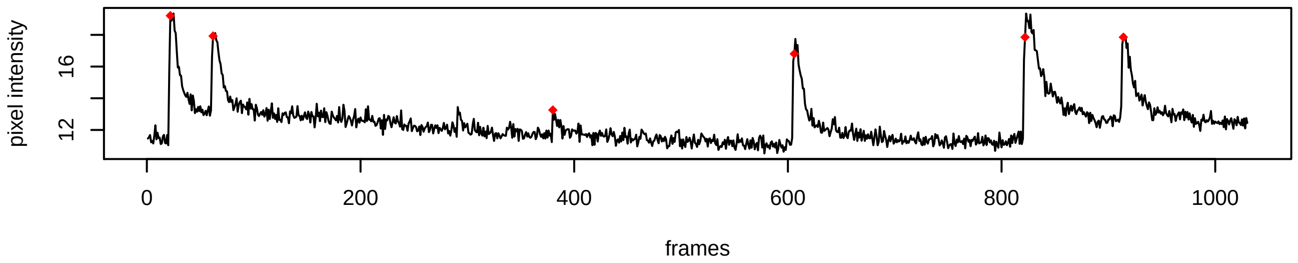

Graph 20 , 7   Total Activity 5   Position in Array 1164

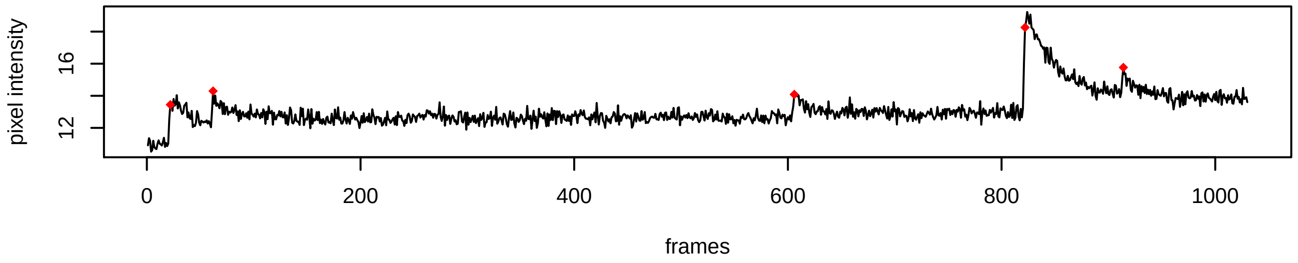

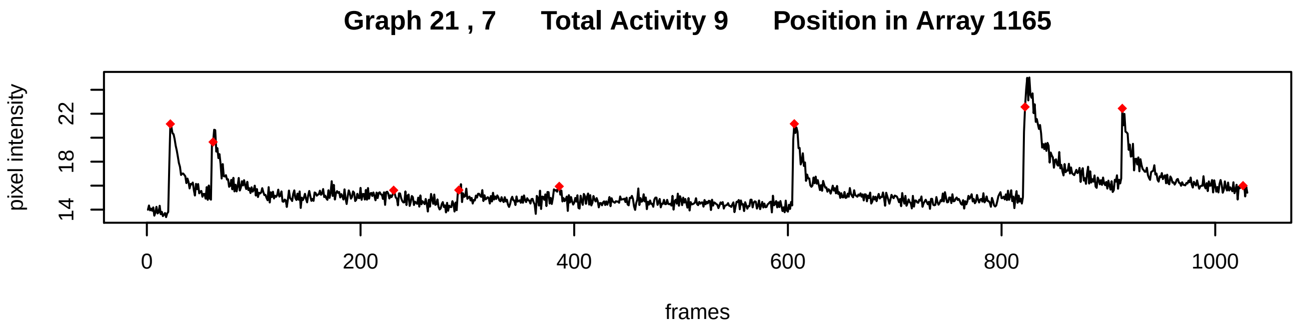
Graph 21 , 7 Total Activity 9 Position in Array 1165
pixel intensity
14
18
22
0
200
400
600
800
1000
frames

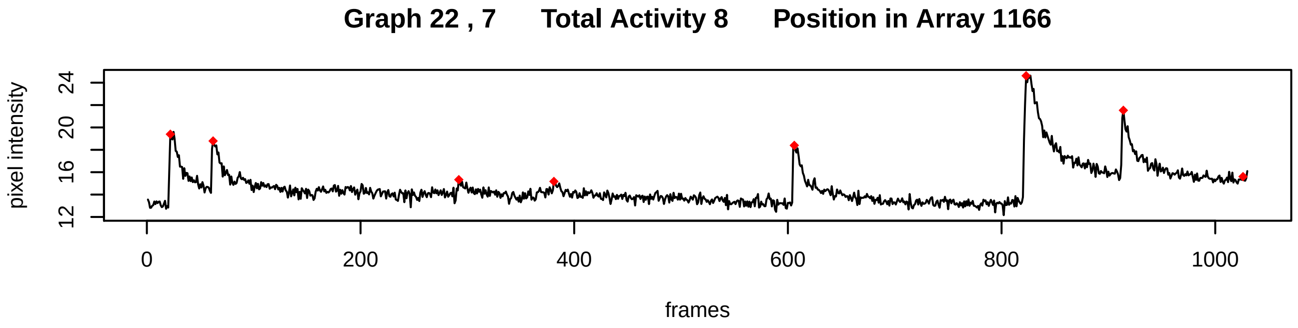
Graph 22 , 7 Total Activity 8 Position in Array 1166
pixel intensity
12
16
20
24
0
200
400
600
800
1000
frames

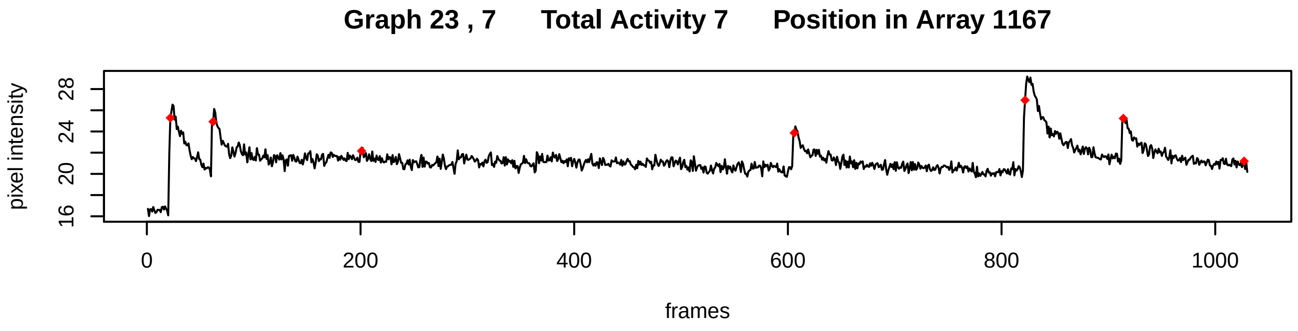
Graph 23 , 7 Total Activity 7 Position in Array 1167
pixel intensity
16
20
24
28
0
200
400
600
800
1000
frames

Graph 24 , 7   Total Activity 7   Position in Array 1168

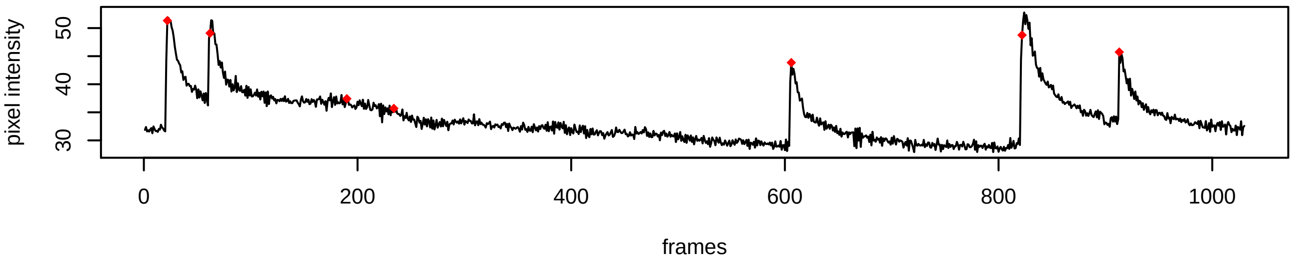

Graph 25 , 7   Total Activity 9   Position in Array 1169

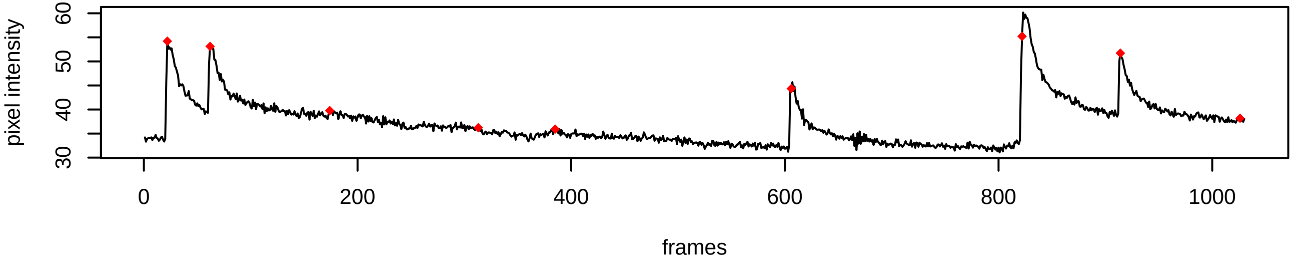

Graph 26 , 7   Total Activity 9   Position in Array 1170

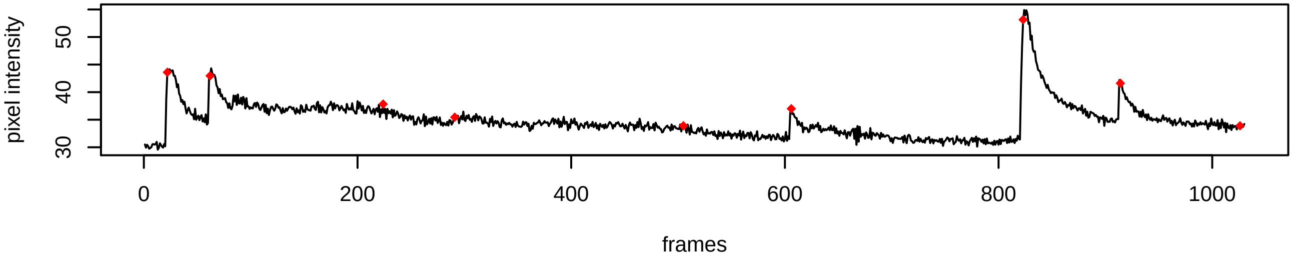

Graph 27 , 7   Total Activity 8   Position in Array 1171

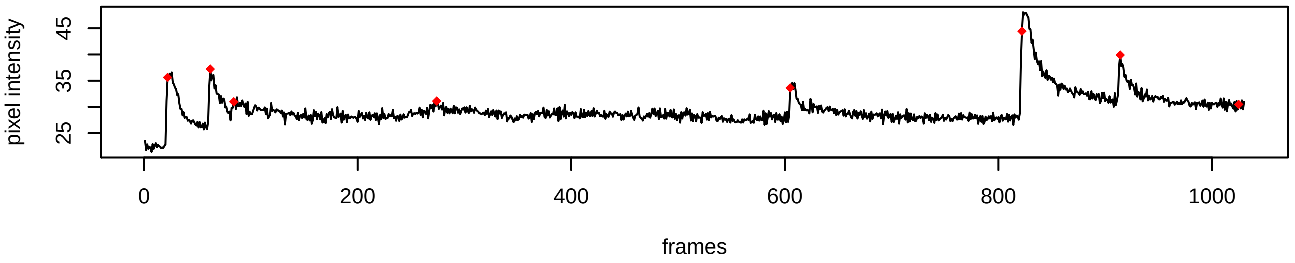

Graph 28 , 7   Total Activity 5   Position in Array 1172

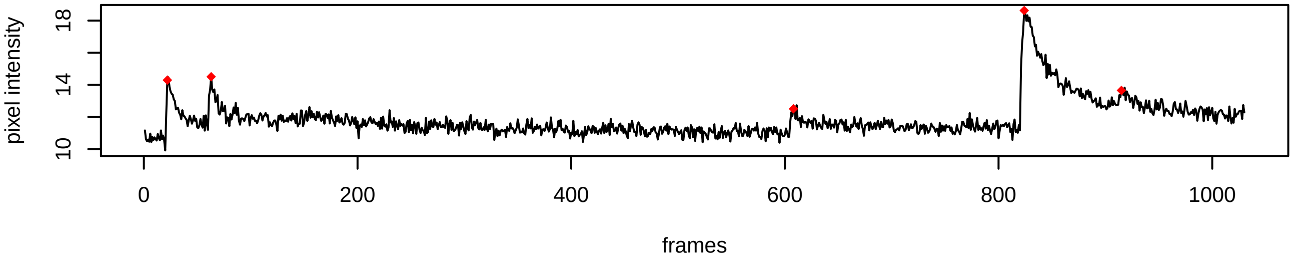

Graph 29 , 7   Total Activity 6   Position in Array 1173

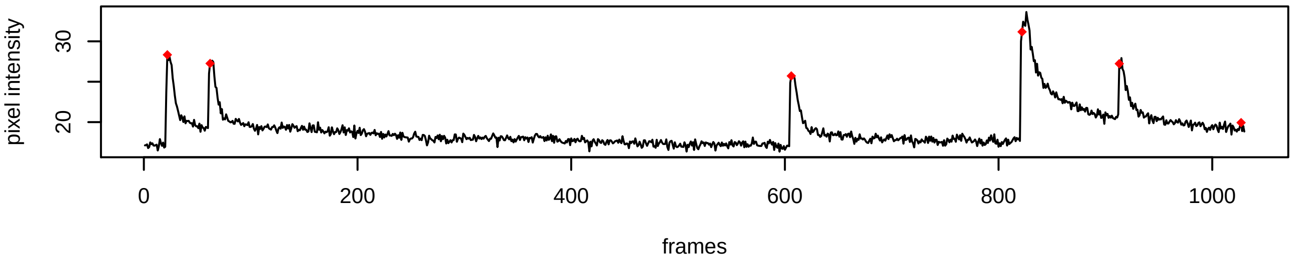

Graph 30 , 7 Total Activity 6 Position in Array 1174

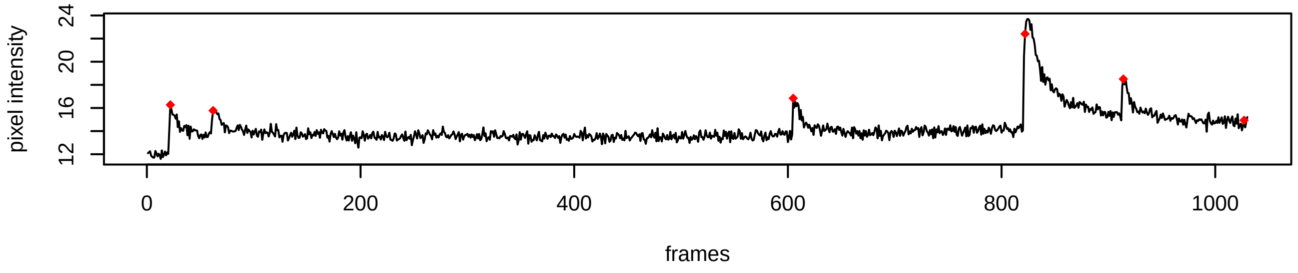

Graph 31 , 7 Total Activity 7 Position in Array 1175

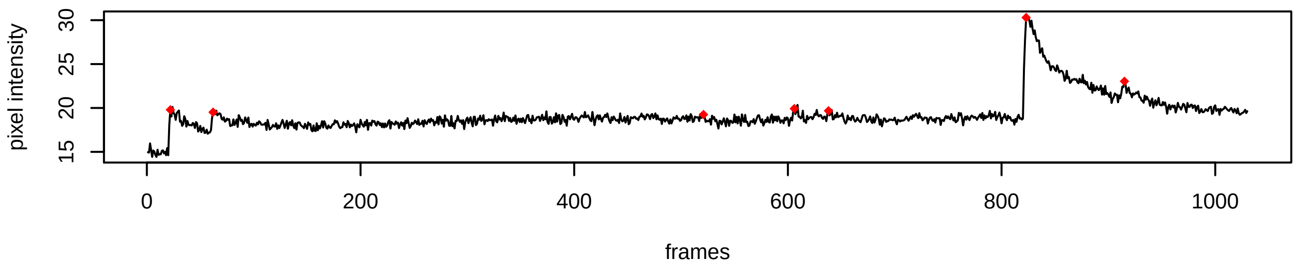

Graph 32 , 7 Total Activity 7 Position in Array 1176

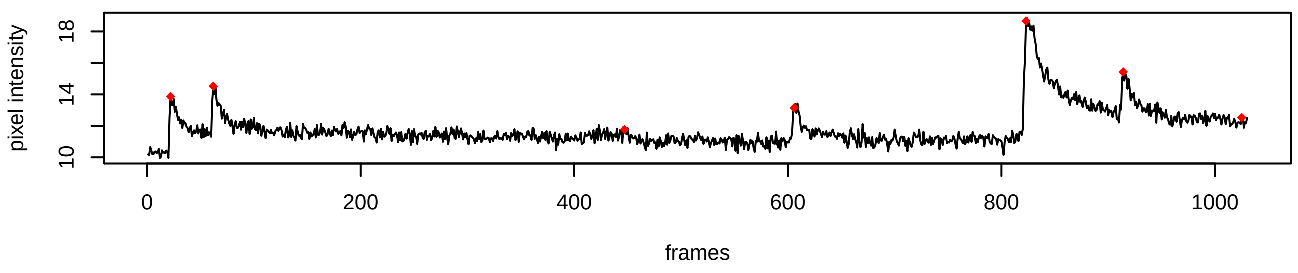

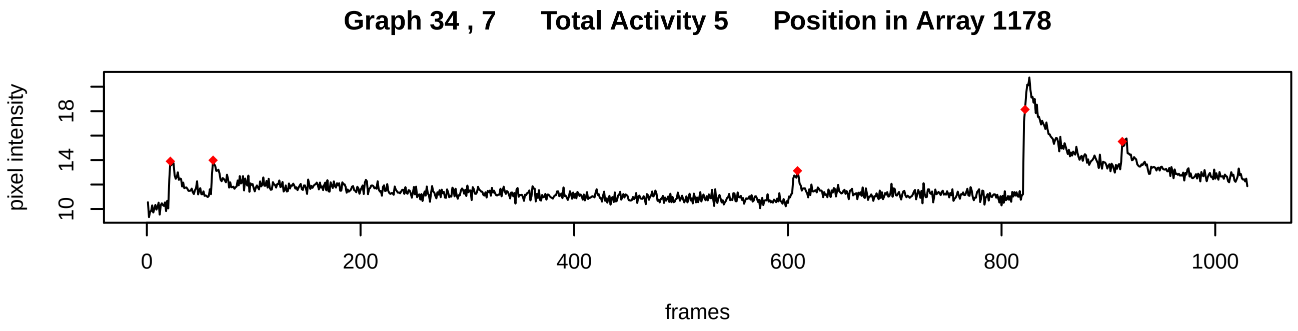
Graph 34 , 7 Total Activity 5 Position in Array 1178
pixel intensity
10
14
18
0
200
400
600
800
1000
frames

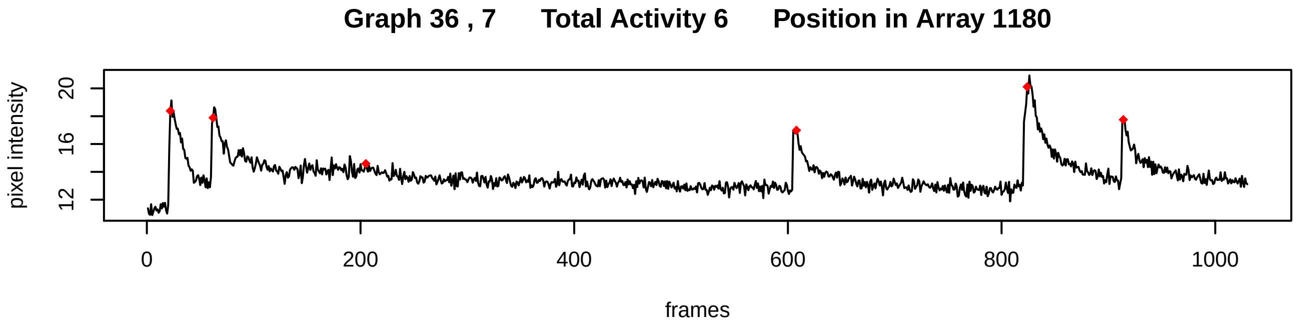
Graph 36 , 7 Total Activity 6 Position in Array 1180
pixel intensity
12
16
20
0
200
400
600
800
1000
frames

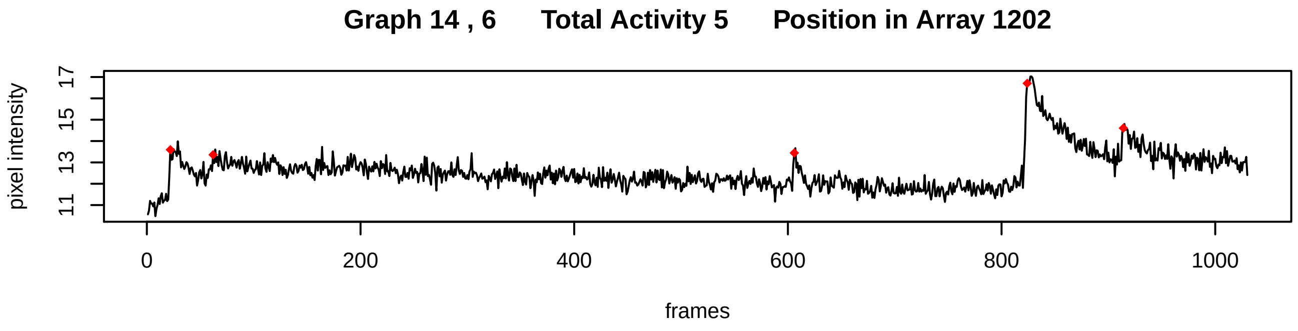
Graph 14 , 6 Total Activity 5 Position in Array 1202
pixel intensity
11
13
15
17
0
200
400
600
800
1000
frames

Graph 16 , 6   Total Activity 8   Position in Array 1204

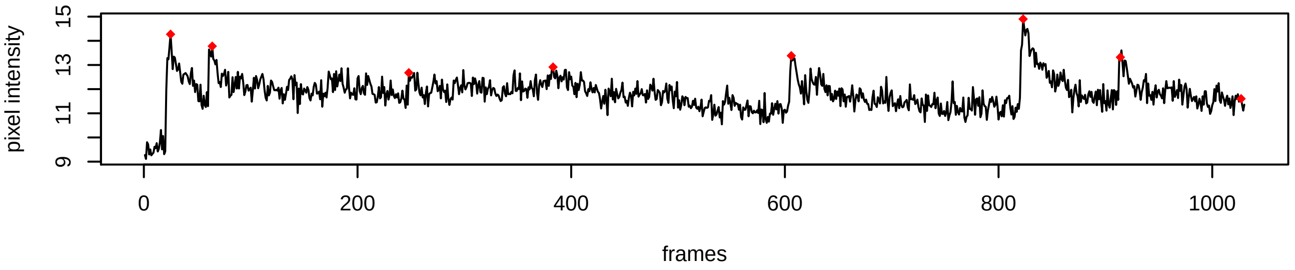

Graph 17 , 6   Total Activity 7   Position in Array 1205

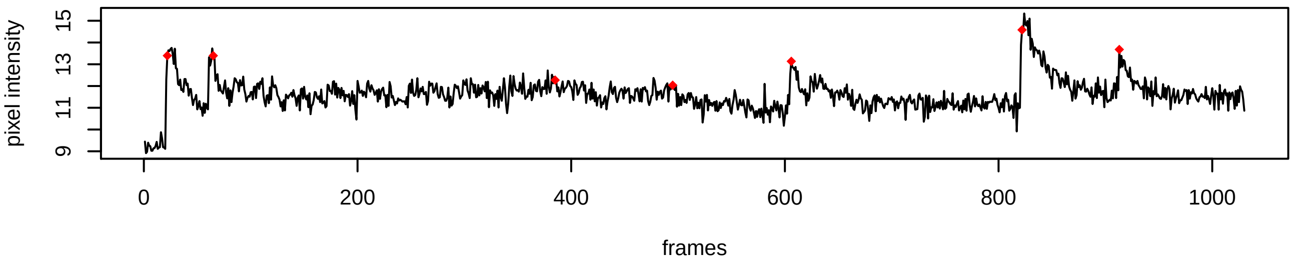

Graph 18 , 6   Total Activity 6   Position in Array 1206

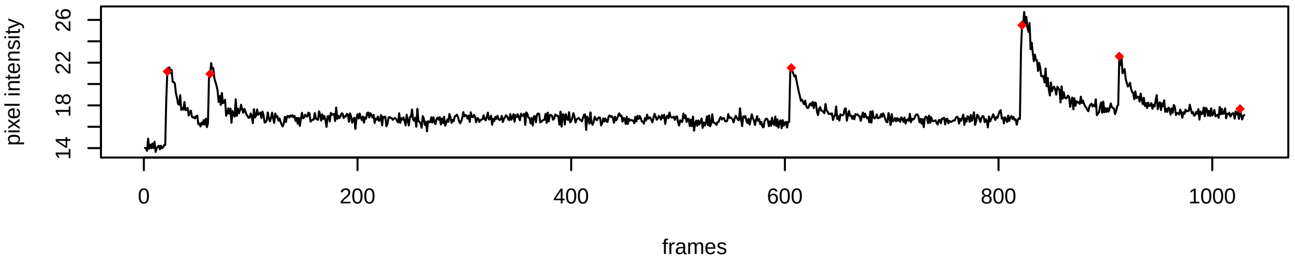

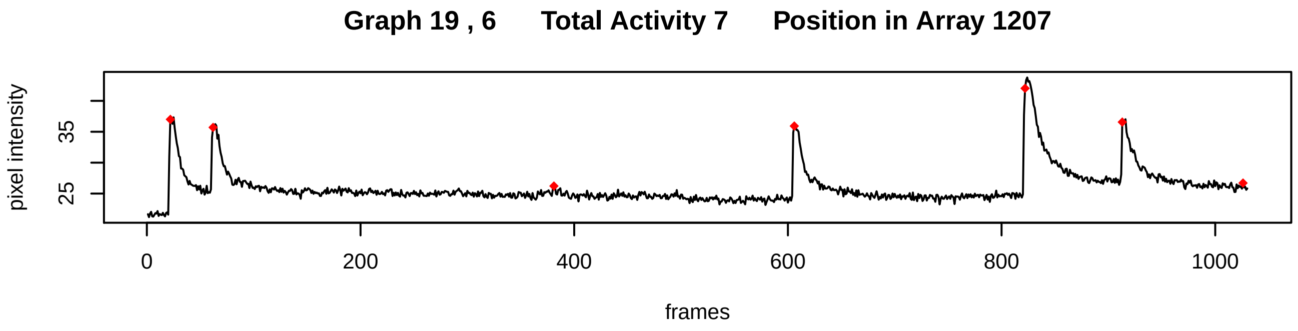
Graph 19 , 6 Total Activity 7 Position in Array 1207
pixel intensity
25
35
0
200
400
600
800
1000
frames

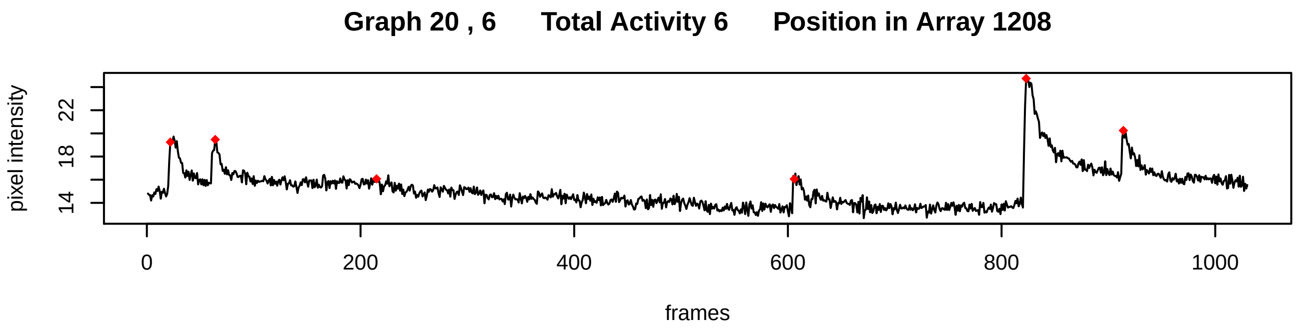
Graph 20 , 6 Total Activity 6 Position in Array 1208
pixel intensity
14
18
22
0
200
400
600
800
1000
frames

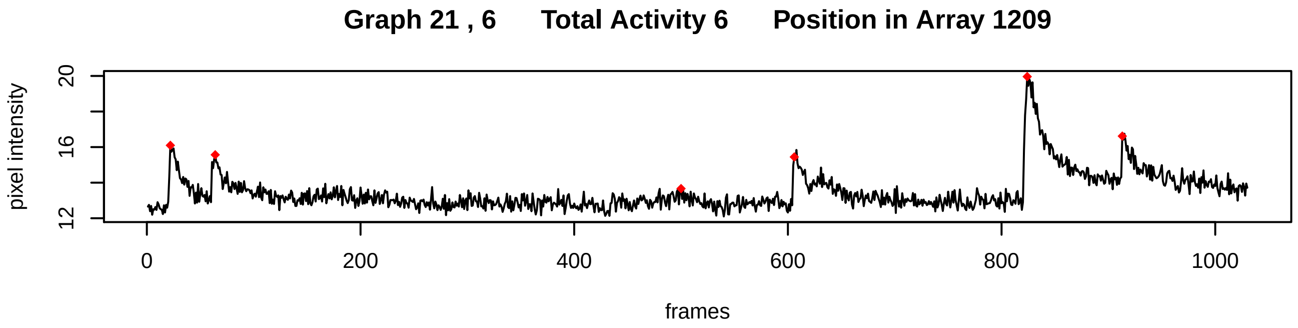
Graph 21 , 6 Total Activity 6 Position in Array 1209
pixel intensity
12
16
20
0
200
400
600
800
1000
frames

Graph 22 , 6   Total Activity 6   Position in Array 1210

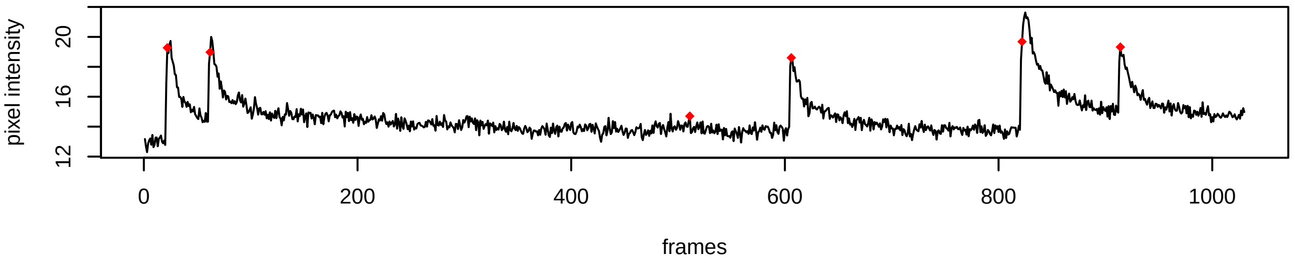

Graph 23 , 6   Total Activity 6   Position in Array 1211

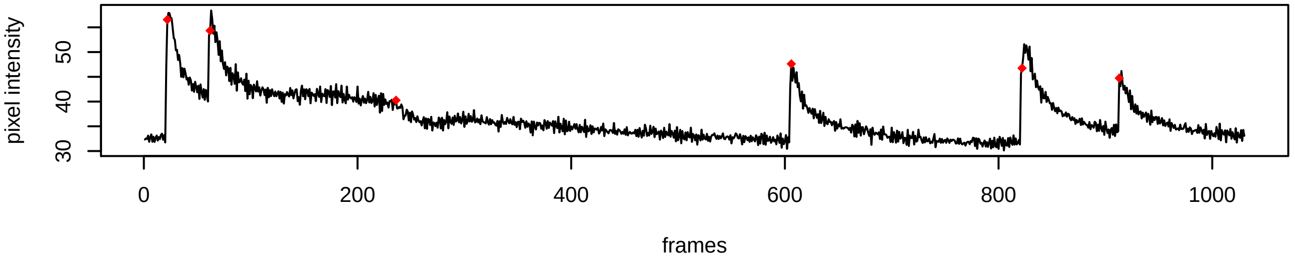

Graph 24 , 6   Total Activity 5   Position in Array 1212

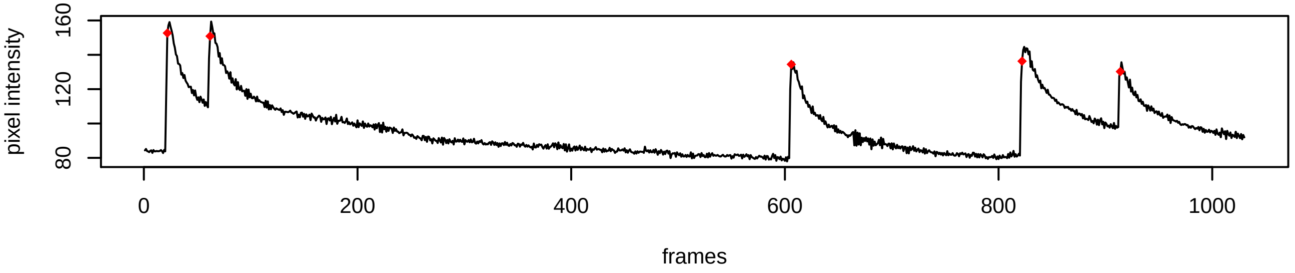

Graph 25 , 6   Total Activity 4   Position in Array 1213

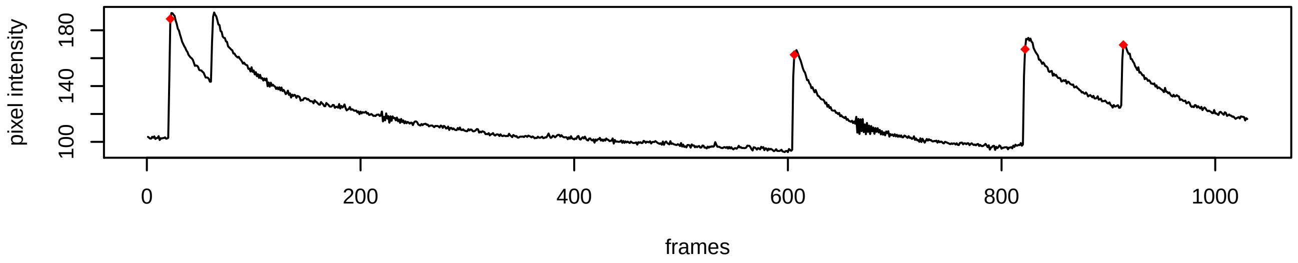

Graph 26 , 6   Total Activity 6   Position in Array 1214

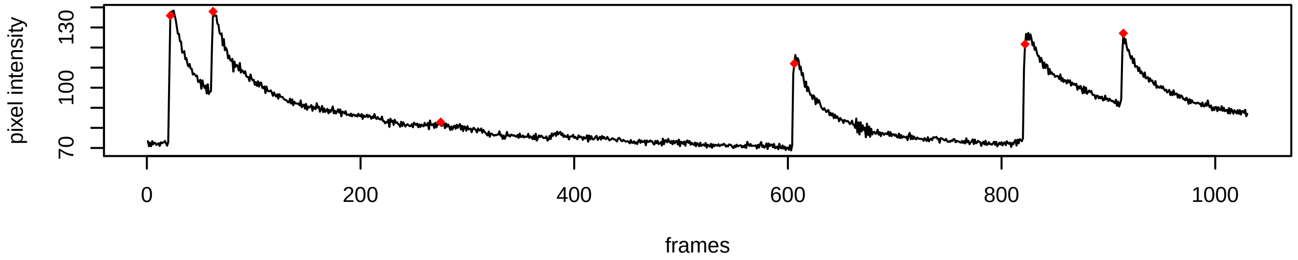

Graph 27 , 6   Total Activity 8   Position in Array 1215

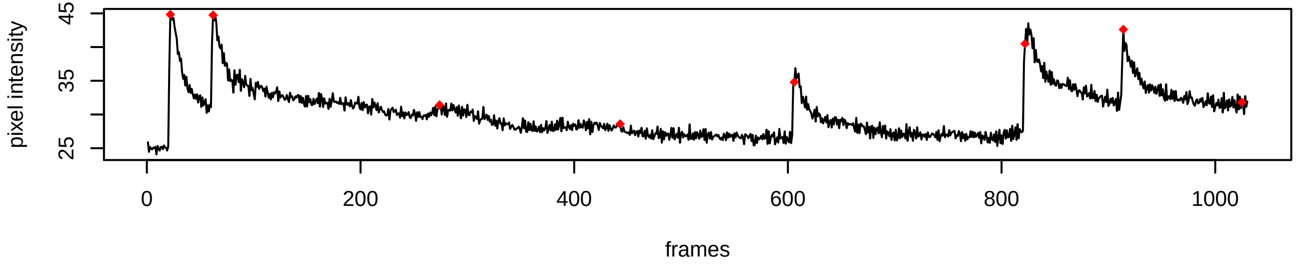

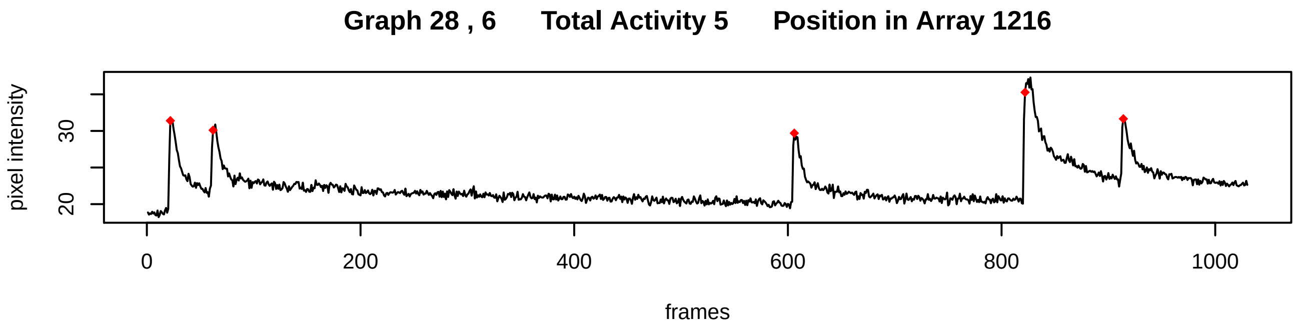
Graph 28 , 6 Total Activity 5 Position in Array 1216
pixel intensity
20
30
0
200
400
600
800
1000
frames

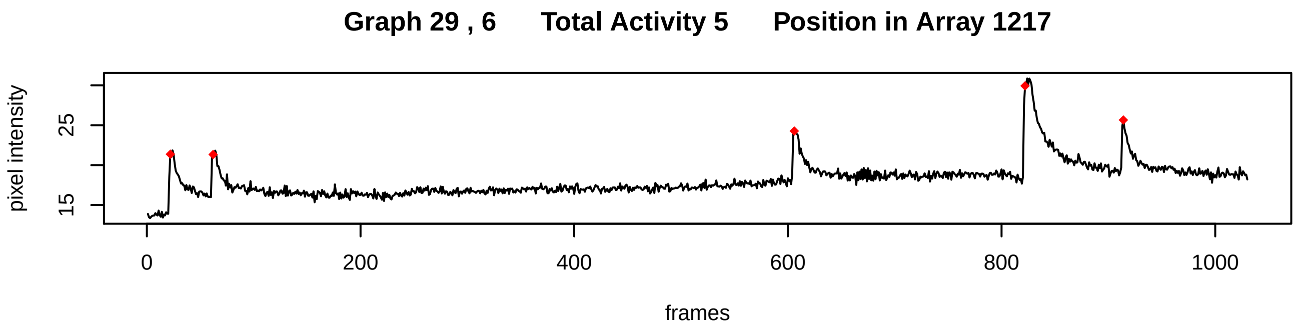
Graph 29 , 6 Total Activity 5 Position in Array 1217
pixel intensity
15
25
0
200
400
600
800
1000
frames

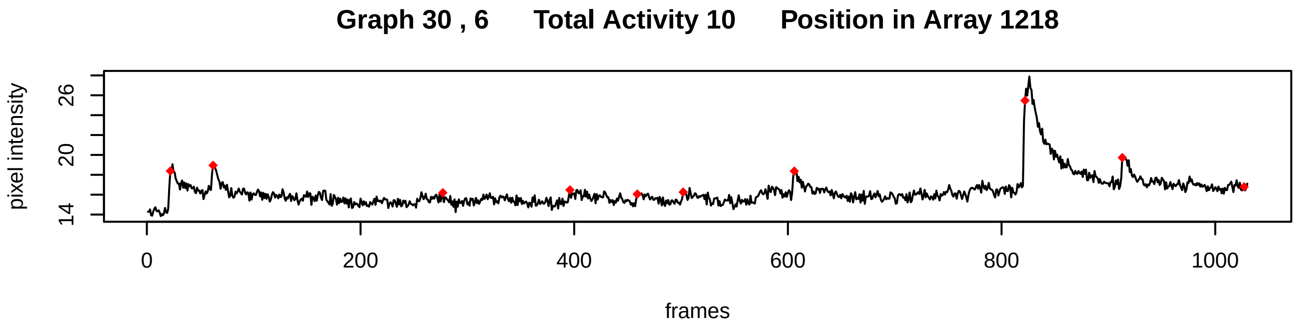
Graph 30 , 6 Total Activity 10 Position in Array 1218
pixel intensity
14
20
26
0
200
400
600
800
1000
frames

Graph 31 , 6   Total Activity 6   Position in Array 1219

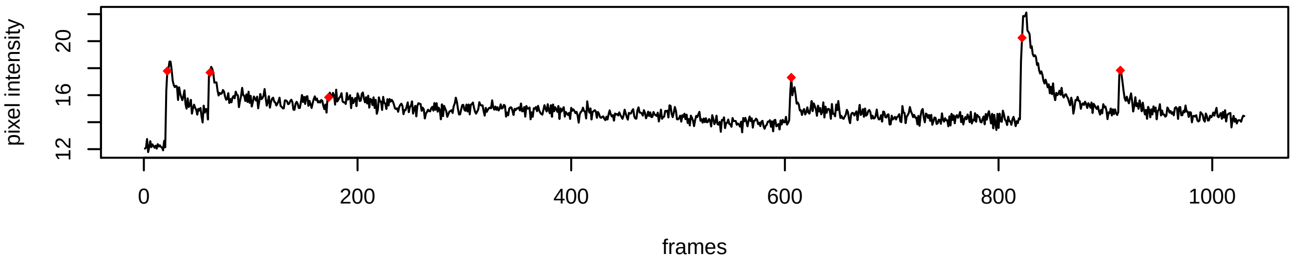

Graph 32 , 6   Total Activity 5   Position in Array 1220

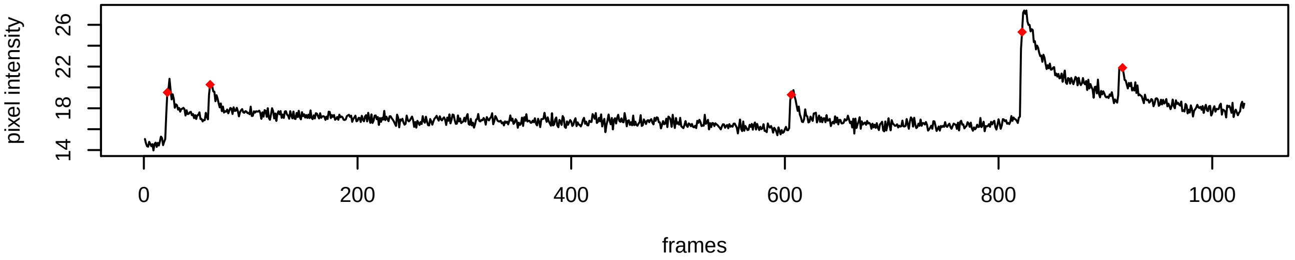

Graph 33 , 6   Total Activity 5   Position in Array 1221

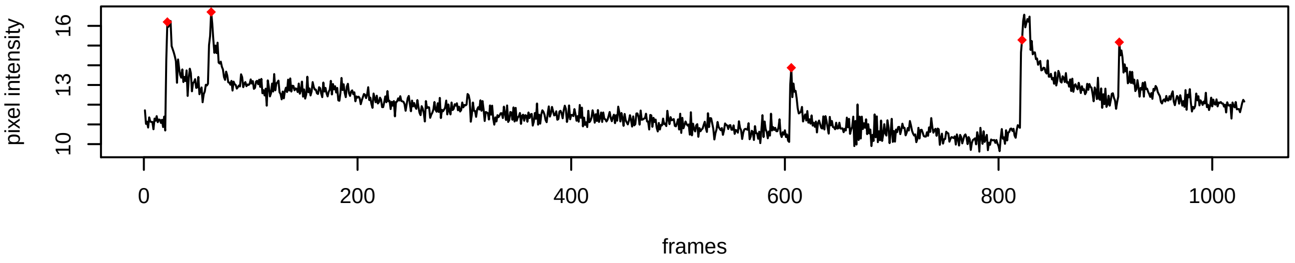

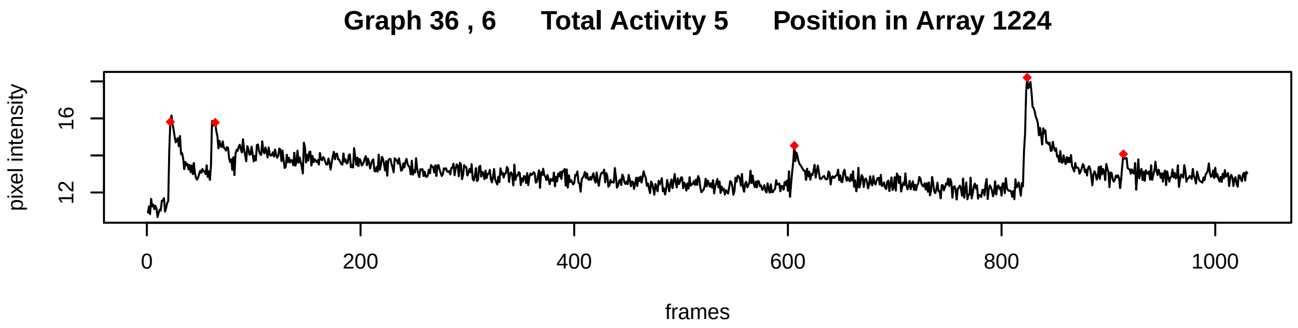
Graph 36 , 6 Total Activity 5 Position in Array 1224
pixel intensity
12
16
0
200
400
600
800
1000
frames

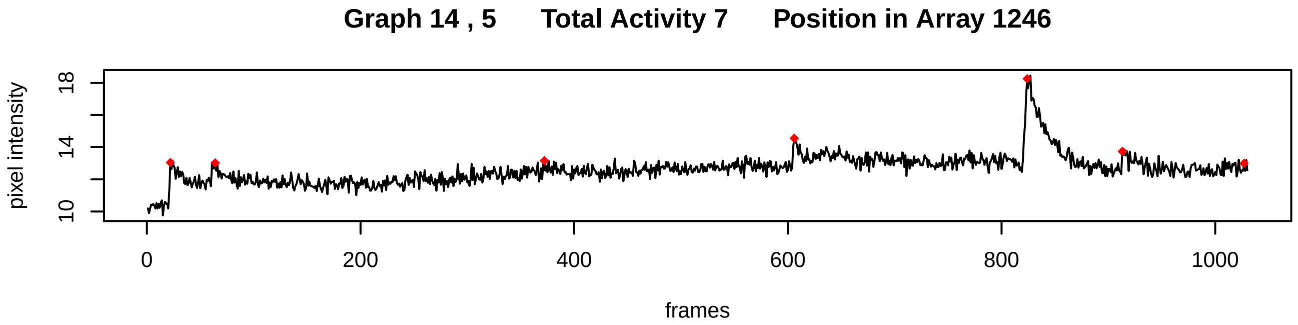
Graph 14 , 5 Total Activity 7 Position in Array 1246
pixel intensity
10
14
18
0
200
400
600
800
1000
frames

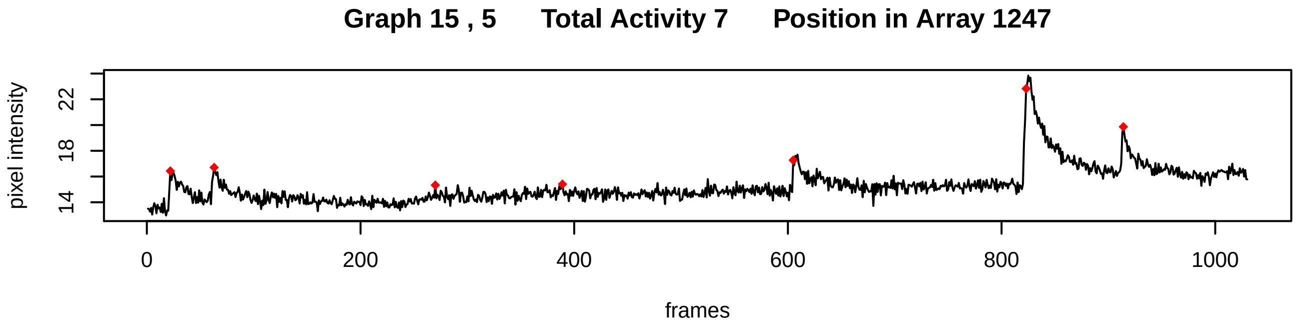
Graph 15 , 5 Total Activity 7 Position in Array 1247
pixel intensity
14
18
22
0
200
400
600
800
1000
frames

Graph 16 , 5 Total Activity 6 Position in Array 1248

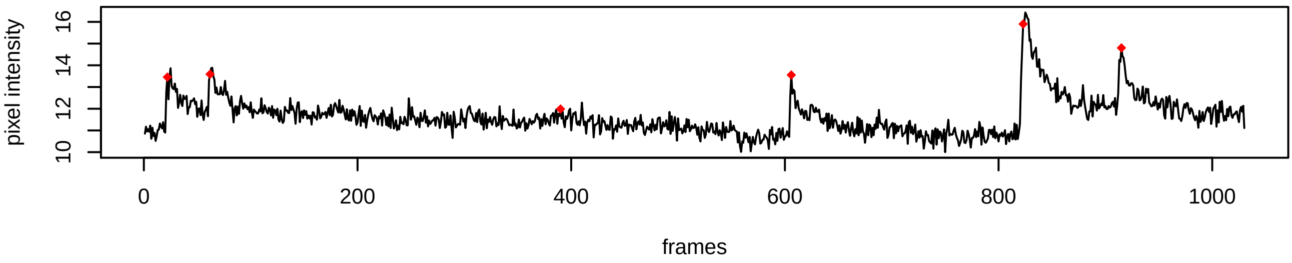

Graph 19 , 5 Total Activity 5 Position in Array 1251

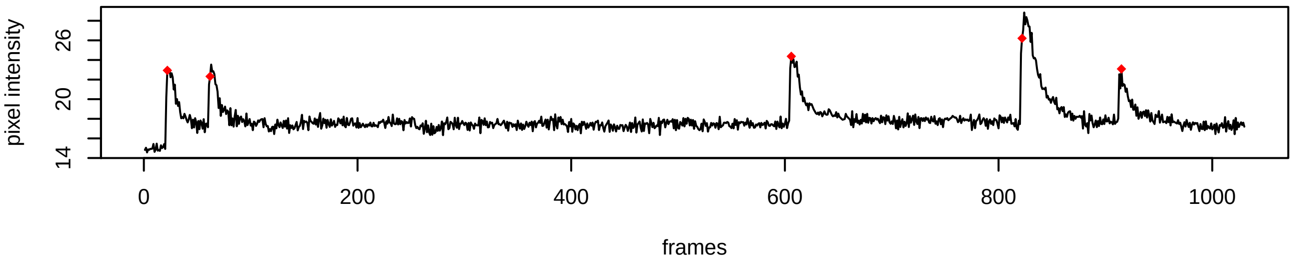

Graph 20 , 5 Total Activity 5 Position in Array 1252

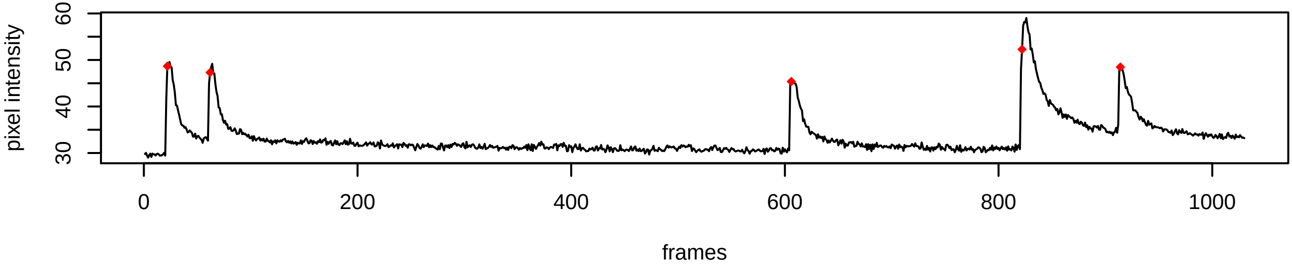

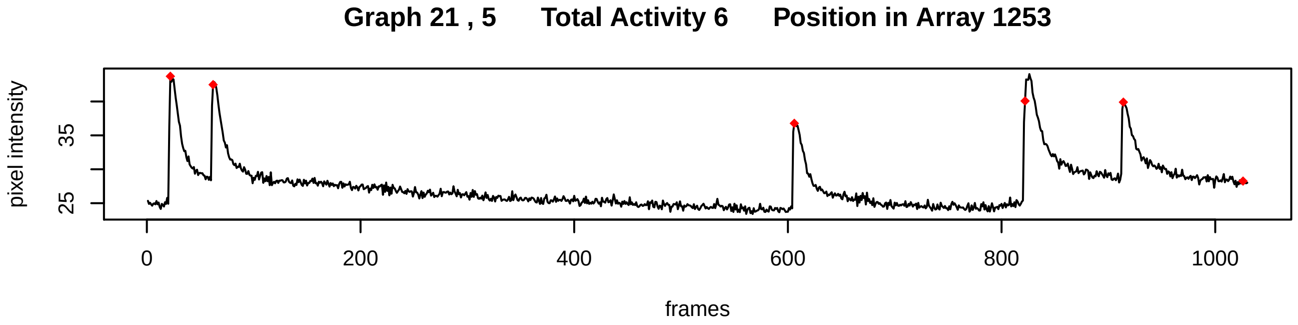
Graph 21 , 5 Total Activity 6 Position in Array 1253
pixel intensity
25
35
0
200
400
600
800
1000
frames

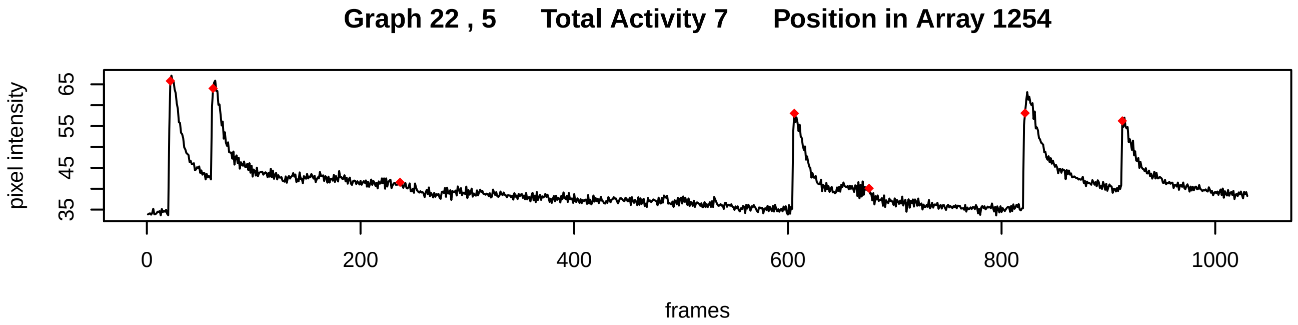
Graph 22 , 5 Total Activity 7 Position in Array 1254
pixel intensity
35
45
55
65
0
200
400
600
800
1000
frames

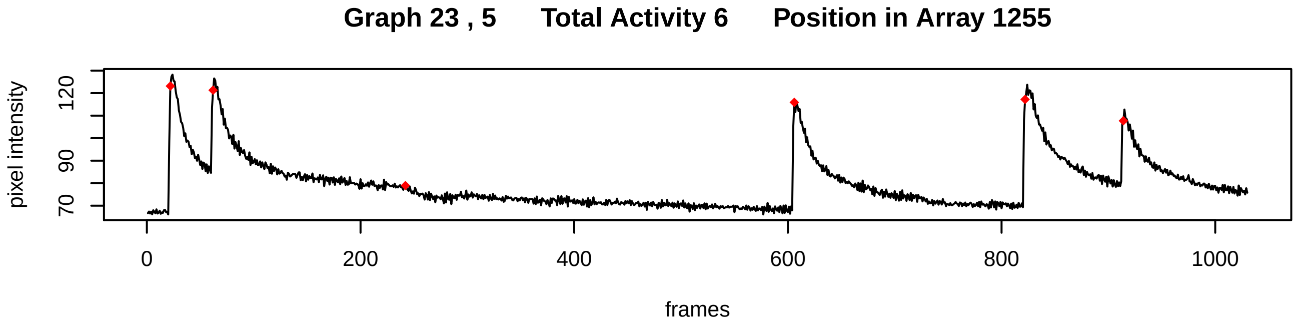
Graph 23 , 5 Total Activity 6 Position in Array 1255
pixel intensity
70
90
120
0
200
400
600
800
1000
frames

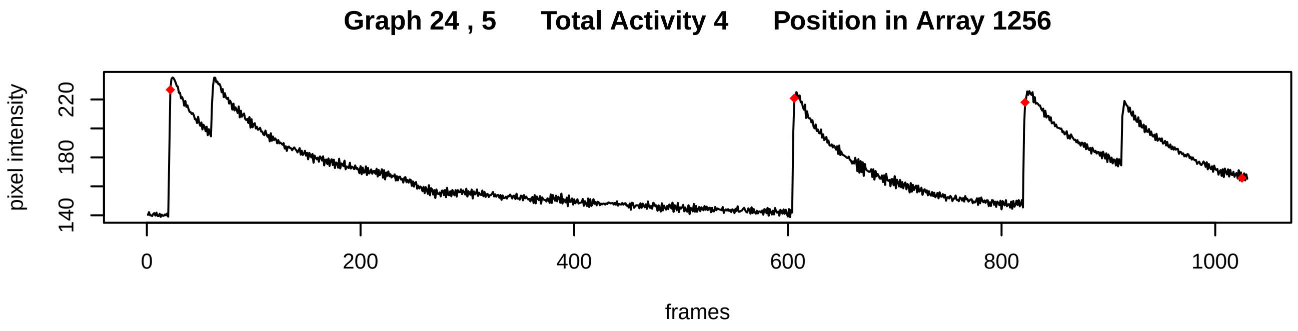
Graph 24 , 5 Total Activity 4 Position in Array 1256
pixel intensity
140
180
220
0
200
400
600
800
1000
frames

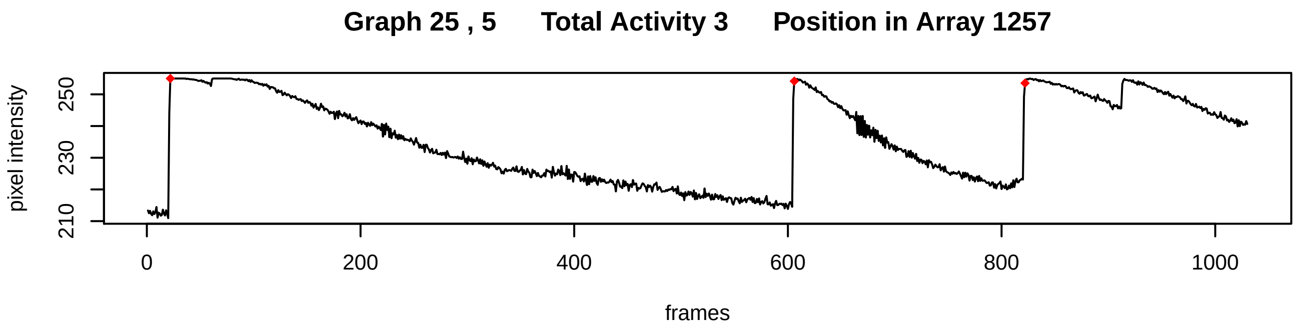
Graph 25 , 5 Total Activity 3 Position in Array 1257
pixel intensity
210
230
250
0
200
400
600
800
1000
frames

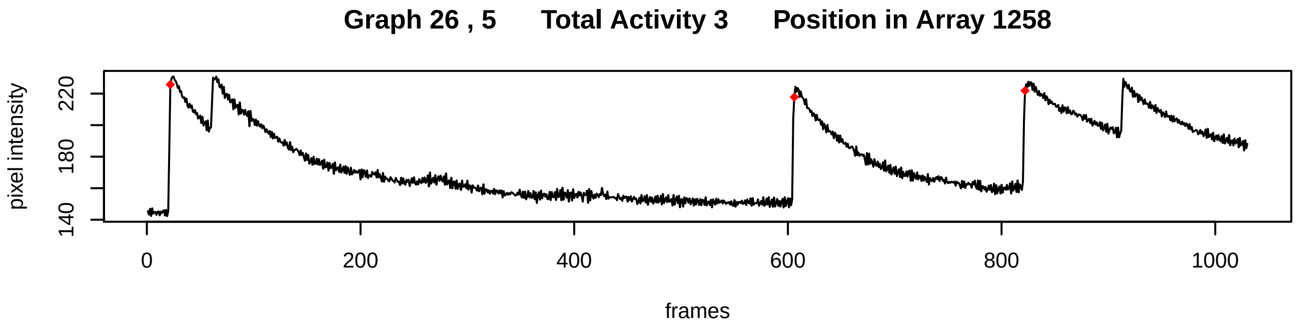
Graph 26 , 5 Total Activity 3 Position in Array 1258
pixel intensity
140
180
220
0
200
400
600
800
1000
frames

Graph 27 , 5   Total Activity 7   Position in Array 1259

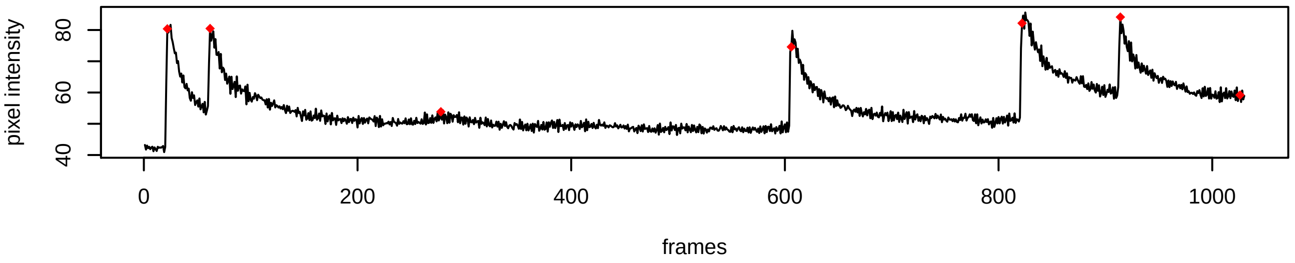

Graph 28 , 5   Total Activity 6   Position in Array 1260

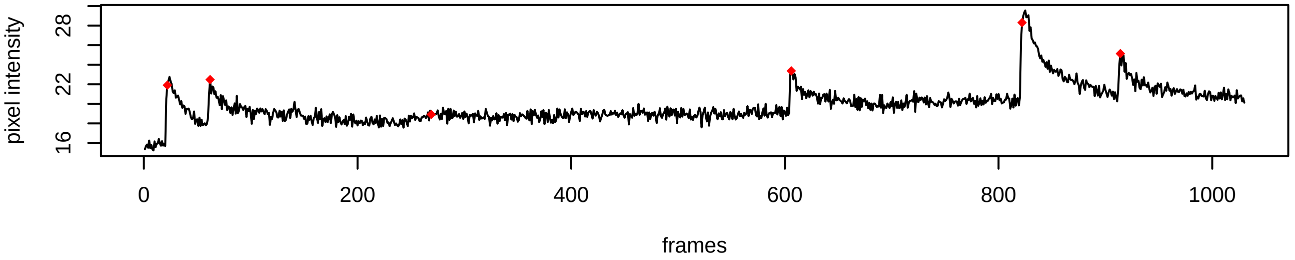

Graph 29 , 5   Total Activity 6   Position in Array 1261

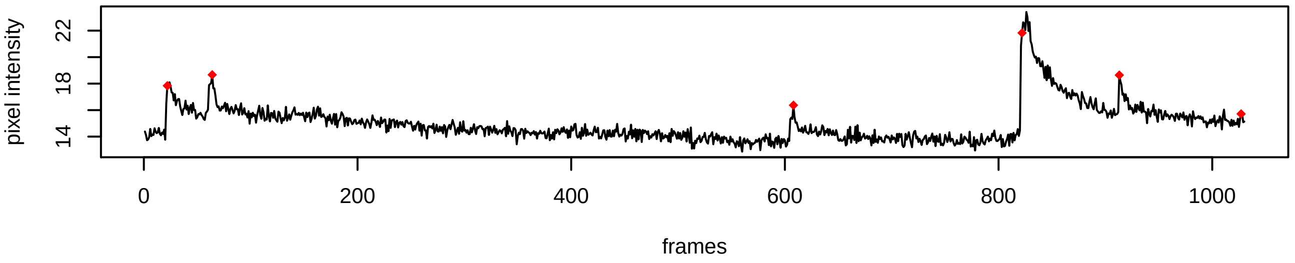

Graph 30 , 5   Total Activity 5   Position in Array 1262

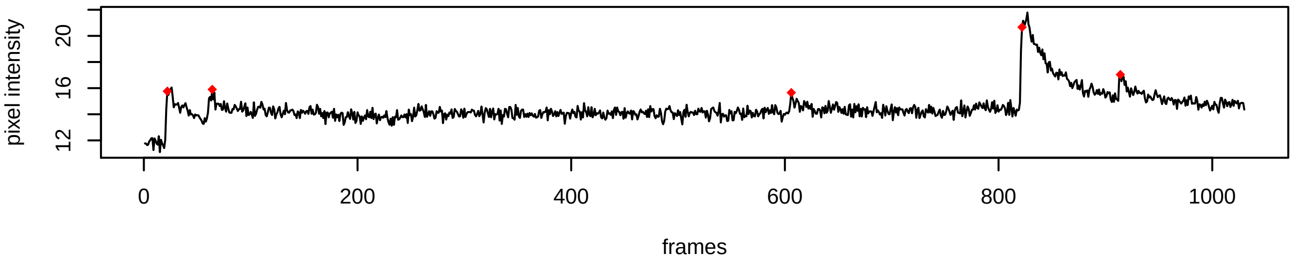

Graph 31 , 5   Total Activity 6   Position in Array 1263

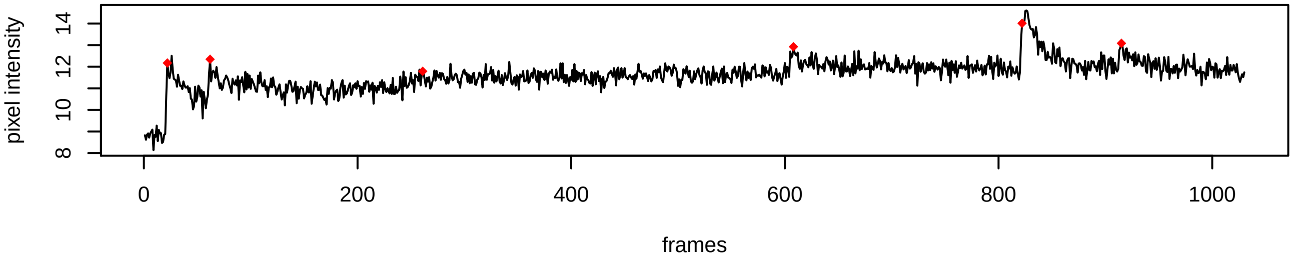

Graph 32 , 5   Total Activity 5   Position in Array 1264

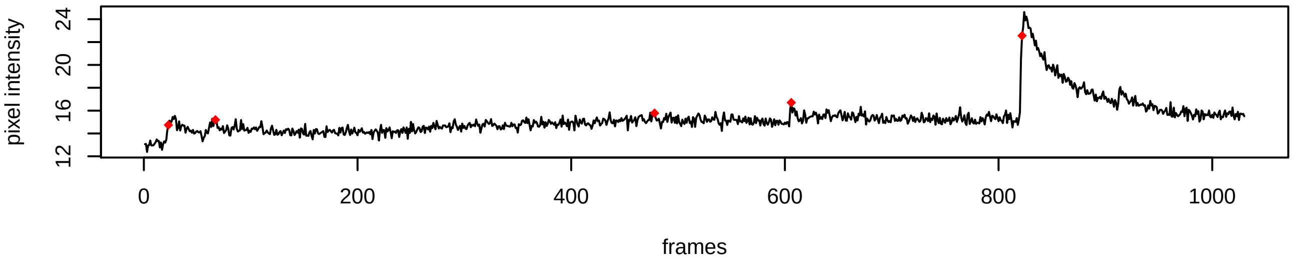

Graph 36 , 5   Total Activity 5   Position in Array 1268

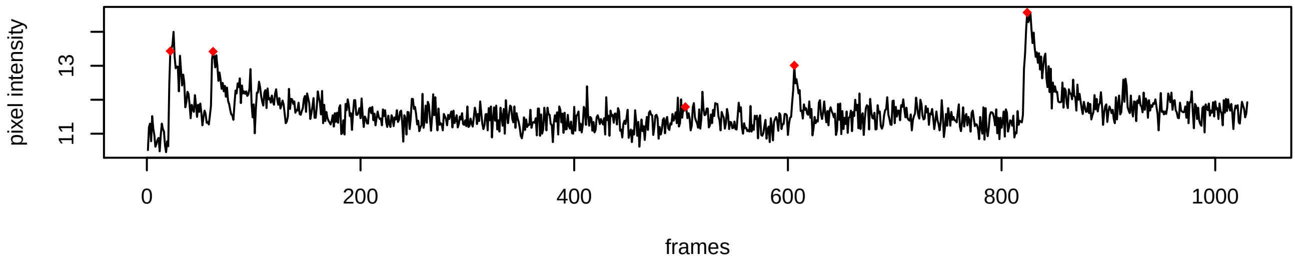

Graph 19 , 4   Total Activity 8   Position in Array 1295

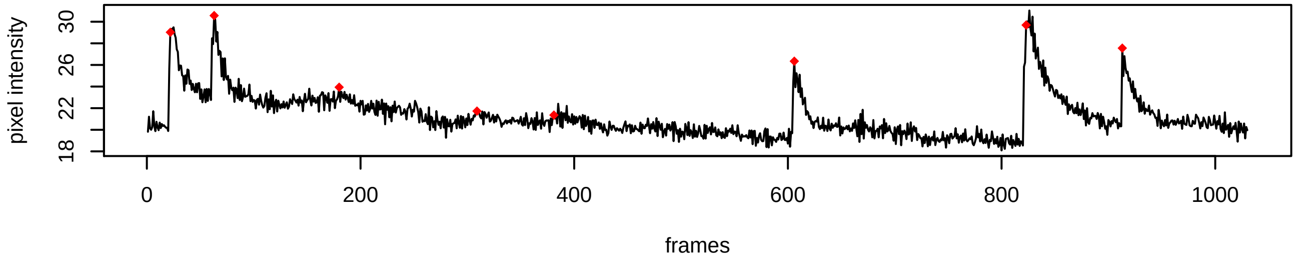

Graph 20 , 4   Total Activity 5   Position in Array 1296

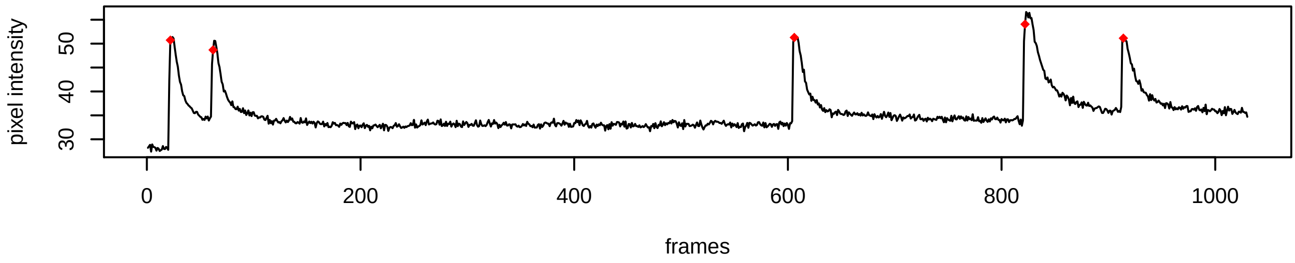

Graph 21 , 4 Total Activity 6 Position in Array 1297

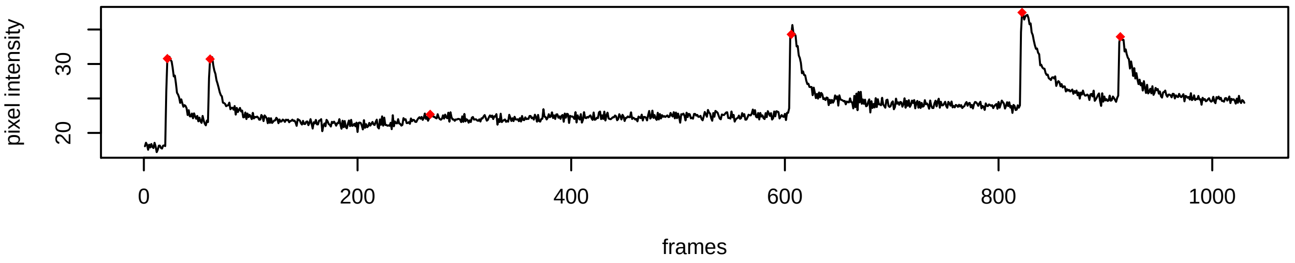

Graph 22 , 4 Total Activity 5 Position in Array 1298

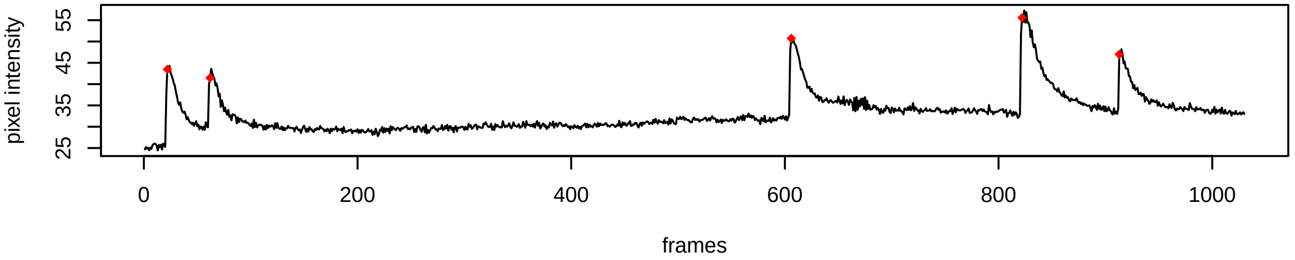

Graph 23 , 4 Total Activity 6 Position in Array 1299

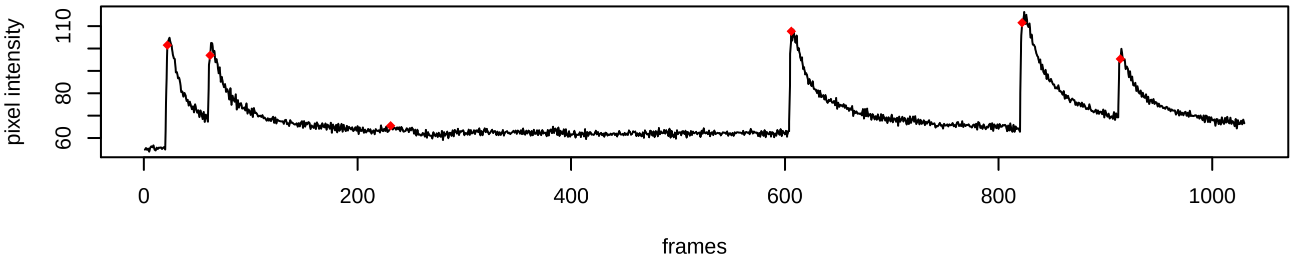

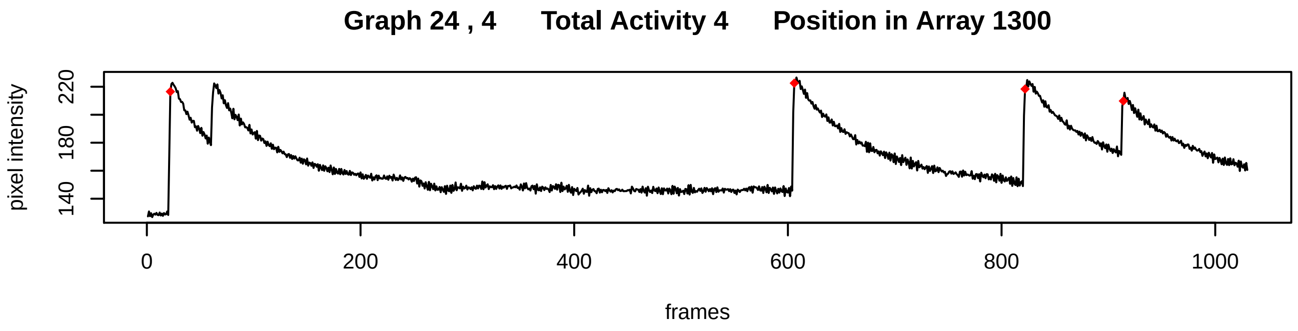
Graph 24 , 4 Total Activity 4 Position in Array 1300
pixel intensity
140
180
220
0
200
400
600
800
1000
frames

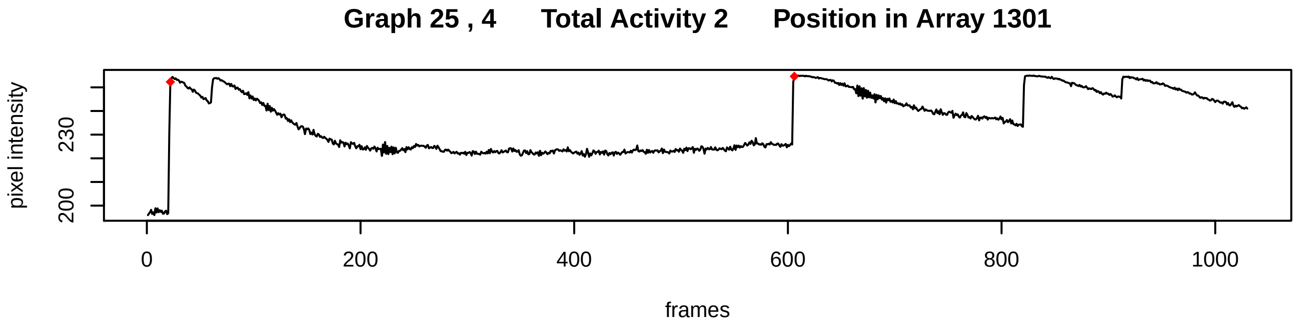
Graph 25 , 4 Total Activity 2 Position in Array 1301
pixel intensity
200
230
0
200
400
600
800
1000
frames

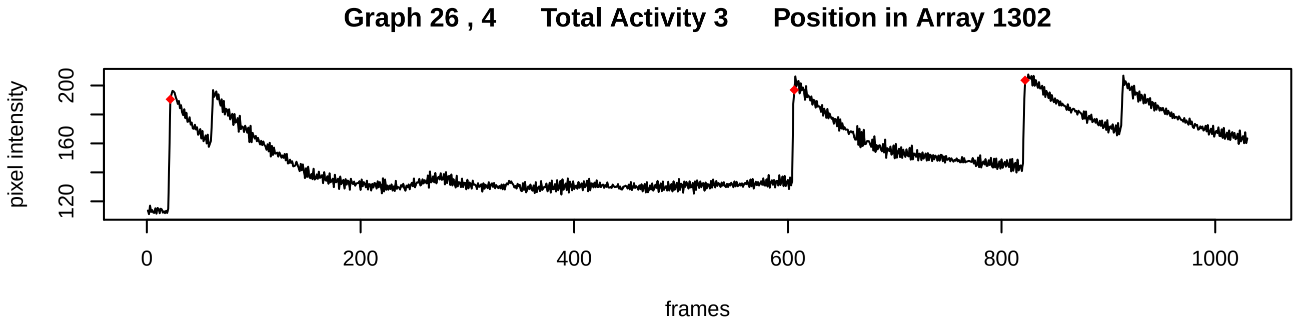
Graph 26 , 4 Total Activity 3 Position in Array 1302
pixel intensity
120
160
200
0
200
400
600
800
1000
frames

Graph 27 , 4 Total Activity 7 Position in Array 1303

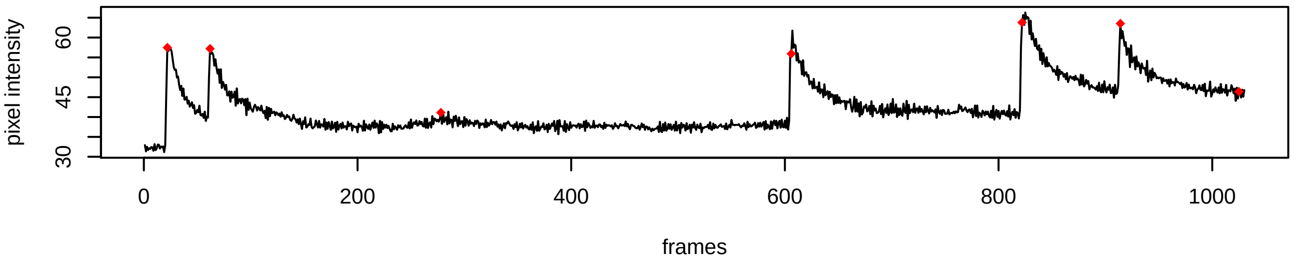

Graph 28 , 4 Total Activity 6 Position in Array 1304

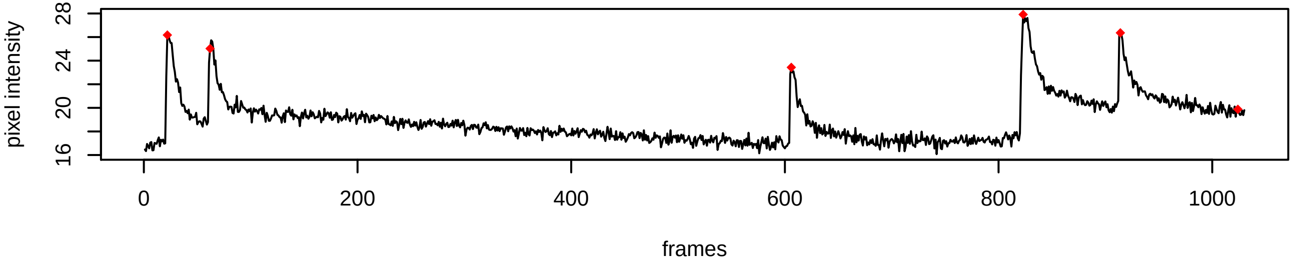

Graph 29 , 4 Total Activity 5 Position in Array 1305

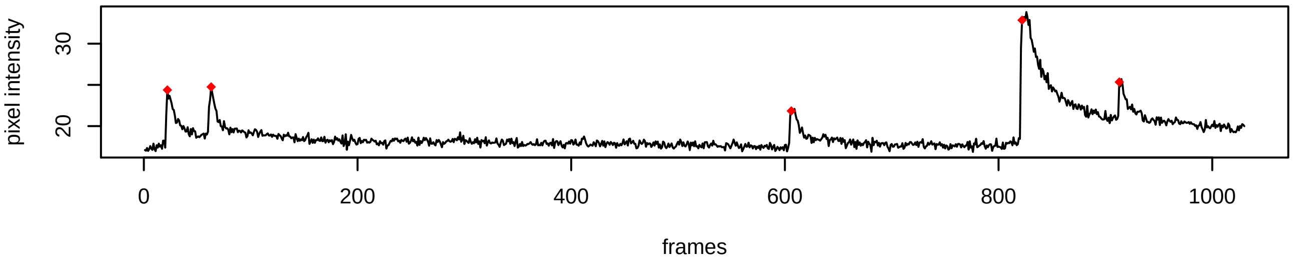

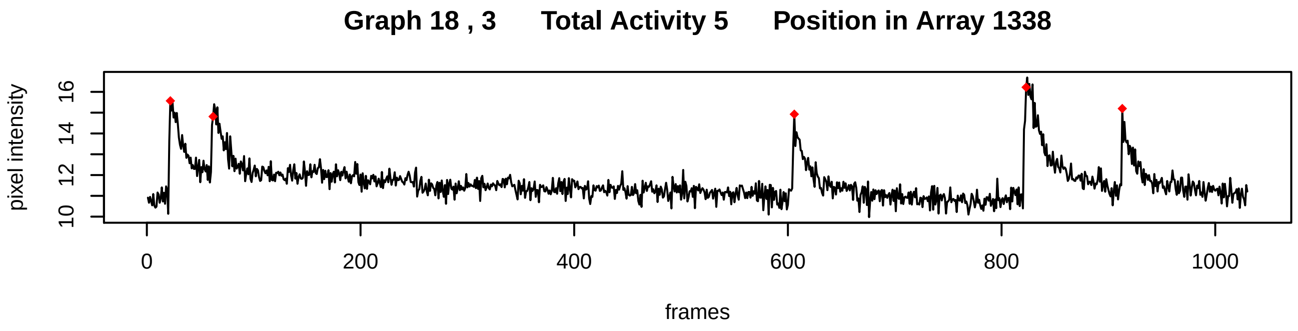
Graph 18 , 3 Total Activity 5 Position in Array 1338
pixel intensity
10
12
14
16
0
200
400
600
800
1000
frames

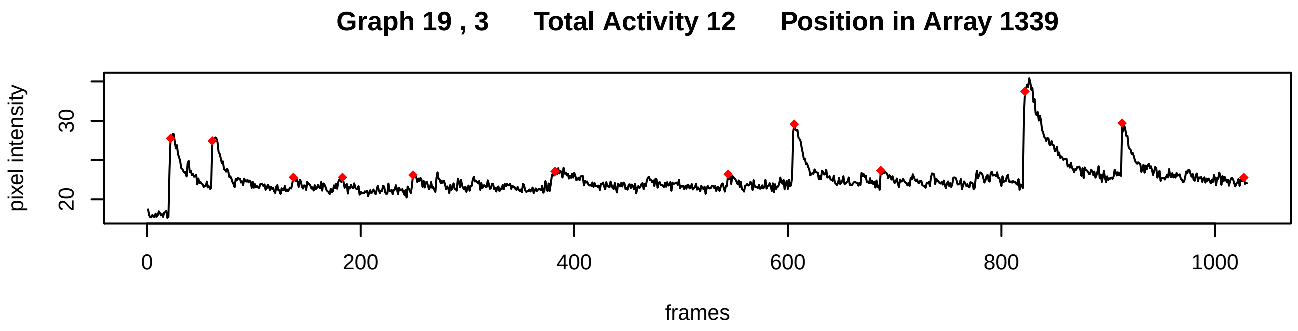
Graph 19 , 3 Total Activity 12 Position in Array 1339
pixel intensity
20
30
0
200
400
600
800
1000
frames

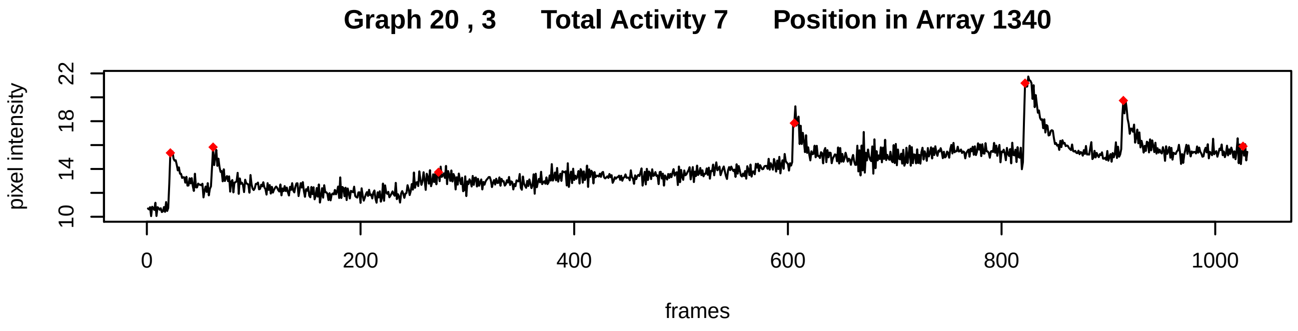
Graph 20 , 3 Total Activity 7 Position in Array 1340
pixel intensity
10
14
18
22
0
200
400
600
800
1000
frames

Graph 22 , 3 Total Activity 5 Position in Array 1342

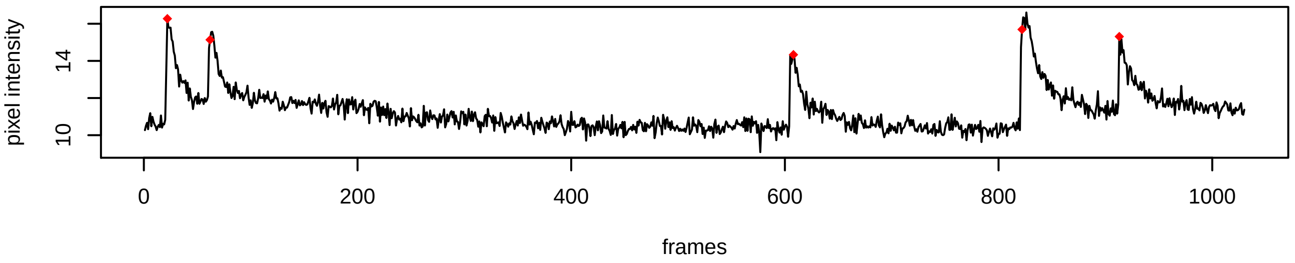

Graph 23 , 3 Total Activity 5 Position in Array 1343

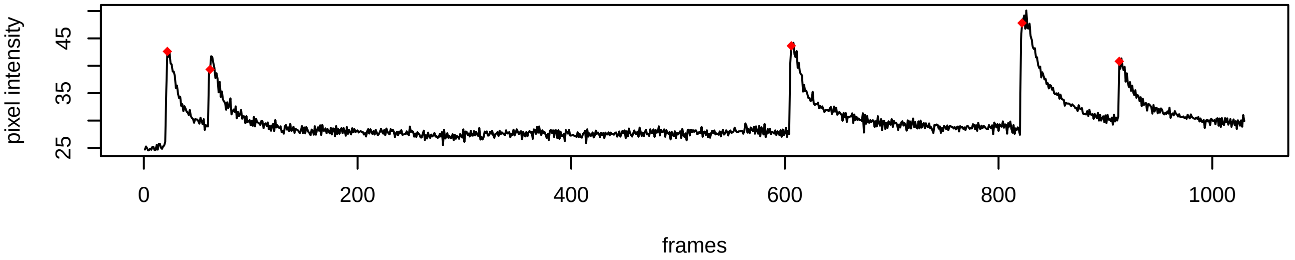

Graph 24 , 3 Total Activity 7 Position in Array 1344

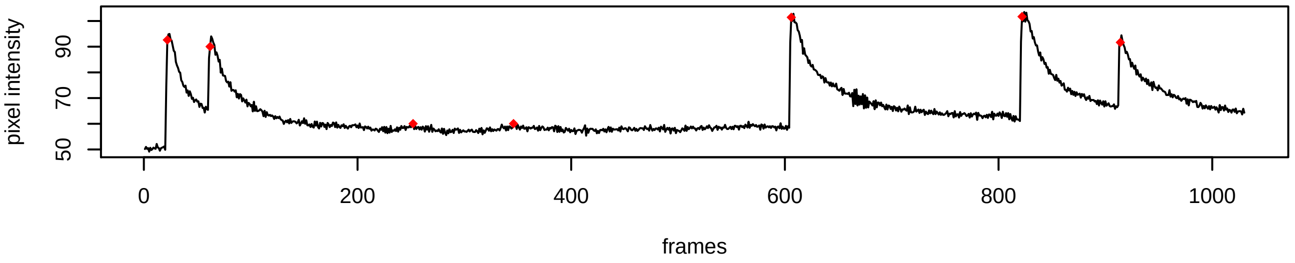

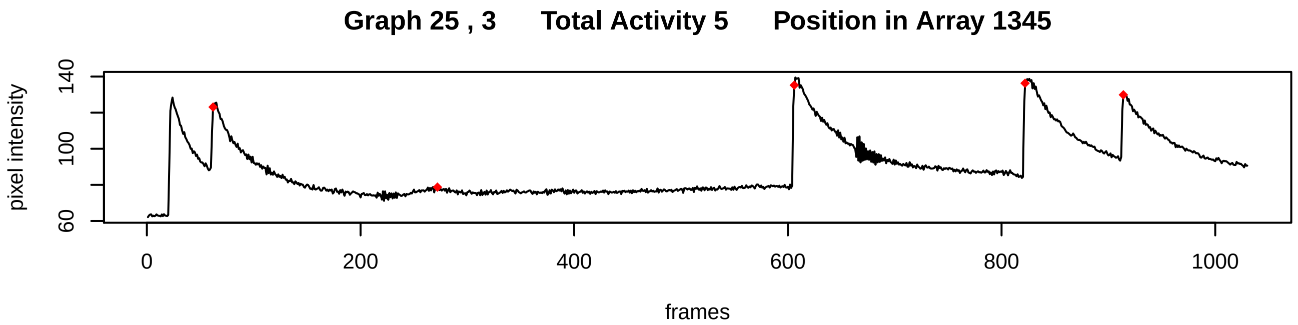
Graph 25 , 3 Total Activity 5 Position in Array 1345
pixel intensity
60
100
140
0
200
400
600
800
1000
frames

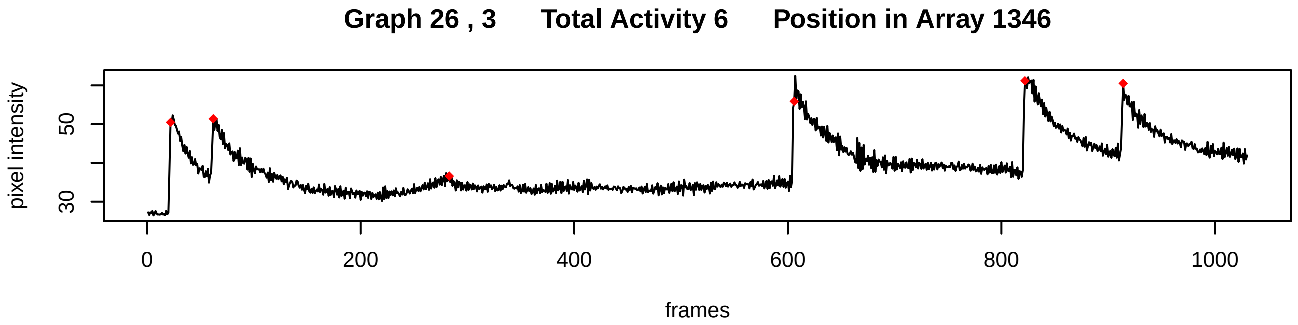
Graph 26 , 3 Total Activity 6 Position in Array 1346
pixel intensity
30
50
0
200
400
600
800
1000
frames

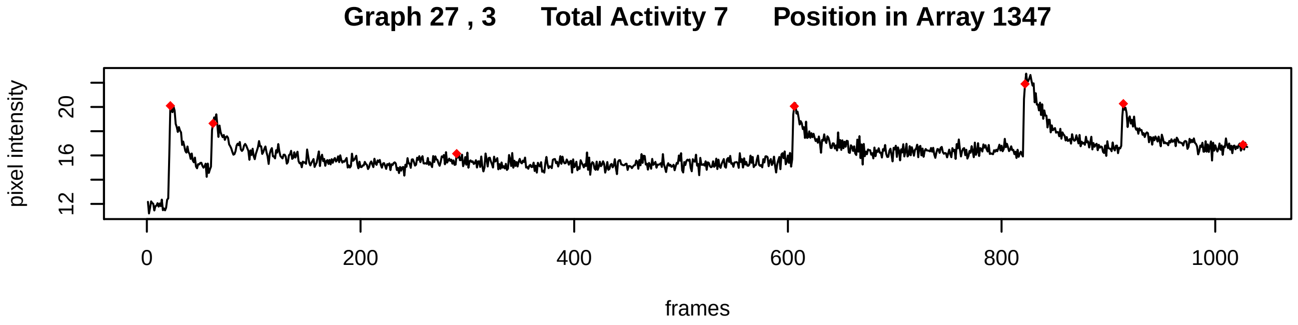
Graph 27 , 3 Total Activity 7 Position in Array 1347
pixel intensity
12
16
20
0
200
400
600
800
1000
frames

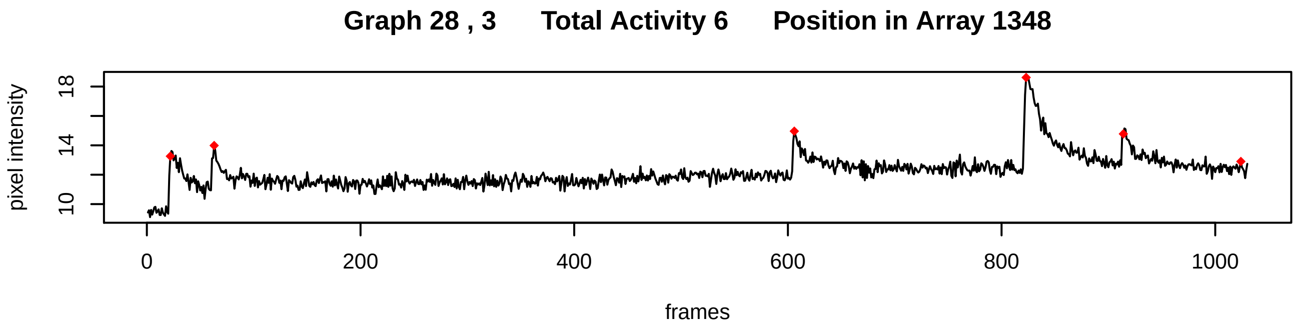
Graph 28 , 3 Total Activity 6 Position in Array 1348
pixel intensity
10
14
18
0
200
400
600
800
1000
frames

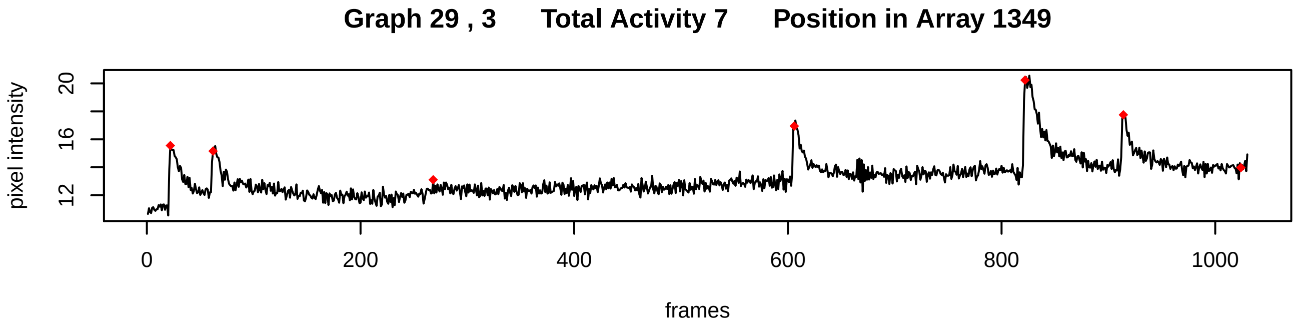
Graph 29 , 3 Total Activity 7 Position in Array 1349
pixel intensity
12
16
20
0
200
400
600
800
1000
frames

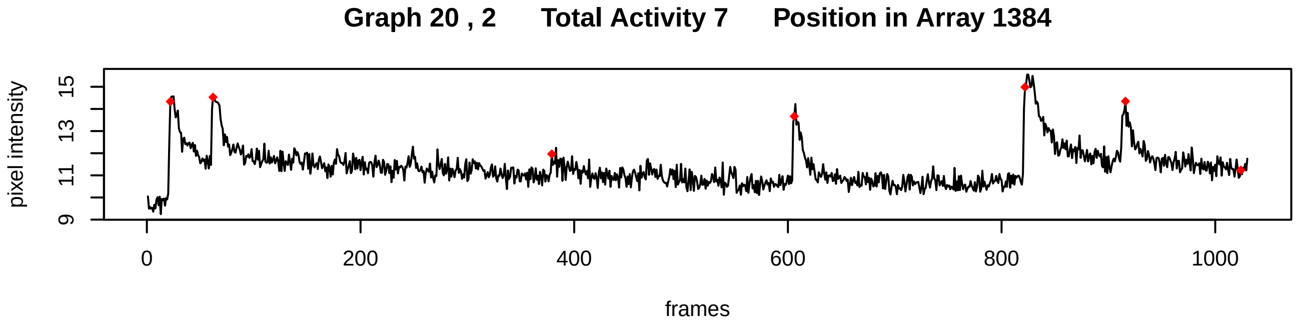
Graph 20 , 2 Total Activity 7 Position in Array 1384
pixel intensity
9
11
13
15
0
200
400
600
800
1000
frames

Graph 22 , 2 Total Activity 5 Position in Array 1386

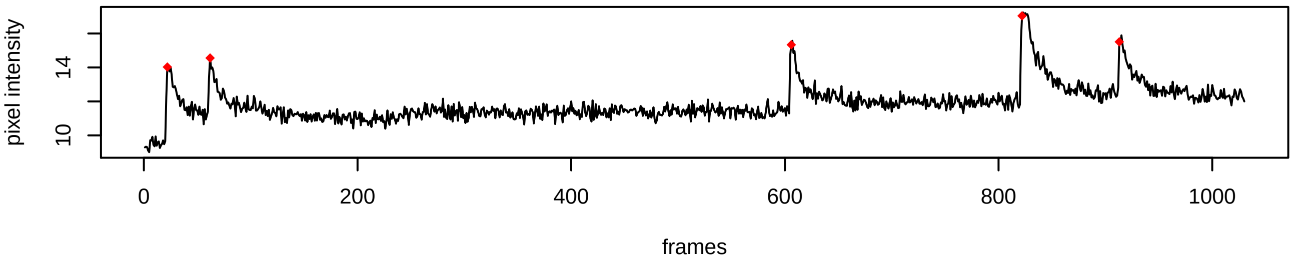

Graph 24 , 2 Total Activity 5 Position in Array 1388

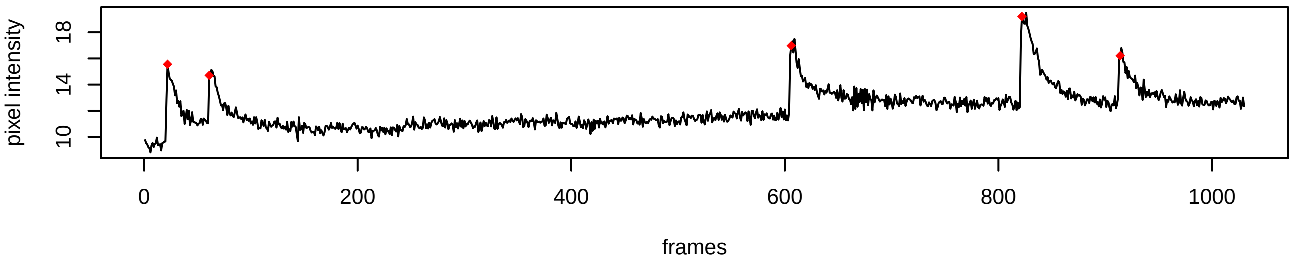

Graph 25 , 2 Total Activity 5 Position in Array 1389

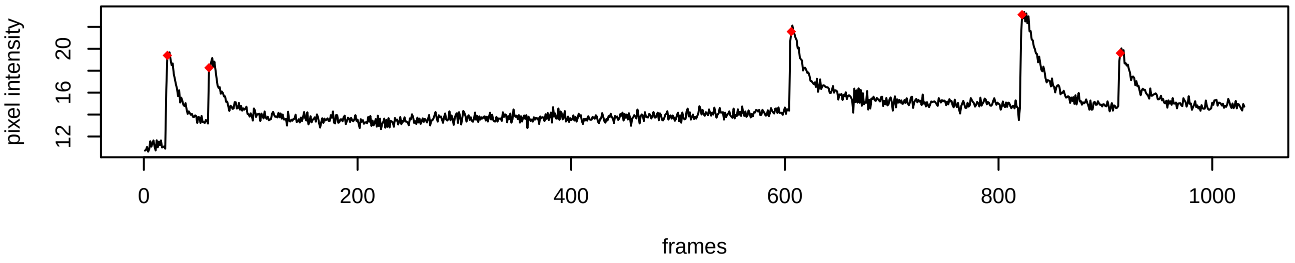

Graph 26 , 2 Total Activity 6 Position in Array 1390

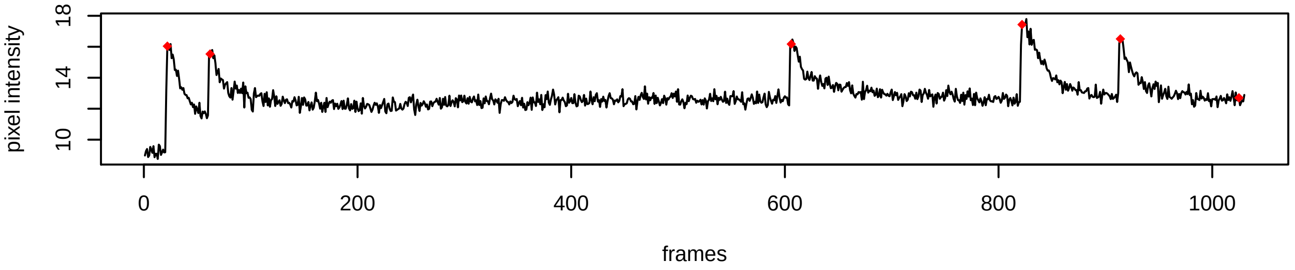

Graph 27 , 2 Total Activity 5 Position in Array 1391

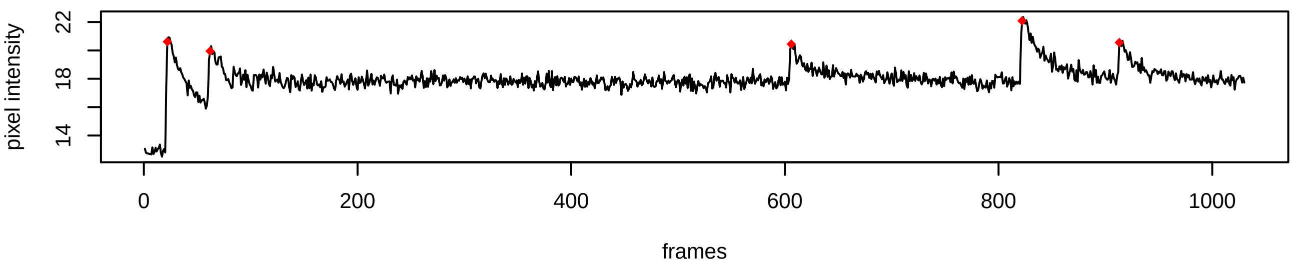

Graph 29 , 2 Total Activity 6 Position in Array 1393

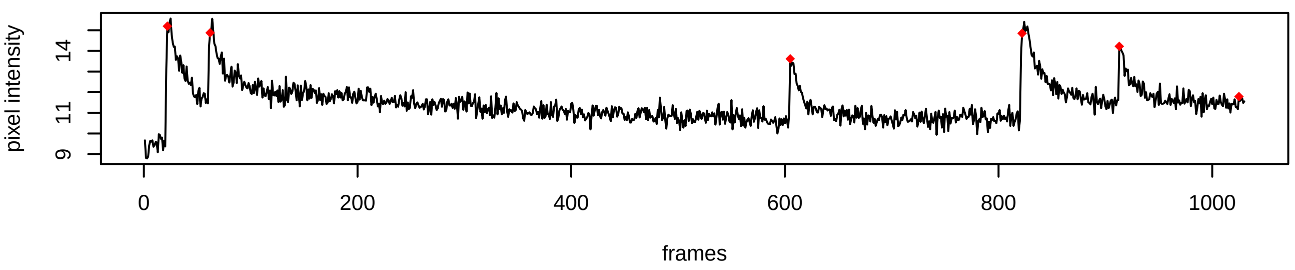

Graph 32 , 2   Total Activity 5   Position in Array 1396

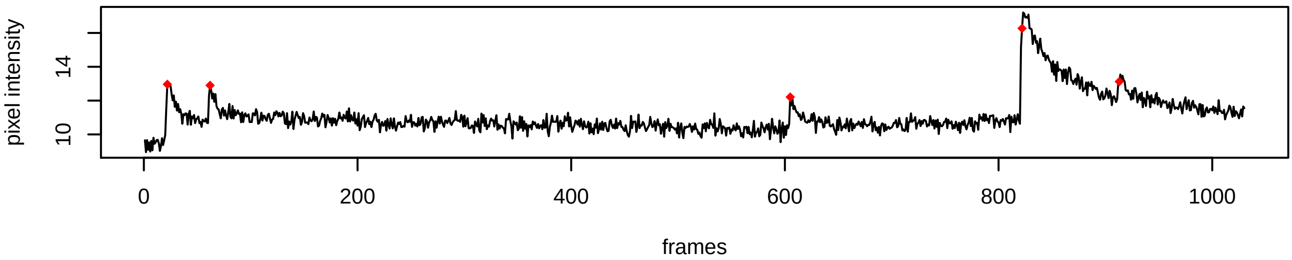

Supplement: S6 Fig — Computation of hippocampal neurons during stimulation with a solution to induce chemical LTP; traces and activity counts. (PDF) [file pcbi.1006054.s006.pdf]
