# Supplementary material for: An open source tool for automatic spatiotemporal assessment of calcium transients and local ‘signal-close-to-noise’ activity in calcium imaging data
Source: PLoS Comput Biol. 2018 Mar 30;14(3):e1006054. doi: 10.1371/journal.pcbi.1006054 (PMC5895056; doi:10.1371/journal.pcbi.1006054)

Total activity 6706

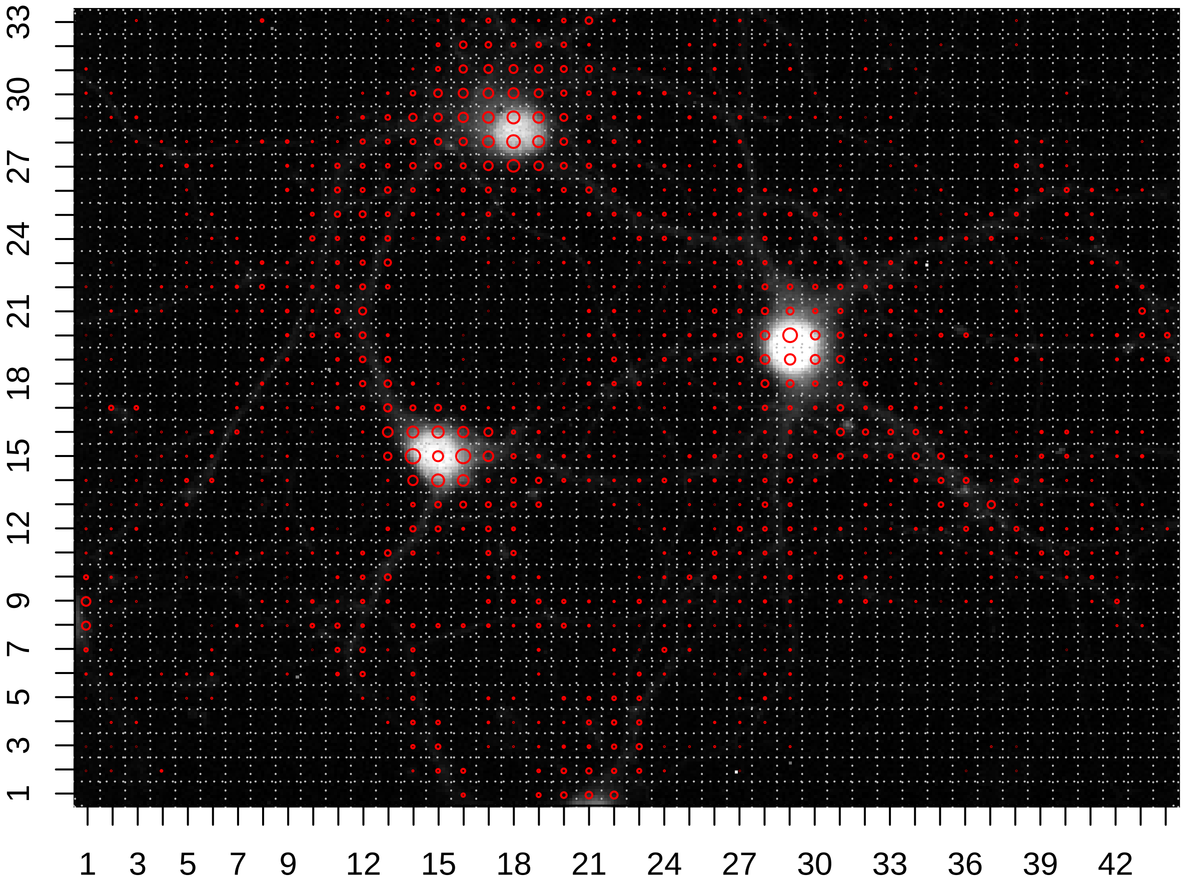

Graph 17 , 33 Total Activity 12 Variance Area 1797

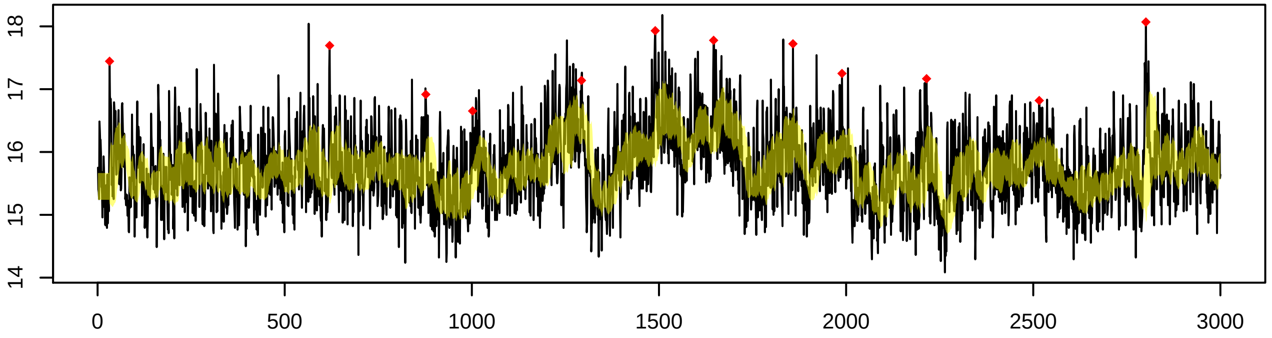

Graph 18 , 33 Total Activity 8 Variance Area 572

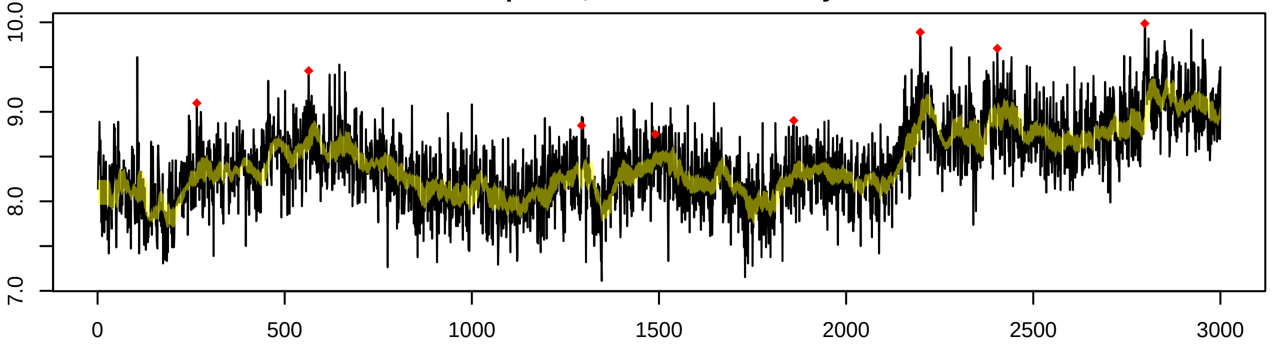

Graph 19 , 33 Total Activity 4 Variance Area 416

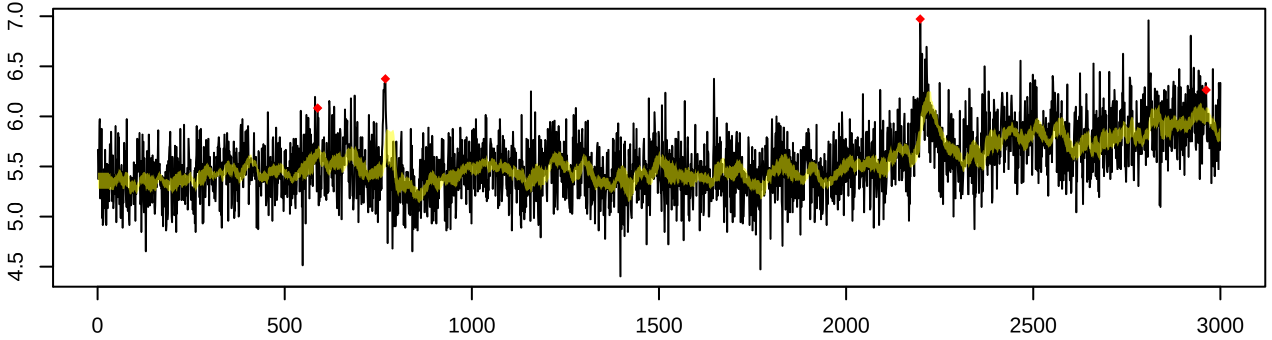

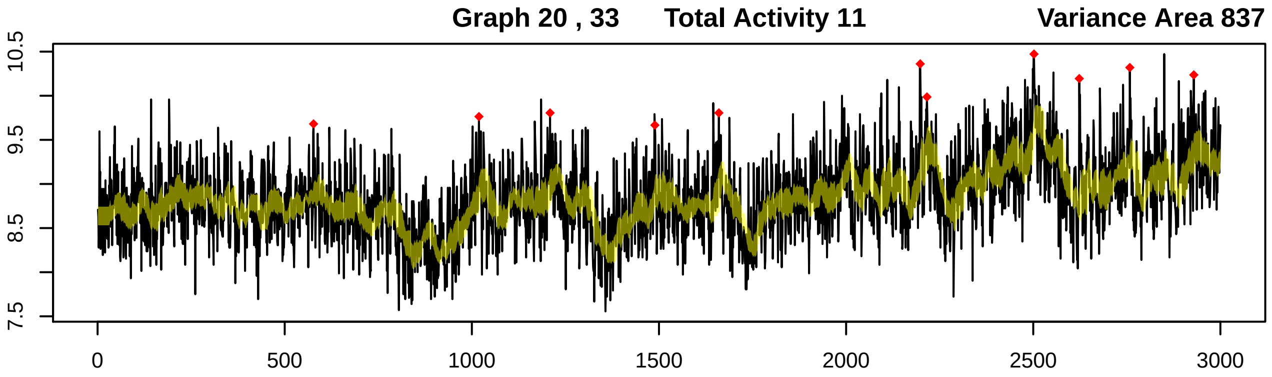
Graph 20 , 33
Total Activity 11
Variance Area 837
7.5
8.5
9.5
10.5
0
500
1000
1500
2000
2500
3000

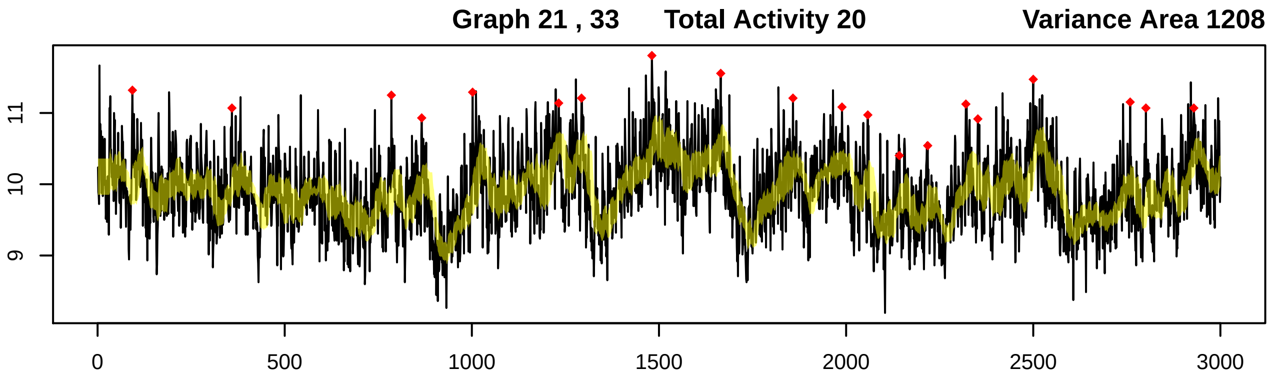
Graph 21 , 33
Total Activity 20
Variance Area 1208
9
10
11
0
500
1000
1500
2000
2500
3000

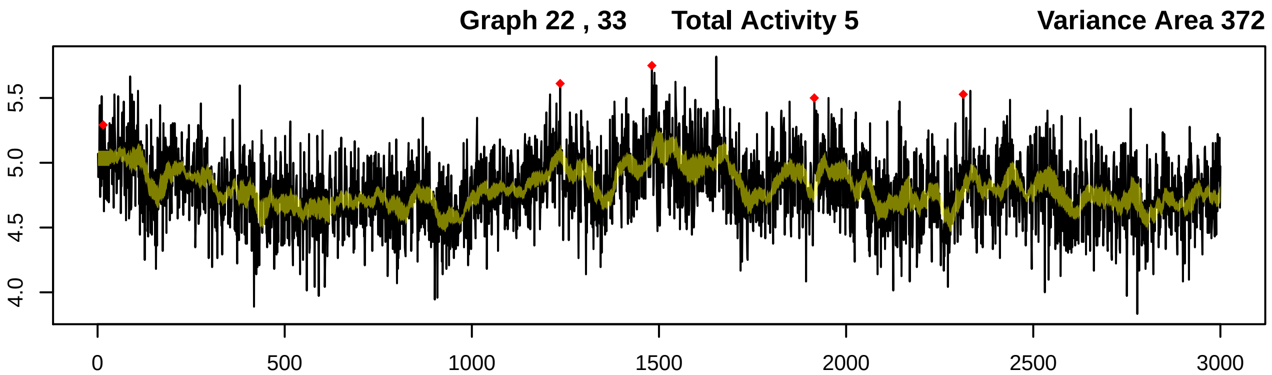
Graph 22 , 33
Total Activity 5
Variance Area 372
4.0
4.5
5.0
5.5
0
500
1000
1500
2000
2500
3000

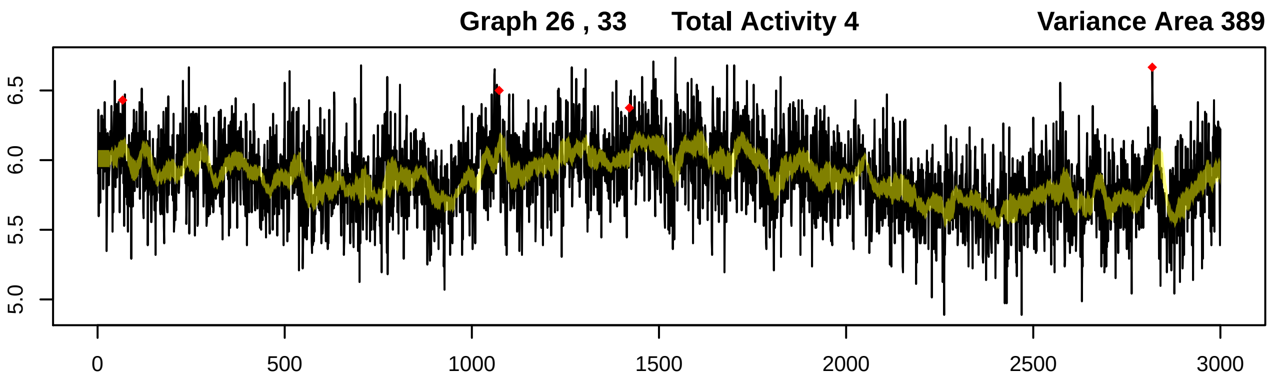
Graph 26 , 33
Total Activity 4
Variance Area 389
6.5
6.0
5.5
5.0
0
500
1000
1500
2000
2500
3000

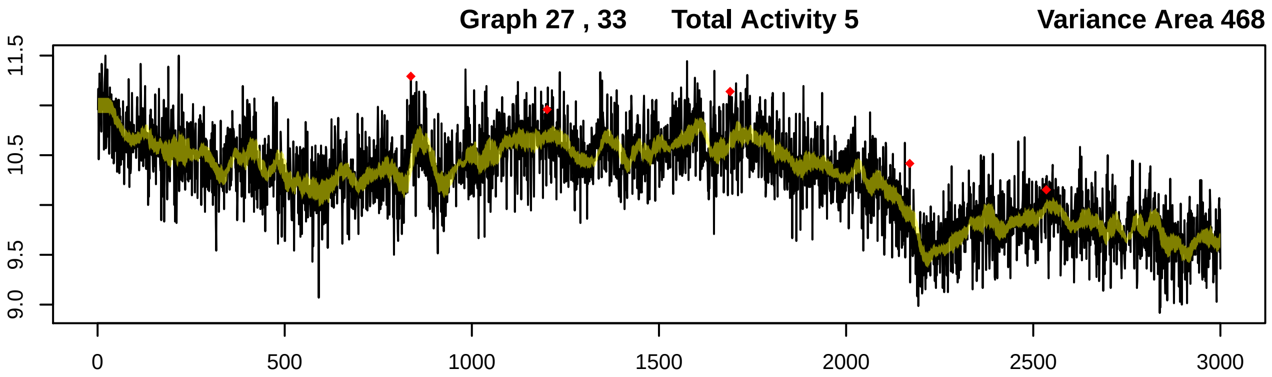
Graph 27 , 33
Total Activity 5
Variance Area 468
11.5
10.5
9.5
9.0
0
500
1000
1500
2000
2500
3000

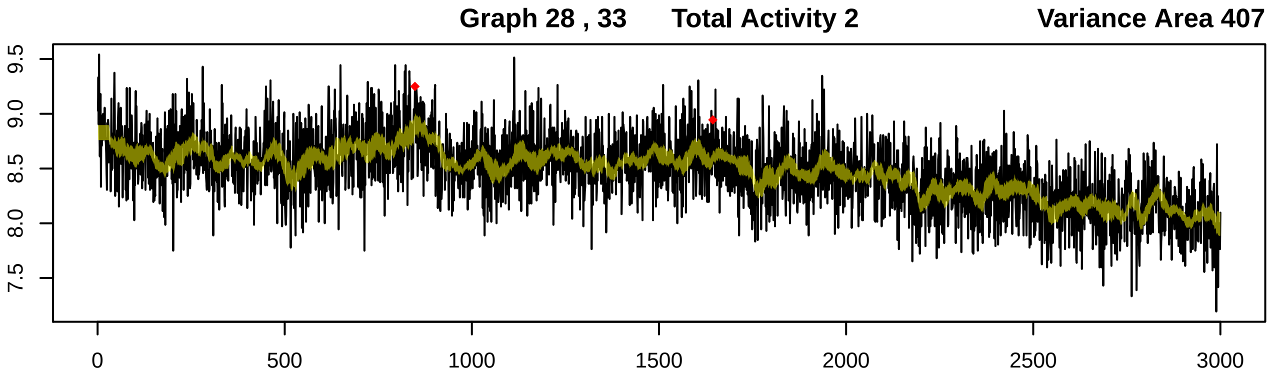
Graph 28 , 33
Total Activity 2
Variance Area 407
9.5
9.0
8.5
8.0
7.5
0
500
1000
1500
2000
2500
3000

Graph 32 , 33 Total Activity 1 Variance Area 334

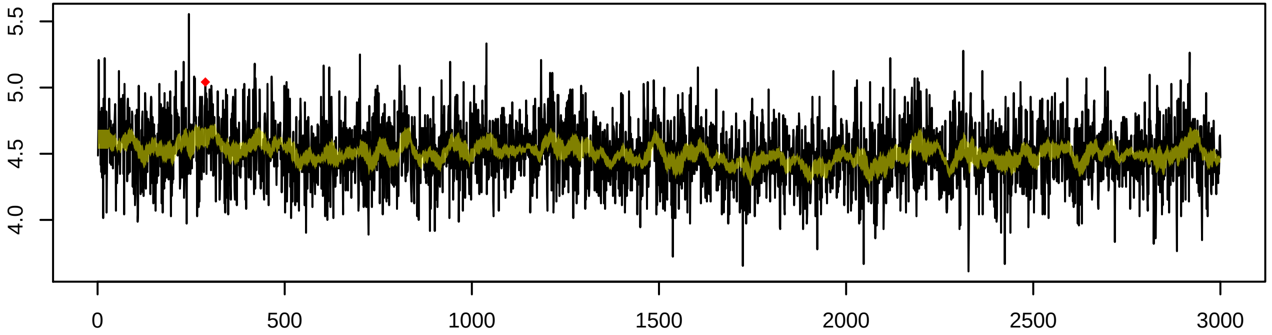

Graph 38 , 33 Total Activity 2 Variance Area 322

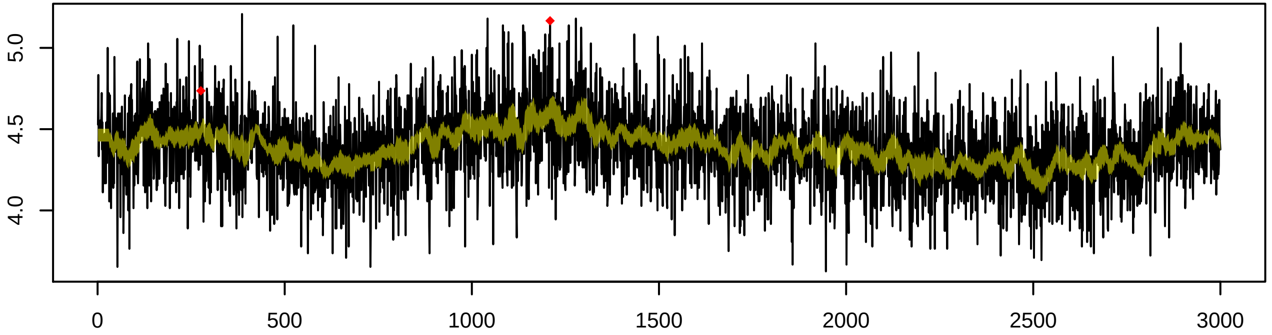

Graph 15 , 32 Total Activity 9 Variance Area 480

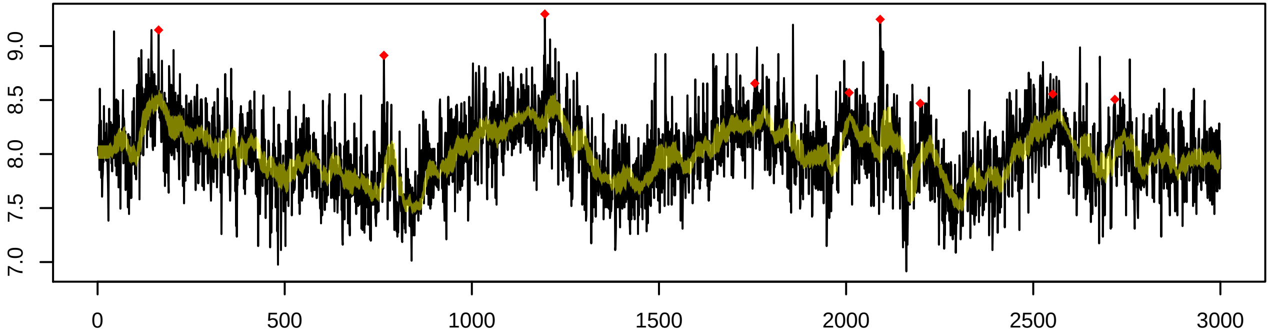

Graph 16 , 32   Total Activity 20   Variance Area 1508

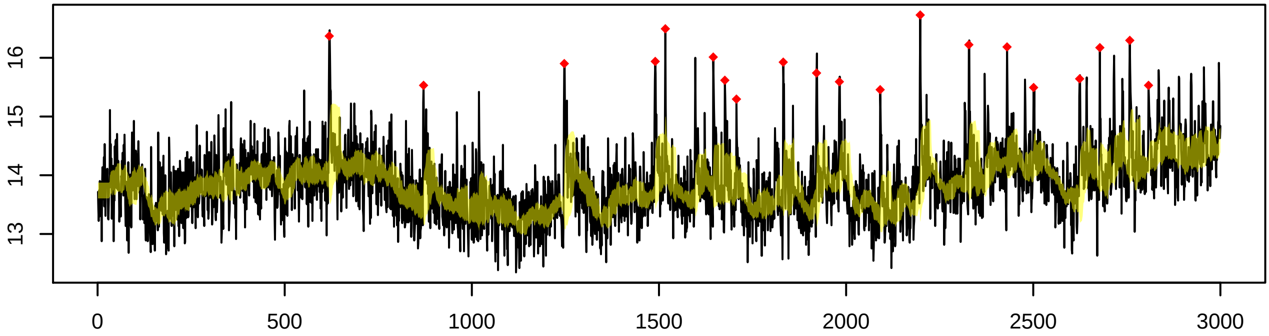

Graph 17 , 32   Total Activity 17   Variance Area 1489

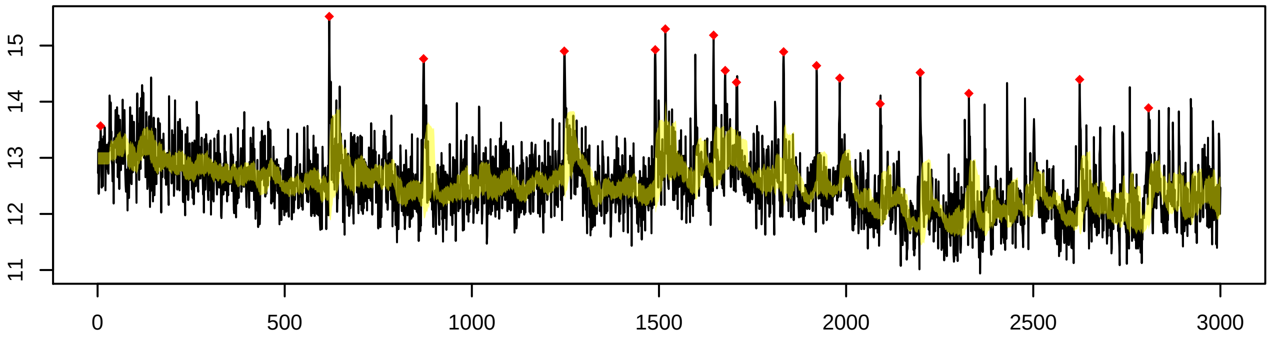

Graph 18 , 32   Total Activity 12   Variance Area 1722

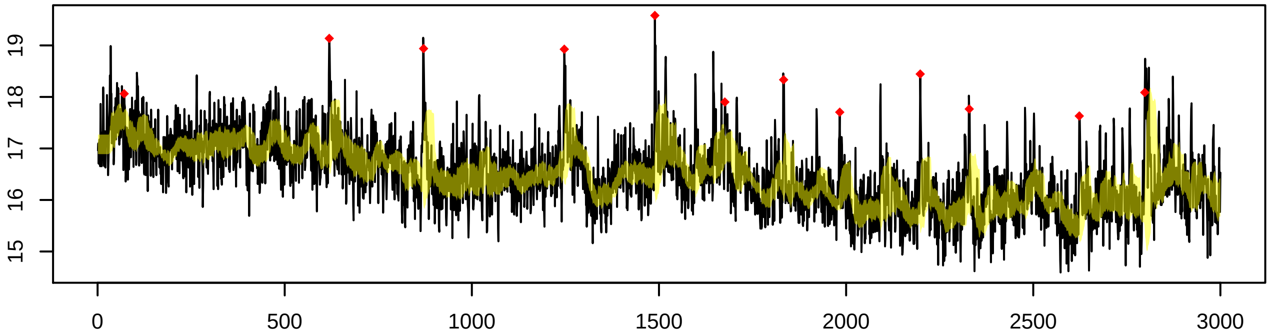

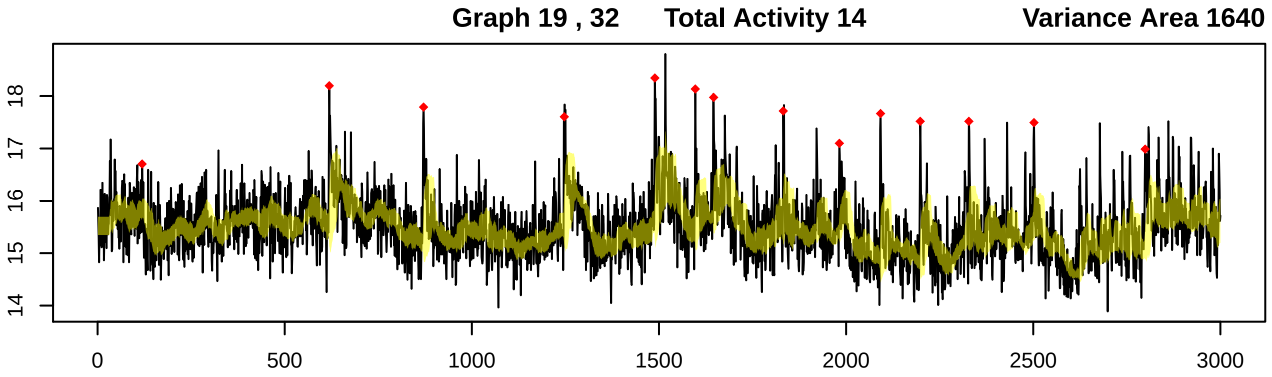
Graph 19 , 32
Total Activity 14
Variance Area 1640
14
15
16
17
18
0
500
1000
1500
2000
2500
3000

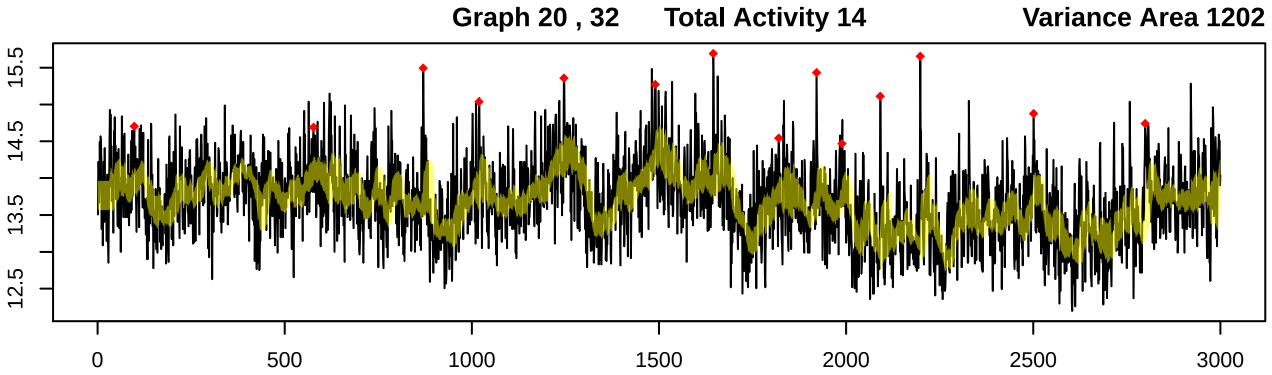
Graph 20 , 32
Total Activity 14
Variance Area 1202
12.5
13.5
14.5
15.5
0
500
1000
1500
2000
2500
3000

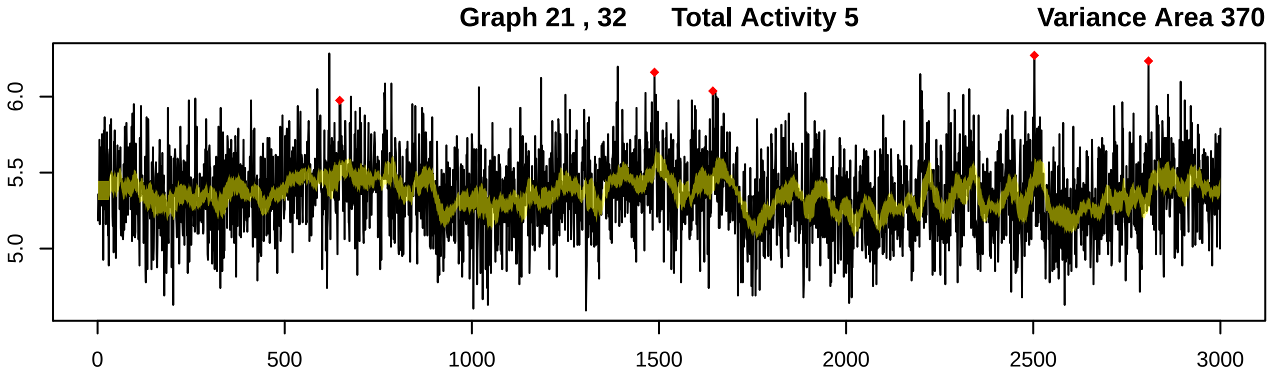
Graph 21 , 32
Total Activity 5
Variance Area 370
5.0
5.5
6.0
0
500
1000
1500
2000
2500
3000

Graph 25 , 32 Total Activity 5 Variance Area 292

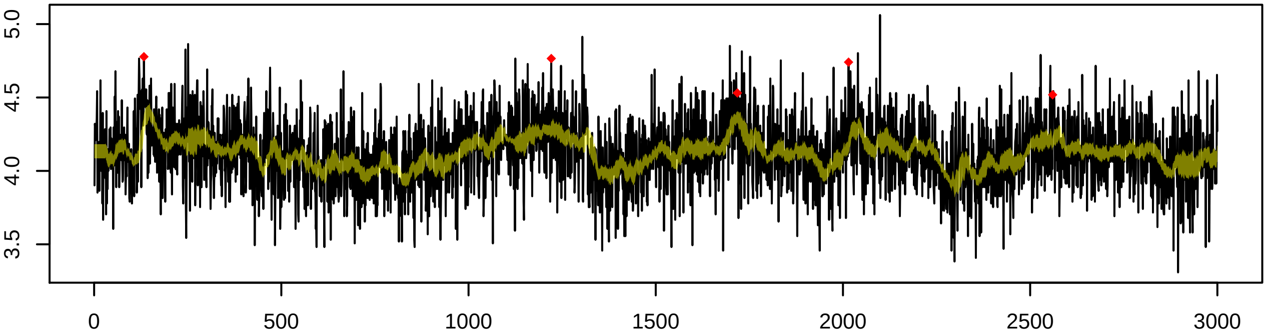

Graph 26 , 32 Total Activity 5 Variance Area 342

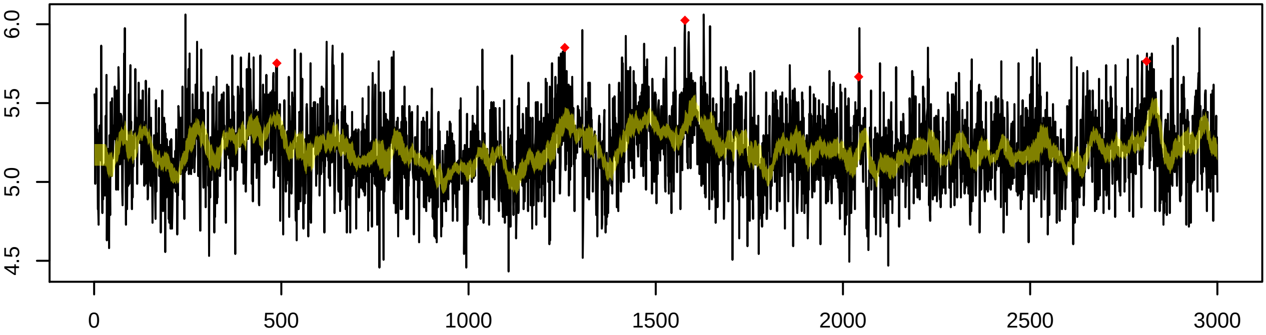

Graph 27 , 32 Total Activity 2 Variance Area 362

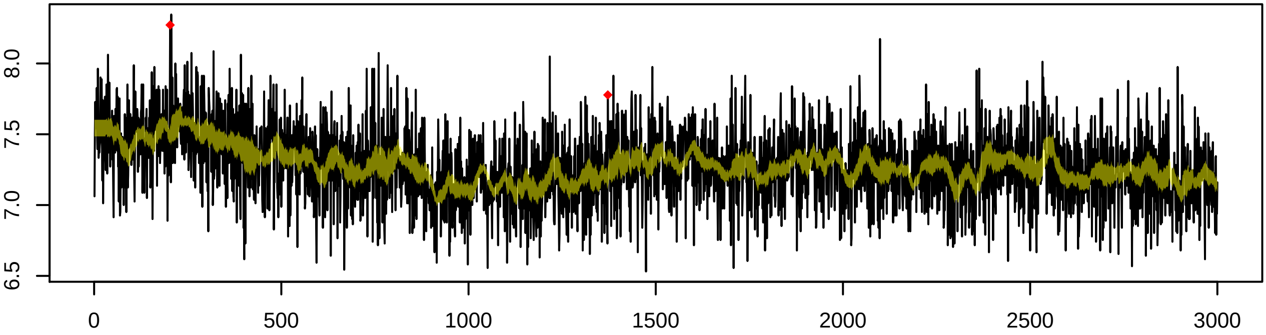

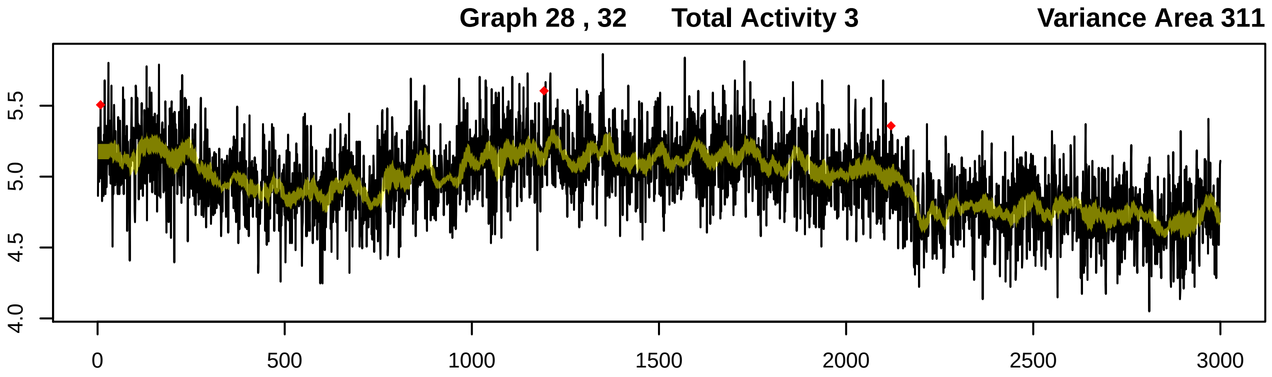
Graph 28 , 32
Total Activity 3
Variance Area 311
4.0
4.5
5.0
5.5
0
500
1000
1500
2000
2500
3000

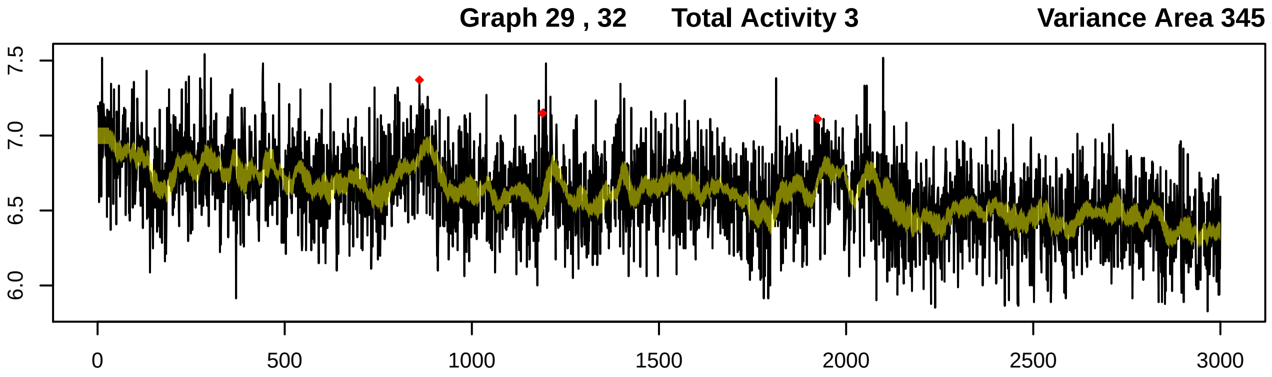
Graph 29 , 32
Total Activity 3
Variance Area 345
6.0
6.5
7.0
7.5
0
500
1000
1500
2000
2500
3000

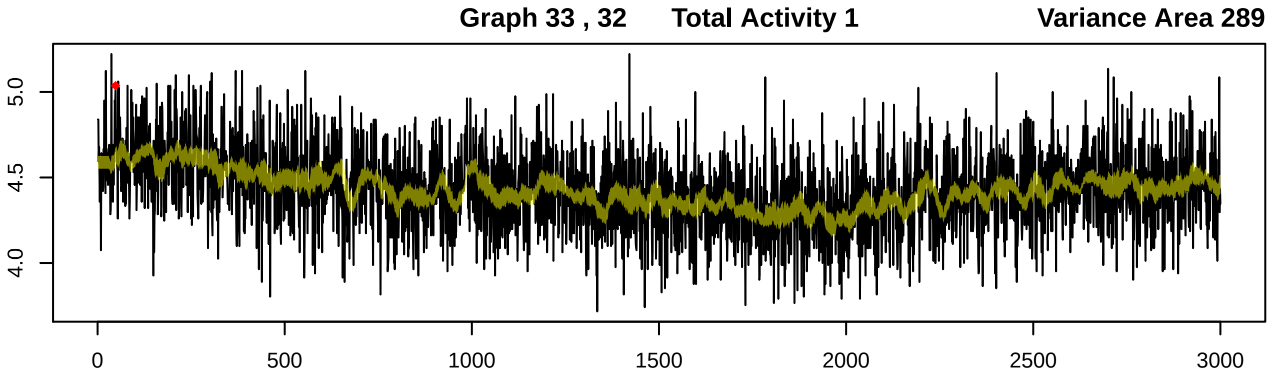
Graph 33 , 32
Total Activity 1
Variance Area 289
4.0
4.5
5.0
0
500
1000
1500
2000
2500
3000

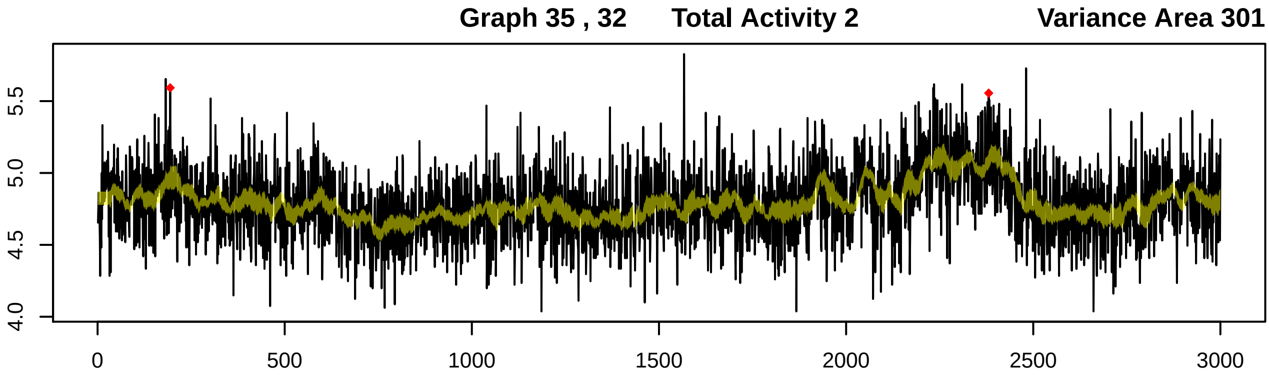
Graph 35 , 32
Total Activity 2
Variance Area 301
4.0
4.5
5.0
5.5
0
500
1000
1500
2000
2500
3000

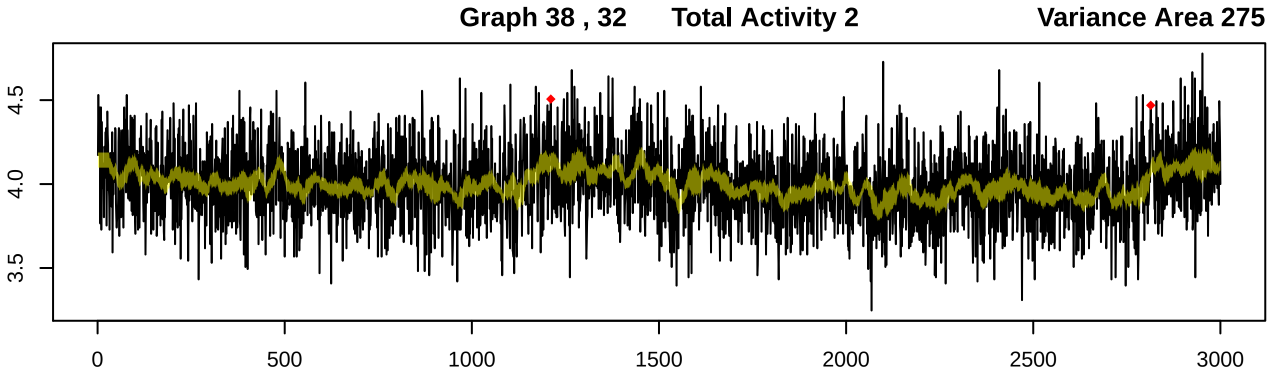
Graph 38 , 32
Total Activity 2
Variance Area 275
3.5
4.0
4.5
0
500
1000
1500
2000
2500
3000

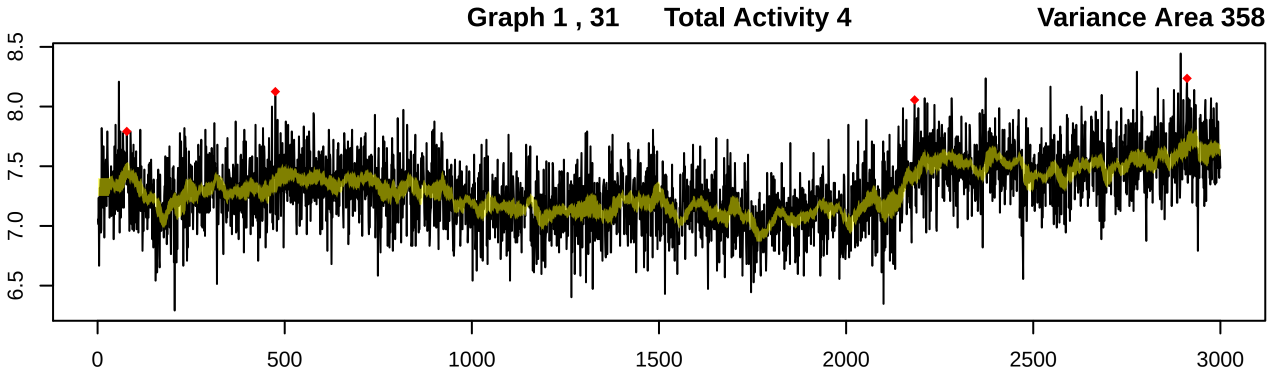
Graph 1 , 31
Total Activity 4
Variance Area 358
6.5
7.0
7.5
8.0
8.5
0
500
1000
1500
2000
2500
3000

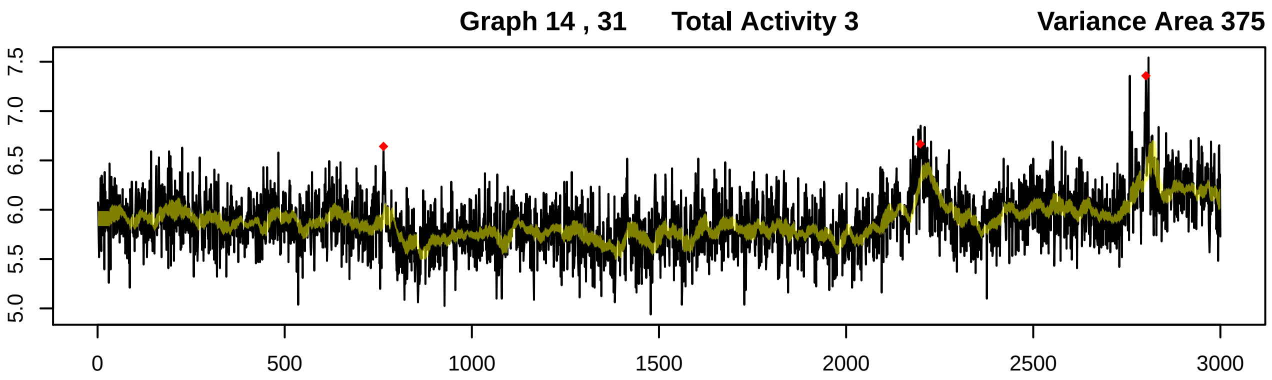
Graph 14 , 31 Total Activity 3 Variance Area 375
5.0 5.5 6.0 6.5 7.0 7.5
0 500 1000 1500 2000 2500 3000

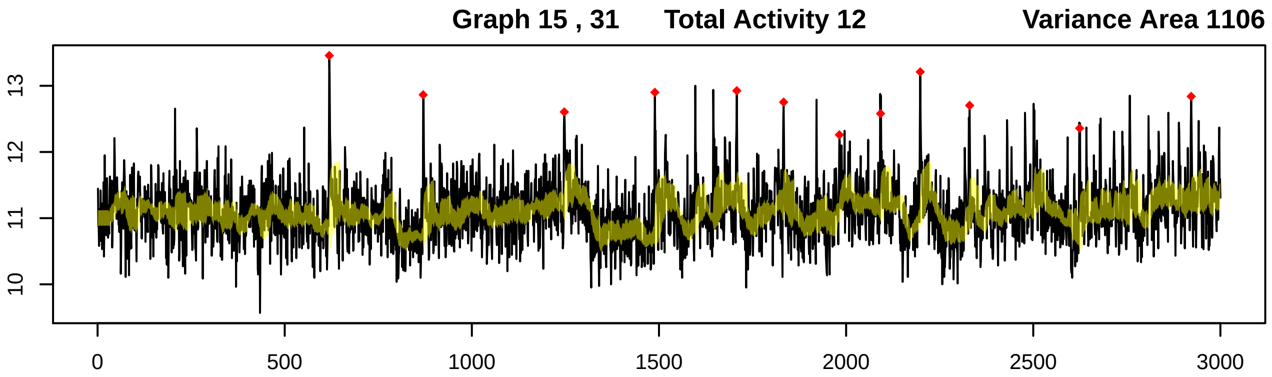
Graph 15 , 31 Total Activity 12 Variance Area 1106
10 11 12 13
0 500 1000 1500 2000 2500 3000

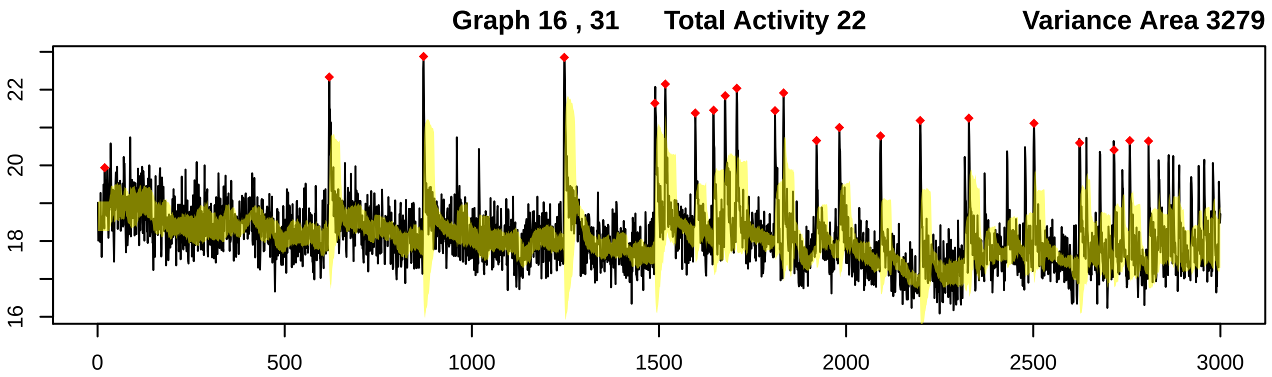
Graph 16 , 31 Total Activity 22 Variance Area 3279
16 18 20 22
0 500 1000 1500 2000 2500 3000

Graph 17 , 31 Total Activity 24 Variance Area 5255

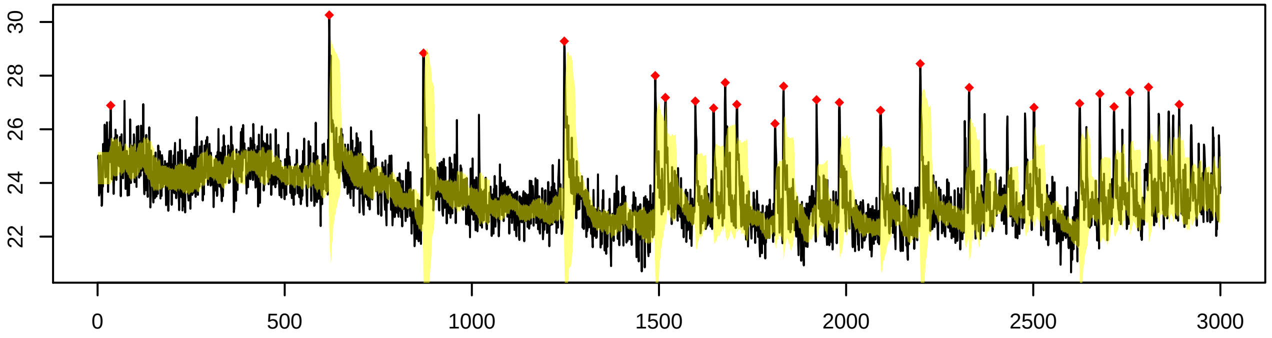

Graph 18 , 31 Total Activity 24 Variance Area 5696

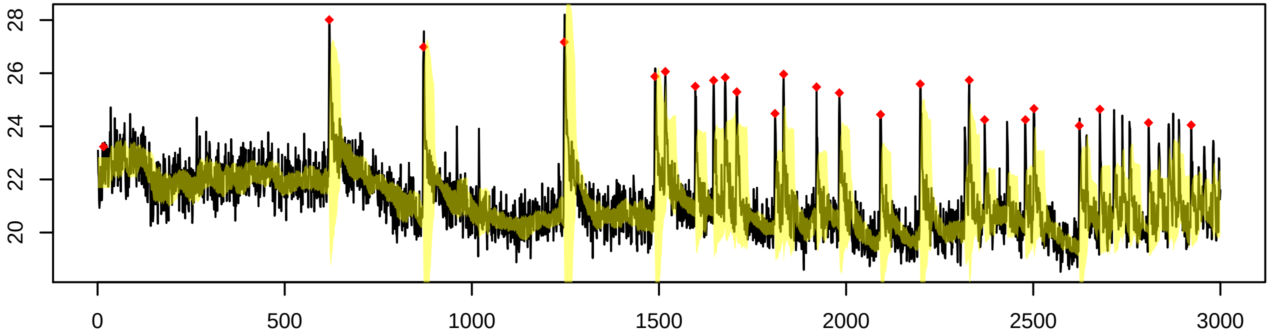

Graph 19 , 31 Total Activity 22 Variance Area 3159

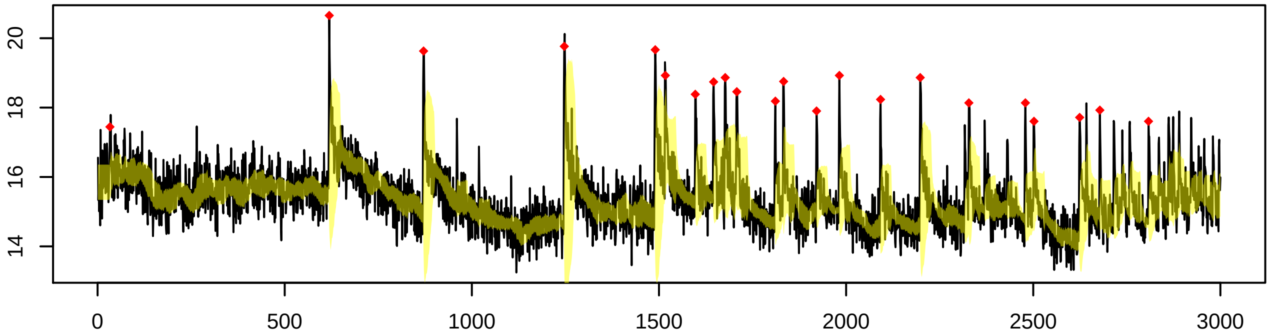

Supplement: S7 Fig — Computation of spontaneously active hippocampal neurons; traces, activity counts, variance area. Shown are 12 of 289 pages of data summary. (PDF) [file pcbi.1006054.s007.pdf]
