# Supplementary material for: An open source tool for automatic spatiotemporal assessment of calcium transients and local ‘signal-close-to-noise’ activity in calcium imaging data
Source: PLoS Comput Biol. 2018 Mar 30;14(3):e1006054. doi: 10.1371/journal.pcbi.1006054 (PMC5895056; doi:10.1371/journal.pcbi.1006054)

Centers of ROI

computed components

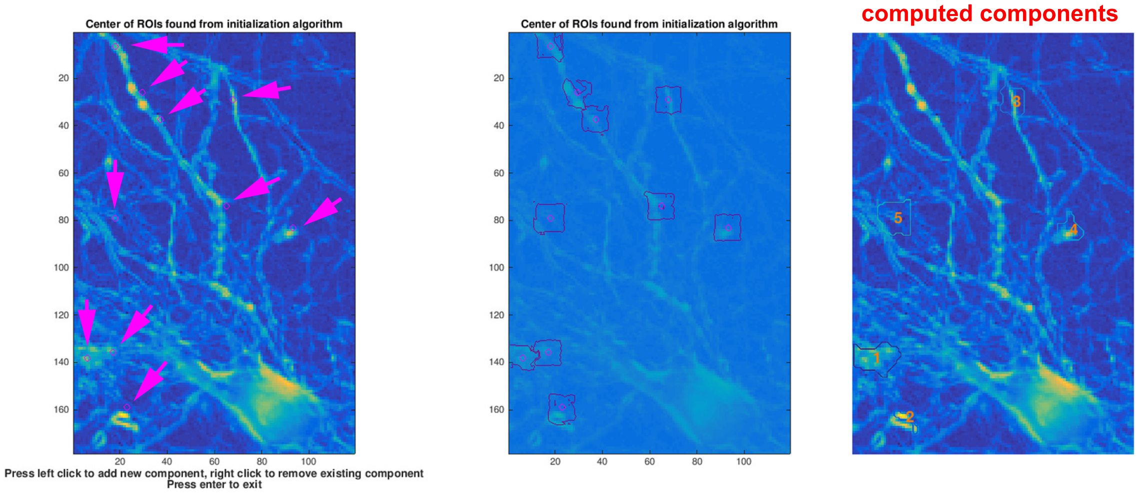

detected component 1

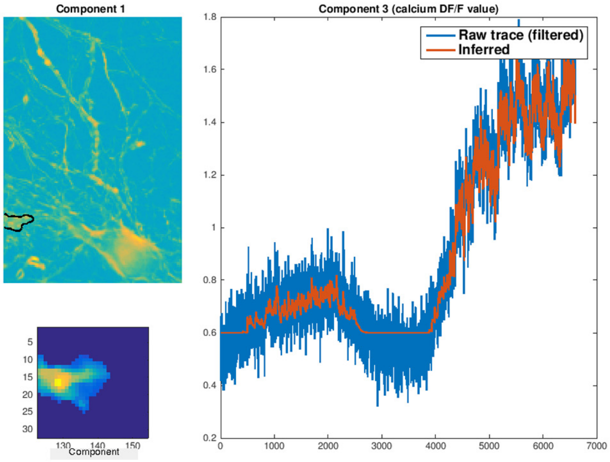

detected component 3

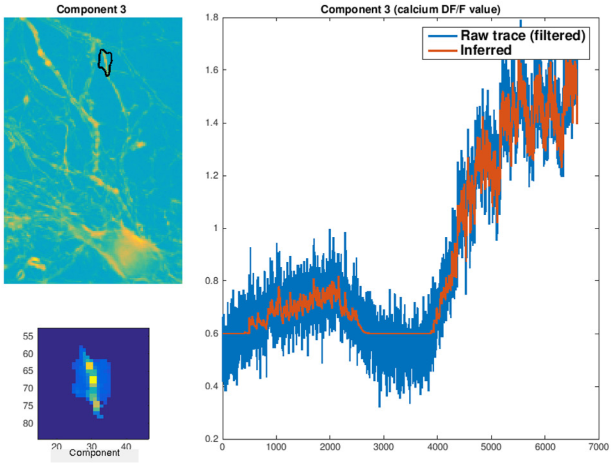

detected component 4

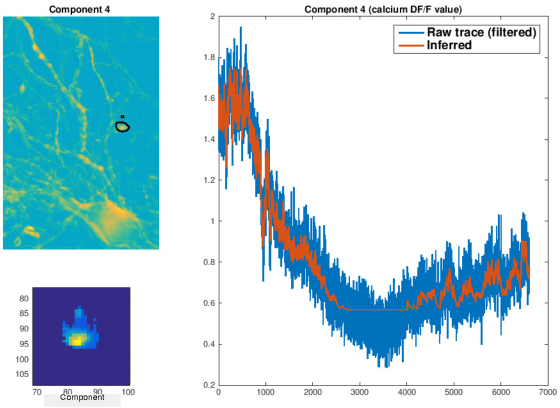

background activity

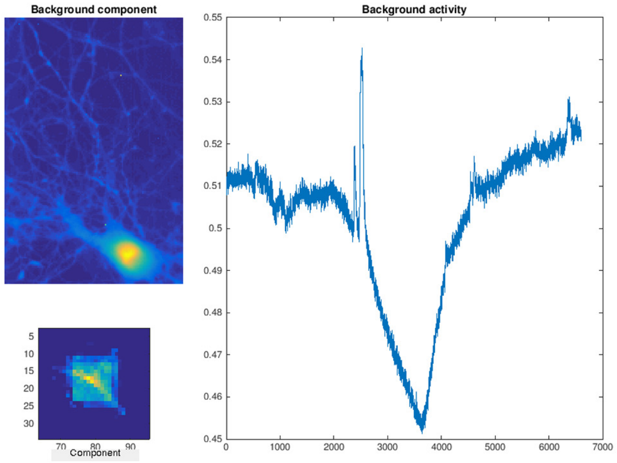

Supplement: S9 Fig — Here, we show the performance of a tool that decomposes the spatiotemporal activity of a neuron into a spatial component to show the local neural structure and temporal components that model the local calcium dynamics [23]. This tool identified ten signal components from the movie (Fig 9) and computed five of these components. (PDF) [file pcbi.1006054.s009.pdf]
