# Supplementary material for: An open source tool for automatic spatiotemporal assessment of calcium transients and local ‘signal-close-to-noise’ activity in calcium imaging data
Source: PLoS Comput Biol. 2018 Mar 30;14(3):e1006054. doi: 10.1371/journal.pcbi.1006054 (PMC5895056; doi:10.1371/journal.pcbi.1006054)
Supplement: S1 Manual — (PDF) [file pcbi.1006054.s010.pdf]

Supplementary material: User manual

An open source tool for automatic spatiotemporal assessment of calcium transients and local 'signal-close-to-noise' activity in calcium imaging data

Juan Prada, Manju Sasi[2], Corinna Martin[2], Sibylle Jablonka[2], Thomas Dandekar[1], and Robert Blum[2]

[1] Department of Bioinformatics, University of Würzburg, Würzburg, Germany

[2] Institute of Clinical Neurobiology, University Hospital, University of Würzburg, Würzburg, Germany

published in PLOS Computational Biology, 2018

The open source tool is available at GitHub, a development platform.

Information, test data and raw data, as well as further developments of the computational tool will be provided at:

https://www.biozentrum.uni-wuerzburg.de/bioinfo/computing/neuralactivitycubic/

NA^3

Automated Calcium Signal Assessment

from x,y-t Calcium Imaging Data

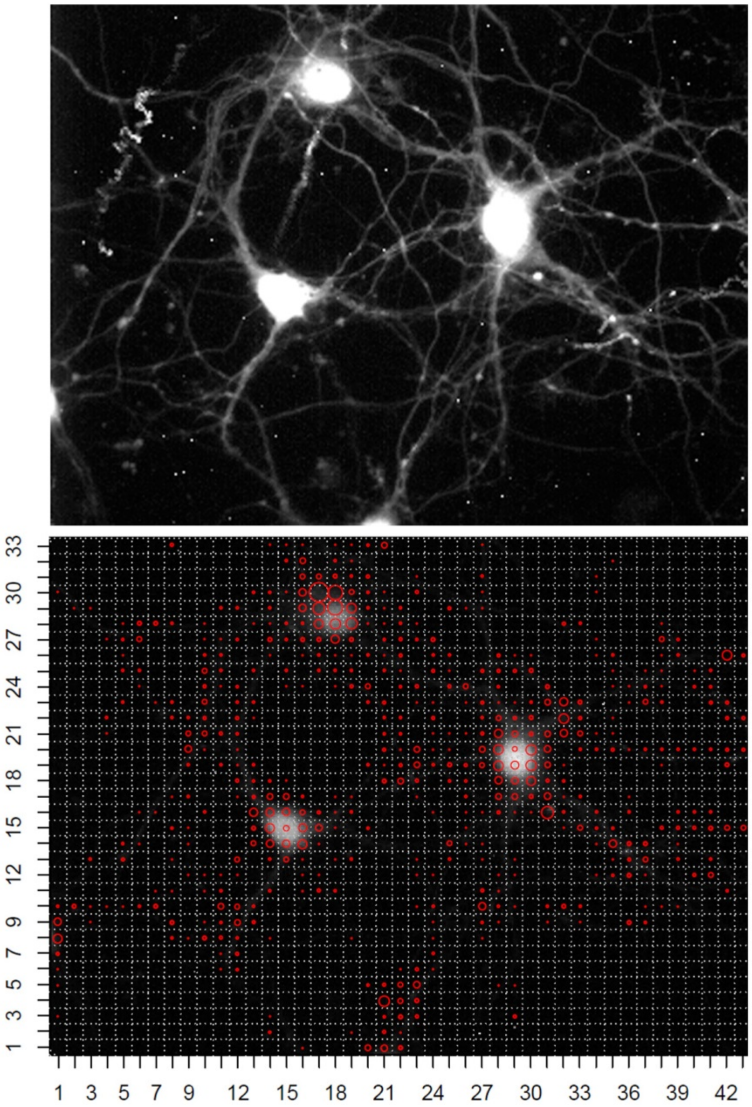

User guide 2018 – I

Written by Juan Prada[1] and Robert Blum[2].

[1] Department of Bioinformatics, University of Würzburg, Würzburg, Germany

[2] Institute of Clinical Neurobiology, University hospital, University of Würzburg, Würzburg, Germany

Content

General Comments 4
I Installation 5
II Starting the program and NA^3 9
III Activity analysis 12
IV Variance area analysis 17
V General Activity Tendency 18
VI The ROI (region of interest) tool: *NeuronActivityROI-2* 19
VII Data Output 22
VIII Branch formation in the CWT algorithm 27
IX Test data 30

General Comments

We created a bioinformatics tool to facilitate the unbiased assessment of calcium signals from x,y-t imaging raw data. The tool can be adapted for use with diverse imaging data sets in which changes in bit-values show a transient-like character.

To compute calcium transients from raw image material the tool has been split in two stages. Signal extraction is computed on ImageJ and activity events are calculated on 'R' (https://www.r-project.org).

Both computations are embedded in the Bio7 environment, an open platform (http://bio7.org/).

To date, the software has been tested on Windows with the BIO7 2.4 for Windows 64 bits.

The system was also proved on a Linux 3.11.10-7. openSUSE 13.1 (Bottle)(x86_64) and a Mac OS X Yosemite 10.10.5, and some other Mac systems.

The application was developed on a Windows X64 Intel core i-7 machine with 16 Gigabyte (GB) of RAM memory. For time series analysis, a Windows X64 Intel core i-5 machine with 4 Gigabyte of RAM memory is sufficient, but we recommend having more RAM (e.g. 8 – 16 GB).

The idea is to use an unbiased x,y-grid, to include all pixels in a imaging video and to offer an activity map. The tool avoids a preferential look at one activity mode of a neuron, e.g. the spiking behavior of neuronal somata, but instead tries to find any activity by detecting signals-close-to-noise.

The tool bases its algorithm on a continuous wavelet transform-guided peak detection to identify calcium signal candidates. Intensity signals are automatically calculated in the whole x,y-field of the image series. For this, a grid with defined pixel size separates the x,y-field in independently analyzed grid windows. The tool can also be used to compute ROI information.

The tool can also compute the signal variance in a sliding window to visualize activity-dependent signal fluctuations.

A dataset is created and shows loci of activity, individual traces of active loci, and calculates a number of activity events.

The tool is powerful to assess calcium spikes, but is more powerful to look for local calcium events and 'signal-close-to-noise' activity.

I Installation

1) Download, unzip and open Bio7 following the instructions in the download section in the Bio7 website (http://bio7.org/). The 64bit version is recommended. Bio7 already includes ImageJ and R.

NOTE 1: *R is already included in Bio7 for Windows. For* ***Linux****, R must be downloaded separately. Please follow the instructions in the Bio7 website. Make sure that a direct access to "R" exists. You can check this in the folder of Bio7 (see next image). If there is no direct access, the user has to create it.*

NOTE 2 for Mac users: *Please find* ***Special Installation recommendations for Mac computers*** *at the end of this chapter.*

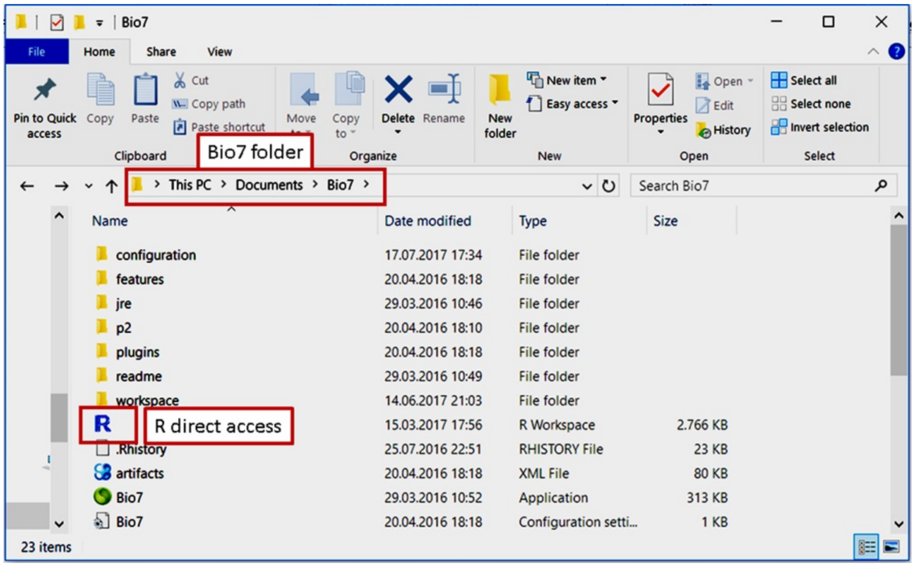

2) Download the Neuron Activity Analysis zip file from GitHub (https://github.com/jpits30/NeuronActivityTool). Another version contains an additional ROI tool https://github.com/jpits30/NeuronActivityTool_ROI (see Chapter VI). Unzip the file in some known location.

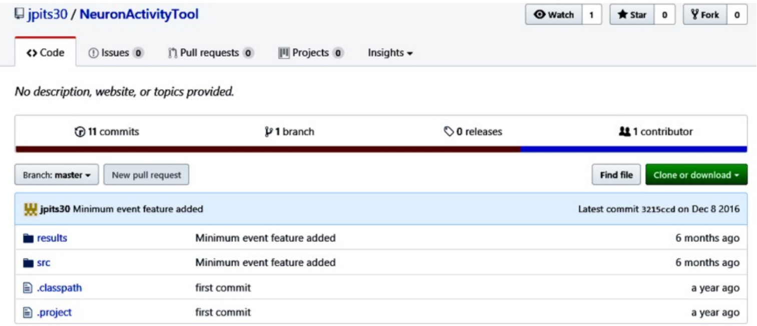

NOTE: *The Zip software will probably change the name of the folder (in most cases adding "-master").*

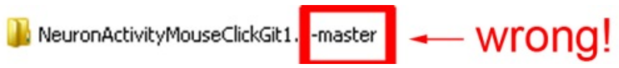

Make sure to rename the folder before moving forward. The name of the folder must be **neuronActivityMouseClickGit1.0**.

3) Open Bio7, go to the **File** menu and click **Import**.

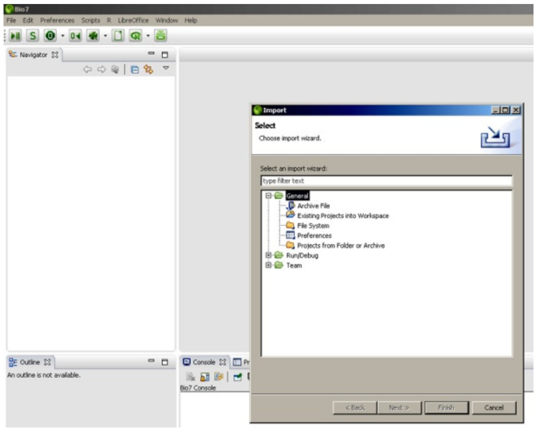

4) Select **Existing Projects into Workspace** option then click **Next.**

5) Browse to the directory where you unzipped the file, select the **neuronActivityMouseClickGit1.0** folder (be careful because the ZIP software might have changed the name). Check the box **Copy projects into workspace** and click **Finish**.

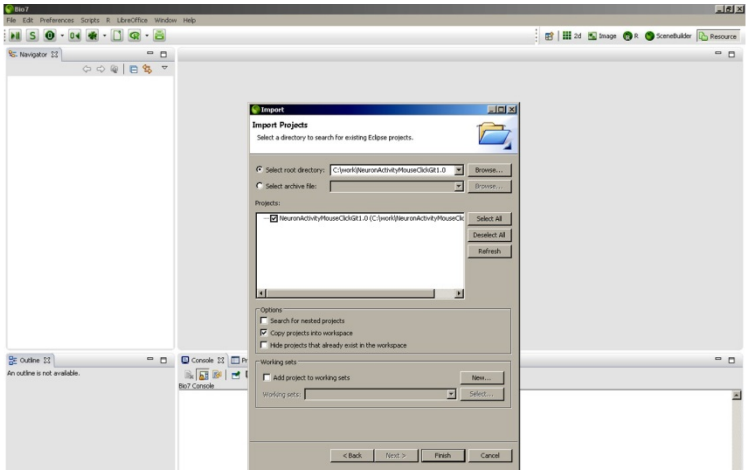

6) The program will ask if he should recalculate paths, make sure you say **YES**

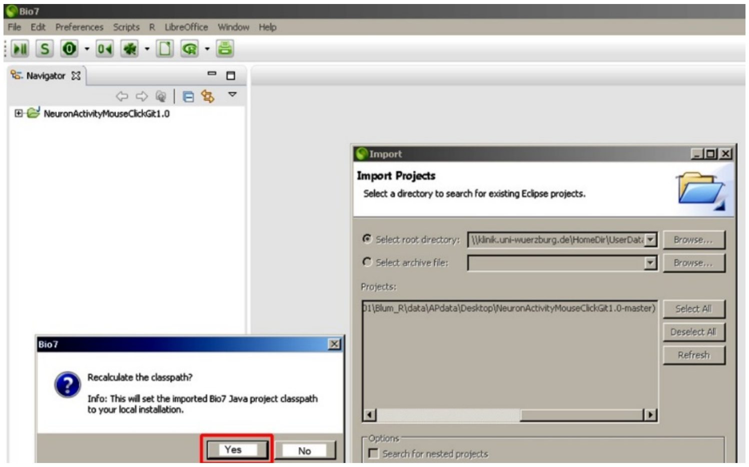

7) File structure when installation is completed.

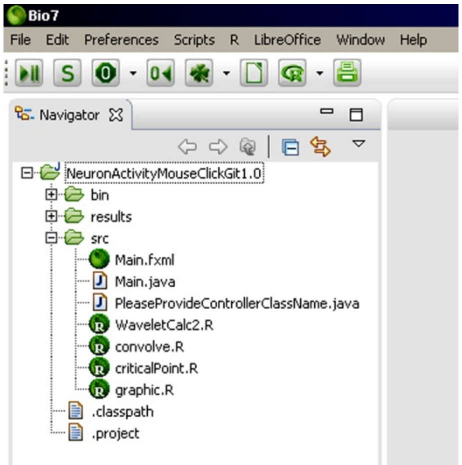

8) Continue to the use instructions.

Note: When you are using the program for a first time, it may ask you to select a HTTPS CRAN mirror. Select one to complete the "R" installation in Bio7.

Special Installation recommendations for Mac computers

Mac Computers have many advantages and provide high quality performance, however to use our software on this operating system, the following recommendations are useful:

1) In case that Bio7 is not recognized as secure software:

This may happen if you try to open Bio7 for the first time. It could be related to the Apple security restrictions. To solve this problem, see this link: http://bio7.622846.n4.nabble.com/Bio-7-2-2-is-not-starting-in-Mac-yosemite-td4640276.html

2) In case that the connection to R is not working:

For Mac versions, R must be downloaded separately. Please follow the instructions in the Bio7 website under this link. http://bio7.org/manual/Main.html#toc-Subsubsection-2.1.2.3.

The default directory where Bio7 looks for ‘R’ might not be correct. To solve this problem, see this link: http://bio7.622846.n4.nabble.com/Rserve-connection-Mac-user-td4640296.html

3) In case that R is not found in the Bio7 directory:

Here the problem is that the working directory in ‘R’ do not correctly point towards the folder called **‘workspace’** from Bio7. In most cases, the path to this folder is the following:
Applications/Bio7.app/Contents/MacOS/workspace/

However , in some Mac systems this folder is at another place. In this case, the user has to find the path to the workspace folder and can copy the path to BIO7, so that this path appears in the indicated line as explained as follows:

1. Users must first open Bio7 and need to navigate to the folder **src in the NeuronActivityMouseClickGit1.0 (see image below)**.
2. Then open the file WaveletCalc2.R (see image below).
3. The following lines must be changed in the „WaveletCalc2.R“.
4. The user has to add a „#“ symbol at the beginning of line 17
5. Remove remove the „#“ symbol at the beginning of the line 19.
   Please see the image below.
6. Change line 19, to show the real path to the workspace folder (see red arrow below).

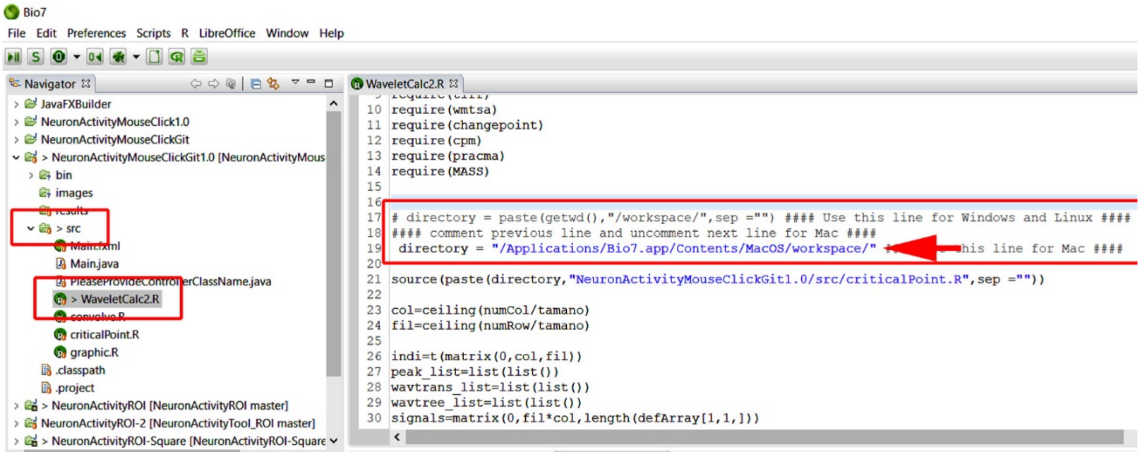

II Starting the program and NA³

1) Open the program Bio7 and open the folder **src**.

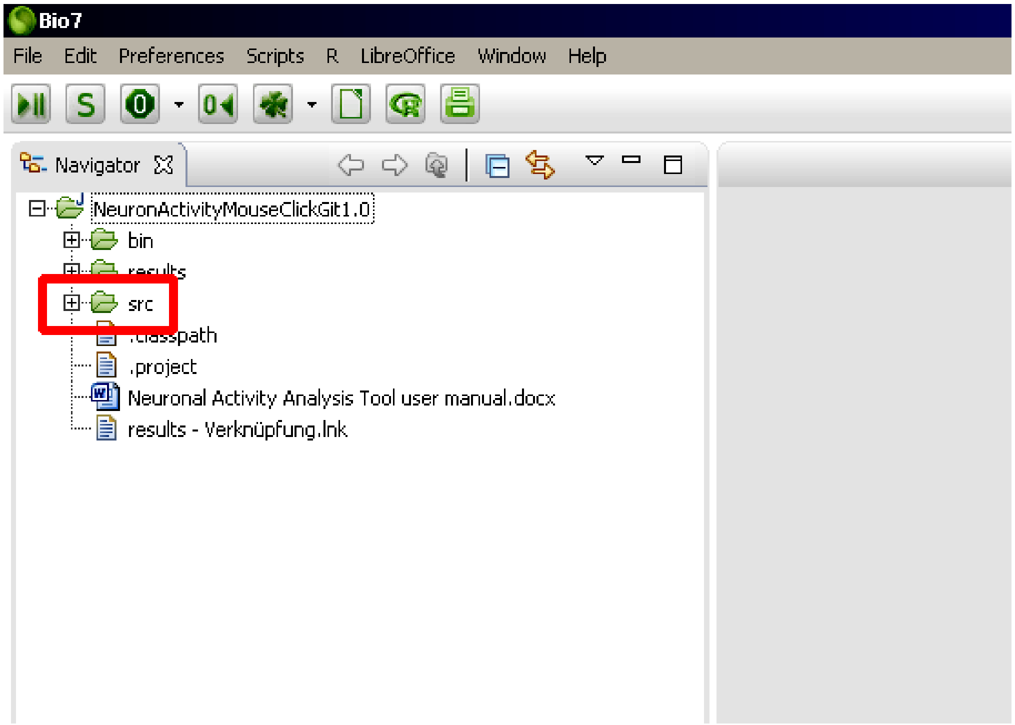

2) Open the **Main.java** file.

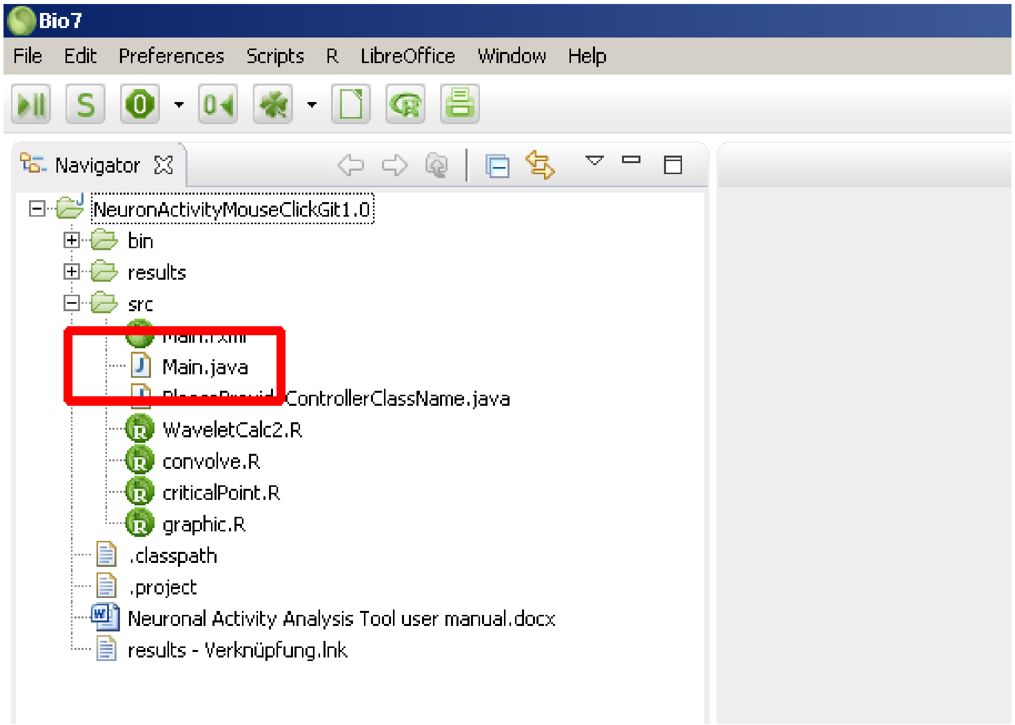

3) With the file open, click on the **Java compiler** icon in the Bio7 tool bar. The program will be compiled. In the lower right corner of the Bio7 window a bar of progress is shown.

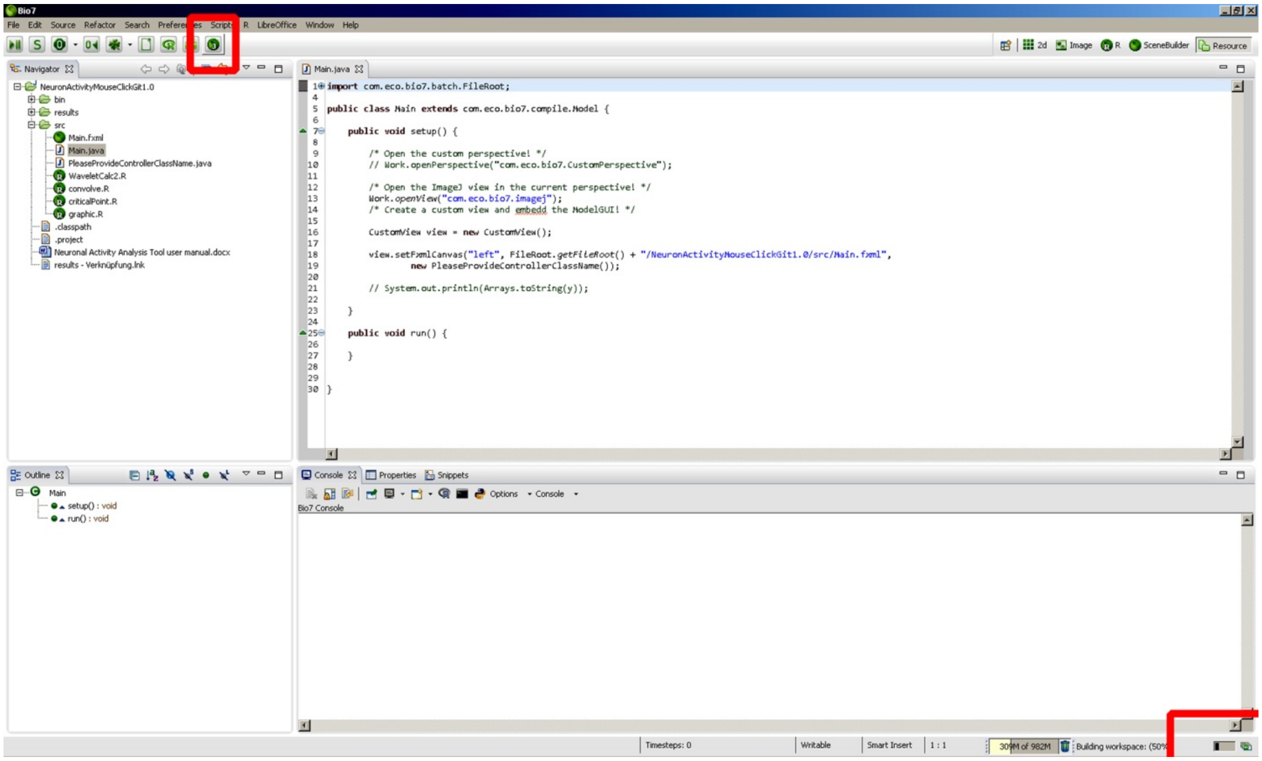

4) Start the tool by a click on the **setup icon**.

The **Neuron Activity Analysis tool** menu will show up.

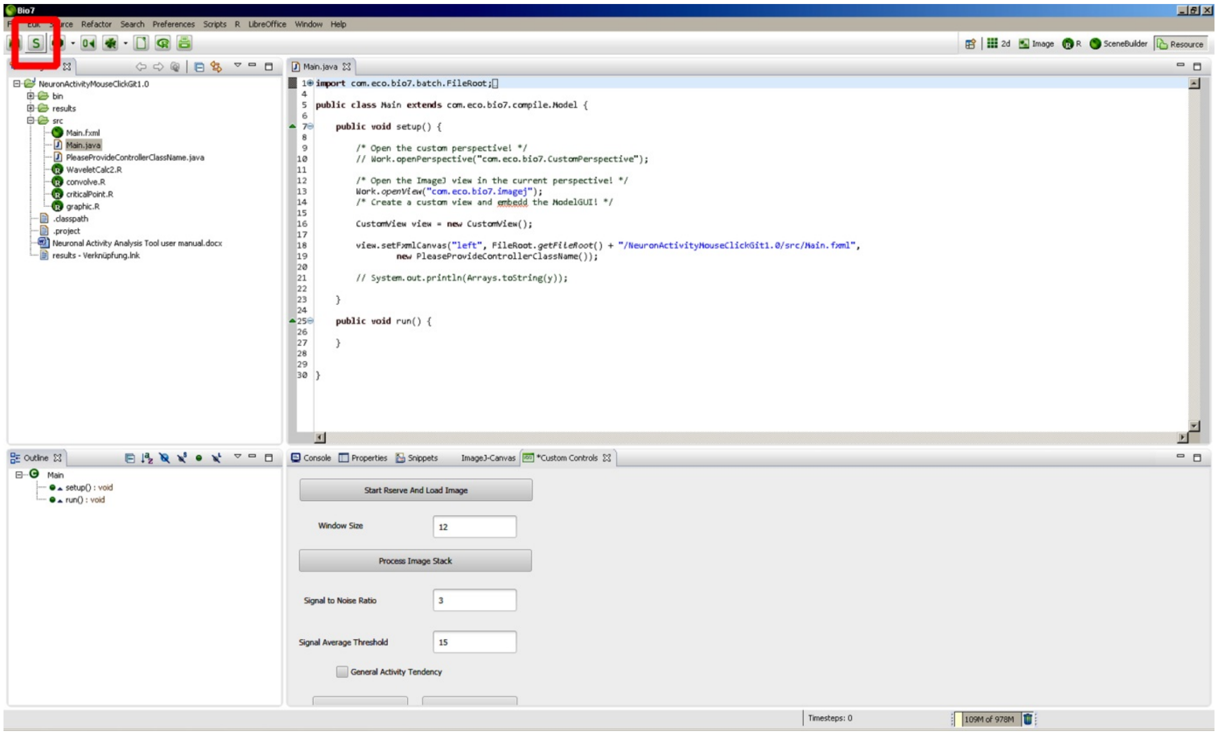

5) For convenience, drag the window to the upper right corner. The activity analysis tool bar and the ImageJ implementation are ready for use.

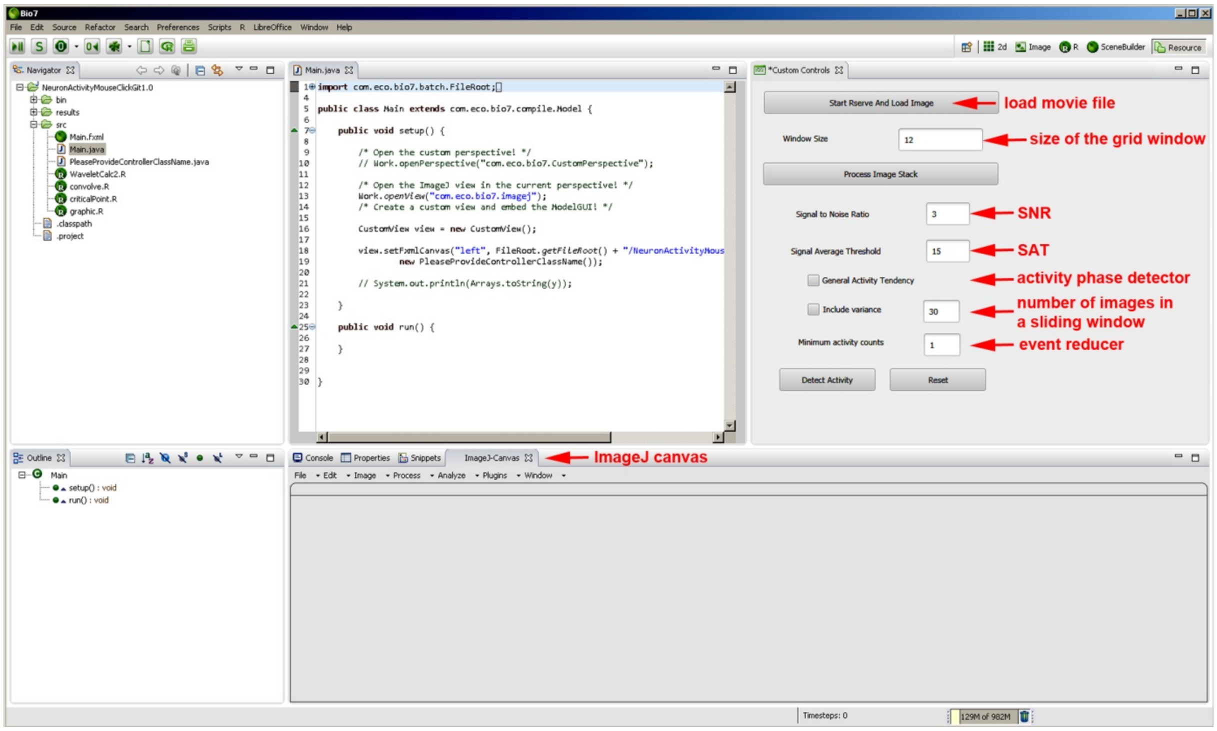

Short description of the menu:

Start Rserve And Load Image: import of the raw image material

Window Size: defines the size of the grid window (12; means 12x12 pixel)

Process Image Stack: calculation of intensity signals

Signal to Noise Ratio: definition of the stringency criterion SNR

Signal Average Threshold: definition of the detection threshold

General Activity Tendency: defines and calculates signal phases.

Include variance: labels and calculates fluctuations in the signal

Minimum activity counts: Event reducer (The number "1" means that only those grid windows are displayed and counted, in which one or more than one activity events were found.

III Activity analysis

1) Click on **Start Rserve And Load Image** window, select an avi-file containing the uncompressed image material. The AVI Reader window appears and asks for the wished frame window (e.g. image 1 to 3000). Confirm with **OK.** The corresponding video appears in the ImageJ window. NOTE: The first time this is done R will have to install the packages it needs to use in the application, simply click OK when asked.

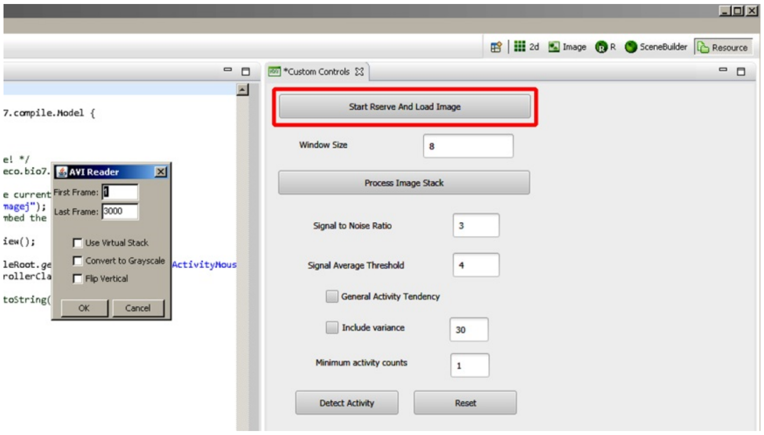

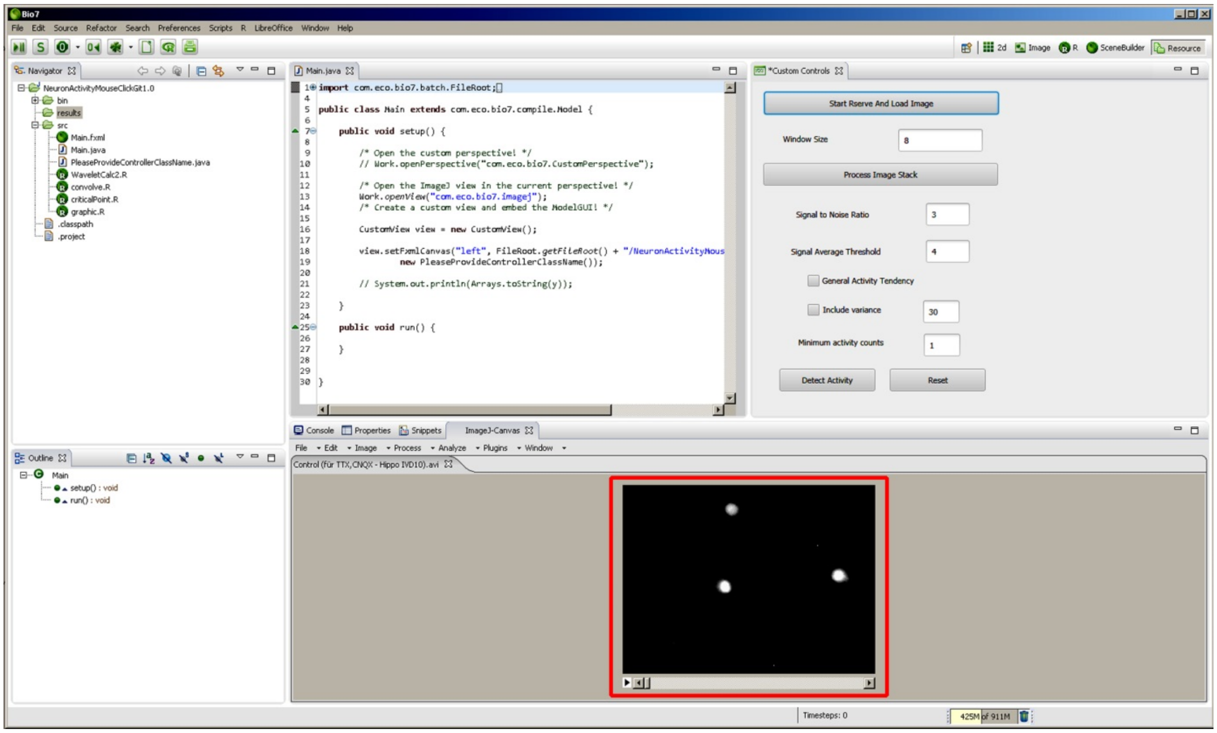

2) Determine a threshold value. This can either been done in BIO7 or in any other image analysis program. In BIO7 select a small ROI (yellow rectangle) either in the background or at any other reference locus in the x,y-field. Open the **Analyze** window of ImageJ. Click on "Measure" to determine the mean grey value (mean intensity bit value) in the background ROI.

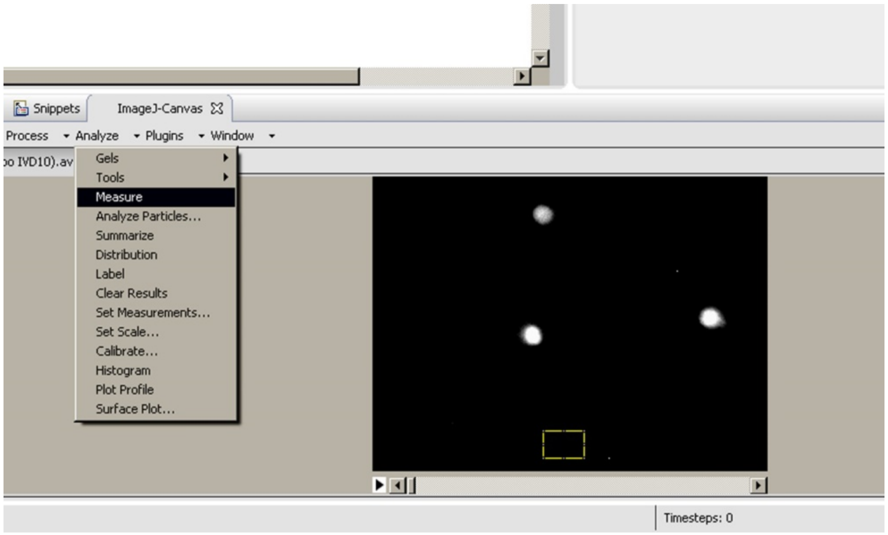

3) Set the **Signal Average Threshold** according to the rule: rounded mean value of e.g. the background or a reference signal. For example: Here, a mean value of 3.391 was determined. This value was rounded up to 4. Therefore, a SAT of 4 is chosen for this analysis. The CWT computation does not depend on a threshold to be accurate. One can use this value to restrict the computation to values above the background, or to restrict the computation to brighter areas.

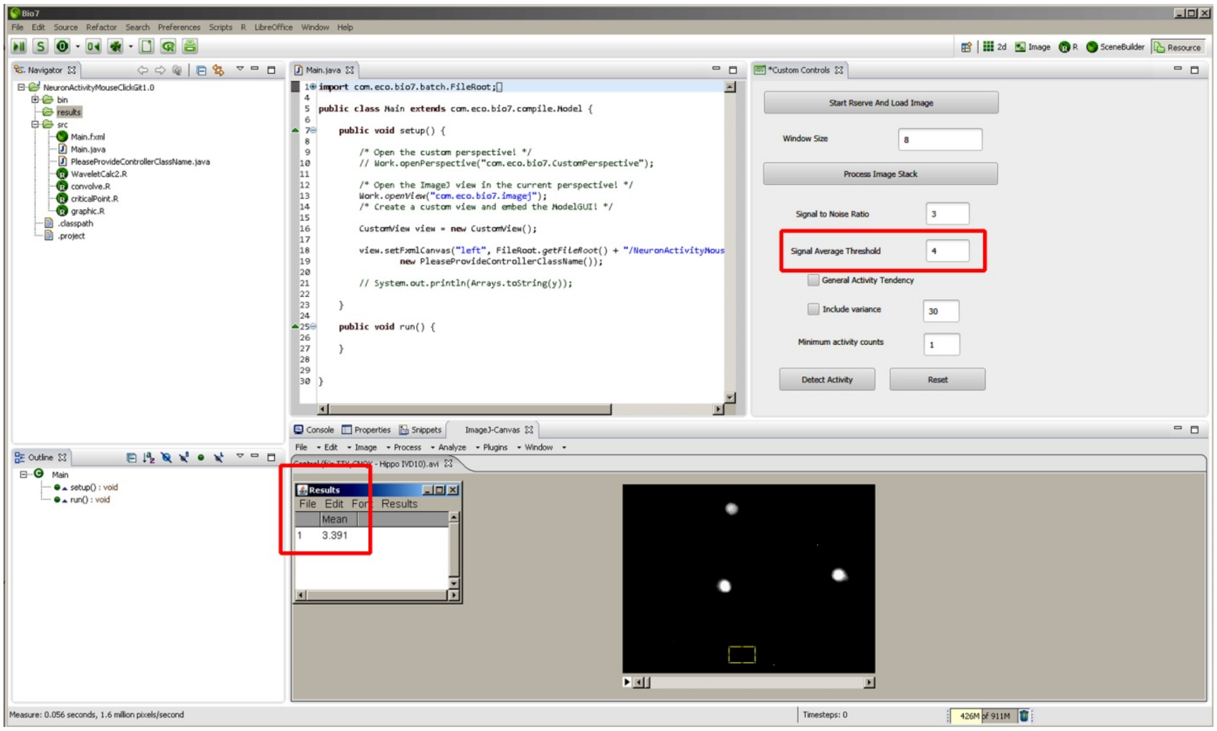

4) Select the size of the grid window according to the size of the neuronal structures. We routinely use a **Window Size** value of 12 – 8, meaning a grid of 12 x 12 or 8 x 8 pixel.

Select the **Signal to Noise Ratio** to tune the stringency of the tool. We recommend a SNR of 2.5 as default setting for an initial assessment of data with indicator dyes such as Oregon Green BAPTA1 (Kd=170 nM). High SNR values of 3 – 4 will preferentially compute “larger” calcium transients, while SNR values of 1.5 – 2 will compute events which are close to the signal noise. Details are explained and discussed in the corresponding publication.

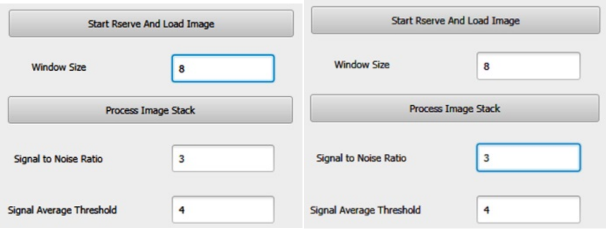

5) Click **Process Image Stack**. The signal intensity values are computed. The progress is seen in the right lower corner. Wait until this calculation is finished.

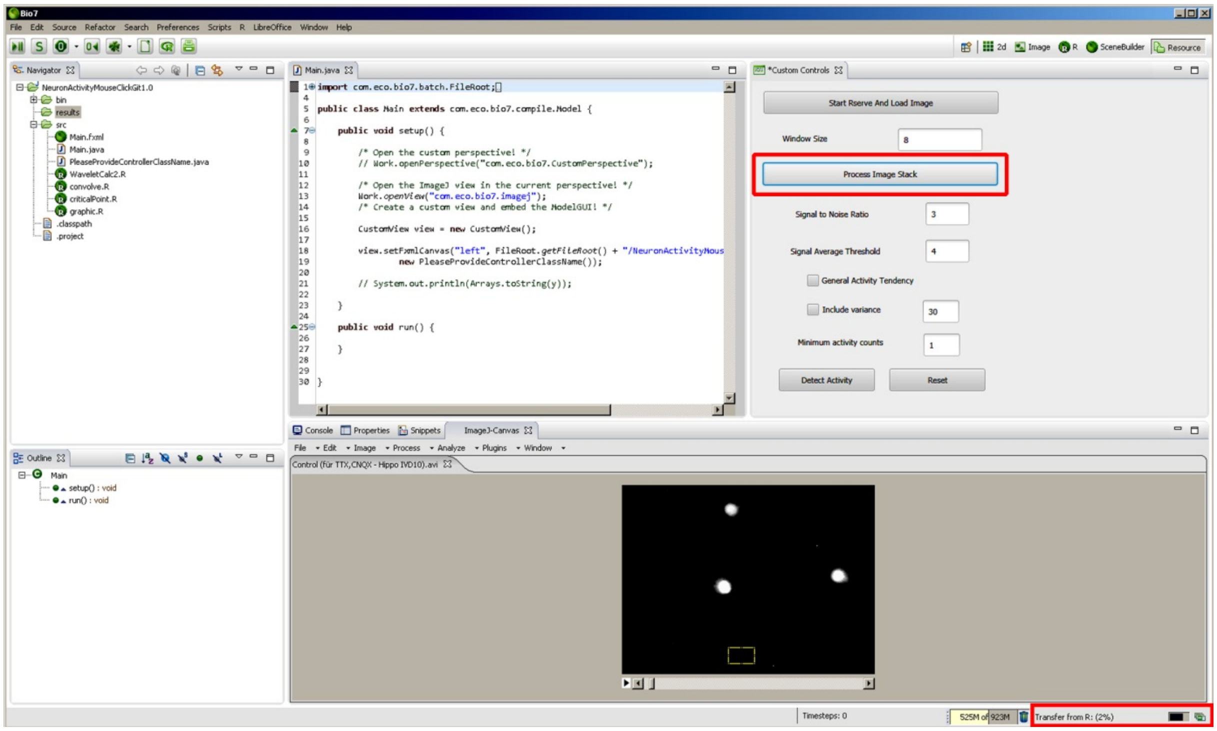

6) Click **Detect Activity**. The calcium signal candidates are computed. The progress is seen in the right lower corner.

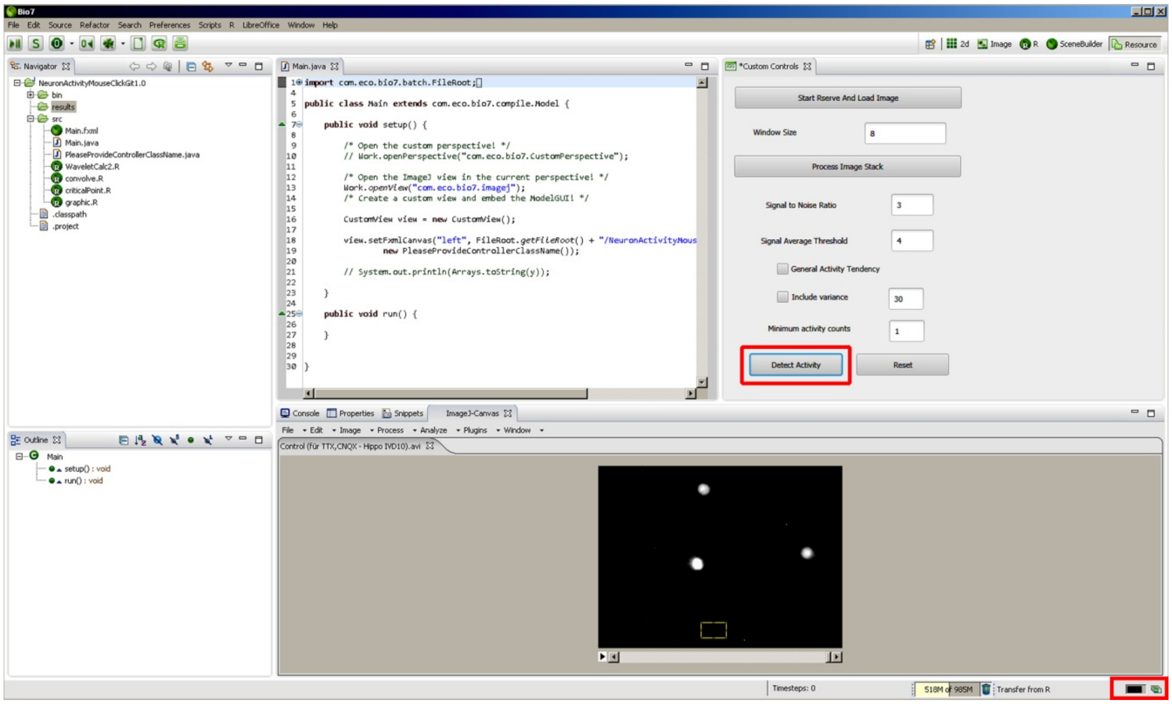

7) When the computation is finished, an image is formed showing the distribution of the computed activity events per grid window as red circles. An output pdf file and a text file are created and put to the results folder. The results are best opened from the file manager system. A mouse click on an individual grid window (red circle) opens an info window. Confirming the Info with **Ok** opens the signal trace in a new plot-window.

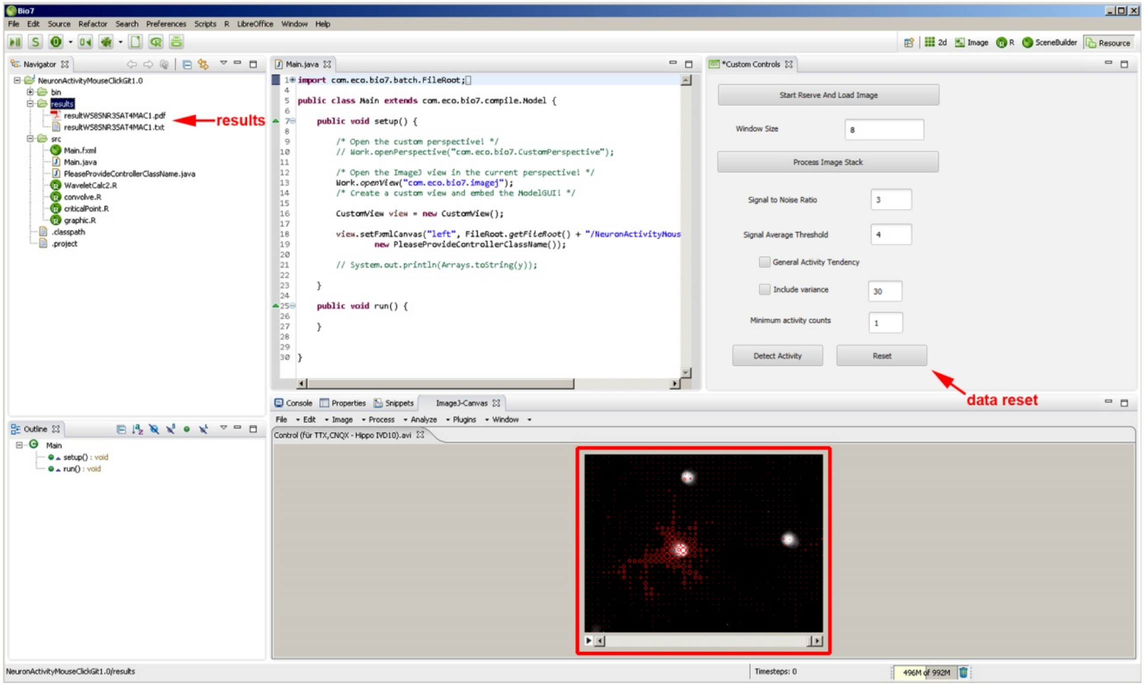

8) Activating a red circle opens an **Info!** window.

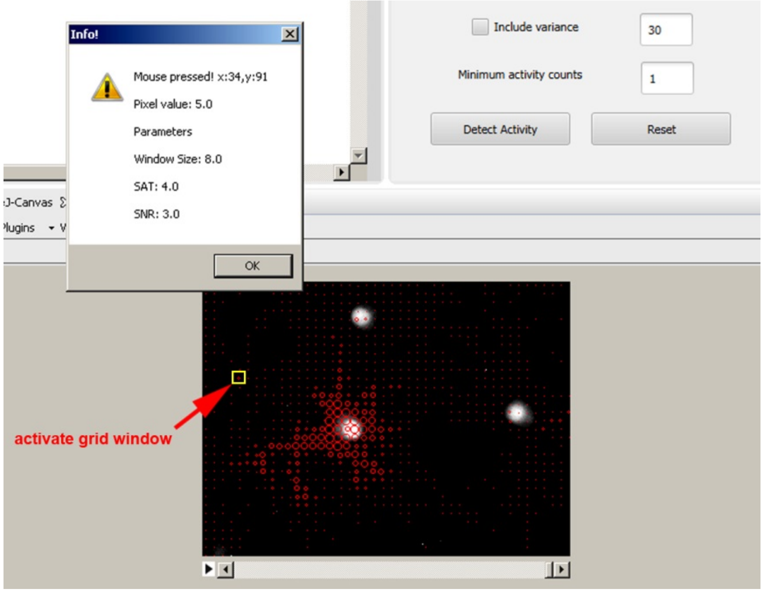

9) The trace underlying this grid window opens.

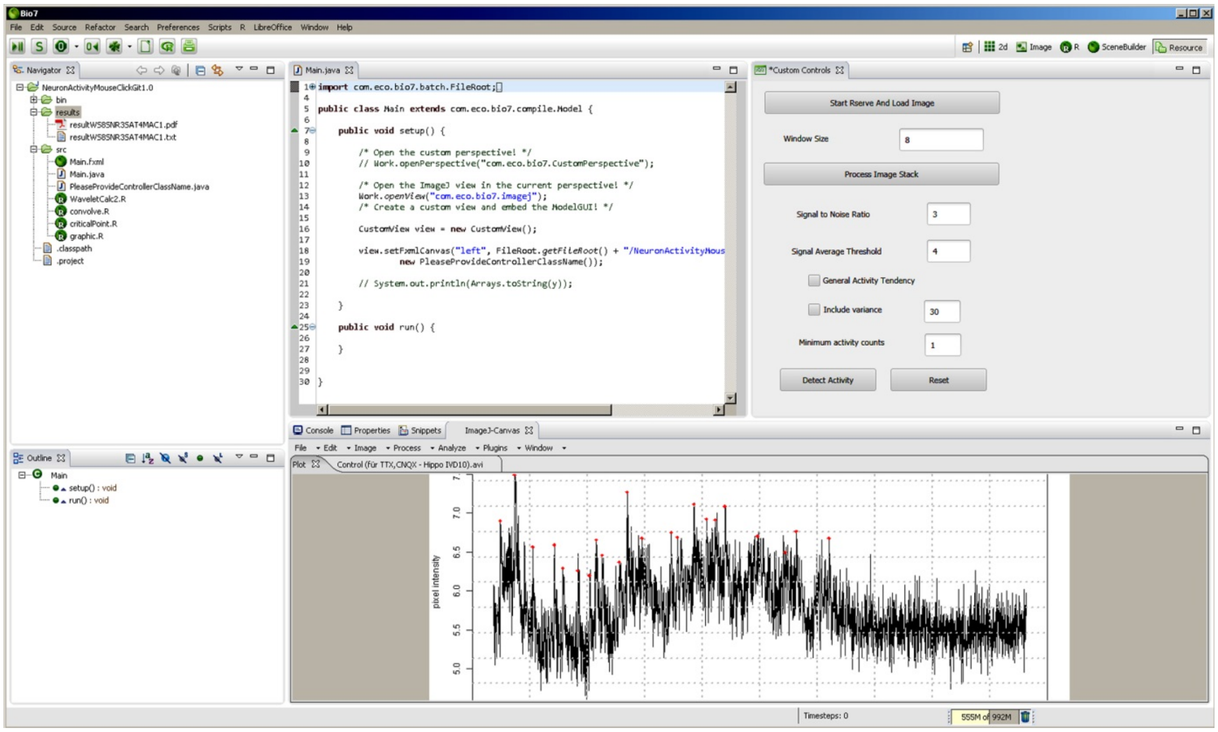

10) The **Reset** function must be used before new SAT or SNR settings can be computed.

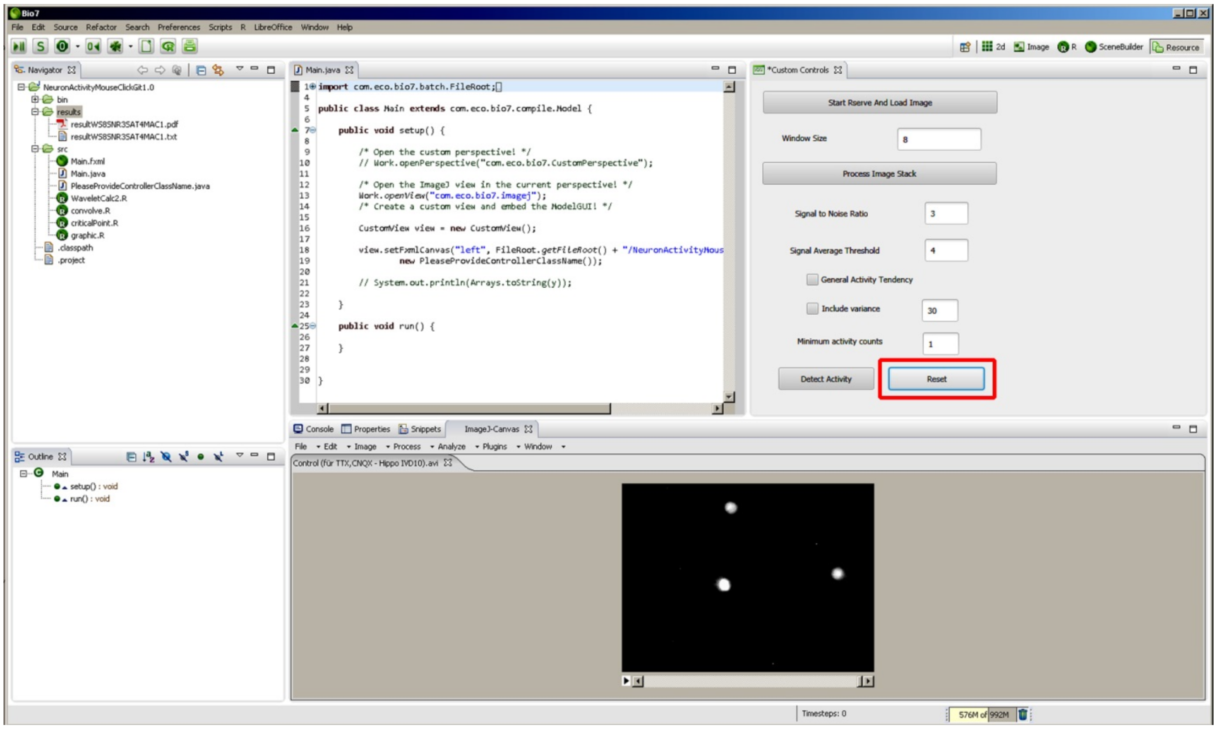

IV Variance area analysis

1) To calculate the variance area, activate the **Include variance** tool. In the output data, the variance area will be calculated on base of a sliding window. The number of images which are used by this tool can be selected by the user (here 30 image frames).

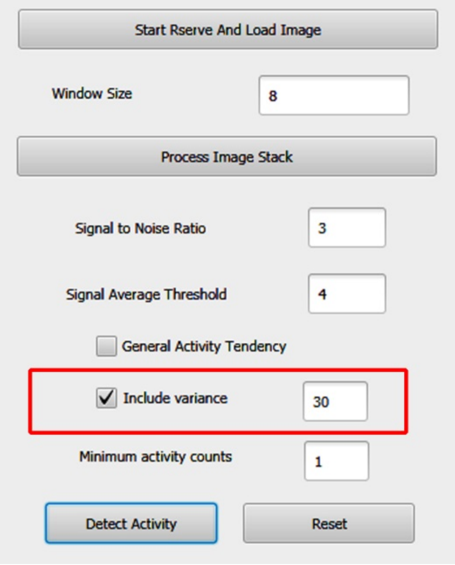

V General Activity Tendency

1) To calculate and determine phases of long-lasting increase in calcium levels, activate the **General Activity Tendency** tool. This tool may be helpful for the identification of grid windows with a long-lasting increase of calcium levels after a stimulus or activity event, e.g. after stimulation of metabotropic receptors. The algorithm is based on a statistical test that defines a start point and end point of the signal. Details are given in the corresponding manuscript. The tool marks and calculates an area under the curve.

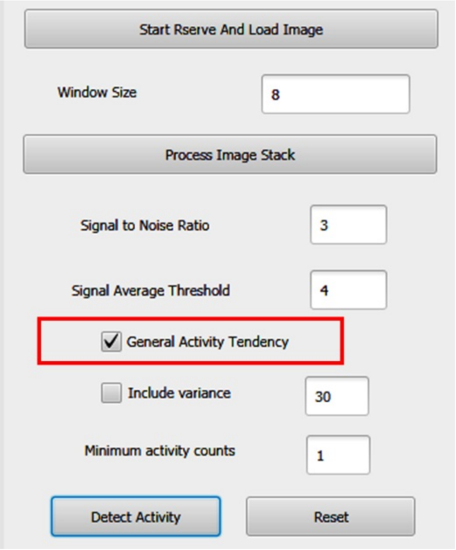

VI The ROI (region of interest) tool: *NeuronActivityROI-2*

In a second version of the tool, a region of interest (ROI) analysis function has been introduced. To use this version, download the Neuron Activity Analysis zip file from github: (https://github.com/jpits30/NeuronActivityTool_ROI.

Unzip the file in some known location and install it as described in Chapter I, and start the tool as described in Chapter II. All tool functions are implemented and can be used as described above.

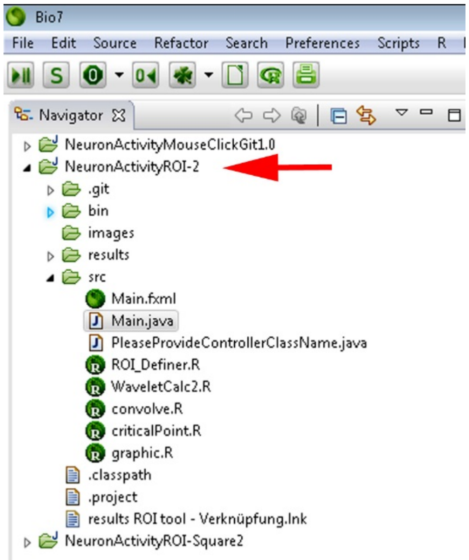

When the **ROI analysis** tool is not active, the tool will run in the standard mode and compute the calcium activity in grid windows.

When the **ROI analysis tool is selected**, the tool will behave as a region of interest analysis tool.

1) To compute selected one region of interest (ROI) or several selected ROIs activate the **ROI.**

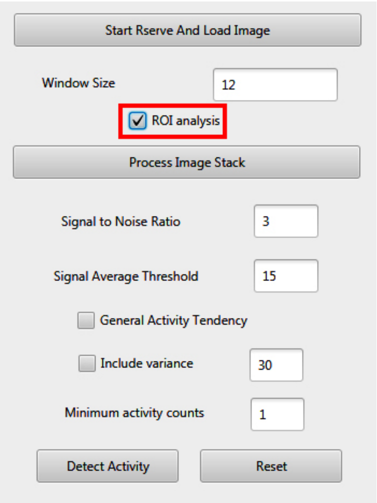

2) Open the ImageJ toolbar and select your preferred settings for activity computation.

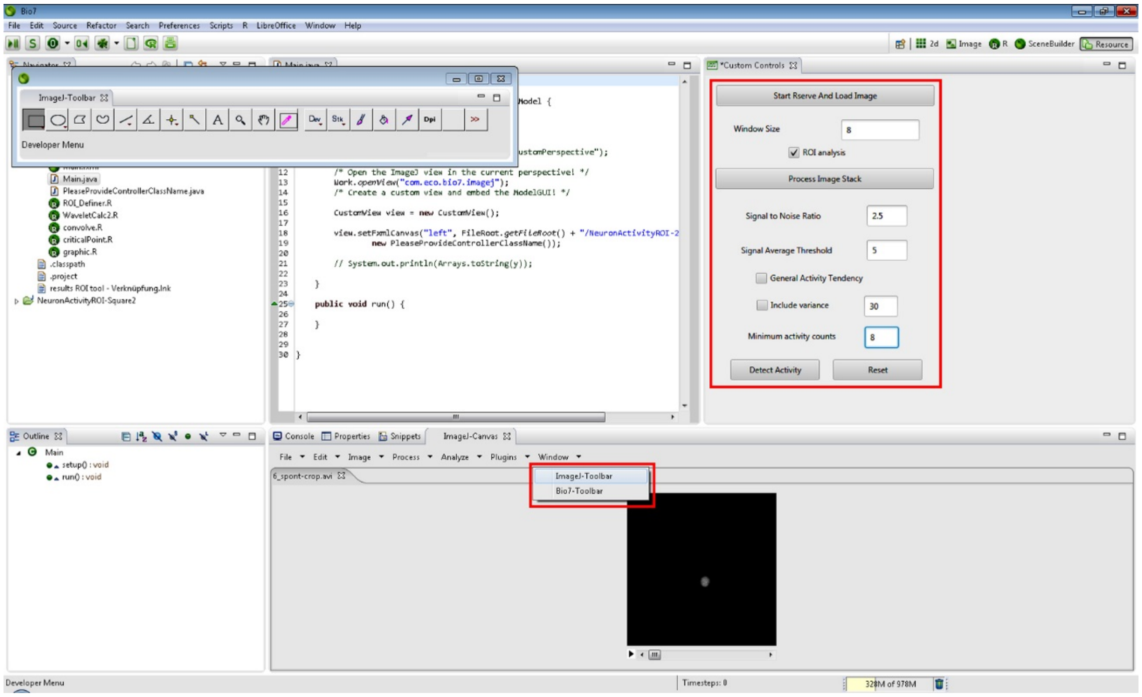

3) Paint your ROIs with the available ImageJ tools and add your ROIs to the ROI manager (in ImageJ: Analyze – Tools – ROI manager).

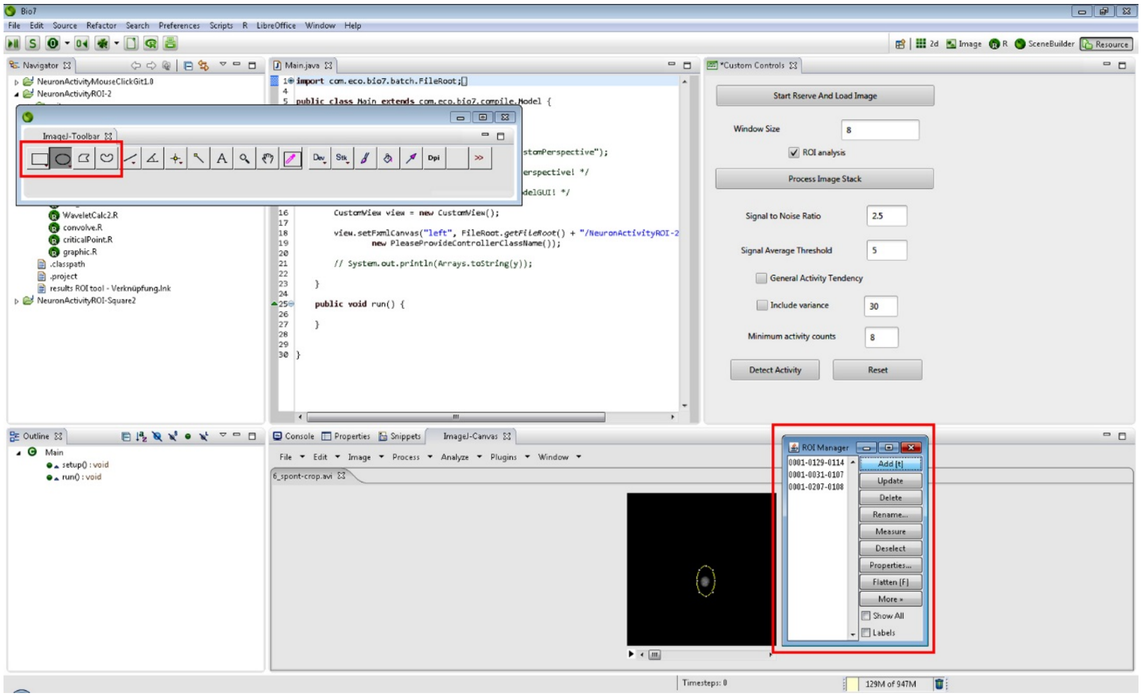

4) Open the **BIO7 toolbar**, activate first **Selection** and then transfer your ROI information to R (**Transfer to R**). A info window will confirm: **Values transferred!**

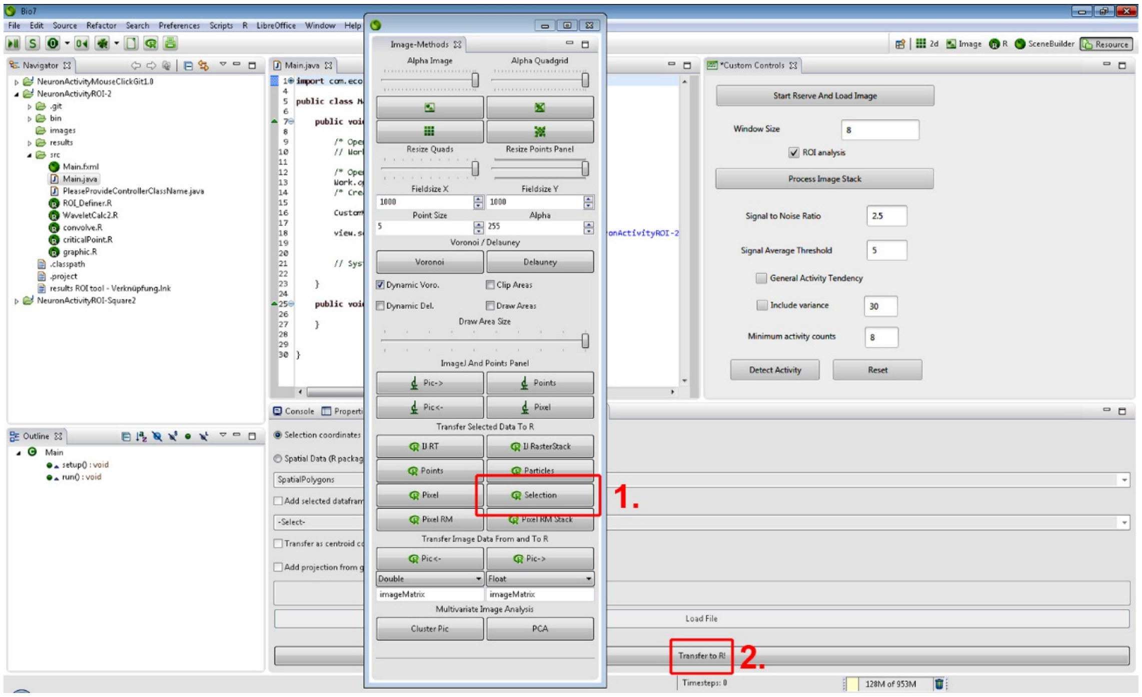

4) Switch to the **ImageJ-Canvas**. Compute activity events by the above-explained functions of the program. First, **Process Image Stack**, then **Detect Activity** to compute the activity events in your ROIs. The results will appear in a pdf-file in the result folder.

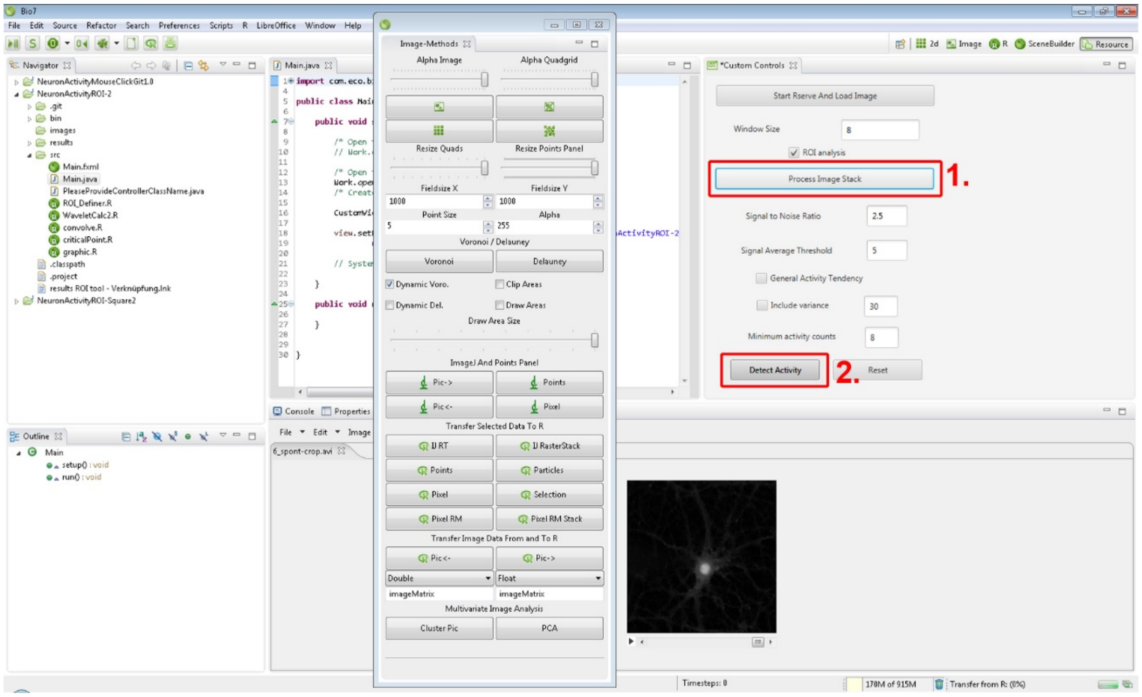

VII Data Output

The summary pdf shows on page 1 the first image of the time series, the grid, and loci of calcium activity (more than one event) indicated by red circles within the grid window. The diameter of the circles is the bigger the more activity the tool found. All activity events are summed up to give a virtual activity number, the 'total activity' value. This value represents all calcium events in the whole x,y-t images series to represent the activity state under a specific experimental condition.

Furthermore, the resulting pdf document shows all traces in which a calcium activity event was found. Activity events are marked and counted.

A txt-file is generated that shows the calculated numbers of activity per grid window in a x,y-table structure which is best opened with a typical editor program (e.g. Windows Editor).

1) Activity map and the virtual activity number "Total activity"

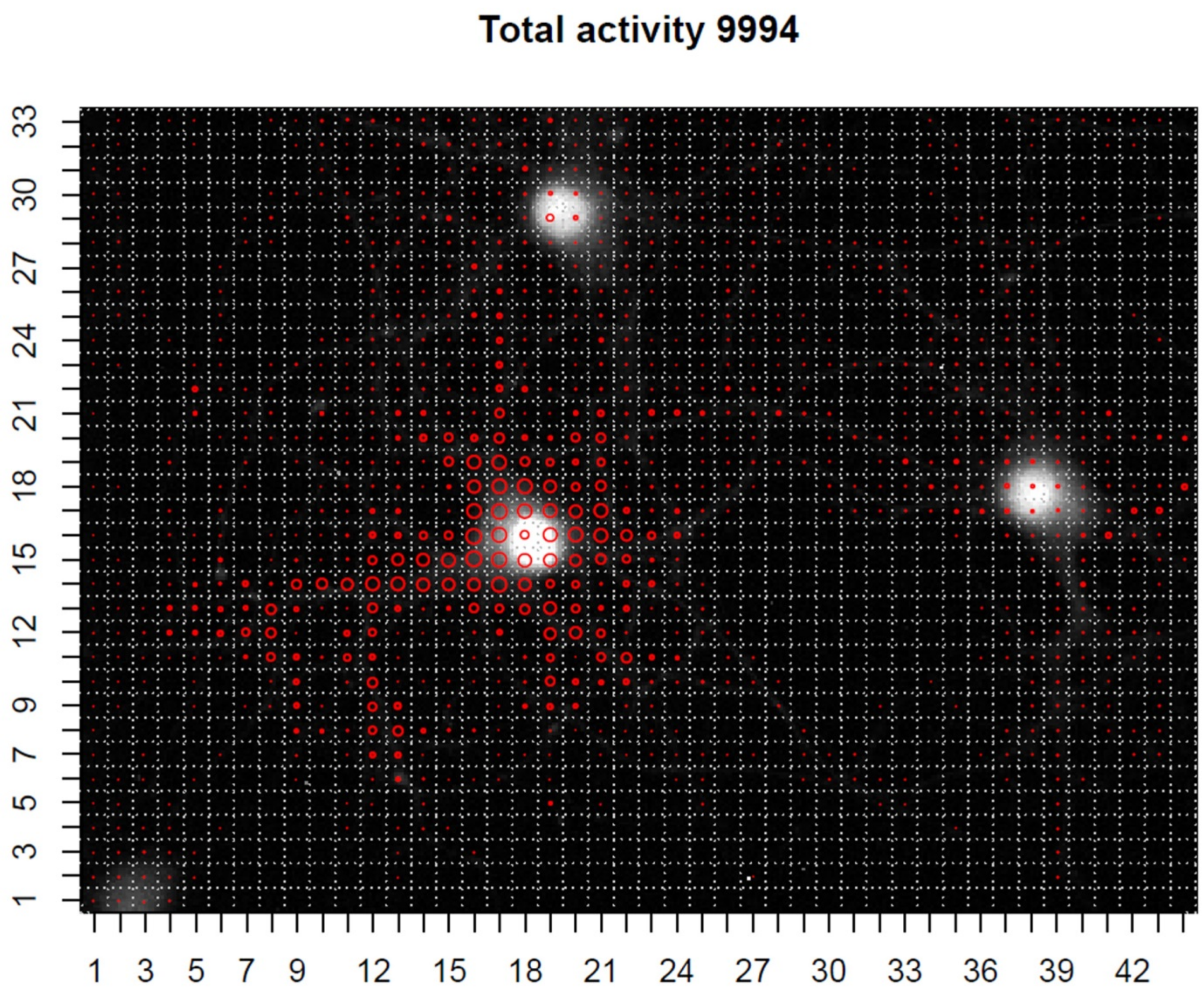

2) Signal raw traces (Graph) showing the mean grey value (pixel intensity) in a grid window (e.g. for the upper trace x,y = 14, 32, position in Array 58). Red squares mark computed activity events which contribute to the total activity number.

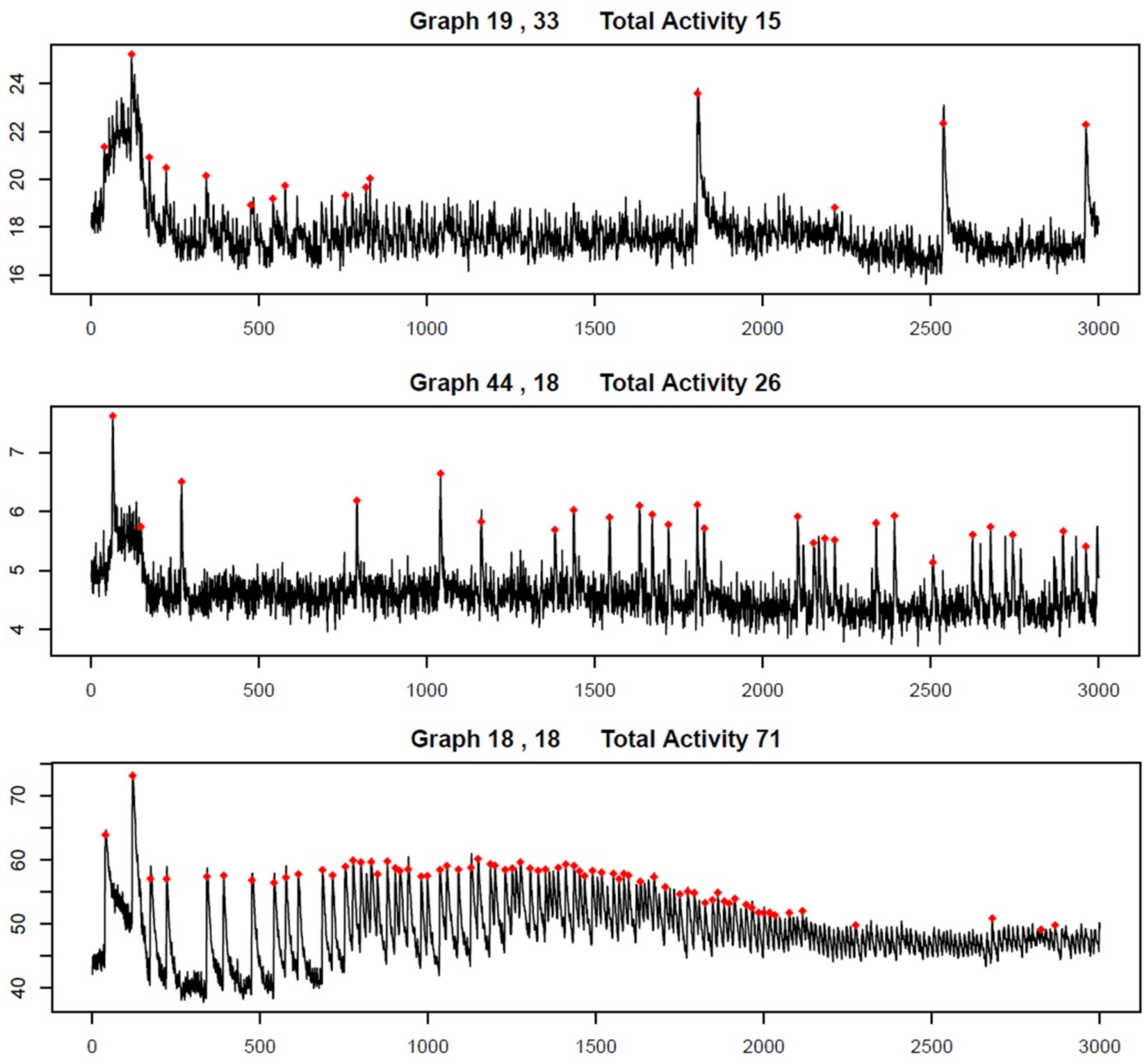

3) Txt-file to give the grid-specific activity event number in a table-like format.

resultWS8SNR3SAT4MAC1-neuron for manual.txt - Editor

Datei Bearbeiten Format Ansicht ?

0	2	0	1	2	0	0	4	1	11	10	11	9	8	4	8	6	8	15	5	7	7	3	1	2	2	0	2	2	0	0	0	0	3	0	0	2	4	7	4	4	4	4	0
2	1	0	0	1	0	0	0	2	8	1	2	6	12	12	8	13	8	13	5	6	5	3	1	0	2	8	10	5	2	0	0	0	3	3	0	0	2	0	0	3	4	0	0
3	2	1	0	0	0	0	0	0	7	3	0	3	0	10	13	12	17	11	8	5	8	1	2	5	5	9	3	4	3	3	0	0	0	5	0	3	0	0	0	0	0	0	0
3	2	0	0	0	0	0	4	4	3	7	5	7	7	8	4	7	12	18	15	7	5	5	6	3	2	6	2	0	0	0	0	0	2	0	0	0	0	0	0	0	0	0	0
1	1	0	0	0	0	2	9	0	0	13	4	5	13	17	7	7	10	35	22	6	5	7	9	7	6	7	2	0	0	0	0	0	0	3	6	0	3	0	5	0	0	6	0
1	5	0	0	0	0	4	4	0	0	4	7	9	5	9	9	13	12	9	8	5	6	11	3	3	3	9	4	3	4	6	7	4	0	4	8	4	7	6	0	0	0	0	0
1	4	0	0	0	2	0	0	0	0	0	10	5	2	10	19	14	6	10	8	6	9	4	1	2	4	8	0	0	2	3	11	4	0	0	8	8	5	0	0	0	0	0	0
1	5	2	0	0	4	0	0	0	0	0	11	2	4	8	12	19	11	6	9	8	6	5	0	0	12	5	0	0	0	0	5	8	0	0	6	8	3	0	0	0	0	0	0
2	5	1	0	0	4	0	0	0	0	0	7	10	8	8	17	22	6	6	7	13	8	0	0	2	9	3	2	3	0	0	0	4	9	6	0	6	4	0	0	0	4	0	0
2	0	0	1	0	4	0	0	0	7	6	9	7	7	5	4	27	7	6	5	16	8	2	1	2	6	2	3	3	3	0	0	0	7	8	7	9	9	4	0	0	0	7	0
1	2	0	0	3	4	0	4	8	2	1	11	7	8	5	2	29	6	0	4	11	7	5	2	3	5	2	2	7	6	6	7	9	10	10	9	6	7	7	0	0	0	0	0
0	0	0	0	22	8	6	7	0	10	5	9	6	9	6	7	30	22	8	10	11	15	6	5	4	15	11	7	5	0	0	0	4	5	11	9	7	6	7	0	0	0	0	0
1	0	0	0	15	0	6	7	0	14	2	9	18	19	7	8	43	3	3	18	29	12	23	22	17	10	12	17	13	11	0	0	3	4	8	8	8	7	7	7	16	0	0	0
0	0	0	3	0	1	5	4	6	3	2	4	17	32	40	31	48	22	17	41	45	13	8	0	2	2	7	0	0	4	12	9	4	0	6	5	12	11	13	8	10	12	14	15
0	0	0	8	0	0	7	0	4	6	6	8	6	5	44	65	68	44	35	25	36	6	7	0	4	6	9	7	9	5	0	5	18	10	17	9	15	17	12	7	3	0	0	0
1	1	0	0	0	3	1	0	0	2	7	7	12	2	14	62	71	71	59	39	49	1	13	8	3	10	3	0	0	8	5	4	7	14	12	11	22	18	17	11	8	5	8	26
0	0	0	7	0	12	0	0	2	8	5	19	18	0	6	70	75	71	67	62	66	25	13	18	10	5	0	0	0	6	7	7	9	7	15	16	19	16	14	10	9	22	25	0
1	0	0	5	3	7	0	2	2	2	12	29	27	36	39	75	73	42	69	70	66	53	35	26	4	0	0	0	0	0	0	0	0	0	6	0	11	10	10	14	27	13	5	0
1	1	0	3	2	15	0	2	12	2	10	40	56	61	66	76	73	67	65	55	49	37	14	7	0	0	0	0	0	0	0	0	0	0	0	0	0	8	9	11	0	5	6	7
1	1	0	5	15	13	26	12	46	52	56	66	66	62	59	61	73	60	37	34	11	27	24	7	4	0	0	0	0	0	0	0	0	0	0	0	0	0	10	17	0	0	5	0
1	2	2	18	18	20	19	48	20	4	3	45	24	7	15	43	38	47	60	39	18	24	3	8	8	0	0	0	0	0	0	0	0	0	0	4	5	0	8	10	4	8	0	0
3	2	6	19	19	27	37	47	2	0	26	35	2	2	2	10	23	0	55	57	38	6	0	5	7	5	0	0	0	0	0	0	0	0	0	0	5	7	8	7	10	10	3	0
1	1	2	2	3	2	14	39	25	8	33	27	4	0	0	4	3	12	34	1	40	48	21	15	0	6	5	0	0	0	0	0	0	0	0	3	5	7	9	5	6	6	5	0
1	1	1	0	1	0	0	4	24	0	7	48	2	4	4	3	3	9	44	25	20	26	11	7	12	7	6	3	0	0	0	0	0	1	0	0	0	7	9	5	4	0	4	0
2	1	0	0	2	0	2	3	23	4	0	40	32	0	6	0	2	16	27	20	0	6	5	0	0	0	0	7	0	0	0	3	0	0	3	0	0	7	8	6	4	0	7	0
1	0	0	0	0	0	0	0	17	15	10	37	47	19	13	12	1	0	5	1	2	2	3	2	0	0	0	0	6	0	0	0	0	0	0	0	6	6	7	0	4	4	4	0
1	0	3	0	0	2	0	0	6	0	3	26	27	1	0	0	2	2	2	0	2	1	4	2	4	2	3	0	4	3	5	0	0	0	0	2	4	4	8	0	0	5	5	0
1	2	2	0	1	0	0	0	1	0	0	4	18	9	1	2	2	0	2	0	0	0	0	1	3	0	0	0	3	4	5	3	3	0	0	0	2	0	6	5	0	0	0	0
1	2	0	2	0	0	0	0	0	0	1	2	0	5	0	0	0	0	14	0	2	0	0	0	4	0	0	0	0	0	0	3	2	0	0	0	0	0	6	0	0	0	0	0
2	3	3	3	0	1	0	0	0	0	3	0	3	6	3	0	0	0	0	0	0	0	0	0	0	0	0	0	0	0	0	0	0	0	2	0	0	0	6	0	0	0	0	0
2	5	8	5	3	0	0	0	0	0	0	0	1	0	0	5	0	0	0	0	0	0	0	0	0	0	0	0	0	0	0	0	0	0	0	0	0	0	7	0	0	0	0	0
5	7	7	5	3	0	0	0	0	0	0	0	2	0	0	0	0	0	0	0	0	0	0	0	0	0	1	0	0	0	0	0	0	0	0	0	0	0	6	0	0	0	0	0
4	5	6	5	0	0	0	0	0	0	0	0	0	0	0	0	0	0	0	0	0	0	0	0	0	0	0	0	0	0	0	0	0	0	0	0	0	0	0	0	0	0	0	0

4) If selected, the variance area will be marked in the imaging trace. The variance area value appears and is used to distinguish fluctuations in the imaging signal. The function of the tool is discussed in the corresponding manuscript.

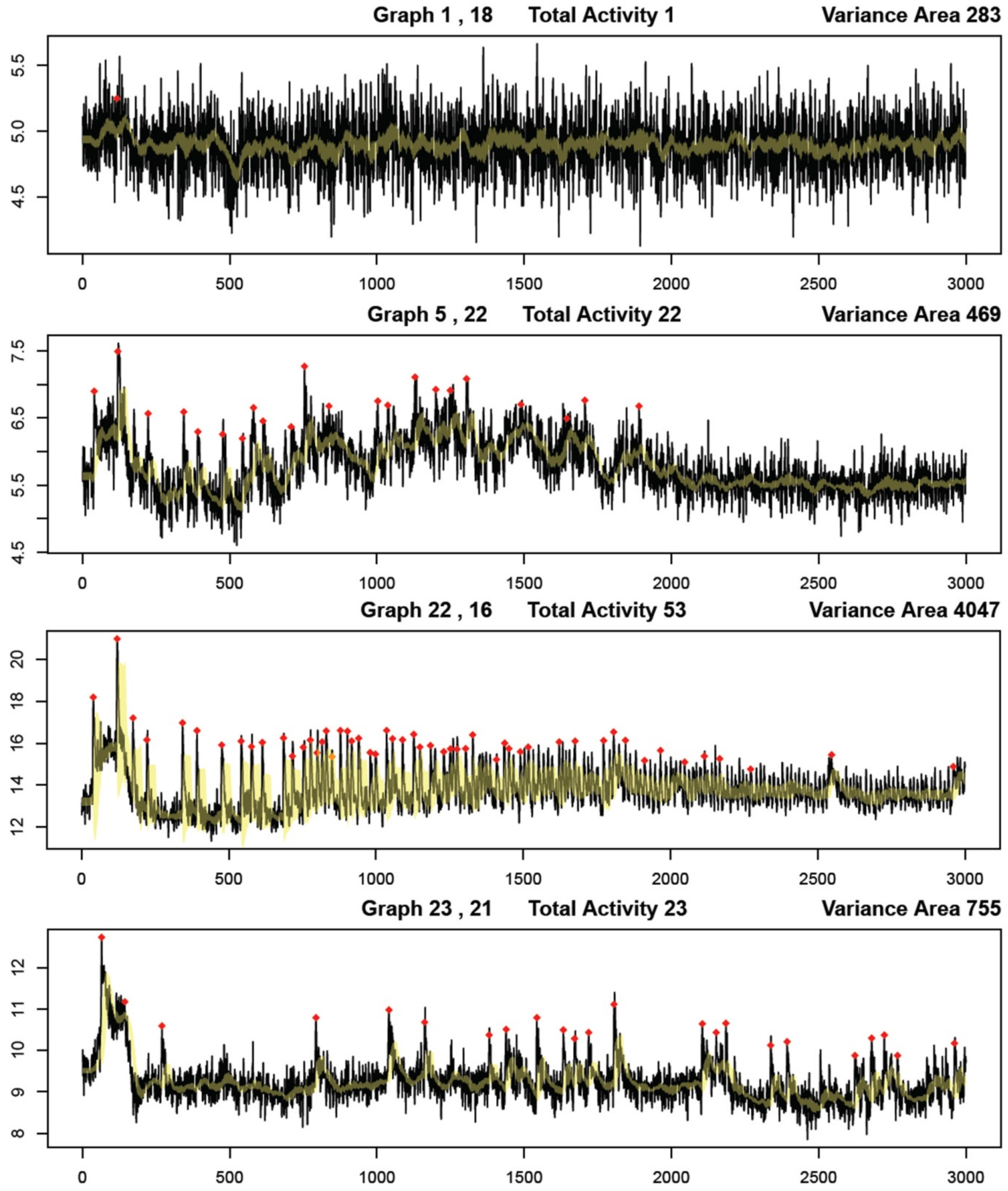

5) Phases of long-lasting activity are visualized by the General Activity Tendency tool. Please note, the activity detector is not well suited to analyze this waveform-like calcium oscillation. The example here shows a response of a monolayer of cultured astrocytes, stimulated with glutamate to activate metabotropic glutamate receptors.

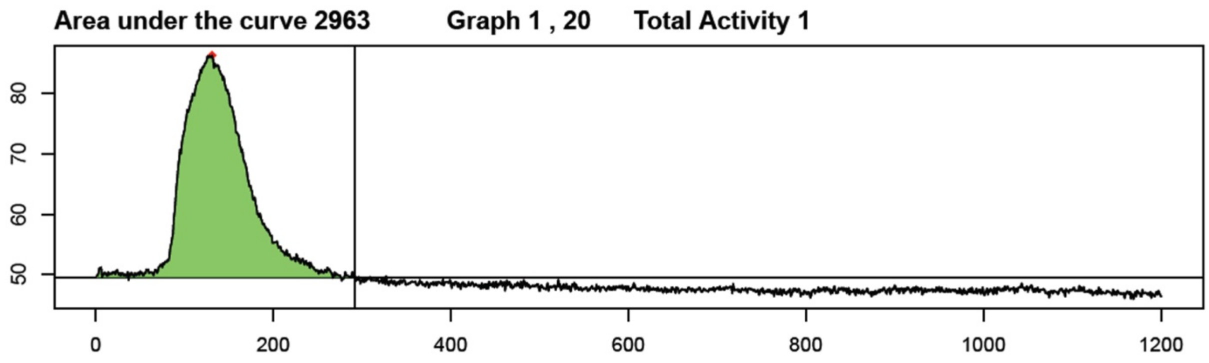

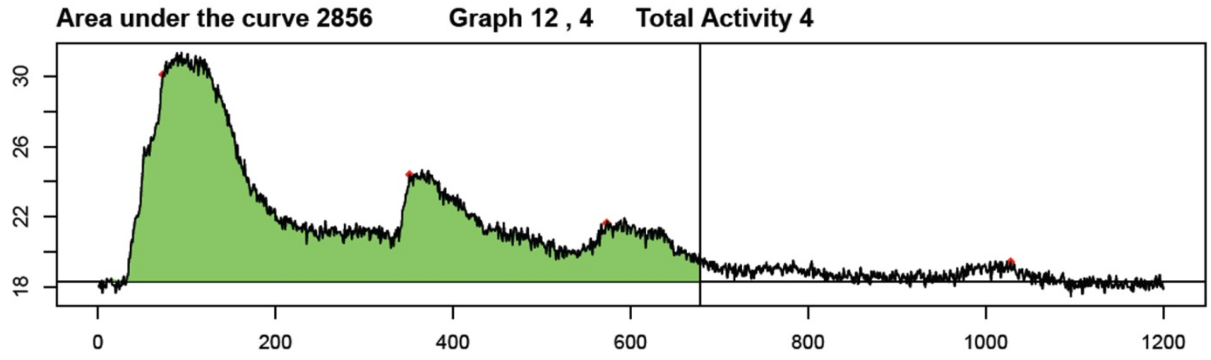

VIII Branch formation in the CWT algorithm

As explained in the paper, the CWT algorithm for peak recognition works in four steps. 1) Wavelet transform calculation, 2) ridge formation, 3) branch formation, and finally 4) peak detection by branch pruning.

Here we want to elucidate how the branch formation occurs. That means we first need to explain what a ridge is.

A ridge is a subset of the wavelet transform coefficients formed by local maxima, which are nearby in time and scale. The ridges of a wavelet transform are shown as blue dots in the next image.

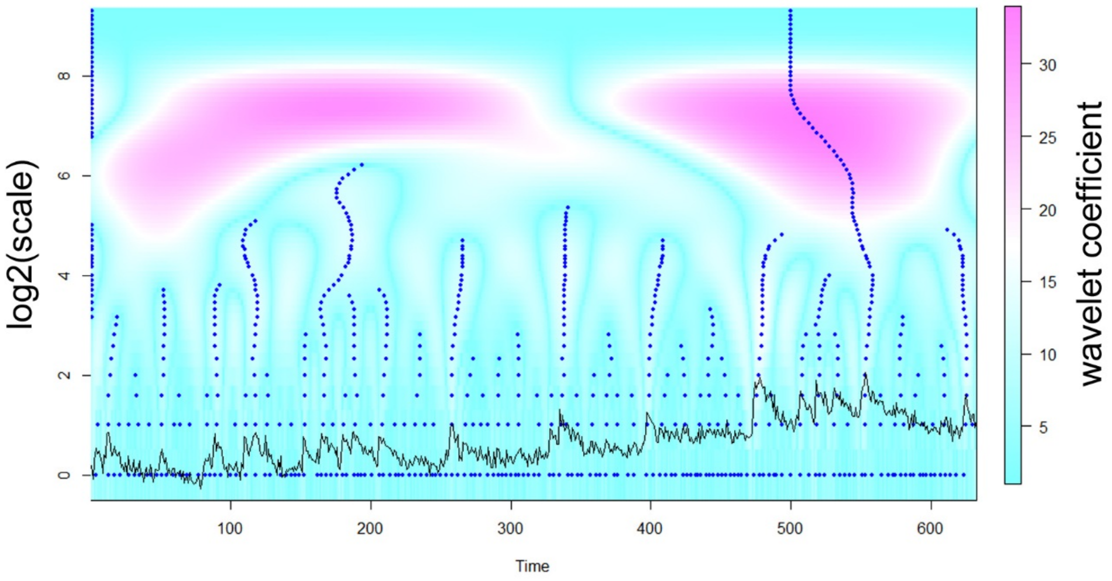

Then, in the step for branch formation, some of these ridges will become a branch. This is crucial, since peaks will be only be searched within the branches. To become a branch, a ridge has to comply with certain characteristics. The most relevant parameter is the length of the ridge. This parameter is called ***n.octave.min*** and it is passed to the wavCWTtree function. This parameter sets a threshold for the minimum length of a set of local maxima in order to be considered as a branch. It sets the minimum number of octaves from the scale range that a ridge has to contain, so it can become a branch.

In summary, a lower value of this parameter will allow more ridges to become branches.

Now we present two examples of the same calcium trace analyzed with two different *n.octave.min* parameters.

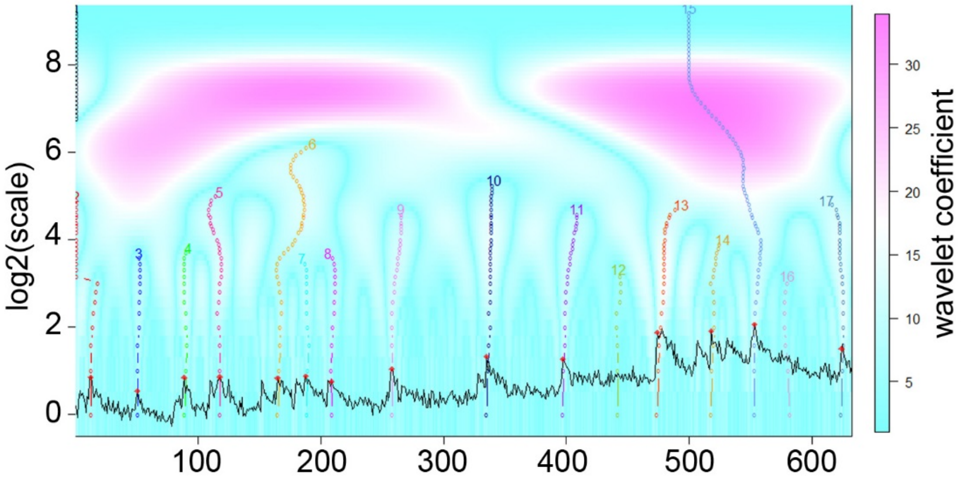

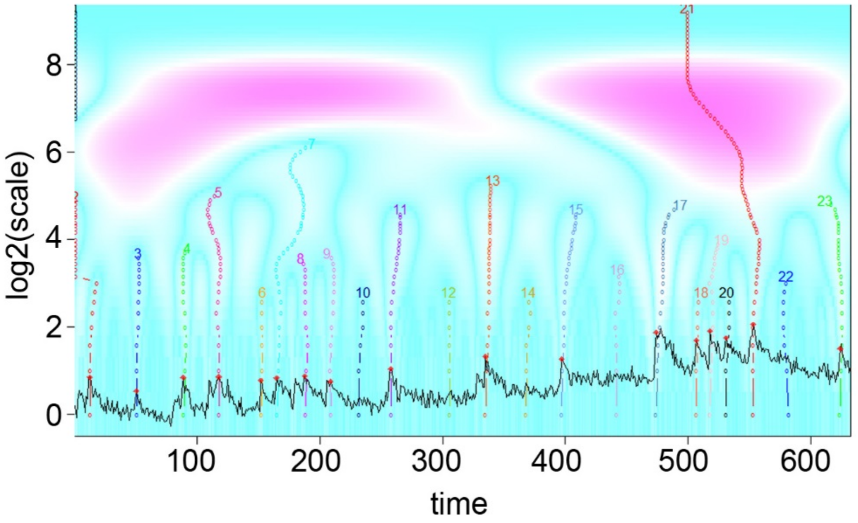

Upper figure: n.octave.min value of 1.0 (default).

Lower figure: n.octave.min value of 0.8 (set by the user) includes more activity candidates here, however on cost of signal detection stringency.

In the upper figure, the parameter ***n.octave.min*** is set to 1. In the right one, ***n.octave.min*** is set to 0.8. The branches are presented as colorful sequences of circles ending on a number. One number is assigned to each branch. It is seen that the lower figure counts 23 branches, while the upper figure counts 17. The extra branches in the lower figure are numbers 6, 10, 12, 14, 18, and 20. All these branches span over less than one octave, which is why they do not appear in the upper figure. Branches 6, 18, and 20 from the lower figure span less than one octave because they lay too close to other peak candidates, which are stronger and take their extrema on higher scales. In the case of branch 6, the extrema is taken by branch 7. In case of the two branches 18 and 20, branch 19 is the one that takes the extrema.

We use ***n.octave.min* = 1.0** as default setting. In our experience, the SNR parameter is powerful enough to tune the tool. However, in case of fast firing or in case of a high signal density over time, changing the *n.octave.min* values to 0.8 or even less can be useful.

The value can be changed in the window: ***WaveletCalc2.R*** in the **code line 44 and 50**.

1) Open ***WaveletCalc2.R*** and change ***n.octave.min*** in the **code line 44 and 50** to value 0.8 or another value to affect the tree pruning process.

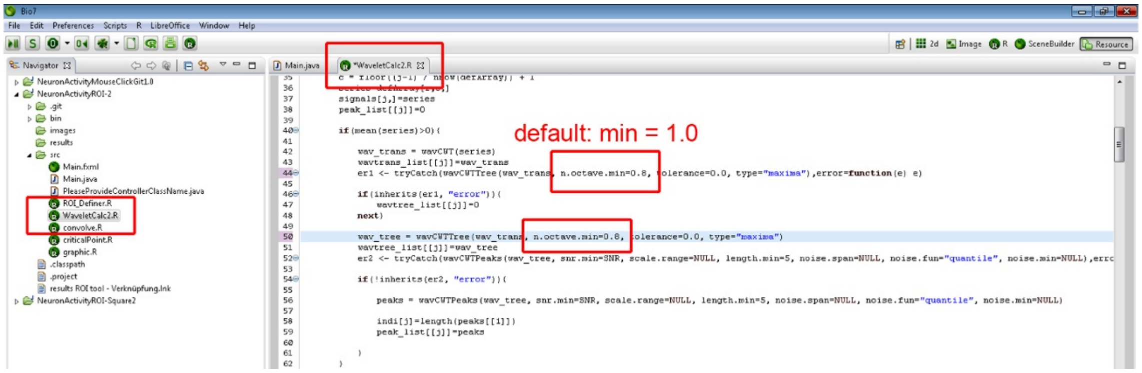

2) Save this change (File, save), **but don't rename the tool!**

IX Test data

Test data and corresponding results are provided in an archive (.7z-format) at:

https://www.biozentrum.uni-wuerzburg.de/bioinfo/computing/neuralactivitycubic/

Test dataset 1: Spiking neuron

Movie file: spiking_neuron.avi (1000 images)

Computed under the following settings.

- Window size: 8
- Signal to noise ratio: 2.5
- Signal average threshold: 3
- Minimum activity count: 2

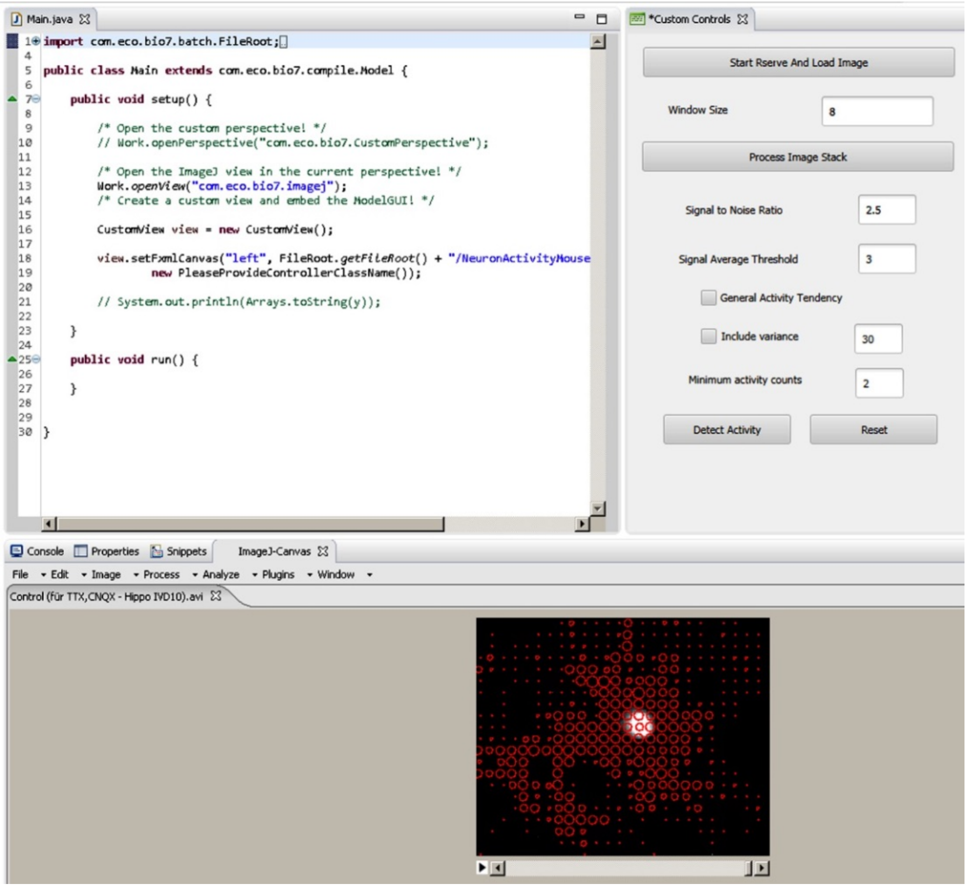

Attachments:

File for result No. 1: Spiking_neuron_summary.pdf

File for result No. 2: Spiking_neuron_activity in grid.txt

Test dataset 2: Neuron in Tetrodotoxin and CNQX to inhibit neuronal spiking

Movie file: Neuron_TTX-CNQX.avi (2251 images)

Computed under the following settings.

- Window size: 8
- Signal to noise ratio: 2.5
- Signal average threshold: 2
- Minimum activity count: 2
- include variance

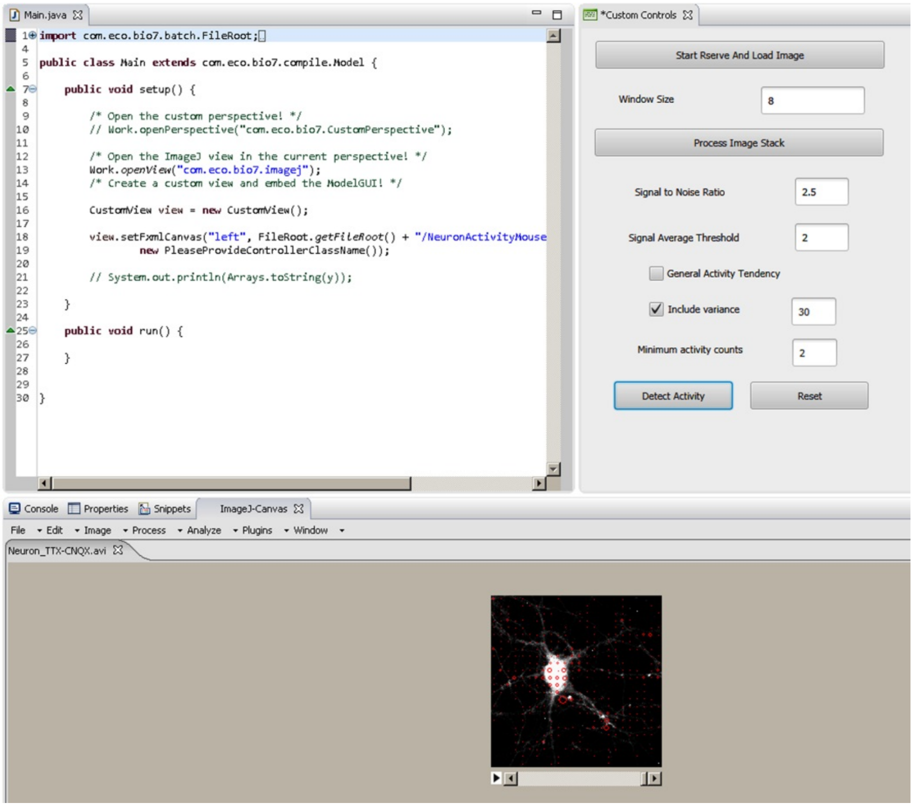

Attachments:

File for result No. 1: Neuron_TTX-CNQX_summary.pdf

File for result No. 2: Neuron_TTX-CNQX_activity in grid.txt
